# Supplementary material for: Limited evidence that body size shrinking and shape-shifting alleviate thermoregulatory pressures in a warmer world
Source: Commun Biol. 2025 May 7;8:707. doi: 10.1038/s42003-025-08131-7 (PMC12059039; doi:10.1038/s42003-025-08131-7)
Supplement: Supplementary file 2 — Supplementary Material [file 42003_2025_8131_MOESM2_ESM.pdf]

Supplemental Material

Joshua Tabh, Elin Persson, Maria Correia, Ciarán Ó Cuív, Elisa Thoral & Andreas Nord

Table of Contents

1.0 Size measurement validations **3**
Overview . . . 4
Validating accuracy of digital appendage length measurements . . . 21

2.0 Data organisation and analysis of growth **31**
Overview . . . 32
Data compilation . . . 32
Modelling effects of the developmental thermal environment on morphology . . . 72
Effects of rearing conditions on allometric relationship between mass and appendage length . . . 170

3.0 Metabolic slope and repeatability analyses **181**
Overview . . . 182
Data compilation . . . 182
Import, collation, and visualisation of resting energy expenditure data . . . 187
Visualising and quantifying metabolic slopes in the cold (juveniles) . . . 203
Visualising and quantifying metabolic slopes in the cold (adults) . . . 224
Analysing effects of morphometry on metabolic slopes in the cold . . . 241
Testing effects of morphometry on metabolic slopes in the heat . . . 373

4.0 Effect of morphology on evaporative cooling **531**
Overview . . . 532
Data import, collation, and filtration . . . 532
Testing effects of morphology and thermal history on evaporative cooling . . . 550

5.0 Estimating lower critical temperature **693**
Overview . . . 694
General methods . . . 694
Data import, collation, and filtration . . . 694
Model construction . . . 699

6.0 Morphology and body temperature responses to heat and cold **718**
Overview . . . 719
Body temperature measurement . . . 719
Description of analyses . . . 719
Data import, collation, and filtration . . . 720
Analysing body temperature responses to cold . . . 734
Analysing body temperature responses to heat . . . 765

1.0 Size measurement validations

Overview

In our study, we use body mass as a proxy for body size (or structural size) in quail. However, tarsus length is also often assumed as a proxy of structural size in other avian species (e.g. Weeks et al, 2020). If the latter is true but not the former, effects of body mass on thermal physiology, conditional upon tarsus length, may be more suitably interpreted as effects of body *condition* rather than body *size* on such. Conversely, effects of tarsus length on thermal physiology, conditional upon body mass, may be interpreted as effects of *inverse* body condition, rather than mere appendage length, on such. For example, a positive effect of tarsus length on metabolic slopes in the cold, when body mass is set at its average (commonly our model intercepts) may indicate a negative effect of body condition on thermal resistance, rather than a positive effect of appendage length.

To assess whether body mass or tarsus length are indeed predictors of structural size, we tested how well each predicted other measures of structural size in a sub-sample of mature and sacrificed Japanese quail (n = 20). To do so, we began by measuring tarsus length (here, digital, as described in Tabh et al, 2025) and five other possible indicators of structural size, including: (1) maximum keel depth (mm), (2) maximum skull height (mm), (3) maximum body width (mm), (4) synsacrum width, and (5) maximum body height (from back to deepest point of keel); measurements were collected by two independent researchers (J.T. and E.P.). Following measurement, we then quantified precision of each structural size metric by calculating the mean coefficient of variation (CV) per metric across all birds measured. Metrics with the lowest mean CV were considered to be the most precise and those with the highest mean CV the least precise. Finally, the two size metrics with the lowest mean CV were regressed against tarsus length and body mass using simple Bayesian linear regressions.

We begin below by loading in packages and functions necessary for our analysis.

```
knitr::knit_hooks$set(chunk = local({
def.chunk.hook <- knitr::knit_hooks$get("chunk")
  function(x, options) {
  x <- def.chunk.hook(x, options)
  ifelse(options$size != "normalsize",
    paste0("\n \\", options$size, "\n\n", x, "\n\n \\normalsize"), x
  )
}
}))

knitr::opts_chunk$set(fig.pos = "H", out.extra = "")
knitr::opts_chunk$set(size = "footnotesize")

# First loading in packages

library("tidyverse")
library("easypackages")

packageList <- c("bayesplot", "brms", "brmsMethods",
                 "doParallel", "foreach", "ggpubr",
                 "kableExtra", "latex2exp", "patchwork",
                 "priorsense", "showtext", "tidybayes",
                 "wesanderson")

libraries(packageList)

caption <- paste0("R packages and their respective versions used for",
                  " data organisation and analysis in this study."
)
```

```
sapply(packageList, function(x) {
  y <- as.character(packageVersion(x))
  return(y)
}, simplify = FALSE) %>%
  enframe(., name = "Package", value = "Version") %>%
  as.data.frame(.) %>%
  kbl(.,
    longtable = T, booktabs = T,
    caption = caption
  ) %>%
  kable_styling(latex_options = "striped")
```

Table 1: *R packages and their respective versions used for data organisation and analysis in this study.*

Package	Version
bayesplot	1.11.1
brms	2.22.7
brmsMethods	0.0.0.9000
doParallel	1.0.17
foreach	1.5.2
ggpubr	0.6.0
kableExtra	1.4.0
latex2exp	0.9.6
patchwork	1.2.0
priorsense	1.0.2
showtext	0.9.7
tidybayes	3.0.6
wesanderson	0.3.7

```
# Loading additional functions

pp_check2 <- function(model, resp = NA, ndraws = 500,
                      xlab = "label", colour = "lightblue") {
  require(brms)
  require(ggplot2)
  stopifnot("Model must be a brmsfit object" = is.brmsfit(model))

  if (is.na(resp)) {
    resp <- model$formula$resp
  }

  p1 <- brms::pp_check(model, ndraws = ndraws, resp = resp) +
    scale_colour_manual(
      values = c("black", colour),
      labels = c("y", "yhat"),
      name = NULL
    ) +
    xlab(xlab) +
    ylab("Density") +
    theme_classic()
  return(p1)
```

```
}

chainCheck <- function(model, rDig = 3) {
  require(brms)
  stopifnot("Model must be a brmsfit object" = is.brmsfit(model))

  Rhat <- paste0(
    "Rhat range: ",
    round(min(rhat(model)), digits = rDig),
    " - ",
    round(max(rhat(model)), digits = rDig)
  )
  Neff <- paste0(
    "Neff/N range: ",
    round(min(neff_ratio(model)), digits = rDig),
    " - ",
    round(max(neff_ratio(model)), digits = rDig)
  )
  cat(paste0(Rhat, "\n", Neff))
}

quantileCIs <- function(x, rnd = 3, cis = c(50, 95), sci_note = FALSE) {
  require(tidyverse)

  if (class(x)[1] != "brmsfit") {
    return("x must be a brmsfit object.")
  }
  if (length(cis) != 2) {
    return("cis must be a vector of integers with length 2")
  }

  prbs = c()
  nColNames = c()
  for (i in 1:length(cis)){
    prbs = c(prbs, c(0.5 - (cis[i]/100)/2, 0.5 + (cis[i]/100)/2))
    nColNames = c(nColNames,
                  paste0("Low_CI_", cis[i]),
                  paste0("High_CI_", cis[i])
    )
  }

  modelFrame = as.data.frame(x)

  Results <- apply(modelFrame, MARGIN = 2, FUN = quantile,
        probs = prbs, type = 8) %>%
    t() %>%
    as.data.frame() %>%
    rownames_to_column(var = "par") %>%
    `colnames<-`(c("Parameter", nColNames))

  if (sci_note == FALSE) {
    Results <- apply(modelFrame, MARGIN = 2, FUN = quantile,
        probs = prbs, type = 8) %>%
```

```
    t() %>%
    as.data.frame() %>%
    rownames_to_column(var = "par") %>%
    `colnames<-`(c("Parameter", nColNames))

  } else if (sci_note == TRUE) {
    Results <- apply(modelFrame, MARGIN = 2, FUN = quantile,
        probs = prbs, type = 8) %>%
    t() %>%
    as.data.frame() %>%
    rownames_to_column(var = "par") %>%
    `colnames<-`(c("Parameter", nColNames)) %>%
    mutate_at(.vars = vars(-Parameter),
              .funs = function(x){
                return(format(x, scientific = TRUE))
              }
    )
  }

  return(Results)
}

# Installing font

font_add_google(name = "Noto Sans", family = "Noto Sans")

# Setting working directory

setwd("/Users/joshuatabh/analyses")
```

Next, we load our validation data and plot CVs by their size metrics.

```
# Loading in data.

structuralData <- read.csv("structuralSizeData.csv") %>%
  dplyr::select(-c("measurer", "date", "headPhoto", "legPhoto", "notes")) %>%
  group_by(ring) %>%
  summarise_all(.funs = list("Mean" = mean, "CV" = function(x) {
    sd(x) / mean(x)
  })) %>%
  merge(., read.csv("digitalDataForStructuralSizeAnalyses.csv"),
    by = "ring"
  ) %>%
  dplyr::select(-wingLength_CV)

# Renaming columns

colnames(structuralData) <- gsub(
  "_", "",
  colnames(structuralData)
)

# Plotting coefficients of variation for structural
# size measures

plotOrder <- structuralData %>%
  dplyr::select(
    bodyWidthCV, bodyHeightCV, keelDepthCV,
    synsacrumWidthCV, skullHeightCV
```

```
  ) %>%
  pivot_longer(everything(),
    names_to = "metric",
    values_to = "CV"
  ) %>%
  merge(., tribble(
    ~Metric, ~metric,
    "Body Width (mm)", "bodyWidthCV",
    "Body Height (mm)", "bodyHeightCV",
    "Keel Depth (mm)", "keelDepthCV",
    "Synsacrum Width\n(mm)", "synsacrumWidthCV",
    "Skull Height (mm)", "skullHeightCV"
  ), by = "metric") %>%
  group_by(Metric) %>%
  summarise("Mean" = mean(CV)) %>%
  arrange(desc(Mean)) %>%
  pull(Metric)

showtext_auto()

cvPlot <- structuralData %>%
  dplyr::select(
    bodyWidthCV, bodyHeightCV, keelDepthCV,
    synsacrumWidthCV, skullHeightCV
  ) %>%
  pivot_longer(everything(),
    names_to = "metric",
    values_to = "CV"
  ) %>%
  merge(., tribble(
    ~Metric, ~metric,
    "Body Width (mm)", "bodyWidthCV",
    "Body Height (mm)", "bodyHeightCV",
    "Keel Depth (mm)", "keelDepthCV",
    "Synsacrum Width\n(mm)", "synsacrumWidthCV",
    "Skull Height (mm)", "skullHeightCV"
  ), by = "metric") %>%
  dplyr::select(Metric, CV) %>%
  mutate(
    "CV" = CV * 100,
    Metric = factor(Metric, levels = plotOrder)
  ) %>%
  ggplot(aes(x = Metric, y = CV, fill = Metric)) +
  geom_point(
    pch = 21, size = 2.5, colour = "black", alpha = 0.5,
    position = position_jitter(width = 0.3)
  ) +
  stat_summary(
    geom = "errorbar", fun.data = "mean_se",
    colour = "black", width = 0.3
  ) +
  stat_summary(
    geom = "point", fun = "mean",
    pch = 21, size = 4, colour = "black"
  ) +
  geom_segment(aes(x = 4.5, y = 7.5, xend = 4.05, yend = 3),
    size = 0.4,
    arrow = arrow(length = unit(0.2, "cm"))
  ) +
  geom_segment(aes(x = 4.5, y = 7.5, xend = 4.95, yend = 2.7),
    size = 0.4,
    arrow = arrow(length = unit(0.2, "cm"))
  ) +
  ylab("Coefficient of Variation (%)") +
  scale_fill_manual(
    values =
      c(
```

```
        "#ff9690", "#ffada9",
        "#fec4c1", "#fee3e2",
        "#fff3f2"
      )
  ) +
  theme_classic() +
  theme(
    axis.title.x = element_blank(),
    legend.position = "none",
    axis.title.y = element_text(family = "Noto Sans"),
    axis.text = element_text(family = "Noto Sans")
  )

cvPlot
```

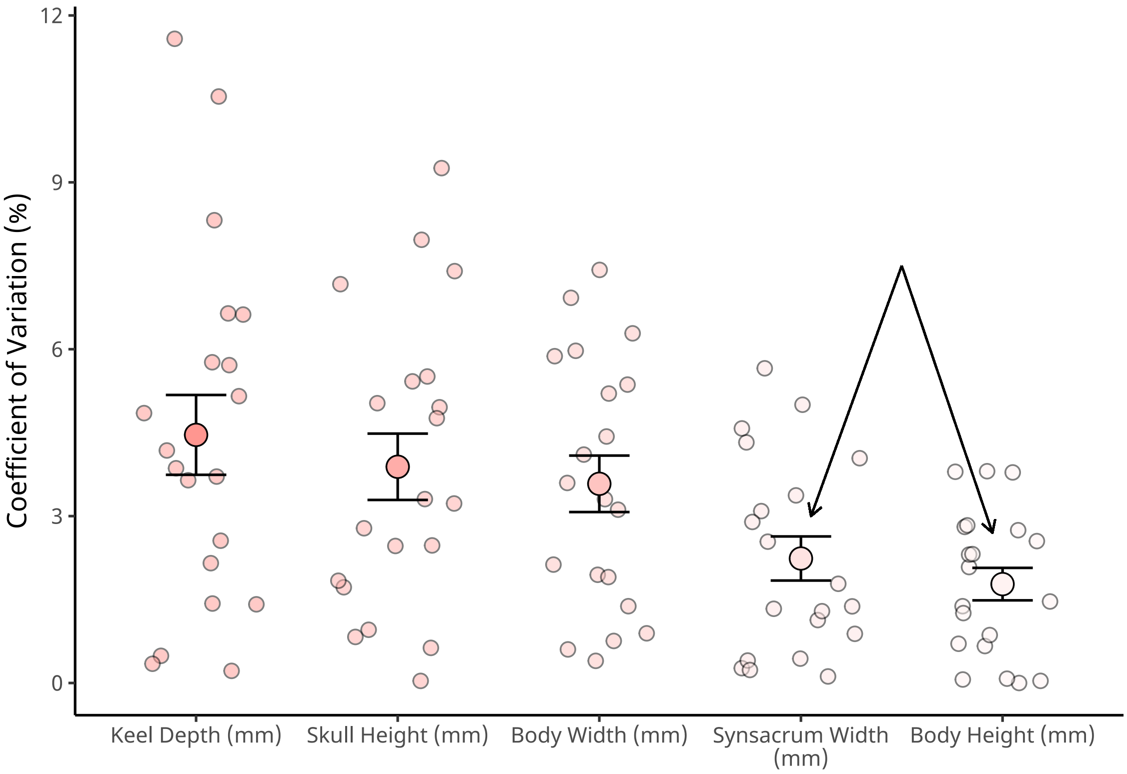

Figure 1: *Precision of skeletal size metrics derived from mature and deceased Japanese quail (n = 20) by two observers. Small dots indicate raw coefficients of variation (CVs) per bird, and large dots indicate mean CVs per size metric. Errorbars indicate +/- one standard error around means. The black errors identify the two skeletal size metrics with highest precision (i.e. lowest mean CV).*

```
ggsave("./plots/structuralSizeCVs.pdf",
  cvPlot,
  dpi = 800, height = 7, width = 8
)

showtext_auto(enable = FALSE)
```

Synsacrum width and maximum body height, being the most precise metrics of skeletal size, are selected for use in further analyses. In those analyses, correlations between tarsus length, body mass, and each selected metric are visualised.

```
# Plotting tarsus and body mass by the two structural size
# metrics with the lowest coefficients of variation
```

```r
# (body height and synsacrum width)

showtext_auto()

p1 <- structuralData %>%
  dplyr::select(ring,
    tarsusLengthMean,
    "Body Height (mm)" = bodyHeightMean,
    "Synsacrum Width\n(mm)" = synsacrumWidthMean
  ) %>%
  pivot_longer(c("Body Height (mm)", "Synsacrum Width\n(mm)"),
    names_to = c("par"), values_to = c("measure")
  ) %>%
  ggplot(aes(x = tarsusLengthMean, y = measure, fill = par)) +
  facet_wrap(~par,
    scales = "free",
    strip.position = "left"
  ) +
  geom_point(
    pch = 21, size = 2.5,
    colour = "black", alpha = 0.7
  ) +
  geom_smooth(
    method = "lm", se = FALSE,
    colour = "black", linetype = "dashed"
  ) +
  scale_fill_manual(values = c("#fff3f2", "#fee3e2")) +
  xlab("Tarsus Length (mm)") +
  theme_classic() +
  theme(
    legend.position = "none",
    strip.background = element_blank(),
    strip.placement = "outside",
    axis.title.y = element_blank(),
    strip.text = element_text(
      size = 12,
      colour = "black",
      family = "Noto Sans"
    ),
    axis.title.x = element_text(
      size = 12,
      colour = "black",
      family = "Noto Sans"
    )
  )

p2 <- structuralData %>%
  dplyr::select(ring,
    massMean,
    "Body Height (mm)" = bodyHeightMean,
    "Synsacrum Width\n(mm)" = synsacrumWidthMean
  ) %>%
  pivot_longer(c("Body Height (mm)", "Synsacrum Width\n(mm)"),
    names_to = c("par"), values_to = c("measure")
  ) %>%
  ggplot(aes(x = massMean, y = measure, fill = par)) +
  facet_wrap(~par, scales = "free", strip.position = "left", ) +
  geom_point(
    pch = 21, size = 2.5,
    colour = "black", alpha = 0.7
  ) +
  geom_smooth(
    method = "lm", se = FALSE,
    colour = "black", linetype = "dashed"
  ) +
  scale_fill_manual(values = c("#fff3f2", "#fee3e2")) +
  xlab("Body Mass (g)") +
```

```
  theme_classic() +
  theme(
    legend.position = "none",
    strip.background = element_blank(),
    strip.placement = "outside",
    axis.title.y = element_blank(),
    strip.text = element_text(
      size = 12,
      colour = "black",
      family = "Noto Sans"
    ),
    axis.title.x = element_text(
      size = 12,
      colour = "black",
      family = "Noto Sans"
    )
  )

structuralSizePlot <- p1 / p2
structuralSizePlot
```

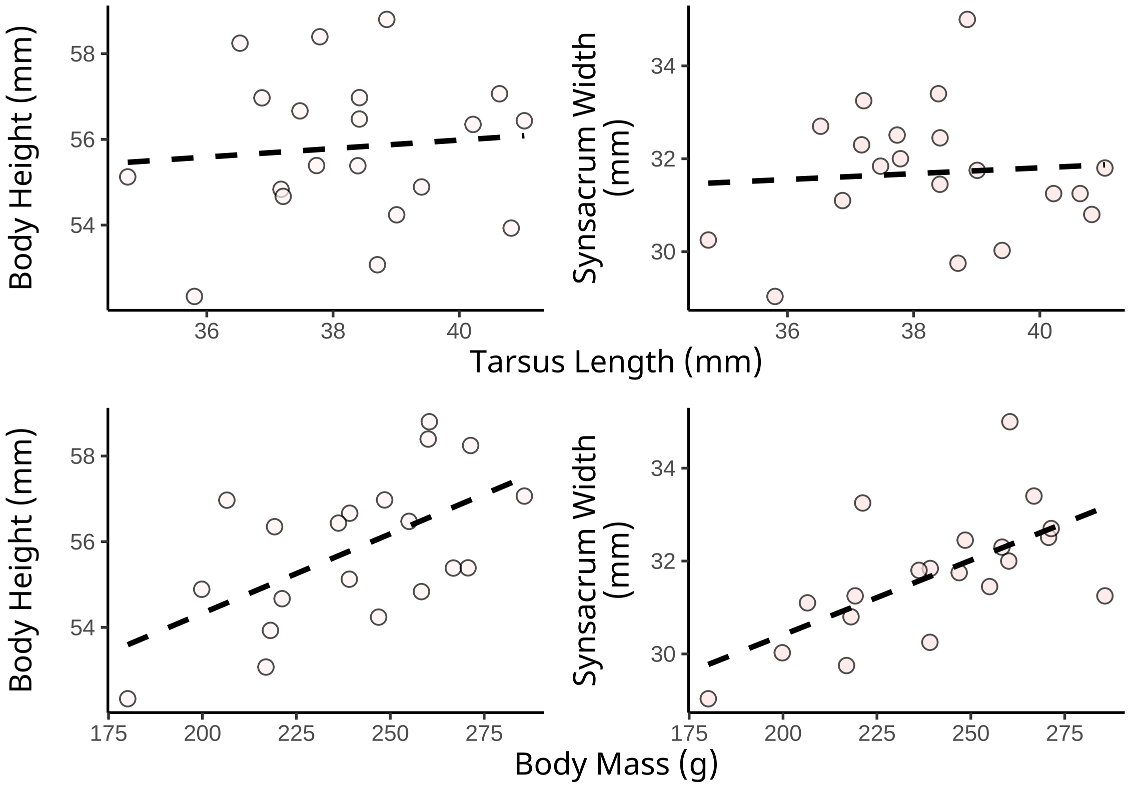

Figure 2: *Relationship between tarsus length (mm) or body mass (g) and both maximum body height (mm) and synsacrum width (mm) in twenty mature and deceased Japanese quail. Dots indicate raw measurements and dashed lines indicate predicted linear relationships estimated by the R package ggplot2 (Wickham, 2011). All measurements were collected using digital and analogue calipers.*

```
ggsave("./plots/tarsusMassByStructuralSizeMeasures.pdf",
  structuralSizePlot,
  dpi = 800, height = 7, width = 8
)

showtext_auto(enable = FALSE)
```

Last, linear regressions between tarsus length and both synsacrum width and body height are constructed, followed by regressions between body mass and both synsacrum width and body height. In these models, all size metrics are mean-centred prior to analysis to simplify interpretation of model intercepts. Priors for slope coefficients were normal, centred at zero, and wide, with standard deviations equaling the range of our observed response variable divided by the range of our observed dependent variable. All intercept priors were also normal, centred at zero, and with standard deviations of 2, and error (ϵ) priors were exponential with scaling factors of 1.

```
# Running basic linear models to compare predictive
# capacity of tarsus length and body mass on
# structural size metrics. Note that only coefficients,
# 95% credible intervals and r2 values are pulled from each model.
# Priors for each slope are weak and wide. Results
# from body mass as predictor are added for comparison
# purposes.

upperLimit <- with(
  structuralData,
  diff(range(bodyHeightMean, na.rm = T)) /
    diff(range(tarsusLengthMean, na.rm = T))
)

tarsusHeightModel <- brm(
  data = structuralData %>%
    dplyr::select(
      "bodyHeight" = bodyHeightMean,
      "tarsus" = tarsusLengthMean
    ) %>%
    mutate(
      "bodyHeight" = bodyHeight -
        mean(bodyHeight, na.rm = T),
      "tarsus" = tarsus - mean(tarsus, na.rm = T)
    ),
  family = "gaussian",
  bodyHeight ~ tarsus,
  prior = c(
    set_prior("normal(0, 2)", class = "Intercept"),
    set_prior(paste0("normal(0, ", upperLimit / 2, ")"),
      class = "b"
    ),
    set_prior("exponential(1)", class = "sigma")
  ),
  iter = 50000, warmup = 10000, cores = 4, chains = 4, thin = 20,
  silent = TRUE, refresh = 0,
  file = "./models/tarsusBodyHeight.Rds"
)

p1 <- pp_check2(tarsusHeightModel,
  xlab = "Max. Body Height (mm;\nMean-Centred)"
) +
  theme(
    legend.position = "none"
  )

p2 <- tarsusHeightModel$data %>%
  mutate(
    "Residuals" =
      residuals(tarsusHeightModel,
        type = "ordinary",
        robust = TRUE
      )[, "Estimate"]
  ) %>%
  ggplot(aes(x = Residuals)) +
  geom_density(
    colour = "black",
    fill = "lightblue", alpha = 0.5
```

```
  ) +
  xlab("Maximum Body Height\nResiduals (mm)") +
  ylab("Density") +
  theme_classic()

p3 <- tarsusHeightModel$data %>%
  mutate(
    "Residuals" =
      residuals(tarsusHeightModel,
        type = "ordinary",
        robust = TRUE
      )[, "Estimate"]
  ) %>%
  ggplot(aes(x = tarsus, y = Residuals)) +
  geom_point(
    size = 2, pch = 21, colour = "black",
    fill = "lightblue", alpha = 0.5
  ) +
  xlab("Tarsus Length (mm)") +
  ylab("Max. Body Height\nResiduals (mm)") +
  theme_classic()

upperLimit <- with(
  structuralData,
  diff(range(synsacrumWidthMean, na.rm = T)) /
    diff(range(tarsusLengthMean, na.rm = T))
)

tarsusSynsacrumModel <- brm(
  data = structuralData %>%
    dplyr::select(
      "synsacrumWidth" = synsacrumWidthMean,
      "tarsus" = tarsusLengthMean
    ) %>%
    mutate(
      "synsacrumWidth" = synsacrumWidth -
        mean(synsacrumWidth, na.rm = T),
      "tarsus" = tarsus - mean(tarsus, na.rm = T)
    ),
  family = "gaussian",
  synsacrumWidth ~ tarsus,
  prior = c(
    set_prior("normal(0, 2)", class = "Intercept"),
    set_prior(paste0("normal(0, ", upperLimit / 2, ")"),
      class = "b"
    ),
    set_prior("exponential(1)", class = "sigma")
  ),
  iter = 50000, warmup = 10000, cores = 4, chains = 4, thin = 20,
  silent = TRUE, refresh = 0,
  file = "./models/tarsusSynsacrumWidth.Rds"
)

p4 <- pp_check2(tarsusSynsacrumModel,
  xlab = "Max. Synsacrum Width\n(mm; Mean-Centred)"
) +
  theme(legend.position = "none")

p5 <- tarsusSynsacrumModel$data %>%
  mutate(
    "Residuals" =
      residuals(tarsusSynsacrumModel,
        type = "ordinary",
        robust = TRUE
      )[, "Estimate"]
  ) %>%
  ggplot(aes(x = Residuals)) +
```

```
  geom_density(
    colour = "black", fill = "lightblue",
    alpha = 0.5
  ) +
  xlab("Max. Synsacrum Width\nResiduals (mm)") +
  ylab("Density") +
  theme_classic()

p6 <- tarsusSynsacrumModel$data %>%
  mutate(
    "Residuals" =
      residuals(tarsusSynsacrumModel,
        type = "ordinary",
        robust = TRUE
      )[, "Estimate"]
  ) %>%
  ggplot(aes(x = tarsus, y = Residuals)) +
  geom_point(
    size = 2, pch = 21, colour = "black",
    fill = "lightblue", alpha = 0.5
  ) +
  xlab("Tarsus Length (mm)") +
  ylab("Max. Synsacrum Width\nResiduals (mm)") +
  theme_classic()

(p1 + p2 + p3)/
  (p4 + p5 + p6) +
  plot_annotation(tag_levels = "A")
```

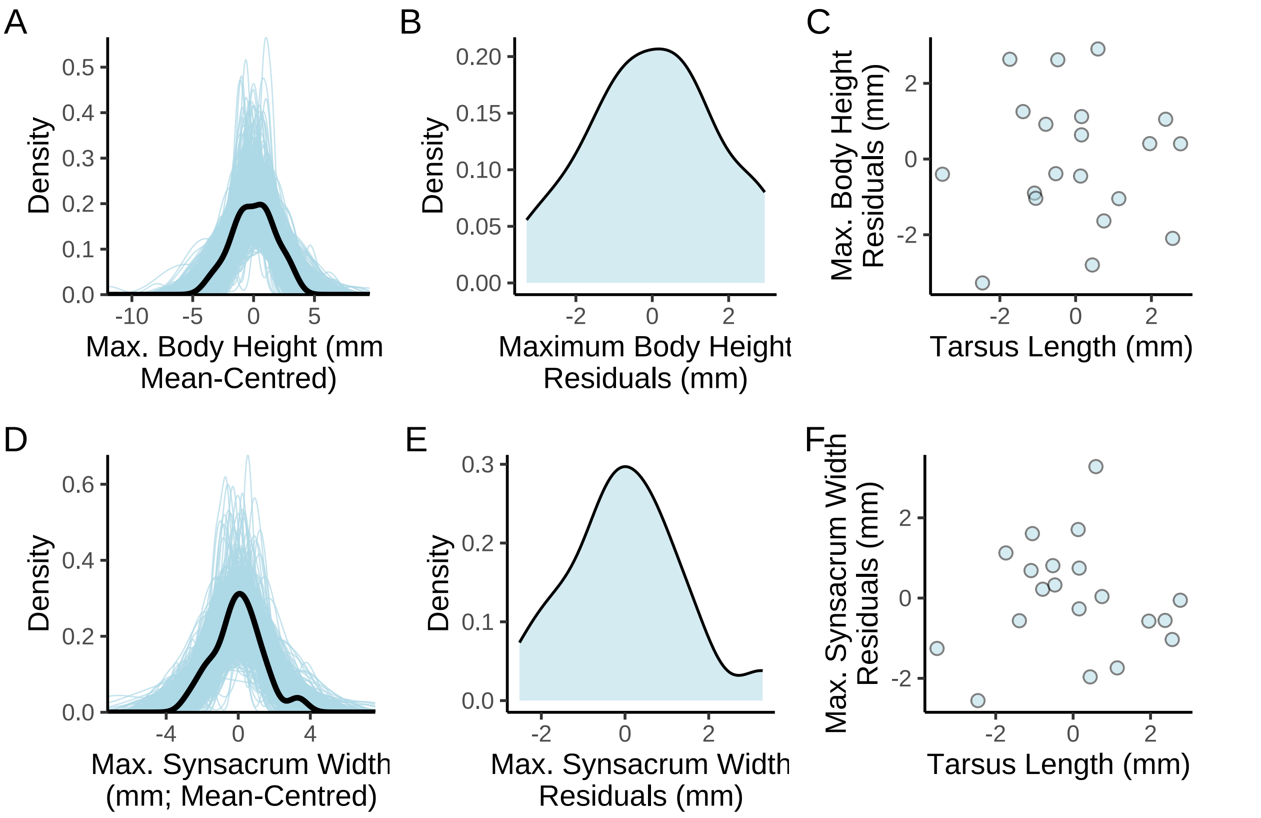

Figure 3: *Validations for linear models with tarsus length (mm) predicting maximum body height (mm) and maximum synsacrum width (mm) in twenty mature and deceased Japanese quail. Maximum body height represents the maximum vertical distance between the keel and back of feathered quail. Maximum synsacrum width represents that measured after dissection. Panels A and D represent posterior predictive checks, with black lines indicating true density of response variable values and light blue lines indicating estimated densities, as drawn from model posteriors. All other panels display spread (density in panels B and E) of model residuals.*

```
# Collating results

tarsus <- data.frame(
  "tarsusHeight" = as.data.frame(tarsusHeightModel)$b_tarsus,
  "tarsusSynsacrum" = as.data.frame(tarsusSynsacrumModel)$b_tarsus
) %>%
  summarise_all(., .funs = median) %>%
  pivot_longer(everything(),
    names_to = "Parameter",
    values_to = "Estimate"
  ) %>%
  merge(.,
    bind_rows(
      quantileCIs(tarsusHeightModel, cis = c(50, 95)) %>%
        mutate(Parameter = ifelse(Parameter == "b_tarsus", "tarsusHeight", Parameter)),
      quantileCIs(tarsusSynsacrumModel, cis = c(50, 95)) %>%
        mutate(Parameter = ifelse(Parameter == "b_tarsus", "tarsusSynsacrum", Parameter))
    ),
    by = "Parameter", all.x = TRUE
  ) %>%
  rowwise() %>%
  mutate("BF" = ifelse(Parameter == "tarsusHeight",
    ifelse(Estimate < 0,
      (2 * mean(as.data.frame(
        tarsusHeightModel
      )[, "b_tarsus"] <= 0)) /
```

```
        (2 * mean(as.data.frame(
          tarsusHeightModel
        )[, "b_tarsus"] >= 0)),
      (2 * mean(as.data.frame(
        tarsusHeightModel
      )[, "b_tarsus"] >= 0)) /
        (2 * mean(as.data.frame(
          tarsusHeightModel
        )[, "b_tarsus"] <= 0))
    ),
    ifelse(Estimate < 0,
      (2 * mean(as.data.frame(
        tarsusSynsacrumModel
      )[, "b_tarsus"] <= 0)) /
        (2 * mean(as.data.frame(
          tarsusSynsacrumModel
        )[, "b_tarsus"] >= 0)),
      (2 * mean(as.data.frame(
        tarsusSynsacrumModel
      )[, "b_tarsus"] >= 0)) /
        (2 * mean(as.data.frame(
          tarsusSynsacrumModel
        )[, "b_tarsus"] <= 0))
    )
  )) %>%
  ungroup() %>%
  mutate(
    "Response" = c("Body Height (mm)", "Synsacrum Width (mm)"),
    "Predictor" = "Tarsus Length (mm)",
    "Estimate" = round(Estimate, digits = 4),
    "BF" = round(BF, digits = 4),
    `50\\% CIs` = paste0(
      "[", round(Low_CI_50, digits = 4),
      ", ", round(High_CI_50, digits = 4),
      "]"
    ),
    `95\\% CIs` = paste0(
      "[", round(Low_CI_95, digits = 4),
      ", ", round(High_CI_95, digits = 4),
      "]"
    ),
    "N" = 20
  ) %>%
  dplyr::select(
    Predictor, Response, N, Estimate,
    `50\\% CIs`, `95\\% CIs`, BF
  )
```

```
# Body mass models

upperLimit <- with(
  structuralData,
  diff(range(bodyHeightMean, na.rm = T)) /
    diff(range(massMean, na.rm = T))
)

massHeightModel <- brm(
  data = structuralData %>%
    dplyr::select(
      "bodyHeight" = bodyHeightMean,
      "mass" = massMean
    ) %>%
    mutate(
      "bodyHeight" = bodyHeight -
        mean(bodyHeight, na.rm = T),
      "mass" = mass - mean(mass, na.rm = T)
    ),
  family = "gaussian",
```

```
  bodyHeight ~ mass,
  prior = c(
    set_prior("normal(0, 2)", class = "Intercept"),
    set_prior(paste0("normal(0, ", upperLimit / 2, ")"),
      class = "b"
    ),
    set_prior("exponential(1)", class = "sigma")
  ),
  iter = 50000, warmup = 10000, cores = 4,
  chains = 4, thin = 20,
  silent = TRUE, refresh = 0,
  file = "./models/massBodyHeightModel.Rds"
)

p1 <- pp_check2(massHeightModel,
  xlab = "Max. Body Height (mm;\nMean-Centred)"
) +
  theme(legend.position = "none")

p2 <- massHeightModel$data %>%
  mutate("Residuals" = residuals(massHeightModel,
    type = "ordinary", robust = TRUE
  )[, "Estimate"]) %>%
  ggplot(aes(x = Residuals)) +
  geom_density(colour = "black", fill = "lightblue", alpha = 0.5) +
  xlab("Max. Body Height\nResiduals (mm)") +
  ylab("Density") +
  theme_classic()

p3 <- massHeightModel$data %>%
  mutate("Residuals" = residuals(massHeightModel,
    type = "ordinary", robust = TRUE
  )[, "Estimate"]) %>%
  ggplot(aes(x = mass, y = Residuals)) +
  geom_point(
    size = 2, pch = 21, colour = "black",
    fill = "lightblue", alpha = 0.5
  ) +
  xlab("Body Mass (g)") +
  ylab("Max. Body Height\nResiduals (mm)") +
  theme_classic()

upperLimit <- with(
  structuralData,
  diff(range(synsacrumWidthMean, na.rm = T)) /
    diff(range(massMean, na.rm = T))
)

massSynsacrumModel <- brm(
  data = structuralData %>%
    dplyr::select(
      "synsacrumWidth" = synsacrumWidthMean,
      "mass" = massMean
    ) %>%
    mutate(
      "synsacrumWidth" = synsacrumWidth -
        mean(synsacrumWidth, na.rm = T),
      "mass" = mass - mean(mass, na.rm = T)
    ),
  family = "gaussian",
  synsacrumWidth ~ mass,
  prior = c(
    set_prior("normal(0, 2)", class = "Intercept"),
    set_prior(paste0(
      "normal(0, ",
      upperLimit / 2, ")"
    ), class = "b"),
```

```
    set_prior("exponential(1)", class = "sigma")
  ),
  iter = 50000, warmup = 10000, cores = 4,
  chains = 4, thin = 20,
  silent = TRUE, refresh = 0,
  file = "./models/massSynsacrumModel.Rds"
)

p4 <- pp_check2(massSynsacrumModel,
  xlab = "Max. Synsacrum Width\n(mm; Mean-Centred)"
) +
  theme(legend.position = "none")

p5 <- massSynsacrumModel$data %>%
  mutate(
    "Residuals" =
      residuals(massSynsacrumModel, type = "ordinary",
                robust = TRUE)[, "Estimate"]
  ) %>%
  ggplot(aes(x = Residuals)) +
  geom_density(colour = "black", fill = "lightblue", alpha = 0.5) +
  xlab("Max. Synsacrum Width\nResiduals (mm)") +
  ylab("Density") +
  theme_classic()

p6 <- massSynsacrumModel$data %>%
  mutate(
    "Residuals" =
      residuals(massSynsacrumModel, type = "ordinary",
                robust = TRUE)[, "Estimate"]
  ) %>%
  ggplot(aes(x = mass, y = Residuals)) +
  geom_point(
    size = 2, pch = 21, colour = "black",
    fill = "lightblue", alpha = 0.5
  ) +
  xlab("Body Mass (g)") +
  ylab("Max. Synsacrum Width\nResiduals (mm)") +
  theme_classic()

(p1 + p2 + p3)/
  (p4 + p5 + p6) +
  plot_annotation(tag_levels = "A")
```

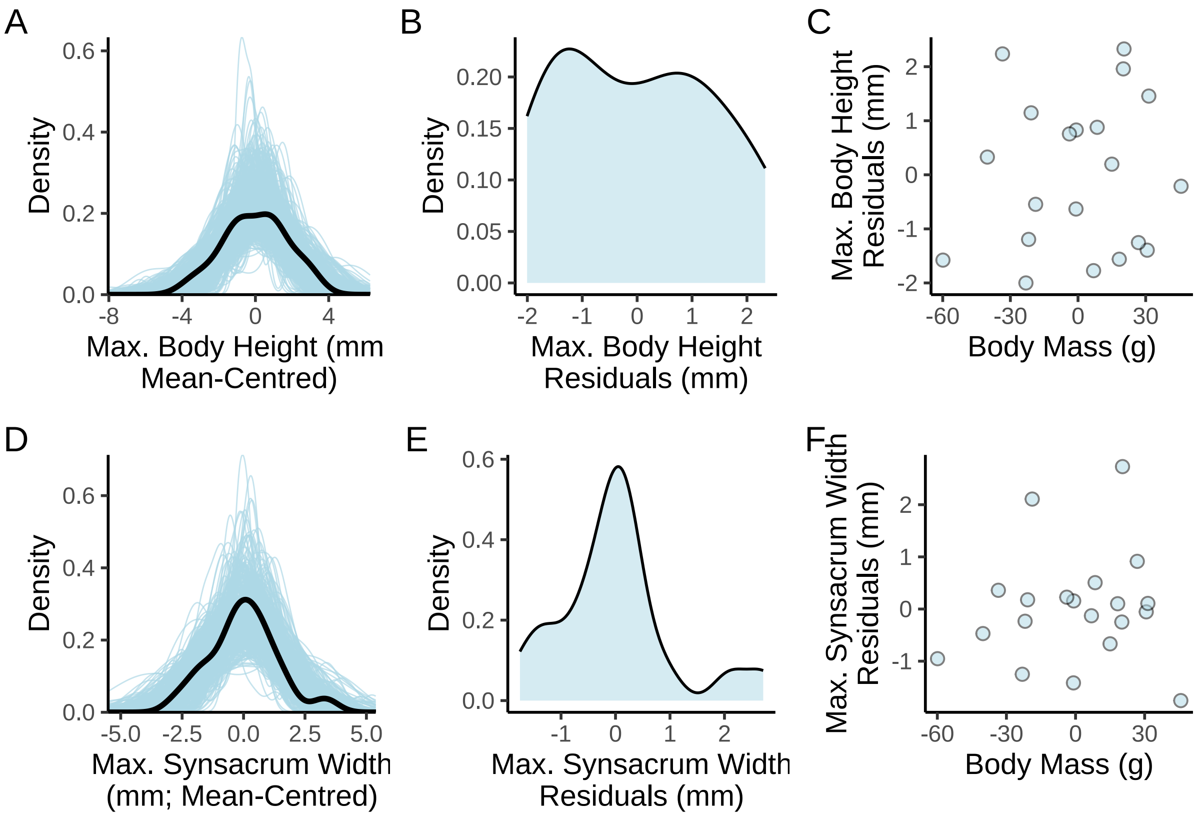

Figure 4: *Validations for linear models with body mass (g) predicting maximum body height (mm) and maximum synsacrum width (mm) in twenty mature and deceased Japanese quail. Maximum body height represents the maximum vertical distance between the keel and back of feathered quail. Maximum synsacrum width represents that measured after dissection. Panels A and D represent posterior predictive checks, with black lines indicating true density of response variable values and light blue lines indicating estimated densities, as drawn from model posteriors. All other panels display spread (density in panels B and E) of model residuals.*

```
# Again, collating results

mass <- data.frame(
  "massHeight" = as.data.frame(massHeightModel)$b_mass,
  "massSynsacrum" = as.data.frame(massSynsacrumModel)$b_mass
) %>%
  summarise_all(., .funs = median) %>%
  pivot_longer(everything(),
    names_to = "Parameter",
    values_to = "Estimate"
  ) %>%
  merge(.,
    bind_rows(
      quantileCIs(massHeightModel, cis = c(50, 95)) %>%
        mutate(Parameter = ifelse(Parameter == "b_mass", "massHeight", Parameter)),
      quantileCIs(massSynsacrumModel, cis = c(50, 95)) %>%
        mutate(Parameter = ifelse(Parameter == "b_mass", "massSynsacrum", Parameter))
    ),
    by = "Parameter", all.x = TRUE
  ) %>%
  rowwise() %>%
  mutate("BF" = ifelse(Parameter == "massHeight",
    ifelse(Estimate < 0,
      (2 * mean(as.data.frame(
        massHeightModel
      )[, "b_mass"] <= 0)) /
```

```
        (2 * mean(as.data.frame(
          massHeightModel
        )[, "b_mass"] >= 0)),
      (2 * mean(as.data.frame(
        massHeightModel
      )[, "b_mass"] >= 0)) /
        (2 * mean(as.data.frame(
          massHeightModel
        )[, "b_mass"] <= 0))
    ),
    ifelse(Estimate < 0,
      (2 * mean(as.data.frame(
        massSynsacrumModel
      )[, "b_mass"] <= 0)) /
        (2 * mean(as.data.frame(
          massSynsacrumModel
        )[, "b_mass"] >= 0)),
      (2 * mean(as.data.frame(
        massSynsacrumModel
      )[, "b_mass"] >= 0)) /
        (2 * mean(as.data.frame(
          massSynsacrumModel
        )[, "b_mass"] <= 0))
    )
  )) %>%
  ungroup() %>%
  mutate(
    "Response" = c("Body Height (mm)", "Synsacrum Width (mm)"),
    "Predictor" = "Body Mass (g)",
    "Estimate" = round(Estimate, digits = 4),
    "BF" = round(BF, digits = 4),
    `50\\% CIs` = paste0(
      "[", round(Low_CI_50, digits = 4),
      ", ", round(High_CI_50, digits = 4),
      "]"
    ),
    `95\\% CIs` = paste0(
      "[", round(Low_CI_95, digits = 4),
      ", ", round(High_CI_95, digits = 4),
      "]"
    ),
    "N" = 20
  ) %>%
  dplyr::select(
    Predictor, Response, N, Estimate,
    `50\\% CIs`, `95\\% CIs`, BF
  )

caption <- paste0(
  "Correlations between tarsus length (mm) ",
  "or body mass (g) and two measures of skeletal size ",
  "(maximum body height [mm] and maximum synsacrum width [mm]) ",
  "in twenty mature and deceased Japanese quail. ",
  "Results are derived from Bayesian linear models. ",
  "'CIs' indicate quantile-based ",
  "credible intervals, and 'BFs' indicate Bayes factors."
)

structuralSizeResults <- rbind(tarsus, mass) %>%
  kbl(format = "latex", escape = FALSE, caption = caption) %>%
  column_spec(column = c(1:2), width = "2.1cm") %>%
  column_spec(column = c(3:10), width = "1.9cm") %>%
  kable_styling(latex_options = "striped")

structuralSizeResults
```

```
# save_kable(structuralSizeResults,
# "../tables/structuralSizePredictionResults.html")
```

Table 2: *Correlations between tarsus length (mm) or body mass (g) and two measures of skeletal size (maximum body height [mm] and maximum synsacrum width [mm]) in twenty mature and deceased Japanese quail. Results are derived from Bayesian linear models. 'CIs' indicate quantile-based credible intervals, and 'BFs' indicate Bayes factors.*

Predictor	Response	N	Estimate	50% CIs	95% CIs	BF
Tarsus Length (mm)	Body Height (mm)	20	0.0804	[-0.0668, 0.2257]	[-0.3627, 0.5242]	1.8369
Tarsus Length (mm)	Synsacrum Width (mm)	20	0.0522	[-0.0732, 0.1735]	[-0.3193, 0.4153]	1.5690
Body Mass (g)	Body Height (mm)	20	0.0320	[0.0241, 0.0396]	[0.0087, 0.054]	194.1220
Body Mass (g)	Synsacrum Width (mm)	20	0.0291	[0.0229, 0.0348]	[0.01, 0.0464]	499.0000

```
# Body mass, but not tarsus length, is
# a good predictor of structural size in our quail.
```

Validating accuracy of digital appendage length measurements

Next, we confirm precision of our digital tarsus length and bill length measurements (mm) by comparing them against analogue measures of each in a subset of mature Japanese quail (n = 43). Comparisons are first done visually, then statistically by a simple Bayesian, measurement error model with analogue tarsus length or bill length as the Gaussian-distributed response and digital tarsus length or bill length as the sole population-level predictor with known measurement error as follows:

$$Analogue\ Tarsus\ Length_i \sim \beta_0 + \beta_1 * Digital\ Tarsus\ Length_{i*} + \epsilon_i$$
$$Digital\ Tarsus\ Length_{i*} \sim Digital\ Tarsus\ Length_i + \eta_i$$

or:

$$Analogue\ Bill\ Length_i \sim \beta_0 + \beta_1 * Digital\ Bill\ Length_{i*} + \epsilon_i$$
$$Digital\ Bill\ Length_{i*} \sim Digital\ Bill\ Length_i + \eta_i$$

where $Digital\ Tarsus\ Length_{i*}$ and $Digital\ Bill\ Length_{i*}$ represents the true but unknown digital tarsus and bill length measurements respectively, $Digital\ Tarsus\ Length_i$ and $Digital\ Bill\ Length_i$ represent mean digital tarsus length measurement or bill length measurements for an individual i, and η_i indicates the standard deviation of digital measurements for individual i.

Priors for these models were:

$$\beta_{0Tarsus} \sim \mathcal{N}(0, 5)$$
$$\beta_{0Bill} \sim \mathcal{N}(0, 2.5)$$

$$\beta_{1Tarsus} \sim N(1, 0.5)$$
$$\beta_{1Bill} \sim N(1, 0.5)$$

$$\epsilon_{iTarsus} \sim exponential(1)$$
$$\epsilon_{iBill} \sim exponential(2.5)$$

Below, we begin by displaying a representative image used for tarsus length calculation. Data are then subsequently loaded in and plotted.

```
plotJpeg <- function(path, add=FALSE) {
  image = jpeg::readJPEG(path, native=T)
  res = dim(image)[2:1]
  if (!add)
    plot(1, 1, xlim=c(1,res[1]),
    ylim=c(1,res[2]), asp=1,type='n',
    xaxs='i', yaxs='i',
    xaxt='n',yaxt='n',
    xlab='',ylab='',
    bty='n'
    )
  rasterImage(image, 1, 1, res[1], res[2])
}

plotJpeg("tarsusImage.jpg")
```

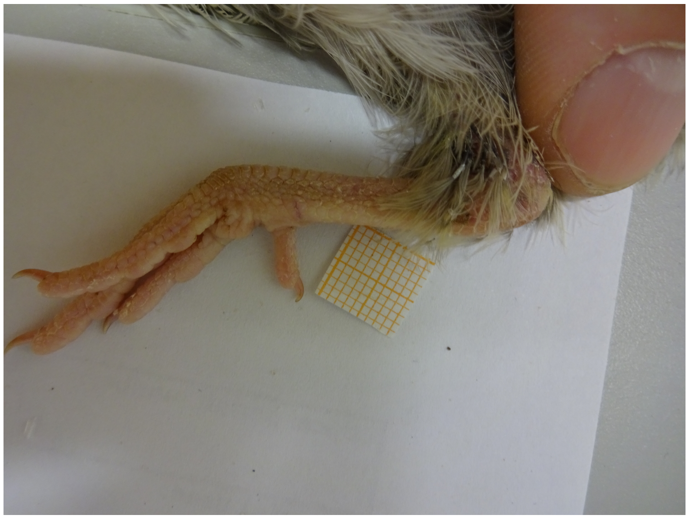

Figure 5: *Photo of a mature (8 weeks) Japaense quail tarsus with 1 mm x 1 mm grid paper used for digital tarsus length measurement.*

```
# Loading in data and correcting typo in header

comparisonData <-
  merge(read.csv("quailAnalogueMeasures20Weeks.csv"),
    read.csv("quailDigitalMeasurements20Weeks.csv"),
    by = "ring", all = TRUE
  ) %>%
  rename("tarsusLengthMean" = tarsusLengthhMean) %>%
  dplyr::select(
    ring, tarsusLength, tarsusLengthMean,
    tarsusLengthSD, tarsusLengthN,
    "billLength" = culmen, billLengthMean, billLengthSD, billLengthN
  ) %>%
  mutate(
    tarsusLengthSD = ifelse(is.na(tarsusLengthSD),
      mean(tarsusLengthSD, na.rm = T),
      tarsusLengthSD
    ),
    billLengthSD = ifelse(is.na(billLengthSD),
      mean(billLengthSD, na.rm = T),
      billLengthSD
    )
```

```
  )

# Plotting digital measures of tarsus length against analogue measures

p1 <- comparisonData %>%
  ggplot(aes(x = tarsusLengthMean, y = tarsusLength)) +
  geom_errorbarh(
    aes(
      xmin = tarsusLengthMean - tarsusLengthSD /
        sqrt(tarsusLengthN),
      xmax = tarsusLengthMean + tarsusLengthSD /
        sqrt(tarsusLengthN),
      y = tarsusLength
    ),
    colour = "black", height = 0.2
  ) +
  geom_point(size = 2.5, colour = "black", pch = 21,
             fill = "#90B1DB", alpha = 0.7) +
  theme_classic() +
  xlab("Digital Tarsus Length\nMeasurement (mm)") +
  ylab("Analogue Tarsus Length\nMeasurement (mm)")

p2 <- comparisonData %>%
  ggplot(aes(x = billLengthMean, y = billLength)) +
  geom_errorbarh(
    aes(
      xmin = billLengthMean - billLengthSD /
        sqrt(billLengthN),
      xmax = billLengthMean + billLengthSD /
        sqrt(billLengthN),
      y = billLength
    ),
    colour = "black", height = 0.2
  ) +
  geom_point(size = 2.5, colour = "black", pch = 21,
             fill = "#90B1DB", alpha = 0.7) +
  theme_classic() +
  xlab("Digital Bill Length\nMeasurement (mm)") +
  ylab("Analogue Bill Length\nMeasurement (mm)")

p1 + p2
```

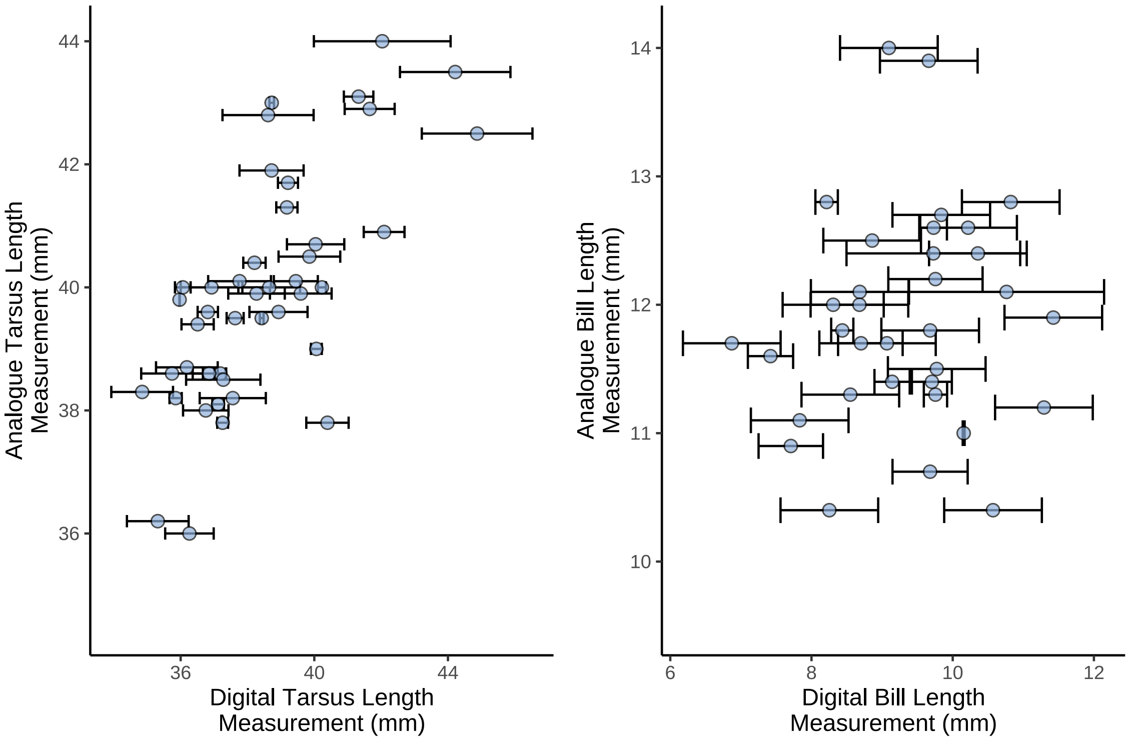

Figure 6: *Analogue tarsus length and bill length measurements as a function of their digital measurements from 43 mature Japanese quail. In most cases, digital tarsus and bill length were measured more than once per individual (range of measurements = 1-4) and dots represent means per individual. Horizontal errorbars represent +/- one standard error around means.*

```
# Modelling

tarsusModel <- brm(
  data = comparisonData %>%
    mutate(
      tarsusLength = tarsusLength -
        mean(tarsusLength, na.rm = T),
      tarsusLengthMean = tarsusLengthMean -
        mean(tarsusLengthMean, na.rm = T)
    ),
  family = "gaussian",
  tarsusLength ~ me(tarsusLengthMean, tarsusLengthSD),
  prior = c(
    set_prior("normal(0, 5)", class = "Intercept"),
    set_prior("normal(1, 0.5)", class = "b"),
    set_prior("exponential(1)", class = "sigma")
  ),
  iter = 50000, warmup = 10000, cores = 4, chains = 4, thin = 20,
  silent = TRUE, refresh = 0,
  file = "./models/_digitalTarsusValidation.Rds"
)

billModel <- brm(
  data = comparisonData %>%
    mutate(
      billLength = billLength -
        mean(billLength, na.rm = T),
      billLengthMean = billLengthMean -
        mean(billLengthMean, na.rm = T)
    ),
  family = "gaussian",
```

```
  billLength ~ me(billLengthMean, billLengthSD),
  prior = c(
    set_prior("normal(0, 2.5)", class = "Intercept"),
    set_prior("normal(1, 0.5)", class = "b"),
    set_prior("exponential(2.5)", class = "sigma")
  ),
  iter = 50000, warmup = 10000, cores = 4, chains = 4, thin = 20,
  silent = TRUE, refresh = 0,
  file = "./models/_digitalBillValidation.Rds"
)
```

We now proceed to simple model validations.

```
# Checking fit and residuals.

p1 <- pp_check2(tarsusModel,
  xlab = "Analogue Tarsus Length (mm)"
) + theme(legend.position = "none")

p2 <- tarsusModel$data %>%
  mutate(
    "Fit" = fitted(tarsusModel)[, "Estimate"],
    "SE" = fitted(tarsusModel)[, "Est.Error"]
  ) %>%
  ggplot(aes(x = Fit, y = tarsusLength)) +
  geom_errorbarh(aes(xmin = Fit - SE, xmax = Fit + SE)) +
  geom_point(
    pch = 21, colour = "black",
    fill = "lightblue", size = 2, alpha = 0.8
  ) +
  xlab("Fitted Tarsus\nLength(mm)") +
  ylab("Tarsus Length\n(mm)") +
  theme_classic()

p3 <- ggplot(
  data =
    data.frame("X" = c(brms::bayes_R2(tarsusModel, summary = FALSE))),
  aes(x = X)
) +
  geom_density(
    colour = "black",
    fill = "lightblue", alpha = 0.7
  ) +
  xlab(
    TeX("$R^{2}$")
  ) +
  ylab("Density") +
  theme_classic()

p4 <- tarsusModel$data %>%
  mutate("Residuals" = residuals(tarsusModel,
    robust = TRUE
  )[, "Estimate"]) %>%
  ggplot(aes(sample = Residuals)) +
  stat_qq(colour = "lightblue") +
  stat_qq_line() +
  xlab("Theoretical Residual\nQuantiles") +
  ylab("Sample Residual\nQuantiles") +
  theme_classic()

p5 <- tarsusModel$data %>%
  mutate("Residuals" = residuals(tarsusModel,
    robust = TRUE
  )[, "Estimate"]) %>%
  ggplot(aes(x = tarsusLengthMean, y = Residuals)) +
  geom_point(
    size = 2, pch = 21,
```

```
    colour = "black", fill = "lightblue", alpha = 0.5
  ) +
  xlab("Digital Tarsus Length\nMeasurement (mm)") +
  ylab("Ordinary Residuals") +
  theme_classic()

(p1 + p2) / (p3 + p4 + p5) +
  plot_annotation(tag_levels = "A")
```

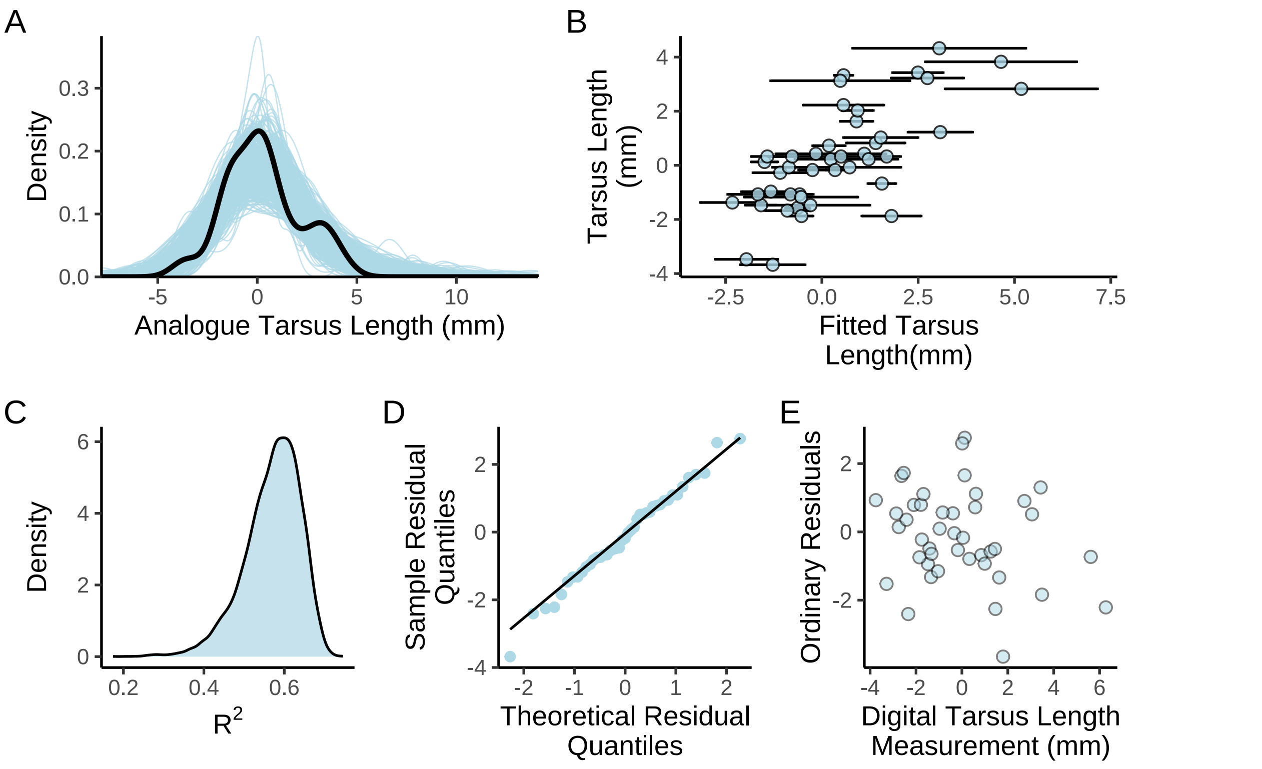

Figure 7: *Spread of fitted and residual values for a simple Bayesian regression predicting analogue tarsus length measurements as a function of digital tarsus length measurements. Panel A displays a posterior predictive check, with the density of true analogue tarsus length measurements indicated by a black line, and estimated densities (drawn from model posteriors) indicated by blue lines. Panel B displays raw analogue tarsus lengths measurements as a function of their fitted values (blue dots) +/- one standard error around fitted values. Panel C displays the esitmated* R^2 *density. Panels D and E display the distribution oresiduals from regression.*

```
# Summarising model.

caption <- paste0(
  "Results from a Bayesian linear model ",
  "predicting analogue tarsus length measurements ",
  "in mature Japanese quail. 'BF' indicates Bayes ",
  "Factor and R\\textsuperscript{2} indicates ",
  "a Bayesian R\\textsuperscript{2}."
)

digitalAnalogueTable <- as.data.frame(tarsusModel) %>%
  dplyr::select(bsp_metarsusLengthMeantarsusLengthSD) %>%
  summarise_all(., .funs = mean) %>%
  pivot_longer(everything(),
    names_to = "Parameter",
    values_to = "Mean"
  ) %>%
```

Table 3: *Results from a Bayesian linear model predicting analogue tarsus length measurements in mature Japanese quail. 'BF' indicates Bayes Factor and R^2 indicates a Bayesian R^2.*

Predictor	N	Estimate	50% CIs	95% CIs	BF	R^2
Digital Tarsus Length (mm)	43	0.7445	[0.6645, 0.8249]	[0.5089, 0.9913]	Inf	0.5697

```
  merge(., quantileCIs(tarsusModel, cis = c(50, 95)),
    by = "Parameter", all.x = TRUE
  ) %>%
  mutate("BF" = ifelse(Mean < 0,
    (2 * mean(as.data.frame(
      tarsusModel
    )[, Parameter] <= 0)) /
      (2 * mean(as.data.frame(
        tarsusModel
      )[, Parameter] >= 0)),
    (2 * mean(as.data.frame(
      tarsusModel
    )[, Parameter] >= 0)) /
      (2 * mean(as.data.frame(
        tarsusModel
      )[, Parameter] <= 0))
  )) %>%
   mutate(
    "Predictor" = "Digital Tarsus\nLength (mm)",
    "Estimate" = round(Mean, digits = 4),
    "BF" = round(BF, digits = 4),
    `50\\% CIs` = paste0(
      "[", round(Low_CI_50, digits = 4),
      ", ", round(High_CI_50, digits = 4),
      "]"
    ),
    `95\\% CIs` = paste0(
      "[", round(Low_CI_95, digits = 4),
      ", ", round(High_CI_95, digits = 4),
      "]"
    ),
     `R\\textsuperscript{2}` =
      round(brms::bayes_R2(tarsusModel)[, "Estimate"], digits = 4),
    "N" = nrow(tarsusModel$data)
  ) %>%
  dplyr::select(Predictor, N, Estimate,
         `50\\% CIs`, `95\\% CIs`,
         BF, `R\\textsuperscript{2}`
  ) %>%
  kbl(., format = "latex", escape = FALSE, caption = caption) %>%
  column_spec(column = c(1:10), width = "2cm") %>%
  kable_styling(latex_options = "striped")

digitalAnalogueTable
```

```
# Checking fit and residuals.

p1 <- pp_check2(billModel,
  xlab = "Analogue Bill Length (mm)"
) + theme(legend.position = "none")

p2 <- billModel$data %>%
  mutate(
    "Fit" = fitted(billModel)[, "Estimate"],
    "SE" = fitted(billModel)[, "Est.Error"]
```

```
  ) %>%
  ggplot(aes(x = Fit, y = billLength)) +
  geom_errorbarh(aes(xmin = Fit - SE, xmax = Fit + SE)) +
  geom_point(
    pch = 21, colour = "black",
    fill = "lightblue", size = 2, alpha = 0.8
  ) +
  xlab("Fitted Bill\nLength(mm)") +
  ylab("Bill Length\n(mm)") +
  theme_classic()

p3 <- ggplot(
  data =
    data.frame("X" = c(brms::bayes_R2(billModel, summary = FALSE))),
  aes(x = X)
) +
  geom_density(
    colour = "black",
    fill = "lightblue", alpha = 0.7
  ) +
  xlab(
    TeX("$R^{2}$")
  ) +
  ylab("Density") +
  theme_classic()

p4 <- billModel$data %>%
  mutate("Residuals" = residuals(billModel,
    robust = TRUE
  )[, "Estimate"]) %>%
  ggplot(aes(sample = Residuals)) +
  stat_qq(colour = "lightblue") +
  stat_qq_line() +
  xlab("Theoretical Residual\nQuantiles") +
  ylab("Sample Residual\nQuantiles") +
  theme_classic()

p5 <- billModel$data %>%
  mutate("Residuals" = residuals(billModel,
    robust = TRUE
  )[, "Estimate"]) %>%
  ggplot(aes(x = billLengthMean, y = Residuals)) +
  geom_point(
    size = 2, pch = 21,
    colour = "black", fill = "lightblue", alpha = 0.5
  ) +
  xlab("Digital Bill Length\nMeasurement (mm)") +
  ylab("Ordinary Residuals") +
  theme_classic()

(p1 + p2) / (p3 + p4 + p5) +
  plot_annotation(tag_levels = "A")
```

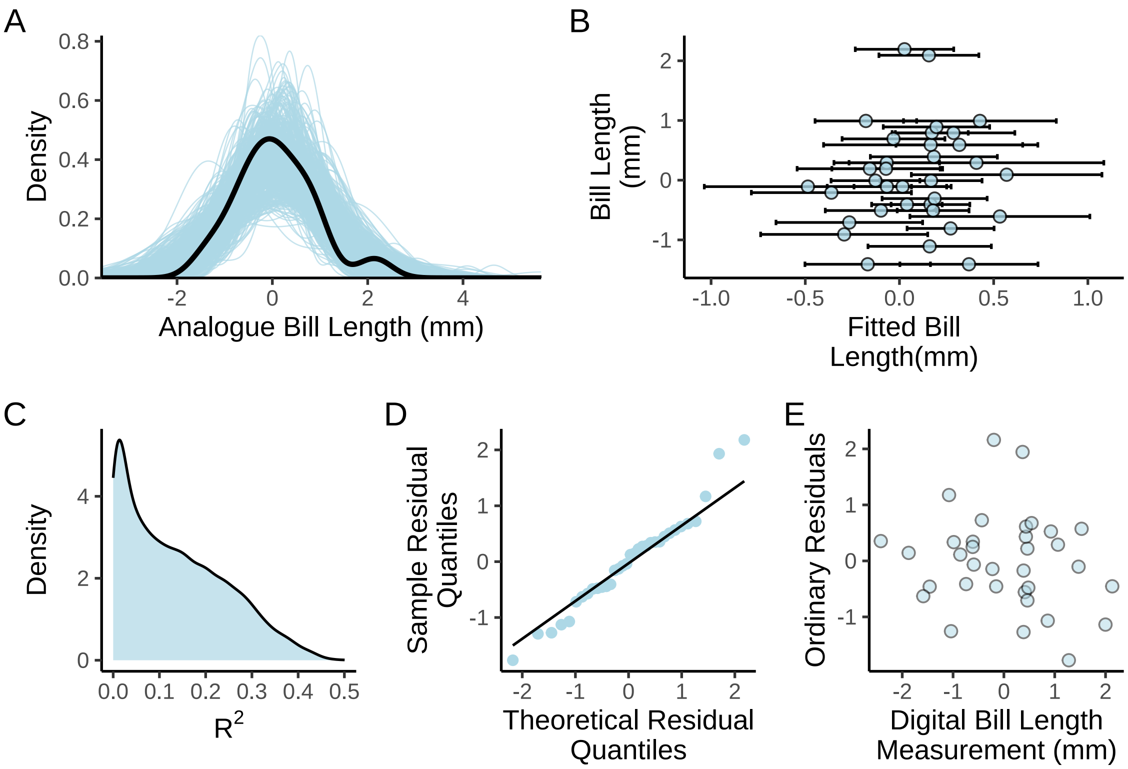

Figure 8: *Spread of fitted and residual values for aBayesian regression predicting analogue bill length measurements as a function of digital bill length measurements. Panel A displays a posterior predictive check, with the density of true analogue bill length measurements indicated by a black line, and estimated densities (drawn from model posteriors) indicated by blue lines. Panel B displays raw analogue bill lengths measurements as a function of their fitted values (blue dots) +/- one standard error around fitted values. Panel C displays the esitmated R^2 density. Panels D and E display the distribution oresiduals from regression.*

```
# Summarising model.

caption <- paste0(
  "Results from a Bayesian linear model ",
  "predicting analogue bill length measurements ",
  "in mature Japanese quail. 'BF' indicates Bayes ",
  "Factor and R\\textsuperscript{2} indicates ",
  "a Bayesian R\\textsuperscript{2}."
)

digitalAnalogueTableBill <- as.data.frame(billModel) %>%
  dplyr::select(bsp_mebillLengthMeanbillLengthSD) %>%
  summarise_all(., .funs = mean) %>%
  pivot_longer(everything(),
    names_to = "Parameter",
    values_to = "Mean"
  ) %>%
  merge(., quantileCIs(billModel, cis = c(50, 95)),
    by = "Parameter", all.x = TRUE
  ) %>%
  mutate("BF" = ifelse(Mean < 0,
    (2 * mean(as.data.frame(
      billModel
    )[, Parameter] <= 0)) /
      (2 * mean(as.data.frame(
        billModel
      )[, Parameter] >= 0)),
    (2 * mean(as.data.frame(
```

Table 4: *Results from a Bayesian linear model predicting analogue bill length measurements in mature Japanese quail. 'BF' indicates Bayes Factor and R^2 indicates a Bayesian R^2.*

Predictor	N	Estimate	50% CIs	95% CIs	BF	R^2
Digital Bill Length (mm)	34	0.2313	[0.0991, 0.36]	[-0.1555, 0.6381]	7.2474	0.134

```
      billModel
    )[, Parameter] >= 0)) /
      (2 * mean(as.data.frame(
        billModel
      )[, Parameter] <= 0))
  )) %>%
   mutate(
    "Predictor" = "Digital Bill\nLength (mm)",
    "Estimate" = round(Mean, digits = 4),
    "BF" = round(BF, digits = 4),
    `50\\% CIs` = paste0(
      "[", round(Low_CI_50, digits = 4),
      ", ", round(High_CI_50, digits = 4),
      "]"
    ),
    `95\\% CIs` = paste0(
      "[", round(Low_CI_95, digits = 4),
      ", ", round(High_CI_95, digits = 4),
      "]"
    ),
     `R\\textsuperscript{2}` =
      round(brms::bayes_R2(billModel)[, "Estimate"], digits = 4),
    "N" = nrow(billModel$data)
  ) %>%
  dplyr::select(Predictor, N, Estimate,
         `50\\% CIs`, `95\\% CIs`,
         BF, `R\\textsuperscript{2}`
  ) %>%
  kbl(., format = "latex", escape = FALSE, caption = caption) %>%
  column_spec(column = c(1:10), width = "2cm") %>%
  kable_styling(latex_options = "striped")

digitalAnalogueTableBill
```

2.0 Data organisation and analysis of growth

Overview

In this document, we compile morphological data from Japanese quail (n = 108) reared across three distinct thermal treatments: (1) "cold" (10°C for 3-8 weeks of age, followed by a constant 20°C), (2) "mild" (20°C until 8 weeks of age), and (3) "warm" (30°C for 3-8 weeks of life, followed by a constant 20°C). Morphological measurements taken include body mass (g) and tarsus length (mm), with mass measured weekly from 0-8 weeks of age, and tarsus length measured weekly between 0-3 weeks of age, then again at 8 weeks of age. A full description of measurement methodology is provided in the 'Methods' of Tabh et al (2025). Once compiled, we proceed by visualising these data for oddities or erroneous values. Then, to test for an effect of rearing temperature treatment on patterns of growth and adulthood phenotypes (here, 8 weeks of age), we follow by building analytical models, scrutinising these models, and visualising outcomes.

Data compilation

Below, we begin by importing all packages and functions required for data compilation, visualisation, and analysis. Versions of R packages loaded into our R session are printed to facilitate reproduction. We recommend that those seeking to reproduce our models and results use the versions reported before proceeding.

```
library("tidyverse")

# 'easypackages' is used to load all other packages in with one simple execution.

library("easypackages")

packageList <- c("bayesplot", "brms", "doParallel",
                 "foreach", "ggpubr", "kableExtra",
                 "latex2exp", "patchwork", "priorsense",
                 "showtext", "tidybayes", "wesanderson")

libraries(packageList)

# R methods from the 'brms' packages that allow for the
# addition of priors in hypothesis tests are loaded from
# a local source (available on GitHub). This first
# requires installation of the dependent package "mgsub".

#install.packages("mgsub")
#install.packages("/Users/joshuatabh/rPackageDevelopment/brmsMethods",
  #repos = NULL, type = "source")

library("brmsMethods")

# Printing package version numbers

caption <- paste0("R packages and their respective versions used for",
                  " data organisation and analysis in this study."
)

sapply(packageList, function(x) {
  y <- as.character(packageVersion(x))
  return(y)
}, simplify = FALSE) %>%
  enframe(., name = "Package", value = "Version") %>%
  as.data.frame(.) %>%
  kbl(.,
    longtable = T, booktabs = T,
    caption = caption
  ) %>%
  kable_styling(latex_options = "striped")
```

Table 5: *R packages and their respective versions used for data organisation and analysis in this study.*

Package	Version

bayesplot	1.11.1
brms	2.22.7
doParallel	1.0.17
foreach	1.5.2
ggpubr	0.6.0
kableExtra	1.4.0
latex2exp	0.9.6
patchwork	1.2.0
priorsense	1.0.2
showtext	0.9.7
tidybayes	3.0.6
wesanderson	0.3.7

```
# Adding custom functions

## A function to calculate the mode of a vector

md <- function(x) {
  all_values <- unique(x)
  all_values[which.max(tabulate(match(x, all_values)))]
}

# A function to cleanly view autocorrelation between posterior
# draws of specified coefficients/variables

clean_ac <- function(x, prs = NA, names = NA) {
  require("rstan")

  if (class(x)[1] != "brmsfit") {
    return("x must be a brmsfit object.")
  }

  if (is.na(prs[1])) {
    return(stan_ac(x$fit))
  }

  if (!is.na(prs[1]) & is.na(names[1])) {
    return(stan_ac(x$fit, pars = prs))
  }

  if (length(prs) != length(names)) {
    return("Length of pars and names must be equal.")
  }

  Base_plot <- stan_ac(x$fit, pars = prs, fill = nice_pink)
  Base_plot$data$parameters <- as.character(Base_plot$data$parameters)

  for (i in 1:length(prs)) {
    Base_plot$data$parameters[c(which(Base_plot$data$parameters == prs[i]))] <-
      names[i]
  }
  Base_plot$data$parameters <- as.factor(Base_plot$data$parameters)

  return(Base_plot)
}

# A function to simplify the output of bayestestR's hdi function.

simple_hdi <- function(x, rnd = 3, cis = c(50, 95), sci_note = FALSE) {
  if (class(x)[1] != "brmsfit") {
    return("x must be a brmsfit object.")
  }
  if (length(cis) != 2) {
    return("cis must be a vector of integers with length 2")
  }
```

```
  HDI_low <- bayestestR::hdi(x, effects = "all", ci = min(cis) / 100)
  HDI_high <- bayestestR::hdi(x, effects = "all", ci = max(cis) / 100)

  if (sci_note == FALSE) {
    Results <- data.frame(
      "Parameter" = HDI_low$Parameter,
      "1" = round(HDI_low$CI_low, rnd),
      "2" = round(HDI_low$CI_high, rnd),
      "3" = round(HDI_high$CI_low, rnd),
      "4" = round(HDI_high$CI_high, rnd)
    )
    colnames(Results)[c(2:5)] <-
      c(paste0("Low_HDI_", min(cis)), paste0("High_HDI_", min(cis)),
        paste0("Low_HDI_", max(cis)), paste0("High_HDI_", max(cis)))
  } else if (sci_note == TRUE) {
    Results <- data.frame(
      "Parameter" = HDI_low$Parameter,
      "1" = format(round(HDI_low$CI_low, rnd), scientific = TRUE),
      "2" = format(round(HDI_low$CI_high, rnd), scientific = TRUE),
      "3" = format(round(HDI_high$CI_low, rnd), scientific = TRUE),
      "4" = format(round(HDI_high$CI_high, rnd), scientific = TRUE)
    )
    colnames(Results)[c(2:5)] <- c(paste0("Low_HDI_", min(cis)),
                                   paste0("High_HDI_", min(cis)),
                                   paste0("Low_HDI_", max(cis)),
                                   paste0("High_HDI_", max(cis)))
  }

  return(Results)
}

modeHDI <- function(x, cis = c(50, 95), rnd = 4, collapse = FALSE){
  stopifnot("x must be a 'brmsfit' object" = class(x) == "brmsfit",
            "collapse must be logical TRUE/FALSE" = is.logical(collapse))

  out <- lapply(X = as.data.frame(x), MARGIN = 2, FUN = ggdist::mode_hdi,
                .width = c(cis/100))
  hold <- names(out)
  out <- out %>%
    map2(hold, ~mutate(.x, name = .y)) %>%
    bind_rows() %>%
    mutate(y = round(y, digits = rnd),
           ymin = round(ymin, digits = rnd),
           ymax = round(ymax, digits = rnd)) %>%
    select("par" = name, "mode" = y, "lcl" = ymin,
           "ucl"= ymax, "confidenceLevel" = .width)

  if (collapse == FALSE){
    return(out)
  } else if (collapse == TRUE){
    out <- out %>%
      mutate("cis" = paste0("[", lcl, ", ", ucl, "]")) %>%
      select(-c(lcl, ucl))

    return(out)
  }
}


# A function to calculate quantile intervals from a brmsfit object

quantileCIs <- function(x, rnd = 3, cis = c(50, 95), sci_note = FALSE) {
  require(tidyverse)

  if (class(x)[1] != "brmsfit") {
    return("x must be a brmsfit object.")
  }
```

```
  if (length(cis) != 2) {
    return("cis must be a vector of integers with length 2")
  }

  prbs = c()
  nColNames = c()
  for (i in 1:length(cis)){
    prbs = c(prbs, c(0.5 - (cis[i]/100)/2, 0.5 + (cis[i]/100)/2))
    nColNames = c(nColNames,
                  paste0("Low_CI_", cis[i]),
                  paste0("High_CI_", cis[i])
    )
  }

  modelFrame = as.data.frame(x)

  Results <- apply(modelFrame, MARGIN = 2, FUN = quantile,
        probs = prbs, type = 8) %>%
    t() %>%
    as.data.frame() %>%
    rownames_to_column(var = "par") %>%
    `colnames<-`(c("Parameter", nColNames))

  if (sci_note == FALSE) {
    Results <- apply(modelFrame, MARGIN = 2, FUN = quantile,
        probs = prbs, type = 8) %>%
    t() %>%
    as.data.frame() %>%
    rownames_to_column(var = "par") %>%
    `colnames<-`(c("Parameter", nColNames))

  } else if (sci_note == TRUE) {
    Results <- apply(modelFrame, MARGIN = 2, FUN = quantile,
        probs = prbs, type = 8) %>%
    t() %>%
    as.data.frame() %>%
    rownames_to_column(var = "par") %>%
    `colnames<-`(c("Parameter", nColNames)) %>%
    mutate_at(.vars = vars(-Parameter),
              .funs = function(x){
                return(format(x, scientific = TRUE))
              }
    )
  }

  return(Results)
}

# A function that allows users to assign multiple objects
# to different variable names at once. The below is reported by "ellbur" at
# https://strugglingthroughproblems.wordpress.com/author/ellbur/page/3/.

{
  "%=%" <- function(l, r, ...) UseMethod("%=%")

  "%=%.lbunch" <- function(l, r, ..., List = NA) {
    Envir <- as.environment(-1)

    if (!is.na(List)) {
      l <- List[[1]]
      r <- List[[2]]
    }

    if (length(r) > length(l)) {
      warning("RHS has more args than LHS. Only first",
              length(l), "used.")
    }
```

```
    if (length(l) > length(r)) {
      warning("LHS has more args than RHS. RHS will be repeated.")
      r <- extendToMatch(r, l)
    }

    for (II in 1:length(l)) {
      do.call("<-", list(l[[II]], r[[II]]), envir = Envir)
    }
  }

  extendToMatch <- function(source, destin) {
    s <- length(source)
    d <- length(destin)

    if (d == 1 && s > 1 && !is.null(as.numeric(destin))) {
      d <- destin
    }

    dif <- d - s
    if (dif > 0) {
      source <- rep(source, ceiling(d / s))[1:d]
    }
    return(source)
  }

  g <- function(...) {
    List <- as.list(substitute(list(...)))[-1L]
    class(List) <- "lbunch"
    return(List)
  }
}

# A function to simplify output of bayes_R2 from brms

simpleR2 <- function(x, ndraws = 1000, roundDigits = 5,
                     robust = TRUE) {
  stopifnot("x must be a 'brmsfit' object" = class(x) == "brmsfit",
            "robust must be logical (TRUE/FALSE)" = is.logical(robust))
  grab <- brms::bayes_R2(x, ndraws = ndraws, robust = robust)
  toPrint <- paste0(
    "R2 = ", round(grab[, "Estimate"], digits = roundDigits),
    " [",
    round(grab[, "Q2.5"], digits = roundDigits),
    ",",
    round(grab[, "Q97.5"], digits = roundDigits),
    "]"
  )
  cat(toPrint)
}

# A function to calculate the position of a skew-normal
# distribution given its mean, omega, and alpha values

skewxi <- function(mean, omega, alpha) {
  delta <- alpha / (sqrt(1 + alpha^2))
  xi <- mean - omega * delta * sqrt(2 / pi)
  return(xi)
}

# A function to produce clean posterior or prior
# predictive checks, based upon "pp_check" from the R-package 'brms'.

pp_check2 <- function(model, resp = NA, ndraws = 500,
                      xlab = "label", colour = "lightblue") {
  require(brms)
  require(ggplot2)
  stopifnot("Model must be a brmsfit object" = is.brmsfit(model))
```

```
  if (is.na(resp)) {
    resp <- model$formula$resp
  }

  p1 <- brms::pp_check(model, ndraws = ndraws, resp = resp) +
    scale_colour_manual(
      values = c("black", colour),
      labels = c("y", "yhat"),
      name = NULL
    ) +
    xlab(xlab) +
    ylab("Density") +
    theme_classic()
  return(p1)
}

# A function to summarise and print Gelman-Rubin statistics and effective
# sample sizes to sample sizes for brmsfit objects.

neffBase <- function(x){
  stopifnot("Model must be a brmsfit object" = is.brmsfit(x))
  out <- as.data.frame(brms::neff_ratio(x)) %>%
    rownames_to_column(var = "var") %>%
    filter(!(var %in% c("lprior", "lp__"))) %>%
    pull(.)
  return(out)
}

chainCheck <- function(model, rDig = 3) {
  require(brms)
  stopifnot("Model must be a brmsfit object" = is.brmsfit(model))

  Rhat <- paste0(
    "Rhat range: ",
    round(min(rhat(model)), digits = rDig),
    " - ",
    round(max(rhat(model)), digits = rDig)
  )
  Neff <- paste0(
    "Neff/N range: ",
    round(min(neffBase(model)), digits = rDig),
    " - ",
    round(max(neffBase(model)), digits = rDig)
  )
  cat(paste0(Rhat, "\n", Neff))
}

# Setting working directory. Note that this Rmarkdown
# file should be placed with the below defined folder
# for scripts to correctly identify other folder and
# file paths referenced in the remaining code.

setwd("/Users/joshuatabh/analyses")
```

Next, data are imported and bound.

```
data <- rbind(
  read.csv("exp1Morphology.csv") %>%
    mutate("exp" = "A"),
  read.csv("exp2Morphology.csv") %>%
    mutate("exp" = "B"),
  read.csv("exp3Morphology.csv") %>%
    mutate("exp" = "C")
) %>%
  mutate(
    sex = tolower(sex),
```

```
    treatment = tolower(treatment),
    pretreatment = tolower(pretreatment),
    posttreatment = tolower(posttreatment)
  )
```

In this study, tarsus and bill length measurements were derived from digital photographs and calibrated to standardised grids within view (see methods of Tabh et al, 2025, for complete details). In some cases, calibration methods varied (e.g. calibration grid layed above or below the subject). To ensure that these variations did not bias our estimates of true appendage lengths, we first modelled appendage length (± its standard deviation when multiple photos were captured per individual) as a function of age (here, categorical) and calibration method, then used mean differences in length measures per calibration method (derived from our model) to correct raw measurement values accordingly. Before modelling, however, raw tarsus and bill length measurements are plotted by age to check for erroneous values that may bias model outcomes.

```
# Checking for aberrant, raw tarsus and bill length measurements

p1 <- data %>%
  filter(week < 10) %>%
  ggplot(aes(x = week, y = tarsusLengthMean)) +
  geom_point(size = 2, pch = 21, fill = "slateblue", colour = "black",
             position = position_jitter(width = 0.5), alpha = 0.3) +
  stat_summary(geom = "errorbar", fun.data = "mean_cl_boot",
               colour = "black", width = 0.3) +
  stat_summary(geom = "point", fun = "mean", size = 3, pch = 21,
               colour = "black", fill = "black") +
  scale_x_continuous(breaks = c(0:8)) +
  theme_classic() +
  xlab("Age (weeks)") +
  ylab("Tarsus Length (mm)")

p2 <- data %>%
  filter(week < 10) %>%
  ggplot(aes(x = week, y = billLengthMean)) +
  geom_point(size = 2, pch = 21, fill = "slateblue", colour = "black",
             position = position_jitter(width = 0.2), alpha = 0.3) +
  stat_summary(geom = "errorbar", fun.data = "mean_cl_boot",
               colour = "black", width = 0.3) +
  stat_summary(geom = "point", fun = "mean", size = 3, pch = 21,
               colour = "black", fill = "black") +
  scale_x_continuous(breaks = c(0:8)) +
  theme_classic() +
  xlab("Age (weeks)") +
  ylab("Bill Length (mm)")

p1 + p2
```

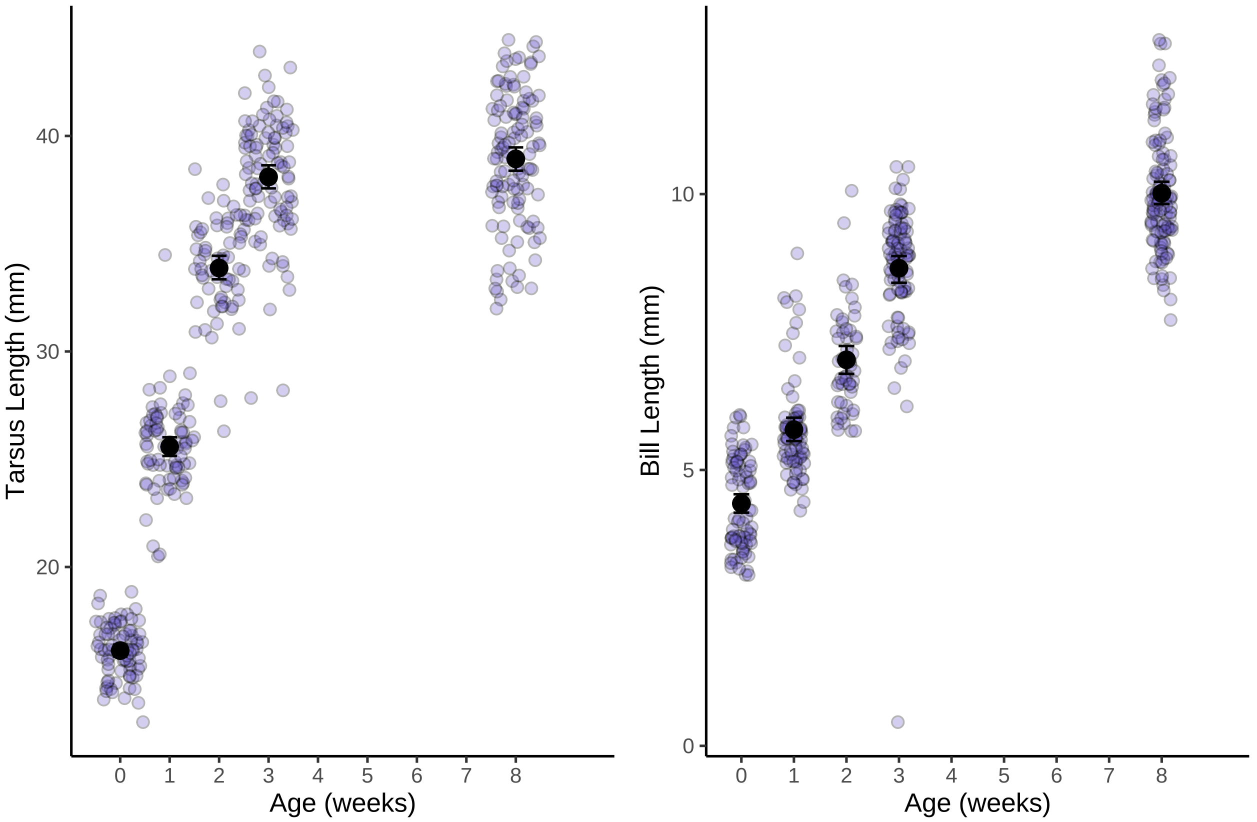

Figure 9: *Effect of post-hatch age on tarsus length (mm) and bill length (mm) in Japanese quail. Black dots represent means and errorbars indicate standard errors; purple dots represent raw values.*

One erroneous bill length measurement (falling below 5 mm) is removed.

```
data <- filter(data, billLengthMean >= 1 | is.na(billLengthMean))
```

We proceed by first constructing our model predicting tarsus length by age and calibration method. Here, flat priors for population level slopes are used, while for our intercept, we use a student-t distributed prior with three degrees of freedom, μ of 35.8 and σ of 6.9.

```
# Adjusting for calibration method in tarsus length
# measurements then replotting. Note that this is achieved
# by modelling mean appendage length (mm; as derived from
# multiple digital photographs per individual) as a function
# of age (weeks; factorial) and calibration method (categorical).
# Uncertainty around mean appendage length is also accounted
# for within these models. All missing values for tarsus
# length measurements are first removed.

tarsusCorrection <- brm(tarsusLengthMean |
                          se(tarsusLengthSD, sigma = TRUE) ~
                          week + tarsusCalibration,
  data = data %>%
    filter(!(is.na(tarsusLengthMean))) %>%
    mutate(
      week = factor(week),
      tarsusLengthSD = ifelse(is.na(tarsusLengthSD),
                              0.001,
                              tarsusLengthSD)
    ),
```

```
  iter = 50000, warmup = 5000, cores = 4, chains = 4,
  inits = 0,
  control = list(adapt_delta = .95),
  silent = TRUE, refresh = 0,
  file = "./models/_tarsusCorrectionModel.Rds"
)

## Checking distribution of calibration coefficients,
# distribution of posterior predictions and leave-one-out "r2".

tarsusCorrection %>%
  as.data.frame() %>%
  mutate(b_Intercept = b_Intercept - mean(b_Intercept)) %>%
  dplyr::select(
    "Grid over" = b_Intercept,
    "Grid under" = b_tarsusCalibrationgridUnder,
    "Ruler under" = b_tarsusCalibrationrulerUnder
  ) %>%
  pivot_longer(everything(),
    names_to = "method",
    values_to = "effect"
  ) %>%
  ggplot(aes(x = effect)) +
  facet_wrap(~method, scales = "free") +
  geom_vline(xintercept = 0, linetype = "dashed", colour = "black") +
  geom_density(colour = "black", fill = "grey50", alpha = 0.5) +
  theme_classic() +
  xlab("Mean Effect of Tarsus Length Estimate (mm)") +
  ylab("Density")
```

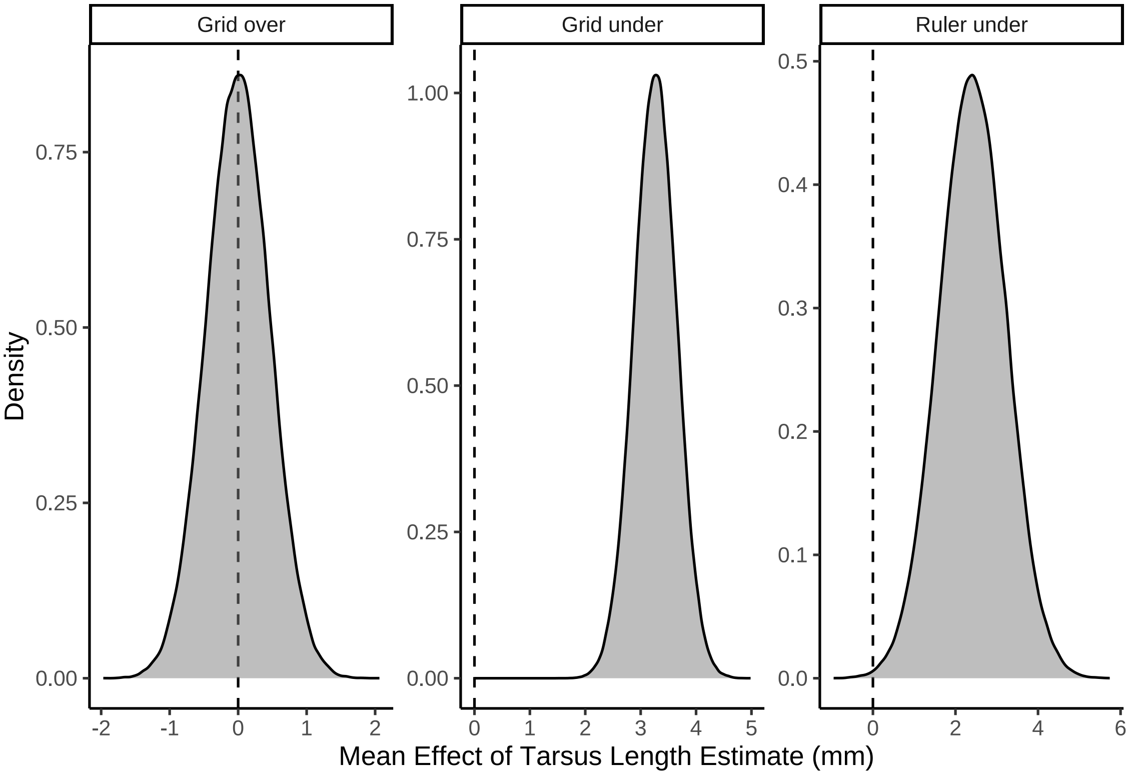

Figure 10: *Effects (model coefficients) of measurement calibration method on tarsus length estimate in Japanese quail. Distributions represent posterior densities from a Bayesian, linear model with tarsus length (mm) as the response variable and age (weeks) and calibration method as predictors.*

Posteriors of our model are briefly visualised, a Bayesian R^2 estimated (here, using posterior median as a measure of centrality) and effects of calibration method displayed.

```
# Clear effects of calibration method.

pp_check2(tarsusCorrection, xlab = "Tarsus Length (mm)")
```

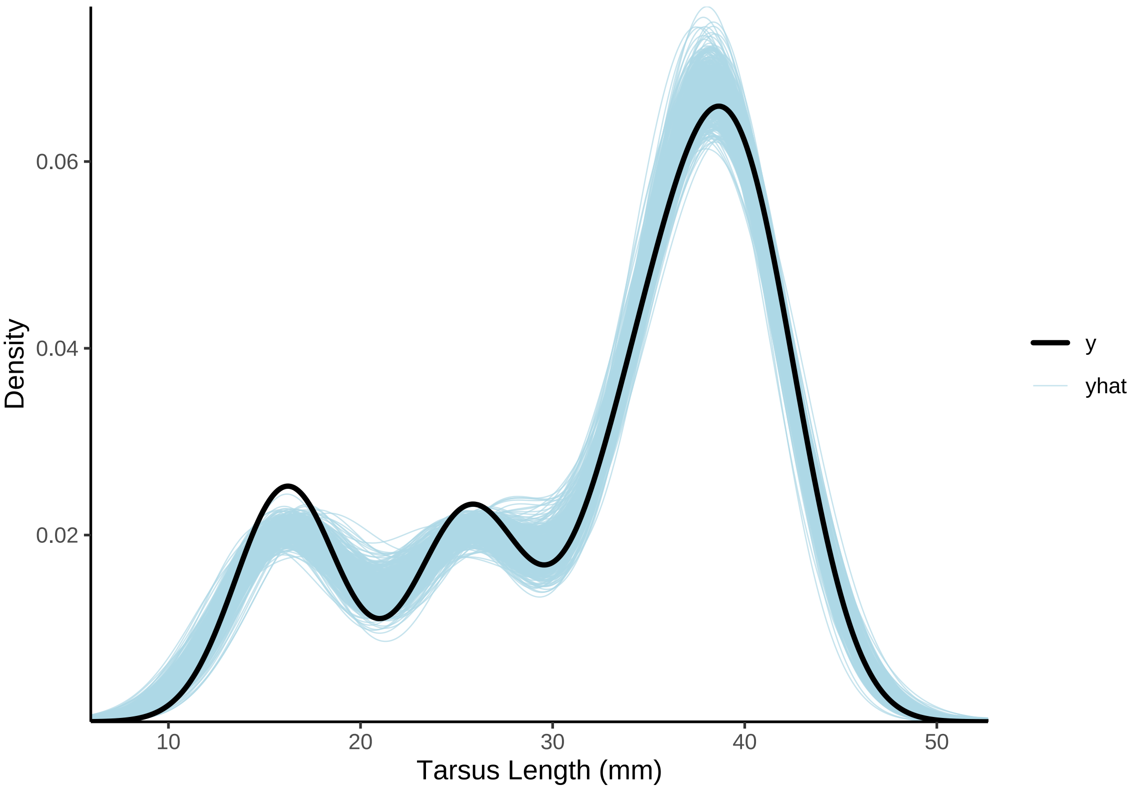

Figure 11: *Density of posterior draws from a Bayesian linear mixed effects model predicting tarsus length in Japanese quail (mm; draws in grey). Density is overlayed with that of true tarsus length measurements (mm; line in black).*

```
simpleR2(tarsusCorrection, robust = TRUE)
```

```
## R2 = 0.93796 [0.9345,0.94058]
```

```
## Normal and remarkably high R2. Proceeding to calculate
# means, and errors around means, by quantiles

tarsusDeltas <- as.data.frame(tarsusCorrection) %>%
  summarise(
    "gridUnder" = mean(b_tarsusCalibrationgridUnder),
    "gridUnderLCL" = quantile(b_tarsusCalibrationgridUnder,
                              0.025, type = 8),
    "gridUnderUCL" = quantile(b_tarsusCalibrationgridUnder,
                              0.975, type = 8),
    "rulerUnder" = mean(b_tarsusCalibrationrulerUnder),
    "rulerUnderLCL" = quantile(b_tarsusCalibrationrulerUnder,
                               0.025, type = 8),
    "rulerUnderUCL" = quantile(b_tarsusCalibrationrulerUnder,
                               0.975, type = 8)
  )

caption <- paste0("Estimated effect of measurement calibration ",
                  "method on tarsus length estimates (mm; posterior means) ",
```

Table 6: *Estimated effect of measurement calibration method on tarsus length estimates (mm; posterior means) across ages in Japanese quail. Credible invervals (2.5 and 97.5%) are in braces.*

Calibration Method	Effect on tarsus length estimate (mm)
Grid Under	3.26472 [2.50613,4.02009]
Ruler Under	2.4127 [0.82261,4.00255]

```
                  "across ages in Japanese quail. Credible invervals ",
                  "(2.5 and 97.5\\%) are in braces."
)

tarsusDeltas %>%
  mutate(
    "Grid Under" = paste0(
      round(gridUnder, digits = 5),
      " [",
      round(gridUnderLCL, digits = 5),
      ",",
      round(gridUnderUCL, digits = 5),
      "]"
    ),
    "Ruler Under" = paste0(
      round(rulerUnder, digits = 5),
      " [",
      round(rulerUnderLCL, digits = 5),
      ",",
      round(rulerUnderUCL, digits = 5),
      "]"
    )
  ) %>%
  select(`Grid Under`, `Ruler Under`) %>%
  pivot_longer(everything(), names_to = "Calibration Method",
               values_to = "Effect on tarsus length estimate (mm)") %>%
  kbl(., format = "latex", caption = caption, escape = FALSE) %>%
  kable_styling()
```

```
rm(tarsusCorrection)

## Correcting with these means

data <- data %>%
  mutate("tarsusLengthMeanAdjusted" =
           ifelse(tarsusCalibration == "gridUnder",
                  tarsusLengthMean - tarsusDeltas$gridUnder,
                  ifelse(tarsusCalibration == "rulerUnder",
                         tarsusLengthMean - tarsusDeltas$rulerUnder,
                         tarsusLengthMean)
                  )
         )
```

We last check for measurement oddities in light of corrections.

```
ggplot(data %>%
         filter(week %in% c(0:3, 8)) %>%
         group_by(week) %>%
         mutate("ID" = 1:n()) %>%
         ungroup() %>%
         mutate("week" = ifelse(week == 1,
                                paste0("Age = ", week, " week"),
                                paste0("Age = ", week, " weeks"))
                ),
       aes(x = ID, y = tarsusLengthMeanAdjusted)) +
  facet_wrap(~week, scales = "free") +
  geom_point(size = 2, colour = "black",
```

```
            fill = "slateblue", alpha = 0.7) +
  theme_classic() +
  xlab("Sample Number") +
  ylab("Tarsus Length (mm)")
```

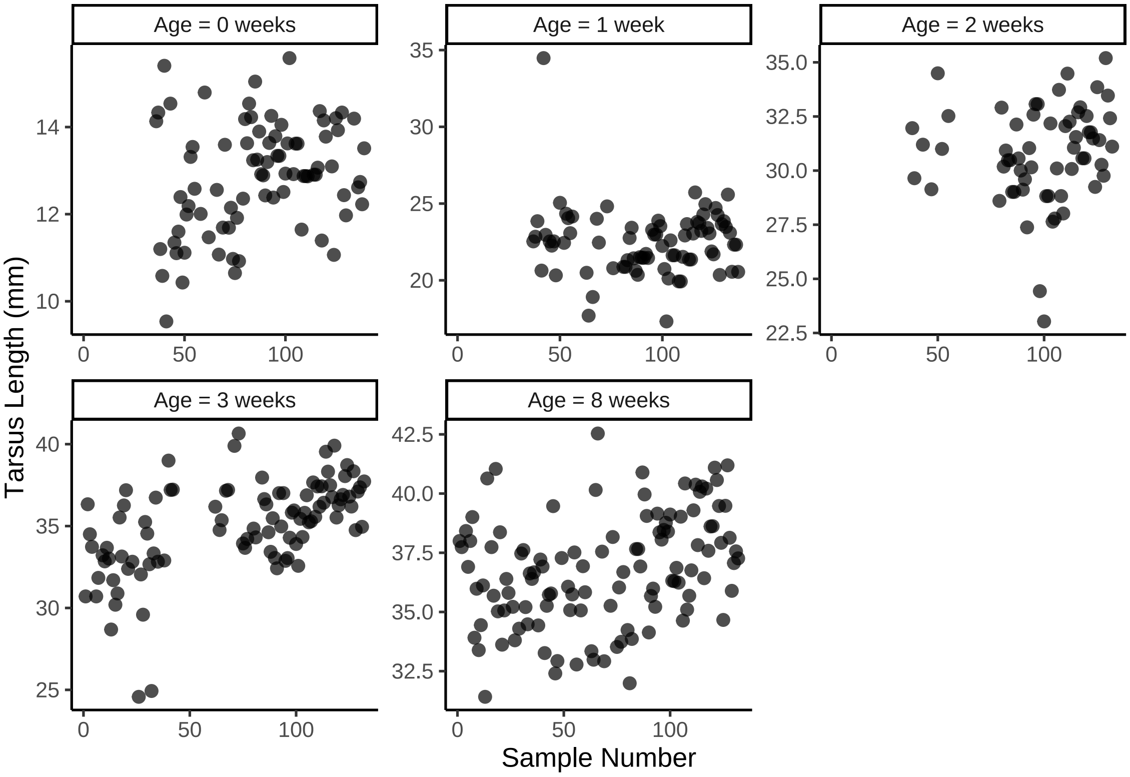

Figure 12: *Cleveland dotplot of adjusted tarsus length estimates (mm) from Japanese quail at 0, 1, 2, 3, and 8 weeks of age.*

One particularly high tarsus length measurement is apparent at 1 week of age, however, its value falls within the distribution of measurements capture at 2 weeks of age. For this reason, this point is retained and visualisations are repeated across ages.

```
data %>%
  filter(week < 10) %>%
  ggplot(aes(x = week, y = tarsusLengthMeanAdjusted)) +
  geom_point(size = 2, pch = 21, fill = "slateblue", colour = "black",
             position = position_jitter(width = 0.5), alpha = 0.3) +
  stat_summary(geom = "errorbar", fun.data = "mean_cl_boot",
               colour = "black", width = 0.3) +
  stat_summary(geom = "point", fun = "mean", size = 3, pch = 21,
               colour = "black", fill = "black") +
  theme_classic() +
  xlab("Age (weeks)") +
  ylab("Tarsus Length (mm)")
```

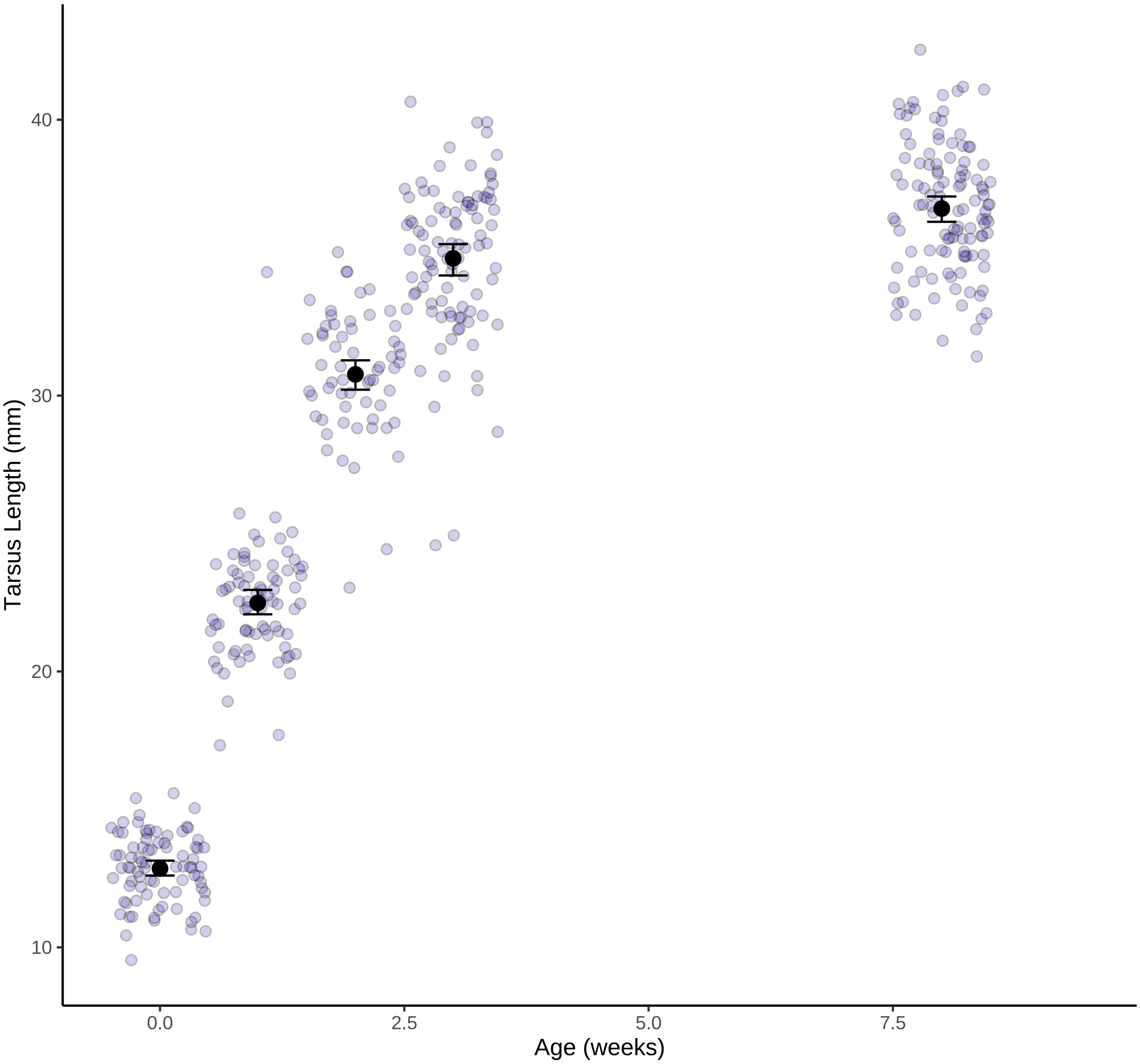

Figure 13: *Raw tarsus length (mm) measurements during growth in Japanese quail (n = 133). Measurements are jittered by age to simplify visualisation.*

No obvious oddities are detected. We therefore proceed to modelling bill length as a function of calibration method and age. Again flat priors are used for all population-level slopes while our intercept is modelled with a Student's-T distribution with three degrees of freedom. Here, μ and *sigma* for our intercept prior are set as 8.3 and 2.6 respectively.

```
billCorrection <- brm(billLengthMean |
                          se(billLengthSD, sigma = TRUE) ~
                          week + billCalibration,
  data = data %>%
    filter(!(is.na(billLengthMean))) %>%
    mutate(
      week = factor(week),
      billLengthSD = ifelse(is.na(billLengthSD),
                            0.001,
                            billLengthSD)
```

```
    ),
  iter = 50000, warmup = 5000, cores = 4, chains = 4,
  inits = 0,
  control = list(adapt_delta = .95),
  silent = TRUE, refresh = 0,
  file = "./models/_billCorrectionModel.Rds"
)

billCorrection %>%
  as.data.frame() %>%
  mutate(b_Intercept = b_Intercept - mean(b_Intercept)) %>%
  dplyr::select(
    "Grid over" = b_Intercept,
    "Grid under" = b_billCalibrationgridUnder,
    "Ruler over" = b_billCalibrationrulerOver,
    "Ruler under" = b_billCalibrationrulerUnder
  ) %>%
  pivot_longer(everything(),
    names_to = "method",
    values_to = "effect"
  ) %>%
  ggplot(aes(x = effect)) +
  facet_wrap(~method, scales = "free") +
  geom_vline(xintercept = 0, linetype = "dashed", colour = "black") +
  geom_density(colour = "black", fill = "grey50", alpha = 0.5) +
  theme_classic() +
  xlab("Mean Effect on Bill Length Estimate (mm)") +
  ylab("Density")
```

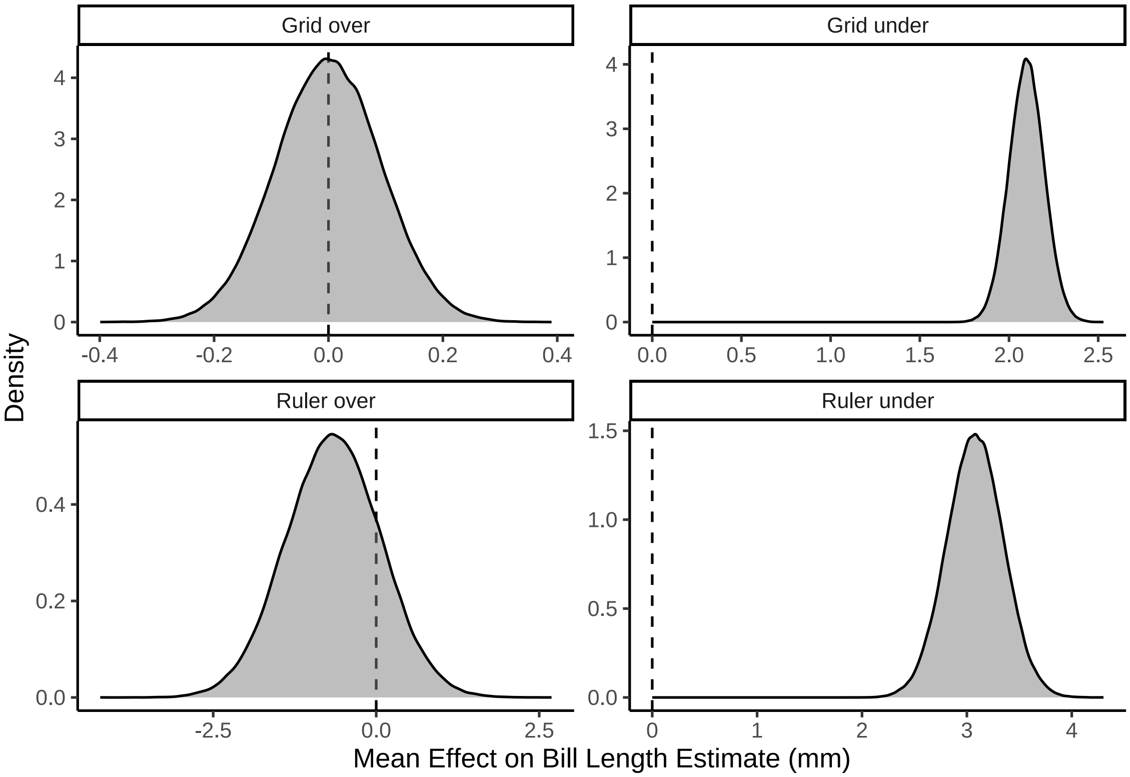

Figure 14: *Effects (model coefficients) of measurement calibration method on bill length estimate in Japanese quail. Distributions represent posterior densities from a Bayesian, linear model with tarsus length (mm) as the response variable and age (weeks) and calibration method as predictors.*

Again, posterior distributions are visualised, a Bayesian R^2 is estimated (using posterior median as a measure

of centrality) and effects of calibration method displayed.

```
pp_check2(billCorrection, xlab = "Bill Length (mm)")
```

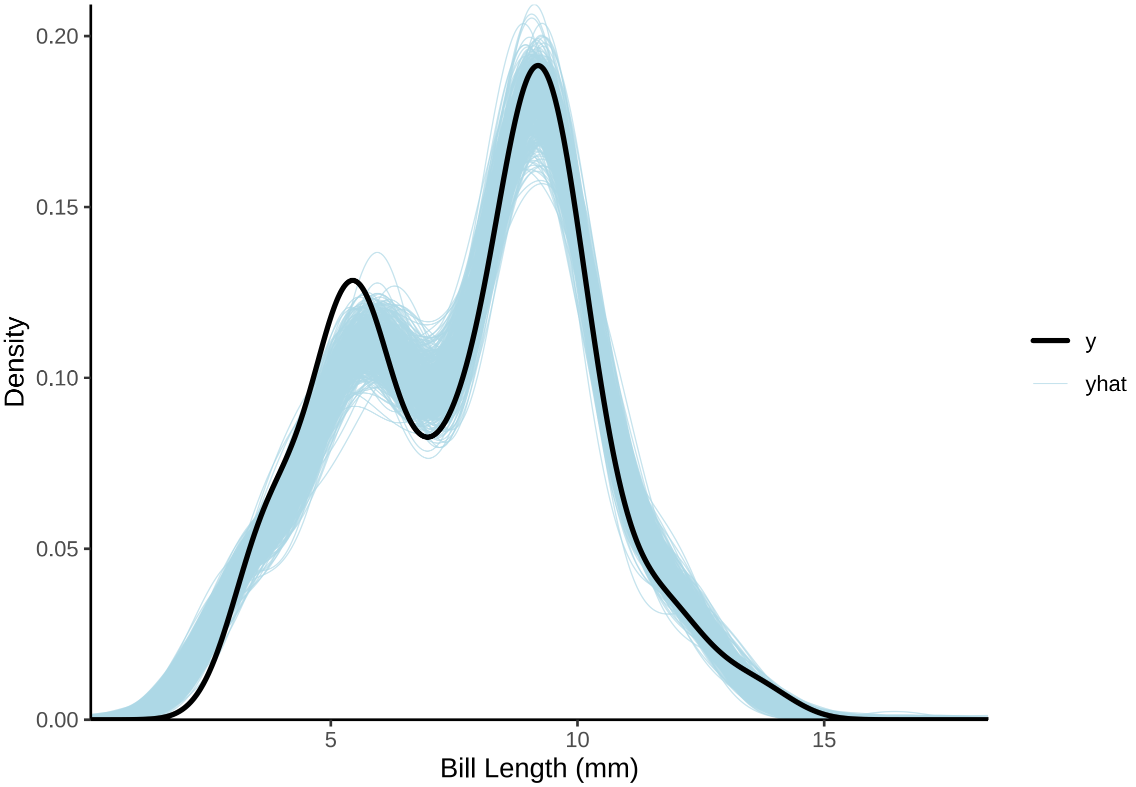

Figure 15: *Density of posterior draws from a Bayesian linear mixed effects model predicting bill length in Japanese quail (mm; draws in grey) as a function of age and calibration method. Density is overlayed with that of true tarsus length measurements (mm; line in black).*

```
simpleR2(billCorrection, robust = TRUE)
```

```
## R2 = 0.9108 [0.90536,0.91459]
```

```
## Suitable R2. Correcting for calibration method, here using posterior
# means given apparent normality of posterior densities.

billDeltas <- as.data.frame(billCorrection) %>%
  summarise(
    "gridUnder" = mean(b_billCalibrationgridUnder),
    "gridUnderLCL" = quantile(b_billCalibrationgridUnder,
                              0.025, type = 8),
    "gridUnderUCL" = quantile(b_billCalibrationgridUnder,
                              0.975, type = 8),
    "rulerOver" = mean(b_billCalibrationrulerOver),
    "rulerOverLCL" = quantile(b_billCalibrationrulerOver,
                               0.025, type = 8),
    "rulerOverUCL" = quantile(b_billCalibrationrulerOver,
                               0.975, type = 8),
    "rulerUnder" = mean(b_billCalibrationrulerUnder),
    "rulerUnderLCL" = quantile(b_billCalibrationrulerUnder,
                               0.025, type = 8),
    "rulerUnderUCL" = quantile(b_billCalibrationrulerUnder,
                               0.975, type = 8)
  )
```

Table 7: *Estimated effect of measurement calibration method on bill length estimates (mm; posterior means) across ages in Japanese quail. Credible invervals (2.5 and 97.5%) are in braces.*

Calibration Method	Effect on bill length estimate (mm)
Grid Under	2.09884 [1.90475,2.29311]
Ruler Over	-0.65285 [-2.08247,0.78486]
Ruler Under	3.07917 [2.55423,3.60595]

```
caption <- paste0("Estimated effect of measurement calibration ",
                  "method on bill length estimates (mm; posterior means) ",
                  "across ages in Japanese quail. Credible invervals ",
                  "(2.5 and 97.5\\%) are in braces."
)

billDeltas %>%
  mutate(
    "Grid Under" = paste0(
      round(gridUnder, digits = 5),
      " [",
      round(gridUnderLCL, digits = 5),
      ",",
      round(gridUnderUCL, digits = 5),
      "]"
    ),
    "Ruler Over" = paste0(
      round(rulerOver, digits = 5),
      " [",
      round(rulerOverLCL, digits = 5),
      ",",
      round(rulerOverUCL, digits = 5),
      "]"
    ),
    "Ruler Under" = paste0(
      round(rulerUnder, digits = 5),
      " [",
      round(rulerUnderLCL, digits = 5),
      ",",
      round(rulerUnderUCL, digits = 5),
      "]"
    )
  ) %>%
  dplyr::select(`Grid Under`, `Ruler Over`, `Ruler Under`) %>%
  pivot_longer(everything(), names_to = "Calibration Method",
               values_to = "Effect on bill length estimate (mm)") %>%
  kbl(., format = "latex", caption = caption, escape = FALSE) %>%
  kable_styling()
```

```
rm(billCorrection)

## Correcting

data <- data %>%
  mutate(
    "billLengthMeanAdjusted" =
      ifelse(billCalibration == "gridUnder",
        billLengthMean - billDeltas$gridUnder,
        ifelse(billCalibration == "rulerOver",
          billLengthMean - billDeltas$rulerOver,
          ifelse(
            billCalibration == "rulerUnder",
            billLengthMean - billDeltas$rulerUnder,
            billLengthMean
          )
        )
      )
```

```
)
```

Measurement oddities are again checked in light of correction.

```
ggplot(data %>%
         filter(week %in% c(0:3, 8)) %>%
         group_by(week) %>%
         mutate("ID" = 1:n()) %>%
         ungroup() %>%
         mutate("week" = ifelse(week == 1,
                                paste0("Age = ", week, " week"),
                                paste0("Age = ", week, " weeks"))
                ),
       aes(x = ID, y = billLengthMeanAdjusted)) +
  facet_wrap(~week, scales = "free") +
  geom_point(size = 2, colour = "black",
             fill = "slateblue", alpha = 0.7) +
  theme_classic() +
  xlab("Sample Number") +
  ylab("Bill Length (mm)")
```

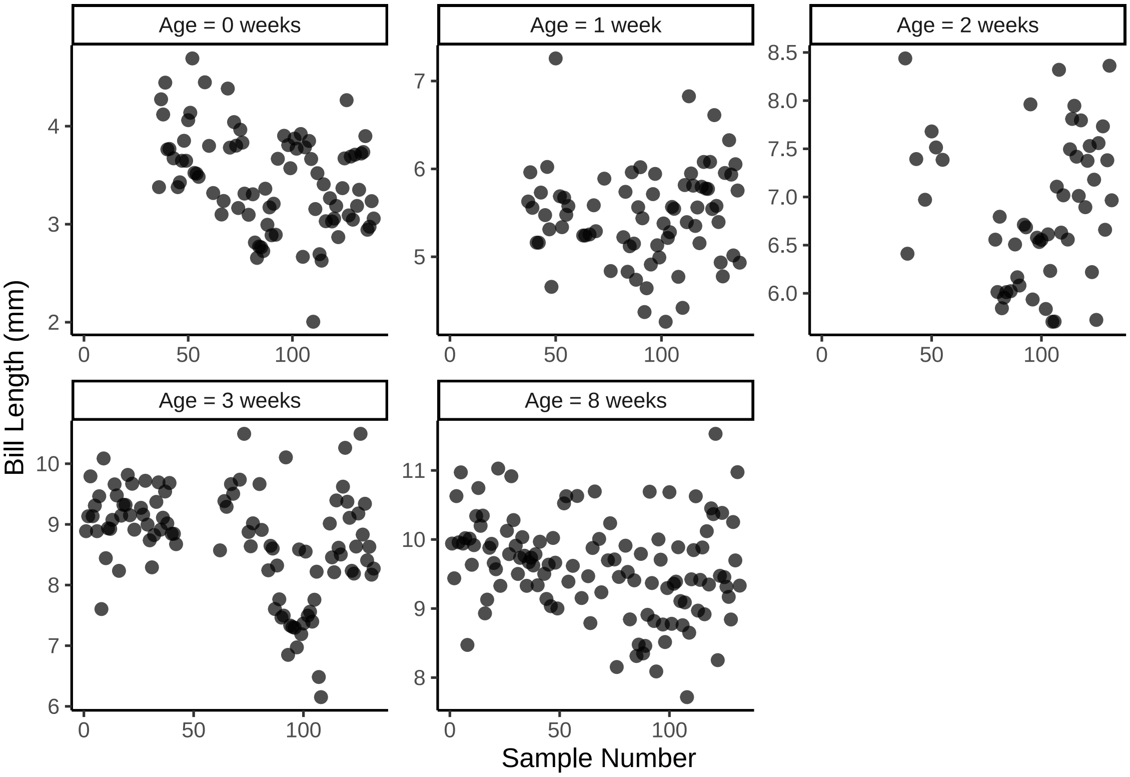

Figure 16: *Cleveland dotplot of adjusted bill length estimates (mm) from Japanese quail at 0, 1, 2, 3, and 8 weeks of age.*

```
data %>%
  filter(week < 10) %>%
  ggplot(aes(x = week, y = billLengthMeanAdjusted)) +
  geom_point(size = 2, pch = 21, fill = "slateblue", colour = "black",
             position = position_jitter(width = 0.5), alpha = 0.3) +
  stat_summary(geom = "errorbar", fun.data = "mean_cl_boot",
               colour = "black", width = 0.3) +
  stat_summary(geom = "point", fun = "mean", size = 3, pch = 21,
               colour = "black", fill = "black") +
```

```
  theme_classic() +
  xlab("Age (weeks)") +
  ylab("Bill Length (mm)")
```

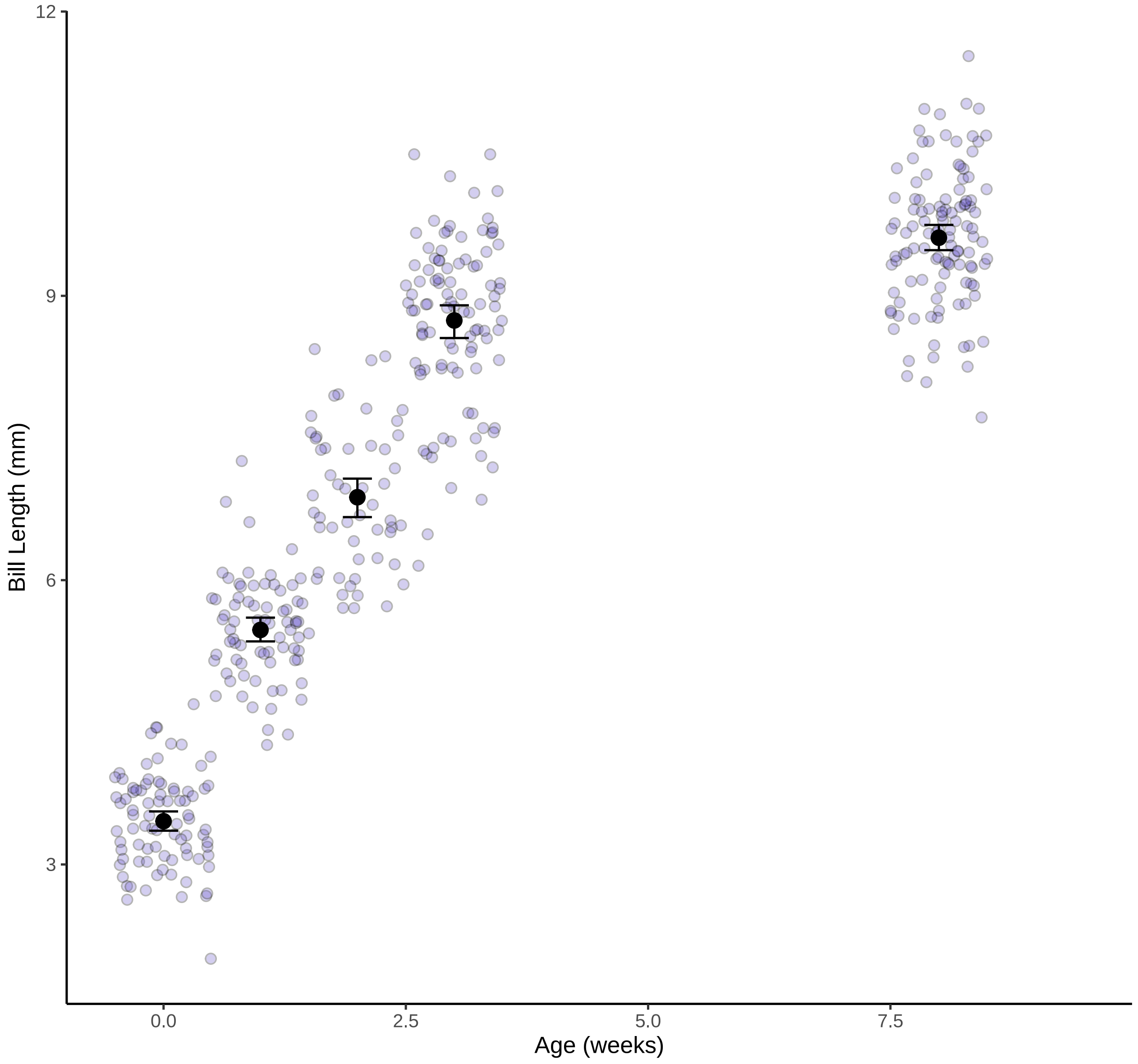

Figure 17: *Adjusted bill length (mm) measurements during growth in Japanese quail (n = 133). Measurements are jittered by age to simplify visualisation.*

With appendange length measurements calibrated, we visualise trends of elongation across ages by treatment type. Here, effects of body mass are not corrected and only raw trends are viewed.

```
showtext_auto()

tarsusPlotA <- data %>%
  filter(!is.na(pretreatment) & !is.na(treatment) & week <= 8) %>%
  mutate(pretreatment = ifelse(pretreatment == "cold", "Cold (10°C)",
    ifelse(pretreatment == "neutral", "Mild (20°C)",
      "Warm (30°C)"
```

```
    )
  )) %>%
  ggplot(aes(x = week, y = tarsusLengthMeanAdjusted, fill = pretreatment)) +
  stat_summary(
    geom = "line", fun.data = "mean_se",
    position = position_dodge(width = 0.8), colour = "black",
    aes(linetype = pretreatment)
  ) +
  stat_summary(
    geom = "errorbar", fun.data = "mean_se", width = 0.3,
    position = position_dodge(width = 0.8)
  ) +
  stat_summary(
    geom = "point", fun = "mean", size = 3, colour = "black",
    pch = 21, position = position_dodge(width = 0.8)
  ) +
  scale_fill_manual(values = c("#7BB4E3", "black", "#CD5C5C"),
                    name = "Rearing\nConditions") +
  scale_linetype_manual(values = c("dashed", "solid", "dotted"),
                        name = "Rearing\nConditions") +
  xlab("Age (weeks)") +
  ylab("Tarsus Length (mm)") +
  ylim(c(15, 45)) +
  theme_classic() +
  theme(
    axis.title = element_text(size = 12, family = "Noto Sans", colour = "black"),
    legend.title = element_text(size = 12, family = "Noto Sans", colour = "black"),
    legend.text = element_text(size = 11, family = "Noto Sans", colour = "black"),
    legend.position = "bottom"
  )

p2 <- ggtexttable(
    data %>%
      filter(!is.na(pretreatment) & !is.na(treatment) &
               !is.na(tarsusLengthMeanAdjusted) & week <= 8 &
        pretreatment == "cold") %>%
      group_by(week) %>%
      dplyr::count() %>%
      rename("Age (weeks)" = week),
    rows = NULL, theme = ttheme("light")
  ) %>%
    tab_add_title(text = "Cold\nRearing", face = "bold")

p3 <- ggtexttable(
      data %>%
        filter(!is.na(pretreatment) & !is.na(treatment) &
                 !is.na(tarsusLengthMeanAdjusted) & week <= 8 &
          pretreatment == "neutral") %>%
        group_by(week) %>%
        rename("Age (weeks)" = week) %>%
        dplyr::count(),
      rows = NULL, theme = ttheme("light")
    ) %>%
      tab_add_title(text = "Mild\nRearing", face = "bold")

p4 <- ggtexttable(
        data %>%
          filter(!is.na(pretreatment) & !is.na(treatment) &
                   !is.na(tarsusLengthMeanAdjusted) & week <= 8 &
            pretreatment == "warm") %>%
          group_by(week) %>%
          rename("Age (weeks)" = week) %>%
          dplyr::count(),
        rows = NULL, theme = ttheme("light")
      ) %>%
      tab_add_title(text = "Warm\nRearing", face = "bold")
```

```r
tarsusPlotA/(p2 + p3 + p4)
```

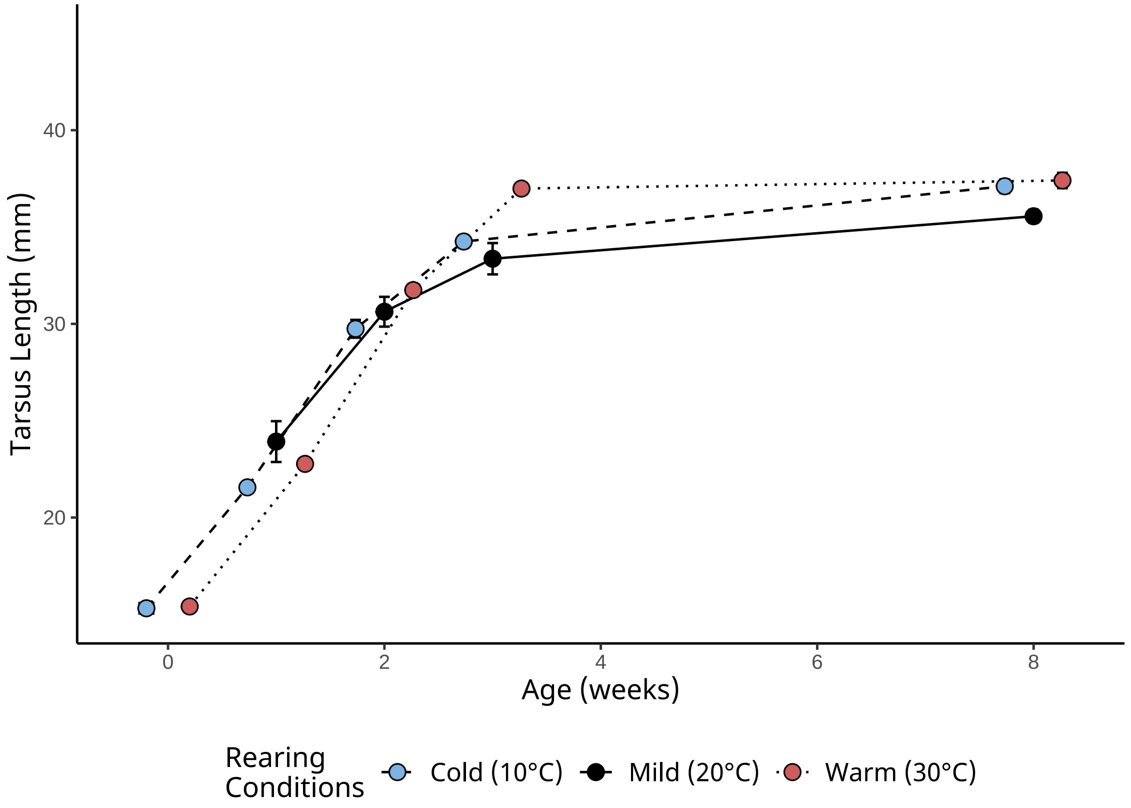

Cold Rearing

Age (weeks)	n
0	26
1	28
2	27
3	41
8	40

Mild Rearing

Age (weeks)	n
0	15
1	12
2	4
3	20
8	33

Warm Rearing

Age (weeks)	n
0	34
1	37
2	29
3	33
8	39

Figure 18: *Effects of age and thermal environment during post-hatch development on tarsus length (mm) in Japanese quail. Dots represent means and errorbars represent standard errors.*

```r
showtext_auto(enable = FALSE)
```

```r
showtext_auto()

billPlotA <- data %>%
  filter(!is.na(pretreatment) & !is.na(treatment) & week <= 8) %>%
  mutate(pretreatment = ifelse(pretreatment == "cold", "Cold (10°C)",
```

```
    ifelse(pretreatment == "neutral", "Mild (20°C)",
      "Warm (30°C)"
    )
  )) %>%
  ggplot(aes(x = week, y = billLengthMeanAdjusted, fill = pretreatment)) +
  stat_summary(
    geom = "line", fun.data = "mean_se",
    position = position_dodge(width = 0.8), colour = "black",
    aes(linetype = pretreatment)
  ) +
  stat_summary(
    geom = "errorbar", fun.data = "mean_se", width = 0.3,
    position = position_dodge(width = 0.8)
  ) +
  stat_summary(
    geom = "point", fun = "mean", size = 3, colour = "black",
    pch = 21, position = position_dodge(width = 0.8)
  ) +
  scale_fill_manual(values = c("#7BB4E3", "black", "#CD5C5C"),
                    name = "Rearing\nConditions") +
  scale_linetype_manual(values = c("dashed", "solid", "dotted"),
                        name = "Rearing\nConditions") +
  xlab("Age (weeks)") +
  ylab("Bill Length (mm)") +
  ylim(c(0, 12)) +
  theme_classic() +
  theme(
    axis.title = element_text(size = 12, family = "Noto Sans", colour = "black"),
    legend.title = element_text(size = 12, family = "Noto Sans", colour = "black"),
    legend.text = element_text(size = 11, family = "Noto Sans", colour = "black"),
    legend.position = "bottom"
  )

p2 <- ggtexttable(
    data %>%
      filter(!is.na(pretreatment) & !is.na(treatment) &
               !is.na(billLengthMeanAdjusted) & week <= 8 &
        pretreatment == "cold") %>%
      group_by(week) %>%
      dplyr::count() %>%
      rename("Age (weeks)" = week),
    rows = NULL, theme = ttheme("light")
  ) %>%
    tab_add_title(text = "Cold\nRearing", face = "bold")

p3 <- ggtexttable(
      data %>%
        filter(!is.na(pretreatment) & !is.na(treatment) &
                 !is.na(billLengthMeanAdjusted) & week <= 8 &
          pretreatment == "neutral") %>%
        group_by(week) %>%
        rename("Age (weeks)" = week) %>%
        dplyr::count(),
      rows = NULL, theme = ttheme("light")
    ) %>%
      tab_add_title(text = "Mild\nRearing", face = "bold")

p4 <- ggtexttable(
        data %>%
          filter(!is.na(pretreatment) & !is.na(treatment) &
                   !is.na(billLengthMeanAdjusted) & week <= 8 &
            pretreatment == "warm") %>%
          group_by(week) %>%
          rename("Age (weeks)" = week) %>%
          dplyr::count(),
        rows = NULL, theme = ttheme("light")
      ) %>%
```

```
    tab_add_title(text = "Warm\nRearing", face = "bold")

billPlotA/(p2 + p3 + p4)
```

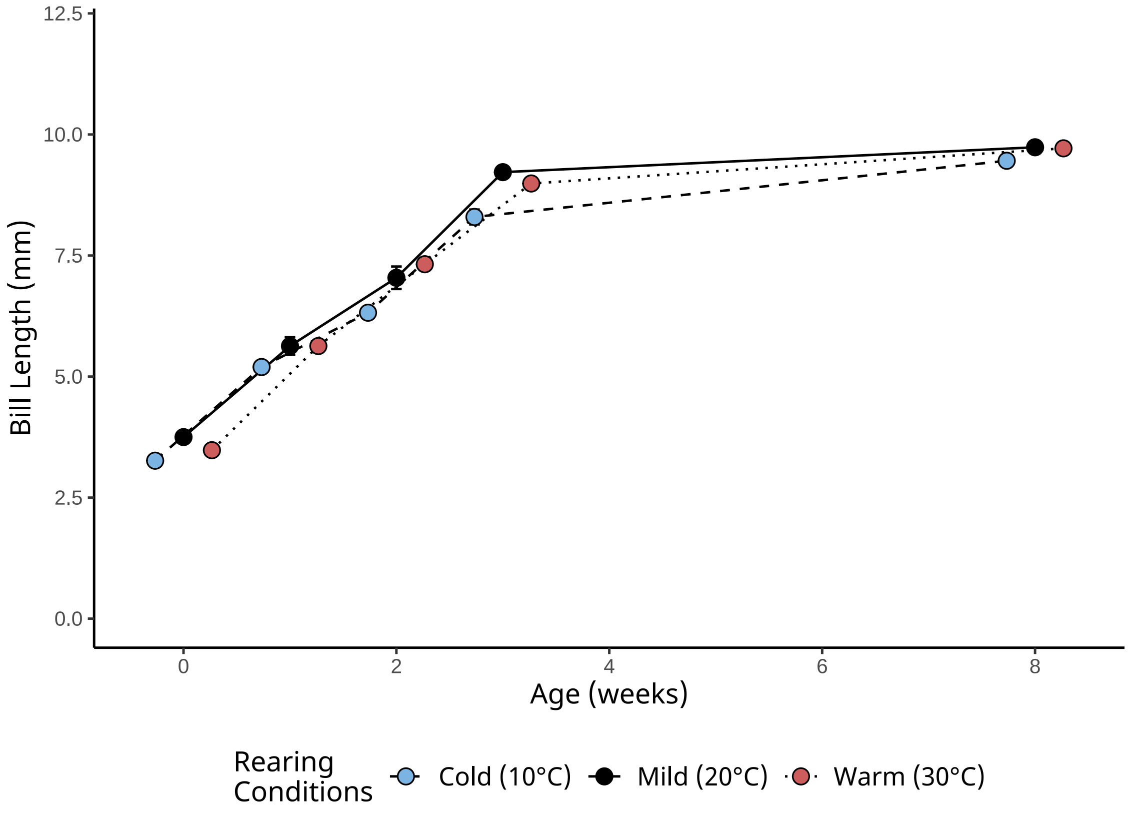

Cold Rearing

Age (weeks)	n
0	21
1	25
2	22
3	43
8	42

Mild Rearing

Age (weeks)	n
0	15
1	12
2	4
3	24
8	34

Warm Rearing

Age (weeks)	n
0	40
1	36
2	26
3	30
8	36

Figure 19: *Effects of age and thermal environment during post-hatch development on bill length (mm) in Japanese quail. Dots represent means and errorbars represent standard errors.*

```
showtext_auto(enable = FALSE)
```

The above plots are reproduced below while only including individuals that were reared at 10°C, 20°C, or 30°C fully until maturity.

```
tarsusPlotB <- data %>%
  filter(!is.na(pretreatment) & !is.na(treatment) & week <= 8) %>%
  filter(!(exp == "A" & pretreatment == "cold") &
           !(exp == "B" & pretreatment == "warm")) %>%
  mutate(pretreatment = ifelse(pretreatment == "cold", "Cold (10°C)",
    ifelse(pretreatment == "neutral", "Mild (20°C)",
      "Warm (30°C)"
    )
  )) %>%
  ggplot(aes(x = week, y = tarsusLengthMeanAdjusted, fill = pretreatment)) +
  stat_summary(
    geom = "line", fun.data = "mean_se",
    position = position_dodge(width = 0.8), colour = "black",
    aes(linetype = pretreatment)
  ) +
  stat_summary(
    geom = "errorbar", fun.data = "mean_se", width = 0.3,
    position = position_dodge(width = 0.8)
  ) +
  stat_summary(
    geom = "point", fun = "mean", size = 3, colour = "black",
    pch = 21, position = position_dodge(width = 0.8)
  ) +
  scale_fill_manual(values = c("#7BB4E3", "black", "#CD5C5C"),
                    name = "Rearing\nConditions") +
  scale_linetype_manual(values = c("dashed", "solid", "dotted"),
                        name = "Rearing\nConditions") +
  xlab("Age (weeks)") +
  ylab("Tarsus Length (mm)") +
  ylim(c(15, 45)) +
  theme_classic() +
  theme(
    axis.title = element_text(size = 12, family = "Noto Sans", colour = "black"),
    legend.title = element_text(size = 12, family = "Noto Sans", colour = "black"),
    legend.text = element_text(size = 11, family = "Noto Sans", colour = "black"),
    legend.position = "bottom"
  )

p2 <- ggtexttable(
    data %>%
      filter(!is.na(pretreatment) & !is.na(treatment) &
               !is.na(tarsusLengthMeanAdjusted) & week <= 8) %>%
      filter(!(exp == "A" & pretreatment == "cold")) %>%
      filter(pretreatment == "cold") %>%
      group_by(week) %>%
      count() %>%
      rename("Age (weeks)" = week),
    rows = NULL, theme = ttheme("light")
  ) %>%
    tab_add_title(text = "Cold\nRearing", face = "bold")

p3 <- ggtexttable(
      data %>%
        filter(!is.na(pretreatment) & !is.na(treatment) &
                 !is.na(tarsusLengthMeanAdjusted) & week <= 8 &
          pretreatment == "neutral") %>%
        group_by(week) %>%
        rename("Age (weeks)" = week) %>%
        count(),
      rows = NULL, theme = ttheme("light")
    ) %>%
      tab_add_title(text = "Mild\nRearing", face = "bold")

p4 <- ggtexttable(
        data %>%
          filter(!is.na(pretreatment) & !is.na(treatment) &
                   !is.na(tarsusLengthMeanAdjusted) & week <= 8) %>%
```

```
          filter(!(exp == "B" & pretreatment == "warm")) %>%
          filter(pretreatment == "warm") %>%
          group_by(week) %>%
          rename("Age (weeks)" = week) %>%
          count(),
        rows = NULL, theme = ttheme("light")
      ) %>%
      tab_add_title(text = "Warm\nRearing", face = "bold")

tarsusPlotB/(p2 + p3 + p4)
```

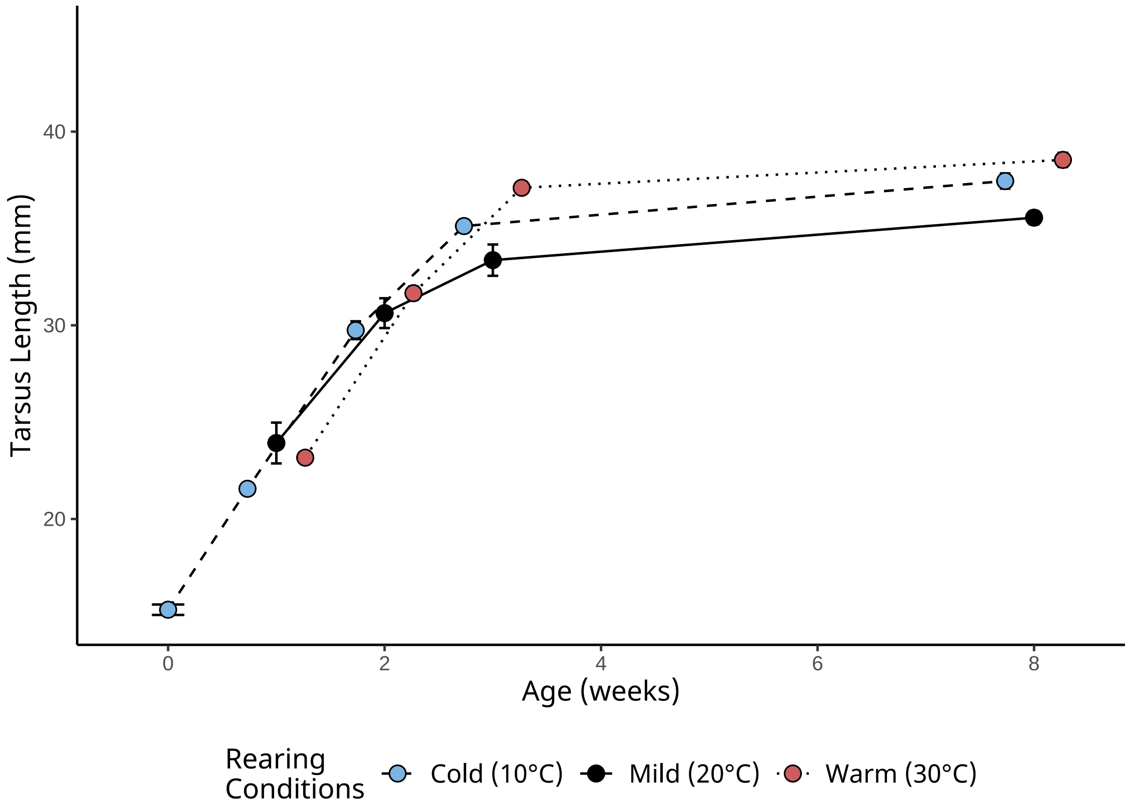

Cold Rearing

Age (weeks)	n
0	26
1	28
2	27
3	25
8	24

Mild Rearing

Age (weeks)	n
0	15
1	12
2	4
3	20
8	33

Warm Rearing

Age (weeks)	n
0	18
1	26
2	26
3	24
8	23

Figure 20: *Effects of age and thermal environment during post-hatch development on tarsus length (mm) in Japanese quail, where only individuals reared in selected thermal environments for 8 weeks of are included. Dots represent group means and errorbars represent group standard errors.*

```
showtext_auto(enable = FALSE)
```

```
billPlotB <- data %>%
  filter(!is.na(pretreatment) & !is.na(treatment) & week <= 8) %>%
  filter(!(exp == "A" & pretreatment == "cold") &
           !(exp == "B" & pretreatment == "warm")) %>%
  mutate(pretreatment = ifelse(pretreatment == "cold", "Cold (10°C)",
    ifelse(pretreatment == "neutral", "Mild (20°C)",
      "Warm (30°C)"
```

```
    )
  )) %>%
  ggplot(aes(x = week, y = billLengthMeanAdjusted, fill = pretreatment)) +
  stat_summary(
    geom = "line", fun.data = "mean_se",
    position = position_dodge(width = 0.8), colour = "black",
    aes(linetype = pretreatment)
  ) +
  stat_summary(
    geom = "errorbar", fun.data = "mean_se", width = 0.3,
    position = position_dodge(width = 0.8)
  ) +
  stat_summary(
    geom = "point", fun = "mean", size = 3, colour = "black",
    pch = 21, position = position_dodge(width = 0.8)
  ) +
  scale_fill_manual(values = c("#7BB4E3", "black", "#CD5C5C"),
                    name = "Rearing\nConditions") +
  scale_linetype_manual(values = c("dashed", "solid", "dotted"),
                        name = "Rearing\nConditions") +
  xlab("Age (weeks)") +
  ylab("Bill Length (mm)") +
  ylim(c(0, 12)) +
  theme_classic() +
  theme(
    axis.title = element_text(size = 12, family = "Noto Sans", colour = "black"),
    legend.title = element_text(size = 12, family = "Noto Sans", colour = "black"),
    legend.text = element_text(size = 11, family = "Noto Sans", colour = "black"),
    legend.position = "bottom"
  )

p2 <- ggtexttable(
    data %>%
      filter(!is.na(pretreatment) & !is.na(treatment) &
               !is.na(billLengthMeanAdjusted) & week <= 8) %>%
      filter(!(exp == "A" & pretreatment == "cold")) %>%
      filter(pretreatment == "cold") %>%
      group_by(week) %>%
      count() %>%
      rename("Age (weeks)" = week),
    rows = NULL, theme = ttheme("light")
  ) %>%
    tab_add_title(text = "Cold\nRearing", face = "bold")

p3 <- ggtexttable(
      data %>%
        filter(!is.na(pretreatment) & !is.na(treatment) &
                 !is.na(billLengthMeanAdjusted) & week <= 8 &
          pretreatment == "neutral") %>%
        group_by(week) %>%
        rename("Age (weeks)" = week) %>%
        count(),
      rows = NULL, theme = ttheme("light")
    ) %>%
      tab_add_title(text = "Mild\nRearing", face = "bold")

p4 <- ggtexttable(
        data %>%
          filter(!is.na(pretreatment) & !is.na(treatment) &
                   !is.na(billLengthMeanAdjusted) & week <= 8) %>%
          filter(!(exp == "B" & pretreatment == "warm")) %>%
          filter(pretreatment == "warm") %>%
          group_by(week) %>%
          rename("Age (weeks)" = week) %>%
          count(),
        rows = NULL, theme = ttheme("light")
      ) %>%
```

```
    tab_add_title(text = "Warm\nRearing", face = "bold")

billPlotB/(p2 + p3 + p4)
```

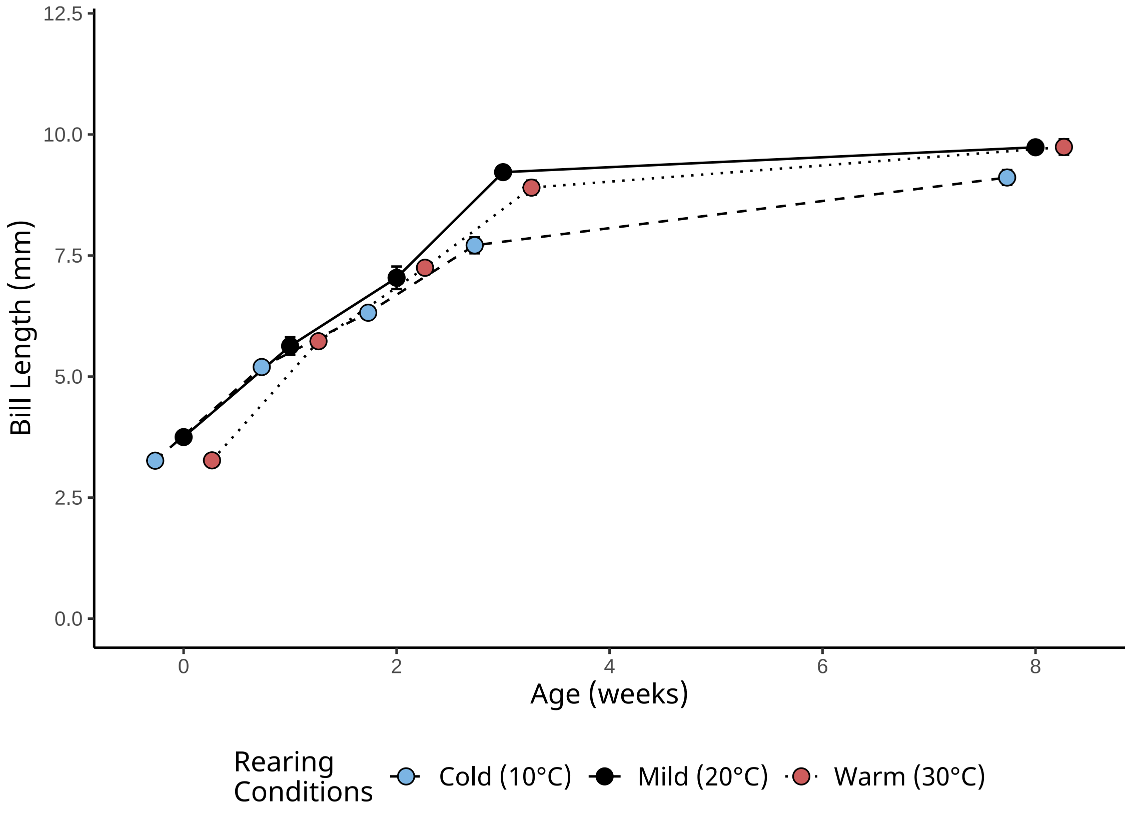

Cold Rearing

Age (weeks)	n
0	21
1	25
2	22
3	25
8	24

Mild Rearing

Age (weeks)	n
0	15
1	12
2	4
3	24
8	34

Warm Rearing

Age (weeks)	n
0	24
1	25
2	23
3	21
8	23

Figure 21: *Effects of age and thermal environment during post-hatch development on bill length (mm) in Japanese quail, where only individuals reared in selected thermal environments for 8 weeks of are included. Dots represent group means and errorbars represent group standard errors.*

```
showtext_auto(enable = FALSE)
```

Data inspection is next continued, with particular focus on the spread of body mass data. A final data-frame

containing all collated data is then saved in a single .csv file for future use.

```
# Checking for aberrant mass values

ggplot(
  data %>%
    filter(week <= 8) %>%
    group_by(week) %>%
    mutate("ID" = 1:n()) %>%
    ungroup() %>%
    mutate("week" = ifelse(week == 1,
                           paste0("Age = ", week, " week"),
                           paste0("Age = ", week, " weeks"))
           ),
  aes(x = ID, y = mass)
) +
  facet_wrap(~week, scales = "free") +
  geom_point(size = 2, colour = "black",
             fill = "slateblue", alpha = 0.7) +
  theme_classic() +
  xlab("Sample Number") +
  ylab("Body Mass (g)")
```

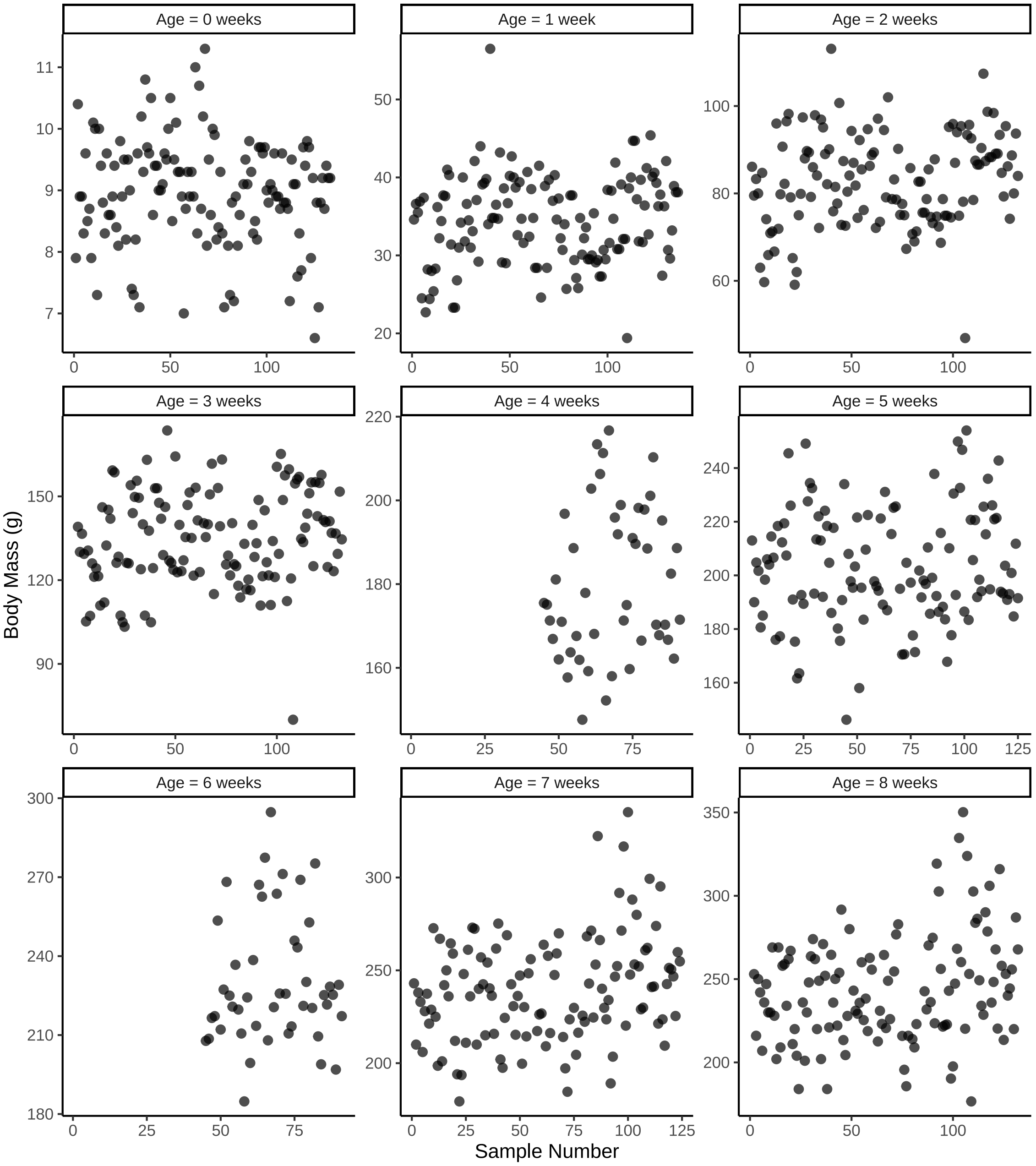

Figure 22: *Cleveland dotplots displaying body mass (g) by sample number of Japanese quail.*

```
# One peculiarly low value (week 3), however, mass still appears to fall within
# the distributions of surrounding weeks. Retaining for this reason.

# Removing raw, unadjusted tarsus and bill length measurements,
# then renaming adjusted values for simplicity.

data <- data %>%
  dplyr::select(-c(tarsusLengthMean, billLengthMean)) %>%
  rename("tarsusLengthMean" = tarsusLengthMeanAdjusted,
         "billLengthMean" = billLengthMeanAdjusted)
```

```
## Visualising change in mass across age

showtext_auto()
```

```
massPlot <- data %>%
  filter(!is.na(pretreatment) & !is.na(treatment) &
    week <= 8) %>%
  mutate(pretreatment = ifelse(pretreatment == "cold", "Cold (10°C)",
    ifelse(pretreatment == "neutral", "Mild (20°C)",
      "Warm (30°C)"
    )
  )) %>%
  ggplot(aes(x = week, y = mass, fill = pretreatment)) +
  stat_summary(
    geom = "line", fun.data = "mean_cl_boot",
    position = position_dodge(width = 0.5), colour = "black",
    aes(linetype = pretreatment)
  ) +
  stat_summary(
    geom = "errorbar", fun.data = "mean_cl_boot", width = 0.3,
    position = position_dodge(width = 0.5)
  ) +
  stat_summary(
    geom = "point", fun = "mean", size = 3, colour = "black",
    pch = 21, position = position_dodge(width = 0.5)
  ) +
  scale_fill_manual(values = c("#7BB4E3", "black", "#CD5C5C"),
                    name = "Rearing\nConditions") +
  scale_linetype_manual(values = c("dashed", "solid", "dotted"),
                        name = "Rearing\nConditions") +
  xlab("Age (weeks)") +
  ylab("Mass (g)") +
  ylim(c(0, 280)) +
  theme_classic() +
  theme(
    axis.title = element_text(size = 12, family = "Noto Sans", colour = "black"),
    legend.title = element_text(size = 12, family = "Noto Sans", colour = "black"),
    legend.text = element_text(size = 11, family = "Noto Sans", colour = "black"),
    legend.position = "bottom"
  )

p2 <- ggtexttable(
    data %>%
      filter(!is.na(pretreatment) & !is.na(treatment) &
               !is.na(mass) & week <= 8 &
        pretreatment == "cold") %>%
      group_by(week) %>%
      count() %>%
      rename("Age (weeks)" = week),
    rows = NULL, theme = ttheme("light")
  ) %>%
    tab_add_title(text = "Cold\nRearing", face = "bold")

p3 <- ggtexttable(
      data %>%
        filter(!is.na(pretreatment) & !is.na(treatment) &
                 !is.na(mass) & week <= 8 &
          pretreatment == "neutral") %>%
        group_by(week) %>%
        rename("Age (weeks)" = week) %>%
        count(),
      rows = NULL, theme = ttheme("light")
    ) %>%
    tab_add_title(text = "Mild\nRearing", face = "bold")

p4 <- ggtexttable(
      data %>%
        filter(!is.na(pretreatment) & !is.na(treatment) &
                 !is.na(mass) & week <= 8 &
          pretreatment == "warm") %>%
        group_by(week) %>%
```

```
        rename("Age (weeks)" = week) %>%
        count(),
      rows = NULL, theme = ttheme("light")
    ) %>%
    tab_add_title(text = "Warm\nRearing", face = "bold")

massPlot/(p2 + p3 + p4)
```

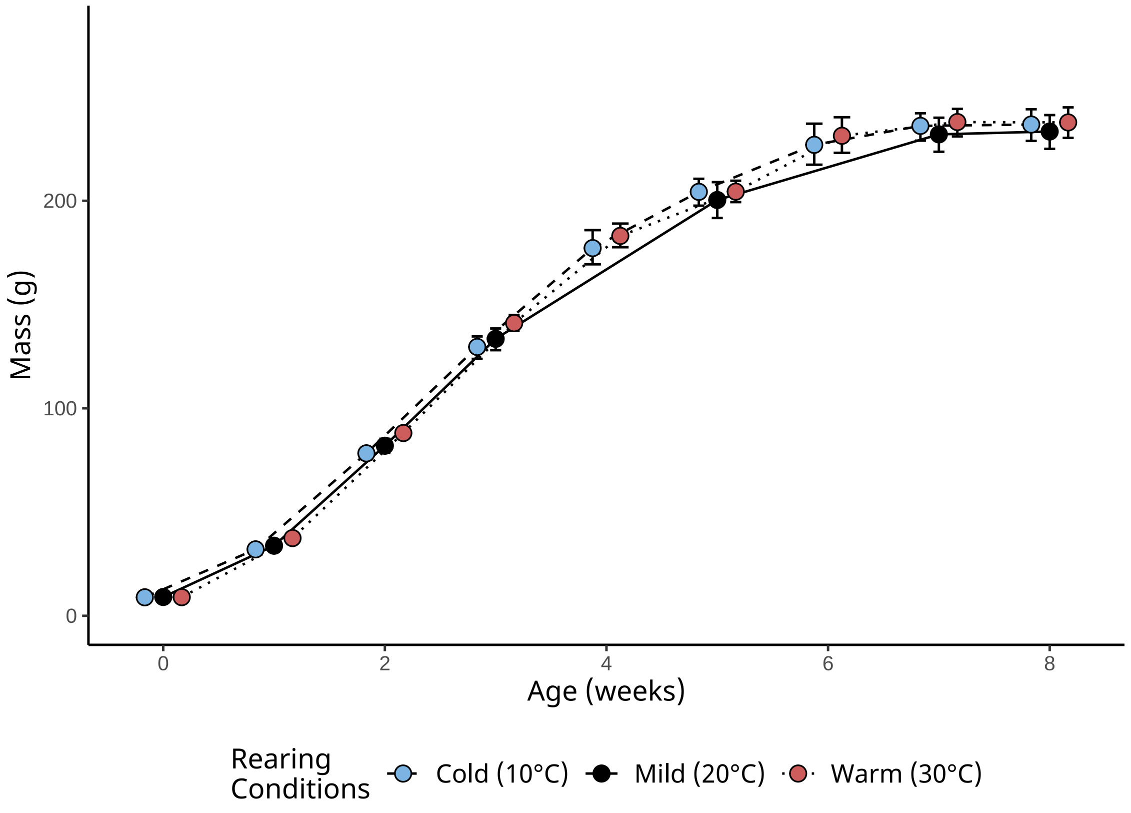

Cold Rearing

Age (weeks)	n
0	44
1	48
2	46
3	43
4	24
5	42
6	24
7	41
8	42

Mild Rearing

Age (weeks)	n
0	36
1	35
2	34
3	38
5	34
7	33
8	36

Warm Rearing

Age (weeks)	n
0	51
1	49
2	48
3	46
4	23
5	42
6	23
7	43
8	43

Figure 23: *Effect of age and rearing conditions on body mass of Japanese quail. Dots represent group means and errorbars represent 2.5% and 97.5% quantiles.*

```
# Combining tarsus, bill, and mass plots.

earlyPanel <- ggarrange(tarsusPlotB, billPlotB, massPlot,
    common.legend = TRUE,
    legend = "bottom", ncol = 3
  )
```

```
print(earlyPanel)
```

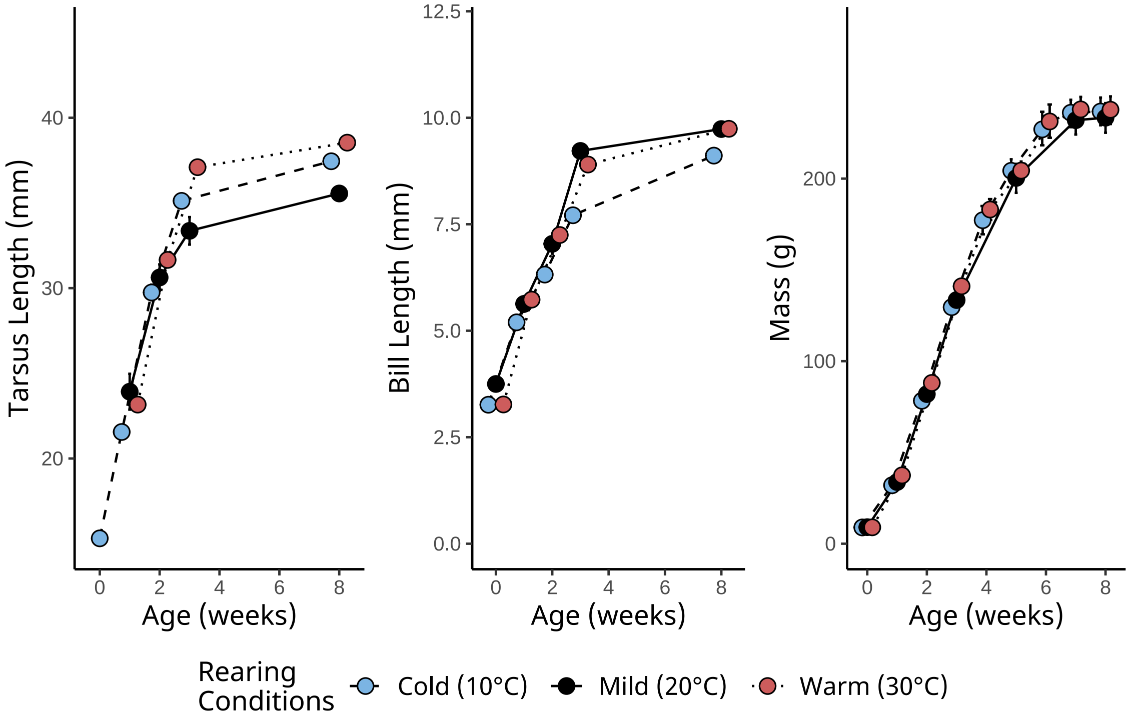

Figure 24: *Effect of age and rearing conditions on morphometry of Japanese quail. Dots represent group means in both plots, while errorbars around tarsus length (mm) means represent standard errors and those around body mass (g) means represent 2.5% and 97.5% quantiles.*

Last, we visualise trends in relative tarsus and bill length by age and treatment type. Length measurements are relativised by estimating either tarsus or bill length residuals from basic linear models. In these models, either tarsus or bill length (mm) is set as the Gaussian-distributed response variable and body mass (g) is set as the sole population-level predictor. Priors for these models are set broadly as follow:

$$Intercept\ (\beta_0) \sim exponential(0.075)$$

$$Body\ Mass\ (\beta_1) \sim \mathcal{N}(1, 2)$$

```
# Exploring residual tarsus and bill length trends

residualTarsus <- data %>%
  drop_na(tarsusLengthMean, mass) %>%
  mutate(
    "residualTarsus" =
      residuals(brm(tarsusLengthMean ~ mass,
        prior = c(
          set_prior("normal(1, 2)", class = "b"),
          set_prior("exponential(0.075)", class = "Intercept")
        ),
        data = .,
        cores = 1, chains = 4,
        seed = 100, family = "gaussian",
        iter = 50000, warmup = 5000, thin = 10,
        control = list(adapt_delta = 0.97, max_treedepth = 14),
```

```
        silent = TRUE, refresh = 0,
        file = "./models/_residualModelPlotA-Median.Rds"
      ), robust = TRUE)[, "Estimate"]
  ) %>%
  filter(!is.na(pretreatment) & !is.na(treatment) & week <= 8) %>%
  mutate(pretreatment = ifelse(pretreatment == "cold", "Cold (10°C)",
    ifelse(pretreatment == "neutral", "Mild (20°C)",
      "Warm (30°C)"
    )
  )) %>%
  ggplot(aes(x = week, y = residualTarsus, fill = pretreatment)) +
  stat_summary(
    geom = "line", fun.data = "mean_cl_boot",
    position = position_dodge(width = 0.4), colour = "black",
    aes(linetype = pretreatment)
  ) +
  stat_summary(
    geom = "errorbar", fun.data = "mean_cl_boot", width = 0.3,
    position = position_dodge(width = 0.4)
  ) +
  stat_summary(
    geom = "point", fun = "mean", size = 3, colour = "black",
    pch = 21, position = position_dodge(width = 0.4)
  ) +
  scale_fill_manual(values = c("#7BB4E3", "black", "#CD5C5C"),
                    name = "Rearing\nConditions") +
  scale_linetype_manual(values = c("dashed", "solid", "dotted"),
                        name = "Rearing\nConditions") +
  xlab("Age (weeks)") +
  ylab("Residual\nTarsus Length (mm)") +
  theme_classic() +
  theme(
    axis.title = element_text(size = 12, family = "Noto Sans", colour = "black"),
    legend.title = element_text(size = 12, family = "Noto Sans", colour = "black"),
    legend.text = element_text(size = 11, family = "Noto Sans", colour = "black"),
    legend.position = "bottom"
  )

p2 <- ggtexttable(
    data %>%
      filter(!is.na(pretreatment) & !is.na(treatment) &
               !is.na(mass) &
        !is.na(tarsusLengthMean) & week <= 8 &
          pretreatment == "cold") %>%
      group_by(week) %>%
      count() %>%
      rename("Age (weeks)" = week),
    rows = NULL, theme = ttheme("light")
  ) %>%
    tab_add_title(text = "Cold\nRearing", face = "bold")

p3 <- ggtexttable(
      data %>%
        filter(!is.na(pretreatment) & !is.na(treatment) &
                 !is.na(mass) &
          !is.na(tarsusLengthMean) & week <= 8 &
            pretreatment == "neutral") %>%
        group_by(week) %>%
        rename("Age (weeks)" = week) %>%
        count(),
      rows = NULL, theme = ttheme("light")
    ) %>%
      tab_add_title(text = "Mild\nRearing", face = "bold")

p4 <- ggtexttable(
        data %>%
          filter(!is.na(pretreatment) & !is.na(treatment) &
```

```
                  !is.na(mass) &
           !is.na(tarsusLengthMean) & week <= 8 &
             pretreatment == "warm") %>%
         group_by(week) %>%
         rename("Age (weeks)" = week) %>%
         count(),
       rows = NULL, theme = ttheme("light")
     ) %>%
     tab_add_title(text = "Warm\nRearing", face = "bold")

residualTarsus/(p2 + p3 + p4)
```

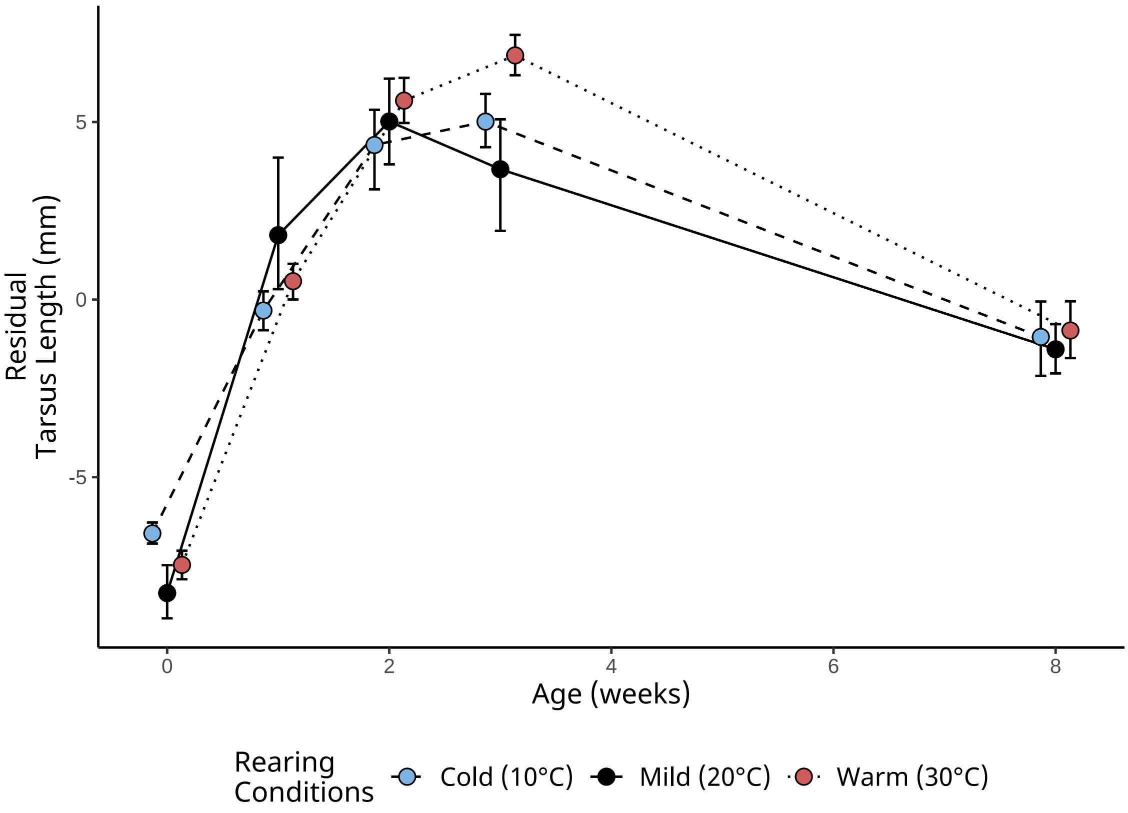

Cold Rearing

Age (weeks)	n
0	25
1	28
2	27
3	41
8	40

Mild Rearing

Age (weeks)	n
0	15
1	12
2	4
3	20
8	33

Warm Rearing

Age (weeks)	n
0	34
1	37
2	29
3	33
8	39

Figure 25: *Effect of age and rearing conditions on relative tarsus length (mm) of Japanese quail. Relative tarsus length represents median residuals from a Bayesian linear model with tarsus length (mm) as as the response variable and body mass (g) as the sole predictor. Dots represent group means and errorbars represent 2.5% and 97.5% quantiles.*

```
residualBill <- data %>%
  drop_na(billLengthMean, mass) %>%
  mutate(
    "residualBill" =
      residuals(brm(billLengthMean ~ mass,
        prior = c(
```

```
          set_prior("normal(1, 2)", class = "b"),
          set_prior("exponential(0.075)", class = "Intercept")
        ),
        data = .,
        cores = 1, chains = 4,
        seed = 100, family = "gaussian",
        iter = 50000, warmup = 5000, thin = 10,
        control = list(adapt_delta = 0.97, max_treedepth = 14),
        silent = TRUE, refresh = 0,
        file = "./models/_residualModelPlotA-MedianBill.Rds"
      ), robust = TRUE)[, "Estimate"]
  ) %>%
  filter(!is.na(pretreatment) & !is.na(treatment) & week <= 8) %>%
  mutate(pretreatment = ifelse(pretreatment == "cold", "Cold (10°C)",
    ifelse(pretreatment == "neutral", "Mild (20°C)",
      "Warm (30°C)"
    )
  )) %>%
  ggplot(aes(x = week, y = residualBill, fill = pretreatment)) +
  stat_summary(
    geom = "line", fun.data = "mean_cl_boot",
    position = position_dodge(width = 0.4), colour = "black",
    aes(linetype = pretreatment)
  ) +
  stat_summary(
    geom = "errorbar", fun.data = "mean_cl_boot", width = 0.3,
    position = position_dodge(width = 0.4)
  ) +
  stat_summary(
    geom = "point", fun = "mean", size = 3, colour = "black",
    pch = 21, position = position_dodge(width = 0.4)
  ) +
  scale_fill_manual(values = c("#7BB4E3", "black", "#CD5C5C"),
                    name = "Rearing\nConditions") +
  scale_linetype_manual(values = c("dashed", "solid", "dotted"),
                        name = "Rearing\nConditions") +
  xlab("Age (weeks)") +
  ylab("Residual\nBill Length (mm)") +
  theme_classic() +
  theme(
    axis.title = element_text(size = 12, family = "Noto Sans", colour = "black"),
    legend.title = element_text(size = 12, family = "Noto Sans", colour = "black"),
    legend.text = element_text(size = 11, family = "Noto Sans", colour = "black"),
    legend.position = "bottom"
  )

p2 <- ggtexttable(
    data %>%
      filter(!is.na(pretreatment) & !is.na(treatment) &
               !is.na(mass) &
        !is.na(billLengthMean) & week <= 8 &
          pretreatment == "cold") %>%
      group_by(week) %>%
      count() %>%
      rename("Age (weeks)" = week),
    rows = NULL, theme = ttheme("light")
  ) %>%
    tab_add_title(text = "Cold\nRearing", face = "bold")

p3 <- ggtexttable(
      data %>%
        filter(!is.na(pretreatment) & !is.na(treatment) &
                 !is.na(mass) &
          !is.na(billLengthMean) & week <= 8 &
            pretreatment == "neutral") %>%
        group_by(week) %>%
        rename("Age (weeks)" = week) %>%
```

```
        count(),
      rows = NULL, theme = ttheme("light")
    ) %>%
      tab_add_title(text = "Mild\nRearing", face = "bold")

p4 <- ggtexttable(
        data %>%
          filter(!is.na(pretreatment) & !is.na(treatment) &
                   !is.na(mass) &
            !is.na(billLengthMean) & week <= 8 &
              pretreatment == "warm") %>%
          group_by(week) %>%
          rename("Age (weeks)" = week) %>%
          count(),
        rows = NULL, theme = ttheme("light")
      ) %>%
      tab_add_title(text = "Warm\nRearing", face = "bold")

residualBill/(p2 + p3 + p4)
```

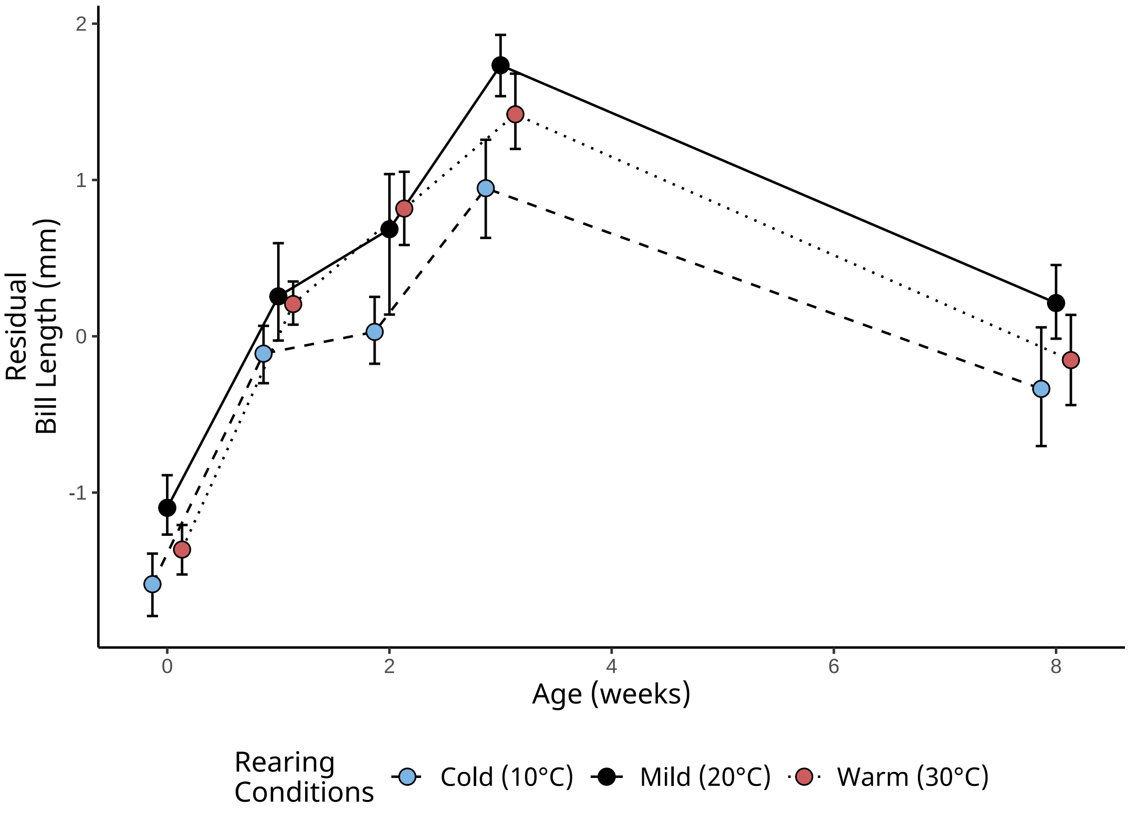

Cold Rearing	
Age (weeks)	n
0	20
1	25
2	22
3	43
8	42

Mild Rearing	
Age (weeks)	n
0	15
1	12
2	4
3	24
8	34

Warm Rearing	
Age (weeks)	n
0	40
1	36
2	26
3	30
8	36

Figure 26: *Effect of age and rearing conditions on relative bill length (mm) of Japanese quail. Relative bill length represents median residuals from a Bayesian linear model with bill length (mm) as as the response variable and body mass (g) as the sole predictor. Dots represent group means and errorbars represent 2.5% and 97.5% quantiles.*

Variance in morphological traits is calculated for reference, then our data-set saved.

```
caption = paste0('Average and variance of size metrics among ',
                 'captive-reared Japanese quail. "SD" indicates ',
                 'standard deviation and "CV" indicates ',
                 'coefficient of variation.')
```

```
data %>%
  filter(week <= 8) %>%
  dplyr::select(week, "tarsus" = tarsusLengthMean, "bill" = billLengthMean, mass) %>%
  pivot_longer(!week, names_to = "Metric", values_to = "Size") %>%
  group_by(week, Metric) %>%
  summarise(
    "Mean" = mean(Size, na.rm = T),
    "SD" = sd(Size, na.rm = T)
  ) %>%
  mutate(
    "CV" = (SD / Mean) * 100,
    Metric = ifelse(Metric == "tarsus",
      "Tarsus Length\n(mm)",
      ifelse(Metric == "bill",
        "Bill Length\n(mm)",
        "Body Mass\n(g)"
      )
    )
  ) %>%
  dplyr::select(Metric, "Age (Weeks)" = week, Mean, SD, "CV (\\%)" = CV) %>%
  arrange(Metric, `Age (Weeks)`) %>%
  kbl(.,
    longtable = T, booktabs = T,
    format = "latex", caption = caption,
    escape = FALSE
  ) %>%
  kable_styling()
```

Table 8: *Average and variance of size metrics among captive-reared Japanese quail. "SD" indicates standard deviation and "CV" indicates coefficient of variation.*

Metric	Age (Weeks)	Mean	SD	CV (%)
Bill Length (mm)	0	3.456283	0.4981479	14.412822
Bill Length (mm)	1	5.474596	0.5379482	9.826263
Bill Length (mm)	2	6.874798	0.7490349	10.895373
Bill Length (mm)	3	8.740267	0.8765772	10.029181
Bill Length (mm)	4	NaN	NA	NA
Bill Length (mm)	5	NaN	NA	NA
Bill Length (mm)	6	NaN	NA	NA
Bill Length (mm)	7	NaN	NA	NA
Bill Length (mm)	8	9.613149	0.7084885	7.369994
Body Mass (g)	0	8.970992	0.8800777	9.810260
Body Mass (g)	1	34.494697	5.7158720	16.570292
Body Mass (g)	2	82.934375	10.6426229	12.832584
Body Mass (g)	3	134.888189	16.8731900	12.509020
Body Mass (g)	4	180.074468	17.9338697	9.959141
Body Mass (g)	5	203.204237	21.2254137	10.445360
Body Mass (g)	6	230.534043	24.6884173	10.709228
Body Mass (g)	7	239.803419	28.6671232	11.954426
Body Mass (g)	8	243.977686	32.4864600	13.315341
Tarsus Length (mm)	0	12.856441	1.2539701	9.753634
Tarsus Length (mm)	1	22.482959	2.1655622	9.632016
Tarsus Length (mm)	2	30.769316	2.2390404	7.276861
Tarsus Length (mm)	3	34.976406	2.8274274	8.083814
Tarsus Length (mm)	4	NaN	NA	NA
Tarsus Length (mm)	5	NaN	NA	NA
Tarsus Length (mm)	6	NaN	NA	NA
Tarsus Length (mm)	7	NaN	NA	NA
Tarsus Length (mm)	8	36.779146	2.3363436	6.352360

```
# Saving compiled data-frame
```

```r
write.csv(data, "compiledDataFull.csv", row.names = F)
data <- read.csv("compiledDataFull.csv")
```

Modelling effects of the developmental thermal environment on morphology

Body mass

In this subsection, we evaluate the effect of the post-hatch thermal environment on body size (here, body mass in g) and appendage length (tarsus and bill length in mm) of captive-reared Japanese quail. As described above, this is achieved by modelling each variable as a Gompertz function of age in weeks, and testing for an effect of rearing condition on any, and all, parameters of the Gompertz curve (i.e. a, b, and c). For body mass, our model was therefore as follows:

$$Mass_{ij} \sim a \cdot e^{-b \cdot e^{-c \cdot Age_{ij}}} + \mu_{0j} + \epsilon_{ij}$$

$$a_{ij} \sim \beta_{a0} + \beta_{a1} \cdot Cold\ Reared_j + \beta_{a2} \cdot Warm\ Reared_j + \mu_{0aj}$$

$$b_{ij} \sim \beta_{b0} + \beta_{b1} \cdot Cold\ Reared_j + \beta_{b2} \cdot Warm\ Reared_j + \mu_{0bj}$$

$$c_{ij} \sim \beta_{c0} + \beta_{c1} \cdot Cold\ Reared_j + \beta_{c2} \cdot Warm\ Reared_j + \mu_{0cj}$$

where a, b, and c represent growth curve parameters implicit in the Gompertz function, *Cold Reared* and *Warmed Reared* represent logical, true/false variables per individual (with true equaling 1 and false equaling 0), β_x values represent model coefficients for growth curve parameters x (a - c), i represents an observation, j represents an individual, μ_0 represents a group-level intercept for individual j, μ_{0a} - μ_{0c} represent group-level intercepts corresponding to the batch of eggs from which an individual was derived from, and ϵ represents the model error structure. Because we expected the standard deviation of body mass to also increase with age, ϵ was therefore modeled as:

$$ln(\epsilon_{ij}) \sim \tau_0 + \tau_1 * ln(Age_{ij} + 1)$$

where τ_0 represents the intercept for ϵ, and τ_1 represents the rate at which ϵ, or its natural logarithm, increases with the natural logarithm of an individual's age in weeks plus 1 (i.e. to limit week 0 being equal to negative infinity).

Priors for all model parameters were moderately informative and selected according to findings by Narinc et al (2010), Burness et al (2013),Haqani et al (2021), and Persson et al (2024). Specifically, our prior for the asymptote of our growth curve (β_{a0}) was normally distributed with a mean of 250 and standard deviation of 25, while that for our x-axis displacement (β_{b0}) was normally distributed with mean of 3 and standard deviation of 1 (i.e. in line with expectations from Narinc et al. 2010). For our growth rate (β_{c0}), we used a skew-normal prior with ξ set to 0.5, ω set to 0.1, and α set to 2.5 (thus assuming that growth rate could not be < 0, and should lay near 0.5). Priors for the effect of rearing condition on growth parameters a, b, and c (i.e. β_{a1-2}, β_{b1-2}, and β_{c1-2} respectively) were all normally distributed. Here, for effects of cold-rearing and warm-rearing on a, we assumed means of 7.5 and -7.5 respectively, given that quail reared at 15°C were approximately 15 g heavier that those reared at 30°C by 66 days in Burness et al (2013); standard deviations were, however, set broadly to 25. Others treatment-specific effects were assigned means of 0 and standard deviations 0.5 (β_{b1-2}), and 0.2 (β_{c1-2}) respectively. For our group-level effects of egg batch, we used exponential priors with lambda values determined from preliminary plots (μ_{0a}: $\lambda = 2.5$; μ_{0b}: $\lambda = 10$; μ_{0c}: $\lambda = 25$). Similarly, for our group-level effect of bird identity, we also used an exponential prior, however, with lambda set broadly to 0.5. Finally, skew-normal priors were used for our error structure parameters τ_0 and τ_1 with ξ values of 1, ω values of 0.5, and α values of 10 and -10 respectively.

For this model, 4 Hamiltonian Monte Carlo (HMC) chains were used, with each run for 50000 iterations and 10000 warm-up iterations, then sampled every 10 iterations. Suitability of priors is evaluated by a prior predictive check below.

First, we check that the standard deviation of body mass does indeed increase with age as anticipated.

```
data %>%
  filter(week <= 8) %>%
  mutate(pretreatment = factor(pretreatment,
    levels = c("neutral", "cold", "warm")
  )) %>%
  select(ring, pretreatment, week, mass) %>%
  distinct() %>%
  mutate(weekB = log(week + 1)) %>%
  group_by(week, weekB) %>%
  summarise(SD = log(sd(mass, na.rm = T))) %>%
  ungroup() %>%
  ggplot(aes(x = weekB, y = SD)) +
  geom_point(size = 3, pch = 21, colour = "black", fill = "grey50") +
  geom_smooth(method = "lm", colour = "black",
              linetype = "dashed", se = FALSE) +
  xlab("Age (Weeks + 1; Natural-log Transformed )") +
  ylab("Standard Deviation of Body Mass (Natural-log Transformed)") +
  theme_classic()
```

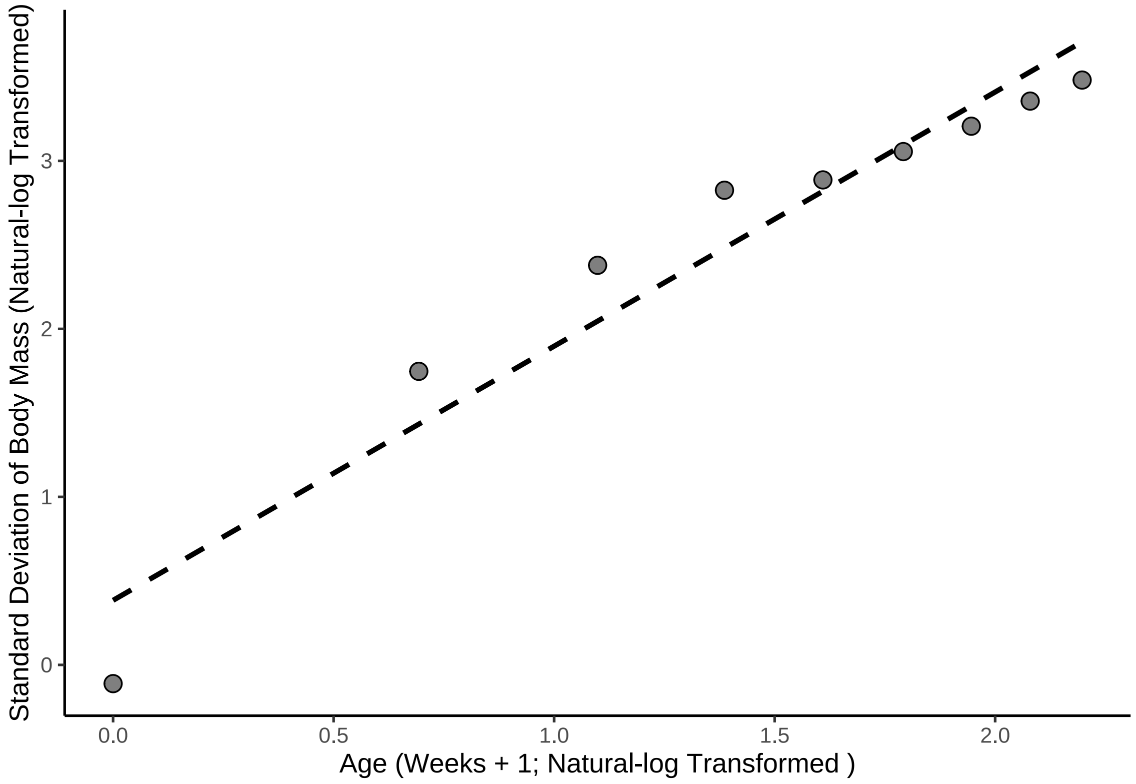

Figure 27: *Effect of natural log-transformed age (in weeks + 1) on the natural log-transformed standard deviation of body mass (g). Dotted line represents a line of body best fit estimated from a linear relationship by internal functions of the R package ggplot (Wickham, 2011).*

```
# Reasonably linear.
```

Our prior predictive check is then produced.

```
growthModel_priorCheck <- brm(
  data = data %>%
    filter(week <= 8) %>%
    mutate(pretreatment = factor(pretreatment,
                                 levels = c("neutral", "cold", "warm")
    )) %>%
```

```
    dplyr::select(ring, pretreatment, week, mass, "batch" = exp) %>%
    distinct() %>%
    mutate(weekB = week + 1),
  formula = bf(mass ~ A * exp(-B * exp(-C * week)) + D,
               A ~ 1 + pretreatment + (1|batch),
               B ~ 1 + pretreatment + (1|batch),
               C ~ 1 + pretreatment + (1|batch),
               D ~ 0 + (1 | ring),
               sigma ~ 0 + intercept + log(weekB),
               nl = TRUE
  ),
  prior = c(
    set_prior("normal(250, 25)",
              class = "b", coef = "Intercept",
              nlpar = "A"
    ),
    set_prior("normal(7.5, 25)",
              class = "b", coef = "pretreatmentcold",
              nlpar = "A"
    ),
    set_prior("normal(-7.5, 25)",
              class = "b", coef = "pretreatmentwarm",
              nlpar = "A"
    ),
    set_prior("exponential(2.5)", class = "sd",
              coef = "Intercept",
              group = "batch", nlpar = "A"
    ),
    set_prior("normal(3, 1)",
              class = "b", coef = "Intercept",
              nlpar = "B"
    ),
    set_prior("normal(0, 0.5)",
              class = "b", coef = "pretreatmentcold",
              nlpar = "B"
    ),
    set_prior("normal(0, 0.5)",
              class = "b", coef = "pretreatmentwarm",
              nlpar = "B"
    ),
    set_prior("exponential(10)", class = "sd",
              coef = "Intercept", group = "batch",
              nlpar = "B"
    ),
    set_prior("skew_normal(0.5, 0.1, 2.5)",
              class = "b", coef = "Intercept",
              nlpar = "C"
    ),
    set_prior("normal(0, 0.2)",
              class = "b", coef = "pretreatmentcold",
              nlpar = "C"
    ),
    set_prior("normal(0, 0.2)",
              class = "b", coef = "pretreatmentwarm",
              nlpar = "C"
    ),
    set_prior("exponential(25)", class = "sd",
              coef = "Intercept", group = "batch",
              nlpar = "C"
    ),
    set_prior("exponential(0.5)", class = "sd",
              coef = "Intercept", group = "ring", nlpar = "D"),
    set_prior("skew_normal(1, 0.5, -10)", class = "b",
              coef = "intercept", dpar = "sigma"),
    set_prior("skew_normal(1, 0.5, 10)", class = "b",
              coef = "logweekB", dpar = "sigma")
  ),
```

```
  family = "gaussian",
  seed = 100,
  cores = 4, chains = 4,
  iter = 50000, warmup = 10000, thin = 10,
  control = list(adapt_delta = 0.98, max_treedepth = 16),
  sample_prior = "only",
  silent = TRUE, refresh = 0,
  file = "./models/_ppCheckMass.Rds"
)

pp_check2(growthModel_priorCheck, xlab = "Body Mass (g)") +
  xlim(c(0, 1000))
```

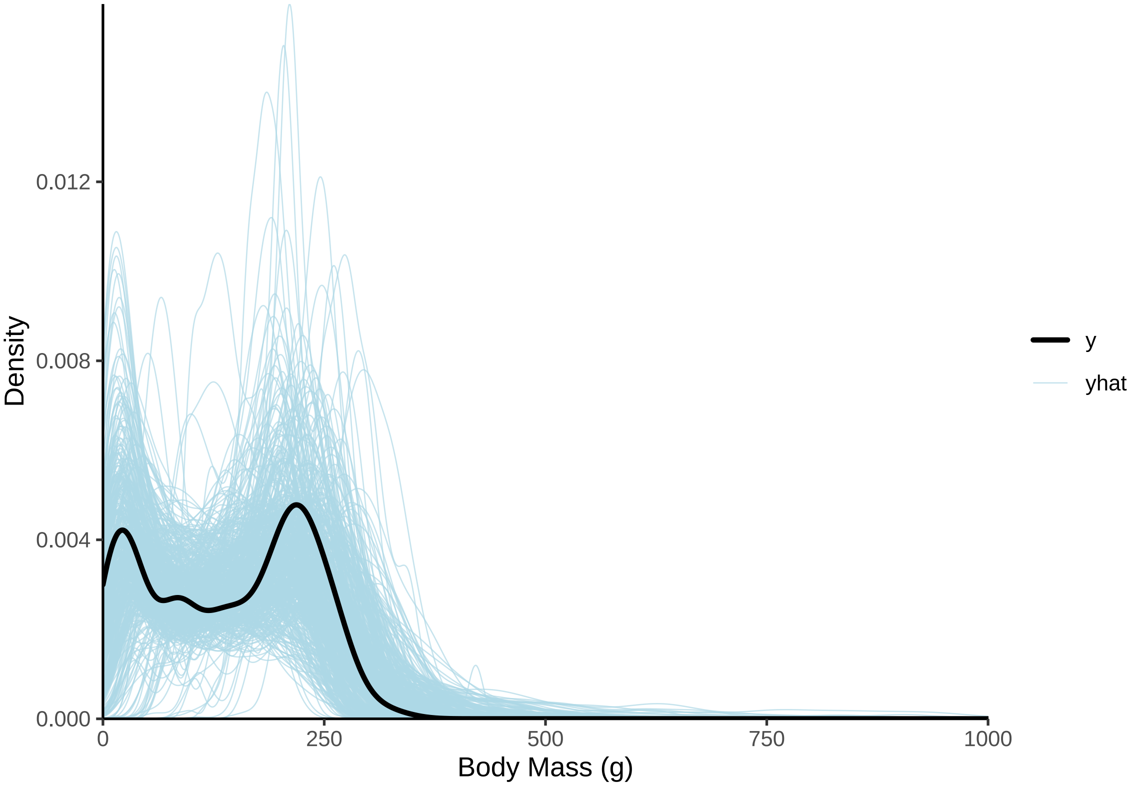

Figure 28: *Overlay of predicted (blue) and true (black) body mass densities, where predicted densities are derived from priors in a Bayesian non-linear model. Clear overlap betwen the black and blue lines suggests that model priors are reasonable with respect to the data.*

```
# Some extreme values permitted, although reasonable overlay.
```

To guide our HMC chains for our full model, initial values are selected from within prior distributions.

```
# Acceptable prior selection. Creating function from
# which to draw initial HMC chain values.

initFunction <- function(chain_id = 1) {
  list(
    "b_A" = c(rnorm(1, 250, 25), 7.5, -7.5),
    "b_B" = c(3, 0, 0),
    "b_C" = c(0.5, 0, 0),
    "b_sigma" = c(rskew_normal(1, xi = 1, omega = 0.5, alpha = -5), 1),
    "sd_1" = rexp(1, 2.5),
    "sd_2" = rexp(1, 10),
    "sd_3" = rexp(1, 25),
```

```
    "sd_4" = rexp(1, 0.5)
  )
}

initList <- lapply(1:4, initFunction)

growthModel <- brm(
  data = data %>%
    filter(week <= 8) %>%
    mutate(pretreatment = factor(pretreatment,
                                 levels = c("neutral", "cold", "warm")
    )) %>%
    dplyr::select(ring, pretreatment, week, mass, "batch" = exp) %>%
    distinct() %>%
    mutate(weekB = week + 1),
  formula = bf(mass ~ A * exp(-B * exp(-C * week)) + D,
               A ~ 1 + pretreatment + (1|batch),
               B ~ 1 + pretreatment + (1|batch),
               C ~ 1 + pretreatment + (1|batch),
               D ~ 0 + (1 | ring),
               sigma ~ 0 + intercept + log(weekB),
               nl = TRUE
  ),
  prior = c(
    set_prior("normal(250, 25)",
              class = "b", coef = "Intercept",
              nlpar = "A"
    ),
    set_prior("normal(7.5, 25)",
              class = "b", coef = "pretreatmentcold",
              nlpar = "A"
    ),
    set_prior("normal(-7.5, 25)",
              class = "b", coef = "pretreatmentwarm",
              nlpar = "A"
    ),
    set_prior("exponential(2.5)", class = "sd",
              coef = "Intercept",
              group = "batch", nlpar = "A"
    ),
    set_prior("normal(3, 1)",
              class = "b", coef = "Intercept",
              nlpar = "B"
    ),
    set_prior("normal(0, 0.5)",
              class = "b", coef = "pretreatmentcold",
              nlpar = "B"
    ),
    set_prior("normal(0, 0.5)",
              class = "b", coef = "pretreatmentwarm",
              nlpar = "B"
    ),
    set_prior("exponential(10)", class = "sd",
              coef = "Intercept", group = "batch",
              nlpar = "B"
    ),
    set_prior("skew_normal(0.5, 0.1, 2.5)",
              class = "b", coef = "Intercept",
              nlpar = "C"
    ),
    set_prior("normal(0, 0.2)",
              class = "b", coef = "pretreatmentcold",
              nlpar = "C"
    ),
    set_prior("normal(0, 0.2)",
              class = "b", coef = "pretreatmentwarm",
              nlpar = "C"
```

```
    ),
    set_prior("exponential(25)", class = "sd",
              coef = "Intercept", group = "batch",
              nlpar = "C"
    ),
    set_prior("exponential(0.5)", class = "sd",
              coef = "Intercept", group = "ring", nlpar = "D"),
    set_prior("skew_normal(1, 0.5, -10)", class = "b",
              coef = "intercept", dpar = "sigma"),
    set_prior("skew_normal(1, 0.5, 10)", class = "b",
              coef = "logweekB", dpar = "sigma")
  ),
  family = "gaussian",
  init = initList,
  seed = 100,
  cores = 4, chains = 4, threads = 2,
  iter = 50000, warmup = 10000, thin = 10,
  backend = "cmdstan",
  control = list(adapt_delta = 0.98, max_treedepth = 16),
  silent = TRUE, refresh = 0,
  file = "./models/_growthModelMass.Rds"
)

# Very few divergences (~0.01%). Checking chain mixing and chain autocorrelation

ggarrange(
  ggplot(data = data.frame("Rhat" = brms::rhat(growthModel)),
         aes(x = Rhat)) +
    geom_density() +
    theme_classic() +
    xlab(
      TeX('$\\hat{R}$')
    ) +
    ylab("Density"),
  ggplot(data = data.frame("Neff" = neffBase(growthModel)),
         aes(x = Neff)) +
    geom_density() +
    theme_classic() +
    xlab(
      TeX('$N_{eff}/N~Ratio$')
      ) +
    ylab("Density")
)
```

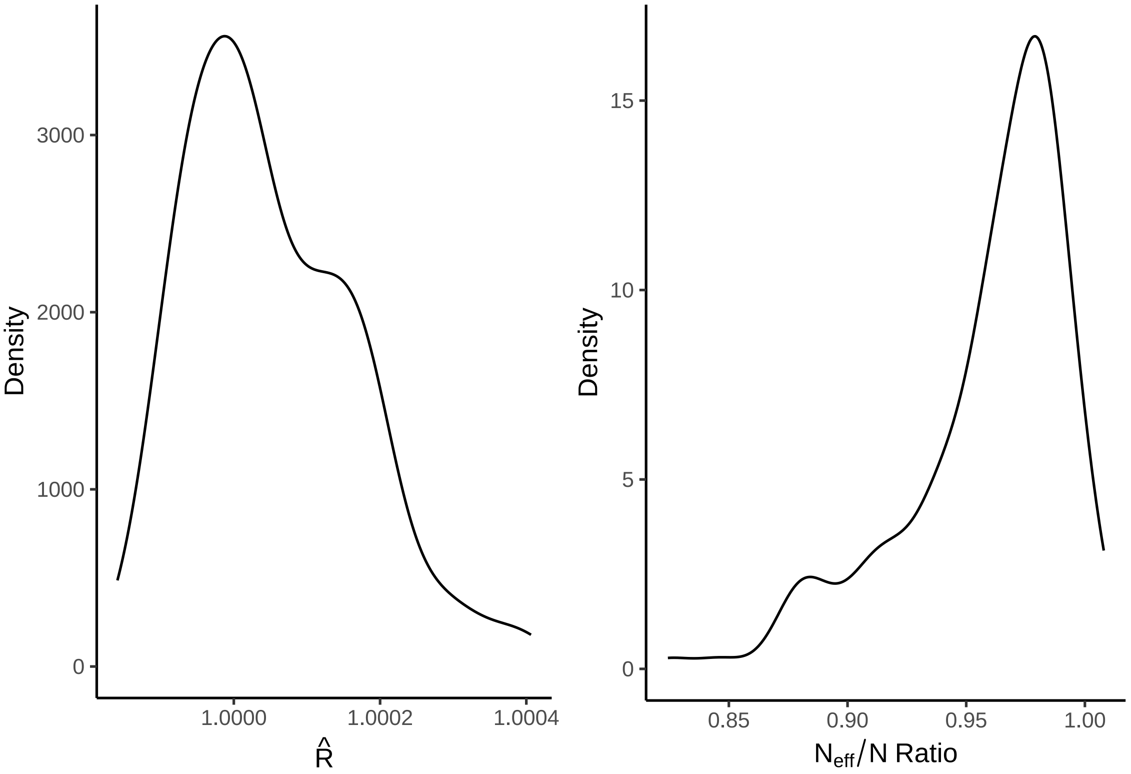

Figure 29: *Gelman-Rubin statistics ($\hat{R}$) and ratio of effective samples sizes by samples sizes per parameter from a Bayesian non-linear model estimating growth of mass (g) among Japanese quail.*

No obvious autocorrelation is detected in our HMC chains, and all appear well converged. We next visualise our posterior predictions.

```
pp_check2(growthModel, xlab = "Body Mass (g)")
```

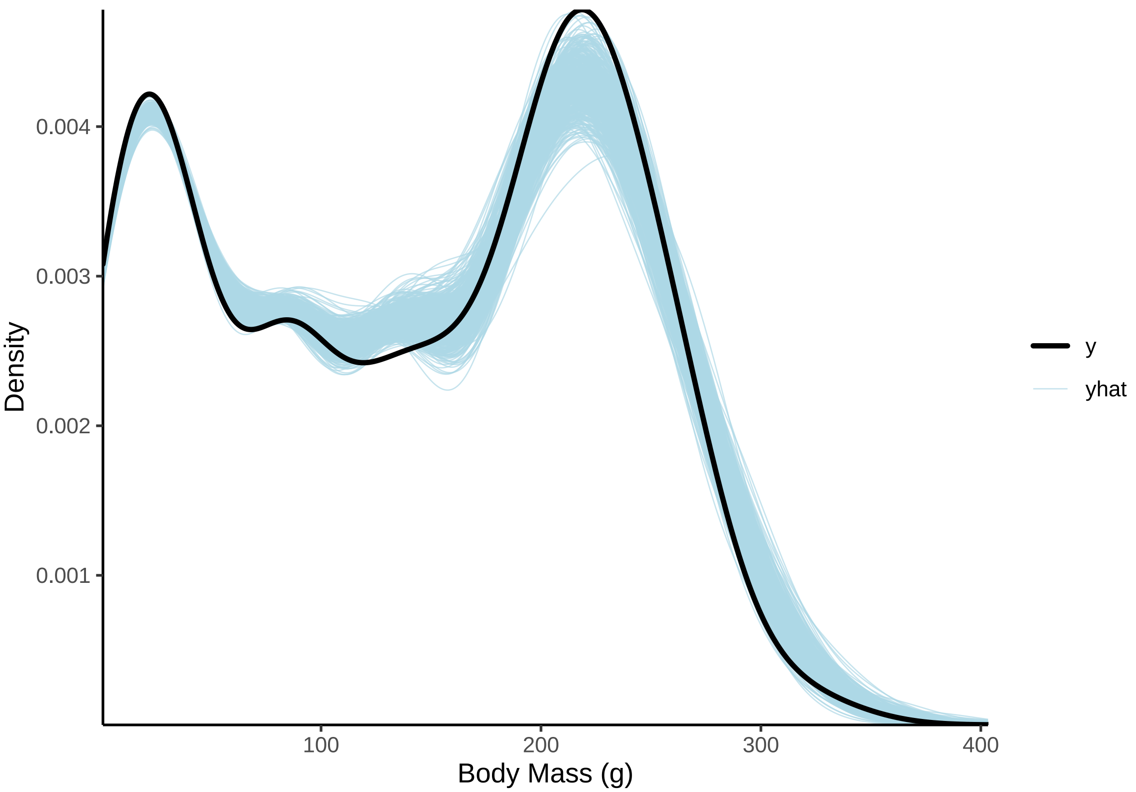

Figure 30: *Posterior predictions of a Bayesian non-linear model predicting Japanese quail mass (g) across time, overlayed with true distributions of quail mass. Blue lines represent predictions from posterior draws, while the black line represents true mass distributions.*

```
# Clear overlay. Checking fitted values using a scatter-plot.
```

```
growthModel$data %>%
  mutate("Fit" = predict(growthModel)[,"Estimate"]) %>%
  ggplot(aes(x = mass, y = Fit, fill = week)) +
  geom_point(pch = 21, colour = "black", size = 2, alpha = 0.5) +
  geom_smooth(method = "lm", colour = "black",
              linetype = "dashed", se = FALSE) +
  scale_fill_gradient2(name = "Age (Weeks)") +
  theme_classic() +
  xlab("Body Mass (g)") +
  ylab("Expected Body Mass (g)")
```

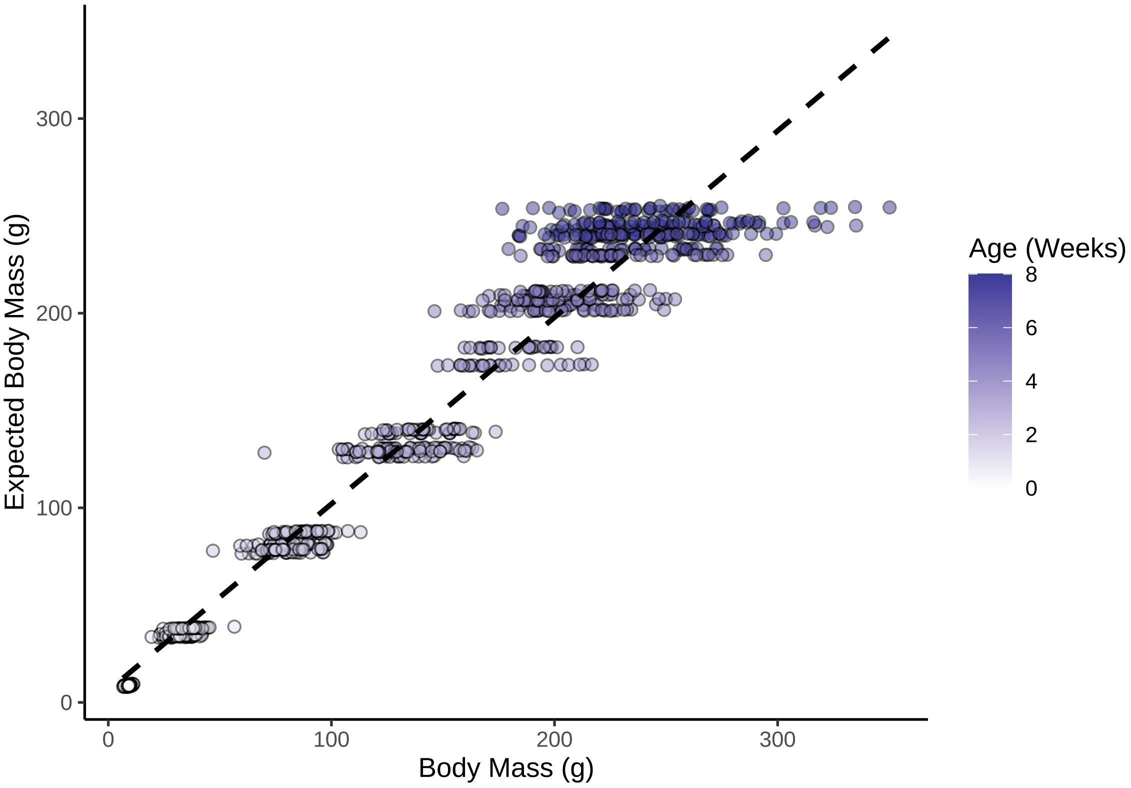

Figure 31: *Scatterplot of Japanese quail body mass (g) against predicted body masses (g) from a Bayesian non-linear model.*

```
with(
  growthModel$data,
  expand.grid(
    "week" = seq(0, 8, by = 0.1),
    "pretreatment" = c("cold", "neutral", "warm")
  )
) %>%
  mutate("weekB" = week + 1) %>%
  mutate(
    "Fit" = predict(growthModel, re_form = NA, robust = TRUE,
                    newdata = .)[, "Estimate"],
    "SE" = predict(growthModel, re_form = NA, robust = TRUE,
                   newdata = .)[, "Est.Error"]
  ) %>%
  mutate(
    "LCL" = Fit - SE,
    "UCL" = Fit + SE
  ) %>%
  mutate(pretreatment = str_to_title(pretreatment)) %>%
  mutate(pretreatment = factor(pretreatment,
                               levels = c("Cold", "Neutral", "Warm"))) %>%
  ggplot(aes(x = week, y = Fit, fill = pretreatment,
             linetype = pretreatment)) +
  # facet_wrap(~sex) +
  geom_ribbon(aes(x = week, ymin = LCL, ymax = UCL),
    colour = NA, alpha = 0.3
  ) +
  geom_line(colour = "black", alpha = 0.7) +
  stat_summary(
    data = growthModel$data %>%
      mutate(pretreatment = str_to_title(pretreatment)) %>%
      mutate(pretreatment = factor(pretreatment,
```

```
                                      levels = c("Cold", "Neutral", "Warm"))),
    aes(x = week, y = mass),
    geom = "errorbar", fun.data = "mean_se",
    colour = "black", alpha = 0.7, width = 0.25,
    position = position_dodge(width = 0.15)
  ) +
  stat_summary(
    data = growthModel$data %>%
      mutate(pretreatment = str_to_title(pretreatment)) %>%
      mutate(pretreatment = factor(pretreatment,
                                      levels = c("Cold", "Neutral", "Warm"))),
    aes(x = week, y = mass),
    geom = "point", fun = "mean", pch = 21, size = 3,
    colour = "black", alpha = 0.7,
    position = position_dodge(width = 0.15)
  ) +
  theme_classic() +
  scale_fill_manual(values = c("#7BB4E3", "black", "#CD5C5C"),
                    name = "Rearing\nConditions",
                    labels = c("Cold (10°C)", "Mild (20°C)", "Warm (30°C)")) +
  scale_linetype_manual(values = c("dotted", "solid", "dashed"),
                        name = "Rearing\nConditions",
                    labels = c("Cold (10°C)", "Mild (20°C)", "Warm (30°C)")) +
  xlab("Age (weeks)") +
  ylab("Body Mass (g)")
```

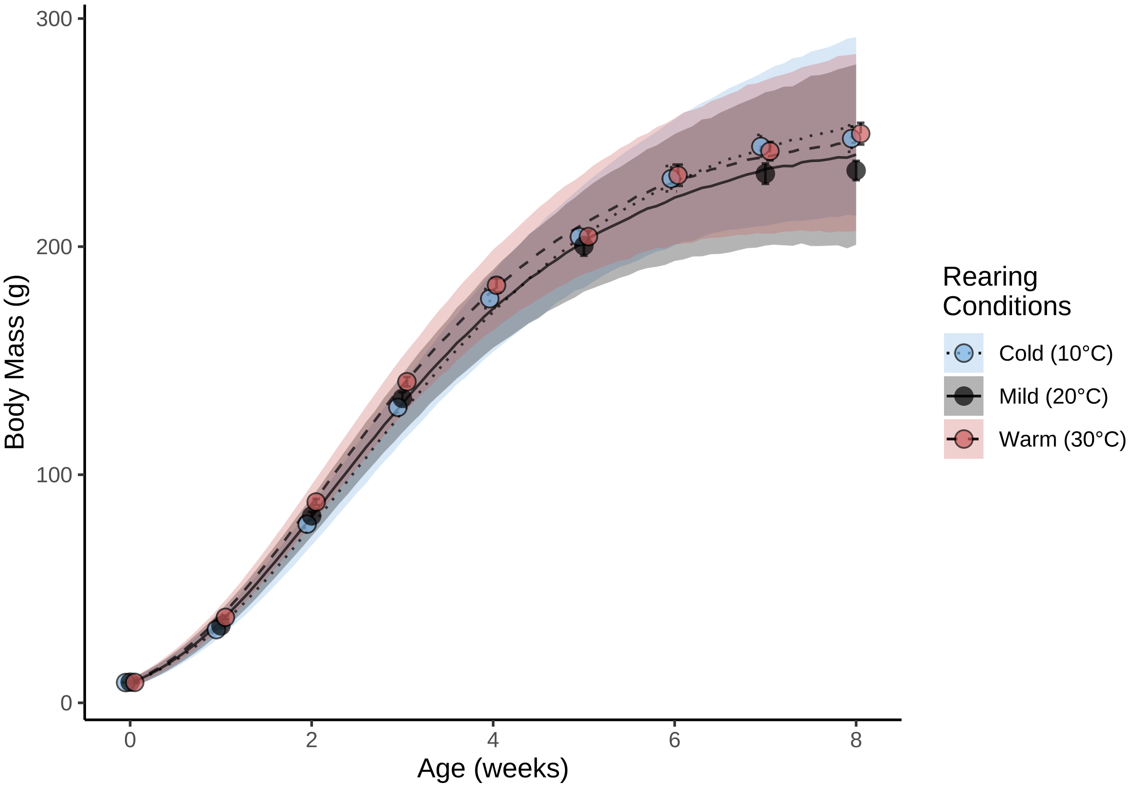

Figure 32: *Body mass (g) growth curves of Japanese quail reared in the cold (10°C), mild conditions (20°C), or the warm (30°C) until at least 3 weeks of age. Dots represent mean values and errorbars represent standard errors around means. Lines represent estimated trends in growth from a Bayesian non-linear model, while ribbons represent confidence around trends (± one standard error).*

```
# Proceeding with further model checking,
# beginning with assessment of residual distributions.
```

Below, we proceed by checking residual distributions.

```
p1 <- growthModel$data %>%
  mutate("residuals" =
           residuals(growthModel,
                     type = "pearson",
                     robust = TRUE)[, "Estimate"]) %>%
  ggplot(aes(x = residuals)) +
  geom_density(colour = "black", fill = "white") +
  xlab("Pearson Residuals") +
  ylab("Density") +
  theme_classic()

p2 <- growthModel$data %>%
  mutate(
    "residuals" = residuals(growthModel,
      type = "pearson",
      robust = TRUE
    )[, "Estimate"],
    "fitted" = fitted(growthModel,
                      robust = TRUE)[, "Estimate"]
  ) %>%
  ggplot(aes(x = fitted, y = residuals)) +
  geom_point(colour = "black", pch = 21,
             size = 2, fill = "grey75", alpha = 0.5) +
  ylab("Pearson Residuals") +
  xlab("Fitted Values (g)") +
  theme_classic()

p3 <- growthModel$data %>%
  mutate("residuals" = residuals(growthModel,
    type = "pearson",
    robust = TRUE
  )[, "Estimate"]) %>%
  ggplot(aes(x = week, y = residuals)) +
  geom_point(
    colour = "black", pch = 21, size = 2,
    fill = "grey75", alpha = 0.5,
    position = position_jitter(width = 0.25)
  ) +
  stat_summary(geom = "errorbar", fun.data = "mean_se",
               colour = "black", width = 0.25) +
  stat_summary(geom = "point", fun = "mean", pch = 21,
               colour = "black", fill = "white", size = 4) +
  ylab("Pearson Residuals") +
  xlab("Age (weeks)") +
  theme_classic()

p4 <- growthModel$data %>%
  mutate("residuals" = residuals(growthModel,
    type = "pearson",
    robust = TRUE
  )[, "Estimate"]) %>%
  mutate(pretreatment = str_to_title(pretreatment)) %>%
  mutate(pretreatment = ifelse(pretreatment == "Cold", "Cold\n(10°C)",
    ifelse(pretreatment == "Neutral", "Mild\n(20°C)",
      "Warm\n(30°C)"
    )
  )) %>%
  mutate(pretreatment = factor(pretreatment,
                               levels = c("Cold\n(10°C)",
                                          "Mild\n(20°C)",
                                          "Warm\n(30°C)"))
        ) %>%
  ggplot(aes(x = pretreatment, y = residuals)) +
  geom_point(
    colour = "black", pch = 21, size = 2, fill = "grey75", alpha = 0.5,
```

```
    position = position_jitter(width = 0.25)
  ) +
  stat_summary(geom = "errorbar", fun.data = "mean_se",
               colour = "black", width = 0.25) +
  stat_summary(geom = "point", fun = "mean", pch = 21,
               colour = "black", fill = "white", size = 4) +
  ylab("Pearson Residuals") +
  xlab("Rearing Conditions)") +
  theme_classic()

(p1 + p2) / (p3 + p4)
```

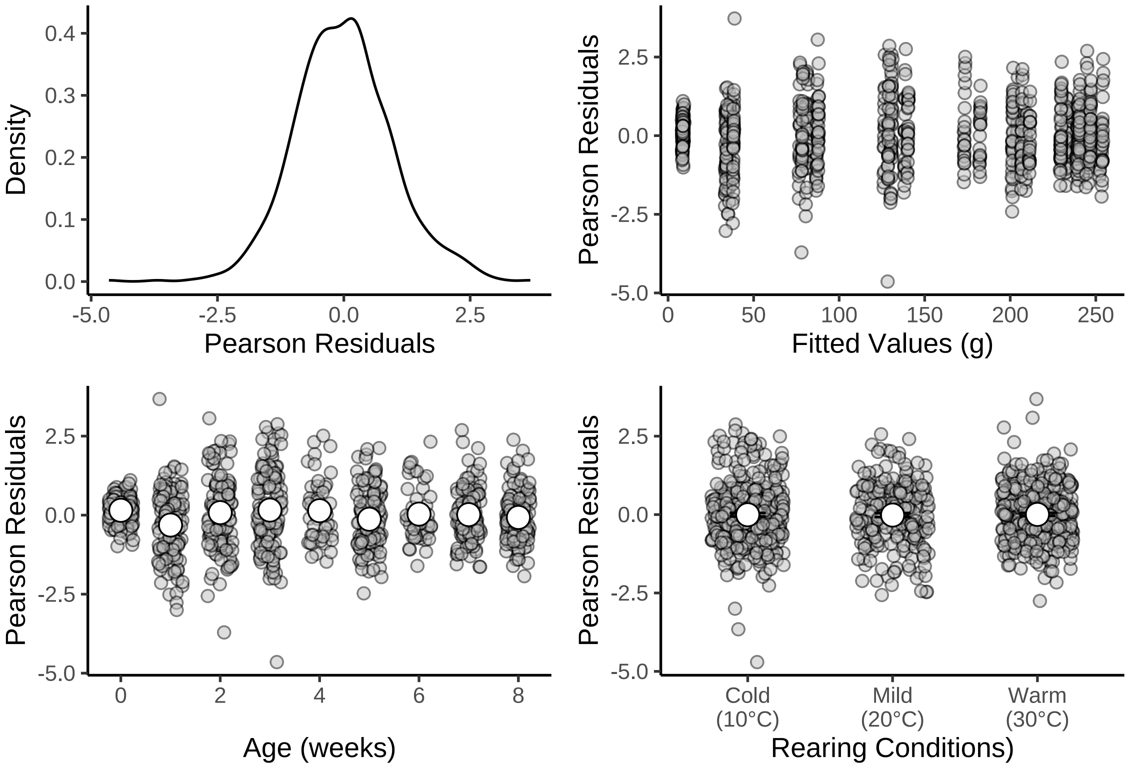

Figure 33: *Density and distributions of Pearson residuals across fitted values and model predictors (including age and rearing conditions). All residuals pertain to those drawn from a Bayesian non-linear model predicting body mass (g) during growth in Japanese quail. Pearson residuals are shown rather than ordinary residuals to correct for the age-dependence of model error.*

```
# No heteroskedasticity and residuals appear normal, albeit large. Plotting model outcomes.
```

Our model outcomes are next visualised.

```
as.data.frame(growthModel) %>%
  pivot_longer(everything(), names_to = "Parameter", values_to = "Values") %>%
  filter(grepl("b_|sd_", Parameter)) %>%
  arrange(Parameter) %>%
  merge(., tribble(
    ~Parameter, ~Par,
    "b_A_Intercept", "Beta a0",
    "b_A_pretreatmentcold", "Beta a1\n(Cold-reared)",
    "b_A_pretreatmentwarm", "Beta a2\n(Warm-reared)",
    "sd_batch__A_Intercept", "Mu 0a",
    "b_B_Intercept", "Beta b0",
    "b_B_pretreatmentcold", "Beta b1\n(Cold-reared)",
```

```
    "b_B_pretreatmentwarm", "Beta b2\n(Warm-reared)",
    "sd_batch__B_Intercept", "Mu 0b",
    "b_C_Intercept", "Beta c0",
    "b_C_pretreatmentcold", "Beta c1\n(Cold-reared)",
    "b_C_pretreatmentwarm", "Beta c2\n(Warm-reared)",
    "sd_batch__C_Intercept", "Mu 0c",
    "sd_ring__D_Intercept", "Mu 0\n(Individual Intercept)",
    "b_sigma_intercept", "Tau 0",
    "b_sigma_logweekB", "Tau 1"
  ),
  by = "Parameter", all.x = TRUE
  ) %>%
  ggplot(aes(x = Values)) +
  facet_wrap(~Par, scales = "free") +
  geom_density() +
  geom_vline(xintercept = 0, linetype = "dashed",
             colour = "firebrick4") +
  scale_x_continuous(n.breaks = 3) +
  ylab("Density") +
  theme_classic() +
  theme(axis.title.x = element_blank())
```

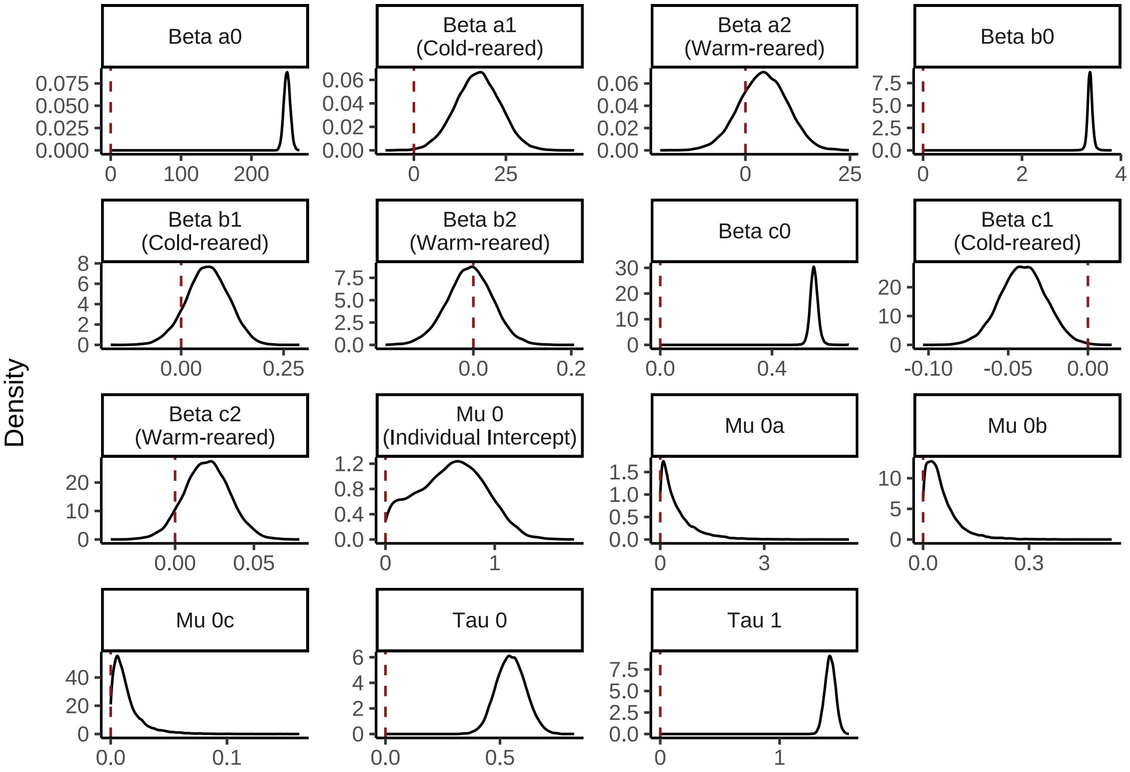

Figure 34: *Density of model coefficients from a Bayesian non-linear model predicting body mass (g) of Japanese quail during growth. Dashed red lines indicate zero values for each coefficient.*

```
# Posterior densities are generally normal, however,
# given that some stray, summarising model coefficients
# by their medians and quantile intervals.

caption <- paste0("Coefficients from a Bayesian non-linear ",
  "effects model predicting body mass of Japanese ",
  "quail as a Gompertz function of age in weeks. ",
  "Coefficients represent posterior medians and are ",
  "estimated from body mass data collected weekly ",
  "between 0 and 8 weeks of age. Credible intervals ",
```

```
  "(CIs) represent quantile intervals."
)

growthModelMassTable <-
  merge(
    as.data.frame(growthModel) %>%
      summarise_all(., .funs = median) %>%
      mutate_all(., .funs = round, 3) %>%
      pivot_longer(everything(), names_to = "Parameter",
                   values_to = "Values") %>%
      filter(grepl("b_|sd_", Parameter)) %>%
      arrange(Parameter),
    quantileCIs(growthModel, cis = c(50, 95)) %>%
    mutate(
    `50\\% CIs` = paste0("[", round(Low_CI_50, digits = 3),
                         ", ", round(High_CI_50, digits = 3), "]"),
    `95\\% CIs` = paste0("[", round(Low_CI_95, digits = 3),
                         ", ", round(High_CI_95, digits = 3), "]")
    ) %>%
      select(Parameter, `50\\% CIs`, `95\\% CIs`),
      by = "Parameter"
  ) %>%
  merge(., tribble(
    ~Parameter, ~Par,
    "b_A_Intercept", "Beta a0",
    "b_A_pretreatmentcold", "Beta a1 Cold-reared)",
    "b_A_pretreatmentwarm", "Beta a2 (Warm-reared)",
    "sd_batch__A_Intercept", "Mu 0a",
    "b_B_Intercept", "Beta b0",
    "b_B_pretreatmentcold", "Beta b1 (Cold-reared)",
    "b_B_pretreatmentwarm", "Beta b2 (Warm-reared)",
    "sd_batch__B_Intercept", "Mu 0b",
    "b_C_Intercept", "Beta c0",
    "b_C_pretreatmentcold", "Beta c1 (Cold-reared)",
    "b_C_pretreatmentwarm", "Beta c2 (Warm-reared)",
    "sd_batch__C_Intercept", "Mu 0c",
    "sd_ring__D_Intercept", "Mu (Individual Intercept)",
    "b_sigma_intercept", "Tau 0",
    "b_sigma_logweekB", "Tau 1"
  ),
  by = "Parameter", all.x = TRUE
  ) %>%
  select(-Parameter) %>%
  select("Parameter" = Par, "Value" = Values,
         `50\\% CIs`, `95\\% CIs`) %>%
  kbl(., longtable = T, booktabs = T, format = "latex",
      caption = caption, escape = FALSE) %>%
  column_spec(column = c(1, 4), width = "2.5cm") %>%
  kable_styling(latex_options = "striped")

growthModelMassTable
```

Table 9: *Coefficients from a Bayesian non-linear effects model predicting body mass of Japanese quail as a Gompertz function of age in weeks. Coefficients represent posterior medians and are estimated from body mass data collected weekly between 0 and 8 weeks of age. Credible intervals (CIs) represent quantile intervals.*

Parameter	Value	50% CIs	95% CIs
Beta a0	250.615	[247.625, 253.615]	[242.067, 259.597]
Beta a1 Cold-reared)	17.441	[13.346, 21.479]	[5.489, 29.133]
Beta a2 (Warm-reared)	4.306	[0.448, 8.141]	[-6.942, 15.115]
Beta b0	3.375	[3.345, 3.406]	[3.276, 3.487]
Beta b1 (Cold-reared)	0.065	[0.03, 0.099]	[-0.039, 0.162]

Beta b2 (Warm-reared)	-0.007	[-0.038, 0.024]	[-0.1, 0.082]
Beta c0	0.550	[0.542, 0.56]	[0.523, 0.581]
Beta c1 (Cold-reared)	-0.041	[-0.05, -0.031]	[-0.069, -0.013]
Beta c2 (Warm-reared)	0.020	[0.01, 0.03]	[-0.008, 0.049]
Tau 0	0.547	[0.504, 0.591]	[0.427, 0.673]
Tau 1	1.424	[1.394, 1.453]	[1.339, 1.508]
Mu 0a	0.319	[0.129, 0.667]	[0.011, 1.902]
Mu 0b	0.040	[0.02, 0.069]	[0.002, 0.178]
Mu 0c	0.010	[0.005, 0.018]	[0.001, 0.051]
Mu (Individual Intercept)	0.611	[0.371, 0.82]	[0.044, 1.176]

```
#save_kable(growthModelMassTable, "../tables/growthModelMassTable.html")
```

Testing effects of rearing condition on growth curve parameters From our model, it is evident that cold-reared individuals (i.e. those raised at 10°C for at least their first three weeks of life) have, on average, a higher asymptote (value for a) than those of both warm-reared (30°C for at least 3 weeks of life) and mild-reared (constant 20°C) individuals. By contrast, the growth rate of cold-reared individuals is lower than warm-reared and mild-reared individuals alike. To test these distinctions formally, we calculated the ratio of probabilities that one asymptote or growth rate (i.e. from one experimental treatment group) was indeed larger than another, as indicated from our model.

```
caption <- paste0("Coefficients from a Bayesian non-linear ",
                  "effects model predicting body mass of ",
                  "Japanese quail as a Gompertz function of ",
                  "age in weeks. Coefficients represent ",
                  "posterior medians and are estimated from ",
                  "body mass data collected weekly between ",
                  "0 and 8 weeks of age. Credible ",
                  "intervals (CIs) represent quantile ",
                  "intervals around medians."
)

hypotheses <- c(
  paste0(
    "(", c("A", "B"), "_Intercept + ", c("A", "B"),
    "_pretreatmentcold) - ", c("A", "B"), "_Intercept > 0"
  ),
  "((C_Intercept+C_pretreatmentcold)-C_Intercept) < 0",
  paste0(
    "(", c("A", "B", "C"), "_Intercept + ", c("A", "B", "C"),
    "_pretreatmentwarm) - ", c("A", "B", "C"), "_Intercept > 0"
  ),
  paste0(
    "(", c("A", "B"), "_Intercept + ", c("A", "B"),
    "_pretreatmentcold) - (", c("A", "B"), "_Intercept + ",
    c("A", "B"), "_pretreatmentwarm) > 0"
  ),
  "(C_Intercept + C_pretreatmentcold) - (C_Intercept + C_pretreatmentwarm) < 0"
)

layHypotheses <- c(
  "Cold-reared Asymptote (a) > Mild-reared Asymptote (a)",
  "Cold-reared Displacement (b) > Mild-reared Displacement (b)",
  "Cold-reared Growth Rate (c) < Mild-reared Growth Rate (c)",
  "Warm-reared Asymptote (a) > Mild-reared Asymptote (a)",
  "Warm-reared Displacement (b) > Mild-reared Displacement (b)",
  "Warm-reared Growth Rate (c) > Mild-reared Growth Rate (c)",
  "Cold-reared Asymptote (a) > Warm-reared Asymptote (a)",
  "Cold-reared Displacement (b) > Warm-reared Displacement (b)",
```

```
  "Cold-reared Growth Rate (c) < Warm-reared Growth Rate (c)"
)

pairwiseTableGrowthMass <- left_join(
  bind_rows(lapply(hypotheses, FUN = function(x) {
    hypothesis(growthModel, hypothesis = x, class = "b", robust = TRUE)$hypothesis
  })) %>% mutate(`95\\% CIs` = paste0(
      "[",
      round(CI.Lower, digits = 3),
      ", ",
      round(CI.Upper, digits = 3),
      "]"
    )) %>%
    dplyr::select(
      Hypothesis, "Delta" = Estimate, `95\\% CIs`, "Evidence Ratio" = Evid.Ratio
      ),
  bind_rows(lapply(hypotheses, FUN = function(x) {
    hypothesis(growthModel,
      hypothesis = x, class = "b",
      robust = TRUE, alpha = 0.2
    )$hypothesis
  })) %>% mutate(`50\\% CIs` = paste0(
      "[",
      round(CI.Lower, digits = 3),
      ", ",
      round(CI.Upper, digits = 3),
      "]"
    )) %>%
    dplyr::select(Hypothesis, `50\\% CIs`),
  by = "Hypothesis"
) %>%
  mutate("hypothesis" = layHypotheses) %>%
  dplyr::select(
    "Hypothesis" = hypothesis, Delta, `50\\% CIs`, `95\\% CIs`,
    `Evidence Ratio`
  ) %>%
  kbl(., longtable = T, booktabs = T, format = "latex",
      caption = caption, escape = FALSE
  ) %>%
  column_spec(column = c(1:10), width = "2.5cm") %>%
  kable_styling(latex_options = "striped")

pairwiseTableGrowthMass
```

Table 10: *Coefficients from a Bayesian non-linear effects model predicting body mass of Japanese quail as a Gompertz function of age in weeks. Coefficients represent posterior medians and are estimated from body mass data collected weekly between 0 and 8 weeks of age. Credible intervals (CIs) represent quantile intervals around medians.*

Hypothesis	Delta	50% CIs	95% CIs	Evidence Ratio
Cold-reared Asymptote (a) > Mild-reared Asymptote (a)	17.4414500	[12.337, 22.492]	[7.453, 27.181]	550.7241379
Cold-reared Displacement (b) > Mild-reared Displacement (b)	0.0647067	[0.022, 0.108]	[-0.022, 0.148]	8.3841642
Cold-reared Growth Rate (c) < Mild-reared Growth Rate (c)	-0.0406110	[-0.053, -0.029]	[-0.064, -0.017]	499.0000000
Warm-reared Asymptote (a) > Mild-reared Asymptote (a)	4.3062050	[-0.522, 9.065]	[-5.096, 13.432]	3.4358192

Warm-reared Displacement (b) > Mild-reared Displacement (b)	-0.0066059	[-0.045, 0.031]	[-0.084, 0.068]	0.7871105
Warm-reared Growth Rate (c) > Mild-reared Growth Rate (c)	0.0202168	[0.008, 0.032]	[-0.003, 0.044]	11.7693536
Cold-reared Asymptote (a) > Warm-reared Asymptote (a)	13.0839550	[8.458, 17.75]	[4.161, 22.186]	133.4537815
Cold-reared Displacement (b) > Warm-reared Displacement (b)	0.0716482	[0.033, 0.109]	[-0.004, 0.146]	15.8243954
Cold-reared Growth Rate (c) < Warm-reared Growth Rate (c)	-0.0607552	[-0.072, -0.05]	[-0.083, -0.039]	Inf

```
#save_kable(pairwiseTableGrowthMass,
# "../tables/pairwiseTableGrowthMass.html")
```

Distinctions in body mass among treatments at 8 weeks of age were evaluated using a Bayesian one-way ANOVA with priors on body mass for our mild treatment being normally distributed with a mean of 250 and standard deviation of 25. Priors for our warm- and cold-reared treatments were assigned means of 242.5 and 257.5 respectively (again, as per Burness et al, 2013), and standard deviations held liberally at 25. Posterior probabilities for differences *between* treatments were calculated using the Savage-Dickey density ratio method.

```
growthAnova <- brm(
  data = data %>%
    filter(week == 8) %>%
    dplyr::select(pretreatment, mass, "batch" = exp) %>%
    drop_na() %>%
    mutate(pretreatment =
             factor(pretreatment,
                    levels = c("neutral", "cold", "warm"))),
  formula = mass ~ 0 + pretreatment + (1|batch),
  prior = c(
    set_prior("normal(250, 25)", class = "b",
              coef = "pretreatmentneutral"),
    set_prior("normal(257.5, 25)", class = "b",
              coef = "pretreatmentcold"),
    set_prior("normal(242.5, 25)", class = "b",
              coef = "pretreatmentwarm"),
    set_prior("exponential(2.5)", class = "sd",
              group = "batch")
  ),
  family = "gaussian",
  seed = 200,
  cores = 4, chains = 4,
  iter = 50000, warmup = 10000, thin = 10,
  control = list(adapt_delta = 0.95, max_treedepth = 13),
  silent = TRUE, refresh = 0,
  file = "./models/_massGrowthAnova.Rds"
)

hypotheses <- c(
  "pretreatmentcold - pretreatmentneutral > 0",
  "pretreatmentwarm - pretreatmentneutral > 0",
  "pretreatmentcold - pretreatmentwarm > 0"
)
```

```
caption <- paste0("Results from a Bayesian, one-way ANOVA comparing ",
                  "body mass at 8 weeks of Japanese quail reared in the ",
                  "cold (10°C until at least 3 weeks of age; n = ",
                  nrow(subset(growthAnova$data, pretreatment == "cold")),
                  "), mild temperature (constant 20°C; n = ",
                  nrow(subset(growthAnova$data, pretreatment == "neutral")),
                  "), or warmth (30°C until at least 3 weeks of age; n = ",
                  nrow(subset(growthAnova$data, pretreatment == "warm")), ")."
                  )

growthMassAnovaTable <- bind_rows(
  lapply(hypotheses, FUN = function(x) {
    hold <- hypothesis(growthAnova,
                       hypothesis = x, class = "b",
                       alpha = 0.5, robust = TRUE)
    return(data.frame(
      "hyp" = x,
      "deltaMass" = hold$hypothesis$Estimate,
      "pProb" = hold$hypothesis$Post.Prob
    ))
  })
) %>%
  mutate("Hypothesis" = c(
    "Cold-Reared Mass > Mild-Reared Mass",
    "Warm-Reared Mass > Mild-Reared Mass",
    "Cold-Reared Mass > Warm-Reared Mass"
  )) %>%
  dplyr::select(Hypothesis, "Delta Mass (g)" = deltaMass,
         "Posterior Probability" = pProb) %>%
  kbl(.,
    longtable = T, booktabs = T,
    caption = caption
  ) %>%
  kable_styling(latex_options = "striped")

growthMassAnovaTable
```

Table 11: *Results from a Bayesian, one-way ANOVA comparing body mass at 8 weeks of Japanese quail reared in the cold (10°C until at least 3 weeks of age; n = 42), mild temperature (constant 20°C; n = 36), or warmth (30°C until at least 3 weeks of age; n = 43).*

Hypothesis	Delta Mass (g)	Posterior Probability
Cold-Reared Mass > Mild-Reared Mass	13.620679	0.9696875
Warm-Reared Mass > Mild-Reared Mass	15.071624	0.9818750
Cold-Reared Mass > Warm-Reared Mass	-1.432595	0.4190625

```
#save_kable(growthMassAnovaTable, "../tables/growthMassAnovaTable.html")

# Convincing evidence that cold and warm reared birds are
# heavier at 8 weeks of age than mild-reared birds, but
# not that cold- and warm-reared birds differ in mass
# (~42% probability that warm-reared birds are largest).
```

Thermal rearing conditions in our study varied in duration, with some warmth (30°C) and cold (10°C) exposures lasting between hatch to three weeks of age and some lasting between hatch and maturity (eight weeks of age). To test whether longer exposure to thermal environments during development differentially shaped growth patterns relative to more transient exposures (3 weeks), we repeated our above analysis but while only including individuals who experience continuous cold (10°C), or warmth (30°C) treatments until maturity. Priors for these analyses remained identical to those used previously, however, effect of egg batch was removed from our model given that all individuals in this analysis were derived from the same batch.

```
initFunction <- function(chain_id = 1) {
  list(
```

```
    "b_A" = c(rnorm(1, 250, 25), -14),
    "b_B" = c(rskew_normal(1, xi = 2.5, omega = 2, alpha = 4), 0),
    "b_C" = c(rbeta(1, 5, 5), 0),
    "b_sigma" = c(rskew_normal(1, xi = 1, omega = 0.5, alpha = -5), 1),
    "sd_1" = rexp(1, 0.5)
  )
}

# Loading initial values into a list.

initList <- lapply(1:4, initFunction)

growthModelStrict <- brm(
  data = data %>%
    filter(week <= 8 & exp == "C") %>%
    mutate(pretreatment = factor(pretreatment,
      levels = c("cold", "warm")
    )) %>%
    dplyr::select(ring, pretreatment, week, mass) %>%
    distinct() %>%
    mutate(weekB = week + 1),
  formula = bf(mass ~ A * exp(-B * exp(-C * week)) + D,
    A ~ 1 + pretreatment,
    B ~ 1 + pretreatment,
    C ~ 1 + pretreatment,
    D ~ 0 + (1 | ring),
    sigma ~ 0 + Intercept + log(weekB),
    nl = TRUE
  ),
  prior = c(
    set_prior("normal(250, 25)",
      class = "b", coef = "Intercept",
      nlpar = "A"
    ),
    set_prior("normal(-14, 25)",
      class = "b", coef = "pretreatmentwarm",
      nlpar = "A"
    ),
    set_prior("normal(3, 1)",
      class = "b", coef = "Intercept",
      nlpar = "B"
    ),
    set_prior("normal(0, 0.5)",
      class = "b", coef = "pretreatmentwarm",
      nlpar = "B"
    ),
    set_prior("skew_normal(0.5, 0.1, 2.5)",
      class = "b", coef = "Intercept",
      nlpar = "C"
    ),
    set_prior("normal(0, 0.2)",
      class = "b", coef = "pretreatmentwarm",
      nlpar = "C"
    ),
    set_prior("exponential(0.5)", class = "sd",
              coef = "Intercept", group = "ring", nlpar = "D"),
    set_prior("skew_normal(1, 0.5, -10)", class = "b",
              coef = "Intercept", dpar = "sigma"),
    set_prior("skew_normal(1, 0.5, 10)", class = "b",
              coef = "logweekB", dpar = "sigma")
  ),
  family = "gaussian",
  init = initList,
  seed = 200,
  cores = 4, chains = 4, threads = 2,
  iter = 50000, warmup = 10000, thin = 10,
  control = list(adapt_delta = 0.98, max_treedepth = 13),
```

```
  backend = "cmdstan",
  silent = TRUE, refresh = 0,
  file = "./models/_growthModelMassStrict.Rds"
)

ggarrange(
  ggplot(data = data.frame("Rhat" = brms::rhat(growthModelStrict)),
         aes(x = Rhat)) +
    geom_density() +
    theme_classic() +
    xlab(
      TeX('$\\hat{R}$')
    ) +
    ylab("Density"),
  ggplot(data = data.frame("Neff" = brms::neff_ratio(growthModelStrict)),
         aes(x = Neff)) +
    geom_density() +
    theme_classic() +
    xlab(
      TeX('$N_{eff}/N~Ratio$')
      ) +
    ylab("Density")
)
```

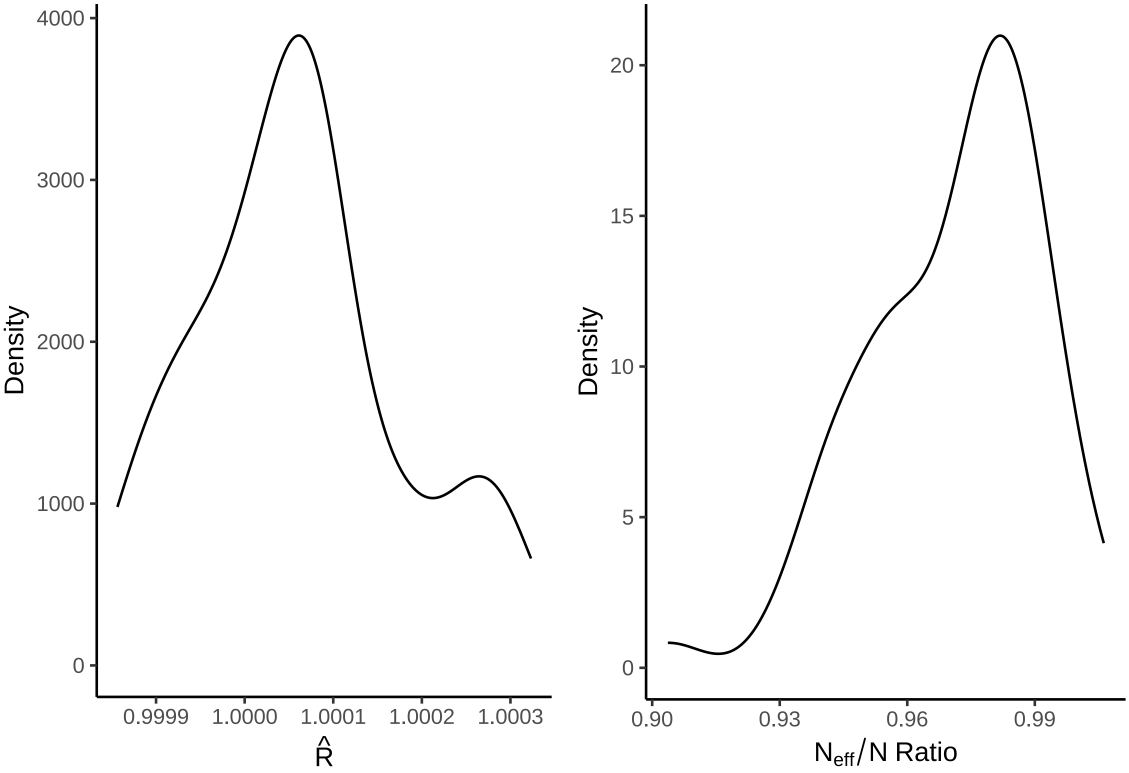

Figure 35: *Gelman-Rubin statistics ($\hat{R}$) and ratio of effective samples sizes by samples sizes per parameter from a Bayesian non-linear model estimating growth of mass (g) among Japanese quail. Here, our sample population is restricted to include only quail reared in cold (10°C), mild (20°C) or warm (30°C) conditions until maturity (8 weeks).*

```
# Checking residuals.
```

```
p1 <- growthModelStrict$data %>%
  mutate("residuals" =
           residuals(growthModelStrict,
```

```
                     type = "pearson",
                     robust = TRUE)[, "Estimate"]) %>%
  ggplot(aes(x = residuals)) +
  geom_density(colour = "black", fill = "white") +
  xlab("Pearson Residuals") +
  ylab("Density") +
  theme_classic()

p2 <- growthModelStrict$data %>%
  mutate(
    "residuals" = residuals(growthModelStrict,
      type = "pearson",
      robust = TRUE
    )[, "Estimate"],
    "fitted" = fitted(growthModelStrict,
                      robust = TRUE)[, "Estimate"]
  ) %>%
  ggplot(aes(x = fitted, y = residuals)) +
  geom_point(colour = "black", pch = 21,
             size = 2, fill = "grey75", alpha = 0.5) +
  ylab("Pearson Residuals") +
  xlab("Fitted Values (g)") +
  theme_classic()

p3 <- growthModelStrict$data %>%
  mutate("residuals" = residuals(growthModelStrict,
    type = "pearson",
    robust = TRUE
  )[, "Estimate"]) %>%
  ggplot(aes(x = week, y = residuals)) +
  geom_point(
    colour = "black", pch = 21, size = 2,
    fill = "grey75", alpha = 0.5,
    position = position_jitter(width = 0.25)
  ) +
  stat_summary(geom = "errorbar", fun.data = "mean_se",
               colour = "black", width = 0.25) +
  stat_summary(geom = "point", fun = "mean", pch = 21,
               colour = "black", fill = "white", size = 4) +
  ylab("Pearson Residuals") +
  xlab("Age (weeks)") +
  theme_classic()

p4 <- growthModelStrict$data %>%
  mutate("residuals" = residuals(growthModelStrict,
    type = "pearson",
    robust = TRUE
  )[, "Estimate"]) %>%
  mutate(pretreatment = str_to_title(pretreatment)) %>%
  mutate(pretreatment = ifelse(pretreatment == "Cold", "Cold\n(10°C)",
      "Warm\n(30°C)"
    )
  ) %>%
  mutate(pretreatment = factor(pretreatment,
                               levels = c("Cold\n(10°C)",
                                          "Warm\n(30°C)"))
         ) %>%
  ggplot(aes(x = pretreatment, y = residuals)) +
  geom_point(
    colour = "black", pch = 21, size = 2, fill = "grey75", alpha = 0.5,
    position = position_jitter(width = 0.25)
  ) +
  stat_summary(geom = "errorbar", fun.data = "mean_se",
               colour = "black", width = 0.25) +
  stat_summary(geom = "point", fun = "mean", pch = 21,
               colour = "black", fill = "white", size = 4) +
  ylab("Pearson Residuals") +
```

```
  xlab("Rearing Conditions)") +
  theme_classic()

(p1 + p2) / (p3 + p4)
```

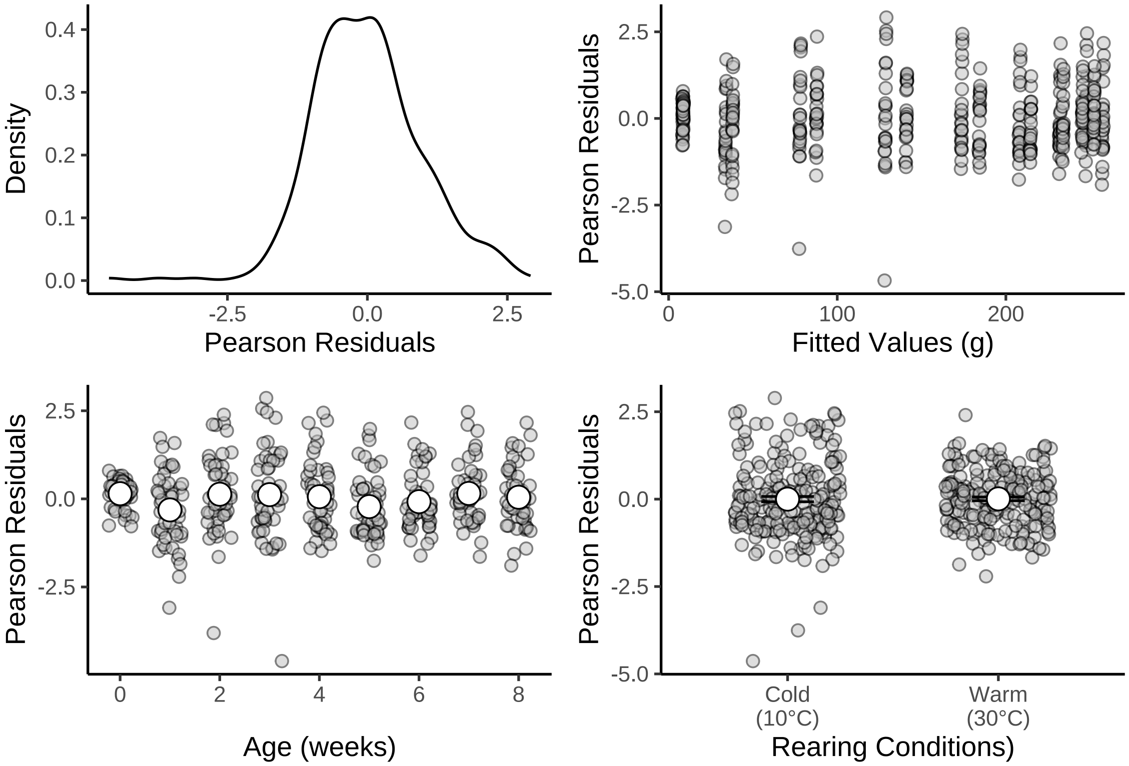

Figure 36: *Density and distributions of Pearson residuals across fitted values and model predictors (including age and rearing conditions). All residuals pertain to those drawn from a Bayesian non-linear model predicting body mass (g) during growth in Japanese quail. Pearson residuals are shown rather than ordinary residuals to correct for the age-dependence of model error. Here, our sample population is restricted to include only quail reared in cold (10°C) or warm (30°C) conditions until maturity (8 weeks).*

```
# Homoskedastic.
# Summarising sample sizes

caption <- paste0("Number of body mass measurements (samples) ",
                  "drawn from Japanese quail between 0 and ",
                  "8 weeks of age (maturity) across three distict ",
                  "thermal rearing conditions.")

growthModelStrict$data %>%
  group_by(week, pretreatment) %>%
  dplyr::count(name = "Samples (n)") %>%
  mutate(pretreatment = ifelse(pretreatment == "cold",
                               "Cold (10°C)", "Warm (30°C)"
                               )
         ) %>%
  arrange(pretreatment, week) %>%
  rename("Age (Weeks)" = week,
         "Rearing Conditions" = pretreatment) %>%
  kbl(.,
    longtable = T, booktabs = T,
    caption = caption
  ) %>%
  kable_styling(latex_options = "striped")
```

Table 12: *Number of body mass measurements (samples) drawn from Japanese quail between 0 and 8 weeks of age (maturity) across three distict thermal rearing conditions.*

Age (Weeks)	Rearing Conditions	Samples (n)
0	Cold (10°C)	24
1	Cold (10°C)	25
2	Cold (10°C)	25
3	Cold (10°C)	25
4	Cold (10°C)	24
5	Cold (10°C)	24
6	Cold (10°C)	24
7	Cold (10°C)	23
8	Cold (10°C)	24
0	Warm (30°C)	24
1	Warm (30°C)	24
2	Warm (30°C)	24
3	Warm (30°C)	24
4	Warm (30°C)	23
5	Warm (30°C)	23
6	Warm (30°C)	23
7	Warm (30°C)	23
8	Warm (30°C)	23

Our predicted growth curve is then replotted.

```
showtext_auto()

growthCurveStrict <- with(
  growthModelStrict$data,
  expand.grid(
    "week" = seq(0, 8, by = 0.1),
    "pretreatment" = c("cold", "warm")
  )
) %>%
  mutate("weekB" = week + 1) %>%
  mutate(
    "Fit" = predict(growthModelStrict, re_form = NA,
                    newdata = .,
                    robust = TRUE)[, "Estimate"],
    "SE" = predict(growthModelStrict, re_form = NA,
                   newdata = .,
                   robust = TRUE)[, "Est.Error"]
  ) %>%
  mutate(
    "LCL" = Fit - SE,
    "UCL" = Fit + SE
  ) %>%
  mutate(pretreatment = str_to_title(pretreatment)) %>%
  mutate(pretreatment = factor(pretreatment,
                               levels = c("Cold", "Warm"))) %>%
  ggplot(aes(x = week, y = Fit, fill = pretreatment,
             linetype = pretreatment)) +
  # facet_wrap(~sex) +
  geom_ribbon(aes(x = week, ymin = LCL, ymax = UCL),
    colour = NA, size = 0.25, alpha = 0.3
  ) +
  geom_line(colour = "black", alpha = 0.7) +
  stat_summary(
    data = growthModelStrict$data %>%
      mutate(pretreatment = str_to_title(pretreatment)) %>%
      mutate(pretreatment = factor(pretreatment,
                                   levels = c("Cold", "Warm"))),
    aes(x = week, y = mass),
```

```
    geom = "errorbar", fun.data = "mean_se",
    colour = "black", alpha = 0.7, width = 0.25,
    position = position_dodge(width = 0.15)
  ) +
  stat_summary(
    data = growthModelStrict$data %>%
      mutate(pretreatment = str_to_title(pretreatment)) %>%
      mutate(pretreatment = factor(pretreatment,
                                   levels = c("Cold", "Warm"))),
    aes(x = week, y = mass),
    geom = "point", fun = "mean", pch = 21, size = 3,
    colour = "black", alpha = 0.7,
    position = position_dodge(width = 0.15)
  ) +
  theme_classic() +
  scale_fill_manual(values = c("#7BB4E3", "#CD5C5C"),
                    name = "Rearing\nConditions",
                    labels = c("Cold (10°C)",
                               "Warm (30°C)")) +
  scale_linetype_manual(values = c("solid", "dashed"),
                        name = "Rearing\nConditions",
                    labels = c("Cold (10°C)",
                               "Warm (30°C)")) +
  xlab("Age (weeks)") +
  ylab("Body Mass (g)")

growthCurveStrict
```

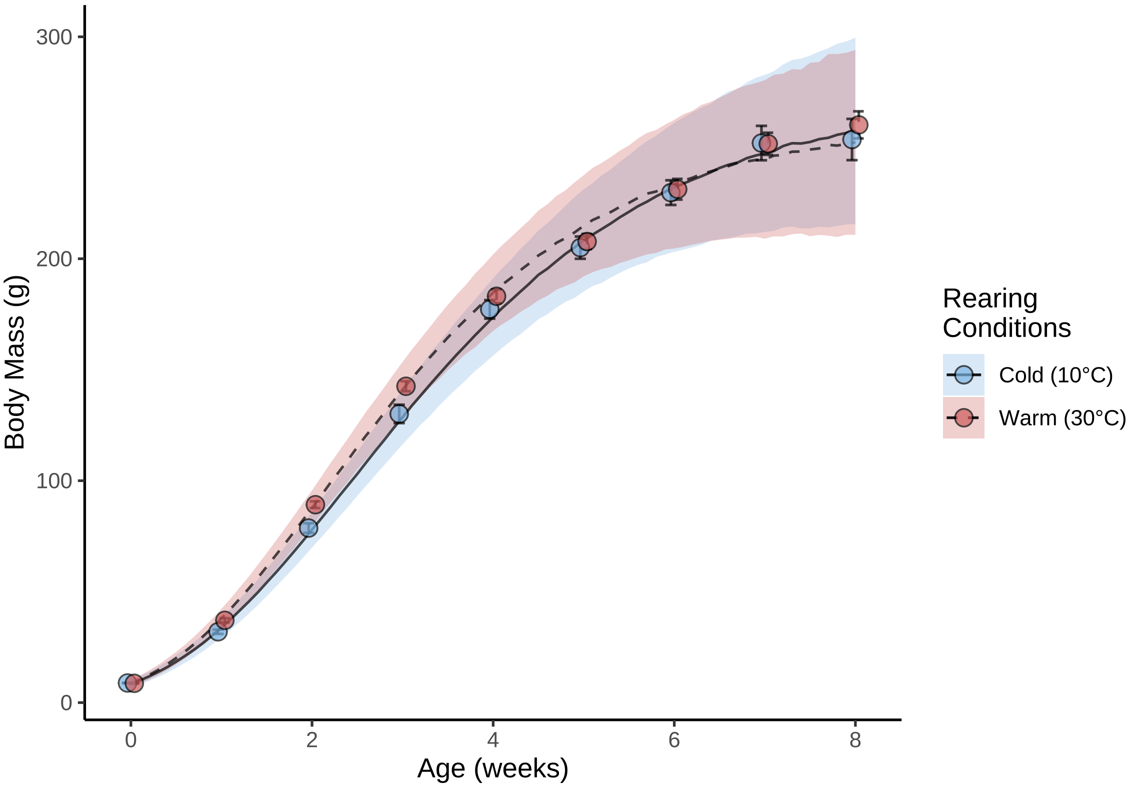

Figure 37: *Body mass (g) growth curves of Japanese quail reared in the cold (10°C), or the warmth (30°C) until at eight weeks of age. Dots represent mean values and errorbars represent standard errors around means. Lines represent estimated trends in growth from a Bayesian non-linear model, while ribbons represent confidence around trends (± one standard error).*

```r
ggsave("./plots/strictGrowthCurve.jpg", dpi = 800,
       width = 7, height = 6.5,
       growthCurveStrict)
showtext_auto(enable = "FALSE")

# Summarising growth curve parameters

caption <- paste0("Coefficients from a Bayesian non-linear effects model ",
                  "predicting body mass of Japanese quail as a Gompertz ",
                  "function of age in weeks. Coefficients represent posterior ",
                  "medians and are estimated from body mass data collected ",
                  "weekly between 0 and 8 weeks of age. Credible intervals ",
                  "(CIs) represent quantile intervals around medians."
)

growthModelStrictMassTable <-
  merge(
    as.data.frame(growthModelStrict) %>%
      summarise_all(., .funs = median) %>%
      mutate_all(., .funs = round, 3) %>%
      pivot_longer(everything(), names_to = "Parameter",
                   values_to = "Values") %>%
      filter(grepl("b_|sd_", Parameter)) %>%
      arrange(Parameter),
    quantileCIs(growthModelStrict, cis = c(50, 95)) %>%
    mutate(
    `50\\% CIs` = paste0("[", round(Low_CI_50, digits = 3),
                         ", ", round(High_CI_50, digits = 3), "]"),
    `95\\% CIs` = paste0("[", round(Low_CI_95, digits = 3),
                         ", ", round(High_CI_95, digits = 3), "]")
    ) %>%
    dplyr::select(Parameter, `50\\% CIs`, `95\\% CIs`),
    by = "Parameter"
  ) %>%
  merge(., tribble(
    ~Parameter, ~Par,
    "b_A_Intercept", "Beta a0 (Cold-reared)",
    "b_A_pretreatmentwarm", "Beta a1 (Warm-reared)",
    "b_B_Intercept", "Beta b0 (Cold-reared)",
    "b_B_pretreatmentwarm", "Beta b1 (Warm-reared)",
    "b_C_Intercept", "Beta c0 (Cold-reared)",
    "b_C_pretreatmentwarm", "Beta c1 (Warm-reared)",
    "sd_ring__D_Intercept", "Mu (Individual Intercept)",
    "b_sigma_Intercept", "Tau 0",
    "b_sigma_logweekB", "Tau 1"
  ),
  by = "Parameter", all.x = TRUE
  ) %>%
  dplyr::select(-Parameter) %>%
  dplyr::select("Parameter" = Par, "Value" = Values,
                `50\\% CIs`,`95\\% CIs`) %>%
  kbl(., longtable = T, booktabs = T, format = "latex",
      caption = caption, escape = FALSE) %>%
  column_spec(column = c(1, 4), width = "2.5cm") %>%
  kable_styling(latex_options = "striped")

growthModelStrictMassTable
```

Table 13: *Coefficients from a Bayesian non-linear effects model predicting body mass of Japanese quail as a Gompertz function of age in weeks. Coefficients represent posterior medians and are estimated from body mass data collected weekly between 0 and 8 weeks of age. Credible intervals (CIs) represent quantile intervals around medians.*

Parameter	Value	50% CIs	95% CIs
Beta a0 (Cold-reared)	273.027	[269.455, 276.622]	[262.924, 283.85]

Beta a1 (Warm-reared)	-11.654	[-16.356, -6.914]	[-25.392, 1.772]
Beta b0 (Cold-reared)	3.479	[3.455, 3.505]	[3.409, 3.555]
Beta b1 (Warm-reared)	-0.063	[-0.098, -0.03]	[-0.164, 0.038]
Beta c0 (Cold-reared)	0.510	[0.503, 0.517]	[0.489, 0.532]
Beta c1 (Warm-reared)	0.061	[0.05, 0.071]	[0.03, 0.091]
Tau 0	0.419	[0.354, 0.487]	[0.229, 0.621]
Tau 1	1.510	[1.465, 1.553]	[1.382, 1.637]
Mu (Individual Intercept)	0.612	[0.342, 0.868]	[0.036, 1.357]

And again, growth curve parameters are formally compared between rearing conditions groups.

```
caption <- paste0("Coefficients from a Bayesian non-linear ",
                  "effects model predicting body mass of Japanese quail as a ",
                  "Gompertz function of age in weeks. Coefficients represent ",
                  "posterior medians and are estimated from body mass data ",
                  "collected weekly between 0 and 8 weeks of age. Credible ",
                  "intervals (CIs) represent quantile intervals. ",
                  "Rearing conditions persisted from ",
                  "hatch to maturity (week 8)."
)

pairwiseTableGrowthMassStrict <- rbind(
  hypothesis(growthModelStrict,
             hypothesis = "A_Intercept - (A_Intercept + A_pretreatmentwarm) > 0",
             class = "b",
             robust = TRUE)$hypothesis,
  hypothesis(growthModelStrict,
             hypothesis = "B_Intercept - (B_Intercept + B_pretreatmentwarm) > 0",
             class = "b",
             robust = TRUE)$hypothesis,
  hypothesis(growthModelStrict,
             hypothesis = "(C_Intercept + C_pretreatmentwarm) - C_Intercept > 0",
             class = "b",
             robust = TRUE)$hypothesis
) %>%
  cbind(., data.frame("hypothesis" = c(
    "Cold-reared Asymptote (a) > Warm-reared Asymptote (a)",
    "Cold-reared Displacement (b) > Warm-reared Displacement (b)",
    "Cold-reared Growth Rate (c) < Warm-reared Growth Rate (c)"
  ))) %>%
  mutate(`95% CIs` = paste0("[", round(CI.Lower, digits = 3), ", ",
                            round(CI.Upper, digits = 3), "]"),
         Estimate = round(Estimate, digits = 3),
         Evid.Ratio = round(Evid.Ratio, digits = 3)) %>%
  dplyr::select("Hypothesis" = hypothesis, "Delta" = Estimate,
         `95% CIs`, "Evidence Ratio" = Evid.Ratio) %>%
  kbl(., longtable = T, booktabs = T, format = "latex", caption = caption) %>%
  column_spec(column = c(1:10), width = "2.5cm")  %>%
  kable_styling(latex_options = "striped")

pairwiseTableGrowthMassStrict
```

Table 14: *Coefficients from a Bayesian non-linear effects model predicting body mass of Japanese quail as a Gompertz function of age in weeks. Coefficients represent posterior medians and are estimated from body mass data collected weekly between 0 and 8 weeks of age. Credible intervals (CIs) represent quantile intervals. Rearing conditions persisted from hatch to maturity (week 8).*

Hypothesis	Delta	95% CIs	Evidence Ratio

Cold-reared Asymptote (a) > Warm-reared Asymptote (a)	11.654	[0.287, 23.122]	21.008
Cold-reared Displacement (b) > Warm-reared Displacement (b)	0.063	[-0.021, 0.147]	8.445
Cold-reared Growth Rate (c) < Warm-reared Growth Rate (c)	0.061	[0.035, 0.086]	5332.333

```
#save_kable(pairwiseTableGrowthMassStrict,
# "../tables/pairwiseTableGrowthMassStrict")
```

```
growthAnovaStrict <- brm(
  data = data %>%
    filter(week == 8 & exp == "C") %>%
    mutate(pretreatment = factor(pretreatment,
      levels = c("cold", "warm")
    )) %>%
    dplyr::select(pretreatment, mass) %>%
    drop_na(),
  formula = mass ~ 0 + pretreatment,
  prior = c(
    set_prior("normal(257.5, 25)", class = "b",
              coef = "pretreatmentcold"),
    set_prior("normal(242.5, 25)", class = "b",
              coef = "pretreatmentwarm")
  ),
  family = "gaussian",
  seed = 200,
  cores = 4, chains = 4,
  iter = 50000, warmup = 10000, thin = 10,
  control = list(adapt_delta = 0.95, max_treedepth = 13),
  silent = TRUE, refresh = 0,
  file = "./models/_massGrowthAnovaStrict.Rds"
)

caption <- paste0("Results from a Bayesian, one-way ANOVA comparing ",
                  "body mass at 8 weeks of Japanese quail reared in the ",
                  "cold (10°C until 8 weeks of age; n = ",
                  nrow(subset(growthAnovaStrict$data, pretreatment == "cold")),
                  "), or warmth (30°C until at least 3 weeks of age; n = ",
                  nrow(subset(growthAnovaStrict$data, pretreatment == "warm")), ")."
                  )

growthMassStrictAnovaTable <-
  data.frame(
    hypothesis(growthAnovaStrict,
               hypothesis = "pretreatmentcold - pretreatmentwarm > 0",
               class = "b", robust = TRUE, alpha = 0.5)$hypothesis
    ) %>%
  dplyr::select("deltaMass" = Estimate,
         "pProb" = Post.Prob) %>%
  mutate("Hypothesis" = "Cold-Reared Mass > Warm-Reared Mass") %>%
  mutate(deltaMass = round(deltaMass, digits = 3),
         pProb = round(pProb, digits = 3)) %>%
  dplyr::select(Hypothesis, "Delta Mass (g)" = deltaMass,
         "Posterior Probability" = pProb) %>%
  kbl(.,
    longtable = T, booktabs = T,
    caption = caption
  ) %>%
  kable_styling(latex_options = "striped")
```

```
growthMassStrictAnovaTable
```

Table 15: *Results from a Bayesian, one-way ANOVA comparing body mass at 8 weeks of Japanese quail reared in the cold (10°C until 8 weeks of age; n = 24), or warmth (30°C until at least 3 weeks of age; n = 23).*

Hypothesis	Delta Mass (g)	Posterior Probability
Cold-Reared Mass > Warm-Reared Mass	-4.632	0.332

Last, we visually assess how well body mass at three weeks predicts body mass at 8 weeks across all quail.

```
data %>%
  filter(week %in% c(3,8) & !(ring == "") & !is.na(pretreatment)) %>%
  group_by(ring, pretreatment, week) %>%
  summarise(mass = mean(mass, na.rm = T)) %>%
  ungroup() %>%
  pivot_wider(id_cols = c("ring", "pretreatment"),
              names_from = "week", values_from = "mass") %>%
  rename("week3" = `3`, "week8" = `8`) %>%
  ggplot(aes(x = week3, y = week8, fill = pretreatment,
             linetype = pretreatment)) +
  geom_point(pch = 21, size = 2, colour = "black", alpha = 0.5) +
  geom_smooth(method = "lm", se = FALSE, colour = "black") +
  scale_fill_manual(values = c("#7BB4E3", "black", "#CD5C5C"),
                    name = "Rearing\nConditions",
                    labels = c("Cold (10°C)",
                               "Neutral (20°C)",
                               "Warm (30°C)")) +
  scale_linetype_manual(values = c("solid", "dotted", "dashed"),
                        name = "Rearing\nConditions",
                    labels = c("Cold (10°C)",
                               "Neutral (20°C)",
                               "Warm (30°C)")) +
  xlab("Mass at Week 3 (g)") +
  ylab("Mass at Week 8 (g)") +
  theme_classic()
```

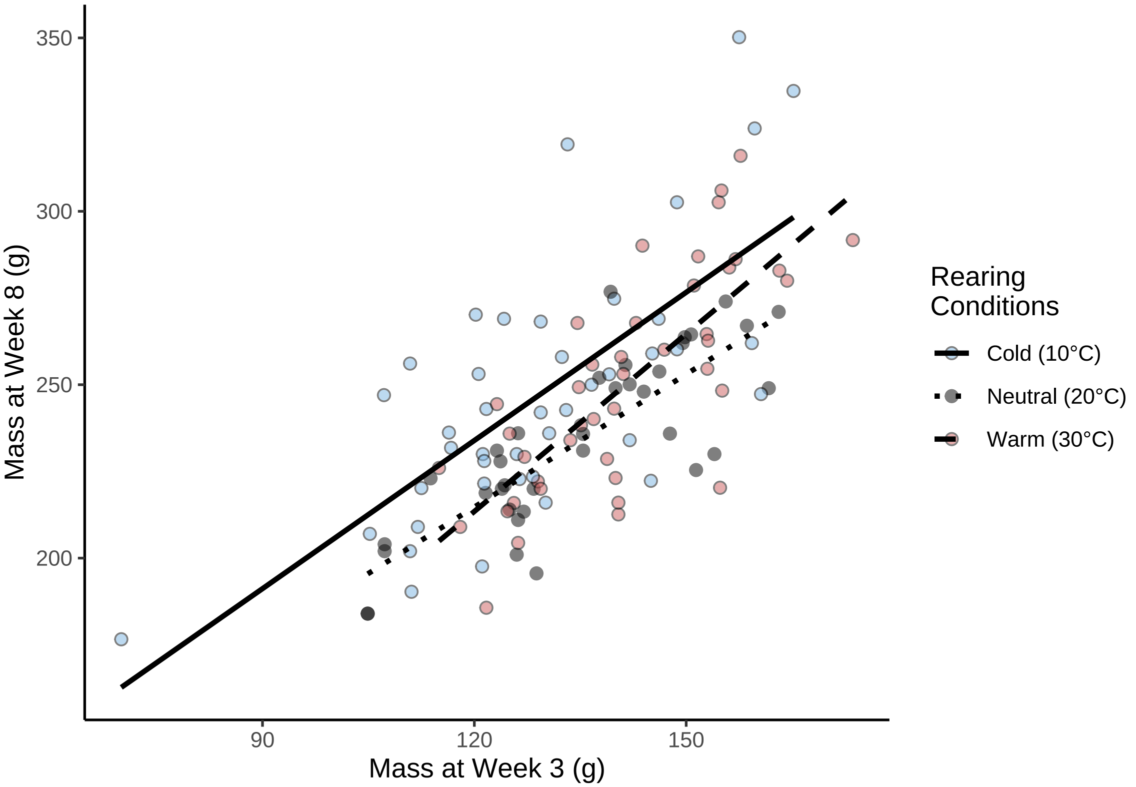

Figure 38: *Relationship between body mass at three weeks of age and eight weeks of age in Japanese quail reared in eith cold (10°C), neutral (20°C) or warm (30°C) conditions. Lines indicate linear relationships estimated via the R package ggplot2 (Wickham, 2011).*

Tarsus length

To evaluate an effect of rearing condition on tarsus elongation during development, we again used a non-linear model with tarsus length being a Gompertz function of age in weeks with a log-log, age-dependent error term. Because body mass may also explain some variance in tarsus length within ages, individual body mass, mean-centred and scaled by standard deviation per week of age, was included as an additional linear predictor of tarsus length. Our model predicting tarsus length was therefore as follows:

$$Tarsus\ Length_{ij} \sim a \cdot e^{-b \cdot e^{-c \cdot Age_{ij}}} + \beta_1 \cdot Scaled\ Mass + \mu_{0j} + \epsilon_{ij}$$
$$a_{ij} \sim \beta_{a0} + \beta_{a1} \cdot Cold\ Reared_j + \beta_{a2} \cdot Warm\ Reared_j + \mu_{0aj}$$
$$b_{ij} \sim \beta_{b0} + \beta_{b1} \cdot Cold\ Reared_j + \beta_{b2} \cdot Warm\ Reared_j + \mu_{0bj}$$
$$c_{ij} \sim \beta_{c0} + \beta_{c1} \cdot Cold\ Reared_j + \beta_{c2} \cdot Warm\ Reared_j + \mu_{0cj}$$
$$ln(\epsilon_{ij}) \sim \tau_0 + \tau_1 * ln(Age_{ij} + 1)$$

where β_1 represents the slope of the predicted relationship between body mass (g) and tarsus length (mm), and all other variables remain as previously described.

Priors for model parameters were again moderately informative and derived from findings of Burness et al (2013) and Persson et al (2024), while assuming a y-intercept (tarsus length at hatching) of approximately 10 mm. For the asymptote (β_{a0}), x-axis displacement (β_{b0}), and growth rate (β_{c0}) of our population growth curve, priors were normal (β_{a0}: mean = 37.5, standard deviation = 2.5; β_{b0}: mean = 1.35, standard deviation = 0.5) and skew-normal (β_{c0}: ξ = 0.5, ω = 0.1, α = 2.5) while those for the effect of rearing condition on growth parameters a, b, and c (i.e. β_{a1-2}, β_{b1-2}, and β_{c1-2} respectively) were all normal-distributed. For

effects of cold- and warming-rearing on asymptotes, means were derived from Burness et al (2013) and represented the calculated difference in tarsus length from mean values among birds raised at 15°C and 30°C until 66 days of age (-0.35 for cold-rearing and 0.35 for warm-rearing respectively). Standard deviations were set broadly at 2. For similar effects of rearing treatment on x-axis displacement and growth rate, we assumed means of 0 and standard deviations of 0.5 and 0.25 respectively. Since we expected the correlation between body mass and tarsus length to be positive and low (< 1) but above 0, we used a skew-normal distribution for our prior on β_1, with ξ equaling 0.5, ω equaling 0.15, and α equaling 5. For our priors on our batch and individual-level effects (μ_{0a} - μ_{0c}, μ_{0j}), we assumed exponential distributions with lambda values of 1 (μ_{0a}), 10 (μ_{0b}), 25 (μ_{0c}), and 1.5 (μ_{0j}). Finally, for our and error structure terms (τ_0 and τ_1), we assumed both exponential ($\lambda = 1.5$) and skew normal distributions (τ_0: $\xi = 0$, $\omega = 0.5$, $\alpha = 10$; τ_1: $\xi = 0$, $\omega = 0.25$, $\alpha = 10$) respectively.

As previous, 4 Hamiltonian Monte Carlo (HMC) chains were used, with each run for 50000 iterations and 10000 warm-up iterations, then sampled every 10 iterations. Prior suitability is evaluated by a prior predictive check below, after graphically evaluating those of τ_0, τ_1, and β_1.

```
# Checking how the standard deviation of tarsus length scales with age

data %>%
  filter(week <= 8) %>%
  mutate(pretreatment = factor(pretreatment,
    levels = c("neutral", "cold", "warm")
  )) %>%
  dplyr::select(ring, pretreatment, week, tarsusLengthMean) %>%
  distinct() %>%
  mutate(weekB = log(week + 1)) %>%
  group_by(week, weekB) %>%
  summarise(SD = log(sd(tarsusLengthMean, na.rm = T))) %>%
  ungroup() %>%
  ggplot(aes(x = weekB, y = SD)) +
  geom_point(size = 3, pch = 21, colour = "black", fill = "grey50") +
  geom_smooth(method = "lm", colour = "black",
              linetype = "dashed", se = FALSE) +
  xlab("Age (Weeks + 1; Natural-log Transformed )") +
  ylab("Standard Deviation of Tarsus Length\n(Natural-log Transformed)") +
  theme_classic()
```

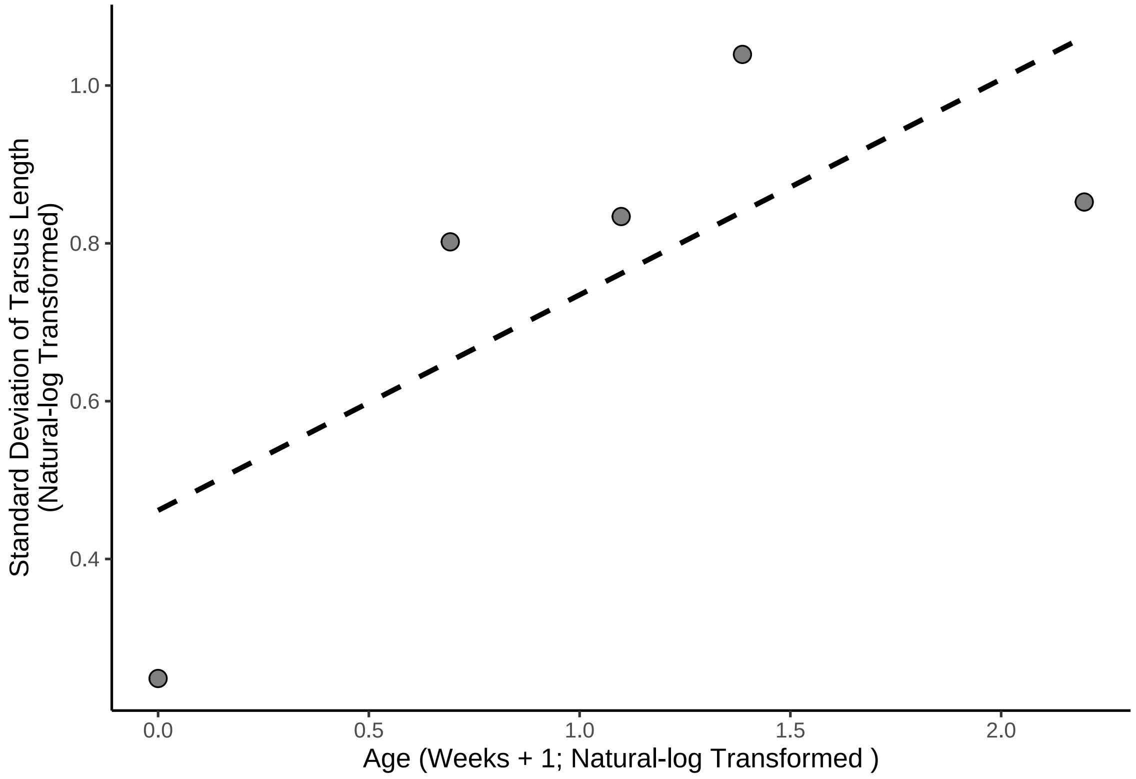

Figure 39: *Effect of natural log-transformed age (in weeks + 1) on the natural log-transformed standard deviation of tarsus length (mm). Dotted line represents a line of body best fit estimated from a linear relationship by internal functions of the R package ggplot (Wickham, 2011).*

```
# Weakly linear, but clearly increasing between 0
# and 3 weeks of age. For this reason, retaining linear expectations.
```

```
# Confirming prior for age-independent effect of natural-log
# transformed body mass (g) on tarsus length (mm).

ggplot(data %>%
  filter(week <= 8) %>%
  mutate(pretreatment = factor(pretreatment,
    levels = c("neutral", "cold", "warm")
  )) %>%
  dplyr::select(ring, pretreatment, week, mass, tarsusLengthMean) %>%
  distinct() %>%
  drop_na() %>%
  group_by(week) %>%
  mutate(
    "adjustedTarsus" = tarsusLengthMean - mean(tarsusLengthMean),
    "centredMass" = (mass - mean(mass, na.rm = T)) / sd(mass, na.rm = T)
  ) %>%
  ungroup(), aes(x = centredMass, y = adjustedTarsus)) +
  stat_summary_bin(geom = "errorbar", fun.data = "mean_se",
                   binwidth = 0.5, colour = "black") +
  stat_summary_bin(geom = "point", fun = "mean", binwidth = 0.5,
                   pch = 21, size = 5, colour = "black",
                   fill = "lightblue2", alpha = 0.5) +
  geom_smooth(method = "lm", colour = "black", se = FALSE,
              linetype = "dashed") +
  xlab("Age-Corrected Body Mass\n(g; Mean-centred Per Week of Age)") +
  ylab("Age-Corrected Tarsus Length\n(mm; Mean-centred Per Week of Age)") +
  theme_classic()
```

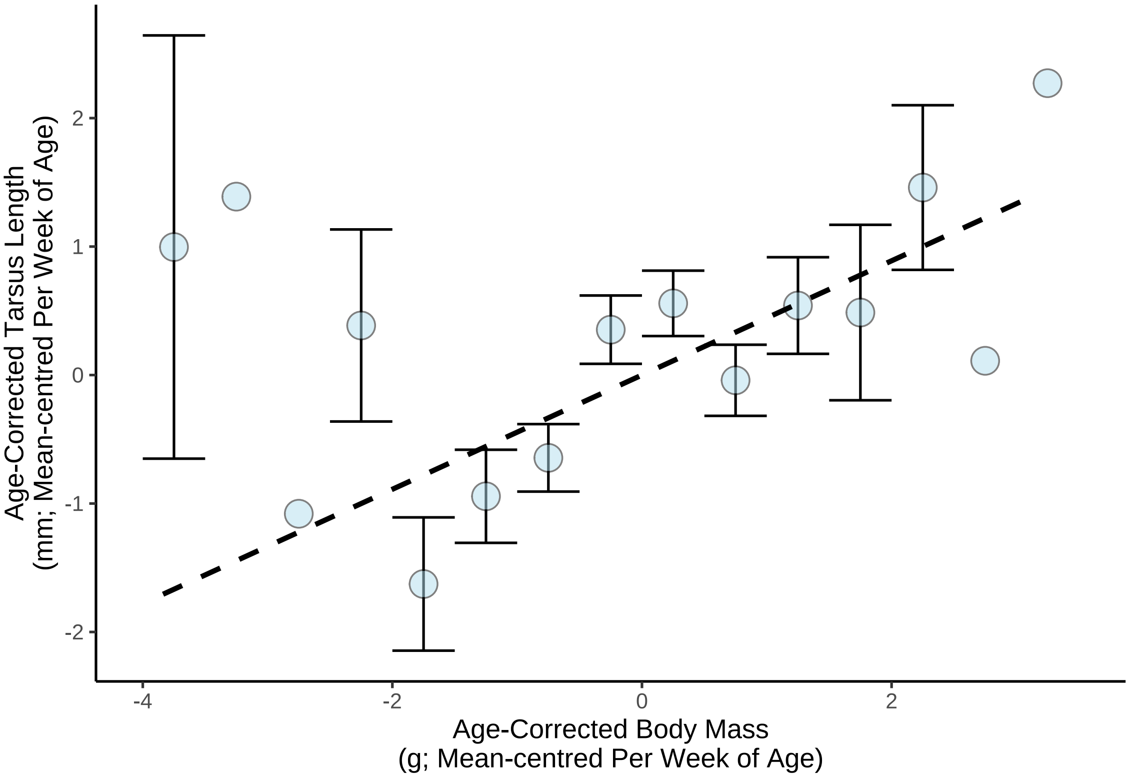

Figure 40: *Effect of natural log-transformed body mass (g) on the tarsus length (mm), mean-centred by age in weeks. Dots represent means binned at intervals of 0.5 and errorbars represent standard errors around means. Dotted line represents a line of body best fit estimated from a linear relationship by internal functions of the R package ggplot (Wickham, 2011).*

```
data %>%
  filter(week == 0) %>%
  summarise(min(tarsusLengthMean, na.rm = T))
```

```
## # A tibble: 1 x 1
##   `min(tarsusLengthMean, na.rm = T)`
##                                <dbl>
## 1                               9.54
```

```
bAtY <- function(a, yint) {
  b <- -log(yint / a)
  return(b)
}

cat(paste0("Estimated b value = ",
           round(bAtY(37.5, 10), digits = 3)))
```

```
## Estimated b value = 1.322
```

```
bXi <- round(skewxi(round(bAtY(37.5, 10), digits = 3),
                    omega = 1, alpha = 5), digits = 3)
cat(paste("xi value for skew-normal distribution on b = ", bXi))
```

```
## xi value for skew-normal distribution on b =  0.54
```

```
growthModelTarsus_ppCheck <- brm(
  data = data %>%
    filter(week <= 8) %>%
    mutate(pretreatment = factor(pretreatment,
      levels = c("neutral", "cold", "warm")
    )) %>%
```

```
    dplyr::select(ring, pretreatment, week, mass,
           "tarsus" = tarsusLengthMean, "batch" = exp) %>%
    distinct() %>%
    mutate(
      weekB = week + 1
    ) %>%
    group_by(week) %>%
    mutate(cScaledMass = (mass - mean(mass, na.rm = T)) /
             sd(mass, na.rm = T)) %>%
    ungroup(),
  formula = bf(tarsus ~ A * exp(-B * exp(-C * week)) + D,
    A ~ 1 + pretreatment + (1|batch),
    B ~ 1 + pretreatment + (1|batch),
    C ~ 1 + pretreatment + (1|batch),
    D ~ 1 + cScaledMass + (1 | ring),
    sigma ~ 0 + intercept + log(weekB),
    nl = TRUE
  ),
  prior = c(
    set_prior("normal(37.5, 2.5)",
              class = "b", coef = "Intercept",
              nlpar = "A"
    ),
    set_prior("normal(-0.35, 2)",
              class = "b", coef = "pretreatmentcold",
              nlpar = "A"
    ),
    set_prior("normal(0.35, 2)",
              class = "b", coef = "pretreatmentwarm",
              nlpar = "A"
    ),
    set_prior("exponential(1)", class = "sd",
              coef = "Intercept",
              group = "batch", nlpar = "A"
    ),
    set_prior("skew_normal(0.6, 0.5, 2.5)",
              class = "b", coef = "Intercept",
              nlpar = "B"
    ),
    set_prior("normal(0, 0.5)",
              class = "b", coef = "pretreatmentcold",
              nlpar = "B"
    ),
    set_prior("normal(0, 0.5)",
              class = "b", coef = "pretreatmentwarm",
              nlpar = "B"
    ),
    set_prior("exponential(10)", class = "sd",
              coef = "Intercept",
              group = "batch", nlpar = "B"
    ),
    set_prior("skew_normal(0.5, 0.1, 2.5)",
              class = "b", coef = "Intercept",
              nlpar = "C"
    ),
    set_prior("normal(0, 0.25)",
              class = "b", coef = "pretreatmentcold",
              nlpar = "C"
    ),
    set_prior("normal(0, 0.25)",
              class = "b", coef = "pretreatmentwarm",
              nlpar = "C"
    ),
    set_prior("exponential(25)", class = "sd",
              coef = "Intercept",
              group = "batch", nlpar = "C"
    ),
```

```
    set_prior("normal(0, 1.5)", class = "b",
              coef = "Intercept", nlpar = "D"),
    set_prior("skew_normal(0.5, 0.15, 5)", class = "b",
              coef = "cScaledMass", nlpar = "D"),
    set_prior("exponential(1.5)", class = "sd",
              coef = "Intercept", group = "ring", nlpar = "D"),
    set_prior("skew_normal(0, 0.25, 10)", class = "b",
              coef = "intercept", dpar = "sigma"),
    set_prior("skew_normal(0, 0.25, 10)", class = "b",
              coef = "logweekB", dpar = "sigma")
  ),
  family = "gaussian",
  seed = 103,
  cores = 4, chains = 4,
  iter = 50000, warmup = 10000, thin = 10,
  control = list(adapt_delta = 0.95, max_treedepth = 13),
  silent = TRUE, refresh = 0,
  sample_prior = "only",
  file = "./models/_ppCheckTarsus.Rds"
)

# Checking how posterior predictions overlay with true tarsus length values

pp_check2(growthModelTarsus_ppCheck, xlab = "Tarsus Length (mm)")
```

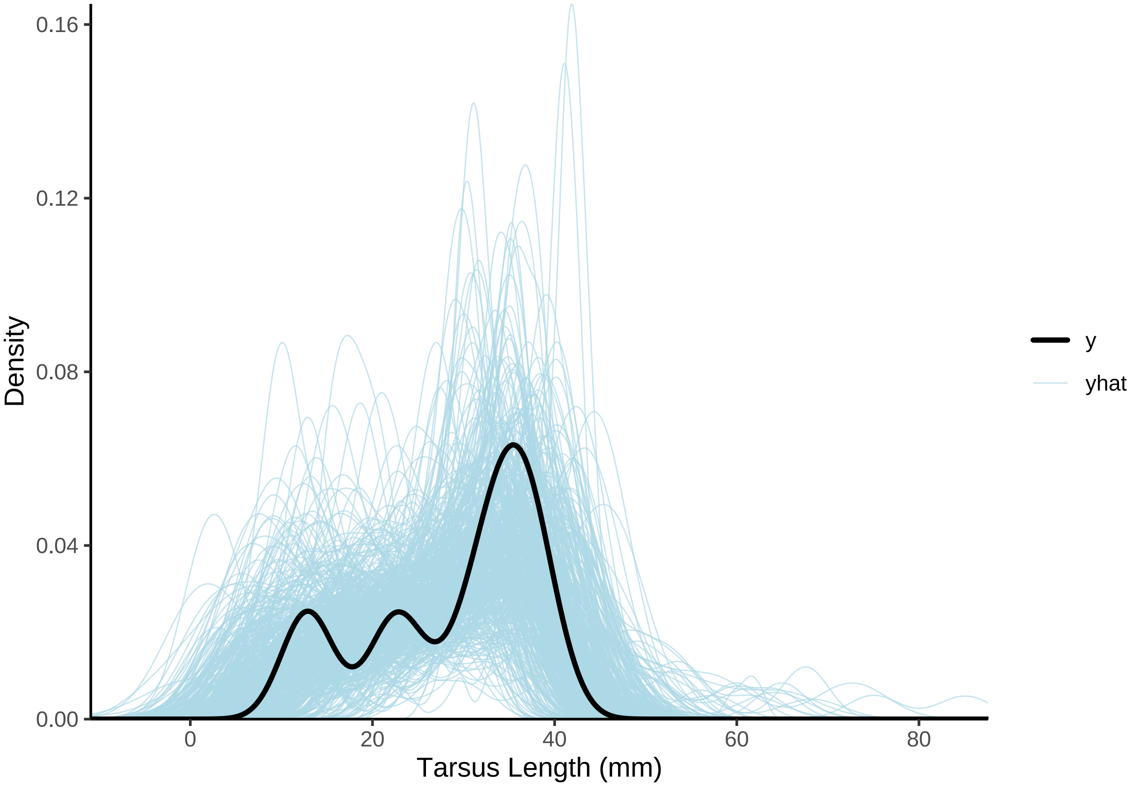

Figure 41: *Overlay of predicted (blue) and true (black) tarsus length densities, where predicted densities are derived from priors in a Bayesian non-linear model. Clear overlap betwen the black and blue lines suggests that model priors are reasonable with respect to the data.*

```
# Reasonable overlap, despite some individuals having
# unrealistically high tarsus length. Proceeding to full model,
# given our relative uncertainty around true parameter values.
```

Similar to our model estimating body mass during growth, initial values for our HMC chains are drawn from

within prior distributions to facilitate chain convergence.

```
initFunction <- function(chain_id = 1) {
  list(
    "b_A" = c(rnorm(1, 37.5, 2.5), -0.35, 0.35),
    "b_B" = c(1.35, 0, 0),
    "b_C" = c(0.5, 0, 0),
    "b_D" = c(rnorm(1, 0, 1.5),
              rskew_normal(1, xi = 0.5, omega = 0.15, alpha = 5)),
    "b_sigma" = rskew_normal(2, xi = 0, omega = 0.25, alpha = 10),
    "sd_1" = rexp(1, 1),
    "sd_2" = rexp(1, 10),
    "sd_3" = rexp(1, 25),
    "sd_4" = rexp(1, 0.5)
  )
}

# Loading initial values into a list.

initList <- lapply(1:4, initFunction)

growthModelTarsus <- brm(
  data = data %>%
    filter(week <= 8) %>%
    mutate(pretreatment = factor(pretreatment,
                                 levels = c("neutral", "cold", "warm")
    )) %>%
    dplyr::select(ring, pretreatment, week, mass,
           "tarsus" = tarsusLengthMean, "batch" = exp) %>%
    distinct() %>%
    mutate(
      weekB = week + 1
    ) %>%
    group_by(week) %>%
    mutate(cScaledMass = (mass - mean(mass, na.rm = T)) /
             sd(mass, na.rm = T)) %>%
    ungroup(),
  formula = bf(tarsus ~ A * exp(-B * exp(-C * week)) + D,
               A ~ 1 + pretreatment + (1|batch),
               B ~ 1 + pretreatment + (1|batch),
               C ~ 1 + pretreatment + (1|batch),
               D ~ 1 + cScaledMass + (1 | ring),
               sigma ~ 0 + intercept + log(weekB),
               nl = TRUE
  ),
  prior = c(
    set_prior("normal(37.5, 2.5)",
              class = "b", coef = "Intercept",
              nlpar = "A"
    ),
    set_prior("normal(-0.35, 2)",
              class = "b", coef = "pretreatmentcold",
              nlpar = "A"
    ),
    set_prior("normal(0.35, 2)",
              class = "b", coef = "pretreatmentwarm",
              nlpar = "A"
    ),
    set_prior("exponential(1)", class = "sd",
              coef = "Intercept",
              group = "batch", nlpar = "A"
    ),
    set_prior("skew_normal(0.6, 0.5, 2.5)",
              class = "b", coef = "Intercept",
              nlpar = "B"
    ),
    set_prior("normal(0, 0.5)",
```

```
              class = "b", coef = "pretreatmentcold",
              nlpar = "B"
    ),
    set_prior("normal(0, 0.5)",
              class = "b", coef = "pretreatmentwarm",
              nlpar = "B"
    ),
    set_prior("exponential(10)", class = "sd",
              coef = "Intercept",
              group = "batch", nlpar = "B"
    ),
    set_prior("skew_normal(0.5, 0.1, 2.5)",
              class = "b", coef = "Intercept",
              nlpar = "C"
    ),
    set_prior("normal(0, 0.25)",
              class = "b", coef = "pretreatmentcold",
              nlpar = "C"
    ),
    set_prior("normal(0, 0.25)",
              class = "b", coef = "pretreatmentwarm",
              nlpar = "C"
    ),
    set_prior("exponential(25)", class = "sd",
              coef = "Intercept",
              group = "batch", nlpar = "C"
    ),
    set_prior("normal(0, 1.5)", class = "b",
              coef = "Intercept", nlpar = "D"),
    set_prior("skew_normal(0.5, 0.15, 5)", class = "b",
              coef = "cScaledMass", nlpar = "D"),
    set_prior("exponential(1.5)", class = "sd",
              coef = "Intercept", group = "ring", nlpar = "D"),
    set_prior("skew_normal(0, 0.25, 10)", class = "b",
              coef = "intercept", dpar = "sigma"),
    set_prior("skew_normal(0, 0.25, 10)", class = "b",
              coef = "logweekB", dpar = "sigma")
  ),
  family = "gaussian",
  seed = 100,
  cores = 4, chains = 4, threads = 2,
  init = initList,
  iter = 50000, warmup = 10000, thin = 10,
  backend = "cmdstan",
  control = list(adapt_delta = 0.95, max_treedepth = 14),
  silent = TRUE, refresh = 0,
  file = "./models/_growthModelTarsus.Rds"
)

# Taking a look at effective sample size to sample size
# ratios and Gelman-Rubin statistics.

ggarrange(
  ggplot(data = data.frame("Rhat" = brms::rhat(growthModelTarsus)),
         aes(x = Rhat)) +
    geom_density() +
    theme_classic() +
    xlab(
      TeX('$\\hat{R}$')
    ) +
    ylab("Density"),
  ggplot(data = data.frame("Neff" = neffBase(growthModelTarsus)),
         aes(x = Neff)) +
    geom_density() +
    theme_classic() +
    xlab(
      TeX('$N_{eff}/N~Ratio$')
```

```
    ) +
    ylab("Density")
)
```

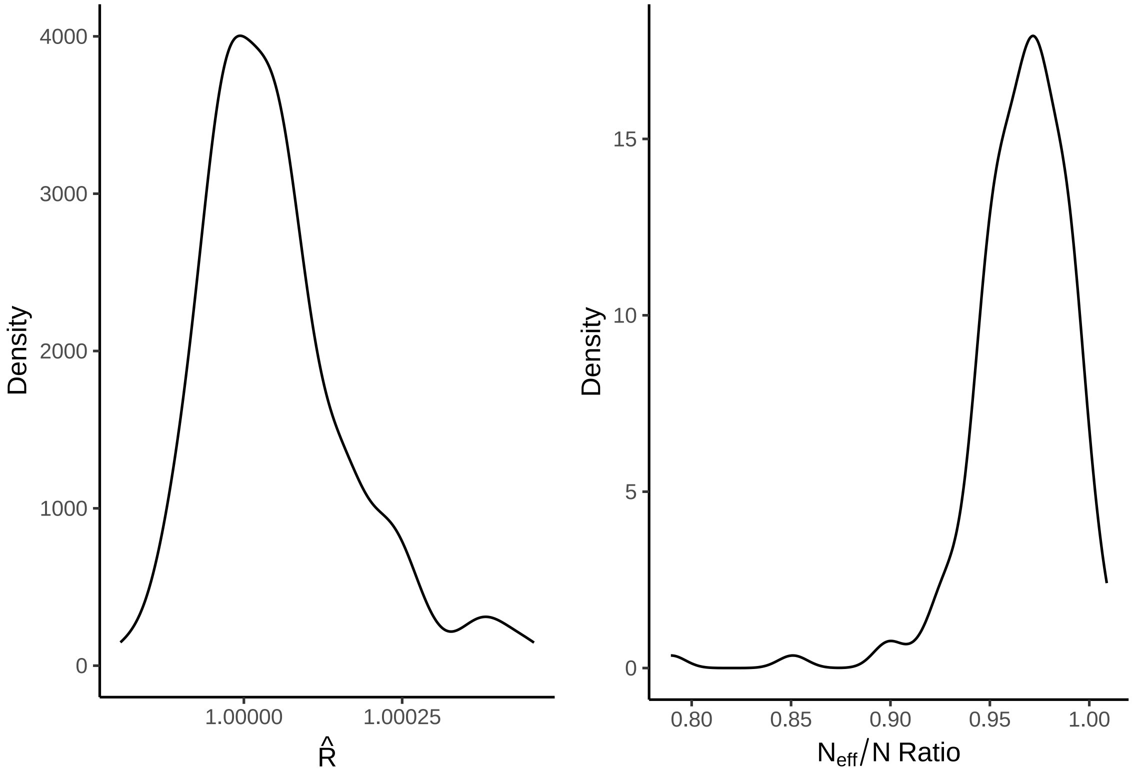

Figure 42: *Gelman-Rubin statistics ($\hat{R}$) and ratio of effective samples sizes by samples sizes per parameter from a Bayesian non-linear model estimating tarsus length (mm) among Japanese quail.*

```
# Some loss in effective sample sizes but acceptable.
```

```
pp_check2(growthModelTarsus, xlab = "Tarsus Length (mm)")
```

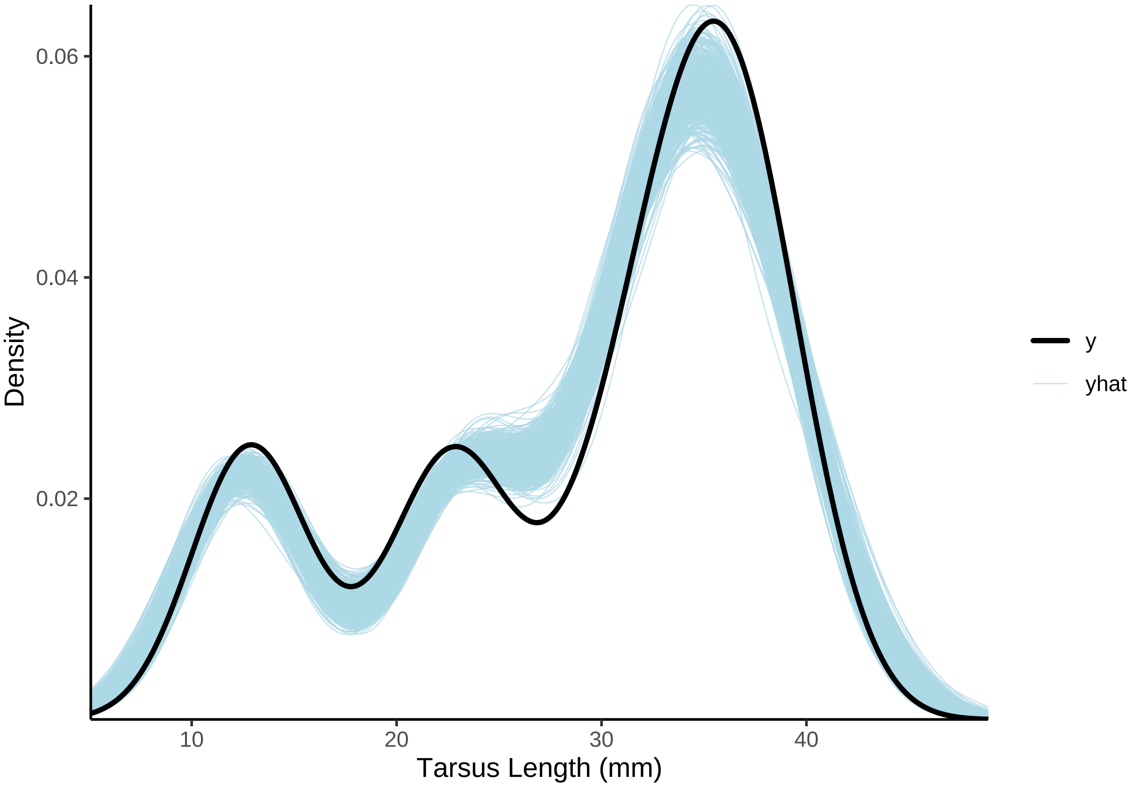

Figure 43: *Posterior predictions of a Bayesian non-linear model predicting Japanese tarsus length (mm) across time, overlayed with true distributions of quail mass. Blue lines represent predictions from posterior draws, while the black line represents true mass distributions.*

```
# Distinct overlay. Checking fit with a scatterplot.
```

```
growthModelTarsus$data %>%
  mutate("Fit" = predict(growthModelTarsus,
                         robust = TRUE)[, "Estimate"]) %>%
  ggplot(aes(x = tarsus, y = Fit, fill = week)) +
  geom_point(pch = 21, colour = "black", size = 2, alpha = 0.5) +
  geom_smooth(method = "lm", colour = "black",
              linetype = "dashed", se = FALSE) +
  scale_fill_gradient2() +
  theme_classic() +
  xlab("Tarsus Length (mm)") +
  ylab("Predicted Tarsus Length (mm)")
```

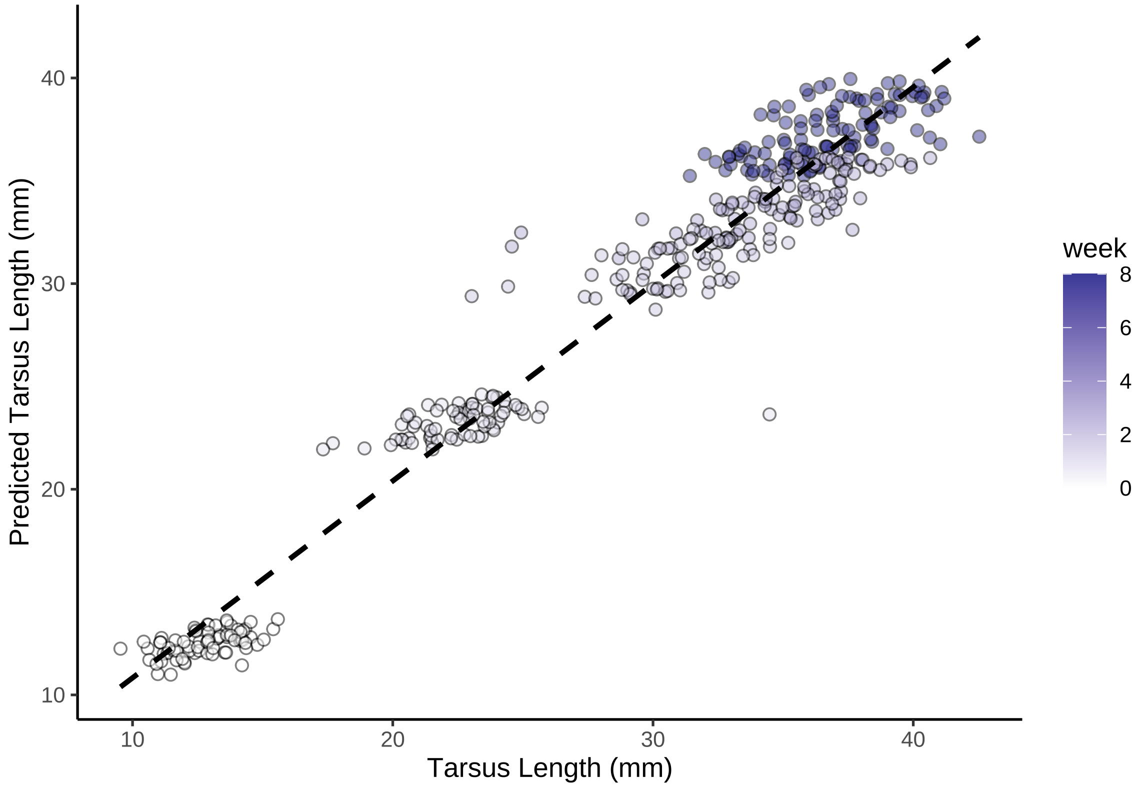

Figure 44: *Scatterplot of Japanese quail tarsus length (mm) against predicted tarsus length (mm) from a Bayesian non-linear model. Dashed line indicates line of best fit, as estimated by the R package ggplot2 (Wickham, 2011).*

```
# Slightly over-fitting at 0 weeks of age (suggesting that
# 'b' may be slightly over-estimated), but otherwise acceptable.
# Plotting mean responses
```

Posterior predictions appear to overlay with raw data well. Below, tarsus elongation curves are predicted from our model for each rearing treatment. These curves are then plotted and overlaid with raw tarsus length means, per treatment, across our observed period of elongation.

```
with(
  growthModelTarsus$data,
  expand.grid(
    "week" = seq(0, 8, by = 0.1),
    "pretreatment" = c("cold", "neutral", "warm"),
    "cScaledMass" = 0
  )
) %>%
  mutate("weekB" = week + 1) %>%
  mutate(
    "Fit" = predict(growthModelTarsus, re_form = NA,
                    newdata = .,
                    robust = TRUE)[, "Estimate"],
    "SE" = predict(growthModelTarsus, re_form = NA,
                   newdata = .,
                    robust = TRUE)[, "Est.Error"]
  ) %>%
  mutate(
    "LCL" = Fit - SE,
    "UCL" = Fit + SE
  ) %>%
```

```
mutate(pretreatment = ifelse(pretreatment == "cold",
                             "Cold (10°C)",
                             ifelse(pretreatment == "neutral",
                                    "Mild (20°C)",
                                    "Warm (30°C)"))) %>%
mutate(pretreatment = factor(pretreatment,
                             levels =
                               c("Cold (10°C)",
                                 "Mild (20°C)",
                                "Warm (30°C)")
)
) %>%
ggplot(aes(x = week, y = Fit,
 fill = pretreatment, linetype = pretreatment)) +
geom_ribbon(aes(x = week, ymin = LCL, ymax = UCL),
  colour = NA, size = 0.25, alpha = 0.3
) +
geom_line(colour = "black", alpha = 0.7) +
stat_summary(
  data = growthModelTarsus$data %>%
  mutate(pretreatment = ifelse(pretreatment == "cold",
                             "Cold (10°C)",
                             ifelse(pretreatment == "neutral",
                                    "Mild (20°C)",
                                    "Warm (30°C)"))) %>%
  mutate(pretreatment = factor(pretreatment,
                             levels =
                               c("Cold (10°C)",
                                "Mild (20°C)",
                                "Warm (30°C)")
)
),
  aes(x = week, y = tarsus),
  geom = "errorbar", fun.data = "mean_cl_boot",
  colour = "black", alpha = 0.7, width = 0.25,
  position = position_dodge(width = 0.15)
) +
stat_summary(
  data = growthModelTarsus$data %>%
  mutate(pretreatment = ifelse(pretreatment == "cold",
                             "Cold (10°C)",
                             ifelse(pretreatment == "neutral",
                                    "Mild (20°C)",
                                    "Warm (30°C)"))) %>%
  mutate(pretreatment = factor(pretreatment,
                             levels =
                               c("Cold (10°C)",
                                "Mild (20°C)",
                                "Warm (30°C)")
)
),
  aes(x = week, y = tarsus),
  geom = "point", fun = "mean", pch = 21, size = 3,
  colour = "black", alpha = 0.7,
  position = position_dodge(width = 0.15)
) +
theme_classic() +
scale_fill_manual(values = c("#7BB4E3", "black", "#CD5C5C"),
                  name = "Rearing\nConditions") +
scale_linetype_manual(values = c("dotted", "solid", "dashed"),
                      name = "Rearing\nConditions") +
xlab("Age (weeks)") +
ylab("Tarsus Length (mm)")
```

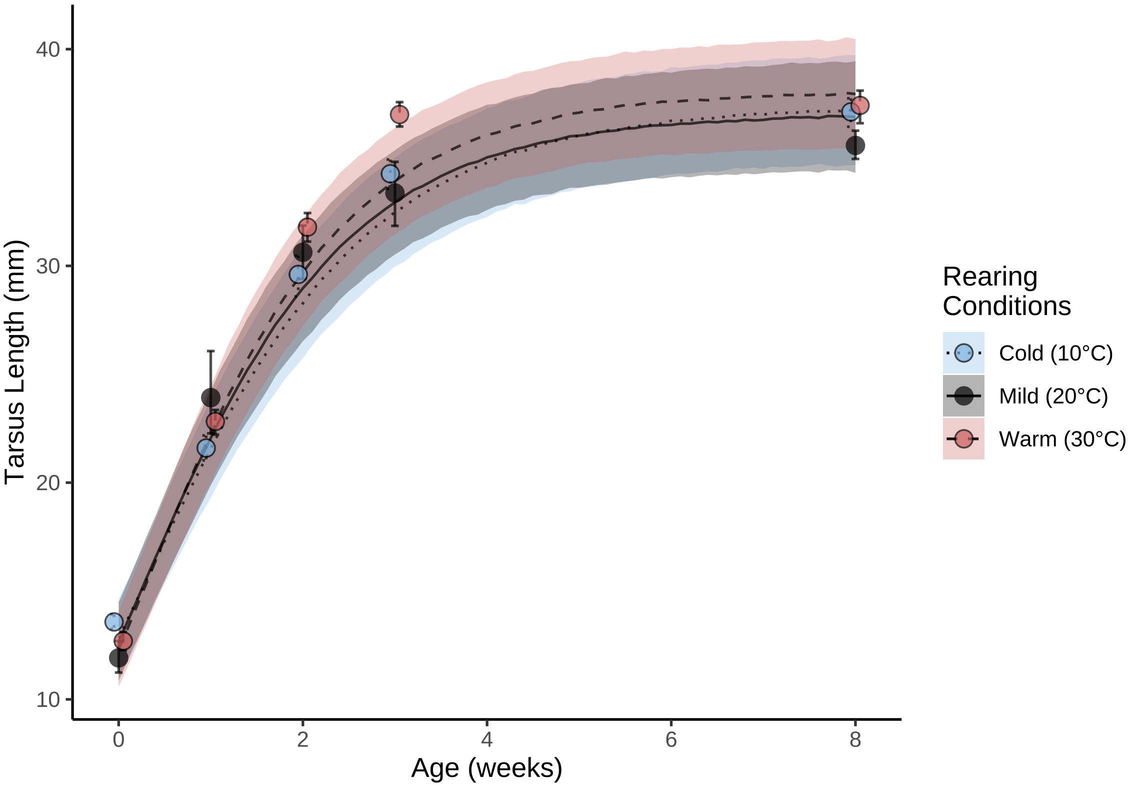

Figure 45: *Tarsus length (mm) elongation curves of Japanese quail reared in the cold (10°C), mild conditions (20°C), or the warm (30°C) until at least 3 weeks of age. Dots represent mean values and errorbars represent quantile-based 95% credible intervals. Lines represent estimated trends in growth from a Bayesian non-linear model, while ribbons represent confidence around trends (± one standard error).*

```
# Good. Very rapid growth proceeded by some weak convergence
# among groups at asymptotes. Checking residuals next.
```

Next, residuals are again visualised to check for heteroskedasticity.

```
p1 <- growthModelTarsus$data %>%
  mutate("residuals" = residuals(growthModelTarsus,
                                 method = "posterior_predict",
                                 type = "pearson",
                                 robust = TRUE)[, "Estimate"]) %>%
  ggplot(aes(x = residuals)) +
  geom_density(colour = "black", fill = "white") +
  xlab("Pearson Residuals") +
  ylab("Density") +
  theme_classic()

p2 <- growthModelTarsus$data %>%
  mutate(
    "residuals" = residuals(growthModelTarsus,
      method = "posterior_predict",
      type = "pearson",
      robust = TRUE
    )[, "Estimate"],
    "fitted" = fitted(growthModelTarsus,
                                 robust = TRUE)[, "Estimate"]
  ) %>%
  ggplot(aes(x = fitted, y = residuals)) +
  geom_point(colour = "black", pch = 21, size = 2,
```

```
           fill = "grey75", alpha = 0.5) +
  ylab("Pearson Residuals") +
  xlab("Fitted Values (mm)") +
  theme_classic()

p3 <- growthModelTarsus$data %>%
  mutate("residuals" =
         residuals(growthModelTarsus,
                   method = "posterior_predict",
                   type = "pearson",
                   robust = TRUE
  )[, "Estimate"]) %>%
  ggplot(aes(x = week, y = residuals)) +
  geom_point(
    colour = "black", pch = 21, size = 2, fill = "grey75", alpha = 0.5,
    position = position_jitter(width = 0.25)
  ) +
  stat_summary(geom = "errorbar", fun.data = "mean_se",
               colour = "black", width = 0.25) +
  stat_summary(geom = "point", fun = "mean", pch = 21,
               colour = "black", fill = "white", size = 4) +
  ylab("Pearson Residuals") +
  xlab("Age (weeks)") +
  theme_classic()

p4 <- growthModelTarsus$data %>%
  mutate("residuals" =
         residuals(growthModelTarsus,
                   method = "posterior_predict",
                   type = "pearson",
                   robust = TRUE
  )[, "Estimate"]) %>%
  ggplot(aes(x = cScaledMass, y = residuals)) +
  geom_point(
    colour = "black", pch = 21, size = 2, fill = "grey75", alpha = 0.5,
    position = position_jitter(width = 0.25)
  ) +
  stat_summary(geom = "errorbar", fun.data = "mean_se",
               colour = "black", width = 0.25) +
  stat_summary(geom = "point", fun = "mean", pch = 21,
               colour = "black", fill = "white", size = 4) +
  ylab("Pearson Residuals") +
  xlab("Centred and Scaled Body Mass") +
  theme_classic()

p5 <- growthModelTarsus$data %>%
  mutate("residuals" =
         residuals(growthModelTarsus,
                   method = "posterior_predict",
                   type = "pearson",
                   robust = TRUE
  )[, "Estimate"]) %>%
  mutate(pretreatment = str_to_title(pretreatment)) %>%
  mutate(pretreatment = ifelse(pretreatment == "Cold",
                               "Cold\n(10°C)",
    ifelse(pretreatment == "Neutral", "Mild\n(20°C)",
      "Warm\n(30°C)"
    )
  )) %>%
  mutate(pretreatment = factor(pretreatment,
                               levels = c("Cold\n(10°C)",
                                          "Mild\n(20°C)",
                                          "Warm\n(30°C)"))) %>%
  ggplot(aes(x = pretreatment, y = residuals)) +
  geom_point(
    colour = "black", pch = 21, size = 2, fill = "grey75", alpha = 0.5,
    position = position_jitter(width = 0.25)
```

```
  ) +
  stat_summary(geom = "errorbar", fun.data = "mean_se",
               colour = "black", width = 0.25) +
  stat_summary(geom = "point", fun = "mean", pch = 21,
               colour = "black", fill = "white", size = 4) +
  ylab("Pearson Residuals") +
  xlab("Rearing Conditions)") +
  theme_classic()

((p1 + p2) / (p3 + p4)) + p5
```

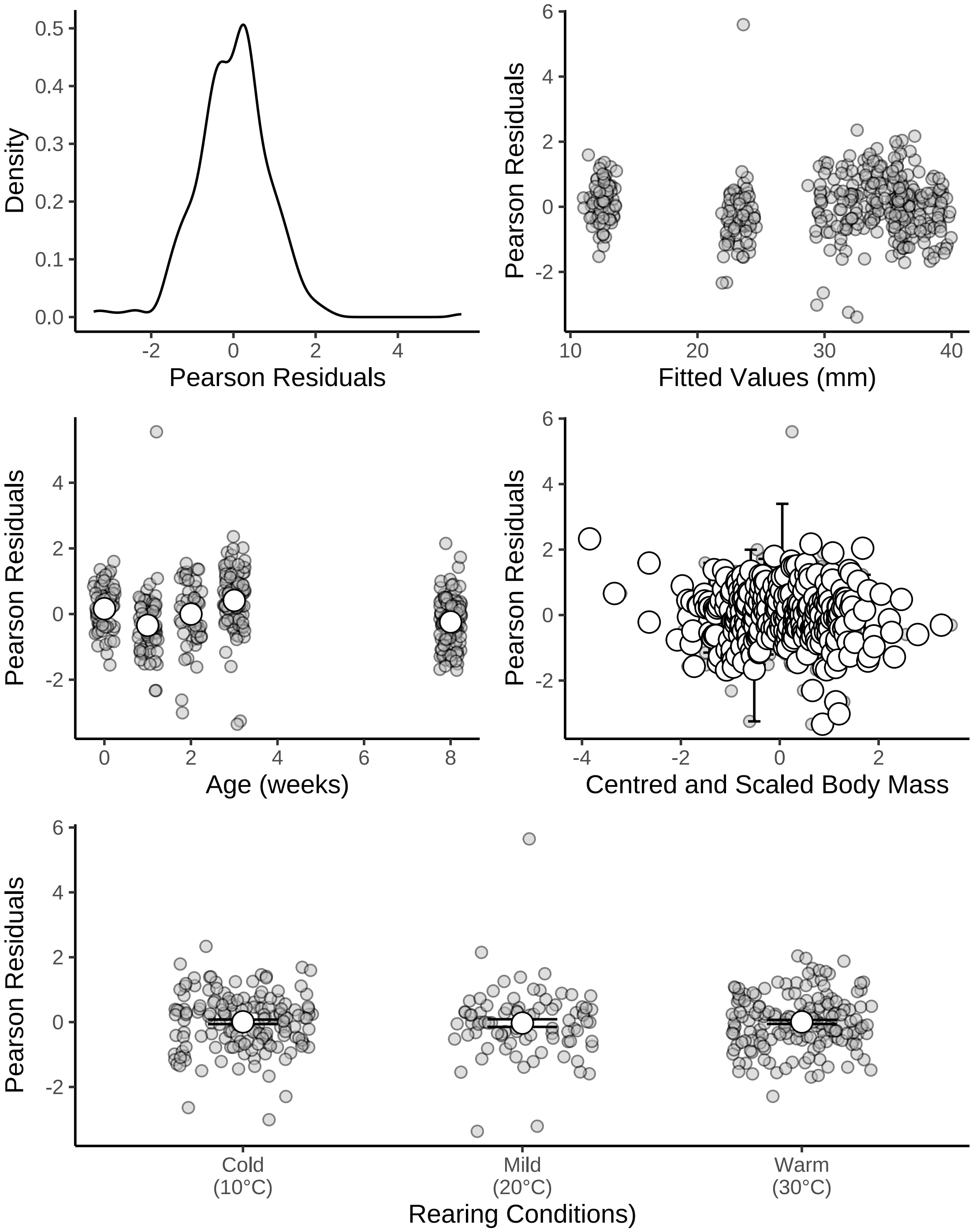

Figure 46: *Density and distributions of Pearson residuals across fitted values and model predictors (including age and rearing conditions). All residuals pertain to those drawn from a Bayesian non-linear model predicting tarsus length (mm) during growth in Japanese quail. Pearson residuals are shown rather than ordinary residuals to correct for the age-dependence of model error.*

Residuals appear homogenous but reveal one potential extreme value. Below, the value is identified and scrutinised.

```
caption <- paste0("Possible outlier identified from Bayesian ",
                  "non-linear model predicting tarsus length (mm) ",
                  "of Japanese quail across growth."
)

growthModelTarsus$data %>%
  mutate("Residuals" = residuals(growthModelTarsus,
                                 type = "pearson",
                                 robust = TRUE)[, "Estimate"]) %>%
  filter(Residuals > 4) %>%
  dplyr::select("Bird Identity" = ring, "Age (weeks)" = week,
         "Centred & Scaled Mass (g)" = cScaledMass,
         "Tarsus Length (mm)" = tarsus) %>%
  kbl(., longtable = T, booktabs = T, format = "latex",
      caption = caption) %>%
  column_spec(column = c(1:10), width = "2.5cm")  %>%
  kable_styling(latex_options = "striped")
```

Table 16: *Possible outlier identified from Bayesian non-linear model predicting tarsus length (mm) of Japanese quail across growth.*

Bird Identity	Age (weeks)	Centred & Scaled Mass (g)	Tarsus Length (mm)
R3	1	0.0516127	34.477

```
# Clearly a large tarsus length for its age. Plotting trends
# in this individuals' tarsus length across time.

growthModelTarsus$data %>%
  filter(ring == "R3") %>%
  ggplot(aes(x = week, y = tarsus)) +
  geom_line(colour = "black", linetype = "dashed", size = 0.5) +
  geom_point(size = 3, pch = 21, colour = "black", fill = "lightblue2") +
  xlab("Age (weeks)") +
  ylab("Tarsus Length (mm)") +
  theme_classic()
```

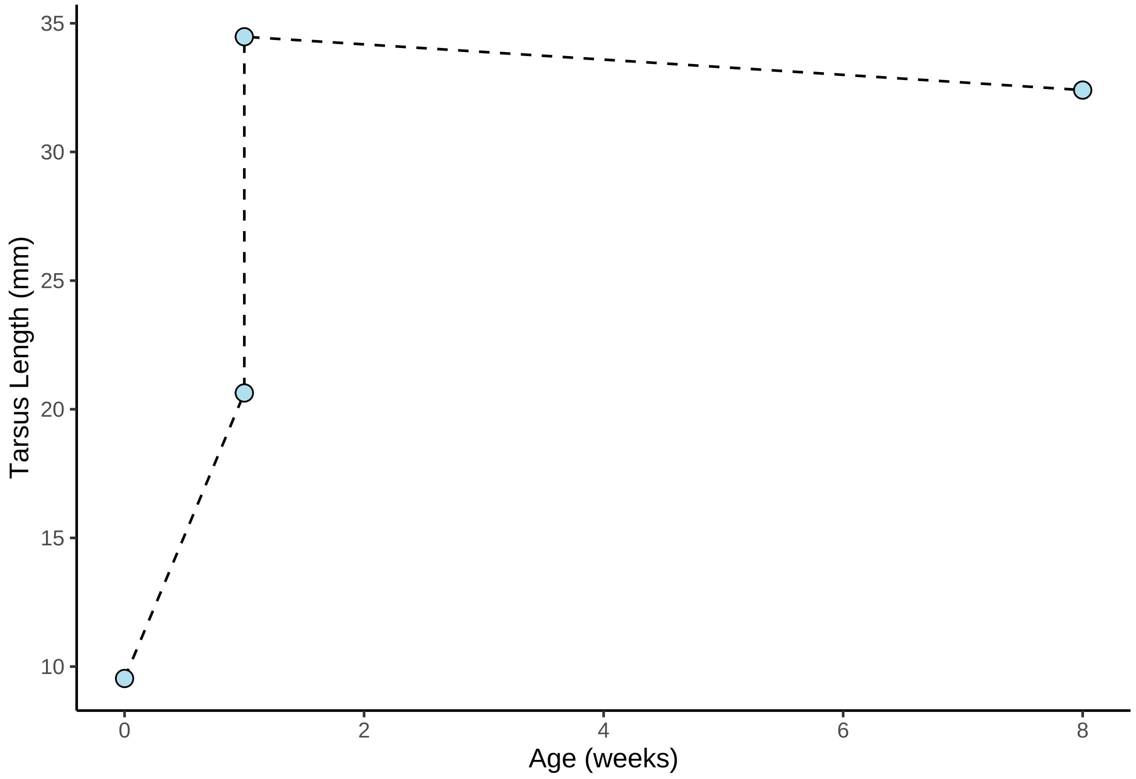

Figure 47: *Tarsus elongation trends of possible outlier identified from Bayesian non-linear model predicting tarsus length (mm) of across growth in Japanese quail.*

```
# Two measurements labelled as being drawn from one. Assessing why.

caption <- paste0("Further information regarding possible tarsus ",
                  "length outlier identified from Bayesian non-linear ",
                  "model predicting tarsus length (mm) of ",
                  "Japanese quail across growth."
)

data %>%
  filter(ring == "R3" & week == 1) %>%
  mutate(
    sex = str_to_title(sex),
    tarsusCalibration = ifelse(tarsusCalibration == "gridUnder", "Grid Under",
      ifelse(tarsusCalibration == "gridOver", "Grid Over",
        tarsusCalibration
      )
    )
  ) %>%
  dplyr::select(
    "Bird Identity" = ring, "Age (weeks)" = week,
    "Sex" = sex, "Body Mass (g)" = mass,
    "Tarsus Length (mm)" = tarsusLengthMean,
    "Calibration Method" = tarsusCalibration
  ) %>%
  kbl(., longtable = T, booktabs = T,
      caption = caption, format = "latex") %>%
  column_spec(column = c(1:10), width = "2cm")  %>%
  kable_styling(latex_options = "striped")
```

Table 17: *Further information regarding possible tarsus length outlier identified from Bayesian non-linear model predicting tarsus length (mm) of Japanese quail across growth.*

Bird Identity	Age (weeks)	Sex	Body Mass (g)	Tarsus Length (mm)	Calibration Method
R3	1	Male	34.8	20.63328	Grid Under
R3	1	Male	34.8	34.47700	Grid Over

```
# Two different calibration methods used, so duplicates
# are not true duplications. Given that both tarsus length
# measurements are within the realistic range of
# Japanese quail morphometrics, both values should be retained.
# Nevertheless, our model is re-run without this data-point to
# dispel concerns about our bias entering our model coefficients.
```

To ensure that potential outliers are not biasing our model outcomes, our model is re-run without their inclusion and coefficients are visually compared between model iterations. Importantly, our revised model is ran with two HMC chains rather than four to improve computational efficiency.

```
initList <- lapply(1:2, initFunction)

growthModelTarsusB <- brm(
  data = data %>%
    filter(!(ring == "R3" & week == 1 &
               tarsusLengthMean > 34)) %>%
    filter(week <= 8) %>%
    mutate(pretreatment = factor(pretreatment,
      levels = c("neutral", "cold", "warm")
    )) %>%
    dplyr::select(ring, pretreatment, week, mass, "batch" = exp,
           "tarsus" = tarsusLengthMean) %>%
    distinct() %>%
    mutate(weekB = week + 1) %>%
    group_by(week) %>%
    mutate(cScaledMass = (mass - mean(mass, na.rm = T)) /
             sd(mass, na.rm = T)) %>%
    ungroup(),
  formula = bf(tarsus ~ A * exp(-B * exp(-C * week)) + D,
               A ~ 1 + pretreatment + (1|batch),
               B ~ 1 + pretreatment + (1|batch),
               C ~ 1 + pretreatment + (1|batch),
               D ~ 1 + cScaledMass + (1 | ring),
               sigma ~ 0 + intercept + log(weekB),
               nl = TRUE
  ),
  prior = c(
    set_prior("normal(37.5, 2.5)",
              class = "b", coef = "Intercept",
              nlpar = "A"
    ),
    set_prior("normal(-0.35, 2)",
              class = "b", coef = "pretreatmentcold",
              nlpar = "A"
    ),
    set_prior("normal(0.35, 2)",
              class = "b", coef = "pretreatmentwarm",
              nlpar = "A"
    ),
    set_prior("exponential(1)", class = "sd",
              coef = "Intercept",
              group = "batch", nlpar = "A"
    ),
    set_prior("skew_normal(0.6, 0.5, 2.5)",
              class = "b", coef = "Intercept",
```

```
              nlpar = "B"
    ),
    set_prior("normal(0, 0.5)",
              class = "b", coef = "pretreatmentcold",
              nlpar = "B"
    ),
    set_prior("normal(0, 0.5)",
              class = "b", coef = "pretreatmentwarm",
              nlpar = "B"
    ),
    set_prior("exponential(10)", class = "sd",
              coef = "Intercept",
              group = "batch", nlpar = "B"
    ),
    set_prior("skew_normal(0.5, 0.1, 2.5)",
              class = "b", coef = "Intercept",
              nlpar = "C"
    ),
    set_prior("normal(0, 0.25)",
              class = "b", coef = "pretreatmentcold",
              nlpar = "C"
    ),
    set_prior("normal(0, 0.25)",
              class = "b", coef = "pretreatmentwarm",
              nlpar = "C"
    ),
    set_prior("exponential(25)", class = "sd",
              coef = "Intercept",
              group = "batch", nlpar = "C"
    ),
    set_prior("normal(0, 1.5)", class = "b",
              coef = "Intercept", nlpar = "D"),
    set_prior("skew_normal(0.5, 0.15, 5)", class = "b",
              coef = "cScaledMass", nlpar = "D"),
    set_prior("exponential(1.5)", class = "sd",
              coef = "Intercept", group = "ring", nlpar = "D"),
    set_prior("skew_normal(0, 0.25, 10)", class = "b",
              coef = "intercept", dpar = "sigma"),
    set_prior("skew_normal(0, 0.25, 10)", class = "b",
              coef = "logweekB", dpar = "sigma")
  ),
  family = "gaussian",
  seed = 100,
  cores = 2, chains = 2, threads = 2,
  init = initList,
  iter = 50000, warmup = 10000, thin = 10,
  backend = "cmdstan",
  control = list(adapt_delta = 0.95, max_treedepth = 13),
  silent = TRUE, refresh = 0,
  file = "./models/_growthModelTarsusB.Rds"
)

# Comparing model coefficients visually.

as.data.frame(growthModelTarsus) %>%
  pivot_longer(everything(), names_to = "Parameter",
               values_to = "Values") %>%
  filter(grepl("b_|sd_", Parameter)) %>%
  arrange(Parameter) %>%
  mutate("model" = "Model A") %>%
  rbind(
    .,
    as.data.frame(growthModelTarsusB) %>%
      pivot_longer(everything(), names_to = "Parameter",
                   values_to = "Values") %>%
      filter(grepl("b_|sd_", Parameter)) %>%
      arrange(Parameter) %>%
```

```
    mutate("model" = "Model B")
) %>%
merge(., tribble(
  ~Parameter, ~Par,
  "b_A_Intercept", "Beta a0",
  "b_A_pretreatmentcold", "Beta a1\n(Cold-reared)",
  "b_A_pretreatmentwarm", "Beta a2\n(Warm-reared)",
  "sd_batch__A_Intercept", "Mu 0a",
  "b_B_Intercept", "Beta b0",
  "b_B_pretreatmentcold", "Beta b1\n(Cold-reared)",
  "b_B_pretreatmentwarm", "Beta b2\n(Warm-reared)",
  "sd_batch__B_Intercept", "Mu 0b",
  "b_C_Intercept", "Beta c0",
  "b_C_pretreatmentcold", "Beta c1\n(Cold-reared)",
  "b_C_pretreatmentwarm", "Beta c2\n(Warm-reared)",
  "sd_batch__C_Intercept", "Mu 0c",
  "b_D_Intercept", "Beta 0",
  "b_D_cScaledMass", "Beta 1",
  "sd_ring__D_Intercept", "Mu 0 (Individual\nIntercept)",
  "b_sigma_intercept", "Tau 0",
  "b_sigma_logweekB", "Tau 1"
),
by = "Parameter", all.x = TRUE
) %>%
ggplot(aes(x = Values, fill = model)) +
facet_wrap(~Par, scales = "free") +
geom_density(colour = "black", alpha = 0.5) +
geom_vline(xintercept = 0, linetype = "dashed", colour = "firebrick4") +
ylab("Density") +
scale_fill_manual(values = c("lightblue2", "grey40"), name = NULL) +
theme_classic() +
theme(axis.title.x = element_blank(),
      legend.position = "bottom")
```

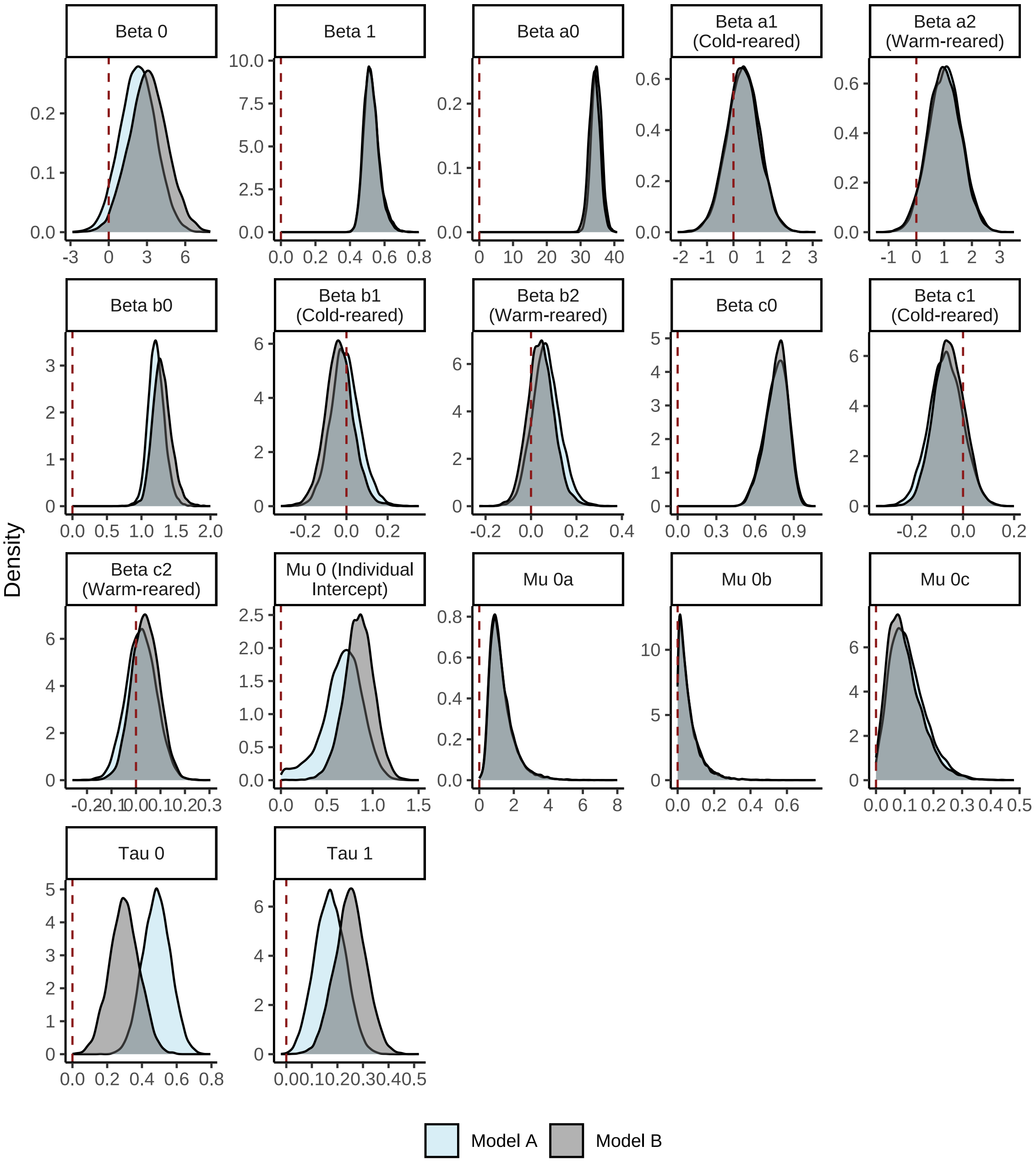

Figure 48: *Comparions of model coefficients derived from two, Bayesian, non-linear models predicting tarsus length (mm) of Japanese quail between 0 and 8 weeks of age. 'Model A' includes one possible outlier (as determined by visually inspecting model residuals), and 'Model B' excludes this outlier. Densities represent posterior densities for each coefficient.*

Some distinctions in variance and error structure emerge after the potential outlier is removed, but all population-level coefficients remain largely unchanged. As such, our initial model is summarised going forward.

```
as.data.frame(growthModelTarsus) %>%
  pivot_longer(everything(), names_to = "Parameter", values_to = "Values") %>%
  filter(grepl("b_|sd_", Parameter)) %>%
  arrange(Parameter) %>%
  merge(., tribble(
```

```
    ~Parameter, ~Par,
    "b_A_Intercept", "Beta a0",
    "b_A_pretreatmentcold", "Beta a1\n(Cold-reared)",
    "b_A_pretreatmentwarm", "Beta a2\n(Warm-reared)",
    "sd_batch__A_Intercept", "Mu 0a",
    "b_B_Intercept", "Beta b0",
    "b_B_pretreatmentcold", "Beta b1\n(Cold-reared)",
    "b_B_pretreatmentwarm", "Beta b2\n(Warm-reared)",
    "sd_batch__B_Intercept", "Mu 0b",
    "b_C_Intercept", "Beta c0",
    "b_C_pretreatmentcold", "Beta c1\n(Cold-reared)",
    "b_C_pretreatmentwarm", "Beta c2\n(Warm-reared)",
    "sd_batch__C_Intercept", "Mu 0c",
    "b_D_Intercept", "Beta 0",
    "b_D_centredMass", "Beta 1",
    "sd_ring__D_Intercept", "Mu 0\n(Individual Intercept)",
    "b_sigma_intercept", "Tau 0",
    "b_sigma_logweekB", "Tau 1"
  ),
  by = "Parameter", all.x = TRUE
  ) %>%
  filter(!is.na(Par)) %>%
  ggplot(aes(x = Values)) +
  facet_wrap(~Par, scales = "free") +
  geom_density() +
  geom_vline(
    xintercept = 0, linetype = "dashed",
    colour = "firebrick4"
  ) +
  ylab("Density") +
  theme_classic() +
  theme(axis.title.x = element_blank())
```

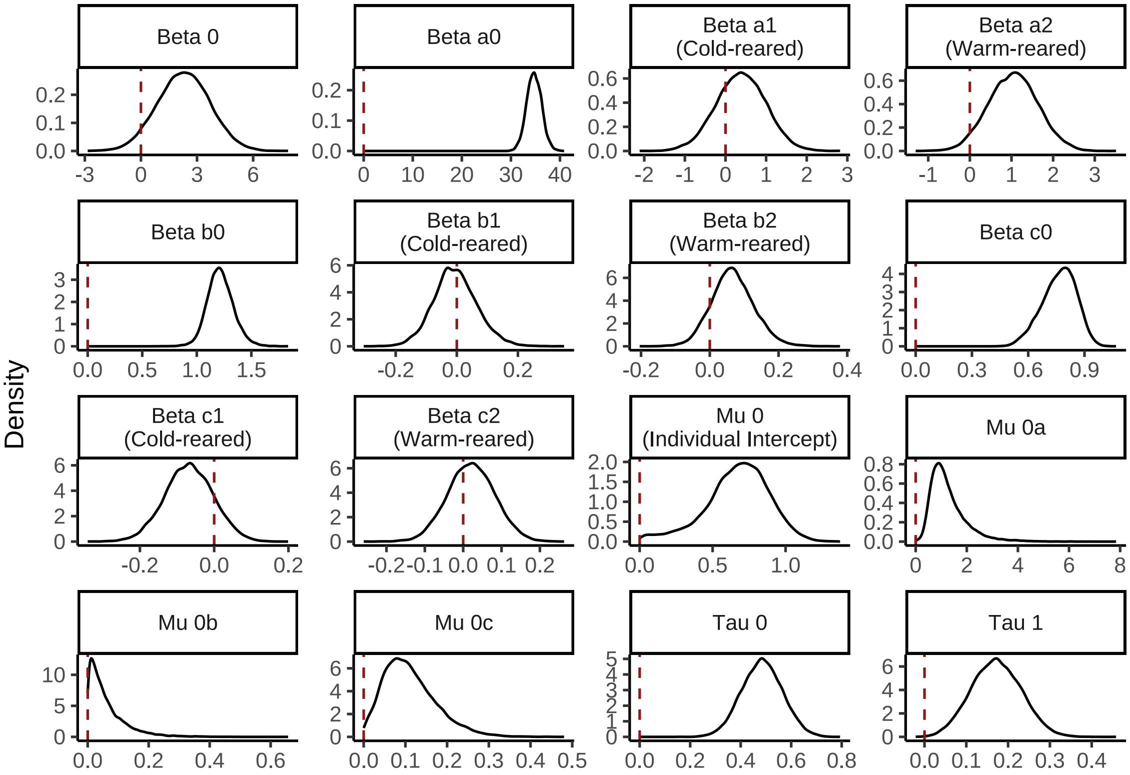

Figure 49: *Density of model coefficients from a Bayesian non-linear model predicting tarsus length (mm) of Japanese quail during growth. Dashed red lines indicate zero values for each coefficient.*

```
# Growth rate (parameter 'c') slowed among cold-reared
# birds, and asymptote evidently raised in warm-reared birds.
# Summarising model outcomes.

caption <- paste0(
  "Coefficients from a Bayesian non-linear effects ",
  "model predicting tarsus length (mm) of Japanese quail as a ",
  "Gompertz function of age in weeks. Coefficients represent ",
  "posterior medians and are estimated from body mass data ",
  "collected weekly between 0 and 8 weeks of age. Credible ",
  "intervals (CIs) represent quantile ",
  "intervals around medians"
)

tarsusGrowthModelTable <-
  merge(
    as.data.frame(growthModelTarsus) %>%
      summarise_all(., .funs = median) %>%
      mutate_all(., .funs = round, 3) %>%
      pivot_longer(everything(),
        names_to = "Parameter",
        values_to = "Values"
      ) %>%
      filter(grepl("b_|sd_", Parameter)) %>%
      arrange(Parameter),
    quantileCIs(growthModelTarsus, cis = c(50, 95)) %>%
      mutate(
        `50\\% CIs` = paste0(
          "[", round(Low_CI_50, digits = 3),
          ", ", round(High_CI_50, digits = 3), "]"
        ),
        `95\\% CIs` = paste0(
          "[", round(Low_CI_95, digits = 3),
          ", ", round(High_CI_95, digits = 3), "]"
        )
      ) %>%
      dplyr::select(Parameter, `50\\% CIs`, `95\\% CIs`),
    by = "Parameter"
  ) %>%
  merge(., tribble(
    ~Parameter, ~Par,
    "b_A_Intercept", "Beta a0",
    "b_A_pretreatmentcold", "Beta a1 (Cold-reared)",
    "b_A_pretreatmentwarm", "Beta a2 (Warm-reared)",
    "sd_batch__A_Intercept", "Mu 0a",
    "b_B_Intercept", "Beta b0",
    "b_B_pretreatmentcold", "Beta b1 (Cold-reared)",
    "b_B_pretreatmentwarm", "Beta b2 (Warm-reared)",
    "sd_batch__B_Intercept", "Mu 0b",
    "b_C_Intercept", "Beta c0",
    "b_C_pretreatmentcold", "Beta c1 (Cold-reared)",
    "b_C_pretreatmentwarm", "Beta c2 (Warm-reared)",
    "sd_batch__C_Intercept", "Mu 0c",
    "b_D_Intercept", "Beta 0",
    "b_D_cScaledMass", "Beta 1",
    "sd_ring__D_Intercept", "Mu 0 (Individual Intercept)",
    "b_sigma_intercept", "Tau 0",
    "b_sigma_logweekB", "Tau 1"
  ),
  by = "Parameter", all.x = TRUE
  ) %>%
  dplyr::select(-Parameter) %>%
  dplyr::select(
    "Parameter" = Par, "Value" = Values, `50\\% CIs`, `95\\% CIs`
  ) %>%
  kbl(.,
    longtable = T, booktabs = T, format = "latex",
```

```
    escape = FALSE, caption = caption
  ) %>%
  column_spec(column = c(1:10), width = "2.5cm") %>%
  kable_styling(latex_options = "striped")

tarsusGrowthModelTable
```

Table 18: *Coefficients from a Bayesian non-linear effects model predicting tarsus length (mm) of Japanese quail as a Gompertz function of age in weeks. Coefficients represent posterior medians and are estimated from body mass data collected weekly between 0 and 8 weeks of age. Credible intervals (CIs) represent quantile intervals around medians*

Parameter	Value	50% CIs	95% CIs
Beta a0	34.722	[33.705, 35.78]	[31.742, 37.802]
Beta a1 (Cold-reared)	0.384	[-0.033, 0.797]	[-0.829, 1.561]
Beta a2 (Warm-reared)	1.049	[0.642, 1.441]	[-0.099, 2.21]
Beta b0	1.211	[1.138, 1.291]	[0.994, 1.468]
Beta b1 (Cold-reared)	-0.007	[-0.052, 0.04]	[-0.143, 0.144]
Beta b2 (Warm-reared)	0.065	[0.026, 0.106]	[-0.049, 0.195]
Beta c0	0.772	[0.705, 0.83]	[0.572, 0.922]
Beta c1 (Cold-reared)	-0.068	[-0.111, -0.023]	[-0.195, 0.06]
Beta c2 (Warm-reared)	0.021	[-0.02, 0.063]	[-0.098, 0.141]
Beta 1	0.522	[0.495, 0.553]	[0.45, 0.634]
Beta 0	2.297	[1.347, 3.24]	[-0.44, 5.025]
Tau 0	0.484	[0.43, 0.539]	[0.327, 0.642]
Tau 1	0.171	[0.131, 0.213]	[0.059, 0.289]
Mu 0a	1.101	[0.785, 1.56]	[0.384, 3.102]
Mu 0b	0.043	[0.018, 0.084]	[0.002, 0.232]
Mu 0c	0.102	[0.065, 0.147]	[0.015, 0.257]
Mu 0 (Individual Intercept)	0.689	[0.547, 0.818]	[0.144, 1.049]

```
# save_kable(tarsusGrowthModelTable,
# "../tables/tarsusGrowthModelTable.html")
```

Effects of rearing condition on growth curve parameters Our coefficients indicate that warm-reared individuals (i.e. those raised at 30°C for at least their first three weeks of life) had a higher asymptote (value for a) than both those who were reared in mild conditions (20°C) and cold conditions (10°C for at least 3 weeks of life). Similarly, the growth rate of warm-reared individuals appeared higher (or more rapid) than those from our other experimental treatments. Evidence ratios for these differences were next calculated formally, again using the Savage-Dickey density ratio method.

```
caption <- paste0(
  "Pairwise comparisons of Gompertz function ",
  "variables between cold-reared (10°C until at least 3 ",
  "weeks of age), mild-reared (constant 20°C), ",
  "and warm-reared (30°C until at least 3 weeks of age) ",
  "Japanese quail. Gompertz function variables being ",
  "compared are indicated in parenthesis, per hypothesis."
)

hypotheses <- c(
  paste0(
    c("A", "B", "C"), "_Intercept - ",
```

```
    "(", c("A", "B"), "_Intercept + ", c("A", "B", "C"),
    "_pretreatmentcold) > 0"
  ),
  paste0(
    "(", c("A", "B", "C"), "_Intercept + ", c("A", "B", "C"),
    "_pretreatmentwarm) - ", c("A", "B", "C"), "_Intercept > 0"
  ),
  paste0(
    "(", c("A", "B", "C"), "_Intercept + ", c("A", "B", "C"),
    "_pretreatmentwarm) - (", c("A", "B", "C"), "_Intercept + ",
    c("A", "B", "C"), "_pretreatmentcold) > 0"
  )
)

layHypotheses <- c(
  "Cold-reared Asymptote (a) < Mild-reared Asymptote (a)",
  "Cold-reared Displacement (b) < Mild-reared Displacement (b)",
  "Cold-reared Growth Rate (c) < Mild-reared Growth Rate (c)",
  "Warm-reared Asymptote (a) > Mild-reared Asymptote (a)",
  "Warm-reared Displacement (b) > Mild-reared Displacement (b)",
  "Warm-reared Growth Rate (c) > Mild-reared Growth Rate (c)",
  "Cold-reared Asymptote (a) < Warm-reared Asymptote (a)",
  "Cold-reared Displacement (b) < Warm-reared Displacement (b)",
  "Cold-reared Growth Rate (a) < Warm-reared Growth Rate (c)"
)

pairwiseTableGrowthTarsus <- left_join(
  bind_rows(lapply(hypotheses, FUN = function(x) {
    hypothesis(growthModelTarsus, hypothesis = x, class = "b", robust = TRUE)$hypothesis
  })) %>% mutate(`95\\% CIs` = paste0(
      "[",
      round(CI.Lower, digits = 3),
      ", ",
      round(CI.Upper, digits = 3),
      "]"
    )) %>%
    dplyr::select(
      Hypothesis, "Delta" = Estimate, `95\\% CIs`, "Evidence Ratio" = Evid.Ratio
      ),
  bind_rows(lapply(hypotheses, FUN = function(x) {
    hypothesis(growthModelTarsus,
      hypothesis = x, class = "b",
      robust = TRUE, alpha = 0.2
    )$hypothesis
  })) %>% mutate(`50\\% CIs` = paste0(
      "[",
      round(CI.Lower, digits = 3),
      ", ",
      round(CI.Upper, digits = 3),
      "]"
    )) %>%
    dplyr::select(Hypothesis, `50\\% CIs`),
  by = "Hypothesis"
) %>%
  mutate("hypothesis" = layHypotheses) %>%
  dplyr::select(
    "Hypothesis" = hypothesis, Delta, `50\\% CIs`, `95\\% CIs`,
    `Evidence Ratio`
  ) %>%
  kbl(., longtable = T, booktabs = T, format = "latex",
      caption = caption, escape = FALSE
  ) %>%
  column_spec(column = c(1:10), width = "2.5cm") %>%
  kable_styling(latex_options = "striped")

pairwiseTableGrowthTarsus
```

Table 19: *Pairwise comparisons of Gompertz function variables between cold-reared (10°C until at least 3 weeks of age), mild-reared (constant 20°C), and warm-reared (30°C until at least 3 weeks of age) Japanese quail. Gompertz function variables being compared are indicated in parenthesis, per hypothesis.*

Hypothesis	Delta	50% CIs	95% CIs	Evidence Ratio
Cold-reared Asymptote (a) < Mild-reared Asymptote (a)	-0.3843930	[-0.893, 0.128]	[-1.376, 0.618]	0.3634427
Cold-reared Displacement (b) < Mild-reared Displacement (b)	0.0071874	[-0.053, 0.063]	[-0.118, 0.118]	1.1795396
Cold-reared Growth Rate (c) < Mild-reared Growth Rate (c)	-33.8891139	[-35.197, -32.616]	[-36.418, -31.408]	0.0000000
Warm-reared Asymptote (a) > Mild-reared Asymptote (a)	1.0490950	[0.543, 1.54]	[0.072, 2.02]	25.8907563
Warm-reared Displacement (b) > Mild-reared Displacement (b)	0.0648697	[0.016, 0.117]	[-0.031, 0.171]	6.4074074
Warm-reared Growth Rate (c) > Mild-reared Growth Rate (c)	0.0211483	[-0.03, 0.073]	[-0.08, 0.123]	1.7220143
Cold-reared Asymptote (a) < Warm-reared Asymptote (a)	0.6613920	[0.205, 1.125]	[-0.247, 1.569]	7.9535534
Cold-reared Displacement (b) < Warm-reared Displacement (b)	0.0710895	[0.031, 0.112]	[-0.008, 0.154]	13.6520147
Cold-reared Growth Rate (a) < Warm-reared Growth Rate (c)	0.0883746	[0.052, 0.125]	[0.019, 0.16]	50.7799353

```
#save_kable(pairwiseTarsusGrowthTable, "../tables/pairwiseTarsusGrowthTable.html")
```

Whether tarsus lengths at maturity (8 weeks of age; the end of our growth curves) differed among rearing treatments was next analysed using a Bayesian one-way ANOVA. Here, priors on tarsus length (mm) for cold-, mild-, and warm-reared birds were normally-distributed with means of 37.1, 37.5, and 37.9 respectively (following findings of Burness et al, 2013), and standard deviations of 2. Our prior for batch effects (included as a group-level intercept) was again exponential with a lambda of 2.5, and that for our error term was half-student t distributed with three degrees of freedom and location and scale parameters of 0 and 2.5 respectively.

```
growthAnovaTarsus <- brm(
  data = data %>%
    filter(week == 8) %>%
    dplyr::select(pretreatment, mass, "tarsus" = tarsusLengthMean,
           "batch" = exp) %>%
    drop_na() %>%
    mutate(pretreatment = factor(pretreatment, levels = c("neutral", "cold", "warm"))),
  formula = tarsus ~ 0 + pretreatment + (1|batch),
  prior = c(
    set_prior("normal(37.5, 2)", class = "b",
```

```
              coef = "pretreatmentneutral"),
    set_prior("normal(37.1, 2)", class = "b",
              coef = "pretreatmentcold"),
    set_prior("normal(37.9, 2)", class = "b",
              coef = "pretreatmentwarm"),
    set_prior("exponential(2.5)", class = "sd",
              group = "batch")
  ),
  family = "gaussian",
  seed = 200,
  cores = 4, chains = 4,
  iter = 50000, warmup = 10000, thin = 10,
  control = list(adapt_delta = 0.95, max_treedepth = 13),
  silent = TRUE, refresh = 0,
  file = "./models/_tarsusAtMaturityANOVA.Rds"
)

hypotheses <- c(
  "pretreatmentcold - pretreatmentneutral > 0",
  "pretreatmentwarm - pretreatmentneutral > 0",
  "pretreatmentwarm - pretreatmentcold > 0"
)

caption <- paste0("Results from a Bayesian, one-way ANOVA ",
                  "comparing tarsus length (mm) at 8 weeks of Japanese ",
                  "quail reared in the cold (10°C until at least ",
                  "3 weeks of age; n = ",
                  nrow(subset(growthAnovaTarsus$data, pretreatment == "cold")),
                  "), mild temperature (constant 20°C; n = ",
                  nrow(subset(growthAnovaTarsus$data, pretreatment == "neutral")),
                  "), or warmth (30°C until at least 3 weeks of age; n = ",
                  nrow(subset(growthAnovaTarsus$data, pretreatment == "warm")),
                  ")."
                  )

tarsusGrowthAnovaTable <- bind_rows(
  lapply(hypotheses, FUN = function(x) {
    hold <- hypothesis(growthAnovaTarsus,
                       hypothesis = x,
                       class = "b",
                       alpha = 0.5,
                       robust = TRUE)
    return(data.frame(
      "hyp" = x,
      "deltaMass" = hold$hypothesis$Estimate,
      "pProb" = hold$hypothesis$Post.Prob
    ))
  })
) %>%
  mutate("Hypothesis" = c(
    "Cold-Reared Tarsus Length > Mild-Reared Tarsus Length",
    "Warm-Reared Tarsus Length > Mild-Reared Tarsus Length",
    "Cold-Reared Tarsus Length < Warm-Reared Tarsus Length"
  )) %>%
  mutate(deltaMass = round(deltaMass, digits = 3),
         pProb = round(pProb, digits = 3)) %>%
  dplyr::select(Hypothesis, "Delta Tarsus Length (mm)" = deltaMass,
         "Posterior Probability" = pProb) %>%
  kbl(.,
    longtable = T, booktabs = T, format = "latex",
    caption = caption
  ) %>%
  column_spec(column = c(1:10), width = "2.5cm")  %>%
  kable_styling(latex_options = "striped")

tarsusGrowthAnovaTable
```

Table 20: *Results from a Bayesian, one-way ANOVA comparing tarsus length (mm) at 8 weeks of Japanese quail reared in the cold (10°C until at least 3 weeks of age; n = 40), mild temperature (constant 20°C; n = 33), or warmth (30°C until at least 3 weeks of age; n = 39).*

Hypothesis	Delta Tarsus Length (mm)	Posterior Probability
Cold-Reared Tarsus Length > Mild-Reared Tarsus Length	0.562	0.826
Warm-Reared Tarsus Length > Mild-Reared Tarsus Length	1.181	0.979
Cold-Reared Tarsus Length < Warm-Reared Tarsus Length	0.621	0.885

```
#save_kable(tarsusGrowthAnovaTable, "../tables/tarsusGrowthAnovaTable.html")
```

Similar to our analyses regarding mass gain during growth, we again tested whether continuous exposure to cold (10°C) or warm (30°C) conditions until maturity (8 weeks of age) differentially shifted appendage elongation trajectories relative to more transient exposure (at least 3 weeks). This is acheived by repeating our above analysis but while only including individuals exposed to their given thermal treatment from hatch until 8 weeks of age. All priors remain the same as above.

```
# Executing model

growthModelTarsusStrict <- brm(
  data = data %>%
    rename("batch" = exp) %>%
    filter(week <= 8 & batch == "C") %>%
    mutate(pretreatment = factor(pretreatment,
                                 levels = c("cold", "warm")
    )) %>%
    dplyr::select(ring, pretreatment, week, mass,
           "tarsus" = tarsusLengthMean) %>%
    distinct() %>%
    mutate(
      weekB = week + 1
    ) %>%
    group_by(week) %>%
    mutate(cScaledMass = (mass - mean(mass, na.rm = T)) /
             sd(mass, na.rm = T)) %>%
    ungroup(),
  formula = bf(tarsus ~ A * exp(-B * exp(-C * week)) + D,
               A ~ 1 + pretreatment,
               B ~ 1 + pretreatment,
               C ~ 1 + pretreatment,
               D ~ 1 + cScaledMass + (1 | ring),
               sigma ~ 0 + Intercept + log(weekB),
               nl = TRUE
  ),
  prior = c(
    set_prior("normal(37.1, 2.5)",
              class = "b", coef = "Intercept",
              nlpar = "A"
    ),
    set_prior("normal(0.8, 2)",
              class = "b", coef = "pretreatmentwarm",
              nlpar = "A"
    ),
```

```
    set_prior("skew_normal(0.6, 0.5, 2.5)",
              class = "b", coef = "Intercept",
              nlpar = "B"
    ),
    set_prior("normal(0, 0.5)",
              class = "b", coef = "pretreatmentwarm",
              nlpar = "B"
    ),
    set_prior("skew_normal(0.5, 0.1, 2.5)",
              class = "b", coef = "Intercept",
              nlpar = "C"
    ),
    set_prior("normal(0, 0.25)",
              class = "b", coef = "pretreatmentwarm",
              nlpar = "C"
    ),
    set_prior("normal(0, 1.5)", class = "b",
              coef = "Intercept", nlpar = "D"),
    set_prior("skew_normal(0.5, 0.15, 5)", class = "b",
              coef = "cScaledMass", nlpar = "D"),
    set_prior("exponential(1.5)", class = "sd",
              coef = "Intercept", group = "ring", nlpar = "D"),
    set_prior("skew_normal(0, 0.25, 10)", class = "b",
              coef = "Intercept", dpar = "sigma"),
    set_prior("skew_normal(0, 0.25, 10)", class = "b",
              coef = "logweekB", dpar = "sigma")
  ),
  family = "gaussian",
  seed = 100,
  cores = 4, chains = 4, init = 0,
  iter = 50000, warmup = 10000, thin = 10,
  control = list(adapt_delta = 0.95, max_treedepth = 14),
  silent = TRUE, refresh = 0,
  file = "./models/_growthModelTarsusStrict.Rds"
  )

ggarrange(
  ggplot(data = data.frame("Rhat" = brms::rhat(growthModelTarsusStrict)),
         aes(x = Rhat)) +
    geom_density() +
    theme_classic() +
    xlab(
      TeX('$\\hat{R}$')
    ) +
    ylab("Density"),
  ggplot(data = data.frame("Neff" = brms::neff_ratio(growthModelTarsusStrict)),
         aes(x = Neff)) +
    geom_density() +
    theme_classic() +
    xlab(
      TeX('$N_{eff}/N~Ratio$')
    ) +
    ylab("Density")
)
```

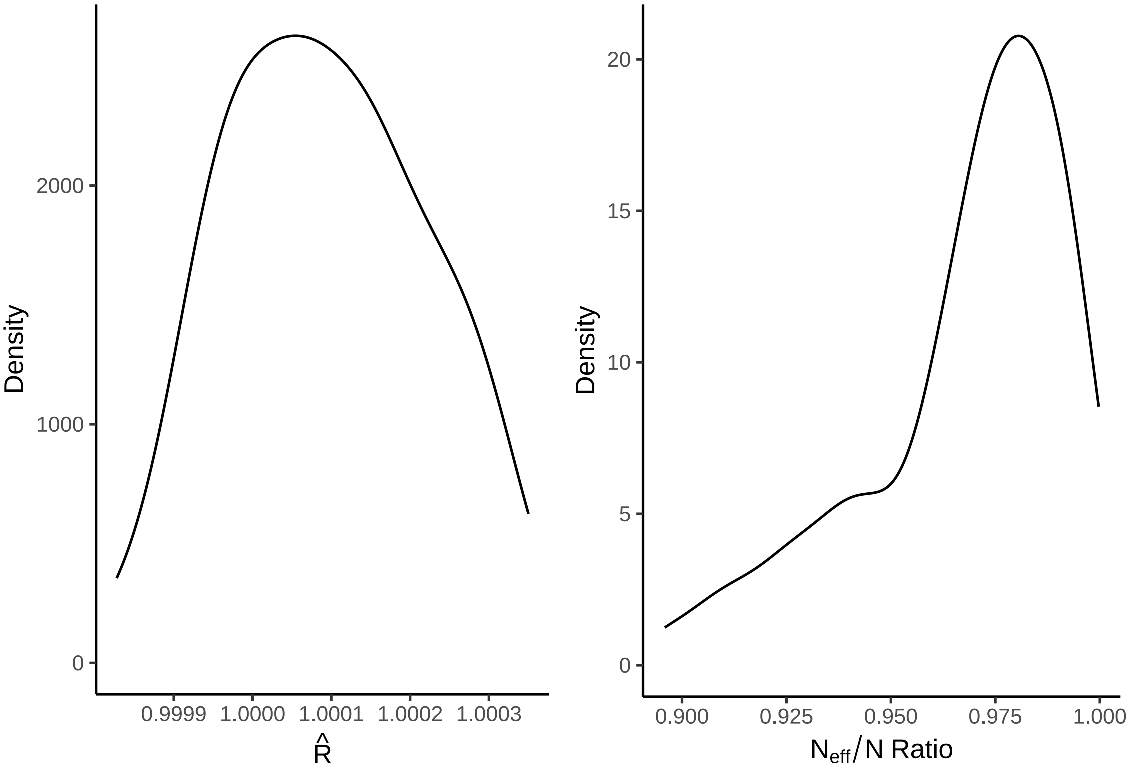

Figure 50: *Gelman-Rubin statistics ($\hat{R}$) and ratio of effective samples sizes by samples sizes per parameter from a Bayesian non-linear model estimating tarsus length (mm) among Japanese quail. Here, only quail exposed to their assigned rearing conditions until 8 weeks of age are included.*

```
# Clear chain convergence and little to no autocorrelation in chains
```

Residuals of this new model are visualised and evaluated.

```
p1 <- growthModelTarsusStrict$data %>%
  mutate("residuals" = residuals(growthModelTarsusStrict,
                                 method = "posterior_predict",
                                 type = "pearson",
                                 robust = TRUE)[, "Estimate"]) %>%
  ggplot(aes(x = residuals)) +
  geom_density(colour = "black", fill = "white") +
  xlab("Pearson Residuals") +
  ylab("Density") +
  theme_classic()

p2 <- growthModelTarsusStrict$data %>%
  mutate(
    "residuals" =
      residuals(growthModelTarsusStrict,
      method = "posterior_predict",
      type = "pearson",
      robust = TRUE
    )[, "Estimate"],
    "fitted" = fitted(growthModelTarsusStrict,
                      robust = TRUE)[, "Estimate"]
  ) %>%
  ggplot(aes(x = fitted, y = residuals)) +
  geom_point(colour = "black", pch = 21, size = 2,
             fill = "grey75", alpha = 0.5) +
  ylab("Pearson Residuals") +
```

```
  xlab("Fitted Values (mm)") +
  theme_classic()

p3 <- growthModelTarsusStrict$data %>%
  mutate("residuals" =
           residuals(growthModelTarsusStrict,
                     method = "posterior_predict",
                     type = "pearson",
                     robust = TRUE
  )[, "Estimate"]) %>%
  ggplot(aes(x = week, y = residuals)) +
  geom_point(
    colour = "black", pch = 21, size = 2, fill = "grey75", alpha = 0.5,
    position = position_jitter(width = 0.25)
  ) +
  stat_summary(geom = "errorbar", fun.data = "mean_se",
               colour = "black", width = 0.25) +
  stat_summary(geom = "point", fun = "mean", pch = 21,
               colour = "black", fill = "white", size = 4) +
  ylab("Pearson Residuals") +
  xlab("Age (weeks)") +
  theme_classic()

p4 <- growthModelTarsusStrict$data %>%
  mutate("residuals" =
           residuals(growthModelTarsusStrict,
                     method = "posterior_predict",
                     type = "pearson",
                     robust = TRUE
  )[, "Estimate"]) %>%
  ggplot(aes(x = cScaledMass, y = residuals)) +
  geom_point(
    colour = "black", pch = 21, size = 2, fill = "grey75", alpha = 0.5,
    position = position_jitter(width = 0.25)
  ) +
  stat_summary(geom = "errorbar", fun.data = "mean_se",
               colour = "black", width = 0.25) +
  stat_summary(geom = "point", fun = "mean", pch = 21,
               colour = "black", fill = "white", size = 4) +
  ylab("Pearson Residuals") +
  xlab("Centred and Scaled Body Mass") +
  theme_classic()

p5 <- growthModelTarsusStrict$data %>%
  mutate("residuals" =
           residuals(growthModelTarsusStrict,
                     method = "posterior_predict",
                     type = "pearson",
                     robust = TRUE
  )[, "Estimate"]) %>%
  mutate(pretreatment = str_to_title(pretreatment)) %>%
  mutate(pretreatment = ifelse(pretreatment == "Cold",
                               "Cold\n(10°C)",
    ifelse(pretreatment == "Neutral", "Mild\n(20°C)",
      "Warm\n(30°C)"
    )
  )) %>%
  mutate(pretreatment = factor(pretreatment,
                               levels = c("Cold\n(10°C)",
                                          "Mild\n(20°C)",
                                          "Warm\n(30°C)"))) %>%
  ggplot(aes(x = pretreatment, y = residuals)) +
  geom_point(
    colour = "black", pch = 21, size = 2, fill = "grey75", alpha = 0.5,
    position = position_jitter(width = 0.25)
  ) +
  stat_summary(geom = "errorbar", fun.data = "mean_se",
```

```
               colour = "black", width = 0.25) +
  stat_summary(geom = "point", fun = "mean", pch = 21,
               colour = "black", fill = "white", size = 4) +
  ylab("Pearson Residuals") +
  xlab("Rearing Conditions)") +
  theme_classic()

((p1 + p2) / (p3 + p4)) + p5
```

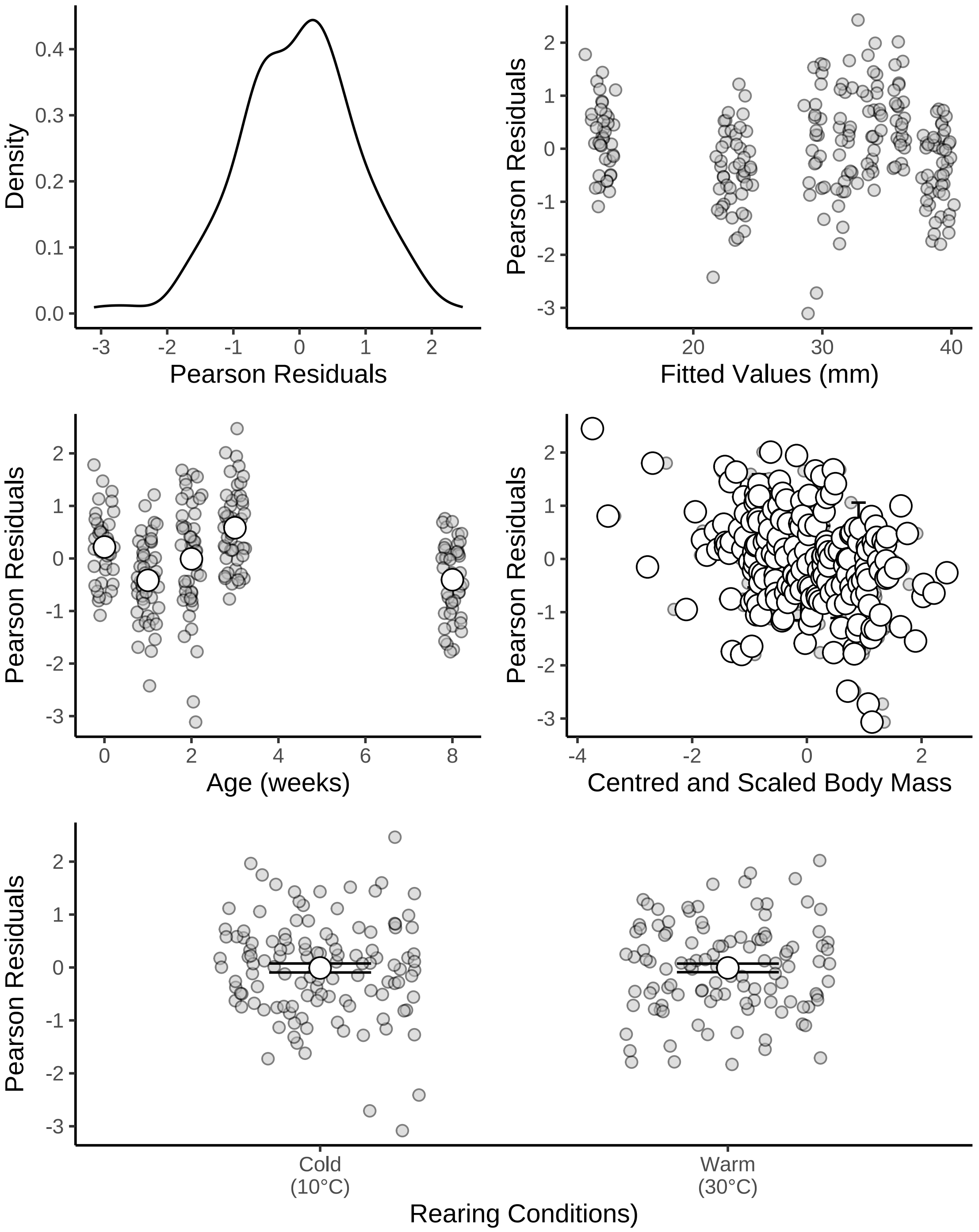

Figure 51: *Density and distributions of Pearson residuals across fitted values and model predictors (including age and rearing conditions). All residuals pertain to those drawn from a Bayesian non-linear model predicting tarsus length (mm) during growth in Japanese quail. Pearson residuals are shown rather than ordinary residuals to correct for the age-dependence of model error. Here, only quail exposed to their assigned rearing conditions until 8 weeks of age were included in our model.*

```
# Again, homoskedastic.
# Summarising sample sizes

caption <- paste0("Number of tarsus length measurements (samples) ",
```

```
                  "drawn from Japanese quail between 0 and ",
                  "8 weeks of age (maturity) across three distict ",
                  "thermal rearing conditions.")

growthModelTarsusStrict$data %>%
  group_by(week, pretreatment) %>%
  dplyr::count(name = "Samples (n)") %>%
  mutate(pretreatment = ifelse(pretreatment == "cold",
                               "Cold (10°C)",
                               ifelse(pretreatment == "neutral",
                                      "Mild (20°C)", "Warm (30°C)")
                               )
         ) %>%
  arrange(pretreatment, week) %>%
  rename("Age (Weeks)" = week,
         "Rearing Conditions" = pretreatment) %>%
  kbl(.,
    longtable = T, booktabs = T,
    caption = caption
  ) %>%
  kable_styling(latex_options = "striped")
```

Table 21: *Number of tarsus length measurements (samples) drawn from Japanese quail between 0 and 8 weeks of age (maturity) across three distict thermal rearing conditions.*

Age (Weeks)	Rearing Conditions	Samples (n)
0	Cold (10°C)	23
1	Cold (10°C)	23
2	Cold (10°C)	24
3	Cold (10°C)	25
8	Cold (10°C)	24
0	Warm (30°C)	16
1	Warm (30°C)	24
2	Warm (30°C)	24
3	Warm (30°C)	24
8	Warm (30°C)	23

Our tarsus elongation curves are replotted.

```
growthCurveTarsusStrict <- with(
  growthModelTarsusStrict$data,
  expand.grid(
    "week" = seq(0, 8, by = 0.1),
    "pretreatment" = c("cold", "warm"),
    "cScaledMass" = 0
  )
) %>%
  mutate("weekB" = week + 1) %>%
  mutate(
    "Fit" = predict(growthModelTarsusStrict, re_form = NA,
                    newdata = .,
                    robust = TRUE)[, "Estimate"],
    "SE" = predict(growthModelTarsusStrict, re_form = NA,
                   newdata = .,
                   robust = TRUE)[, "Est.Error"]
  ) %>%
  mutate(
    "LCL" = Fit - SE,
    "UCL" = Fit + SE
  ) %>%
  mutate(pretreatment = ifelse(pretreatment == "cold",
                               "Cold (10°C)",
                               "Warm (30°C)")
```

```
         ) %>%
  mutate(pretreatment = factor(pretreatment,
                               levels =
                                 c("Cold (10°C)",
                                  "Warm (30°C)")
  )
  ) %>%
  ggplot(aes(x = week, y = Fit,
   fill = pretreatment, linetype = pretreatment)) +
  geom_ribbon(aes(x = week, ymin = LCL, ymax = UCL),
    colour = NA, size = 0.25, alpha = 0.3
  ) +
  geom_line(colour = "black", alpha = 0.7) +
  stat_summary(
    data = growthModelTarsusStrict$data %>%
    mutate(pretreatment = ifelse(pretreatment == "cold",
                               "Cold (10°C)",
                               "Warm (30°C)")
           ) %>%
    mutate(pretreatment = factor(pretreatment,
                               levels =
                                 c("Cold (10°C)",
                                  "Warm (30°C)")
  )
  ),
    aes(x = week, y = tarsus),
    geom = "errorbar", fun.data = "mean_cl_boot",
    colour = "black", alpha = 0.7, width = 0.25,
    position = position_dodge(width = 0.15)
  ) +
  stat_summary(
    data = growthModelTarsusStrict$data %>%
    mutate(pretreatment = ifelse(pretreatment == "cold",
                               "Cold (10°C)",
                               "Warm (30°C)")
           ) %>%
    mutate(pretreatment = factor(pretreatment,
                               levels =
                                 c("Cold (10°C)",
                                  "Warm (30°C)")
  )
  ),
    aes(x = week, y = tarsus),
    geom = "point", fun = "mean", pch = 21, size = 3,
    colour = "black", alpha = 0.7,
    position = position_dodge(width = 0.15)
  ) +
  theme_classic() +
  scale_fill_manual(values = c("#7BB4E3", "#CD5C5C"),
                    name = "Rearing\nConditions") +
  scale_linetype_manual(values = c("dotted", "solid", "dashed"),
                        name = "Rearing\nConditions") +
  xlab("Age (weeks)") +
  ylab("Tarsus Length (mm)")

showtext_auto()
growthCurveTarsusStrict
```

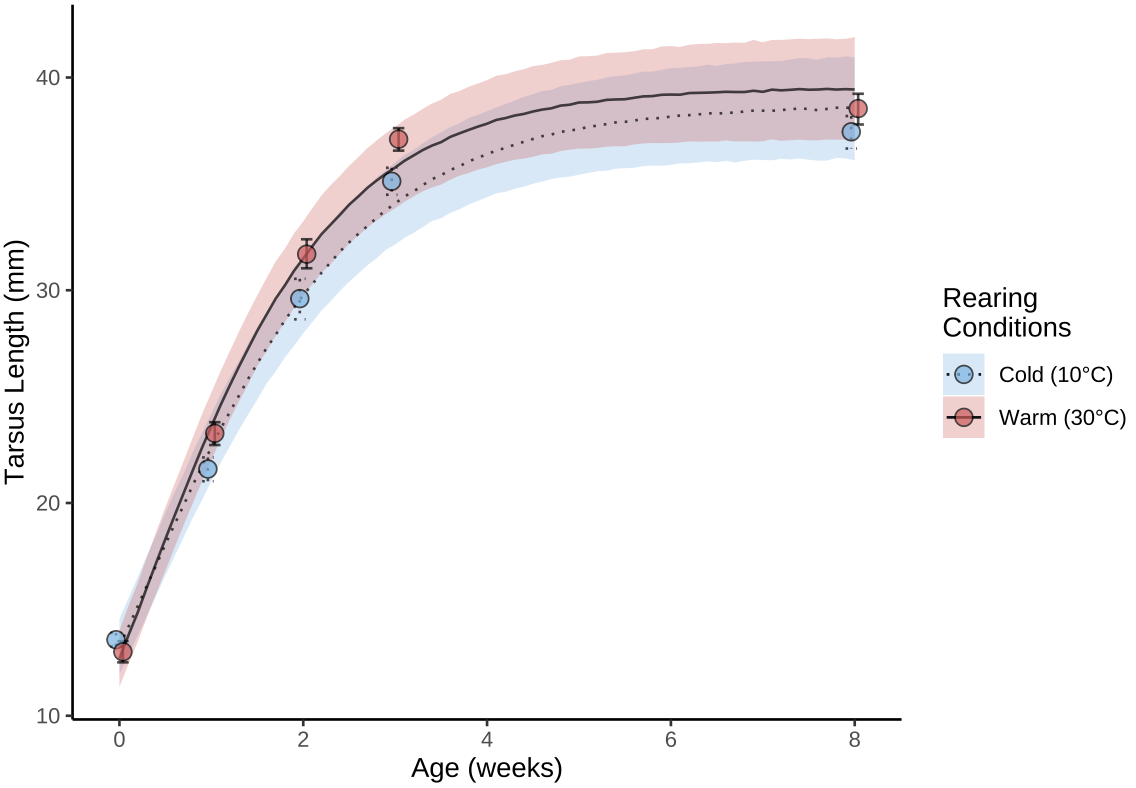

Figure 52: *Tarsus length (mm) elongation curves of Japanese quail reared in the cold (10°C), mild conditions (20°C), or the warm (30°C) until at least 8 weeks of age. Dots represent mean values and errorbars represent quantile-based 95% credible intervals. Lines represent estimated trends in growth from a Bayesian non-linear model, while ribbons represent confidence around trends (± one standard error).*

```
ggsave("./plots/growthCurveTarsusStrict.jpg", dpi = 800,
       width = 7, height = 6.5,
       growthCurveTarsusStrict)
showtext_auto(enable = "FALSE")

# Summarising growth curve parameters

caption <- paste0('Coefficients from a Bayesian non-linear effects ',
                  "model predicting tarsus length (mm) of Japanese quail as a ",
                  "Gompertz function of age in weeks. Coefficients represent ",
                  "posterior medians and are estimated from body mass data ",
                  "collected weekly between 0 and 8 weeks of age. Credible ",
                  "intervals (CIs) represent quantile ",
                  'intervals around medians Here, only individuals ',
                  'who experienced continuous exposure to thermal treatment ',
                  'until maturity (8 weeks of age) were included in our ',
                  'analysis.'
)

tarsusGrowthModelStrictTable <-
  merge(
    as.data.frame(growthModelTarsusStrict) %>%
      summarise_all(., .funs = median) %>%
      mutate_all(., .funs = round, 3) %>%
      pivot_longer(everything(), names_to = "Parameter",
                   values_to = "Values") %>%
      filter(grepl("b_|sd_", Parameter)) %>%
      arrange(Parameter),
    quantileCIs(growthModelTarsusStrict, cis = c(50, 95)),
```

```
    by = "Parameter"
  ) %>%
    mutate(`50\\% CI` = paste0("[",
                               round(Low_CI_50, digits = 3),
                               ", ",
                               round(High_CI_50, digits = 3),
                               "]"),
           `95\\% CI` = paste0("[",
                               round(Low_CI_95, digits = 3),
                               ", ",
                               round(High_CI_95, digits = 3),
                               "]"),
           ) %>%
    dplyr::select(Parameter, Values, `50\\% CI`, `95\\% CI`) %>%
  merge(., tribble(
    ~Parameter, ~Par,
    "b_A_Intercept", "Beta a0",
    "b_A_pretreatmentwarm", "Beta a2 (Warm-reared)",
    "b_B_Intercept", "Beta b0",
    "b_B_pretreatmentwarm", "Beta b2 (Warm-reared)",
    "b_C_Intercept", "Beta c0",
    "b_C_pretreatmentwarm", "Beta c2 (Warm-reared)",
    "b_D_Intercept", "Beta 0",
    "b_D_cScaledMass", "Beta 1",
    "sd_ring__D_Intercept", "Mu 0 (Individual Intercept)",
    "b_sigma_Intercept", "Tau 0",
    "b_sigma_logweekB", "Tau 1"
  ),
  by = "Parameter", all.x = TRUE
  ) %>%
  dplyr::select(-Parameter) %>%
  dplyr::select(
    "Parameter" = Par, "Value" = Values, `50\\% CI`, `95\\% CI`
  ) %>%
  kbl(., longtable = T, booktabs = T, format = "latex",
      escape = FALSE, caption = caption) %>%
  column_spec(column = c(1:10), width = "2.5cm")  %>%
  kable_styling(latex_options = "striped")

tarsusGrowthModelStrictTable
```

Table 22: *Coefficients from a Bayesian non-linear effects model predicting tarsus length (mm) of Japanese quail as a Gompertz function of age in weeks. Coefficients represent posterior medians and are estimated from body mass data collected weekly between 0 and 8 weeks of age. Credible intervals (CIs) represent quantile intervals around medians Here, only individuals who experienced continuous exposure to thermal treatment until maturity (8 weeks of age) were included in our analysis.*

Parameter	Value	50% CI	95% CI
Beta a0	36.225	[35.269, 37.174]	[33.44, 39.041]
Beta a2 (Warm-reared)	0.799	[0.385, 1.204]	[-0.423, 2.013]
Beta b0	1.218	[1.159, 1.281]	[1.059, 1.426]
Beta b2 (Warm-reared)	0.074	[0.045, 0.104]	[-0.008, 0.166]
Beta c0	0.729	[0.708, 0.749]	[0.671, 0.791]
Beta c2 (Warm-reared)	0.106	[0.08, 0.132]	[0.031, 0.183]
Beta 1	0.486	[0.463, 0.511]	[0.423, 0.565]
Beta 0	2.508	[1.595, 3.406]	[-0.207, 5.169]
Tau 0	0.254	[0.184, 0.325]	[0.062, 0.458]
Tau 1	0.277	[0.223, 0.333]	[0.122, 0.43]
Mu 0 (Individual Intercept)	0.689	[0.565, 0.808]	[0.252, 1.04]

Bill length

In this subsection, we estimate the effect of the post-hatch thermal environment on bill length (in mm) of our captive-reared Japanese quail. Our approach to do so reflects that described in the above subsection, with bill length modeled as a Gompertz function of age, adjusted for age-dependent body mass, and rearing treatment predicting each Gompertz parameter. Formulae for this model were therefore as follows:

$$Bill\ Length_{ij} \sim a \cdot e^{-b \cdot e^{-c \cdot Age_{ij}}} + \beta_1 \cdot Scaled\ Mass + \mu_{0j} + \epsilon_{ij}$$
$$a_{ij} \sim \beta_{a0} + \beta_{a1} \cdot Cold\ Reared_j + \beta_{a2} \cdot Warm\ Reared_j + \mu_{0aj}$$
$$b_{ij} \sim \beta_{b0} + \beta_{b1} \cdot Cold\ Reared_j + \beta_{b2} \cdot Warm\ Reared_j + \mu_{0bj}$$
$$c_{ij} \sim \beta_{c0} + \beta_{c1} \cdot Cold\ Reared_j + \beta_{c2} \cdot Warm\ Reared_j + \mu_{0cj}$$

with all variables remaining as described previously.

Here again, priors for our model parameters were informed by Burness et al (2013) and Persson et al (2024). More specifically, our prior for our growth curve asymptote (β_{a0}) was normal with a mean of 15.7 mm (the mean bill length of all Japanese quail at 123 days, as reported in raw data of Burness et al, 2013) and a standard deviation of 2.5, while that for our x-axis displacement (β_{b0}) was skew-normal distributed with a ξ value of 1.7, ω of 0.5 and α of 2.5 (assuming a y-intercept of our minimum bill length and asymptote described above). For our growth rate parameter (β_{c0}), a skew-normal prior was used with parameterisation equaling that used for our prior of tarsus length growth rate ($\xi = 0.5$, $\omega = 0.1$, and $\alpha = 2.5$). Priors for the effect of rearing condition on growth parameters a, b, and c (i.e. β_{a1-2}, β_{b1-2}, and β_{c1-2} respectively) were each normally-distributed, with those predicting a being moderately informed by raw data of Burness et al (2013; means for cold-rearing and warm-rearing respectively being -0.22 and 0.22 [representing the cumulative mean difference in mean bill length between warm and cold-reared quail as adults in their study] and standard deviations of 1) and those predicting b and c being conservatively set with means of 0 and standard deviations of 0.5 and 0.25 respectively. For our group-level effects of egg batch, we again used exponential priors with lambda values determined from preliminary plots (μ_{0a}: $\lambda = 5$; μ_{0b}: $\lambda = 10$; μ_{0c}: $\lambda = 25$). Similarly, for our group-level effect of bird identity, we also used an exponential prior, but with lambda set broadly to 2.5. Last, a skew-normal prior was used for the effect of scaled body mass on bill length, with ξ, ω, and α values of 0.25, 0.15 and 5 respectively. Skew-normal priors were used for our error structure parameters τ_0 and τ_1 with ξ values of 1 and 0.2 respectively, ω values of 0.5, and α values of 5 and -5 respectively (estimated from preliminary plots).

For this model, four Hamiltonian Monte Carlo (HMC) chains were used with each run for 50000 iterations and 10000 warm-up iterations, then sampled every 10 iterations. Suitability of priors is evaluated by a prior predictive check below.

```
ahat <- 15.7 # Estimated asymptote which here equals the mean exposed culmen
# length of quail at 123 days of age, as reported in the raw data of Burness
# et al (2013).

b0hat <- min(data$billLength, na.rm = T)

bAtY <- function(a, yint) {
  b <- -log(yint / a)
  return(b)
}

cat(paste0("Estimated b value = ",
           round(bAtY(ahat, b0hat), digits = 3)))
```

```
## Estimated b value = -Inf
```

```
bXi <- round(skewxi(round(bAtY(ahat, b0hat), digits = 3),
                    omega = 0.5, alpha = 2.5), digits = 3)
cat(paste("xi value for skew-normal distribution on b = ", bXi))
```

```
## xi value for skew-normal distribution on b =  -Inf
```

```
growthModelBill_ppCheck <- brm(
  data = data %>%
    filter(week <= 8) %>%
    mutate(pretreatment = factor(pretreatment,
      levels = c("neutral", "cold", "warm")
    )) %>%
    dplyr::select(
      ring, pretreatment, week, mass, billLengthMean, "batch" = exp
    ) %>%
    distinct() %>%
    mutate(
      weekB = week + 1
    ) %>%
    group_by(week) %>%
    mutate(cScaledMass = (mass - mean(mass, na.rm = T)) /
             sd(mass, na.rm = T)) %>%
    ungroup(),
  formula = bf(billLengthMean ~ A * exp(-B * exp(-C * week)) + D,
    A ~ 1 + pretreatment + (1|batch),
    B ~ 1 + pretreatment + (1|batch),
    C ~ 1 + pretreatment + (1|batch),
    D ~ 1 + cScaledMass + (1 | ring),
    sigma ~ 0 + Intercept + log(weekB),
    nl = TRUE
  ),
  prior = c(
    set_prior(paste0("normal(", ahat,", 2.5)"),
              class = "b", coef = "Intercept",
              nlpar = "A"
    ),
    set_prior("normal(-0.22, 1)",
              class = "b", coef = "pretreatmentcold",
              nlpar = "A"
    ),
    set_prior("normal(0.22, 1)",
              class = "b", coef = "pretreatmentwarm",
              nlpar = "A"
    ),
    set_prior("exponential(5)", class = "sd",
              coef = "Intercept",
              group = "batch", nlpar = "A"
    ),
    set_prior("skew_normal(1.7, 0.5, 2.5)",
              class = "b", coef = "Intercept",
              nlpar = "B"
    ),
    set_prior("normal(0, 0.5)",
              class = "b", coef = "pretreatmentcold",
              nlpar = "B"
    ),
    set_prior("normal(0, 0.5)",
              class = "b", coef = "pretreatmentwarm",
              nlpar = "B"
    ),
    set_prior("exponential(10)", class = "sd",
              coef = "Intercept",
              group = "batch", nlpar = "B"
    ),
    set_prior("skew_normal(0.5, 0.1, 2.5)",
              class = "b", coef = "Intercept",
              nlpar = "C"
    ),
    set_prior("normal(0, 0.25)",
              class = "b", coef = "pretreatmentcold",
              nlpar = "C"
    ),
    set_prior("normal(0, 0.25)",
```

```
              class = "b", coef = "pretreatmentwarm",
              nlpar = "C"
    ),
    set_prior("exponential(25)", class = "sd",
              coef = "Intercept",
              group = "batch", nlpar = "C"
    ),
    set_prior("normal(0, 1.5)", class = "b",
              coef = "Intercept", nlpar = "D"),
    set_prior("skew_normal(0.5, 0.15, 5)", class = "b",
              coef = "cScaledMass", nlpar = "D"),
    set_prior("exponential(2.5)", class = "sd",
              coef = "Intercept", group = "ring", nlpar = "D"),
    set_prior("skew_normal(1, 0.25, 5)", class = "b",
              coef = "Intercept", dpar = "sigma"),
    set_prior("skew_normal(0.2, 0.25, 5)", class = "b",
              coef = "logweekB", dpar = "sigma")
  ),
  family = "gaussian",
  seed = 103,
  cores = 4, chains = 4,
  iter = 50000, warmup = 10000, thin = 10,
  control = list(adapt_delta = 0.95, max_treedepth = 13),
  silent = TRUE, refresh = 0,
  sample_prior = "only",
  file = "./models/_ppCheckBillLength.Rds"
)

# Assessing posterior predictions

pp_check2(growthModelBill_ppCheck, xlab = "Bill Length (mm)")
```

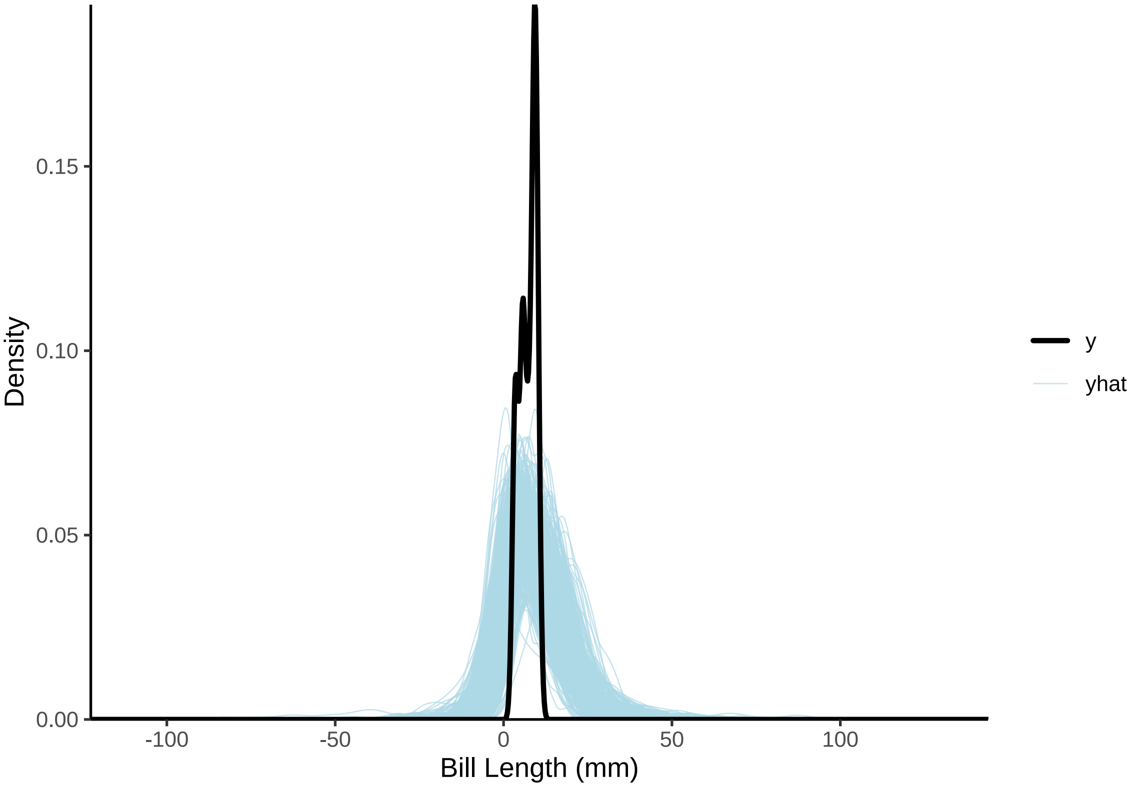

Figure 53: *Overlay of predicted (blue) and true (black) bill length densities, where predicted densities are derived from priors in a Bayesian non-linear model. Overlap betwen the black and blue lines indicates that model priors are reasonable with respect to the data.*

```
# Central values captured, but priors notable weak.
# Proceeding as is to avoid over-asserting prior effects.
```

We proceed with the selected priors and set initial parameter values to ease HMC chains initiation. Here, parameter values are selected from within our prior distributions.

```
initFunction <- function(chain_id = 1) {
  list(
    "b_A" = c(rnorm(1, 15.7, 2.5), -0.22, 0.22),
    "b_B" = c(1.7, 0, 0),
    "b_C" = c(0.5, 0, 0),
    "b_D" = c(rnorm(1, 0, 1.5),
              rskew_normal(1, xi = 0.2, omega = 0.15, alpha = 5)),
    "b_sigma" = rskew_normal(2, xi = 0, omega = 0.25, alpha = 5),
    "sd_1" = rexp(1, 5),
    "sd_2" = rexp(1, 10),
    "sd_3" = rexp(1, 25),
    "sd_4" = rexp(1, 2.5)
  )
}

# Loading initial values into a list.

initList <- list(initFunction(),
                 initFunction(),
                 initFunction(),
                 initFunction())

growthModelBill <- brm(
```

```
data = data %>%
  filter(week <= 8) %>%
  mutate(pretreatment = factor(pretreatment,
                               levels = c("neutral", "cold", "warm")
  )) %>%
  dplyr::select(ring, pretreatment, week, mass,
         billLengthMean, "batch" = exp) %>%
  distinct() %>%
  mutate(
    weekB = week + 1
  ) %>%
  group_by(week) %>%
  mutate(cScaledMass = (mass - mean(mass, na.rm = T)) /
           sd(mass, na.rm = T)) %>%
  ungroup(),
formula = bf(billLengthMean ~ A * exp(-B * exp(-C * week)) + D,
             A ~ 1 + pretreatment + (1|batch),
             B ~ 1 + pretreatment + (1|batch),
             C ~ 1 + pretreatment + (1|batch),
             D ~ 1 + cScaledMass + (1 | ring),
             sigma ~ 0 + Intercept + log(weekB),
             nl = TRUE
),
prior = c(
  set_prior(paste0("normal(", ahat,", 2.5)"),
            class = "b", coef = "Intercept",
            nlpar = "A"
  ),
  set_prior("normal(-0.22, 1)",
            class = "b", coef = "pretreatmentcold",
            nlpar = "A"
  ),
  set_prior("normal(0.22, 1)",
            class = "b", coef = "pretreatmentwarm",
            nlpar = "A"
  ),
  set_prior("exponential(5)", class = "sd",
            coef = "Intercept",
            group = "batch", nlpar = "A"
  ),
  set_prior("skew_normal(1.7, 0.5, 2.5)",
            class = "b", coef = "Intercept",
            nlpar = "B"
  ),
  set_prior("normal(0, 0.5)",
            class = "b", coef = "pretreatmentcold",
            nlpar = "B"
  ),
  set_prior("normal(0, 0.5)",
            class = "b", coef = "pretreatmentwarm",
            nlpar = "B"
  ),
  set_prior("exponential(10)", class = "sd",
            coef = "Intercept",
            group = "batch", nlpar = "B"
  ),
  set_prior("skew_normal(0.5, 0.1, 2.5)",
            class = "b", coef = "Intercept",
            nlpar = "C"
  ),
  set_prior("normal(0, 0.25)",
            class = "b", coef = "pretreatmentcold",
            nlpar = "C"
  ),
  set_prior("normal(0, 0.25)",
            class = "b", coef = "pretreatmentwarm",
            nlpar = "C"
```

```
    ),
    set_prior("exponential(25)", class = "sd",
              coef = "Intercept",
              group = "batch", nlpar = "C"
    ),
    set_prior("normal(0, 1.5)", class = "b",
              coef = "Intercept", nlpar = "D"),
    set_prior("skew_normal(0.5, 0.15, 5)", class = "b",
              coef = "cScaledMass", nlpar = "D"),
    set_prior("exponential(2.5)", class = "sd",
              coef = "Intercept", group = "ring", nlpar = "D"),
    set_prior("skew_normal(1, 0.25, 5)", class = "b",
              coef = "Intercept", dpar = "sigma"),
    set_prior("skew_normal(0.2, 0.25, 5)", class = "b",
              coef = "logweekB", dpar = "sigma")
  ),
  family = "gaussian",
  seed = 100,
  cores = 4, chains = 4,
  init = initList,
  backend = "cmdstan", threads = 2,
  iter = 50000, warmup = 10000, thin = 10,
  control = list(adapt_delta = 0.95, max_treedepth = 14),
  silent = TRUE, refresh = 0,
  file = "./models/_growthModelBillLength.Rds"
)

# Visualising Gelman-Rubin statistics and ratio of effective sample
# size to sample size ratios.

ggarrange(
  ggplot(data = data.frame("Rhat" = brms::rhat(growthModelBill)),
         aes(x = Rhat)) +
    geom_density() +
    theme_classic() +
    xlab(
      TeX('$\\hat{R}$')
    ) +
    ylab("Density"),
  ggplot(data = data.frame("Neff" = neffBase(growthModelBill)),
         aes(x = Neff)) +
    geom_density() +
    theme_classic() +
    xlab(
      TeX('$N_{eff}/N~Ratio$')
    ) +
    ylab("Density")
)
```

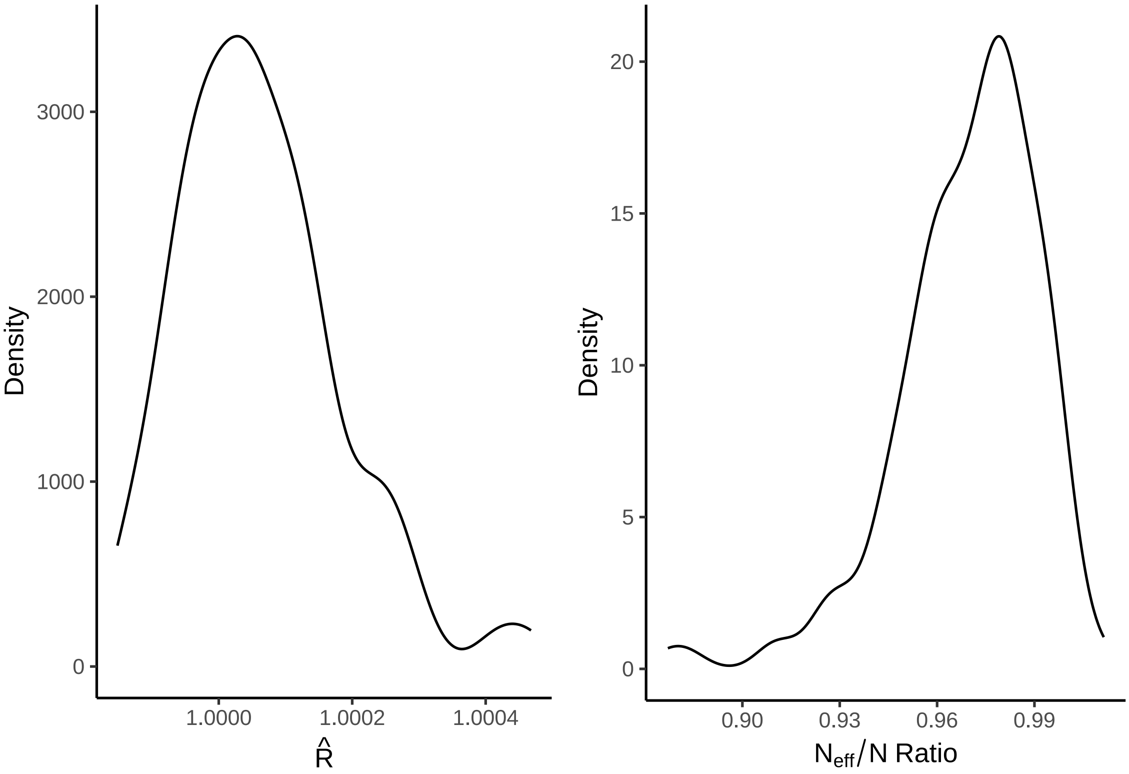

Figure 54: *Gelman-Rubin statistics ($\hat{R}$) and ratio of effective samples sizes by samples sizes per parameter from a Bayesian non-linear model estimating bill length (mm) among Japanese quail.*

Chains have converged and samples within chains appears independent. We proceed by visualising: (1) the density of posterior predictions against the true response variable density, and (2) a scatterplot of predicted bill length values against true bill length values.

```
pp_check2(growthModelBill, xlab = "Bill Length (mm)")
```

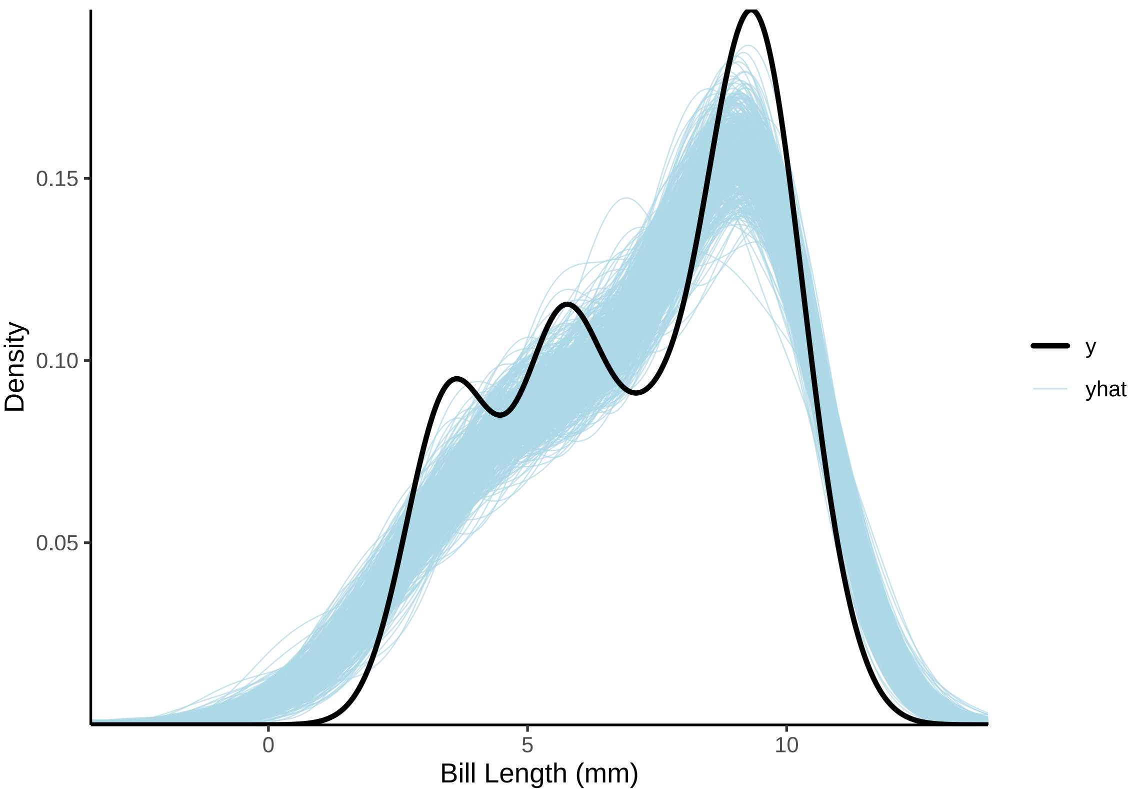

Figure 55: *Posterior predictions of a Bayesian non-linear model predicting Japanese bill length (mm) across time, overlayed with true distributions of quail bill length. Blue lines represent predictions from posterior draws, while the black line represents true bill length distributions.*

```
growthModelBill$data %>%
  mutate("Fit" = predict(growthModelBill,
                         robust = TRUE)[, "Estimate"]) %>%
  ggplot(aes(x = billLengthMean, y = Fit, fill = week)) +
  geom_point(pch = 21, colour = "black", size = 2, alpha = 0.5) +
  geom_smooth(method = "lm", colour = "black",
              linetype = "dashed", se = FALSE) +
  scale_fill_gradient2() +
  theme_classic() +
  xlab("Bill Length (mm)") +
  ylab("Predicted Bill Length (mm)")
```

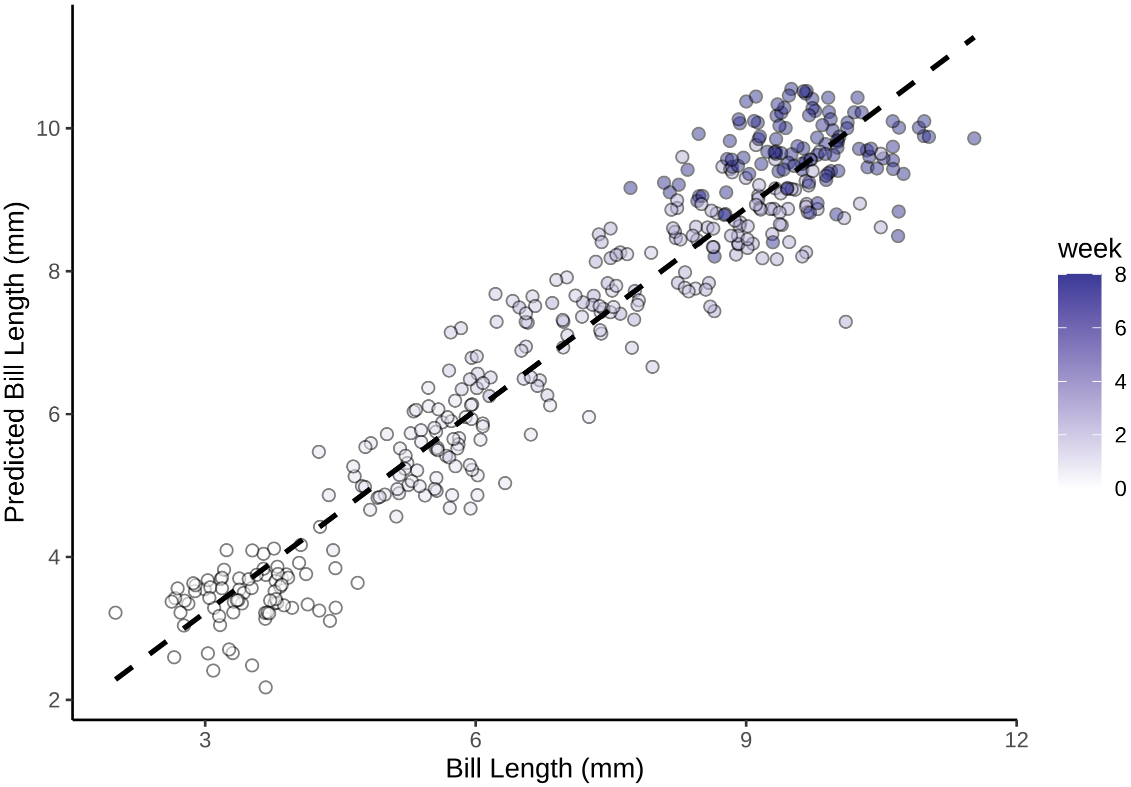

Figure 56: *Scatterplot of Japanese quail bill length (mm) against predicted bill length (mm) from a Bayesian non-linear model. Dashed line indicates line of best fit, as estimated by the R package ggplot2 (Wickham, 2011).*

Posterior predictions evidently capture variation in bill length measurements well. Spread of mean Pearson residuals is next visualised to check for oddities.

```
p1 <- growthModelBill$data %>%
  mutate("residuals" = residuals(growthModelBill,
                                 method = "posterior_predict",
                                 type = "pearson",
                                 robust = TRUE)[, "Estimate"]) %>%
  ggplot(aes(x = residuals)) +
  geom_density(colour = "black", fill = "white") +
  xlab("Pearson Residuals") +
  ylab("Density") +
  theme_classic()

p2 <- growthModelBill$data %>%
  mutate(
    "residuals" = residuals(growthModelBill,
      method = "posterior_predict",
      type = "pearson",
      robust = TRUE
    )[, "Estimate"],
    "fitted" = fitted(growthModelBill,
                                 robust = TRUE)[, "Estimate"]
  ) %>%
  ggplot(aes(x = fitted, y = residuals)) +
  geom_point(colour = "black", pch = 21, size = 2,
             fill = "grey75", alpha = 0.5) +
  ylab("Pearson Residuals") +
  xlab("Fitted Values (mm)") +
```

```
  theme_classic()

p3 <- growthModelBill$data %>%
  mutate("residuals" =
           residuals(growthModelBill,
                     method = "posterior_predict",
                     type = "pearson",
                     robust = TRUE
  )[, "Estimate"]) %>%
  ggplot(aes(x = week, y = residuals)) +
  geom_point(
    colour = "black", pch = 21, size = 2, fill = "grey75", alpha = 0.5,
    position = position_jitter(width = 0.25)
  ) +
  stat_summary(geom = "errorbar", fun.data = "mean_se",
               colour = "black", width = 0.25) +
  stat_summary(geom = "point", fun = "mean", pch = 21,
               colour = "black", fill = "white", size = 4) +
  ylab("Pearson Residuals") +
  xlab("Age (weeks)") +
  theme_classic()

p4 <- growthModelBill$data %>%
  mutate("residuals" =
           residuals(growthModelBill,
                     method = "posterior_predict",
                     type = "pearson",
                     robust = TRUE
  )[, "Estimate"]) %>%
  ggplot(aes(x = cScaledMass, y = residuals)) +
  geom_point(
    colour = "black", pch = 21, size = 2, fill = "grey75", alpha = 0.5,
    position = position_jitter(width = 0.25)
  ) +
  stat_summary(geom = "errorbar", fun.data = "mean_se",
               colour = "black", width = 0.25) +
  stat_summary(geom = "point", fun = "mean", pch = 21,
               colour = "black", fill = "white", size = 4) +
  ylab("Pearson Residuals") +
  xlab("Centred and Scaled Body Mass") +
  theme_classic()

p5 <- growthModelBill$data %>%
  mutate("residuals" =
           residuals(growthModelBill,
                     method = "posterior_predict",
                     type = "pearson",
                     robust = TRUE
  )[, "Estimate"]) %>%
  mutate(pretreatment = str_to_title(pretreatment)) %>%
  mutate(pretreatment = ifelse(pretreatment == "Cold",
                               "Cold\n(10°C)",
    ifelse(pretreatment == "Neutral", "Mild\n(20°C)",
      "Warm\n(30°C)"
    )
  )) %>%
  mutate(pretreatment = factor(pretreatment,
                               levels = c("Cold\n(10°C)",
                                          "Mild\n(20°C)",
                                          "Warm\n(30°C)"))) %>%
  ggplot(aes(x = pretreatment, y = residuals)) +
  geom_point(
    colour = "black", pch = 21, size = 2, fill = "grey75", alpha = 0.5,
    position = position_jitter(width = 0.25)
  ) +
  stat_summary(geom = "errorbar", fun.data = "mean_se",
               colour = "black", width = 0.25) +
```

```
  stat_summary(geom = "point", fun = "mean", pch = 21,
               colour = "black", fill = "white", size = 4) +
  ylab("Pearson Residuals") +
  xlab("Rearing Conditions)") +
  theme_classic()

((p1 + p2) / (p3 + p4)) + p5
```

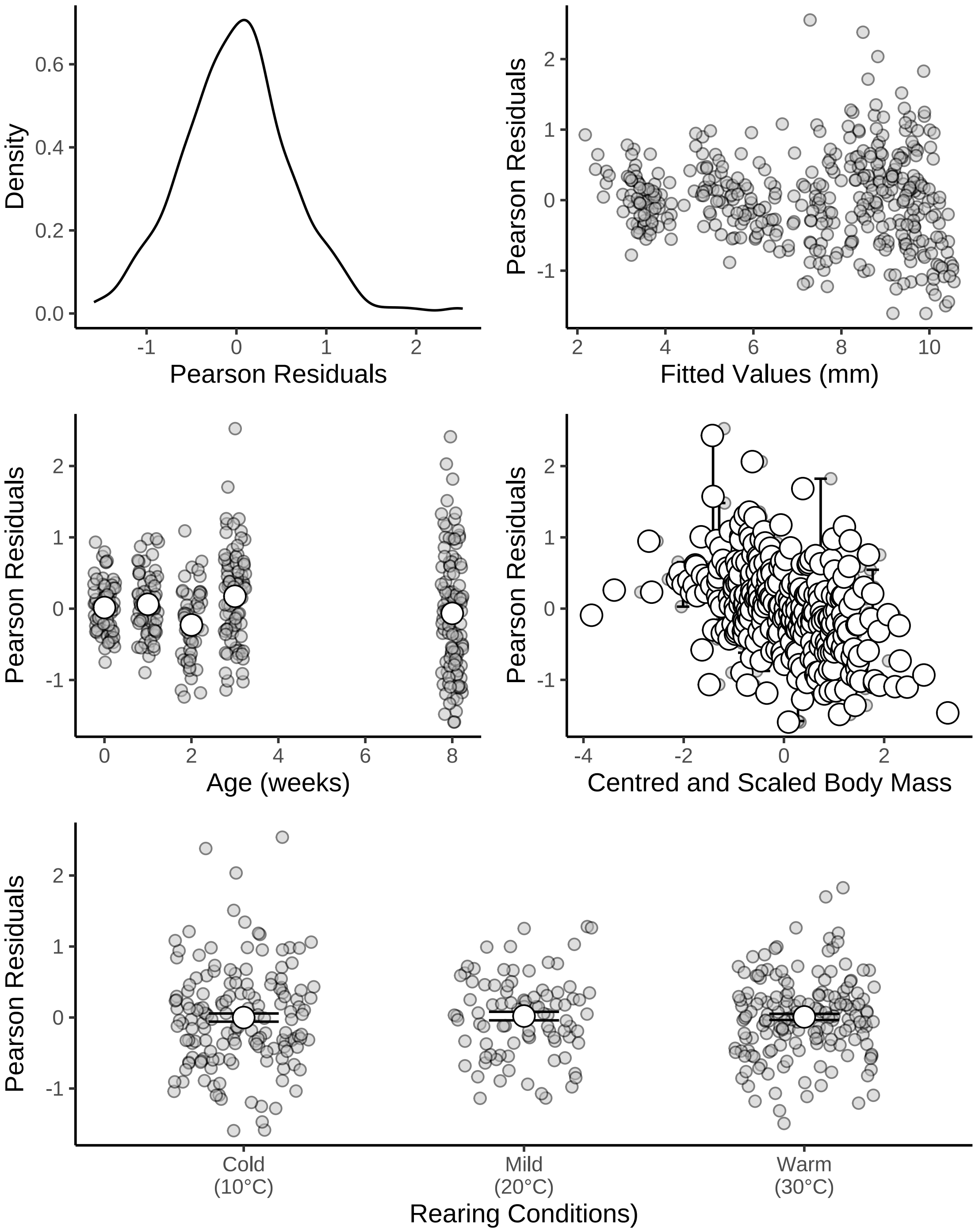

Figure 57: *Density and distributions of Pearson residuals across fitted values and model predictors (including age and rearing conditions). All residuals pertain to those drawn from a Bayesian non-linear model predicting bill length (mm) during growth in Japanese quail. Pearson residuals are shown rather than ordinary residuals to correct for the age-dependence of model error.*

Pearson residuals are homogenous across model predictors. Our model is therefore summarised below, both visually and numerically. Here, we use posterior medians as our measure of central tendency in parameter summaries.

```
as.data.frame(growthModelBill) %>%
  pivot_longer(everything(), names_to = "Parameter", values_to = "Values") %>%
  filter(grepl("b_|sd_", Parameter)) %>%
  arrange(Parameter) %>%
  merge(., tribble(
    ~Parameter, ~Par,
    "b_A_Intercept", "Beta a0",
    "b_A_pretreatmentcold", "Beta a1\n(Cold-reared)",
    "b_A_pretreatmentwarm", "Beta a2\n(Warm-reared)",
    "sd_batch__A_Intercept", "Mu 0a",
    "b_B_Intercept", "Beta b0",
    "b_B_pretreatmentcold", "Beta b1\n(Cold-reared)",
    "b_B_pretreatmentwarm", "Beta b2\n(Warm-reared)",
    "sd_batch__B_Intercept", "Mu 0b",
    "b_C_Intercept", "Beta c0",
    "b_C_pretreatmentcold", "Beta c1\n(Cold-reared)",
    "b_C_pretreatmentwarm", "Beta c2\n(Warm-reared)",
    "sd_batch__C_Intercept", "Mu 0c",
    "b_D_Intercept", "Beta 0",
    "b_D_centredMass", "Beta 1",
    "sd_ring__D_Intercept", "Mu 0\n(Individual Intercept)",
    "b_sigma_intercept", "Tau 0",
    "b_sigma_logweekB", "Tau 1"
  ),
  by = "Parameter", all.x = TRUE
  ) %>%
  filter(!is.na(Par)) %>%
  ggplot(aes(x = Values)) +
  facet_wrap(~Par, scales = "free") +
  geom_density() +
  geom_vline(
    xintercept = 0, linetype = "dashed",
    colour = "firebrick4"
  ) +
  ylab("Density") +
  theme_classic() +
  theme(axis.title.x = element_blank())
```

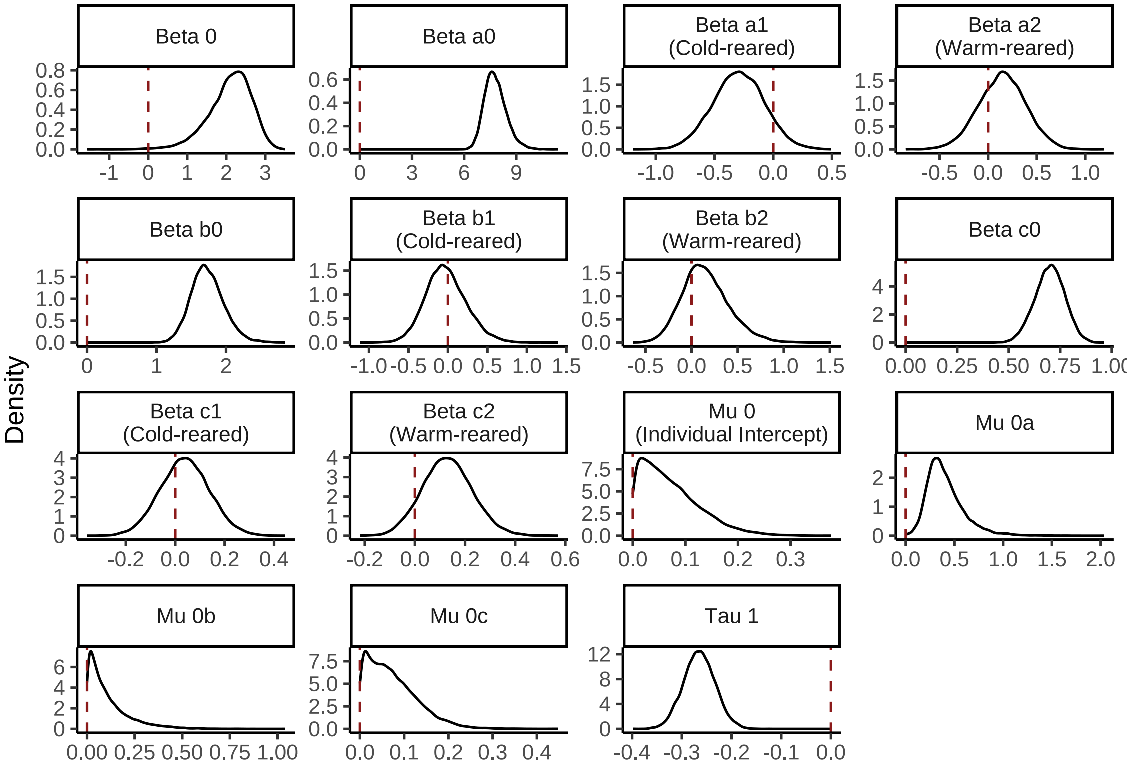

Figure 58: *Density of model coefficients from a Bayesian non-linear model predicting bill length (mm) of Japanese quail during growth. Dashed red lines indicate zero values for each coefficient.*

```
# Possible weak asymptotic reduction among cold-reared quail.
# Summarising model outcomes.

caption <- paste0(
  "Coefficients from a Bayesian non-linear effects ",
  "model predicting bill length (mm) of Japanese quail as a ",
  "Gompertz function of age in weeks. Coefficients represent ",
  "posterior medians and are estimated from bill length data ",
  "collected weekly between 0 and 8 weeks of age. Credible ",
  "intervals (CIs) represent quantile ",
  "intervals around medians"
)

billGrowthModelTable <-
  merge(
    as.data.frame(growthModelBill) %>%
      summarise_all(., .funs = median) %>%
      mutate_all(., .funs = round, 3) %>%
      pivot_longer(everything(),
        names_to = "Parameter",
        values_to = "Values"
      ) %>%
      filter(grepl("b_|sd_", Parameter)) %>%
      arrange(Parameter),
    quantileCIs(growthModelBill, cis = c(50, 95)) %>%
      mutate(
        `50\\% CIs` = paste0(
          "[", round(Low_CI_50, digits = 3),
          ", ", round(High_CI_50, digits = 3), "]"
        ),
        `95\\% CIs` = paste0(
          "[", round(Low_CI_95, digits = 3),
          ", ", round(High_CI_95, digits = 3), "]"
```

```
        )
      ) %>%
      dplyr::select(Parameter, `50\\% CIs`, `95\\% CIs`),
    by = "Parameter"
  ) %>%
  merge(., tribble(
    ~Parameter, ~Par,
    "b_A_Intercept", "Beta a0",
    "b_A_pretreatmentcold", "Beta a1 (Cold-reared)",
    "b_A_pretreatmentwarm", "Beta a2 (Warm-reared)",
    "sd_batch__A_Intercept", "Mu 0a",
    "b_B_Intercept", "Beta b0",
    "b_B_pretreatmentcold", "Beta b1 (Cold-reared)",
    "b_B_pretreatmentwarm", "Beta b2 (Warm-reared)",
    "sd_batch__B_Intercept", "Mu 0b",
    "b_C_Intercept", "Beta c0",
    "b_C_pretreatmentcold", "Beta c1 (Cold-reared)",
    "b_C_pretreatmentwarm", "Beta c2 (Warm-reared)",
    "sd_batch__C_Intercept", "Mu 0c",
    "b_D_Intercept", "Beta 0",
    "b_D_cScaledMass", "Beta 1",
    "sd_ring__D_Intercept", "Mu 0 (Individual Intercept)",
    "b_sigma_Intercept", "Tau 0",
    "b_sigma_logweekB", "Tau 1"
  ),
  by = "Parameter", all.x = TRUE
  ) %>%
  dplyr::select(-Parameter) %>%
  dplyr::select(
    "Parameter" = Par, "Value" = Values, `50\\% CIs`, `95\\% CIs`
  ) %>%
  kbl(.,
    longtable = T, booktabs = T, format = "latex",
    escape = FALSE, caption = caption
  ) %>%
  column_spec(column = c(1:10), width = "2.5cm") %>%
  kable_styling(latex_options = "striped")

billGrowthModelTable
```

Table 23: *Coefficients from a Bayesian non-linear effects model predicting bill length (mm) of Japanese quail as a Gompertz function of age in weeks. Coefficients represent posterior medians and are estimated from bill length data collected weekly between 0 and 8 weeks of age. Credible intervals (CIs) represent quantile intervals around medians*

Parameter	Value	50% CIs	95% CIs
Beta a0	7.739	[7.36, 8.176]	[6.723, 9.205]
Beta a1 (Cold-reared)	-0.294	[-0.441, -0.146]	[-0.725, 0.133]
Beta a2 (Warm-reared)	0.148	[-0.017, 0.309]	[-0.338, 0.617]
Beta b0	1.719	[1.572, 1.881]	[1.308, 2.242]
Beta b1 (Cold-reared)	-0.038	[-0.199, 0.141]	[-0.501, 0.528]
Beta b2 (Warm-reared)	0.132	[-0.02, 0.308]	[-0.292, 0.71]
Beta c0	0.701	[0.651, 0.749]	[0.555, 0.835]
Beta c1 (Cold-reared)	0.039	[-0.028, 0.106]	[-0.154, 0.237]
Beta c2 (Warm-reared)	0.128	[0.062, 0.196]	[-0.059, 0.332]
Beta 1	0.422	[0.403, 0.442]	[0.368, 0.479]
Beta 0	2.160	[1.779, 2.481]	[0.832, 2.98]
Tau 0	0.458	[0.429, 0.487]	[0.372, 0.543]
Tau 1	-0.265	[-0.286, -0.244]	[-0.327, -0.201]

Mu 0a	0.370	[0.276, 0.498]	[0.131, 0.88]
Mu 0b	0.074	[0.03, 0.15]	[0.003, 0.412]
Mu 0c	0.063	[0.028, 0.106]	[0.002, 0.207]
Mu 0 (Individual Intercept)	0.061	[0.028, 0.104]	[0.003, 0.207]

Our model indicates a tendency for bill lengths of cold-reared quail to be smaller than warm-reared quail at their asymptotes. To explicitly test this and other possible differences among treatment groups, we use the Savage-Dickey density ratio method to conduct pairwise comparisons between all treatment groups across all Gompertz parameters.

```
caption <- paste0(
  "Pairwise comparisons of Gompertz function ",
  "variables between cold-reared (10°C until at least 3 ",
  "weeks of age), mild-reared (constant 20°C), ",
  "and warm-reared (30°C until at least 3 weeks of age) ",
  "Japanese quail. Gompertz function variables being ",
  "compared are indicated in parenthesis, per hypothesis."
)

hypotheses <- c(
  paste0(
    c("A", "B", "C"), "_Intercept - ",
    "(", c("A", "B"), "_Intercept + ", c("A", "B", "C"),
    "_pretreatmentcold) > 0"
  ),
  paste0(
    "(", c("A", "B", "C"), "_Intercept + ", c("A", "B", "C"),
    "_pretreatmentwarm) - ", c("A", "B", "C"), "_Intercept > 0"
  ),
  paste0(
    "(", c("A", "B", "C"), "_Intercept + ", c("A", "B", "C"),
    "_pretreatmentwarm) - (", c("A", "B", "C"), "_Intercept + ",
    c("A", "B", "C"), "_pretreatmentcold) > 0"
  )
)

layHypotheses <- c(
  "Cold-reared Asymptote (a) < Mild-reared Asymptote (a)",
  "Cold-reared Displacement (b) < Mild-reared Displacement (b)",
  "Cold-reared Growth Rate (c) < Mild-reared Growth Rate (c)",
  "Warm-reared Asymptote (a) > Mild-reared Asymptote (a)",
  "Warm-reared Displacement (b) > Mild-reared Displacement (b)",
  "Warm-reared Growth Rate (c) > Mild-reared Growth Rate (c)",
  "Cold-reared Asymptote (a) < Warm-reared Asymptote (a)",
  "Cold-reared Displacement (b) < Warm-reared Displacement (b)",
  "Cold-reared Growth Rate (a) < Warm-reared Growth Rate (c)"
)

pairwiseTableGrowthBill <- left_join(
  bind_rows(lapply(hypotheses, FUN = function(x) {
    hypothesis(growthModelBill, hypothesis = x, class = "b", robust = TRUE)$hypothesis
  })) %>% mutate(`95\\% CIs` = paste0(
      "[",
      round(CI.Lower, digits = 3),
      ", ",
      round(CI.Upper, digits = 3),
      "]"
    )) %>%
    dplyr::select(
      Hypothesis, "Delta" = Estimate, `95\\% CIs`, "Evidence Ratio" = Evid.Ratio
      ),
  bind_rows(lapply(hypotheses, FUN = function(x) {
    hypothesis(growthModelBill,
```

```
      hypothesis = x, class = "b",
      robust = TRUE, alpha = 0.2
    )$hypothesis
  })) %>% mutate(`50\\% CIs` = paste0(
      "[",
      round(CI.Lower, digits = 3),
      ", ",
      round(CI.Upper, digits = 3),
      "]"
    )) %>%
    dplyr::select(Hypothesis, `50\\% CIs`),
  by = "Hypothesis"
) %>%
  mutate("hypothesis" = layHypotheses) %>%
  dplyr::select(
    "Hypothesis" = hypothesis, Delta, `50\\% CIs`, `95\\% CIs`,
    `Evidence Ratio`
  ) %>%
  kbl(., longtable = T, booktabs = T, format = "latex",
      caption = caption, escape = FALSE
  ) %>%
  column_spec(column = c(1:10), width = "2.5cm") %>%
  kable_styling(latex_options = "striped")

pairwiseTableGrowthBill
```

Table 24: *Pairwise comparisons of Gompertz function variables between cold-reared (10°C until at least 3 weeks of age), mild-reared (constant 20°C), and warm-reared (30°C until at least 3 weeks of age) Japanese quail. Gompertz function variables being compared are indicated in parenthesis, per hypothesis.*

Hypothesis	Delta	50% CIs	95% CIs	Evidence Ratio
Cold-reared Asymptote (a) < Mild-reared Asymptote (a)	0.2935750	[0.112, 0.477]	[-0.063, 0.656]	10.436741
Cold-reared Displacement (b) < Mild-reared Displacement (b)	0.0384036	[-0.188, 0.236]	[-0.425, 0.423]	1.267574
Cold-reared Growth Rate (c) < Mild-reared Growth Rate (c)	-7.0864187	[-7.636, -6.598]	[-8.253, -6.183]	0.000000
Warm-reared Asymptote (a) > Mild-reared Asymptote (a)	0.1484725	[-0.058, 0.349]	[-0.256, 0.545]	2.662165
Warm-reared Displacement (b) > Mild-reared Displacement (b)	0.1319460	[-0.055, 0.351]	[-0.228, 0.603]	2.539823
Warm-reared Growth Rate (c) > Mild-reared Growth Rate (c)	0.1283300	[0.046, 0.213]	[-0.032, 0.297]	9.596027
Cold-reared Asymptote (a) < Warm-reared Asymptote (a)	0.4412230	[0.249, 0.634]	[0.057, 0.818]	32.402923
Cold-reared Displacement (b) < Warm-reared Displacement (b)	0.1670580	[-0.036, 0.391]	[-0.259, 0.628]	3.163414

Cold-reared Growth Rate (a) < Warm-reared Growth Rate (c)	0.0917135	[0.001, 0.18]	[-0.083, 0.267]	4.036198

Next, we analyse whether bill lengths at maturity (8 weeks of age; the end of our growth curves) differed among rearing treatments using a Bayesian one-way ANOVA, as described previously. Priors on bill length (mm) for cold-, mild-, and warm-reared birds were normally-distributed with means of 9.6 (the raw mean for 8 week old quail in our data), 9.4, and 9.8 respectively (following raw data of Burness et al, 2013), and standard deviations of 1. Our prior for batch effects (included as a group-level intercept) was exponential with a lambda of 5, and that for our error term was half-student t distributed with three degrees of freedom and location and scale parameters of 0 and 2.5 respectively.

```
growthAnovaBill <- brm(
  data = data %>%
    filter(week == 8) %>%
    dplyr::select(pretreatment, mass, billLengthMean,
           "batch" = exp) %>%
    drop_na() %>%
    mutate(pretreatment = factor(pretreatment, levels = c("neutral", "cold", "warm"))),
  formula = billLengthMean ~ 0 + pretreatment + (1|batch),
  prior = c(
    set_prior("normal(9.6, 1)", class = "b",
              coef = "pretreatmentneutral"),
    set_prior("normal(9.4, 1)", class = "b",
              coef = "pretreatmentcold"),
    set_prior("normal(9.8, 1)", class = "b",
              coef = "pretreatmentwarm"),
    set_prior("exponential(5)", class = "sd",
              group = "batch")
  ),
  family = "gaussian",
  seed = 200,
  cores = 4, chains = 4,
  iter = 50000, warmup = 10000, thin = 10,
  control = list(adapt_delta = 0.95, max_treedepth = 13),
  silent = TRUE, refresh = 0,
  file = "./models/_billAtMaturityANOVA.Rds"
)

hypotheses <- c(
  "pretreatmentneutral - pretreatmentcold > 0",
  "pretreatmentwarm - pretreatmentneutral > 0",
  "pretreatmentwarm - pretreatmentcold > 0"
)

caption <- paste0("Results from a Bayesian, one-way ANOVA ",
                  "comparing bill length (mm) at 8 weeks of Japanese ",
                  "quail reared in the cold (10°C until at least ",
                  "3 weeks of age; n = ",
                  nrow(subset(growthAnovaBill$data, pretreatment == "cold")),
                  "), mild temperature (constant 20°C; n = ",
                  nrow(subset(growthAnovaBill$data, pretreatment == "neutral")),
                  "), or warmth (30°C until at least 3 weeks of age; n = ",
                  nrow(subset(growthAnovaBill$data, pretreatment == "warm")),
                  ")."
                  )

billGrowthAnovaTable <- bind_rows(
  lapply(hypotheses, FUN = function(x) {
    hold <- hypothesis(growthAnovaBill,
                       hypothesis = x,
                       class = "b",
                       alpha = 0.5,
```

```
                      robust = TRUE)
    return(data.frame(
      "hyp" = x,
      "deltaMass" = hold$hypothesis$Estimate,
      "pProb" = hold$hypothesis$Post.Prob
    ))
  })
) %>%
  mutate("Hypothesis" = c(
    "Cold-Reared Bill Length < Mild-Reared Bill Length",
    "Warm-Reared Bill Length > Mild-Reared Bill Length",
    "Cold-Reared Bill Length < Warm-Reared Bill Length"
  )) %>%
  mutate(deltaMass = round(deltaMass, digits = 3),
         pProb = round(pProb, digits = 3)) %>%
  dplyr::select(Hypothesis, "Delta Bill Length (mm)" = deltaMass,
         "Posterior Probability" = pProb) %>%
  kbl(.,
    longtable = T, booktabs = T, format = "latex",
    caption = caption
  ) %>%
  column_spec(column = c(1:10), width = "2.5cm")  %>%
  kable_styling(latex_options = "striped")

billGrowthAnovaTable
```

Table 25: *Results from a Bayesian, one-way ANOVA comparing bill length (mm) at 8 weeks of Japanese quail reared in the cold (10°C until at least 3 weeks of age; $n = 42$), mild temperature (constant 20°C; $n = 34$), or warmth (30°C until at least 3 weeks of age; $n = 36$).*

Hypothesis	Delta Bill Length (mm)	Posterior Probability
Cold-Reared Bill Length < Mild-Reared Bill Length	0.180	0.841
Warm-Reared Bill Length > Mild-Reared Bill Length	0.249	0.899
Cold-Reared Bill Length < Warm-Reared Bill Length	0.428	0.994

Effect of rearing treatment on bill length growth curves are below visualised.

```
with(
  growthModelBill$data,
  expand.grid(
    "week" = seq(0, 8, by = 0.1),
    "pretreatment" = c("cold", "neutral", "warm"),
    "cScaledMass" = 0
  )
) %>%
  mutate("weekB" = week + 1) %>%
  mutate(
    "Fit" = predict(growthModelBill,
      re_form = NA,
      newdata = .,
      robust = TRUE
    )[, "Estimate"],
    "SE" = predict(growthModelBill,
      re_form = NA,
```

```
    newdata = .,
    robust = TRUE
  )[, "Est.Error"]
) %>%
mutate(
  "LCL" = Fit - SE,
  "UCL" = Fit + SE
) %>%
mutate(pretreatment = ifelse(pretreatment == "cold",
  "Cold (10°C)",
  ifelse(pretreatment == "neutral",
    "Mild (20°C)",
    "Warm (30°C)"
  )
)) %>%
mutate(pretreatment = factor(pretreatment,
  levels =
    c(
      "Cold (10°C)",
      "Mild (20°C)",
      "Warm (30°C)"
    )
)) %>%
ggplot(aes(
  x = week, y = Fit,
  fill = pretreatment, linetype = pretreatment
)) +
geom_ribbon(aes(x = week, ymin = LCL, ymax = UCL),
  colour = NA, size = 0.25, alpha = 0.3
) +
geom_line(colour = "black", alpha = 0.7) +
stat_summary(
  data = growthModelBill$data %>%
    mutate(pretreatment = ifelse(pretreatment == "cold",
      "Cold (10°C)",
      ifelse(pretreatment == "neutral",
        "Mild (20°C)",
        "Warm (30°C)"
      )
    )) %>%
    mutate(pretreatment = factor(pretreatment,
      levels =
        c(
          "Cold (10°C)",
          "Mild (20°C)",
          "Warm (30°C)"
        )
    )),
  aes(x = week, y = billLengthMean),
  geom = "errorbar", fun.data = "mean_cl_boot",
  colour = "black", alpha = 0.7, width = 0.25,
  position = position_dodge(width = 0.15)
) +
stat_summary(
  data = growthModelBill$data %>%
    mutate(pretreatment = ifelse(pretreatment == "cold",
      "Cold (10°C)",
      ifelse(pretreatment == "neutral",
        "Mild (20°C)",
        "Warm (30°C)"
      )
    )) %>%
    mutate(pretreatment = factor(pretreatment,
      levels =
        c(
          "Cold (10°C)",
          "Mild (20°C)",
```

```
          "Warm (30°C)"
        )
    )),
    aes(x = week, y = billLengthMean),
    geom = "point", fun = "mean", pch = 21, size = 3,
    colour = "black", alpha = 0.7,
    position = position_dodge(width = 0.15)
  ) +
  theme_classic() +
  scale_fill_manual(
    values = c("#7BB4E3", "black", "#CD5C5C"),
    name = "Rearing\nConditions"
  ) +
  scale_linetype_manual(
    values = c("dotted", "solid", "dashed"),
    name = "Rearing\nConditions"
  ) +
  xlab("Age (weeks)") +
  ylab("Bill Length (mm)")
```

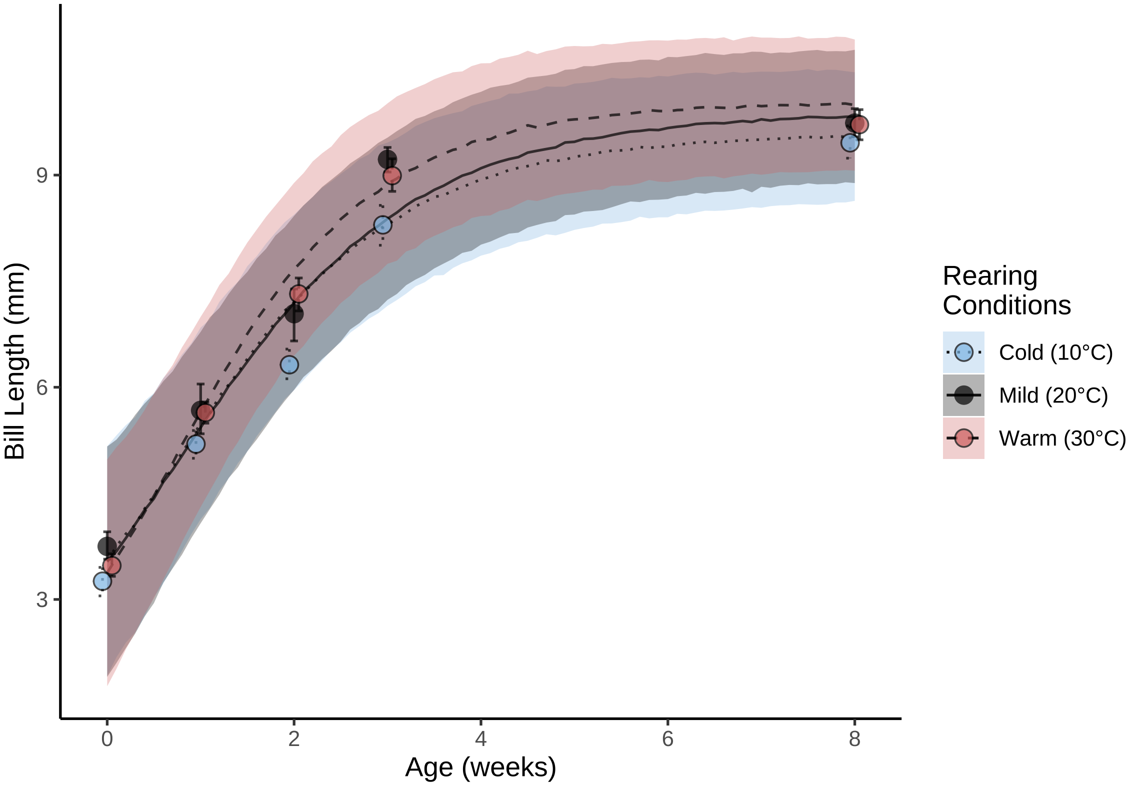

Figure 59: *Bill length (mm) elongation curves of Japanese quail reared in the cold (10°C), mild conditions (20°C), or the warm (30°C) until at least 3 weeks of age. Dots represent mean values and errorbars represent quantile-based 95% credible intervals. Lines represent estimated trends in growth from a Bayesian non-linear model, while ribbons represent confidence around trends (± one standard error).*

Finally, we repeat the above analysis but while restricting our sample to individuals raised in their respective treatments until 8 weeks of age.

```
initFunction <- function(chain_id = 1) {
  list(
    "b_A" = c(rnorm(1, 15.7, 2.5), -0.22),
    "b_B" = c(1.7, 0),
```

```
    "b_C" = c(0.5, 0),
    "b_D" = c(rnorm(1, 0, 1.5),
              rskew_normal(1, xi = 0.2, omega = 0.15, alpha = 5)),
    "b_sigma" = rskew_normal(2, xi = 0, omega = 0.25, alpha = 5),
    "sd_1" = rexp(1, 5)
  )
}

initList <- list(initFunction(),
                 initFunction(),
                 initFunction(),
                 initFunction())

growthModelBillStrict <- brm(
  data =  data %>%
    rename("batch" = exp) %>%
    filter(week <= 8 & batch == "C") %>%
    mutate(pretreatment = factor(pretreatment,
                                 levels = c("cold", "warm")
    )) %>%
    dplyr::select(ring, pretreatment, week, mass, billLengthMean) %>%
    distinct() %>%
    mutate(
      weekB = week + 1
    ) %>%
    group_by(week) %>%
    mutate(cScaledMass = (mass - mean(mass, na.rm = T)) /
             sd(mass, na.rm = T)) %>%
    ungroup(),
  formula = bf(billLengthMean ~ A * exp(-B * exp(-C * week)) + D,
               A ~ 1 + pretreatment,
               B ~ 1 + pretreatment,
               C ~ 1 + pretreatment,
               D ~ 1 + cScaledMass + (1 | ring),
               sigma ~ 0 + Intercept + log(weekB),
               nl = TRUE
  ),
  prior = c(
    set_prior(paste0("normal(", ahat,", 2.5)"),
              class = "b", coef = "Intercept",
              nlpar = "A"
    ),
    set_prior("normal(0.44, 1)",
              class = "b", coef = "pretreatmentwarm",
              nlpar = "A"
    ),
    set_prior("skew_normal(1.7, 0.5, 2.5)",
              class = "b", coef = "Intercept",
              nlpar = "B"
    ),
    set_prior("normal(0, 0.5)",
              class = "b", coef = "pretreatmentwarm",
              nlpar = "B"
    ),
    set_prior("skew_normal(0.5, 0.1, 2.5)",
              class = "b", coef = "Intercept",
              nlpar = "C"
    ),
    set_prior("normal(0, 0.25)",
              class = "b", coef = "pretreatmentwarm",
              nlpar = "C"
    ),
    set_prior("normal(0, 1.5)", class = "b",
              coef = "Intercept", nlpar = "D"),
    set_prior("skew_normal(0.5, 0.15, 5)", class = "b",
              coef = "cScaledMass", nlpar = "D"),
    set_prior("exponential(2.5)", class = "sd",
```

```
              coef = "Intercept", group = "ring", nlpar = "D"),
    set_prior("skew_normal(1, 0.25, 5)", class = "b",
              coef = "Intercept", dpar = "sigma"),
    set_prior("skew_normal(0.2, 0.25, 5)", class = "b",
              coef = "logweekB", dpar = "sigma")
  ),
  family = "gaussian",
  seed = 100,
  cores = 4, chains = 4,
  init = initList,
  backend = "cmdstan", threads = 2,
  iter = 50000, warmup = 10000, thin = 10,
  control = list(adapt_delta = 0.95, max_treedepth = 14),
  #silent = TRUE, refresh = 0,
  file = "./models/_growthModelBillLengthStrict.Rds"
)

# Assessing model diagnostics

ggarrange(
  ggplot(data = data.frame("Rhat" = brms::rhat(growthModelBillStrict)),
         aes(x = Rhat)) +
    geom_density() +
    theme_classic() +
    xlab(
      TeX('$\\hat{R}$')
    ) +
    ylab("Density"),
  ggplot(data = data.frame("Neff" = neffBase(growthModelBillStrict)),
         aes(x = Neff)) +
    geom_density() +
    theme_classic() +
    xlab(
      TeX('$N_{eff}/N~Ratio$')
    ) +
    ylab("Density")
)
```

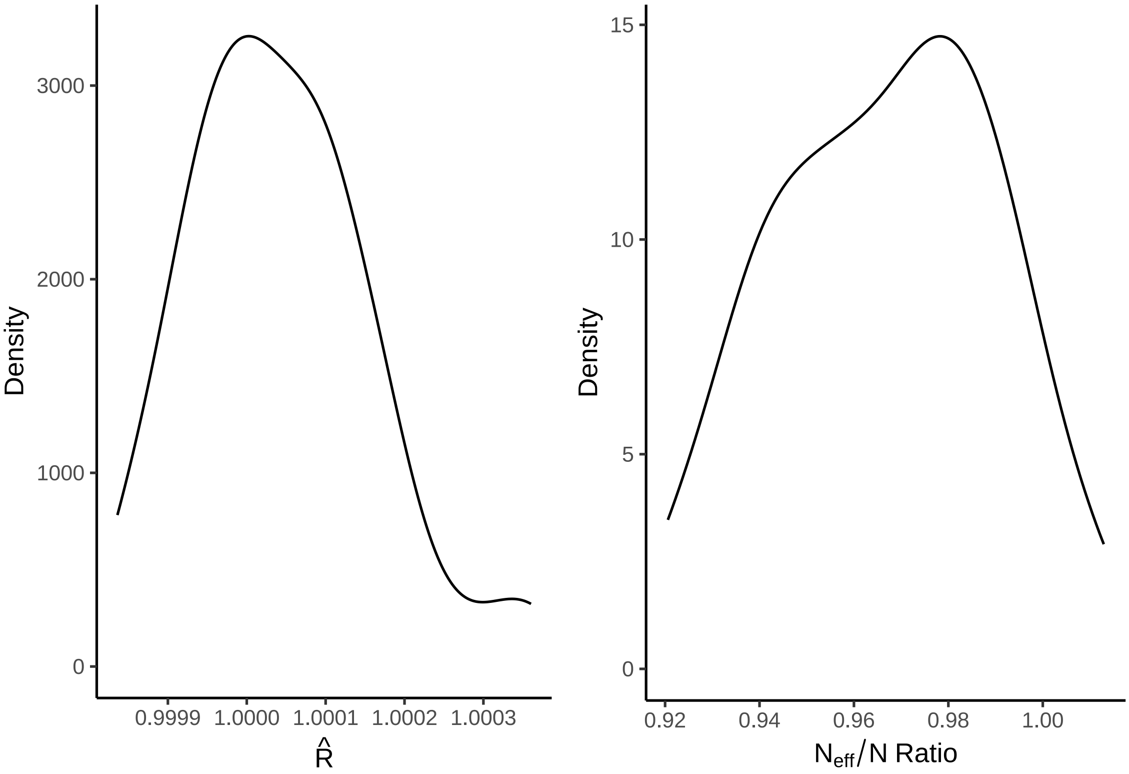

Figure 60: *Gelman-Rubin statistics ($\hat{R}$) and ratio of effective samples sizes by samples sizes per parameter from a Bayesian non-linear model estimating bill length (mm) among Japanese quail reared until maturity in either cold (10°C), mild (20°C) or warm (30°C) conditions.*

Fit of our model and homoskedasticity of residuals are assessed visually.

```
p1 <- pp_check2(growthModelBillStrict, xlab = "Bill Length (mm)")
p2 <- growthModelBillStrict$data %>%
  mutate("Fit" = predict(growthModelBillStrict,
                         robust = TRUE)[, "Estimate"]) %>%
  ggplot(aes(x = billLengthMean, y = Fit, fill = week)) +
  geom_point(pch = 21, colour = "black", size = 2, alpha = 0.5) +
  geom_smooth(method = "lm", colour = "black",
              linetype = "dashed", se = FALSE) +
  scale_fill_gradient2() +
  theme_classic() +
  xlab("Bill Length (mm)") +
  ylab("Predicted Bill Length (mm)")

p1 + p2
```

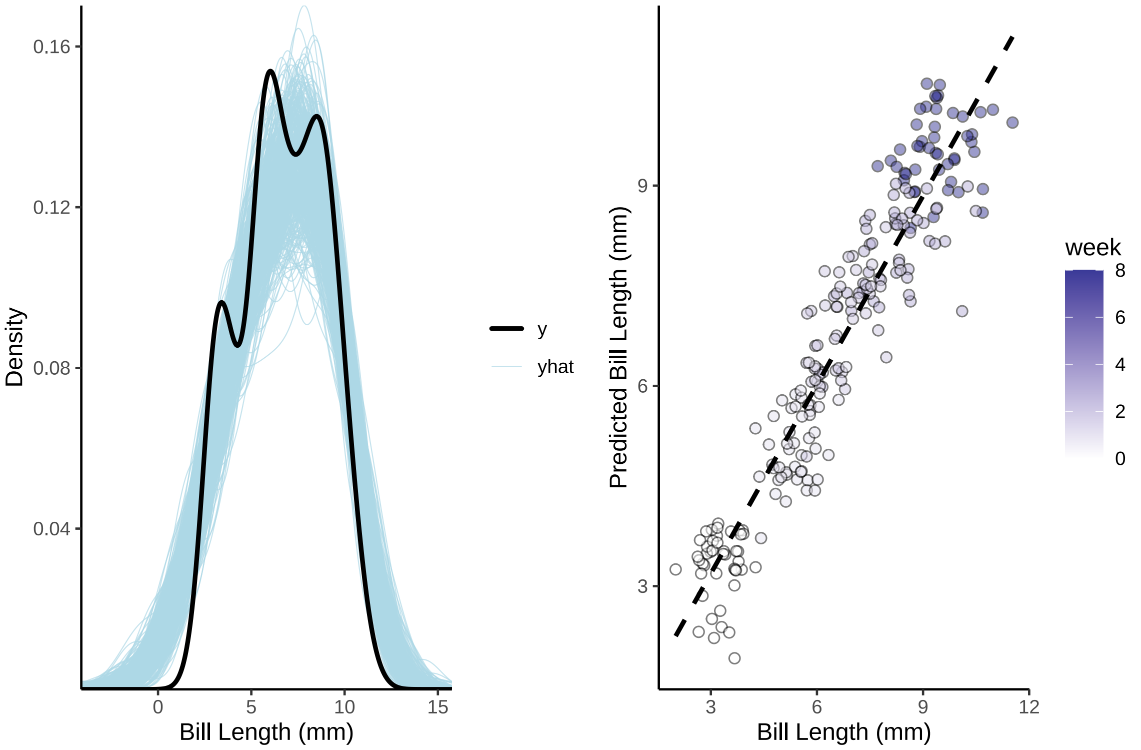

Figure 61: *Posterior predictions of a Bayesian non-linear model predicting Japanese bill length (mm) across time. Predictions are either overlayed with true distributions of quail bill length (panel A) or plotted against true bill length measurements (panel B). Blue lines in panel A represent predictions from posterior draws, while the black line represents true bill length distributions.*

```
p1 <- growthModelBillStrict$data %>%
  mutate("residuals" = residuals(growthModelBillStrict,
    method = "posterior_predict",
    type = "pearson",
    robust = TRUE
  )[, "Estimate"]) %>%
  ggplot(aes(x = residuals)) +
  geom_density(colour = "black", fill = "white") +
  xlab("Pearson Residuals") +
  ylab("Density") +
  theme_classic()

p2 <- growthModelBillStrict$data %>%
  mutate(
    "residuals" = residuals(growthModelBillStrict,
      method = "posterior_predict",
      type = "pearson",
      robust = TRUE
    )[, "Estimate"],
    "fitted" = fitted(growthModelBillStrict, robust = TRUE)[, "Estimate"]
  ) %>%
  ggplot(aes(x = fitted, y = residuals)) +
  geom_point(
    colour = "black", pch = 21, size = 2,
    fill = "grey75", alpha = 0.5
  ) +
  ylab("Pearson Residuals") +
  xlab("Fitted Values (mm)") +
  theme_classic()

p3 <- growthModelBillStrict$data %>%
```

```
  mutate(
    "residuals" =
      residuals(growthModelBillStrict,
        method = "posterior_predict",
        type = "pearson",
        robust = TRUE
      )[, "Estimate"]
  ) %>%
  ggplot(aes(x = week, y = residuals)) +
  geom_point(
    colour = "black", pch = 21, size = 2, fill = "grey75", alpha = 0.5,
    position = position_jitter(width = 0.25)
  ) +
  stat_summary(
    geom = "errorbar", fun.data = "mean_se",
    colour = "black", width = 0.25
  ) +
  stat_summary(
    geom = "point", fun = "mean", pch = 21,
    colour = "black", fill = "white", size = 4
  ) +
  ylab("Pearson Residuals") +
  xlab("Age (weeks)") +
  theme_classic()

p4 <- growthModelBillStrict$data %>%
  mutate(
    "residuals" =
      residuals(growthModelBillStrict,
        method = "posterior_predict",
        type = "pearson",
        robust = TRUE
      )[, "Estimate"]
  ) %>%
  ggplot(aes(x = cScaledMass, y = residuals)) +
  geom_point(
    colour = "black", pch = 21, size = 2, fill = "grey75", alpha = 0.5,
    position = position_jitter(width = 0.25)
  ) +
  stat_summary(
    geom = "errorbar", fun.data = "mean_se",
    colour = "black", width = 0.25
  ) +
  stat_summary(
    geom = "point", fun = "mean", pch = 21,
    colour = "black", fill = "white", size = 4
  ) +
  ylab("Pearson Residuals") +
  xlab("Centred and Scaled Body Mass") +
  theme_classic()

p5 <- growthModelBillStrict$data %>%
  mutate(
    "residuals" =
      residuals(growthModelBillStrict,
        method = "posterior_predict",
        type = "pearson",
        robust = TRUE
      )[, "Estimate"]
  ) %>%
  mutate(pretreatment = str_to_title(pretreatment)) %>%
  mutate(pretreatment = ifelse(pretreatment == "Cold",
    "Cold\n(10°C)",
    ifelse(pretreatment == "Neutral", "Mild\n(20°C)",
      "Warm\n(30°C)"
    )
  )) %>%
```

```
  mutate(pretreatment = factor(pretreatment,
    levels = c(
      "Cold\n(10°C)",
      "Mild\n(20°C)",
      "Warm\n(30°C)"
    )
  )) %>%
  ggplot(aes(x = pretreatment, y = residuals)) +
  geom_point(
    colour = "black", pch = 21, size = 2, fill = "grey75", alpha = 0.5,
    position = position_jitter(width = 0.25)
  ) +
  stat_summary(
    geom = "errorbar", fun.data = "mean_se",
    colour = "black", width = 0.25
  ) +
  stat_summary(
    geom = "point", fun = "mean", pch = 21,
    colour = "black", fill = "white", size = 4
  ) +
  ylab("Pearson Residuals") +
  xlab("Rearing Conditions)") +
  theme_classic()

((p1 + p2) / (p3 + p4)) + p5
```

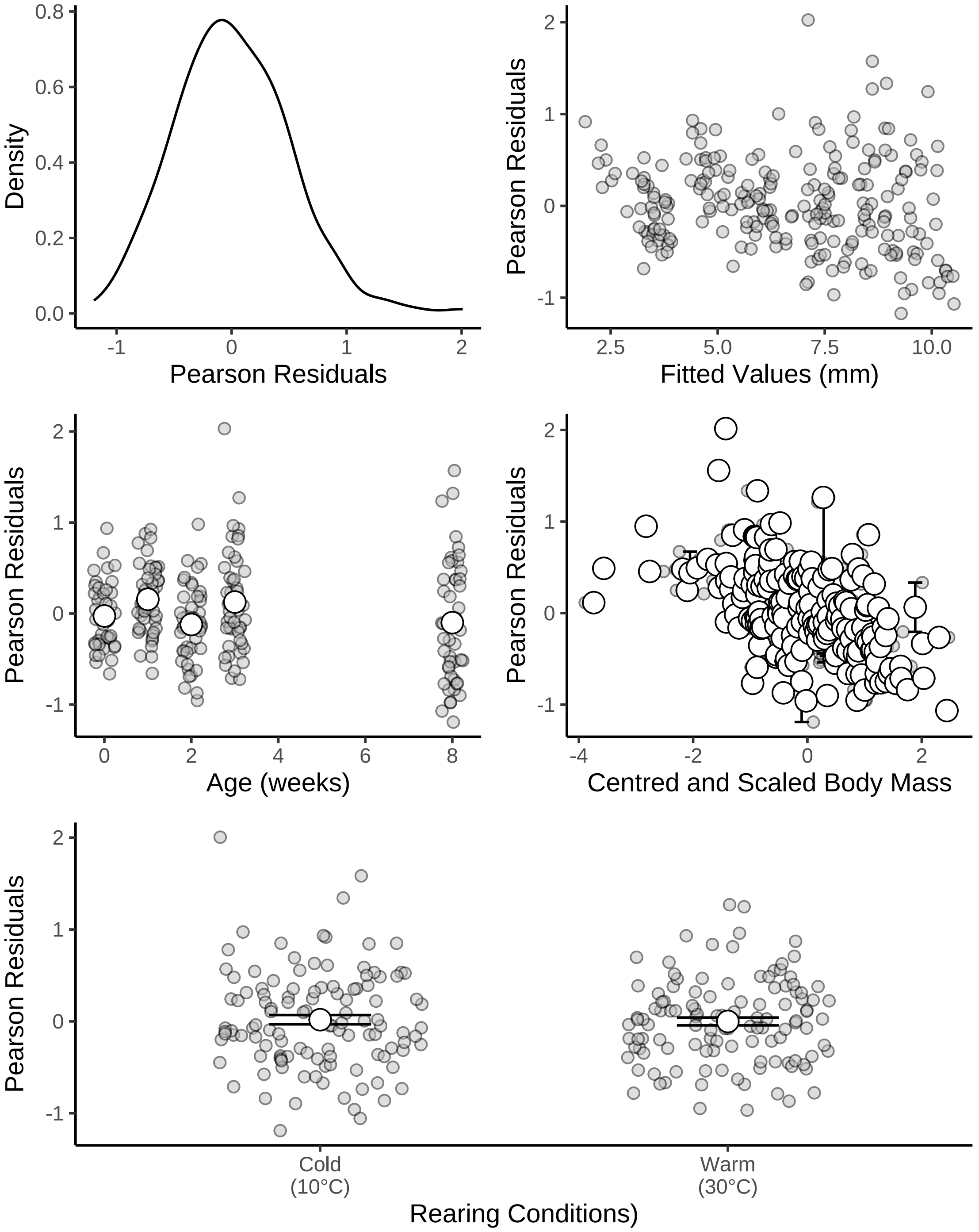

Figure 62: *Density and distributions of Pearson residuals across fitted values and model predictors (including age and rearing conditions). All residuals pertain to those drawn from a Bayesian non-linear model predicting bill length (mm) during growth in Japanese quail. Samples are restricted to individuals that were raised in their respective temperature treatments until adulthood. Pearson residuals are shown rather than ordinary residuals to correct for the age-dependence of model error.*

Predictions from our model correlate clearly with true bill length values and Pearson residuals are homogenous across predictors. Below, we therefore summarise our model outcomes and repeat pairwise comparisons between treatment groups and across Gompertz parameters as described previously.

```
as.data.frame(growthModelBillStrict) %>%
  pivot_longer(everything(), names_to = "Parameter", values_to = "Values") %>%
  filter(grepl("b_|sd_", Parameter)) %>%
  arrange(Parameter) %>%
  merge(., tribble(
    ~Parameter, ~Par,
    "b_A_Intercept", "Beta a0",
    "b_A_pretreatmentcold", "Beta a1\n(Cold-reared)",
    "b_A_pretreatmentwarm", "Beta a2\n(Warm-reared)",
    "sd_batch__A_Intercept", "Mu 0a",
    "b_B_Intercept", "Beta b0",
    "b_B_pretreatmentcold", "Beta b1\n(Cold-reared)",
    "b_B_pretreatmentwarm", "Beta b2\n(Warm-reared)",
    "sd_batch__B_Intercept", "Mu 0b",
    "b_C_Intercept", "Beta c0",
    "b_C_pretreatmentcold", "Beta c1\n(Cold-reared)",
    "b_C_pretreatmentwarm", "Beta c2\n(Warm-reared)",
    "sd_batch__C_Intercept", "Mu 0c",
    "b_D_Intercept", "Beta 0",
    "b_D_centredMass", "Beta 1",
    "sd_ring__D_Intercept", "Mu 0\n(Individual Intercept)",
    "b_sigma_intercept", "Tau 0",
    "b_sigma_logweekB", "Tau 1"
  ),
  by = "Parameter", all.x = TRUE
  ) %>%
  filter(!is.na(Par)) %>%
  ggplot(aes(x = Values)) +
  facet_wrap(~Par, scales = "free") +
  geom_density() +
  geom_vline(
    xintercept = 0, linetype = "dashed",
    colour = "firebrick4"
  ) +
  ylab("Density") +
  theme_classic() +
  theme(axis.title.x = element_blank())
```

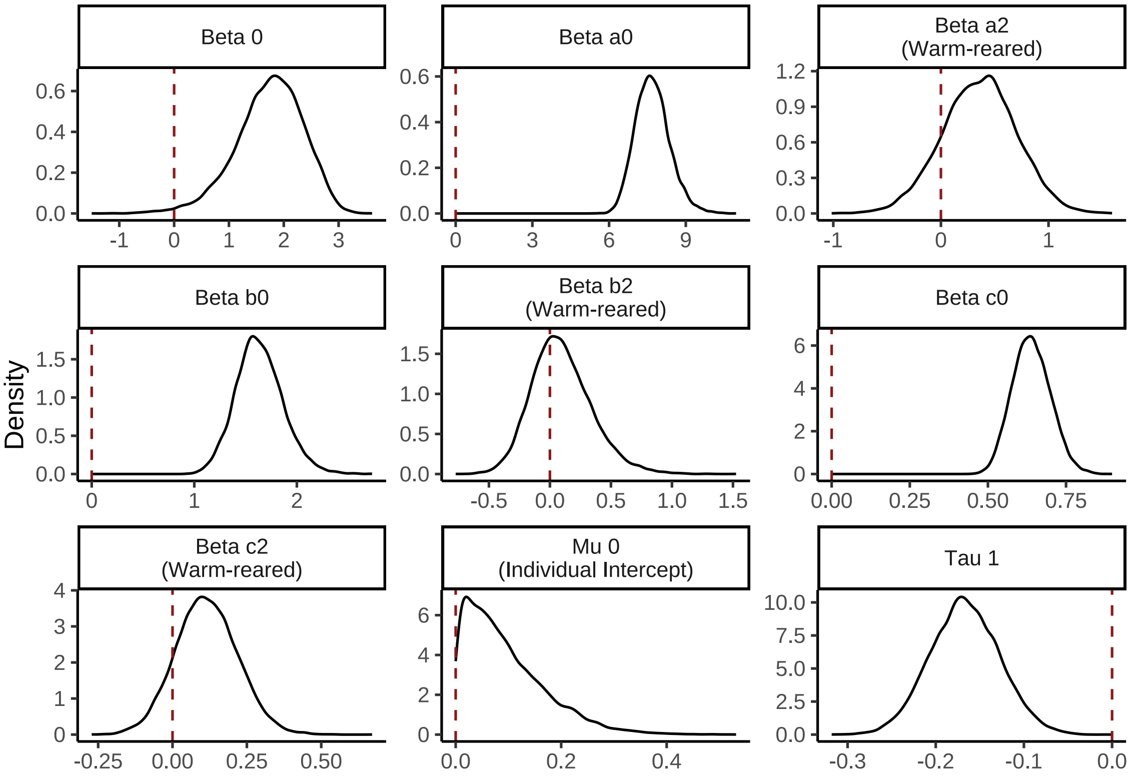

Figure 63: *Density of model coefficients from a Bayesian non-linear model predicting bill length (mm) of Japanese quail during growth. Samples are restricted to individuals that were raised in their respective temperature treatments until adulthood. Dashed red lines indicate zero values for each coefficient.*

```
# Little evidence of effects.

caption <- paste0(
  "Coefficients from a Bayesian non-linear effects ",
  "model predicting bill length (mm) of Japanese quail as a ",
  "Gompertz function of age in weeks. Coefficients represent ",
  "posterior medians and are estimated from bill length data ",
  "collected weekly between 0 and 8 weeks of age. Credible ",
  "intervals (CIs) represent quantile ",
  "intervals around medians. Samples are restricted to individuals ",
  "that were raised in their respective temperature treatments ",
  "until adulthood."
)

billGrowthModelStrictTable <-
  merge(
    as.data.frame(growthModelBillStrict) %>%
      summarise_all(., .funs = median) %>%
      mutate_all(., .funs = round, 3) %>%
      pivot_longer(everything(),
        names_to = "Parameter",
        values_to = "Values"
      ) %>%
      filter(grepl("b_|sd_", Parameter)) %>%
      arrange(Parameter),
    quantileCIs(growthModelBillStrict, cis = c(50, 95)) %>%
      mutate(
        `50\\% CIs` = paste0(
          "[", round(Low_CI_50, digits = 3),
          ", ", round(High_CI_50, digits = 3), "]"
        ),
```

```
        `95\\% CIs` = paste0(
          "[", round(Low_CI_95, digits = 3),
          ", ", round(High_CI_95, digits = 3), "]"
        )
      ) %>%
      dplyr::select(Parameter, `50\\% CIs`, `95\\% CIs`),
    by = "Parameter"
  ) %>%
  merge(., tribble(
    ~Parameter, ~Par,
    "b_A_Intercept", "Beta a0",
    "b_A_pretreatmentcold", "Beta a1 (Cold-reared)",
    "b_A_pretreatmentwarm", "Beta a2 (Warm-reared)",
    "sd_batch__A_Intercept", "Mu 0a",
    "b_B_Intercept", "Beta b0",
    "b_B_pretreatmentcold", "Beta b1 (Cold-reared)",
    "b_B_pretreatmentwarm", "Beta b2 (Warm-reared)",
    "sd_batch__B_Intercept", "Mu 0b",
    "b_C_Intercept", "Beta c0",
    "b_C_pretreatmentcold", "Beta c1 (Cold-reared)",
    "b_C_pretreatmentwarm", "Beta c2 (Warm-reared)",
    "sd_batch__C_Intercept", "Mu 0c",
    "b_D_Intercept", "Beta 0",
    "b_D_cScaledMass", "Beta 1",
    "sd_ring__D_Intercept", "Mu 0 (Individual Intercept)",
    "b_sigma_Intercept", "Tau 0",
    "b_sigma_logweekB", "Tau 1"
  ),
  by = "Parameter", all.x = TRUE
  ) %>%
  dplyr::select(-Parameter) %>%
  dplyr::select(
    "Parameter" = Par, "Value" = Values, `50\\% CIs`, `95\\% CIs`
  ) %>%
  kbl(.,
    longtable = T, booktabs = T, format = "latex",
    escape = FALSE, caption = caption
  ) %>%
  column_spec(column = c(1:10), width = "2.5cm") %>%
  kable_styling(latex_options = "striped")

billGrowthModelStrictTable
```

Table 26: *Coefficients from a Bayesian non-linear effects model predicting bill length (mm) of Japanese quail as a Gompertz function of age in weeks. Coefficients represent posterior medians and are estimated from bill length data collected weekly between 0 and 8 weeks of age. Credible intervals (CIs) represent quantile intervals around medians. Samples are restricted to individuals that were raised in their respective temperature treatments until adulthood.*

Parameter	Value	50% CIs	95% CIs
Beta a0	7.663	[7.23, 8.117]	[6.515, 9.138]
Beta a2 (Warm-reared)	0.363	[0.125, 0.59]	[-0.34, 1.024]
Beta b0	1.623	[1.48, 1.781]	[1.213, 2.125]
Beta b2 (Warm-reared)	0.076	[-0.074, 0.244]	[-0.338, 0.642]
Beta c0	0.637	[0.597, 0.68]	[0.527, 0.763]
Beta c2 (Warm-reared)	0.113	[0.044, 0.183]	[-0.083, 0.324]
Beta 1	0.485	[0.463, 0.509]	[0.423, 0.561]
Beta 0	1.785	[1.371, 2.168]	[0.397, 2.781]
Tau 0	0.610	[0.579, 0.641]	[0.522, 0.7]
Tau 1	-0.167	[-0.194, -0.14]	[-0.243, -0.089]

Mu 0 (Individual Intercept)	0.076	[0.035, 0.135]	[0.003, 0.279]

Pairwise comparisons follow below.

```
caption <- paste0(
  "Pairwise comparisons of Gompertz function ",
  "variables between cold-reared (10°C until at least 8 ",
  "weeks of age), mild-reared (constant 20°C), ",
  "and warm-reared (30°C until at least 8 weeks of age) ",
  "Japanese quail. Gompertz function variables being ",
  "compared are indicated in parenthesis, per hypothesis."
)

hypotheses <- c(
  paste0(
    "(", c("A", "B", "C"), "_Intercept + ", c("A", "B", "C"),
    "_pretreatmentwarm) - ", c("A", "B", "C"), "_Intercept > 0"
  )
)

layHypotheses <- c(
  "Cold-reared Growth Rate (c) < Warm-reared Growth Rate (c)",
  "Cold-reared Displacement (b) < Warm-reared Displacement (b)",
  "Cold-reared Growth Rate (c) < Warm-reared Growth Rate (c)"
)

pairwiseTableGrowthBillStrict <- left_join(
  bind_rows(lapply(hypotheses, FUN = function(x) {
    hypothesis(
      growthModelBillStrict, hypothesis = x, class = "b", robust = TRUE
    )$hypothesis
  })) %>% mutate(`95\\% CIs` = paste0(
      "[",
      round(CI.Lower, digits = 3),
      ", ",
      round(CI.Upper, digits = 3),
      "]"
    )) %>%
    dplyr::select(
      Hypothesis, "Delta" = Estimate, `95\\% CIs`, "Evidence Ratio" = Evid.Ratio
      ),
  bind_rows(lapply(hypotheses, FUN = function(x) {
    hypothesis(growthModelBillStrict,
      hypothesis = x, class = "b",
      robust = TRUE, alpha = 0.2
    )$hypothesis
  })) %>% mutate(`50\\% CIs` = paste0(
      "[",
      round(CI.Lower, digits = 3),
      ", ",
      round(CI.Upper, digits = 3),
      "]"
    )) %>%
    dplyr::select(Hypothesis, `50\\% CIs`),
  by = "Hypothesis"
) %>%
  mutate("hypothesis" = layHypotheses) %>%
  dplyr::select(
    "Hypothesis" = hypothesis, Delta, `50\\% CIs`, `95\\% CIs`,
    `Evidence Ratio`
  ) %>%
  kbl(., longtable = T, booktabs = T, format = "latex",
      caption = caption, escape = FALSE
  ) %>%
  column_spec(column = c(1:10), width = "2.5cm") %>%
```

```
  kable_styling(latex_options = "striped")

pairwiseTableGrowthBill
```

Table 27: *Pairwise comparisons of Gompertz function variables between cold-reared (10°C until at least 3 weeks of age), mild-reared (constant 20°C), and warm-reared (30°C until at least 3 weeks of age) Japanese quail. Gompertz function variables being compared are indicated in parenthesis, per hypothesis.*

Hypothesis	Delta	50% CIs	95% CIs	Evidence Ratio
Cold-reared Asymptote (a) < Mild-reared Asymptote (a)	0.2935750	[0.112, 0.477]	[-0.063, 0.656]	10.436741
Cold-reared Displacement (b) < Mild-reared Displacement (b)	0.0384036	[-0.188, 0.236]	[-0.425, 0.423]	1.267574
Cold-reared Growth Rate (c) < Mild-reared Growth Rate (c)	-7.0864187	[-7.636, -6.598]	[-8.253, -6.183]	0.000000
Warm-reared Asymptote (a) > Mild-reared Asymptote (a)	0.1484725	[-0.058, 0.349]	[-0.256, 0.545]	2.662165
Warm-reared Displacement (b) > Mild-reared Displacement (b)	0.1319460	[-0.055, 0.351]	[-0.228, 0.603]	2.539823
Warm-reared Growth Rate (c) > Mild-reared Growth Rate (c)	0.1283300	[0.046, 0.213]	[-0.032, 0.297]	9.596027
Cold-reared Asymptote (a) < Warm-reared Asymptote (a)	0.4412230	[0.249, 0.634]	[0.057, 0.818]	32.402923
Cold-reared Displacement (b) < Warm-reared Displacement (b)	0.1670580	[-0.036, 0.391]	[-0.259, 0.628]	3.163414
Cold-reared Growth Rate (a) < Warm-reared Growth Rate (c)	0.0917135	[0.001, 0.18]	[-0.083, 0.267]	4.036198

Effects of rearing conditions on allometric relationship between mass and appendage length

Our above-described analyses compares absolute appendage lengths among treatment groups at specific ages. As such, they do not consider how *allometry* with mass may vary by treatment groups. To address this limitation, we then construct simple linear models with absolute tarsus length (mm) or bill length (mm) at maturity as the Gaussian-distributed responses, rearing condition (categorical), body mass (g) and the interaction between rearing condition and body mass as population level predictors, and egg batch as a group-level predictor. The models allow us to test whether the relationship between body size and extremity length may shift according to environmental temperature (as may be expected for interaction coefficients that do not cross zero). To simplify prior construction for our linear models, tarsus length, bill length, and body mass are first mean-centred (placing our intercept near or at 0).

For both of our linear models, priors for intercepts are centred at zero, and normally-distributed (s.d. = 2.5 for model predicting tarsus length and s.d. = 0.5 for that predicting bill length). For the effect of body

mass on tarsus length, we assumed a skew-normal prior with ξ equaling 0, ω equaling 0.25, and α equaling 5, while for the effect of mass on bill length, we assumed a normally-distributed prior with mean of 0 and standard deviation of 0.01. Next, for the effects of cold- and warm-rearing on tarsus length, we assumed normal distributions with means of -0.4 and 0.4 respectively, and standard deviations of 2. For equivalent effects on bill length, we again assumed normally distributed priors but with means of -0.2 and 0.2 and standard deviations of 1. Given we had no *a prior* information about how rearing conditions and body mass might interact to shape tarsus length or bill length, we assumed broad and normal priors with means of 0 and standard deviations of 0.1 for parameters predicting tarsus length and 0.05 for parameters predicting bill length. Finally, for our priors on egg batch, we used exponential distributions with lambda values of 2.5 and 5 (for tarsus and bill length respectively), while for those of ϵ (our error terms), we also used an exponential distribution with a lambda of 1.

Results of our models are summarised and visualised after execution below.

```
tarsusAllometryModel <- brm(
  data = data %>%
    filter(week == 8) %>%
    dplyr::select(pretreatment, mass,
           "tarsus" = tarsusLengthMean,
           "batch" = exp) %>%
    drop_na() %>%
    mutate(pretreatment = factor(pretreatment,
                                 levels = c("neutral", "cold", "warm")
                                 )
           ) %>%
    group_by(pretreatment) %>%
    mutate(mass = mass - mean(mass, na.rm = T),
           tarsus = tarsus - mean(tarsus, na.rm = T)
           ) %>% ungroup(),
  formula = tarsus ~ mass*pretreatment + (1|batch),
  prior = c(
    set_prior("normal(0, 2.5)", class = "Intercept"),
    set_prior("skew_normal(0, 0.25, 5)", class = "b",
              coef = "mass"),
    set_prior("normal(-0.4, 2)", class = "b",
              coef = "pretreatmentcold"),
    set_prior("normal(0, 0.1)", class = "b",
              coef = "mass:pretreatmentwarm"),
    set_prior("normal(0, 0.1)", class = "b",
              coef = "mass:pretreatmentcold"),
    set_prior("normal(0.4, 2)", class = "b",
              coef = "pretreatmentwarm"),
    set_prior("exponential(2.5)", class = "sd",
              group = "batch"),
    set_prior("exponential(1)", class = "sigma")
  ),
  family = "gaussian",
  seed = 200,
  cores = 4, chains = 4,
  iter = 50000, warmup = 10000, thin = 10,
  control = list(adapt_delta = 0.95, max_treedepth = 13),
  silent = TRUE, refresh = 0,
  file = "./models/_tarsusAllometryModel.Rds"
)

caption <- paste0(
  "Results from a Bayesian linear model ",
  "predicting tarsus length (mm) of mature Japanese quail ",
  "as a function of body mass (g), rearing conditions ",
  "and interactions between body mass and rearing conditions. ",
  "week old Japanese quail. Physiological measurements ",
  "Cold rearing indicates ",
  "post-hatch rearing at 10°C, ",
  "relative to 20°C (intercept), ",
  "or 30°C ('warm rearing'). ",
```

```
  "CI indicates quantile ",
  "intervals and BF indicates Bayes Factors."
)

tarsusAllometryModelResults <-
  as.data.frame(tarsusAllometryModel) %>%
  summarise_all(., .funs = median) %>%
  pivot_longer(everything(),
    names_to = "Parameter",
    values_to = "Estimate"
  ) %>%
  merge(., quantileCIs(tarsusAllometryModel, cis = c(50, 95)),
    by = "Parameter", all.x = TRUE
  ) %>%
  filter(grepl("b_|sd_", Parameter)) %>%
  rowwise() %>%
  mutate("BF" = ifelse(Estimate < 0,
    (2 * mean(as.data.frame(
      tarsusAllometryModel
    )[, Parameter] <= 0)) /
      (2 * mean(as.data.frame(
        tarsusAllometryModel
      )[, Parameter] >= 0)),
    (2 * mean(as.data.frame(
      tarsusAllometryModel
    )[, Parameter] >= 0)) /
      (2 * mean(as.data.frame(
        tarsusAllometryModel
      )[, Parameter] <= 0))
  )) %>%
  ungroup() %>%
  mutate(
    "Estimate" = round(Estimate, digits = 4),
    "BF" = round(BF, digits = 4),
    "N" = nrow(tarsusAllometryModel$data)
  ) %>%
  mutate("Parameter" = ifelse(grepl("b_", Parameter),
    gsub("b_", "", Parameter),
    gsub(
      "Intercept", "batch",
      gsub(".*__", "", Parameter)
    )
  )) %>%
  merge(., tribble(
    ~Parameter, ~parameter, ~level,
    "Intercept", "Intercept", "A",
    "mass", "Body Mass (g)", "B",
    "mass:pretreatmentcold", "Body Mass:Cold Rearing", "C",
    "mass:pretreatmentwarm", "Body Mass:Warm Rearing", "D",
    "pretreatmentcold", "Cold Rearing", "E",
    "pretreatmentwarm", "Warm Rearing", "F",
    "batch", "Egg Batch [mu]", "G"
  ),
  by = "Parameter"
  ) %>%
  mutate(
    `50\\% HDI` = paste0("(", paste(
      round(Low_CI_50, digits = 4),
      round(High_CI_50, digits = 4),
      sep = ", "
    ), ")"),
    `95\\% HDI` = paste0("(", paste(
      round(Low_CI_95, digits = 4),
      round(High_CI_95, digits = 4),
      sep = ", "
    ), ")")
  ) %>%
```

```
  dplyr::select(-c(Low_CI_50, High_CI_50, Low_CI_95, High_CI_95)) %>%
  dplyr::select(
    "Parameter" = "parameter", N,
    Estimate, `50\\% HDI`, `95\\% HDI`, BF, level
  ) %>%
  arrange(level) %>%
  dplyr::select(-c(level)) %>%
  kbl(.,
    longtable = T, booktabs = T, format = "latex", escape = FALSE,
    caption = caption
  ) %>%
  column_spec(column = c(1:2), width = "2.2cm") %>%
  column_spec(column = c(3:10), width = "1.9cm") %>%
  kable_styling(latex_options = "striped")

tarsusAllometryModelResults
```

Table 28: *Results from a Bayesian linear model predicting tarsus length (mm) of mature Japanese quail as a function of body mass (g), rearing conditions and interactions between body mass and rearing conditions. week old Japanese quail. Physiological measurements Cold rearing indicates post-hatch rearing at 10°C, relative to 20°C (intercept), or 30°C ('warm rearing'). CI indicates quantile intervals and BF indicates Bayes Factors.*

Parameter	N	Estimate	50% HDI	95% HDI	BF
Intercept	112	0.2491	(-0.0953, 0.6056)	(-0.7898, 1.4599)	2.1911
Body Mass (g)	112	0.0344	(0.0254, 0.0434)	(0.0081, 0.0603)	194.1220
Body Mass:Cold Rearing	112	-0.0299	(-0.0406, -0.0195)	(-0.0606, 0.0011)	33.1151
Body Mass:Warm Rearing	112	-0.0048	(-0.0164, 0.0067)	(-0.0385, 0.0289)	1.5782
Cold Rearing	112	-0.6216	(-1.0074, -0.2317)	(-1.7376, 0.5044)	6.2926
Warm Rearing	112	-0.3595	(-0.7256, -4e-04)	(-1.4074, 0.6693)	3.0090
Egg Batch [mu]	112	0.6132	(0.4051, 0.8616)	(0.0651, 1.5608)	Inf

```
tarsusAllometryPlot <- expand.grid(
  "mass" = seq(min(tarsusAllometryModel$data$mass),
    max(tarsusAllometryModel$data$mass),
    by = 1
  ),
  "pretreatment" = unique(tarsusAllometryModel$data$pretreatment)
) %>%
  mutate(
    "tarsus" = predict(tarsusAllometryModel,
      newdata = .,
      re_form = NA
    )[, "Estimate"],
    "tarsusSE" = predict(tarsusAllometryModel,
      newdata = .,
      re_form = NA
    )[, "Est.Error"]
  ) %>%
  merge(., subset(data, week == 8) %>%
          group_by(pretreatment) %>%
          summarise("deltaMass" = mean(mass, na.rm = T),
                    "deltaTarsus" = mean(tarsusLengthMean, na.rm = T)
                    ) %>%
          drop_na(),
        by = "pretreatment", all.x = TRUE
        ) %>%
```

```
  mutate(
    mass = mass + deltaMass,
    tarsus = tarsus + deltaTarsus,
    pretreatment = factor(pretreatment,
      levels = c("cold", "neutral", "warm")
    )
  ) %>%
  ggplot(aes(
    x = mass, y = tarsus, fill = pretreatment,
    linetype = pretreatment
  )) +
  geom_ribbon(aes(ymin = tarsus - tarsusSE, ymax = tarsus + tarsusSE),
    alpha = 0.5
  ) +
  geom_point(data = subset(data, week == "8") %>%
    dplyr::select(pretreatment, mass,
      "tarsus" = tarsusLengthMean
    ) %>%
    drop_na() %>%
    mutate(pretreatment = factor(pretreatment,
      levels = c("cold", "neutral", "warm")
    )), pch = 21, colour = "black", size = 2, alpha = 0.5) +
  geom_line(colour = "black") +
  xlab("Body Mass (g)") +
  ylab("Tarsus Length (mm)") +
  scale_fill_manual(
    values = c("#7BB4E3", "black", "#CD5C5C"),
    name = "Rearing\nConditions",
    labels = c("Cold (10°C)", "Mild (20°C)", "Warm (30°C)")
  ) +
  scale_linetype_manual(
    values = c("solid", "dashed", "dotted"),
    name = "Rearing\nConditions",
    labels = c("Cold (10°C)", "Mild (20°C)", "Warm (30°C)")
  ) +
  theme_classic() +
  theme(axis.title = element_text(family = "Noto Sans"),
        axis.text = element_text(family = "Noto Sans"),
        legend.title = element_text(family = "Noto Sans"),
        legend.text = element_text(family = "Noto Sans")
        )

showtext_auto(enable = TRUE)
tarsusAllometryPlot
```

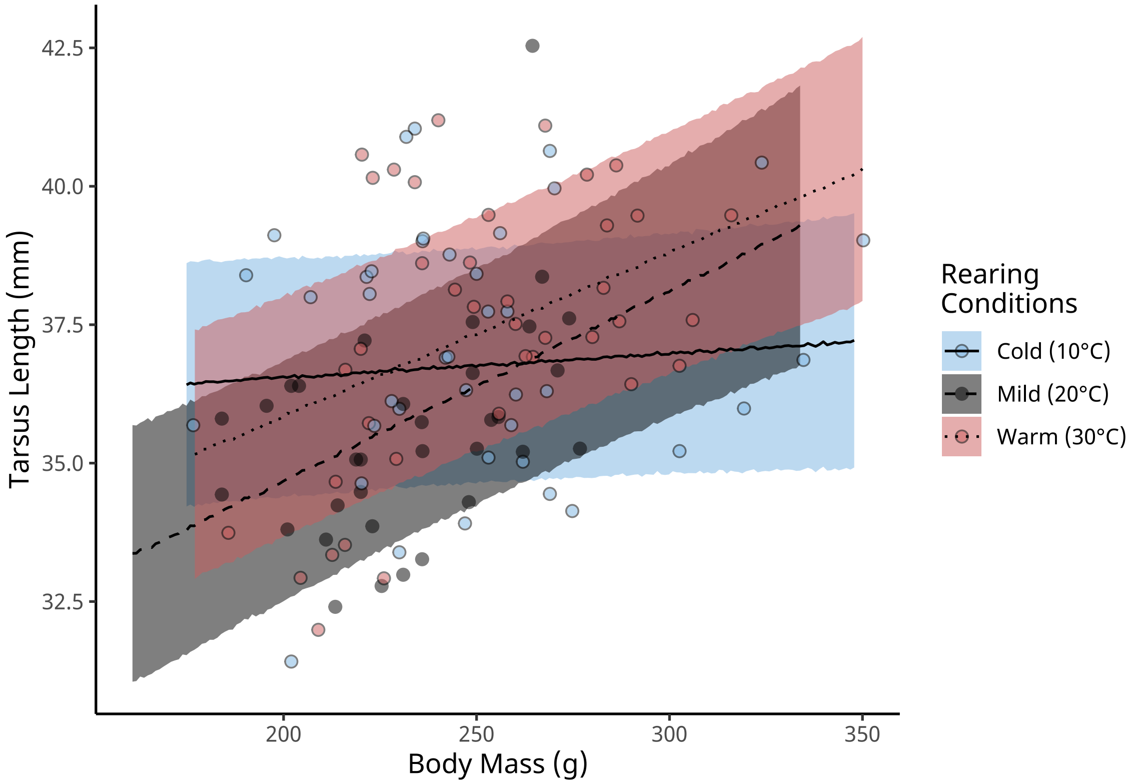

Figure 64: *Effect of body mass (g) and rearing temperature on tarsus length (mm) in eight week old Japanese quail. Rearing temperatures were applied from hatching to at least three weeks of age, after which, temperatures to switched to 20°C in approximately two-thirds half of individuals. Lines represent predicted relationships from a Bayesian mixed effects model and ribbons represent +/- one standard deviation around predicted relationships.*

```
ggsave("./plots/tarsusAllometryPlot.pdf",
  dpi = 800, width = 8, height = 8,
  tarsusAllometryPlot
)
showtext_auto(enable = FALSE)
```

```
file.remove("./models/_billAllometryModel.Rds")
```

```
## [1] TRUE
```

```
billAllometryModel <- brm(
  data = data %>%
    filter(week == 8) %>%
    dplyr::select(pretreatment, mass,
           "bill" = billLengthMean,
           "batch" = exp) %>%
    drop_na() %>%
    mutate(pretreatment = factor(pretreatment,
                                 levels = c("neutral", "cold", "warm")
                                 )
           ) %>%
    group_by(pretreatment) %>%
    mutate(mass = mass - mean(mass, na.rm = T),
           bill = bill - mean(bill, na.rm = T)
           ) %>% ungroup(),
  formula = bill ~ mass*pretreatment + (1|batch),
  prior = c(
    set_prior("normal(0, 0.5)", class = "Intercept"),
    set_prior("normal(0, 0.01)", class = "b",
```

```
                coef = "mass"),
    set_prior("normal(-0.2, 1)", class = "b",
                coef = "pretreatmentcold"),
    set_prior("normal(0, 0.05)", class = "b",
                coef = "mass:pretreatmentwarm"),
    set_prior("normal(0, 0.05)", class = "b",
                coef = "mass:pretreatmentcold"),
    set_prior("normal(0.2, 1)", class = "b",
                coef = "pretreatmentwarm"),
    set_prior("exponential(5)", class = "sd",
                group = "batch"),
    set_prior("exponential(1)", class = "sigma")
  ),
  family = "gaussian",
  seed = 200,
  cores = 4, chains = 4,
  iter = 50000, warmup = 10000, thin = 10,
  control = list(adapt_delta = 0.95, max_treedepth = 13),
  silent = TRUE, refresh = 0,
  file = "./models/_billAllometryModel.Rds"
)
```

```
## Running /Library/Frameworks/R.framework/Resources/bin/R CMD SHLIB foo.c
## Error: object 'LdFlags' not found
## Execution halted
## using C compiler: ‘Apple clang version 16.0.0 (clang-1600.0.26.6)’
## using SDK: ‘’
## clang -arch arm64 -I"/Library/Frameworks/R.framework/Resources/include" -DNDEBUG   -I"/Library/Frameworks/R.framework/Versio
## Error: object 'LdFlags' not found
## Execution halted
## In file included from <built-in>:1:
## In file included from /Library/Frameworks/R.framework/Versions/4.4-arm64/Resources/library/StanHeaders/include/stan/math/pri
## In file included from /Library/Frameworks/R.framework/Versions/4.4-arm64/Resources/library/RcppEigen/include/Eigen/Dense:1:
## In file included from /Library/Frameworks/R.framework/Versions/4.4-arm64/Resources/library/RcppEigen/include/Eigen/Core:19:
## /Library/Frameworks/R.framework/Versions/4.4-arm64/Resources/library/RcppEigen/include/Eigen/src/Core/util/Macros.h:679:10: f
##   679 | #include <cmath>
##       |          ^~~~~~~
## 1 error generated.
## make: *** [foo.o] Error 1
```

```
caption <- paste0(
  "Results from a Bayesian linear model ",
  "predicting bill length (mm) of mature Japanese quail ",
  "as a function of body mass (g), rearing conditions ",
  "and interactions between body mass and rearing conditions. ",
  "week old Japanese quail. Physiological measurements ",
  "Cold rearing indicates ",
  "post-hatch rearing at 10°C, ",
  "relative to 20°C (intercept), ",
  "or 30°C ('warm rearing'). ",
  "CI indicates quantile ",
  "intervals and BF indicates Bayes Factors."
)

billAllometryModelResults <-
  as.data.frame(billAllometryModel) %>%
  summarise_all(., .funs = median) %>%
  pivot_longer(everything(),
    names_to = "Parameter",
    values_to = "Estimate"
  ) %>%
  merge(., quantileCIs(billAllometryModel, cis = c(50, 95)),
    by = "Parameter", all.x = TRUE
  ) %>%
  filter(grepl("b_|sd_", Parameter)) %>%
  rowwise() %>%
  mutate("BF" = ifelse(Estimate < 0,
    (2 * mean(as.data.frame(
```

```
      billAllometryModel
    )[, Parameter] <= 0)) /
      (2 * mean(as.data.frame(
        billAllometryModel
      )[, Parameter] >= 0)),
    (2 * mean(as.data.frame(
      billAllometryModel
    )[, Parameter] >= 0)) /
      (2 * mean(as.data.frame(
        billAllometryModel
      )[, Parameter] <= 0))
  )) %>%
  ungroup() %>%
  mutate(
    "Estimate" = round(Estimate, digits = 4),
    "BF" = round(BF, digits = 4),
    "N" = nrow(billAllometryModel$data)
  ) %>%
  mutate("Parameter" = ifelse(grepl("b_", Parameter),
    gsub("b_", "", Parameter),
    gsub(
      "Intercept", "batch",
      gsub(".*__", "", Parameter)
    )
  )) %>%
  merge(., tribble(
    ~Parameter, ~parameter, ~level,
    "Intercept", "Intercept", "A",
    "mass", "Body Mass (g)", "B",
    "mass:pretreatmentcold", "Body Mass:Cold Rearing", "C",
    "mass:pretreatmentwarm", "Body Mass:Warm Rearing", "D",
    "pretreatmentcold", "Cold Rearing", "E",
    "pretreatmentwarm", "Warm Rearing", "F",
    "batch", "Egg Batch [mu]", "G"
  ),
  by = "Parameter"
  ) %>%
  mutate(
    `50\\% HDI` = paste0("(", paste(
      round(Low_CI_50, digits = 4),
      round(High_CI_50, digits = 4),
      sep = ", "
    ), ")"),
    `95\\% HDI` = paste0("(", paste(
      round(Low_CI_95, digits = 4),
      round(High_CI_95, digits = 4),
      sep = ", "
    ), ")")
  ) %>%
  dplyr::select(-c(Low_CI_50, High_CI_50, Low_CI_95, High_CI_95)) %>%
  dplyr::select(
    "Parameter" = "parameter", N,
    Estimate, `50\\% HDI`, `95\\% HDI`, BF, level
  ) %>%
  arrange(level) %>%
  dplyr::select(-c(level)) %>%
  kbl(.,
    longtable = T, booktabs = T, format = "latex", escape = FALSE,
    caption = caption
  ) %>%
  column_spec(column = c(1:2), width = "2.2cm") %>%
  column_spec(column = c(3:10), width = "1.9cm") %>%
  kable_styling(latex_options = "striped")

billAllometryModelResults
```

Table 29: *Results from a Bayesian linear model predicting bill length (mm) of mature Japanese quail as a function of body mass (g), rearing conditions and interactions between body mass and rearing conditions. week old Japanese quail. Physiological measurements Cold rearing indicates post-hatch rearing at 10°C, relative to 20°C (intercept), or 30°C ('warm rearing'). CI indicates quantile intervals and BF indicates Bayes Factors.*

Parameter	N	Estimate	50% HDI	95% HDI	BF
Intercept	112	-0.0949	(-0.2249, 0.0282)	(-0.5219, 0.2885)	2.3431
Body Mass (g)	112	0.0033	(5e-04, 0.0062)	(-0.005, 0.0116)	3.6702
Body Mass:Cold Rearing	112	-0.0080	(-0.0114, -0.0046)	(-0.018, 0.0021)	16.0758
Body Mass:Warm Rearing	112	-0.0001	(-0.0038, 0.0036)	(-0.0109, 0.0109)	1.0174
Cold Rearing	112	0.0939	(-0.0208, 0.2131)	(-0.2452, 0.4375)	2.4460
Warm Rearing	112	0.2520	(0.1227, 0.3806)	(-0.1269, 0.623)	9.2498
Egg Batch [mu]	112	0.2492	(0.175, 0.3476)	(0.0571, 0.6513)	Inf

```
billAllometryPlot <- expand.grid(
  "mass" = seq(min(billAllometryModel$data$mass),
    max(billAllometryModel$data$mass),
    by = 1
  ),
  "pretreatment" = unique(billAllometryModel$data$pretreatment)
) %>%
  mutate(
    "bill" = predict(billAllometryModel,
      newdata = .,
      re_form = NA
    )[, "Estimate"],
    "billSE" = predict(billAllometryModel,
      newdata = .,
      re_form = NA
    )[, "Est.Error"]
  ) %>%
  merge(., subset(data, week == 8) %>%
          group_by(pretreatment) %>%
          summarise("deltaMass" = mean(mass, na.rm = T),
                    "deltaBill" = mean(billLengthMean, na.rm = T)
                    ) %>%
          drop_na(),
        by = "pretreatment", all.x = TRUE
        ) %>%
  mutate(
    mass = mass + deltaMass,
    bill = bill + deltaBill,
    pretreatment = factor(pretreatment,
      levels = c("cold", "neutral", "warm")
    )
  ) %>%
  ggplot(aes(
    x = mass, y = bill, fill = pretreatment,
    linetype = pretreatment
  )) +
  geom_ribbon(aes(ymin = bill - billSE, ymax = bill + billSE),
    alpha = 0.5
  ) +
  geom_point(data = subset(data, week == "8") %>%
    dplyr::select(pretreatment, mass,
      "bill" = billLengthMean
```

```
    ) %>%
    drop_na() %>%
    mutate(pretreatment = factor(pretreatment,
      levels = c("cold", "neutral", "warm")
    )), pch = 21, colour = "black", size = 2, alpha = 0.5) +
  geom_line(colour = "black") +
  xlab("Body Mass (g)") +
  ylab("Bill Length (mm)") +
  scale_fill_manual(
    values = c("#7BB4E3", "black", "#CD5C5C"),
    name = "Rearing\nConditions",
    labels = c("Cold (10°C)", "Mild (20°C)", "Warm (30°C)")
  ) +
  scale_linetype_manual(
    values = c("solid", "dashed", "dotted"),
    name = "Rearing\nConditions",
    labels = c("Cold (10°C)", "Mild (20°C)", "Warm (30°C)")
  ) +
  theme_classic() +
  theme(axis.title = element_text(family = "Noto Sans"),
        axis.text = element_text(family = "Noto Sans"),
        legend.title = element_text(family = "Noto Sans"),
        legend.text = element_text(family = "Noto Sans")
        )

showtext_auto(enable = TRUE)
billAllometryPlot
```

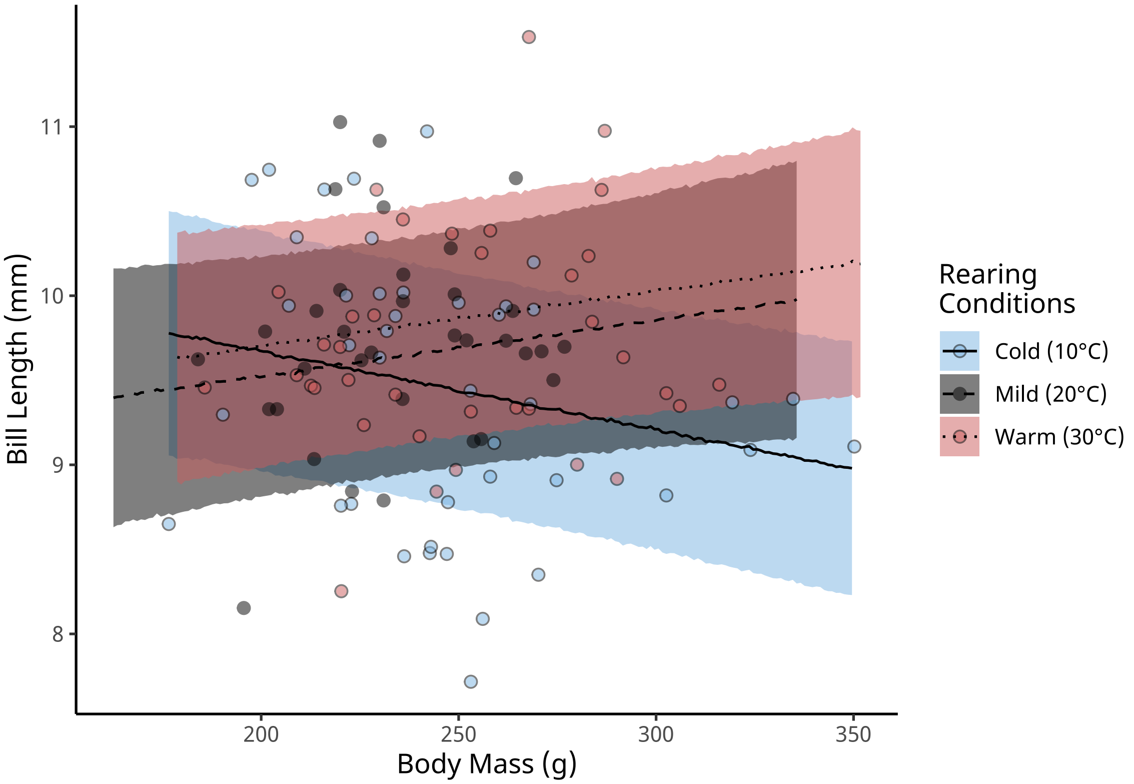

Figure 65: *Effect of body mass (g) and rearing temperature on bill length (mm) in eight week old Japanese quail. Rearing temperatures were applied from hatching to at least three weeks of age, after which, temperatures to switched to 20°C in approximately two-thirds half of individuals. Lines represent predicted relationships from a Bayesian mixed effects model and ribbons represent +/- one standard deviation around predicted relationships.*

```
ggsave("./plots/billAllometryPlot.pdf",
  dpi = 800, width = 8, height = 8,
  billAllometryPlot
)
showtext_auto(enable = FALSE)
```

3.0 Metabolic slope and repeatability analyses

Overview

In the previous section, we showed that patterns of growth and limb elongation in Japanese quail (n = 108) depend on the thermal environment experienced during post-hatch development. Quail reared in cold environmental (10°C) grew more slowly than those reared in warm environments (30°C) but reached higher asymptotic masses and displayed relatively shorter tarsi (and, to a lesser extent, bills) by adulthood. In this document we test whether morphology during development (here, 3 weeks of age) or at maturity (8 weeks of age) influenced thermoregulatory costs in these quail, while also accounting for direct effects of rearing temperature (and thus, physiological acclimation) on such costs.

To approximate thermoregulatory costs in quail, we measured resting energy expenditure at thermoneutrality (30°C), throughout a cold challenge (10°C - 30°C) and throughout a heat challenge (30°C - 40°C). Details of these measurements are described in the methods of Tabh et al (2025). We then quantified the rate at which metabolism increased across cold and heat challenges (using simple Bayesian linear mixed-effects models) assuming that higher rates (or steeper slopes) indicate lower thermoregulatory efficiency and thus, higher thermoregulatory costs relative to lower rates (or shallower slopes). Next, we tested whether morphology and rearing conditions influenced these rates. Throughout this document, we described the steps taken: (1) to quantify metabolic responses to cold and heat challenges, (2) estimate how consistently variable these responses are among individuals, and (3) analyse their potential dependence on morphology and thermal history (i.e. rearing conditions).

Data compilation

We again begin by importing packages and functions required to execute the below analyses. Versions of R packages used are displayed for code reproduction. We recommend that packages versions used here and installed for the those wishing to reproduce our results.

```
library("tidyverse")
library("easypackages")

packageList <- c("bayesplot", "brms", "doParallel",
                 "foreach", "ggpubr", "kableExtra",
                 "latex2exp", "patchwork", "priorsense",
                 "showtext", "tidybayes", "wesanderson")

libraries(packageList)
library("brmsMethods")

# Printing package version numbers

caption <- paste0("R packages and their respective versions used for",
                  " data organisation and analysis in this study."
)

sapply(packageList, function(x) {
  y <- as.character(packageVersion(x))
  return(y)
}, simplify = FALSE) %>%
  enframe(., name = "Package", value = "Version") %>%
  as.data.frame(.) %>%
  kbl(.,
    longtable = T, booktabs = T,
    caption = caption
  ) %>%
  kable_styling(latex_options = "striped")
```

Table 30: *R packages and their respective versions used for data organisation and analysis in this study.*

Package	Version
bayesplot	1.11.1
brms	2.22.7

doParallel	1.0.17
foreach	1.5.2
ggpubr	0.6.0
kableExtra	1.4.0
latex2exp	0.9.6
patchwork	1.2.0
priorsense	1.0.2
showtext	0.9.7
tidybayes	3.0.6
wesanderson	0.3.7

```
# Adding custom functions

## A function to calculate the mode of a vector

md <- function(x) {
  all_values <- unique(x)
  all_values[which.max(tabulate(match(x, all_values)))]
}

## A function to cleanly view autocorrelation between posterior
## draws of specified coefficients/variables

clean_ac <- function(x, prs = NA, names = NA) {
  require("rstan")

  if (class(x)[1] != "brmsfit") {
    return("x must be a brmsfit object.")
  }

  if (is.na(prs[1])) {
    return(stan_ac(x$fit))
  }

  if (!is.na(prs[1]) & is.na(names[1])) {
    return(stan_ac(x$fit, pars = prs))
  }

  if (length(prs) != length(names)) {
    return("Length of pars and names must be equal.")
  }

  Base_plot <- stan_ac(x$fit, pars = prs, fill = nice_pink)
  Base_plot$data$parameters <- as.character(Base_plot$data$parameters)

  for (i in 1:length(prs)) {
    Base_plot$data$parameters[c(which(Base_plot$data$parameters == prs[i]))] <-
      names[i]
  }
  Base_plot$data$parameters <- as.factor(Base_plot$data$parameters)

  return(Base_plot)
}

## A function to simplify the output of bayestestR's hdi function.

simple_hdi <- function(x, rnd = 3, cis = c(50, 95), sci_note = FALSE) {
  if (class(x)[1] != "brmsfit") {
    return("x must be a brmsfit object.")
  }
  if (length(cis) != 2) {
    return("cis must be a vector of integers with length 2")
  }

  HDI_low <- bayestestR::hdi(x, effects = "all", ci = min(cis) / 100)
  HDI_high <- bayestestR::hdi(x, effects = "all", ci = max(cis) / 100)
```

```
  if (sci_note == FALSE) {
    Results <- data.frame(
      "Parameter" = HDI_low$Parameter,
      "1" = round(HDI_low$CI_low, rnd),
      "2" = round(HDI_low$CI_high, rnd),
      "3" = round(HDI_high$CI_low, rnd),
      "4" = round(HDI_high$CI_high, rnd)
    )
    colnames(Results)[c(2:5)] <-
      c(paste0("Low_HDI_", min(cis)), paste0("High_HDI_", min(cis)),
        paste0("Low_HDI_", max(cis)), paste0("High_HDI_", max(cis)))
  } else if (sci_note == TRUE) {
    Results <- data.frame(
      "Parameter" = HDI_low$Parameter,
      "1" = format(round(HDI_low$CI_low, rnd), scientific = TRUE),
      "2" = format(round(HDI_low$CI_high, rnd), scientific = TRUE),
      "3" = format(round(HDI_high$CI_low, rnd), scientific = TRUE),
      "4" = format(round(HDI_high$CI_high, rnd), scientific = TRUE)
    )
    colnames(Results)[c(2:5)] <- c(paste0("Low_HDI_", min(cis)),
                                   paste0("High_HDI_", min(cis)),
                                   paste0("Low_HDI_", max(cis)),
                                   paste0("High_HDI_", max(cis)))
  }

  return(Results)
}

modeHDI <- function(x, cis = c(50, 95), rnd = 4, collapse = FALSE){
  stopifnot("x must be a 'brmsfit' object" = class(x) == "brmsfit",
            "collapse must be logical TRUE/FALSE" = is.logical(collapse))

  out <- lapply(X = as.data.frame(x), MARGIN = 2, FUN = ggdist::mode_hdi,
                .width = c(cis/100))
  hold <- names(out)
  out <- out %>%
    map2(hold, ~mutate(.x, name = .y)) %>%
    bind_rows() %>%
    mutate(y = round(y, digits = rnd),
           ymin = round(ymin, digits = rnd),
           ymax = round(ymax, digits = rnd)) %>%
    select("par" = name, "mode" = y, "lcl" = ymin,
           "ucl"= ymax, "confidenceLevel" = .width)

  if (collapse == FALSE){
    return(out)
  } else if (collapse == TRUE){
    out <- out %>%
      mutate("cis" = paste0("[", lcl, ", ", ucl, "]")) %>%
      select(-c(lcl, ucl))

    return(out)
  }
}


## A function to calculate quantile intervals from a brmsfit object

quantileCIs <- function(x, rnd = 3, cis = c(50, 95), sci_note = FALSE) {
  require(tidyverse)

  if (class(x)[1] != "brmsfit") {
    return("x must be a brmsfit object.")
  }
  if (length(cis) != 2) {
    return("cis must be a vector of integers with length 2")
  }
```

```
  prbs = c()
  nColNames = c()
  for (i in 1:length(cis)){
    prbs = c(prbs, c(0.5 - (cis[i]/100)/2, 0.5 + (cis[i]/100)/2))
    nColNames = c(nColNames,
                  paste0("Low_CI_", cis[i]),
                  paste0("High_CI_", cis[i])
    )
  }

  modelFrame = as.data.frame(x)

  Results <- apply(modelFrame, MARGIN = 2, FUN = quantile,
        probs = prbs, type = 8) %>%
    t() %>%
    as.data.frame() %>%
    rownames_to_column(var = "par") %>%
    `colnames<-`(c("Parameter", nColNames))

  if (sci_note == FALSE) {
    Results <- apply(modelFrame, MARGIN = 2, FUN = quantile,
        probs = prbs, type = 8) %>%
    t() %>%
    as.data.frame() %>%
    rownames_to_column(var = "par") %>%
    `colnames<-`(c("Parameter", nColNames))

  } else if (sci_note == TRUE) {
    Results <- apply(modelFrame, MARGIN = 2, FUN = quantile,
        probs = prbs, type = 8) %>%
    t() %>%
    as.data.frame() %>%
    rownames_to_column(var = "par") %>%
    `colnames<-`(c("Parameter", nColNames)) %>%
    mutate_at(.vars = vars(-Parameter),
              .funs = function(x){
                return(format(x, scientific = TRUE))
              }
    )
  }

  return(Results)
}

## A function that allows users to assign multiple objects
## to different variable names at once. The below is reported by "ellbur" at
## https://strugglingthroughproblems.wordpress.com/author/ellbur/page/3/.

{
  "%=%" <- function(l, r, ...) UseMethod("%=%")

  "%=%.lbunch" <- function(l, r, ..., List = NA) {
    Envir <- as.environment(-1)

    if (!is.na(List)) {
      l <- List[[1]]
      r <- List[[2]]
    }

    if (length(r) > length(l)) {
      warning("RHS has more args than LHS. Only first",
              length(l), "used.")
    }

    if (length(l) > length(r)) {
      warning("LHS has more args than RHS. RHS will be repeated.")
      r <- extendToMatch(r, l)
```

```
    }

    for (II in 1:length(l)) {
      do.call("<-", list(l[[II]], r[[II]]), envir = Envir)
    }
  }

  extendToMatch <- function(source, destin) {
    s <- length(source)
    d <- length(destin)

    if (d == 1 && s > 1 && !is.null(as.numeric(destin))) {
      d <- destin
    }

    dif <- d - s
    if (dif > 0) {
      source <- rep(source, ceiling(d / s))[1:d]
    }
    return(source)
  }

  g <- function(...) {
    List <- as.list(substitute(list(...)))[-1L]
    class(List) <- "lbunch"
    return(List)
  }
}

## A function to simplify output of bayes_R2 from brms

simpleR2 <- function(x, ndraws = 1000, roundDigits = 5,
                     robust = TRUE) {
  stopifnot("x must be a 'brmsfit' object" = class(x) == "brmsfit",
            "robust must be logical (TRUE/FALSE)" = is.logical(robust))
  grab <- brms::bayes_R2(x, ndraws = ndraws, robust = robust)
  toPrint <- paste0(
    "R2 = ", round(grab[, "Estimate"], digits = roundDigits),
    " [",
    round(grab[, "Q2.5"], digits = roundDigits),
    ",",
    round(grab[, "Q97.5"], digits = roundDigits),
    "]"
  )
  cat(toPrint)
}

## A function to calculate the position of a skew-normal
## distribution given its mean, omega, and alpha values

skewxi <- function(mean, omega, alpha) {
  delta <- alpha / (sqrt(1 + alpha^2))
  xi <- mean - omega * delta * sqrt(2 / pi)
  return(xi)
}

## A function to produce clean posterior or prior
## predictive checks, based upon "pp_check" from the R-package 'brms'.

pp_check2 <- function(model, resp = NA, ndraws2 = 500,
                      xlab = "label", colour = "lightblue") {
  require(brms)
  require(ggplot2)
  stopifnot("Model must be a brmsfit object" = is.brmsfit(model))

  if (is.na(resp)) {
    resp <- model$formula$resp
```

```
  }

  p1 <- brms::pp_check(model, ndraws = ndraws, resp = resp) +
    scale_colour_manual(
      values = c("black", colour),
      labels = c("y", "yhat"),
      name = NULL
    ) +
    xlab(xlab) +
    ylab("Density") +
    theme_classic()
  return(p1)
}

## A function to summarise and print Gelman-Rubin statistics and effective
## sample sizes to sample sizes for brmsfit objects.

neffBase <- function(x){
  stopifnot("Model must be a brmsfit object" = is.brmsfit(x))
  out <- as.data.frame(brms::neff_ratio(x)) %>%
    rownames_to_column(var = "var") %>%
    filter(!(var %in% c("lprior", "lp__"))) %>%
    pull(.)
  return(out)
}

chainCheck <- function(model, rDig = 3) {
  require(brms)
  stopifnot("Model must be a brmsfit object" = is.brmsfit(model))

  Rhat <- paste0(
    "Rhat range: ",
    round(min(rhat(model)), digits = rDig),
    " - ",
    round(max(rhat(model)), digits = rDig)
  )
  Neff <- paste0(
    "Neff/N range: ",
    round(min(neffBase(model)), digits = rDig),
    " - ",
    round(max(neffBase(model)), digits = rDig)
  )
  cat(paste0(Rhat, "\n", Neff))
}

## Setting working directory.

setwd("/Users/joshuatabh/analyses")
```

Import, collation, and visualisation of resting energy expenditure data

Data pertaining to both morphometry and resting energy expenditure of our quail are first loaded, organised, and quality-checked. Visualisations of data and identification of erroneous data points is conducted below as above. Following quality checks, energy expenditure data is bound to morphometric data that was previously compiled above.

Compilation of morphometric data is described fully in the previous section (2.0) of this document.

```
# Loading in morphological data

data <- read.csv("compiledDataFull.csv")

# Loading in raw metabolic rate data

vo2Data <- bind_rows(
```

```
  read.csv("exp1VO2.csv"),
  read.csv("exp2VO2.csv"),
  read.csv("exp3VO2.csv")
) %>%
  dplyr::select(-c(startTime, endTime, initialTb, fileName)) %>%
  mutate(pretreatment = ifelse(pretreatment == "control",
                               "neutral", pretreatment))

# Joining metabolic data with morphology data

all <- merge(
  data %>%
    dplyr::select(-Ta),
  vo2Data %>%
    distinct() %>%
    dplyr::select(-c(pretreatment, posttreatment, birdID)),
  by = c("ring", "week", "exp"),
  all.x = TRUE
) %>%
  distinct()

# Re-ordering columns more logically

all <- all %>%
  dplyr::select(ring, birdID, sex, exp, week, pretreatment,
         posttreatment, treatment, Ta, mass, wingLength,
         meanTb, tarsusLengthMean, tarsusLengthSD,
         tarsusCalibration, billLengthMean, billLengthSD,
         billCalibration, VO2, RMR)

# Ambient temperature measurements from experiment C are binned and
# only metabolic data obtained at 30°C and 40°C are retained
# for consistency with experiments A and B.

all <- all %>%
  mutate(Ta = ifelse(exp == "C" & Ta > 28 & Ta < 31, 30, Ta)) %>%
  mutate(Ta = ifelse(exp == "C" & Ta > 38 & Ta < 41, 40, Ta)) %>%
  filter(Ta %in% c(10, 20, 30, 40))

# Checking for spurious VO2 measurements with Cleveland dotplot

ggplot(
  all %>%
    filter(week %in% c(3, 8)) %>%
    group_by(week) %>%
    mutate("ID" = 1:n()) %>%
    ungroup() %>%
    mutate(
      "week" = paste0("Age = ", week, " weeks"),
      "Ta" = paste0(Ta, "°C")
    ),
  aes(x = ID, y = VO2)
) +
  facet_grid(week ~ Ta, scales = "free") +
  geom_point(size = 2, colour = "black",
             fill = "slateblue", alpha = 0.7) +
  geom_rect(
    data = all %>%
      filter(week %in% c(3, 8)) %>%
      group_by(week, Ta) %>%
      summarise(
        "Mean" = mean(VO2, na.rm = T),
        "LCL" = Mean - 3 * sd(VO2, na.rm = T),
        "UCL" = Mean + 3 * sd(VO2, na.rm = T),
        "ID" = 1, "VO2" = 1
      ) %>%
      ungroup() %>%
```

```
    mutate(
      "week" = paste0("Age = ", week, " weeks"),
      "Ta" = paste0(Ta, "°C")
    ) %>%
    as.data.frame(),
  aes(xmin = -Inf, xmax = Inf, ymin = LCL, ymax = UCL),
  colour = "black", fill = "grey70", alpha = 0.5
) +
theme_classic() +
xlab("Sample Number") +
ylab(TeX('$Resting~Metabolism~(mL~O_{2}/min)$'))
```

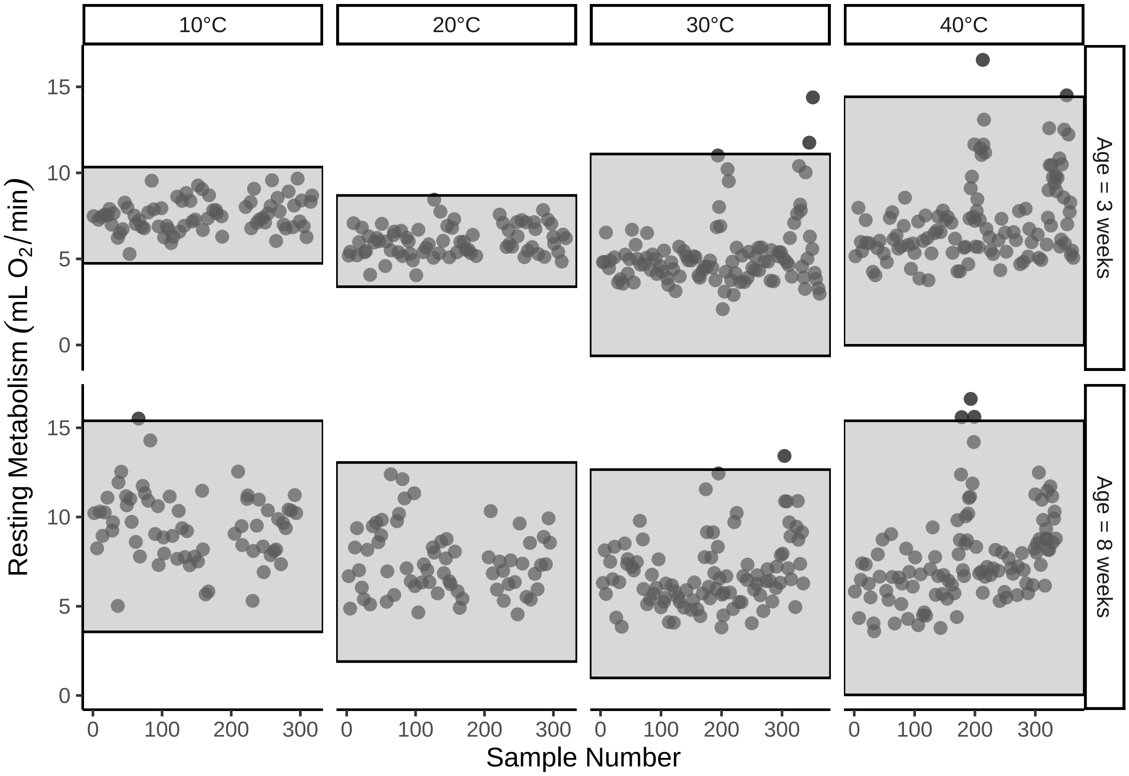

Figure 66: *Cleveland dotplot of resting metabolism measures drawn from 3 and 8 week old Japanese quail. Measurements are divided by ambient temperature during collection. Grey boxes represent means ± three standard deviations. Dots outside of grey rectangles represent tentative outliers.*

A few data points observed at 30°C and 40°C appear high relative to mean values, but are not particularly abnormal from general trends. We retain these values given that we have no reason to expect error.

Next, we check for duplications in our data and further visualise resting metabolism as a function of morphology (here, body mass, tarsus length, bill length, relative [or residual] tarsus length, and relative bill length) across rearing treatments and ambient temperature of measurement.

```
# Checking for, and removing, duplicates

all %>%
  filter(week %in% c(3, 8)) %>%
  group_by(ring, week, Ta, VO2) %>%
  dplyr::count() %>%
  filter(n > 1) %>%
  dplyr::select("ID" = ring, "Age (weeks)" = week,
                "Ambient Temp. (°C)" = Ta, "RMR (mL O2/min)" = VO2,
```

```
                "Count" = n) %>%
  kbl(.,
    longtable = T, booktabs = T, format = "latex",
    caption = paste0("Individual quail with duplicate ",
                     "measurements - part A."), escape = FALSE,
  ) %>%
  kable_styling(latex_options = "striped")
```

Table 31: *Individual quail with duplicate measurements - part A.*

ID	Age (weeks)	Ambient Temp. (°C)	RMR (mL O2/min)	Count
B14	3	10	7.834142	2
B14	3	20	5.528916	2
B14	3	30	4.545016	2
B14	3	40	4.277006	2

```
# Duplicate measurements for one individual at each temperature treatment

all %>%
  mutate("Row" = 1:n()) %>%
  filter((ring == "B14" & week == "3")) %>%
  dplyr::select(-c(
    birdID, sex, exp, posttreatment, treatment, meanTb,
    tarsusLengthSD, tarsusCalibration, wingLength,
    billLengthSD, billCalibration, RMR, Row
  )) %>%
  mutate(pretreatment = ifelse(pretreatment == "warm", "Warm (30°C)",
                               ifelse(pretreatment == "cold", "Cold (10°C)",
                                      "Mild (20°C)")
                               ),
         tarsusLengthMean = round(tarsusLengthMean, digits = 3),
         billLengthMean = round(billLengthMean, digits = 3),
         VO2 = round(VO2, digits = 3)
         ) %>%
  arrange(Ta) %>%
  dplyr::select("ID" = ring, "Age (weeks)" = week,
                "Rearing Conditions" = pretreatment,
                "Body Mass (g)" = mass,
                "Tarsus Length (mm)" = tarsusLengthMean,
                "Bill Length (mm)" = billLengthMean,
                "Ambient Temp. (°C)" = Ta, "RMR (mL O2/min)" = VO2) %>%
  kbl(.,
    longtable = T, booktabs = T, format = "latex",
    caption = paste0("Individual quail with duplicate ",
                     "measurements - part B."), escape = FALSE,
  ) %>%
  column_spec(column = c(1:2), width = "1.4cm") %>%
  column_spec(column = c(3:10), width = "1.8cm") %>%
  kable_styling(latex_options = "striped")
```

Table 32: *Individual quail with duplicate measurements - part B.*

ID	Age (weeks)	Rearing Conditions	Body Mass (g)	Tarsus Length (mm)	Bill Length (mm)	Ambient Temp. (°C)	RMR (mL O2/min)
B14	3	Warm (30°C)	152.9	38.996	8.845	10	7.834
B14	3	Warm (30°C)	152.9	37.206	8.845	10	7.834
B14	3	Warm (30°C)	152.9	38.996	8.845	20	5.529
B14	3	Warm (30°C)	152.9	37.206	8.845	20	5.529
B14	3	Warm (30°C)	152.9	38.996	8.845	30	4.545
B14	3	Warm (30°C)	152.9	37.206	8.845	30	4.545
B14	3	Warm (30°C)	152.9	38.996	8.845	40	4.277
B14	3	Warm (30°C)	152.9	37.206	8.845	40	4.277

```
# Tarsus measurements uniquely doubled. Averaging values and proceeding.

all <- rbind(
  all %>%
    filter(ring == "B14" & week == "3") %>%
    group_by(Ta) %>%
    mutate(
      "tarsusLengthMean" = mean(tarsusLengthMean, na.rm = T)
    ) %>%
    mutate("n" = 1:2) %>%
    ungroup() %>%
    arrange(n, Ta) %>%
    slice(1:4) %>%
    dplyr::select(-n),
  all %>%
    filter(!(ring == "B14" & week == "3"))
) %>%
  arrange(exp, week, ring, Ta)

# Plotting VO2 as kJ/day across tarsus length and mass

mass3Weeks <- ggplot(all %>%
  mutate(
    Ta = paste0(Ta, "°C"),
    pretreatment = ifelse(pretreatment == "cold", "Cold (10°C)",
      ifelse(pretreatment == "neutral", "Mild (20°C)",
        "Warm (30°C)"
      )
    )
  ) %>%
  filter(week == 3) %>%
  drop_na(pretreatment), aes(x = mass, y = VO2)) +
  facet_wrap(~Ta, scale = "free") +
  geom_point(pch = 21, colour = "black", size = 2, aes(fill = pretreatment)) +
  theme_classic() +
  scale_fill_manual(values = c("#7BB4E3", "black", "#CD5C5C"),
                    name = "Rearing\nConditions") +
  xlab("Body Mass at 3 Weeks (g)")  +
  ylab(TeX('$Resting~Metabolism~(mL~O_{2}/min)$'))

mass8Weeks <- ggplot(all %>%
  mutate(
    Ta = paste0(Ta, "°C"),
    pretreatment = ifelse(pretreatment == "cold", "Cold (10°C)",
      ifelse(pretreatment == "neutral", "Mild (20°C)",
        "Warm (30°C)"
      )
    )
  ) %>%
  filter(week == 8) %>%
  drop_na(pretreatment), aes(x = mass, y = VO2)) +
  facet_wrap(~Ta, scales = "free") +
  geom_point(pch = 21, colour = "black", size = 2, aes(fill = pretreatment)) +
  theme_classic() +
  scale_fill_manual(values = c("#7BB4E3", "black", "#CD5C5C"),
                    name = "Rearing\nConditions") +
  xlab("Body Mass at Maturity (g)")  +
  ylab(TeX('$Resting~Metabolism~(mL~O_{2}/min)$'))

showtext_auto()
ggarrange(mass3Weeks, mass8Weeks, ncol = 1,
  common.legend = T
)
```

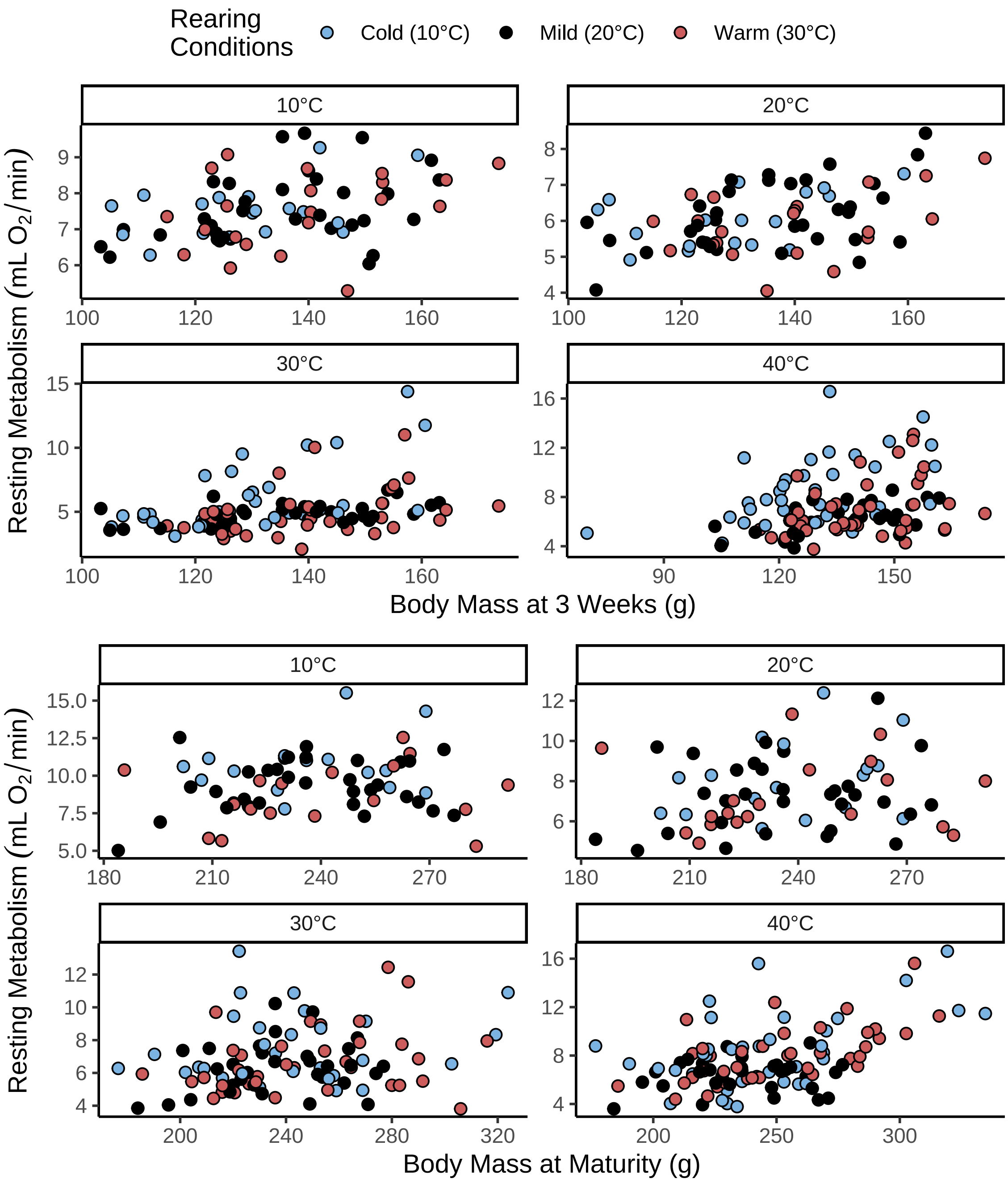

Figure 67: *Resting metabolic rate (mL O_2/min) as a function of body mass (g) in 3 and 8 week old Japanese quail. Resting metabolic rate was measured at four different thermal environments (10°C, 20°C, 30°C and 40°C), with individuals measured in at least two environments. Measured quail were reared in one of three difference rearing conditions: (1) cold (10°C) until at least three weeks of age, (2) mild conditions (20°C) for eight weeks of age, or (3) warmth (30°C) until at least three weeks of age.*

```
showtext_auto(enable = FALSE)
```

```
tarsus3Weeks <- ggplot(all %>%
  mutate(
    Ta = paste0(Ta, "°C"),
    pretreatment = ifelse(pretreatment == "cold", "Cold (10°C)",
      ifelse(pretreatment == "neutral", "Mild (20°C)",
```

```
        "Warm (30°C)"
      )
    )
  ) %>%
  filter(week == 3) %>%
  drop_na(pretreatment), aes(x = tarsusLengthMean, y = VO2)) +
  facet_wrap(~Ta, scale = "free") +
  geom_point(pch = 21, colour = "black", size = 2, aes(fill = pretreatment)) +
  theme_classic() +
  scale_fill_manual(values = c("#7BB4E3", "black", "#CD5C5C"),
                    name = "Rearing\nConditions") +
  xlab("Tarsus Length at Week 3 (mm)")  +
  ylab(TeX('$Resting~Metabolism~(mL~O_{2}/min)$')) +
  theme(legend.position = "none")

tarsus8Weeks <- ggplot(all %>%
  mutate(
    Ta = paste0(Ta, "°C"),
    pretreatment = ifelse(pretreatment == "cold", "Cold (10°C)",
      ifelse(pretreatment == "neutral", "Mild (20°C)",
        "Warm (30°C)"
      )
    )
  ) %>%
  filter(week == 8) %>%
  drop_na(pretreatment), aes(x = tarsusLengthMean, y = VO2)) +
  facet_wrap(~Ta, scales = "free") +
  geom_point(pch = 21, colour = "black", size = 2, aes(fill = pretreatment)) +
  theme_classic() +
  scale_fill_manual(values = c("#7BB4E3", "black", "#CD5C5C"),
                    name = "Rearing\nConditions") +
  xlab("Tarsus Length at Maturity (mm)")  +
  ylab(TeX('$Resting~Metabolism~(mL~O_{2}/min)$')) +
  theme(legend.position = "bottom")

showtext_auto()
tarsus3Weeks/tarsus8Weeks
```

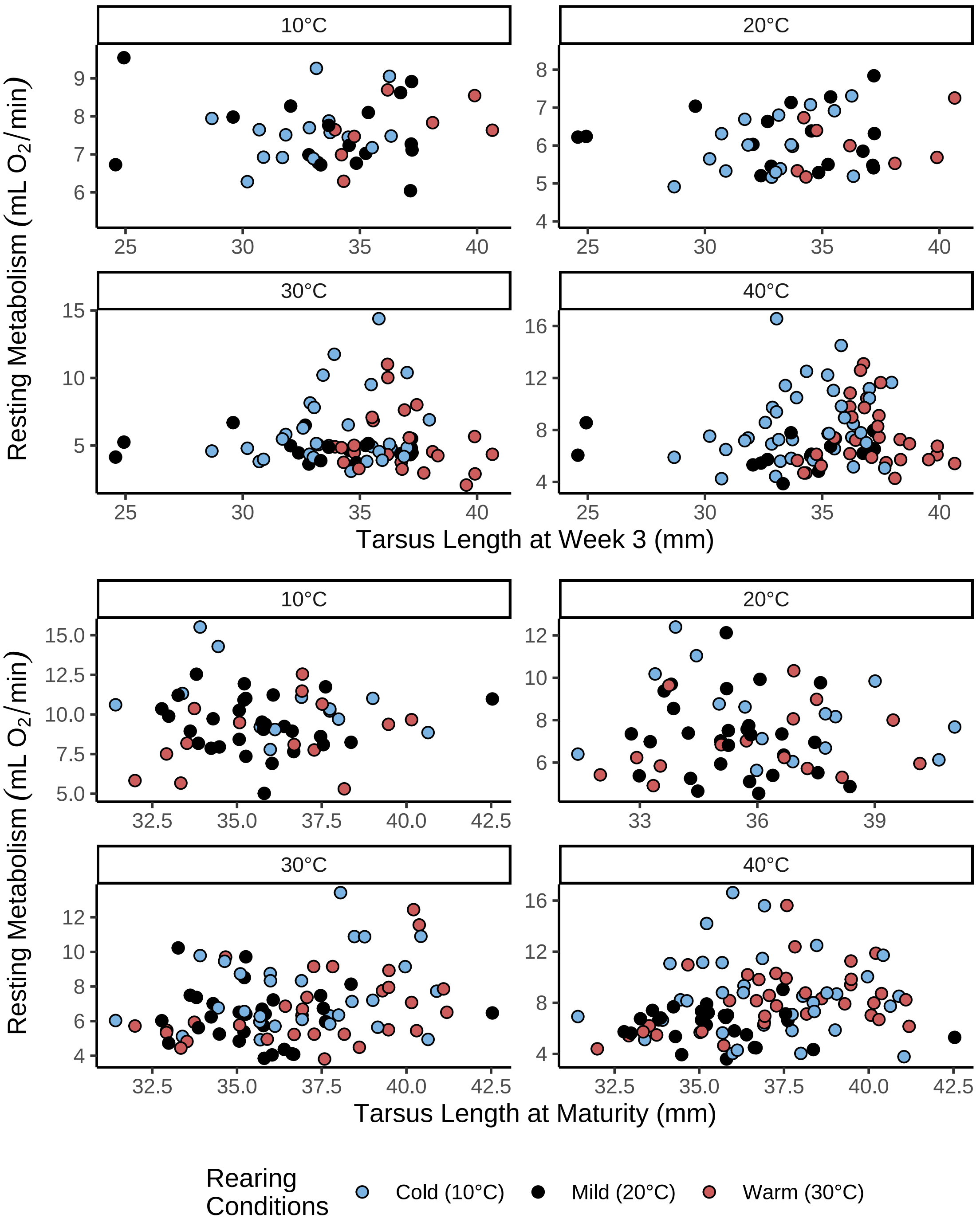

Figure 68: *Resting metabolic rate (mL O_2/min) as a function of tarsus length (mm) in 3 and 8 week old Japanese quail. Resting metabolic rate was measured at four different thermal environments (10°C, 20°C, 30°C and 40°C), with individuals measured in at least two environments. Measured quail were reared in one of three difference rearing conditions: (1) cold (10°C) until at least three weeks of age, (2) mild conditions (20°C) for eight weeks of age, or (3) warmth (30°C) until at least three weeks of age.*

```
showtext_auto(enable = FALSE)
```

```
# Repeating exploratory plots with mass adjusted energy expenditure in place
# of un-adjusted energy expenditure.
```

```
residualVO2Tarsus3Weeks <- ggplot(all %>%
  filter(week == 3) %>%
  mutate(Ta = paste0(Ta, "°C")) %>%
  mutate(
    "VO2" = ((VO2 * 20 / 60) / 1000) * (3600 * 24),
    pretreatment = ifelse(pretreatment == "cold", "Cold (10°C)",
      ifelse(pretreatment == "neutral", "Mild (20°C)",
        "Warm (30°C)"
      )
    )
  ) %>%
  drop_na(mass, tarsusLengthMean, VO2, Ta) %>%
  mutate(
    "residualTarsus" =
      residuals(brm(tarsusLengthMean ~ mass,
        prior = c(
          set_prior("normal(1, 2)", class = "b"),
          set_prior("exponential(0.05)", class = "Intercept")
        ),
        data = .,
        cores = 1, chains = 4,
        seed = 100, family = "gaussian",
        iter = 50000, warmup = 5000, thin = 10,
        control = list(adapt_delta = 0.99, max_treedepth = 16),
        silent = TRUE, refresh = 0,
        file = "./models/_vo2ResidualPlotTarsus3Weeks-Median.Rds"
      ),
      robust = TRUE)[, "Estimate"]
  ) %>%
  drop_na(pretreatment), aes(x = residualTarsus, y = VO2)) +
  facet_wrap(~Ta, scales = "free") +
  geom_point(pch = 21, colour = "black", size = 2, aes(fill = pretreatment)) +
  theme_classic() +
  scale_fill_manual(values = c("#7BB4E3", "black", "#CD5C5C"),
                    name = "Rearing\nConditions") +
  xlab("Residual Tarsus Length at Week 3 (mm)")  +
  ylab(TeX('$Resting~Metabolism~(mL~O_{2}/min)$'))

residualVO2Tarsus8Weeks <- ggplot(all %>%
  filter(week == 8) %>%
  mutate(Ta = paste0(Ta, "°C")) %>%
  mutate(
    "VO2" = ((VO2 * 20 / 60) / 1000) * (3600 * 24),
    pretreatment = ifelse(pretreatment == "cold", "Cold (10°C)",
      ifelse(pretreatment == "neutral", "Mild (20°C)",
        "Warm (30°C)"
      )
    )
  ) %>%
  drop_na(mass, tarsusLengthMean, VO2, Ta) %>%
  mutate(
    "residualTarsus" =
      residuals(brm(tarsusLengthMean ~ mass,
        prior = c(
          set_prior("normal(1, 2)", class = "b"),
          set_prior("exponential(0.05)", class = "Intercept")
        ),
        data = .,
        cores = 1, chains = 4,
        seed = 100, family = "gaussian",
        iter = 50000, warmup = 5000, thin = 10,
        control = list(adapt_delta = 0.99, max_treedepth = 16),
        silent = TRUE, refresh = 0,
        file = "./models/_vo2ResidualPlotTarsus8Weeks-Median.Rds"
      ),
      robust = TRUE)[, "Estimate"]
  ) %>%
```

```
  drop_na(pretreatment), aes(x = residualTarsus, y = VO2)) +
  facet_wrap(~Ta, scales = "free") +
  geom_point(pch = 21, colour = "black", size = 2, aes(fill = pretreatment)) +
  theme_classic() +
  scale_fill_manual(values = c("#7BB4E3", "black", "#CD5C5C"),
                    name = "Rearing\nConditions") +
  xlab("Residual Tarsus Length at Maturity (mm)")  +
  ylab(TeX('$Resting~Metabolism~(mL~O_{2}/min)$'))

showtext_auto()
ggarrange(residualVO2Tarsus3Weeks,
  residualVO2Tarsus8Weeks,
  ncol = 1,
  common.legend = TRUE
)
```

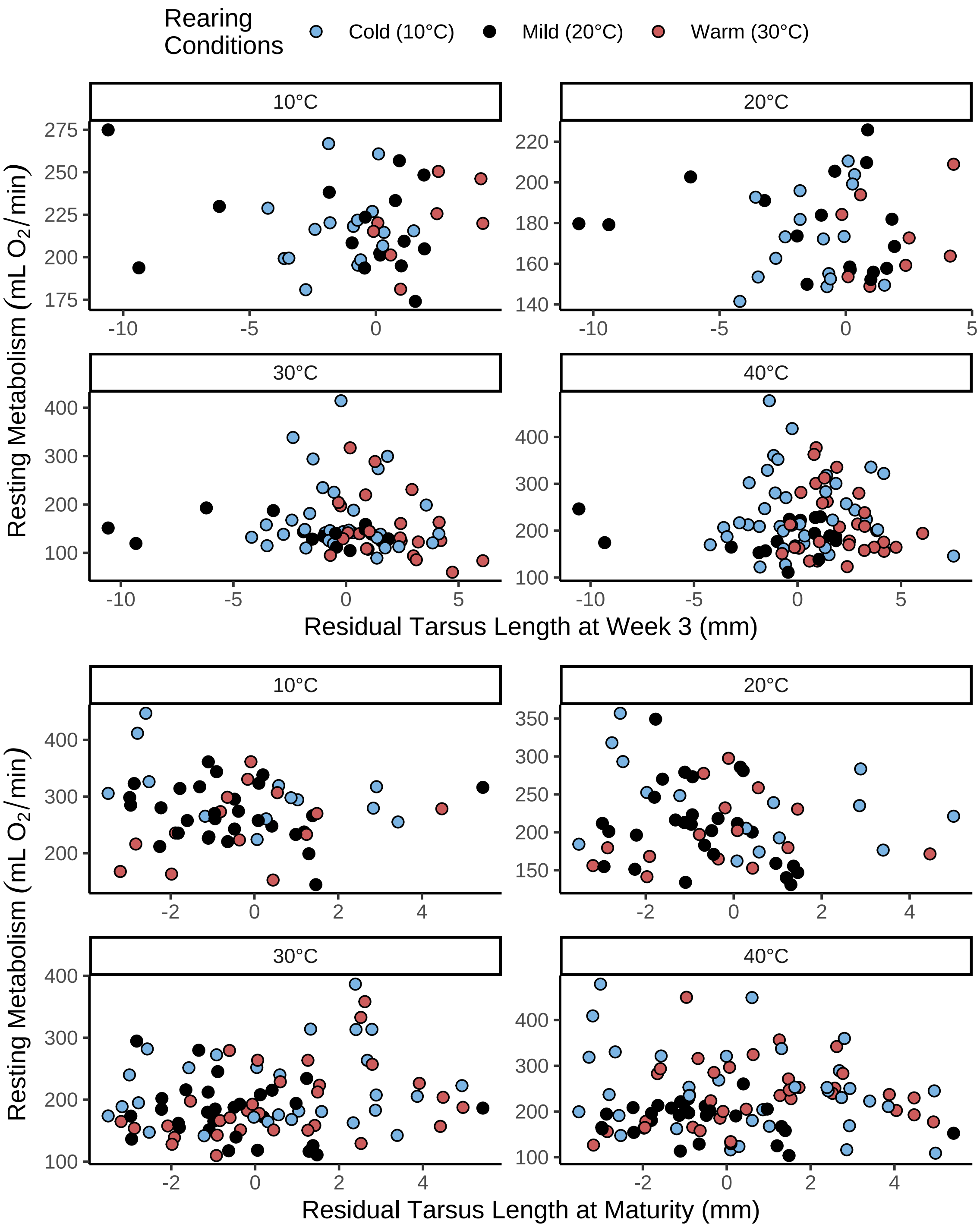

Figure 69: *Resting metabolic rate (mL O_2/min) as a function of relative tarsus length (mm; residuals from a tarsus length [mm] by body mass [g] regression) in 3 and 8 week old Japanese quail. Resting metabolic rate was measured at four different thermal environments (10°C, 20°C, 30°C and 40°C), with individuals measured in at least two environments. Measured quail were reared in one of three difference rearing conditions: (1) cold (10°C) until at least three weeks of age, (2) mild conditions (20°C) for eight weeks of age, or (3) warmth (30°C) until at least three weeks of age.*

```
showtext_auto(enable = FALSE)
```

Finally, viewing raw correlations between resting metabolism and both bill length and relative bill length.

```
bill3Weeks <- ggplot(all %>%
  mutate(
    Ta = paste0(Ta, "°C"),
    pretreatment = ifelse(pretreatment == "cold", "Cold (10°C)",
      ifelse(pretreatment == "neutral", "Mild (20°C)",
        "Warm (30°C)"
      )
    )
  ) %>%
  filter(week == 3) %>%
  drop_na(pretreatment), aes(x = billLengthMean, y = VO2)) +
  facet_wrap(~Ta, scale = "free") +
  geom_point(pch = 21, colour = "black", size = 2, aes(fill = pretreatment)) +
  theme_classic() +
  scale_fill_manual(values = c("#7BB4E3", "black", "#CD5C5C"),
                    name = "Rearing\nConditions") +
  xlab("Bill Length at Week 3 (mm)")  +
  ylab(TeX('$Resting~Metabolism~(mL~O_{2}/min)$')) +
  theme(legend.position = "none")

bill8Weeks <- ggplot(all %>%
  mutate(
    Ta = paste0(Ta, "°C"),
    pretreatment = ifelse(pretreatment == "cold", "Cold (10°C)",
      ifelse(pretreatment == "neutral", "Mild (20°C)",
        "Warm (30°C)"
      )
    )
  ) %>%
  filter(week == 8) %>%
  drop_na(pretreatment), aes(x = billLengthMean, y = VO2)) +
  facet_wrap(~Ta, scales = "free") +
  geom_point(pch = 21, colour = "black", size = 2, aes(fill = pretreatment)) +
  theme_classic() +
  scale_fill_manual(values = c("#7BB4E3", "black", "#CD5C5C"),
                    name = "Rearing\nConditions") +
  xlab("Bill Length at Maturity (mm)")  +
  ylab(TeX('$Resting~Metabolism~(mL~O_{2}/min)$')) +
  theme(legend.position = "bottom")

showtext_auto()
bill3Weeks/bill8Weeks
```

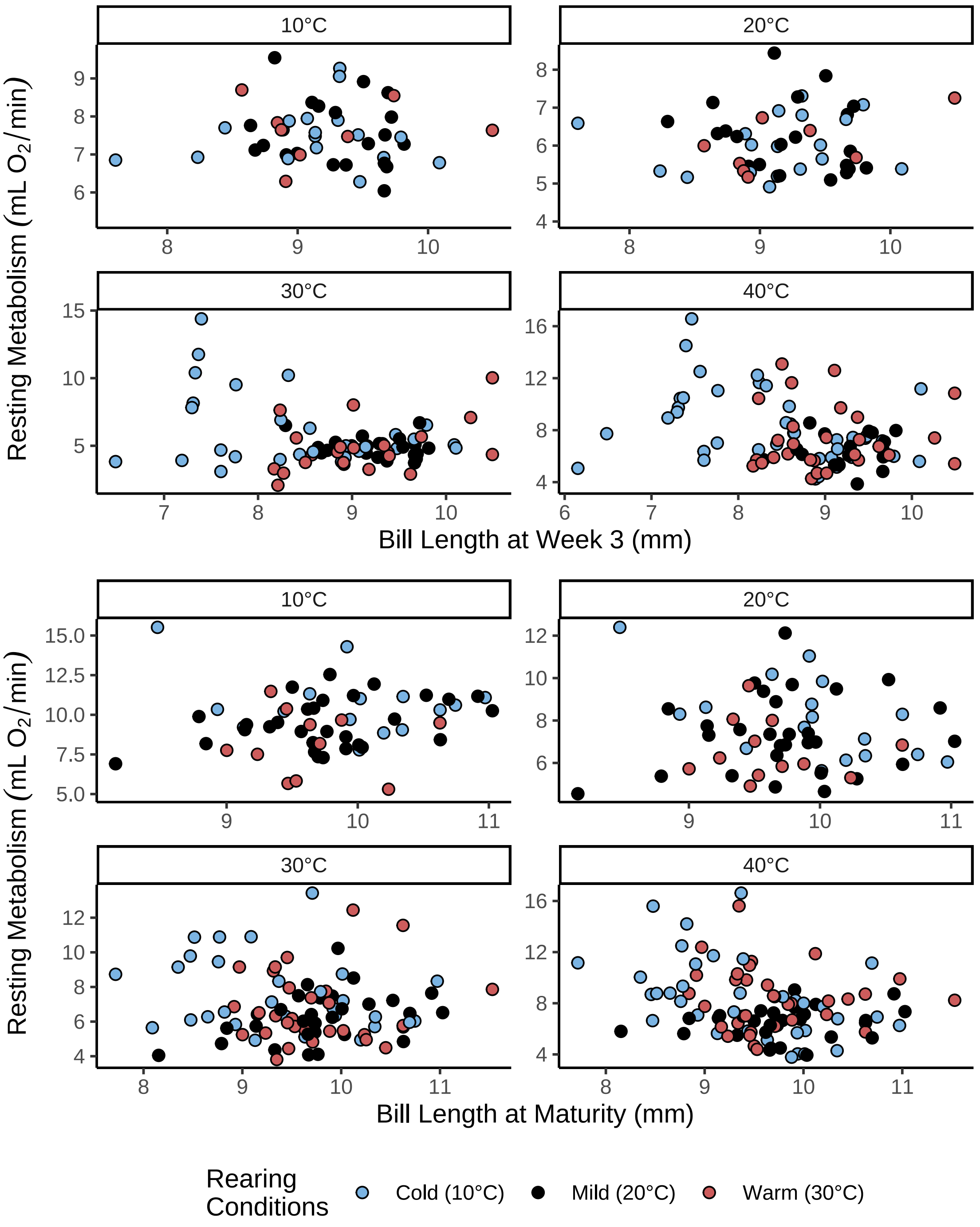

Figure 70: *Resting metabolic rate (mL O_2/min) as a function of bill length (mm) in 3 and 8 week old Japanese quail. Resting metabolic rate was measured at four different thermal environments (10°C, 20°C, 30°C and 40°C), with individuals measured in at least two environments. Measured quail were reared in one of three difference rearing conditions: (1) cold (10°C) until at least three weeks of age, (2) mild conditions (20°C) for eight weeks of age, or (3) warmth (30°C) until at least three weeks of age.*

```
showtext_auto(enable = FALSE)
```

```
# Repeating exploratory plots with mass adjusted energy expenditure in place
# of un-adjusted energy expenditure.
```

```
residualVO2Bill3Weeks <- ggplot(all %>%
  filter(week == 3) %>%
  mutate(Ta = paste0(Ta, "°C")) %>%
  mutate(
    "VO2" = ((VO2 * 20 / 60) / 1000) * (3600 * 24),
    pretreatment = ifelse(pretreatment == "cold", "Cold (10°C)",
      ifelse(pretreatment == "neutral", "Mild (20°C)",
        "Warm (30°C)"
      )
    )
  ) %>%
  drop_na(mass, billLengthMean, VO2, Ta) %>%
  mutate(
    "residualBill" =
      residuals(brm(billLengthMean ~ mass,
        prior = c(
          set_prior("normal(1, 2)", class = "b"),
          set_prior("exponential(0.05)", class = "Intercept")
        ),
        data = .,
        cores = 1, chains = 4,
        seed = 100, family = "gaussian",
        iter = 50000, warmup = 5000, thin = 10,
        control = list(adapt_delta = 0.99, max_treedepth = 16),
        silent = TRUE, refresh = 0,
        file = "./models/_vo2ResidualPlotBill3Weeks-Median.Rds"
      ),
      robust = TRUE)[, "Estimate"]
  ) %>%
  drop_na(pretreatment), aes(x = residualBill, y = VO2)) +
  facet_wrap(~Ta, scales = "free") +
  geom_point(pch = 21, colour = "black", size = 2, aes(fill = pretreatment)) +
  theme_classic() +
  scale_fill_manual(values = c("#7BB4E3", "black", "#CD5C5C"),
                    name = "Rearing\nConditions") +
  xlab("Residual Bill Length at Week 3 (mm)")  +
  ylab(TeX('$Resting~Metabolism~(mL~O_{2}/min)$'))

residualVO2Bill8Weeks <- ggplot(all %>%
  filter(week == 8) %>%
  mutate(Ta = paste0(Ta, "°C")) %>%
  mutate(
    "VO2" = ((VO2 * 20 / 60) / 1000) * (3600 * 24),
    pretreatment = ifelse(pretreatment == "cold", "Cold (10°C)",
      ifelse(pretreatment == "neutral", "Mild (20°C)",
        "Warm (30°C)"
      )
    )
  ) %>%
  drop_na(mass, billLengthMean, VO2, Ta) %>%
  mutate(
    "residualBill" =
      residuals(brm(billLengthMean ~ mass,
        prior = c(
          set_prior("normal(1, 2)", class = "b"),
          set_prior("exponential(0.05)", class = "Intercept")
        ),
        data = .,
        cores = 1, chains = 4,
        seed = 100, family = "gaussian",
        iter = 50000, warmup = 5000, thin = 10,
        control = list(adapt_delta = 0.99, max_treedepth = 16),
        silent = TRUE, refresh = 0,
        file = "./models/_vo2ResidualPlotBill8Weeks-Median.Rds"
      ),
      robust = TRUE)[, "Estimate"]
  ) %>%
```

```
  drop_na(pretreatment), aes(x = residualBill, y = VO2)) +
  facet_wrap(~Ta, scales = "free") +
  geom_point(pch = 21, colour = "black", size = 2, aes(fill = pretreatment)) +
  theme_classic() +
  scale_fill_manual(values = c("#7BB4E3", "black", "#CD5C5C"),
                    name = "Rearing\nConditions") +
  xlab("Residual Bill Length at Maturity (mm)")  +
  ylab(TeX('$Resting~Metabolism~(mL~O_{2}/min)$'))

showtext_auto()
ggarrange(residualVO2Bill3Weeks,
  residualVO2Bill8Weeks,
  ncol = 1,
  common.legend = TRUE
)
```

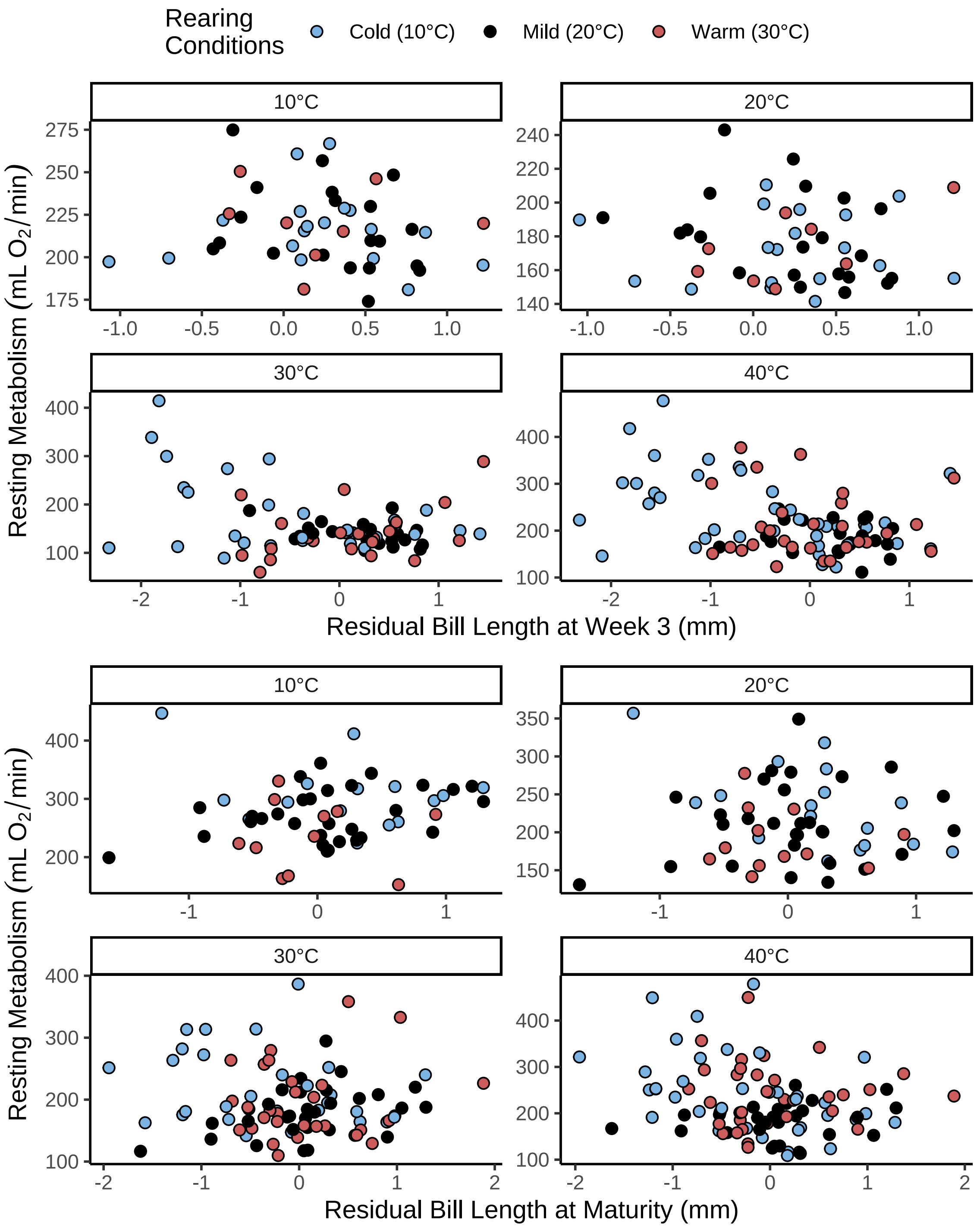

Figure 71: *Resting metabolic rate (mL O_2/min) as a function of relative bill length (mm; residuals from a bill length [mm] by body mass [g] regression) in 3 and 8 week old Japanese quail. Resting metabolic rate was measured at four different thermal environments (10°C, 20°C, 30°C and 40°C), with individuals measured in at least two environments. Measured quail were reared in one of three difference rearing conditions: (1) cold (10°C) until at least three weeks of age, (2) mild conditions (20°C) for eight weeks of age, or (3) warmth (30°C) until at least three weeks of age.*

```
showtext_auto(enable = FALSE)
```

Visualising and quantifying metabolic slopes in the cold (juveniles)

With data collated, we proceed by visualising the rates at which resting metabolism increases in cold (i.e. metabolic slopes), depending on individual morphology (here, body mass, tarsus length, and bill length) and rearing conditions. Visualisation are first conducted using raw resting metabolism data obtained from quail during development (three weeks of age) and at adulthood (eight weeks of age).

```
## Visualising metabolic slopes by tarsus size at
# temperatures below thermoneutrality

rSlopesMass <- all %>%
  filter(week %in% c(3, 8) & Ta < 35) %>%
  mutate("Age" = paste0("Age = ", week, " weeks")) %>%
  drop_na(Ta, week, mass, VO2, pretreatment) %>%
  group_by(Age) %>%
  mutate(
    massScaledA = (mass - min(mass)) /
      (max(mass) - min(mass)),
    massScaledB = (mass - mean(mass)) / sd(mass)
  ) %>%
  mutate(massScaledA = exp(massScaledA)) %>%
  ungroup() %>%
  ggplot(aes(
    x = Ta, y = VO2,
    colour = massScaledB,
    group = ring
  )) +
  facet_wrap(~Age, scales = "free", ncol = 1) +
  geom_smooth(
    method = "lm", linetype = "solid", alpha = 0.5, se = FALSE,
    aes(linewidth = massScaledA)
  ) +
  scale_colour_gradient(
    low = "black", high = "grey99", name = "Relative\nBody\nMass",
    breaks = c(-2, 0, 2),
    labels = c("Mean -\n2 SDs", "Mean", "Mean +\n2 SDs")
  ) +
  scale_linewidth_continuous(range = c(0.1, 2), guide = "none") +
  xlab("Ambient Temperature (°C)") +
  ylab(
   TeX('$\\overset{Resting~Energy~Expenditure}{(mL~O_{2}/min)}$')
   ) +
  theme_classic() +
  theme(
    axis.title = element_text(family = "Noto Sans"),
    legend.title = element_text(family = "Noto Sans"),
    legend.position = "bottom"
  )

rSlopesTarsus <- all %>%
  filter(week %in% c(3, 8) & Ta < 35) %>%
  mutate("Age" = paste0("Age = ", week, " weeks")) %>%
  drop_na(Ta, week, tarsusLengthMean, VO2, pretreatment) %>%
  group_by(Age) %>%
  mutate(
    tarsusScaledA = (tarsusLengthMean - min(tarsusLengthMean)) /
      (max(tarsusLengthMean) - min(tarsusLengthMean)),
    tarsusScaledB = (tarsusLengthMean - mean(tarsusLengthMean)) /
      sd(tarsusLengthMean)
  ) %>%
  mutate(tarsusScaledA = exp(tarsusScaledA)) %>%
  ungroup() %>%
  ggplot(aes(
```

```
    x = Ta, y = VO2,
    colour = tarsusScaledB,
    group = ring
  )) +
  facet_wrap(~Age, scales = "free") +
  geom_smooth(
    method = "lm", linetype = "solid", alpha = 0.5, se = FALSE,
    aes(linewidth = tarsusScaledA)
  ) +
  scale_colour_gradient(
    low = "black", high = "grey99",
    name = "Relative\nTarsus Length",
    breaks = c(-2, -1, 0, 1, 2),
    labels = c("Mean - 2 SDs", "Mean - 1 SD", "Mean", "Mean + 1 SD", "Mean + 2 SDs")
  ) +
  scale_linewidth_continuous(range = c(0.1, 2), guide = "none") +
  xlab("Ambient Temperature (°C)") +
  ylab(
   TeX('$\\overset{Resting~Energy~Expenditure}{(mL~O_{2}/min)}$')
   ) +
  theme_classic() +
  theme(
    axis.title = element_text(family = "Noto Sans"),
    legend.title = element_text(family = "Noto Sans")
  )

rSlopesBill <- all %>%
  filter(week %in% c(3, 8) & Ta < 35) %>%
  mutate("Age" = paste0("Age = ", week, " weeks")) %>%
  drop_na(Ta, week, billLengthMean, VO2, pretreatment) %>%
  group_by(Age) %>%
  mutate(
    billScaledA = (billLengthMean - min(billLengthMean)) /
      (max(billLengthMean) - min(billLengthMean)),
    billScaledB = (billLengthMean - mean(billLengthMean)) /
      sd(billLengthMean)
  ) %>%
  mutate(billScaledA = exp(billScaledA)) %>%
  ungroup() %>%
  ggplot(aes(
    x = Ta, y = VO2,
    colour = billScaledB,
    group = ring
  )) +
  facet_wrap(~Age, scales = "free") +
  geom_smooth(
    method = "lm", linetype = "solid", alpha = 0.5, se = FALSE,
    aes(linewidth = billScaledA)
  ) +
  scale_colour_gradient(
    low = "black", high = "grey99",
    name = "Relative\nBill Length",
    breaks = c(-2, -1, 0, 1, 2),
    labels = c("Mean - 2 SDs", "Mean - 1 SD", "Mean", "Mean + 1 SD", "Mean + 2 SDs")
  ) +
  scale_linewidth_continuous(range = c(0.1, 2), guide = "none") +
  xlab("Ambient Temperature (°C)") +
  ylab(
   TeX('$\\overset{Resting~Energy~Expenditure}{(mL~O_{2}/min)}$')
   ) +
  theme_classic() +
  theme(
    axis.title = element_text(family = "Noto Sans"),
    legend.title = element_text(family = "Noto Sans")
  )

rSlopesPlot <- rSlopesMass | (rSlopesTarsus/rSlopesBill)
```

```
showtext_auto()
print(rSlopesPlot)
```

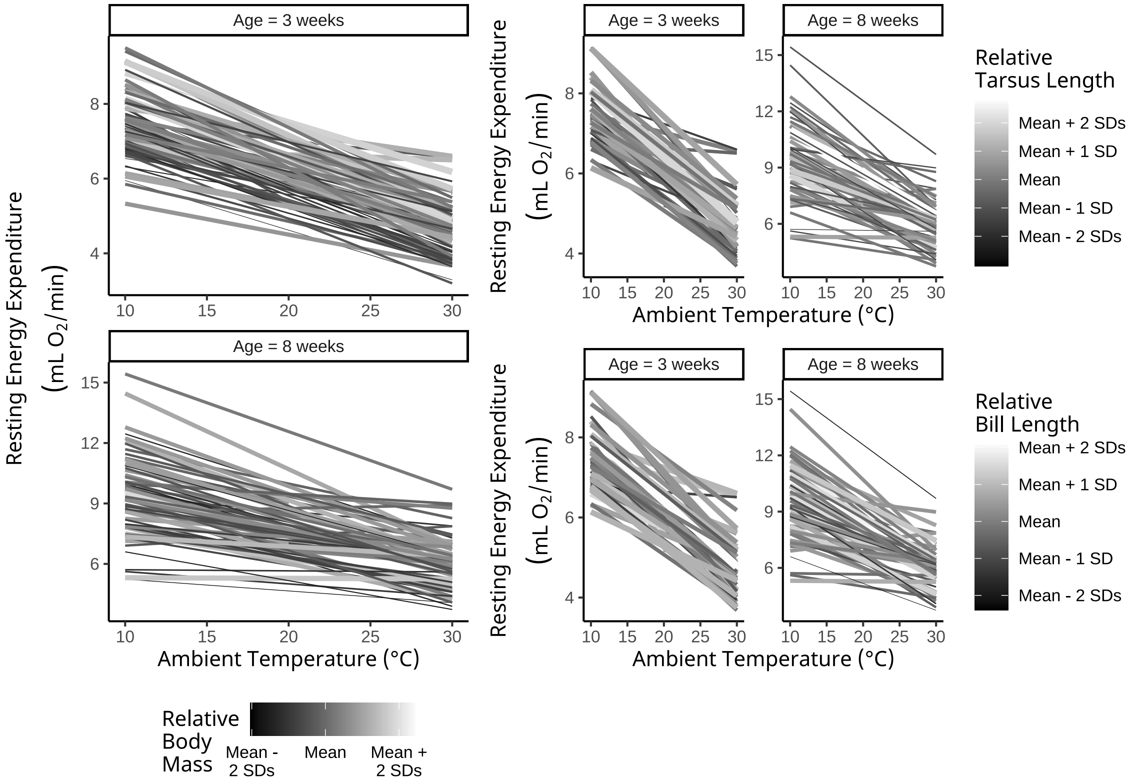

Figure 72: *Resting energy expenditure of Japanese quail at various ambient temperatures (°C) and according to body mass (g), tarsus lengths (mm), and bill length (mm). Each line represents energy expenditure for one individual. Line size is scaled by relative body mass or appendage length for each plot respectively, with thicker lines representing relatively large phenotype and thinner lines representing relative small phenotype. Lines are scaled per age of measurement.*

```
showtext_auto(enable = FALSE)

ggsave("./plots/resistanceSlopes.jpg",
  rSlopesPlot,
  dpi = 800, height = 7, width = 9
)
```

Tentative correlations between tarsus or bill length and metabolic slopes are further investigated by relativising each measure with respect to body mass (here, as residuals from simple Bayesian linear models with broad priors).

```
rSlopesResidualTarsus <- all %>%
  filter(week %in% c(3, 8) & Ta < 35) %>%
  drop_na(tarsusLengthMean, mass, week) %>%
  mutate(
    "residualTarsus" =
      residuals(
        brm(tarsusLengthMean ~ mass + week,
          prior = c(
            set_prior("normal(1, 2)", class = "b"),
            set_prior("exponential(0.05)", class = "Intercept")
          ),
```

```
          data = .,
          cores = 4, chains = 4,
          seed = 100, family = "gaussian",
          iter = 50000, warmup = 5000, thin = 10,
          control = list(adapt_delta = 0.99, max_treedepth = 16),
          silent = TRUE, refresh = 0,
          file = "./models/_residualTarsusPlotModel-Median.Rds"
        ),
        robust = TRUE
      )[, "Estimate"]
  ) %>%
  mutate("tarsusBin" = ifelse(residualTarsus < 0,
    "Below Average", "Above Average"
  )) %>%
  mutate(
    "Age" = paste0("Age = ", week, " weeks")
  ) %>%
  drop_na(tarsusBin) %>%
  ggplot(aes(
    x = Ta, y = VO2,
    colour = tarsusBin,
    group = ring
  )) +
  facet_wrap(~Age, scales = "free") +
  geom_smooth(method = "lm", linetype = "solid", alpha = 0.5, se = FALSE) +
  scale_colour_manual(
    values = c("grey80", "black"),
    name = "Relative\nTarsus Length"
  ) +
  xlab("Ambient Temperature (°C)") +
  ylab(TeX("$\\overset{Resting~Energy~Expenditure}{(mL~O_{2}/min)}$")) +
  theme_classic() +
  theme(
    axis.title = element_text(family = "Noto Sans", size = 14),
    legend.title = element_text(family = "Noto Sans", size = 14)
  )

rSlopesResidualBill <- all %>%
  filter(week %in% c(3, 8) & Ta < 35) %>%
  drop_na(billLengthMean, mass, week) %>%
  mutate(
    "residualBill" =
      residuals(
        brm(billLengthMean ~ mass + week,
          prior = c(
            set_prior("normal(1, 2)", class = "b"),
            set_prior("exponential(0.05)", class = "Intercept")
          ),
          data = .,
          cores = 4, chains = 4,
          seed = 100, family = "gaussian",
          iter = 50000, warmup = 5000, thin = 10,
          control = list(adapt_delta = 0.99, max_treedepth = 16),
          silent = TRUE, refresh = 0,
          file = "./models/_residualBillPlotModel-Median.Rds"
        ),
        robust = TRUE
      )[, "Estimate"]
  ) %>%
  mutate("billBin" = ifelse(residualBill < 0,
    "Below Average", "Above Average"
  )) %>%
  mutate(
    "Age" = paste0("Age = ", week, " weeks")
  ) %>%
  filter(!is.na(billBin)) %>%
  ggplot(aes(
```

```
    x = Ta, y = VO2,
    colour = billBin,
    group = ring
  )) +
  facet_wrap(~Age, scales = "free") +
  geom_smooth(method = "lm", linetype = "solid", alpha = 0.5, se = FALSE) +
  scale_colour_manual(
    values = c("grey80", "black"),
    name = "Relative\nBill Length"
  ) +
  xlab("Ambient Temperature (°C)") +
  ylab(TeX("$\\overset{Resting~Energy~Expenditure}{(mL~O_{2}/min)}$")) +
  theme_classic() +
  theme(
    axis.title = element_text(family = "Noto Sans", size = 14),
    legend.title = element_text(family = "Noto Sans", size = 14)
  )

rSlopesPlotResiduals <- rSlopesResidualTarsus/rSlopesResidualBill

showtext_auto()
print(rSlopesPlotResiduals)
```

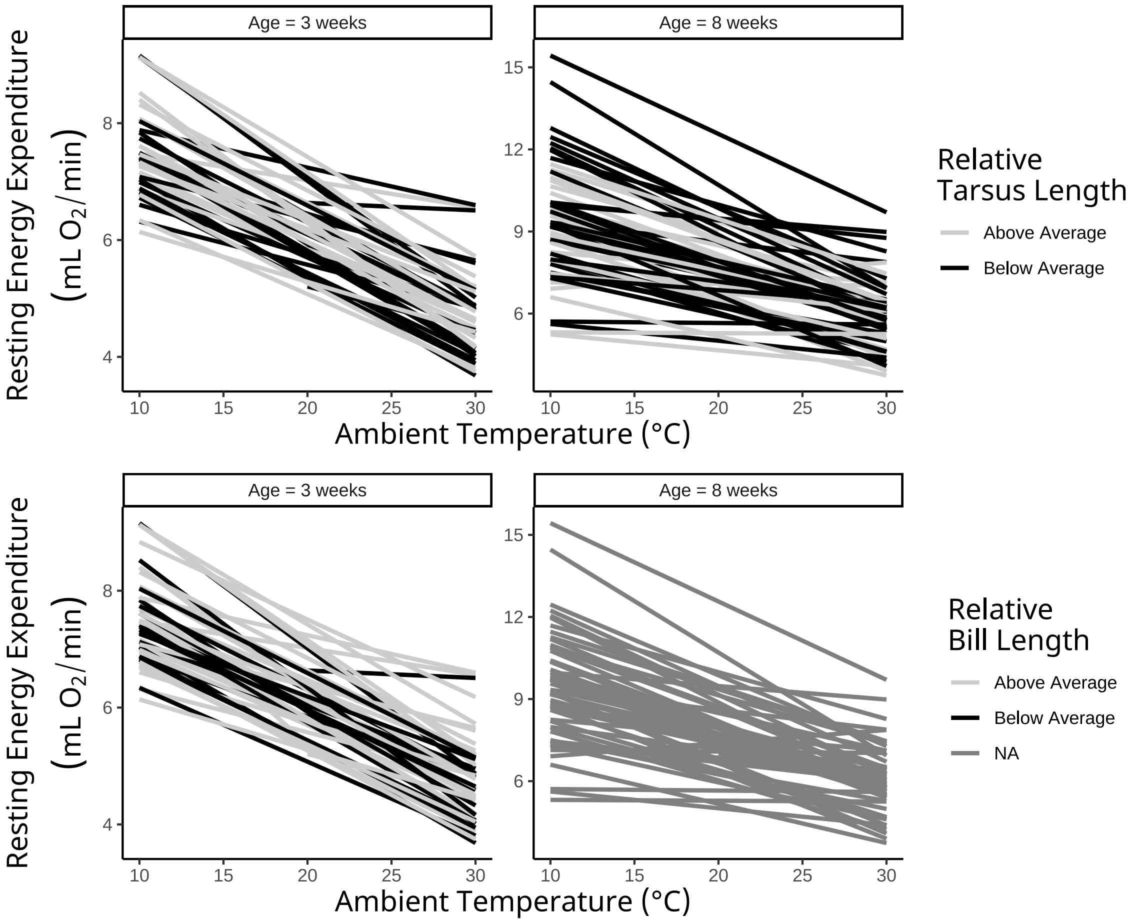

Figure 73: *Resting energy expenditure of Japanese quail at various ambient temperatures (°C) and according to relative tarsus length or bill length (mm; residuals from a regression against body mass [g]). Each line represents energy expenditure for one individual and lines are now coloured according to whether an individual's relative appendage length fell above or below the average for its age.*

```
showtext_auto(enable = FALSE)

ggsave("./plots/resistanceSlopesResiduals.jpg",
  rSlopesPlotResiduals,
  dpi = 800, height = 7, width = 9
)
```

Next, we formally quantify metabolic slopes in response to our cold challenge per individual and during development (3 weeks of age). To do so, we model resting metabolism as a function of air temperatures between 10°C - 30°C while allowing slopes to vary by individual. However, given that: **(1)** morphology can influence both intercepts and slopes of temperature-metabolism relationships, and **(2)** intercepts and slopes may be inter-related (thus complicating analyses), wefirst relativised resting metabolism measurements as fold-changes from those observed at thermoneutrality (30°C; Persson et al, 2024) per individual; thus, resting metabolism at thermoneutrality for all individuals was rooted at 1. To accommodate this rooting, we then set thermoneutrality (30°C) as our model intercept and proceeded to estimating slopes of temperature-metabolism relationships alone. Since variance in metabolism measurements can vary greatly by measurement temperature, we further allowed our error structure to vary by categorical ambient temperature while assuming an increase in error at lower ambient temperatures. Formulae for our model were therefore as follows:

$$Relative\ VO_{2ij} \sim 1 + (\beta_1 + v_j) \cdot Ta_{ij} + \epsilon_{ij}$$

where:

$$Relative\ VO_{2ij} = VO_{2ij}/Thermoneutral\ VO_{2j}$$
$$v_j \sim \mathcal{N}(0, V_v)$$
$$\epsilon_{ij} \sim S\mathcal{N}(0, Ve)$$
$$Ve \sim e^{(\tau_0 + \tau_1 \cdot Ta_{10°C;ij})}$$

and where i represents an observation, j represents an individual, v represents an individual's deviance from the population metabolic slope (β_1), which is normally-distributed around 0 with a standard deviation of V_v, ϵ represents the model error term with standard deviation *Ve*, τ_0 indicates the natural-log transformed standard deviation of ϵ for measures at 20°C, $Ta_{10°C;\ ij}$ indicates whether an observation was made at 10°C (true $= 1$, false $= 0$) and τ_1 indicates the natural-log transformed adjustment to ϵ made for these observations. Metabolic slopes are then calculated per individual as follows:

$$Metabolic\ Slope_j = \beta_1 + v_j$$

Priors for β_1 were informed by Saarela and Heldmaier (1987) and set as skew-normal with an ξ value of -0.018, ω value of 0.02, and α value of -5 (thus setting the mean for our prior at approximately that reported by Saarela and Heldmaier, 1987). Priors for other variables were weak, with that for V_v set as exponential (*Exp*) with a λ of 10, and those for τ_0, τ_1, and τ_2 set as normal with means of -1.5, 0, and -5 respectively, and standard deviations of 1, 0.25, and 1 respectively.

Only individuals for whom resting metabolism was measured at the lower critical temperature (~30°C) and at least one ambient temperature below (10°C or 20°) were used in this model and subsequent analyses. Below, a linear relationship between ambient temperature and resting metabolism is first visually confirmed.

```
birdVector <- merge(
  all %>%
    drop_na(VO2) %>%
    filter(week == 3 & Ta == 30) %>%
    select(ring) %>%
```

```
    distinct(),
  all %>%
    drop_na(VO2) %>%
    filter(week == 3 & Ta %in% c(10, 20)) %>%
    select(ring) %>%
    distinct(),
  by = "ring", all = FALSE
) %>% pull(ring)

modData <- subset(all, week == 3 & Ta < 35 & ring %in% birdVector)

# Checking correlation between ambient temperature
# and metabolic rate visually.

modData %>%
  ggplot(aes(x = Ta, y = VO2)) +
  geom_point(
    pch = 21, size = 1.5, alpha = 0.5, colour = "black", fill = "grey50",
    position = position_jitter(width = 0.2)
  ) +
  stat_summary(geom = "errorbar", fun.data = "mean_se",
               width = 0.3, colour = "black") +
  stat_summary(
    geom = "point", fun = "mean", size = 4, pch = 21,
    colour = "black",
    fill = "grey50"
  ) +
  geom_smooth(method = "lm", colour = "black", linetype = "dashed", se = FALSE) +
  xlab("Ambient Temperature (°C)") +
  ylab(TeX('$\\overset{Resting~Energy~Expenditure}{(mL~O_{2}/min)}$')) +
  theme_classic()
```

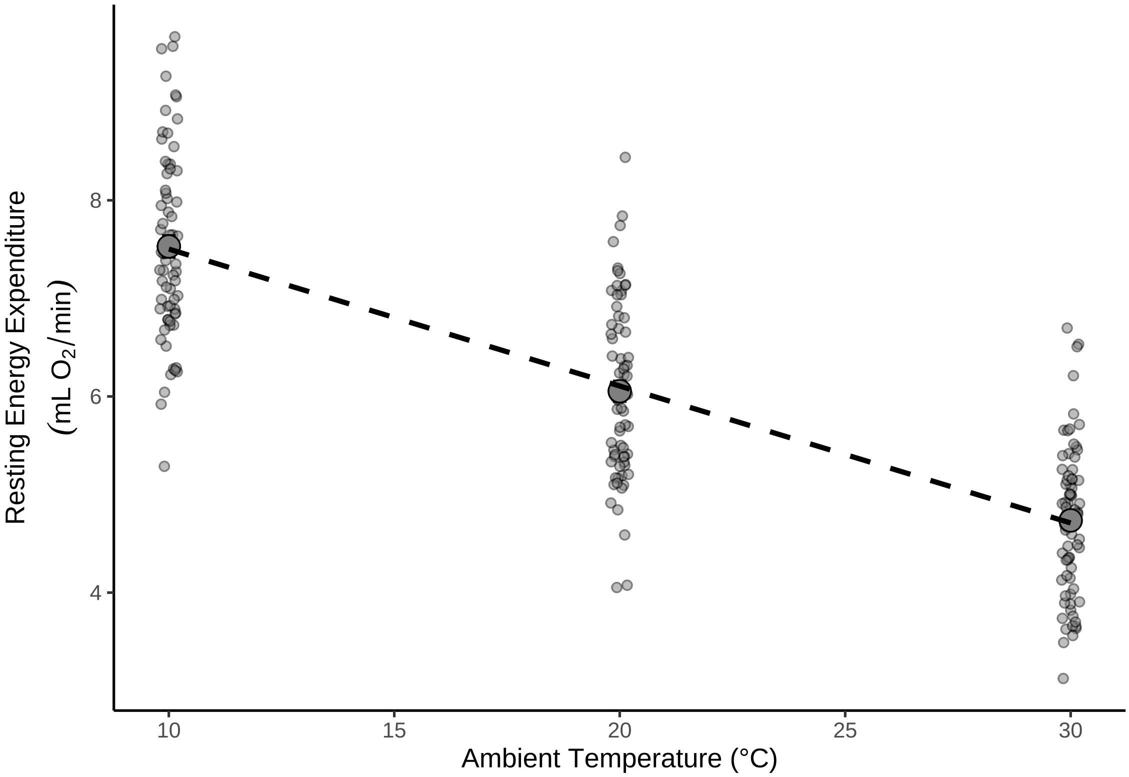

Figure 74: *Relationship between resting metabolism and ambient temperature in three week old Japanese quail. Large dots represent means across quail and small dots represent individual measurements. Errorbars around large dots represent standard errors and the dotted line represents the line of best fit as estimated by the R package ggplot2 (Wickham, 2011).*

To confirm suitability of our priors, we visualise how well predictions from our priors alone capture distributions of resting metabolism measurements in our data.

```
# Setting 30°C as 0°C and checking suitability of priors.

slopes3Weeks_ppCheck <- brm(
  data = modData %>%
    dplyr::select(ring, Ta, VO2) %>%
    pivot_wider(id_cols = "ring", names_from = Ta, values_from = VO2) %>%
    mutate(`10` = `10`/`30`,
           `20` = `20`/`30`,
           `30` = 1) %>%
    pivot_longer(-c("ring"), names_to = "Ta", values_to = "VO2") %>%
    mutate(Ta = as.integer(Ta)) %>%
    mutate(Ta = Ta - 30) %>%
    filter(Ta < 0) %>%
    mutate("discreteTa" = ifelse(Ta == -20, "A", "B")) %>%
    drop_na(),
  family = "gaussian",
  bf(VO2 ~ 1 + B,
     B ~ 0 + Ta + (0 + Ta | ring),
     sigma ~ discreteTa,
     nl = TRUE),
  prior = c(
    set_prior("skew_normal(-0.018, 0.02, -2.5)", nlpar = "B",
              class = "b", coef = "Ta"),
    set_prior("exponential(2.5)", nlpar = "B", class = "sd",
              coef = "Ta", group = "ring"),
```

```
    set_prior("skew_normal(-0.2, 1, -5)", dpar = "sigma",
              class = "Intercept"),
    set_prior("normal(0.25, 0.25)", dpar = "sigma",
              class = "b")
  ),
  iter = 50000, warmup = 10000, thin = 10,
  chains = 4, cores = 4,
  control = list(adapt_delta = .96),
  silent = TRUE, refresh = 0,
  sample_prior = "only",
  file = "./models/_threeWeekSlopesPPCheck.Rds"
)

pp_check2(slopes3Weeks_ppCheck,
          xlab = "Fold Metabolism at 30°C") +
  xlim(c(-10, 10))
```

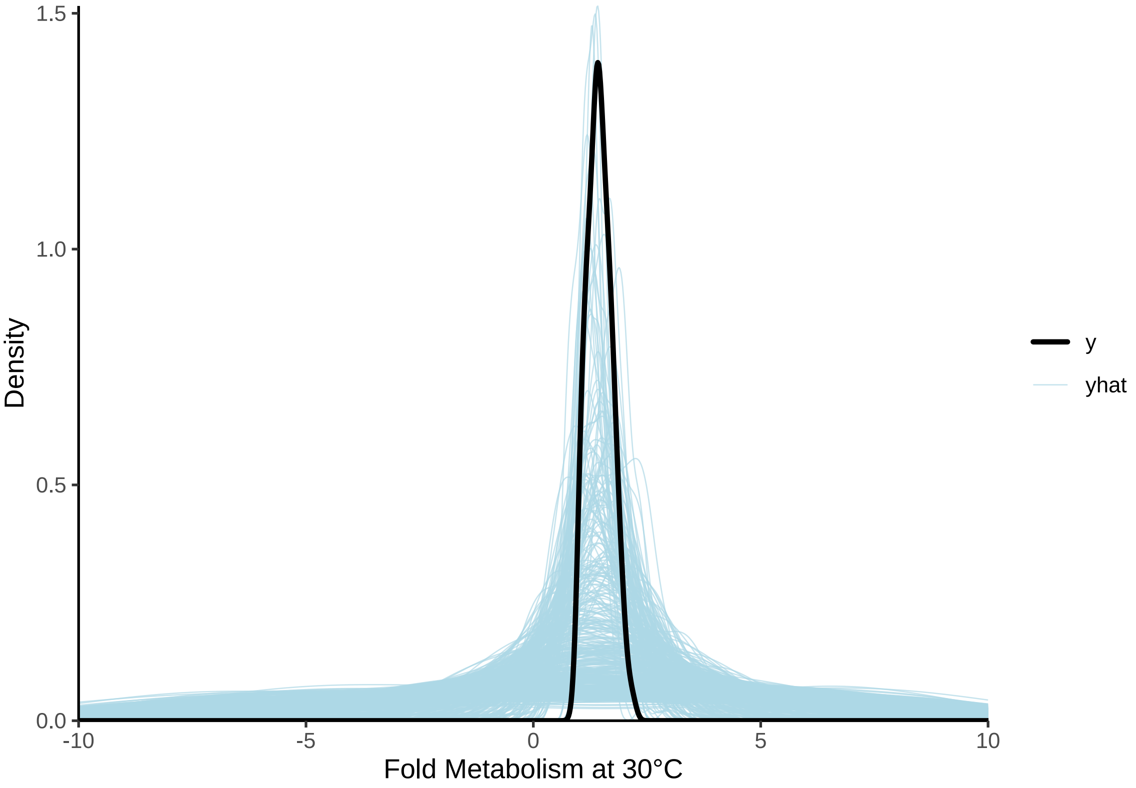

Figure 75: *Prior predictive check for a Bayesian linear mixed-effects model predicting relative resting metabolism (fold metabolism at thermoneutrality) of three week old Japanese quail by ambient temperature (raw ambient temperatures ranging from 10°C - 30°C). Light blue lines represent densities of resting metabolism values as predicted by model priors alone. The dark blue line represents the true density of resting metabolism values. Clear overlap between the dark blue and light blue lines indicates that priors are suitable.*

Priors are wide but capture our peak density well. We proceed to constructing our full model accordingly and evaluating chain mixing and intra-chain autocorrelation.

```
slopes3Weeks <- brm(
  data = modData %>%
    select(ring, Ta, VO2) %>%
    pivot_wider(id_cols = "ring", names_from = Ta, values_from = VO2) %>%
    mutate(`10` = `10`/`30`,
           `20` = `20`/`30`,
```

```
           `30` = 1) %>%
    pivot_longer(-c("ring"), names_to = "Ta", values_to = "VO2") %>%
    mutate(Ta = as.integer(Ta)) %>%
    mutate(Ta = Ta - 30) %>%
    filter(Ta < 0) %>%
    mutate("discreteTa" = ifelse(Ta == -20, "A", "B")),
  family = "gaussian",
  bf(VO2 ~ 1 + B,
     B ~ 0 + Ta + (0 + Ta | ring),
     sigma ~ discreteTa,
     nl = TRUE),
  prior = c(
    set_prior("skew_normal(-0.018, 0.02, -2.5)", nlpar = "B",
              class = "b", coef = "Ta"),
    set_prior("exponential(2.5)", nlpar = "B", class = "sd",
              coef = "Ta", group = "ring"),
    set_prior("skew_normal(-0.2, 1, -5)", dpar = "sigma",
              class = "Intercept"),
    set_prior("normal(0.25, 0.25)", dpar = "sigma",
              class = "b")
  ),
  iter = 100000, warmup = 50000, thin = 10,
  chains = 4, cores = 4,
  control = list(adapt_delta = .98, max_treedepth = 14),
  silent = TRUE, refresh = 0,
  file = "./models/_threeWeekSlopes.Rds"
)

# Checking Gelman-Rubin statistic and ratio of effective sample sizes to sample sizes.

ggarrange(
  mcmc_rhat(rhat(slopes3Weeks)[names(rhat(slopes3Weeks)) != "lp__"]) +
    theme(legend.position = "none") +
    xlab(
      TeX('$\\hat{R}$')
    ),
  mcmc_neff(
    neff_ratio(slopes3Weeks)[names(neff_ratio(slopes3Weeks)) != "lp__"],
    size = 2
  ) +
    theme(legend.position = "none") +
    xlab(
      TeX('$N_{eff}/N~Ratio$')
    )
)
```

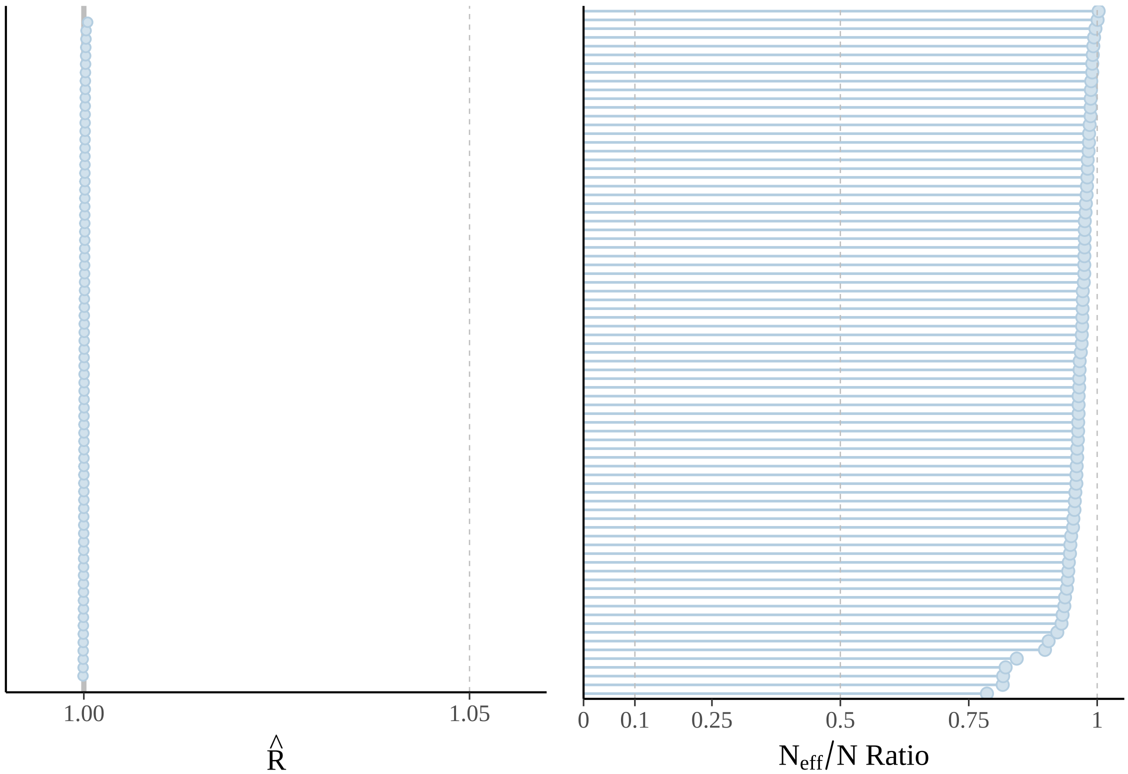

Figure 76: *Gelman-Rubin statistics ($\hat{R}$s) and ratio of effective sample sizes to sample sizes for coefficients from a Bayesian, linear mixed-effects model predicting relative resting metabolism (fold metabolism at thermoneutrality) of three week old Japanese quail across 10°C - 30°C thermal environemnts.*

Chains appear well mixed (Gelman-Rubin statistics [$\hat{R}$] laying near 1) and high effective sample size to sample size ratios (>0.75) indicate minimal chain autocorrelation. We proceed by visualising posterior densities and predictions, alongside estimating fit (here, using a Bayesian "R^2").

```
pp_check2(slopes3Weeks,
          xlab = paste0("Relative Resting Energy Expenditure\n",
          "(Fold Metabolism at 30°C)")
)
```

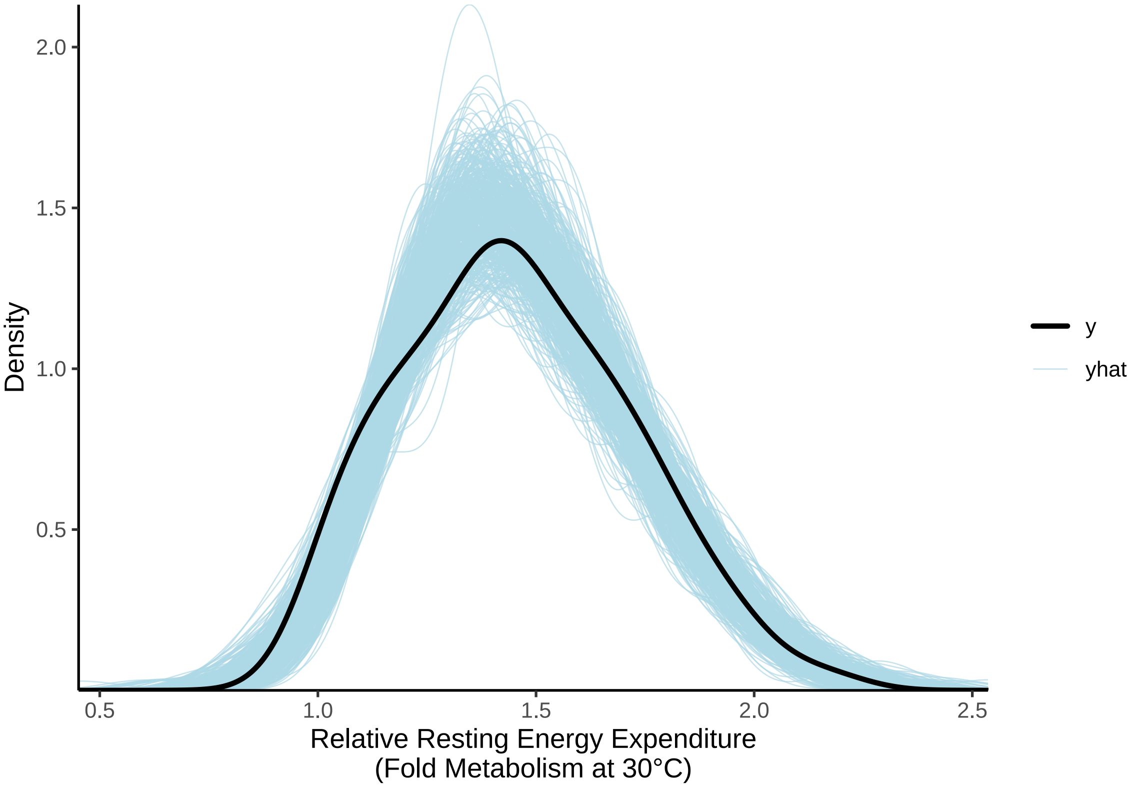

Figure 77: *Posterior predictive check for a Bayesian linear, mixed-effects model predicting relative resting metabolism (fold resting metabolism at thermoneutrality) of three week old Japanese quail by ambient temperature (raw ambient temperatures ranging from 10°C - 30°C). Light blue lines represent densities of resting metabolism values as drawn from model posteriors. The dark blue line represents the true density of resting metabolism values. Clear overlap between the dark blue and light blue lines indicates strong model fit.*

```
# Good. Checking distributions of metabolic slope estimates per bird
```

```
as.data.frame(slopes3Weeks) %>%
  select(starts_with("r_")) %>%
  pivot_longer(everything(), names_to = "ring",
               values_to = "slope") %>%
  mutate("ring" =
           gsub("\\,.*", "",
                gsub(".*\\[", "", ring)
                )
  ) %>%
  mutate(slope = fixef(slopes3Weeks)[1,"Estimate"] +
           slope) %>%
  ggplot(aes(x = slope)) +
  facet_wrap(~ring) +
  geom_density() +
  xlab("Metabolic Slope") +
  ylab("Density") +
  theme_classic() +
  theme(axis.text.x = element_blank(),
        axis.ticks.x = element_blank(),
        axis.text.y = element_blank(),
        axis.ticks.y = element_blank()
        )
```

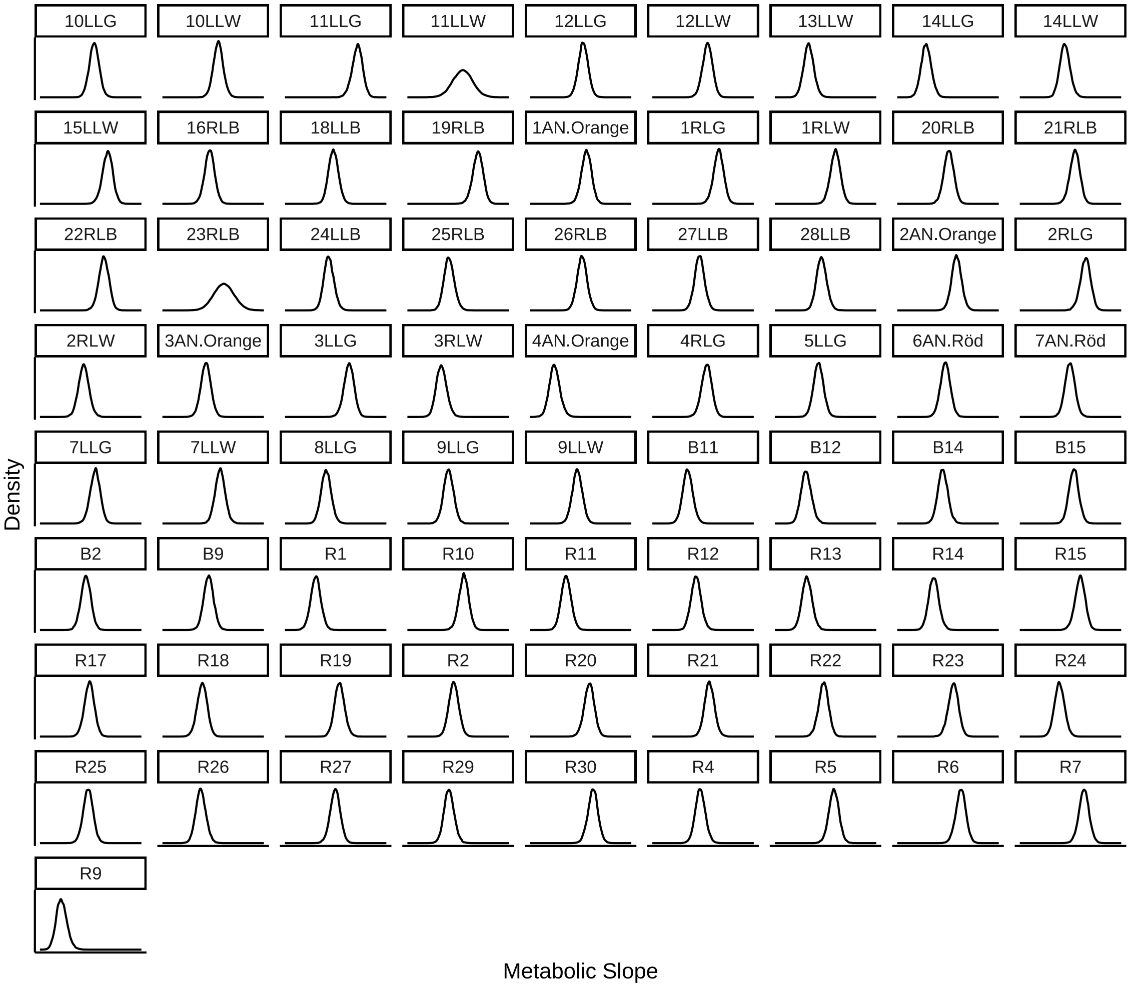

Figure 78: *Posterior densities of metabolic slopes, per individual, from a Bayesian linear mixed-effects model predicting relative resting metabolism (fold resting metabolism at thermoneutrality) of three week old Japanese quail by ambient temperature (raw ambient temperatures ranging from 10°C - 30°C).*

```
# Reasonably normal and no multimodality. Using medians for measure of
# central tendency.

caption <- paste0("R\\textsuperscript{2} for Bayesian linear, mixed-effects",
                  " model predicting relative resting metabolism (fold ",
                  "resting metabolism at thermoneutrality) ",
                  "of three week old Japanese quail by ambient ",
                  "temperature (raw ambient temperatures ranging from ",
                  "10°C - ",
                  "30°C)."
)

brms::bayes_R2(slopes3Weeks,
               robust = TRUE, ndraws = 1000) %>%
  as.data.frame() %>%
  remove_rownames() %>%
  mutate(Estimate = round(Estimate, digits = 4),
         Est.Error = round(Est.Error, digits = 4),
         Q2.5 = round(Q2.5, digits = 4),
         Q97.5 = round(Q97.5, digits = 4)
         ) %>%
  rename(
    "R\\textsuperscript{2}" = Estimate, "Standard Error" = Est.Error,
```

```
    `2.5\\% CI` = `Q2.5`, `97.5\\% CI` = `Q97.5`
  ) %>%
  kbl(.,
    longtable = T, booktabs = T, format = "latex",
    caption = caption, escape = FALSE,
  ) %>%
  kable_styling(latex_options = "striped")
```

Table 33: *R^2 for Bayesian linear, mixed-effects model predicting relative resting metabolism (fold resting metabolism at thermoneutrality) of three week old Japanese quail by ambient temperature (raw ambient temperatures ranging from 10°C - 30°C).*

R^2	Standard Error	2.5% CI	97.5% CI
0.7822	0.03	0.697	0.822

```
# Good and R2 reasonably high. Checking residuals.
```

```
p1 <- slopes3Weeks$data %>%
  mutate("Residuals" = residuals(slopes3Weeks,
    method = "posterior_predict",
    type = "pearson",
    robust = TRUE
  )[, "Estimate"]) %>%
  ggplot(aes(x = Residuals)) +
  geom_density(colour = "black", fill = "grey50", adjust = 1) +
  theme_classic() +
  xlab("Pearson Residuals") +
  ylab("Density")

p2 <- as.data.frame(slopes3Weeks) %>%
  select("Ta" = b_B_Ta, "ring" = sd_ring__B_Ta) %>%
  pivot_longer(everything(),
    names_to = "var",
    values_to = "value"
  ) %>%
  ggplot(aes(sample = value)) +
  facet_wrap(~var, scales = "free") +
  stat_qq(colour = "grey50") +
  stat_qq_line() +
  xlab("Theoretical") +
  ylab("Sample") +
  theme_classic()

p3 <- slopes3Weeks$data %>%
  mutate(
    "Residuals" = residuals(slopes3Weeks,
      type = "pearson",
      robust = TRUE
    )[, "Estimate"],
    "Fitted" = fitted(slopes3Weeks,
      robust = TRUE
    )[, "Estimate"]
  ) %>%
  ggplot(aes(x = Fitted, y = Residuals)) +
  geom_point(size = 2, pch = 21, colour = "black", fill = "grey50") +
  xlab("Fitted Values") +
  ylab("Pearson Residuals") +
  theme_classic()

(p1 + p2) / p3
```

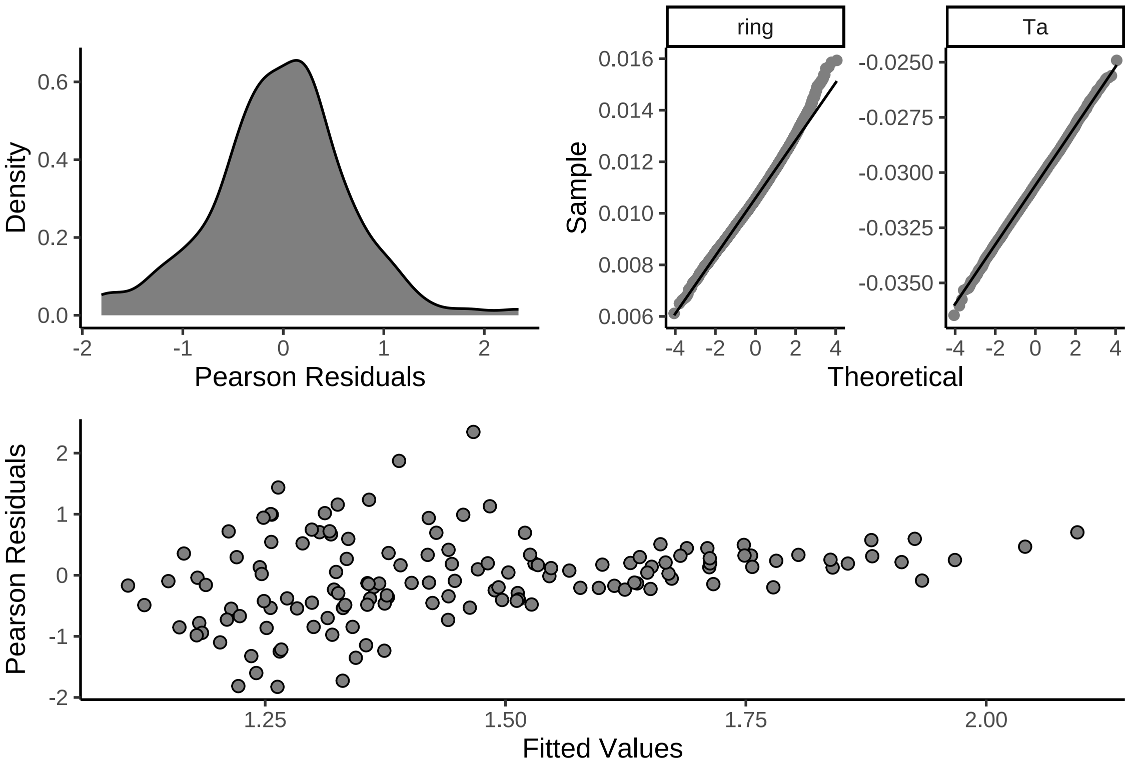

Figure 79: *Distributions of Pearson residuals and posterior densities drawn from Bayesian linear, mixed-effects model predicting relative resting metabolism (fold resting metabolism at thermoneutrality) of three week old Japanese quail by ambient temperature (raw ambient temperatures ranging from 10°C - 30°C).*

```
# Good. Some straying from normality among group-level effect of ring, although minor.
```

Conditional repeatability

For metabolic slopes to vary by morphology or thermal history, we first expect that consistent differences in slopes among individual are detectable. To test this expectation, we quantify conditional repeatability of our metabolic slopes according to Schielzeth and Nakagawa (2022), then visualise and formally test its deviance from null expectations.

```
# Note that intercept repeatabilities are calculated classically,
# and slope repeatabilities calculated as per Schielzeth and Nakagawa (2022).

as.data.frame(slopes3Weeks) %>%
  mutate(
    "Vs" = sd_ring__B_Ta^2 * var(slopes3Weeks$data$Ta) +
      mean(slopes3Weeks$data$Ta)^2 * sd_ring__B_Ta^2,
    "Vf" = b_B_Ta^2 * var(slopes3Weeks$data$Ta)
  ) %>%
  mutate("Slope" = Vs / (Vf + exp(b_sigma_Intercept)^2 +
    exp(b_sigma_Intercept + b_sigma_discreteTaB)^2)) %>%
  ggplot(aes(x = Slope)) +
  geom_density(alpha = 0.5, colour = "black", fill = "grey80") +
  theme_classic() +
  xlab("Metabolic Slope Repeatability") +
  ylab("Density") +
  theme(legend.position = "none")
```

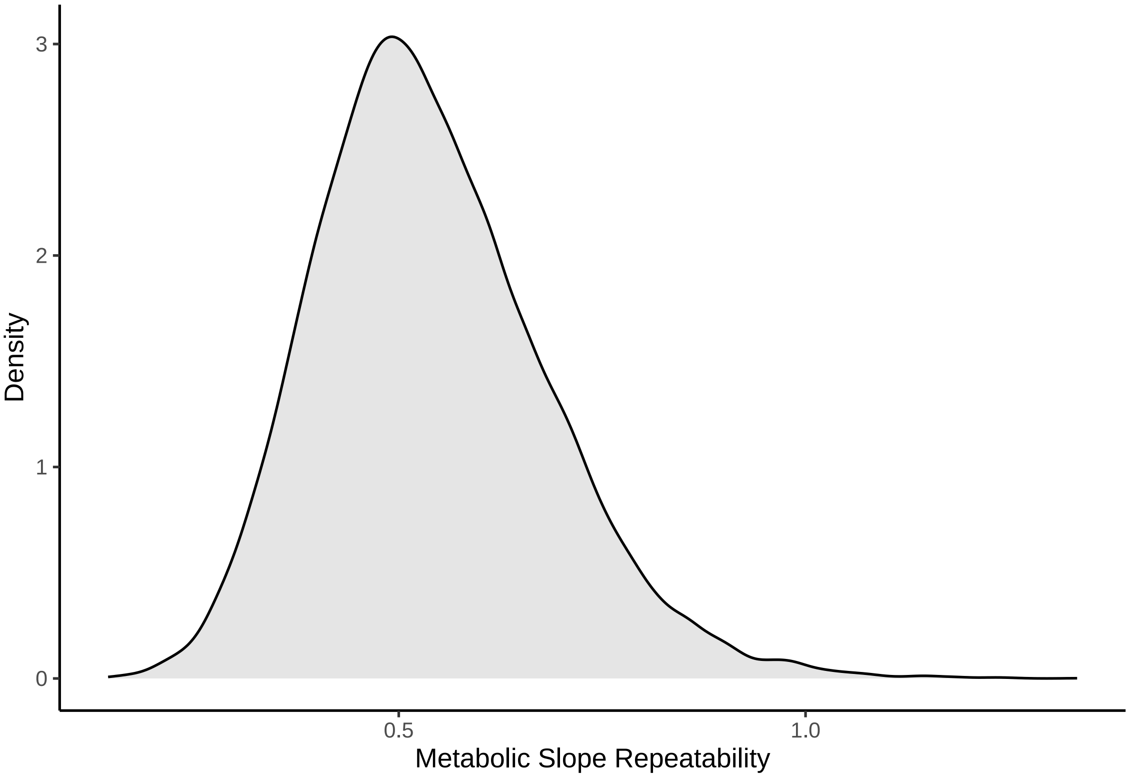

Figure 80: *Conditional repeatability of group-level (here, individual-level) slopes derived from a Bayesian, linear mixed-effects model predicting relative resting metabolism (fold metabolism at thermoneutrality) of three week old Japanese quail across ambient temperature (10°C - 30°C). Trends are estimated per individual and repeatabilities calculated from variability within and among trend parameters, as per Schielzeth and Nakagawa (2022).*

```
# Quantifying median and 95% quantile intervals.

repeatabilityFrame <-
  as.data.frame(slopes3Weeks) %>%
  mutate(
    "Vs" = sd_ring__B_Ta^2 * var(slopes3Weeks$data$Ta) +
      mean(slopes3Weeks$data$Ta)^2 * sd_ring__B_Ta^2,
    "Vf" = b_B_Ta^2 * var(slopes3Weeks$data$Ta)
  ) %>%
  mutate("Slope" = Vs / (Vf + exp(b_sigma_Intercept)^2 +
                         exp(b_sigma_Intercept + b_sigma_discreteTaB)^2)
        ) %>%
  dplyr::select(Slope)

caption <- paste0('Conditional repeatability of individual-level ',
                  "slopes for the relationship between ",
                  "relative resting metabolism ",
                  "(fold metabolism at thermoneutrality) ",
                  "across ambient temperature (10°C ",
                  "- 30°C) in three week old ",
                  "Japanese quail. Repeatability is calculated following ",
                  "Schielzeth and Nakagawa (2022) and represents ",
                  "the posterior median. 95\\% credible intervals indicate ",
                  "quantile intervals."
)

repeatability3Weeks <-
    repeatabilityFrame %>%
```

```
    summarise("Median" = round(median(Slope), digits = 4),
              "95% CIs" =
                paste0("[",
                       round(quantile(Slope, 0.025, type = 8),
                             digits = 4),
                       ", ",
                       round(quantile(Slope, 0.975, type = 8),
                             digits = 4),
                       "]")
              ) %>%
  kbl(.,
    longtable = T, booktabs = T, format = "latex",
    caption = caption
  ) %>%
  kable_styling(latex_options = "striped")

repeatability3Weeks
```

Table 34: *Conditional repeatability of individual-level slopes for the relationship between relative resting metabolism (fold metabolism at thermoneutrality) across ambient temperature (10°C - 30°C) in three week old Japanese quail. Repeatability is calculated following Schielzeth and Nakagawa (2022) and represents the posterior median. 95% credible intervals indicate quantile intervals.*

Median	95% CIs
0.5238	[0.2994, 0.8538]

```
#save_kable(repeatability3Weeks, "../tables/VO2Repeatability3Weeks.html")
```

To formally test an increase in repeatability from null expectations, we follow the approach described by Tabh et al (2022). Specifically, we randomly scramble individual identities, re-execute our model described above, recalculate conditional repeatability (referred to as a “null repeatability”) then test for a relative increase in true conditional repeatability from null repeatabilities.

```
slopes3WeeksScrambled <- brm(
  data = modData %>%
    select(ring, Ta, VO2) %>%
    pivot_wider(id_cols = "ring", names_from = Ta, values_from = VO2) %>%
    mutate(`10` = `10`/`30`,
           `20` = `20`/`30`,
           `30` = 1) %>%
    pivot_longer(-c("ring"), names_to = "Ta", values_to = "VO2") %>%
    mutate(Ta = as.integer(Ta)) %>%
    mutate(Ta = Ta - 30) %>%
    filter(Ta < 0) %>%
    mutate("ringScrambled" = sample(ring, nrow(.), replace = FALSE)) %>%
    mutate("discreteTa" = ifelse(Ta == -20, "A", "B")),
  family = "gaussian",
  bf(VO2 ~ 1 + B,
     B ~ 0 + Ta + (0 + Ta | ringScrambled),
     sigma ~ discreteTa,
     nl = TRUE),
  prior = c(
    set_prior("skew_normal(-0.018, 0.02, -2.5)", nlpar = "B",
              class = "b", coef = "Ta"),
    set_prior("exponential(2.5)", nlpar = "B", class = "sd",
              coef = "Ta", group = "ringScrambled"),
    set_prior("skew_normal(-0.2, 1, -5)", dpar = "sigma",
              class = "Intercept"),
    set_prior("normal(0.25, 0.25)", dpar = "sigma",
              class = "b")
  ),
  iter = 100000, warmup = 50000, thin = 10,
  chains = 4, cores = 4,
```

```
  control = list(adapt_delta = .98, max_treedepth = 14),
  silent = TRUE, refresh = 0,
  file = "./models/_threeWeekSlopesScrambled.Rds"
)

# Visually comparing first

repeatabilityFrameScrambled <-
  as.data.frame(slopes3WeeksScrambled) %>%
  mutate(
    "Vs" = sd_ringScrambled__B_Ta^2 *
      var(slopes3WeeksScrambled$data$Ta) +
      mean(slopes3WeeksScrambled$data$Ta)^2 *
        sd_ringScrambled__B_Ta^2,
    "Vf" = b_B_Ta^2 * var(slopes3WeeksScrambled$data$Ta)
  ) %>%
  mutate("Slope" = Vs / (Vf + exp(b_sigma_Intercept)^2 +
    exp(b_sigma_Intercept + b_sigma_discreteTaB)^2)) %>%
  select(Slope)

repeatPlot <- rbind(
  repeatabilityFrame %>%
    mutate("Type" = "True Model"),
  repeatabilityFrameScrambled %>%
    mutate("Type" = "Null Model")
) %>%
  ggplot(aes(x = Slope, fill = Type)) +
  geom_density(colour = "black", adjust = 1, alpha = 0.8) +
  scale_fill_manual(values = c("grey20", "grey80")) +
  xlab("Conditional Repeatability at 3 Weeks") +
  ylab("Density") +
  theme_classic() +
  theme(
    axis.text = element_text(family = "Noto Sans"),
    axis.title = element_text(family = "Noto Sans"),
    strip.text.x = element_text(family = "Noto Sans", colour = "black")
  )

showtext_auto()
repeatPlot
```

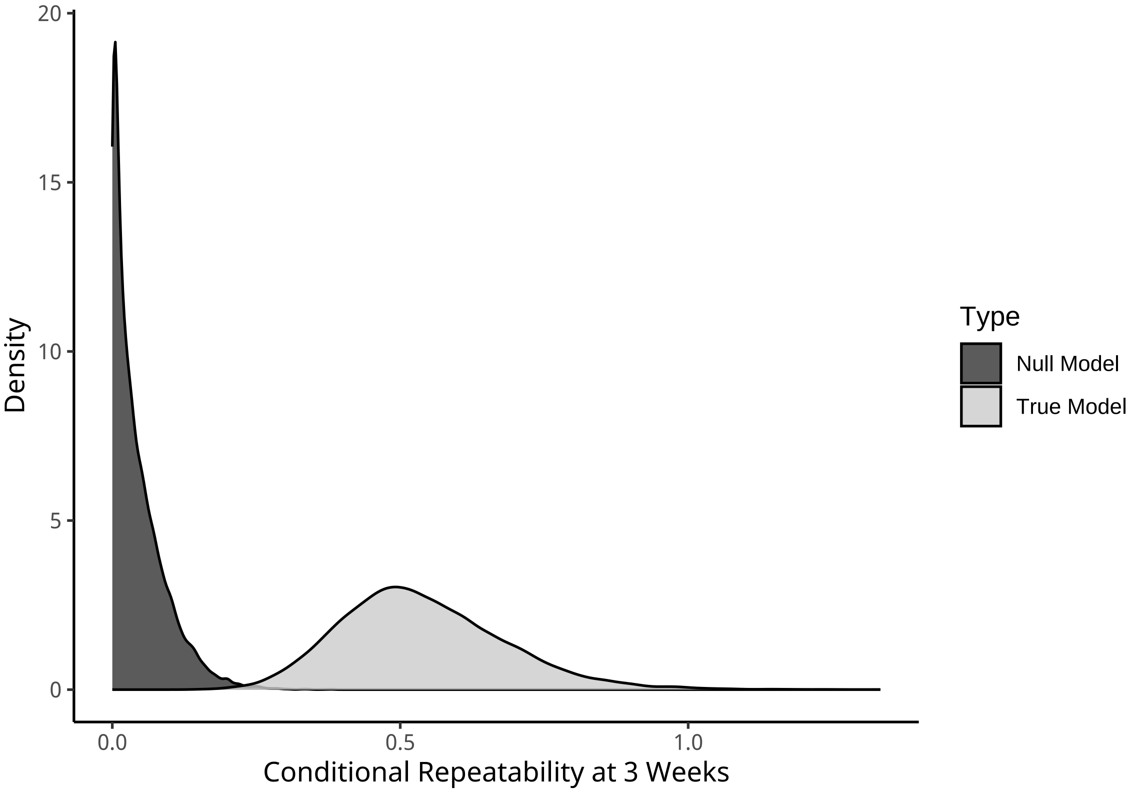

Figure 81: *Density of slope repeatabilites calculated from coefficients of a Bayesian, linear mixed-effects model predicting relative resting metabolism (fold resting metabolism at thermoneutrality) across ambient temperature (10°C - 30°C) in three week old Japanese quail. Light grey densities represent true repeatability estimates and dark grey densities represent those calculated from models with scrambled individual identities (null model). All repeatabilities are calculated as per Schielzeth and Nakagawa (2022).*

```
ggsave("./plots/traitRepeatability3Weeks_3.pdf",
  repeatPlot,
  dpi = 800, height = 5, width = 7.5
)
showtext_auto(enable = FALSE)
```

Comparisons between true and null conditional repeatabilities are achieved using a one-way hypothesis test via the Savage-Dickey density comparison method. Priors for our null and true repeatabilies are assigned generous beta distributions with $\alpha = 1$ and $\beta = 4$.

```
# Clear effect of identity on metabolic slopes.

require(loo)

u1RepeatabilityTestFrame <- build_hdf(
  vars = list(
    repeatabilityFrame$Slope,
    repeatabilityFrameScrambled$Slope
  ),
  priors = list(
    rbeta(nrow(repeatabilityFrame), 1, 4),
    rbeta(nrow(repeatabilityFrameScrambled), 1, 4)
  ),
  names = c("True", "Null")
)
```

```r
u1RepeatabilityTest <- hypothesis_df("True > Null",
                                     u1RepeatabilityTestFrame,
                                     class = "b", alpha = 0.05
)

# Relabelling for clearer interpretation of plots

u1RepeatabilityTest$hypothesis$Hypothesis <- paste0(
  "Slope: ",
  u1RepeatabilityTest$hypothesis$Hypothesis
)

# Viewing outcome and summarising results

repeatTestPlot <-
    data.frame(
      "values" = c(
        u1RepeatabilityTest$samples$H1,
        u1RepeatabilityTest$prior_samples$H1
      ),
      "Type" = c(
        rep("Posterior", length(u1RepeatabilityTest$samples$H1)),
        rep("Prior", length(u1RepeatabilityTest$prior_samples$H1))
      ),
      "Hypothesis" = "Slope Repeatability > Null"
    ) %>%
  ggplot(aes(x = values, fill = Type)) +
  facet_wrap(~Hypothesis) +
  geom_density(colour = "black", alpha = 0.4) +
  geom_vline(xintercept = 0, colour = "black", linetype = "longdash") +
  scale_fill_manual(values = c("black", "grey80")) +
  xlab("True - Null Repeatability") +
  ylab("Density") +
  theme_classic()

repeatTestPlot
```

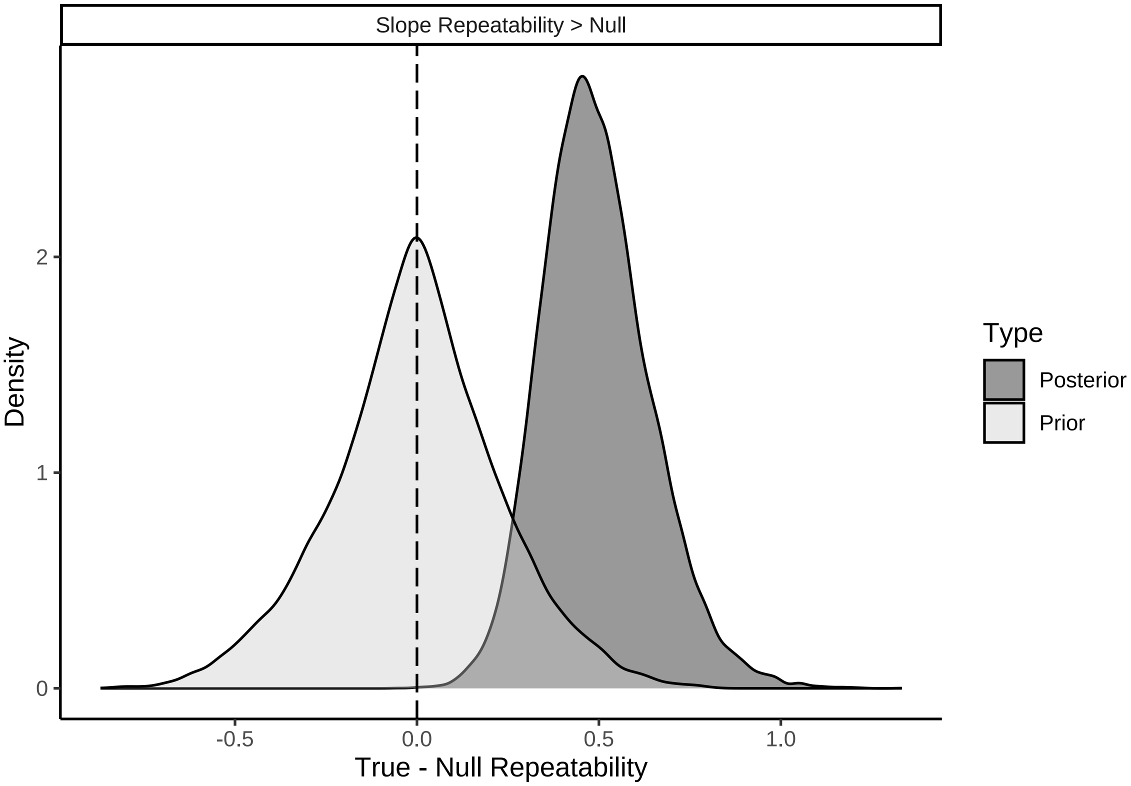

Figure 82: *Differences between true and null repeatability estimates for individual-level slopes of relative resting metabolism (fold metabolism at thermoneutrality) by ambient temperature (°C; between 10-30°C) curves in three week old Japanese quail.*

```
caption <- paste0("Results of a non-linear hypothesis ",
                  "tests comparing true and null repeatabilities ",
                  "of individual-level slopes from relative ",
                  "resting metabolism (fold metabolism at ",
                  "thermoneutrality) by ambient temperature ",
                  "(10-30°C) curves. ",
                  "Curves are derived from three week old ",
                  "Japanese quail. Posterior probabilities are ",
                  "calculated using the Savage-Dickey ",
                  "density ratio method."
)

repeatabilityTest3Weeks <- u1RepeatabilityTest$hypothesis %>%
  mutate(Hypothesis = c(
    "True Slope Repeatability > Null"
  )) %>%
  select(-c(Evid.Ratio, Star)) %>%
  mutate(Estimate = round(Estimate, digits = 4),
         "Est.Error" = round(Est.Error, digits = 4),
         "CI.Lower" = round(CI.Lower, digits = 4),
         "CI.Upper" = round(CI.Upper, digits = 4)) %>%
  rename(
    "Difference Between True and Null Repeatabilities" = Estimate,
    "Standard Error" = Est.Error,
    `2.5 % CI` = "CI.Lower",
    `97.5 % CI` = "CI.Upper",
    "Posterior Probability" = Post.Prob
  ) %>%
  kbl(.,
    longtable = T, booktabs = T, format = "latex",
    caption = caption
```

```
  ) %>%
  column_spec(column = c(1:2), width = "2.5cm")  %>%
  column_spec(column = c(3:10), width = "2cm")  %>%
  kable_styling(latex_options = "striped")

repeatabilityTest3Weeks
```

Table 35: *Results of a non-linear hypothesis tests comparing true and null repeatabilities of individual-level slopes from relative resting metabolism (fold metabolism at thermoneutrality) by ambient temperature (10-30°C) curves. Curves are derived from three week old Japanese quail. Posterior probabilities are calculated using the Savage-Dickey density ratio method.*

Hypothesis	Difference Between True and Null Repeatabilities	Standard Error	2.5 % CI	97.5 % CI	Posterior Probability
True Slope Repeatability > Null	0.4943	0.1495	0.2715	0.7553	0.9999

```
#save_kable(repeatabilityTest3Weeks, "../tables/repeatabilityTest3Weeks.html")
```

Visualising and quantifying metabolic slopes in the cold (adults)

Models including only eight week old individuals are next constructed. Again, only individuals for whom resting metabolism was measured at thermoneutrality (30°C) and at least one ambient temperature below were used here.

Using data derived from other adult Japanese quail in our sample population, we have estimated the lower critical temperature of our quail strain to be approximately 24°C (see section 5.0 of this document). For this reason, slopes of resting metabolism by ambient temperature are not likely to be linear at and below 30°C, potentially biasing estimation of metabolic slopes. To therefore better estimate metabolic slopes in our sample population, we reassigned our metabolism measurements at 30°C to 24°C, since: (1) metabolism at these temperatures is expect to be constant given that both temperatures fall within thermoneutrality, and (2) doing so will linearise metabolism by ambient temperature measurements as expected below thermoneutrality. Below, we visually test whether linearisation does occur with ambient temperature reassignment.

```
## Repeating model for 8 week measurements while retaining
# equivalent priors

birdVector <- merge(
  all %>%
    drop_na(VO2) %>%
    filter(week == 8 & Ta == 30) %>%
    select(ring) %>%
    distinct(),
  all %>%
    drop_na(VO2) %>%
    filter(week == 8 & Ta %in% c(10, 20)) %>%
    select(ring) %>%
    distinct(),
  by = "ring", all = FALSE
) %>% pull(ring)

modData <- all %>%
  filter(week == 8 & Ta < 35 & ring %in% birdVector)

vo2ByTaAdjusted <- modData %>%
  mutate(Ta = ifelse(Ta == 30, 24, Ta)) %>%
  ggplot(aes(x = Ta, y = VO2)) +
  geom_point(
```

```
    pch = 21, size = 1.5, alpha = 0.5,
    colour = "black", fill = "grey50",
    position = position_jitter(width = 0.2)
  ) +
  stat_summary(geom = "errorbar", fun.data = "mean_se",
               width = 0.5, colour = "black", size = 1) +
  stat_summary(
    geom = "point", fun = "mean", size = 4, pch = 21, colour = "black",
    fill = "grey50"
  ) +
  geom_smooth(method = "lm", colour = "black",
              linetype = "dashed", se = FALSE) +
  xlab("Ambient Temperature (°C)") +
  ylab(bquote(Metabolism ~ at ~ 8 ~ weeks ~ (mL ~ O[2] / min))) +
  theme_classic() +
  theme(axis.title = element_text(family = "Noto Sans", size = 12))

showtext_auto()
vo2ByTaAdjusted
```

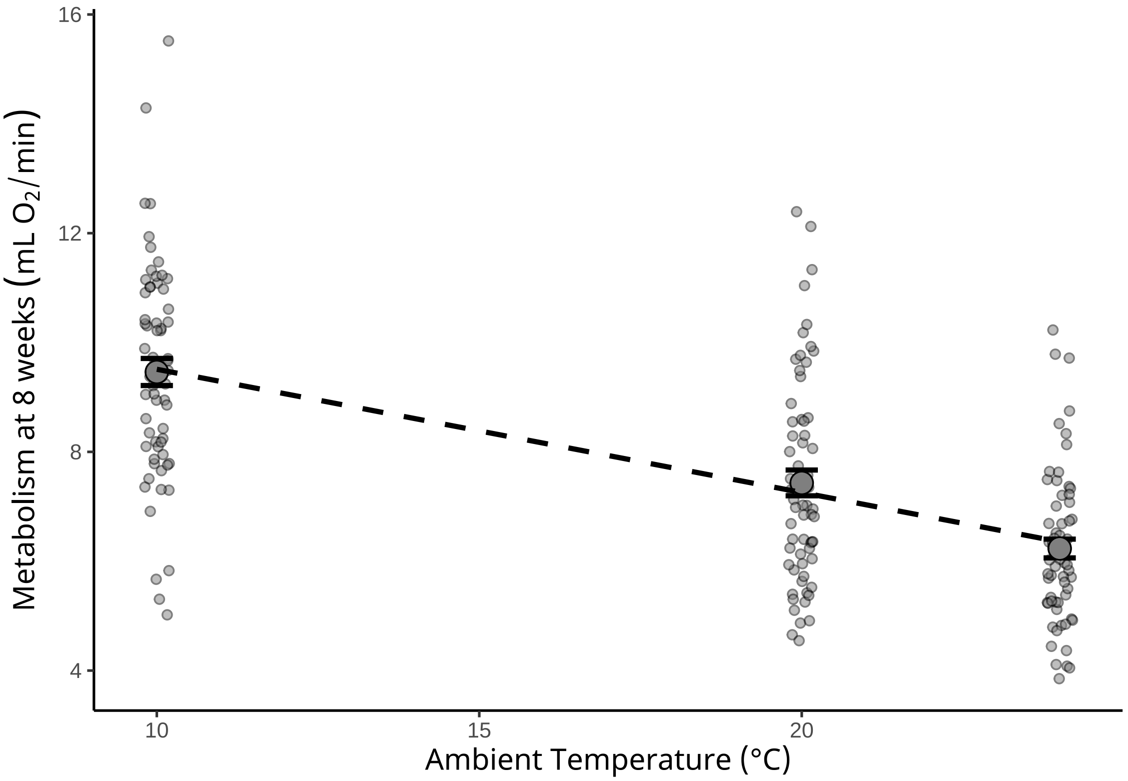

Figure 83: *Relationship between resting metabolism and ambient temperature in eight week old Japanese quail. Large dots represent means across quail and small dots represent individual measurements. Errorbars around large dots represent standard errors and the dotted line represents the linear, line of best fit as estimated by the R package ggplot2 (Wickham, 2011). Metabolism measurements at 24°C were collected at 30°C and expected to be constant between 24°C and 30°C.*

```
showtext_auto(enable = FALSE)
```

Above, the correlation between resting metabolism and ambient temperature is largely linearised after reassigning measurements at 30°C to 24°C (with our line of best fit passing through one standard error around means). We proceed by constructing a model of fold-metabolism by ambient temperature using these data as previously described. Priors remain the same as those used for our model addressing metabolic trends in

three week old Japanese quail. However, despite this constancy of priors, we still begin by checking their suitability against data obtained from mature quail.

```
# Running prior predictive check.

slopes8WeeksPPCheck <- brm(
  data = modData %>%
    select(ring, Ta, VO2) %>%
    pivot_wider(id_cols = "ring", names_from = Ta, values_from = VO2) %>%
    mutate(`10` = `10`/`30`,
           `20` = `20`/`30`,
           `30` = 1) %>%
    rename(`24` = `30`) %>%
    pivot_longer(-c("ring"), names_to = "Ta", values_to = "VO2") %>%
    mutate(Ta = as.integer(Ta)) %>%
    mutate(Ta = Ta - 24) %>%
    filter(Ta < 0) %>%
    mutate("discreteTa" = ifelse(Ta == -14, "A", "B")),
  family = "gaussian",
  bf(VO2 ~ 1 + B,
     B ~ 0 + Ta + (0 + Ta | ring),
     sigma ~ discreteTa,
     nl = TRUE),
  prior = c(
    set_prior("skew_normal(-0.018, 0.02, -2.5)", nlpar = "B",
              class = "b", coef = "Ta"),
    set_prior("exponential(2.5)", nlpar = "B", class = "sd",
              coef = "Ta", group = "ring"),
    set_prior("skew_normal(-0.2, 1, -5)", dpar = "sigma",
              class = "Intercept"),
    set_prior("normal(0.25, 0.25)", dpar = "sigma",
              class = "b")
  ),
  iter = 50000, warmup = 10000, thin = 10,
  chains = 4, cores = 4,
  control = list(adapt_delta = .96),
  silent = TRUE, refresh = 0,
  sample_prior = "only",
  file = "./models/_eightWeekSlopesPPCheck.Rds"
)

pp_check2(slopes8WeeksPPCheck,
          xlab = TeX('$Relative~Resting~Metabolism$')) +
  xlim(c(-10, 10))
```

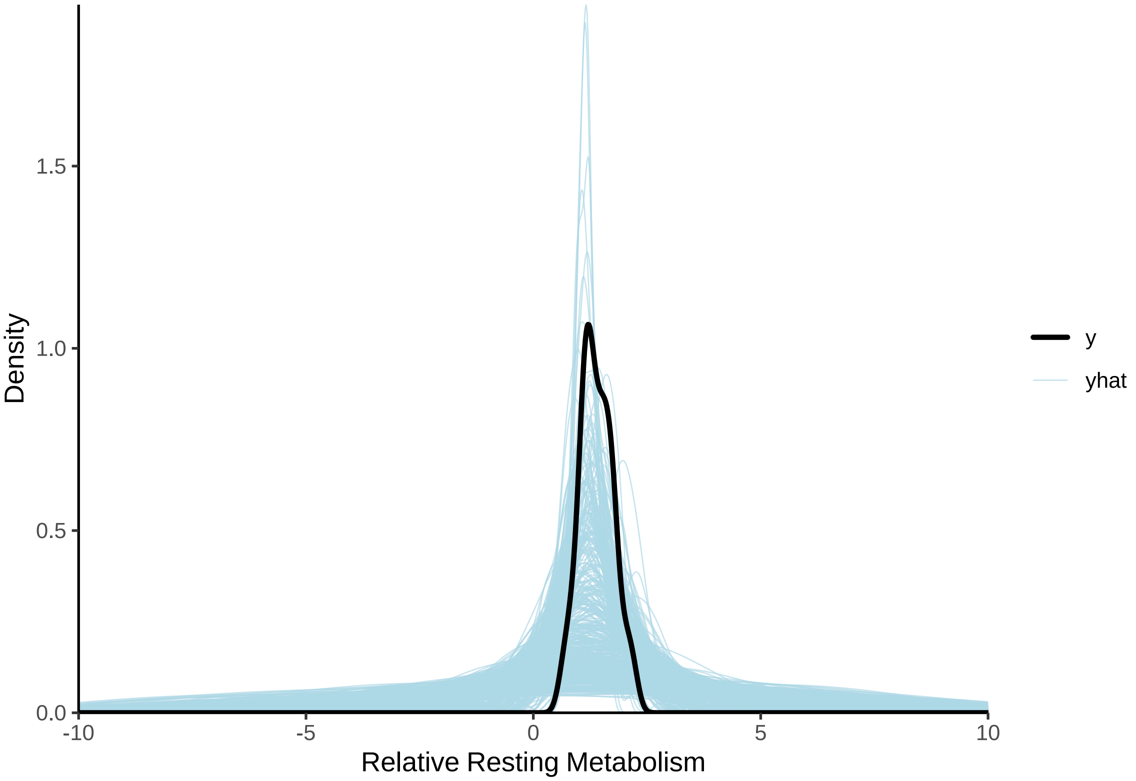

Figure 84: *Prior predictive check for a Bayesian linear, mixed-effects model predicting relative resting metabolism (fold metabolism at thermoneutrality) of eight week old Japanese quail by mean-centred ambient temperature (raw ambient temperatures ranging from 10°C - 24°C). Light blue lines represent densities of resting metabolism values as predicted by model priors alone. The dark blue line represent the true density of resting metabolism values. Clear overlap between the dark blue and light blue lines indicates that priors are suitable.*

Again, centrality of relative metabolism measures is well predicted. We proceed with the selected priors.

```
slopes8Weeks <- brm(
  data = modData %>%
    select(ring, Ta, VO2) %>%
    pivot_wider(id_cols = "ring", names_from = Ta, values_from = VO2) %>%
    mutate(`10` = `10`/`30`,
           `20` = `20`/`30`,
           `30` = 1) %>%
    rename(`24` = `30`) %>%
    pivot_longer(-c("ring"), names_to = "Ta", values_to = "VO2") %>%
    mutate(Ta = as.integer(Ta)) %>%
    mutate(Ta = Ta - 24) %>%
    filter(Ta < 0) %>%
    mutate("discreteTa" = ifelse(Ta == -14, "A", "B")),
  family = "gaussian",
  bf(VO2 ~ 1 + B,
     B ~ 0 + Ta + (0 + Ta | ring),
     sigma ~ discreteTa,
     nl = TRUE),
  prior = c(
    set_prior("skew_normal(-0.018, 0.02, -2.5)", nlpar = "B",
              class = "b", coef = "Ta"),
    set_prior("exponential(2.5)", nlpar = "B", class = "sd",
              coef = "Ta", group = "ring"),
    set_prior("skew_normal(-0.2, 1, -5)", dpar = "sigma",
```

```
              class = "Intercept"),
    set_prior("normal(0.25, 0.25)", dpar = "sigma",
              class = "b")
  ),
  iter = 50000, warmup = 10000, thin = 10, cores = 4, chains = 4,
  control = list(adapt_delta = .96),
  silent = TRUE, refresh = 0,
  file = "./models/_eightWeekSlopes.Rds"
)

# Again, assessing Rubin-Gelman statistic, ratio of effective
# sample sizes to sample sizes, and distribution of posterior predictions.

p1 <- mcmc_neff(
  neff_ratio(slopes8Weeks)[names(neff_ratio(slopes8Weeks)) != "lp__"], size = 2) +
  theme(legend.position = "none") +
  xlab(
    TeX('$N_{eff}/N~Ratio$')
  )
p2 <- mcmc_rhat(
  rhat(slopes8Weeks)[names(rhat(slopes8Weeks)) != "lp__"]) +
  theme(legend.position = "none") +
  xlab(
    TeX('$\\hat{R}$')
  )

p3 <- pp_check2(slopes8Weeks,
                xlab = TeX('$Relative~Resting~Metabolism$'))
p4 <- ppc_scatter_avg_grouped(
  slopes8Weeks$data %>%
    pull(VO2),
  posterior_predict(slopes8Weeks,
                    ndraws = 100, robust = TRUE),
  group = slopes8Weeks$data %>%
    merge(., modData %>%
            select(ring, pretreatment) %>%
            distinct(),
          by = "ring", all.x = TRUE) %>%
    mutate(pretreatment = ifelse(pretreatment == "cold", "Cold\n(10°C)",
      ifelse(pretreatment == "neutral", "Mild\n(20°C)",
        "Warm\n(30°C)"
      )
    )) %>%
    pull(pretreatment)
) +
  geom_smooth(method = "lm", colour = "black") +
  xlab("Predicted Metabolism\n(Fold from Thermoneutrality)") +
  ylab("True Metabolism\n(Fold from Thermoneutrality") +
  scale_fill_manual(values = "lightblue", guide = "none") +
  theme_classic()

(p1 + p2 + p3) / (p4) + plot_annotation(tag_levels = "A")
```

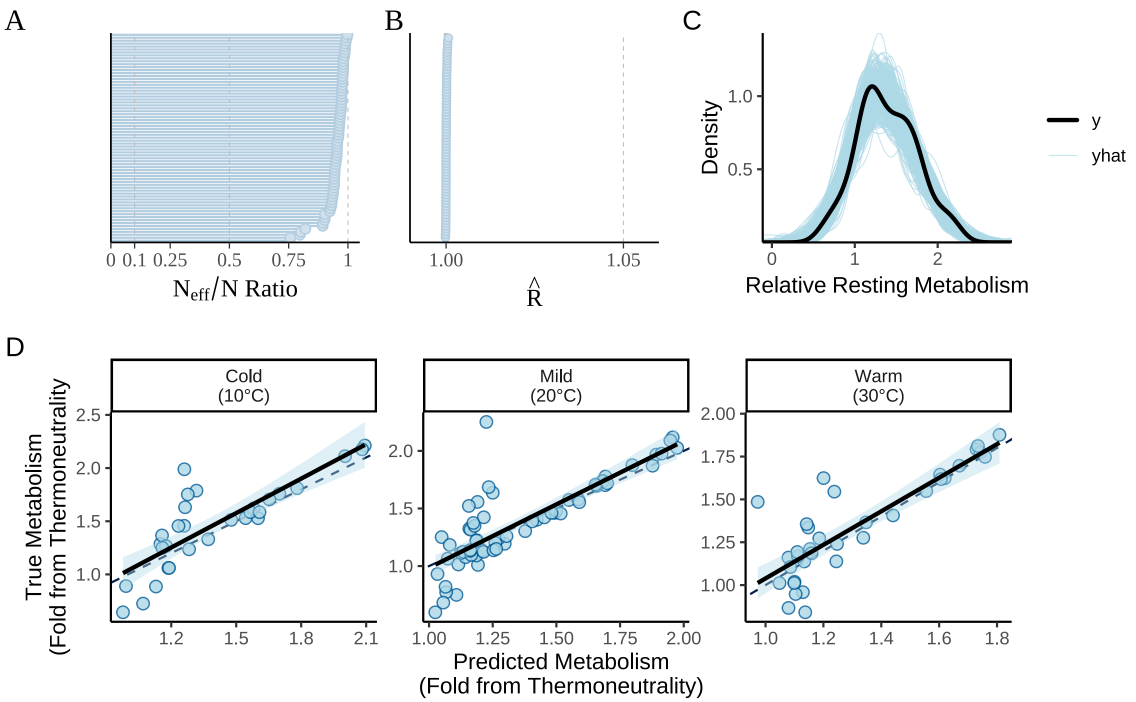

Figure 85: *Validations for a Bayesian mixed effects model predicting relative resting metabolism (fold metabolism at thermoneutrality) of eight week old Japanese quail across 10°C - 24°C. Quail were reared across three different thermal environments: (1) cold (10°C until at least 3 weeks of age), mild (20°C until 8 weeks of age), or warm (30°C until at least 3 weeks of age). Panel A displays the ratio of effective sample sizes to sample sizes for all model parameters. Panel B displays Gelman-Rubin statistics, again, for each model parameter. Panel C displays densities of true (y) and predicted (yhat) values of resting metabolism (mL O_2/min). Panel D displays true and predicted resting metabolism (mL O_2/min) values across each rearing treatment.*

```
rm(p1, p2, p3, p4)
```

Residuals from this model are next visualised and relative fit (Bayesian R^2) evaluated.

```
# No obvious bias by treatment groups. Checking residuals.

g(p1, p2, p3) %=% list(
  ggplot(
    slopes8Weeks$data %>%
      mutate(
        "residuals" =
          residuals(slopes8Weeks,
            type = "pearson",
            robust = TRUE
          )[, "Estimate"]
      ),
    aes(x = residuals)
  ) +
    geom_density(colour = "black") +
    theme_classic() +
    xlab("Pearson Residuals") +
    ylab("Denstiy"),
  ggplot(
    slopes8Weeks$data %>%
      mutate("residuals" = residuals(slopes8Weeks,
        robust = TRUE
      )[, "Estimate"]),
```

```r
    aes(x = ring, y = residuals)
  ) +
    geom_boxplot(fill = "lightblue", colour = "black", width = 0.3) +
    geom_point(
      pch = 21, size = 3, fill = "lightblue",
      alpha = 0.5, position = position_jitter(width = 0.2)
    ) +
    xlab("Bird Identity") +
    ylab("Ordinary Residuals") +
    theme_classic() +
    theme(
      axis.text.x = element_blank(),
      axis.ticks.x = element_blank()
    ),
  ggplot(
    slopes8Weeks$data %>%
      mutate(
        "residuals" = residuals(slopes8Weeks,
          type = "pearson",
          robust = TRUE
        )[, "Estimate"],
        "residualSE" = residuals(slopes8Weeks,
          type = "pearson",
          robust = TRUE
        )[, "Est.Error"],
        "fitted" = fitted(slopes8Weeks,
          robust = TRUE
        )[, "Estimate"],
        "fittedSE" = fitted(slopes8Weeks,
          robust = TRUE
        )[, "Est.Error"]
      ),
    aes(x = fitted, y = residuals)
  ) +
    geom_errorbar(
      aes(
        ymin = residuals - residualSE,
        ymax = residuals + residualSE
      ),
      width = 0.1, alpha = 0.5
    ) +
    geom_errorbar(
      aes(
        xmin = fitted - fittedSE,
        xmax = fitted + fittedSE
      ),
      height = 0.5, alpha = 0.5
    ) +
    geom_point(pch = 21, size = 3, fill = "lightblue", alpha = 0.5) +
    theme_classic() +
    xlab("Fitted Values") +
    ylab("Pearson Residuals")
)

(p1 + p3) / p2 + plot_annotation(tag_levels = "A")
```

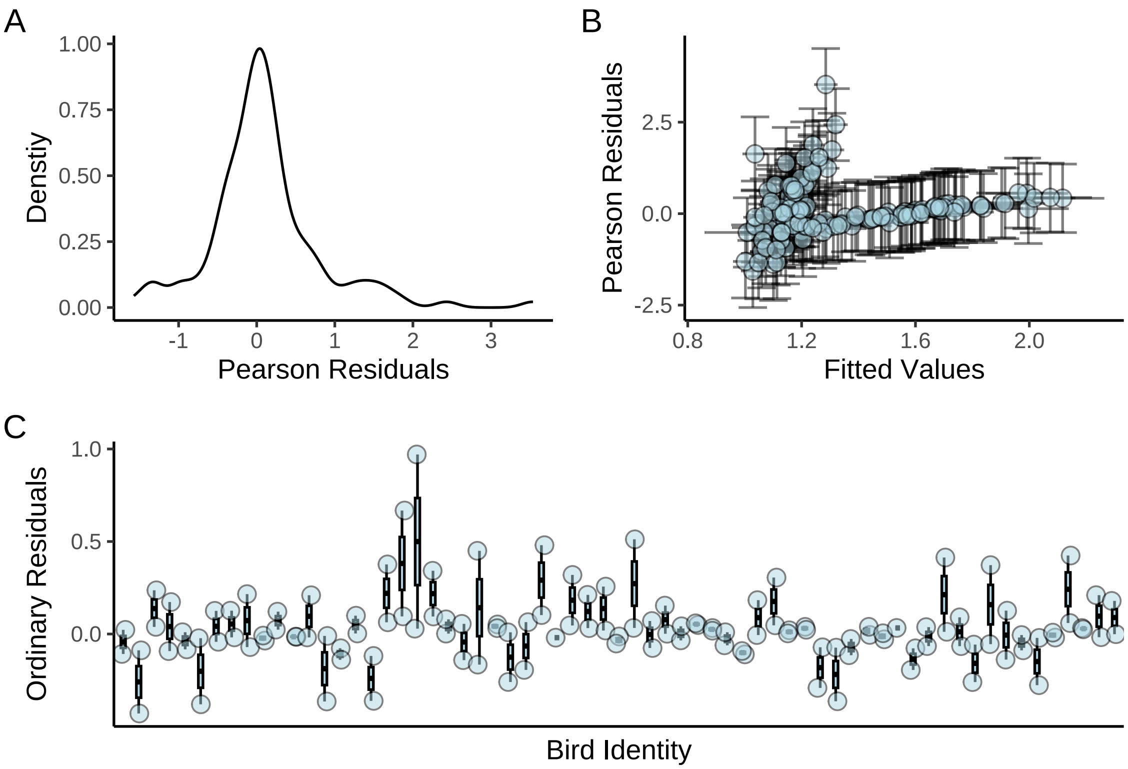

Figure 86: *Ordinary residuals from a Bayesian mixed-effects model predicting relative resting metabolism (fold metabolism at thermoneutrality) of eight week old Japanese quail across 10°C - 24°C. Panel A displays residuals against mean-centred ambient temperature values, while panel B displays residuals against fitted values. Panel C displays residuals across individual bird identities.*

```
# Checking R2 values

caption <- paste0(
  "R\\textsuperscript{2} for Bayesian, linear mixed-effects ",
  "model predicting relative resting metabolism (fold ",
  "metabolism at thermoneutrality) of eight week old Japanese ",
  "quail by mean-centred ambient temperature ",
  "(raw ambient temperatures ranging from ",
  "10°C - ",
  "24°C)."
)

brms::bayes_R2(slopes8Weeks,
               robust = TRUE, ndraws = 1000) %>%
  as.data.frame() %>%
  remove_rownames() %>%
  mutate(Estimate = round(Estimate, digits = 4),
         Est.Error = round(Est.Error, digits = 4),
         Q2.5 = round(Q2.5, digits = 4),
         Q97.5 = round(Q97.5, digits = 4)
         ) %>%
  rename(
    "R\\textsuperscript{2}" = Estimate,
    "Standard Error" = Est.Error,
    `2.5\\%CI` = "Q2.5", `97.5\\% CI` = "Q97.5"
  ) %>%
  kbl(.,
    longtable = T, booktabs = T, format = "latex",
    caption = caption, escape = FALSE
  ) %>%
```

```
  kable_styling(latex_options = "striped")
```

Table 36: *R^2 for Bayesian, linear mixed-effects model predicting relative resting metabolism (fold metabolism at thermoneutrality) of eight week old Japanese quail by mean-centred ambient temperature (raw ambient temperatures ranging from 10°C - 24°C).*

R^2	Standard Error	2.5%CI	97.5% CI
0.648	0.0407	0.5205	0.706

Posterior densities per individual are plotted for visual evaluation of spread and skeweness.

```
as.data.frame(slopes8Weeks) %>%
  select(starts_with("r_")) %>%
  pivot_longer(everything(), names_to = "ring",
               values_to = "slope") %>%
  mutate("ring" =
           gsub("\\,.*", "",
                gsub(".*\\[", "", ring)
                )
  ) %>%
  mutate(slope = fixef(slopes8Weeks)[1,"Estimate"] +
           slope) %>%
  ggplot(aes(x = slope)) +
  facet_wrap(~ring) +
  geom_density() +
  xlab("Metabolic Slope") +
  ylab("Density") +
  theme_classic() +
  theme(axis.text.x = element_blank(),
        axis.ticks.x = element_blank(),
        axis.text.y = element_blank(),
        axis.ticks.y = element_blank())
```

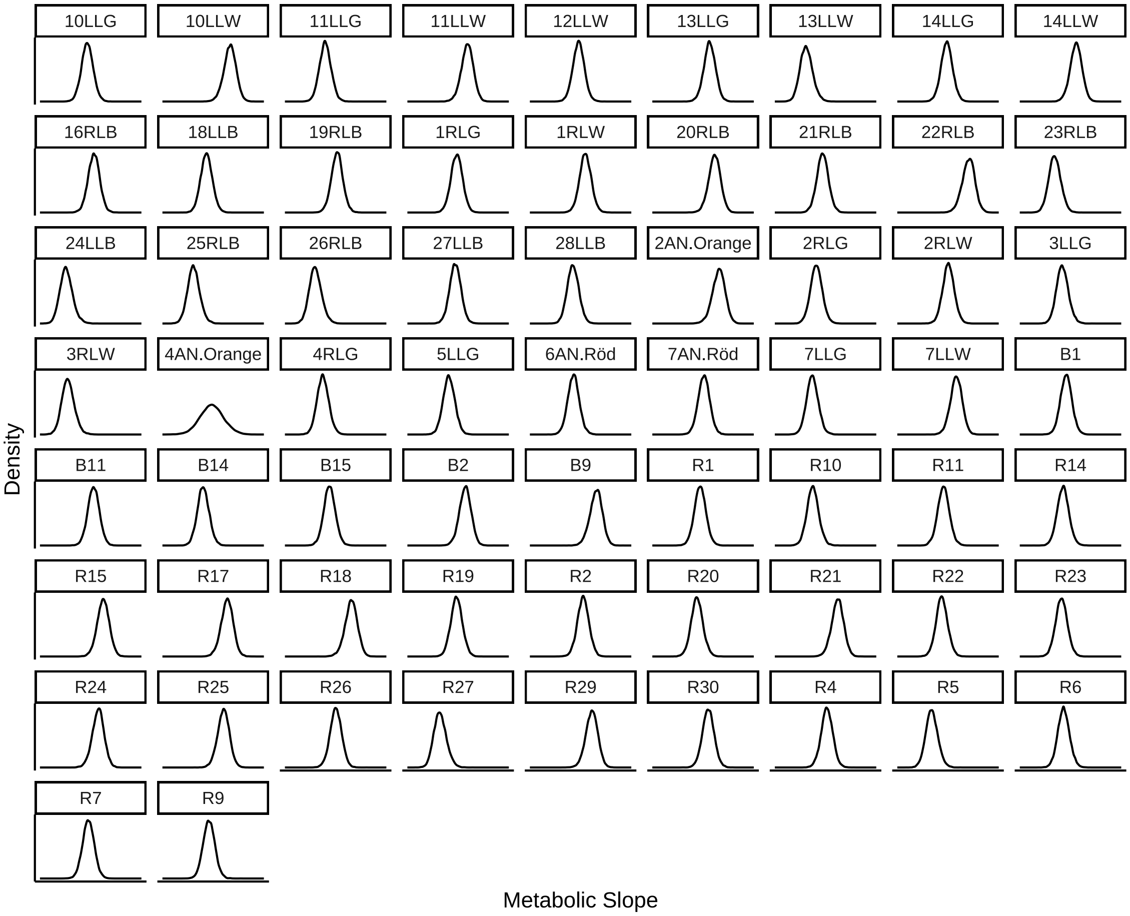

Figure 87: *Posterior densities of metabolic slopes, per individual, from a Bayesian linear mixed-effects model predicting relative resting metabolism (fold resting metabolism at thermoneutrality) of eight week old Japanese quail by ambient temperature (raw ambient temperatures ranging from 10°C - 24°C).*

```
# Means appear to be good estimates of central tendency.
```

Conditional repeatability

As done for metabolic slopes during development, we next test whether individuals consistently vary in their metabolic responses to decreasing temperature. This is achieved by quantifying conditional repeatability of metabolic slopes, then testing whether this repeatability differs from null expectations (as described above).

```
as.data.frame(slopes8Weeks) %>%
  mutate(
    "Vs" = sd_ring__B_Ta^2 * var(slopes8Weeks$data$Ta) +
      mean(slopes8Weeks$data$Ta)^2 * sd_ring__B_Ta^2,
    "Vf" = b_B_Ta^2 * var(slopes8Weeks$data$Ta)
  ) %>%
  mutate("Slope" = Vs / (Vf + exp(b_sigma_Intercept)^2 +
                                 exp(b_sigma_Intercept + b_sigma_discreteTaB)^2)
         ) %>%
  ggplot(aes(x = Slope)) +
  geom_density(alpha = 0.5, colour = "black", fill = "grey80") +
  theme_classic() +
  xlab("Metabolic Slope Repeatability") +
  ylab("Density") +
  theme(legend.position = "none")
```

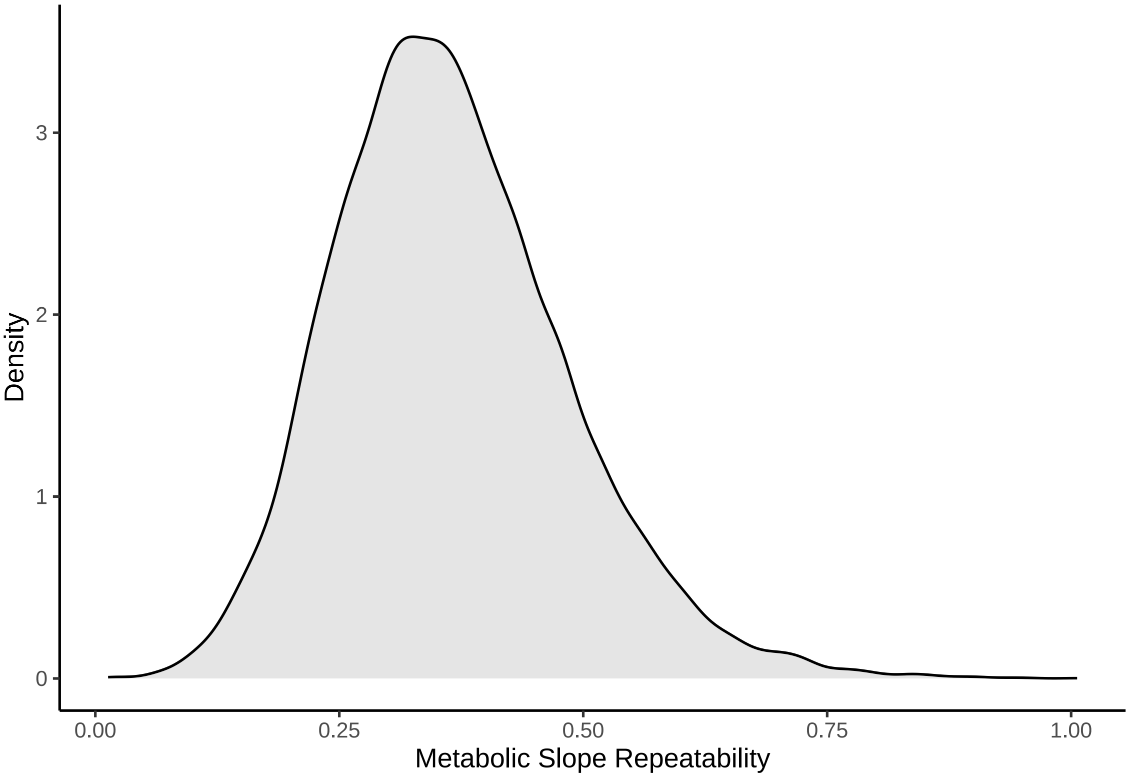

Figure 88: *Repeatability of group-level (here, individual-level) slopes derived from a Bayesian, linear mixed-effects model predicting relative resting metabolism (fold metabolism at thermoneutrality) of eight week old Japanese quail across ambient temperature (10°C - 24°C). Trends are estimated per individual and repeatabilities calculated from variability within and among trend parameters, as per Schielzeth and Nakagawa (2022).*

Again, repeatability values appear quite high but are formally tested for biological significance following Tabh et al (2022).

```
repeatabilityFrame <- as.data.frame(slopes8Weeks) %>%
  mutate(
    "Vs" = sd_ring__B_Ta^2 * var(slopes8Weeks$data$Ta) +
      mean(slopes8Weeks$data$Ta)^2 * sd_ring__B_Ta^2,
    "Vf" = b_B_Ta^2 * var(slopes8Weeks$data$Ta)
  ) %>%
  mutate("Slope" = Vs / (Vf + exp(b_sigma_Intercept)^2 +
         exp(b_sigma_Intercept + b_sigma_discreteTaB)^2)
         ) %>%
  dplyr::select(Slope)

caption <- paste("Repeatability of individual-level ",
                 "slopes for the relationship ",
                 "between relative resting metabolism (fold ",
                 "metabolism at thermoneutrality) and ambient ",
                 "temperature (10°C ",
                 "- 24°C) in eight week ",
                 "old Japanese quail. Repeatabilities are ",
                 "calculated using group-level coefficients ",
                 "derived from a Bayesian linear-mixed effects ",
                 "model. Calculations follow Schielzeth and ",
                 "Nakagawa (2022)."
)
```

```
repeatability8Weeks <-
    repeatabilityFrame %>%
      summarise("Median" = median(Slope),
                "95% CIs" =
                  paste0("[",
                         round(quantile(Slope, 0.025, type = 8),
                               digits = 4),
                         ", ",
                         round(quantile(Slope, 0.975, type = 8),
                               digits = 4),
                         "]")
                ) %>%
  kbl(.,
    longtable = T, booktabs = T, format = "latex",
    caption = caption
  ) %>%
  kable_styling(latex_options = "striped")

repeatability8Weeks
```

Table 37: *Repeatability of individual-level slopes for the relationship between relative resting metabolism (fold metabolism at thermoneutrality) and ambient temperature (10°C - 24°C) in eight week old Japanese quail. Repeatabilities are calculated using group-level coefficients derived from a Bayesian linear-mixed effects model. Calculations follow Schielzeth and Nakagawa (2022).*

Median	95% CIs
0.3541923	[0.1614, 0.6275]

```
#save_kable(repeatability8Weeks,
# "../tables/VO2Repeatability8Weeks.html")

# And testing formally with scrambled model.

slopes8WeeksScrambled <- brm(
data = modData %>%
    select(ring, Ta, VO2) %>%
    pivot_wider(id_cols = "ring", names_from = Ta, values_from = VO2) %>%
    mutate(`10` = `10`/`30`,
           `20` = `20`/`30`,
           `30` = 1) %>%
    rename(`24` = `30`) %>%
    pivot_longer(-c("ring"), names_to = "Ta", values_to = "VO2") %>%
    mutate(Ta = as.integer(Ta)) %>%
    mutate(Ta = Ta - 24) %>%
    filter(Ta < 0) %>%
    mutate("discreteTa" = ifelse(Ta == -14, "A", "B"),
           "ringScrambled" = sample(ring, nrow(.), replace = FALSE)),
  family = "gaussian",
  bf(VO2 ~ 1 + B,
     B ~ 0 + Ta + (0 + Ta | ringScrambled),
     sigma ~ discreteTa,
     nl = TRUE),
  prior = c(
    set_prior("skew_normal(-0.018, 0.02, -2.5)", nlpar = "B",
              class = "b", coef = "Ta"),
    set_prior("exponential(2.5)", nlpar = "B", class = "sd",
              coef = "Ta", group = "ringScrambled"),
    set_prior("skew_normal(-0.2, 1, -5)", dpar = "sigma",
              class = "Intercept"),
    set_prior("normal(0.25, 0.25)", dpar = "sigma",
              class = "b")
  ),
  iter = 50000, warmup = 10000, thin = 10, cores = 4, chains = 4,
  control = list(adapt_delta = .96),
```

```
  silent = TRUE, refresh = 0,
  file = "./models/_eightWeekSlopesScrambled.Rds"
)

repeatabilityFrameScrambled =
  as.data.frame(slopes8WeeksScrambled) %>%
  mutate(
    "Vs" = sd_ringScrambled__B_Ta^2 *
      var(slopes8WeeksScrambled$data$Ta) +
      mean(slopes8WeeksScrambled$data$Ta)^2 *
      sd_ringScrambled__B_Ta^2,
    "Vf" = b_B_Ta^2 * var(slopes8WeeksScrambled$data$Ta)
  ) %>%
  mutate("Slope" = Vs / (Vf + exp(b_sigma_Intercept)^2 +
         exp(b_sigma_Intercept + b_sigma_discreteTaB)^2)
         ) %>%
  dplyr::select(Slope)

repeatPlot8Weeks <- rbind(
  repeatabilityFrame %>%
    mutate("Type" = "True"),
  repeatabilityFrameScrambled %>%
    mutate("Type" = "Null")
) %>%
  pivot_longer(!c("Type"), names_to = "Par",
               values_to = "Repeatability") %>%
  mutate(Par = "Individual Slope") %>%
  ggplot(aes(x = Repeatability, fill = Type)) +
  geom_density(colour = "black", adjust = 1, alpha = 0.7) +
  scale_fill_manual(
    values = c("grey20", "#BBD8F0"),
    labels = c("Null Model", "True Model"),
    name = NULL
  ) +
  ylab("Density") +
  theme_classic() +
  theme(axis.text = element_text(family = "Noto Sans"),
        legend.text = element_text(family = "Noto Sans")
        )

showtext_auto()
repeatPlot8Weeks
```

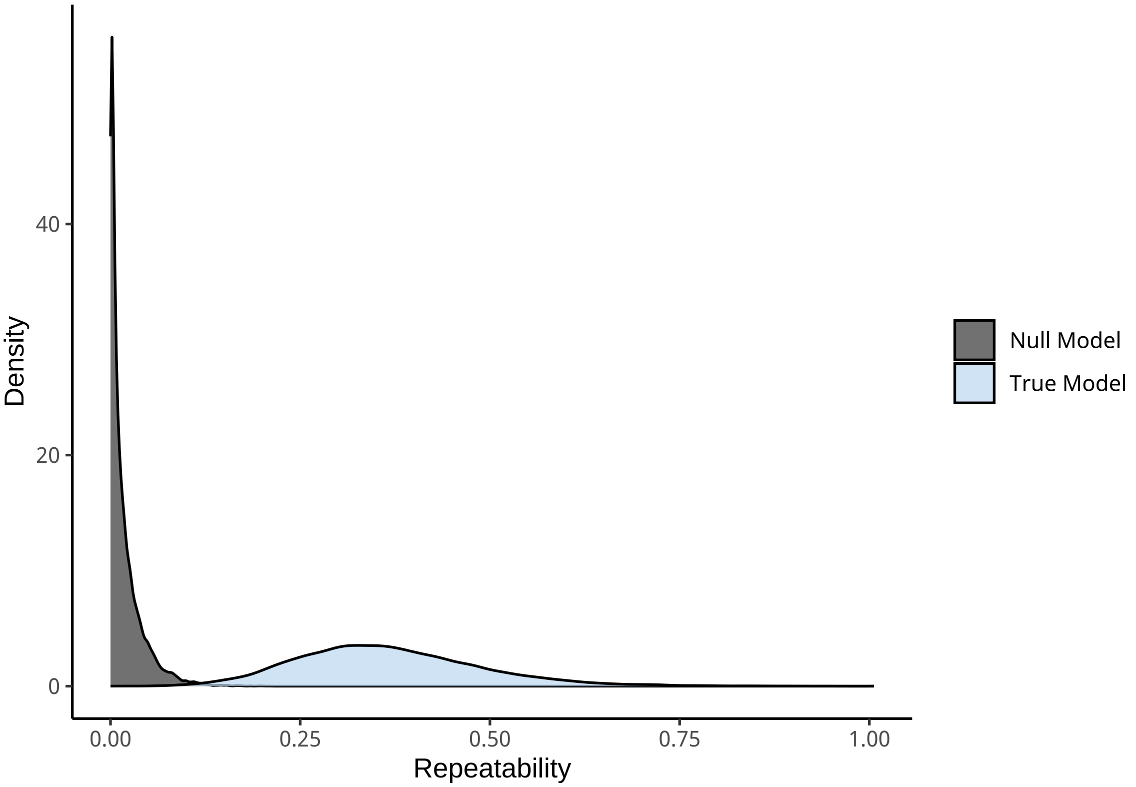

Figure 89: *Density of conditional repeatabilities calculated from coefficients of a Bayesian, linear mixed-effects model predicting relative resting metabolism (fold metabolism at thermoneutrality) across ambient temperature (10°C - 24°C) in eight week old Japanese quail. Light grey densities represent true repeatability estimates and dark grey densities represent those calculated from models with scrambled individual identities (null model). All repeatabilities are calculated as per Schielzeth and Nakagawa (2022).*

```
ggsave("./plots/traitRepeatability8Weeks_2.pdf",
  repeatPlot8Weeks,
  dpi = 800, height = 5, width = 7.5
)
showtext_auto(enable = FALSE)

# Formally testing possible distinctions.
```

```
u1RepeatabilityTestFrame <- build_hdf(
  vars = list(
    repeatabilityFrame$Slope,
    repeatabilityFrameScrambled$Slope
  ),
  priors = list(
    rbeta(nrow(repeatabilityFrame), 1, 4),
    rbeta(nrow(repeatabilityFrameScrambled), 1, 4)
  ),
  names = c("True", "Null")
)

u1RepeatabilityTest <- hypothesis_df("True > Null",
                                     u1RepeatabilityTestFrame,
  class = "b", alpha = 0.05
)

u1RepeatabilityTest$hypothesis$Hypothesis <- paste0(
  "Slope: ",
  u1RepeatabilityTest$hypothesis$Hypothesis
```

```
)

# Visualising and summarising outcomes

repeatTestPlot <-
    data.frame(
      "values" = c(
        u1RepeatabilityTest$samples$H1,
        u1RepeatabilityTest$prior_samples$H1
      ),
      "Type" = c(
        rep("Posterior", length(u1RepeatabilityTest$samples$H1)),
        rep("Prior", length(u1RepeatabilityTest$prior_samples$H1))
      ),
      "Hypothesis" = "Slope Repeatability > Null"
    ) %>%
  ggplot(aes(x = values, fill = Type)) +
  facet_wrap(~Hypothesis) +
  geom_density(colour = "black", alpha = 0.4) +
  geom_vline(xintercept = 0, colour = "black", linetype = "longdash") +
  scale_fill_manual(values = c("black", "grey80")) +
  xlab("True - Null Repeatability") +
  ylab("Density") +
  theme_classic()

repeatTestPlot
```

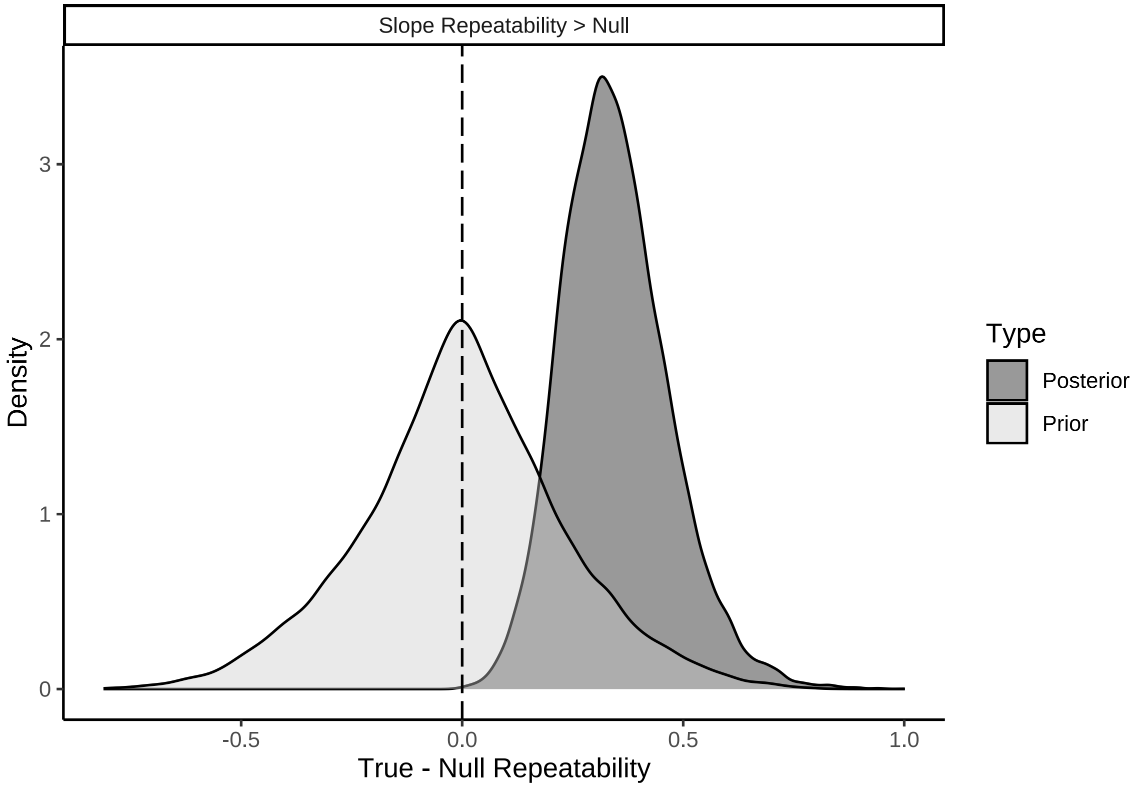

Figure 90: *Differences between true and null repeatability estimates for individual-level slopes of relative resting metabolism (fold metabolism at thermoneutrality) by ambient temperature (°C; between 10-24°C) curves in eight week old Japanese quail.*

```
caption <- paste0("Results of two, non-linear hypothesis ",
                  "tests comparing true and null repeatabilities of ",
                  "individual-level slopes from relative ",
```

```
                 "resting metabolism (fold metabolism at thermoneutrality) ",
                 "by ambient temperature (°C; ",
                 "10-24°C) curves. ",
                 "Curves are calculated for eight week old ",
                 "Japanese quail. Posterior probabilities are ",
                 "calculated using the Savage-Dickey density ratio method."
)

repeatabilityTest8Weeks <-
  u1RepeatabilityTest$hypothesis %>%
  mutate(Hypothesis =
    "True Slope Repeatability > Null"
    ) %>%
  select(-c(Evid.Ratio, Star)) %>%
  mutate(Estimate = round(Estimate, digits = 4),
         "Est.Error" = round(Est.Error, digits = 4),
         "CI.Lower" = round(CI.Lower, digits = 4),
         "CI.Upper" = round(CI.Upper, digits = 4)) %>%
  rename(
    "Difference Between True and Null Repeatabilities" = Estimate,
    "Standard Error" = Est.Error,
    `2.5\\% CI` = CI.Lower,
    `97.5\\% CI` = CI.Upper,
    "Posterior Probability" = Post.Prob
  ) %>%
  kbl(.,
    longtable = T, booktabs = T, format = "latex",
    caption = caption, escape = FALSE
  ) %>%
  column_spec(column = c(1:10), width = "2.1cm")  %>%
  kable_styling(latex_options = "striped")

repeatabilityTest8Weeks
```

Table 38: *Results of two, non-linear hypothesis tests comparing true and null repeatabilities of individual-level slopes from relative resting metabolism (fold metabolism at thermoneutrality) by ambient temperature (°C; 10-24°C) curves. Curves are calculated for eight week old Japanese quail. Posterior probabilities are calculated using the Savage-Dickey density ratio method.*

Hypothesis	Difference Between True and Null Repeatabilities	Standard Error	2.5% CI	97.5% CI	Posterior Probability
True Slope Repeatability > Null	0.3475	0.1209	0.1686	0.5603	0.999875

```
#save_kable(repeatabilityTest8Weeks,
# "../tables/VO2RepeatabilityTest8Weeks.html")
```

Finally, all metabolic slope values are extracted from models and appended to a data frame for use in subsequent analyses.

```
u1sTrue <- rbind(
  as.data.frame(slopes3Weeks) %>%
    select(c(contains("r_ring") |
             contains("b_B_Ta"))
           ) %>%
    mutate_at(vars(contains("r_ring")), ~ b_B_Ta + .) %>%
    select(-b_B_Ta) %>%
    summarise_all(.funs = median) %>%
    pivot_longer(everything(), names_to = "ring",
                 values_to = "mslope") %>%
```

```
    mutate(
      "ring" = gsub(
        "\\,.*", "",
        gsub(".*\\[", "", ring)
      ),
      "week" = "3"
    ),
  as.data.frame(slopes8Weeks) %>%
    select(c(contains("r_ring") |
             contains("b_B_Ta"))
           ) %>%
    mutate_at(vars(contains("r_ring")), ~ b_B_Ta + .) %>%
    select(-b_B_Ta) %>%
    summarise_all(.funs = median) %>%
    pivot_longer(everything(), names_to = "ring",
                 values_to = "mslope") %>%
    mutate(
      "ring" = gsub(
        "\\,.*", "",
        gsub(".*\\[", "", ring)
      ),
      "week" = "8"
    )
)

u1sRaw <-  rbind(
  as.data.frame(slopes3Weeks) %>%
    select(contains("r_ring")) %>%
    summarise_all(.funs = median) %>%
    pivot_longer(everything(), names_to = "ring",
                 values_to = "rawSlope") %>%
    mutate(
      "ring" = gsub(
        "\\,.*", "",
        gsub(".*\\[", "", ring)
      ),
      "week" = "3"
    ),
    as.data.frame(slopes8Weeks) %>%
    select(contains("r_ring")) %>%
    summarise_all(.funs = median) %>%
    pivot_longer(everything(), names_to = "ring",
                 values_to = "rawSlope") %>%
    mutate(
      "ring" = gsub(
        "\\,.*", "",
        gsub(".*\\[", "", ring)
      ),
      "week" = "8"
    )
)

# Note that ring identities are corrected to bind properly

slopeData <-
  merge(u1sTrue, u1sRaw, by = c("ring", "week")) %>%
  mutate(ring = gsub("\\.", " ", ring)) %>%
  merge(
    all %>%
      filter(Ta < 35 & week %in% c(3, 8)) %>%
      distinct(ring, week, mass, tarsusLengthMean, billLengthMean, pretreatment),
    .,
    by = c("ring", "week"), all = TRUE
  )

# Summarising slope values
```

```
caption = paste0("Summary of metabolic slope values estimated for ",
                 "captive reared Japanese quail.")

slopeData %>%
  group_by(week) %>%
  summarise("Mean" = mean(mslope, na.rm = T),
    "SD" = sd(rawSlope, na.rm = T)
  ) %>%
  mutate("CV" = abs((SD/Mean))*100,
    "Parameter" = "Metabolic Slope (RMR/°C)"
  ) %>%
  dplyr::select(Parameter, "Age (weeks)" = week, Mean, "S.D." = SD, "CV (\\%)" = CV) %>%
  kbl(.,
    longtable = T, booktabs = T, format = "latex",
    caption = caption, escape = FALSE
  ) %>%
  column_spec(column = c(1:10), width = "2.1cm")  %>%
  kable_styling(latex_options = "striped")
```

Table 39: *Summary of metabolic slope values estimated for captive reared Japanese quail.*

Parameter	Age (weeks)	Mean	S.D.	CV (%)
Metabolic Slope (RMR/°C)	3	-0.0306060	0.0095244	31.11957
Metabolic Slope (RMR/°C)	8	-0.0399378	0.0185398	46.42174

Analysing effects of morphometry on metabolic slopes in the cold

In this subsection, we test whether and how body mass and appendage length affect metabolic slopes of Japanese quail in the cold. These potential effects are contrasted against those of thermal history (here, rearing temperature treatment). Our conceptual model for this subsection is therefore as shown below.

```
data.frame(x = c(-5:5), y = c(-5:5)) %>%
  ggplot(aes(x = x, y = y)) +
  annotate("text", x = 0, y = 5, label = paste0("Developmental, ",
           "Thermal\nEnvironment"), colour = "black") +
  annotate("text", x = -2.5, y = 2.5, label = "Body Mass (g)",
           colour = "black") +
  annotate("text", x = 2.5, y = 2.5, label = "Appendage Length\n(mm)",
           colour = "black") +
  annotate("text", x = 0, y = -0.1,
           label = "Metabolic Slope (fold RMR/°C)",
           colour = "black") +
  geom_segment(
    lineend = "round", linejoin = "round",
    size = 0.3, linetype = "solid", colour = "grey10",
    aes(x = -0.25, y = 4.6, xend = -2.5, yend = 2.75),
    arrow = arrow(length = unit(0.2, "cm"))
  ) +
  geom_segment(
    lineend = "round", linejoin = "round",
    size = 0.3, linetype = "solid", colour = "grey10",
    aes(x = 0.25, y = 4.6, xend = 2.5, yend = 2.75),
    arrow = arrow(length = unit(0.2, "cm"))
  ) +
  geom_segment(
    lineend = "round", linejoin = "round",
    size = 0.3, linetype = "solid", colour = "grey10",
    aes(x = -1.25, y = 2.5, xend = 1, yend = 2.5),
    arrow = arrow(length = unit(0.2, "cm"))
  ) +
  geom_segment(
```

```
    lineend = "round", linejoin = "round",
    size = 0.3, linetype = "dotted", colour = "grey10",
    aes(x = -2.5, y = 2.25, xend = -0.25, yend = 0.25),
    arrow = arrow(length = unit(0.2, "cm"))
  ) +
  geom_segment(
    lineend = "round", linejoin = "round",
    size = 0.3, linetype = "longdash", colour = "grey10",
    aes(x = 2.5, y = 2.25, xend = 0.25, yend = 0.25),
    arrow = arrow(length = unit(0.2, "cm"))
  ) +
  geom_curve(
    lineend = "round",
    size = 0.3, linetype = "solid", colour = "grey10",
    aes(x = -1.75, y = 4.75, xend = -1.75, yend = 0.25),
    arrow = arrow(length = unit(0.2, "cm"))
  ) +
  xlim(c(-6, 6)) +
  ylim(c(-0.5, 6)) +
  theme_void()
```

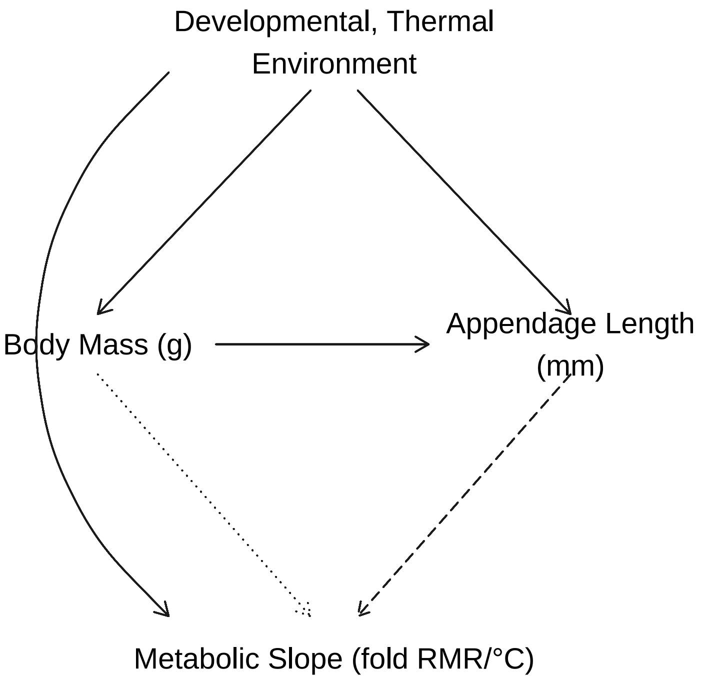

Figure 91: *Flow-chart describing expected effects of developmental, thermal environment on morphology, and subsequently, thermal physiology of Japanese quail. Dashed and dotted lines represent effects predicted under a adaptive hypotheses of Allen's rule and Bergmann's rule respectively.*

Adaptive hypotheses of Bergmann's and Allen's rule predict direct effects of body size (here, body mass) and appendage length on metabolic slopes. However, appendage length is likely to be correlated with body mass, and each are clearly influenced by the thermal environment during rearing (shown in Tabh et al 2025). For these reasons, rather than analysing effects of body mass or appendage length on metabolic slopes independently, we chose to analyse each as part of a comprehensive path analysis, terminating in metabolic slope. This path analysis was composed of four separate models as follows:

$$Body\ Mass_j \sim \beta_{a0} + \beta_{a1} \cdot Cold\ Rearing_j + \beta_{a2} \cdot Warm\ Rearing_j + \mu_{0a} + \epsilon_a$$

$$Tarsus\ Length_j \sim \beta_{b0} + \beta_{b1} \cdot Cold\ Rearing_j + \beta_{b2} \cdot Warm\ Rearing_j + \beta_{b3} \cdot Body\ Mass_j +$$
$$\mu_{0b} + \epsilon_b$$
$$Bill\ Length_j \sim \beta_{c0} + \beta_{c1} \cdot Cold\ Rearing_j + \beta_{c2} \cdot Warm\ Rearing_j + \beta_{c3} \cdot Body\ Mass_j +$$
$$\mu_{0c} + \epsilon_c$$

and:

$$Metabolic\ Slope_j \sim \beta_{d0} + \beta_{d1} \cdot Cold\ Rearing_j + \beta_{d2} \cdot Warm\ Rearing_j + \beta_{d3} \cdot Body\ Mass_j +$$
$$\beta_{d4} \cdot Tarsus\ Length_j + \beta_{d5} \cdot Bill\ Length_j + \mu_{0d} + \epsilon_d$$

with j representing individual (and thus, observation) identity, "warm rearing" and "cold rearing" representing logical terms with "0" corresponding to "false" and "1" corresponding to "true", μ_{0a} - μ_{0c} representing group-level intercepts, per response variable, of egg batch, and body mass, bill length, and tarsus length measures being mean-centred to simplify interpretation of model intercepts (β_0 terms). Error terms (ϵ) were normally distributed with a mean of zero for each model, and residuals for all models were assumed to be uncorrelated with each response values.

Since we were interested in testing age effects on the functional significance of Bergmann's and Allen's rules, we ran our path analysis for data drawn from both three week and eight week old Japanese quail, then compare outcomes thereafter. This approach was chosen to avoid over-parameterising a single model.

Modelling effects in juveniles

Below, we begin by constructing and running our path analysis for data from juvenile quail (three weeks old). Priors for each model parameter here were moderately informative and as follows:

$$\beta_{a0} \sim \mathcal{N}(0, 5)$$
$$\beta_{a1} \sim \mathcal{N}(0, 15)$$
$$\beta_{a2} \sim \mathcal{N}(0, 15)$$
$$\mu 0a \sim \exp(2.5)$$
$$\epsilon_a \sim \exp(0.15)$$

$$\beta_{b0} \sim \mathcal{N}(0, 2.5)$$
$$\beta_{b1} \sim \mathcal{N}(0, 2.5)$$
$$\beta_{b2} \sim \mathcal{N}(0, 2.5)$$
$$\beta_{b3} \sim S\mathcal{N}(0, 0.25, 5)$$
$$\mu 0b \sim \exp(2)$$
$$\epsilon_b \sim \exp(1)$$

$$\beta_{c0} \sim \mathcal{N}(0, 1)$$
$$\beta_{c1} \sim \mathcal{N}(0, 0.5)$$

$$\beta_{c2} \sim \mathcal{N}(0, 0.5)$$
$$\beta_{c3} \sim S\mathcal{N}(0, 0.25, 5)$$
$$\mu 0c \sim \exp(5)$$
$$\epsilon_c \sim \exp(2.5)$$

$$\beta_{d0} \sim \mathcal{N}(0, 0.01)$$
$$\beta_{d1} \sim \mathcal{N}(0, 0.025)$$
$$\beta_{d2} \sim \mathcal{N}(0, 0.025)$$
$$\beta_{d3} \sim \mathcal{N}(0, 0.001)$$
$$\beta_{d4} \sim \mathcal{N}(0, 0.0025)$$
$$\beta_{d5} \sim \mathcal{N}(0, 0.001)$$
$$\mu 0d \sim \exp(25)$$
$$\epsilon_d \sim \exp(10)$$

with distributions for group-level effects of egg batch on body mass, tarsus length and bill length length derived from growth models described in the previous section of this document (2.0).

Suitability of priors is examined using prior predictive checks (as described above).

```
# Testing how appendage length and body mass predict metabolic slopes
# Beginning with prior predictive check

functionModel3WeeksPPCheck <-
  brm(
    data = slopeData %>%
      filter(week == "3") %>%
      mutate(
        mass = mass - mean(mass, na.rm = T),
        tarsus = tarsusLengthMean - mean(tarsusLengthMean, na.rm = T),
        bill = billLengthMean - mean(billLengthMean, na.rm = T),
        pretreatment = ifelse(pretreatment == "cold", "A",
          ifelse(pretreatment == "neutral", "B", "C")
        )
      ) %>%
      mutate(pretreatment = factor(pretreatment, levels = c("B", "A", "C"))) %>%
      drop_na() %>%
      merge(., data %>%
        dplyr::select(ring, "batch" = exp) %>%
        distinct(),
      by = "ring", all.x = TRUE
      ),
    family = "gaussian",
    bf(mass ~ pretreatment + (1 | batch)) +
      bf(tarsus ~ mass + pretreatment + (1 | batch)) +
      bf(bill ~ mass + pretreatment + (1 | batch)) +
      bf(rawSlope ~ tarsus + bill + mass + pretreatment + (1 | batch)) +
      set_rescor(FALSE),
        prior = c(
      set_prior("normal(0, 5)",
        class = "Intercept",
        resp = "mass"
      ),
      set_prior("normal(0, 15)",
        class = "b",
```

```
      coef = "pretreatmentA",
      resp = "mass"
    ),
    set_prior("normal(0, 15)",
      class = "b",
      coef = "pretreatmentC",
      resp = "mass"
    ),
    set_prior("exponential(2.5)",
      class = "sd",
      group = "batch",
      resp = "mass"
    ),
    set_prior("exponential(0.15)",
      class = "sigma",
      resp = "mass"
    ),
    set_prior("normal(0, 2.5)",
      class = "Intercept",
      resp = "tarsus"
    ),
    set_prior("normal(0, 2.5)",
      class = "b",
      coef = "pretreatmentA",
      resp = "tarsus"
    ),
    set_prior("normal(0, 2.5)",
      class = "b",
      coef = "pretreatmentC",
      resp = "tarsus"
    ),
    set_prior("skew_normal(0, 0.25, 5)",
      class = "b",
      coef = "mass",
      resp = "tarsus"
    ),
    set_prior("exponential(2)",
      class = "sd",
      group = "batch",
      resp = "tarsus"
    ),
    set_prior("exponential(1)",
      class = "sigma",
      resp = "tarsus"
    ),
    set_prior("normal(0, 1)",
      class = "Intercept",
      resp = "bill"
    ),
    set_prior("normal(0, 0.5)",
      class = "b",
      coef = "pretreatmentA",
      resp = "bill"
    ),
    set_prior("normal(0, 0.5)",
      class = "b",
      coef = "pretreatmentC",
      resp = "bill"
    ),
    set_prior("skew_normal(0, 0.25, 5)",
      class = "b",
      coef = "mass",
      resp = "bill"
    ),
    set_prior("exponential(5)",
      class = "sd",
      group = "batch", resp = "bill"
```

```
      ),
      set_prior(
        "exponential(2.5)",
        class = "sigma",
        resp = "bill"
      ),
      set_prior("normal(0, 0.01)",
        class = "Intercept",
        resp = "rawSlope"
      ),
      set_prior("normal(0, 0.025)",
        class = "b",
        coef = "pretreatmentA",
        resp = "rawSlope"
      ),
      set_prior("normal(0, 0.025)",
        class = "b",
        coef = "pretreatmentC",
        resp = "rawSlope"
      ),
      set_prior("normal(0, 0.001)",
        class = "b",
        coef = "mass",
        resp = "rawSlope"
      ),
      set_prior("normal(0, 0.0025)",
        class = "b",
        coef = "tarsus",
        resp = "rawSlope"
      ),
      set_prior("normal(0, 0.001)",
        class = "b",
        coef = "bill",
        resp = "rawSlope"
      ),
      set_prior("exponential(50)",
        class = "sd",
        group = "batch",
        resp = "rawSlope"
      ),
      set_prior("exponential(10)",
        class = "sigma",
        resp = "rawSlope"
      )
    ),
    iter = 50000, warmup = 10000, cores = 4, chains = 4, thin = 20,
    control = list(adapt_delta = .97, max_treedepth = 14),
    silent = TRUE, refresh = 0,
    sample_prior = "only",
    file = "./models/_threeWeekFunctionModelPPCheck.Rds"
  )

(
  pp_check2(functionModel3WeeksPPCheck,
    resp = "mass",
    xlab = "Relative Body Mass (g)"
  ) +
    pp_check2(functionModel3WeeksPPCheck,
      resp = "tarsus",
      xlab = "Relative Tarsus Length (mm)"
    )
) /
  (
    pp_check2(functionModel3WeeksPPCheck,
      resp = "bill",
      xlab = "Relative Bill Length (mm)"
    ) +
```

```
    pp_check2(functionModel3WeeksPPCheck,
      resp = "rawSlope",
      xlab = "Metabolic Slope (fold RMR/°C)"
    )
)
```

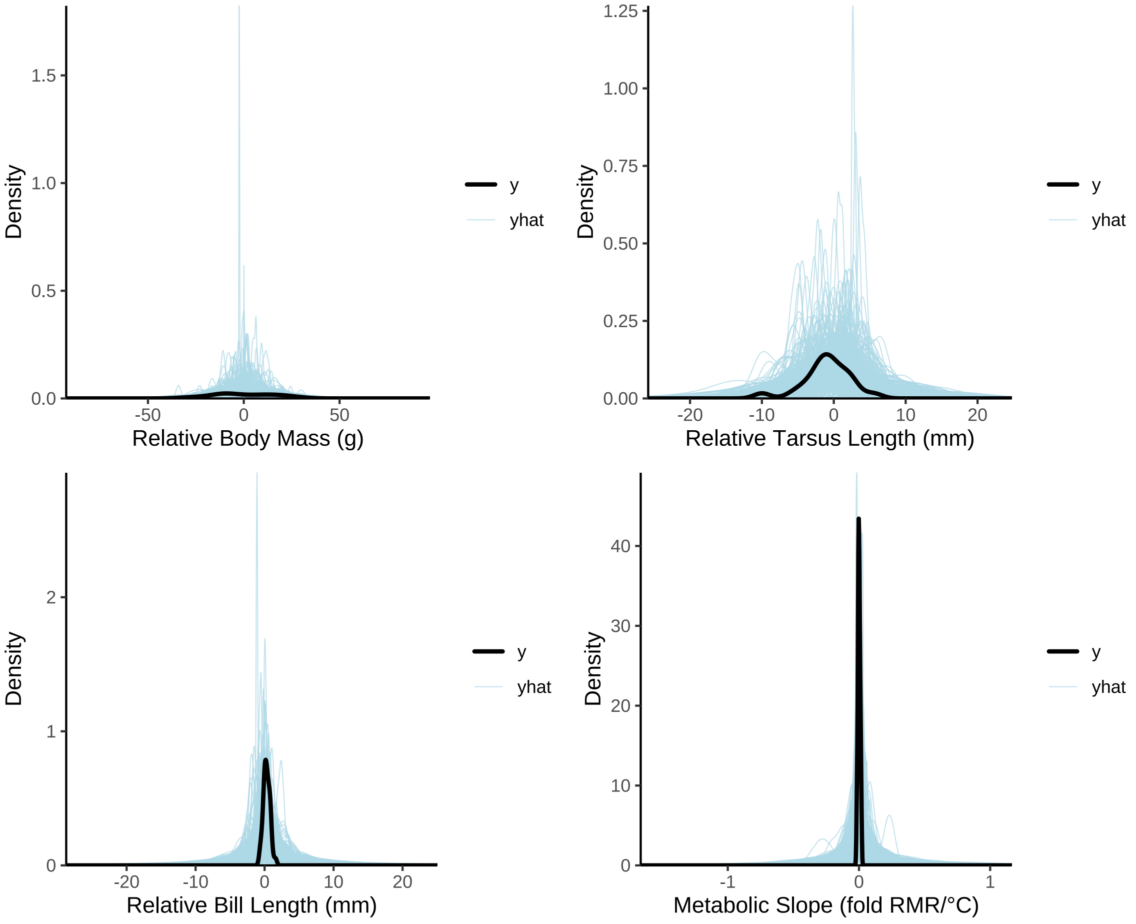

Figure 92: *Prior predictive check for a Bayesian path analysis predicting metabolic slope in the cold (fold metabolism at thermoneutrality/°C) as a direct and indirect function of body mass (g), tarsus length (mm), and bill length (mm) in three week old Japanese quail. Light blue lines represent densities of resting metabolism values as predicted by model priors alone. The dark blue line represents the true density of resting metabolism values. Clear overlap between the dark blue and light blue lines indicates that priors are suitable.*

Priors are clearly suitable and broad. We therefore continue to full model construction.

```
functionModel3Weeks <-
  brm(
    data = slopeData %>%
      filter(week == "3") %>%
      mutate(
        mass = mass - mean(mass, na.rm = T),
        tarsus = tarsusLengthMean - mean(tarsusLengthMean, na.rm = T),
        bill = billLengthMean - mean(billLengthMean, na.rm = T),
        pretreatment = ifelse(pretreatment == "cold", "A",
          ifelse(pretreatment == "neutral", "B", "C")
        )
      ) %>%
      mutate(pretreatment = factor(pretreatment, levels = c("B", "A", "C"))) %>%
```

```
      drop_na() %>%
      merge(., data %>%
        dplyr::select(ring, "batch" = exp) %>%
        distinct(),
      by = "ring", all.x = TRUE
      ),
    family = "gaussian",
    bf(mass ~ pretreatment + (1 | batch)) +
      bf(tarsus ~ mass + pretreatment + (1 | batch)) +
      bf(bill ~ mass + pretreatment + (1 | batch)) +
      bf(rawSlope ~ tarsus + bill + mass + pretreatment + (1 | batch)) +
      set_rescor(FALSE),
    prior = c(
      set_prior("normal(0, 5)",
        class = "Intercept",
        resp = "mass"
      ),
      set_prior("normal(0, 15)",
        class = "b",
        coef = "pretreatmentA",
        resp = "mass"
      ),
      set_prior("normal(0, 15)",
        class = "b",
        coef = "pretreatmentC",
        resp = "mass"
      ),
      set_prior("exponential(2.5)",
        class = "sd",
        group = "batch",
        resp = "mass"
      ),
      set_prior("exponential(0.15)",
        class = "sigma",
        resp = "mass"
      ),
      set_prior("normal(0, 2.5)",
        class = "Intercept",
        resp = "tarsus"
      ),
      set_prior("normal(0, 2.5)",
        class = "b",
        coef = "pretreatmentA",
        resp = "tarsus"
      ),
      set_prior("normal(0, 2.5)",
        class = "b",
        coef = "pretreatmentC",
        resp = "tarsus"
      ),
      set_prior("skew_normal(0, 0.25, 5)",
        class = "b",
        coef = "mass",
        resp = "tarsus"
      ),
      set_prior("exponential(2)",
        class = "sd",
        group = "batch",
        resp = "tarsus"
      ),
      set_prior("exponential(1)",
        class = "sigma",
        resp = "tarsus"
      ),
      set_prior("normal(0, 1)",
        class = "Intercept",
        resp = "bill"
```

```
      ),
      set_prior("normal(0, 0.5)",
        class = "b",
        coef = "pretreatmentA",
        resp = "bill"
      ),
      set_prior("normal(0, 0.5)",
        class = "b",
        coef = "pretreatmentC",
        resp = "bill"
      ),
      set_prior("skew_normal(0, 0.25, 5)",
        class = "b",
        coef = "mass",
        resp = "bill"
      ),
      set_prior("exponential(5)",
        class = "sd",
        group = "batch",
        resp = "bill"
      ),
      set_prior("exponential(2.5)",
        class = "sigma",
        resp = "bill"
      ),
      set_prior("normal(0, 0.01)",
        class = "Intercept",
        resp = "rawSlope"
      ),
      set_prior("normal(0, 0.025)",
        class = "b",
        coef = "pretreatmentA",
        resp = "rawSlope"
      ),
      set_prior("normal(0, 0.025)",
        class = "b",
        coef = "pretreatmentC",
        resp = "rawSlope"
      ),
      set_prior("normal(0, 0.001)",
        class = "b",
        coef = "mass",
        resp = "rawSlope"
      ),
      set_prior("normal(0, 0.0025)",
        class = "b",
        coef = "tarsus",
        resp = "rawSlope"
      ),
      set_prior("normal(0, 0.001)",
        class = "b",
        coef = "bill",
        resp = "rawSlope"
      ),
      set_prior("exponential(50)",
        class = "sd",
        group = "batch",
        resp = "rawSlope"
      ),
      set_prior("exponential(10)",
        class = "sigma",
        resp = "rawSlope"
      )
    ),
    iter = 50000, warmup = 10000, cores = 4, chains = 4, thin = 20,
    control = list(adapt_delta = .97, max_treedepth = 14),
    silent = TRUE, refresh = 0,
```

```
    file = "./models/_threeWeekFunctionModel.Rds"
  )

# Some divergent transitions, but relatively minimal.

chainCheck(functionModel3Weeks)
```

```
## Rhat range: 1 - 1.001
## Neff/N range: 0.862 - 1.017
```

```
# Chains converged and loss to autocorrelation within chains minimal. R2 values?

caption <- paste0(
  "R\\textsuperscript{2} for Bayesian ",
  "path analysis predicting metabolic slopes ",
  "(fold metabolism at thermoneutrality/°C) ",
  "of three week old Japanese quail as a function ",
  "of body mass (g), tarsus length (mm), and bill length (mm). ",
  "Metabolic slopes are measured below thermoneutrality ",
  "(<30°C)."
)

brms::bayes_R2(functionModel3Weeks, ndraws = 1000,
               robust = TRUE) %>%
  as.data.frame() %>%
  rownames_to_column("var") %>%
  merge(., tribble(
    ~var, ~Var,
    "R2mass", "Body Mass (g)",
    "R2tarsus", "Tarsus Length (mm)",
    "R2bill", "Bill Length (mm)",
    "R2rawSlope",
    "Metabolic Slope (fold RMR/°C)"
  ), by = c("var")) %>%
  mutate(Estimate = round(Estimate, digits = 4),
         Est.Error = round(Est.Error, digits = 4),
         Q2.5 = round(Q2.5, digits = 4),
         Q97.5 = round(Q97.5, digits = 4),
         Var = factor(Var, levels = c("Body Mass (g)", "Tarsus Length (mm)",
                                      "Bill Length (mm)",
                                      "Metabolic Slope (fold RMR/°C)")
                      )
         ) %>%
  dplyr::select(
    "Response" = Var, "R\\textsuperscript{2}" = Estimate,
    "Standard Error" = Est.Error,
    `2.5\\% CI` = Q2.5, `97.5\\% CI` = Q97.5
  ) %>%
  arrange(Response) %>%
  kbl(.,
    longtable = T, booktabs = T, format = "latex",
    caption = caption, escape = FALSE
  ) %>%
  column_spec(column = 1, width = "3.5cm")  %>%
  column_spec(column = c(2:10), width = "2.25cm") %>%
  kable_styling(latex_options = "striped")
```

Table 40: *R^2 for Bayesian path analysis predicting metabolic slopes (fold metabolism at thermoneutrality/°C) of three week old Japanese quail as a function of body mass (g), tarsus length (mm), and bill length (mm). Metabolic slopes are measured below thermoneutrality (<30°C).*

Response	R^2	Standard Error	2.5% CI	97.5% CI
Body Mass (g)	0.0794	0.0626	0.0042	0.2308
Tarsus Length (mm)	0.3832	0.0979	0.1723	0.5434
Bill Length (mm)	0.1272	0.0754	0.0215	0.2903

Metabolic Slope (fold RMR/°C)	0.1798	0.0815	0.0500	0.3464

```
pp_check2(functionModel3Weeks, resp = "rawSlope",
          xlab = "Metabolic Slope (fold RMR/°C)"
          )
```

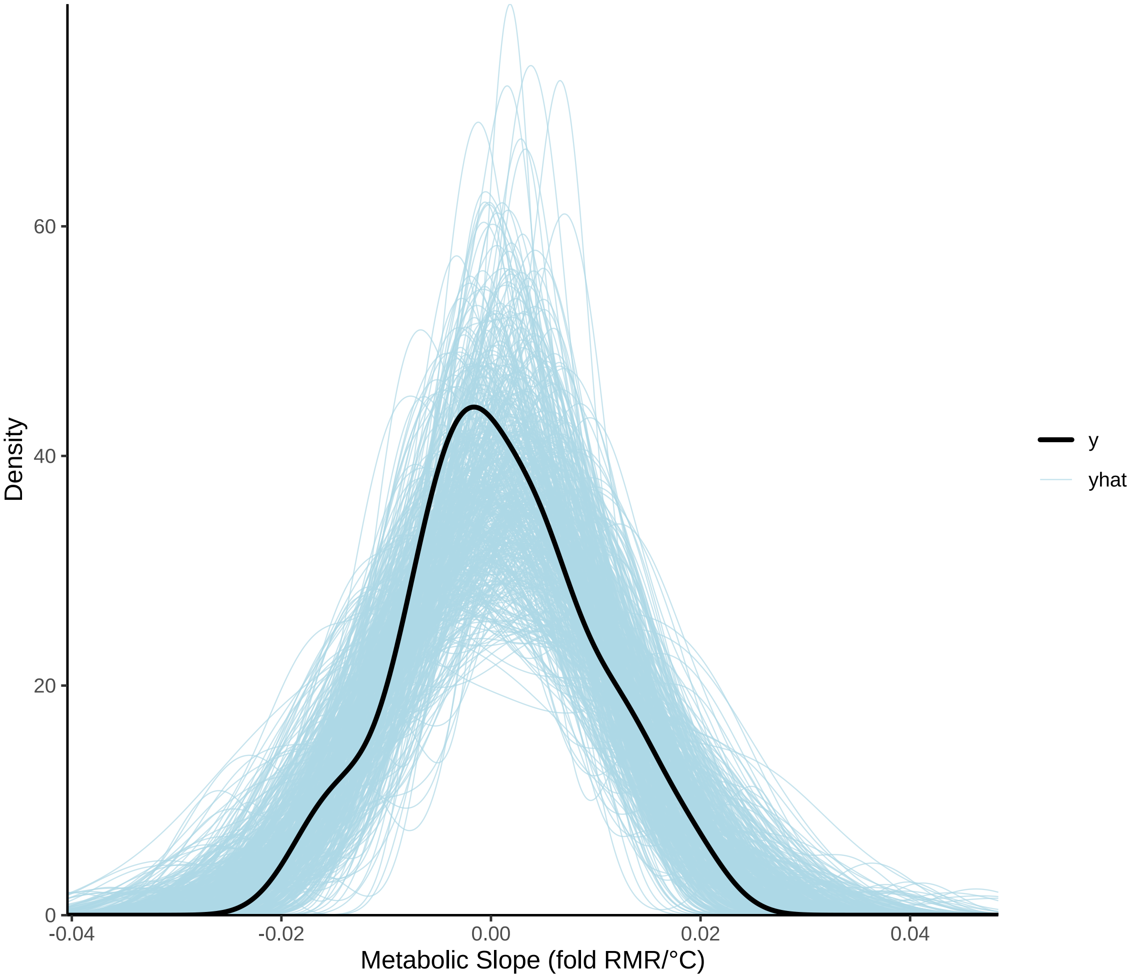

Figure 93: *Densities of posterior predictions from a Bayesian path analysis predicting metabolic slopes (fold metabolism at thermoneutrality/°C) as a direct and indirect function of body mass (g), tarsus length (mm), and bill length (mm) in three week old Japanese quail. Prediction are overlayed with true distributions of quail metabolic slopes. Blue lines represent predictions from posterior draws, while the black line represents true, mean-centred resitance density.*

Residuals of this model are visualised as before.

```
p1 <- functionModel3Weeks$data %>%
  mutate("Residuals" = residuals(functionModel3Weeks,
    resp = "rawSlope",
    robust = TRUE
  )[, "Estimate"]) %>%
  ggplot(aes(x = mass, y = Residuals)) +
  geom_point(size = 2, pch = 21, colour = "black", fill = "grey50") +
  xlab("Relative Body\nMass (g)") +
  ylab("Ordinary Residuals") +
```

```
  theme_classic()

p2 <- functionModel3Weeks$data %>%
  mutate("Residuals" = residuals(functionModel3Weeks,
    resp = "rawSlope",
    robust = TRUE
  )[, "Estimate"]) %>%
  ggplot(aes(x = tarsus, y = Residuals)) +
  geom_point(size = 2, pch = 21, colour = "black", fill = "grey50") +
  xlab("Relative Tarsus\nLength (mm)") +
  ylab("Ordinary Residuals") +
  theme_classic()

p3 <- functionModel3Weeks$data %>%
  mutate("Residuals" = residuals(functionModel3Weeks,
    resp = "rawSlope",
    robust = TRUE
  )[, "Estimate"]) %>%
  ggplot(aes(x = bill, y = Residuals)) +
  geom_point(size = 2, pch = 21, colour = "black", fill = "grey50") +
  xlab("Relative Bill\nLength (mm)") +
  ylab("Ordinary Residuals") +
  theme_classic()

p4 <- functionModel3Weeks$data %>%
  mutate("Residuals" = residuals(functionModel3Weeks,
    resp = "rawSlope",
    robust = TRUE
  )[, "Estimate"]) %>%
  ggplot(aes(x = factor(pretreatment), y = Residuals)) +
  geom_boxplot(width = 0.5) +
  geom_point(
    size = 2, pch = 21,
    position = position_jitter(width = 0.25),
    colour = "black", fill = "grey50"
  ) +
  xlab("Rearing Conditions") +
  ylab("Ordinary Residuals") +
  theme_classic()

p5 <- functionModel3Weeks$data %>%
  mutate(
    "Residuals" = residuals(functionModel3Weeks,
      resp = "rawSlope",
      robust = TRUE
    )[, "Estimate"],
    "Fitted" = fitted(functionModel3Weeks,
      resp = "rawSlope",
      robust = TRUE
    )[, "Estimate"]
  ) %>%
  ggplot(aes(x = Fitted, y = Residuals)) +
  geom_point(size = 2, pch = 21, colour = "black", fill = "grey50") +
  xlab("Fitted Values") +
  ylab("Ordinary Residuals") +
  theme_classic()

p6 <- functionModel3Weeks$data %>%
  mutate("Residuals" = residuals(functionModel3Weeks,
    resp = "rawSlope",
    robust = TRUE
  )[, "Estimate"]) %>%
  ggplot(aes(sample = Residuals)) +
  stat_qq(colour = "grey50") +
  stat_qq_line() +
  xlab("Theoretical") +
  ylab("Sample") +
```

```
  theme_classic()

((p1 + p2 + p3) / (p4 + p5 + p6)) + plot_annotation(tag_levels = "A")
```

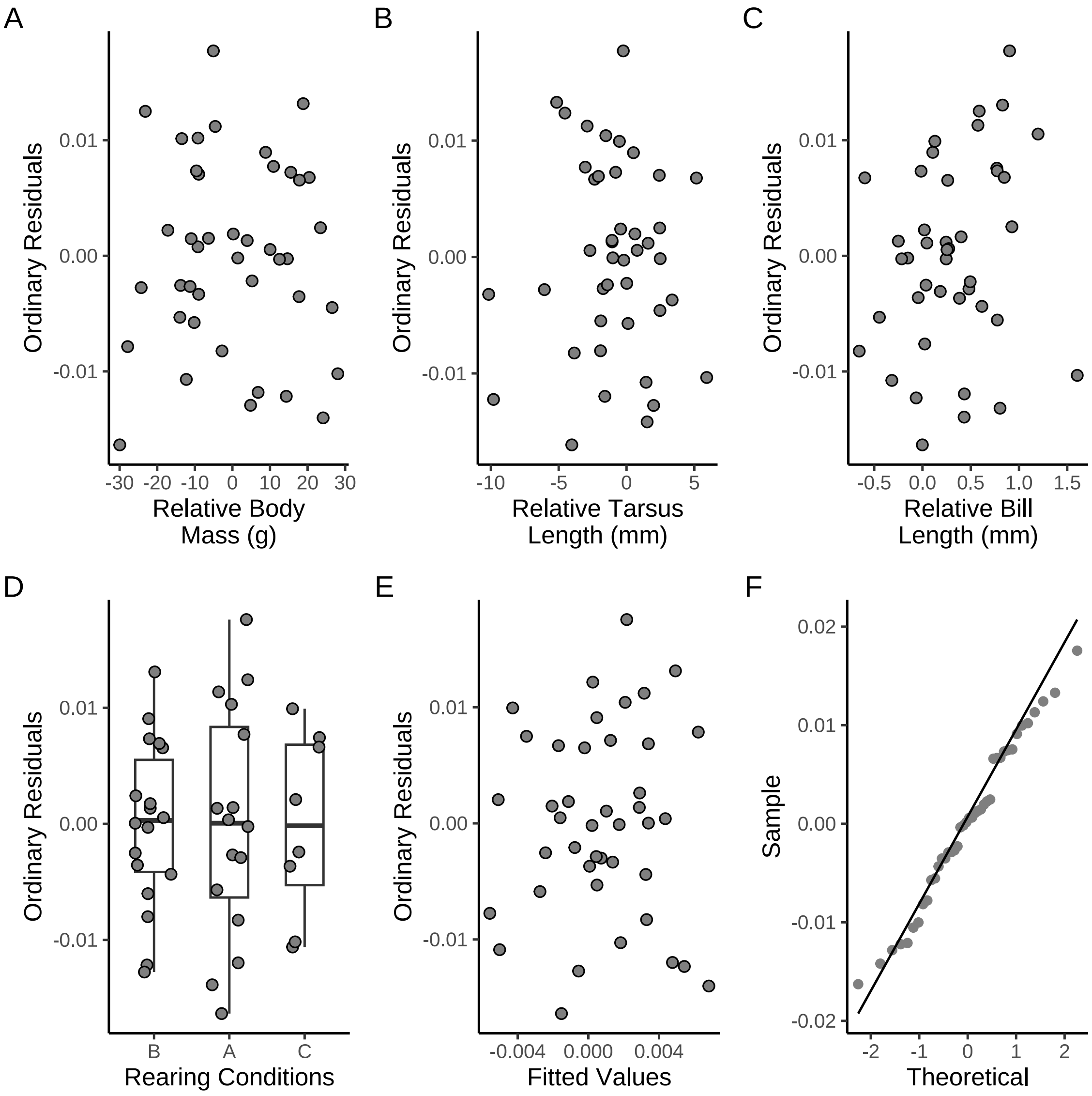

Figure 94: *Ordinary residuals from a Bayesian path analysis predicting metabolic slopes below thermoneutrality (fold metabolism at thermoneutrality/°C) as a direct and indirect function of body mass (g), tarsus length (mm), and bill length (mm) in three week old Japanese quail. Panels A-C displays residuals against mean-centred body mass, tarsus length, and bill length values respectively, panel D displays residuals across post-hatch rearing conditions, panel E displays residuals against fitted, metabolic slope values, and panel F displays theoretical against sample residuals (qq-plot).*

Next, predicted effects of thermal history (here, rearing treatment) on morphology and metabolic slopes are visualised.

```
p1 <- data.frame("pretreatment" = c("A", "B", "C")) %>%
  mutate(
    "mass" = predict(functionModel3Weeks,
      newdata = ., robust = "TRUE",
```

```
      resp = "mass", re_form = NA
    )[, "Estimate"],
    "SE" = predict(functionModel3Weeks,
      newdata = ., robust = "TRUE",
      resp = "mass", re_form = NA
    )[, "Est.Error"]
  ) %>%
  mutate("Mass" = mass +
    mean(subset(slopeData, week == "3")$mass,
      na.rm = T
    )) %>%
  ggplot(aes(x = pretreatment, y = Mass)) +
  geom_errorbar(aes(
    x = pretreatment, ymin = Mass - SE,
    ymax = Mass + SE
  ), colour = "black", width = 0.3) +
  geom_point(
    size = 5, pch = 21, colour = "black",
    aes(x = pretreatment, fill = factor(pretreatment))
  ) +
  geom_line(linetype = "dashed", colour = "black") +
  geom_point(
    size = 2, pch = 21, colour = "black",
    data = subset(slopeData, week == "3") %>%
      mutate("pretreatment" = ifelse(pretreatment == "cold", "A",
        ifelse(pretreatment == "neutral", "B", "C")
      )),
    aes(x = pretreatment, y = mass, fill = factor(pretreatment)),
    position = position_jitter(width = 0.3)
  ) +
  scale_fill_manual(values = c("#7BB4E3", "black", "#CD5C5C")) +
  scale_x_discrete(
    breaks = c("A", "B", "C"),
    labels = c(
      "Cold\n(10°C)",
      "Mild\n(20°C)",
      "Warm\n(30°C)"
    )
  ) +
  theme_classic() +
  theme(
    legend.position = "none",
    axis.text = element_text(family = "Noto Sans"),
    axis.title = element_text(family = "Noto Sans")
  ) +
  xlab("Rearing Conditions") +
  ylab("Mass (g)")

p2 <- data.frame(
  "pretreatment" = c("A", "B", "C"),
  "mass" = 0
) %>%
  mutate(
    "tarsus" = predict(functionModel3Weeks,
      newdata = ., robust = TRUE,
      resp = "tarsus", re_form = NA
    )[, "Estimate"],
    "SE" = predict(functionModel3Weeks,
      newdata = ., robust = TRUE,
      resp = "tarsus", re_form = NA
    )[, "Est.Error"]
  ) %>%
  mutate("Tarsus" = tarsus +
    mean(subset(slopeData, week == "3")$tarsusLengthMean,
      na.rm = T
    )) %>%
  ggplot(aes(x = pretreatment, y = Tarsus)) +
```

```
  geom_errorbar(aes(
    x = pretreatment, ymin = Tarsus - SE,
    ymax = Tarsus + SE
  ), colour = "black", width = 0.3) +
  geom_point(
    size = 5, pch = 21, colour = "black",
    aes(x = pretreatment, fill = factor(pretreatment))
  ) +
  geom_line(linetype = "dashed", colour = "black") +
  geom_point(
    size = 2, pch = 21, colour = "black",
    data = subset(slopeData, week == "3") %>%
      mutate("pretreatment" = ifelse(pretreatment == "cold", "A",
        ifelse(pretreatment == "neutral", "B", "C")
      )),
    aes(
      x = pretreatment, y = tarsusLengthMean,
      fill = factor(pretreatment)
    ),
    position = position_jitter(width = 0.3)
  ) +
  scale_fill_manual(values = c("#7BB4E3", "black", "#CD5C5C")) +
  scale_x_discrete(
    breaks = c("A", "B", "C"),
    labels = c(
      "Cold\n(10°C)",
      "Mild\n(20°C)",
      "Warm\n(30°C)"
    )
  ) +
  theme_classic() +
  theme(
    legend.position = "none",
    axis.text = element_text(family = "Noto Sans"),
    axis.title = element_text(family = "Noto Sans")
  ) +
  xlab("Rearing Conditions") +
  ylab("Tarsus Length (mm)")

p3 <- data.frame(
  "pretreatment" = c("A", "B", "C"),
  "mass" = 0
) %>%
  mutate(
    "bill" = predict(functionModel3Weeks,
      newdata = ., robust = TRUE,
      resp = "bill", re_form = NA
    )[, "Estimate"],
    "SE" = predict(functionModel3Weeks,
      newdata = ., robust = TRUE,
      resp = "bill", re_form = NA
    )[, "Est.Error"]
  ) %>%
  mutate("Bill" = bill +
    mean(subset(slopeData, week == "3")$billLengthMean,
      na.rm = T
    )) %>%
  ggplot(aes(x = pretreatment, y = Bill)) +
  geom_errorbar(aes(
    x = pretreatment, ymin = Bill - SE,
    ymax = Bill + SE
  ), colour = "black", width = 0.3) +
  geom_point(
    size = 5, pch = 21, colour = "black",
    aes(x = pretreatment, fill = factor(pretreatment))
  ) +
  geom_line(linetype = "dashed", colour = "black") +
```

```
  geom_point(
    size = 2, pch = 21, colour = "black",
    data = subset(slopeData, week == "3") %>%
      mutate("pretreatment" = ifelse(pretreatment == "cold", "A",
        ifelse(pretreatment == "neutral", "B", "C")
      )),
    aes(
      x = pretreatment, y = billLengthMean,
      fill = factor(pretreatment)
    ),
    position = position_jitter(width = 0.3)
  ) +
  scale_fill_manual(values = c("#7BB4E3", "black", "#CD5C5C")) +
  scale_x_discrete(
    breaks = c("A", "B", "C"),
    labels = c(
      "Cold\n(10°C)",
      "Mild\n(20°C)",
      "Warm\n(30°C)"
    )
  ) +
  theme_classic() +
  theme(
    legend.position = "none",
    axis.text = element_text(family = "Noto Sans"),
    axis.title = element_text(family = "Noto Sans")
  ) +
  xlab("Rearing Conditions") +
  ylab("Bill Length (mm)")

p4 <- data.frame(
  "pretreatment" = c("A", "B", "C"),
  "mass" = 0,
  "tarsus" = 0,
  "bill" = 0
) %>%
  mutate(
    "slope" = predict(functionModel3Weeks,
      newdata = ., robust = TRUE,
      resp = "rawSlope", re_form = NA
    )[, "Estimate"],
    "SE" = predict(functionModel3Weeks,
      newdata = ., robust = TRUE,
      resp = "rawSlope", re_form = NA
    )[, "Est.Error"]
  ) %>%
  mutate(slope = slope +
    mean(subset(slopeData, week == "3")$mslope,
      na.rm = T
    )) %>%
  ggplot(aes(x = pretreatment, y = slope)) +
  geom_errorbar(
    aes(
      x = pretreatment,
      ymin = slope - SE, ymax = slope + SE
    ),
    colour = "black", width = 0.3
  ) +
  geom_point(
    size = 5, pch = 21, colour = "black",
    aes(x = pretreatment, fill = factor(pretreatment))
  ) +
  geom_line(linetype = "dashed", colour = "black") +
  geom_point(
    size = 2, pch = 21, colour = "black",
    data = subset(slopeData, week == "3") %>%
      mutate("pretreatment" = ifelse(pretreatment == "cold", "A",
```

```
        ifelse(pretreatment == "neutral", "B", "C")
      )),
    aes(
      x = pretreatment, y = mslope,
      fill = factor(pretreatment)
    ),
    position = position_jitter(width = 0.3)
  ) +
  scale_fill_manual(values = c("#7BB4E3", "black", "#CD5C5C")) +
  scale_x_discrete(
    breaks = c("A", "B", "C"),
    labels = c(
      "Cold\n(10°C)",
      "Mild\n(20°C)",
      "Warm\n(30°C)"
    )
  ) +
  theme_classic() +
  theme(
    legend.position = "none",
    axis.text = element_text(family = "Noto Sans"),
    axis.title = element_text(family = "Noto Sans")
  ) +
  xlab("Rearing Conditions") +
  ylab("Metabolic Slope\n(fold Metabolism at\nThermoneutrality/°C)")

pretreatmentPanel <- ((p1 / p2) | (p3 / p4)) +
  plot_annotation(tag_levels = "A")

showtext_auto()
pretreatmentPanel
```

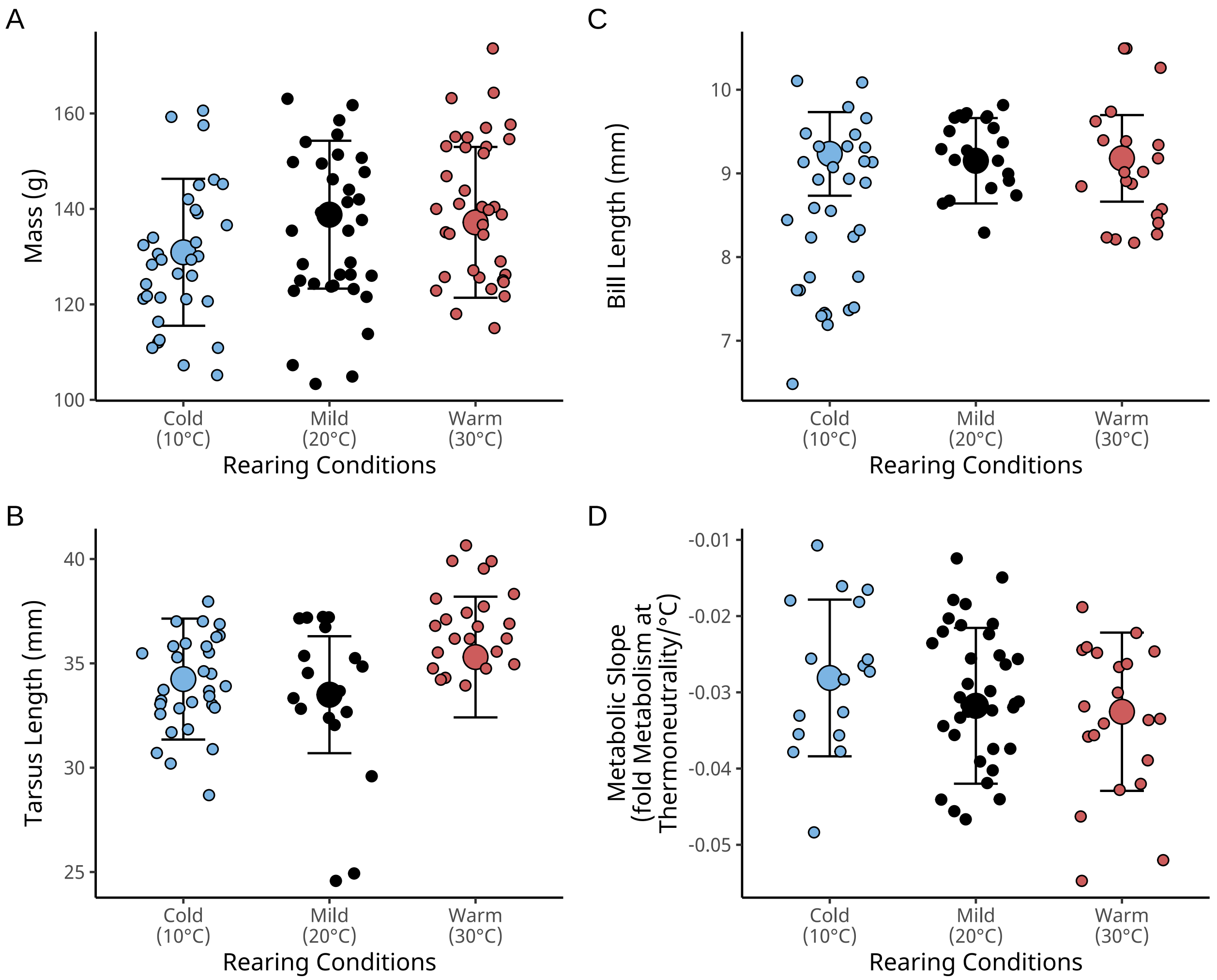

Figure 95: *Effect of post-hatch thermal environment on morphology (body mass, g, tarsus length, mm, bill length, mm) and thermal physiology (metabolic slope, fold RMR at thermoneutrality/°C) in three week old Japanese quail. Metabolic slope is measured at ambient temperatures at and below 30°C (10°C - 30°C).*

```
ggsave("./plots/rearingConditionEffects3Weeks_3fold.pdf",
  pretreatmentPanel,
  dpi = 800, height = 7, width = 9
)
showtext_auto(enable = "FALSE")
```

And predicted effects of morphology on metabolic slopes are also visualised.

```
# And morphology effects on thermal physiology

p1 <- data.frame(
  "pretreatment" = "B",
  "mass" = seq(min(functionModel3Weeks$data$mass, na.rm = T),
    max(functionModel3Weeks$data$mass, na.rm = T),
    by = 0.1
  ),
  "tarsus" = 0,
  "bill" = 0
) %>%
  mutate(
    "slope" = predict(functionModel3Weeks,
```

```
      newdata = ., robust = TRUE,
      resp = "rawSlope", re_form = NA
    )[, "Estimate"],
    "SE" = predict(functionModel3Weeks,
      newdata = ., robust = TRUE,
      resp = "rawSlope", re_form = NA
    )[, "Est.Error"]
  ) %>%
  mutate("Mass" = mass +
    mean(subset(slopeData, week == "3")$mass,
      na.rm = T
    )) %>%
  mutate("slope" = slope +
    mean(subset(slopeData, week == "3")$mslope,
      na.rm = T
    )) %>%
  ggplot(aes(x = Mass, y = slope)) +
  geom_ribbon(
    aes(
      x = Mass, ymin = slope - SE,
      ymax = slope + SE
    ),
    fill = "grey50", alpha = 0.5
  ) +
  geom_smooth(
    method = "lm", linetype = "dashed",
    se = FALSE, colour = "black"
  ) +
  geom_point(
    size = 2.5, pch = 21, colour = "black", alpha = 0.5,
    fill = "grey50",
    data = subset(slopeData, week == "3"),
    aes(x = mass, y = mslope)
  ) +
  theme_classic() +
  theme(
    legend.position = "none",
    axis.text = element_text(family = "Noto Sans"),
    axis.title = element_text(family = "Noto Sans")
  ) +
  xlab("Body Mass (g)") +
  ylab("Metabolic Slope\n(Fold Metabolism\nat Thermoneutrality/°C)")

p2 <- data.frame(
  "pretreatment" = "B",
  "tarsus" = seq(min(functionModel3Weeks$data$tarsus, na.rm = T),
    max(functionModel3Weeks$data$tarsus, na.rm = T),
    by = 0.1
  ),
  "mass" = 0,
  "bill" = 0
) %>%
  mutate(
    "slope" = predict(functionModel3Weeks,
      newdata = ., robust = TRUE,
      resp = "rawSlope", re_form = NA
    )[, "Estimate"],
    "SE" = predict(functionModel3Weeks,
      newdata = ., robust = TRUE,
      resp = "rawSlope", re_form = NA
    )[, "Est.Error"]
  ) %>%
  mutate("Tarsus" = tarsus +
    mean(subset(slopeData, week == "3")$tarsusLengthMean,
      na.rm = T
    )) %>%
  mutate("slope" = slope +
```

```
    mean(subset(slopeData, week == "3")$mslope,
      na.rm = T
    )) %>%
  ggplot(aes(x = Tarsus, y = slope)) +
  geom_ribbon(aes(x = Tarsus, ymin = slope - SE, ymax = slope + SE),
    fill = "grey50", alpha = 0.5
  ) +
  geom_smooth(method = "lm", linetype = "dashed", se = FALSE, colour = "black") +
  geom_point(
    size = 2.5, pch = 21, colour = "black", alpha = 0.5, fill = "grey50",
    data = subset(slopeData, week == "3"),
    aes(x = tarsusLengthMean, y = mslope)
  ) +
  theme_classic() +
  theme(
    legend.position = "none",
    axis.text = element_text(family = "Noto Sans"),
    axis.title = element_text(family = "Noto Sans")
  ) +
  xlab("Tarsus Length (mm)") +
  ylab("Metabolic Slope\n(Fold Metabolism\nat Thermoneutrality/°C)")

p3 <- data.frame(
  "pretreatment" = "B",
  "bill" = seq(min(functionModel3Weeks$data$bill, na.rm = T),
    max(functionModel3Weeks$data$bill, na.rm = T),
    by = 0.1
  ),
  "mass" = 0,
  "tarsus" = 0
) %>%
  mutate(
    "slope" = predict(functionModel3Weeks,
      newdata = ., robust = TRUE,
      resp = "rawSlope", re_form = NA
    )[, "Estimate"],
    "SE" = predict(functionModel3Weeks,
      newdata = ., robust = TRUE,
      resp = "rawSlope", re_form = NA
    )[, "Est.Error"]
  ) %>%
  mutate("Bill" = bill +
    mean(subset(slopeData, week == "3")$billLengthMean,
      na.rm = T
    )) %>%
  mutate("slope" = slope +
    mean(subset(slopeData, week == "3")$mslope,
      na.rm = T
    )) %>%
  ggplot(aes(x = Bill, y = slope)) +
  geom_ribbon(aes(x = Bill, ymin = slope - SE, ymax = slope + SE),
    fill = "grey50", alpha = 0.5
  ) +
  geom_smooth(method = "lm", linetype = "dashed", se = FALSE, colour = "black") +
  geom_point(
    size = 2.5, pch = 21, colour = "black", alpha = 0.5, fill = "grey50",
    data = subset(slopeData, week == "3"),
    aes(x = billLengthMean, y = mslope)
  ) +
  xlim(c(7, 12)) +
  theme_classic() +
  theme(
    legend.position = "none",
    axis.text = element_text(family = "Noto Sans"),
    axis.title = element_text(family = "Noto Sans")
  ) +
  xlab("Bill Length (mm)") +
```

```
  ylab("Metabolic Slope\n(Fold Metabolism\nat Thermoneutrality/°C)")

massPred <- data.frame(
  "pretreatment" = "B",
  "mass" = c(
    mean(functionModel3Weeks$data$mass, na.rm = T) -
      sd(functionModel3Weeks$data$mass, na.rm = T),
    mean(functionModel3Weeks$data$mass, na.rm = T),
    mean(functionModel3Weeks$data$mass, na.rm = T) +
      sd(functionModel3Weeks$data$mass, na.rm = T)
  ),
  "tarsus" = 0,
  "bill" = 0
) %>%
  predict(functionModel3Weeks,
    re_form = NA,
    resp = "rawSlope", newdata = .,
    summary = TRUE, robust = TRUE
  ) %>%
  as.data.frame() %>%
  mutate("slope" = Estimate +
    mean(
      subset(
        slopeData,
        week == 3 & pretreatment == "neutral"
      )$mslope,
      na.rm = TRUE
    )) %>%
  mutate("Size" = c("Small", "Average", "Large")) %>%
  select(Size, slope, "slopeSE" = Est.Error) %>%
  slice(rep(1:n(), each = length(seq(-20, -0.1, by = 0.1)))) %>%
  mutate("Ta" = rep(seq(-20, -0.1, by = 0.1), 3)) %>%
  mutate(
    "VO2" = 1 + Ta * slope,
    "LL" = 1 + Ta * (slope - slopeSE),
    "UL" = 1 + Ta * (slope + slopeSE)
  ) %>%
  mutate(
    "Ta" = Ta + 30,
    Size = factor(Size, levels = c("Small", "Average", "Large"))
  )

massPlot <- massPred %>%
  ggplot(aes(x = Ta, y = VO2)) +
  geom_point(
    data = slopes3Weeks$data %>%
      merge(., subset(all, week == "3") %>%
        select(ring, mass),
      by = "ring", all.x = TRUE
      ) %>%
      mutate(Ta = Ta + 30),
    aes(x = Ta, y = VO2, colour = mass),
    position = position_jitter(width = 1), alpha = 0.5
  ) +
  geom_point(
    data = data.frame("Ta" = 30, "VO2" = 1),
    pch = 21, colour = "black", fill = "grey15",
    size = 3
  ) +
  geom_ribbon(aes(x = Ta, ymin = LL, ymax = UL, fill = Size),
    alpha = 0.4, colour = "black"
  ) +
  geom_line(
    aes(linetype = Size)
  ) +
  theme_classic() +
  xlab("Ambient Temperature (°C)") +
```

```
  ylab("Fold Metabolism at\nThermoneutrality") +
  scale_fill_manual(
    values = c("#DECCC1", "#855E46", "#231709"),
    labels = c(
      paste0(
        "Mean Mass -\n1 SD (",
        round(
          mean(subset(slopeData, week == "3")$mass, na.rm = T) -
            sd(subset(slopeData, week == "3")$mass, na.rm = T),
          digits = 0.1
        ),
        "g)"
      ),
      paste0(
        "Mean Mass\n(",
        round(mean(subset(slopeData, week == "3")$mass, na.rm = T),
          digits = 0.1
        ),
        "g)"
      ),
      paste0(
        "Mean Mass +\n1 SD (",
        round(
          mean(subset(slopeData, week == "3")$mass, na.rm = T) +
            sd(subset(slopeData, week == "3")$mass, na.rm = T),
          digits = 0.1
        ),
        "g)"
      )
    ), name = NULL
  ) +
  scale_linetype_manual(
    values = c("solid", "dashed", "dotted"),
    labels = c(
      paste0(
        "Mean Mass -\n1 SD (",
        round(
          mean(subset(slopeData, week == "3")$mass, na.rm = T) -
            sd(subset(slopeData, week == "3")$mass, na.rm = T),
          digits = 0.1
        ),
        "g)"
      ),
      paste0(
        "Mean Mass\n(",
        round(mean(subset(slopeData, week == "3")$mass, na.rm = T),
          digits = 0.1
        ),
        "g)"
      ),
      paste0(
        "Mean Mass +\n1 SD (",
        round(
          mean(subset(slopeData, week == "3")$mass, na.rm = T) +
            sd(subset(slopeData, week == "3")$mass, na.rm = T),
          digits = 0.1
        ),
        "g)"
      )
    ), name = NULL
  ) +
  scale_colour_gradient(
    low = "#DECCC1", high = "#231709",
    guide = NULL
  ) +
  theme(
    axis.text = element_text(family = "Noto Sans"),
```

```
    axis.title = element_text(family = "Noto Sans"),
    legend.position = "bottom",
    legend.key.size = unit(0.5, 'cm'),
    legend.text = element_text(size = 6)
  )

tarsusPred <- data.frame(
  "pretreatment" = "B",
  "tarsus" = c(
    mean(functionModel3Weeks$data$tarsus, na.rm = T) -
      sd(functionModel3Weeks$data$tarsus, na.rm = T),
    mean(functionModel3Weeks$data$tarsus, na.rm = T),
    mean(functionModel3Weeks$data$tarsus, na.rm = T) +
      sd(functionModel3Weeks$data$tarsus, na.rm = T)
  ),
  "mass" = 0,
  "bill" = 0
) %>%
  predict(functionModel3Weeks,
    re_form = NA,
    resp = "rawSlope", newdata = .,
    summary = TRUE, robust = TRUE
  ) %>%
  as.data.frame() %>%
  mutate("slope" = Estimate +
    mean(
      subset(
        slopeData,
        week == 3 & pretreatment == "neutral"
      )$mslope,
      na.rm = TRUE
    )) %>%
  mutate("Size" = c("Small", "Average", "Large")) %>%
  select(Size, slope, "slopeSE" = Est.Error) %>%
  slice(rep(1:n(), each = length(seq(-20, -0.1, by = 0.1)))) %>%
  mutate("Ta" = rep(seq(-20, -0.1, by = 0.1), 3)) %>%
  mutate(
    "VO2" = 1 + Ta * slope,
    "LL" = 1 + Ta * (slope - slopeSE),
    "UL" = 1 + Ta * (slope + slopeSE)
  ) %>%
  mutate(
    "Ta" = Ta + 30,
    Size = factor(Size, levels = c("Small", "Average", "Large"))
  )

tarsusPlot <- tarsusPred %>%
  ggplot(aes(x = Ta, y = VO2)) +
  geom_point(
    data = slopes3Weeks$data %>%
      merge(., subset(all, week == "3") %>%
        select(ring, "tarsus" = tarsusLengthMean),
      by = "ring", all.x = TRUE
      ) %>%
      mutate(Ta = Ta + 30),
    aes(x = Ta, y = VO2, colour = tarsus),
    position = position_jitter(width = 1), alpha = 0.5
  ) +
  geom_point(
    data = data.frame("Ta" = 30, "VO2" = 1),
    pch = 21, colour = "black", fill = "grey15",
    size = 3
  ) +
  geom_ribbon(aes(x = Ta, ymin = LL, ymax = UL, fill = Size),
    alpha = 0.4, colour = "black"
  ) +
  geom_line(
```

```
    aes(linetype = Size)
  ) +
  theme_classic() +
  xlab("Ambient Temperature (°C)") +
  ylab("Fold Metabolism at\nThermoneutrality") +
  scale_fill_manual(
    values = c("#DECCC1", "#855E46", "#231709"),
    labels = c(
      paste0(
        "Mean Tarsus\nLength -\n1 SD (",
        round(
          mean(subset(slopeData, week == "3")$tarsusLengthMean,
            na.rm = T
          ) -
            sd(subset(slopeData, week == "3")$tarsusLengthMean,
              na.rm = T
            ),
          digits = 0.1
        ),
        "g)"
      ),
      paste0(
        "Mean Tarsus\nLength (",
        round(
          mean(subset(slopeData, week == "3")$tarsusLengthMean,
            na.rm = T
          ),
          digits = 0.1
        ),
        "g)"
      ),
      paste0(
        "Mean Tarsus\nLength +\n1 SD (",
        round(
          mean(subset(slopeData, week == "3")$tarsusLengthMean,
            na.rm = T
          ) +
            sd(subset(slopeData, week == "3")$tarsusLengthMean,
              na.rm = T
            ),
          digits = 0.1
        ),
        "g)"
      )
    ), name = NULL
  ) +
  scale_linetype_manual(
    values = c("solid", "dashed", "dotted"),
    labels = c(
      paste0(
        "Mean Tarsus\nLength -\n1 SD (",
        round(
          mean(subset(slopeData, week == "3")$tarsusLengthMean,
            na.rm = T
          ) -
            sd(subset(slopeData, week == "3")$tarsusLengthMean,
              na.rm = T
            ),
          digits = 0.1
        ),
        "g)"
      ),
      paste0(
        "Mean Tarsus\nLength (",
        round(
          mean(subset(slopeData, week == "3")$tarsusLengthMean,
            na.rm = T
```

```
          ),
          digits = 0.1
        ),
        "g)"
      ),
      paste0(
        "Mean Tarsus\nLength +\n1 SD (",
        round(
          mean(subset(slopeData, week == "3")$tarsusLengthMean,
            na.rm = T
          ) +
            sd(subset(slopeData, week == "3")$tarsusLengthMean,
              na.rm = T
            ),
          digits = 0.1
        ),
        "g)"
      )
    ), name = NULL
  ) +
  scale_colour_gradient(
    low = "#DECCC1", high = "#231709",
    guide = NULL
  ) +
  theme(
    axis.text = element_text(family = "Noto Sans"),
    axis.title = element_text(family = "Noto Sans"),
    legend.position = "bottom",
    legend.key.size = unit(1, 'cm'),
    legend.text = element_text(size = 6)
  )

billPred <- data.frame(
  "pretreatment" = "B",
  "bill" = c(
    mean(functionModel3Weeks$data$bill, na.rm = T) -
      sd(functionModel3Weeks$data$bill, na.rm = T),
    mean(functionModel3Weeks$data$bill, na.rm = T),
    mean(functionModel3Weeks$data$bill, na.rm = T) +
      sd(functionModel3Weeks$data$bill, na.rm = T)
  ),
  "mass" = 0,
  "tarsus" = 0
) %>%
  predict(functionModel3Weeks,
    re_form = NA,
    resp = "rawSlope", newdata = .,
    summary = TRUE, robust = TRUE
  ) %>%
  as.data.frame() %>%
  mutate("slope" = Estimate +
    mean(
      subset(
        slopeData,
        week == 3 & pretreatment == "neutral"
      )$mslope,
      na.rm = TRUE
    )) %>%
  mutate("Size" = c("Small", "Average", "Large")) %>%
  select(Size, slope, "slopeSE" = Est.Error) %>%
  slice(rep(1:n(), each = length(seq(-20, -0.1, by = 0.1)))) %>%
  mutate("Ta" = rep(seq(-20, -0.1, by = 0.1), 3)) %>%
  mutate(
    "VO2" = 1 + Ta * slope,
    "LL" = 1 + Ta * (slope - slopeSE),
    "UL" = 1 + Ta * (slope + slopeSE)
  ) %>%
```

```
  mutate(
    "Ta" = Ta + 30,
    Size = factor(Size, levels = c("Small", "Average", "Large"))
  )

billPlot <- billPred %>%
  ggplot(aes(x = Ta, y = VO2)) +
  geom_point(
    data = slopes3Weeks$data %>%
      merge(., subset(all, week == "3") %>%
        select(ring, "bill" = billLengthMean),
      by = "ring", all.x = TRUE
      ) %>%
      mutate(Ta = Ta + 30),
    aes(x = Ta, y = VO2, colour = bill),
    position = position_jitter(width = 1), alpha = 0.5
  ) +
  geom_point(
    data = data.frame("Ta" = 30, "VO2" = 1),
    pch = 21, colour = "black", fill = "grey15",
    size = 3
  ) +
  geom_ribbon(aes(x = Ta, ymin = LL, ymax = UL, fill = Size),
    alpha = 0.4, colour = "black"
  ) +
  geom_line(
    aes(linetype = Size)
  ) +
  theme_classic() +
  xlab("Ambient Temperature (°C)") +
  ylab("Fold Metabolism at\nThermoneutrality") +
  scale_fill_manual(
    values = c("#DECCC1", "#855E46", "#231709"),
    labels = c(
      paste0(
        "Mean Bill\nLength -\n1 SD (",
        round(
          mean(subset(slopeData, week == "3")$billLengthMean,
            na.rm = T
          ) -
            sd(subset(slopeData, week == "3")$billLengthMean,
              na.rm = T
            ),
          digits = 0.1
        ),
        "g)"
      ),
      paste0(
        "Mean Bill\nLength (",
        round(
          mean(subset(slopeData, week == "3")$billLengthMean,
            na.rm = T
          ),
          digits = 0.1
        ),
        "g)"
      ),
      paste0(
        "Mean Bill\nLength +\n1 SD (",
        round(
          mean(subset(slopeData, week == "3")$billLengthMean,
            na.rm = T
          ) +
            sd(subset(slopeData, week == "3")$billLengthMean,
              na.rm = T
            ),
          digits = 0.1
```

```
        ),
        "g)"
      )
    ), name = NULL
  ) +
  scale_linetype_manual(
    values = c("solid", "dashed", "dotted"),
    labels = c(
      paste0(
        "Mean Bill\nLength -\n1 SD (",
        round(
          mean(subset(slopeData, week == "3")$billLengthMean,
            na.rm = T
          ) -
            sd(subset(slopeData, week == "3")$billLengthMean,
              na.rm = T
            ),
          digits = 0.1
        ),
        "g)"
      ),
      paste0(
        "Mean Bill\nLength (",
        round(
          mean(subset(slopeData, week == "3")$billLengthMean,
            na.rm = T
          ),
          digits = 0.1
        ),
        "g)"
      ),
      paste0(
        "Mean Bill\nLength +\n1 SD (",
        round(
          mean(subset(slopeData, week == "3")$billLengthMean,
            na.rm = T
          ) +
            sd(subset(slopeData, week == "3")$billLengthMean,
              na.rm = T
            ),
          digits = 0.1
        ),
        "g)"
      )
    ), name = NULL
  ) +
  scale_colour_gradient(
    low = "#DECCC1", high = "#231709",
    guide = NULL
  ) +
  theme(
    axis.text = element_text(family = "Noto Sans"),
    axis.title = element_text(family = "Noto Sans"),
    legend.position = "bottom",
    legend.key.size = unit(1, 'cm'),
    legend.text = element_text(size = 6)
  )

showtext.auto()

allMorphology <- (p1 + massPlot) / (p2 + tarsusPlot) / (p3 + billPlot) +
  plot_annotation(tag_levels = "A")

allMorphology
```

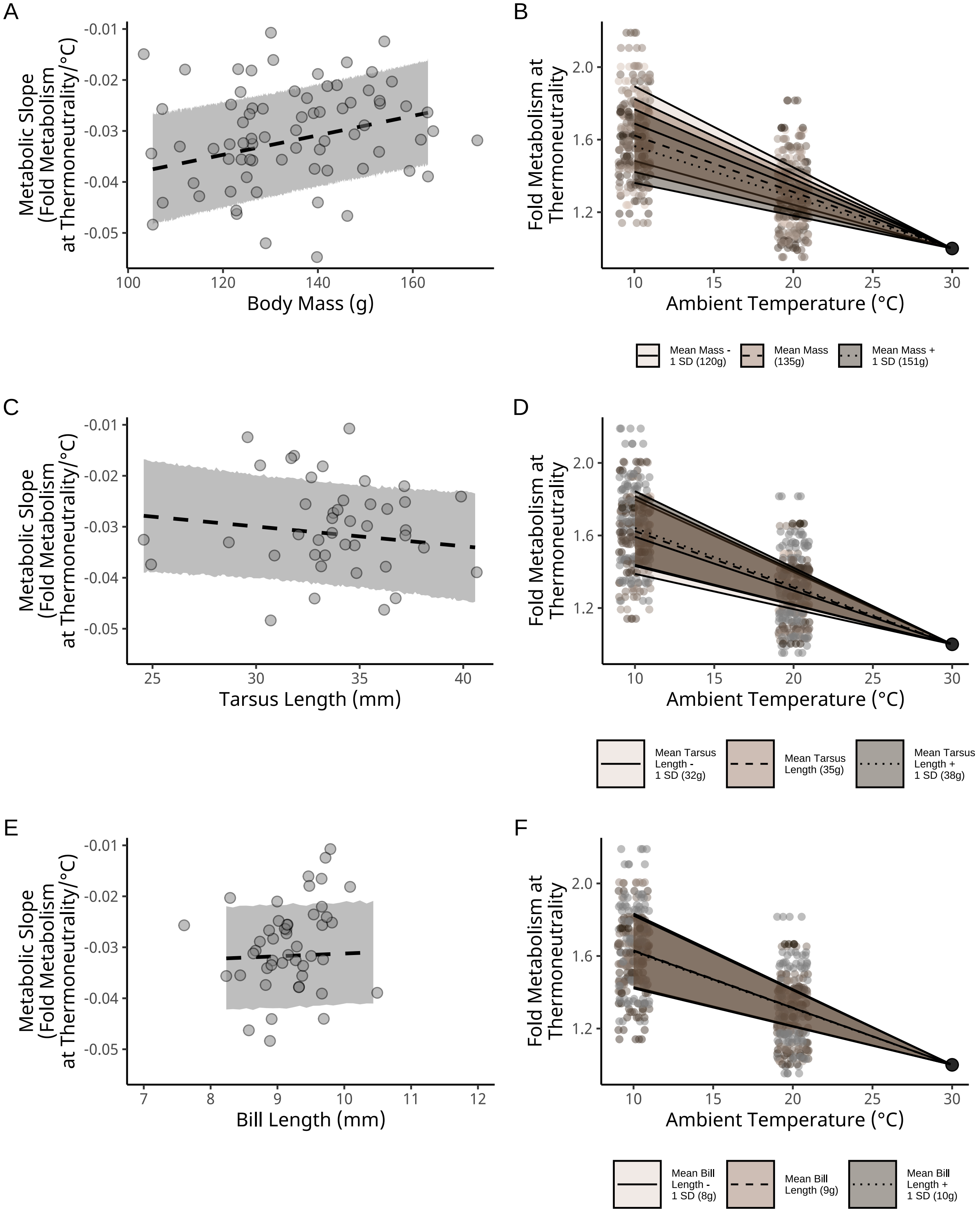

Figure 96: *Effect of morphology (body mass, g, tarsus length, mm, bill length, mm) on metabolic slopes (fold RMR at thermoneutrality/°C) at ambient temperatures below thermoneutrality (<30°C) in three week old Japanese quail. Dots represent raw data points and lines and ribbons represent predicted relationships +/- 1 standard error from a Bayesian path analysis.*

```
ggsave("./plots/morphologyEffectsResistance3Weeks_3Fold.pdf",
  allMorphology,
  dpi = 800, height = 8, width = 8
)

showtext.auto(enable = "FALSE")
```

```
# As fold increase at 10°C

massPlot10 <- massPred %>%
  filter(Ta == 10) %>%
  ggplot(aes(x = Size, y = VO2)) +
  geom_point(
    data = slopes3Weeks$data %>%
      filter(Ta == -20) %>%
      merge(., subset(all, week == "3") %>%
        select(ring, mass) %>%
        distinct() %>%
        mutate(Size = ifelse(mass < (mean(mass, na.rm = T) -
          sd(mass, na.rm = T)),
        "Small",
        ifelse((mass > (mean(mass, na.rm = T) -
          sd(mass, na.rm = T))) &
          (mass < (mean(mass, na.rm = T) +
            sd(mass, na.rm = T))),
        "Average", "Large"
        )
        )),
      by = "ring", all.x = TRUE
      ) %>%
      mutate(Size = factor(Size, levels = c("Small", "Average", "Large"))),
    aes(x = Size, y = VO2, colour = Size),
    position = position_jitter(width = 0.25), alpha = 0.5
  ) +
  geom_errorbar(aes(ymin = LL, ymax = UL),
    colour = "black", width = 0.25
  ) +
  geom_point(
    size = 3, pch = 21, colour = "black",
    aes(fill = Size)
  ) +
  xlab("Body Mass") +
  ylab("Fold Increase in Metabolism at 10°C\n(From Thermoneutrality)") +
  scale_x_discrete(labels = c("Mean - 1 SD", "Mean", "Mean + 1 SD")) +
  scale_fill_manual(values = c("#DECCC1", "#855E46", "#231709")) +
  scale_colour_manual(values = c("#DECCC1", "#855E46", "#231709")) +
  theme_classic() +
  theme(
    legend.position = "none",
    axis.text = element_text(family = "Noto Sans"),
    axis.title = element_text(family = "Noto Sans")
  )

tarsusPlot10 <- tarsusPred %>%
  filter(Ta == 10) %>%
  ggplot(aes(x = Size, y = VO2)) +
  geom_point(
    data = slopes3Weeks$data %>%
      filter(Ta == -20) %>%
      merge(., subset(all, week == "3") %>%
        select(ring, "tarsus" = tarsusLengthMean) %>%
        distinct() %>%
        mutate(Size = ifelse(tarsus < (mean(tarsus, na.rm = T) -
          sd(tarsus, na.rm = T)),
        "Small",
        ifelse((tarsus > (mean(tarsus, na.rm = T) -
          sd(tarsus, na.rm = T))) &
          (tarsus < (mean(tarsus, na.rm = T) +
```

```
          sd(tarsus, na.rm = T))),
        "Average", "Large"
        )
        )),
      by = "ring", all.x = TRUE
      ) %>%
      drop_na(tarsus) %>%
      mutate(Size = factor(Size, levels = c("Small", "Average", "Large"))),
    aes(x = Size, y = VO2, colour = Size),
    position = position_jitter(width = 0.25), alpha = 0.5
  ) +
  geom_errorbar(aes(ymin = LL, ymax = UL),
    colour = "black", width = 0.25
  ) +
  geom_point(
    size = 3, pch = 21, colour = "black",
    aes(fill = Size)
  ) +
  xlab("Tarsus Length") +
  ylab("Fold Increase in Metabolism at 10°C\n(From Thermoneutrality)") +
  scale_x_discrete(labels = c(
    "Mean - 1 SD",
    "Mean",
    "Mean + 1 SD"
  )) +
  scale_fill_manual(values = c("#DECCC1", "#855E46", "#231709")) +
  scale_colour_manual(values = c("#DECCC1", "#855E46", "#231709")) +
  theme_classic() +
  theme(
    legend.position = "none",
    axis.text = element_text(family = "Noto Sans"),
    axis.title = element_text(family = "Noto Sans")
  )

billPlot10 <- billPred %>%
  filter(Ta == 10) %>%
  ggplot(aes(x = Size, y = VO2)) +
  geom_point(
    data = slopes3Weeks$data %>%
      filter(Ta == -20) %>%
      merge(., subset(all, week == "3") %>%
        select(ring, "bill" = billLengthMean) %>%
        distinct() %>%
        mutate(Size = ifelse(bill < (mean(bill, na.rm = T) -
          sd(bill, na.rm = T)),
        "Small",
        ifelse((bill > (mean(bill, na.rm = T) -
          sd(bill, na.rm = T))) &
          (bill < (mean(bill, na.rm = T) +
            sd(bill, na.rm = T))),
        "Average", "Large"
        )
        )),
      by = "ring", all.x = TRUE
      ) %>%
      drop_na(bill) %>%
      mutate(Size = factor(Size, levels = c("Small", "Average", "Large"))),
    aes(x = Size, y = VO2, colour = Size),
    position = position_jitter(width = 0.25), alpha = 0.5
  ) +
  geom_errorbar(aes(ymin = LL, ymax = UL),
    colour = "black", width = 0.25
  ) +
  geom_point(
    size = 3, pch = 21, colour = "black",
    aes(fill = Size)
  ) +
```

```
  xlab("Bill Length") +
  ylab("Fold Increase in Metabolism at 10°C\n(From Thermoneutrality)") +
  scale_x_discrete(labels = c(
    "Mean - 1 SD",
    "Mean",
    "Mean + 1 SD"
  )) +
  scale_fill_manual(values = c("#DECCC1", "#855E46", "#231709")) +
  scale_colour_manual(values = c("#DECCC1", "#855E46", "#231709")) +
  theme_classic() +
  theme(
    legend.position = "none",
    axis.text = element_text(family = "Noto Sans"),
    axis.title = element_text(family = "Noto Sans")
  )

allMorphology10 <- (p1 + massPlot10) / (p2 + tarsusPlot10) / (p3 + billPlot10) +
  plot_annotation(tag_levels = "A")

ggsave("./plots/morphologyEffectsResistance3Weeks_3Fold10.pdf",
  allMorphology10,
  dpi = 800, height = 8, width = 8
)
```

Finally, we summarise model outcomes and estimate partial R^2 values for population-level model predictors.

```
caption <- paste0(
  "Results from a Bayesian path analysis ",
  "predicting metabolic slopes (fold resting ",
  "metabolism at thermoneutrality/°C) ",
  "as a function of morphometry in three ",
  "week old Japanese quail. ",
  "Cold rearing indicates post-hatch rearing at ",
  "10°C, relative to ",
  "20°C (intercept), or ",
  "30°C ('warm rearing'). ",
  "Coefficients represent medians and credible intervals ",
  "(CIs) represent quantile intervals. ",
  "BF indicates Bayes Factors."
)

week3Results <-
  as.data.frame(functionModel3Weeks) %>%
  summarise_all(., .funs = median) %>%
  pivot_longer(everything(),
    names_to = "Parameter",
    values_to = "Estimate"
  ) %>%
  merge(., quantileCIs(functionModel3Weeks, cis = c(50, 95)),
    by = "Parameter", all.x = TRUE
  ) %>%
  filter(grepl("b_|sd_", Parameter)) %>%
  rowwise() %>%
  mutate("BF" = ifelse(Estimate < 0,
    (2 * mean(as.data.frame(
      functionModel3Weeks
    )[, Parameter] <= 0)) /
      (2 * mean(as.data.frame(
        functionModel3Weeks
      )[, Parameter] >= 0)),
    (2 * mean(as.data.frame(
      functionModel3Weeks
    )[, Parameter] >= 0)) /
      (2 * mean(as.data.frame(
        functionModel3Weeks
      )[, Parameter] <= 0))
  )) %>%
```

```
ungroup() %>%
mutate(
  "Estimate" = round(Estimate, digits = 4),
  "BF" = round(BF, digits = 4),
  "N" = nrow(functionModel3Weeks$data)
) %>%
mutate("Parameter" = ifelse(grepl("b_", Parameter),
  gsub(
    "b_", "",
    Parameter
  ),
  gsub(
    "Intercept", "batch",
    gsub("sd_batch__", "", Parameter)
  )
)) %>%
mutate(
  "Response" = gsub("_.*", "", Parameter),
  "Parameter" = gsub(".*_", "", Parameter)
) %>%
merge(., tribble(
  ~Response, ~response, ~level,
  "mass", "Body Mass (g)", "A",
  "rawSlope",
  "Metabolic Slope", "D",
  "tarsus", "Tarsus Length (mm)", "B",
  "bill", "Bill Length (mm)", "C"
),
by = "Response"
) %>%
merge(., tribble(
  ~Parameter, ~parameter, ~number,
  "Intercept", "Intercept", "1",
  "mass", "Body Mass (g)", "4",
  "tarsus", "Tarsus Length (mm)", "5",
  "bill", "Bill Length (mm)", "6",
  "pretreatmentA", "Cold Rearing", "2",
  "pretreatmentC", "Warm Rearing", "3",
  "batch", "Egg Batch [mu]", "7"
),
by = "Parameter"
) %>%
mutate(
  `50\\% HDI` = paste0("(", paste(
    round(Low_CI_50, digits = 4),
    round(High_CI_50, digits = 4),
    sep = ", "
  ), ")"),
  `95\\% HDI` = paste0("(", paste(
    round(Low_CI_95, digits = 4),
    round(High_CI_95, digits = 4),
    sep = ", "
  ), ")")
) %>%
dplyr::select(-c(Low_CI_50, High_CI_50, Low_CI_95, High_CI_95)) %>%
dplyr::select(
  "Response" = "response", "Parameter" = "parameter", N,
  Estimate, `50\\% HDI`, `95\\% HDI`, BF, level, number
) %>%
arrange(level, number) %>%
dplyr::select(-c(level, number)) %>%
kbl(.,
  longtable = T, booktabs = T, format = "latex", escape = FALSE,
  caption = caption
) %>%
column_spec(column = c(1:2), width = "2.7cm") %>%
column_spec(column = 3, width = "1cm") %>%
```

```
  column_spec(column = c(4:10), width = "1.9cm") %>%
  kable_styling(latex_options = "striped")

week3Results
```

Table 41: *Results from a Bayesian path analysis predicting metabolic slopes (fold resting metabolism at thermoneutrality/°C) as a function of morphometry in three week old Japanese quail. Cold rearing indicates post-hatch rearing at 10°C, relative to 20°C (intercept), or 30°C ('warm rearing'). Coefficients represent medians and credible intervals (CIs) represent quantile intervals. BF indicates Bayes Factors.*

Response	Parameter	N	Estimate	50% HDI	95% HDI	BF
Body Mass (g)	Intercept	42	3.7142	(1.4661, 5.9338)	(-2.8758, 10.161)	6.4419
Body Mass (g)	Cold Rearing	42	-8.3080	(-11.5071, -5.0491)	(-17.6774, 1.4015)	20.2202
Body Mass (g)	Warm Rearing	42	-1.5588	(-5.5567, 2.2592)	(-13.2447, 9.9407)	1.5486
Body Mass (g)	Egg Batch [mu]	42	0.2754	(0.1146, 0.5486)	(0.0101, 1.4357)	Inf
Tarsus Length (mm)	Intercept	42	-1.2771	(-1.8303, -0.6674)	(-2.9882, 0.8348)	9.7383
Tarsus Length (mm)	Cold Rearing	42	0.6847	(0.0378, 1.3098)	(-1.238, 2.5282)	3.2261
Tarsus Length (mm)	Warm Rearing	42	1.8362	(0.9778, 2.6717)	(-0.6475, 4.1378)	12.7457
Tarsus Length (mm)	Body Mass (g)	42	0.0911	(0.0738, 0.1094)	(0.0389, 0.1434)	2665.6667
Tarsus Length (mm)	Egg Batch [mu]	42	0.8549	(0.4452, 1.3209)	(0.0419, 2.4552)	Inf
Bill Length (mm)	Intercept	42	0.2591	(0.167, 0.354)	(-0.0872, 0.5897)	19.2020
Bill Length (mm)	Cold Rearing	42	0.0836	(-0.0249, 0.1963)	(-0.2427, 0.4182)	2.3099
Bill Length (mm)	Warm Rearing	42	0.0438	(-0.0905, 0.1782)	(-0.3597, 0.439)	1.4309
Bill Length (mm)	Body Mass (g)	42	0.0097	(0.0064, 0.0132)	(-1e-04, 0.0196)	37.8350
Bill Length (mm)	Egg Batch [mu]	42	0.0920	(0.0388, 0.1776)	(0.0032, 0.5041)	Inf
Metabolic Slope	Intercept	42	-0.0012	(-0.0037, 0.0013)	(-0.0111, 0.0088)	1.6864
Metabolic Slope	Cold Rearing	42	0.0036	(0.0014, 0.0059)	(-0.003, 0.0105)	6.1174
Metabolic Slope	Warm Rearing	42	-0.0009	(-0.004, 0.0021)	(-0.0097, 0.0078)	1.3419
Metabolic Slope	Body Mass (g)	42	0.0002	(1e-04, 3e-04)	(0, 4e-04)	27.5714
Metabolic Slope	Tarsus Length (mm)	42	-0.0004	(-8e-04, 0)	(-0.0015, 7e-04)	3.0754
Metabolic Slope	Bill Length (mm)	42	0.0004	(-2e-04, 0.0011)	(-0.0014, 0.0024)	2.0852
Metabolic Slope	Egg Batch [mu]	42	0.0038	(0.0017, 0.0081)	(1e-04, 0.027)	Inf

```
#save_kable(week3Results, "../tables/threeWeekResults_3.html")

# And checking partial R2 values of morphometry on metabolic slopes

functionModel3R2Mass <-
  brm(
    data = slopeData %>%
      filter(week == "3") %>%
      mutate(
```

```
        mass = mass - mean(mass, na.rm = T),
        tarsus = tarsusLengthMean - mean(tarsusLengthMean, na.rm = T),
        bill = billLengthMean - mean(billLengthMean, na.rm = T),
        pretreatment = ifelse(pretreatment == "cold", "A",
          ifelse(pretreatment == "neutral", "B", "C")
        )
      ) %>%
      mutate(pretreatment = factor(pretreatment, levels = c("B", "A", "C"))) %>%
      drop_na() %>%
      merge(., data %>%
        dplyr::select(ring, "batch" = exp) %>%
        distinct(),
      by = "ring", all.x = TRUE
      ),
    family = "gaussian",
    bf(mass ~ pretreatment + (1 | batch)) +
      bf(tarsus ~ mass + pretreatment + (1 | batch)) +
      bf(bill ~ mass + pretreatment + (1 | batch)) +
      bf(rawSlope ~ tarsus + bill + pretreatment + (1 | batch)) +
      set_rescor(FALSE),
    prior = c(
      set_prior("normal(0, 5)",
        class = "Intercept",
        resp = "mass"
      ),
      set_prior("normal(0, 15)",
        class = "b",
        coef = "pretreatmentA",
        resp = "mass"
      ),
      set_prior("normal(0, 15)",
        class = "b",
        coef = "pretreatmentC",
        resp = "mass"
      ),
      set_prior("exponential(2.5)",
        class = "sd",
        group = "batch",
        resp = "mass"
      ),
      set_prior("exponential(0.15)",
        class = "sigma",
        resp = "mass"
      ),
      set_prior("normal(0, 2.5)",
        class = "Intercept",
        resp = "tarsus"
      ),
      set_prior("normal(0, 2.5)",
        class = "b",
        coef = "pretreatmentA",
        resp = "tarsus"
      ),
      set_prior("normal(0, 2.5)",
        class = "b",
        coef = "pretreatmentC",
        resp = "tarsus"
      ),
      set_prior("skew_normal(0, 0.25, 5)",
        class = "b",
        coef = "mass",
        resp = "tarsus"
      ),
      set_prior("exponential(2)",
        class = "sd",
        group = "batch",
        resp = "tarsus"
```

```
    ),
    set_prior("exponential(1)",
      class = "sigma",
      resp = "tarsus"
    ),
    set_prior("normal(0, 1)",
      class = "Intercept",
      resp = "bill"
    ),
    set_prior("normal(0, 0.5)",
      class = "b",
      coef = "pretreatmentA",
      resp = "bill"
    ),
    set_prior("normal(0, 0.5)",
      class = "b",
      coef = "pretreatmentC",
      resp = "bill"
    ),
    set_prior("skew_normal(0, 0.25, 5)",
      class = "b",
      coef = "mass",
      resp = "bill"
    ),
    set_prior("exponential(5)",
      class = "sd",
      group = "batch",
      resp = "bill"
    ),
    set_prior("exponential(2.5)",
      class = "sigma",
      resp = "bill"
    ),
    set_prior("normal(0, 0.01)",
      class = "Intercept",
      resp = "rawSlope"
    ),
    set_prior("normal(0, 0.025)",
      class = "b",
      coef = "pretreatmentA",
      resp = "rawSlope"
    ),
    set_prior("normal(0, 0.025)",
      class = "b",
      coef = "pretreatmentC",
      resp = "rawSlope"
    ),
    set_prior("normal(0, 0.0025)",
      class = "b",
      coef = "tarsus",
      resp = "rawSlope"
    ),
    set_prior("normal(0, 0.001)",
      class = "b",
      coef = "bill",
      resp = "rawSlope"
    ),
    set_prior("exponential(50)",
      class = "sd",
      group = "batch",
      resp = "rawSlope"
    ),
    set_prior("exponential(10)",
      class = "sigma",
      resp = "rawSlope"
    )
  ),
```

```
    iter = 50000, warmup = 10000, cores = 4, chains = 4, thin = 20,
    control = list(adapt_delta = .97, max_treedepth = 14),
    silent = TRUE, refresh = 0,
    file = "./models/_threeWeekFunctionModelMassR2.Rds"
  )

functionModel3WeeksR2Tarsus <-
  brm(
    data = slopeData %>%
      filter(week == "3") %>%
      mutate(
        mass = mass - mean(mass, na.rm = T),
        tarsus = tarsusLengthMean - mean(tarsusLengthMean, na.rm = T),
        bill = billLengthMean - mean(billLengthMean, na.rm = T),
        pretreatment = ifelse(pretreatment == "cold", "A",
          ifelse(pretreatment == "neutral", "B", "C")
        )
      ) %>%
      mutate(pretreatment = factor(pretreatment, levels = c("B", "A", "C"))) %>%
      drop_na() %>%
      merge(., data %>%
        select(ring, "batch" = exp) %>%
        distinct(),
      by = "ring", all.x = TRUE
      ),
    family = "gaussian",
    bf(mass ~ pretreatment + (1 | batch)) +
      bf(tarsus ~ mass + pretreatment + (1 | batch)) +
      bf(bill ~ mass + pretreatment + (1 | batch)) +
      bf(rawSlope ~ mass + bill + pretreatment + (1 | batch)) +
      set_rescor(FALSE),
   prior = c(
      set_prior("normal(0, 5)",
        class = "Intercept",
        resp = "mass"
      ),
      set_prior("normal(0, 15)",
        class = "b",
        coef = "pretreatmentA",
        resp = "mass"
      ),
      set_prior("normal(0, 15)",
        class = "b",
        coef = "pretreatmentC",
        resp = "mass"
      ),
      set_prior("exponential(2.5)",
        class = "sd",
        group = "batch",
        resp = "mass"
      ),
      set_prior("exponential(0.15)",
        class = "sigma",
        resp = "mass"
      ),
      set_prior("normal(0, 2.5)",
        class = "Intercept",
        resp = "tarsus"
      ),
      set_prior("normal(0, 2.5)",
        class = "b",
        coef = "pretreatmentA",
        resp = "tarsus"
      ),
      set_prior("normal(0, 2.5)",
        class = "b",
        coef = "pretreatmentC",
```

```
      resp = "tarsus"
    ),
    set_prior("skew_normal(0, 0.25, 5)",
      class = "b",
      coef = "mass",
      resp = "tarsus"
    ),
    set_prior("exponential(2)",
      class = "sd",
      group = "batch",
      resp = "tarsus"
    ),
    set_prior("exponential(1)",
      class = "sigma",
      resp = "tarsus"
    ),
    set_prior("normal(0, 1)",
      class = "Intercept",
      resp = "bill"
    ),
    set_prior("normal(0, 0.5)",
      class = "b",
      coef = "pretreatmentA",
      resp = "bill"
    ),
    set_prior("normal(0, 0.5)",
      class = "b",
      coef = "pretreatmentC",
      resp = "bill"
    ),
    set_prior("skew_normal(0, 0.25, 5)",
      class = "b",
      coef = "mass",
      resp = "bill"
    ),
    set_prior("exponential(5)",
      class = "sd",
      group = "batch",
      resp = "bill"
    ),
    set_prior("exponential(2.5)",
      class = "sigma",
      resp = "bill"
    ),
    set_prior("normal(0, 0.01)",
      class = "Intercept",
      resp = "rawSlope"
    ),
    set_prior("normal(0, 0.025)",
      class = "b",
      coef = "pretreatmentA",
      resp = "rawSlope"
    ),
    set_prior("normal(0, 0.025)",
      class = "b",
      coef = "pretreatmentC",
      resp = "rawSlope"
    ),
    set_prior("normal(0, 0.001)",
      class = "b",
      coef = "mass",
      resp = "rawSlope"
    ),
    set_prior("normal(0, 0.001)",
      class = "b",
      coef = "bill",
      resp = "rawSlope"
```

```
      ),
      set_prior("exponential(50)",
        class = "sd",
        group = "batch",
        resp = "rawSlope"
      ),
      set_prior("exponential(10)",
        class = "sigma",
        resp = "rawSlope"
      )
    ),
    iter = 50000, warmup = 10000, cores = 4, chains = 4, thin = 20,
    control = list(adapt_delta = .97, max_treedepth = 14),
    silent = TRUE, refresh = 0,
    file = "./models/_threeWeekFunctionModelTarsusR2.Rds"
  )

functionModel3WeeksR2Bill <-
  brm(
    data = slopeData %>%
      filter(week == "3") %>%
      mutate(
        mass = mass - mean(mass, na.rm = T),
        tarsus = tarsusLengthMean - mean(tarsusLengthMean, na.rm = T),
        bill = billLengthMean - mean(billLengthMean, na.rm = T),
        pretreatment = ifelse(pretreatment == "cold", "A",
          ifelse(pretreatment == "neutral", "B", "C")
        )
      ) %>%
      mutate(pretreatment = factor(pretreatment, levels = c("B", "A", "C"))) %>%
      drop_na() %>%
      merge(., data %>%
        select(ring, "batch" = exp) %>%
        distinct(),
      by = "ring", all.x = TRUE
      ),
    family = "gaussian",
    bf(mass ~ pretreatment + (1 | batch)) +
      bf(tarsus ~ mass + pretreatment + (1 | batch)) +
      bf(bill ~ mass + pretreatment + (1 | batch)) +
      bf(rawSlope ~ mass + tarsus + pretreatment + (1 | batch)) +
      set_rescor(FALSE),
   prior = c(
      set_prior("normal(0, 5)",
        class = "Intercept",
        resp = "mass"
      ),
      set_prior("normal(0, 15)",
        class = "b",
        coef = "pretreatmentA",
        resp = "mass"
      ),
      set_prior("normal(0, 15)",
        class = "b",
        coef = "pretreatmentC",
        resp = "mass"
      ),
      set_prior("exponential(2.5)",
        class = "sd",
        group = "batch",
        resp = "mass"
      ),
      set_prior("exponential(0.15)",
        class = "sigma",
        resp = "mass"
      ),
      set_prior("normal(0, 2.5)",
```

```
    class = "Intercept",
    resp = "tarsus"
  ),
  set_prior("normal(0, 2.5)",
    class = "b",
    coef = "pretreatmentA",
    resp = "tarsus"
  ),
  set_prior("normal(0, 2.5)",
    class = "b",
    coef = "pretreatmentC",
    resp = "tarsus"
  ),
  set_prior("skew_normal(0, 0.25, 5)",
    class = "b",
    coef = "mass",
    resp = "tarsus"
  ),
  set_prior("exponential(2)",
    class = "sd",
    group = "batch",
    resp = "tarsus"
  ),
  set_prior("exponential(1)",
    class = "sigma",
    resp = "tarsus"
  ),
  set_prior("normal(0, 1)",
    class = "Intercept",
    resp = "bill"
  ),
  set_prior("normal(0, 0.5)",
    class = "b",
    coef = "pretreatmentA",
    resp = "bill"
  ),
  set_prior("normal(0, 0.5)",
    class = "b",
    coef = "pretreatmentC",
    resp = "bill"
  ),
  set_prior("skew_normal(0, 0.25, 5)",
    class = "b",
    coef = "mass",
    resp = "bill"
  ),
  set_prior("exponential(5)",
    class = "sd",
    group = "batch",
    resp = "bill"
  ),
  set_prior("exponential(2.5)",
    class = "sigma",
    resp = "bill"
  ),
  set_prior("normal(0, 0.01)",
    class = "Intercept",
    resp = "rawSlope"
  ),
  set_prior("normal(0, 0.025)",
    class = "b",
    coef = "pretreatmentA",
    resp = "rawSlope"
  ),
  set_prior("normal(0, 0.025)",
    class = "b",
    coef = "pretreatmentC",
```

```
        resp = "rawSlope"
      ),
      set_prior("normal(0, 0.001)",
        class = "b",
        coef = "mass",
        resp = "rawSlope"
      ),
      set_prior("normal(0, 0.0025)",
        class = "b",
        coef = "tarsus",
        resp = "rawSlope"
      ),
      set_prior("exponential(50)",
        class = "sd",
        group = "batch",
        resp = "rawSlope"
      ),
      set_prior("exponential(10)",
        class = "sigma",
        resp = "rawSlope"
      )
    ),
    iter = 50000, warmup = 10000, cores = 4, chains = 4, thin = 20,
    control = list(adapt_delta = .97, max_treedepth = 14),
    silent = TRUE, refresh = 0,
    file = "./models/_threeWeekFunctionModelBillR2.Rds"
  )

# And combined appendage lengths

functionModel3WeeksR2Appendage <-
  brm(
    data = slopeData %>%
      filter(week == "3") %>%
      mutate(
        mass = mass - mean(mass, na.rm = T),
        tarsus = tarsusLengthMean - mean(tarsusLengthMean, na.rm = T),
        bill = billLengthMean - mean(billLengthMean, na.rm = T),
        pretreatment = ifelse(pretreatment == "cold", "A",
          ifelse(pretreatment == "neutral", "B", "C")
        )
      ) %>%
      mutate(pretreatment = factor(pretreatment, levels = c("B", "A", "C"))) %>%
      drop_na() %>%
      merge(., data %>%
        select(ring, "batch" = exp) %>%
        distinct(),
      by = "ring", all.x = TRUE
      ),
    family = "gaussian",
    bf(mass ~ pretreatment + (1 | batch)) +
      bf(tarsus ~ mass + pretreatment + (1 | batch)) +
      bf(bill ~ mass + pretreatment + (1 | batch)) +
      bf(rawSlope ~ mass + pretreatment + (1 | batch)) +
      set_rescor(FALSE),
   prior = c(
      set_prior("normal(0, 5)",
        class = "Intercept",
        resp = "mass"
      ),
      set_prior("normal(0, 15)",
        class = "b",
        coef = "pretreatmentA",
        resp = "mass"
      ),
      set_prior("normal(0, 15)",
        class = "b",
```

```
    coef = "pretreatmentC",
    resp = "mass"
  ),
  set_prior("exponential(2.5)",
    class = "sd",
    group = "batch",
    resp = "mass"
  ),
  set_prior("exponential(0.15)",
    class = "sigma",
    resp = "mass"
  ),
  set_prior("normal(0, 2.5)",
    class = "Intercept",
    resp = "tarsus"
  ),
  set_prior("normal(0, 2.5)",
    class = "b",
    coef = "pretreatmentA",
    resp = "tarsus"
  ),
  set_prior("normal(0, 2.5)",
    class = "b",
    coef = "pretreatmentC",
    resp = "tarsus"
  ),
  set_prior("skew_normal(0, 0.25, 5)",
    class = "b",
    coef = "mass",
    resp = "tarsus"
  ),
  set_prior("exponential(2)",
    class = "sd",
    group = "batch",
    resp = "tarsus"
  ),
  set_prior("exponential(1)",
    class = "sigma",
    resp = "tarsus"
  ),
  set_prior("normal(0, 1)",
    class = "Intercept",
    resp = "bill"
  ),
  set_prior("normal(0, 0.5)",
    class = "b",
    coef = "pretreatmentA",
    resp = "bill"
  ),
  set_prior("normal(0, 0.5)",
    class = "b",
    coef = "pretreatmentC",
    resp = "bill"
  ),
  set_prior("skew_normal(0, 0.25, 5)",
    class = "b",
    coef = "mass",
    resp = "bill"
  ),
  set_prior("exponential(5)",
    class = "sd",
    group = "batch",
    resp = "bill"
  ),
  set_prior("exponential(2.5)",
    class = "sigma",
    resp = "bill"
```

```
      ),
      set_prior("normal(0, 0.01)",
        class = "Intercept",
        resp = "rawSlope"
      ),
      set_prior("normal(0, 0.025)",
        class = "b",
        coef = "pretreatmentA",
        resp = "rawSlope"
      ),
      set_prior("normal(0, 0.025)",
        class = "b",
        coef = "pretreatmentC",
        resp = "rawSlope"
      ),
      set_prior("normal(0, 0.001)",
        class = "b",
        coef = "mass",
        resp = "rawSlope"
      ),
      set_prior("exponential(50)",
        class = "sd",
        group = "batch",
        resp = "rawSlope"
      ),
      set_prior("exponential(10)",
        class = "sigma",
        resp = "rawSlope"
      )
    ),
    iter = 50000, warmup = 10000, cores = 4, chains = 4, thin = 20,
    control = list(adapt_delta = .97, max_treedepth = 14),
    silent = TRUE, refresh = 0,
    file = "./models/_threeWeekFunctionModelAppendageR2.Rds"
  )

pR2Pull <- function(x) {
  baseR2 <- as.data.frame(
    brms::bayes_R2(functionModel3Weeks,
      ndraws = 1000,
      resp = "rawSlope", summary = FALSE,
      robust = TRUE
    )
  )$R2rawSlope
  redR2 <- as.data.frame(
    brms::bayes_R2(x,
      ndraws = 1000,
      resp = "rawSlope", summary = FALSE,
      robust = TRUE
    )
  )$R2rawSlope
  pR2 <- round(baseR2 - redR2, digits = 3)

  ciFrame <- t(
    quantile(pR2, probs = c(0.025, 0.975), type = 8)
  ) %>% as.data.frame()

  ciFrame <- ciFrame %>%
    mutate(`2.5%` = ifelse(`2.5%` < 0, 0, `2.5%`)) %>%
    mutate("95\\% CI" = paste0(
      "[",
      round(`2.5%`, digits = 3),
      ",",
      round(`97.5%`, digits = 3),
      "]"
    )) %>%
    mutate("Partial R2" = round(median(pR2), digits = 3)) %>%
```

```
    mutate(`Partial R2` = ifelse(`Partial R2` < 0, 0, `Partial R2`)) %>%
    dplyr::select("Partial R\\textsuperscript{2}" = `Partial R2`, "95\\% CI")

  return(ciFrame)
}

caption = paste0('Variance in metabolic slope ',
  '(fold resting metabolsim at thermoneutrality/°C) explained by ',
  'morphometry in three week old Japanese quail. Metabolic ',
  'slopes are measured below thermoneutrality (<30°C).'
  )

models <- list(functionModel3R2Mass, functionModel3WeeksR2Tarsus,
            functionModel3WeeksR2Bill, functionModel3WeeksR2Appendage)
bind_rows(lapply(models, pR2Pull)) %>%
  mutate("Variable" = c("Body Mass", "Tarsus Length",
                        "Bill Length", "Appendage Length")
        ) %>%
  dplyr::select(Variable, "Partial R\\textsuperscript{2}", `95\\% CI`) %>%
  kbl(.,
    longtable = T, booktabs = T, format = "latex",
    caption = caption, escape = FALSE
  ) %>%
  column_spec(column = c(1:10), width = "2.5cm")  %>%
  kable_styling(latex_options = "striped")
```

Table 42: *Variance in metabolic slope (fold resting metabolsim at thermoneutrality/°C) explained by morphometry in three week old Japanese quail. Metabolic slopes are measured below thermoneutrality (<30°C).*

Variable	Partial R^2	95% CI
Body Mass	0.082	[0,0.276]
Tarsus Length	0.024	[0,0.225]
Bill Length	0.002	[0,0.232]
Appendage Length	0.016	[0,0.231]

```
rm(models)
```

To better understand the potential of selection on thermoregulatory efficiency to influence morphology in quail, we estimate the metabolic costs of misalignment with Bergmann's and Allen's rules (i.e. by falling 2 standard deviations below average mass, or 2 standard deviations above average tarsus length or bill length respectively).

```
data.frame(
  "Size" = c("Average", "Small (2x s.d. < mean)"),
  "pretreatment" = "B",
  "mass" = c(
    mean(functionModel3Weeks$data$mass, na.rm = T),
    mean(functionModel3Weeks$data$mass, na.rm = T) -
      2 * sd(functionModel3Weeks$data$mass, na.rm = T)
  ),
  "tarsus" = 0,
  "bill" = 0
) %>%
  mutate("slope" = predict(functionModel3Weeks,
    newdata = .,
    robust = TRUE, re_form = NA,
    resp = "rawSlope"
  )[, "Estimate"]) %>%
  mutate(slope = slope +
    mean(
      subset(
        slopeData,
        week == 3 & pretreatment == "neutral"
```

```
    )$mslope,
    na.rm = T
  )) %>%
  mutate(cost = 1 + slope * -20) %>%
  dplyr::select("Body Size" = Size, "Expenditure Rate at 10°C\n(fold RMR)" = cost) %>%
  kbl(.,
    longtable = T, booktabs = T, format = "latex",
    caption = paste0("Comparison of estimated energy expenditure of ",
                     "three week old Japanese quail at 10°C ",
                     "and varying body mass sizes."),
    escape = FALSE
  ) %>%
  column_spec(column = c(1:10), width = "2.5cm")  %>%
  kable_styling(latex_options = "striped")
```

Table 43: *Comparison of estimated energy expenditure of three week old Japanese quail at 10°C and varying body mass sizes.*

Body Size	Expenditure Rate at 10°C (fold RMR)
Average	1.622611
Small (2x s.d. < mean)	1.746864

```
data.frame(
  "Size" = c("Average", "Long (2x s.d. > mean)"),
  "pretreatment" = "B",
  "tarsus" = c(
    mean(functionModel3Weeks$data$tarsus, na.rm = T),
    mean(functionModel3Weeks$data$tarsus, na.rm = T) +
      2 * sd(functionModel3Weeks$data$tarsus, na.rm = T)
  ),
  "mass" = 0,
  "bill" = 0
) %>%
  mutate("slope" = predict(functionModel3Weeks,
    newdata = .,
    robust = TRUE, re_form = NA,
    resp = "rawSlope"
  )[, "Estimate"]) %>%
  mutate(slope = slope +
    mean(subset(slopeData, week == 3)$mslope, na.rm = T)) %>%
  mutate(cost = 1 + slope * -20) %>%
  dplyr::select(
    "Tarsus Length" = Size,
    "Expenditure Rate at 10°C\n(fold RMR)" = cost
  ) %>%
  kbl(.,
    longtable = T, booktabs = T, format = "latex",
    caption = paste0("Comparison of estimated energy expenditure of ",
                     "three week old Japanese quail at 10°C ",
                     "and varying tarsus lengths."),
    escape = FALSE
  ) %>%
  column_spec(column = c(1:10), width = "2.5cm")  %>%
  kable_styling(latex_options = "striped")
```

Table 44: *Comparison of estimated energy expenditure of three week old Japanese quail at 10°C and varying tarsus lengths.*

Tarsus Length	Expenditure Rate at 10°C (fold RMR)

Average	1.627986
Long (2x s.d. > mean)	1.681426

```
data.frame(
  "Size" = c("Average", "Long (2x s.d. > mean)"),
  "pretreatment" = "B",
  "bill" = c(
    mean(functionModel3Weeks$data$bill, na.rm = T),
    mean(functionModel3Weeks$data$bill, na.rm = T) +
      2 * sd(functionModel3Weeks$data$bill, na.rm = T)
  ),
  "mass" = 0,
  "tarsus" = 0
) %>%
  mutate("slope" = predict(functionModel3Weeks,
    newdata = .,
    robust = TRUE, re_form = NA,
    resp = "rawSlope"
  )[, "Estimate"]) %>%
  mutate(slope = slope +
    mean(subset(slopeData, week == 3)$mslope, na.rm = T)) %>%
  mutate(cost = 1 + slope * -20) %>%
  dplyr::select(
    "Bill Length" = Size,
    "Expenditure Rate at 10°C\n(fold RMR)" = cost
  ) %>%
  kbl(.,
    longtable = T, booktabs = T, format = "latex",
    caption = paste0("Comparison of estimated energy expenditure of ",
                     "three week old Japanese quail at 10°C ",
                     "and varying bill lengths."),
    escape = FALSE
  ) %>%
  column_spec(column = c(1:10), width = "2.5cm")  %>%
  kable_styling(latex_options = "striped")
```

Table 45: *Comparison of estimated energy expenditure of three week old Japanese quail at 10°C and varying bill lengths.*

Bill Length	Expenditure Rate at 10°C (fold RMR)
Average	1.636745
Long (2x s.d. > mean)	1.622014

```
# Checking whether these differences are statistically clear

mismatchDFMass <- data.frame(
  "Size" = c("Average", "Small (2x s.d. < mean)"),
  "pretreatment" = "B",
  "mass" = c(
    mean(functionModel3Weeks$data$mass, na.rm = T),
    mean(functionModel3Weeks$data$mass, na.rm = T) -
      2 * sd(functionModel3Weeks$data$mass, na.rm = T)
  ),
  "tarsus" = 0,
  "bill" = 0
)

mismatchDFTarsus <- data.frame(
  "Size" = c("Average", "Large (2x s.d. > mean)"),
  "pretreatment" = "B",
```

```
  "tarsus" = c(
    mean(functionModel3Weeks$data$tarsus, na.rm = T),
    mean(functionModel3Weeks$data$tarsus, na.rm = T) +
      2 * sd(functionModel3Weeks$data$tarsus, na.rm = T)
  ),
  "mass" = 0,
  "bill" = 0
)

mismatchDFBill <- data.frame(
  "Size" = c("Average", "Large (2x s.d. > mean)"),
  "pretreatment" = "B",
  "bill" = c(
    mean(functionModel3Weeks$data$bill, na.rm = T),
    mean(functionModel3Weeks$data$bill, na.rm = T) +
      2 * sd(functionModel3Weeks$data$bill, na.rm = T)
  ),
  "mass" = 0,
  "tarsus" = 0
)

mismatchTestFun <- function(x) {
  df <- as.data.frame(
    predict(functionModel3Weeks,
    newdata = x,
    robust = TRUE, re_form = NA,
    resp = "rawSlope", summary = FALSE
  )) %>%
    rename("Average" = "V1", "Mismatch" = "V2") %>%
    mutate_all(.funs = function(x) {
      x + mean(subset(slopeData, week == 3)$mslope, na.rm = T)
    })

  mismatchDFTest <- build_hdf(
    vars = list(
      df$Average,
      df$Mismatch
    ),
    priors = list(
      rnorm(nrow(df), 0, 0.01),
      rnorm(nrow(df), 0, 0.01)
    ),
    names = c("Average", "Mismatch")
  )
  mismatchTest <- hypothesis_df("Mismatch > Average",
    mismatchDFTest,
    class = "b", alpha = 0.05
  )

  mismatchTest$hypothesis$Hypothesis <-
    "Mismatch Metabolic Slope > Average Slope"

  return(mismatchTest)
}

mismatchTests <- lapply(
  list(mismatchDFMass, mismatchDFTarsus, mismatchDFBill),
  mismatchTestFun
  )

# Summarising

caption <- paste0(
  "Results of a non-linear hypothesis ",
  "tests comparing predicted metabolic slopes in the cold (30°C - 10°C) ",
  "among three week old Japanese quail of average or atypically small ",
  "body size (mean - 2 standard deviations). Posterior probabilities are ",
```

```
  "calculated using the Savage-Dickey ",
  "density ratio method."
)

mismatchTestMassTable <- mismatchTests[[1]]$hypothesis %>%
  mutate(Hypothesis = c(
    "Small Metabolic Slope > Average Slope"
  )) %>%
  dplyr::select(-c(Evid.Ratio, Star)) %>%
  mutate(
    Estimate = round(Estimate, digits = 4),
    "Est.Error" = round(Est.Error, digits = 4),
    "CI.Lower" = round(CI.Lower, digits = 4),
    "CI.Upper" = round(CI.Upper, digits = 4),
    "Posterior Probability" = round(Post.Prob, digits = 4)
  ) %>%
  rename(
    "Difference Between Metabolic Slopes" = Estimate,
    "Standard Error" = Est.Error,
    `2.5 % CI` = "CI.Lower",
    `97.5 % CI` = "CI.Upper"
  ) %>%
  dplyr::select(-Post.Prob) %>%
  kbl(.,
    longtable = T, booktabs = T, format = "latex",
    caption = caption
  ) %>%
  column_spec(column = c(1:2), width = "2.5cm") %>%
  column_spec(column = c(3:10), width = "2cm") %>%
  kable_styling(latex_options = "striped")

mismatchTestMassTable
```

Table 46: *Results of a non-linear hypothesis tests comparing predicted metabolic slopes in the cold (30°C - 10°C) among three week old Japanese quail of average or atypically small body size (mean - 2 standard deviations). Posterior probabilities are calculated using the Savage-Dickey density ratio method.*

Hypothesis	Difference Between Metabolic Slopes	Standard Error	2.5 % CI	97.5 % CI	Posterior Probability
Small Metabolic Slope > Average Slope	-0.006	0.0132	-0.0272	0.0155	0.325

```
# For tarsus length

caption <- paste0(
  "Results of a non-linear hypothesis ",
  "tests comparing predicted metabolic slopes in the cold (30°C - 10°C) ",
  "among three week old Japanese quail of average or atypically long ",
  "tarsus length (mean + 2 standard deviations). Posterior probabilities are ",
  "calculated using the Savage-Dickey ",
  "density ratio method."
)

mismatchTestTarsusTable <- mismatchTests[[2]]$hypothesis %>%
  mutate(Hypothesis = c(
    "Long Metabolic Slope > Average Slope"
  )) %>%
  dplyr::select(-c(Evid.Ratio, Star)) %>%
  mutate(
    Estimate = round(Estimate, digits = 4),
    "Est.Error" = round(Est.Error, digits = 4),
    "CI.Lower" = round(CI.Lower, digits = 4),
    "CI.Upper" = round(CI.Upper, digits = 4),
    "Posterior Probability" = round(Post.Prob, digits = 4)
```

```
  ) %>%
  rename(
    "Difference Between Metabolic Slopes" = Estimate,
    "Standard Error" = Est.Error,
    `2.5 % CI` = "CI.Lower",
    `97.5 % CI` = "CI.Upper"
  ) %>%
  dplyr::select(-Post.Prob) %>%
  kbl(.,
    longtable = T, booktabs = T, format = "latex",
    caption = caption
  ) %>%
  column_spec(column = c(1:2), width = "2.5cm") %>%
  column_spec(column = c(3:10), width = "2cm") %>%
  kable_styling(latex_options = "striped")

mismatchTestTarsusTable
```

Table 47: *Results of a non-linear hypothesis tests comparing predicted metabolic slopes in the cold (30°C - 10°C) among three week old Japanese quail of average or atypically long tarsus length (mean + 2 standard deviations). Posterior probabilities are calculated using the Savage-Dickey density ratio method.*

Hypothesis	Difference Between Metabolic Slopes	Standard Error	2.5 % CI	97.5 % CI	Posterior Probability
Long Metabolic Slope > Average Slope	-0.0023	0.0136	-0.0249	0.0195	0.4367

```
## Bill length

caption <- paste0(
  "Results of a non-linear hypothesis ",
  "tests comparing predicted metabolic slopes in the cold (30°C - 10°C) ",
  "among three week old Japanese quail of average or atypically long ",
  "bill length (mean + 2 standard deviations). Posterior probabilities are ",
  "calculated using the Savage-Dickey ",
  "density ratio method."
)

mismatchTestBillTable <- mismatchTests[[3]]$hypothesis %>%
  mutate(Hypothesis = c(
    "Long Metabolic Slope > Average Slope"
  )) %>%
  dplyr::select(-c(Evid.Ratio, Star)) %>%
  mutate(
    Estimate = round(Estimate, digits = 4),
    "Est.Error" = round(Est.Error, digits = 4),
    "CI.Lower" = round(CI.Lower, digits = 4),
    "CI.Upper" = round(CI.Upper, digits = 4),
    "Posterior Probability" = round(Post.Prob, digits = 4)
  ) %>%
  rename(
    "Difference Between Metabolic Slopes" = Estimate,
    "Standard Error" = Est.Error,
    `2.5 % CI` = "CI.Lower",
    `97.5 % CI` = "CI.Upper"
  ) %>%
  dplyr::select(-Post.Prob) %>%
  kbl(.,
    longtable = T, booktabs = T, format = "latex",
    caption = caption
  ) %>%
  column_spec(column = c(1:2), width = "2.5cm") %>%
  column_spec(column = c(3:10), width = "2cm") %>%
  kable_styling(latex_options = "striped")
```

```
mismatchTestBillTable
```

Table 48: *Results of a non-linear hypothesis tests comparing predicted metabolic slopes in the cold (30°C - 10°C) among three week old Japanese quail of average or atypically long bill length (mean + 2 standard deviations). Posterior probabilities are calculated using the Savage-Dickey density ratio method.*

Hypothesis	Difference Between Metabolic Slopes	Standard Error	2.5 % CI	97.5 % CI	Posterior Probability
Long Metabolic Slope > Average Slope	4e-04	0.0127	-0.0205	0.0211	0.5124

Any effects of prior thermal acclimation or thermal history (i.e. via our distinct rearing treatments) on metabolic slopes may be direct, indirect through their influence on morphology, or both. Using outcomes from our path analysis, we visualise possible contributions of each pathway below. To both ease interpretation and comparison of effects, each are first scaled to represent the effect that a change in one standard deviation (or one categorical level) of a variable is predicted to have on another.

```
# Visualising indirect and direct effects

scaledBetas <- as.data.frame(functionModel3Weeks) %>%
  mutate(
    b_mass_pretreatmentA = b_mass_pretreatmentA /
      sd(functionModel3Weeks$data$mass),
    b_mass_pretreatmentC = b_mass_pretreatmentC /
      sd(functionModel3Weeks$data$mass),
    b_tarsus_pretreatmentA = b_tarsus_pretreatmentA /
      sd(functionModel3Weeks$data$tarsus),
    b_tarsus_pretreatmentC = b_tarsus_pretreatmentC /
      sd(functionModel3Weeks$data$tarsus),
    b_tarsus_mass =
      (b_tarsus_mass * sd(functionModel3Weeks$data$mass)) /
        sd(functionModel3Weeks$data$tarsus),
    b_bill_pretreatmentA = b_bill_pretreatmentA /
      sd(functionModel3Weeks$data$bill),
    b_bill_pretreatmentC = b_tarsus_pretreatmentC /
      sd(functionModel3Weeks$data$bill),
    b_bill_mass =
      (b_bill_mass * sd(functionModel3Weeks$data$mass)) /
        sd(functionModel3Weeks$data$bill),
    b_rawSlope_mass =
      (b_rawSlope_mass * sd(functionModel3Weeks$data$mass)) /
        sd(functionModel3Weeks$data$rawSlope),
    b_rawSlope_tarsus =
      (b_rawSlope_tarsus * sd(functionModel3Weeks$data$tarsus)) /
        sd(functionModel3Weeks$data$rawSlope),
    b_rawSlope_bill =
      (b_rawSlope_bill * sd(functionModel3Weeks$data$bill)) /
        sd(functionModel3Weeks$data$rawSlope),
    b_rawSlope_pretreatmentA = b_rawSlope_pretreatmentA /
      sd(functionModel3Weeks$data$rawSlope),
    b_rawSlope_pretreatmentC = b_rawSlope_pretreatmentC /
      sd(functionModel3Weeks$data$rawSlope),
  )

fullEffectThreeWeeksBetas <- scaledBetas %>%
  mutate("Effects" = "Direct Effects") %>%
  mutate(
    "Body Mass" = b_rawSlope_mass,
    "Tarsus Length" = b_rawSlope_tarsus,
    "Bill Length" = b_rawSlope_bill,
    "Cold Rearing\n(10°C)" = b_rawSlope_pretreatmentA,
```

```
    "Warm Rearing\n(30°C)" = b_rawSlope_pretreatmentC
  ) %>%
  dplyr::select(
    Effects, `Body Mass`, `Tarsus Length`, `Bill Length`,
    `Cold Rearing\n(10°C)`,
    `Warm Rearing\n(30°C)`
  ) %>%
  rbind(
    .,
    scaledBetas %>%
      mutate("Effects" = "Indirect Effects") %>%
      mutate(
        "Body Mass" = b_tarsus_mass *
          b_rawSlope_tarsus +
          b_bill_mass *
            b_rawSlope_bill,
        "Tarsus Length" = NA,
        "Bill Length" = NA,
        "Cold Rearing\n(10°C)" =
          b_mass_pretreatmentA * b_rawSlope_mass +
            b_tarsus_pretreatmentA * b_rawSlope_tarsus +
            b_tarsus_pretreatmentA * b_rawSlope_bill +
            b_mass_pretreatmentA * b_tarsus_mass *
              b_rawSlope_tarsus +
            b_mass_pretreatmentA * b_bill_mass *
              b_rawSlope_bill,
        "Warm Rearing\n(30°C)" =
          b_mass_pretreatmentC * b_rawSlope_mass +
            b_tarsus_pretreatmentC * b_rawSlope_tarsus +
            b_tarsus_pretreatmentC * b_rawSlope_bill +
            b_mass_pretreatmentC * b_tarsus_mass *
              b_rawSlope_tarsus +
            b_mass_pretreatmentC * b_bill_mass *
              b_rawSlope_bill
      ) %>%
      dplyr::select(
        Effects, `Body Mass`, `Tarsus Length`, `Bill Length`,
        `Cold Rearing\n(10°C)`, `Warm Rearing\n(30°C)`
      )
  ) %>%
  rbind(., scaledBetas %>%
    mutate("Effects" = "Total Effects") %>%
    mutate(
      "Body Mass" =
        b_rawSlope_mass +
          b_tarsus_mass * b_rawSlope_tarsus +
          b_bill_mass * b_rawSlope_bill,
      "Tarsus Length" =
        b_rawSlope_tarsus,
      "Bill Length" =
        b_rawSlope_bill,
      "Cold Rearing\n(10°C)" =
        b_rawSlope_pretreatmentA +
          b_mass_pretreatmentA *
            b_rawSlope_mass +
          b_tarsus_pretreatmentA *
            b_rawSlope_tarsus +
          b_bill_pretreatmentA *
            b_rawSlope_bill +
          b_mass_pretreatmentA *
            b_tarsus_mass * b_rawSlope_tarsus +
          b_mass_pretreatmentA *
            b_bill_mass * b_rawSlope_bill,
      "Warm Rearing\n(30°C)" =
        b_rawSlope_pretreatmentC +
          b_mass_pretreatmentC *
            b_rawSlope_mass +
```

```
          b_tarsus_pretreatmentC *
            b_rawSlope_tarsus +
          b_bill_pretreatmentC *
            b_rawSlope_bill +
          b_mass_pretreatmentC *
            b_tarsus_mass * b_rawSlope_tarsus +
          b_mass_pretreatmentC *
            b_bill_mass * b_rawSlope_bill,
    ) %>%
    dplyr::select(
      Effects, `Body Mass`, `Tarsus Length`, `Bill Length`,
      `Cold Rearing\n(10°C)`, `Warm Rearing\n(30°C)`
    )) %>%
  pivot_longer(c(-Effects), names_to = "var", values_to = "values") %>%
  mutate(var = factor(var,
    levels = c(
      "Body Mass",
      "Tarsus Length",
      "Bill Length",
      "Warm Rearing\n(30°C)",
      "Cold Rearing\n(10°C)"
    )
  ))

fullEffectThreeWeeks <- fullEffectThreeWeeksBetas %>%
  ggplot(aes(x = values, y = var, fill = var)) +
  facet_wrap(~Effects) +
  stat_halfeye(normalize = "xy", colour = "black", alpha = 0.7) +
  geom_vline(xintercept = 0, linetype = "dashed",
             colour = "black") +
  xlab(".                  Effect on Metabolic Slope (standard deviations)") +
  scale_fill_manual(values = c("black", "grey40", "grey80", "#CD5C5C", "#7BB4E3")) +
  theme_classic() +
  theme(
    legend.position = "none", axis.title.y = element_blank(),
    axis.text.y = element_text(size = 11, colour = "black",
                               family = "Noto Sans"),
    axis.title.x = element_text(family = "Noto Sans", hjust = -0.005),
    axis.text.x = element_text(family = "Noto Sans")
  )

showtext_auto(enable = TRUE)

fullEffectThreeWeeks
```

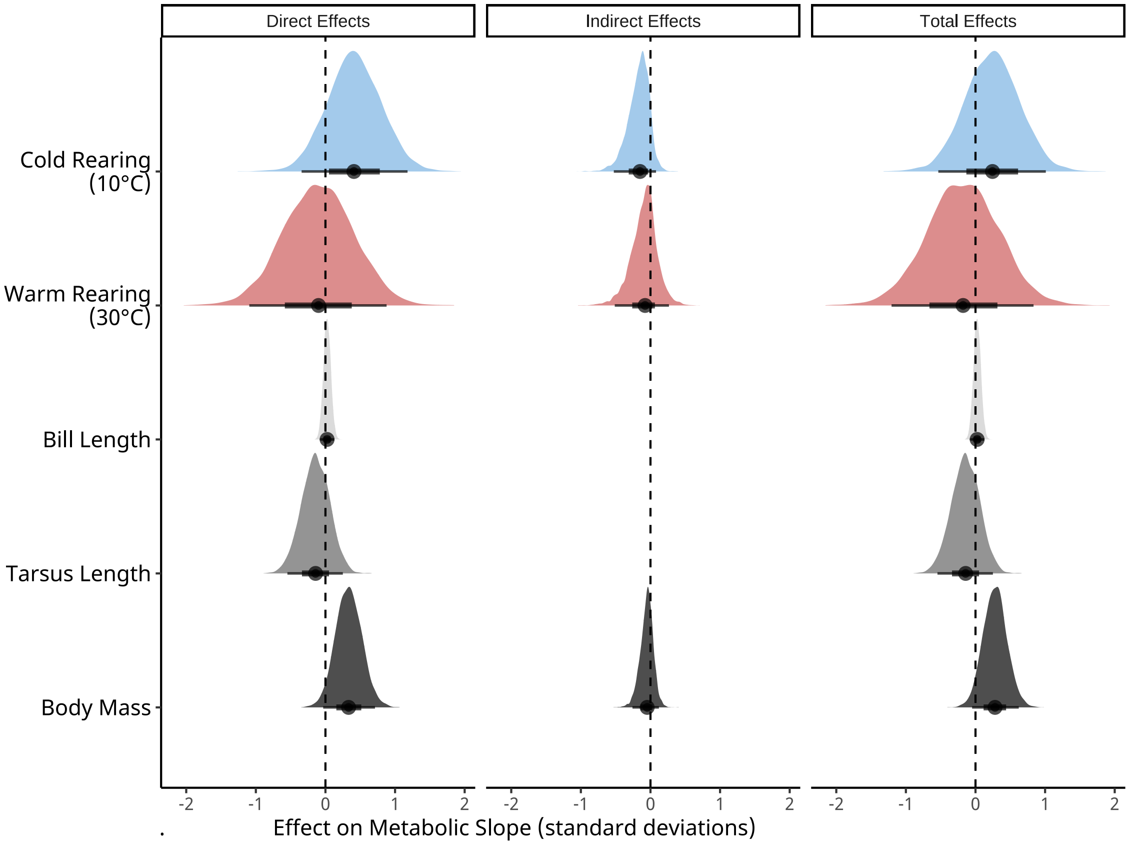

Figure 97: *Effects of morphology (body mass, g, tarsus length, mm, bill length, mm) on metabolic slope (in standard deviations) at ambient temperatures below thermoneutrality (<30°C) in three week old Japanese quail. Distributions represent those derived from posteriors of a Bayesian path analysis.*

```
ggsave("./plots/indirectEffectsOnResistance3Weeks_3ScaledFold.pdf",
       fullEffectThreeWeeks,
  dpi = 800, width = 8, height = 6
)
showtext_auto(enable = FALSE)

# Summarising results

caption <- paste0(
  "Direct, indirect, and total effects of morphology and ",
  "rearing temperature on metabolic slope ",
  "(fold metabolism at thermoneutrality/°C) ",
  "of three week old Japanese quail. Effects are derived from ",
  "a Bayesian path analysis and represent those predicted for a ",
  "change in one standard deviation (or categorical level) of ",
  "a given predictor on the standard deviation of metabolic ",
  "slopes. Estimates indicate posterior medians and credible ",
  "intervels (CIs) indicate quantile intervals."
)

week3ResultsScaled <-
  fullEffectThreeWeeksBetas %>%
  filter(!is.na(values) & !is.nan(values)) %>%
  group_by(Effects, var) %>%
  summarise(
    "Estimate" = median(values),
    "50\\% CIs" = paste0(
      "[",
```

```
      round(
        quantile(values, probs = 0.1, type = 8),
        digits = 4
      ),
      ", ",
      round(
        quantile(values, probs = 0.9, type = 8),
        digits = 4
      ),
      "]"
    ),
    "95\\% CIs" = paste0(
      "[",
      round(
        quantile(values, probs = 0.025, type = 8),
        digits = 4
      ),
      ", ",
      round(
        quantile(values, probs = 0.975, type = 8),
        digits = 4
      ),
      "]"
    )
  ) %>%
  mutate("Effects" = gsub("[[:space:]].*", "", Effects)) %>%
  dplyr::select(
    "Predictor" = "var", "Effect Level" = "Effects",
    Estimate, `50\\% CIs`, `95\\% CIs`
  ) %>%
  arrange(Predictor, `Effect Level`) %>%
  kbl(.,
    longtable = T, booktabs = T, format = "latex", escape = FALSE,
    caption = caption
  ) %>%
  column_spec(column = c(1:2), width = "2.4cm") %>%
  column_spec(column = c(3:10), width = "2.2cm") %>%
  kable_styling(latex_options = "striped")

week3ResultsScaled
```

Table 49: *Direct, indirect, and total effects of morphology and rearing temperature on metabolic slope (fold metabolism at thermoneutrality/°C) of three week old Japanese quail. Effects are derived from a Bayesian path analysis and represent those predicted for a change in one standard deviation (or categorical level) of a given predictor on the standard deviation of metabolic slopes. Estimates indicate posterior medians and credible intervels (CIs) indicate quantile intervals.*

Predictor	Effect Level	Estimate	50% CIs	95% CIs
Body Mass	Direct	0.3327955	[0.0974, 0.574]	[-0.0325, 0.7124]
Body Mass	Indirect	-0.0469351	[-0.1789, 0.0585]	[-0.2589, 0.1212]
Body Mass	Total	0.2806079	[0.063, 0.4988]	[-0.0489, 0.6218]
Tarsus Length	Direct	-0.1416251	[-0.4059, 0.1143]	[-0.5472, 0.2505]
Tarsus Length	Total	-0.1416251	[-0.4059, 0.1143]	[-0.5472, 0.2505]
Bill Length	Direct	0.0236645	[-0.0426, 0.0904]	[-0.0763, 0.1273]
Bill Length	Total	0.0236645	[-0.0426, 0.0904]	[-0.0763, 0.1273]
Warm Rearing (30°C)	Direct	-0.0994331	[-0.7415, 0.5526]	[-1.0951, 0.8784]
Warm Rearing (30°C)	Indirect	-0.0773322	[-0.3317, 0.1252]	[-0.5128, 0.2654]
Warm Rearing (30°C)	Total	-0.1780980	[-0.839, 0.4749]	[-1.2069, 0.8365]
Cold Rearing (10°C)	Direct	0.4091021	[-0.084, 0.9097]	[-0.343, 1.1795]
Cold Rearing (10°C)	Indirect	-0.1508374	[-0.3766, -1e-04]	[-0.5269, 0.0798]

Cold Rearing (10°C)	Total	0.2442511	[-0.2566, 0.7455]	[-0.5346, 1.0102]

```
#save_kable(week3ResultsScaled, "../tables/threeWeekResults_3Scaled.html")
```

To alleviate concerns over possible collinearity between body mass and tarsus length or bill length in our previous model, our path analysis is re-run but while relativising both tarsus length and bill length by body mass prior to use in our models. This was achieved by first regressing each measure against mean-centred body mass (tarsus length priors: intercept = $\mathcal{N}[30, 2.5]$; body mass = $\mathcal{SN}[0, 0.25, 5]$; bill length priors: intercept = $\mathcal{N}[9, 1]$; body mass = $\mathcal{SN}[0, 0.25, 5]$), then extracting ordinary residuals from the regression and replacing true length values with these values in our path analysis; body mass is then removed as a predictor of tarsus and bill length in our analysis. Priors for all model parameters remained the same as described for our previous path analysis (with variables unscaled).

```
# Note that our prior for the intercept of tarsus length (here, the mean,
# since body mass is mean-centred) is informed from Gebhardt-Henrich
# & Mark (1993: Gen Res Camb) and van der Ziel & Visser
# (2001; Physiol Biochem Zool).

resModData <- filter(slopeData, week == "3") %>%
    mutate(
      massCentred = mass - mean(mass, na.rm = T),
      pretreatment = ifelse(pretreatment == "cold", "A",
        ifelse(pretreatment == "neutral", "B", "C")
      )
    ) %>%
    mutate(pretreatment = factor(pretreatment, levels = c("B", "A", "C"))) %>%
    dplyr::select(-mass) %>%
    rename("mass" = massCentred) %>%
    drop_na() %>%
    merge(., data %>%
      dplyr::select(ring, "batch" = exp) %>%
      distinct(),
    by = "ring", all.x = TRUE
    )

residTarsusModel <- brm(
  bf(tarsusLengthMean ~ mass),
  data = resModData,
  prior = c(
    set_prior("skew_normal(0, 0.25, 5)",
      class = "b"
    ),
    set_prior("normal(30, 2.5)",
      class = "Intercept"
    )
  ),
  family = "gaussian",
  iter = 50000, warmup = 10000, cores = 1,
  chains = 4, thin = 20,
  silent = TRUE, refresh = 0,
  file = "./models/_resTarsus3WeeksCold.Rds"
)

residBillModel <- brm(
  bf(billLengthMean ~ mass),
  data = resModData,
  prior = c(
    set_prior("skew_normal(0, 0.25, 5)",
      class = "b"
    ),
    set_prior("normal(9, 1)",
      class = "Intercept"
    )
```

```
  ),
  family = "gaussian",
  iter = 50000, warmup = 10000, cores = 1,
  chains = 4, thin = 20,
  silent = TRUE, refresh = 0,
  file = "./models/_resBill3WeeksCold.Rds"
)

functionModel3WeeksResidual <- brm(
  data = resModData %>%
    mutate(
      "residualTarsus" =
        residuals(residTarsusModel)[,"Estimate"],
      "residualBill" =
        residuals(residBillModel)[,"Estimate"]
    ),
  family = "gaussian",
  bf(mass ~ pretreatment + (1 | batch)) +
    bf(residualTarsus ~ pretreatment + (1 | batch)) +
    bf(residualBill ~ pretreatment + (1 | batch)) +
    bf(rawSlope ~ mass + residualTarsus + residualBill + pretreatment + (1 | batch)) +
    set_rescor(rescor = FALSE),
  prior = c(
      set_prior("normal(0, 5)",
        class = "Intercept",
        resp = "mass"
      ),
      set_prior("normal(0, 15)",
        class = "b",
        coef = "pretreatmentA",
        resp = "mass"
      ),
      set_prior("normal(0, 15)",
        class = "b",
        coef = "pretreatmentC",
        resp = "mass"
      ),
      set_prior("exponential(2.5)",
        class = "sd",
        group = "batch",
        resp = "mass"
      ),
      set_prior("exponential(0.15)",
        class = "sigma",
        resp = "mass"
      ),
      set_prior("normal(0, 2.5)",
        class = "Intercept",
        resp = "residualTarsus"
      ),
      set_prior("normal(0, 2.5)",
        class = "b",
        coef = "pretreatmentA",
        resp = "residualTarsus"
      ),
      set_prior("normal(0, 2.5)",
        class = "b",
        coef = "pretreatmentC",
        resp = "residualTarsus"
      ),
      set_prior("exponential(2)",
        class = "sd",
        group = "batch",
        resp = "residualTarsus"
      ),
      set_prior("exponential(1)",
        class = "sigma",
```

```
        resp = "residualTarsus"
      ),
      set_prior("normal(0, 1)",
        class = "Intercept",
        resp = "residualBill"
      ),
      set_prior("normal(0, 0.5)",
        class = "b",
        coef = "pretreatmentA",
        resp = "residualBill"
      ),
      set_prior("normal(0, 0.5)",
        class = "b",
        coef = "pretreatmentC",
        resp = "residualBill"
      ),
      set_prior("exponential(5)",
        class = "sd",
        group = "batch",
        resp = "residualBill"
      ),
      set_prior("exponential(2.5)",
        class = "sigma",
        resp = "residualBill"
      ),
      set_prior("normal(0, 0.01)",
        class = "Intercept",
        resp = "rawSlope"
      ),
      set_prior("normal(0, 0.025)",
        class = "b",
        coef = "pretreatmentA",
        resp = "rawSlope"
      ),
      set_prior("normal(0, 0.025)",
        class = "b",
        coef = "pretreatmentC",
        resp = "rawSlope"
      ),
      set_prior("normal(0, 0.001)",
        class = "b",
        coef = "mass",
        resp = "rawSlope"
      ),
      set_prior("normal(0, 0.0025)",
        class = "b",
        coef = "residualTarsus",
        resp = "rawSlope"
      ),
      set_prior("normal(0, 0.001)",
        class = "b",
        coef = "residualBill",
        resp = "rawSlope"
      ),
      set_prior("exponential(50)",
        class = "sd",
        group = "batch",
        resp = "rawSlope"
      ),
      set_prior("exponential(10)",
        class = "sigma",
        resp = "rawSlope"
      )
    ),
  iter = 50000, warmup = 10000, cores = 4, chains = 4, thin = 20,
  control = list(adapt_delta = .96),
  silent = TRUE, refresh = 0,
```

```
  file = "./models/_threeWeekFunctionModelResidual.Rds"
)

# Running quick diagnostics. Note that these are not fulsome,
# given the supplemental nature of the analysis.

chainCheck(functionModel3WeeksResidual)
```

```
## Rhat range: 1 - 1.001
## Neff/N range: 0.913 - 1.007
```

```
pp_check2(functionModel3WeeksResidual,
  resp = "rawSlope",
  xlab = "Metabolic Slope\n(fold Metabolism at Thermoneutrality)"
  )
```

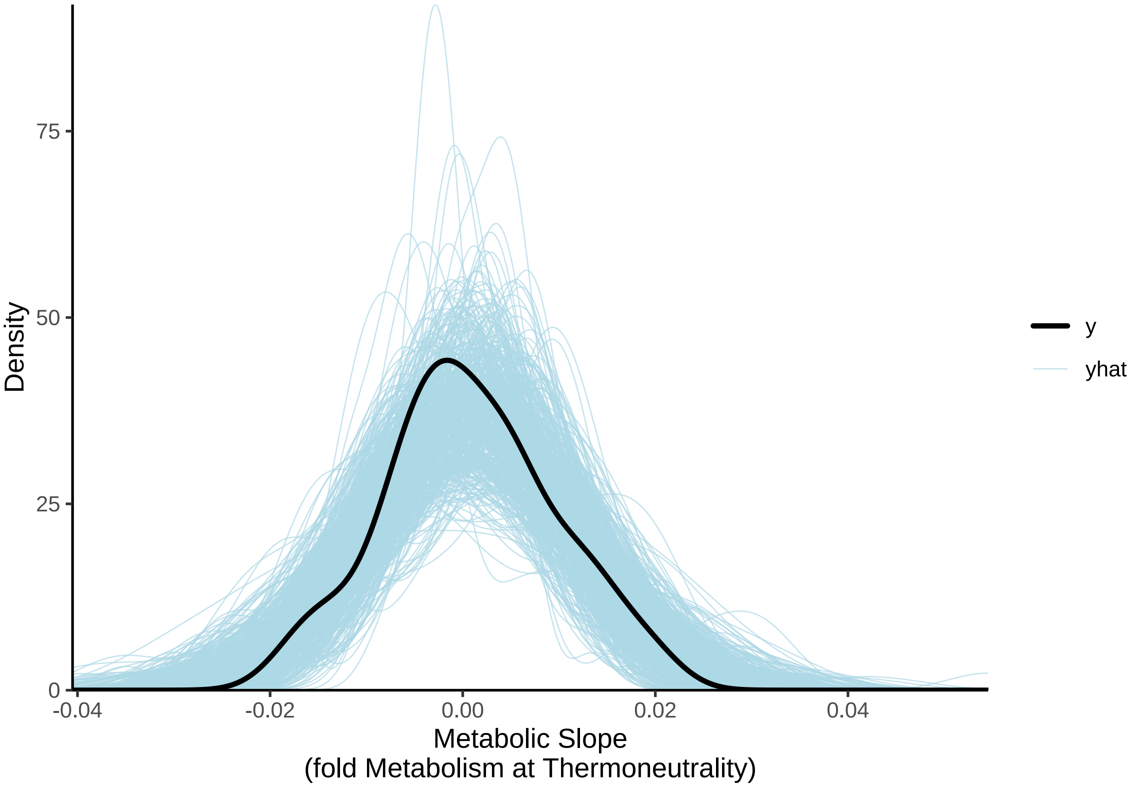

Figure 98: *Posterior predictive check for a Bayesian path analysis predicting metabolic slope (fold metabolism at thermoneutrality/°C) of three week old Japanese quail by morphometry. Light blue lines represent densities of resting metabolism values as drawn from model posteriors. The dark blue line represents the true density of resting metabolism values. Clear overlap between the dark blue and light blue lines indicates strong model fit. Morphometry measures include body mass (g), tarsus length residuals (mm), and bill length residuals (mm).*

Density of posterior predictions overlays clearly with true density of metabolic slope values. Below, we briefly visualise the spread of residuals, autocorrelation factors in chains (per predictor) and posterior densities (again, per predictor).

```
lbs <- c("Mass ~ Cold Rearing")
names(lbs) <- c("b_mass_pretreatmentA")
p1 <- mcmc_acf(functionModel3WeeksResidual,
  pars = "b_mass_pretreatmentA", lags = 10,
  facet_args = list(labeller = labeller(Parameter = lbs))
```

```
) +
  theme_classic()

lbs <- c("Mass ~ Warm Rearing")
names(lbs) <- c("b_mass_pretreatmentC")
p2 <- mcmc_acf(functionModel3WeeksResidual,
  pars = "b_mass_pretreatmentC", lags = 10,
  facet_args = list(labeller = labeller(Parameter = lbs))
) +
  theme_classic()

lbs <- c("Tarsus ~ Cold Rearing")
names(lbs) <- c("b_residualTarsus_pretreatmentA")
p3 <- mcmc_acf(functionModel3WeeksResidual,
  pars = "b_residualTarsus_pretreatmentA", lags = 10,
  facet_args = list(labeller = labeller(Parameter = lbs))
) +
  theme_classic()

lbs <- c("Tarsus ~ Warm Rearing")
names(lbs) <- c("b_residualTarsus_pretreatmentC")
p4 <- mcmc_acf(functionModel3WeeksResidual,
  pars = "b_residualTarsus_pretreatmentC", lags = 10,
  facet_args = list(labeller = labeller(Parameter = lbs))
) +
  theme_classic()

lbs <- c("Bill ~ Cold Rearing")
names(lbs) <- c("b_residualBill_pretreatmentA")
p5 <- mcmc_acf(functionModel3WeeksResidual,
  pars = "b_residualBill_pretreatmentA", lags = 10,
  facet_args = list(labeller = labeller(Parameter = lbs))
) +
  theme_classic()

lbs <- c("Bill ~ Warm Rearing")
names(lbs) <- c("b_residualBill_pretreatmentC")
p6 <- mcmc_acf(functionModel3WeeksResidual,
  pars = "b_residualBill_pretreatmentC", lags = 10,
  facet_args = list(labeller = labeller(Parameter = lbs))
) +
  theme_classic()

lbs <- c("Slope ~ Mass")
names(lbs) <- c("b_rawSlope_mass")
p7 <- mcmc_acf(functionModel3WeeksResidual,
  pars = "b_rawSlope_mass", lags = 10,
  facet_args = list(labeller = labeller(Parameter = lbs))
) +
  theme_classic()

lbs <- c("Slope ~ Tarsus")
names(lbs) <- c("b_rawSlope_residualTarsus")
p8 <- mcmc_acf(functionModel3WeeksResidual,
  pars = "b_rawSlope_residualTarsus", lags = 10,
  facet_args = list(labeller = labeller(Parameter = lbs))
) +
  theme_classic()

lbs <- c("Slope ~ Bill")
names(lbs) <- c("b_rawSlope_residualBill")
p9 <- mcmc_acf(functionModel3WeeksResidual,
  pars = "b_rawSlope_residualBill", lags = 10,
  facet_args = list(labeller = labeller(Parameter = lbs))
) +
  theme_classic()
```

```
lbs <- c("Slope ~ Cold")
names(lbs) <- c("b_rawSlope_pretreatmentA")
p10 <- mcmc_acf(functionModel3WeeksResidual,
  pars = "b_rawSlope_pretreatmentA", lags = 10,
  facet_args = list(labeller = labeller(Parameter = lbs))
) +
  theme_classic()

lbs <- c("Slope ~ Warm")
names(lbs) <- c("b_rawSlope_pretreatmentC")
p11 <- mcmc_acf(functionModel3WeeksResidual,
  pars = "b_rawSlope_pretreatmentC", lags = 10,
  facet_args = list(labeller = labeller(Parameter = lbs))
) +
  theme_classic()

p12 <- functionModel3WeeksResidual$data %>%
  mutate("Res" =
           residuals(functionModel3WeeksResidual,
                     resp = "rawSlope",
                     robust = TRUE)[, "Estimate"]) %>%
  ggplot(aes(sample = Res)) +
  stat_qq(colour = "grey50") +
  stat_qq_line() +
  xlab("Theoretical") +
  ylab("Sample") +
  theme_classic()

(p1 + p2 + p3) /
  (p4 + p5 + p6) /
  (p7 + p8 + p9) /
  (p10 + p11 + p12)
```

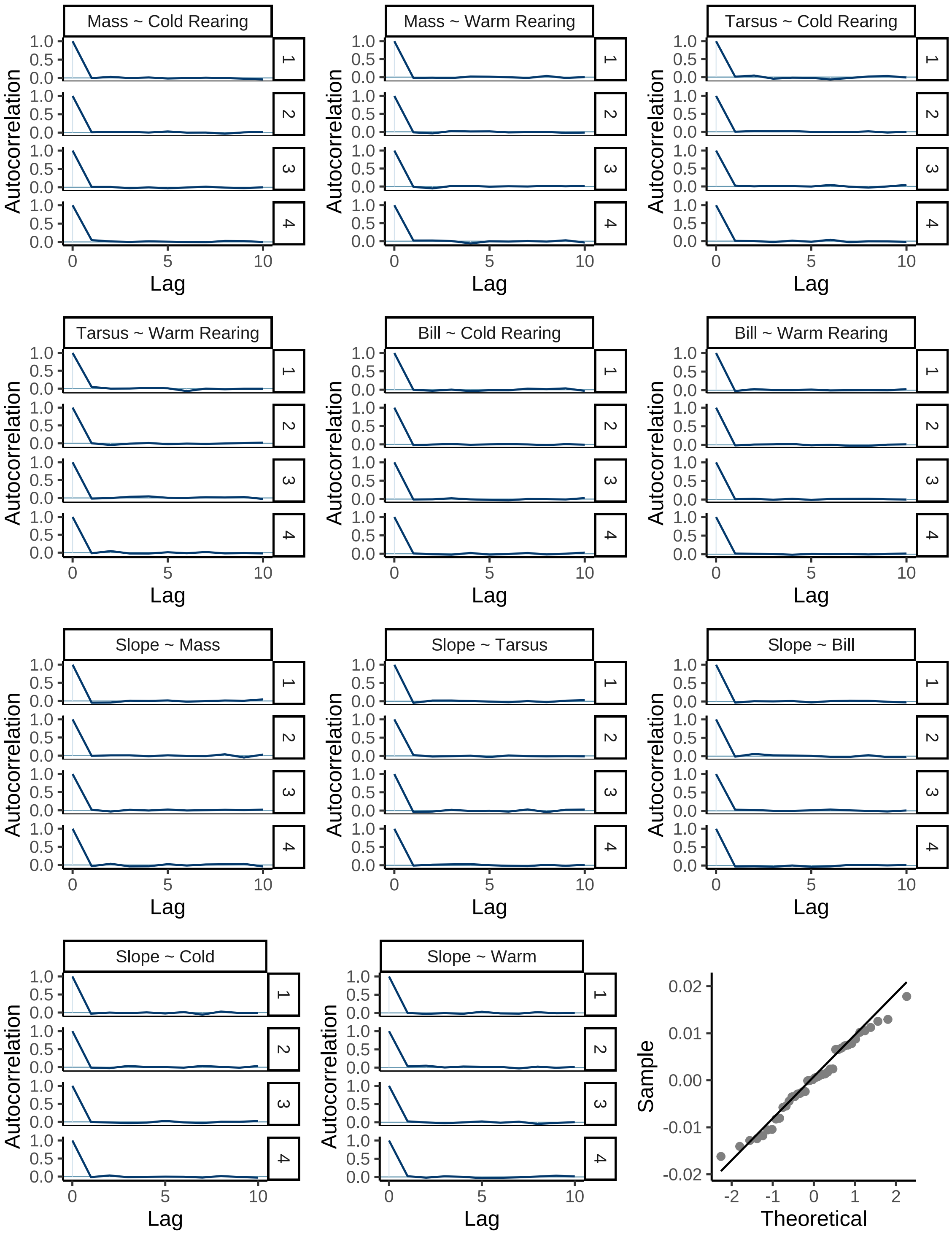

Figure 99: *Diagnostics for a Bayesian path analysis predicting metabolic slope in the cold (fold metabolism at thermoneutrality/°C) of three week old Japanese quail by morphometry (here, body mass [g], residual tarsus length [mm], residual bill length [mm]). Autocorrelation plots display autocorrelation between adjacent Hamiltonian Monte Carlo chain draws, divided by chain. QQ-plot displays sample ordinary residuals by theoretical residual quantiles, and fitted values are displayed against true, mean-centred metabolic slope values.*

```
# Plotting coefficients

as.data.frame(functionModel3WeeksResidual) %>%
    dplyr::select(
      "Intercept" = b_rawSlope_Intercept,
      "Body Mass (g)" = b_rawSlope_mass,
      "Residual Tarsus (mm)" =
        b_rawSlope_residualTarsus,
      "Residual Bill (mm)" =
        b_rawSlope_residualBill,
      "Cold Rearing\n(10°C)" =
        b_rawSlope_pretreatmentA,
      "Warm Rearing\n(30°C)" =
        b_rawSlope_pretreatmentC
    ) %>%
    pivot_longer(everything(), names_to = "Par",
                 values_to = "Coefs") %>%
    ggplot(aes(x = Coefs)) +
    facet_wrap(~Par, scales = "free", ncol = 2) +
    geom_density(colour = "black", alpha = 0.5,
                 fill = "grey70") +
    geom_vline(xintercept = 0, linetype = "dashed",
               colour = "black") +
    ylab("Density") +
    theme_classic() +
    theme(axis.title.x = element_blank())
```

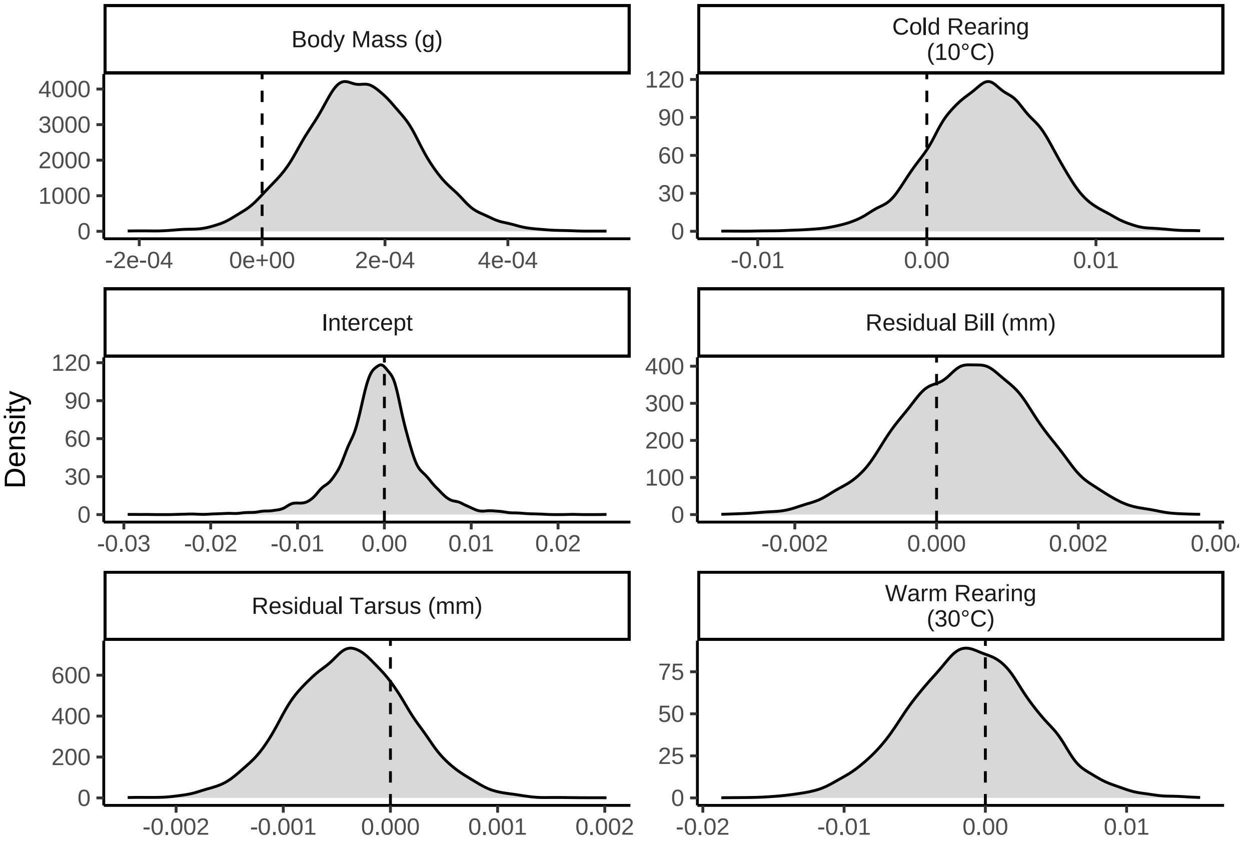

Figure 100: *Posterior densities for model coefficients derived from a Bayesian path analysis predicting metabolic slope (fold metabolism at thermoneutrality/°C) of three week old Japanese quail in the cold (10°C - 30°C). Vertical dashed lines indicate 0.*

```
ggarrange(
  as.data.frame(functionModel3WeeksResidual) %>%
    dplyr::select(
      "Intercept" = b_mass_Intercept,
      "Cold Rearing" = b_mass_pretreatmentA,
      "Warm Rearing" = b_mass_pretreatmentC,
      "Sigma" = sigma_mass
    ) %>%
    pivot_longer(everything(), names_to = "Par", values_to = "Coefs") %>%
    ggplot(aes(x = Coefs)) +
    facet_wrap(~Par, scales = "free") +
    geom_density(colour = "black", alpha = 0.5, fill = "grey70") +
    geom_vline(xintercept = 0, linetype = "dashed", colour = "black") +
    ylab("Density") +
    theme_classic() +
    theme(axis.title.x = element_blank()) +
    ggtitle("Body Mass (g)"),

  as.data.frame(functionModel3WeeksResidual) %>%
    dplyr::select(
      "Intercept" = b_residualTarsus_Intercept,
      "Cold Rearing" = b_residualTarsus_pretreatmentA,
      "Warm Rearing" = b_residualTarsus_pretreatmentC,
      "Sigma" = sigma_residualTarsus
    ) %>%
    pivot_longer(everything(), names_to = "Par",
                 values_to = "Coefs") %>%
    ggplot(aes(x = Coefs)) +
    facet_wrap(~Par, scales = "free") +
    geom_density(colour = "black", alpha = 0.5, fill = "grey70") +
    geom_vline(xintercept = 0, linetype = "dashed", colour = "black") +
    ylab("Density") +
    theme_classic() +
    theme(axis.title.x = element_blank()) +
    ggtitle("Residual Tarsus Length (mm)"),

  as.data.frame(functionModel3WeeksResidual) %>%
    dplyr::select(
      "Intercept" = b_residualBill_Intercept,
      "Cold Rearing" = b_residualBill_pretreatmentA,
      "Warm Rearing" = b_residualBill_pretreatmentC,
      "Sigma" = sigma_residualBill
    ) %>%
    pivot_longer(everything(), names_to = "Par",
                 values_to = "Coefs") %>%
    ggplot(aes(x = Coefs)) +
    facet_wrap(~Par, scales = "free") +
    geom_density(colour = "black", alpha = 0.5, fill = "grey70") +
    geom_vline(xintercept = 0, linetype = "dashed", colour = "black") +
    ylab("Density") +
    theme_classic() +
    theme(axis.title.x = element_blank()) +
    ggtitle("Residual Bill Length (mm)"),
  ncol = 1
)
```

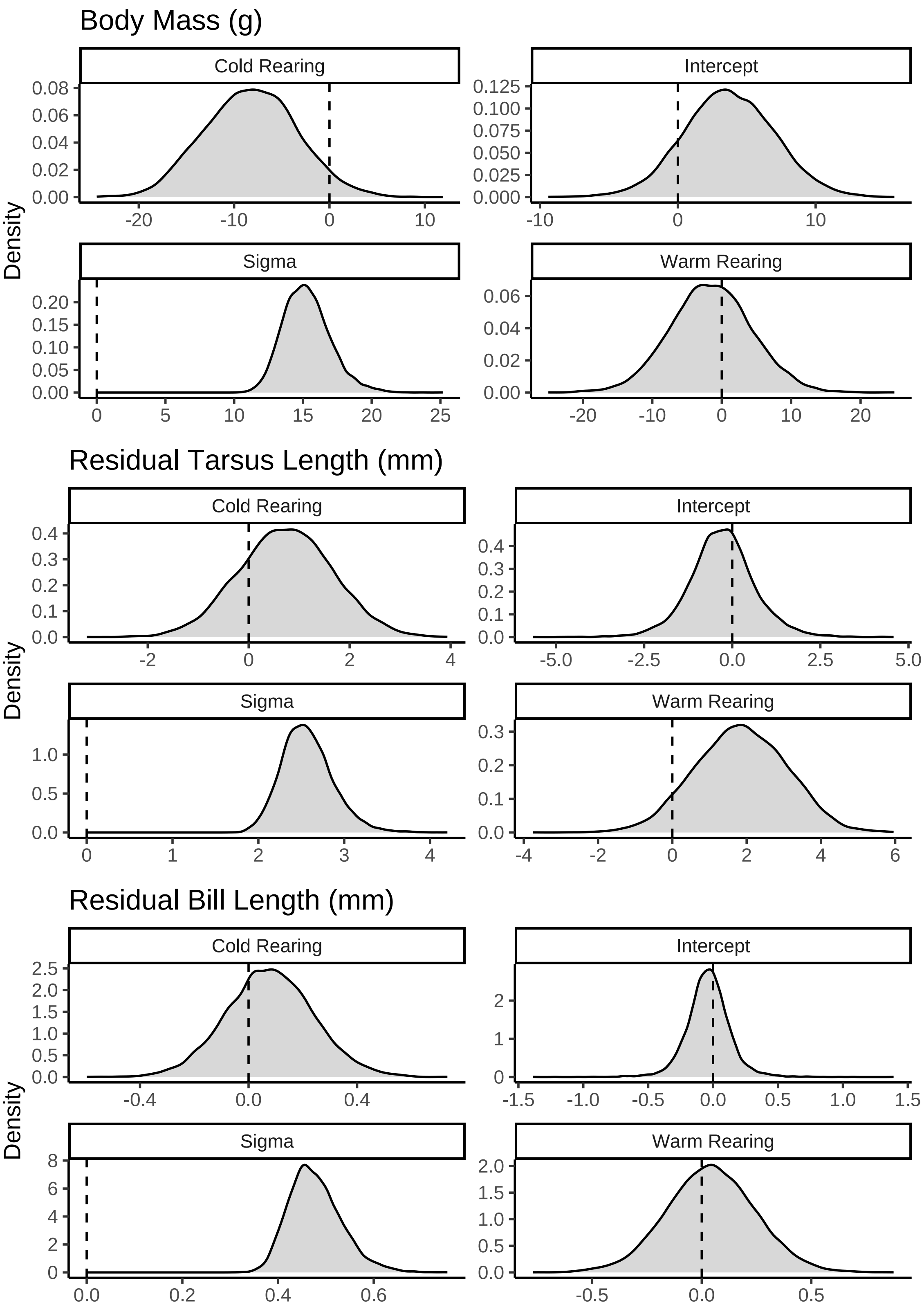

Figure 101: *Continued. Posterior densities for model coefficients derived from a Bayesian path analysis predicting metabolic slopes (fold metabolism at thermoneutrality/°C) of three week old Japanese quail in the cold (10°C - 30°C). Densities are split by their respective response values (indicated with titles). Vertical dashed lines indicate 0.*

Slight skewing in predictors is evident. As such, we proceed by using medians as our metric of central tendency for posterior summaries.

```
caption <- paste0(
  "Results of a Bayesian path analysis ",
  "predicting metabolic slopes (fold metabolism ",
  "at thermoneutrality) of three week old Japanese quail by ",
  "morphometry and thermal history. Tarsus length and bill ",
  "length are relativised by body mass (tarsus and bill ",
  "length residuals) as estimated from Bayesian ",
  "linear models. Metabolic slopes represent those in response ",
  "to cold (10°C - 30°C). Estimates are posterior medians, ",
  "credible intervals are quantile intervals, and BF indicates ",
  "Bayes Factors for each model parameter."
)

functionModel3WeeksResidualTable <-
  as.data.frame(functionModel3WeeksResidual) %>%
  summarise_all(., .funs = median) %>%
  pivot_longer(everything(),
    names_to = "Parameter",
    values_to = "Estimate"
  ) %>%
  merge(., quantileCIs(functionModel3WeeksResidual, cis = c(50, 95)),
    by = "Parameter", all.x = TRUE
  ) %>%
  filter(grepl("b_|sd_", Parameter)) %>%
  rowwise() %>%
  mutate("BF" = ifelse(Estimate < 0,
    (2 * mean(as.data.frame(
      functionModel3WeeksResidual
    )[, Parameter] <= 0)) /
      (2 * mean(as.data.frame(
        functionModel3WeeksResidual
      )[, Parameter] >= 0)),
    (2 * mean(as.data.frame(
      functionModel3WeeksResidual
    )[, Parameter] >= 0)) /
      (2 * mean(as.data.frame(
        functionModel3WeeksResidual
      )[, Parameter] <= 0))
  )) %>%
  ungroup() %>%
  mutate(
    "Estimate" = round(Estimate, digits = 4),
    "BF" = round(BF, digits = 4),
    "N" = nrow(functionModel3WeeksResidual$data)
  ) %>%
  mutate("Parameter" = ifelse(grepl("b_", Parameter),
    gsub("b_", "", Parameter),
    gsub(
      "Intercept", "batch",
      gsub(".*__", "", Parameter)
    )
  )) %>%
  mutate(
    "Response" = gsub("_.*", "", Parameter),
    "Parameter" = gsub(".*_", "", Parameter)
  ) %>%
  merge(., tribble(
    ~Response, ~response, ~level,
    "mass", "Body Mass (g)", "A",
    "rawSlope",
    "Metabolic Slope", "D",
    "residualTarsus", "Residual Tarsus Length (mm)", "B",
    "residualBill", "Residual Bill Length (mm)", "C"
  ),
```

```
  by = "Response"
  ) %>%
  merge(., tribble(
    ~Parameter, ~parameter, ~number,
    "Intercept", "Intercept", "1",
    "mass", "Body Mass (g)", "4",
    "residualTarsus", "Residual Tarsus Length (mm)", "5",
    "residualBill", "Residual Bill Length (mm)", "6",
    "pretreatmentA", "Cold Rearing", "2",
    "pretreatmentC", "Warm Rearing", "3",
    "batch", "Egg Batch [mu]", "7"
  ),
  by = "Parameter"
  ) %>%
  mutate(
    `50\\% CI` = paste0("(", paste(
      round(Low_CI_50, digits = 4),
      round(High_CI_50, digits = 4),
      sep = ", "
    ), ")"),
    `95\\% CI` = paste0("(", paste(
      round(Low_CI_95, digits = 4),
      round(High_CI_95, digits = 4),
      sep = ", "
    ), ")")
  ) %>%
  dplyr::select(-c(Low_CI_50, High_CI_50, Low_CI_95, High_CI_95)) %>%
  dplyr::select(
    "Response" = "response", "Parameter" = "parameter", N,
    Estimate, `50\\% CI`, `95\\% CI`, BF, level, number
  ) %>%
  arrange(level, number) %>%
  dplyr::select(-c(level, number)) %>%
  kbl(.,
    longtable = T, booktabs = T, format = "latex", escape = FALSE,
    caption = caption
  ) %>%
  column_spec(column = c(1:2), width = "2.2cm") %>%
  column_spec(column = c(3:10), width = "1.9cm") %>%
  kable_styling(latex_options = "striped")

functionModel3WeeksResidualTable
```

Table 50: *Results of a Bayesian path analysis predicting metabolic slopes (fold metabolism at thermoneutrality) of three week old Japanese quail by morphometry and thermal history. Tarsus length and bill length are relativised by body mass (tarsus and bill length residuals) as estimated from Bayesian linear models. Metabolic slopes represent those in response to cold (10°C - 30°C). Estimates are posterior medians, credible intervals are quantile intervals, and BF indicates Bayes Factors for each model parameter.*

Response	Parameter	N	Estimate	50% CI	95% CI	BF
Body Mass (g)	Intercept	42	3.6892	(1.4869, 5.9428)	(-2.7933, 10.2441)	6.8818
Body Mass (g)	Cold Rearing	42	-8.1935	(-11.5241, -4.9357)	(-17.3977, 1.4123)	21.5352
Body Mass (g)	Warm Rearing	42	-1.5614	(-5.4139, 2.3878)	(-12.9901, 10.0742)	1.5276
Body Mass (g)	Egg Batch [mu]	42	0.2741	(0.1118, 0.5492)	(0.0104, 1.5039)	Inf
Residual Tarsus Length (mm)	Intercept	42	-0.2943	(-0.8483, 0.2644)	(-2.1514, 1.6493)	1.7749
Residual Tarsus Length (mm)	Cold Rearing	42	0.7414	(0.1142, 1.3574)	(-1.1054, 2.5598)	3.6243
Residual Tarsus Length (mm)	Warm Rearing	42	1.8498	(1.0076, 2.7008)	(-0.5643, 4.2069)	13.6252

Residual Tarsus Length (mm)	Egg Batch [mu]	42	0.8587	(0.4582, 1.3239)	(0.0375, 2.4378)	Inf
Residual Bill Length (mm)	Intercept	42	-0.0378	(-0.1321, 0.0569)	(-0.3631, 0.2991)	1.5633
Residual Bill Length (mm)	Cold Rearing	42	0.0784	(-0.0279, 0.1858)	(-0.2363, 0.4038)	2.2747
Residual Bill Length (mm)	Warm Rearing	42	0.0416	(-0.0911, 0.1757)	(-0.3549, 0.4412)	1.3981
Residual Bill Length (mm)	Egg Batch [mu]	42	0.0912	(0.0392, 0.1827)	(0.0035, 0.5005)	Inf
Metabolic Slope	Intercept	42	-0.0005	(-0.0028, 0.0017)	(-0.0105, 0.0087)	1.2851
Metabolic Slope	Cold Rearing	42	0.0037	(0.0014, 0.006)	(-0.0032, 0.0104)	6.0922
Metabolic Slope	Warm Rearing	42	-0.0010	(-0.004, 0.002)	(-0.0099, 0.0079)	1.4024
Metabolic Slope	Body Mass (g)	42	0.0002	(1e-04, 2e-04)	(0, 4e-04)	22.0548
Metabolic Slope	Residual Tarsus Length (mm)	42	-0.0004	(-8e-04, 0)	(-0.0014, 7e-04)	3.1885
Metabolic Slope	Residual Bill Length (mm)	42	0.0005	(-2e-04, 0.0011)	(-0.0015, 0.0023)	2.1032
Metabolic Slope	Egg Batch [mu]	42	0.0039	(0.0016, 0.0081)	(2e-04, 0.0265)	Inf

```
#save_kable(resistanceResidual3WeeksTable,
# "../residualTarsusAnalysisResults3Weeks.html")
```

Modelling effects during adulthood

Here, we repeat the above described path analyses but while only using data obtained from mature Japanese quail (8 weeks of age). Priors used in our previous analyses are retained, except for a broadening of our body mass priors (intercept: $\mathcal{N}[0, 10]$; cold rearing: $\mathcal{N}[0, 25]$; warm rearing: $\mathcal{N}[0, 25]$). Again, priors are tested for suitability via a prior predictive check.

```
functionModel8WeeksPPCheck <-
  brm(
    data = slopeData %>%
      filter(week == "8") %>%
      mutate(
        mass = mass - mean(mass, na.rm = T),
        tarsus = tarsusLengthMean - mean(tarsusLengthMean, na.rm = T),
        bill = billLengthMean - mean(billLengthMean, na.rm = T),
        pretreatment = ifelse(pretreatment == "cold", "A",
          ifelse(pretreatment == "neutral", "B", "C")
        )
      ) %>%
      mutate(pretreatment = factor(pretreatment, levels = c("B", "A", "C"))) %>%
      drop_na() %>%
      merge(., data %>%
        dplyr::select(ring, "batch" = exp) %>%
        distinct(),
      by = "ring", all.x = TRUE
      ),
    family = "gaussian",
    bf(mass ~ pretreatment + (1 | batch)) +
      bf(tarsus ~ mass + pretreatment + (1 | batch)) +
      bf(bill ~ mass + pretreatment + (1 | batch)) +
      bf(rawSlope ~ mass + tarsus + bill + pretreatment + (1 | batch)) +
      set_rescor(FALSE),
    prior = c(
      set_prior("normal(0, 10)",
        class = "Intercept",
        resp = "mass"
```

```
    ),
    set_prior("normal(0, 25)",
      class = "b",
      coef = "pretreatmentA",
      resp = "mass"
    ),
    set_prior("normal(0, 25)",
      class = "b",
      coef = "pretreatmentC",
      resp = "mass"
    ),
    set_prior("exponential(2.5)",
      class = "sd",
      group = "batch",
      resp = "mass"
    ),
    set_prior("exponential(0.15)",
      class = "sigma",
      resp = "mass"
    ),
    set_prior("normal(0, 2.5)",
      class = "Intercept",
      resp = "tarsus"
    ),
    set_prior("normal(0, 2.5)",
      class = "b",
      coef = "pretreatmentA",
      resp = "tarsus"
    ),
    set_prior("normal(0, 2.5)",
      class = "b",
      coef = "pretreatmentC",
      resp = "tarsus"
    ),
    set_prior("skew_normal(0, 0.25, 5)",
      class = "b",
      coef = "mass",
      resp = "tarsus"
    ),
    set_prior("exponential(2)",
      class = "sd",
      group = "batch",
      resp = "tarsus"
    ),
    set_prior("exponential(1)",
      class = "sigma",
      resp = "tarsus"
    ),
    set_prior("normal(0, 1)",
      class = "Intercept",
      resp = "bill"
    ),
    set_prior("normal(0, 0.5)",
      class = "b",
      coef = "pretreatmentA",
      resp = "bill"
    ),
    set_prior("normal(0, 0.5)",
      class = "b",
      coef = "pretreatmentC",
      resp = "bill"
    ),
    set_prior("skew_normal(0, 0.25, 5)",
      class = "b",
      coef = "mass",
      resp = "bill"
    ),
```

```
      set_prior("exponential(5)",
        class = "sd",
        group = "batch",
        resp = "bill"
      ),
      set_prior("exponential(2.5)",
        class = "sigma",
        resp = "bill"
      ),
      set_prior("normal(0, 0.01)",
        class = "Intercept",
        resp = "rawSlope"
      ),
      set_prior("normal(0, 0.025)",
        class = "b",
        coef = "pretreatmentA",
        resp = "rawSlope"
      ),
      set_prior("normal(0, 0.025)",
        class = "b",
        coef = "pretreatmentC",
        resp = "rawSlope"
      ),
      set_prior("normal(0, 0.001)",
        class = "b",
        coef = "mass",
        resp = "rawSlope"
      ),
      set_prior("normal(0, 0.0025)",
        class = "b",
        coef = "tarsus",
        resp = "rawSlope"
      ),
      set_prior("normal(0, 0.001)",
        class = "b",
        coef = "bill",
        resp = "rawSlope"
      ),
      set_prior("exponential(50)",
        class = "sd",
        group = "batch",
        resp = "rawSlope"
      ),
      set_prior("exponential(10)",
        class = "sigma",
        resp = "rawSlope"
      )
    ),
    iter = 50000, warmup = 10000, cores = 4, chains = 4, thin = 20,
    control = list(adapt_delta = .97, max_treedepth = 14),
    silent = TRUE, refresh = 0,
    sample_prior = "only",
    file = "./models/_eightWeekFunctionModelPPCheck.Rds"
  )

# Plotting

p1 <- pp_check2(functionModel8WeeksPPCheck, resp = "mass",
            xlab = "Body Mass (g; Mean-Centred)")
p2 <- pp_check2(functionModel8WeeksPPCheck, resp = "tarsus",
            xlab = "Tarsus Length (mm; Mean-Centred)")
p3 <- pp_check2(functionModel8WeeksPPCheck, resp = "bill",
            xlab = "Bill Length (mm; Mean-Centred)")
p4 <- pp_check2(functionModel8WeeksPPCheck,
    resp = "rawSlope",
    xlab =
    "Metabolic Slope (fold RMR at\nThermoneutrality/°C)"
```

```
  )

(p1/p2) | (p3/p4)
```

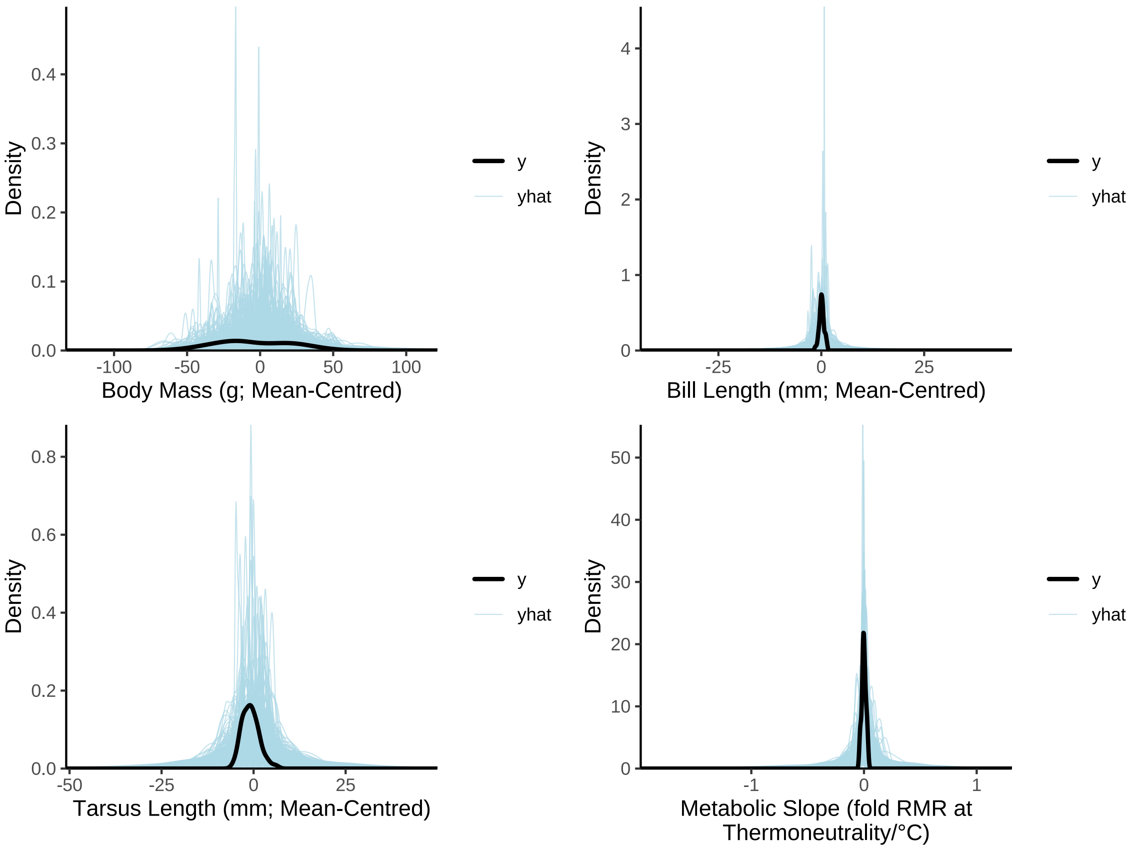

Figure 102: *Prior predictive check for a Bayesian path analysis predicting metabolic slopes (fold metabolism at thermoneutrality/°C) as a direct and indirect function of body mass (g), tarsus length (mm) and bill length (mm) in eight week old Japanese quail. Metabolic slopes pertain to those estimated in response to cold (10°C - 30°C). Light blue lines represent densities of resting metabolism values as predicted by model priors alone. The dark blue line represent the true density of resting metabolism values. Clear overlap between the dark blue and light blue lines indicates that priors are suitable.*

```
# Proceeding.
```

```
functionModel8Weeks <-
  brm(
    data = slopeData %>%
      filter(week == "8") %>%
      mutate(
        mass = mass - mean(mass, na.rm = T),
        tarsus = tarsusLengthMean - mean(tarsusLengthMean, na.rm = T),
        bill = billLengthMean - mean(billLengthMean, na.rm = T),
        pretreatment = ifelse(pretreatment == "cold", "A",
          ifelse(pretreatment == "neutral", "B", "C")
        )
      ) %>%
      mutate(pretreatment = factor(pretreatment, levels = c("B", "A", "C"))) %>%
      drop_na() %>%
      merge(., data %>%
        dplyr::select(ring, "batch" = exp) %>%
        distinct(),
      by = "ring", all.x = TRUE
```

```
    ),
    family = "gaussian",
    bf(mass ~ pretreatment + (1 | batch)) +
      bf(tarsus ~ mass + pretreatment + (1 | batch)) +
      bf(bill ~ mass + pretreatment + (1 | batch)) +
      bf(rawSlope ~ mass + tarsus + bill + pretreatment + (1 | batch)) +
      set_rescor(FALSE),
    prior = c(
      set_prior("normal(0, 10)",
        class = "Intercept",
        resp = "mass"
      ),
      set_prior("normal(0, 25)",
        class = "b",
        coef = "pretreatmentA",
        resp = "mass"
      ),
      set_prior("normal(0, 25)",
        class = "b",
        coef = "pretreatmentC",
        resp = "mass"
      ),
      set_prior("exponential(2.5)",
        class = "sd",
        group = "batch",
        resp = "mass"
      ),
      set_prior("exponential(0.15)",
        class = "sigma",
        resp = "mass"
      ),
      set_prior("normal(0, 2.5)",
        class = "Intercept",
        resp = "tarsus"
      ),
      set_prior("normal(0, 2.5)",
        class = "b",
        coef = "pretreatmentA",
        resp = "tarsus"
      ),
      set_prior("normal(0, 2.5)",
        class = "b",
        coef = "pretreatmentC",
        resp = "tarsus"
      ),
      set_prior("skew_normal(0, 0.25, 5)",
        class = "b",
        coef = "mass",
        resp = "tarsus"
      ),
      set_prior("exponential(2)",
        class = "sd",
        group = "batch",
        resp = "tarsus"
      ),
      set_prior("exponential(1)",
        class = "sigma",
        resp = "tarsus"
      ),
      set_prior("normal(0, 1)",
        class = "Intercept",
        resp = "bill"
      ),
      set_prior("normal(0, 0.5)",
        class = "b",
        coef = "pretreatmentA",
        resp = "bill"
```

```
      ),
      set_prior("normal(0, 0.5)",
        class = "b",
        coef = "pretreatmentC",
        resp = "bill"
      ),
      set_prior("skew_normal(0, 0.25, 5)",
        class = "b",
        coef = "mass",
        resp = "bill"
      ),
      set_prior("exponential(5)",
        class = "sd",
        group = "batch",
        resp = "bill"
      ),
      set_prior("exponential(2.5)",
        class = "sigma",
        resp = "bill"
      ),
      set_prior("normal(0, 0.01)",
        class = "Intercept",
        resp = "rawSlope"
      ),
      set_prior("normal(0, 0.025)",
        class = "b",
        coef = "pretreatmentA",
        resp = "rawSlope"
      ),
      set_prior("normal(0, 0.025)",
        class = "b",
        coef = "pretreatmentC",
        resp = "rawSlope"
      ),
      set_prior("normal(0, 0.001)",
        class = "b",
        coef = "mass",
        resp = "rawSlope"
      ),
      set_prior("normal(0, 0.0025)",
        class = "b",
        coef = "tarsus",
        resp = "rawSlope"
      ),
      set_prior("normal(0, 0.001)",
        class = "b",
        coef = "bill",
        resp = "rawSlope"
      ),
      set_prior("exponential(50)",
        class = "sd",
        group = "batch",
        resp = "rawSlope"
      ),
      set_prior("exponential(10)",
        class = "sigma",
        resp = "rawSlope"
      )
    ),
    iter = 50000, warmup = 10000, cores = 4, chains = 4, thin = 20,
    control = list(adapt_delta = .97, max_treedepth = 14),
    silent = TRUE, refresh = 0,
    file = "./models/_eightWeekFunctionModel.Rds"
  )

# Negligible divergences. Checking Rhat values, Neff/N ratios,
# and posterior distribution
```

```
chainCheck(functionModel8Weeks)

## Rhat range: 1 - 1.001
## Neff/N range: 0.793 - 1.018
```

```
pp_check2(
  functionModel8Weeks, resp = "rawSlope",
  xlab = "Metabolic Slope (fold RMR at Thermoneutrality/°C)"
)
```

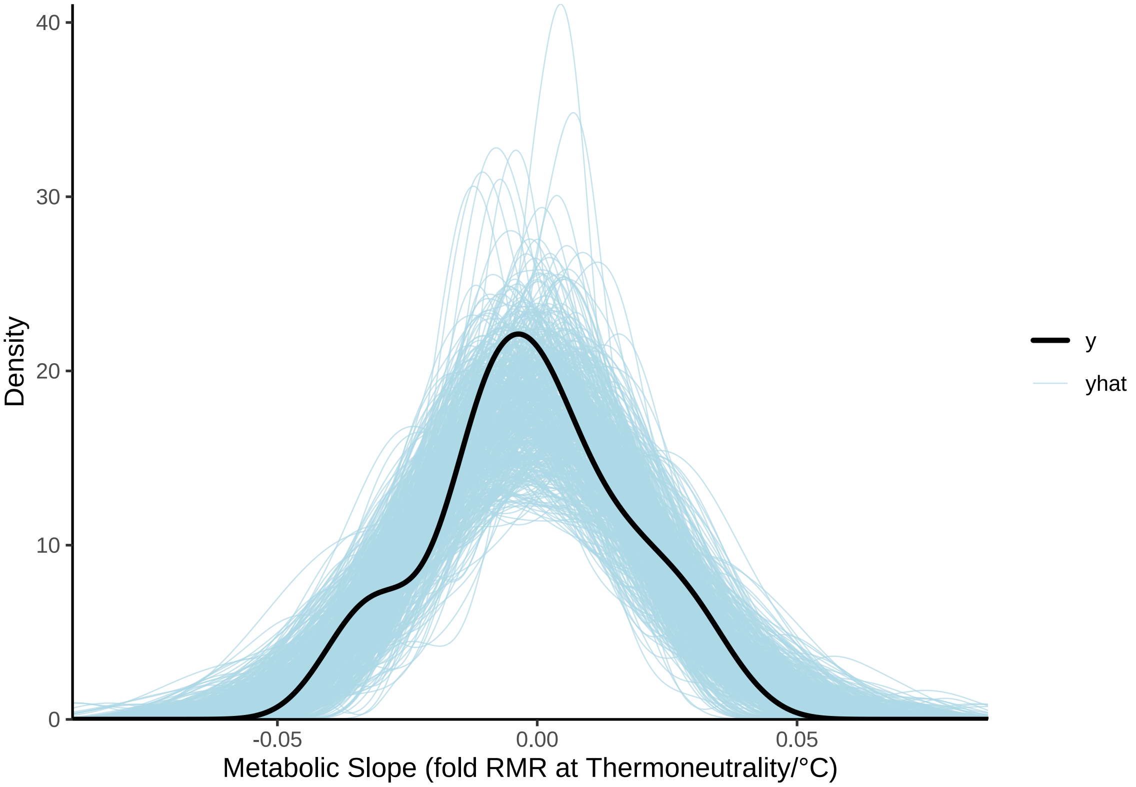

Figure 103: *Posterior predictive check for a Bayesian path analysis predicting metabolic slopes of eight week old Japanese quail, in response to cold (10°C - 30°C), by mean-centred morphometry (body mass, tarsus length, and bill length). Light blue lines represent densities of resting metabolism values as drawn from model posteriors. The dark blue line represent the true density of resting metabolism values. Clear overlap between the dark blue and light blue lines indicates strong model fit.*

Bayesian R^2 values for each element of our path analysis are next estimated.

```
caption <- paste0(
  "Estimates of fit for each element of a Bayesian path ",
  "analysis predicting metabolic slopes (fold metabolism at ",
  "thermoneutrality/°C) of mature Japanese quail by morphometry ",
  "and thermal history. Metabolic slopes are measured in ",
  "response to cold (10°C - 30°C). Credible intervals are ",
  "quantile intervals. "
)

brms::bayes_R2(functionModel8Weeks,
  ndraws = 1000,
  robust = TRUE
) %>%
  as.data.frame() %>%
```

```
  rownames_to_column("var") %>%
  merge(., tribble(
    ~var, ~Var,
    "R2mass", "Body Mass (g)",
    "R2tarsus", "Tarsus Length (mm)",
    "R2bill", "Bill Length (mm)",
    "R2rawSlope",
    "Metabolic Slope"
  ), by = c("var")) %>%
  mutate(
    Estimate = round(Estimate, digits = 4),
    Est.Error = round(Est.Error, digits = 4),
    "95\\% CI" = paste0(
      "[", round(Q2.5, digits = 4),
      ", ", round(Q97.5, digits = 4),
      "]"
    ),
    Var = factor(Var, levels = c("Body Mass (g)",
                                 "Tarsus Length (mm)",
                                 "Bill Length (mm)",
                                 "Metabolic Slope")
                 )
  ) %>%
  dplyr::select(
    "Response" = Var, "R\\textsuperscript{2}" = Estimate,
    "Standard Error" = Est.Error,
    `95\\% CI`
  ) %>%
  arrange(Response) %>%
  kbl(.,
    longtable = T, booktabs = T, format = "latex",
    caption = caption, escape = FALSE
  ) %>%
  column_spec(column = 1, width = "3cm") %>%
  column_spec(column = c(2:10), width = "2.5cm") %>%
  kable_styling(latex_options = "striped")
```

Table 51: *Estimates of fit for each element of a Bayesian path analysis predicting metabolic slopes (fold metabolism at thermoneutrality/°C) of mature Japanese quail by morphometry and thermal history. Metabolic slopes are measured in response to cold (10°C - 30°C). Credible intervals are quantile intervals.*

Response	R^2	Standard Error	95% CI
Body Mass (g)	0.0243	0.0245	[7e-04, 0.1113]
Tarsus Length (mm)	0.3199	0.0816	[0.1338, 0.4723]
Bill Length (mm)	0.0584	0.0416	[0.0075, 0.1692]
Metabolic Slope	0.1031	0.0625	[0.0241, 0.2389]

Residuals and posterior densities are again visualised as before.

```
p1 <- functionModel8Weeks$data %>%
    mutate("Residuals" =
             residuals(functionModel8Weeks,
                       resp = "rawSlope",
                       robust = TRUE)[, "Estimate"]) %>%
    ggplot(aes(x = mass, y = Residuals)) +
    geom_point(size = 2, pch = 21, colour = "black", fill = "grey50") +
    xlab("Relative Body Mass (g)") +
    ylab("Ordinary Residuals") +
    theme_classic()

p2 <- functionModel8Weeks$data %>%
    mutate("Residuals" =
             residuals(functionModel8Weeks,
```

```
                         resp = "rawSlope",
                         robust = TRUE)[, "Estimate"]) %>%
    ggplot(aes(x = tarsus, y = Residuals)) +
    geom_point(size = 2, pch = 21, colour = "black", fill = "grey50") +
    xlab("Relative Tarsus\nLength (mm)") +
    ylab("Ordinary Residuals") +
    theme_classic()

p3 <- functionModel8Weeks$data %>%
    mutate("Residuals" =
             residuals(functionModel8Weeks,
                       resp = "rawSlope",
                       robust = TRUE)[, "Estimate"]) %>%
    ggplot(aes(x = bill, y = Residuals)) +
    geom_point(size = 2, pch = 21, colour = "black", fill = "grey50") +
    xlab("Relative Bill\nLength (mm)") +
    ylab("Ordinary Residuals") +
    theme_classic()

p4 <- functionModel8Weeks$data %>%
    mutate("Residuals" =
             residuals(functionModel8Weeks,
                       resp = "rawSlope",
                       robust = TRUE)[, "Estimate"]) %>%
    ggplot(aes(x = factor(pretreatment), y = Residuals)) +
    geom_boxplot(width = 0.5) +
    geom_point(size = 2, pch = 21,
               position = position_jitter(width = 0.25),
               colour = "black", fill = "grey50") +
    xlab("Rearing Conditions") +
    ylab("Ordinary Residuals") +
    theme_classic()

p5 <- functionModel8Weeks$data %>%
    mutate("Residuals" =
             residuals(functionModel8Weeks,
                       resp = "rawSlope",
                       robust = TRUE)[, "Estimate"]) %>%
    ggplot(aes(sample = Residuals)) +
    stat_qq(colour = "grey50") +
    stat_qq_line() +
    xlab("Theoretical") +
    ylab("Sample") +
    theme_classic()

p6 <- functionModel8Weeks$data %>%
    mutate(
      "Residuals" =
        residuals(functionModel8Weeks,
                  resp = "rawSlope",
                       robust = TRUE)[, "Estimate"],
      "Fitted" =
        fitted(functionModel8Weeks,
               resp = "rawSlope",
                       robust = TRUE)[, "Estimate"]
    ) %>%
    ggplot(aes(x = Fitted, y = Residuals)) +
    geom_point(size = 2, pch = 21, colour = "black", fill = "grey50") +
    xlab("Fitted Values") +
    ylab("Ordinary Residuals") +
    theme_classic()

(p1 + p2 + p3)/(p4 + p5 + p6)
```

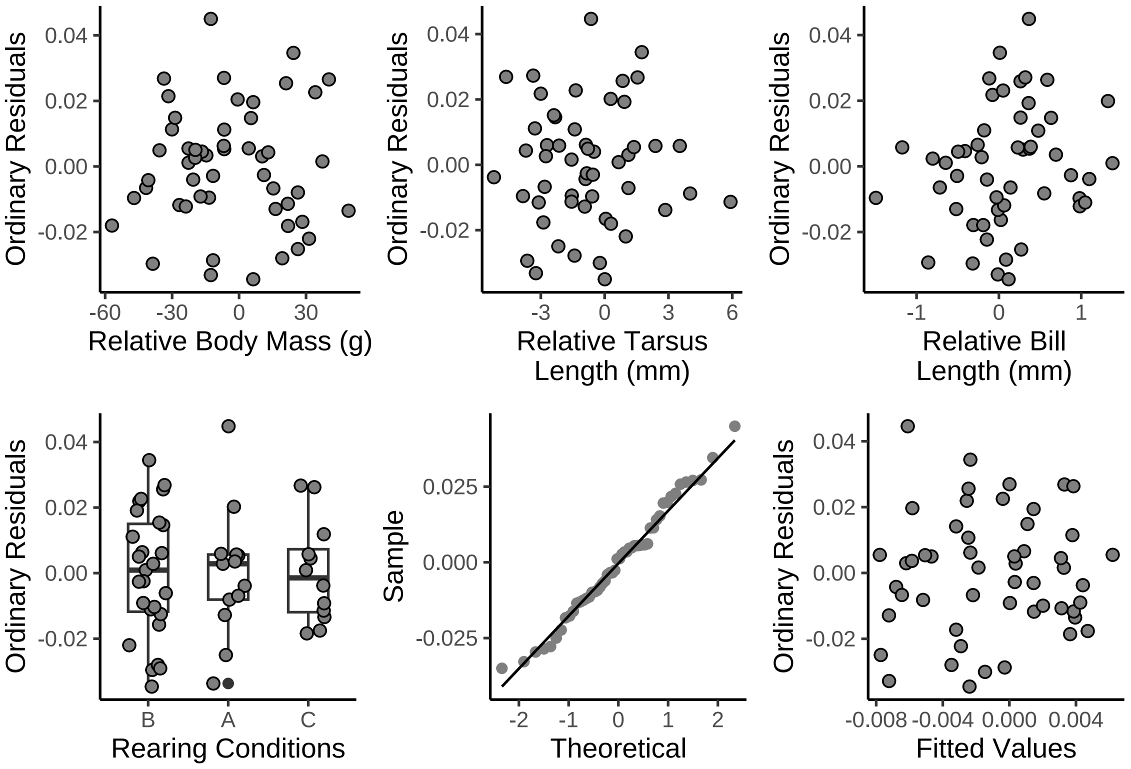

Figure 104: *Ordinary residuals from a Bayesian path analysis predicting metabolic slopes (fold metabolism at thermoneutrality/°C) of mature Japanese quail as a function of body mass, tarsus length, bill length, and thermal history (here, rearing temperature). Metabolic slopes are measures in response to cold (10°C - 30°C). Residuals are displayed against model predictors, as a function of theoretical quantiles, and against model fitted values.*

```
# Evenly dispersed. Plotting model coefficients
```

```
ggarrange(
  as.data.frame(functionModel8Weeks) %>%
    dplyr::select(
      "Body Mass (g)" = b_rawSlope_mass,
      "Tarsus Length\n(mm)" = b_rawSlope_tarsus,
      "Bill Length\n(mm)" = b_rawSlope_bill,
      "Cold Rearing\n(10°C)" = b_rawSlope_pretreatmentA,
      "Warm Rearing\n(10°C)" = b_rawSlope_pretreatmentC
    ) %>%
    pivot_longer(everything(), names_to = "Par",
                 values_to = "Coefs") %>%
    ggplot(aes(x = Coefs)) +
    facet_wrap(~Par, scales = "free", ncol = 2) +
    geom_density(colour = "black", alpha = 0.5, fill = "grey70") +
    geom_vline(xintercept = 0, linetype = "dashed",
               colour = "black") +
    ylab("Density") +
    scale_x_continuous(n.breaks = 3) +
    theme_classic() +
    theme(axis.title.x = element_blank()) +
    ggtitle("Metabolic Slope"),

  as.data.frame(functionModel8Weeks) %>%
    dplyr::select(
      "Intercept" = b_mass_Intercept,
      "Cold Rearing\n(10°C)" = b_mass_pretreatmentA,
      "Warm Rearing\n(30°C)" = b_mass_pretreatmentC,
```

```
      "Sigma" = sigma_mass
    ) %>%
    pivot_longer(everything(), names_to = "Par",
                 values_to = "Coefs") %>%
    ggplot(aes(x = Coefs)) +
    facet_wrap(~Par, scales = "free") +
    geom_density(colour = "black",
                 alpha = 0.5, fill = "grey70") +
    geom_vline(xintercept = 0, linetype = "dashed",
               colour = "black") +
    ylab("Density") +
    theme_classic() +
    theme(axis.title.x = element_blank()) +
    ggtitle("Body Mass (g)")
)
```

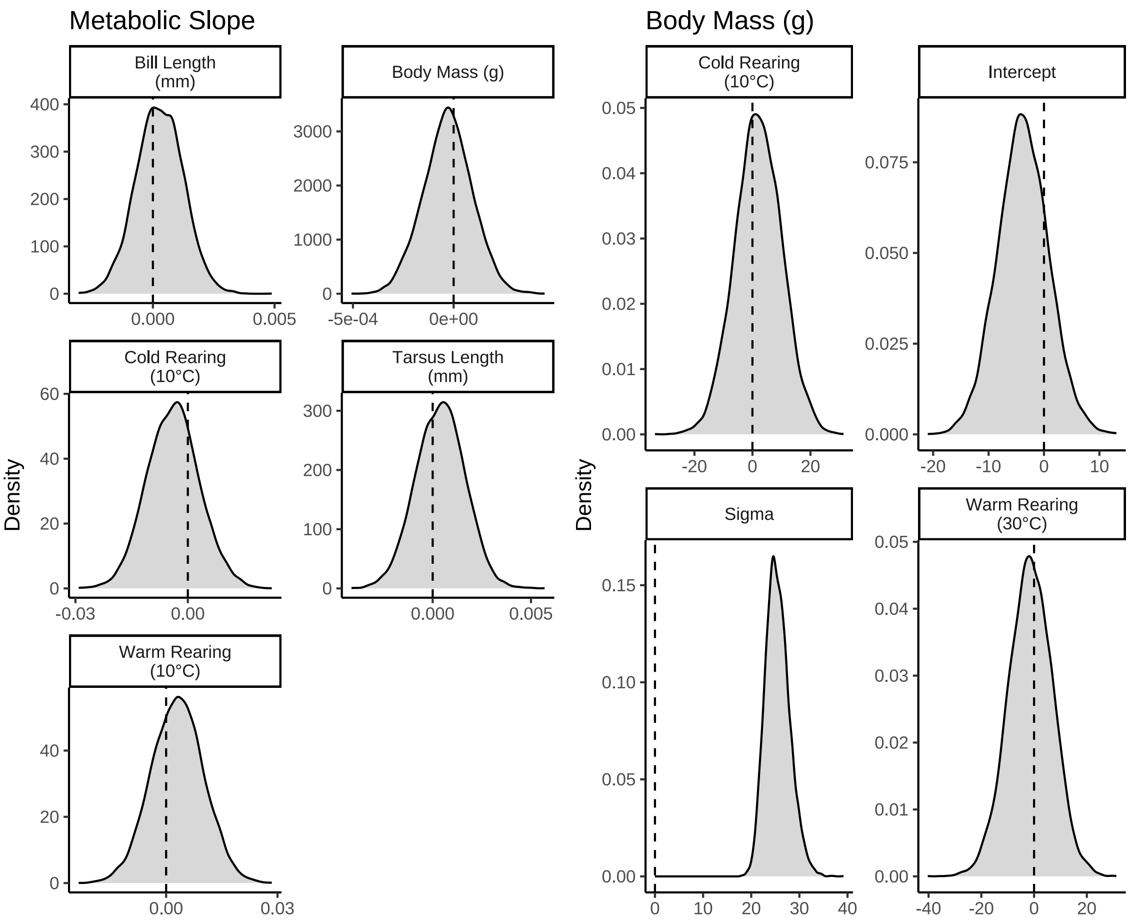

Figure 105: *Coefficient densities (posteriors) from a Bayesian path analysis predicting metabolic slopes (fold metabolism at thermoneutrality/°C) as a function of body mass, tarsus length, and bill length in eight week old Japanese quail.*

```
ggarrange(
  as.data.frame(functionModel8Weeks) %>%
    dplyr::select(
      "Intercept" = b_tarsus_Intercept,
      "Cold Rearing\n(10°C)" = b_tarsus_pretreatmentA,
      "Warm Rearing\n(30°C)" = b_tarsus_pretreatmentC,
      "Sigma" = sigma_tarsus
    ) %>%
    pivot_longer(everything(), names_to = "Par",
```

```
                 values_to = "Coefs") %>%
    ggplot(aes(x = Coefs)) +
    facet_wrap(~Par, scales = "free") +
    geom_density(colour = "black",
                 alpha = 0.5, fill = "grey70") +
    geom_vline(xintercept = 0, linetype = "dashed",
               colour = "black") +
    ylab("Density") +
    theme_classic() +
    theme(axis.title.x = element_blank()) +
    ggtitle("Tarsus Length (mm)"),

  as.data.frame(functionModel8Weeks) %>%
    dplyr::select(
      "Intercept" = b_bill_Intercept,
      "Cold Rearing\n(10°C)" = b_bill_pretreatmentA,
      "Warm Rearing\n(30°C)" = b_bill_pretreatmentC,
      "Sigma" = sigma_bill
    ) %>%
    pivot_longer(everything(), names_to = "Par",
                 values_to = "Coefs") %>%
    ggplot(aes(x = Coefs)) +
    facet_wrap(~Par, scales = "free") +
    geom_density(colour = "black",
                 alpha = 0.5, fill = "grey70") +
    geom_vline(xintercept = 0, linetype = "dashed",
               colour = "black") +
    ylab("Density") +
    theme_classic() +
    theme(axis.title.x = element_blank()) +
    ggtitle("Bill Length (mm)")
)
```

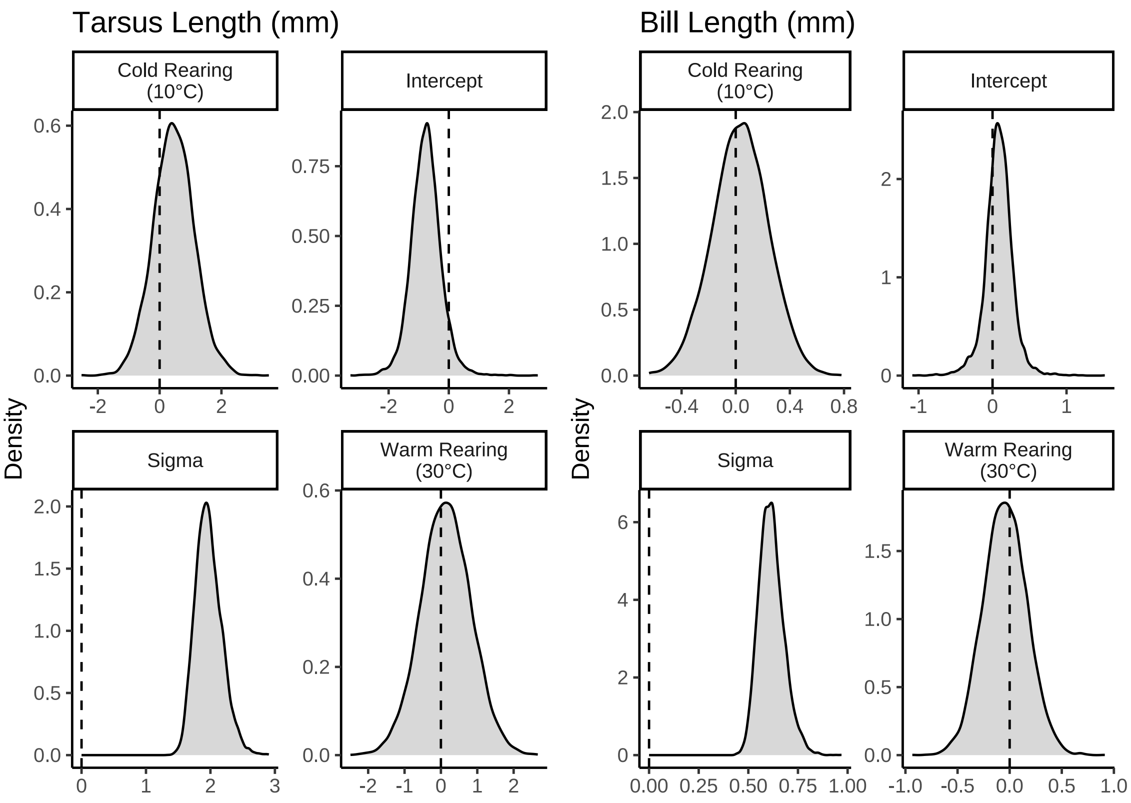

Figure 106: *Coefficient densities (posteriors) from a Bayesian path analysis predicting metabolic slopes (fold metabolism at thermoneutrality/°C) as a function of body mass, tarsus length and bill length in eight week old Japanese quail. Continued from previous figure.*

```
# Very little evidence of biological effects. Summarising results

caption <- paste0(
  "Results from a Bayesian path analysis ",
  "predicting metabolic slope (fold  ",
  "metabolism at thermoneutrality/{\\circ}C) as a function of ",
  "morphometry and rearing temperature in ",
  "eight week old Japanese quail. Metabolic slopes are ",
  "measured in response to cold (10°C - 30°C). Cold rearing ",
  "indicates post-hatch rearing at ",
  "10°C, relative to 20°C (intercept), or ",
  "30°C ('warm rearing). Estimates indicate medians, ",
  "CIs indicate quantile intervals, ",
  "and BF indicates Bayes Factors."
)

functionModel8WeeksTable <-
  as.data.frame(functionModel8Weeks) %>%
  summarise_all(., .funs = median) %>%
  pivot_longer(everything(),
    names_to = "Parameter",
    values_to = "Estimate"
  ) %>%
  merge(., quantileCIs(functionModel8Weeks, cis = c(50, 95)),
    by = "Parameter", all.x = TRUE
  ) %>%
  filter(grepl("b_|sd_", Parameter)) %>%
  rowwise() %>%
  mutate("BF" = ifelse(Estimate < 0,
    (2 * mean(as.data.frame(
```

```
    functionModel8Weeks
  )[, Parameter] <= 0)) /
    (2 * mean(as.data.frame(
      functionModel8Weeks
    )[, Parameter] >= 0)),
  (2 * mean(as.data.frame(
    functionModel8Weeks
  )[, Parameter] >= 0)) /
    (2 * mean(as.data.frame(
      functionModel8Weeks
    )[, Parameter] <= 0))
)) %>%
ungroup() %>%
mutate(
  "Estimate" = round(Estimate, digits = 4),
  "BF" = round(BF, digits = 4),
  "N" = nrow(functionModel8Weeks$data)
) %>%
mutate("Parameter" = ifelse(grepl("b_", Parameter),
  gsub("b_", "", Parameter),
  gsub(
    "Intercept", "batch",
    gsub(".*__", "", Parameter)
  )
)) %>%
mutate(
  "Response" = gsub("_.*", "", Parameter),
  "Parameter" = gsub(".*_", "", Parameter)
) %>%
merge(., tribble(
  ~Response, ~response, ~level,
  "mass", "Body Mass (g)", "A",
  "rawSlope",
  "Metabolic Slope", "D",
  "tarsus", "Tarsus Length (mm)", "B",
  "bill", "Bill Length (mm)", "C"
),
by = "Response"
) %>%
merge(., tribble(
  ~Parameter, ~parameter, ~number,
  "Intercept", "Intercept", "1",
  "mass", "Body Mass (g)", "4",
  "tarsus", "Tarsus Length (mm)", "5",
  "bill", "Bill Length (mm)", "6",
  "pretreatmentA", "Cold Rearing", "2",
  "pretreatmentC", "Warm Rearing", "3",
  "batch", "Egg Batch [mu]", "7"
),
by = "Parameter"
) %>%
mutate(
  `50\\% CI` = paste0("(", paste(
    round(Low_CI_50, digits = 4),
    round(High_CI_50, digits = 4),
    sep = ", "
  ), ")"),
  `95\\% CI` = paste0("(", paste(
    round(Low_CI_95, digits = 4),
    round(High_CI_95, digits = 4),
    sep = ", "
  ), ")")
) %>%
dplyr::select(-c(Low_CI_50, High_CI_50, Low_CI_95, High_CI_95)) %>%
dplyr::select(
  "Response" = "response", "Parameter" = "parameter", N,
  Estimate, `50\\% CI`, `95\\% CI`, BF, level, number
```

```
  ) %>%
  arrange(level, number) %>%
  dplyr::select(-c(level, number)) %>%
  kbl(.,
    longtable = T, booktabs = T, format = "latex", escape = FALSE,
    caption = caption
  ) %>%
  column_spec(column = 1, width = "2.2cm") %>%
  column_spec(column = 2, width = "2cm") %>%
  column_spec(column = c(3:10), width = "1.9cm") %>%
  kable_styling(latex_options = "striped")

functionModel8WeeksTable
```

Table 52: *Results from a Bayesian path analysis predicting metabolic slope (fold metabolism at thermoneutrality/ C) as a function of morphometry and rearing temperature in eight week old Japanese quail. Metabolic slopes are measured in response to cold (10°C - 30°C). Cold rearing indicates post-hatch rearing at 10°C, relative to 20°C (intercept), or 30°C ('warm rearing). Estimates indicate medians, CIs indicate quantile intervals, and BF indicates Bayes Factors.*

Response	Parameter	N	Estimate	50% CI	95% CI	BF
Body Mass (g)	Intercept	52	-3.8048	(-6.82, -0.6951)	(-12.861, 5.4242)	3.8751
Body Mass (g)	Cold Rearing	52	2.0049	(-3.2985, 7.5949)	(-13.685, 18.0906)	1.4984
Body Mass (g)	Warm Rearing	52	-1.3041	(-6.9568, 4.3378)	(-18.0757, 14.8938)	1.3075
Body Mass (g)	Egg Batch [mu]	52	0.2750	(0.1184, 0.5462)	(0.0091, 1.4585)	Inf
Tarsus Length (mm)	Intercept	52	-0.7596	(-1.0692, -0.4611)	(-1.7373, 0.2638)	15.6667
Tarsus Length (mm)	Cold Rearing	52	0.4513	(0.0157, 0.8948)	(-0.846, 1.8016)	3.1216
Tarsus Length (mm)	Warm Rearing	52	0.1335	(-0.3225, 0.6021)	(-1.2264, 1.5043)	1.3606
Tarsus Length (mm)	Body Mass (g)	52	0.0491	(0.0419, 0.0562)	(0.0276, 0.0706)	Inf
Tarsus Length (mm)	Egg Batch [mu]	52	0.2490	(0.1052, 0.4895)	(0.0088, 1.3823)	Inf
Bill Length (mm)	Intercept	52	0.0853	(-0.018, 0.1902)	(-0.3157, 0.4501)	2.4965
Bill Length (mm)	Cold Rearing	52	0.0361	(-0.1026, 0.1739)	(-0.3786, 0.4374)	1.3168
Bill Length (mm)	Warm Rearing	52	-0.0449	(-0.184, 0.0946)	(-0.4454, 0.3705)	1.4075
Bill Length (mm)	Body Mass (g)	52	-0.0004	(-0.0027, 0.0018)	(-0.0073, 0.006)	1.2516
Bill Length (mm)	Egg Batch [mu]	52	0.1145	(0.0498, 0.2197)	(0.0049, 0.5585)	Inf
Metabolic Slope	Intercept	52	-0.0004	(-0.0039, 0.0031)	(-0.0119, 0.012)	1.1237
Metabolic Slope	Cold Rearing	52	-0.0038	(-0.0086, 6e-04)	(-0.0174, 0.0101)	2.5242
Metabolic Slope	Warm Rearing	52	0.0031	(-0.0018, 0.0078)	(-0.0108, 0.0169)	2.0120
Metabolic Slope	Body Mass (g)	52	0.0000	(-1e-04, 1e-04)	(-3e-04, 2e-04)	1.4806
Metabolic Slope	Tarsus Length (mm)	52	0.0004	(-5e-04, 0.0012)	(-0.002, 0.0028)	1.6560
Metabolic Slope	Bill Length (mm)	52	0.0003	(-4e-04, 9e-04)	(-0.0016, 0.0021)	1.5478
Metabolic Slope	Egg Batch [mu]	52	0.0055	(0.0024, 0.0106)	(3e-04, 0.0307)	Inf

```
#save_kable(resistance8WeeksTable,
# "../tables/pathAnalysisResults8Weeks.html")

# And calculating partial R2 of morphometry on metabolic slopes

{
  functionModel8WeeksMassR2 <-
    brm(
      data = slopeData %>%
        filter(week == "8") %>%
        mutate(
          mass = mass - mean(mass, na.rm = T),
          tarsus = tarsusLengthMean - mean(tarsusLengthMean, na.rm = T),
          bill = billLengthMean - mean(billLengthMean, na.rm = T),
          pretreatment = ifelse(pretreatment == "cold", "A",
            ifelse(pretreatment == "neutral", "B", "C")
          )
        ) %>%
        mutate(pretreatment = factor(pretreatment, levels = c("B", "A", "C"))) %>%
        drop_na() %>%
        merge(., data %>%
          dplyr::select(ring, "batch" = exp) %>%
          distinct(),
        by = "ring", all.x = TRUE
        ),
      family = "gaussian",
      bf(mass ~ pretreatment + (1 | batch)) +
        bf(tarsus ~ mass + pretreatment + (1 | batch)) +
        bf(bill ~ mass + pretreatment + (1 | batch)) +
        bf(rawSlope ~ tarsus + bill + pretreatment + (1 | batch)) +
        set_rescor(FALSE),
      prior = c(
        set_prior("normal(0, 10)",
          class = "Intercept",
          resp = "mass"
        ),
        set_prior("normal(0, 25)",
          class = "b",
          coef = "pretreatmentA",
          resp = "mass"
        ),
        set_prior("normal(0, 25)",
          class = "b",
          coef = "pretreatmentC",
          resp = "mass"
        ),
        set_prior("exponential(2.5)",
          class = "sd",
          group = "batch",
          resp = "mass"
        ),
        set_prior("exponential(0.15)",
          class = "sigma",
          resp = "mass"
        ),
        set_prior("normal(0, 2.5)",
          class = "Intercept",
          resp = "tarsus"
        ),
        set_prior("normal(0, 2.5)",
          class = "b",
          coef = "pretreatmentA",
          resp = "tarsus"
        ),
        set_prior("normal(0, 2.5)",
          class = "b",
          coef = "pretreatmentC",
```

```
    resp = "tarsus"
  ),
  set_prior("skew_normal(0, 0.25, 5)",
    class = "b",
    coef = "mass",
    resp = "tarsus"
  ),
  set_prior("exponential(2)",
    class = "sd",
    group = "batch",
    resp = "tarsus"
  ),
  set_prior("exponential(1)",
    class = "sigma",
    resp = "tarsus"
  ),
  set_prior("normal(0, 1)",
    class = "Intercept",
    resp = "bill"
  ),
  set_prior("normal(0, 0.5)",
    class = "b",
    coef = "pretreatmentA",
    resp = "bill"
  ),
  set_prior("normal(0, 0.5)",
    class = "b",
    coef = "pretreatmentC",
    resp = "bill"
  ),
  set_prior("skew_normal(0, 0.25, 5)",
    class = "b",
    coef = "mass",
    resp = "bill"
  ),
  set_prior("exponential(5)",
    class = "sd",
    group = "batch",
    resp = "bill"
  ),
  set_prior("exponential(2.5)",
    class = "sigma",
    resp = "bill"
  ),
  set_prior("normal(0, 0.01)",
    class = "Intercept",
    resp = "rawSlope"
  ),
  set_prior("normal(0, 0.025)",
    class = "b",
    coef = "pretreatmentA",
    resp = "rawSlope"
  ),
  set_prior("normal(0, 0.025)",
    class = "b",
    coef = "pretreatmentC",
    resp = "rawSlope"
  ),
  set_prior("normal(0, 0.0025)",
    class = "b",
    coef = "tarsus",
    resp = "rawSlope"
  ),
  set_prior("normal(0, 0.001)",
    class = "b",
    coef = "bill",
    resp = "rawSlope"
```

```
      ),
      set_prior("exponential(50)",
        class = "sd",
        group = "batch",
        resp = "rawSlope"
      ),
      set_prior("exponential(10)",
        class = "sigma",
        resp = "rawSlope"
      )
    ),
    iter = 50000, warmup = 10000, cores = 4, chains = 4, thin = 20,
    control = list(adapt_delta = .97, max_treedepth = 14),
    silent = TRUE, refresh = 0,
    file = "./models/_eightWeekFunctionModelMassR2.Rds"
  )

functionModel8WeeksTarsusR2 <-
  brm(
    data = slopeData %>%
      filter(week == "8") %>%
      mutate(
        mass = mass - mean(mass, na.rm = T),
        tarsus = tarsusLengthMean - mean(tarsusLengthMean, na.rm = T),
        bill = billLengthMean - mean(billLengthMean, na.rm = T),
        pretreatment = ifelse(pretreatment == "cold", "A",
          ifelse(pretreatment == "neutral", "B", "C")
        )
      ) %>%
      mutate(pretreatment = factor(pretreatment, levels = c("B", "A", "C"))) %>%
      drop_na() %>%
      merge(., data %>%
        dplyr::select(ring, "batch" = exp) %>%
        distinct(),
      by = "ring", all.x = TRUE
      ),
    family = "gaussian",
    bf(mass ~ pretreatment + (1 | batch)) +
      bf(tarsus ~ mass + pretreatment + (1 | batch)) +
      bf(bill ~ mass + pretreatment + (1 | batch)) +
      bf(rawSlope ~ mass + bill + pretreatment + (1 | batch)) +
      set_rescor(FALSE),
    prior = c(
      set_prior("normal(0, 10)",
        class = "Intercept",
        resp = "mass"
      ),
      set_prior("normal(0, 25)",
        class = "b",
        coef = "pretreatmentA",
        resp = "mass"
      ),
      set_prior("normal(0, 25)",
        class = "b",
        coef = "pretreatmentC",
        resp = "mass"
      ),
      set_prior("exponential(2.5)",
        class = "sd",
        group = "batch",
        resp = "mass"
      ),
      set_prior("exponential(0.15)",
        class = "sigma",
        resp = "mass"
      ),
      set_prior("normal(0, 2.5)",
```

```
      class = "Intercept",
      resp = "tarsus"
    ),
    set_prior("normal(0, 2.5)",
      class = "b",
      coef = "pretreatmentA",
      resp = "tarsus"
    ),
    set_prior("normal(0, 2.5)",
      class = "b",
      coef = "pretreatmentC",
      resp = "tarsus"
    ),
    set_prior("skew_normal(0, 0.25, 5)",
      class = "b",
      coef = "mass",
      resp = "tarsus"
    ),
    set_prior("exponential(2)",
      class = "sd",
      group = "batch",
      resp = "tarsus"
    ),
    set_prior("exponential(1)",
      class = "sigma",
      resp = "tarsus"
    ),
    set_prior("normal(0, 1)",
      class = "Intercept",
      resp = "bill"
    ),
    set_prior("normal(0, 0.5)",
      class = "b",
      coef = "pretreatmentA",
      resp = "bill"
    ),
    set_prior("normal(0, 0.5)",
      class = "b",
      coef = "pretreatmentC",
      resp = "bill"
    ),
    set_prior("skew_normal(0, 0.25, 5)",
      class = "b",
      coef = "mass",
      resp = "bill"
    ),
    set_prior("exponential(5)",
      class = "sd",
      group = "batch",
      resp = "bill"
    ),
    set_prior("exponential(2.5)",
      class = "sigma",
      resp = "bill"
    ),
    set_prior("normal(0, 0.01)",
      class = "Intercept",
      resp = "rawSlope"
    ),
    set_prior("normal(0, 0.025)",
      class = "b",
      coef = "pretreatmentA",
      resp = "rawSlope"
    ),
    set_prior("normal(0, 0.025)",
      class = "b",
      coef = "pretreatmentC",
```

```
        resp = "rawSlope"
      ),
      set_prior("normal(0, 0.001)",
        class = "b",
        coef = "mass",
        resp = "rawSlope"
      ),
      set_prior("normal(0, 0.001)",
        class = "b",
        coef = "bill",
        resp = "rawSlope"
      ),
      set_prior("exponential(50)",
        class = "sd",
        group = "batch",
        resp = "rawSlope"
      ),
      set_prior("exponential(10)",
        class = "sigma",
        resp = "rawSlope"
      )
    ),
    iter = 50000, warmup = 10000, cores = 4, chains = 4, thin = 20,
    control = list(adapt_delta = .97, max_treedepth = 14),
    silent = TRUE, refresh = 0,
    file = "./models/_eightWeekFunctionModelTarsusR2.Rds"
  )

functionModel8WeeksBillR2 <-
  brm(
    data = slopeData %>%
      filter(week == "8") %>%
      mutate(
        mass = mass - mean(mass, na.rm = T),
        tarsus = tarsusLengthMean - mean(tarsusLengthMean, na.rm = T),
        bill = billLengthMean - mean(billLengthMean, na.rm = T),
        pretreatment = ifelse(pretreatment == "cold", "A",
          ifelse(pretreatment == "neutral", "B", "C")
        )
      ) %>%
      mutate(pretreatment = factor(pretreatment, levels = c("B", "A", "C"))) %>%
      drop_na() %>%
      merge(., data %>%
        dplyr::select(ring, "batch" = exp) %>%
        distinct(),
      by = "ring", all.x = TRUE
      ),
    family = "gaussian",
    bf(mass ~ pretreatment + (1 | batch)) +
      bf(tarsus ~ mass + pretreatment + (1 | batch)) +
      bf(bill ~ mass + pretreatment + (1 | batch)) +
      bf(rawSlope ~ mass + tarsus + pretreatment + (1 | batch)) +
      set_rescor(FALSE),
    prior = c(
      set_prior("normal(0, 10)",
        class = "Intercept",
        resp = "mass"
      ),
      set_prior("normal(0, 25)",
        class = "b",
        coef = "pretreatmentA",
        resp = "mass"
      ),
      set_prior("normal(0, 25)",
        class = "b",
        coef = "pretreatmentC",
        resp = "mass"
```

```
    ),
    set_prior("exponential(2.5)",
      class = "sd",
      group = "batch",
      resp = "mass"
    ),
    set_prior("exponential(0.15)",
      class = "sigma",
      resp = "mass"
    ),
    set_prior("normal(0, 2.5)",
      class = "Intercept",
      resp = "tarsus"
    ),
    set_prior("normal(0, 2.5)",
      class = "b",
      coef = "pretreatmentA",
      resp = "tarsus"
    ),
    set_prior("normal(0, 2.5)",
      class = "b",
      coef = "pretreatmentC",
      resp = "tarsus"
    ),
    set_prior("skew_normal(0, 0.25, 5)",
      class = "b",
      coef = "mass",
      resp = "tarsus"
    ),
    set_prior("exponential(2)",
      class = "sd",
      group = "batch",
      resp = "tarsus"
    ),
    set_prior("exponential(1)",
      class = "sigma",
      resp = "tarsus"
    ),
    set_prior("normal(0, 1)",
      class = "Intercept",
      resp = "bill"
    ),
    set_prior("normal(0, 0.5)",
      class = "b",
      coef = "pretreatmentA",
      resp = "bill"
    ),
    set_prior("normal(0, 0.5)",
      class = "b",
      coef = "pretreatmentC",
      resp = "bill"
    ),
    set_prior("skew_normal(0, 0.25, 5)",
      class = "b",
      coef = "mass",
      resp = "bill"
    ),
    set_prior("exponential(5)",
      class = "sd",
      group = "batch",
      resp = "bill"
    ),
    set_prior("exponential(2.5)",
      class = "sigma",
      resp = "bill"
    ),
    set_prior("normal(0, 0.01)",
```

```
        class = "Intercept",
        resp = "rawSlope"
      ),
      set_prior("normal(0, 0.025)",
        class = "b",
        coef = "pretreatmentA",
        resp = "rawSlope"
      ),
      set_prior("normal(0, 0.025)",
        class = "b",
        coef = "pretreatmentC",
        resp = "rawSlope"
      ),
      set_prior("normal(0, 0.001)",
        class = "b",
        coef = "mass",
        resp = "rawSlope"
      ),
      set_prior("normal(0, 0.0025)",
        class = "b",
        coef = "tarsus",
        resp = "rawSlope"
      ),
      set_prior("exponential(50)",
        class = "sd",
        group = "batch",
        resp = "rawSlope"
      ),
      set_prior("exponential(10)",
        class = "sigma",
        resp = "rawSlope"
      )
    ),
    iter = 50000, warmup = 10000, cores = 4, chains = 4, thin = 20,
    control = list(adapt_delta = .97, max_treedepth = 14),
    silent = TRUE, refresh = 0,
    file = "./models/_eightWeekFunctionModelBillR2.Rds"
  )

functionModel8WeeksAppendageR2 <-
  brm(
    data = slopeData %>%
      filter(week == "8") %>%
      mutate(
        mass = mass - mean(mass, na.rm = T),
        tarsus = tarsusLengthMean - mean(tarsusLengthMean, na.rm = T),
        bill = billLengthMean - mean(billLengthMean, na.rm = T),
        pretreatment = ifelse(pretreatment == "cold", "A",
          ifelse(pretreatment == "neutral", "B", "C")
        )
      ) %>%
      mutate(pretreatment = factor(pretreatment, levels = c("B", "A", "C"))) %>%
      drop_na() %>%
      merge(., data %>%
        dplyr::select(ring, "batch" = exp) %>%
        distinct(),
      by = "ring", all.x = TRUE
      ),
    family = "gaussian",
    bf(mass ~ pretreatment + (1 | batch)) +
      bf(tarsus ~ mass + pretreatment + (1 | batch)) +
      bf(bill ~ mass + pretreatment + (1 | batch)) +
      bf(rawSlope ~ mass + pretreatment + (1 | batch)) +
      set_rescor(FALSE),
    prior = c(
      set_prior("normal(0, 10)",
        class = "Intercept",
```

```
    resp = "mass"
  ),
  set_prior("normal(0, 25)",
    class = "b",
    coef = "pretreatmentA",
    resp = "mass"
  ),
  set_prior("normal(0, 25)",
    class = "b",
    coef = "pretreatmentC",
    resp = "mass"
  ),
  set_prior("exponential(2.5)",
    class = "sd",
    group = "batch",
    resp = "mass"
  ),
  set_prior("exponential(0.15)",
    class = "sigma",
    resp = "mass"
  ),
  set_prior("normal(0, 2.5)",
    class = "Intercept",
    resp = "tarsus"
  ),
  set_prior("normal(0, 2.5)",
    class = "b",
    coef = "pretreatmentA",
    resp = "tarsus"
  ),
  set_prior("normal(0, 2.5)",
    class = "b",
    coef = "pretreatmentC",
    resp = "tarsus"
  ),
  set_prior("skew_normal(0, 0.25, 5)",
    class = "b",
    coef = "mass",
    resp = "tarsus"
  ),
  set_prior("exponential(2)",
    class = "sd",
    group = "batch",
    resp = "tarsus"
  ),
  set_prior("exponential(1)",
    class = "sigma",
    resp = "tarsus"
  ),
  set_prior("normal(0, 1)",
    class = "Intercept",
    resp = "bill"
  ),
  set_prior("normal(0, 0.5)",
    class = "b",
    coef = "pretreatmentA",
    resp = "bill"
  ),
  set_prior("normal(0, 0.5)",
    class = "b",
    coef = "pretreatmentC",
    resp = "bill"
  ),
  set_prior("skew_normal(0, 0.25, 5)",
    class = "b",
    coef = "mass",
    resp = "bill"
```

```
        ),
        set_prior("exponential(5)",
          class = "sd",
          group = "batch",
          resp = "bill"
        ),
        set_prior("exponential(2.5)",
          class = "sigma",
          resp = "bill"
        ),
        set_prior("normal(0, 0.01)",
          class = "Intercept",
          resp = "rawSlope"
        ),
        set_prior("normal(0, 0.025)",
          class = "b",
          coef = "pretreatmentA",
          resp = "rawSlope"
        ),
        set_prior("normal(0, 0.025)",
          class = "b",
          coef = "pretreatmentC",
          resp = "rawSlope"
        ),
        set_prior("normal(0, 0.001)",
          class = "b",
          coef = "mass",
          resp = "rawSlope"
        ),
        set_prior("exponential(50)",
          class = "sd",
          group = "batch",
          resp = "rawSlope"
        ),
        set_prior("exponential(10)",
          class = "sigma",
          resp = "rawSlope"
        )
      ),
      iter = 50000, warmup = 10000, cores = 4, chains = 4, thin = 20,
      control = list(adapt_delta = .97, max_treedepth = 14),
      silent = TRUE, refresh = 0,
      file = "./models/_eightWeekFunctionModelAppendageR2.Rds"
    )
}

pR2Pull <- function(x) {
  baseR2 <- as.data.frame(
    brms::bayes_R2(functionModel8Weeks,
      ndraws = 1000,
      resp = "rawSlope", summary = FALSE,
      robust = TRUE
    )
  )$R2rawSlope
  redR2 <- as.data.frame(
    brms::bayes_R2(x,
      ndraws = 1000,
      resp = "rawSlope", summary = FALSE,
      robust = TRUE
    )
  )$R2rawSlope
  pR2 <- round(baseR2 - redR2, digits = 3)

  ciFrame <- t(
    quantile(pR2, probs = c(0.025, 0.975), type = 8)
  ) %>% as.data.frame()
```

```
  ciFrame <- ciFrame %>%
    mutate(`2.5%` = ifelse(`2.5%` < 0, 0, `2.5%`)) %>%
    mutate("95\\% CI" = paste0(
      "[",
      round(`2.5%`, digits = 3),
      ",",
      round(`97.5%`, digits = 3),
      "]"
    )) %>%
    mutate("Partial R2" = round(median(pR2), digits = 3)) %>%
    mutate(`Partial R2` = ifelse(`Partial R2` < 0, 0, `Partial R2`)) %>%
    dplyr::select("Partial R\\textsuperscript{2}" = `Partial R2`, "95\\% CI")

  return(ciFrame)
}

caption = paste0('Variance in metabolic slope ',
  '(fold resting metabolsim at thermoneutrality/°C) explained by ',
  'morphometry in three week old Japanese quail. Metabolic ',
  'slopes are measured below thermoneutrality (<30°C).'
  )

models <- list(functionModel8WeeksMassR2, functionModel8WeeksTarsusR2,
            functionModel8WeeksBillR2, functionModel8WeeksAppendageR2)
bind_rows(lapply(models, pR2Pull)) %>%
  mutate("Variable" = c("Body Mass (g)", "Tarsus Length (mm)",
                        "Bill Length (mm)", "Appendage Length (mm)")
        ) %>%
  dplyr::select(Variable, `Partial R\\textsuperscript{2}`, `95\\% CI`) %>%
  kbl(.,
    longtable = T, booktabs = T, format = "latex",
    caption = caption, escape = FALSE
  ) %>%
  column_spec(column = c(1:10), width = "2.5cm")  %>%
  kable_styling(latex_options = "striped")
```

Table 53: *Variance in metabolic slope (fold resting metabolsim at thermoneutrality/°C) explained by morphometry in three week old Japanese quail. Metabolic slopes are measured below thermoneutrality (<30°C).*

Variable	Partial R^2	95% CI
Body Mass (g)	0.014	[0,0.173]
Tarsus Length (mm)	0.016	[0,0.172]
Bill Length (mm)	0.000	[0,0.167]
Appendage Length (mm)	0.014	[0,0.161]

```
rm(models)
```

Similar to above, we estimate the costs of misaligning with Bergmann's and Allen's rule at 10°C (i.e. by being two standard deviations below average mass, or two standard deviations above average tarsus length).

```
data.frame(
  "Size" = c("Average", "Small (2x s.d. < mean)"),
  "pretreatment" = "B",
  "mass" = c(
    mean(functionModel8Weeks$data$mass, na.rm = T),
    mean(functionModel8Weeks$data$mass, na.rm = T) -
      2 * sd(functionModel8Weeks$data$mass, na.rm = T)
  ),
  "tarsus" = 0,
  "bill" = 0
) %>%
  mutate("slope" = predict(functionModel8Weeks,
```

```
    newdata = .,
    robust = TRUE, re_form = NA,
    resp = "rawSlope"
  )[, "Estimate"]) %>%
  mutate(slope = slope +
    mean(
      subset(
        slopeData,
        week == 8 & pretreatment == "neutral"
      )$mslope,
      na.rm = T
    )) %>%
  mutate(cost = 1 + slope * -14) %>%
  dplyr::select("Body Size" = Size, "Expenditure Rate at 10°C\n(fold RMR)" = cost) %>%
  kbl(.,
    longtable = T, booktabs = T, format = "latex",
    caption = paste0("Comparison of estimated energy expenditure of ",
                     "eight week old Japanese quail at 10°C ",
                     "and varying body mass sizes."),
    escape = FALSE
  ) %>%
  column_spec(column = c(1:10), width = "2.5cm")  %>%
  kable_styling(latex_options = "striped")
```

Table 54: *Comparison of estimated energy expenditure of eight week old Japanese quail at 10°C and varying body mass sizes.*

Body Size	Expenditure Rate at 10°C (fold RMR)
Average	1.554506
Small (2x s.d. < mean)	1.533971

```
data.frame(
  "Size" = c("Average", "Long (2x s.d. > mean)"),
  "pretreatment" = "B",
  "tarsus" = c(
    mean(functionModel8Weeks$data$tarsus, na.rm = T),
    mean(functionModel8Weeks$data$tarsus, na.rm = T) +
      2 * sd(functionModel8Weeks$data$tarsus, na.rm = T)
  ),
  "mass" = 0,
  "bill" = 0
) %>%
  mutate("slope" = predict(functionModel8Weeks,
    newdata = .,
    robust = TRUE, re_form = NA,
    resp = "rawSlope"
  )[, "Estimate"]) %>%
  mutate(slope = slope +
    mean(subset(slopeData, week == 8)$mslope, na.rm = T)) %>%
  mutate(cost = 1 + slope * -14) %>%
  dplyr::select(
    "Tarsus Length" = Size,
    "Expenditure Rate at 10°C\n(fold RMR)" = cost
  ) %>%
  kbl(.,
    longtable = T, booktabs = T, format = "latex",
    caption = paste0("Comparison of estimated energy expenditure of ",
                     "eight week old Japanese quail at 10°C ",
                     "and varying tarsus lengths."),
    escape = FALSE
  ) %>%
  column_spec(column = c(1:10), width = "2.5cm")  %>%
```

```
  kable_styling(latex_options = "striped")
```

Table 55: *Comparison of estimated energy expenditure of eight week old Japanese quail at 10°C and varying tarsus lengths.*

Tarsus Length	Expenditure Rate at 10°C (fold RMR)
Average	1.563541
Long (2x s.d. > mean)	1.539351

```
data.frame(
  "Size" = c("Average", "Long (2x s.d. > mean)"),
  "pretreatment" = "B",
  "bill" = c(
    mean(functionModel8Weeks$data$bill, na.rm = T),
    mean(functionModel8Weeks$data$bill, na.rm = T) +
      2 * sd(functionModel8Weeks$data$bill, na.rm = T)
  ),
  "mass" = 0,
  "tarsus" = 0
) %>%
  mutate("slope" = predict(functionModel8Weeks,
    newdata = .,
    robust = TRUE, re_form = NA,
    resp = "rawSlope"
  )[, "Estimate"]) %>%
  mutate(slope = slope +
    mean(subset(slopeData, week == 8)$mslope, na.rm = T)) %>%
  mutate(cost = 1 + slope * -14) %>%
  dplyr::select(
    "Bill Length" = Size,
    "Expenditure Rate at 10°C\n(fold RMR)" = cost
  ) %>%
  kbl(.,
    longtable = T, booktabs = T, format = "latex",
    caption = paste0("Comparison of estimated energy expenditure of ",
                     "eight week old Japanese quail at 10°C ",
                     "and varying bill lengths."),
    escape = FALSE
  ) %>%
  column_spec(column = c(1:10), width = "2.5cm")  %>%
  kable_styling(latex_options = "striped")
```

Table 56: *Comparison of estimated energy expenditure of eight week old Japanese quail at 10°C and varying bill lengths.*

Bill Length	Expenditure Rate at 10°C (fold RMR)
Average	1.564126
Long (2x s.d. > mean)	1.562842

```
# Checking whether these differences are statistically clear

mismatchDFMass <- data.frame(
  "Size" = c("Average", "Small (2x s.d. < mean)"),
  "pretreatment" = "B",
  "mass" = c(
    mean(functionModel8Weeks$data$mass, na.rm = T),
```

```
    mean(functionModel8Weeks$data$mass, na.rm = T) -
      2 * sd(functionModel8Weeks$data$mass, na.rm = T)
  ),
  "tarsus" = 0,
  "bill" = 0
)

mismatchDFTarsus <- data.frame(
  "Size" = c("Average", "Large (2x s.d. > mean)"),
  "pretreatment" = "B",
  "tarsus" = c(
    mean(functionModel8Weeks$data$tarsus, na.rm = T),
    mean(functionModel8Weeks$data$tarsus, na.rm = T) +
      2 * sd(functionModel8Weeks$data$tarsus, na.rm = T)
  ),
  "mass" = 0,
  "bill" = 0
)

mismatchDFBill <- data.frame(
  "Size" = c("Average", "Large (2x s.d. > mean)"),
  "pretreatment" = "B",
  "bill" = c(
    mean(functionModel8Weeks$data$bill, na.rm = T),
    mean(functionModel8Weeks$data$bill, na.rm = T) +
      2 * sd(functionModel8Weeks$data$bill, na.rm = T)
  ),
  "mass" = 0,
  "tarsus" = 0
)

mismatchTestFun <- function(x) {
  df <- predict(functionModel8Weeks,
    newdata = x,
    robust = TRUE, re_form = NA,
    resp = "rawSlope", summary = FALSE
  ) %>%
    as.data.frame() %>%
    rename("Average" = "V1", "Mismatch" = "V2") %>%
    mutate_all(.funs = function(x) {
      x + mean(subset(slopeData, week == 8)$mslope, na.rm = T)
    })

  mismatchDFTest <- build_hdf(
    vars = list(
      df$Average,
      df$Mismatch
    ),
    priors = list(
      rnorm(nrow(df), 0, 0.01),
      rnorm(nrow(df), 0, 0.01)
    ),
    names = c("Average", "Mismatch")
  )
  mismatchTest <- hypothesis_df("Mismatch > Average",
    mismatchDFTest,
    class = "b", alpha = 0.05
  )

  mismatchTest$hypothesis$Hypothesis <-
    "Mismatch Metabolic Slope > Average Slope"

  return(mismatchTest)
}

mismatchTests <- lapply(
  list(mismatchDFMass, mismatchDFTarsus, mismatchDFBill),
```

```
  mismatchTestFun
  )

# Summarising

caption <- paste0(
  "Results of a non-linear hypothesis ",
  "tests comparing predicted metabolic slopes in the cold (30°C - 10°C) ",
  "among eight week old Japanese quail of average or atypically small ",
  "body size (mean - 2 standard deviations). Posterior probabilities are ",
  "calculated using the Savage-Dickey ",
  "density ratio method."
)

mismatchTestMassTable <- mismatchTests[[1]]$hypothesis %>%
  mutate(Hypothesis = c(
    "Small Metabolic Slope > Average Slope"
  )) %>%
  dplyr::select(-c(Evid.Ratio, Star)) %>%
  mutate(
    Estimate = round(Estimate, digits = 4),
    "Est.Error" = round(Est.Error, digits = 4),
    "CI.Lower" = round(CI.Lower, digits = 4),
    "CI.Upper" = round(CI.Upper, digits = 4),
    "Posterior Probability" = round(Post.Prob, digits = 4)
  ) %>%
  rename(
    "Difference Between Metabolic Slopes" = Estimate,
    "Standard Error" = Est.Error,
    `2.5 % CI` = "CI.Lower",
    `97.5 % CI` = "CI.Upper"
  ) %>%
  dplyr::select(-Post.Prob) %>%
  kbl(.,
    longtable = T, booktabs = T, format = "latex",
    caption = caption
  ) %>%
  column_spec(column = c(1:2), width = "2.5cm") %>%
  column_spec(column = c(3:10), width = "2cm") %>%
  kable_styling(latex_options = "striped")

mismatchTestMassTable
```

Table 57: *Results of a non-linear hypothesis tests comparing predicted metabolic slopes in the cold (30°C - 10°C) among eight week old Japanese quail of average or atypically small body size (mean - 2 standard deviations). Posterior probabilities are calculated using the Savage-Dickey density ratio method.*

Hypothesis	Difference Between Metabolic Slopes	Standard Error	2.5 % CI	97.5 % CI	Posterior Probability
Small Metabolic Slope > Average Slope	0.0013	0.0284	-0.0453	0.0475	0.5174

```
# For tarsus length

caption <- paste0(
  "Results of a non-linear hypothesis ",
  "tests comparing predicted metabolic slopes in the cold (30°C - 10°C) ",
  "among eight week old Japanese quail of average or atypically long ",
  "tarsus length (mean + 2 standard deviations). Posterior probabilities are ",
  "calculated using the Savage-Dickey ",
  "density ratio method."
)

mismatchTestTarsusTable <- mismatchTests[[2]]$hypothesis %>%
```

```
  mutate(Hypothesis = c(
    "Long Metabolic Slope > Average Slope"
  )) %>%
  dplyr::select(-c(Evid.Ratio, Star)) %>%
  mutate(
    Estimate = round(Estimate, digits = 4),
    "Est.Error" = round(Est.Error, digits = 4),
    "CI.Lower" = round(CI.Lower, digits = 4),
    "CI.Upper" = round(CI.Upper, digits = 4),
    "Posterior Probability" = round(Post.Prob, digits = 4)
  ) %>%
  rename(
    "Difference Between Metabolic Slopes" = Estimate,
    "Standard Error" = Est.Error,
    `2.5 % CI` = "CI.Lower",
    `97.5 % CI` = "CI.Upper"
  ) %>%
  dplyr::select(-Post.Prob) %>%
  kbl(.,
    longtable = T, booktabs = T, format = "latex",
    caption = caption
  ) %>%
  column_spec(column = c(1:2), width = "2.5cm") %>%
  column_spec(column = c(3:10), width = "2cm") %>%
  kable_styling(latex_options = "striped")

mismatchTestTarsusTable
```

Table 58: *Results of a non-linear hypothesis tests comparing predicted metabolic slopes in the cold (30°C - 10°C) among eight week old Japanese quail of average or atypically long tarsus length (mean + 2 standard deviations). Posterior probabilities are calculated using the Savage-Dickey density ratio method.*

Hypothesis	Difference Between Metabolic Slopes	Standard Error	2.5 % CI	97.5 % CI	Posterior Probability
Long Metabolic Slope > Average Slope	0.0022	0.0278	-0.0427	0.0475	0.5308

```
## Bill length

caption <- paste0(
  "Results of a non-linear hypothesis ",
  "tests comparing predicted metabolic slopes in the cold (30°C - 10°C) ",
  "among eight week old Japanese quail of average or atypically long ",
  "bill length (mean + 2 standard deviations). Posterior probabilities are ",
  "calculated using the Savage-Dickey ",
  "density ratio method."
)

mismatchTestBillTable <- mismatchTests[[3]]$hypothesis %>%
  mutate(Hypothesis = c(
    "Long Metabolic Slope > Average Slope"
  )) %>%
  dplyr::select(-c(Evid.Ratio, Star)) %>%
  mutate(
    Estimate = round(Estimate, digits = 4),
    "Est.Error" = round(Est.Error, digits = 4),
    "CI.Lower" = round(CI.Lower, digits = 4),
    "CI.Upper" = round(CI.Upper, digits = 4),
    "Posterior Probability" = round(Post.Prob, digits = 4)
  ) %>%
  rename(
    "Difference Between Metabolic Slopes" = Estimate,
    "Standard Error" = Est.Error,
    `2.5 % CI` = "CI.Lower",
```

```
    `97.5 % CI` = "CI.Upper"
  ) %>%
  dplyr::select(-Post.Prob) %>%
  kbl(.,
    longtable = T, booktabs = T, format = "latex",
    caption = caption
  ) %>%
  column_spec(column = c(1:2), width = "2.5cm") %>%
  column_spec(column = c(3:10), width = "2cm") %>%
  kable_styling(latex_options = "striped")

mismatchTestBillTable
```

Table 59: *Results of a non-linear hypothesis tests comparing predicted metabolic slopes in the cold (30°C - 10°C) among eight week old Japanese quail of average or atypically long bill length (mean + 2 standard deviations). Posterior probabilities are calculated using the Savage-Dickey density ratio method.*

Hypothesis	Difference Between Metabolic Slopes	Standard Error	2.5 % CI	97.5 % CI	Posterior Probability
Long Metabolic Slope > Average Slope	1e-04	0.0283	-0.0462	0.0471	0.5004

We then visualise effects of the thermal environment and morphometry on metabolic slopes in eight week old Japanese quail.

```
# Plotting outcomes

p1_8 <- data.frame("pretreatment" = c("B", "A", "C")) %>%
  mutate(
    "mass" = predict(functionModel8Weeks,
      newdata = ., robust = TRUE,
      resp = "mass", re_form = NA
    )[, "Estimate"],
    "SE" = predict(functionModel8Weeks,
      newdata = ., robust = TRUE,
      resp = "mass", re_form = NA
    )[, "Est.Error"]
  ) %>%
  mutate("Mass" = mass +
    mean(subset(slopeData, week == "8")$mass,
      na.rm = T
    )) %>%
  mutate("pretreatment" = ifelse(pretreatment == "A",
    "Cold\n(10°C)",
    ifelse(pretreatment == "B", "Mild\n(20°C)",
      "Warm\n(30°C)"
    )
  )) %>%
  ggplot(aes(x = pretreatment, y = Mass)) +
  geom_errorbar(aes(
    x = pretreatment, ymin = Mass - SE,
    ymax = Mass + SE
  ), colour = "black", width = 0.3) +
  geom_point(
    size = 5, pch = 21, colour = "black",
    aes(x = pretreatment, fill = factor(pretreatment))
  ) +
  geom_line(linetype = "dashed", colour = "black") +
  geom_point(
    size = 2, pch = 21, colour = "black",
    data = subset(slopeData, week == "8") %>%
      mutate("pretreatment" = ifelse(pretreatment == "cold",
```

```
        "Cold\n(10°C)",
        ifelse(pretreatment == "neutral", "Mild\n(20°C)",
          "Warm\n(30°C)"
        )
      )),
    aes(x = pretreatment, y = mass, fill = factor(pretreatment)),
    position = position_jitter(width = 0.3)
  ) +
  scale_fill_manual(values = c("#7BB4E3", "black", "#CD5C5C")) +
  theme_classic() +
  theme(
    legend.position = "none",
    axis.text = element_text(family = "Noto Sans"),
    axis.title = element_text(family = "Noto Sans")
  ) +
  xlab("Rearing Conditions") +
  ylab("Mass (g)")

p2_8 <- data.frame(
  "pretreatment" = c("A", "B", "C"),
  "mass" = 0
) %>%
  mutate(
    "tarsus" = predict(functionModel8Weeks,
      newdata = ., robust = TRUE,
      resp = "tarsus", re_form = NA
    )[, "Estimate"],
    "SE" = predict(functionModel8Weeks,
      newdata = ., robust = TRUE,
      resp = "tarsus", re_form = NA
    )[, "Est.Error"]
  ) %>%
  mutate("Tarsus" = tarsus +
    mean(subset(slopeData, week == "8")$tarsusLengthMean,
      na.rm = T
    )) %>%
  mutate("Mass" = mass +
    mean(subset(slopeData, week == "8")$mass,
      na.rm = T
    )) %>%
  mutate("pretreatment" = ifelse(pretreatment == "A",
    "Cold\n(10°C)",
    ifelse(pretreatment == "B", "Mild\n(20°C)",
      "Warm\n(30°C)"
    )
  )) %>%
  ggplot(aes(x = pretreatment, y = Tarsus)) +
  geom_errorbar(aes(
    x = pretreatment, ymin = Tarsus - SE,
    ymax = Tarsus + SE
  ), colour = "black", width = 0.3) +
  geom_point(
    size = 5, pch = 21, colour = "black",
    aes(x = pretreatment, fill = factor(pretreatment))
  ) +
  geom_line(linetype = "dashed", colour = "black") +
  geom_point(
    size = 2, pch = 21, colour = "black",
    data = subset(slopeData, week == "8") %>%
      mutate("pretreatment" = ifelse(pretreatment == "cold", "Cold\n(10°C)",
        ifelse(pretreatment == "neutral", "Mild\n(20°C)", "Warm\n(30°C)")
      )),
    aes(
      x = pretreatment, y = tarsusLengthMean,
      fill = factor(pretreatment)
    ),
    position = position_jitter(width = 0.3)
```

```
  ) +
  scale_fill_manual(values = c("#7BB4E3", "black", "#CD5C5C")) +
  theme_classic() +
  theme(
    legend.position = "none",
    axis.text = element_text(family = "Noto Sans"),
    axis.title = element_text(family = "Noto Sans")
  ) +
  xlab("Rearing Conditions") +
  ylab("Tarsus Length (mm)")

p3_8 <- data.frame(
  "pretreatment" = c("A", "B", "C"),
  "mass" = 0
) %>%
  mutate(
    "bill" = predict(functionModel8Weeks,
      newdata = ., robust = TRUE,
      resp = "bill", re_form = NA
    )[, "Estimate"],
    "SE" = predict(functionModel8Weeks,
      newdata = ., robust = TRUE,
      resp = "bill", re_form = NA
    )[, "Est.Error"]
  ) %>%
  mutate("Bill" = bill +
    mean(subset(slopeData, week == "8")$billLengthMean,
      na.rm = T
    )) %>%
  mutate("Mass" = mass +
    mean(subset(slopeData, week == "8")$mass,
      na.rm = T
    )) %>%
  mutate("pretreatment" = ifelse(pretreatment == "A",
    "Cold\n(10°C)",
    ifelse(pretreatment == "B", "Mild\n(20°C)",
      "Warm\n(30°C)"
    )
  )) %>%
  ggplot(aes(x = pretreatment, y = Bill)) +
  geom_errorbar(aes(
    x = pretreatment, ymin = Bill - SE,
    ymax = Bill + SE
  ), colour = "black", width = 0.3) +
  geom_point(
    size = 5, pch = 21, colour = "black",
    aes(x = pretreatment, fill = factor(pretreatment))
  ) +
  geom_line(linetype = "dashed", colour = "black") +
  geom_point(
    size = 2, pch = 21, colour = "black",
    data = subset(slopeData, week == "8") %>%
      mutate("pretreatment" = ifelse(pretreatment == "cold", "Cold\n(10°C)",
        ifelse(pretreatment == "neutral", "Mild\n(20°C)", "Warm\n(30°C)")
      )),
    aes(
      x = pretreatment, y = billLengthMean,
      fill = factor(pretreatment)
    ),
    position = position_jitter(width = 0.3)
  ) +
  scale_fill_manual(values = c("#7BB4E3", "black", "#CD5C5C")) +
  theme_classic() +
  theme(
    legend.position = "none",
    axis.text = element_text(family = "Noto Sans"),
    axis.title = element_text(family = "Noto Sans")
```

```
  ) +
  xlab("Rearing Conditions") +
  ylab("Bill Length (mm)")

p4_8 <- data.frame(
  "pretreatment" = c("A", "B", "C"),
  "mass" = 0,
  "tarsus" = 0,
  "bill" = 0
) %>%
  mutate(
    "slope" = predict(functionModel8Weeks,
      newdata = ., robust = TRUE, re_form = NA,
      resp = "rawSlope"
    )[, "Estimate"],
    "SE" = predict(functionModel8Weeks,
      newdata = ., robust = TRUE, re_form = NA,
      resp = "rawSlope"
    )[, "Est.Error"]
  ) %>%
  mutate("pretreatment" = ifelse(pretreatment == "A",
    "Cold\n(10°C)",
    ifelse(pretreatment == "B",
      "Mild\n(20°C)",
      "Warm\n(30°C)"
    )
  )) %>%
  mutate("slope" = slope +
    mean(subset(slopeData, week == "8")$mslope,
      na.rm = T
    )) %>%
  ggplot(aes(x = pretreatment, y = slope)) +
  geom_errorbar(
    aes(
      x = pretreatment,
      ymin = slope - SE, ymax = slope + SE
    ),
    colour = "black", width = 0.3
  ) +
  geom_point(
    size = 5, pch = 21, colour = "black",
    aes(x = pretreatment, fill = factor(pretreatment))
  ) +
  geom_line(linetype = "dashed", colour = "black") +
  geom_point(
    size = 2, pch = 21, colour = "black",
    data = subset(slopeData, week == "8") %>%
      mutate("pretreatment" = ifelse(pretreatment == "cold",
        "Cold\n(10°C)",
        ifelse(pretreatment == "neutral",
          "Mild\n(20°C)",
          "Warm\n(30°C)"
        )
      )),
    aes(
      x = pretreatment, y = mslope,
      fill = factor(pretreatment)
    ),
    position = position_jitter(width = 0.3)
  ) +
  scale_fill_manual(values = c("#7BB4E3", "black", "#CD5C5C")) +
  theme_classic() +
  theme(
    legend.position = "none",
    axis.text = element_text(family = "Noto Sans"),
    axis.title = element_text(family = "Noto Sans")
  ) +
```

```
  xlab("Rearing Conditions") +
  ylab("Metabolic Slope\n(Fold Metabolism at\nThermoneutrality)")

pretreatmentPanel8 <- ((p1_8/p2_8) | (p3_8/p4_8)) +
  plot_annotation(tag_levels = "A")

showtext_auto()
pretreatmentPanel8
```

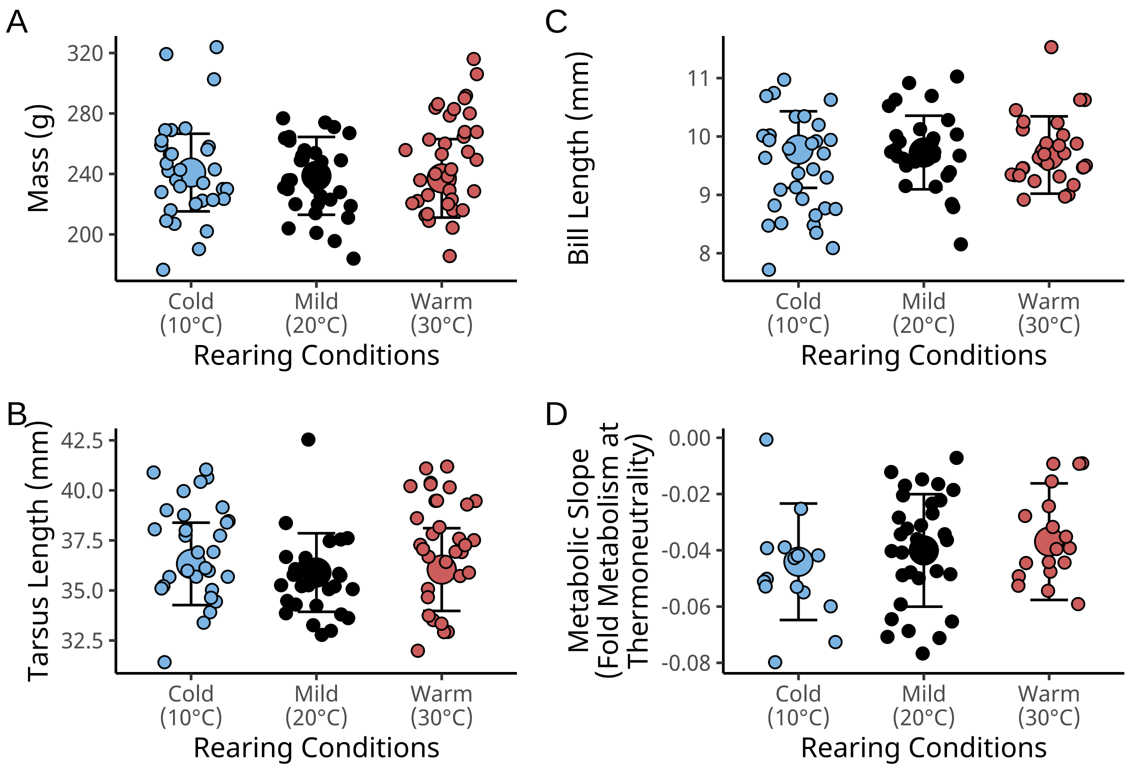

Figure 107: *Effects of post-hatch thermal conditions on morphometry and thermal physiology in eight week old Japanese quail. Small dots represent raw data points while large dots represent estimated effectgs per grouping from a Bayesian path analysis. Errorbars represent +/- one standard error. Panels A to C display effects of rearing conditions on morphology, while panel D represents effects of rearing conditions on thermal physiology. Cold and warm rearing treatments persisted for at least 3 weeks of life, followed by holding at 20°C.*

```
ggsave("./plots/rearingConditionEffects8Weeks_2Fold.pdf",
  pretreatmentPanel8,
  dpi = 800, height = 7, width = 9
)
showtext_auto(enable = "FALSE")
```

```
p1 <- data.frame(
  "pretreatment" = "B",
  "mass" = seq(min(functionModel8Weeks$data$mass, na.rm = T),
    max(functionModel8Weeks$data$mass, na.rm = T),
    by = 0.1
  ),
  "tarsus" = 0,
  "bill" = 0
) %>%
  mutate(
    "slope" = predict(functionModel8Weeks,
      newdata = ., robust = TRUE, re_form = NA,
```

```
      resp = "rawSlope"
    )[, "Estimate"],
    "SE" = predict(functionModel8Weeks,
      newdata = ., robust = TRUE, re_form = NA,
      resp = "rawSlope"
    )[, "Est.Error"]
  ) %>%
  mutate(slope = slope +
    mean(subset(slopeData, week == 8)$mslope,
      na.rm = T
    )) %>%
  mutate("Mass" = mass +
    mean(subset(slopeData, week == "8")$mass,
      na.rm = T
    )) %>%
  ggplot(aes(x = Mass, y = slope)) +
  geom_ribbon(
    aes(
      x = Mass, ymin = slope - SE,
      ymax = slope + SE
    ),
    fill = "grey50", alpha = 0.5
  ) +
  geom_smooth(
    method = "lm", linetype = "dashed",
    se = FALSE, colour = "black"
  ) +
  geom_point(
    size = 2.5, pch = 21, colour = "black", alpha = 0.5, fill = "grey50",
    data = subset(slopeData, week == "8"),
    aes(x = mass, y = mslope)
  ) +
  xlim(c(180, 300)) +
  theme_classic() +
  theme(
    axis.text = element_text(family = "Noto Sans"),
    axis.title = element_text(family = "Noto Sans"),
    legend.position = "none"
  ) +
  xlab("Body Mass (g)") +
  ylab("Metabolic Slope < 30°C\n(Fold Metabolism/°C)")

p2 <- data.frame(
  "pretreatment" = "B",
  "tarsus" = seq(
    min(functionModel8Weeks$data$tarsus,
      na.rm = T
    ),
    max(functionModel8Weeks$data$tarsus,
      na.rm = T
    ),
    by = 0.1
  ),
  "mass" = 0,
  "bill" = 0
) %>%
  mutate(
    "slope" = predict(functionModel8Weeks,
      newdata = ., robust = TRUE, re_form = NA,
      resp = "rawSlope"
    )[, "Estimate"],
    "SE" = predict(functionModel8Weeks,
      newdata = ., robust = TRUE, re_form = NA,
      resp = "rawSlope"
    )[, "Est.Error"]
  ) %>%
  mutate("Tarsus" = tarsus +
```

```
    mean(subset(slopeData, week == "8")$tarsusLengthMean,
      na.rm = T
    )) %>%
  mutate(slope = slope +
    mean(subset(slopeData, week == "8")$mslope,
      na.rm = T
    )) %>%
  ggplot(aes(x = Tarsus, y = slope)) +
  geom_ribbon(
    aes(
      x = Tarsus, ymin = slope - SE,
      ymax = slope + SE
    ),
    fill = "grey50", alpha = 0.5
  ) +
  geom_smooth(
    method = "lm", linetype = "dashed",
    se = FALSE, colour = "black"
  ) +
  geom_point(
    size = 2.5, pch = 21, colour = "black",
    alpha = 0.5, fill = "grey50",
    data = subset(slopeData, week == "8"),
    aes(x = tarsusLengthMean, y = mslope)
  ) +
  xlim(c(30, 42)) +
  theme_classic() +
  theme(
    legend.position = "none",
    axis.text = element_text(family = "Noto Sans"),
    axis.title = element_text(family = "Noto Sans")
  ) +
  xlab("Tarsus Length (mm)") +
  ylab("Metabolic Slope < 30°C\n(Fold Metabolism/°C)")

p3 <- data.frame(
  "pretreatment" = "B",
  "bill" = seq(
    min(functionModel8Weeks$data$bill,
      na.rm = T
    ),
    max(functionModel8Weeks$data$bill,
      na.rm = T
    ),
    by = 0.1
  ),
  "mass" = 0,
  "tarsus" = 0
) %>%
  mutate(
    "slope" = predict(functionModel8Weeks,
      newdata = ., robust = TRUE, re_form = NA,
      resp = "rawSlope"
    )[, "Estimate"],
    "SE" = predict(functionModel8Weeks,
      newdata = ., robust = TRUE, re_form = NA,
      resp = "rawSlope"
    )[, "Est.Error"]
  ) %>%
  mutate("Bill" = bill +
    mean(subset(slopeData, week == "8")$billLengthMean,
      na.rm = T
    )) %>%
  mutate(slope = slope +
    mean(subset(slopeData, week == "8")$mslope,
      na.rm = T
    )) %>%
```

```
  ggplot(aes(x = Bill, y = slope)) +
  geom_ribbon(
    aes(
      x = Bill, ymin = slope - SE,
      ymax = slope + SE
    ),
    fill = "grey50", alpha = 0.5
  ) +
  geom_smooth(
    method = "lm", linetype = "dashed",
    se = FALSE, colour = "black"
  ) +
  geom_point(
    size = 2.5, pch = 21, colour = "black",
    alpha = 0.5, fill = "grey50",
    data = subset(slopeData, week == "8"),
    aes(x = billLengthMean, y = mslope)
  ) +
  xlim(c(7, 12)) +
  theme_classic() +
  theme(
    legend.position = "none",
    axis.text = element_text(family = "Noto Sans"),
    axis.title = element_text(family = "Noto Sans")
  ) +
  xlab("Bill Length (mm)") +
  ylab("Metabolic Slope < 30°C\n(Fold Metabolism/°C)")

massPred <- data.frame(
  "pretreatment" = "B",
  "mass" = c(
    mean(functionModel8Weeks$data$mass, na.rm = T) -
      sd(functionModel8Weeks$data$mass, na.rm = T),
    mean(functionModel8Weeks$data$mass, na.rm = T),
    mean(functionModel8Weeks$data$mass, na.rm = T) +
      sd(functionModel8Weeks$data$mass, na.rm = T)
  ),
  "tarsus" = 0,
  "bill" = 0
) %>%
  predict(functionModel8Weeks,
    re_form = NA,
    resp = "rawSlope", newdata = .,
    summary = TRUE, robust = TRUE
  ) %>%
  as.data.frame() %>%
  mutate("slope" = Estimate +
    mean(
      subset(
        slopeData,
        week == 8 & pretreatment == "neutral"
      )$mslope,
      na.rm = TRUE
    )) %>%
  mutate("Size" = c("Small", "Average", "Large")) %>%
  dplyr::select(Size, slope, "slopeSE" = Est.Error) %>%
  slice(rep(1:n(), each = length(seq(-20, -0.1, by = 0.1)))) %>%
  mutate("Ta" = rep(seq(-20, -0.1, by = 0.1), 3)) %>%
  mutate(
    "VO2" = 1 + Ta * slope,
    "LL" = 1 + Ta * (slope - slopeSE),
    "UL" = 1 + Ta * (slope + slopeSE)
  ) %>%
  mutate(
    "Ta" = Ta + 22,
    Size = factor(Size, levels = c("Small", "Average", "Large"))
  )
```

```
jitterer <- position_jitter(width = 1, seed = 100)

massPlot <- massPred %>%
  ggplot(aes(x = Ta, y = VO2)) +
  geom_point(
    data = data.frame("Ta" = 22, "VO2" = 1),
    pch = 21, colour = "black", fill = "grey15",
    size = 3
  ) +
  geom_point(
    data = slopes8Weeks$data %>%
      merge(., subset(all, week == "8") %>%
        dplyr::select(ring, mass),
      by = "ring", all.x = TRUE
      ) %>%
      mutate(Ta = Ta + 24),
    aes(x = Ta, y = VO2),
    position = jitterer,
    pch = 21, colour = "black", fill = "transparent"
  ) +
  geom_point(
    data = slopes8Weeks$data %>%
      merge(., subset(all, week == "8") %>%
        dplyr::select(ring, mass),
      by = "ring", all.x = TRUE
      ) %>%
      mutate(Ta = Ta + 24),
    aes(x = Ta, y = VO2, colour = mass),
    position = jitterer, alpha = 0.5
  ) +
  scale_colour_gradient2(
    low = "#19242E",
    mid = "#BBD8F0",
    high = "grey98",
    midpoint = mean(subset(slopeData, week == "8")$mass, na.rm = T),
    guide = NULL
  ) +
  geom_ribbon(aes(x = Ta, ymin = LL, ymax = UL, fill = Size),
    alpha = 0.4, colour = "black"
  ) +
  scale_fill_manual(
    values = c("#194A7A", "#BBD8F0", "grey98"),
    labels = c(
      paste0(
        "Mean Mass -\n1 SD (",
        round(
          mean(subset(slopeData, week == "8")$mass, na.rm = T) -
            sd(subset(slopeData, week == "8")$mass, na.rm = T),
          digits = 0.1
        ),
        "g)"
      ),
      paste0(
        "Mean Mass\n(",
        round(mean(subset(slopeData, week == "8")$mass, na.rm = T),
          digits = 0.1
        ),
        "g)"
      ),
      paste0(
        "Mean Mass +\n1 SD (",
        round(
          mean(subset(slopeData, week == "8")$mass, na.rm = T) +
            sd(subset(slopeData, week == "8")$mass, na.rm = T),
          digits = 0.1
        ),
        "g)"
```

```
      )
    ), name = NULL
  ) +
  scale_linetype_manual(
    values = c("solid", "dashed", "dotted"),
    labels = c(
      paste0(
        "Mean Mass -\n1 SD (",
        round(
          mean(subset(slopeData, week == "8")$mass, na.rm = T) -
            sd(subset(slopeData, week == "8")$mass, na.rm = T),
          digits = 0.1
        ),
        "g)"
      ),
      paste0(
        "Mean Mass\n(",
        round(mean(subset(slopeData, week == "8")$mass, na.rm = T),
          digits = 0.1
        ),
        "g)"
      ),
      paste0(
        "Mean Mass +\n1 SD (",
        round(
          mean(subset(slopeData, week == "8")$mass, na.rm = T) +
            sd(subset(slopeData, week == "8")$mass, na.rm = T),
          digits = 0.1
        ),
        "g)"
      )
    ), name = NULL
  ) +
  geom_line(
    aes(linetype = Size)
  ) +
  theme_classic() +
  xlab("Ambient Temperature (°C)") +
  ylab("Resting Metabolism\n(Fold Change from Thermoneutrality)") +
  scale_linetype_manual(
    values = c("solid", "dashed", "dotted"),
    labels = c(
      paste0(
        "Mean Mass -\n1 SD (",
        round(
          mean(subset(slopeData, week == "8")$mass, na.rm = T) -
            sd(subset(slopeData, week == "8")$mass, na.rm = T),
          digits = 0.1
        ),
        "g)"
      ),
      paste0(
        "Mean Mass\n(",
        round(mean(subset(slopeData, week == "8")$mass, na.rm = T),
          digits = 0.1
        ),
        "g)"
      ),
      paste0(
        "Mean Mass +\n1 SD (",
        round(
          mean(subset(slopeData, week == "8")$mass, na.rm = T) +
            sd(subset(slopeData, week == "8")$mass, na.rm = T),
          digits = 0.1
        ),
        "g)"
      )
```

```
    ), name = NULL
  ) +
  guides(
    fill = guide_legend(override.aes = list(size = 3)),
    linetype = guide_legend(override.aes = list(size = 4))
  ) +
  theme(
    axis.text = element_text(family = "Noto Sans"),
    axis.title = element_text(family = "Noto Sans"),
    legend.text = element_text(family = "Noto Sans", size = 7),
    legend.title = element_text(family = "Noto Sans"),
    legend.position = "bottom"
  )

showtext_auto()
ggsave("./plots/massPlot8Weeks.pdf",
  massPlot,
  dpi = 800, height = 7, width = 7
)
showtext_auto(enable = FALSE)

tarsusPred <- data.frame(
  "pretreatment" = "B",
  "tarsus" = c(
    mean(functionModel8Weeks$data$tarsus, na.rm = T) -
      sd(functionModel8Weeks$data$tarsus, na.rm = T),
    mean(functionModel8Weeks$data$tarsus, na.rm = T),
    mean(functionModel8Weeks$data$tarsus, na.rm = T) +
      sd(functionModel8Weeks$data$tarsus, na.rm = T)
  ),
  "mass" = 0,
  "bill" = 0
) %>%
  predict(functionModel8Weeks,
    re_form = NA,
    resp = "rawSlope", newdata = .,
    summary = TRUE, robust = TRUE
  ) %>%
  as.data.frame() %>%
  mutate("slope" = Estimate +
    mean(
      subset(
        slopeData,
        week == 8 & pretreatment == "neutral"
      )$mslope,
      na.rm = TRUE
    )) %>%
  mutate("Size" = c("Small", "Average", "Large")) %>%
  dplyr::select(Size, slope, "slopeSE" = Est.Error) %>%
  slice(rep(1:n(), each = length(seq(-20, -0.1, by = 0.1)))) %>%
  mutate("Ta" = rep(seq(-20, -0.1, by = 0.1), 3)) %>%
  mutate(
    "VO2" = 1 + Ta * slope,
    "LL" = 1 + Ta * (slope - slopeSE),
    "UL" = 1 + Ta * (slope + slopeSE)
  ) %>%
  mutate(
    "Ta" = Ta + 22,
    Size = factor(Size, levels = c("Small", "Average", "Large"))
  )

tarsusPlot <- tarsusPred %>%
  ggplot(aes(x = Ta, y = VO2)) +
  geom_point(
    data = slopes8Weeks$data %>%
      merge(., subset(all, week == "8") %>%
        select(ring, "tarsus" = tarsusLengthMean),
```

```
      by = "ring", all.x = TRUE
      ) %>%
      mutate(Ta = Ta + 24),
    aes(x = Ta, y = VO2),
    position = jitterer, alpha = 0.5,
    pch = 21, colour = "black", fill = "transparent"
  ) +
  geom_point(
    data = slopes8Weeks$data %>%
      merge(., subset(all, week == "8") %>%
        select(ring, "tarsus" = tarsusLengthMean),
      by = "ring", all.x = TRUE
      ) %>%
      mutate(Ta = Ta + 24),
    aes(x = Ta, y = VO2, colour = tarsus),
    position = jitterer, alpha = 0.5
  ) +
  geom_point(
    data = data.frame("Ta" = 22, "VO2" = 1),
    pch = 21, colour = "black", fill = "grey15",
    size = 3
  ) +
  geom_ribbon(aes(x = Ta, ymin = LL, ymax = UL, fill = Size),
    alpha = 0.4, colour = "black"
  ) +
  geom_line(
    aes(linetype = Size)
  ) +
  theme_classic() +
  xlab("Ambient Temperature (°C)") +
  ylab("Resting Metabolism\n(Fold Change from Thermoneutrality)") +
  scale_fill_manual(
    values = c("#194A7A", "#BBD8F0", "grey98"),
    labels = c(
      paste0(
        "Mean Tarsus\nLength -\n1 SD (",
        round(
          mean(subset(slopeData, week == "8")$tarsusLengthMean,
            na.rm = T
          ) -
            sd(subset(slopeData, week == "8")$tarsusLengthMean,
              na.rm = T
            ),
          digits = 0.1
        ),
        "mm)"
      ),
      paste0(
        "Mean Tarsus\nLength (",
        round(
          mean(subset(slopeData, week == "8")$tarsusLengthMean,
            na.rm = T
          ),
          digits = 0.1
        ),
        "mm)"
      ),
      paste0(
        "Mean Tarsus\nLength +\n1 SD (",
        round(
          mean(subset(slopeData, week == "8")$tarsusLengthMean,
            na.rm = T
          ) +
            sd(subset(slopeData, week == "8")$tarsusLengthMean,
              na.rm = T
            ),
          digits = 0.1
```

```
        ),
        "mm)"
      )
    ), name = NULL
  ) +
  scale_linetype_manual(
    values = c("solid", "dashed", "dotted"),
    labels = c(
      paste0(
        "Mean Tarsus\nLength -\n1 SD (",
        round(
          mean(subset(slopeData, week == "8")$tarsusLengthMean,
            na.rm = T
          ) -
            sd(subset(slopeData, week == "8")$tarsusLengthMean,
              na.rm = T
            ),
          digits = 0.1
        ),
        "mm)"
      ),
      paste0(
        "Mean Tarsus\nLength (",
        round(
          mean(subset(slopeData, week == "8")$tarsusLengthMean,
            na.rm = T
          ),
          digits = 0.1
        ),
        "mm)"
      ),
      paste0(
        "Mean Tarsus\nLength +\n1 SD (",
        round(
          mean(subset(slopeData, week == "8")$tarsusLengthMean,
            na.rm = T
          ) +
            sd(subset(slopeData, week == "8")$tarsusLengthMean,
              na.rm = T
            ),
          digits = 0.1
        ),
        "mm)"
      )
    ), name = NULL
  ) +
  scale_colour_gradient2(
    low = "#19242E",
    mid = "#BBD8F0",
    high = "grey98",
    midpoint = mean(subset(slopeData, week == "8")$tarsus, na.rm = T),
    guide = NULL
  ) +
  guides(
    fill = guide_legend(override.aes = list(size = 3)),
    linetype = guide_legend(override.aes = list(size = 4))
  ) +
  theme(
    axis.text = element_text(family = "Noto Sans"),
    axis.title = element_text(family = "Noto Sans"),
    legend.text = element_text(family = "Noto Sans", size = 7),
    legend.title = element_text(family = "Noto Sans"),
    legend.position = "bottom"
  )

showtext_auto()
ggsave("./plots/tarsusPlot8Weeks.pdf",
```

```
  tarsusPlot,
  dpi = 800, height = 7, width = 7
)
showtext_auto(enable = FALSE)

billPred <- data.frame(
  "pretreatment" = "B",
  "bill" = c(
    mean(functionModel8Weeks$data$bill, na.rm = T) -
      sd(functionModel8Weeks$data$bill, na.rm = T),
    mean(functionModel8Weeks$data$bill, na.rm = T),
    mean(functionModel8Weeks$data$bill, na.rm = T) +
      sd(functionModel8Weeks$data$bill, na.rm = T)
  ),
  "mass" = 0,
  "tarsus" = 0
) %>%
  predict(functionModel8Weeks,
    re_form = NA,
    resp = "rawSlope", newdata = .,
    summary = TRUE, robust = TRUE
  ) %>%
  as.data.frame() %>%
  mutate("slope" = Estimate +
    mean(
      subset(
        slopeData,
        week == 8 & pretreatment == "neutral"
      )$mslope,
      na.rm = TRUE
    )) %>%
  mutate("Size" = c("Small", "Average", "Large")) %>%
  dplyr::select(Size, slope, "slopeSE" = Est.Error) %>%
  slice(rep(1:n(), each = length(seq(-20, -0.1, by = 0.1)))) %>%
  mutate("Ta" = rep(seq(-20, -0.1, by = 0.1), 3)) %>%
  mutate(
    "VO2" = 1 + Ta * slope,
    "LL" = 1 + Ta * (slope - slopeSE),
    "UL" = 1 + Ta * (slope + slopeSE)
  ) %>%
  mutate(
    "Ta" = Ta + 22,
    Size = factor(Size, levels = c("Small", "Average", "Large"))
  )

billPlot <- billPred %>%
  ggplot(aes(x = Ta, y = VO2)) +
  geom_point(
    data = slopes8Weeks$data %>%
      merge(., subset(all, week == "8") %>%
        dplyr::select(ring, "bill" = billLengthMean),
      by = "ring", all.x = TRUE
      ) %>%
      mutate(Ta = Ta + 24),
    aes(x = Ta, y = VO2),
    position = jitterer, alpha = 0.5,
    pch = 21, colour = "black", fill = "transparent"
  ) +
  geom_point(
    data = slopes8Weeks$data %>%
      merge(., subset(all, week == "8") %>%
        select(ring, "bill" = billLengthMean),
      by = "ring", all.x = TRUE
      ) %>%
      mutate(Ta = Ta + 24),
    aes(x = Ta, y = VO2, colour = bill),
    position = jitterer, alpha = 0.5
```

```
  ) +
  geom_point(
    data = data.frame("Ta" = 22, "VO2" = 1),
    pch = 21, colour = "black", fill = "grey15",
    size = 3
  ) +
  geom_ribbon(aes(x = Ta, ymin = LL, ymax = UL, fill = Size),
    alpha = 0.4, colour = "black"
  ) +
  geom_line(
    aes(linetype = Size)
  ) +
  theme_classic() +
  xlab("Ambient Temperature (°C)") +
  ylab("Resting Metabolism\n(Fold Change from Thermoneutrality)") +
  scale_fill_manual(
    values = c("#194A7A", "#BBD8F0", "grey98"),
    labels = c(
      paste0(
        "Mean Bill\nLength -\n1 SD (",
        round(
          mean(subset(slopeData, week == "8")$billLengthMean,
            na.rm = T
          ) -
            sd(subset(slopeData, week == "8")$billLengthMean,
              na.rm = T
            ),
          digits = 0.1
        ),
        "mm)"
      ),
      paste0(
        "Mean Bill\nLength (",
        round(
          mean(subset(slopeData, week == "8")$billLengthMean,
            na.rm = T
          ),
          digits = 0.1
        ),
        "mm)"
      ),
      paste0(
        "Mean Bill\nLength +\n1 SD (",
        round(
          mean(subset(slopeData, week == "8")$billLengthMean,
            na.rm = T
          ) +
            sd(subset(slopeData, week == "8")$billLengthMean,
              na.rm = T
            ),
          digits = 0.1
        ),
        "mm)"
      )
    ), name = NULL
  ) +
  scale_linetype_manual(
    values = c("solid", "dashed", "dotted"),
    labels = c(
      paste0(
        "Mean Bill\nLength -\n1 SD (",
        round(
          mean(subset(slopeData, week == "8")$billLengthMean,
            na.rm = T
          ) -
            sd(subset(slopeData, week == "8")$billLengthMean,
              na.rm = T
```

```
          ),
          digits = 0.1
        ),
        "mm)"
      ),
      paste0(
        "Mean Bill\nLength (",
        round(
          mean(subset(slopeData, week == "8")$billLengthMean,
            na.rm = T
          ),
          digits = 0.1
        ),
        "mm)"
      ),
      paste0(
        "Mean Bill\nLength +\n1 SD (",
        round(
          mean(subset(slopeData, week == "8")$billLengthMean,
            na.rm = T
          ) +
            sd(subset(slopeData, week == "8")$billLengthMean,
              na.rm = T
            ),
          digits = 0.1
        ),
        "mm)"
      )
    ), name = NULL
  ) +
  scale_colour_gradient2(
    low = "#19242E",
    mid = "#BBD8F0",
    high = "grey98",
    midpoint = mean(subset(slopeData, week == "8")$bill, na.rm = T),
    guide = NULL
  ) +
  guides(
    fill = guide_legend(override.aes = list(size = 3)),
    linetype = guide_legend(override.aes = list(size = 4))
  ) +
  theme(
    axis.text = element_text(family = "Noto Sans"),
    axis.title = element_text(family = "Noto Sans"),
    legend.text = element_text(family = "Noto Sans", size = 7),
    legend.title = element_text(family = "Noto Sans"),
    legend.position = "bottom"
  )

showtext_auto()
ggsave("./plots/billPlot8Weeks.pdf",
  billPlot,
  dpi = 800, height = 7, width = 7
)
showtext_auto(enable = FALSE)


allMorphology <- (
  (p1 + massPlot) /
  (p2 + tarsusPlot) /
  (p3 + billPlot)
  ) +
  plot_annotation(tag_levels = "A")

allMorphology
```

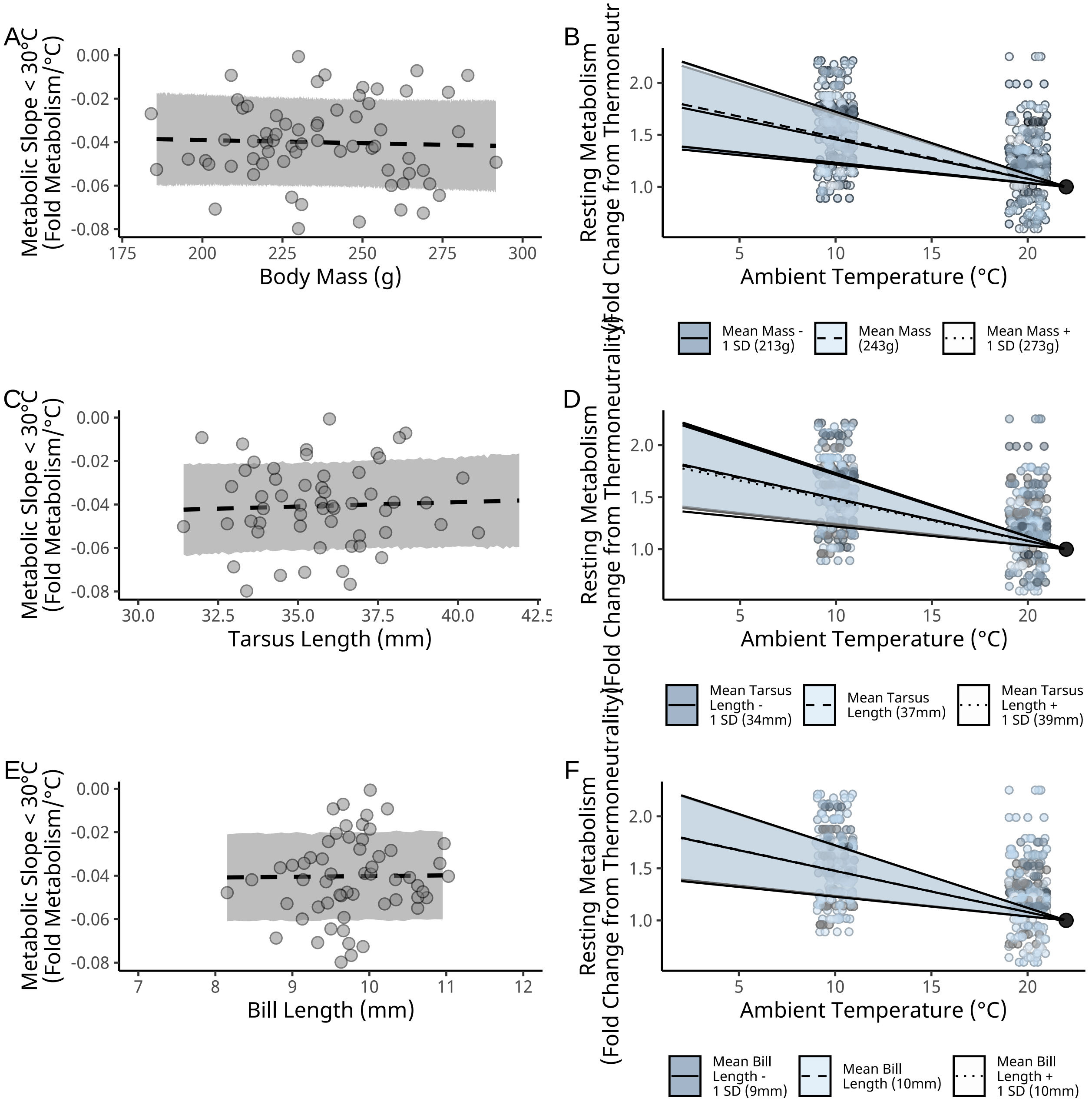

Figure 108: *Effect of morphology on metabolic slopes (fold metabolism at thermoneutrality/°C) of eight week old Japanese quail in response to cold (10°C - 30°C). Dots represent raw values, trend lines are predicted from a Bayesian path analysis, and ribbons represent one standard error around predictions. Predictions in panels B and D assume rearing at mild conditions (20°C).*

```
ggsave("./plots/morphologyEffectsResistance8Weeks_3Fold.pdf",
  allMorphology,
  dpi = 800, height = 7, width = 9
)

showtext_auto(enable = FALSE)
```

```
# As fold increase at 10°C

massPlot10 <- massPred %>%
  filter(Ta == 10) %>%
```

```
  ggplot(aes(x = Size, y = VO2)) +
  geom_point(
    data = slopes8Weeks$data %>%
      filter(Ta == -16) %>%
      merge(., subset(all, week == "8") %>%
        select(ring, mass) %>%
        distinct() %>%
        mutate(Size = ifelse(mass < (mean(mass, na.rm = T) -
          sd(mass, na.rm = T)),
        "Small",
        ifelse((mass > (mean(mass, na.rm = T) -
          sd(mass, na.rm = T))) &
          (mass < (mean(mass, na.rm = T) +
            sd(mass, na.rm = T))),
        "Average", "Large"
        )
        )),
      by = "ring", all.x = TRUE
      ) %>%
      mutate(Size = factor(Size, levels = c("Small", "Average", "Large"))),
    aes(x = Size, y = VO2, colour = Size),
    position = position_jitter(width = 0.25), alpha = 0.5
  ) +
  geom_errorbar(aes(ymin = LL, ymax = UL),
    colour = "black", width = 0.25
  ) +
  geom_point(
    size = 3, pch = 21, colour = "black",
    aes(fill = Size)
  ) +
  xlab("Body Mass") +
  ylab("Fold Metabolism at 10°C\n(From Thermoneutrality)") +
  scale_x_discrete(labels = c("Mean - 1 SD", "Mean", "Mean + 1 SD")) +
  scale_fill_manual(values = c("#DECCC1", "#855E46", "#231709")) +
  scale_colour_manual(values = c("#DECCC1", "#855E46", "#231709")) +
  theme_classic() +
  theme(
    legend.position = "none",
    axis.text = element_text(family = "Noto Sans"),
    axis.title = element_text(family = "Noto Sans")
  )

tarsusPlot10 <- tarsusPred %>%
  filter(Ta == 10) %>%
  ggplot(aes(x = Size, y = VO2)) +
  geom_point(
    data = slopes8Weeks$data %>%
      filter(Ta == -16) %>%
      merge(., subset(all, week == "8") %>%
        select(ring, "tarsus" = tarsusLengthMean) %>%
        distinct() %>%
        mutate(Size = ifelse(tarsus < (mean(tarsus, na.rm = T) -
          sd(tarsus, na.rm = T)),
        "Small",
        ifelse((tarsus > (mean(tarsus, na.rm = T) -
          sd(tarsus, na.rm = T))) &
          (tarsus < (mean(tarsus, na.rm = T) +
            sd(tarsus, na.rm = T))),
        "Average", "Large"
        )
        )),
      by = "ring", all.x = TRUE
      ) %>%
      drop_na(tarsus) %>%
      mutate(Size = factor(Size, levels = c("Small", "Average", "Large"))),
    aes(x = Size, y = VO2, colour = Size),
    position = position_jitter(width = 0.25), alpha = 0.5
```

```
  ) +
  geom_errorbar(aes(ymin = LL, ymax = UL),
    colour = "black", width = 0.25
  ) +
  geom_point(
    size = 3, pch = 21, colour = "black",
    aes(fill = Size)
  ) +
  xlab("Tarsus Length") +
  ylab("Fold Metabolism at 10°C\n(From Thermoneutrality)") +
  scale_x_discrete(labels = c(
    "Mean - 1 SD",
    "Mean",
    "Mean + 1 SD"
  )) +
  scale_fill_manual(values = c("#DECCC1", "#855E46", "#231709")) +
  scale_colour_manual(values = c("#DECCC1", "#855E46", "#231709")) +
  theme_classic() +
  theme(
    legend.position = "none",
    axis.text = element_text(family = "Noto Sans"),
    axis.title = element_text(family = "Noto Sans")
  )

billPlot10 <- billPred %>%
  filter(Ta == 10) %>%
  ggplot(aes(x = Size, y = VO2)) +
  geom_point(
    data = slopes8Weeks$data %>%
      filter(Ta == -16) %>%
      merge(., subset(all, week == "8") %>%
        select(ring, "bill" = billLengthMean) %>%
        distinct() %>%
        mutate(Size = ifelse(bill < (mean(bill, na.rm = T) -
          sd(bill, na.rm = T)),
        "Small",
        ifelse((bill > (mean(bill, na.rm = T) -
          sd(bill, na.rm = T))) &
          (bill < (mean(bill, na.rm = T) +
            sd(bill, na.rm = T))),
        "Average", "Large"
        )
        )),
      by = "ring", all.x = TRUE
      ) %>%
      drop_na(bill) %>%
      mutate(Size = factor(Size, levels = c("Small", "Average", "Large"))),
    aes(x = Size, y = VO2, colour = Size),
    position = position_jitter(width = 0.25), alpha = 0.5
  ) +
  geom_errorbar(aes(ymin = LL, ymax = UL),
    colour = "black", width = 0.25
  ) +
  geom_point(
    size = 3, pch = 21, colour = "black",
    aes(fill = Size)
  ) +
  xlab("Bill Length") +
  ylab("Fold Metabolism at 10°C\n(From Thermoneutrality)") +
  scale_x_discrete(labels = c(
    "Mean - 1 SD",
    "Mean",
    "Mean + 1 SD"
  )) +
  scale_fill_manual(values = c("#DECCC1", "#855E46", "#231709")) +
  scale_colour_manual(values = c("#DECCC1", "#855E46", "#231709")) +
  theme_classic() +
```

```
  theme(
    legend.position = "none",
    axis.text = element_text(family = "Noto Sans"),
    axis.title = element_text(family = "Noto Sans")
  )

allMorphology10 <- (p1 + massPlot10) /
  (p2 + tarsusPlot10) /
  (p3 + billPlot10) +
  plot_annotation(tag_levels = "A")

allMorphology10
```

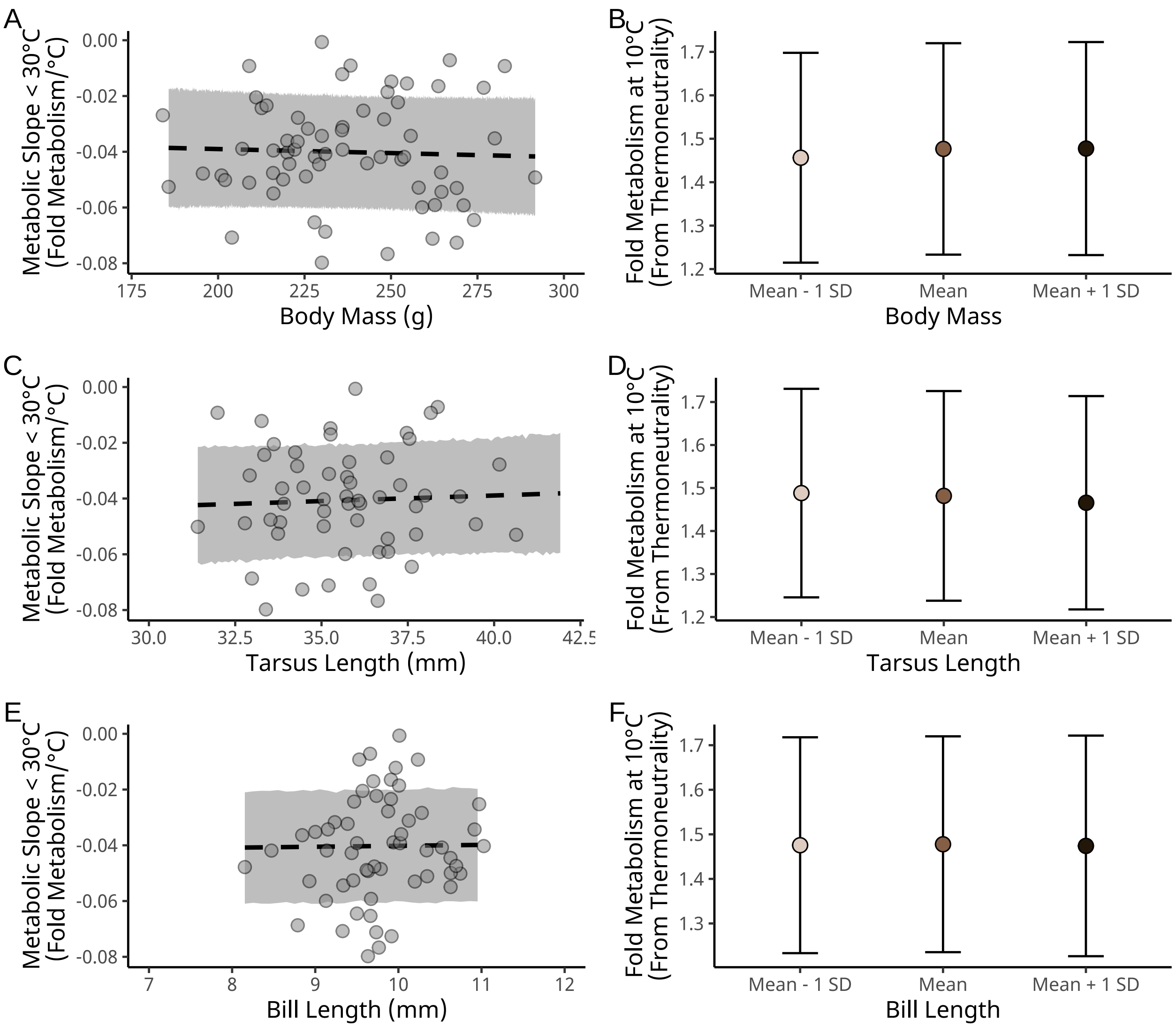

Figure 109: *Effect of morphology and the thermal environment on fold resting metabolism at 10°C in eight week old Japanese quail. Dots represent raw values, trend lines are predicted from a Bayesian path analysis, and ribbons represent one standard error around predictions.*

```
ggsave("./plots/morphologyEffectsResistance8Weeks_Fold10.pdf",
  allMorphology10,
  dpi = 800, height = 7, width = 9
)
```

Again, we visualise and summarise direct and indirect effects of thermal history (here, rearing conditions) and morphometry on metabolic slopes in our quail. To simplify comparisons, model coefficients are scaled to represent the effect that a change in one standard deviation (or categorical level) of a predictor elicits on metabolic slopes (in standard deviations).

```
scaledBetas <- as.data.frame(functionModel8Weeks) %>%
  mutate(
    b_mass_pretreatmentA = b_mass_pretreatmentA /
      sd(functionModel8Weeks$data$mass),
    b_mass_pretreatmentC = b_mass_pretreatmentC /
      sd(functionModel8Weeks$data$mass),
    b_tarsus_pretreatmentA = b_tarsus_pretreatmentA /
      sd(functionModel8Weeks$data$tarsus),
    b_tarsus_pretreatmentC = b_tarsus_pretreatmentC /
      sd(functionModel8Weeks$data$tarsus),
    b_tarsus_mass =
      (b_tarsus_mass * sd(functionModel8Weeks$data$mass)) /
        sd(functionModel8Weeks$data$tarsus),
    b_bill_pretreatmentA = b_bill_pretreatmentA /
      sd(functionModel8Weeks$data$bill),
    b_bill_pretreatmentC = b_tarsus_pretreatmentC /
      sd(functionModel8Weeks$data$bill),
    b_bill_mass =
      (b_bill_mass * sd(functionModel8Weeks$data$mass)) /
        sd(functionModel8Weeks$data$bill),
    b_rawSlope_mass =
      (b_rawSlope_mass * sd(functionModel8Weeks$data$mass)) /
        sd(functionModel8Weeks$data$rawSlope),
    b_rawSlope_tarsus =
      (b_rawSlope_tarsus * sd(functionModel8Weeks$data$tarsus)) /
        sd(functionModel8Weeks$data$rawSlope),
    b_rawSlope_bill =
      (b_rawSlope_bill * sd(functionModel8Weeks$data$bill)) /
        sd(functionModel8Weeks$data$rawSlope),
    b_rawSlope_pretreatmentA = b_rawSlope_pretreatmentA /
      sd(functionModel8Weeks$data$rawSlope),
    b_rawSlope_pretreatmentC = b_rawSlope_pretreatmentC /
      sd(functionModel8Weeks$data$rawSlope),
  )

fullEffectEightWeeksBetas <- scaledBetas %>%
  mutate("Effects" = "Direct Effects") %>%
  mutate(
    "Body Mass" = b_rawSlope_mass,
    "Tarsus Length" = b_rawSlope_tarsus,
    "Bill Length" = b_rawSlope_bill,
    "Cold Rearing\n(10°C)" = b_rawSlope_pretreatmentA,
    "Warm Rearing\n(30°C)" = b_rawSlope_pretreatmentC
  ) %>%
  dplyr::select(
    Effects, `Body Mass`, `Tarsus Length`, `Bill Length`,
    `Cold Rearing\n(10°C)`,
    `Warm Rearing\n(30°C)`
  ) %>%
  rbind(
    .,
    scaledBetas %>%
      mutate("Effects" = "Indirect Effects") %>%
      mutate(
        "Body Mass" = b_tarsus_mass *
          b_rawSlope_tarsus +
          b_bill_mass *
            b_rawSlope_bill,
        "Tarsus Length" = NA,
        "Bill Length" = NA,
        "Cold Rearing\n(10°C)" =
```

```
          b_mass_pretreatmentA * b_rawSlope_mass +
            b_tarsus_pretreatmentA * b_rawSlope_tarsus +
            b_tarsus_pretreatmentA * b_rawSlope_bill +
            b_mass_pretreatmentA * b_tarsus_mass *
              b_rawSlope_tarsus +
            b_mass_pretreatmentA * b_bill_mass *
              b_rawSlope_bill,
        "Warm Rearing\n(30°C)" =
          b_mass_pretreatmentC * b_rawSlope_mass +
            b_tarsus_pretreatmentC * b_rawSlope_tarsus +
            b_tarsus_pretreatmentC * b_rawSlope_bill +
            b_mass_pretreatmentC * b_tarsus_mass *
              b_rawSlope_tarsus +
            b_mass_pretreatmentC * b_bill_mass *
              b_rawSlope_bill
    ) %>%
    dplyr::select(
      Effects, `Body Mass`, `Tarsus Length`, `Bill Length`,
      `Cold Rearing\n(10°C)`, `Warm Rearing\n(30°C)`
    )
) %>%
rbind(., scaledBetas %>%
  mutate("Effects" = "Total Effects") %>%
  mutate(
    "Body Mass" =
      b_rawSlope_mass +
        b_tarsus_mass * b_rawSlope_tarsus +
        b_bill_mass * b_rawSlope_bill,
    "Tarsus Length" =
      b_rawSlope_tarsus,
    "Bill Length" =
      b_rawSlope_bill,
    "Cold Rearing\n(10°C)" =
      b_rawSlope_pretreatmentA +
        b_mass_pretreatmentA *
          b_rawSlope_mass +
        b_tarsus_pretreatmentA *
          b_rawSlope_tarsus +
        b_bill_pretreatmentA *
          b_rawSlope_bill +
        b_mass_pretreatmentA *
          b_tarsus_mass * b_rawSlope_tarsus +
        b_mass_pretreatmentA *
          b_bill_mass * b_rawSlope_bill,
    "Warm Rearing\n(30°C)" =
      b_rawSlope_pretreatmentC +
        b_mass_pretreatmentC *
          b_rawSlope_mass +
        b_tarsus_pretreatmentC *
          b_rawSlope_tarsus +
        b_bill_pretreatmentC *
          b_rawSlope_bill +
        b_mass_pretreatmentC *
          b_tarsus_mass * b_rawSlope_tarsus +
        b_mass_pretreatmentC *
          b_bill_mass * b_rawSlope_bill,
  ) %>%
  dplyr::select(
    Effects, `Body Mass`, `Tarsus Length`, `Bill Length`,
    `Cold Rearing\n(10°C)`, `Warm Rearing\n(30°C)`
  )) %>%
pivot_longer(c(-Effects), names_to = "var", values_to = "values") %>%
mutate(var = factor(var,
  levels = c(
    "Body Mass",
    "Tarsus Length",
    "Bill Length",
```

```
      "Warm Rearing\n(30°C)",
      "Cold Rearing\n(10°C)"
    )
  ))

fullEffectEightWeeks <- fullEffectEightWeeksBetas %>%
  ggplot(aes(x = values, y = var, fill = var)) +
  facet_wrap(~Effects) +
  stat_halfeye(normalize = "xy", colour = "black", alpha = 0.7) +
  geom_vline(xintercept = 0, linetype = "dashed",
             colour = "black") +
  xlab(".                   Effect on Metabolic Slope (standard deviations)") +
  scale_fill_manual(values = c("black", "grey40", "grey80", "#CD5C5C", "#7BB4E3")) +
  theme_classic() +
  theme(
    legend.position = "none", axis.title.y = element_blank(),
    axis.text.y = element_text(size = 11, colour = "black",
                               family = "Noto Sans"),
    axis.title.x = element_text(family = "Noto Sans", hjust = -0.005),
    axis.text.x = element_text(family = "Noto Sans")
  )

showtext_auto(enable = TRUE)

fullEffectEightWeeks
```

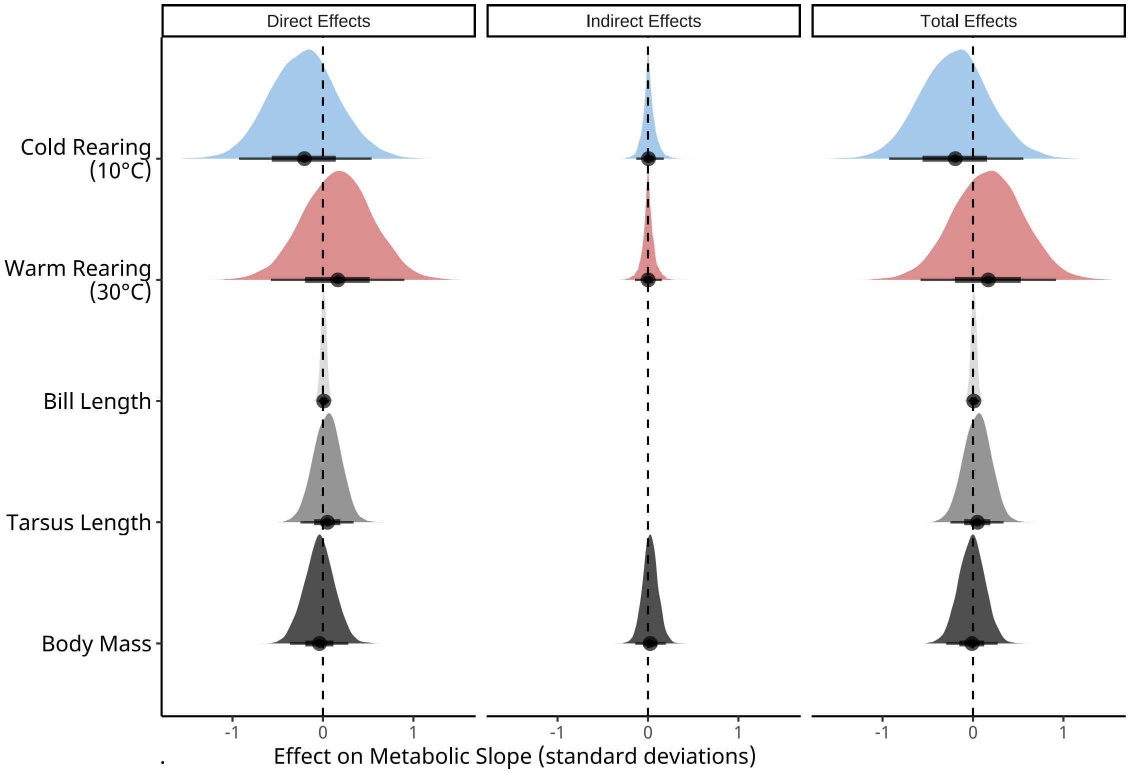

Figure 110: *Effects of morphology (body mass, g, and tarsus length, mm) on metabolic slopes (in standard deviations) at ambient temperatures below thermoneutrality (<30°C) in eight week old Japanese quail. Distributions represent those derived from posteriors of a Bayesian path analysis.*

```
ggsave("./plots/indirectEffectsOnResistance8Weeks_3ScaledFold.pdf",
       fullEffectEightWeeks,
  dpi = 800, width = 8, height = 6
)
```

```
showtext_auto(enable = FALSE)

# Summarising results

caption <- paste0(
  "Direct, indirect, and total effects of morphology and ",
  "rearing temperature on metabolic slope ",
  "(fold metabolism at thermoneutrality/°C) ",
  "of eight week old Japanese quail. Effects are derived from ",
  "a Bayesian path analysis and represent those predicted for a ",
  "change in one standard deviation (or categorical level) of ",
  "a given predictor on the standard deviation of metabolic ",
  "slopes. Estimates indicate posterior medians and credible ",
  "intervels (CIs) indicate quantile intervals."
)

week8ResultsScaled <-
  fullEffectEightWeeksBetas %>%
  filter(!is.na(values) & !is.nan(values)) %>%
  group_by(Effects, var) %>%
  summarise(
    "Estimate" = median(values),
    "50\\% CIs" = paste0(
      "[",
      round(
        quantile(values, probs = 0.1, type = 8),
        digits = 4
      ),
      ", ",
      round(
        quantile(values, probs = 0.9, type = 8),
        digits = 4
      ),
      "]"
    ),
    "95\\% CIs" = paste0(
      "[",
      round(
        quantile(values, probs = 0.025, type = 8),
        digits = 4
      ),
      ", ",
      round(
        quantile(values, probs = 0.975, type = 8),
        digits = 4
      ),
      "]"
    )
  ) %>%
  mutate("Effects" = gsub("[[:space:]].*", "", Effects)) %>%
  dplyr::select(
    "Predictor" = "var", "Effect Level" = "Effects",
    Estimate, `50\\% CIs`, `95\\% CIs`
  ) %>%
  arrange(Predictor, `Effect Level`) %>%
  kbl(.,
    longtable = T, booktabs = T, format = "latex", escape = FALSE,
    caption = caption
  ) %>%
  column_spec(column = c(1:2), width = "2.2cm") %>%
  column_spec(column = c(3:10), width = "1.9cm") %>%
  kable_styling(latex_options = "striped")

week8ResultsScaled
```

Table 60: *Direct, indirect, and total effects of morphology and rearing temperature on metabolic slope (fold metabolism at thermoneutrality/°C) of eight week old Japanese quail. Effects are derived from a Bayesian path analysis and represent those predicted for a change in one standard deviation (or categorical level) of a given predictor on the standard deviation of metabolic slopes. Estimates indicate posterior medians and credible intervels (CIs) indicate quantile intervals.*

Predictor	Effect Level	Estimate	50% CIs	95% CIs
Body Mass	Direct	-0.0377293	[-0.2519, 0.1728]	[-0.3647, 0.2809]
Body Mass	Indirect	0.0250604	[-0.08, 0.1341]	[-0.1408, 0.1971]
Body Mass	Total	-0.0110589	[-0.2, 0.1712]	[-0.2966, 0.2725]
Tarsus Length	Direct	0.0499266	[-0.1466, 0.2411]	[-0.2498, 0.3391]
Tarsus Length	Total	0.0499266	[-0.1466, 0.2411]	[-0.2498, 0.3391]
Bill Length	Direct	0.0087913	[-0.0305, 0.0479]	[-0.0524, 0.0687]
Bill Length	Total	0.0087913	[-0.0305, 0.0479]	[-0.0524, 0.0687]
Warm Rearing (30°C)	Direct	0.1647997	[-0.3152, 0.6437]	[-0.576, 0.9006]
Warm Rearing (30°C)	Indirect	0.0019248	[-0.0745, 0.0815]	[-0.1435, 0.1533]
Warm Rearing (30°C)	Total	0.1693794	[-0.3183, 0.6569]	[-0.5808, 0.9191]
Cold Rearing (10°C)	Direct	-0.2049041	[-0.6811, 0.2747]	[-0.9289, 0.5387]
Cold Rearing (10°C)	Indirect	0.0058015	[-0.0715, 0.0979]	[-0.1295, 0.1758]
Cold Rearing (10°C)	Total	-0.1957164	[-0.6761, 0.2861]	[-0.9283, 0.558]

Similar to our analysis of data from three week old quail, we now repeat our model using tarsus and bill length residuals in place of tarsus and bill length as our predictor of thermal physiology in these quail. Priors from our initial analysis of metabolic slopes across eight week old individuals are retained.

```r
resModData <- filter(slopeData, week == "8") %>%
    mutate(
      massCentred = mass - mean(mass, na.rm = T),
      pretreatment = ifelse(pretreatment == "cold", "A",
        ifelse(pretreatment == "neutral", "B", "C")
      )
    ) %>%
    mutate(pretreatment = factor(pretreatment, levels = c("B", "A", "C"))) %>%
    dplyr::select(-mass) %>%
    rename("mass" = massCentred) %>%
    drop_na() %>%
    merge(., data %>%
      dplyr::select(ring, "batch" = exp) %>%
      distinct(),
    by = "ring", all.x = TRUE
    )

residTarsusModel <- brm(
  bf(tarsusLengthMean ~ mass),
  data = resModData,
  prior = c(
    set_prior("skew_normal(0, 0.25, 5)",
      class = "b"
    ),
```

```
    set_prior("normal(37, 2.5)",
      class = "Intercept"
    )
  ),
  family = "gaussian",
  iter = 50000, warmup = 10000, cores = 1,
  chains = 4, thin = 20,
  silent = TRUE, refresh = 0,
  file = "./models/_tarsusResiduals8WeeksCold.Rds"
)

residBillModel <- brm(
  bf(billLengthMean ~ mass),
  data = resModData,
  prior = c(
    set_prior("skew_normal(0, 0.25, 5)",
      class = "b"
    ),
    set_prior("normal(10, 1)",
      class = "Intercept"
    )
  ),
  family = "gaussian",
  iter = 50000, warmup = 10000, cores = 1,
  chains = 4, thin = 20,
  silent = TRUE, refresh = 0,
  file = "./models/_billResiduals8WeeksCold.Rds"
)

functionModel8WeeksResidual <- brm(
  data = resModData %>%
    mutate(
      "residualTarsus" =
        residuals(residTarsusModel)[,"Estimate"],
      "residualBill" =
        residuals(residBillModel)[,"Estimate"]
    ),
  family = "gaussian",
  bf(mass ~ pretreatment + (1 | batch)) +
    bf(residualTarsus ~ pretreatment + (1 | batch)) +
    bf(residualBill ~ pretreatment + (1 | batch)) +
    bf(rawSlope ~ mass + residualTarsus + residualBill + pretreatment +
         (1 | batch)) +
    set_rescor(rescor = FALSE),
    prior = c(
      set_prior("normal(0, 10)",
        class = "Intercept",
        resp = "mass"
      ),
      set_prior("normal(0, 25)",
        class = "b",
        coef = "pretreatmentA",
        resp = "mass"
      ),
      set_prior("normal(0, 25)",
        class = "b",
        coef = "pretreatmentC",
        resp = "mass"
      ),
      set_prior("exponential(2.5)",
        class = "sd",
        group = "batch",
        resp = "mass"
      ),
      set_prior("exponential(0.15)",
        class = "sigma",
        resp = "mass"
```

```
    ),
    set_prior("normal(0, 2.5)",
      class = "Intercept",
      resp = "residualTarsus"
    ),
    set_prior("normal(0, 2.5)",
      class = "b",
      coef = "pretreatmentA",
      resp = "residualTarsus"
    ),
    set_prior("normal(0, 2.5)",
      class = "b",
      coef = "pretreatmentC",
      resp = "residualTarsus"
    ),
    set_prior("exponential(2)",
      class = "sd",
      group = "batch",
      resp = "residualTarsus"
    ),
    set_prior("exponential(1)",
      class = "sigma",
      resp = "residualTarsus"
    ),
    set_prior("normal(0, 1)",
      class = "Intercept",
      resp = "residualBill"
    ),
    set_prior("normal(0, 0.5)",
      class = "b",
      coef = "pretreatmentA",
      resp = "residualBill"
    ),
    set_prior("normal(0, 0.5)",
      class = "b",
      coef = "pretreatmentC",
      resp = "residualBill"
    ),
    set_prior("exponential(5)",
      class = "sd",
      group = "batch",
      resp = "residualBill"
    ),
    set_prior("exponential(2.5)",
      class = "sigma",
      resp = "residualBill"
    ),
    set_prior("normal(0, 0.01)",
      class = "Intercept",
      resp = "rawSlope"
    ),
    set_prior("normal(0, 0.025)",
      class = "b",
      coef = "pretreatmentA",
      resp = "rawSlope"
    ),
    set_prior("normal(0, 0.025)",
      class = "b",
      coef = "pretreatmentC",
      resp = "rawSlope"
    ),
    set_prior("normal(0, 0.001)",
      class = "b",
      coef = "mass",
      resp = "rawSlope"
    ),
    set_prior("normal(0, 0.0025)",
```

```
        class = "b",
        coef = "residualTarsus",
        resp = "rawSlope"
      ),
      set_prior("normal(0, 0.001)",
        class = "b",
        coef = "residualBill",
        resp = "rawSlope"
      ),
      set_prior("exponential(50)",
        class = "sd",
        group = "batch",
        resp = "rawSlope"
      ),
      set_prior("exponential(10)",
        class = "sigma",
        resp = "rawSlope"
      )
    ),
  iter = 50000, warmup = 10000, cores = 4, chains = 4, thin = 20,
  control = list(adapt_delta = .96),
  silent = TRUE, refresh = 0,
  file = "./models/_eightWeekFunctionModelResidual.Rds"
)

chainCheck(functionModel8WeeksResidual)
```

```
## Rhat range: 1 - 1.001
## Neff/N range: 0.863 - 1.008
```

```
pp_check2(functionModel8WeeksResidual,
  resp = "rawSlope",
  xlab = "Metabolic Slope\n(fold Metabolism at Thermoneutrality)"
  )
```

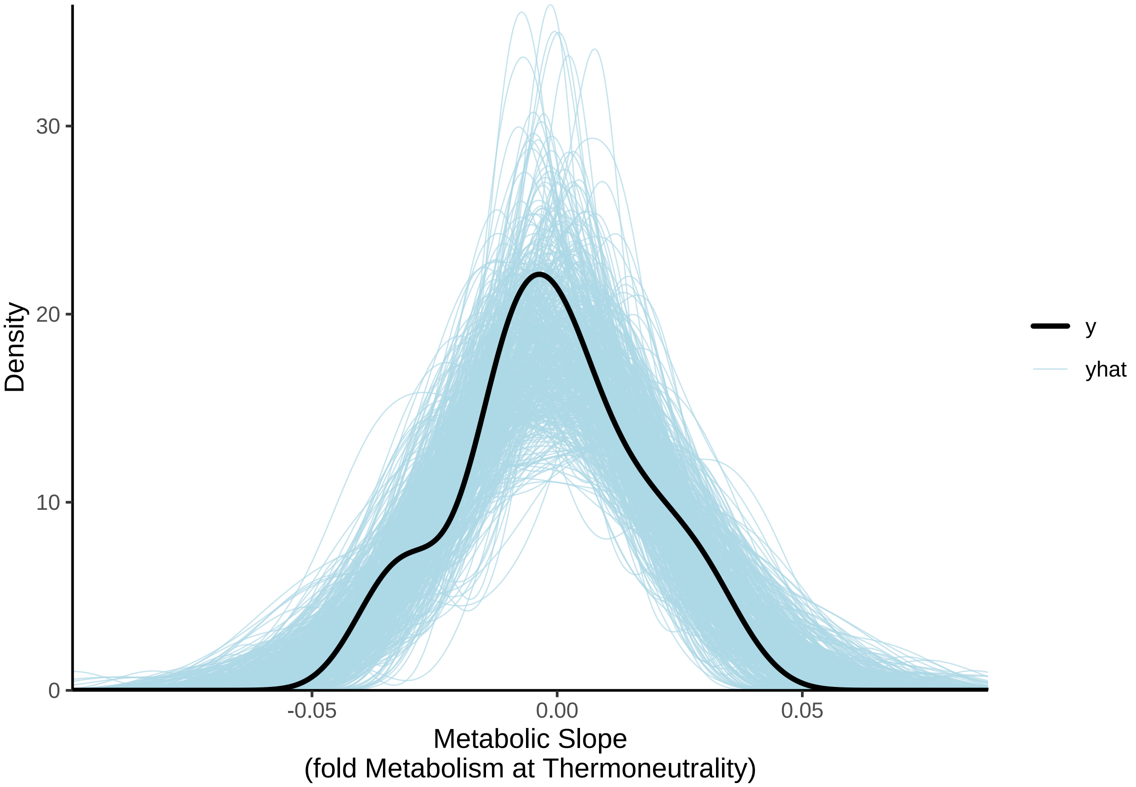

Figure 111: *Posterior predictive check for a Bayesian path analysis predicting metabolic slopes (fold metabolism at thermoneutrality/°C) of eight week old Japanese quail by morphometry and thermal history. Metabolic slopes are measured in the cold (10°C - 30°C). Light blue lines represent densities of resting metabolism values as drawn from model posteriors. The dark blue line represents the true density of resting metabolism values. Clear overlap between the dark blue and light blue lines indicates strong model fit. Morphometry measures include body mass (g) and tarsus length residuals.*

```
lbs <- c("Mass ~ Cold Rearing")
names(lbs) <- c("b_mass_pretreatmentA")
p1 <- mcmc_acf(functionModel8WeeksResidual,
  pars = "b_mass_pretreatmentA", lags = 10,
  facet_args = list(labeller = labeller(Parameter = lbs))
) +
  theme_classic()

lbs <- c("Mass ~ Warm Rearing")
names(lbs) <- c("b_mass_pretreatmentC")
p2 <- mcmc_acf(functionModel8WeeksResidual,
  pars = "b_mass_pretreatmentC", lags = 10,
  facet_args = list(labeller = labeller(Parameter = lbs))
) +
  theme_classic()

lbs <- c("Tarsus ~ Cold Rearing")
names(lbs) <- c("b_residualTarsus_pretreatmentA")
p3 <- mcmc_acf(functionModel8WeeksResidual,
  pars = "b_residualTarsus_pretreatmentA", lags = 10,
  facet_args = list(labeller = labeller(Parameter = lbs))
) +
  theme_classic()

lbs <- c("Tarsus ~ Warm Rearing")
names(lbs) <- c("b_residualTarsus_pretreatmentC")
p4 <- mcmc_acf(functionModel8WeeksResidual,
```

```
  pars = "b_residualTarsus_pretreatmentC", lags = 10,
  facet_args = list(labeller = labeller(Parameter = lbs))
) +
  theme_classic()

lbs <- c("Bill ~ Cold Rearing")
names(lbs) <- c("b_residualBill_pretreatmentA")
p5 <- mcmc_acf(functionModel8WeeksResidual,
  pars = "b_residualBill_pretreatmentA", lags = 10,
  facet_args = list(labeller = labeller(Parameter = lbs))
) +
  theme_classic()

lbs <- c("Bill ~ Warm Rearing")
names(lbs) <- c("b_residualBill_pretreatmentC")
p6 <- mcmc_acf(functionModel8WeeksResidual,
  pars = "b_residualBill_pretreatmentC", lags = 10,
  facet_args = list(labeller = labeller(Parameter = lbs))
) +
  theme_classic()

lbs <- c("Slope ~ Mass")
names(lbs) <- c("b_rawSlope_mass")
p7 <- mcmc_acf(functionModel8WeeksResidual,
  pars = "b_rawSlope_mass", lags = 10,
  facet_args = list(labeller = labeller(Parameter = lbs))
) +
  theme_classic()

lbs <- c("Slope ~ Tarsus")
names(lbs) <- c("b_rawSlope_residualTarsus")
p8 <- mcmc_acf(functionModel8WeeksResidual,
  pars = "b_rawSlope_residualTarsus", lags = 10,
  facet_args = list(labeller = labeller(Parameter = lbs))
) +
  theme_classic()

lbs <- c("Slope ~ Bill")
names(lbs) <- c("b_rawSlope_residualBill")
p9 <- mcmc_acf(functionModel8WeeksResidual,
  pars = "b_rawSlope_residualBill", lags = 10,
  facet_args = list(labeller = labeller(Parameter = lbs))
) +
  theme_classic()

lbs <- c("Slope ~\nCold Rearing")
names(lbs) <- c("b_rawSlope_pretreatmentA")
p10 <- mcmc_acf(functionModel8WeeksResidual,
  pars = "b_rawSlope_pretreatmentA", lags = 10,
  facet_args = list(labeller = labeller(Parameter = lbs))
) +
  theme_classic()

lbs <- c("Slope ~\nWarm Rearing")
names(lbs) <- c("b_rawSlope_pretreatmentC")
p11 <- mcmc_acf(functionModel8WeeksResidual,
  pars = "b_rawSlope_pretreatmentC", lags = 10,
  facet_args = list(labeller = labeller(Parameter = lbs))
) +
  theme_classic()

p12 <- functionModel8WeeksResidual$data %>%
  mutate("Res" = residuals(functionModel8WeeksResidual,
                           resp = "rawSlope",
                           robust = TRUE)[, "Estimate"]) %>%
  ggplot(aes(sample = Res)) +
  stat_qq(colour = "grey50") +
```

```
  stat_qq_line() +
  xlab("Theoretical") +
  ylab("Sample") +
  theme_classic()

(p1 + p2 + p3) /
  (p4 + p5 + p6) /
  (p7 + p8 + p9) /
  (p10 + p11 + p12)
```

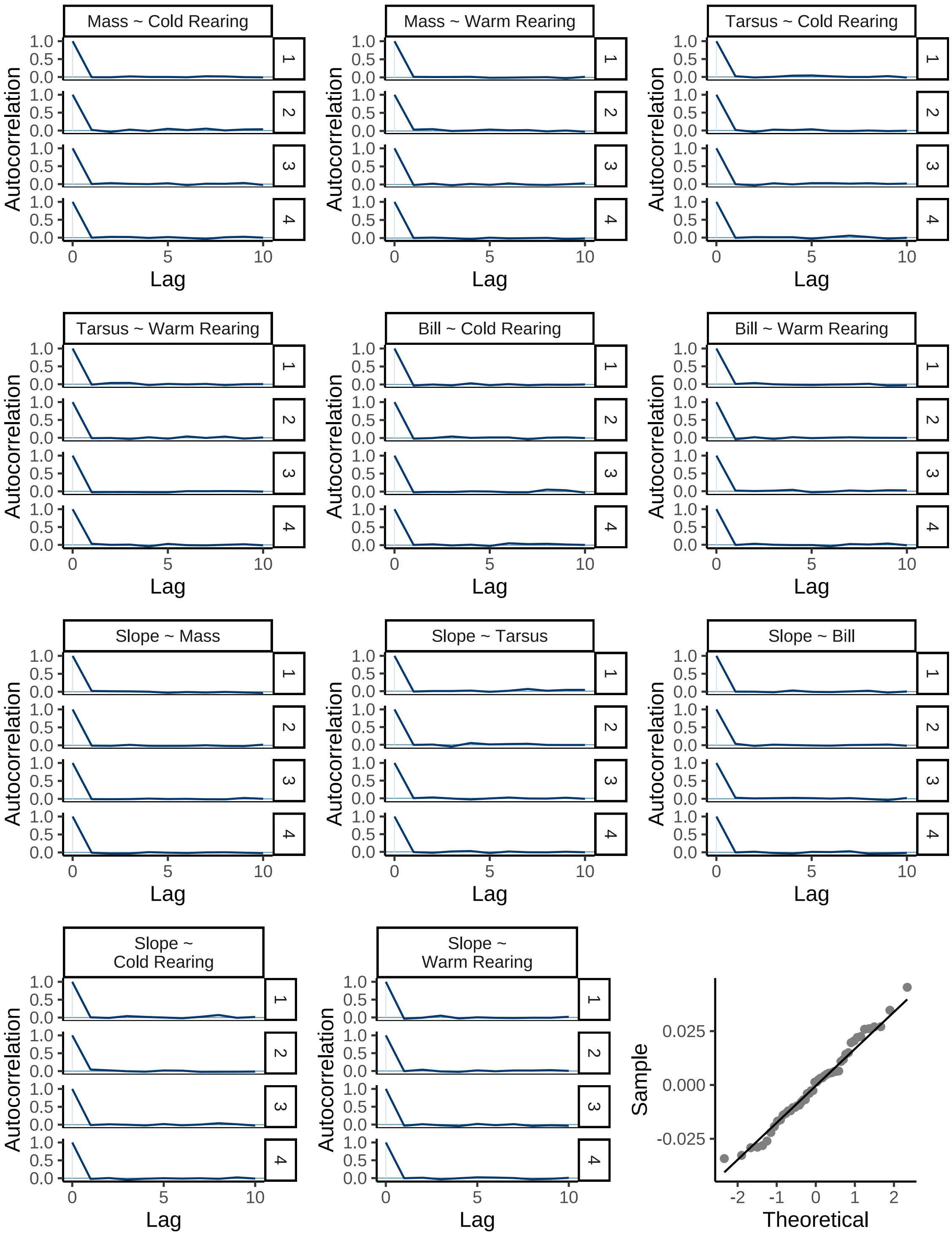

Figure 112: *Diagnostics for a Bayesian path analysis predicting metabolic slopes (fold metabolism at thermoneutrality/°C) of eight week old Japanese quail by morphometry (here, body mass [g], residual tarsus length [mm], residual bill length [mm]) and thermal history. Metabolic slopes are measured in the cold (10°C - 30°C). Autocorrelation plots display autocorrelation between adjacent Hamiltonian Monte Carlo chain draws, divided by chain. QQ-plot displays sample ordinary residuals by theoretical residual quantiles, and fitted values are displayed against true, mean-centred metabolic slope values.*

Below, we plot posterior densities per predictor.

```
# Plotting coefficients

  as.data.frame(functionModel8WeeksResidual) %>%
    dplyr::select(
      "Intercept" = b_rawSlope_Intercept,
      "Body Mass (g)" = b_rawSlope_mass,
      "Residual Tarsus\n(mm)" = b_rawSlope_residualTarsus,
      "Residual Bill\n(mm)" = b_rawSlope_residualBill,
      "Cold Rearing\n(10°C)" = b_rawSlope_pretreatmentA,
      "Warm Rearing\n(30°C)" = b_rawSlope_pretreatmentC,
      "Sigma" = sigma_rawSlope
    ) %>%
    pivot_longer(everything(), names_to = "Par",
                 values_to = "Coefs") %>%
    ggplot(aes(x = Coefs)) +
    facet_wrap(~Par, scales = "free", ncol = 2) +
    geom_density(colour = "black", alpha = 0.5, fill = "grey70") +
    geom_vline(xintercept = 0, linetype = "dashed", colour = "black") +
    ylab("Density") +
    theme_classic() +
    theme(axis.title.x = element_blank())
```

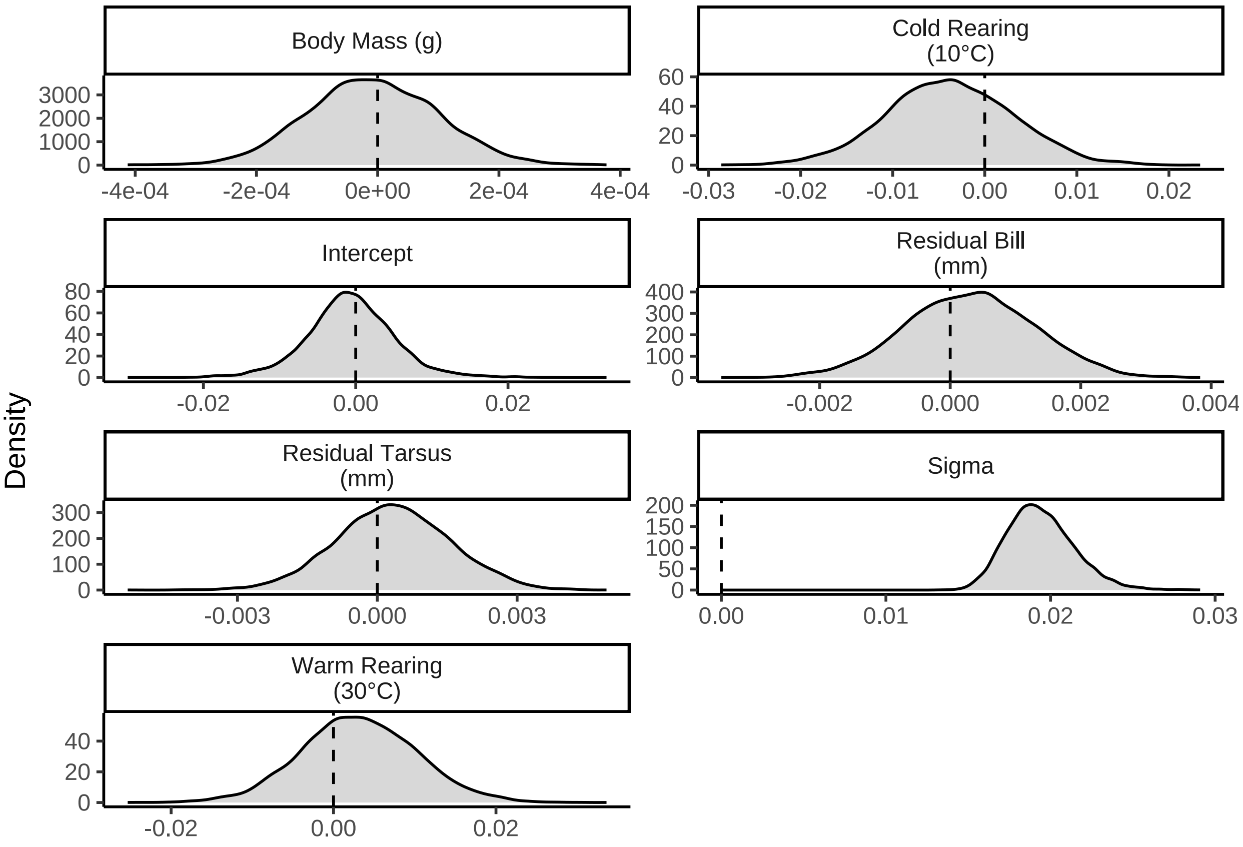

Figure 113: *Posterior densities for model coefficients derived from a Bayesian path analysis predicting metabolic slopes (fold metabolism at thermoneutrality/°C) of eight week old Japanese quail. Metabolic slopes represent those displayed in the cold (10°C - 30°C). Densities are split by their respective response values (indicated with titles). Vertical dashed lines indicate 0.*

```
ggarrange(
  as.data.frame(functionModel8WeeksResidual) %>%
```

```
    select(
      "Intercept" = b_residualTarsus_Intercept,
      "Cold Rearing" = b_mass_pretreatmentA,
      "Warm Rearing" = b_mass_pretreatmentC,
      "Sigma" = sigma_mass
    ) %>%
    pivot_longer(everything(), names_to = "Par",
                 values_to = "Coefs") %>%
    ggplot(aes(x = Coefs)) +
    facet_wrap(~Par, scales = "free") +
    geom_density(colour = "black", alpha = 0.5, fill = "grey70") +
    geom_vline(xintercept = 0, linetype = "dashed",
               colour = "black") +
    ylab("Density") +
    theme_classic() +
    theme(axis.title.x = element_blank()) +
    ggtitle("Body Mass (g)"),

  as.data.frame(functionModel8WeeksResidual) %>%
    select(
      "Intercept" = b_residualTarsus_Intercept,
      "Cold Rearing" = b_residualTarsus_pretreatmentA,
      "Warm Rearing" = b_residualTarsus_pretreatmentC,
      "Sigma" = sigma_residualTarsus
    ) %>%
    pivot_longer(everything(), names_to = "Par",
                 values_to = "Coefs") %>%
    ggplot(aes(x = Coefs)) +
    facet_wrap(~Par, scales = "free") +
    geom_density(colour = "black", alpha = 0.5, fill = "grey70") +
    geom_vline(xintercept = 0, linetype = "dashed",
               colour = "black") +
    ylab("Density") +
    theme_classic() +
    theme(axis.title.x = element_blank()) +
    ggtitle("Residual Tarsus Length (mm)"),

    as.data.frame(functionModel8WeeksResidual) %>%
    select(
      "Intercept" = b_residualBill_Intercept,
      "Cold Rearing" = b_residualBill_pretreatmentA,
      "Warm Rearing" = b_residualBill_pretreatmentC,
      "Sigma" = sigma_residualBill
    ) %>%
    pivot_longer(everything(), names_to = "Par",
                 values_to = "Coefs") %>%
    ggplot(aes(x = Coefs)) +
    facet_wrap(~Par, scales = "free") +
    geom_density(colour = "black", alpha = 0.5, fill = "grey70") +
    geom_vline(xintercept = 0, linetype = "dashed",
               colour = "black") +
    ylab("Density") +
    theme_classic() +
    theme(axis.title.x = element_blank()) +
    ggtitle("Residual Bill Length (mm)"),
  ncol = 1
)
```

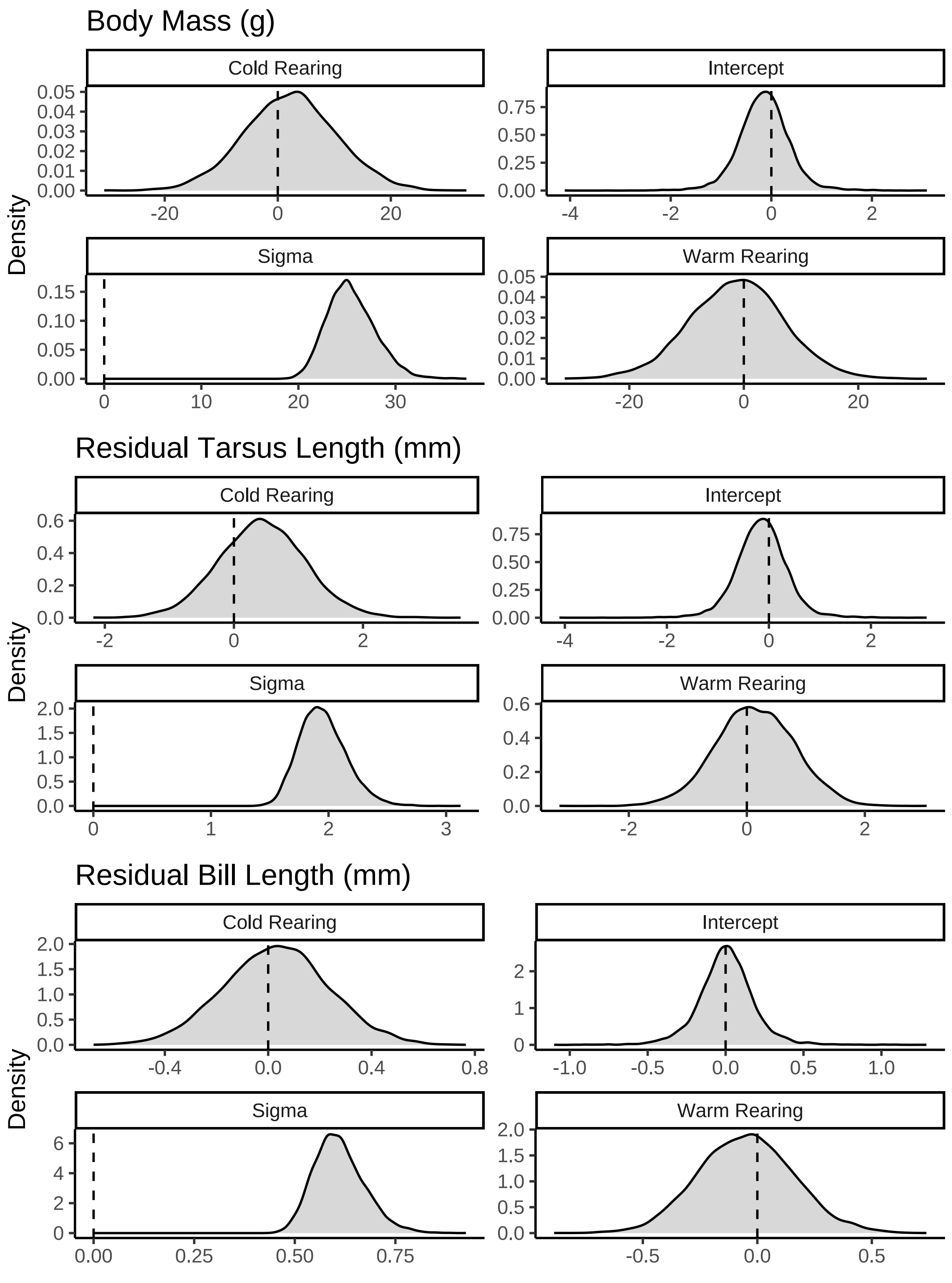

Figure 114: *Continued. Posterior densities for model coefficients derived from a Bayesian path analysis predicting metabolic slopes (fold metabolism at thermoneutrality/°C) of eight week old Japanese quail. Densities are split by their respective response values (indicated with titles). Vertical dashed lines indicate 0.*

```
# Summarising model
```

```
caption <- paste0(
  "Results of a Bayesian path analysis ",
  "predicting metabolic slopes (fold metabolism  ",
  "at thermoneutrality/°C) ",
  "of eight week old Japanese quail by morphology and ",
  "thermal history (here, rearing temperature). ",
  "Tarsus length and bill length are relativised by body mass",
  "(ordinary residuals) as estimated from a Bayesian ",
  "linear model. Metabolic slopes are measured in the cold ",
  "(10°C - 30°C). Credible intervals are quantile ",
  "intervals. BF indicates ",
  "Bayes Factors for each model parameter."
)

functionModel8WeeksResidualTable <-
  as.data.frame(functionModel8WeeksResidual) %>%
  summarise_all(., .funs = median) %>%
  pivot_longer(everything(),
    names_to = "Parameter",
    values_to = "Estimate"
  ) %>%
  merge(., quantileCIs(functionModel8WeeksResidual, cis = c(50, 95)),
    by = "Parameter", all.x = TRUE
  ) %>%
  filter(grepl("b_|sd_", Parameter)) %>%
  rowwise() %>%
  mutate("BF" = ifelse(Estimate < 0,
    (2 * mean(as.data.frame(
      functionModel8WeeksResidual
    )[, Parameter] <= 0)) /
      (2 * mean(as.data.frame(
        functionModel8WeeksResidual
      )[, Parameter] >= 0)),
    (2 * mean(as.data.frame(
      functionModel8WeeksResidual
    )[, Parameter] >= 0)) /
      (2 * mean(as.data.frame(
        functionModel8WeeksResidual
      )[, Parameter] <= 0))
  )) %>%
  ungroup() %>%
  mutate(
    "Estimate" = round(Estimate, digits = 4),
    "BF" = round(BF, digits = 4),
    "N" = nrow(functionModel8WeeksResidual$data)
  ) %>%
  mutate("Parameter" = ifelse(grepl("b_", Parameter),
    gsub("b_", "", Parameter),
    gsub(
      "Intercept", "batch",
      gsub(".*__", "", Parameter)
    )
  )) %>%
  mutate(
    "Response" = gsub("_.*", "", Parameter),
    "Parameter" = gsub(".*_", "", Parameter)
  ) %>%
  merge(., tribble(
    ~Response, ~response, ~level,
    "mass", "Body Mass (g)", "A",
    "rawSlope",
    "Metabolic Slope", "D",
    "residualTarsus", "Residual Tarsus Length (mm)", "B",
    "residualBill", "Residual Bill Length (mm)", "C"
  ),
  by = "Response"
  ) %>%
```

```
  merge(., tribble(
    ~Parameter, ~parameter, ~number,
    "Intercept", "Intercept", "1",
    "mass", "Body Mass (g)", "4",
    "residualTarsus", "Residual Tarsus Length (mm)", "5",
    "residualBill", "Residual Tarsus Length (mm)", "6",
    "pretreatmentA", "Cold Rearing", "2",
    "pretreatmentC", "Warm Rearing", "3",
    "batch", "Egg Batch [mu]", "7"
  ),
  by = "Parameter"
  ) %>%
  mutate(
    `50\\% CI` = paste0("(", paste(
      round(Low_CI_50, digits = 4),
      round(High_CI_50, digits = 4),
      sep = ", "
    ), ")"),
    `95\\% CI` = paste0("(", paste(
      round(Low_CI_95, digits = 4),
      round(High_CI_95, digits = 4),
      sep = ", "
    ), ")")
  ) %>%
  dplyr::select(-c(Low_CI_50, High_CI_50, Low_CI_95, High_CI_95)) %>%
  dplyr::select(
    "Response" = "response", "Parameter" = "parameter", N,
    Estimate, `50\\% CI`, `95\\% CI`, BF, level, number
  ) %>%
  arrange(level, number) %>%
  dplyr::select(-c(level, number)) %>%
  kbl(.,
    longtable = T, booktabs = T, format = "latex", escape = FALSE,
    caption = caption
  ) %>%
  column_spec(column = c(1:2), width = "2.2cm") %>%
  column_spec(column = c(3:10), width = "1.9cm") %>%
  kable_styling(latex_options = "striped")

functionModel8WeeksResidualTable
```

Table 61: *Results of a Bayesian path analysis predicting metabolic slopes (fold metabolism at thermoneutrality/°C) of eight week old Japanese quail by morphology and thermal history (here, rearing temperature). Tarsus length and bill length are relativised by body mass(ordinary residuals) as estimated from a Bayesian linear model. Metabolic slopes are measured in the cold (10°C - 30°C). Credible intervals are quantile intervals. BF indicates Bayes Factors for each model parameter.*

Response	Parameter	N	Estimate	50% CI	95% CI	BF
Body Mass (g)	Intercept	52	-3.9234	(-6.9339, -0.7548)	(-13.0666, 5.3277)	3.8780
Body Mass (g)	Cold Rearing	52	2.3250	(-3.2179, 7.6629)	(-13.9317, 18.2852)	1.5518
Body Mass (g)	Warm Rearing	52	-1.1161	(-6.7571, 4.219)	(-17.7008, 15.0491)	1.2504
Body Mass (g)	Egg Batch [mu]	52	0.2821	(0.1209, 0.5499)	(0.0108, 1.468)	Inf
Residual Tarsus Length (mm)	Intercept	52	-0.1449	(-0.4471, 0.1508)	(-1.1454, 0.8402)	1.6891
Residual Tarsus Length (mm)	Cold Rearing	52	0.4320	(-0.0116, 0.8808)	(-0.8689, 1.7766)	2.9024
Residual Tarsus Length (mm)	Warm Rearing	52	0.1308	(-0.3048, 0.5936)	(-1.1805, 1.4442)	1.3648
Residual Tarsus Length (mm)	Egg Batch [mu]	52	0.2509	(0.1071, 0.4885)	(0.0088, 1.3462)	Inf

Residual Bill Length (mm)	Intercept	52	0.0036	(-0.0981, 0.1046)	(-0.3614, 0.371)	1.0393
Residual Bill Length (mm)	Cold Rearing	52	0.0333	(-0.105, 0.1653)	(-0.3676, 0.4386)	1.3035
Residual Bill Length (mm)	Warm Rearing	52	-0.0481	(-0.1921, 0.0887)	(-0.4456, 0.3732)	1.4783
Residual Bill Length (mm)	Egg Batch [mu]	52	0.1160	(0.0518, 0.2173)	(0.0044, 0.5354)	Inf
Metabolic Slope	Intercept	52	-0.0008	(-0.0041, 0.0028)	(-0.0124, 0.0114)	1.2612
Metabolic Slope	Cold Rearing	52	-0.0039	(-0.0085, 9e-04)	(-0.0175, 0.0097)	2.4438
Metabolic Slope	Warm Rearing	52	0.0030	(-0.0016, 0.0079)	(-0.0106, 0.0174)	2.0211
Metabolic Slope	Body Mass (g)	52	0.0000	(-1e-04, 1e-04)	(-2e-04, 2e-04)	1.1581
Metabolic Slope	Residual Tarsus Length (mm)	52	0.0003	(-5e-04, 0.0012)	(-0.002, 0.0027)	1.5773
Metabolic Slope	Residual Tarsus Length (mm)	52	0.0003	(-4e-04, 0.001)	(-0.0016, 0.0022)	1.6247
Metabolic Slope	Egg Batch [mu]	52	0.0056	(0.0024, 0.0109)	(2e-04, 0.0326)	Inf

Testing effects of morphometry on metabolic slopes in the heat

Here, we evaluate the degree to which body size and appendage length (i.e. tarsus length and bill length) influence metabolic responses to heat in our captive reared Japanese quail. To achieve this end, we first calculate metabolic slopes displayed in response to a heat challenge (30°C - 40°C) on an individual basis. These slopes are then regressed against morphometry, while controlling for thermal history (i.e. our experimental rearing treatment). Contrasting our approach used to quantify metabolic slopes in the cold, slopes are here calculated manually as fold increase in resting metabolism between 30°C and 40°C divided by 10°C. After calculation, data are visually inspected for erroneous values before proceeding to any further analysis.

Modelling for juveniles

```
# Beginning with metabolic data from birds three weeks of age
# Subsetting data to include only metabolism measurements
# at and above 30°C.

birdVector <- merge(
  all %>%
    drop_na(VO2) %>%
    filter(week == 3 & Ta == 30) %>%
    select(ring) %>%
    distinct(),
  all %>%
    drop_na(VO2) %>%
    filter(week == 3 & Ta == 40) %>%
    select(ring) %>%
    distinct(),
  by = "ring", all = FALSE
) %>% pull(ring)

modDataHot <- subset(all, week == 3 & Ta > 25 &
                       ring %in% birdVector)

# Calculating metabolic slopes manually.

slopeDataHot3 <- modDataHot %>%
  dplyr::select(ring, Ta, VO2) %>%
  pivot_wider(id_cols = ring, names_from = Ta,
              values_from = VO2) %>%
```

```
  mutate(
    slope = (`40`/`30`)/10
  ) %>%
  dplyr::select(ring, slope) %>%
  merge(modDataHot %>%
    dplyr::select(
      ring, sex, exp, week, pretreatment,
      posttreatment, treatment, mass, wingLength,
      tarsusLengthMean, billLengthMean
    ) %>%
    distinct(), ., by = "ring")

# Checking for peculiar values.

slopeDataHot3 %>%
  ggplot(aes(x = 1:nrow(.), y = slope)) +
  geom_rect(
    colour = "black", fill = "grey80", alpha = 0.5,
    aes(
      xmin = -Inf, xmax = Inf,
      ymin = mean(slope, na.rm = T) -
        3.5 * sd(slope, na.rm = T),
      ymax = mean(slope, na.rm = T) +
        3.5 * sd(slope, na.rm = T)
    )
  ) +
  geom_point() +
  xlab("Sample Number") +
  ylab("Metabolic Slope\n(Fold Metabolsim at Thermoneutrality/°C)") +
  theme_classic()
```

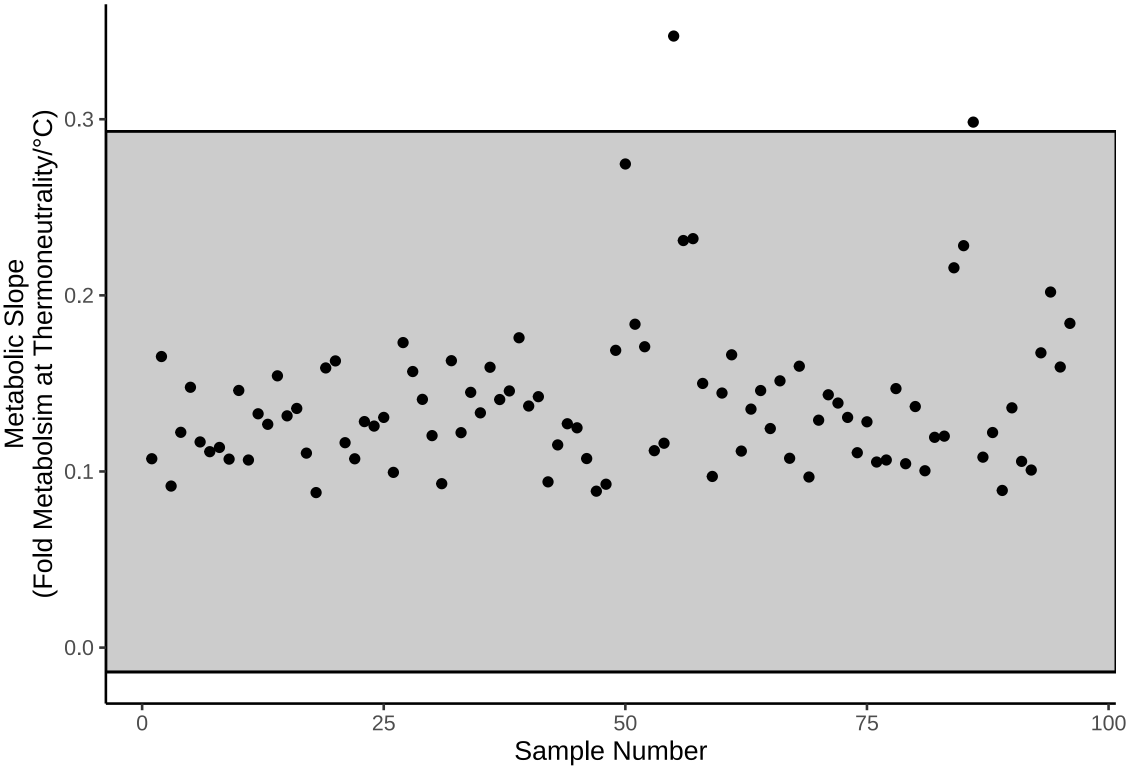

Figure 115: *Cleveland dotplot displaying metabolic slope values of three week old Japanese quail at 30°C - 40°C. Dots represent raw values and the grey rectangle represents the range covered by mean metabolic slope +/- 3.5x the standard deviation.*

Two values appears particularly high and are investigated further below.

```
caption <- paste0(
  "Potential metabolic slope outliers ",
  "among three week old Japanese quail with ",
  "metabolism measured at ambient temperatures ",
  "between 30°C and 40°C."
)

slopeDataHot3 %>%
  filter(slope > 0.29) %>%
  left_join(., modDataHot %>%
    dplyr::select(ring, Ta, VO2),
  by = "ring"
  ) %>%
  mutate(
    pretreatment = ifelse(pretreatment == "warm",
    "Warm (30°C)", ifelse(pretreatment == "cold",
      "Cold (10°C)", "Mild (20°C)"
     )),
    tarsusLengthMean = round(tarsusLengthMean, digits = 2),
    billLengthMean = round(tarsusLengthMean, digits = 2),
    VO2 = round(VO2, digits = 2),
    slope = round(slope, digits = 2)
    ) %>%
  dplyr::select(
    "Bird Identity" = ring,
    "Rearing Conditions" = pretreatment,
    "Mass (g)" = mass,
    "Tarsus Length (mm)" = tarsusLengthMean,
    "Bill Length (mm)" = billLengthMean,
    "Ambient Temperature (°C)" = Ta,
    "Resting Metabolism (mL O\\textsubscript{2}/min)" =
      VO2,
    "Metabolic Slope\n(Fold RMR at thermoneutrality/°C)" =
      slope
  ) %>%
  kbl(.,
    longtable = T, booktabs = T, format = "latex", escape = FALSE,
    caption = caption
  ) %>%
  column_spec(colum = c(1:2), width = "2cm") %>%
  column_spec(colum = c(3:10), width = "1.6cm") %>%
  kable_styling(latex_options = "striped")
```

Table 62: *Potential metabolic slope outliers among three week old Japanese quail with metabolism measured at ambient temperatures between 30°C and 40°C.*

Bird Identity	Rearing Conditions	Mass (g)	Tarsus Length (mm)	Bill Length (mm)	Ambient Temperature (°C)	Resting Metabolism (mL O_2/min)	Metabolic Slope (Fold RMR at thermoneutrality/°C)
LLW26	Warm (30°C)	155.0	36.76	36.76	30	3.77	0.35
LLW26	Warm (30°C)	155.0	36.76	36.76	40	13.09	0.35
RLG28	Warm (30°C)	124.7	36.80	36.80	30	3.26	0.30
RLG28	Warm (30°C)	124.7	36.80	36.80	40	9.72	0.30

Resting metabolism values for these individuals are quite high at 40°C and quite low and 30°C. We next plot how these values fit within the distribution of others.

```
modDataHot %>%
  ggplot(aes(x = VO2, y = Ta)) +
```

```
  geom_line(aes(group = ring),
    colour = "grey70", alpha = 0.5
  ) +
  geom_line(
    data = slopeDataHot3 %>%
      filter(ring == "LLW26") %>%
      merge(., modDataHot %>%
        dplyr::select(ring, Ta, VO2),
      by = "ring", all.x = TRUE
      ),
    colour = "black", alpha = 0.5, size = 1.5
  ) +
  geom_line(
    data = slopeDataHot3 %>%
      filter(ring == "RLG28") %>%
      merge(., modDataHot %>%
        dplyr::select(ring, Ta, VO2),
      by = "ring", all.x = TRUE
      ),
    colour = "black", alpha = 0.5, size = 1.5
  ) +
  geom_point(
    pch = 21, colour = "black",
    fill = "lightblue", alpha = 0.5
  ) +
  geom_errorbarh(
    data = modDataHot %>%
      group_by(Ta) %>%
      summarise(
        "vo2" = mean(VO2, na.rm = T),
        "SE" = sd(VO2, na.rm = T) / sqrt(n())
      ) %>%
      rename("VO2" = vo2),
    aes(xmin = VO2 - 1.96 * SE, xmax = VO2 + 1.96 * SE, y = Ta),
    colour = "black", height = 1
  ) +
  geom_point(
    data = modDataHot %>%
      group_by(Ta) %>%
      summarise("mean" = mean(VO2, na.rm = T)),
    aes(x = mean, y = Ta), pch = 21,
    colour = "black", fill = "lightblue", size = 5
  ) +
  scale_y_continuous(limits = c(28, 43), breaks = c(30, 40)) +
  xlab(TeX("$\\overset{Resting~Metabolism}{(mL~O_{2}/min)}$")) +
  ylab("Ambient Temperature (°C)") +
  theme_classic()
```

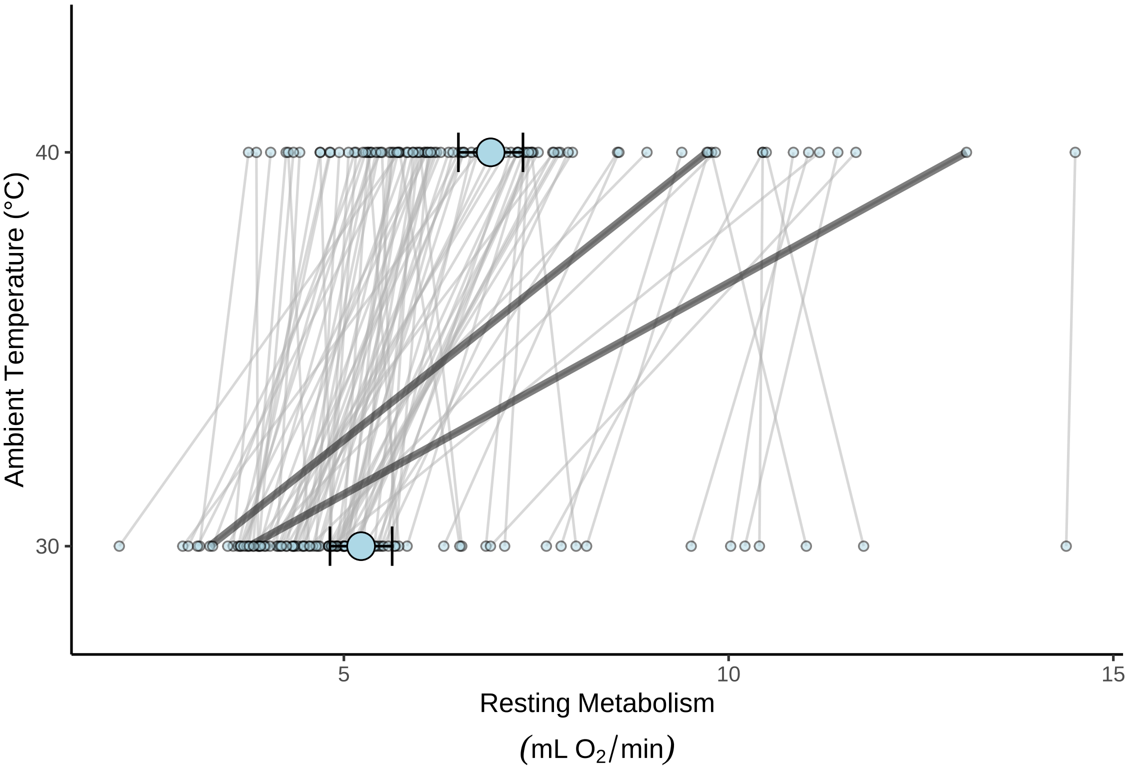

Figure 116: *Spread of resting metabolism values among three week old Japanese quail at 30°C and 40°C. Small dots represent raw values and lines connect dots for individual birds. The black line indicates possible metabolic slope (fold metabolism at thermoneutrality/°C) outliers. Large dots represent mean values at given ambient temperatures and errorbars indicate means +/- 1.96x the standard error.*

```
# Quite extreme. Checking how many standard deviations from
# the mean slope values lies.

cat(paste0(
  "Slope values for LLW26 lies ",
  round(
    (subset(slopeDataHot3, ring == "LLW26")$slope -
      mean(slopeDataHot3$slope, na.rm = T)) /
      sd(slopeDataHot3$slope, na.rm = T),
    digits = 3
  ),
  " standard deviations above the mean"
))
```

```
## Slope values for LLW26 lies 4.736 standard deviations above the mean
```

```
cat(paste0(
  "Slope values for RLG28 lies ",
  round(
    (subset(slopeDataHot3, ring == "RLG28")$slope -
      mean(slopeDataHot3$slope, na.rm = T)) /
      sd(slopeDataHot3$slope, na.rm = T),
    digits = 3
  ),
  " standard deviations above the mean"
))
```

```
## Slope values for RLG28 lies 3.621 standard deviations above the mean
```

Although these metabolic slopes are relatively large with respect to mean values, we retain them in the absence of other reasons for removal.

Below, we summarise metabolic slope values at their means, medians, and standard deviations.

```
caption = paste0("Summary of metabolic parameters estimated for ",
                 "captive-reared Japanese quail above ",
                 "thermoneutrality (30°C).")

slopeDataHot3 %>%
  group_by(week) %>%
  summarise(
    "Mean" = mean(slope, na.rm = T),
    "Median" = median(slope, na.rm = T),
    "SD" = sd(slope, na.rm = T)
  ) %>%
  mutate(
    "CV" = (SD / Mean) * 100,
    "Parameter" = "Metabolic Slope (fold metabolism at thermoneutrality/°C)"
  ) %>%
  dplyr::select(Parameter,
    "Age (weeks)" = week,
    Mean, Median, "S.D." = SD, "CV (\\%)" = CV
  ) %>%
  kbl(.,
    longtable = T, booktabs = T, format = "latex",
    caption = caption, escape = FALSE
  ) %>%
  column_spec(column = c(1:10), width = "2.1cm") %>%
  kable_styling(latex_options = "striped")
```

Table 63: *Summary of metabolic parameters estimated for captive-reared Japanese quail above thermoneutrality (30°C).*

Parameter	Age (weeks)	Mean	Median	S.D.	CV (%)
Metabolic Slope (fold metabolism at thermoneutrality/°C)	3	0.13967	0.1306982	0.0438293	31.38061

Next, distributions of, and trends in, our data are broadly explored to better inform model construction.

```
# Visualising data

p1 <- ggplot(slopeDataHot3, aes(x = slope)) +
  geom_density(colour = "black", fill = "grey50", alpha = 0.5) +
  theme_classic() +
  xlab("Metabolic Slope\n(Fold Metabolism at\nThermoneutrality/°C)") +
  ylab("Density")

p2 <- ggplot(
  modDataHot %>%
    mutate(
      "massGroup" =
        ifelse(mass <=
          mean(mass, na.rm = T) -
            sd(mass, na.rm = T),
        "Small",
        ifelse(mass >=
          mean(mass, na.rm = T) +
            sd(mass, na.rm = T),
        "Large", "Average"
        )
        )
    ) %>%
```

```
    mutate(
      massGroup =
        factor(massGroup,
          levels = c("Small", "Average", "Large")
        )
    ) %>%
    mutate(
      pretreatment =
        ifelse(pretreatment == "cold", "Cold\n(10°C)",
          ifelse(pretreatment == "neutral", "Mild\n(20°C)",
            "Warm\n(30°C)"
          )
        )
    ),
  aes(x = Ta, y = VO2, group = ring, colour = massGroup)
) +
  facet_wrap(~pretreatment) +
  geom_line(aes(size = massGroup)) +
  scale_size_manual(
    values = c("Small" = 0.5, "Average" = 1, "Large" = 2),
    name = "Relative Body\nMass",
    labels = c("< Mean - 1SD", "Mean", "> Mean + 1SD"),
  ) +
  scale_colour_manual(
    values = c(
      "Small" = "grey80",
      "Average" = "grey30",
      "Large" = "black"
    ),
    name = "Relative Body\nMass",
    labels = c(
      "< Mean - 1SD", "Mean",
      "> Mean + 1SD"
    ),
  ) +
  scale_x_continuous(breaks = c(30, 40)) +
  theme_classic() +
  theme(legend.position = "bottom") +
  xlab("Ambient Temperature (°C)") +
  ylab(TeX(
    "$\\overset{Resting~Metabolism}{(mL~O_{2}/min)}$"
  ))

p3 <- ggplot(
  modDataHot %>%
    mutate(
      "tarsusGroup" =
        ifelse(tarsusLengthMean <
          mean(tarsusLengthMean, na.rm = T) -
            sd(tarsusLengthMean, na.rm = T),
        "Small",
        ifelse(tarsusLengthMean >
          mean(tarsusLengthMean, na.rm = T) +
            sd(tarsusLengthMean, na.rm = T),
        "Large", "Average"
        )
        )
    ) %>%
    mutate(
      tarsusGroup =
        factor(tarsusGroup,
          levels = c("Small", "Average", "Large")
        )
    ) %>%
    mutate(
      pretreatment =
        ifelse(pretreatment == "cold", "Cold\n(10°C)",
```

```
          ifelse(pretreatment == "neutral", "Mild\n(20°C)",
            "Warm\n(30°C)"
          )
        )
    ) %>%
    filter(!is.na(tarsusGroup)),
  aes(x = Ta, y = VO2, group = ring, colour = tarsusGroup)
) +
  facet_wrap(~pretreatment) +
  geom_line(aes(size = tarsusGroup)) +
  scale_size_manual(
    values = c("Small" = 0.5, "Average" = 1, "Large" = 2),
    labels = c("< Mean - 1SD", "Mean", "> Mean + 1SD"),
    name = "Relative Tarsus\nLength"
  ) +
  scale_colour_manual(
    values = c(
      "Small" = "grey80",
      "Average" = "grey30",
      "Large" = "black"
    ),
    name = "Relative Tarsus\nLength",
    labels = c("< Mean - 1SD", "Mean", "> Mean + 1SD"),
  ) +
  scale_x_continuous(breaks = c(30, 40)) +
  theme_classic() +
  theme(legend.position = "right") +
  xlab("Ambient Temperature (°C)") +
  ylab(TeX(
    "$\\overset{Resting~Metabolism}{(mL~O_{2}/min)}$"
  ))

p4 <- ggplot(
  modDataHot %>%
    mutate(
      "billGroup" =
        ifelse(billLengthMean <
          mean(billLengthMean, na.rm = T) -
            sd(billLengthMean, na.rm = T),
        "Small",
        ifelse(billLengthMean >
          mean(billLengthMean, na.rm = T) +
            sd(billLengthMean, na.rm = T),
        "Large", "Average"
        )
        )
    ) %>%
    mutate(
      billGroup =
        factor(billGroup,
          levels = c("Small", "Average", "Large")
        )
    ) %>%
    mutate(
      pretreatment =
        ifelse(pretreatment == "cold", "Cold\n(10°C)",
          ifelse(pretreatment == "neutral", "Mild\n(20°C)",
            "Warm\n(30°C)"
          )
        )
    ) %>%
    filter(!is.na(billGroup)),
  aes(x = Ta, y = VO2, group = ring, colour = billGroup)
) +
  facet_wrap(~pretreatment) +
  geom_line(aes(size = billGroup)) +
  scale_size_manual(
```

```
    values = c("Small" = 0.5, "Average" = 1, "Large" = 2),
    labels = c("< Mean - 1SD", "Mean", "> Mean + 1SD"),
    name = "Relative Bill\nLength"
  ) +
  scale_colour_manual(
    values = c(
      "Small" = "grey80",
      "Average" = "grey30",
      "Large" = "black"
    ),
    name = "Relative Bill\nLength",
    labels = c("< Mean - 1SD", "Mean", "> Mean + 1SD"),
  ) +
  scale_x_continuous(breaks = c(30, 40)) +
  theme_classic() +
  theme(legend.position = "right") +
  xlab("Ambient Temperature (°C)") +
  ylab(TeX(
    "$\\overset{Resting~Metabolism}{(mL~O_{2}/min)}$"
  ))

((p1 / p2) | (p3 / p4)) +
  plot_annotation(tag_levels = "A")
```

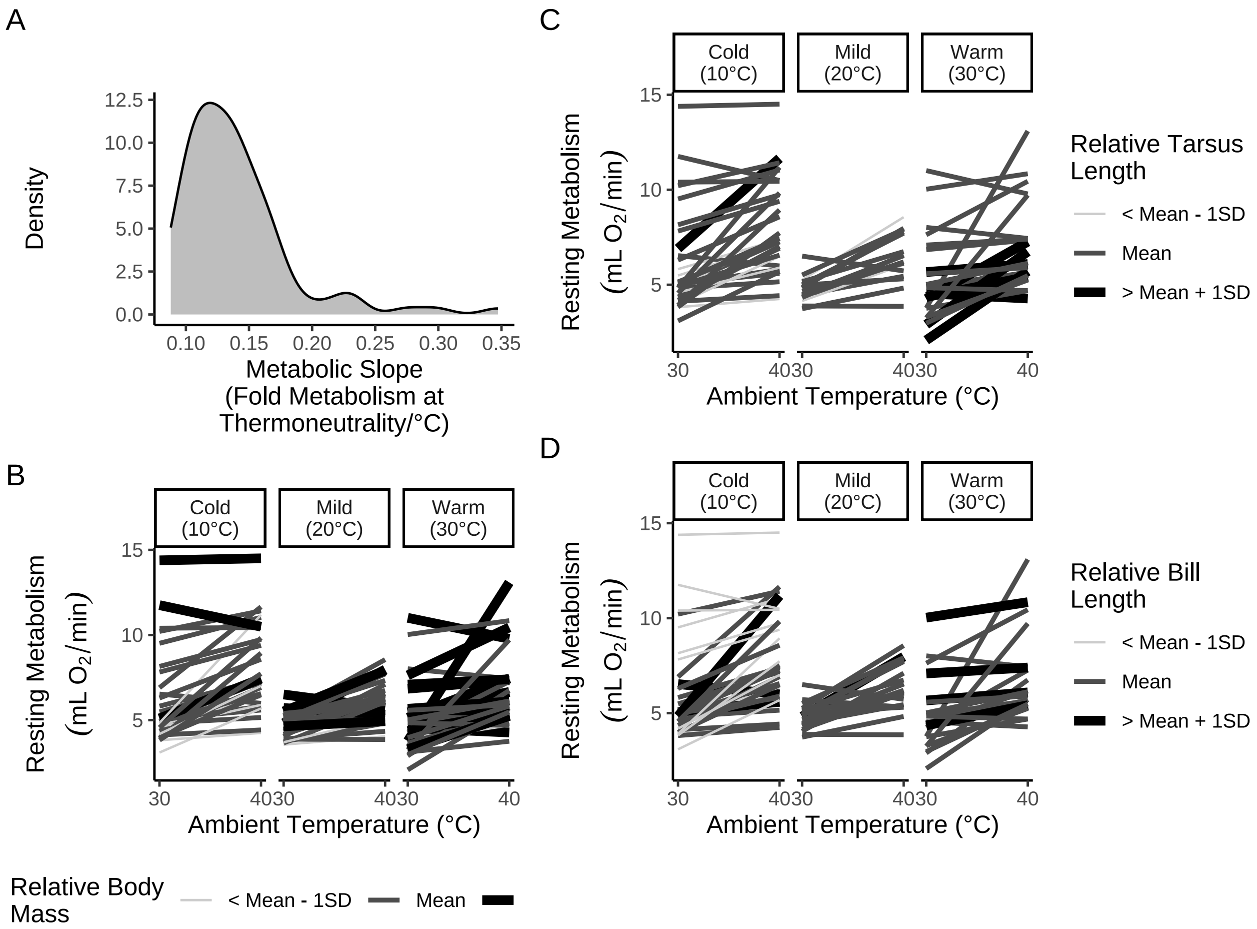

Figure 117: *Thermal physiology of three week old Japanese quail at ambient temperature within and above thermoneutrality ($\geq$ 30°C). Panel A displays density of metabolic slopes at this temperature range. Panels B and C display correlations between ambient temperature and resting metabolism by morphology. In panels B and C, each line represents one individual with line colour and line size scaled by relative size (tarsus length [mm] or body mass [g]); data is divided by rearing conditions up to three months of age.*

```
p1 <- ggplot(slopeDataHot3,
  aes(x = mass, y = slope)) +
  geom_point(pch = 21, size = 1.5,
             colour = "black", fill = "grey50") +
  geom_smooth(method = "lm", se = FALSE,
              colour = "black", linetype = "dashed") +
  xlab("Body Mass (g)") +
  ylab("Metabolic Slope\n(Fold Metabolism at\nThermoneutrality/°C)") +
  theme_classic()

p2 <- ggplot(slopeDataHot3,
             aes(x = tarsusLengthMean, y = slope)) +
  geom_point(pch = 21, size = 1.5,
             colour = "black", fill = "grey50") +
  geom_smooth(method = "lm", se = FALSE,
              colour = "black", linetype = "dashed") +
  xlab("Tarsus Length (mm)") +
  ylab("Metabolic Slope\n(Fold Metabolism at\nThermoneutrality/°C)") +
  theme_classic()

p3 <- ggplot(slopeDataHot3,
             aes(x = tarsusLengthMean, y = slope)) +
  geom_point(pch = 21, size = 1.5,
```

```r
             colour = "black", fill = "grey50") +
  geom_smooth(method = "lm", se = FALSE,
              colour = "black", linetype = "dashed") +
  xlab("Bill Length (mm)") +
  ylab("Metabolic Slope\n(Fold Metabolism at\nThermoneutrality/°C)") +
  theme_classic()

p4 <- ggplot(
  slopeDataHot3 %>%
    drop_na(tarsusLengthMean, mass) %>%
    mutate("tarsusResiduals" = residuals(
      brm(tarsusLengthMean ~ mass,
        data = .,
        prior = c(set_prior("gamma(1, 1)",
                            class = "b", coef = "mass")),
        iter = 50000, warmup = 5000, cores = 4, chains = 4,
        silent = TRUE, refresh = 0,
        file = "./models/_tarsusResidualPlotModel.Rds"
      ), robust = TRUE
    )[, "Estimate"]),
  aes(x = tarsusResiduals, y = slope)
) +
  geom_point(pch = 21, size = 1.5,
             colour = "black", fill = "grey50") +
  geom_smooth(method = "lm", se = FALSE,
              colour = "black", linetype = "dashed") +
  xlab("Residual Tarsus\nLength (mm)") +
  ylab("Metabolic Slope\n(Fold Metabolism at\nThermoneutrality/°C)") +
  theme_classic()

p5 <- ggplot(
  slopeDataHot3 %>%
    drop_na(billLengthMean, mass) %>%
    mutate("billResiduals" = residuals(
      brm(billLengthMean ~ mass,
        data = .,
        prior = c(set_prior("normal(1, 2)",
                            class = "b", coef = "mass")),
        iter = 50000, warmup = 5000, cores = 4, chains = 4,
        silent = TRUE, refresh = 0,
        file = "./models/_billResidualPlotModel.Rds"
      ), robust = TRUE
    )[, "Estimate"]),
  aes(x = billResiduals, y = slope)
) +
  geom_point(pch = 21, size = 1.5,
             colour = "black", fill = "grey50") +
  geom_smooth(method = "lm", se = FALSE,
              colour = "black", linetype = "dashed") +
  xlab("Residual Tarsus\nLength (mm)") +
  ylab("Metabolic Slope\n(Fold Metabolism at\nThermoneutrality/°C)") +
  theme_classic()

(p1/p2/p3) | (p4/p5)
```

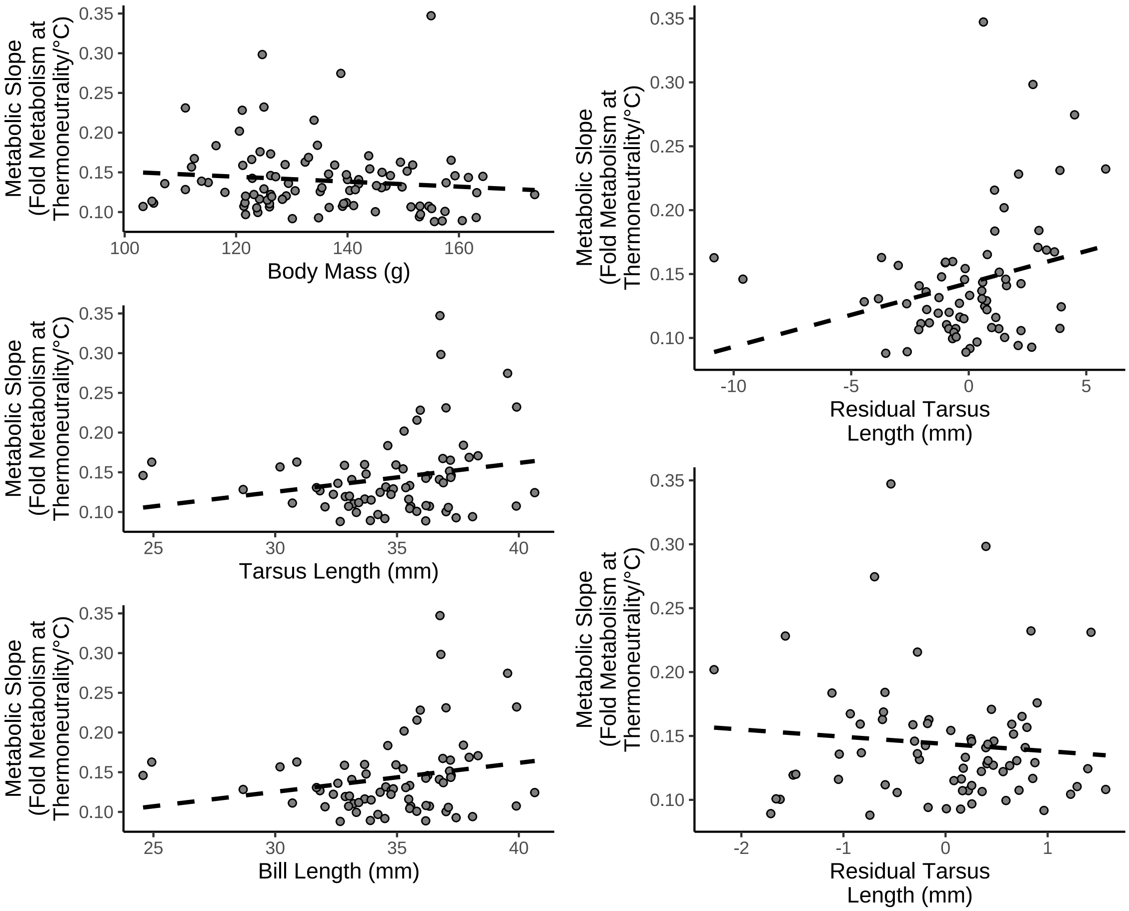

Figure 118: *Raw correlations between morphology and metabolic slopes above thermoneutrality in 3 week old Japanese quail. Metabolic slopes are measured as the fold change in resting metabolism between 30°C and 40°C. Relative tarsus and bill length indicate ordinary residuals from Bayesian regressions of tarsus length (mm) or bill length (mm) against body mass (g). Dots indicate raw values per individual and dashed lines indicate estimated relationships as predicted by the R package ggplot2 (Wickham, 2011).*

Because flow-rates differed notably between experimental batch 3 and batches 1 and 2 we visualise the spread of metabolic slope values, excluding the extreme value noted above.

```
slopeDataHot3 %>%
  rename("batch" = exp) %>%
  filter(slope < 0.3) %>%
  ggplot(aes(x = slope, fill = batch)) +
  geom_density(colour = "black", alpha = 0.5) +
  scale_fill_manual(values = c("black", "firebrick", "beige"),
                    labels = c("1", "2", "3"),
                    name = "Experimental\nBatch") +
  theme_classic() +
  xlab("Metabolic Slope\n(Fold Metabolism at Thermoneutrality") +
  ylab("Density")
```

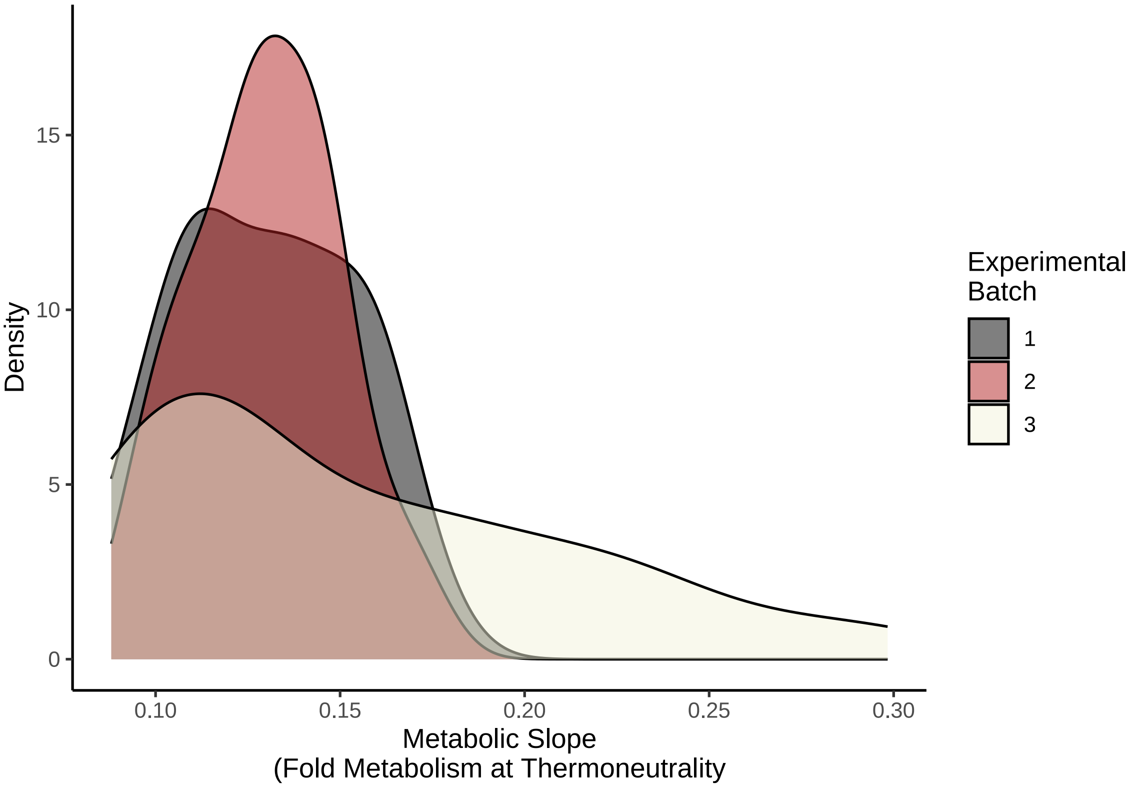

Figure 119: *Densities of metabolic slope values below 30°C from three week old Japanese quail. Densities are divided by experimental batches.*

Variance in slopes among experimental batch 3 is notably larger. For this reason, error (ϵ) around metabolic slopes must be permitted to vary by batch.

To test for an effect of morphometry on metabolic slopes of Japanese quail in response to heat, we construct a new path analysis similar to that described for responses to the cold. Models encompassed within this analysis were identical to those previously and are as follows:

$$Body\ Mass_j \sim \beta_{a0} + \beta_{a1} \cdot Cold\ Rearing_j + \beta_{a2} \cdot Warm\ Rearing_j + \mu_{0a} + \epsilon_a$$

$$Tarsus\ Length_j \sim \beta_{b0} + \beta_{b1} \cdot Cold\ Rearing_j + \beta_{b2} \cdot Warm\ Rearing_j + \beta_{b3} \cdot Body\ Mass_j + \mu_{0b} + \epsilon_b$$

$$Bill\ Length_j \sim \beta_{c0} + \beta_{c1} \cdot Cold\ Rearing_j + \beta_{c2} \cdot Warm\ Rearing_j + \beta_{c3} \cdot Body\ Mass_j + \mu_{0c} + \epsilon_c$$

and:

$$Metabolic\ Slope_j \sim \beta_{d0} + \beta_{d1} \cdot Cold\ Rearing_j + \beta_{d2} \cdot Warm\ Rearing_j + \beta_{d3} \cdot Body\ Mass_j + \\ \beta_{d4} \cdot Tarsus\ Length_j + \beta_{d5} \cdot Bill\ Length_j + \mu_{0d} + \epsilon_{dj}$$

where:

$$\epsilon_{cj} \sim e^{(\tau_0+\tau_1\cdot Batch_{2j}+\tau_2\cdot Batch_{3j})} \tag{1}$$

Variable names largely remain as described above. τ_0 indicates the natural log-transformed error term for experimental batch 1, τ_1 indicates the change in error associated with measures derived from experimental batch 2, and τ_2 indicates the change in error associated with measures derived from experimental batch 3. $Batch_{2j}$ and $Batch_{3j}$ indicate logical variables pertaining to whether a slope from individual j was derived from experimental batch 2 or 3 respectively (0 = no; 1 = yes).

Model coefficients are mean-centred to ease interpretation of model intercepts. Again, priors for this path analysis were also weakly informed by Persson et al (2022) and Saarela and Heldmaier (1987), but were broaden owing to wider uncertainty around how quail at three weeks of age respond to heat exposure. Further, standard deviations for effects of morphometry on metabolic slopes were set to approximately half the metabolic slope range divided by the morphometric measurement range. Priors were therefore as follows:

$$\beta_{a0} \sim \mathcal{N}(0,5)$$
$$\beta_{a1} \sim \mathcal{N}(0,15)$$
$$\beta_{a2} \sim \mathcal{N}(0,15)$$
$$\mu_{0a} \sim \exp(2.5)$$
$$\epsilon_a \sim \exp(0.15)$$

$$\beta_{b0} \sim \mathcal{N}(0,1.5)$$
$$\beta_{b1} \sim \mathcal{N}(0,2.5)$$
$$\beta_{b2} \sim \mathcal{N}(0,2.5)$$
$$\beta_{b3} \sim S\mathcal{N}(0,0.25,5)$$

$$\mu_{0b} \sim \exp(2)$$
$$\epsilon_b \sim \exp(1)$$

$$\beta_{c0} \sim \mathcal{N}(0,1)$$
$$\beta_{c1} \sim \mathcal{N}(0,0.5)$$
$$\beta_{c2} \sim \mathcal{N}(0,0.5)$$
$$\beta_{c3} \sim S\mathcal{N}0,0.25,5$$
$$\mu 0c \sim \exp(5)$$
$$\epsilon_c \sim \exp(2.5)$$

$$\beta_{d0} \sim \mathcal{N}(0,0.125)$$
$$\beta_{d1} \sim \mathcal{N}(0,0.125)$$
$$\beta_{d2} \sim \mathcal{N}(0,0.125)$$

$$\beta_{d3} \sim \mathcal{N}(0, 0.004)$$
$$\beta_{d4} \sim \mathcal{N}(0, 0.015)$$
$$\beta_{d5} \sim \mathcal{N}(0, 0.015)$$
$$\mu_{d0} \sim \exp(50)$$

$$\tau_0 \sim \mathcal{N}(-3, 1.5)$$
$$\tau_1 \sim \mathcal{N}(0, 0.5)$$
$$\tau_2 \sim \mathcal{N}(1, 1.5)$$

Below, we test the suitability of our priors using a prior predictive check. In the event that priors are suitable, we continue with executing our full path analysis and: (1) assessing chain convergence (here, by calculating and summarising Gelman-Rubin statistics), (2) checking for evidence of autocorrelation in chains, and (3) summarising the general fit of each model within our path analysis.

```
# Running prior predictive check

functionModel3WeeksHotPPCheck <-
  brm(
    data = slopeDataHot3 %>%
      mutate(
        mass = mass - mean(mass, na.rm = T),
        tarsus = tarsusLengthMean -
          mean(tarsusLengthMean, na.rm = T),
        bill = billLengthMean -
          mean(billLengthMean, na.rm = T),
        pretreatment = ifelse(pretreatment == "cold", "A",
          ifelse(pretreatment == "neutral", "B", "C")
        ),
        slope = slope -
          mean(slope, na.rm = T)
      ) %>%
      mutate(pretreatment = factor(pretreatment,
        levels = c("B", "A", "C")
      )) %>%
      drop_na() %>%
      merge(., data %>%
        select(ring, "batch" = exp) %>%
        distinct(),
      by = "ring", all.x = TRUE
      ),
    family = "gaussian",
    bf(mass ~ pretreatment + (1 | batch)) +
      bf(tarsus ~ mass + pretreatment + (1 | batch)) +
      bf(bill ~ mass + pretreatment + (1 | batch)) +
      bf(
        slope ~ mass + tarsus + bill + pretreatment + (1 | batch),
        sigma ~ batch
      ) +
      set_rescor(FALSE),
    prior = c(
      set_prior("normal(0, 5)",
        class = "Intercept",
        resp = "mass"
      ),
      set_prior("normal(0, 15)",
        class = "b",
        coef = "pretreatmentA",
        resp = "mass"
      ),
      set_prior("normal(0, 15)",
```

```
      class = "b",
      coef = "pretreatmentC",
      resp = "mass"
    ),
    set_prior("exponential(2.5)",
      class = "sd",
      group = "batch",
      resp = "mass"
    ),
    set_prior("exponential(0.15)",
      class = "sigma",
      resp = "mass"
    ),
    set_prior("normal(0, 2.5)",
      class = "Intercept",
      resp = "tarsus"
    ),
    set_prior("normal(0, 2.5)",
      class = "b",
      coef = "pretreatmentA",
      resp = "tarsus"
    ),
    set_prior("normal(0, 2.5)",
      class = "b",
      coef = "pretreatmentC",
      resp = "tarsus"
    ),
    set_prior("skew_normal(0, 0.25, 5)",
      class = "b",
      coef = "mass",
      resp = "tarsus"
    ),
    set_prior("exponential(2)",
      class = "sd",
      group = "batch",
      resp = "tarsus"
    ),
    set_prior("exponential(1)",
      class = "sigma",
      resp = "tarsus"
    ),
    set_prior("normal(0, 1)",
      class = "Intercept",
      resp = "bill"
    ),
    set_prior("normal(0, 0.5)",
      class = "b",
      coef = "pretreatmentA",
      resp = "bill"
    ),
    set_prior("normal(0, 0.5)",
      class = "b",
      coef = "pretreatmentC",
      resp = "bill"
    ),
    set_prior("skew_normal(0, 0.25, 5)",
      class = "b",
      coef = "mass",
      resp = "bill"
    ),
    set_prior("exponential(5)",
      class = "sd",
      group = "batch",
      resp = "bill"
    ),
    set_prior("exponential(2.5)",
      class = "sigma",
```

```
        resp = "bill"
      ),
      set_prior("normal(0, 0.125)",
        class = "Intercept",
        resp = "slope"
      ),
      set_prior("normal(0, 0.125)",
        class = "b",
        coef = "pretreatmentA",
        resp = "slope"
      ),
      set_prior("normal(0, 0.125)",
        class = "b",
        coef = "pretreatmentC",
        resp = "slope"
      ),
      set_prior("normal(0, 0.004)",
        class = "b",
        coef = "mass",
        resp = "slope"
      ),
      set_prior("normal(0, 0.015)",
        class = "b",
        coef = "tarsus",
        resp = "slope"
      ),
      set_prior("normal(0, 0.06)",
        class = "b",
        coef = "bill",
        resp = "slope"
      ),
      set_prior("exponential(50)",
        class = "sd",
        group = "batch",
        resp = "slope"
      ),
      set_prior("normal(-3, 1.5)",
        dpar = "sigma",
        class = "Intercept",
        resp = "slope"
      ),
      set_prior("normal(0, 0.5)",
        dpar = "sigma",
        class = "b",
        coef = "batchB",
        resp = "slope"
      ),
      set_prior("normal(1, 1.5)",
        dpar = "sigma",
        class = "b",
        coef = "batchC",
        resp = "slope"
      )
    ),
    iter = 50000, warmup = 10000, cores = 4, chains = 4, thin = 20,
    control = list(adapt_delta = .97, max_treedepth = 14),
    sample_prior = "only",
    silent = TRUE, refresh = 0,
    file = "./models/_threeWeekFunctionModelHotPPCheckBill.Rds"
  )

pp_check2(functionModel3WeeksHotPPCheck, resp = "slope",
   xlab = "Metabolic Slope\n(Fold Metabolism at Thermoneutrality/°C)"
   ) + xlim(c(-5, 5))
```

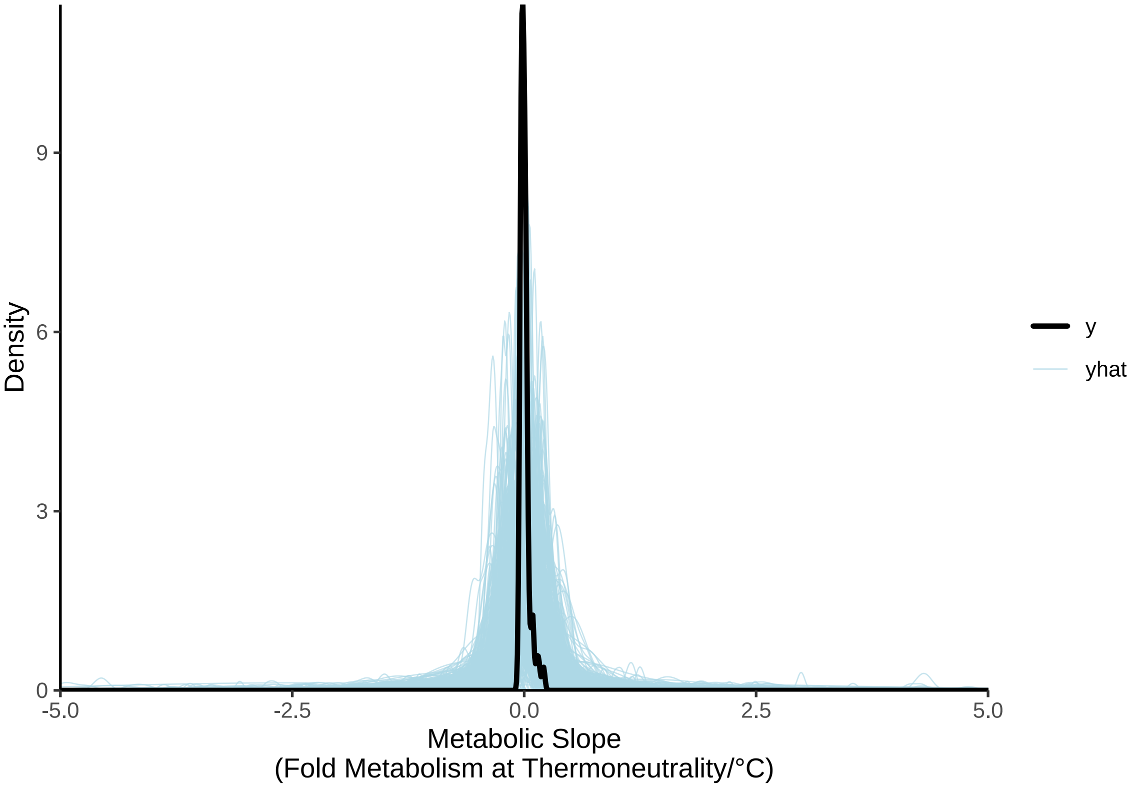

Figure 120: *Prior predictive check for a Bayesian path analysis predicting physiological responses to heat in three week old Japanese quail. Black lines represent true densities of metabolic slopes (fold metabolism at thermoneutrality/°C) while blue lines represent densities estimated from model priors alone.*

```
# Broad, but acceptable. Running full model.

functionModel3WeeksHot <-
  brm(
    data = slopeDataHot3 %>%
      mutate(
        mass = mass - mean(mass, na.rm = T),
        tarsus = tarsusLengthMean -
          mean(tarsusLengthMean, na.rm = T),
        bill = billLengthMean -
          mean(billLengthMean, na.rm = T),
        pretreatment = ifelse(pretreatment == "cold", "A",
          ifelse(pretreatment == "neutral", "B", "C")
        ),
        slope = slope -
          mean(slope, na.rm = T)
      ) %>%
      mutate(pretreatment = factor(pretreatment,
        levels = c("B", "A", "C")
      )) %>%
      drop_na() %>%
      merge(., data %>%
        dplyr::select(ring, "batch" = exp) %>%
        distinct(),
      by = "ring", all.x = TRUE
      ),
    family = "gaussian",
    bf(mass ~ pretreatment + (1 | batch)) +
      bf(tarsus ~ mass + pretreatment + (1 | batch)) +
      bf(bill ~ mass + pretreatment + (1 | batch)) +
      bf(
```

```
        slope ~ mass + tarsus + bill + pretreatment + (1 | batch),
        sigma ~ batch
      ) +
      set_rescor(FALSE),
    prior = c(
      set_prior("normal(0, 5)",
        class = "Intercept",
        resp = "mass"
      ),
      set_prior("normal(0, 15)",
        class = "b",
        coef = "pretreatmentA",
        resp = "mass"
      ),
      set_prior("normal(0, 15)",
        class = "b",
        coef = "pretreatmentC",
        resp = "mass"
      ),
      set_prior("exponential(2.5)",
        class = "sd",
        group = "batch",
        resp = "mass"
      ),
      set_prior("exponential(0.15)",
        class = "sigma",
        resp = "mass"
      ),
      set_prior("normal(0, 2.5)",
        class = "Intercept",
        resp = "tarsus"
      ),
      set_prior("normal(0, 2.5)",
        class = "b",
        coef = "pretreatmentA",
        resp = "tarsus"
      ),
      set_prior("normal(0, 2.5)",
        class = "b",
        coef = "pretreatmentC",
        resp = "tarsus"
      ),
      set_prior("skew_normal(0, 0.25, 5)",
        class = "b",
        coef = "mass",
        resp = "tarsus"
      ),
      set_prior("exponential(2)",
        class = "sd",
        group = "batch",
        resp = "tarsus"
      ),
      set_prior("exponential(1)",
        class = "sigma",
        resp = "tarsus"
      ),
      set_prior("normal(0, 1)",
        class = "Intercept",
        resp = "bill"
      ),
      set_prior("normal(0, 0.5)",
        class = "b",
        coef = "pretreatmentA",
        resp = "bill"
      ),
      set_prior("normal(0, 0.5)",
        class = "b",
```

```
        coef = "pretreatmentC",
        resp = "bill"
      ),
      set_prior("skew_normal(0, 0.25, 5)",
        class = "b",
        coef = "mass",
        resp = "bill"
      ),
      set_prior("exponential(5)",
        class = "sd",
        group = "batch",
        resp = "bill"
      ),
      set_prior("exponential(2.5)",
        class = "sigma",
        resp = "bill"
      ),
      set_prior("normal(0, 0.125)",
        class = "Intercept",
        resp = "slope"
      ),
      set_prior("normal(0, 0.125)",
        class = "b",
        coef = "pretreatmentA",
        resp = "slope"
      ),
      set_prior("normal(0, 0.125)",
        class = "b",
        coef = "pretreatmentC",
        resp = "slope"
      ),
      set_prior("normal(0, 0.004)",
        class = "b",
        coef = "mass",
        resp = "slope"
      ),
      set_prior("normal(0, 0.015)",
        class = "b",
        coef = "tarsus",
        resp = "slope"
      ),
      set_prior("normal(0, 0.06)",
        class = "b",
        coef = "bill",
        resp = "slope"
      ),
      set_prior("exponential(50)",
        class = "sd",
        group = "batch",
        resp = "slope"
      ),
      set_prior("normal(-3, 1.5)",
        dpar = "sigma",
        class = "Intercept",
        resp = "slope"
      ),
      set_prior("normal(0, 0.5)",
        dpar = "sigma",
        class = "b",
        coef = "batchB",
        resp = "slope"
      ),
      set_prior("normal(1, 1.5)",
        dpar = "sigma",
        class = "b",
        coef = "batchC",
        resp = "slope"
```

```
      )
    ),
    iter = 50000, warmup = 10000, cores = 4, chains = 4, thin = 20,
    control = list(adapt_delta = .97, max_treedepth = 14),
    silent = TRUE, refresh = 0,
    file = "./models/_threeWeekFunctionModelHot.Rds"
  )

chainCheck(functionModel3WeeksHot)
```

```
## Rhat range: 1 - 1.001
## Neff/N range: 0.866 - 1.004
```

```
# Good. Checking r2 and posterior density/fit

caption <- paste0(
  "R\\textsuperscript{2} for a Bayesian ",
  "path analysis predicting metabolic slopes ",
  "(fold metabolism at thermoneutrality; 30°C) ",
  "of three week old Japanese quail as a ",
  "function of body mass (g), tarsus length (mm)",
  "bill length (mm), and rearing ",
  "temperature. Metabolic slopes are measured above ",
  "thermoneutrality (30°C - 40°C)."
)

brms::bayes_R2(functionModel3WeeksHot,
               ndraws = 1000,
               robust = TRUE) %>%
  as.data.frame() %>%
  rownames_to_column("var") %>%
  merge(., tribble(
    ~var, ~Var,
    "R2mass", "Body Mass (g)",
    "R2tarsus", "Tarsus Length (mm)",
    "R2bill", "Bill Length (mm)",
    "R2slope", "Metabolic Slope"
  ), by = c("var")) %>%
  mutate(Var = factor(Var, levels = c(
    "Body Mass (g)", "Tarsus Length (mm)",
    "Bill Length (mm)", "Metabolic Slope"
  ))) %>%
  mutate(Estimate = round(Estimate, digits = 4),
         Est.Error = round(Est.Error, digits = 4),
         `95\\% CI` = paste0("[", round(Q2.5, digits = 4),
                             ", ",
                             round(Q97.5, digits = 4),
                             "]"
         )
         ) %>%
  dplyr::select(
    "Response" = Var, "R\\textsuperscript{2}" = Estimate,
    "Standard Error" = Est.Error, `95\\% CI`
  ) %>%
  arrange(Response) %>%
  kbl(.,
    longtable = T, booktabs = T, format = "latex",
    caption = caption, escape = FALSE
  ) %>%
  column_spec(column = c(1:10), width = "2.5cm")  %>%
  kable_styling(latex_options = "striped")
```

Table 64: *R^2 for a Bayesian path analysis predicting metabolic slopes (fold metabolism at thermoneutrality; 30°C) of three week old Japanese quail as a function of body mass (g), tarsus length (mm)bill length (mm), and rearing temperature. Metabolic slopes are measured above thermoneutrality (30°C - 40°C).*

Response	R^2	Standard Error	95% CI

Body Mass (g)	0.0994	0.0572	[0.0126, 0.2387]
Tarsus Length (mm)	0.3891	0.0797	[0.2246, 0.523]
Bill Length (mm)	0.3638	0.0816	[0.1872, 0.4848]
Metabolic Slope	0.1002	0.0512	[0.0287, 0.234]

The fit of our path analysis is next broadly assessed.

```
ggarrange(
  pp_check2(functionModel3WeeksHot,
            resp = "slope",
            xlab = paste0("Metabolic Slope\n(Fold Metabolism ",
                          "at\nThermoneutrality/°C)")
            ),

  functionModel3WeeksHot$data %>%
    mutate("Fit" = fitted(functionModel3WeeksHot,
                          resp = "slope",
                          robust = TRUE)[, "Estimate"]) %>%
    ggplot(aes(x = Fit, y = slope)) +
    geom_point() +
    geom_line(data = data.frame("Fit" = c(-0.05, 0.05),
                                "slope" = c(-0.05, 0.05))) +
    xlab(
      "Fitted Metabolic Slope\n(Fold Metabolism at\nThermoneutrality/°C)"
      ) +
    ylab(
      "True Metabolic Slope\n(Fold Metabolism at\nThermoneutrality/°C)"
      ) +
    theme_classic(),
  labels = c("A", "B")
)
```

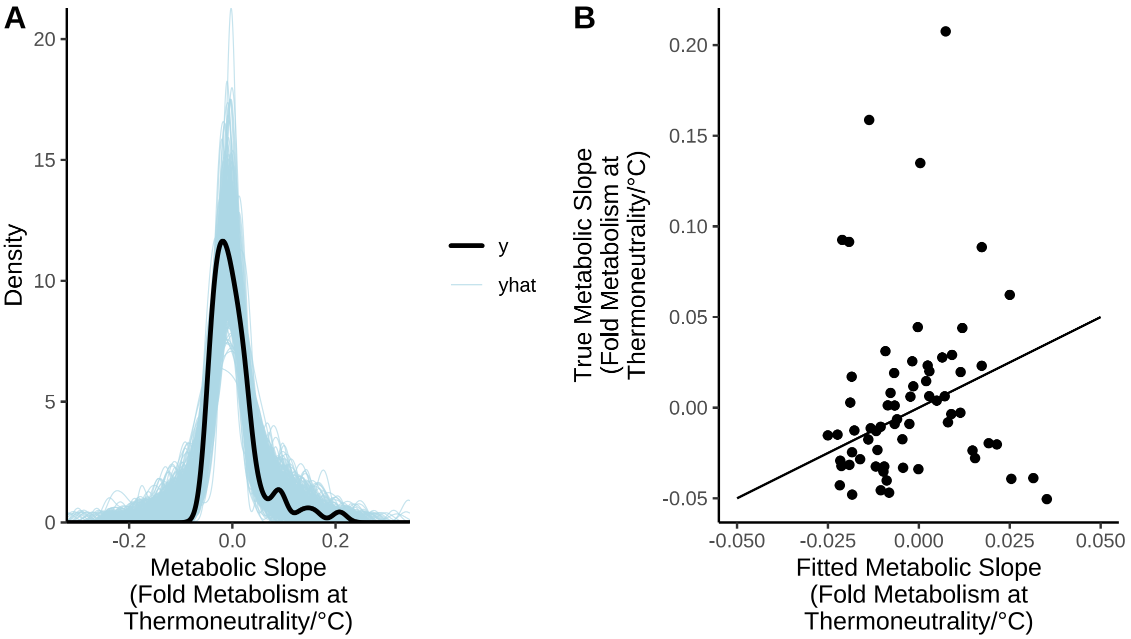

Figure 121: *Characteristics of Bayesian path analysis predicting physiological responses to heat in three week old Japanese quail. Panels A displays a posterior predictive check with the black line indicating the true density of metabolic slopes and blue lines indicating densities estimated from posterior draws. Panel B displays model fitted values by true values with dots indicating individual data points and the black line indicating a 1:1 relationship.*

Residuals of this path analysis are diagnosed for pathologies as above.

```
p1 <- functionModel3WeeksHot$data %>%
  mutate(
    "Residuals" =
      residuals(functionModel3WeeksHot,
        resp = "slope",
        robust = TRUE
      )[, "Estimate"]
  ) %>%
  ggplot(aes(sample = Residuals)) +
  stat_qq(colour = "grey50") +
  stat_qq_line() +
  xlab("Theoretical Quantiles") +
  ylab(TeX(
    "$\\overset{Sample~Quantiles}{(Slope)}$"
  )) +
  theme_classic()

p2 <- functionModel3WeeksHot$data %>%
  mutate(
    "Residuals" =
      residuals(functionModel3WeeksHot,
        resp = "slope",
        robust = TRUE
      )[, "Estimate"]
  ) %>%
  ggplot(aes(x = mass, y = Residuals)) +
  geom_point(
    pch = 21, colour = "black", size = 2,
    fill = "lightblue", alpha = 0.5
  ) +
  xlab("Body Mass (g; Mean-Centred)") +
  ylab("Metabolic Slope\nResiduals") +
  theme_classic()

p3 <- functionModel3WeeksHot$data %>%
  mutate(
    "Residuals" =
      residuals(functionModel3WeeksHot,
        resp = "slope",
        robust = TRUE
      )[, "Estimate"]
  ) %>%
  ggplot(aes(x = tarsus, y = Residuals)) +
  geom_point(
    pch = 21, colour = "black", size = 2,
    fill = "lightblue", alpha = 0.5
  ) +
  xlab("Tarsus Length (mm; Mean-Centred)") +
  ylab("Metabolic Slope\nResiduals") +
  theme_classic()

p4 <- functionModel3WeeksHot$data %>%
  mutate(
    "Residuals" =
      residuals(functionModel3WeeksHot,
        resp = "slope",
        robust = TRUE
      )[, "Estimate"]
  ) %>%
  ggplot(aes(x = bill, y = Residuals)) +
  geom_point(
    pch = 21, colour = "black", size = 2,
    fill = "lightblue", alpha = 0.5
  ) +
  xlab("Bill Length (mm; Mean-Centred)") +
```

```
  ylab("Metabolic Slope\nResiduals") +
  theme_classic()

p5 <- functionModel3WeeksHot$data %>%
  mutate(
    "Residuals" =
      residuals(functionModel3WeeksHot,
        resp = "slope",
        robust = TRUE
      )[, "Estimate"]
  ) %>%
  mutate("pretreatment" = ifelse(pretreatment == "A", "Cold (10°C)",
    ifelse(pretreatment == "B", "Mild (20°C)",
      "Warm (30°C)"
    )
  )) %>%
  ggplot(aes(x = pretreatment, y = Residuals)) +
  geom_point(
    pch = 21, colour = "black", size = 2,
    fill = "lightblue", alpha = 0.5
  ) +
  stat_summary(
    geom = "errorbar", fun.data = "mean_se",
    colour = "black", width = 0.25
  ) +
  stat_summary(
    geom = "point", fun = "mean", size = 4,
    pch = 21, colour = "black", fill = "lightblue"
  ) +
  xlab("Rearing Conditions") +
  ylab("Metabolic Slope\nResiduals") +
  theme_classic()

p6 <- functionModel3WeeksHot$data %>%
  mutate(
    "Residuals" = residuals(functionModel3WeeksHot,
      resp = "slope",
      robust = TRUE
    )[, "Estimate"],
    "Fit" = fitted(functionModel3WeeksHot,
      resp = "slope",
      robust = TRUE
    )[, "Estimate"]
  ) %>%
  ggplot(aes(x = Fit, y = Residuals)) +
  geom_point(
    pch = 21, colour = "black", size = 2,
    fill = "lightblue", alpha = 0.5
  ) +
  xlab("Fitted Metabolic Slope\n(Fold Metabolism at Thermoneutrality/°C)") +
  ylab("Metabolic Slope\nResiduals") +
  theme_classic()

(p1 + p2) / (p3 + p4) / (p5 + p6) +
  plot_annotation(tag_levels = "A")
```

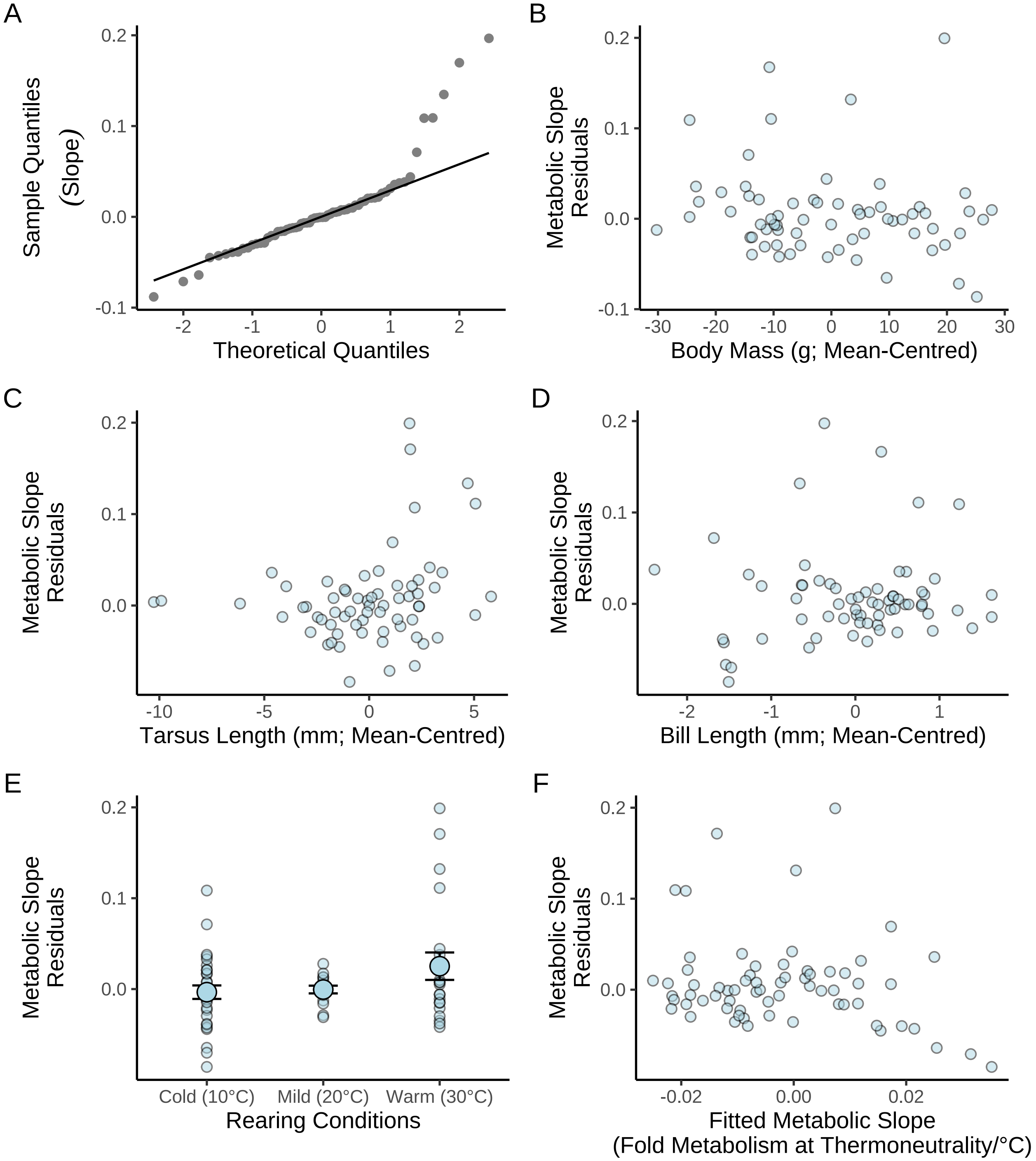

Figure 122: *Spread of ordinary residuals from Bayesian path analysis predicting physiological responses to heat in three week old Japanese quail. Quail were reared in one of three thermal conditions: (1) cold (10°C), (2) mild (20°C), or warm (30°C). Panels A displays a qq-plot of theoretical residual quantiles against their true values. Panels B to F display ordinary residuals against predictors and fitted values; dots in these panels represent values per individual.*

Evidently, there is some right skewing in our median residuals. This is arguably expected given that our slope values are manually calculated and therefore not balanced by a population-averaged response. Below, we check whether these values have undue influence on our model by first using leave-one-out ("LOO") cross-

validation via Pareto-smoothed importance sampling, then evaluating importance weights (here, Pareto K) per data point (Vehtari et al, 2017).

```
loo(functionModel3WeeksHot)$diagnostics$pareto_k %>%
  as_tibble() %>%
  ggplot(aes(x = 1:nrow(.), y = value)) +
  geom_point(pch = 21, colour = "black", fill = "grey50", size = 2) +
  geom_hline(yintercept = 0.5, colour = "black", linetype = "dashed") +
  geom_hline(yintercept = 0.7, colour = "red4", linetype = "dashed") +
  xlab("Sample") +
  ylab("Pareto K") +
  theme_classic()
```

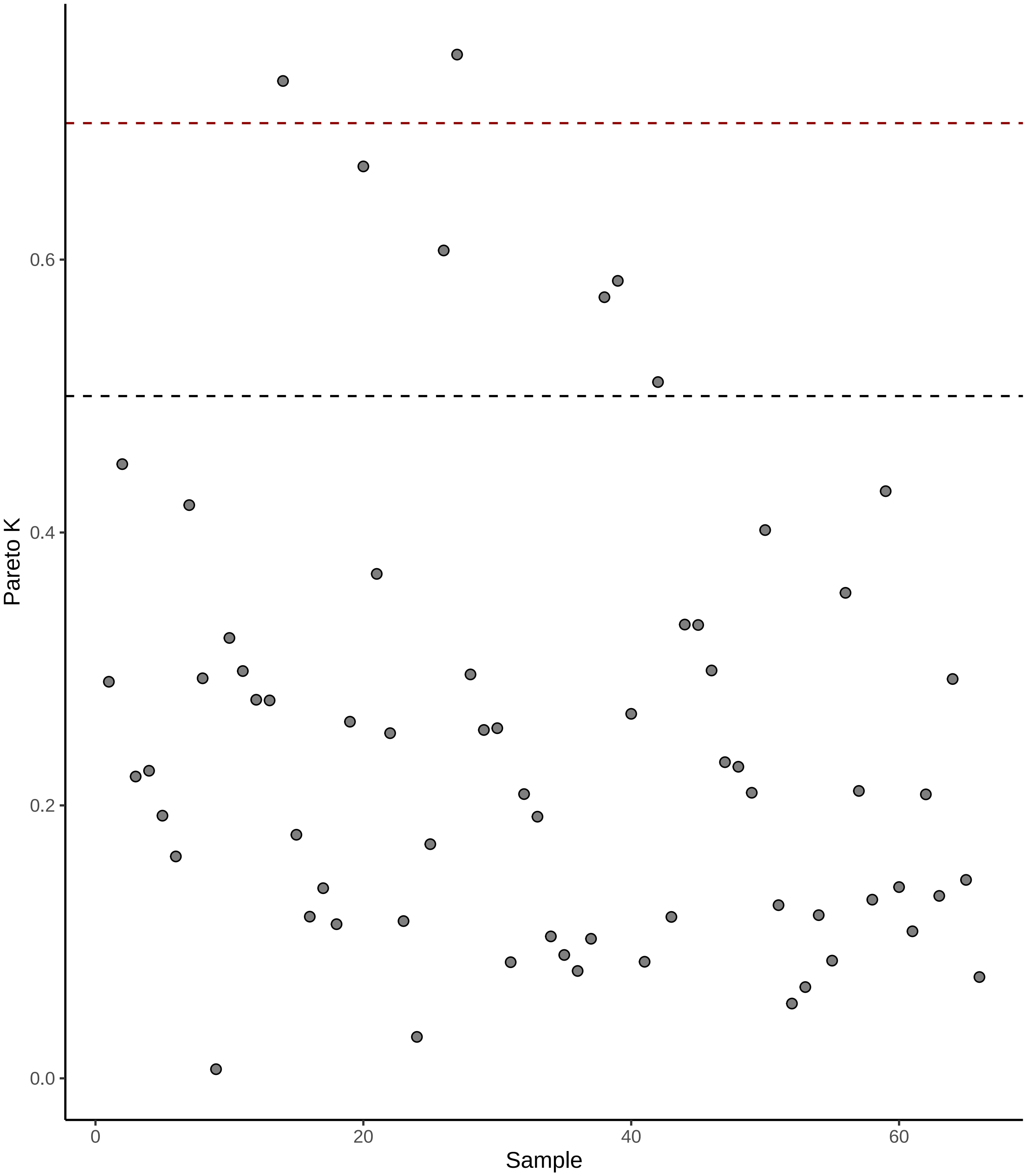

Figure 123: *Relative importance of individual metabolic slope values (dots) to outcomes of our Bayesian path analysis, as estimated using leave-one-out cross validations. Importance is measured here as Pareto K (Vehtari et al, 2017). The black, dashed horizontal line indicated a Pareto K of 0.5 and the red horizontal dashed line indicates as Pareto K of 0.7, above which, samples hold significant importance on analysis outcomes.*

A few data points are identified as potentially problematic (> 0.7; see Vehtari et al 2020). There points are examined below, along with others wiht pareto K values exceeding 0.5.

```r
functionModel3WeeksHot$data %>%
  mutate(
    "id" = 1:nrow(.),
    "paretoK" = loo(functionModel3WeeksHot)$diagnostics$pareto_k
  ) %>%
  mutate("Influence" = ifelse(paretoK > 0.5, "High", "Low")) %>%
  dplyr::select(c(id, Influence, mass, tarsus, bill, slope)) %>%
  pivot_longer(-c(id, Influence), names_to = "var", values_to = "vals") %>%
  left_join(., tribble(
    ~var, ~Var,
    "mass", "Mass (g)",
    "tarsus", "Tarsus Length (mm)",
    "bill", "Bill Length (mm)",
    "slope", "Metabolic Slope\n(fold RMR/°C)"
  ),
  by = "var"
  ) %>%
  ggplot(aes(x = vals, fill = Influence)) +
  facet_wrap(~Var, scales = "free") +
  geom_density(alpha = 0.5, colour = "black") +
  scale_fill_manual(values = c("grey20", "grey80")) +
  xlab("Measurement") +
  ylab("Density") +
  theme_classic()
```

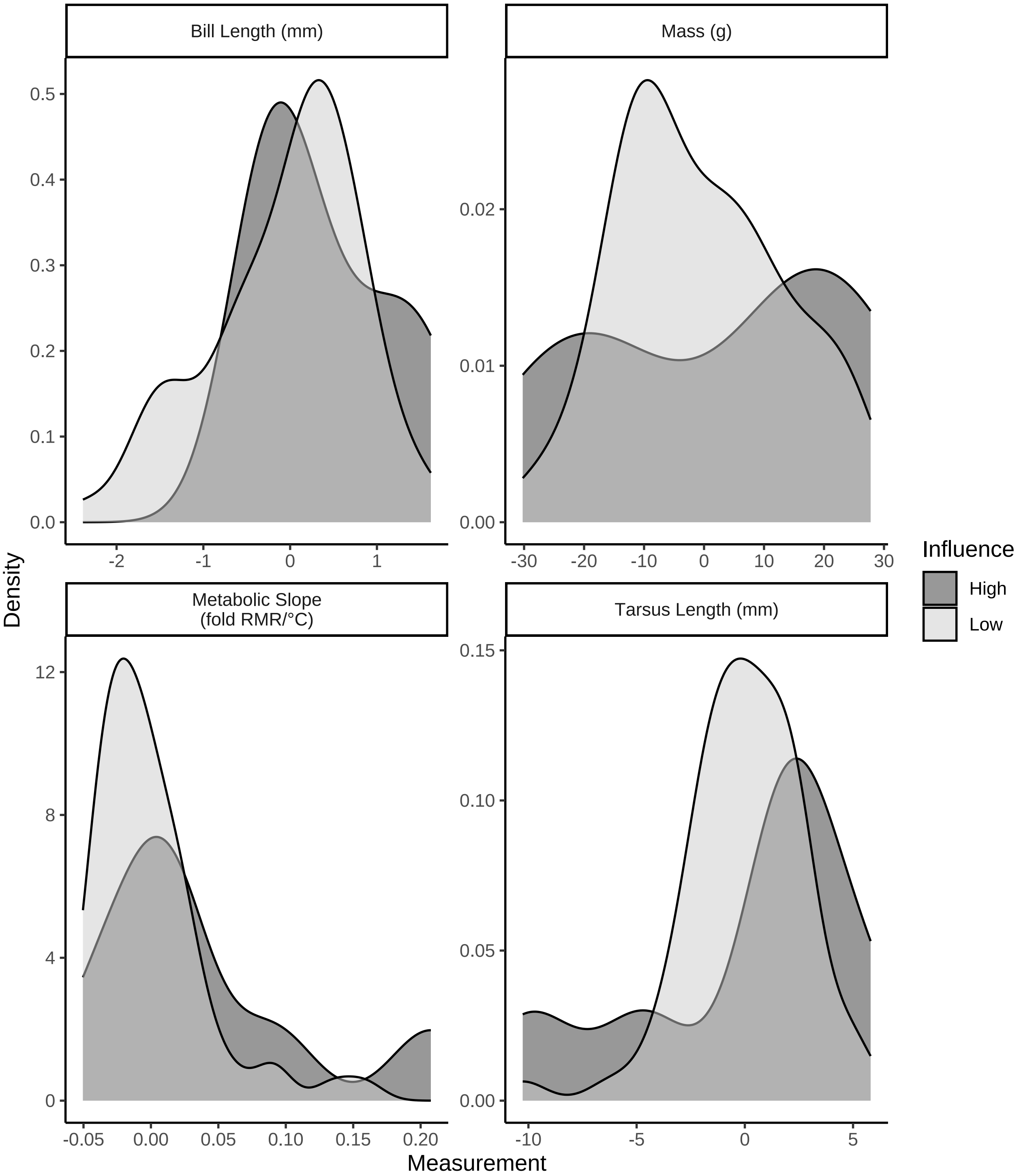

Figure 124: *Density of various morphometric and metabolic parameters for three week old Japanese quail according to their level of influence on a path analysis ultimately predicting fold change in resting metabolism (as measured at thermoneutrality)/°C in the heat. "High" influence represents values with corresponding pareto K values exceeding 0.5.*

Distinctions between high and low influence values are not obvious, except for a possible tendency for high and low masses to have high influence on our model. Below we use a power-scaling sensitivity analysis to

check whether our body mass priors conflict with our data.

```
data.frame(
  powerscale_sensitivity(functionModel3WeeksHot, component = "prior")
) %>%
  filter(grepl("mass", variable)) %>%
  `colnames<-`((str_to_title(colnames(.)))) %>%
  mutate(Diagnosis = ifelse(Diagnosis == "-", "", Diagnosis)) %>%
  mutate(Diagnosis = ifelse(Diagnosis == "strong prior / weak likelihood",
    "Strong prior and weak likelihood", Diagnosis
  )) %>%
  left_join(., tribble(
    ~Variable, ~Var,
    "b_mass_Intercept", "Mass Intercept",
    "b_mass_pretreatmentA", "Mass by Cold Rearing",
    "b_mass_pretreatmentC", "Mass by Warm Rearing",
    "b_tarsus_mass", "Tarsus Length by Mass",
    "b_bill_mass", "Bill Length by Mass",
    "b_slope_mass", "Metabolic Slope by Mass",
    "sd_batch__mass_Intercept", "Batch Effects on Mass",
    "sigma_mass", "Mass epsilon"
  ),
  by = "Variable"
  ) %>%
  drop_na(Var) %>%
  dplyr::select("Variable" = Var, Prior, Likelihood, Diagnosis) %>%
  kbl(.,
    longtable = T, booktabs = T, format = "latex",
    caption = paste0(
      "Results of a power-scaling sensitivity analysis applied ",
      "to a Bayesian path analysis predicting metabolic responses ",
      "of three week old Japanese quail to a heat challenge."
    ), escape = FALSE
  ) %>%
  column_spec(column = c(1:10), width = "2.5cm") %>%
  kable_styling(latex_options = "striped")
```

Table 65: *Results of a power-scaling sensitivity analysis applied to a Bayesian path analysis predicting metabolic responses of three week old Japanese quail to a heat challenge.*

Variable	Prior	Likelihood	Diagnosis
Mass Intercept	0.0314002	0.0827643	
Mass by Cold Rearing	0.0340974	0.0990896	
Mass by Warm Rearing	0.0230812	0.0819614	
Tarsus Length by Mass	0.0153783	0.1097678	
Bill Length by Mass	0.0285407	0.1033856	
Metabolic Slope by Mass	0.0060299	0.0976821	
Batch Effects on Mass	0.3955019	0.0770597	prior-data conflict
Mass epsilon	0.0448493	0.1491111	

Prior for the effect of batch on body mass could be relaxed Below, we do so by decreasing λ of our exponential prior to 1.5 from 2.5. Model R^2 values are then recalculated and both posterior predictive check and analysis of influence repeated.

```
functionModel3WeeksHot <-
  brm(
```

```
  data = slopeDataHot3 %>%
    mutate(
      mass = mass - mean(mass, na.rm = T),
      tarsus = tarsusLengthMean -
        mean(tarsusLengthMean, na.rm = T),
      bill = billLengthMean -
        mean(billLengthMean, na.rm = T),
      pretreatment = ifelse(pretreatment == "cold", "A",
        ifelse(pretreatment == "neutral", "B", "C")
      ),
      slope = slope -
        mean(slope, na.rm = T)
    ) %>%
    mutate(pretreatment = factor(pretreatment,
      levels = c("B", "A", "C")
    )) %>%
    drop_na() %>%
    merge(., data %>%
      dplyr::select(ring, "batch" = exp) %>%
      distinct(),
    by = "ring", all.x = TRUE
    ),
  family = "gaussian",
  bf(mass ~ pretreatment + (1 | batch)) +
    bf(tarsus ~ mass + pretreatment + (1 | batch)) +
    bf(bill ~ mass + pretreatment + (1 | batch)) +
    bf(
      slope ~ mass + tarsus + bill + pretreatment + (1 | batch),
      sigma ~ batch
    ) +
    set_rescor(FALSE),
  prior = c(
    set_prior("normal(0, 5)",
      class = "Intercept",
      resp = "mass"
    ),
    set_prior("normal(0, 15)",
      class = "b",
      coef = "pretreatmentA",
      resp = "mass"
    ),
    set_prior("normal(0, 15)",
      class = "b",
      coef = "pretreatmentC",
      resp = "mass"
    ),
    set_prior("exponential(1.5)",
      class = "sd",
      group = "batch",
      resp = "mass"
    ),
    set_prior("exponential(0.15)",
      class = "sigma",
      resp = "mass"
    ),
    set_prior("normal(0, 2.5)",
      class = "Intercept",
      resp = "tarsus"
    ),
    set_prior("normal(0, 2.5)",
      class = "b",
      coef = "pretreatmentA",
      resp = "tarsus"
    ),
    set_prior("normal(0, 2.5)",
      class = "b",
      coef = "pretreatmentC",
```

```
    resp = "tarsus"
  ),
  set_prior("skew_normal(0, 0.25, 5)",
    class = "b",
    coef = "mass",
    resp = "tarsus"
  ),
  set_prior("exponential(2)",
    class = "sd",
    group = "batch",
    resp = "tarsus"
  ),
  set_prior("exponential(1)",
    class = "sigma",
    resp = "tarsus"
  ),
  set_prior("normal(0, 1)",
    class = "Intercept",
    resp = "bill"
  ),
  set_prior("normal(0, 0.5)",
    class = "b",
    coef = "pretreatmentA",
    resp = "bill"
  ),
  set_prior("normal(0, 0.5)",
    class = "b",
    coef = "pretreatmentC",
    resp = "bill"
  ),
  set_prior("skew_normal(0, 0.25, 5)",
    class = "b",
    coef = "mass",
    resp = "bill"
  ),
  set_prior("exponential(5)",
    class = "sd",
    group = "batch",
    resp = "bill"
  ),
  set_prior("exponential(2.5)",
    class = "sigma",
    resp = "bill"
  ),
  set_prior("normal(0, 0.125)",
    class = "Intercept",
    resp = "slope"
  ),
  set_prior("normal(0, 0.125)",
    class = "b",
    coef = "pretreatmentA",
    resp = "slope"
  ),
  set_prior("normal(0, 0.125)",
    class = "b",
    coef = "pretreatmentC",
    resp = "slope"
  ),
  set_prior("normal(0, 0.004)",
    class = "b",
    coef = "mass",
    resp = "slope"
  ),
  set_prior("normal(0, 0.015)",
    class = "b",
    coef = "tarsus",
    resp = "slope"
```

```
      ),
      set_prior("normal(0, 0.06)",
        class = "b",
        coef = "bill",
        resp = "slope"
      ),
      set_prior("exponential(50)",
        class = "sd",
        group = "batch",
        resp = "slope"
      ),
      set_prior("normal(-3, 1.5)",
        dpar = "sigma",
        class = "Intercept",
        resp = "slope"
      ),
      set_prior("normal(0, 0.5)",
        dpar = "sigma",
        class = "b",
        coef = "batchB",
        resp = "slope"
      ),
      set_prior("normal(1, 1.5)",
        dpar = "sigma",
        class = "b",
        coef = "batchC",
        resp = "slope"
      )
    ),
    iter = 50000, warmup = 10000, cores = 4, chains = 4, thin = 20,
    control = list(adapt_delta = .98, max_treedepth = 14),
    silent = TRUE, refresh = 0,
    file = "./models/_threeWeekFunctionModelHotB.Rds"
  )

chainCheck(functionModel3WeeksHot)
```

```
## Rhat range: 1 - 1.001
## Neff/N range: 0.903 - 1.033
```

```
pp_check2(functionModel3WeeksHot, resp = "slope",
   xlab = "Metabolic Slope\n(Fold Metabolism at Thermoneutrality/°C)"
   ) + xlim(c(-5, 5))
```

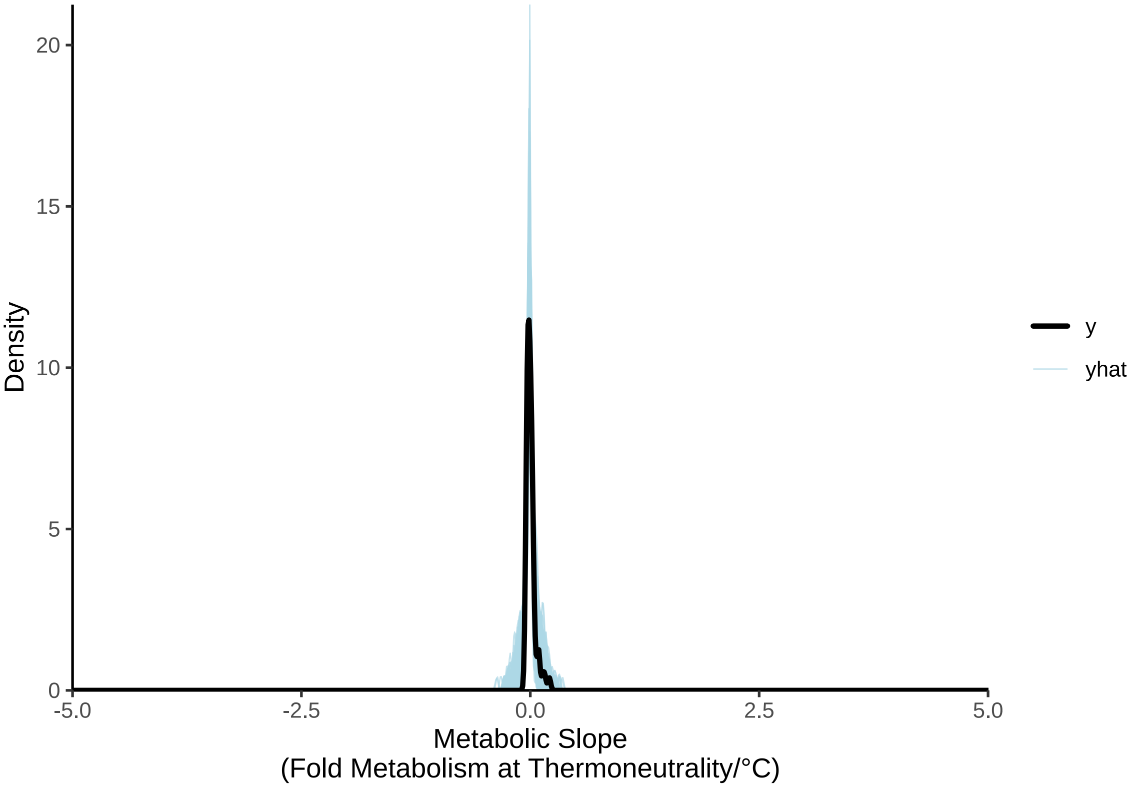

Figure 125: *Posterior predictive check for a Bayesian path analysis predicting physiological responses to heat in three week old Japanese quail. Black lines represent true densities of metabolic slopes (fold metabolism at thermoneutrality/°C) while blue lines represent densities estimated from the path analysis.*

```
caption <- paste0(
  "R\\textsuperscript{2} for a Bayesian ",
  "path analysis predicting metabolic slopes ",
  "(fold metabolism at thermoneutrality; 30°C) ",
  "of three week old Japanese quail as a ",
  "function of body mass (g), tarsus length (mm)" ,
  "bill length (mm), and rearing ",
  "temperature. Metabolic slopes are measured above ",
  "thermoneutrality (30°C - 40°C)."
)

brms::bayes_R2(functionModel3WeeksHot,
               ndraws = 1000,
               robust = TRUE) %>%
  as.data.frame() %>%
  rownames_to_column("var") %>%
  merge(., tribble(
    ~var, ~Var,
    "R2mass", "Body Mass (g)",
    "R2tarsus", "Tarsus Length (mm)",
    "R2bill", "Bill Length (mm)",
    "R2slope", "Metabolic Slope"
  ), by = c("var")) %>%
  mutate(Var = factor(Var, levels = c(
    "Body Mass (g)", "Tarsus Length (mm)",
    "Bill Length (mm)", "Metabolic Slope"
  ))) %>%
  mutate(Estimate = round(Estimate, digits = 4),
         Est.Error = round(Est.Error, digits = 4),
         `95\\% CI` = paste0("[", round(Q2.5, digits = 4),
                             ", ",
```

```
                              round(Q97.5, digits = 4),
                              "]"
        )
        ) %>%
  dplyr::select(
    "Response" = Var, "R\\textsuperscript{2}" = Estimate,
    "Standard Error" = Est.Error, `95\\% CI`
  ) %>%
  arrange(Response) %>%
  kbl(.,
    longtable = T, booktabs = T, format = "latex",
    caption = caption, escape = FALSE
  ) %>%
  column_spec(column = c(1:10), width = "2.5cm")  %>%
  kable_styling(latex_options = "striped")
```

Table 66: *R^2 for a Bayesian path analysis predicting metabolic slopes (fold metabolism at thermoneutrality; 30°C) of three week old Japanese quail as a function of body mass (g), tarsus length (mm)bill length (mm), and rearing temperature. Metabolic slopes are measured above thermoneutrality (30°C - 40°C).*

Response	R^2	Standard Error	95% CI
Body Mass (g)	0.1057	0.0611	[0.017, 0.2309]
Tarsus Length (mm)	0.3896	0.0748	[0.2228, 0.5145]
Bill Length (mm)	0.3586	0.0735	[0.2037, 0.4852]
Metabolic Slope	0.0993	0.0461	[0.031, 0.2322]

```
loo(functionModel3WeeksHot)$diagnostics$pareto_k %>%
  as_tibble() %>%
  ggplot(aes(x = 1:nrow(.), y = value)) +
  geom_point(pch = 21, colour = "black", fill = "grey50", size = 2) +
  geom_hline(yintercept = 0.5, colour = "black", linetype = "dashed") +
  geom_hline(yintercept = 0.7, colour = "red4", linetype = "dashed") +
  xlab("Sample") +
  ylab("Pareto K") +
  theme_classic()
```

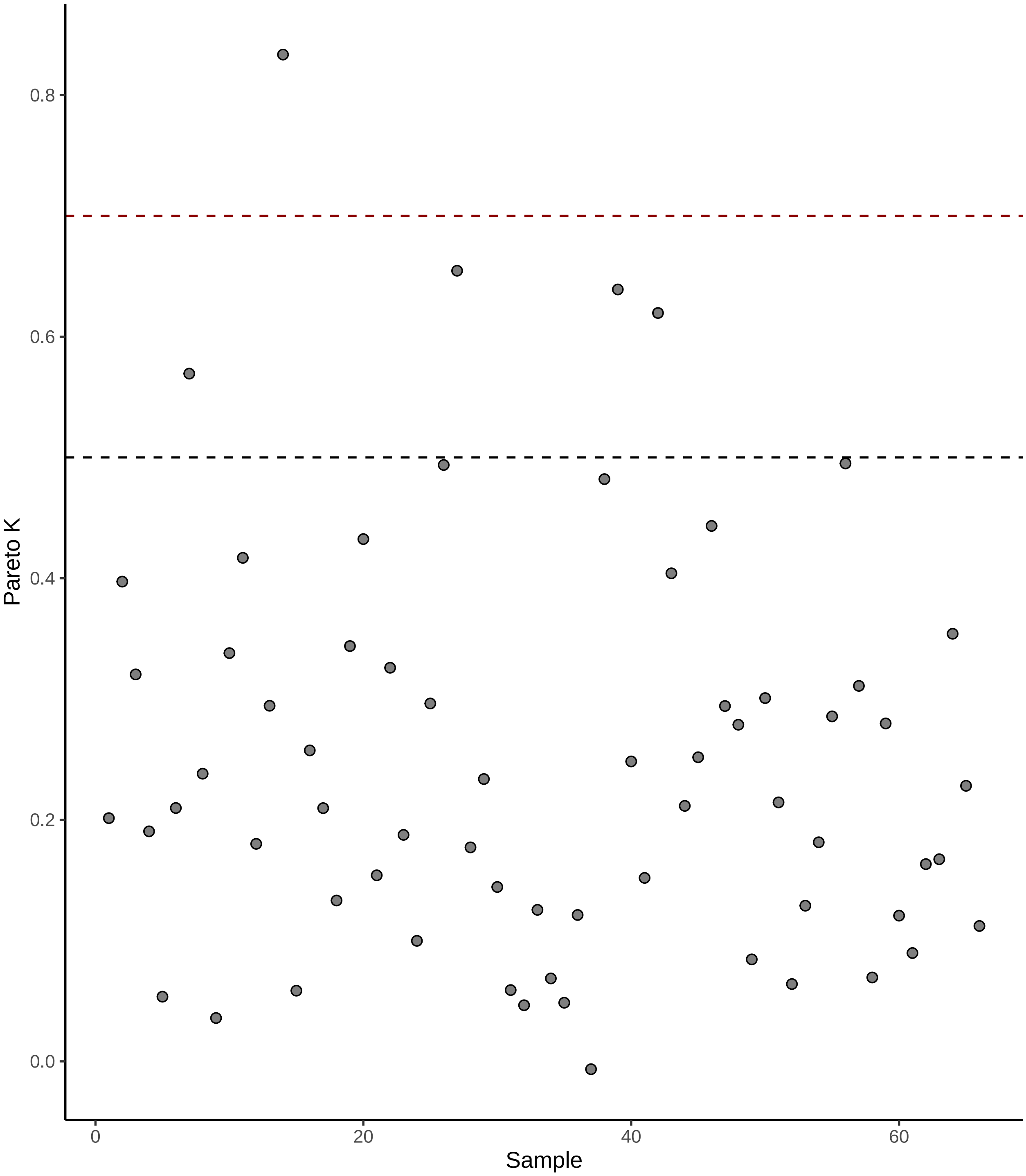

Figure 126: *Importance of individual data points (dots) to outcomes of our Bayesian path analysis, as estimated using leave-one-out cross validations. Importance is measured here as Pareto K (Vehtari et al, 2017). The black, dashed horizontal line indicated a Pareto K of 0.5 and the red horizontal dashed line indicates as Pareto K of 0.7, above which, samples hold significant importance on analysis outcomes.*

One value remains considerably influential. This value is investigated.

```
functionModel3WeeksHot$data %>%
  mutate(
    "Pareto K" = round(
      loo(functionModel3WeeksHot)$diagnostics$pareto_k, digits = 2
      )
  ) %>%
  filter(`Pareto K` > 0.7) %>%
  mutate(pretreatment = ifelse(pretreatment == "A", "Cold (10°C)",
    ifelse(pretreatment == "B", "Mild (20°C)",
      "Warm (30°C)"
    )
  )) %>%
  mutate(mass = round(mass, digits = 2),
         tarsus = round(tarsus, digits = 2),
         bill = round(bill, digits = 2),
         slope = round(slope, digits = 2),
         ) %>%
  dplyr::select(
    "Rearing Treatment" = pretreatment,
    "Body Mass (g)" = mass,
    "Tarsus Length (mm)" = tarsus,
    "Bill Length (mm)" = bill,
    "Metabolic Slope" = slope,
    "Pareto K"
  ) %>%
  kbl(.,
    longtable = T, booktabs = T, format = "latex",
    caption = paste0(
      "Morphology and physiology of an individual identified ",
      "as having high influence (Pareto K > 0.7) on the outcome ",
      "of a Bayesian path analysis predicting metabolic ",
      "responses of three week old Japanese quail to a heat ",
      "exposure. Morphometric measurements are mean centred."
    ),
    escape = FALSE
  ) %>%
  column_spec(column = c(1:10), width = "2.1cm") %>%
  kable_styling(latex_options = "striped")
```

Table 67: *Morphology and physiology of an individual identified as having high influence (Pareto K > 0.7) on the outcome of a Bayesian path analysis predicting metabolic responses of three week old Japanese quail to a heat exposure. Morphometric measurements are mean centred.*

Rearing Treatment	Body Mass (g)	Tarsus Length (mm)	Bill Length (mm)	Metabolic Slope	Pareto K
Mild (20°C)	14.07	-9.91	-0.05	0.02	0.83

The identified individual appears to have a relatively small tarsus length for its body mass, although not to the degree of being erroneous. We therefore retain this individual but repeat our model with a student-t distributed error term to reduce its influence on total model outcomes. Here, we use a conservative gamma-distributed prior for ν of our error term, with α equaling 5 and β equaling 1.

```
functionModel3WeeksHot <-
  brm(
    data = slopeDataHot3 %>%
      mutate(
        mass = mass - mean(mass, na.rm = T),
        tarsus = tarsusLengthMean -
          mean(tarsusLengthMean, na.rm = T),
        bill = billLengthMean -
          mean(billLengthMean, na.rm = T),
        pretreatment = ifelse(pretreatment == "cold", "A",
          ifelse(pretreatment == "neutral", "B", "C")
```

```
      ),
      slope = slope -
        mean(slope, na.rm = T)
    ) %>%
    mutate(pretreatment = factor(pretreatment,
      levels = c("B", "A", "C")
    )) %>%
    drop_na() %>%
    merge(., data %>%
      dplyr::select(ring, "batch" = exp) %>%
      distinct(),
    by = "ring", all.x = TRUE
    ),
  bf(mass ~ pretreatment + (1 | batch),
    family = "gaussian") +
    bf(tarsus ~ mass + pretreatment + (1 | batch),
    family = "gaussian") +
    bf(bill ~ mass + pretreatment + (1 | batch),
    family = "gaussian") +
    bf(
      slope ~ mass + tarsus + bill + pretreatment + (1 | batch),
      sigma ~ batch, family = "student"
    ) +
    set_rescor(FALSE),
  prior = c(
    set_prior("normal(0, 5)",
      class = "Intercept",
      resp = "mass"
    ),
    set_prior("normal(0, 15)",
      class = "b",
      coef = "pretreatmentA",
      resp = "mass"
    ),
    set_prior("normal(0, 15)",
      class = "b",
      coef = "pretreatmentC",
      resp = "mass"
    ),
    set_prior("exponential(1.5)",
      class = "sd",
      group = "batch",
      resp = "mass"
    ),
    set_prior("exponential(0.15)",
      class = "sigma",
      resp = "mass"
    ),
    set_prior("normal(0, 2.5)",
      class = "Intercept",
      resp = "tarsus"
    ),
    set_prior("normal(0, 2.5)",
      class = "b",
      coef = "pretreatmentA",
      resp = "tarsus"
    ),
    set_prior("normal(0, 2.5)",
      class = "b",
      coef = "pretreatmentC",
      resp = "tarsus"
    ),
    set_prior("skew_normal(0, 0.25, 5)",
      class = "b",
      coef = "mass",
      resp = "tarsus"
    ),
```

```
    set_prior("exponential(2)",
      class = "sd",
      group = "batch",
      resp = "tarsus"
    ),
    set_prior("exponential(1)",
      class = "sigma",
      resp = "tarsus"
    ),
    set_prior("normal(0, 1)",
      class = "Intercept",
      resp = "bill"
    ),
    set_prior("normal(0, 0.5)",
      class = "b",
      coef = "pretreatmentA",
      resp = "bill"
    ),
    set_prior("normal(0, 0.5)",
      class = "b",
      coef = "pretreatmentC",
      resp = "bill"
    ),
    set_prior("skew_normal(0, 0.25, 5)",
      class = "b",
      coef = "mass",
      resp = "bill"
    ),
    set_prior("exponential(5)",
      class = "sd",
      group = "batch",
      resp = "bill"
    ),
    set_prior("exponential(2.5)",
      class = "sigma",
      resp = "bill"
    ),
    set_prior("normal(0, 0.125)",
      class = "Intercept",
      resp = "slope"
    ),
    set_prior("normal(0, 0.125)",
      class = "b",
      coef = "pretreatmentA",
      resp = "slope"
    ),
    set_prior("normal(0, 0.125)",
      class = "b",
      coef = "pretreatmentC",
      resp = "slope"
    ),
    set_prior("normal(0, 0.004)",
      class = "b",
      coef = "mass",
      resp = "slope"
    ),
    set_prior("normal(0, 0.015)",
      class = "b",
      coef = "tarsus",
      resp = "slope"
    ),
    set_prior("normal(0, 0.06)",
      class = "b",
      coef = "bill",
      resp = "slope"
    ),
    set_prior("exponential(50)",
```

```
        class = "sd",
        group = "batch",
        resp = "slope"
      ),
      set_prior("normal(-3, 1.5)",
        dpar = "sigma",
        class = "Intercept",
        resp = "slope"
      ),
      set_prior("normal(0, 0.5)",
        dpar = "sigma",
        class = "b",
        coef = "batchB",
        resp = "slope"
      ),
      set_prior("normal(1, 1.5)",
        dpar = "sigma",
        class = "b",
        coef = "batchC",
        resp = "slope"
      ),
      set_prior("gamma(5, 1)",
        class = "nu",
        resp = "slope"
      )
    ),
    iter = 50000, warmup = 10000, cores = 4, chains = 4, thin = 20,
    control = list(adapt_delta = .98, max_treedepth = 14),
    silent = TRUE, refresh = 0,
    file = "./models/_threeWeekFunctionModelHotC.Rds"
  )

chainCheck(functionModel3WeeksHot)
```

```
## Rhat range: 1 - 1.002
## Neff/N range: 0.132 - 1.001
```

```
caption <- paste0(
  "R\\textsuperscript{2} for a Bayesian ",
  "path analysis predicting metabolic slopes ",
  "(fold metabolism at thermoneutrality; 30°C) ",
  "of three week old Japanese quail as a ",
  "function of body mass (g), tarsus length (mm) ",
  "bill length (mm), and rearing ",
  "temperature. Metabolic slopes are measured above ",
  "thermoneutrality (30°C - 40°C)."
)

brms::bayes_R2(functionModel3WeeksHot,
               ndraws = 1000,
               robust = TRUE) %>%
  as.data.frame() %>%
  rownames_to_column("var") %>%
  merge(., tribble(
    ~var, ~Var,
    "R2mass", "Body Mass (g)",
    "R2tarsus", "Tarsus Length (mm)",
    "R2bill", "Bill Length (mm)",
    "R2slope", "Metabolic Slope"
  ), by = c("var")) %>%
  mutate(Var = factor(Var, levels = c(
    "Body Mass (g)", "Tarsus Length (mm)",
    "Bill Length (mm)", "Metabolic Slope"
  ))) %>%
  mutate(Estimate = round(Estimate, digits = 4),
         Est.Error = round(Est.Error, digits = 4),
         `95\\% CI` = paste0("[", round(Q2.5, digits = 4),
                             ", ",
```

```
                              round(Q97.5, digits = 4),
                              "]"
        )
        ) %>%
  dplyr::select(
    "Response" = Var, "R\\textsuperscript{2}" = Estimate,
    "Standard Error" = Est.Error, `95\\% CI`
  ) %>%
  arrange(Response) %>%
  kbl(.,
    longtable = T, booktabs = T, format = "latex",
    caption = caption, escape = FALSE
  ) %>%
  column_spec(column = c(1:10), width = "2.5cm")  %>%
  kable_styling(latex_options = "striped")
```

Table 68: *R^2 for a Bayesian path analysis predicting metabolic slopes (fold metabolism at thermoneutrality; 30°C) of three week old Japanese quail as a function of body mass (g), tarsus length (mm) bill length (mm), and rearing temperature. Metabolic slopes are measured above thermoneutrality (30°C - 40°C).*

Response	R^2	Standard Error	95% CI
Body Mass (g)	0.1007	0.0603	[0.011, 0.2162]
Tarsus Length (mm)	0.3967	0.0789	[0.2061, 0.5196]
Bill Length (mm)	0.3655	0.0768	[0.1895, 0.4872]
Metabolic Slope	0.0888	0.0429	[0.0282, 0.1973]

```
loo(functionModel3WeeksHot)$diagnostics$pareto_k %>%
  as_tibble() %>%
  ggplot(aes(x = 1:nrow(.), y = value)) +
  geom_point(pch = 21, colour = "black", fill = "grey50", size = 2) +
  geom_hline(yintercept = 0.5, colour = "black", linetype = "dashed") +
  geom_hline(yintercept = 0.7, colour = "red4", linetype = "dashed") +
  xlab("Sample") +
  ylab("Pareto K") +
  theme_classic()
```

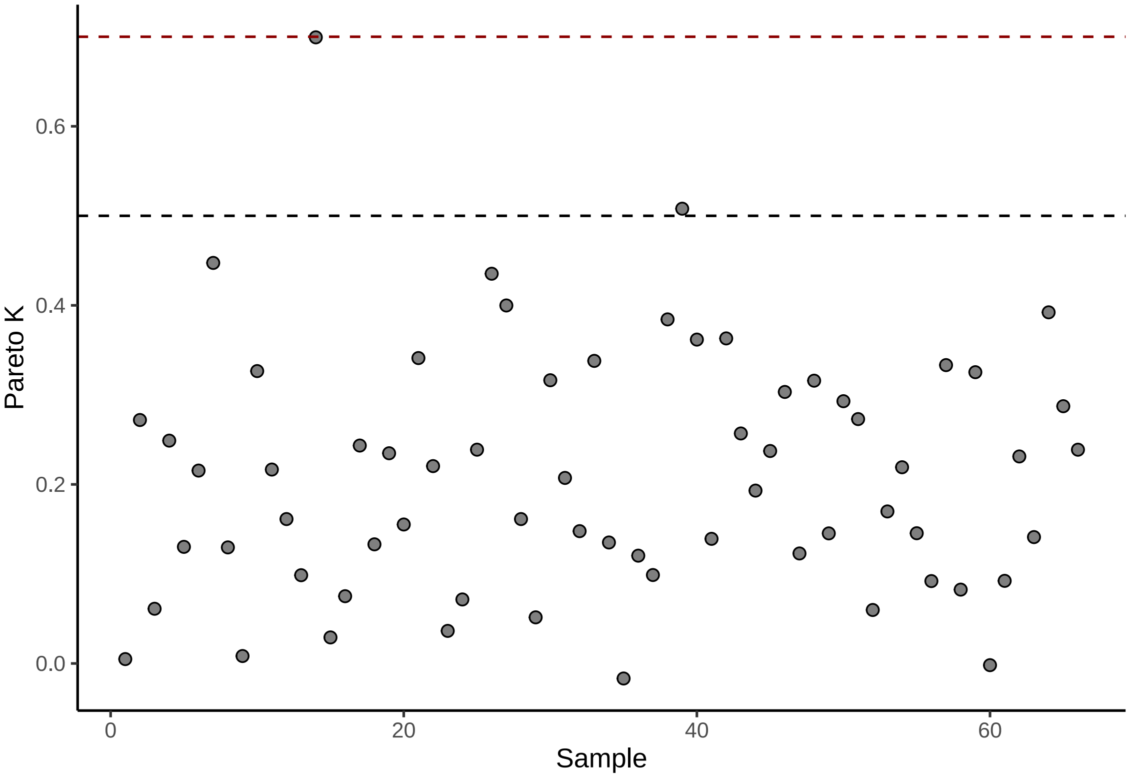

Figure 127: *Importance of individual data points (dots) to outcomes of our Bayesian path analysis, as estimated using leave-one-out cross validations. Importance is measured as described previously. The black, dashed horizontal line indicated a Pareto K of 0.5 and the red horizontal dashed line indicates as Pareto K of 0.7. Here, the error term for our model predicting metabolic responses to heat exposure is students-t distributed.*

Influence of certain extreme points is reduced and now arguably acceptable. Coefficient estimates are therefore next drawn from posteriors and visualised for normality. After-hand, these coefficients are summarised then used to plot conditional effects.

```
as.data.frame(functionModel3WeeksHot) %>%
    dplyr::select(
      "Intercept" = b_slope_Intercept,
      "Body Mass\n(g; Mean-Centred)" = b_slope_mass,
      "Tarsus Length\n(mm; Mean-Centred)" = b_slope_tarsus,
      "Bill Length\n(mm; Mean-Centred)" = b_slope_bill,
      "Cold Rearing\n(10°C)" = b_slope_pretreatmentA,
      "Warm Rearing\n(30°C)" = b_slope_pretreatmentC,
      #"Sigma" = sigma_slope
    ) %>%
    pivot_longer(everything(), names_to = "Par",
                 values_to = "Coefs") %>%
    ggplot(aes(x = Coefs)) +
    facet_wrap(~Par, scales = "free", ncol = 2) +
    geom_density(colour = "black",
                 alpha = 0.5, fill = "grey70") +
    geom_vline(xintercept = 0, linetype = "dashed",
               colour = "black") +
    ylab("Density") +
    scale_x_continuous(n.breaks = 4) +
    theme_classic() +
    theme(axis.title.x = element_blank()) +
    ggtitle(paste0("Metabolic Slope\n(Fold Metabolism ",
```

```
                  "at Thermoneutrality/°C)")
      )
```

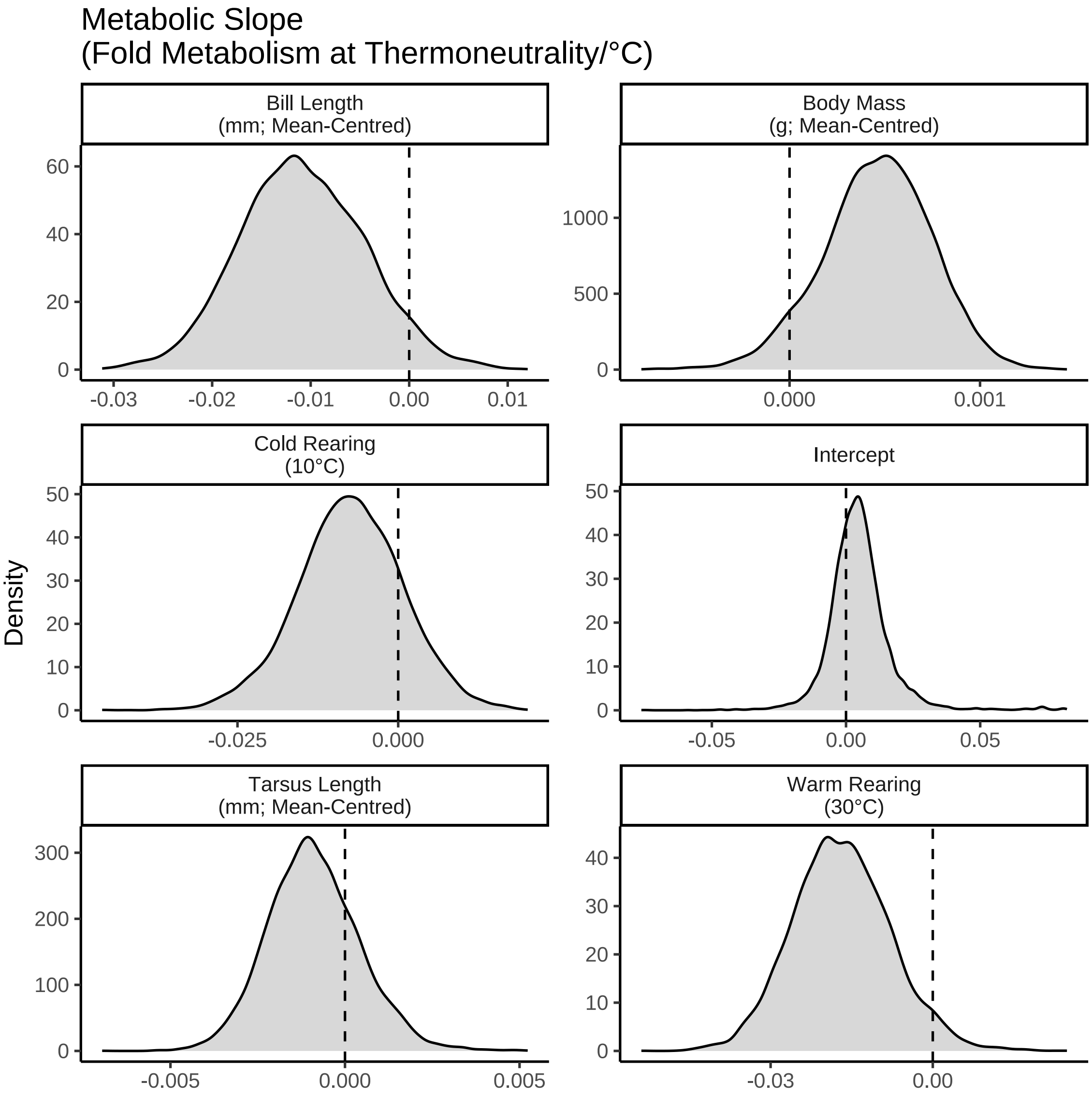

Figure 128: *Posterior densities of coefficients for a Bayesian path analysis predicting physiological responses to heat in three week old Japanese quail. Quail were reared in one of three thermal conditions: (1) cold (10°C), (2) mild (20°C), or warm (30°C).*

```
caption <- paste0(
  "Results from a Bayesian path analysis ",
  "predicting metabolic responses to heat (metabolic slope ",
  "[fold metabolism at thermoneutrality/°C]) as ",
  "a function of morphometry in three week old Japanese quail. ",
  "Physiological measurements are made at ambient temperatures ",
  "between 30°C and 40°C. Cold rearing indicates ",
  "post-hatch rearing at 10°C, relative to 20°C (intercept), ",
  "or 30°C ('warm rearing'). CIs indicates quantile intervals ",
  "and BF indicates Bayes Factors."
)

week3ResultsHeat <-
```

```
as.data.frame(functionModel3WeeksHot) %>%
summarise_all(., .funs = median) %>%
pivot_longer(everything(),
  names_to = "Parameter",
  values_to = "Estimate"
) %>%
merge(., quantileCIs(functionModel3WeeksHot, cis = c(50, 95)),
  by = "Parameter", all.x = TRUE
) %>%
filter(grepl("b_|sd_", Parameter)) %>%
rowwise() %>%
mutate("BF" = ifelse(Estimate < 0,
  (2 * mean(as.data.frame(
    functionModel3WeeksHot
  )[, Parameter] <= 0)) /
    (2 * mean(as.data.frame(
      functionModel3WeeksHot
    )[, Parameter] >= 0)),
  (2 * mean(as.data.frame(
    functionModel3WeeksHot
  )[, Parameter] >= 0)) /
    (2 * mean(as.data.frame(
      functionModel3WeeksHot
    )[, Parameter] <= 0))
)) %>%
ungroup() %>%
mutate(
  "Estimate" = round(Estimate, digits = 4),
  "BF" = round(BF, digits = 4),
  "N" = nrow(functionModel3WeeksHot$data)
) %>%
mutate("Parameter" = ifelse(grepl("b_", Parameter),
  gsub("b_", "", Parameter),
  gsub(
    "Intercept", "batch",
    gsub(".*__", "", Parameter)
  )
)) %>%
mutate(
  "Response" = gsub("_.*", "", Parameter),
  "Parameter" = gsub(".*_", "", Parameter)
) %>%
merge(., tribble(
  ~Response, ~response, ~level,
  "mass", "Body Mass (g)", "A",
  "slope",
  "Metabolic Slope", "D",
  "tarsus", "Tarsus Length (mm)", "B",
  "bill", "Bill Length (mm)", "C"
),
by = "Response"
) %>%
merge(., tribble(
  ~Parameter, ~parameter, ~number,
  "Intercept", "Intercept", "1",
  "mass", "Body Mass (g)", "4",
  "tarsus", "Tarsus Length (mm)", "5",
  "bill", "Bill Length (mm)", "6",
  "pretreatmentA", "Cold Rearing", "2",
  "pretreatmentC", "Warm Rearing", "3",
  "batch", "Egg Batch [mu]", "7"
),
by = "Parameter"
) %>%
mutate(
  `50\\% HDI` = paste0("(", paste(
    round(Low_CI_50, digits = 4),
```

```
      round(High_CI_50, digits = 4),
      sep = ", "
    ), ")"),
    `95\\% HDI` = paste0("(", paste(
      round(Low_CI_95, digits = 4),
      round(High_CI_95, digits = 4),
      sep = ", "
    ), ")")
  ) %>%
  dplyr::select(-c(Low_CI_50, High_CI_50, Low_CI_95, High_CI_95)) %>%
  dplyr::select(
    "Response" = "response", "Parameter" = "parameter", N,
    Estimate, `50\\% HDI`, `95\\% HDI`, BF, level, number
  ) %>%
  arrange(level, number) %>%
  dplyr::select(-c(level, number)) %>%
  kbl(.,
    longtable = T, booktabs = T, format = "latex", escape = FALSE,
    caption = caption
  ) %>%
  column_spec(column = c(1:2), width = "2.2cm") %>%
  column_spec(column = c(3:10), width = "1.9cm") %>%
  kable_styling(latex_options = "striped")

week3ResultsHeat
```

Table 69: *Results from a Bayesian path analysis predicting metabolic responses to heat (metabolic slope [fold metabolism at thermoneutrality/°C]) as a function of morphometry in three week old Japanese quail. Physiological measurements are made at ambient temperatures between 30°C and 40°C. Cold rearing indicates post-hatch rearing at 10°C, relative to 20°C (intercept), or 30°C ('warm rearing'). CIs indicates quantile intervals and BF indicates Bayes Factors.*

Response	Parameter	N	Estimate	50% HDI	95% HDI	BF
Body Mass (g)	Intercept	66	3.6790	(1.4086, 5.9185)	(-3.053, 10.5009)	6.2202
Body Mass (g)	Cold Rearing	66	-8.6318	(-11.3618, -5.7476)	(-16.943, -0.2365)	44.7143
Body Mass (g)	Warm Rearing	66	-0.0696	(-3.0992, 2.9184)	(-8.8111, 8.9828)	1.0274
Body Mass (g)	Egg Batch [mu]	66	0.4300	(0.1788, 0.8333)	(0.0146, 2.1229)	Inf
Tarsus Length (mm)	Intercept	66	-0.7166	(-1.2824, -0.1427)	(-2.4035, 1.1135)	3.8960
Tarsus Length (mm)	Cold Rearing	66	0.4412	(-0.0733, 0.9546)	(-1.0702, 1.9931)	2.5320
Tarsus Length (mm)	Warm Rearing	66	1.7794	(1.1783, 2.3872)	(0.056, 3.5214)	46.0588
Tarsus Length (mm)	Body Mass (g)	66	0.0639	(0.0499, 0.0778)	(0.0246, 0.1051)	3999.0000
Tarsus Length (mm)	Egg Batch [mu]	66	0.9704	(0.6867, 1.3082)	(0.2003, 2.2182)	Inf
Bill Length (mm)	Intercept	66	0.0549	(-0.145, 0.2552)	(-0.5867, 0.6519)	1.3516
Bill Length (mm)	Cold Rearing	66	-0.3695	(-0.5114, -0.2265)	(-0.7872, 0.051)	22.2558
Bill Length (mm)	Warm Rearing	66	0.4204	(0.262, 0.5744)	(-0.0462, 0.8698)	24.9740
Bill Length (mm)	Body Mass (g)	66	0.0038	(-1e-04, 0.0079)	(-0.0083, 0.0156)	2.8816
Bill Length (mm)	Egg Batch [mu]	66	0.4193	(0.3269, 0.5421)	(0.1965, 0.9017)	Inf
Metabolic Slope	Intercept	66	0.0041	(-0.0014, 0.0098)	(-0.0152, 0.0293)	2.2520

Metabolic Slope	Cold Rearing	66	-0.0075	(-0.0128, -0.002)	(-0.0242, 0.0085)	4.6259
Metabolic Slope	Warm Rearing	66	-0.0171	(-0.023, -0.011)	(-0.034, 0.0019)	25.0586
Metabolic Slope	Body Mass (g)	66	0.0005	(3e-04, 7e-04)	(-1e-04, 0.001)	15.1943
Metabolic Slope	Tarsus Length (mm)	66	-0.0010	(-0.0018, -1e-04)	(-0.0033, 0.0018)	3.2061
Metabolic Slope	Bill Length (mm)	66	-0.0110	(-0.0152, -0.0065)	(-0.023, 0.0019)	19.9424
Metabolic Slope	Egg Batch [mu]	66	0.0076	(0.0033, 0.0147)	(3e-04, 0.0407)	Inf

```
#save_kable(week3ResultsHeat,
# "../tables/pathAnalysisInHeat3Weeks.html")

{
  functionModel3WeeksHotMassR2 <-
    brm(
      data = slopeDataHot3 %>%
        mutate(
          mass = mass - mean(mass, na.rm = T),
          tarsus = tarsusLengthMean -
            mean(tarsusLengthMean, na.rm = T),
          bill = billLengthMean -
            mean(billLengthMean, na.rm = T),
          pretreatment = ifelse(pretreatment == "cold", "A",
            ifelse(pretreatment == "neutral", "B", "C")
          ),
          slope = slope -
            mean(slope, na.rm = T)
        ) %>%
        mutate(pretreatment = factor(pretreatment,
          levels = c("B", "A", "C")
        )) %>%
        drop_na() %>%
        merge(., data %>%
          dplyr::select(ring, "batch" = exp) %>%
          distinct(),
        by = "ring", all.x = TRUE
        ),
      bf(mass ~ pretreatment + (1 | batch),
        family = "gaussian"
      ) +
        bf(tarsus ~ mass + pretreatment + (1 | batch),
          family = "gaussian"
        ) +
        bf(bill ~ mass + pretreatment + (1 | batch),
          family = "gaussian"
        ) +
        bf(
          slope ~ tarsus + bill + pretreatment + (1 | batch),
          sigma ~ batch,
          family = "student"
        ) +
        set_rescor(FALSE),
      prior = c(
        set_prior("normal(0, 5)",
          class = "Intercept",
          resp = "mass"
        ),
        set_prior("normal(0, 15)",
          class = "b",
          coef = "pretreatmentA",
          resp = "mass"
        ),
        set_prior("normal(0, 15)",
```

```
      class = "b",
      coef = "pretreatmentC",
      resp = "mass"
    ),
    set_prior("exponential(1.5)",
      class = "sd",
      group = "batch",
      resp = "mass"
    ),
    set_prior("exponential(0.15)",
      class = "sigma",
      resp = "mass"
    ),
    set_prior("normal(0, 2.5)",
      class = "Intercept",
      resp = "tarsus"
    ),
    set_prior("normal(0, 2.5)",
      class = "b",
      coef = "pretreatmentA",
      resp = "tarsus"
    ),
    set_prior("normal(0, 2.5)",
      class = "b",
      coef = "pretreatmentC",
      resp = "tarsus"
    ),
    set_prior("skew_normal(0, 0.25, 5)",
      class = "b",
      coef = "mass",
      resp = "tarsus"
    ),
    set_prior("exponential(2)",
      class = "sd",
      group = "batch",
      resp = "tarsus"
    ),
    set_prior("exponential(1)",
      class = "sigma",
      resp = "tarsus"
    ),
    set_prior("normal(0, 1)",
      class = "Intercept",
      resp = "bill"
    ),
    set_prior("normal(0, 0.5)",
      class = "b",
      coef = "pretreatmentA",
      resp = "bill"
    ),
    set_prior("normal(0, 0.5)",
      class = "b",
      coef = "pretreatmentC",
      resp = "bill"
    ),
    set_prior("skew_normal(0, 0.25, 5)",
      class = "b",
      coef = "mass",
      resp = "bill"
    ),
    set_prior("exponential(5)",
      class = "sd",
      group = "batch",
      resp = "bill"
    ),
    set_prior("exponential(2.5)",
      class = "sigma",
```

```
        resp = "bill"
      ),
      set_prior("normal(0, 0.125)",
        class = "Intercept",
        resp = "slope"
      ),
      set_prior("normal(0, 0.125)",
        class = "b",
        coef = "pretreatmentA",
        resp = "slope"
      ),
      set_prior("normal(0, 0.125)",
        class = "b",
        coef = "pretreatmentC",
        resp = "slope"
      ),
      set_prior("normal(0, 0.015)",
        class = "b",
        coef = "tarsus",
        resp = "slope"
      ),
      set_prior("normal(0, 0.06)",
        class = "b",
        coef = "bill",
        resp = "slope"
      ),
      set_prior("exponential(50)",
        class = "sd",
        group = "batch",
        resp = "slope"
      ),
      set_prior("normal(-3, 1.5)",
        dpar = "sigma",
        class = "Intercept",
        resp = "slope"
      ),
      set_prior("normal(0, 0.5)",
        dpar = "sigma",
        class = "b",
        coef = "batchB",
        resp = "slope"
      ),
      set_prior("normal(1, 1.5)",
        dpar = "sigma",
        class = "b",
        coef = "batchC",
        resp = "slope"
      ),
      set_prior("gamma(5, 1)",
        class = "nu",
        resp = "slope"
      )
    ),
    iter = 50000, warmup = 10000, cores = 4, chains = 4, thin = 20,
    control = list(adapt_delta = .98, max_treedepth = 14),
    silent = TRUE, refresh = 0,
    file = "./models/_threeWeekFunctionModelHotMassR2.Rds"
  )

functionModel3WeeksHotTarsusR2 <-
  brm(
    data = slopeDataHot3 %>%
      mutate(
        mass = mass - mean(mass, na.rm = T),
        tarsus = tarsusLengthMean -
          mean(tarsusLengthMean, na.rm = T),
        bill = billLengthMean -
```

```
          mean(billLengthMean, na.rm = T),
        pretreatment = ifelse(pretreatment == "cold", "A",
          ifelse(pretreatment == "neutral", "B", "C")
        ),
        slope = slope -
          mean(slope, na.rm = T)
      ) %>%
      mutate(pretreatment = factor(pretreatment,
        levels = c("B", "A", "C")
      )) %>%
      drop_na() %>%
      merge(., data %>%
        dplyr::select(ring, "batch" = exp) %>%
        distinct(),
      by = "ring", all.x = TRUE
      ),
    bf(mass ~ pretreatment + (1 | batch),
      family = "gaussian"
    ) +
      bf(tarsus ~ mass + pretreatment + (1 | batch),
        family = "gaussian"
      ) +
      bf(bill ~ mass + pretreatment + (1 | batch),
        family = "gaussian"
      ) +
      bf(
        slope ~ mass + bill + pretreatment + (1 | batch),
        sigma ~ batch,
        family = "student"
      ) +
      set_rescor(FALSE),
    prior = c(
      set_prior("normal(0, 5)",
        class = "Intercept",
        resp = "mass"
      ),
      set_prior("normal(0, 15)",
        class = "b",
        coef = "pretreatmentA",
        resp = "mass"
      ),
      set_prior("normal(0, 15)",
        class = "b",
        coef = "pretreatmentC",
        resp = "mass"
      ),
      set_prior("exponential(1.5)",
        class = "sd",
        group = "batch",
        resp = "mass"
      ),
      set_prior("exponential(0.15)",
        class = "sigma",
        resp = "mass"
      ),
      set_prior("normal(0, 2.5)",
        class = "Intercept",
        resp = "tarsus"
      ),
      set_prior("normal(0, 2.5)",
        class = "b",
        coef = "pretreatmentA",
        resp = "tarsus"
      ),
      set_prior("normal(0, 2.5)",
        class = "b",
        coef = "pretreatmentC",
```

```
      resp = "tarsus"
    ),
    set_prior("skew_normal(0, 0.25, 5)",
      class = "b",
      coef = "mass",
      resp = "tarsus"
    ),
    set_prior("exponential(2)",
      class = "sd",
      group = "batch",
      resp = "tarsus"
    ),
    set_prior("exponential(1)",
      class = "sigma",
      resp = "tarsus"
    ),
    set_prior("normal(0, 1)",
      class = "Intercept",
      resp = "bill"
    ),
    set_prior("normal(0, 0.5)",
      class = "b",
      coef = "pretreatmentA",
      resp = "bill"
    ),
    set_prior("normal(0, 0.5)",
      class = "b",
      coef = "pretreatmentC",
      resp = "bill"
    ),
    set_prior("skew_normal(0, 0.25, 5)",
      class = "b",
      coef = "mass",
      resp = "bill"
    ),
    set_prior("exponential(5)",
      class = "sd",
      group = "batch",
      resp = "bill"
    ),
    set_prior("exponential(2.5)",
      class = "sigma",
      resp = "bill"
    ),
    set_prior("normal(0, 0.125)",
      class = "Intercept",
      resp = "slope"
    ),
    set_prior("normal(0, 0.125)",
      class = "b",
      coef = "pretreatmentA",
      resp = "slope"
    ),
    set_prior("normal(0, 0.125)",
      class = "b",
      coef = "pretreatmentC",
      resp = "slope"
    ),
    set_prior("normal(0, 0.004)",
      class = "b",
      coef = "mass",
      resp = "slope"
    ),
    set_prior("normal(0, 0.06)",
      class = "b",
      coef = "bill",
      resp = "slope"
```

```
      ),
      set_prior("exponential(50)",
        class = "sd",
        group = "batch",
        resp = "slope"
      ),
      set_prior("normal(-3, 1.5)",
        dpar = "sigma",
        class = "Intercept",
        resp = "slope"
      ),
      set_prior("normal(0, 0.5)",
        dpar = "sigma",
        class = "b",
        coef = "batchB",
        resp = "slope"
      ),
      set_prior("normal(1, 1.5)",
        dpar = "sigma",
        class = "b",
        coef = "batchC",
        resp = "slope"
      ),
      set_prior("gamma(5, 1)",
        class = "nu",
        resp = "slope"
      )
    ),
    iter = 50000, warmup = 10000, cores = 4, chains = 4, thin = 20,
    control = list(adapt_delta = .98, max_treedepth = 14),
    silent = TRUE, refresh = 0,
    file = "./models/_threeWeekFunctionModelHotTarsusR2.Rds"
  )

functionModel3WeeksHotBillR2 <-
  brm(
    data = slopeDataHot3 %>%
      mutate(
        mass = mass - mean(mass, na.rm = T),
        tarsus = tarsusLengthMean -
          mean(tarsusLengthMean, na.rm = T),
        bill = billLengthMean -
          mean(billLengthMean, na.rm = T),
        pretreatment = ifelse(pretreatment == "cold", "A",
          ifelse(pretreatment == "neutral", "B", "C")
        ),
        slope = slope -
          mean(slope, na.rm = T)
      ) %>%
      mutate(pretreatment = factor(pretreatment,
        levels = c("B", "A", "C")
      )) %>%
      drop_na() %>%
      merge(., data %>%
        dplyr::select(ring, "batch" = exp) %>%
        distinct(),
      by = "ring", all.x = TRUE
      ),
    bf(mass ~ pretreatment + (1 | batch),
      family = "gaussian"
    ) +
      bf(tarsus ~ mass + pretreatment + (1 | batch),
        family = "gaussian"
      ) +
      bf(bill ~ mass + pretreatment + (1 | batch),
        family = "gaussian"
      ) +
```

```
        bf(
          slope ~ mass + tarsus + pretreatment + (1 | batch),
          sigma ~ batch,
          family = "student"
        ) +
        set_rescor(FALSE),
      prior = c(
        set_prior("normal(0, 5)",
          class = "Intercept",
          resp = "mass"
        ),
        set_prior("normal(0, 15)",
          class = "b",
          coef = "pretreatmentA",
          resp = "mass"
        ),
        set_prior("normal(0, 15)",
          class = "b",
          coef = "pretreatmentC",
          resp = "mass"
        ),
        set_prior("exponential(1.5)",
          class = "sd",
          group = "batch",
          resp = "mass"
        ),
        set_prior("exponential(0.15)",
          class = "sigma",
          resp = "mass"
        ),
        set_prior("normal(0, 2.5)",
          class = "Intercept",
          resp = "tarsus"
        ),
        set_prior("normal(0, 2.5)",
          class = "b",
          coef = "pretreatmentA",
          resp = "tarsus"
        ),
        set_prior("normal(0, 2.5)",
          class = "b",
          coef = "pretreatmentC",
          resp = "tarsus"
        ),
        set_prior("skew_normal(0, 0.25, 5)",
          class = "b",
          coef = "mass",
          resp = "tarsus"
        ),
        set_prior("exponential(2)",
          class = "sd",
          group = "batch",
          resp = "tarsus"
        ),
        set_prior("exponential(1)",
          class = "sigma",
          resp = "tarsus"
        ),
        set_prior("normal(0, 1)",
          class = "Intercept",
          resp = "bill"
        ),
        set_prior("normal(0, 0.5)",
          class = "b",
          coef = "pretreatmentA",
          resp = "bill"
        ),
```

```
        set_prior("normal(0, 0.5)",
          class = "b",
          coef = "pretreatmentC",
          resp = "bill"
        ),
        set_prior("skew_normal(0, 0.25, 5)",
          class = "b",
          coef = "mass",
          resp = "bill"
        ),
        set_prior("exponential(5)",
          class = "sd",
          group = "batch",
          resp = "bill"
        ),
        set_prior("exponential(2.5)",
          class = "sigma",
          resp = "bill"
        ),
        set_prior("normal(0, 0.125)",
          class = "Intercept",
          resp = "slope"
        ),
        set_prior("normal(0, 0.125)",
          class = "b",
          coef = "pretreatmentA",
          resp = "slope"
        ),
        set_prior("normal(0, 0.125)",
          class = "b",
          coef = "pretreatmentC",
          resp = "slope"
        ),
        set_prior("normal(0, 0.004)",
          class = "b",
          coef = "mass",
          resp = "slope"
        ),
        set_prior("normal(0, 0.015)",
          class = "b",
          coef = "tarsus",
          resp = "slope"
        ),
        set_prior("exponential(50)",
          class = "sd",
          group = "batch",
          resp = "slope"
        ),
        set_prior("normal(-3, 1.5)",
          dpar = "sigma",
          class = "Intercept",
          resp = "slope"
        ),
        set_prior("normal(0, 0.5)",
          dpar = "sigma",
          class = "b",
          coef = "batchB",
          resp = "slope"
        ),
        set_prior("normal(1, 1.5)",
          dpar = "sigma",
          class = "b",
          coef = "batchC",
          resp = "slope"
        ),
        set_prior("gamma(5, 1)",
          class = "nu",
```

```
        resp = "slope"
      )
    ),
    iter = 50000, warmup = 10000, cores = 4, chains = 4, thin = 20,
    control = list(adapt_delta = .98, max_treedepth = 14),
    silent = TRUE, refresh = 0,
    file = "./models/_threeWeekFunctionModelHotBillR2.Rds"
  )

functionModel3WeeksHotAppendageR2 <-
  brm(
    data = slopeDataHot3 %>%
      mutate(
        mass = mass - mean(mass, na.rm = T),
        tarsus = tarsusLengthMean -
          mean(tarsusLengthMean, na.rm = T),
        bill = billLengthMean -
          mean(billLengthMean, na.rm = T),
        pretreatment = ifelse(pretreatment == "cold", "A",
          ifelse(pretreatment == "neutral", "B", "C")
        ),
        slope = slope -
          mean(slope, na.rm = T)
      ) %>%
      mutate(pretreatment = factor(pretreatment,
        levels = c("B", "A", "C")
      )) %>%
      drop_na() %>%
      merge(., data %>%
        dplyr::select(ring, "batch" = exp) %>%
        distinct(),
      by = "ring", all.x = TRUE
      ),
    bf(mass ~ pretreatment + (1 | batch),
      family = "gaussian"
    ) +
      bf(tarsus ~ mass + pretreatment + (1 | batch),
        family = "gaussian"
      ) +
      bf(bill ~ mass + pretreatment + (1 | batch),
        family = "gaussian"
      ) +
      bf(
        slope ~ mass + pretreatment + (1 | batch),
        sigma ~ batch,
        family = "student"
      ) +
      set_rescor(FALSE),
    prior = c(
      set_prior("normal(0, 5)",
        class = "Intercept",
        resp = "mass"
      ),
      set_prior("normal(0, 15)",
        class = "b",
        coef = "pretreatmentA",
        resp = "mass"
      ),
      set_prior("normal(0, 15)",
        class = "b",
        coef = "pretreatmentC",
        resp = "mass"
      ),
      set_prior("exponential(1.5)",
        class = "sd",
        group = "batch",
        resp = "mass"
```

```
    ),
    set_prior("exponential(0.15)",
      class = "sigma",
      resp = "mass"
    ),
    set_prior("normal(0, 2.5)",
      class = "Intercept",
      resp = "tarsus"
    ),
    set_prior("normal(0, 2.5)",
      class = "b",
      coef = "pretreatmentA",
      resp = "tarsus"
    ),
    set_prior("normal(0, 2.5)",
      class = "b",
      coef = "pretreatmentC",
      resp = "tarsus"
    ),
    set_prior("skew_normal(0, 0.25, 5)",
      class = "b",
      coef = "mass",
      resp = "tarsus"
    ),
    set_prior("exponential(2)",
      class = "sd",
      group = "batch",
      resp = "tarsus"
    ),
    set_prior("exponential(1)",
      class = "sigma",
      resp = "tarsus"
    ),
    set_prior("normal(0, 1)",
      class = "Intercept",
      resp = "bill"
    ),
    set_prior("normal(0, 0.5)",
      class = "b",
      coef = "pretreatmentA",
      resp = "bill"
    ),
    set_prior("normal(0, 0.5)",
      class = "b",
      coef = "pretreatmentC",
      resp = "bill"
    ),
    set_prior("skew_normal(0, 0.25, 5)",
      class = "b",
      coef = "mass",
      resp = "bill"
    ),
    set_prior("exponential(5)",
      class = "sd",
      group = "batch",
      resp = "bill"
    ),
    set_prior("exponential(2.5)",
      class = "sigma",
      resp = "bill"
    ),
    set_prior("normal(0, 0.125)",
      class = "Intercept",
      resp = "slope"
    ),
    set_prior("normal(0, 0.125)",
      class = "b",
```

```
          coef = "pretreatmentA",
          resp = "slope"
        ),
        set_prior("normal(0, 0.125)",
          class = "b",
          coef = "pretreatmentC",
          resp = "slope"
        ),
        set_prior("normal(0, 0.004)",
          class = "b",
          coef = "mass",
          resp = "slope"
        ),
        set_prior("exponential(50)",
          class = "sd",
          group = "batch",
          resp = "slope"
        ),
        set_prior("normal(-3, 1.5)",
          dpar = "sigma",
          class = "Intercept",
          resp = "slope"
        ),
        set_prior("normal(0, 0.5)",
          dpar = "sigma",
          class = "b",
          coef = "batchB",
          resp = "slope"
        ),
        set_prior("normal(1, 1.5)",
          dpar = "sigma",
          class = "b",
          coef = "batchC",
          resp = "slope"
        ),
        set_prior("gamma(5, 1)",
          class = "nu",
          resp = "slope"
        )
      ),
      iter = 50000, warmup = 10000, cores = 4, chains = 4, thin = 20,
      control = list(adapt_delta = .98, max_treedepth = 14),
      silent = TRUE, refresh = 0,
      file = "./models/_threeWeekFunctionModelHotAppendageR2.Rds"
    )
}

pR2Pull <- function(x) {
  baseR2 <- as.data.frame(
    brms::bayes_R2(functionModel3WeeksHot,
      ndraws = 1000,
      resp = "slope", summary = FALSE,
      robust = TRUE
    )
  )$R2slope
  redR2 <- as.data.frame(
    brms::bayes_R2(x,
      ndraws = 1000,
      resp = "slope", summary = FALSE,
      robust = TRUE
    )
  )$R2slope
  pR2 <- round(baseR2 - redR2, digits = 3)

  ciFrame <- t(
    quantile(pR2, probs = c(0.025, 0.975), type = 8)
  ) %>% as.data.frame()
```

```
  ciFrame <- ciFrame %>%
    mutate(`2.5%` = ifelse(`2.5%` < 0, 0, `2.5%`)) %>%
    mutate("95\\% CI" = paste0(
      "[",
      round(`2.5%`, digits = 3),
      ",",
      round(`97.5%`, digits = 3),
      "]"
    )) %>%
    mutate("Partial R\\textsuperscript{2}" = round(median(pR2), digits = 3)) %>%
    mutate(`Partial R\\textsuperscript{2}` =
             ifelse(`Partial R\\textsuperscript{2}` < 0, 0,
                    `Partial R\\textsuperscript{2}`)
           ) %>%
    dplyr::select(`Partial R\\textsuperscript{2}`, "95\\% CI")

  return(ciFrame)
}

caption = paste0('Variance in metabolic slope ',
  '(fold resting metabolsim at thermoneutrality/°C) explained by ',
  'morphometry in three week old Japanese quail. Metabolic ',
  'slopes are measured above thermoneutrality (<30°C).'
  )

models <- list(functionModel3WeeksHotMassR2, functionModel3WeeksHotTarsusR2,
            functionModel3WeeksHotBillR2, functionModel3WeeksHotAppendageR2)
bind_rows(lapply(models, pR2Pull)) %>%
  mutate("Variable" = c("Body Mass", "Tarsus Length",
                        "Bill Length", "Appendage Length")
         ) %>%
  dplyr::select(Variable, `Partial R\\textsuperscript{2}`, `95\\% CI`) %>%
  kbl(.,
    longtable = T, booktabs = T, format = "latex",
    caption = caption, escape = FALSE
  ) %>%
  column_spec(column = c(1:10), width = "2.5cm")  %>%
  kable_styling(latex_options = "striped")
```

Table 70: *Variance in metabolic slope (fold resting metabolsim at thermoneutrality/°C) explained by morphometry in three week old Japanese quail. Metabolic slopes are measured above thermoneutrality (<30°C).*

Variable	Partial R^2	95% CI
Body Mass	0.015	[0,0.136]
Tarsus Length	0.003	[0,0.126]
Bill Length	0.028	[0,0.142]
Appendage Length	0.041	[0,0.155]

```
rm(models)
```

Similar to analysis of metabolic responses to the cold, we estimate costs of misaligning with Bergmann's and Allen's rule in the heat. This is achieved by calculating the relative increase in fold metabolism among large- (2 standard deviations above mean body mass) and short-limbed (2 standard deviations below mean tarsus length) quail at 40°C compared with average quail.

```
data.frame(
  "Size" = c("Average", "Large (2x s.d. > mean)"),
  "pretreatment" = "B",
  "mass" = c(
    mean(functionModel3WeeksHot$data$mass, na.rm = T),
    mean(functionModel3WeeksHot$data$mass, na.rm = T) +
      2 * sd(functionModel3WeeksHot$data$mass, na.rm = T)
  ),
```

```
  "tarsus" = 0,
  "bill" = 0,
  "batch" = "A"
) %>%
  mutate("slope" = predict(functionModel3WeeksHot,
    newdata = .,
    robust = TRUE, re_form = NA,
    resp = "slope"
  )[, "Estimate"]) %>%
  mutate(slope = slope +
    mean(
      subset(
        slopeDataHot3,
        pretreatment == "neutral"
      )$slope,
      na.rm = T
    )) %>%
  dplyr::select("Body Size" = Size, "Metabolic Slope (fold RMR/°C)" = slope) %>%
  kbl(.,
    longtable = T, booktabs = T, format = "latex",
    caption = paste0("Comparison of metabolic slopes among ",
                     "three week old Japanese quail > 30°C ",
                     "and varying body sizes."),
    escape = FALSE
  ) %>%
  column_spec(column = c(1:10), width = "2.5cm")  %>%
  kable_styling(latex_options = "striped")
```

Table 71: *Comparison of metabolic slopes among three week old Japanese quail > 30°C and varying body sizes.*

Body Size	Metabolic Slope (fold RMR/°C)
Average	0.1363282
Large (2x s.d. > mean)	0.1501585

```
data.frame(
  "Size" = c("Average", "Short (2x s.d. < mean)"),
  "pretreatment" = "B",
  "tarsus" = c(
    mean(functionModel3WeeksHot$data$tarsus, na.rm = T),
    mean(functionModel3WeeksHot$data$tarsus, na.rm = T) -
      2 * sd(functionModel3WeeksHot$data$tarsus, na.rm = T)
  ),
  "mass" = 0,
  "bill" = 0,
  "batch" = "A"
) %>%
  mutate("slope" = predict(functionModel3WeeksHot,
    newdata = .,
    robust = TRUE, re_form = NA,
    resp = "slope"
  )[, "Estimate"]) %>%
  mutate(slope = slope +
        mean(
      subset(
        slopeDataHot3,
        pretreatment == "neutral"
      )$slope,
      na.rm = T
    )) %>%
  dplyr::select("Tarsus Length" = Size,
                "Metabolic Slope (fold RMR/°C)" = slope) %>%
  kbl(.,
```

```
    longtable = T, booktabs = T, format = "latex",
    caption = paste0("Comparison of metabolic slopes among ",
                     "three week old Japanese quail > 30°C ",
                     "and varying tarsus lengths."),
    escape = FALSE
  ) %>%
  column_spec(column = c(1:10), width = "2.5cm")  %>%
  kable_styling(latex_options = "striped")
```

Table 72: *Comparison of metabolic slopes among three week old Japanese quail > 30°C and varying tarsus lengths.*

Tarsus Length	Metabolic Slope (fold RMR/°C)
Average	0.1365908
Short (2x s.d. < mean)	0.1422780

```
data.frame(
  "Size" = c("Average", "Short (2x s.d. < mean)"),
  "pretreatment" = "B",
  "bill" = c(
    mean(functionModel3WeeksHot$data$bill, na.rm = T),
    mean(functionModel3WeeksHot$data$bill, na.rm = T) -
      2 * sd(functionModel3WeeksHot$data$bill, na.rm = T)
  ),
  "mass" = 0,
  "tarsus" = 0,
  "batch" = "A"
) %>%
  mutate("slope" = predict(functionModel3WeeksHot,
    newdata = .,
    robust = TRUE, re_form = NA,
    resp = "slope"
  )[, "Estimate"]) %>%
  mutate(slope = slope +
        mean(
      subset(
        slopeDataHot3,
        pretreatment == "neutral"
      )$slope,
      na.rm = T
    )) %>%
  dplyr::select("Bill Length" = Size,
              "Metabolic Slope (fold RMR/°C)" = slope) %>%
  kbl(.,
    longtable = T, booktabs = T, format = "latex",
    caption = paste0("Comparison of metabolic slopes among ",
                     "three week old Japanese quail > 30°C ",
                     "and varying bill lengths."),
    escape = FALSE
  ) %>%
  column_spec(column = c(1:10), width = "2.5cm")  %>%
  kable_styling(latex_options = "striped")
```

Table 73: *Comparison of metabolic slopes among three week old Japanese quail > 30°C and varying bill lengths.*

Bill Length	Metabolic Slope (fold RMR/°C)
Average	0.1365381
Short (2x s.d. < mean)	0.1553530

```
# Checking whether these differences are statistically clear

mismatchDFMass <- data.frame(
  "Size" = c("Average", "Large (2x s.d. > mean)"),
  "pretreatment" = "B",
  "mass" = c(
    mean(functionModel3WeeksHot$data$mass, na.rm = T),
    mean(functionModel3WeeksHot$data$mass, na.rm = T) +
      2 * sd(functionModel3WeeksHot$data$mass, na.rm = T)
  ),
  "tarsus" = 0,
  "bill" = 0,
  "batch" = "A"
)

mismatchDFTarsus <- data.frame(
  "Size" = c("Average", "Short (2x s.d. < mean)"),
  "pretreatment" = "B",
  "tarsus" = c(
    mean(functionModel3WeeksHot$data$tarsus, na.rm = T),
    mean(functionModel3WeeksHot$data$tarsus, na.rm = T) -
      2 * sd(functionModel3WeeksHot$data$tarsus, na.rm = T)
  ),
  "mass" = 0,
  "bill" = 0,
  "batch" = "A"
)

mismatchDFBill <- data.frame(
  "Size" = c("Average", "Short (2x s.d. < mean)"),
  "pretreatment" = "B",
  "bill" = c(
    mean(functionModel3WeeksHot$data$bill, na.rm = T),
    mean(functionModel3WeeksHot$data$bill, na.rm = T) -
      2 * sd(functionModel3WeeksHot$data$bill, na.rm = T)
  ),
  "mass" = 0,
  "tarsus" = 0,
  "batch" = "A"
)

mismatchTestFun <- function(x) {
  df <- predict(functionModel3WeeksHot,
    newdata = x,
    robust = TRUE, re_form = NA,
    resp = "slope", summary = FALSE
  ) %>%
    as.data.frame() %>%
    rename("Average" = "V1", "Mismatch" = "V2") %>%
    mutate_all(.funs = function(x) {
      x + mean(slopeDataHot3$slope, na.rm = T)
    })

  mismatchDFTest <- build_hdf(
    vars = list(
      df$Average,
      df$Mismatch
    ),
    priors = list(
      rnorm(nrow(df), 0, 0.01),
      rnorm(nrow(df), 0, 0.01)
    ),
    names = c("Average", "Mismatch")
  )
  mismatchTest <- hypothesis_df("Mismatch > Average",
    mismatchDFTest,
    class = "b", alpha = 0.05
```

```
  )

  mismatchTest$hypothesis$Hypothesis <-
    "Mismatch Metabolic Slope > Average Slope"

  return(mismatchTest)
}

mismatchTests <- lapply(
  list(mismatchDFMass, mismatchDFTarsus, mismatchDFBill),
  mismatchTestFun
  )

# Summarising

caption <- paste0(
  "Results of a non-linear hypothesis ",
  "tests comparing predicted metabolic slopes in the warmth (30°C - 40°C) ",
  "among three week old Japanese quail of average or atypically large ",
  "body size (mean + 2 standard deviations). Posterior probabilities are ",
  "calculated using the Savage-Dickey ",
  "density ratio method."
)

mismatchTestMassTable <- mismatchTests[[1]]$hypothesis %>%
  mutate(Hypothesis = c(
    "Large Metabolic Slope > Average Slope"
  )) %>%
  dplyr::select(-c(Evid.Ratio, Star)) %>%
  mutate(
    Estimate = round(Estimate, digits = 4),
    "Est.Error" = round(Est.Error, digits = 4),
    "CI.Lower" = round(CI.Lower, digits = 4),
    "CI.Upper" = round(CI.Upper, digits = 4),
    "Posterior Probability" = round(Post.Prob, digits = 4)
  ) %>%
  rename(
    "Difference Between Metabolic Slopes" = Estimate,
    "Standard Error" = Est.Error,
    `2.5 % CI` = "CI.Lower",
    `97.5 % CI` = "CI.Upper"
  ) %>%
  dplyr::select(-Post.Prob) %>%
  kbl(.,
    longtable = T, booktabs = T, format = "latex",
    caption = caption
  ) %>%
  column_spec(column = c(1:2), width = "2.5cm") %>%
  column_spec(column = c(3:10), width = "2cm") %>%
  kable_styling(latex_options = "striped")

mismatchTestMassTable
```

Table 74: *Results of a non-linear hypothesis tests comparing predicted metabolic slopes in the warmth (30°C - 40°C) among three week old Japanese quail of average or atypically large body size (mean + 2 standard deviations). Posterior probabilities are calculated using the Savage-Dickey density ratio method.*

Hypothesis	Difference Between Metabolic Slopes	Standard Error	2.5 % CI	97.5 % CI	Posterior Probability
Large Metabolic Slope > Average Slope	0.014	0.0343	-0.0406	0.067	0.6888

```
# For tarsus length
```

```r
caption <- paste0(
  "Results of a non-linear hypothesis ",
  "tests comparing predicted metabolic slopes in the warmth (30°C - 40°C) ",
  "among three week old Japanese quail of average or atypically short ",
  "tarsus length (mean - 2 standard deviations). Posterior probabilities are ",
  "calculated using the Savage-Dickey ",
  "density ratio method."
)

mismatchTestTarsusTable <- mismatchTests[[2]]$hypothesis %>%
  mutate(Hypothesis = c(
    "Short Metabolic Slope > Average Slope"
  )) %>%
  dplyr::select(-c(Evid.Ratio, Star)) %>%
  mutate(
    Estimate = round(Estimate, digits = 4),
    "Est.Error" = round(Est.Error, digits = 4),
    "CI.Lower" = round(CI.Lower, digits = 4),
    "CI.Upper" = round(CI.Upper, digits = 4),
    "Posterior Probability" = round(Post.Prob, digits = 4)
  ) %>%
  rename(
    "Difference Between Metabolic Slopes" = Estimate,
    "Standard Error" = Est.Error,
    `2.5 % CI` = "CI.Lower",
    `97.5 % CI` = "CI.Upper"
  ) %>%
  dplyr::select(-Post.Prob) %>%
  kbl(.,
    longtable = T, booktabs = T, format = "latex",
    caption = caption
  ) %>%
  column_spec(column = c(1:2), width = "2.5cm") %>%
  column_spec(column = c(3:10), width = "2cm") %>%
  kable_styling(latex_options = "striped")

mismatchTestTarsusTable
```

Table 75: *Results of a non-linear hypothesis tests comparing predicted metabolic slopes in the warmth (30°C - 40°C) among three week old Japanese quail of average or atypically short tarsus length (mean - 2 standard deviations). Posterior probabilities are calculated using the Savage-Dickey density ratio method.*

Hypothesis	Difference Between Metabolic Slopes	Standard Error	2.5 % CI	97.5 % CI	Posterior Probability
Short Metabolic Slope > Average Slope	0.0052	0.0338	-0.0477	0.0569	0.5715

```r
## Bill length

caption <- paste0(
  "Results of a non-linear hypothesis ",
  "tests comparing predicted metabolic slopes in the warmth (30°C - 40°C) ",
  "among three week old Japanese quail of average or short long ",
  "bill length (mean - 2 standard deviations). Posterior probabilities are ",
  "calculated using the Savage-Dickey ",
  "density ratio method."
)

mismatchTestBillTable <- mismatchTests[[3]]$hypothesis %>%
  mutate(Hypothesis = c(
    "Short Metabolic Slope > Average Slope"
  )) %>%
  dplyr::select(-c(Evid.Ratio, Star)) %>%
  mutate(
```

```
    Estimate = round(Estimate, digits = 4),
    "Est.Error" = round(Est.Error, digits = 4),
    "CI.Lower" = round(CI.Lower, digits = 4),
    "CI.Upper" = round(CI.Upper, digits = 4),
    "Posterior Probability" = round(Post.Prob, digits = 4)
  ) %>%
  rename(
    "Difference Between Metabolic Slopes" = Estimate,
    "Standard Error" = Est.Error,
    `2.5 % CI` = "CI.Lower",
    `97.5 % CI` = "CI.Upper"
  ) %>%
  dplyr::select(-Post.Prob) %>%
  kbl(.,
    longtable = T, booktabs = T, format = "latex",
    caption = caption
  ) %>%
  column_spec(column = c(1:2), width = "2.5cm") %>%
  column_spec(column = c(3:10), width = "2cm") %>%
  kable_styling(latex_options = "striped")

mismatchTestBillTable
```

Table 76: *Results of a non-linear hypothesis tests comparing predicted metabolic slopes in the warmth (30°C - 40°C) among three week old Japanese quail of average or short long bill length (mean - 2 standard deviations). Posterior probabilities are calculated using the Savage-Dickey density ratio method.*

Hypothesis	Difference Between Metabolic Slopes	Standard Error	2.5 % CI	97.5 % CI	Posterior Probability
Short Metabolic Slope > Average Slope	0.0181	0.0355	-0.0373	0.0734	0.7244

Predicted effects of morphology and rearing conditions on metabolic slopes in the heat are next visualised.

```
showtext.auto(enable = TRUE)

p1 <- data.frame(
  "pretreatment" = c("A", "B", "C"),
  "mass" = 0,
  "tarsus" = 0,
  "bill" = 0,
  "batch" = "A"
) %>%
  mutate(
    "slope" = predict(functionModel3WeeksHot,
      newdata = .,
      resp = "slope",
      robust = TRUE,
      re_form = NA
    )[, "Estimate"],
    "SE" = predict(functionModel3WeeksHot,
      newdata = .,
      resp = "slope",
      robust = TRUE,
      re_form = NA
    )[, "Est.Error"]
  ) %>%
  mutate(slope = slope +
    mean(slopeDataHot3$slope,
      na.rm = T
    )) %>%
  ggplot(aes(x = pretreatment, y = slope)) +
  geom_errorbar(
```

```
    aes(
      x = pretreatment, ymin = slope - SE,
      ymax = slope + SE
    ),
    colour = "black", width = 0.3
  ) +
  geom_point(
    size = 5, pch = 21, colour = "black",
    aes(x = pretreatment, fill = factor(pretreatment))
  ) +
  geom_line(linetype = "dashed", colour = "black") +
  geom_point(
    size = 2, pch = 21, colour = "black",
    data = slopeDataHot3 %>%
      mutate(
        "pretreatment" =
          ifelse(pretreatment == "cold", "A",
            ifelse(pretreatment == "neutral", "B", "C")
          )
      ),
    aes(
      x = pretreatment, y = slope,
      fill = factor(pretreatment)
    ),
    position = position_jitter(width = 0.3)
  ) +
  scale_fill_manual(values = c("#7BB4E3", "black", "#CD5C5C")) +
  scale_x_discrete(
    breaks = c("A", "B", "C"),
    labels = c(
      "Cold\n(10°C)",
      "Mild\n(20°C)",
      "Warm\n(30°C)"
    )
  ) +
  theme_classic() +
  theme(
    legend.position = "none",
    axis.text = element_text(family = "Noto Sans"),
    axis.title = element_text(family = "Noto Sans")
  ) +
  xlab("Rearing Conditions") +
  ylab("Metabolic Slope\n(Fold Metabolism\nat Thermoneutrality/°C)")

p2 <- data.frame(
  "pretreatment" = "B",
  "mass" = seq(min(functionModel3WeeksHot$data$mass, na.rm = T),
    max(functionModel3WeeksHot$data$mass, na.rm = T),
    by = 0.1
  ),
  "tarsus" = 0,
  "bill" = 0,
  "batch" = "A"
) %>%
  mutate(
    "slope" = predict(functionModel3WeeksHot,
      newdata = .,
      resp = "slope",
      robust = TRUE,
      re_form = NA
    )[, "Estimate"],
    "SE" = predict(functionModel3WeeksHot,
      newdata = .,
      resp = "slope",
      robust = TRUE,
      re_form = NA
    )[, "Est.Error"]
```

```
  ) %>%
  mutate("Mass" = mass +
    mean(slopeDataHot3$mass, na.rm = T)) %>%
  mutate("slope" = slope +
    mean(slopeDataHot3$slope, na.rm = T)) %>%
  ggplot(aes(x = Mass, y = slope)) +
  geom_ribbon(
    aes(
      x = Mass, ymin = slope - SE,
      ymax = slope + SE
    ),
    fill = "grey50", alpha = 0.5
  ) +
  geom_smooth(
    method = "lm", linetype = "dashed",
    se = FALSE, colour = "black"
  ) +
  geom_point(
    size = 2.5, pch = 21, colour = "black", alpha = 0.5, fill = "grey50",
    data = slopeDataHot3,
    aes(x = mass, y = slope)
  ) +
  theme_classic() +
  theme(
    legend.position = "none",
    axis.text = element_text(family = "Noto Sans"),
    axis.title = element_text(family = "Noto Sans")
  ) +
  xlab("Body Mass (g)") +
  ylab("Metabolic Slope\n(Fold Metabolism at\nThermoneutrality/°C)")

p3 <- data.frame(
  "pretreatment" = "B",
  "tarsus" = seq(min(functionModel3WeeksHot$data$tarsus, na.rm = T),
    max(functionModel3WeeksHot$data$tarsus, na.rm = T),
    by = 0.1
  ),
  "mass" = 0,
  "bill" = 0,
  "batch" = "A"
) %>%
  mutate(
    "slope" = predict(functionModel3WeeksHot,
      newdata = .,
      resp = "slope",
      robust = TRUE,
      re_form = NA
    )[, "Estimate"],
    "SE" = predict(functionModel3WeeksHot,
      newdata = .,
      resp = "slope",
      robust = TRUE,
      re_form = NA
    )[, "Est.Error"]
  ) %>%
  mutate("Tarsus" = tarsus +
    mean(slopeDataHot3$tarsusLengthMean, na.rm = T)) %>%
  mutate("slope" = slope +
    mean(slopeDataHot3$slope, na.rm = T)) %>%
  ggplot(aes(x = Tarsus, y = slope)) +
  geom_ribbon(
    aes(
      x = Tarsus, ymin = slope - SE,
      ymax = slope + SE
    ),
    fill = "grey50", alpha = 0.5
  ) +
```

```
  geom_smooth(
    method = "lm", linetype = "dashed",
    se = FALSE, colour = "black"
  ) +
  geom_point(
    size = 2.5, pch = 21, colour = "black",
    alpha = 0.5, fill = "grey50",
    data = slopeDataHot3,
    aes(x = tarsusLengthMean, y = slope)
  ) +
  theme_classic() +
  theme(
    legend.position = "none",
    axis.text = element_text(family = "Noto Sans"),
    axis.title = element_text(family = "Noto Sans")
  ) +
  xlab("Tarsus Length (mm)") +
  ylab("Metabolic Slope\n(Fold Metabolism at\nThermoneutrality/°C)")

p4 <- data.frame(
  "pretreatment" = "B",
  "bill" = seq(min(functionModel3WeeksHot$data$bill, na.rm = T),
    max(functionModel3WeeksHot$data$bill, na.rm = T),
    by = 0.1
  ),
  "mass" = 0,
  "tarsus" = 0,
  "batch" = "A"
) %>%
  mutate(
    "slope" = predict(functionModel3WeeksHot,
      newdata = .,
      resp = "slope",
      robust = TRUE,
      re_form = NA
    )[, "Estimate"],
    "SE" = predict(functionModel3WeeksHot,
      newdata = .,
      resp = "slope",
      robust = TRUE,
      re_form = NA
    )[, "Est.Error"]
  ) %>%
  mutate("Bill" = bill +
    mean(slopeDataHot3$billLengthMean, na.rm = T)) %>%
  mutate("slope" = slope +
    mean(slopeDataHot3$slope, na.rm = T)) %>%
  ggplot(aes(x = Bill, y = slope)) +
  geom_ribbon(
    aes(
      x = Bill, ymin = slope - SE,
      ymax = slope + SE
    ),
    fill = "grey50", alpha = 0.5
  ) +
  geom_smooth(
    method = "lm", linetype = "dashed",
    se = FALSE, colour = "black"
  ) +
  geom_point(
    size = 2.5, pch = 21, colour = "black",
    alpha = 0.5, fill = "grey50",
    data = slopeDataHot3,
    aes(x = billLengthMean, y = slope)
  ) +
  theme_classic() +
  theme(
```

```
    legend.position = "none",
    axis.text = element_text(family = "Noto Sans"),
    axis.title = element_text(family = "Noto Sans")
  ) +
  xlab("Bill Length (mm)") +
  ylab("Metabolic Slope\n(Fold Metabolism at\nThermoneutrality/°C)")

massPred <- data.frame(
  "pretreatment" = "B",
  "mass" = c(
    mean(functionModel3WeeksHot$data$mass, na.rm = T) -
      sd(functionModel3WeeksHot$data$mass, na.rm = T),
    mean(functionModel3WeeksHot$data$mass, na.rm = T),
    mean(functionModel3WeeksHot$data$mass, na.rm = T) +
      sd(functionModel3WeeksHot$data$mass, na.rm = T)
  ),
  "tarsus" = 0,
  "bill" = 0,
  "batch" = "A"
) %>%
  predict(functionModel3WeeksHot,
    re_form = NA,
    resp = "slope", newdata = .,
    summary = TRUE, robust = TRUE
  ) %>%
  as.data.frame() %>%
  mutate("slope" = Estimate +
    mean(
      subset(
        slopeDataHot3,
        week == 3 & pretreatment == "neutral"
      )$slope,
      na.rm = TRUE
    )) %>%
  mutate("Size" = c("Small", "Average", "Large")) %>%
  select(Size, slope, "slopeSE" = Est.Error) %>%
  slice(rep(1:n(), each = 2)) %>%
  mutate("Ta" = rep(c(30, 40), 3)) %>%
  mutate(
    "foldRMR" = ifelse(Ta == 30, 1, 10 * slope),
    "LL" = ifelse(Ta == 30, 1, 10 * (slope - slopeSE)),
    "UL" = ifelse(Ta == 30, 1, 10 * (slope + slopeSE))
  ) %>%
  mutate(Size = factor(Size, levels = c("Small", "Average", "Large")))

massPlot <- massPred %>%
  filter(Ta == 40) %>%
  ggplot(aes(x = Size, y = foldRMR)) +
  geom_point(
    data = modDataHot %>%
      select(ring, mass, Ta, VO2) %>%
      pivot_wider(
        id_cols = c("ring", "mass"),
        names_from = "Ta",
        values_from = "VO2"
      ) %>%
      mutate(
        "foldRMR" = `40` / `30`,
        "Ta" = 40,
        "Size" = ifelse(mass < mean(mass, na.rm = T) -
          sd(mass, na.rm = T),
        "Small",
        ifelse(mass > mean(mass, na.rm = T) +
          sd(mass, na.rm = T),
        "Large", "Average"
        )
        )
```

```
      ) %>%
      mutate(Size = factor(Size, levels = c("Small", "Average", "Large"))),
    aes(x = Size, y = foldRMR, colour = mass),
    position = position_jitter(width = 0.25), alpha = 0.5
  ) +
  geom_hline(yintercept = 1, colour = "grey30", linetype = "dashed") +
  geom_errorbar(aes(ymin = LL, ymax = UL),
    colour = "grey80",
    width = 0.25
  ) +
  geom_errorbar(aes(ymin = LL, ymax = UL),
    colour = "black",
    width = 0.25
  ) +
  geom_point(aes(x = Size, y = foldRMR, fill = Size),
    size = 4, colour = "black", pch = 21
  ) +
  ylim(c(0, 4.5)) +
  theme_classic() +
  ylab("Fold Resting\nMetabolism") +
  scale_fill_manual(
    values = c("#DECCC1", "#855E46", "#231709"),
    guide = NULL
  ) +
  scale_colour_gradient(
    low = "#DECCC1", high = "#231709",
    name = "Body Mass (g)"
  ) +
  scale_x_discrete(
    labels = c("Mean - 1 SD", "Mean", "Mean + 1 SD"),
    name = "Body Mass"
  ) +
  theme(
    axis.text = element_text(family = "Noto Sans"),
    axis.title = element_text(family = "Noto Sans"),
    legend.text = element_text(family = "Noto Sans", size = 8),
    legend.title = element_text(family = "Noto Sans", size = 8),
    legend.position = "right"
  )

rm(massPred)

tarsusPred <- data.frame(
  "pretreatment" = "B",
  "tarsus" = c(
    mean(functionModel3WeeksHot$data$tarsus, na.rm = T) -
      sd(functionModel3WeeksHot$data$tarsus, na.rm = T),
    mean(functionModel3WeeksHot$data$tarsus, na.rm = T),
    mean(functionModel3WeeksHot$data$tarsus, na.rm = T) +
      sd(functionModel3WeeksHot$data$tarsus, na.rm = T)
  ),
  "mass" = 0,
  "bill" = 0,
  "batch" = "A"
) %>%
  predict(functionModel3WeeksHot,
    re_form = NA,
    resp = "slope", newdata = .,
    summary = TRUE, robust = TRUE
  ) %>%
  as.data.frame() %>%
  mutate("slope" = Estimate +
    mean(
      subset(
        slopeDataHot3,
        pretreatment == "neutral"
      )$slope,
```

```
      na.rm = TRUE
    )) %>%
  mutate("Size" = c("Small", "Average", "Large")) %>%
  select(Size, slope, "slopeSE" = Est.Error) %>%
  slice(rep(1:n(), each = 2)) %>%
  mutate("Ta" = rep(c(30, 40), 3)) %>%
  mutate(
    "foldRMR" = ifelse(Ta == 30, 1, 10 * slope),
    "LL" = ifelse(Ta == 30, 1, 10 * (slope - slopeSE)),
    "UL" = ifelse(Ta == 30, 1, 10 * (slope + slopeSE))
  ) %>%
  mutate(Size = factor(Size, levels = c("Small", "Average", "Large")))

tarsusPlot <- tarsusPred %>%
  filter(Ta == 40) %>%
  ggplot(aes(x = Size, y = foldRMR)) +
  geom_point(
    data = modDataHot %>%
      select(ring, "tarsus" = tarsusLengthMean, Ta, VO2) %>%
      pivot_wider(
        id_cols = c("ring", "tarsus"),
        names_from = "Ta",
        values_from = "VO2"
      ) %>%
      mutate(
        "foldRMR" = `40` / `30`,
        "Ta" = 40,
        "Size" = ifelse(tarsus < mean(tarsus, na.rm = T) -
          sd(tarsus, na.rm = T),
        "Small",
        ifelse(tarsus > mean(tarsus, na.rm = T) +
          sd(tarsus, na.rm = T),
        "Large", "Average"
        )
        )
      ) %>%
      drop_na() %>%
      mutate(Size = factor(Size, levels = c("Small", "Average", "Large"))),
    aes(x = Size, y = foldRMR, colour = tarsus),
    position = position_jitter(width = 0.25), alpha = 0.5
  ) +
  geom_hline(yintercept = 1, colour = "grey30", linetype = "dashed") +
  geom_errorbar(aes(ymin = LL, ymax = UL),
    colour = "grey80",
    width = 0.25
  ) +
  geom_errorbar(aes(ymin = LL, ymax = UL),
    colour = "black",
    width = 0.25
  ) +
  geom_point(aes(x = Size, y = foldRMR, fill = Size),
    size = 4, colour = "black", pch = 21
  ) +
  theme_classic() +
  xlab("Ambient Temperature (°C)") +
  ylab("Fold Resting\nMetabolism") +
  ylim(c(0, 4.5)) +
  scale_fill_manual(
    values = c("#DECCC1", "#855E46", "#231709"),
    guide = NULL
  ) +
  scale_colour_gradient(
    low = "#DECCC1", high = "#231709",
    name = "Tarsus Length\n(mm)"
  ) +
  theme(
    axis.text = element_text(family = "Noto Sans"),
```

```
    axis.title = element_text(family = "Noto Sans"),
    legend.text = element_text(family = "Noto Sans", size = 8),
    legend.title = element_text(family = "Noto Sans", size = 8),
    legend.position = "right"
  )

billPred <- data.frame(
  "pretreatment" = "B",
  "bill" = c(
    mean(functionModel3WeeksHot$data$bill, na.rm = T) -
      sd(functionModel3WeeksHot$data$bill, na.rm = T),
    mean(functionModel3WeeksHot$data$bill, na.rm = T),
    mean(functionModel3WeeksHot$data$bill, na.rm = T) +
      sd(functionModel3WeeksHot$data$bill, na.rm = T)
  ),
  "mass" = 0,
  "tarsus" = 0,
  "batch" = "A"
) %>%
  predict(functionModel3WeeksHot,
    re_form = NA,
    resp = "slope", newdata = .,
    summary = TRUE, robust = TRUE
  ) %>%
  as.data.frame() %>%
  mutate("slope" = Estimate +
    mean(
      subset(
        slopeDataHot3,
        pretreatment == "neutral"
      )$slope,
      na.rm = TRUE
    )) %>%
  mutate("Size" = c("Small", "Average", "Large")) %>%
  select(Size, slope, "slopeSE" = Est.Error) %>%
  slice(rep(1:n(), each = 2)) %>%
  mutate("Ta" = rep(c(30, 40), 3)) %>%
  mutate(
    "foldRMR" = ifelse(Ta == 30, 1, 10 * slope),
    "LL" = ifelse(Ta == 30, 1, 10 * (slope - slopeSE)),
    "UL" = ifelse(Ta == 30, 1, 10 * (slope + slopeSE))
  ) %>%
  mutate(Size = factor(Size, levels = c("Small", "Average", "Large")))

billPlot <- billPred %>%
  filter(Ta == 40) %>%
  ggplot(aes(x = Size, y = foldRMR)) +
  geom_point(
    data = modDataHot %>%
      select(ring, "bill" = billLengthMean, Ta, VO2) %>%
      pivot_wider(
        id_cols = c("ring", "bill"),
        names_from = "Ta",
        values_from = "VO2"
      ) %>%
      mutate(
        "foldRMR" = `40` / `30`,
        "Ta" = 40,
        "Size" = ifelse(bill < mean(bill, na.rm = T) -
          sd(bill, na.rm = T),
        "Small",
        ifelse(bill > mean(bill, na.rm = T) +
          sd(bill, na.rm = T),
        "Large", "Average"
        )
        )
      ) %>%
```

```
      drop_na() %>%
      mutate(Size = factor(Size, levels = c("Small", "Average", "Large"))),
    aes(x = Size, y = foldRMR, colour = bill),
    position = position_jitter(width = 0.25), alpha = 0.5
  ) +
  geom_hline(yintercept = 1, colour = "grey30", linetype = "dashed") +
  geom_errorbar(aes(ymin = LL, ymax = UL),
    colour = "grey80",
    width = 0.25
  ) +
  geom_errorbar(aes(ymin = LL, ymax = UL),
    colour = "black",
    width = 0.25
  ) +
  geom_point(aes(x = Size, y = foldRMR, fill = Size),
    size = 4, colour = "black", pch = 21
  ) +
  theme_classic() +
  xlab("Ambient Temperature (°C)") +
  ylab("Fold Resting\nMetabolism") +
  ylim(c(0, 4.5)) +
  scale_fill_manual(
    values = c("#DECCC1", "#855E46", "#231709"),
    guide = NULL
  ) +
  scale_colour_gradient(
    low = "#DECCC1", high = "#231709",
    name = "Bill Length\n(mm)"
  ) +
  theme(
    axis.text = element_text(family = "Noto Sans"),
    axis.title = element_text(family = "Noto Sans"),
    legend.text = element_text(family = "Noto Sans", size = 8),
    legend.title = element_text(family = "Noto Sans", size = 8),
    legend.position = "right"
  )

allMorphology <- (p1 / (p2 + massPlot) / (p3 + tarsusPlot) / (p4 + billPlot)) +
  plot_annotation(tag_levels = "A")

allMorphology
```

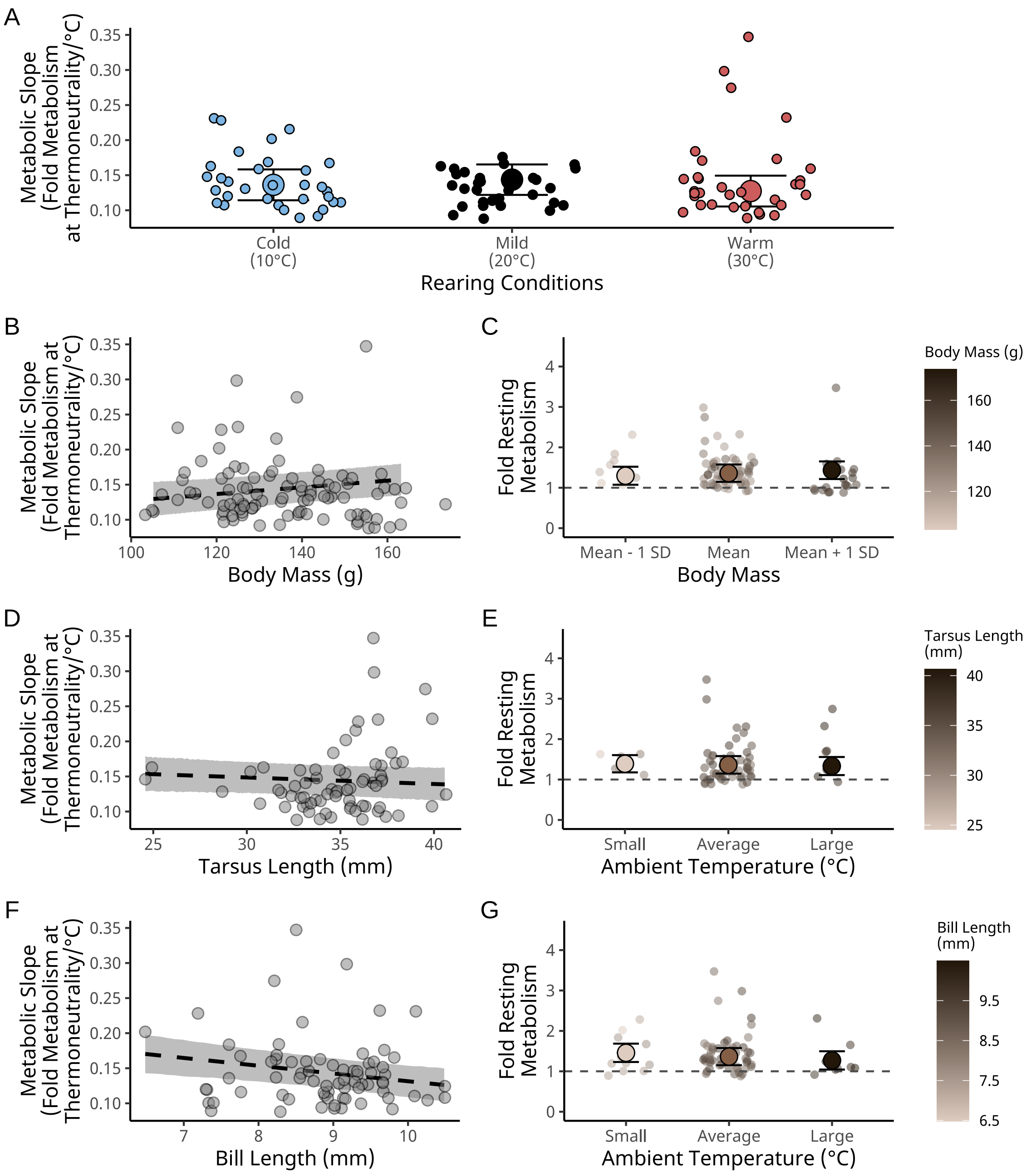

Figure 129: *Conditional effects of post-hatch rearing environments (10°C, 20°C or 30°C) and morphometry (body mass [g], tarsus length [mm], bill length [mm]) on physiological responses to heat in three week old Japanese quail. Dots represent raw data points per individual. Large dots in panels C, E, and G represent estimated effects at average morphometric values and errorbars indicate +/- one standard error around means. Lines in panels B, D and F indicate estimate effects across given parameters and grouping (assuming mild [20°C] rearing conditions) and ribbons indicate standard errors around effects. All estimated effects are drawn from a Bayesian path analysis.*

```
ggsave("./plots/morphologyEffectsResistanceHeat3Weeks_3.pdf",
  allMorphology,
  dpi = 800, height = 7, width = 9
)

showtext.auto(enable = "FALSE")
```

Modelling for adults

Next, the above analysis is repeated for data derived from eight week old Japanese quail. Model parameterisation and priors remain as described but with slight modifications described below within this subsection. We begin by calculating metabolic slopes manually and visualising resulting values for oddities. Then, raw trends in the data are inspected with plots, and our model constructed.

```
birdVector <- merge(
  all %>%
    drop_na(VO2) %>%
    filter(week == 8 & Ta == 30) %>%
    select(ring) %>%
    distinct(),
  all %>%
    drop_na(VO2) %>%
    filter(week == 8 & Ta == 40) %>%
    select(ring) %>%
    distinct(),
  by = "ring", all = FALSE
) %>% pull(ring)

modData8 <- subset(all, week == 8 & Ta > 25 &
                     ring %in% birdVector)

# Calculating metabolic slopes for each
# bird. Again, 30°C is set as 0 to root slopes.

slopeDataHot8 <- modData8 %>%
  dplyr::select(ring, Ta, VO2) %>%
  pivot_wider(id_cols = ring, names_from = Ta,
              values_from = VO2) %>%
  mutate(
    slope = (`40`/`30`)/10
  ) %>%
  dplyr::select(ring, slope) %>%
  merge(modData8 %>%
    dplyr::select(
      ring, sex, exp, week, pretreatment,
      posttreatment, treatment, mass, wingLength,
      tarsusLengthMean, billLengthMean
    ) %>%
    distinct(), ., by = "ring")

# Building dotplot

slopeDataHot8 %>%
  ggplot(aes(x = 1:nrow(.), y = slope)) +
  geom_rect(
    colour = "black", fill = "grey80", alpha = 0.5,
    aes(
      xmin = -Inf, xmax = Inf,
      ymin = mean(slope, na.rm = T) -
        3.5 * sd(slope, na.rm = T),
      ymax = mean(slope, na.rm = T) +
        3.5 * sd(slope, na.rm = T)
    )
  ) +
  geom_point() +
  xlab("Sample Number") +
  ylab("Metabolic Slope\n(Fold Resting Metabolism/°C)") +
  theme_classic()
```

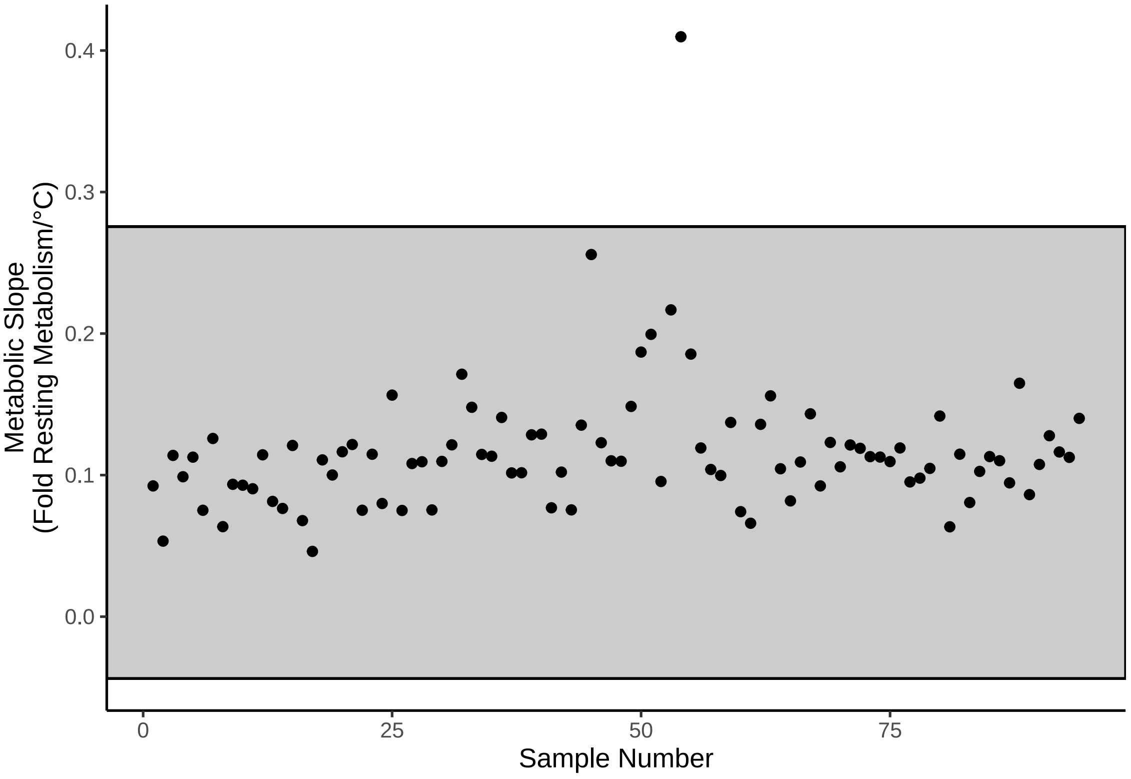

Figure 130: *Cleveland dotplot displaying metabolic slope values of eight week old Japanese quail at 30°C - 40°C. Dots represent raw values and the grey rectangle represents the range covered by mean metabolic slope +/- 3.5x the standard deviation.*

Again, a potential outlier evident in our dotplot is investigated.

```
# Identifying.

caption <- paste0(
  "Potential metabolic slope outlier ",
  "among eight week old Japanese quail with metabolism ",
  "measured at ambient temperatures between 30°C and 40°C."
)

slopeDataHot8 %>%
  filter(slope > 0.4) %>%
  merge(., modData8 %>%
    dplyr::select(ring, Ta, VO2),
  by = "ring", all.x = TRUE
  ) %>%
  mutate(pretreatment = ifelse(pretreatment == "cold",
    "Cold (10°C)",
    ifelse(pretreatment == "warm",
      "Warm (30°C)", "Mild (20°C)"
    )
  )) %>%
  dplyr::select(
    "Bird Identity" = ring,
    "Rearing Conditions" = pretreatment,
    "Mass (g)" = mass,
    "Tarsus Length (mm)" = tarsusLengthMean,
    "Bill Length (mm)" = billLengthMean,
    "Ambient Temperature (°C)" = Ta,
    "Metabolic Slope\n(Fold Resting Metabolism at Thermoneutrality/°C)" =
```

```
    slope
  ) %>%
  kbl(.,
    longtable = T, booktabs = T,
    format = "latex", escape = FALSE,
    caption = caption
  ) %>%
  column_spec(column = c(2, 4:7), width = "2.1cm") %>%
  kable_styling(latex_options = "striped")
```

Table 77: *Potential metabolic slope outlier among eight week old Japanese quail with metabolism measured at ambient temperatures between 30°C and 40°C.*

Bird Identity	Rearing Conditions	Mass (g)	Tarsus Length (mm)	Bill Length (mm)	Ambient Temperature (°C)	Metabolic Slope (Fold Resting Metabolism at Thermoneutrality/°C)
LLW26	Warm (30°C)	306	37.58428	9.348	30	0.4097247
LLW26	Warm (30°C)	306	37.58428	9.348	40	0.4097247

The individual from which this extreme point came was also flagged as extreme at three weeks of age as well. At this time, however, its response now lays more than six standard deviations above the mean. Below, we retain the value but again check its relative importance on our analysis outcomes, again, using Pareto K as our metric of importance.

```
# Visualising data

p1 <- ggplot(slopeDataHot8, aes(x = slope)) +
  geom_density(colour = "black", fill = "grey50", alpha = 0.5) +
  theme_classic() +
  xlab(
    paste0(
      "Metabolic Slope\n(Fold Resting ",
      "Metabolism at\nThermoneutrality/°C)"
    )
  ) +
  ylab("Density")

p2<- ggplot(
  modData8 %>%
    mutate(
      "massGroup" =
        ifelse(mass <=
          mean(mass, na.rm = T) -
            sd(mass, na.rm = T),
        "Small",
        ifelse(mass >=
          mean(mass, na.rm = T) +
            sd(mass, na.rm = T),
        "Large", "Average"
        )
        )
    ) %>%
    mutate(
      massGroup =
        factor(massGroup,
          levels = c("Small", "Average", "Large")
        )
    ) %>%
    mutate(
      pretreatment =
        ifelse(pretreatment == "cold", "Cold\n(10°C)",
```

```
          ifelse(pretreatment == "neutral", "Mild\n(20°C)",
            "Warm\n(30°C)"
          )
        )
    ),
  aes(x = Ta, y = VO2, group = ring, colour = massGroup)
) +
  facet_wrap(~pretreatment) +
  geom_line(aes(size = massGroup)) +
  scale_size_manual(
    values = c("Small" = 0.5, "Average" = 1, "Large" = 2),
    name = "Relative Body\nMass",
    labels = c("< Mean - 1SD", "Mean", "> Mean + 1SD"),
  ) +
  scale_colour_manual(
    values = c(
      "Small" = "grey80",
      "Average" = "grey30",
      "Large" = "black"
    ),
    name = "Relative Body\nMass",
    labels = c("< Mean - 1SD", "Mean", "> Mean + 1SD"),
  ) +
  scale_x_continuous(breaks = c(30, 40)) +
  theme_classic() +
  theme(legend.position = "bottom") +
  xlab("Ambient Temperature (°C)") +
  ylab(
    TeX(
      "$\\overset{Resting~Metabolism}{(mL~O_{2}/min)}$"
    )
  )

p3 <- ggplot(
  modData8 %>%
    mutate(
      "tarsusGroup" =
        ifelse(tarsusLengthMean <
          mean(tarsusLengthMean, na.rm = T) -
            sd(tarsusLengthMean, na.rm = T),
        "Small",
        ifelse(tarsusLengthMean >
          mean(tarsusLengthMean, na.rm = T) +
            sd(tarsusLengthMean, na.rm = T),
        "Large", "Average"
        )
        )
    ) %>%
    mutate(
      tarsusGroup =
        factor(tarsusGroup,
          levels = c("Small", "Average", "Large")
        )
    ) %>%
    mutate(pretreatment = ifelse(pretreatment == "cold",
      "Cold\n(10°C)",
      ifelse(pretreatment == "neutral", "Mild\n(20°C)",
        "Warm\n(30°C)"
      )
    )),
  aes(x = Ta, y = VO2, group = ring, colour = tarsusGroup)
) +
  facet_wrap(~pretreatment) +
  geom_line(aes(size = tarsusGroup)) +
  scale_size_manual(
    values = c("Small" = 0.5, "Average" = 1, "Large" = 2),
    labels = c("< Mean - 1SD", "Mean", "> Mean + 1SD"),
```

```
    name = "Relative Tarsus\nLength"
  ) +
  scale_colour_manual(
    values = c(
      "Small" = "grey80",
      "Average" = "grey30",
      "Large" = "black"
    ),
    name = "Relative Tarsus\nLength",
    labels = c("< Mean - 1SD", "Mean", "> Mean + 1SD"),
  ) +
  scale_x_continuous(breaks = c(30, 40)) +
  theme_classic() +
  theme(legend.position = "bottom") +
  xlab("Ambient Temperature (°C)") +
  ylab(
    TeX(
      "$\\overset{Resting~Metabolism}{(mL~O_{2}/min)}$"
    )
  )

p4 <- ggplot(
  modData8 %>%
    mutate(
      "billGroup" =
        ifelse(billLengthMean <
          mean(billLengthMean, na.rm = T) -
            sd(billLengthMean, na.rm = T),
        "Small",
        ifelse(billLengthMean >
          mean(billLengthMean, na.rm = T) +
            sd(billLengthMean, na.rm = T),
        "Large", "Average"
        )
        )
    ) %>%
    mutate(
      billGroup =
        factor(billGroup,
          levels = c("Small", "Average", "Large")
        )
    ) %>%
    mutate(pretreatment = ifelse(pretreatment == "cold",
      "Cold\n(10°C)",
      ifelse(pretreatment == "neutral", "Mild\n(20°C)",
        "Warm\n(30°C)"
      )
    )),
  aes(x = Ta, y = VO2, group = ring, colour = billGroup)
) +
  facet_wrap(~pretreatment) +
  geom_line(aes(size = billGroup)) +
  scale_size_manual(
    values = c("Small" = 0.5, "Average" = 1, "Large" = 2),
    labels = c("< Mean - 1SD", "Mean", "> Mean + 1SD"),
    name = "Relative Bill\nLength"
  ) +
  scale_colour_manual(
    values = c(
      "Small" = "grey80",
      "Average" = "grey30",
      "Large" = "black"
    ),
    name = "Relative Bill\nLength",
    labels = c("< Mean - 1SD", "Mean", "> Mean + 1SD"),
  ) +
  scale_x_continuous(breaks = c(30, 40)) +
```

```
  theme_classic() +
  theme(legend.position = "bottom") +
  xlab("Ambient Temperature (°C)") +
  ylab(
    TeX(
      "$\\overset{Resting~Metabolism}{(mL~O_{2}/min)}$"
    )
  )

(p1 | (p2/p3/p4)) +
  plot_annotation(tag_levels = "A")
```

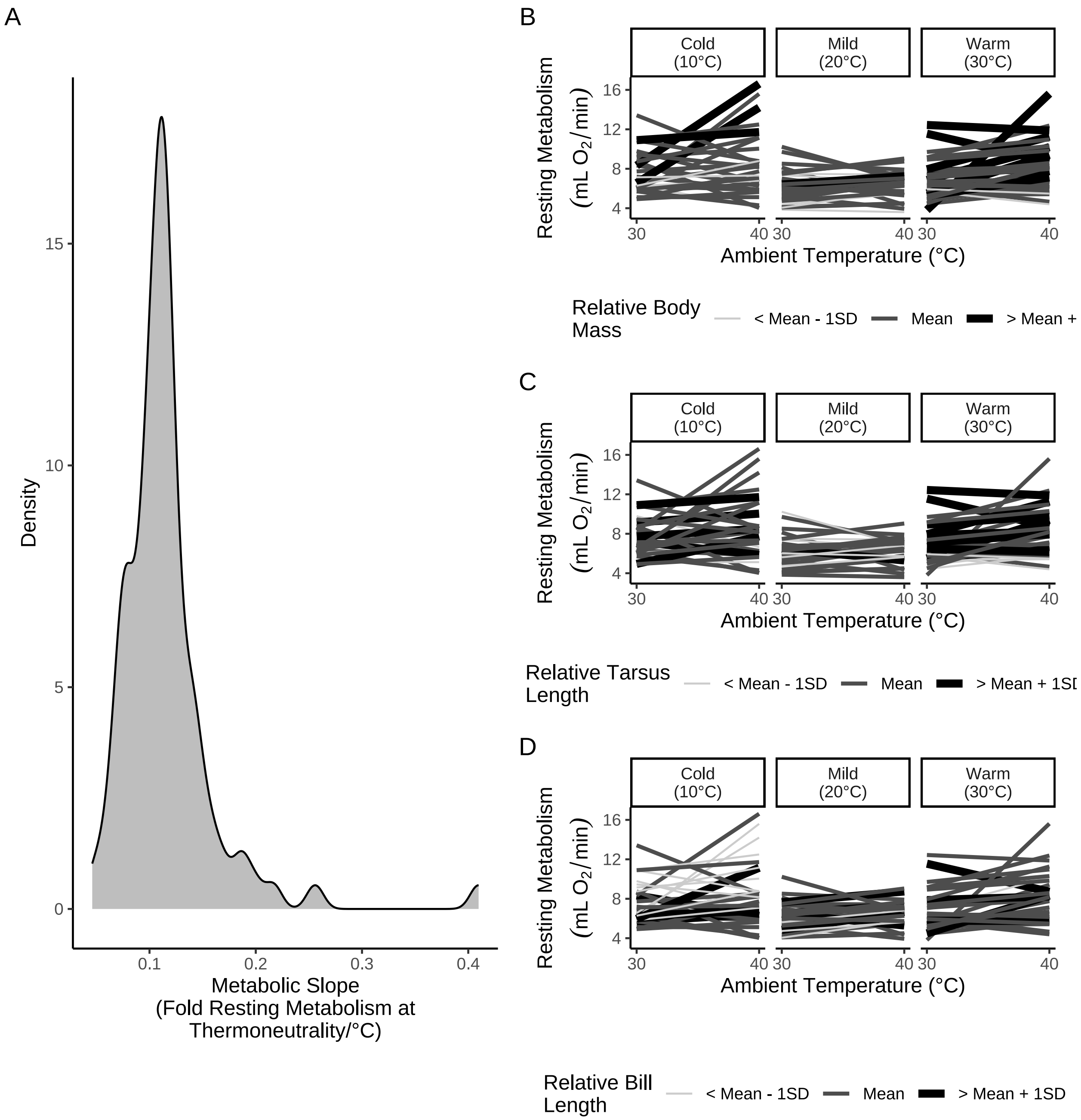

Figure 131: *Thermal physiology of eight week old Japanese quail at ambient temperature within and about thermoneutral (30°C). Panel A displays the density of metabolic slopes at this temperature range. Panels B and C display correlations between ambient temperature and resting metabolism. Each line represents one individual with line colour and line size scaled by relative size (tarsus length [mm] or body mass [g]); data is divided by rearing conditions up to at least three months of age.*

```
p1 <- ggplot(
  slopeDataHot8,
  aes(x = mass, y = slope)
) +
  geom_point(
    pch = 21, size = 1.5,
    colour = "black", fill = "grey50"
  ) +
```

```
  geom_smooth(
    method = "lm", se = FALSE,
    colour = "black", linetype = "dashed"
  ) +
  xlab("Body Mass (g)") +
  ylab("Metabolic Slope\n(Fold Metabolism at\nThermoneutrality/°C)") +
  theme_classic()

p2 <- ggplot(
  slopeDataHot8,
  aes(x = tarsusLengthMean, y = slope)
) +
  geom_point(
    pch = 21, size = 1.5,
    colour = "black", fill = "grey50"
  ) +
  geom_smooth(
    method = "lm", se = FALSE,
    colour = "black", linetype = "dashed"
  ) +
  theme_classic() +
  scale_x_continuous(n.breaks = 4) +
  theme(
    axis.title.y = element_blank(),
    axis.text.y = element_blank(),
    axis.ticks.y = element_blank(),
    axis.line.y = element_blank()
  )

p3 <- ggplot(
  slopeDataHot8,
  aes(x = billLengthMean, y = slope)
) +
  geom_point(
    pch = 21, size = 1.5,
    colour = "black", fill = "grey50"
  ) +
  geom_smooth(
    method = "lm", se = FALSE,
    colour = "black", linetype = "dashed"
  ) +
  theme_classic() +
  scale_x_continuous(n.breaks = 4) +
  xlab("Bill Length\n(mm)") +
  ylab("Metabolic Slope\n(Fold Metabolism at\nThermoneutrality/°C)")

p4 <- ggplot(
  slopeDataHot8 %>%
    drop_na(tarsusLengthMean, mass) %>%
    mutate("tarsusResiduals" = residuals(
      brm(tarsusLengthMean ~ mass,
        data = .,
        prior = c(set_prior("gamma(1, 1)",
          class = "b", coef = "mass"
        )),
        iter = 50000, warmup = 5000,
        cores = 4, chains = 4,
        silent = TRUE, refresh = 0,
        file = "./models/_resPlotModel8WeeksTarsus.Rds"
      )
    )[, "Estimate"]),
  aes(x = tarsusResiduals, y = slope)
) +
  geom_point(
    pch = 21, size = 1.5,
    colour = "black", fill = "grey50"
  ) +
```

```
  geom_smooth(
    method = "lm", se = FALSE,
    colour = "black", linetype = "dashed"
  ) +
  xlab("Residual Tarsus\nLength (mm)") +
  theme_classic() +
  theme(
    axis.title.y = element_blank(),
    axis.text.y = element_blank(),
    axis.ticks.y = element_blank(),
    axis.line.y = element_blank()
  )

p5 <- ggplot(
  slopeDataHot8 %>%
    drop_na(billLengthMean, mass) %>%
    mutate("billResiduals" = residuals(
      brm(billLengthMean ~ mass,
        data = .,
        prior = c(set_prior("normal(1, 2)",
          class = "b", coef = "mass"
        )),
        iter = 50000, warmup = 5000,
        cores = 4, chains = 4,
        silent = TRUE, refresh = 0,
        file = "./models/_resPlotModel8WeeksBill.Rds"
      )
    )[, "Estimate"]),
  aes(x = billLengthMean, y = slope)
) +
  geom_point(
    pch = 21, size = 1.5,
    colour = "black", fill = "grey50"
  ) +
  geom_smooth(
    method = "lm", se = FALSE,
    colour = "black", linetype = "dashed"
  ) +
  xlab("Residual Bill\nLength (mm)") +
  ylab("Metabolic Slope\n(Fold Metabolism at\nThermoneutrality/°C)") +
  theme_classic()

((p1 + p2) / (p3 + p4) / p5)
```

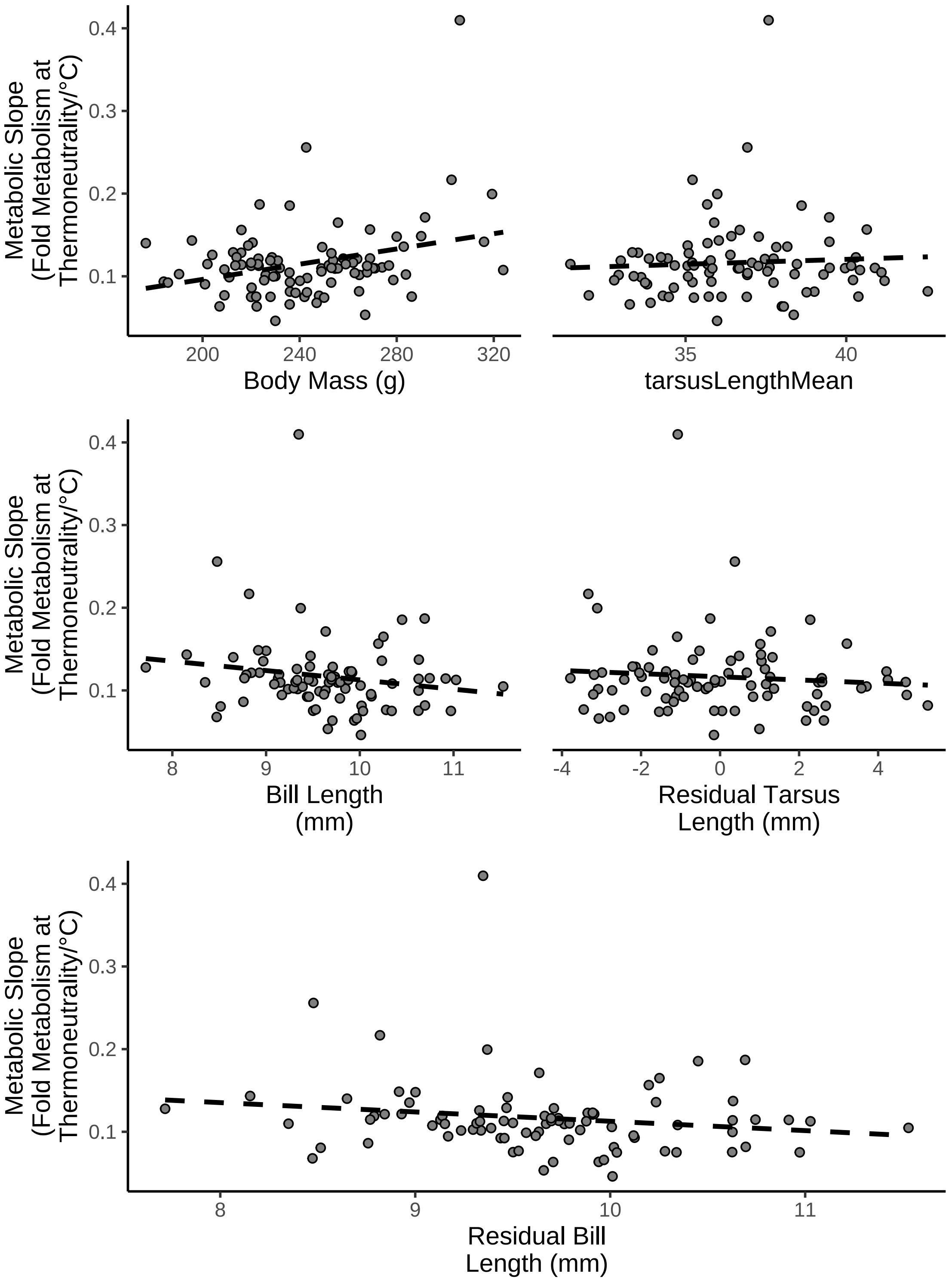

Figure 132: *Raw correlations between morphology and metabolic slopes above thermoneutrality in 8 week old Japanese quail. Metabolic slopes are measured as the fold change in resting metabolism between 30°C and 40°C, divided by 10°C. Relative tarsus and bill length indicate ordinary residuals from Bayesian regressions of either measure (in mm) against body mass (g). Dots indicate raw values per individual and dashed lines indicate estimated relationships as predicted by the R package ggplot2 (Wickham, 2011).*

And again, we check whether variance in metabolic slopes differs between experimental batches owing to differences in flow-rates.

```
slopeDataHot8 %>%
  rename("batch" = exp) %>%
  filter(slope < 0.4) %>%
  ggplot(aes(x = slope, fill = batch)) +
  geom_density(colour = "black", alpha = 0.5) +
  scale_fill_manual(values = c("black", "firebrick", "beige"),
                    labels = c("1", "2", "3"),
                    name = "Experimental\nBatch") +
  theme_classic() +
  xlab("Metabolic Slope\n(Fold Metabolism at Thermoneutrality") +
  ylab("Density")
```

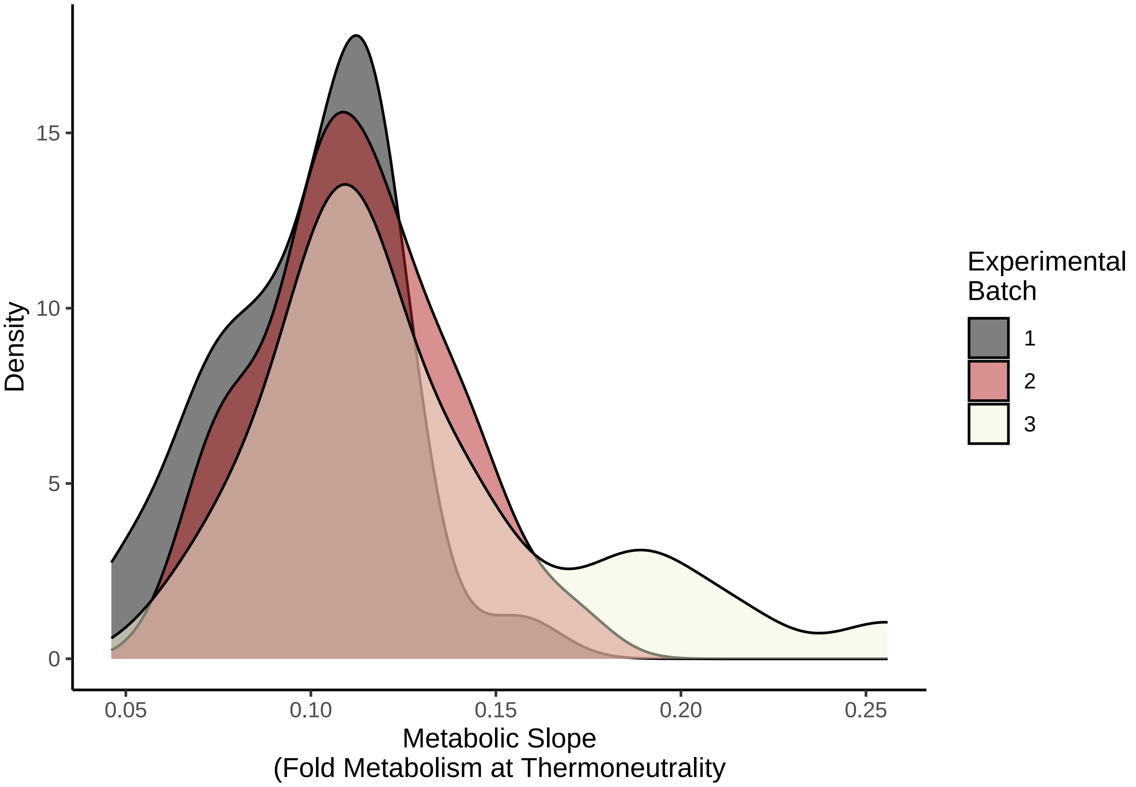

Figure 133: *Densities of metabolic slope values below 30°C from eight week old Japanese quail. Densities are divided by experimental batches.*

Here, variance appears largely similar between batches. As such, no adjustments to the error term of our model predicting metabolic slope at this age are made.

For this path analysis, we used models and priors largely equivalent to those used to analyse data from three week old quail. Minor adjustments are made to priors to accommodate: (1) an increased variance in body size and appendage length, and (2) a decrease in metabolic slope at this age (see Persson et al, 2024). These adjusted priors were therefore as follows:

$$\beta_{a0} \sim \mathcal{N}(0, 10)$$

$$\beta_{a1} \sim \mathcal{N}(0, 25)$$

$$\beta_{a2} \sim \mathcal{N}(0, 25)$$

$$\mu_{0a} \sim exp(1.5)$$
$$\epsilon_a \sim exp(0.05)$$

$$\beta_{b0} \sim \mathcal{N}(0, 3)$$
$$\beta_{b1} \sim \mathcal{N}(0, 3)$$
$$\beta_{b2} \sim \mathcal{N}(0, 3)$$
$$\beta_{b3} \sim S\mathcal{N}(0, 0.25, 5)$$
$$\mu_{0b} \sim exp(2)$$
$$\epsilon_b \sim exp(0.75)$$

$$\beta_{c0} \sim \mathcal{N}(0, 1)$$
$$\beta_{c1} \sim \mathcal{N}(0, 1)$$
$$\beta_{c2} \sim \mathcal{N}(0, 1)$$
$$\beta_{c3} \sim S\mathcal{N}(0, 0.25, 5)$$
$$\mu_{0c} \sim exp(5)$$
$$\epsilon_c \sim exp(2.5)$$

and:

$$\beta_{d0} \sim \mathcal{N}(0, 0.1)$$
$$\beta_{d1} \sim \mathcal{N}(0, 0.1)$$
$$\beta_{d2} \sim \mathcal{N}(0, 0.1)$$
$$\beta_{d3} \sim \mathcal{N}(0, 0.0025)$$
$$\beta_{d4} \sim \mathcal{N}(0, 0.03)$$
$$\beta_{d5} \sim \mathcal{N}(0, 0.1)$$
$$\mu_{0d} \sim exp(50)$$
$$\epsilon_d \sim exp(10)$$

Again, we assumed a Student's-T distributed error with a gamma-distributed prior on ν ($\alpha = 10$, $\beta = 1$).

```
# Running prior predictive check

functionModel8WeeksHotppCheck <-
  brm(
    data = slopeDataHot8 %>%
      mutate(
        mass = mass - mean(mass, na.rm = T),
        tarsus = tarsusLengthMean -
          mean(tarsusLengthMean, na.rm = T),
        bill = billLengthMean -
          mean(billLengthMean, na.rm = T),
        pretreatment = ifelse(pretreatment == "cold", "A",
          ifelse(pretreatment == "neutral", "B", "C")
        ),
```

```
      slope = slope -
        mean(slope, na.rm = T)
    ) %>%
    mutate(pretreatment = factor(pretreatment,
      levels = c("B", "A", "C")
    )) %>%
    drop_na() %>%
    merge(., data %>%
      dplyr::select(ring, "batch" = exp) %>%
      distinct(),
    by = "ring", all.x = TRUE
    ),
    bf(mass ~ pretreatment + (1 | batch),
       family = "gaussian") +
    bf(tarsus ~ mass + pretreatment + (1 | batch),
       family = "gaussian") +
    bf(bill ~ mass + pretreatment + (1 | batch),
       family = "gaussian") +
    bf(slope ~ mass + tarsus + bill + pretreatment + (1 | batch),
       family = student) +
    set_rescor(FALSE),
  prior = c(
    set_prior("normal(0, 10)",
      class = "Intercept",
      resp = "mass"
    ),
    set_prior("normal(0, 25)",
      class = "b",
      coef = "pretreatmentA",
      resp = "mass"
    ),
    set_prior("normal(0, 25)",
      class = "b",
      coef = "pretreatmentC",
      resp = "mass"
    ),
    set_prior("exponential(1.5)",
      class = "sd",
      group = "batch",
      resp = "mass"
    ),
    set_prior("exponential(0.05)",
      class = "sigma",
      resp = "mass"
    ),
    set_prior("normal(0, 3)",
      class = "Intercept",
      resp = "tarsus"
    ),
    set_prior("normal(0, 3)",
      class = "b",
      coef = "pretreatmentA",
      resp = "tarsus"
    ),
    set_prior("normal(0, 3)",
      class = "b",
      coef = "pretreatmentC",
      resp = "tarsus"
    ),
    set_prior("skew_normal(0, 0.25, 5)",
      class = "b",
      coef = "mass",
      resp = "tarsus"
    ),
    set_prior("exponential(2)",
      class = "sd",
      group = "batch",
```

```
      resp = "tarsus"
    ),
    set_prior("exponential(0.75)",
      class = "sigma",
      resp = "tarsus"
    ),
    set_prior("normal(0, 1)",
      class = "Intercept",
      resp = "bill"
    ),
    set_prior("normal(0, 0.5)",
      class = "b",
      coef = "pretreatmentA",
      resp = "bill"
    ),
    set_prior("normal(0, 0.5)",
      class = "b",
      coef = "pretreatmentC",
      resp = "bill"
    ),
    set_prior("skew_normal(0, 0.25, 5)",
      class = "b",
      coef = "mass",
      resp = "bill"
    ),
    set_prior("exponential(5)",
      class = "sd",
      group = "batch",
      resp = "bill"
    ),
    set_prior("exponential(2.5)",
      class = "sigma",
      resp = "bill"
    ),
    set_prior("normal(0, 0.1)",
      class = "Intercept",
      resp = "slope"
    ),
    set_prior("normal(0, 0.1)",
      class = "b",
      coef = "pretreatmentA",
      resp = "slope"
    ),
    set_prior("normal(0, 0.1)",
      class = "b",
      coef = "pretreatmentC",
      resp = "slope"
    ),
    set_prior("normal(0, 0.0025)",
      class = "b",
      coef = "mass",
      resp = "slope"
    ),
    set_prior("normal(0, 0.03)",
      class = "b",
      coef = "tarsus",
      resp = "slope"
    ),
    set_prior("normal(0, 0.1)",
      class = "b",
      coef = "bill",
      resp = "slope"
    ),
    set_prior("exponential(50)",
      class = "sd",
      group = "batch",
      resp = "slope"
```

```
      ),
      set_prior("exponential(10)",
        class = "sigma",
        resp = "slope"
      ),
      set_prior("gamma(10, 1)",
                class = "nu",
                resp = "slope"
      )
    ),
    iter = 50000, warmup = 10000, cores = 4, chains = 4, thin = 20,
    control = list(adapt_delta = .98, max_treedepth = 14),
    silent = TRUE, refresh = 0,
    sample_prior = "only",
    file = "./models/_eightWeekFunctionModelHotppCheck.Rds"
  )

pp_check2(functionModel8WeeksHotppCheck,
  resp = "slope",
  xlab = paste0(
    "Metabolic Slope\n",
    "(Fold Metabolism at Thermoneutrality/°C)"
  )
)
```

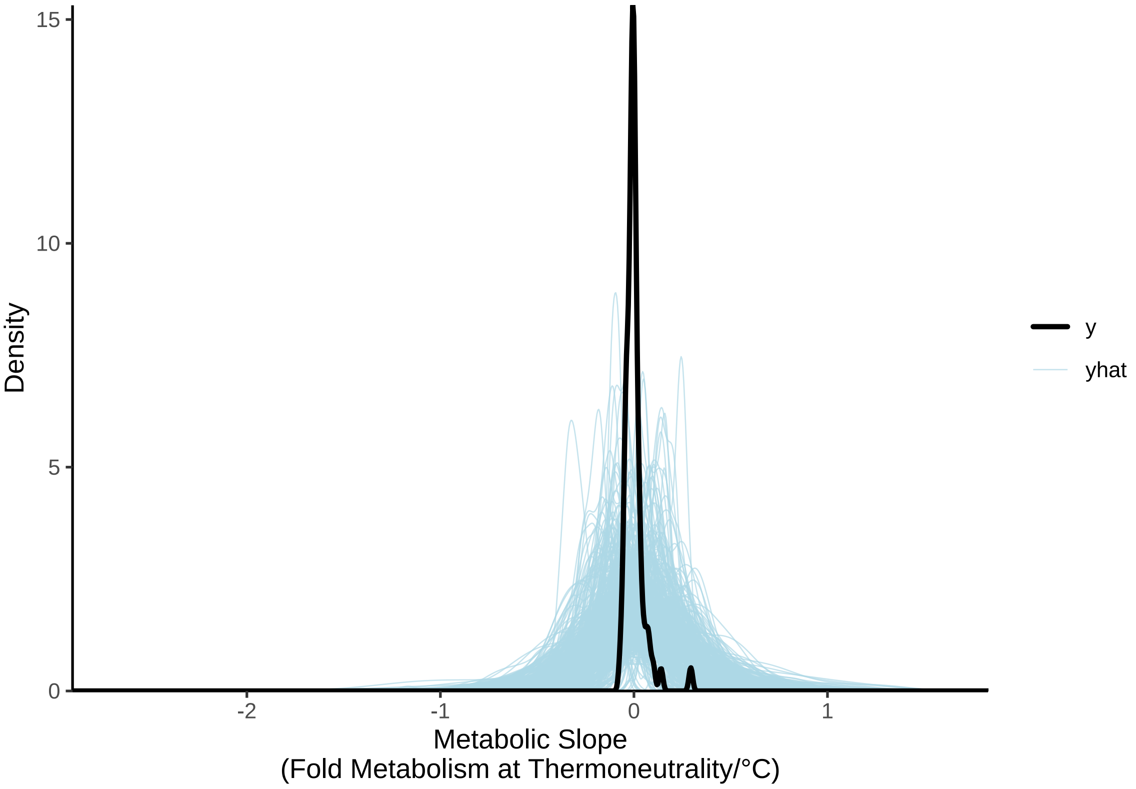

Figure 134: *Prior predictive check for a Bayesian path analysis predicting metabolic slope in the heat (fold metabolism at thermoneutrality/°C) as a direct and indirect function of body mass (g), tarsus length (mm) and bill length in eight week old Japanese quail. Light blue lines represent densities of resting metabolism values as predicted by model priors alone. The dark blue line represents the true density of resting metabolism values. Clear overlap between the dark blue and light blue lines indicates that priors are suitable.*

```
# Priors clearly not constraining. Constructing full model.
```

```
functionModel8WeeksHot <-
  brm(
   data = slopeDataHot8 %>%
     mutate(
       mass = mass - mean(mass, na.rm = T),
       tarsus = tarsusLengthMean -
         mean(tarsusLengthMean, na.rm = T),
       bill = billLengthMean -
         mean(billLengthMean, na.rm = T),
       pretreatment = ifelse(pretreatment == "cold", "A",
         ifelse(pretreatment == "neutral", "B", "C")
       ),
       slope = slope -
         mean(slope, na.rm = T)
     ) %>%
     mutate(pretreatment = factor(pretreatment,
       levels = c("B", "A", "C")
     )) %>%
     drop_na() %>%
     merge(., data %>%
       dplyr::select(ring, "batch" = exp) %>%
       distinct(),
     by = "ring", all.x = TRUE
     ),
     bf(mass ~ pretreatment + (1 | batch),
        family = "gaussian") +
     bf(tarsus ~ mass + pretreatment + (1 | batch),
        family = "gaussian") +
     bf(bill ~ mass + pretreatment + (1 | batch),
        family = "gaussian") +
     bf(slope ~ mass + tarsus + bill + pretreatment + (1 | batch),
        family = student) +
     set_rescor(FALSE),
   prior = c(
     set_prior("normal(0, 10)",
       class = "Intercept",
       resp = "mass"
     ),
     set_prior("normal(0, 25)",
       class = "b",
       coef = "pretreatmentA",
       resp = "mass"
     ),
     set_prior("normal(0, 25)",
       class = "b",
       coef = "pretreatmentC",
       resp = "mass"
     ),
     set_prior("exponential(1.5)",
       class = "sd",
       group = "batch",
       resp = "mass"
     ),
     set_prior("exponential(0.05)",
       class = "sigma",
       resp = "mass"
     ),
     set_prior("normal(0, 3)",
       class = "Intercept",
       resp = "tarsus"
     ),
     set_prior("normal(0, 3)",
       class = "b",
       coef = "pretreatmentA",
       resp = "tarsus"
     ),
     set_prior("normal(0, 3)",
```

```
      class = "b",
      coef = "pretreatmentC",
      resp = "tarsus"
    ),
    set_prior("skew_normal(0, 0.25, 5)",
      class = "b",
      coef = "mass",
      resp = "tarsus"
    ),
    set_prior("exponential(2)",
      class = "sd",
      group = "batch",
      resp = "tarsus"
    ),
    set_prior("exponential(0.75)",
      class = "sigma",
      resp = "tarsus"
    ),
    set_prior("normal(0, 1)",
      class = "Intercept",
      resp = "bill"
    ),
    set_prior("normal(0, 0.5)",
      class = "b",
      coef = "pretreatmentA",
      resp = "bill"
    ),
    set_prior("normal(0, 0.5)",
      class = "b",
      coef = "pretreatmentC",
      resp = "bill"
    ),
    set_prior("skew_normal(0, 0.25, 5)",
      class = "b",
      coef = "mass",
      resp = "bill"
    ),
    set_prior("exponential(5)",
      class = "sd",
      group = "batch",
      resp = "bill"
    ),
    set_prior("exponential(2.5)",
      class = "sigma",
      resp = "bill"
    ),
    set_prior("normal(0, 0.1)",
      class = "Intercept",
      resp = "slope"
    ),
    set_prior("normal(0, 0.1)",
      class = "b",
      coef = "pretreatmentA",
      resp = "slope"
    ),
    set_prior("normal(0, 0.1)",
      class = "b",
      coef = "pretreatmentC",
      resp = "slope"
    ),
    set_prior("normal(0, 0.0025)",
      class = "b",
      coef = "mass",
      resp = "slope"
    ),
    set_prior("normal(0, 0.03)",
      class = "b",
```

```
        coef = "tarsus",
        resp = "slope"
      ),
      set_prior("normal(0, 0.1)",
        class = "b",
        coef = "bill",
        resp = "slope"
      ),
      set_prior("exponential(50)",
        class = "sd",
        group = "batch",
        resp = "slope"
      ),
      set_prior("exponential(10)",
        class = "sigma",
        resp = "slope"
      ),
      set_prior("gamma(10, 1)",
                class = "nu",
                resp = "slope"
      )
    ),
    iter = 50000, warmup = 10000, cores = 4, chains = 4, thin = 20,
    control = list(adapt_delta = .98, max_treedepth = 14),
    silent = TRUE, refresh = 0,
    file = "./models/_eightWeekFunctionModelHot.Rds"
  )

chainCheck(functionModel8WeeksHot)
```

```
## Rhat range: 1 - 1.001
## Neff/N range: 0.815 - 1.007
```

```
# Very little attrition in effective sample sizes. Neff/N > 0.75.

caption <- paste0(
  "R\\textsuperscript{2} for a Bayesian ",
  "path analysis predicting metabolic slopes ",
  "(fold metabolism at thermoneutrality/°C) of ",
  "eight week old Japanese quail as a function of ",
  "body mass (g), tarsus length (mm) and bill length (mm). Metabolic slopes ",
  "are measured above thermoneutrality (30°C - 40°C)."
)

brms::bayes_R2(functionModel8WeeksHot,
  ndraws = 1000,
  robust = TRUE
) %>%
  as.data.frame() %>%
  rownames_to_column("var") %>%
  merge(., tribble(
    ~var, ~Var,
    "R2mass", "Body Mass (g)",
    "R2tarsus", "Tarsus Length (mm)",
    "R2bill", "Bill Length (mm)",
    "R2slope",
    "Metabolic Slope"
  ), by = c("var")) %>%
  mutate(
    Estimate = round(Estimate, digits = 4),
    Est.Error = round(Est.Error, digits = 4),
    `95\\% CI` = paste0(
      "[",
      round(Q2.5, digits = 4),
      ", ",
      round(Q97.5, digits = 4),
      "]"
    )
```

```
) %>%
dplyr::select(
  "Response" = Var, "R\\textsuperscript{2}" = Estimate,
  "Standard Error" = Est.Error, `95\\% CI`
) %>%
kbl(.,
  longtable = T, booktabs = T, format = "latex",
  caption = caption, escape = FALSE
) %>%
column_spec(column = c(1:10), width = "2.7cm") %>%
kable_styling(latex_options = "striped")
```

Table 78: *R^2 for a Bayesian path analysis predicting metabolic slopes (fold metabolism at thermoneutrality/°C) of eight week old Japanese quail as a function of body mass (g), tarsus length (mm) and bill length (mm). Metabolic slopes are measured above thermoneutrality (30°C - 40°C).*

Response	R^2	Standard Error	95% CI
Bill Length (mm)	0.1328	0.0682	[0.0313, 0.2711]
Body Mass (g)	0.0382	0.0344	[0.0023, 0.1281]
Metabolic Slope	0.1100	0.0480	[0.0349, 0.221]
Tarsus Length (mm)	0.2979	0.0710	[0.1391, 0.428]

```
ggarrange(
  pp_check2(functionModel8WeeksHot,
    resp = "slope",
    xlab = "Metabolic Slope\n(Fold Metabolism at\nThermoneutrality/°C)"
  ),
  functionModel8WeeksHot$data %>%
    mutate(
      "Fit" = fitted(functionModel8WeeksHot,
        resp = "slope",
        robust = TRUE
      )[, "Estimate"],
      "SE" = fitted(functionModel8WeeksHot,
        resp = "slope",
        robust = TRUE
      )[, "Est.Error"]
    ) %>%
    ggplot(aes(x = Fit, y = slope)) +
    geom_errorbarh(
      aes(
        y = slope, xmin = Fit - SE,
        xmax = Fit + SE
      ),
      colour = "black", height = 0.01
    ) +
    geom_point() +
    geom_line(
      data = data.frame(
        "Fit" = c(-0.1, 0.1),
        "slope" = c(-0.1, 0.1)
      ),
      colour = "grey50"
    ) +
    xlab("Fitted Metabolic Slope\n(Fold Metabolism at\nThermoneutrality/°C)") +
    ylab("Metabolic Slope\n(Fold Metabolism at\nThermoneutrality/°C)") +
    theme_classic(),
  labels = c("A", "B")
)
```

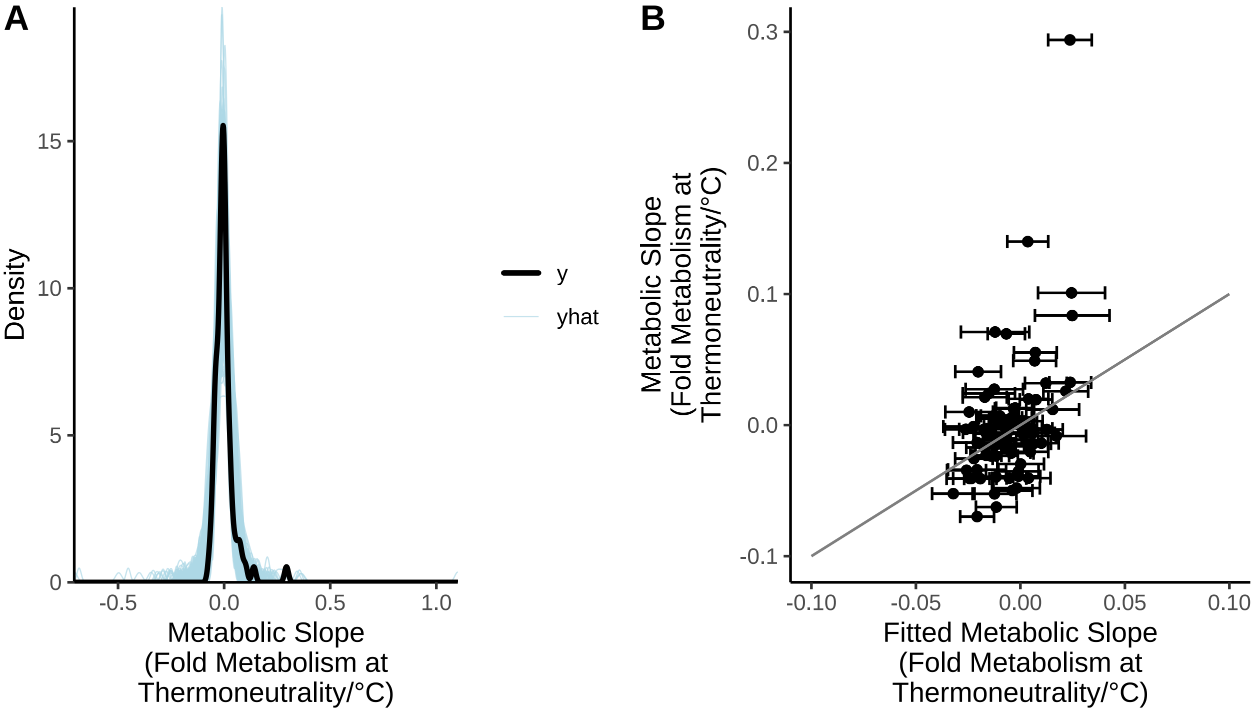

__Figure 135:__ Measures of fit for a Bayesian path analysis predicting physiological responses to heat in eight week old Japanese quail. Panel A displays a posterior predictive check from the path analysis with the black line indicating the true deensity of metabolic responses to heat among birds, and blue lines indicating densities estimated from posterior draws. Panel B displays fitted values by true values with dots indicating individual data points, errorbars indicating standard errors around fitted values, and the grey line indicating a theoretical 1:1 relationship.

Model residuals are next visualised.

```
p1 <- functionModel8WeeksHot$data %>%
  mutate("Residuals" = residuals(functionModel8WeeksHot,
    resp = "slope",
    robust = TRUE
  )[, "Estimate"]) %>%
  ggplot(aes(x = Residuals)) +
  geom_density(colour = "black", fill = "grey50", alpha = 0.5) +
  xlab("Metabolic Slope\nResiduals") +
  ylab("Density") +
  theme_classic()

p2 <- functionModel8WeeksHot$data %>%
  mutate("Residuals" = residuals(functionModel8WeeksHot,
    resp = "slope",
    robust = TRUE
  )[, "Estimate"]) %>%
  ggplot(aes(x = mass, y = Residuals)) +
  geom_point(
    pch = 21, colour = "black", size = 2,
    fill = "lightblue", alpha = 0.5
  ) +
  xlab("Body Mass (g; Mean-Centred)") +
  ylab("Metabolic Slope\nResiduals") +
  theme_classic()

p3 <- functionModel8WeeksHot$data %>%
  mutate("Residuals" = residuals(functionModel8WeeksHot,
```

```
    resp = "slope",
    robust = TRUE
  )[, "Estimate"]) %>%
  ggplot(aes(x = tarsus, y = Residuals)) +
  geom_point(
    pch = 21, colour = "black", size = 2,
    fill = "lightblue", alpha = 0.5
  ) +
  xlab("Tarsus Length (mm; Mean-Centred)") +
  ylab("Metabolic Slope\nResiduals") +
  theme_classic()

p4 <- functionModel8WeeksHot$data %>%
  mutate("Residuals" = residuals(functionModel8WeeksHot,
    resp = "slope",
    robust = TRUE
  )[, "Estimate"]) %>%
  ggplot(aes(x = bill, y = Residuals)) +
  geom_point(
    pch = 21, colour = "black", size = 2,
    fill = "lightblue", alpha = 0.5
  ) +
  xlab("Bill Length (mm; Mean-Centred)") +
  ylab("Metabolic Slope\nResiduals") +
  theme_classic()

p5 <- functionModel8WeeksHot$data %>%
  mutate(
    "Residuals" =
      residuals(functionModel8WeeksHot,
        resp = "slope",
        robust = TRUE
      )[, "Estimate"]
  ) %>%
  mutate("pretreatment" = ifelse(pretreatment == "A", "Cold (10°C)",
    ifelse(pretreatment == "B", "Mild (20°C)",
      "Warm (30°C)"
    )
  )) %>%
  ggplot(aes(x = pretreatment, y = Residuals)) +
  geom_point(
    pch = 21, colour = "black", size = 2,
    fill = "lightblue", alpha = 0.5
  ) +
  stat_summary(
    geom = "errorbar", fun.data = "mean_se",
    colour = "black", width = 0.25
  ) +
  stat_summary(
    geom = "point", fun = "mean", size = 4,
    pch = 21, colour = "black", fill = "lightblue"
  ) +
  xlab("Rearing Conditions") +
  ylab("Metabolic Slope\nResiduals") +
  theme_classic()

p6 <- functionModel8WeeksHot$data %>%
  mutate(
    "Residuals" = residuals(functionModel8WeeksHot,
      resp = "slope",
      robust = TRUE
    )[, "Estimate"],
    "Fit" = fitted(functionModel8WeeksHot,
      resp = "slope",
      robust = TRUE
    )[, "Estimate"]
  ) %>%
```

```
  ggplot(aes(x = Fit, y = Residuals)) +
  geom_point(
    pch = 21, colour = "black", size = 2,
    fill = "lightblue", alpha = 0.5
  ) +
  xlab("Fitted Metabolic Slope\n(Fold Metabolism at\nThermoneutrality/°C)") +
  ylab("Metabolic Slope\nResiduals") +
  theme_classic()

(p1 + p2) / (p3 + p4) / (p5 + p6) +
  plot_annotation(tag_levels = "A")
```

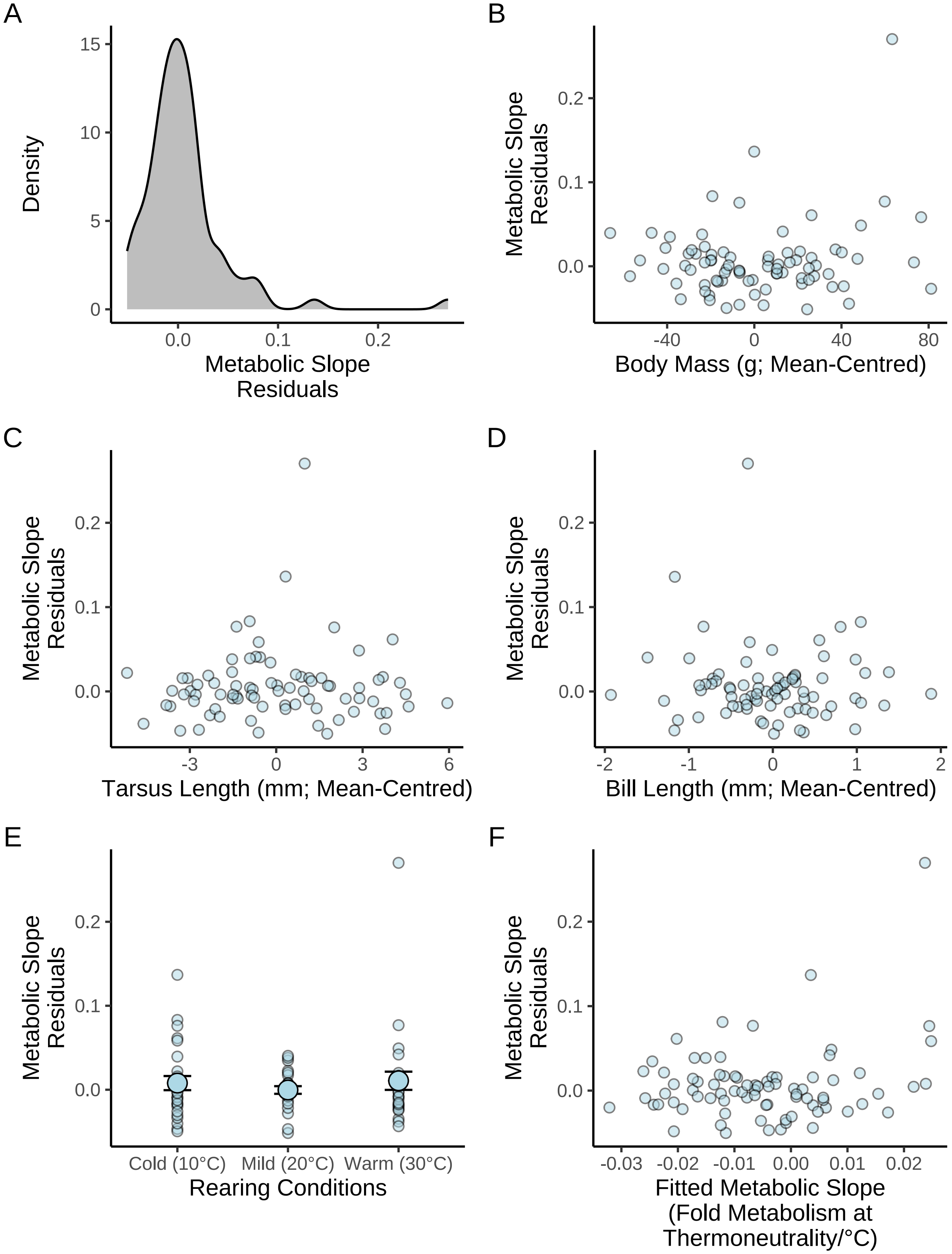

Figure 136: *Spread of ordinary residuals from Bayesian path analysis predicting physiological responses to heat in eight week old Japanese quail. Quail were reared in one of three thermal conditions: (1) cold (10°C), (2) mild (20°C), or warm (30°C). Panel A displays density of ordinary residuals of metabolic slopes (fold metabolism at thermoneutrality/°C). Panels B to F display ordinary residuals against predictors and fitted values; dots in these panels represent values per individual.*

Again, skewing in our median residuals is observed, as expected from our data-generating process (discussed above) and from our observation of a tentative outlying individual. We visualise the importance of individual data points to our analysis outcome as described for metabolic responses to heat in three-week old individuals.

```
loo(functionModel8WeeksHot)$diagnostics$pareto_k %>%
  as_tibble() %>%
  ggplot(aes(x = 1:nrow(.), y = value)) +
  geom_point(pch = 21, colour = "black", fill = "grey50", size = 2) +
  geom_hline(yintercept = 0.5, colour = "black", linetype = "dashed") +
  geom_hline(yintercept = 0.7, colour = "red4", linetype = "dashed") +
  xlab("Sample") +
  ylab("Pareto K") +
  theme_classic()
```

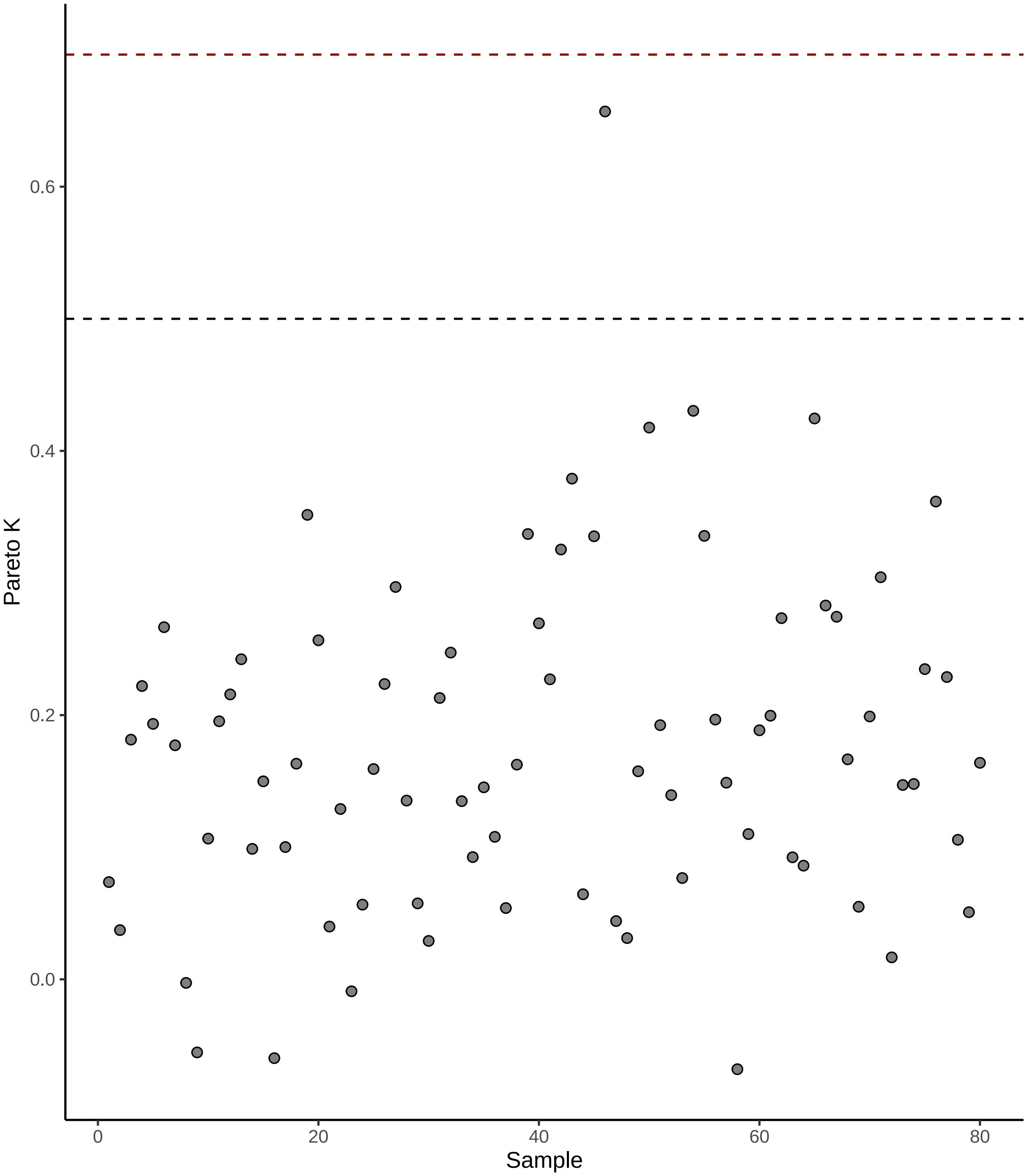

Figure 137: *Relative importance of individual metabolic slope values (dots; derived from mature quail) to outcomes of our Bayesian path analysis, as estimated using leave-one-out cross validations. Importance is measured here as Pareto K (Vehtari et al, 2017). The black, dashed horizontal line indicated a Pareto K of 0.5 and the red horizontal dashed line indicates as Pareto K of 0.7, above which, samples hold significant importance on analysis outcomes.*

One sample has a moderately strong (Pareto K > 0.5) influence on our model outcome. However, we have no *a priori* reason to exclude this sample and we therefore proceed by visualising the posterior densities for

our model coefficients and summarising their spread.

```
as.data.frame(functionModel8WeeksHot) %>%
  dplyr::select(
    "Intercept" = b_slope_Intercept,
    "Body Mass\n(g; Mean-Centred)" = b_slope_mass,
    "Tarsus Length\n(mm; Mean-Centred)" = b_slope_tarsus,
    "Bill Length\n(mm; Mean-Centred)" = b_slope_bill,
    "Cold Rearing\n(10°C)" = b_slope_pretreatmentA,
    "Warm Rearing\n(30°C)" = b_slope_pretreatmentC,
    # "Sigma" = sigma_slope
  ) %>%
  pivot_longer(everything(),
    names_to = "Par",
    values_to = "Coefs"
  ) %>%
  ggplot(aes(x = Coefs)) +
  facet_wrap(~Par, scales = "free", ncol = 2) +
  geom_density(
    colour = "black",
    alpha = 0.5, fill = "grey70"
  ) +
  geom_vline(
    xintercept = 0, linetype = "dashed",
    colour = "black"
  ) +
  ylab("Density") +
  scale_x_continuous(n.breaks = 4) +
  theme_classic() +
  theme(axis.title.x = element_blank()) +
  ggtitle(paste0(
    "Metabolic Slope\n(Fold Metabolism ",
    "at Thermoneutrality/°C)"
  ))
```

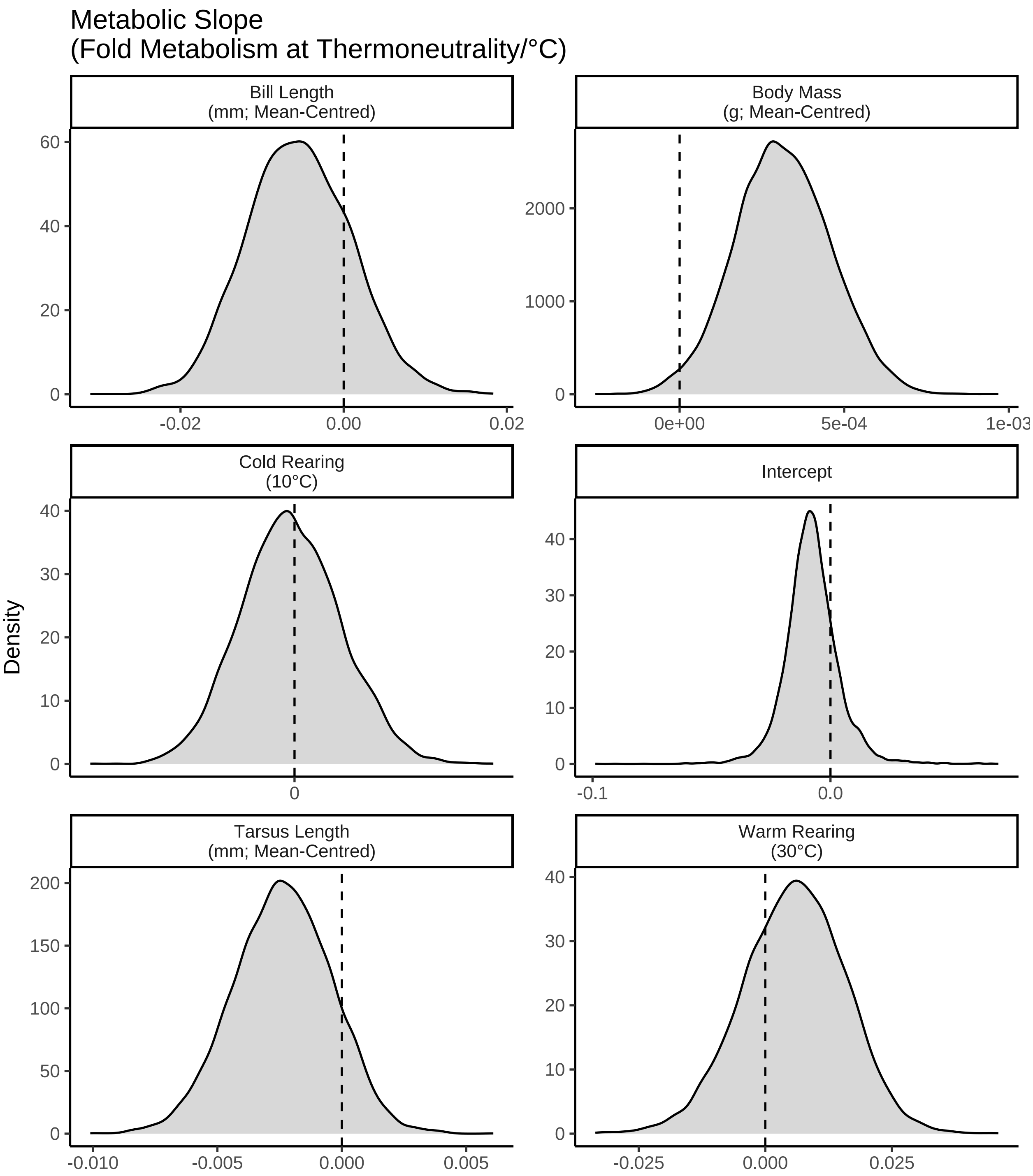

Figure 138: *Posterior densities of coefficients for a Bayesian path analysis predicting physiological responses to heat in eight week old Japanese quail. Quail were reared in one of three thermal conditions: (1) cold (10°C), (2) mild (20°C), or warm (30°C).*

```
caption <- paste0(
  "Results from a Bayesian path analysis ",
  "predicting metabolic responses to heat (metabolic slope ",
  "[fold metabolism at thermoneutrality/°C]) as ",
  "a function of morphometry in eight week old Japanese quail. ",
```

```
  "Physiological measurements are made at ambient temperatures ",
  "between 30°C and 40°C. Cold rearing indicates ",
  "post-hatch rearing at 10°C, relative to 20°C (intercept), ",
  "or 30°C ('warm rearing'). CIs indicates quantile intervals ",
  "and BF indicates Bayes Factors."
)

week8ResultsHeat <-
  as.data.frame(functionModel8WeeksHot) %>%
  summarise_all(., .funs = median) %>%
  pivot_longer(everything(),
    names_to = "Parameter",
    values_to = "Estimate"
  ) %>%
  merge(., quantileCIs(functionModel8WeeksHot, cis = c(50, 95)),
    by = "Parameter", all.x = TRUE
  ) %>%
  filter(grepl("b_|sd_", Parameter)) %>%
  rowwise() %>%
  mutate("BF" = ifelse(Estimate < 0,
    (2 * mean(as.data.frame(
      functionModel8WeeksHot
    )[, Parameter] <= 0)) /
      (2 * mean(as.data.frame(
        functionModel8WeeksHot
      )[, Parameter] >= 0)),
    (2 * mean(as.data.frame(
      functionModel8WeeksHot
    )[, Parameter] >= 0)) /
      (2 * mean(as.data.frame(
        functionModel8WeeksHot
      )[, Parameter] <= 0))
  )) %>%
  ungroup() %>%
  mutate(
    "Estimate" = round(Estimate, digits = 4),
    "BF" = round(BF, digits = 4),
    "N" = nrow(functionModel8WeeksHot$data)
  ) %>%
  mutate("Parameter" = ifelse(grepl("b_", Parameter),
    gsub("b_", "", Parameter),
    gsub(
      "Intercept", "batch",
      gsub(".*__", "", Parameter)
    )
  )) %>%
  mutate(
    "Response" = gsub("_.*", "", Parameter),
    "Parameter" = gsub(".*_", "", Parameter)
  ) %>%
  merge(., tribble(
    ~Response, ~response, ~level,
    "mass", "Body Mass (g)", "A",
    "tarsus", "Tarsus Length (mm)", "B",
    "bill", "Bill Length (mm)", "C",
    "slope",
    "Metabolic Slope", "D"
  ),
  by = "Response"
  ) %>%
  merge(., tribble(
    ~Parameter, ~parameter, ~number,
    "Intercept", "Intercept", "1",
    "mass", "Body Mass (g)", "4",
    "tarsus", "Tarsus Length (mm)", "5",
    "bill", "Bill Length (mm)", "6",
    "pretreatmentA", "Cold Rearing", "2",
```

```
    "pretreatmentC", "Warm Rearing", "3",
    "batch", "Egg Batch [mu]", "7"
  ),
  by = "Parameter"
  ) %>%
  mutate(
    `50\\% HDI` = paste0("(", paste(
      round(Low_CI_50, digits = 4),
      round(High_CI_50, digits = 4),
      sep = ", "
    ), ")"),
    `95\\% HDI` = paste0("(", paste(
      round(Low_CI_95, digits = 4),
      round(High_CI_95, digits = 4),
      sep = ", "
    ), ")")
  ) %>%
  dplyr::select(-c(Low_CI_50, High_CI_50, Low_CI_95, High_CI_95)) %>%
  dplyr::select(
    "Response" = "response", "Parameter" = "parameter", N,
    Estimate, `50\\% HDI`, `95\\% HDI`, BF, level, number
  ) %>%
  arrange(level, number) %>%
  dplyr::select(-c(level, number)) %>%
  kbl(.,
    longtable = T, booktabs = T, format = "latex", escape = FALSE,
    caption = caption
  ) %>%
  column_spec(column = c(1:2), width = "2.2cm") %>%
  column_spec(column = c(3:10), width = "1.9cm") %>%
  kable_styling(latex_options = "striped")

week8ResultsHeat
```

Table 79: *Results from a Bayesian path analysis predicting metabolic responses to heat (metabolic slope [fold metabolism at thermoneutrality/°C]) as a function of morphometry in eight week old Japanese quail. Physiological measurements are made at ambient temperatures between 30°C and 40°C. Cold rearing indicates post-hatch rearing at 10°C, relative to 20°C (intercept), or 30°C ('warm rearing'). CIs indicates quantile intervals and BF indicates Bayes Factors.*

Response	Parameter	N	Estimate	50% HDI	95% HDI	BF
Body Mass (g)	Intercept	80	-4.4920	(-8.3769, -0.6335)	(-15.7041, 7.1044)	3.6323
Body Mass (g)	Cold Rearing	80	4.5032	(-0.7963, 9.9309)	(-11.0828, 20.3915)	2.5149
Body Mass (g)	Warm Rearing	80	12.2417	(6.8788, 17.6248)	(-3.6347, 28.21)	13.6252
Body Mass (g)	Egg Batch [mu]	80	0.4868	(0.1974, 0.9724)	(0.0182, 2.4942)	Inf
Tarsus Length (mm)	Intercept	80	-0.5995	(-0.9822, -0.1952)	(-1.764, 0.7836)	5.1350
Tarsus Length (mm)	Cold Rearing	80	0.5786	(0.1044, 1.0365)	(-0.7524, 1.9299)	4.0031
Tarsus Length (mm)	Warm Rearing	80	0.8721	(0.4292, 1.3086)	(-0.4309, 2.199)	9.5402
Tarsus Length (mm)	Body Mass (g)	80	0.0294	(0.024, 0.0347)	(0.014, 0.0458)	2665.6667
Tarsus Length (mm)	Egg Batch [mu]	80	0.5898	(0.3519, 0.8897)	(0.0366, 1.7067)	Inf
Bill Length (mm)	Intercept	80	0.0018	(-0.1333, 0.1292)	(-0.457, 0.4271)	1.0095
Bill Length (mm)	Cold Rearing	80	-0.2694	(-0.3926, -0.1437)	(-0.6349, 0.0988)	12.4454
Bill Length (mm)	Warm Rearing	80	0.2010	(0.0684, 0.3329)	(-0.1778, 0.5793)	5.5574

Bill Length (mm)	Body Mass (g)	80	-0.0004	(-0.002, 0.0013)	(-0.0051, 0.0044)	1.2773
Bill Length (mm)	Egg Batch [mu]	80	0.2455	(0.1655, 0.352)	(0.0281, 0.6684)	Inf
Metabolic Slope	Intercept	80	-0.0083	(-0.0142, -0.0019)	(-0.0294, 0.0155)	4.1118
Metabolic Slope	Cold Rearing	80	-0.0010	(-0.0076, 0.006)	(-0.0201, 0.0194)	1.1876
Metabolic Slope	Warm Rearing	80	0.0058	(-0.0011, 0.0125)	(-0.0145, 0.0247)	2.5119
Metabolic Slope	Body Mass (g)	80	0.0003	(2e-04, 4e-04)	(0, 6e-04)	66.2269
Metabolic Slope	Tarsus Length (mm)	80	-0.0024	(-0.0037, -0.001)	(-0.0063, 0.0014)	7.7623
Metabolic Slope	Bill Length (mm)	80	-0.0056	(-0.0099, -0.0011)	(-0.0175, 0.0072)	3.9875
Metabolic Slope	Egg Batch [mu]	80	0.0101	(0.0049, 0.0175)	(4e-04, 0.0412)	Inf

Next, we calculate partial R^2 values for our morphometric measures (as predictors of metabolic responses to a heat exposure).

```
r2modData <- slopeDataHot8 %>%
  mutate(
    mass = mass - mean(mass, na.rm = T),
    tarsus = tarsusLengthMean -
      mean(tarsusLengthMean, na.rm = T),
    bill = billLengthMean -
      mean(billLengthMean, na.rm = T),
    pretreatment = ifelse(pretreatment == "cold", "A",
      ifelse(pretreatment == "neutral", "B", "C")
    ),
    slope = slope -
      mean(slope, na.rm = T)
  ) %>%
  mutate(pretreatment = factor(pretreatment,
    levels = c("B", "A", "C")
  )) %>%
  drop_na() %>%
  merge(., data %>%
    dplyr::select(ring, "batch" = exp) %>%
    distinct(),
  by = "ring", all.x = TRUE
  )

{
  functionModel8WeeksHotR2Mass <-
    brm(
      data = r2modData,
      bf(mass ~ pretreatment + (1 | batch),
        family = "gaussian"
      ) +
        bf(tarsus ~ mass + pretreatment + (1 | batch),
          family = "gaussian"
        ) +
        bf(bill ~ mass + pretreatment + (1 | batch),
          family = "gaussian"
        ) +
        bf(slope ~ tarsus + bill + pretreatment + (1 | batch),
          family = student
        ) +
        set_rescor(FALSE),
      prior = c(
        set_prior("normal(0, 10)",
          class = "Intercept",
```

```
      resp = "mass"
    ),
    set_prior("normal(0, 25)",
      class = "b",
      coef = "pretreatmentA",
      resp = "mass"
    ),
    set_prior("normal(0, 25)",
      class = "b",
      coef = "pretreatmentC",
      resp = "mass"
    ),
    set_prior("exponential(1.5)",
      class = "sd",
      group = "batch",
      resp = "mass"
    ),
    set_prior("exponential(0.05)",
      class = "sigma",
      resp = "mass"
    ),
    set_prior("normal(0, 3)",
      class = "Intercept",
      resp = "tarsus"
    ),
    set_prior("normal(0, 3)",
      class = "b",
      coef = "pretreatmentA",
      resp = "tarsus"
    ),
    set_prior("normal(0, 3)",
      class = "b",
      coef = "pretreatmentC",
      resp = "tarsus"
    ),
    set_prior("skew_normal(0, 0.25, 5)",
      class = "b",
      coef = "mass",
      resp = "tarsus"
    ),
    set_prior("exponential(2)",
      class = "sd",
      group = "batch",
      resp = "tarsus"
    ),
    set_prior("exponential(0.75)",
      class = "sigma",
      resp = "tarsus"
    ),
    set_prior("normal(0, 1)",
      class = "Intercept",
      resp = "bill"
    ),
    set_prior("normal(0, 0.5)",
      class = "b",
      coef = "pretreatmentA",
      resp = "bill"
    ),
    set_prior("normal(0, 0.5)",
      class = "b",
      coef = "pretreatmentC",
      resp = "bill"
    ),
    set_prior("skew_normal(0, 0.25, 5)",
      class = "b",
      coef = "mass",
      resp = "bill"
```

```
      ),
      set_prior("exponential(5)",
        class = "sd",
        group = "batch",
        resp = "bill"
      ),
      set_prior("exponential(2.5)",
        class = "sigma",
        resp = "bill"
      ),
      set_prior("normal(0, 0.1)",
        class = "Intercept",
        resp = "slope"
      ),
      set_prior("normal(0, 0.1)",
        class = "b",
        coef = "pretreatmentA",
        resp = "slope"
      ),
      set_prior("normal(0, 0.1)",
        class = "b",
        coef = "pretreatmentC",
        resp = "slope"
      ),
      set_prior("normal(0, 0.03)",
        class = "b",
        coef = "tarsus",
        resp = "slope"
      ),
      set_prior("normal(0, 0.1)",
        class = "b",
        coef = "bill",
        resp = "slope"
      ),
      set_prior("exponential(50)",
        class = "sd",
        group = "batch",
        resp = "slope"
      ),
      set_prior("exponential(10)",
        class = "sigma",
        resp = "slope"
      ),
      set_prior("gamma(10, 1)",
        class = "nu",
        resp = "slope"
      )
    ),
    iter = 50000, warmup = 10000, cores = 4, chains = 4, thin = 20,
    control = list(adapt_delta = .98, max_treedepth = 14),
    silent = TRUE, refresh = 0,
    file = "./models/_eightWeekFunctionModelHotR2Mass.Rds"
  )

functionModel8WeeksHotR2Tarsus <-
  brm(data = r2modData,
    bf(mass ~ pretreatment + (1 | batch),
      family = "gaussian"
    ) +
      bf(tarsus ~ mass + pretreatment + (1 | batch),
        family = "gaussian"
      ) +
      bf(bill ~ mass + pretreatment + (1 | batch),
        family = "gaussian"
      ) +
      bf(slope ~ mass + bill + pretreatment + (1 | batch),
        family = student
```

```
    ) +
    set_rescor(FALSE),
  prior = c(
    set_prior("normal(0, 10)",
      class = "Intercept",
      resp = "mass"
    ),
    set_prior("normal(0, 25)",
      class = "b",
      coef = "pretreatmentA",
      resp = "mass"
    ),
    set_prior("normal(0, 25)",
      class = "b",
      coef = "pretreatmentC",
      resp = "mass"
    ),
    set_prior("exponential(1.5)",
      class = "sd",
      group = "batch",
      resp = "mass"
    ),
    set_prior("exponential(0.05)",
      class = "sigma",
      resp = "mass"
    ),
    set_prior("normal(0, 3)",
      class = "Intercept",
      resp = "tarsus"
    ),
    set_prior("normal(0, 3)",
      class = "b",
      coef = "pretreatmentA",
      resp = "tarsus"
    ),
    set_prior("normal(0, 3)",
      class = "b",
      coef = "pretreatmentC",
      resp = "tarsus"
    ),
    set_prior("skew_normal(0, 0.25, 5)",
      class = "b",
      coef = "mass",
      resp = "tarsus"
    ),
    set_prior("exponential(2)",
      class = "sd",
      group = "batch",
      resp = "tarsus"
    ),
    set_prior("exponential(0.75)",
      class = "sigma",
      resp = "tarsus"
    ),
    set_prior("normal(0, 1)",
      class = "Intercept",
      resp = "bill"
    ),
    set_prior("normal(0, 0.5)",
      class = "b",
      coef = "pretreatmentA",
      resp = "bill"
    ),
    set_prior("normal(0, 0.5)",
      class = "b",
      coef = "pretreatmentC",
      resp = "bill"
```

```
      ),
      set_prior("skew_normal(0, 0.25, 5)",
        class = "b",
        coef = "mass",
        resp = "bill"
      ),
      set_prior("exponential(5)",
        class = "sd",
        group = "batch",
        resp = "bill"
      ),
      set_prior("exponential(2.5)",
        class = "sigma",
        resp = "bill"
      ),
      set_prior("normal(0, 0.1)",
        class = "Intercept",
        resp = "slope"
      ),
      set_prior("normal(0, 0.1)",
        class = "b",
        coef = "pretreatmentA",
        resp = "slope"
      ),
      set_prior("normal(0, 0.1)",
        class = "b",
        coef = "pretreatmentC",
        resp = "slope"
      ),
      set_prior("normal(0, 0.0025)",
        class = "b",
        coef = "mass",
        resp = "slope"
      ),
      set_prior("normal(0, 0.1)",
        class = "b",
        coef = "bill",
        resp = "slope"
      ),
      set_prior("exponential(50)",
        class = "sd",
        group = "batch",
        resp = "slope"
      ),
      set_prior("exponential(10)",
        class = "sigma",
        resp = "slope"
      ),
      set_prior("gamma(10, 1)",
        class = "nu",
        resp = "slope"
      )
    ),
    iter = 50000, warmup = 10000, cores = 4, chains = 4, thin = 20,
    control = list(adapt_delta = .98, max_treedepth = 14),
    silent = TRUE, refresh = 0,
    file = "./models/_eightWeekFunctionModelHotR2Tarsus.Rds"
  )

functionModel8WeeksHotR2Bill <-
  brm(data = r2modData,
    bf(mass ~ pretreatment + (1 | batch),
      family = "gaussian"
    ) +
      bf(tarsus ~ mass + pretreatment + (1 | batch),
        family = "gaussian"
      ) +
```

```
    bf(bill ~ mass + pretreatment + (1 | batch),
      family = "gaussian"
    ) +
    bf(slope ~ mass + tarsus + pretreatment + (1 | batch),
      family = student
    ) +
    set_rescor(FALSE),
  prior = c(
    set_prior("normal(0, 10)",
      class = "Intercept",
      resp = "mass"
    ),
    set_prior("normal(0, 25)",
      class = "b",
      coef = "pretreatmentA",
      resp = "mass"
    ),
    set_prior("normal(0, 25)",
      class = "b",
      coef = "pretreatmentC",
      resp = "mass"
    ),
    set_prior("exponential(1.5)",
      class = "sd",
      group = "batch",
      resp = "mass"
    ),
    set_prior("exponential(0.05)",
      class = "sigma",
      resp = "mass"
    ),
    set_prior("normal(0, 3)",
      class = "Intercept",
      resp = "tarsus"
    ),
    set_prior("normal(0, 3)",
      class = "b",
      coef = "pretreatmentA",
      resp = "tarsus"
    ),
    set_prior("normal(0, 3)",
      class = "b",
      coef = "pretreatmentC",
      resp = "tarsus"
    ),
    set_prior("skew_normal(0, 0.25, 5)",
      class = "b",
      coef = "mass",
      resp = "tarsus"
    ),
    set_prior("exponential(2)",
      class = "sd",
      group = "batch",
      resp = "tarsus"
    ),
    set_prior("exponential(0.75)",
      class = "sigma",
      resp = "tarsus"
    ),
    set_prior("normal(0, 1)",
      class = "Intercept",
      resp = "bill"
    ),
    set_prior("normal(0, 0.5)",
      class = "b",
      coef = "pretreatmentA",
      resp = "bill"
```

```
      ),
      set_prior("normal(0, 0.5)",
        class = "b",
        coef = "pretreatmentC",
        resp = "bill"
      ),
      set_prior("skew_normal(0, 0.25, 5)",
        class = "b",
        coef = "mass",
        resp = "bill"
      ),
      set_prior("exponential(5)",
        class = "sd",
        group = "batch",
        resp = "bill"
      ),
      set_prior("exponential(2.5)",
        class = "sigma",
        resp = "bill"
      ),
      set_prior("normal(0, 0.1)",
        class = "Intercept",
        resp = "slope"
      ),
      set_prior("normal(0, 0.1)",
        class = "b",
        coef = "pretreatmentA",
        resp = "slope"
      ),
      set_prior("normal(0, 0.1)",
        class = "b",
        coef = "pretreatmentC",
        resp = "slope"
      ),
      set_prior("normal(0, 0.0025)",
        class = "b",
        coef = "mass",
        resp = "slope"
      ),
      set_prior("normal(0, 0.03)",
        class = "b",
        coef = "tarsus",
        resp = "slope"
      ),
      set_prior("exponential(50)",
        class = "sd",
        group = "batch",
        resp = "slope"
      ),
      set_prior("exponential(10)",
        class = "sigma",
        resp = "slope"
      ),
      set_prior("gamma(10, 1)",
        class = "nu",
        resp = "slope"
      )
    ),
    iter = 50000, warmup = 10000, cores = 4, chains = 4, thin = 20,
    control = list(adapt_delta = .98, max_treedepth = 14),
    silent = TRUE, refresh = 0,
    file = "./models/_eightWeekFunctionModelHotR2Bill.Rds"
  )

functionModel8WeeksHotR2Appendage <-
  brm(data = r2modData,
    bf(mass ~ pretreatment + (1 | batch),
```

```
      family = "gaussian"
    ) +
      bf(tarsus ~ mass + pretreatment + (1 | batch),
        family = "gaussian"
      ) +
      bf(bill ~ mass + pretreatment + (1 | batch),
        family = "gaussian"
      ) +
      bf(slope ~ mass + pretreatment + (1 | batch),
        family = student
      ) +
      set_rescor(FALSE),
    prior = c(
      set_prior("normal(0, 10)",
        class = "Intercept",
        resp = "mass"
      ),
      set_prior("normal(0, 25)",
        class = "b",
        coef = "pretreatmentA",
        resp = "mass"
      ),
      set_prior("normal(0, 25)",
        class = "b",
        coef = "pretreatmentC",
        resp = "mass"
      ),
      set_prior("exponential(1.5)",
        class = "sd",
        group = "batch",
        resp = "mass"
      ),
      set_prior("exponential(0.05)",
        class = "sigma",
        resp = "mass"
      ),
      set_prior("normal(0, 3)",
        class = "Intercept",
        resp = "tarsus"
      ),
      set_prior("normal(0, 3)",
        class = "b",
        coef = "pretreatmentA",
        resp = "tarsus"
      ),
      set_prior("normal(0, 3)",
        class = "b",
        coef = "pretreatmentC",
        resp = "tarsus"
      ),
      set_prior("skew_normal(0, 0.25, 5)",
        class = "b",
        coef = "mass",
        resp = "tarsus"
      ),
      set_prior("exponential(2)",
        class = "sd",
        group = "batch",
        resp = "tarsus"
      ),
      set_prior("exponential(0.75)",
        class = "sigma",
        resp = "tarsus"
      ),
      set_prior("normal(0, 1)",
        class = "Intercept",
        resp = "bill"
```

```
        ),
        set_prior("normal(0, 0.5)",
          class = "b",
          coef = "pretreatmentA",
          resp = "bill"
        ),
        set_prior("normal(0, 0.5)",
          class = "b",
          coef = "pretreatmentC",
          resp = "bill"
        ),
        set_prior("skew_normal(0, 0.25, 5)",
          class = "b",
          coef = "mass",
          resp = "bill"
        ),
        set_prior("exponential(5)",
          class = "sd",
          group = "batch",
          resp = "bill"
        ),
        set_prior("exponential(2.5)",
          class = "sigma",
          resp = "bill"
        ),
        set_prior("normal(0, 0.1)",
          class = "Intercept",
          resp = "slope"
        ),
        set_prior("normal(0, 0.1)",
          class = "b",
          coef = "pretreatmentA",
          resp = "slope"
        ),
        set_prior("normal(0, 0.1)",
          class = "b",
          coef = "pretreatmentC",
          resp = "slope"
        ),
        set_prior("normal(0, 0.0025)",
          class = "b",
          coef = "mass",
          resp = "slope"
        ),
        set_prior("exponential(50)",
          class = "sd",
          group = "batch",
          resp = "slope"
        ),
        set_prior("exponential(10)",
          class = "sigma",
          resp = "slope"
        ),
        set_prior("gamma(10, 1)",
          class = "nu",
          resp = "slope"
        )
      ),
      iter = 50000, warmup = 10000, cores = 4, chains = 4, thin = 20,
      control = list(adapt_delta = .98, max_treedepth = 14),
      silent = TRUE, refresh = 0,
      file = "./models/_eightWeekFunctionModelHotR2Appendage.Rds"
    )
}

pR2Pull <- function(x) {
  baseR2 <- as.data.frame(
```

```
    brms::bayes_R2(functionModel8WeeksHot,
      ndraws = 1000,
      resp = "slope", summary = FALSE,
      robust = TRUE
    )
  )$R2slope
  redR2 <- as.data.frame(
    brms::bayes_R2(x,
      ndraws = 1000,
      resp = "slope", summary = FALSE,
      robust = TRUE
    )
  )$R2slope
  pR2 <- round(baseR2 - redR2, digits = 3)

  ciFrame <- t(
    quantile(pR2, probs = c(0.025, 0.975), type = 8)
  ) %>% as.data.frame()

  ciFrame <- ciFrame %>%
    mutate(`2.5%` = ifelse(`2.5%` < 0, 0, `2.5%`)) %>%
    mutate("95\\% CI" = paste0(
      "[",
      round(`2.5%`, digits = 3),
      ",",
      round(`97.5%`, digits = 3),
      "]"
    )) %>%
    mutate("Partial R2" = round(median(pR2), digits = 3)) %>%
    mutate(`Partial R2` = ifelse(`Partial R2` < 0, 0, `Partial R2`)) %>%
    dplyr::select("Partial R\\textsuperscript{2}" = `Partial R2`, "95\\% CI")

  return(ciFrame)
}

caption = paste0('Variance in metabolic slope ',
  '(fold resting metabolsim at thermoneutrality/°C) explained by ',
  'morphometry in eight week old Japanese quail. Metabolic ',
  'slopes are measured above thermoneutrality (<30°C).'
  )

models <- list(functionModel8WeeksHotR2Mass, functionModel8WeeksHotR2Tarsus,
            functionModel8WeeksHotR2Bill, functionModel8WeeksHotR2Appendage)
bind_rows(lapply(models, pR2Pull)) %>%
  mutate("Variable" = c("Body Mass", "Tarsus Length",
                        "Bill Length", "Appendage Length")
         ) %>%
  dplyr::select(Variable, `Partial R\\textsuperscript{2}`, `95\\% CI`) %>%
  kbl(.,
    longtable = T, booktabs = T, format = "latex",
    caption = caption, escape = FALSE
  ) %>%
  column_spec(column = c(1:10), width = "2.5cm")  %>%
  kable_styling(latex_options = "striped")
```

Table 80: *Variance in metabolic slope (fold resting metabolsim at thermoneutrality/°C) explained by morphometry in eight week old Japanese quail. Metabolic slopes are measured above thermoneutrality (<30°C).*

Variable	Partial R^2	95% CI
Body Mass	0.046	[0,0.169]
Tarsus Length	0.025	[0,0.147]
Bill Length	0.000	[0,0.153]
Appendage Length	0.036	[0,0.168]

```
rm(models)
```

To fully visualise the above effects, we below calculate conditional effects of body mass (g), tarsus length (mm), bill length (mm), and rearing temperature (10°C or 30°C) on metabolic responses to heating in our 8 week old quail.

```
showtext.auto(enable = TRUE)

p1 <- data.frame(
  "pretreatment" = c("A", "B", "C"),
  "mass" = 0,
  "tarsus" = 0,
  "bill" = 0
) %>%
  mutate(
    "slope" = predict(functionModel8WeeksHot,
      newdata = .,
      resp = "slope",
      robust = TRUE,
      re_form = NA
    )[, "Estimate"],
    "SE" = predict(functionModel8WeeksHot,
      newdata = .,
      resp = "slope",
      robust = TRUE,
      re_form = NA
    )[, "Est.Error"]
  ) %>%
  mutate(slope = slope + mean(slopeDataHot8$slope, na.rm = T)) %>%
  ggplot(aes(x = pretreatment, y = slope)) +
  geom_errorbar(
    aes(x = pretreatment, ymin = slope - SE, ymax = slope + SE),
    colour = "black", width = 0.3
  ) +
  geom_point(
    size = 5, pch = 21, colour = "black",
    aes(x = pretreatment, fill = factor(pretreatment))
  ) +
  geom_line(linetype = "dashed", colour = "black") +
  geom_point(
    size = 2, pch = 21, colour = "black",
    data = slopeDataHot8 %>%
      mutate(
        "pretreatment" =
          ifelse(pretreatment == "cold", "A",
            ifelse(pretreatment == "neutral", "B", "C")
          )
      ),
    aes(
      x = pretreatment, y = slope,
      fill = factor(pretreatment)
    ),
    position = position_jitter(width = 0.3)
  ) +
  scale_fill_manual(values = c("#7BB4E3", "black", "#CD5C5C")) +
  scale_x_discrete(
    breaks = c("A", "B", "C"),
    labels = c(
      "Cold\n(10°C)",
      "Mild\n(20°C)",
      "Warm\n(30°C)"
    )
  ) +
  xlab("Rearing Conditions") +
  ylab("Metabolic Slope\n(Fold Metabolism at Thermoneutrality/°C)") +
  theme_classic() +
```

```
  theme(
    legend.position = "none",
    axis.text = element_text(family = "Noto Sans"),
    axis.title = element_text(family = "Noto Sans")
  )

p2 <- data.frame(
  "pretreatment" = "B",
  "mass" = seq(min(functionModel8WeeksHot$data$mass, na.rm = T),
    max(functionModel8WeeksHot$data$mass, na.rm = T),
    by = 0.1
  ),
  "tarsus" = 0,
  "bill" = 0
) %>%
  mutate(
    "slope" = predict(functionModel8WeeksHot,
      newdata = .,
      resp = "slope",
      robust = TRUE,
      re_form = NA
    )[, "Estimate"],
    "SE" = predict(functionModel8WeeksHot,
      newdata = .,
      resp = "slope",
      robust = TRUE,
      re_form = NA
    )[, "Est.Error"]
  ) %>%
  mutate("Mass" = mass +
    mean(slopeDataHot8$mass, na.rm = T)) %>%
  mutate("slope" = slope +
    mean(slopeDataHot8$slope, na.rm = T)) %>%
  ggplot(aes(x = Mass, y = slope)) +
  geom_ribbon(
    aes(x = Mass, ymin = slope - SE, ymax = slope + SE),
    fill = "grey50", alpha = 0.5
  ) +
  geom_smooth(
    method = "lm", linetype = "dashed",
    se = FALSE, colour = "black"
  ) +
  geom_point(
    size = 2.5, pch = 21, colour = "black", alpha = 0.5, fill = "grey50",
    data = slopeDataHot8,
    aes(x = mass, y = slope)
  ) +
  xlab("Body Mass (g)") +
  ylab("Metabolic Slope\n(Fold Metabolism at\nThermoneutrality/°C)") +
  theme_classic() +
  theme(
    legend.position = "none",
    axis.text = element_text(family = "Noto Sans"),
    axis.title = element_text(family = "Noto Sans")
  )

p3 <- data.frame(
  "pretreatment" = "B",
  "tarsus" = seq(min(functionModel8WeeksHot$data$tarsus, na.rm = T),
    max(functionModel8WeeksHot$data$tarsus, na.rm = T),
    by = 0.1
  ),
  "mass" = 0,
  "bill" = 0
) %>%
  mutate(
    "slope" = predict(functionModel8WeeksHot,
```

```
      newdata = .,
      resp = "slope",
      robust = TRUE,
      re_form = NA
    )[, "Estimate"],
    "SE" = predict(functionModel8WeeksHot,
      newdata = .,
      resp = "slope",
      robust = TRUE,
      re_form = NA
    )[, "Est.Error"]
  ) %>%
  mutate("Tarsus" = tarsus +
    mean(slopeDataHot8$tarsusLengthMean, na.rm = T)) %>%
  mutate("slope" = slope +
    mean(slopeDataHot8$slope, na.rm = T)) %>%
  ggplot(aes(x = Tarsus, y = slope)) +
  geom_ribbon(
    aes(x = Tarsus, ymin = slope - SE, ymax = slope + SE),
    fill = "grey50", alpha = 0.5
  ) +
  geom_smooth(
    method = "lm", linetype = "dashed",
    se = FALSE, colour = "black"
  ) +
  geom_point(
    size = 2.5, pch = 21, colour = "black",
    alpha = 0.5, fill = "grey50",
    data = slopeDataHot8,
    aes(x = tarsusLengthMean, y = slope)
  ) +
  xlab("Tarsus Length (mm)") +
  ylab("Metabolic Slope\n(Fold Metabolism at\nThermoneutrality/°C)") +
  theme_classic() +
  theme(
    legend.position = "none",
    axis.text = element_text(family = "Noto Sans"),
    axis.title = element_text(family = "Noto Sans")
  )

p4 <- data.frame(
  "pretreatment" = "B",
  "bill" = seq(min(functionModel8WeeksHot$data$bill, na.rm = T),
    max(functionModel8WeeksHot$data$bill, na.rm = T),
    by = 0.1
  ),
  "mass" = 0,
  "tarsus" = 0
) %>%
  mutate(
    "slope" = predict(functionModel8WeeksHot,
      newdata = .,
      resp = "slope",
      robust = TRUE,
      re_form = NA
    )[, "Estimate"],
    "SE" = predict(functionModel8WeeksHot,
      newdata = .,
      resp = "slope",
      robust = TRUE,
      re_form = NA
    )[, "Est.Error"]
  ) %>%
  mutate("Bill" = bill +
    mean(slopeDataHot8$billLengthMean, na.rm = T)) %>%
  mutate("slope" = slope +
    mean(slopeDataHot8$slope, na.rm = T)) %>%
```

```
  ggplot(aes(x = Bill, y = slope)) +
  geom_ribbon(
    aes(x = Bill, ymin = slope - SE, ymax = slope + SE),
    fill = "grey50", alpha = 0.5
  ) +
  geom_smooth(
    method = "lm", linetype = "dashed",
    se = FALSE, colour = "black"
  ) +
  geom_point(
    size = 2.5, pch = 21, colour = "black",
    alpha = 0.5, fill = "grey50",
    data = slopeDataHot8,
    aes(x = billLengthMean, y = slope)
  ) +
  xlab("Bill Length (mm)") +
  ylab("Metabolic Slope\n(Fold Metabolism at\nThermoneutrality/°C)") +
  theme_classic() +
  theme(
    legend.position = "none",
    axis.text = element_text(family = "Noto Sans"),
    axis.title = element_text(family = "Noto Sans")
  )

massPred <- data.frame(
  "pretreatment" = "B",
  "mass" = c(
    mean(functionModel8WeeksHot$data$mass, na.rm = T) -
      sd(functionModel8WeeksHot$data$mass, na.rm = T),
    mean(functionModel8WeeksHot$data$mass, na.rm = T),
    mean(functionModel8WeeksHot$data$mass, na.rm = T) +
      sd(functionModel8WeeksHot$data$mass, na.rm = T)
  ),
  "tarsus" = 0,
  "bill" = 0,
  "batch" = "A"
) %>%
  predict(functionModel8WeeksHot,
    re_form = NA,
    resp = "slope", newdata = .,
    summary = TRUE, robust = TRUE
  ) %>%
  as.data.frame() %>%
  mutate("slope" = Estimate +
    mean(
      subset(
        slopeDataHot8,
        pretreatment == "neutral"
      )$slope,
      na.rm = TRUE
    )) %>%
  mutate("Size" = c("Small", "Average", "Large")) %>%
  dplyr::select(Size, slope, "slopeSE" = Est.Error) %>%
  slice(rep(1:n(), each = 2)) %>%
  mutate("Ta" = rep(c(30, 40), 3)) %>%
  mutate(
    "foldRMR" = ifelse(Ta == 30, 1, 10 * slope),
    "LL" = ifelse(Ta == 30, 1, 10 * (slope - slopeSE)),
    "UL" = ifelse(Ta == 30, 1, 10 * (slope + slopeSE))
  ) %>%
  mutate(Size = factor(Size, levels = c("Small", "Average", "Large")))

massPlot <- massPred %>%
  filter(Ta == 40) %>%
  ggplot(aes(x = Size, y = foldRMR)) +
  geom_point(
    data = modData8 %>%
```

```
      dplyr::select(ring, mass, Ta, VO2) %>%
      pivot_wider(
        id_cols = c("ring", "mass"),
        names_from = "Ta",
        values_from = "VO2"
      ) %>%
      mutate(
        "foldRMR" = `40` / `30`,
        "Ta" = 40,
        "Size" = ifelse(mass < mean(mass, na.rm = T) -
          sd(mass, na.rm = T),
        "Small",
        ifelse(mass > mean(mass, na.rm = T) +
          sd(mass, na.rm = T),
        "Large", "Average"
        )
        )
      ) %>%
      mutate(Size = factor(Size, levels = c("Small", "Average", "Large"))),
    aes(x = Size, y = foldRMR, fill = Size),
    position = position_jitter(width = 0.25), alpha = 0.5,
    colour = "black", pch = 21
  ) +
  geom_hline(yintercept = 1, colour = "grey30", linetype = "dashed") +
  geom_errorbar(aes(ymin = LL, ymax = UL),
    colour = "grey80",
    width = 0.25
  ) +
  geom_errorbar(aes(ymin = LL, ymax = UL),
    colour = "black",
    width = 0.25
  ) +
  geom_point(aes(x = Size, y = foldRMR, fill = Size),
    size = 4, colour = "black", pch = 21
  ) +
  ylim(c(0, 2)) +
  theme_classic() +
  ylab("Resting Metabolism\n(Fold Change from Thermoneutrality)") +
  scale_fill_manual(
    values = c("#B35050", "#EABCBC", "white"),
    guide = NULL
  ) +
  scale_x_discrete(
    labels = c("Mean -\n1 SD", "Mean", "Mean +\n1 SD"),
    name = "Body Mass"
  ) +
  theme(
    axis.text = element_text(family = "Noto Sans"),
    axis.title = element_text(family = "Noto Sans"),
    legend.text = element_text(family = "Noto Sans", size = 8),
    legend.title = element_text(family = "Noto Sans", size = 8),
    legend.position = "right"
  )

ggsave("./plots/massPlotHot8Weeks.pdf",
  dpi = 800,
  width = 7, height = 7,
  massPlot
)

rm(massPred)

tarsusPred <- data.frame(
  "pretreatment" = "B",
  "tarsus" = c(
    mean(functionModel8WeeksHot$data$tarsus, na.rm = T) -
      sd(functionModel8WeeksHot$data$tarsus, na.rm = T),
```

```
    mean(functionModel8WeeksHot$data$tarsus, na.rm = T),
    mean(functionModel8WeeksHot$data$tarsus, na.rm = T) +
      sd(functionModel8WeeksHot$data$tarsus, na.rm = T)
  ),
  "mass" = 0,
  "bill" = 0,
  "batch" = "A"
) %>%
  predict(functionModel8WeeksHot,
    re_form = NA,
    resp = "slope", newdata = .,
    summary = TRUE, robust = TRUE
  ) %>%
  as.data.frame() %>%
  mutate("slope" = Estimate +
    mean(
      subset(
        slopeDataHot8,
        pretreatment == "neutral"
      )$slope,
      na.rm = TRUE
    )) %>%
  mutate("Size" = c("Small", "Average", "Large")) %>%
  dplyr::select(Size, slope, "slopeSE" = Est.Error) %>%
  slice(rep(1:n(), each = 2)) %>%
  mutate("Ta" = rep(c(30, 40), 3)) %>%
  mutate(
    "foldRMR" = ifelse(Ta == 30, 1, 10 * slope),
    "LL" = ifelse(Ta == 30, 1, 10 * (slope - slopeSE)),
    "UL" = ifelse(Ta == 30, 1, 10 * (slope + slopeSE))
  ) %>%
  mutate(Size = factor(Size, levels = c("Small", "Average", "Large")))

tarsusPlot <- tarsusPred %>%
  filter(Ta == 40) %>%
  ggplot(aes(x = Size, y = foldRMR)) +
  geom_point(
    data = modData8 %>%
      dplyr::select(ring, "tarsus" = tarsusLengthMean, Ta, VO2) %>%
      pivot_wider(
        id_cols = c("ring", "tarsus"),
        names_from = "Ta",
        values_from = "VO2"
      ) %>%
      mutate(
        "foldRMR" = `40` / `30`,
        "Ta" = 40,
        "Size" = ifelse(tarsus < mean(tarsus, na.rm = T) -
          sd(tarsus, na.rm = T),
        "Small",
        ifelse(tarsus > mean(tarsus, na.rm = T) +
          sd(tarsus, na.rm = T),
        "Large", "Average"
        )
        )
      ) %>%
      drop_na() %>%
      mutate(Size = factor(Size, levels = c("Small", "Average", "Large"))),
    aes(x = Size, y = foldRMR, fill = Size),
    position = position_jitter(width = 0.25), alpha = 0.5,
    pch = 21, colour = "black"
  ) +
  geom_hline(yintercept = 1, colour = "grey30", linetype = "dashed") +
  geom_errorbar(aes(ymin = LL, ymax = UL),
    colour = "grey80",
    width = 0.25
  ) +
```

```
  geom_errorbar(aes(ymin = LL, ymax = UL),
    colour = "black",
    width = 0.25
  ) +
  geom_point(aes(x = Size, y = foldRMR, fill = Size),
    size = 4, colour = "black", pch = 21
  ) +
  theme_classic() +
  xlab("Tarsus Length") +
  scale_x_discrete(labels = c(
    "Mean -\n1 SD",
    "Mean",
    "Mean +\n1 SD"
  )) +
  ylim(c(0, 3)) +
  ylab("Resting Metabolism\n(Fold Change from Thermoneutrality)") +
  scale_fill_manual(
    values = c("#B35050", "#EABCBC", "white"),
    guide = NULL
  ) +
  theme(
    axis.text = element_text(family = "Noto Sans"),
    axis.title = element_text(family = "Noto Sans"),
    legend.text = element_text(family = "Noto Sans", size = 8),
    legend.title = element_text(family = "Noto Sans", size = 8),
    legend.position = "right"
  )

ggsave("./plots/tarsusPlotHot8Weeks.pdf",
  dpi = 800,
  width = 7, height = 7,
  tarsusPlot
)

billPred <- data.frame(
  "pretreatment" = "B",
  "bill" = c(
    mean(functionModel8WeeksHot$data$bill, na.rm = T) -
      sd(functionModel8WeeksHot$data$bill, na.rm = T),
    mean(functionModel8WeeksHot$data$bill, na.rm = T),
    mean(functionModel8WeeksHot$data$bill, na.rm = T) +
      sd(functionModel8WeeksHot$data$bill, na.rm = T)
  ),
  "mass" = 0,
  "tarsus" = 0,
  "batch" = "A"
) %>%
  predict(functionModel8WeeksHot,
    re_form = NA,
    resp = "slope", newdata = .,
    summary = TRUE, robust = TRUE
  ) %>%
  as.data.frame() %>%
  mutate("slope" = Estimate +
    mean(
      subset(
        slopeDataHot8,
        pretreatment == "neutral"
      )$slope,
      na.rm = TRUE
    )) %>%
  mutate("Size" = c("Small", "Average", "Large")) %>%
  dplyr::select(Size, slope, "slopeSE" = Est.Error) %>%
  slice(rep(1:n(), each = 2)) %>%
  mutate("Ta" = rep(c(30, 40), 3)) %>%
  mutate(
    "foldRMR" = ifelse(Ta == 30, 1, 10 * slope),
```

```
    "LL" = ifelse(Ta == 30, 1, 10 * (slope - slopeSE)),
    "UL" = ifelse(Ta == 30, 1, 10 * (slope + slopeSE))
  ) %>%
  mutate(Size = factor(Size, levels = c("Small", "Average", "Large")))

billPlot <- billPred %>%
  filter(Ta == 40) %>%
  ggplot(aes(x = Size, y = foldRMR)) +
  geom_point(
    data = modData8 %>%
      select(ring, "bill" = billLengthMean, Ta, VO2) %>%
      pivot_wider(
        id_cols = c("ring", "bill"),
        names_from = "Ta",
        values_from = "VO2"
      ) %>%
      mutate(
        "foldRMR" = `40` / `30`,
        "Ta" = 40,
        "Size" = ifelse(bill < mean(bill, na.rm = T) -
          sd(bill, na.rm = T),
        "Small",
        ifelse(bill > mean(bill, na.rm = T) +
          sd(bill, na.rm = T),
        "Large", "Average"
        )
        )
      ) %>%
      drop_na() %>%
      mutate(Size = factor(Size, levels = c("Small", "Average", "Large"))),
    aes(x = Size, y = foldRMR, fill = Size),
    position = position_jitter(width = 0.25), alpha = 0.5,
    pch = 21, colour = "black"
  ) +
  geom_hline(yintercept = 1, colour = "grey30", linetype = "dashed") +
  geom_errorbar(aes(ymin = LL, ymax = UL),
    colour = "grey80",
    width = 0.25
  ) +
  geom_errorbar(aes(ymin = LL, ymax = UL),
    colour = "black",
    width = 0.25
  ) +
  geom_point(aes(x = Size, y = foldRMR, fill = Size),
    size = 4, colour = "black", pch = 21
  ) +
  theme_classic() +
  xlab("Bill Length") +
  scale_x_discrete(labels = c(
    "Mean -\n1 SD",
    "Mean",
    "Mean +\n1 SD"
  )) +
  ylim(c(0, 3)) +
  ylab("Resting Metabolism\n(Fold Change from Thermoneutrality)") +
  scale_fill_manual(
    values = c("#B35050", "#EABCBC", "white"),
    guide = NULL
  ) +
  theme(
    axis.text = element_text(family = "Noto Sans"),
    axis.title = element_text(family = "Noto Sans"),
    legend.text = element_text(family = "Noto Sans", size = 8),
    legend.title = element_text(family = "Noto Sans", size = 8),
    legend.position = "right"
  )
```

```r
layout <- "
  AABC
  AADE
  ##FG
"

allMorphology <-
  p1 + p2 + massPlot + p3 + tarsusPlot + p4 + billPlot +
  plot_annotation(tag_levels = "A") +
  plot_layout(design = layout)

allMorphology
```

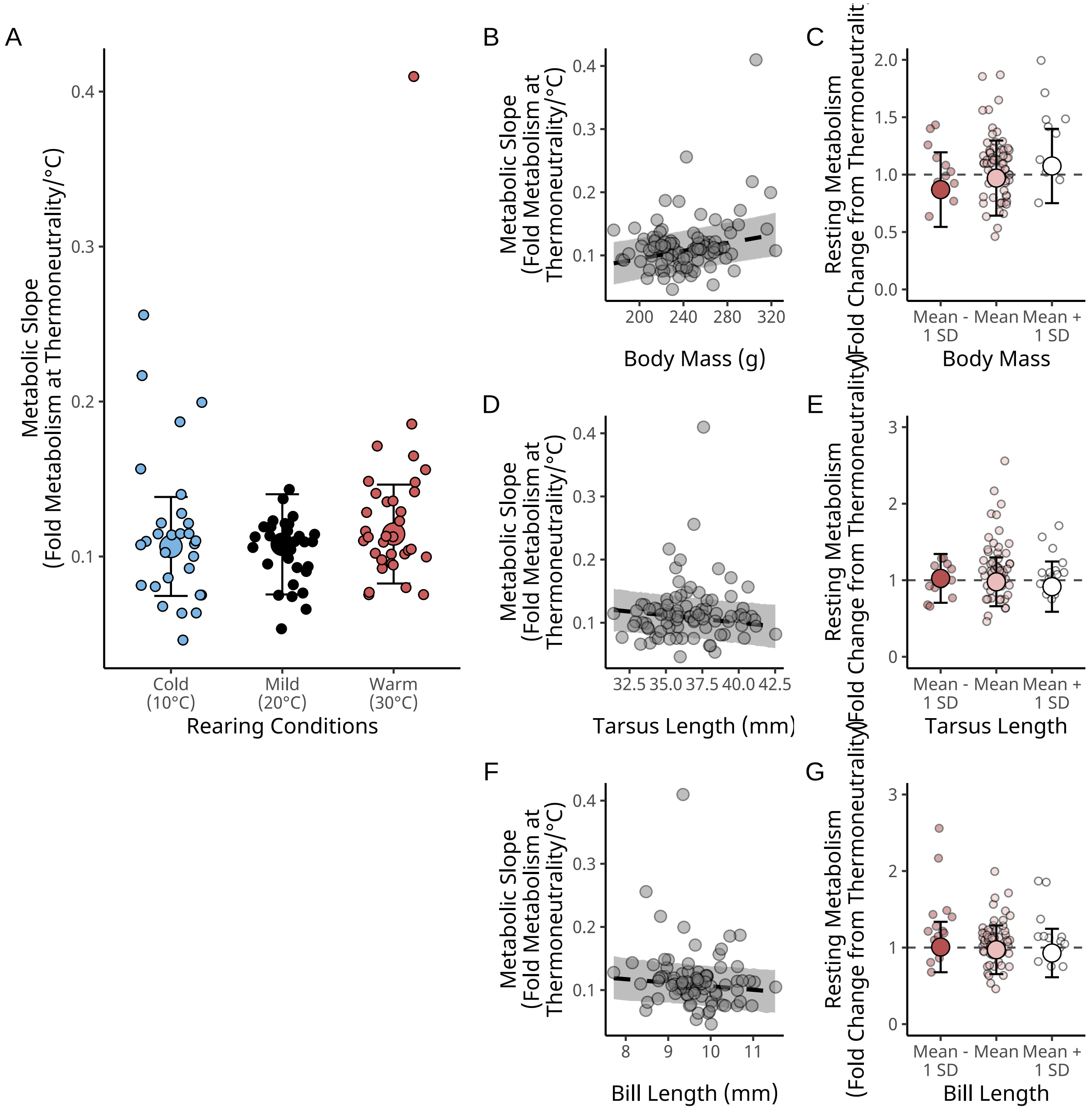

Figure 139: *Conditional effects of post-hatch rearing environments (10°C, 20°C or 30°C) and morphometry (body mass [g], tarsus length [mm], bill length [mm]) on physiological responses to heat in eight week old Japanese quail. Dots represent raw data points per individual. Large dots in panel A represent estimated effects at average body mass, tarsus length, and bill length values, and errorbars indicate +/- one standard error around means. Large dots in panels C, E, and G represent estimated effects at mild rearing temperatures and otherwise average morphometry. Again, errorbars indicate +/- one standard error around means. Lines in panels B, D and F indicate estimate effects across given parameters and grouping (assuming mild [20°C] rearing conditions) and ribbons indicate standard errors around effects. All estimated effects are drawn from a Bayesian path analysis.*

```
ggsave("./plots/morphologyEffectsResistanceHeat8Weeks.pdf",
  allMorphology,
  dpi = 800, height = 7, width = 9
)
```

```
showtext.auto(enable = "FALSE")
```

Similar to above, we then estimate costs of mismatching morphology with expectations of Bergmann's and Allen's rules in the heat. Here, mismatches represent having a body mass two standard deviations above the mean, relative tarsus length two standard deviations below the mean, or bill length to standard deviations below the mean.

```
data.frame(
  "Size" = c("Average", "Large (2x s.d. > mean)"),
  "pretreatment" = "B",
  "mass" = c(
    mean(functionModel8WeeksHot$data$mass, na.rm = T),
    mean(functionModel8WeeksHot$data$mass, na.rm = T) +
      2 * sd(functionModel8WeeksHot$data$mass, na.rm = T)
  ),
  "tarsus" = 0,
  "bill" = 0,
  "batch" = "A"
) %>%
  mutate("slope" = predict(functionModel8WeeksHot,
    newdata = .,
    robust = TRUE, re_form = NA,
    resp = "slope"
  )[, "Estimate"]) %>%
  mutate(slope =
           (slope + mean(slopeDataHot8$slope, na.rm = T))*10
         ) %>%
  dplyr::select("Body Size" = Size, "Metabolic Slope (fold RMR/°C)" = slope) %>%
  kbl(.,
    longtable = T, booktabs = T, format = "latex",
    caption = paste0("Comparison of predicted metabolic responses to 40°C ",
                     "among eight week old Japanese quail > 30°C of ",
                     "varying body sizes."),
    escape = FALSE
  ) %>%
  column_spec(column = c(1:10), width = "2.5cm")  %>%
  kable_styling(latex_options = "striped")
```

Table 81: *Comparison of predicted metabolic responses to 40°C among eight week old Japanese quail > 30°C of varying body sizes.*

Body Size	Metabolic Slope (fold RMR/°C)
Average	1.088742
Large (2x s.d. > mean)	1.277988

```
data.frame(
  "Size" = c("Average", "Short (2x s.d. < mean)"),
  "pretreatment" = "B",
  "tarsus" = c(
    mean(functionModel8WeeksHot$data$tarsus, na.rm = T),
    mean(functionModel8WeeksHot$data$tarsus, na.rm = T) -
      2 * sd(functionModel8WeeksHot$data$tarsus, na.rm = T)
  ),
  "mass" = 0,
  "bill" = 0,
  "batch" = "A"
) %>%
  mutate("slope" = predict(functionModel8WeeksHot,
    newdata = .,
    robust = TRUE, re_form = NA,
    resp = "slope"
```

```
  )[, "Estimate"]) %>%
  mutate(slope = slope +
        mean(
      subset(
        slopeDataHot8,
        pretreatment == "neutral"
      )$slope,
      na.rm = T
    )) %>%
  dplyr::select("Tarsus Length" = Size,
                "Metabolic Slope (fold RMR/°C)" = slope) %>%
  kbl(.,
    longtable = T, booktabs = T, format = "latex",
    caption = paste0("Comparison of metabolic slopes among ",
                     "eight week old Japanese quail > 30°C ",
                     "and varying tarsus lengths."),
    escape = FALSE
  ) %>%
  column_spec(column = c(1:10), width = "2.5cm")  %>%
  kable_styling(latex_options = "striped")
```

Table 82: *Comparison of metabolic slopes among eight week old Japanese quail > 30°C and varying tarsus lengths.*

Tarsus Length	Metabolic Slope (fold RMR/°C)
Average	0.0970832
Short (2x s.d. < mean)	0.1082584

```
data.frame(
  "Size" = c("Average", "Short (2x s.d. < mean)"),
  "pretreatment" = "B",
  "bill" = c(
    mean(functionModel8WeeksHot$data$bill, na.rm = T),
    mean(functionModel8WeeksHot$data$bill, na.rm = T) -
      2 * sd(functionModel8WeeksHot$data$bill, na.rm = T)
  ),
  "mass" = 0,
  "tarsus" = 0,
  "batch" = "A"
) %>%
  mutate("slope" = predict(functionModel8WeeksHot,
    newdata = .,
    robust = TRUE, re_form = NA,
    resp = "slope"
  )[, "Estimate"]) %>%
  mutate(slope = slope +
        mean(
      subset(
        slopeDataHot8,
        pretreatment == "neutral"
      )$slope,
      na.rm = T
    )) %>%
  dplyr::select("Bill Length" = Size,
                "Metabolic Slope (fold RMR/°C)" = slope) %>%
  kbl(.,
    longtable = T, booktabs = T, format = "latex",
    caption = paste0("Comparison of metabolic slopes among ",
                     "eight week old Japanese quail > 30°C ",
                     "and varying bill lengths."),
    escape = FALSE
  ) %>%
  column_spec(column = c(1:10), width = "2.5cm")  %>%
```

```
  kable_styling(latex_options = "striped")
```

Table 83: *Comparison of metabolic slopes among eight week old Japanese quail > 30°C and varying bill lengths.*

Bill Length	Metabolic Slope (fold RMR/°C)
Average	0.0970988
Short (2x s.d. < mean)	0.1049616

```
# Checking whether these differences are statistically clear

mismatchDFMass <- data.frame(
  "Size" = c("Average", "Large (2x s.d. > mean)"),
  "pretreatment" = "B",
  "mass" = c(
    mean(functionModel8WeeksHot$data$mass, na.rm = T),
    mean(functionModel8WeeksHot$data$mass, na.rm = T) +
      2 * sd(functionModel8WeeksHot$data$mass, na.rm = T)
  ),
  "tarsus" = 0,
  "bill" = 0,
  "batch" = "A"
)

mismatchDFTarsus <- data.frame(
  "Size" = c("Average", "Short (2x s.d. < mean)"),
  "pretreatment" = "B",
  "tarsus" = c(
    mean(functionModel8WeeksHot$data$tarsus, na.rm = T),
    mean(functionModel8WeeksHot$data$tarsus, na.rm = T) -
      2 * sd(functionModel8WeeksHot$data$tarsus, na.rm = T)
  ),
  "mass" = 0,
  "bill" = 0,
  "batch" = "A"
)

mismatchDFBill <- data.frame(
  "Size" = c("Average", "Short (2x s.d. < mean)"),
  "pretreatment" = "B",
  "bill" = c(
    mean(functionModel8WeeksHot$data$bill, na.rm = T),
    mean(functionModel8WeeksHot$data$bill, na.rm = T) -
      2 * sd(functionModel8WeeksHot$data$bill, na.rm = T)
  ),
  "mass" = 0,
  "tarsus" = 0,
  "batch" = "A"
)

mismatchTestFun <- function(x) {
  df <- predict(functionModel8WeeksHot,
    newdata = x,
    robust = TRUE, re_form = NA,
    resp = "slope", summary = FALSE
  ) %>%
    as.data.frame() %>%
    rename("Average" = "V1", "Mismatch" = "V2") %>%
    mutate_all(.funs = function(x) {
      x + mean(slopeDataHot8$slope, na.rm = T)
    })

  mismatchDFTest <- build_hdf(
```

```
    vars = list(
      df$Average,
      df$Mismatch
    ),
    priors = list(
      rnorm(nrow(df), 0, 0.01),
      rnorm(nrow(df), 0, 0.01)
    ),
    names = c("Average", "Mismatch")
  )
  mismatchTest <- hypothesis_df("Mismatch > Average",
    mismatchDFTest,
    class = "b", alpha = 0.05
  )

  mismatchTest$hypothesis$Hypothesis <-
    "Mismatch Metabolic Slope > Average Slope"

  return(mismatchTest)
}

mismatchTests <- lapply(
  list(mismatchDFMass, mismatchDFTarsus, mismatchDFBill),
  mismatchTestFun
  )

# Summarising

caption <- paste0(
  "Results of a non-linear hypothesis ",
  "tests comparing predicted metabolic slopes in the warmth (30°C - 40°C) ",
  "among eight week old Japanese quail of average or atypically large ",
  "body size (mean + 2 standard deviations). Posterior probabilities are ",
  "calculated using the Savage-Dickey ",
  "density ratio method."
)

mismatchTestMassTable <- mismatchTests[[1]]$hypothesis %>%
  mutate(Hypothesis = c(
    "Large Metabolic Slope > Average Slope"
  )) %>%
  dplyr::select(-c(Evid.Ratio, Star)) %>%
  mutate(
    Estimate = round(Estimate, digits = 4),
    "Est.Error" = round(Est.Error, digits = 4),
    "CI.Lower" = round(CI.Lower, digits = 4),
    "CI.Upper" = round(CI.Upper, digits = 4),
    "Posterior Probability" = round(Post.Prob, digits = 4)
  ) %>%
  rename(
    "Difference Between Metabolic Slopes" = Estimate,
    "Standard Error" = Est.Error,
    `2.5 % CI` = "CI.Lower",
    `97.5 % CI` = "CI.Upper"
  ) %>%
  dplyr::select(-Post.Prob) %>%
  kbl(.,
    longtable = T, booktabs = T, format = "latex",
    caption = caption
  ) %>%
  column_spec(column = c(1:2), width = "2.5cm") %>%
  column_spec(column = c(3:10), width = "2cm") %>%
  kable_styling(latex_options = "striped")

mismatchTestMassTable
```

Table 84: *Results of a non-linear hypothesis tests comparing predicted metabolic slopes in the warmth (30°C - 40°C) among eight week old Japanese quail of average or atypically large body size (mean + 2 standard deviations). Posterior probabilities are calculated using the Savage-Dickey density ratio method.*

Hypothesis	Difference Between Metabolic Slopes	Standard Error	2.5 % CI	97.5 % CI	Posterior Probability
Large Metabolic Slope > Average Slope	0.0188	0.0529	-0.062	0.1031	0.6611

```
# For tarsus length

caption <- paste0(
  "Results of a non-linear hypothesis ",
  "tests comparing predicted metabolic slopes in the warmth (30°C - 14°C) ",
  "among eight week old Japanese quail of average or atypically short ",
  "tarsus length (mean - 2 standard deviations). Posterior probabilities are ",
  "calculated using the Savage-Dickey ",
  "density ratio method."
)

mismatchTestTarsusTable <- mismatchTests[[2]]$hypothesis %>%
  mutate(Hypothesis = c(
    "Short Metabolic Slope < Average Slope"
  )) %>%
  dplyr::select(-c(Evid.Ratio, Star)) %>%
  mutate(
    Estimate = round(Estimate, digits = 4),
    "Est.Error" = round(Est.Error, digits = 4),
    "CI.Lower" = round(CI.Lower, digits = 4),
    "CI.Upper" = round(CI.Upper, digits = 4),
    "Posterior Probability" = round(Post.Prob, digits = 4)
  ) %>%
  rename(
    "Difference Between Metabolic Slopes" = Estimate,
    "Standard Error" = Est.Error,
    `2.5 % CI` = "CI.Lower",
    `97.5 % CI` = "CI.Upper"
  ) %>%
  dplyr::select(-Post.Prob) %>%
  kbl(.,
    longtable = T, booktabs = T, format = "latex",
    caption = caption
  ) %>%
  column_spec(column = c(1:2), width = "2.5cm") %>%
  column_spec(column = c(3:10), width = "2cm") %>%
  kable_styling(latex_options = "striped")

mismatchTestTarsusTable
```

Table 85: *Results of a non-linear hypothesis tests comparing predicted metabolic slopes in the warmth (30°C - 14°C) among eight week old Japanese quail of average or atypically short tarsus length (mean - 2 standard deviations). Posterior probabilities are calculated using the Savage-Dickey density ratio method.*

Hypothesis	Difference Between Metabolic Slopes	Standard Error	2.5 % CI	97.5 % CI	Posterior Probability
Short Metabolic Slope < Average Slope	0.0116	0.0534	-0.07	0.0939	0.5978

```
## Bill length
```

```
caption <- paste0(
  "Results of a non-linear hypothesis ",
  "tests comparing predicted metabolic slopes in the warmth (30°C - 40°C) ",
  "among eight week old Japanese quail of average or short long ",
  "bill length (mean - 2 standard deviations). Posterior probabilities are ",
  "calculated using the Savage-Dickey ",
  "density ratio method."
)

mismatchTestBillTable <- mismatchTests[[3]]$hypothesis %>%
  mutate(Hypothesis = c(
    "Short Metabolic Slope > Average Slope"
  )) %>%
  dplyr::select(-c(Evid.Ratio, Star)) %>%
  mutate(
    Estimate = round(Estimate, digits = 4),
    "Est.Error" = round(Est.Error, digits = 4),
    "CI.Lower" = round(CI.Lower, digits = 4),
    "CI.Upper" = round(CI.Upper, digits = 4),
    "Posterior Probability" = round(Post.Prob, digits = 4)
  ) %>%
  rename(
    "Difference Between Metabolic Slopes" = Estimate,
    "Standard Error" = Est.Error,
    `2.5 % CI` = "CI.Lower",
    `97.5 % CI` = "CI.Upper"
  ) %>%
  dplyr::select(-Post.Prob) %>%
  kbl(.,
    longtable = T, booktabs = T, format = "latex",
    caption = caption
  ) %>%
  column_spec(column = c(1:2), width = "2.5cm") %>%
  column_spec(column = c(3:10), width = "2cm") %>%
  kable_styling(latex_options = "striped")

mismatchTestBillTable
```

Table 86: *Results of a non-linear hypothesis tests comparing predicted metabolic slopes in the warmth (30°C - 40°C) among eight week old Japanese quail of average or short long bill length (mean - 2 standard deviations). Posterior probabilities are calculated using the Savage-Dickey density ratio method.*

Hypothesis	Difference Between Metabolic Slopes	Standard Error	2.5 % CI	97.5 % CI	Posterior Probability
Short Metabolic Slope > Average Slope	0.0081	0.0513	-0.0733	0.0886	0.5724

```
# Further, plotting estimated effects of body size enlargement, relative to
# average, on estimated rates of metabolic heat production

posteriorProbabilities <- bind_rows(
  lapply(X = seq(0, 5, by = 0.05),
      FUN = function(x){
        data <- data.frame(
         "tarsus" = 0,
         "pretreatment" = "B",
         "mass" = c(0, x * sd(
           functionModel8WeeksHot$data$mass,
           na.rm = T
         )),
         "bill" = 0,
         "batch" = "B"
       ) %>% predict(functionModel8WeeksHot, newdata = .,
           robust = TRUE, resp = "slope",
```

```
              summary = FALSE) %>%
           as.data.frame() %>%
           rename("Average" = "V1", "Large" = "V2") %>%
           mutate_all(.funs = function(x)
             {x + mean(slopeDataHot8$slope, na.rm = T)}
             )

         testDF <- build_hdf(
           vars = list(
             data$Average,
             data$Large
             ),
           priors = list(
             rnorm(nrow(data), 0, 0.01),
             rnorm(nrow(data), 0, 0.01)
             ),
           names = c("Average", "Large")
         )

         pp <- hypothesis_df("Large > Average", testDF,
                  class = "b", alpha = 0.5)$hypothesis$Post.Prob

         return(data.frame("sd" = x, "pp" = pp))
       }
  )
)

bind_rows(
    lapply(
      X = seq(0.1, 5, by = 0.1),
      FUN = function(x) {
        data <- data.frame(
          "tarsus" = 0,
          "pretreatment" = "B",
          "mass" = c(0, x * sd(
            functionModel8WeeksHot$data$mass,
            na.rm = T
          )),
          "bill" = 0,
          "batch" = "B"
        ) %>%
          predict(functionModel8WeeksHot,
            newdata = .,
            robust = TRUE, ndraws = 8000,
            resp = "slope", summary = FALSE
          ) %>%
          as.data.frame() %>%
          mutate_all(.funs = function(x){x + mean(slopeDataHot8$slope)}) %>%
          mutate("deltaRaw" = ((V2/V1)-1)*100) %>%
          summarise(
            "delta" = median(deltaRaw),
            "LL80" = quantile(deltaRaw, 0.1, type = 8),
            "UL80" = quantile(deltaRaw, 0.9, type = 8),
            "LL95" = quantile(deltaRaw, 0.025, type = 8),
            "UL95" = quantile(deltaRaw, 0.975, type = 8)
          ) %>%
          mutate("sd" = x) %>%
          select(sd, delta, LL80, UL80, LL95, UL95)
        return(data)
      }
    )
) %>%
  ggplot(aes(x = sd, y = delta)) +
  geom_ribbon(aes(ymin = LL95, ymax = UL95),
              fill = "#DECCC1", alpha = 0.5) +
  geom_ribbon(aes(ymin = LL80, ymax = UL80),
              fill = "#DECCC1", alpha = 0.8) +
```

```
  geom_smooth(method = "lm", colour = "black", linetype = "dashed",
              se = FALSE) +
  geom_line(data = posteriorProbabilities, colour = "black",
            linetype = "solid", aes(x = sd, y = pp*600)) +
  geom_hline(yintercept = 0, linetype = "dotted",
             colour = "grey50") +
  xlab("Standard Deviations\nAbove Mean Mass") +
  scale_y_continuous(name = "Increase in\nMetabolic Slopes (%)",
                     sec.axis = sec_axis(trans=~./6,
                                         name="Posterior Probability (%)")
                     ) +
  theme_classic()
```

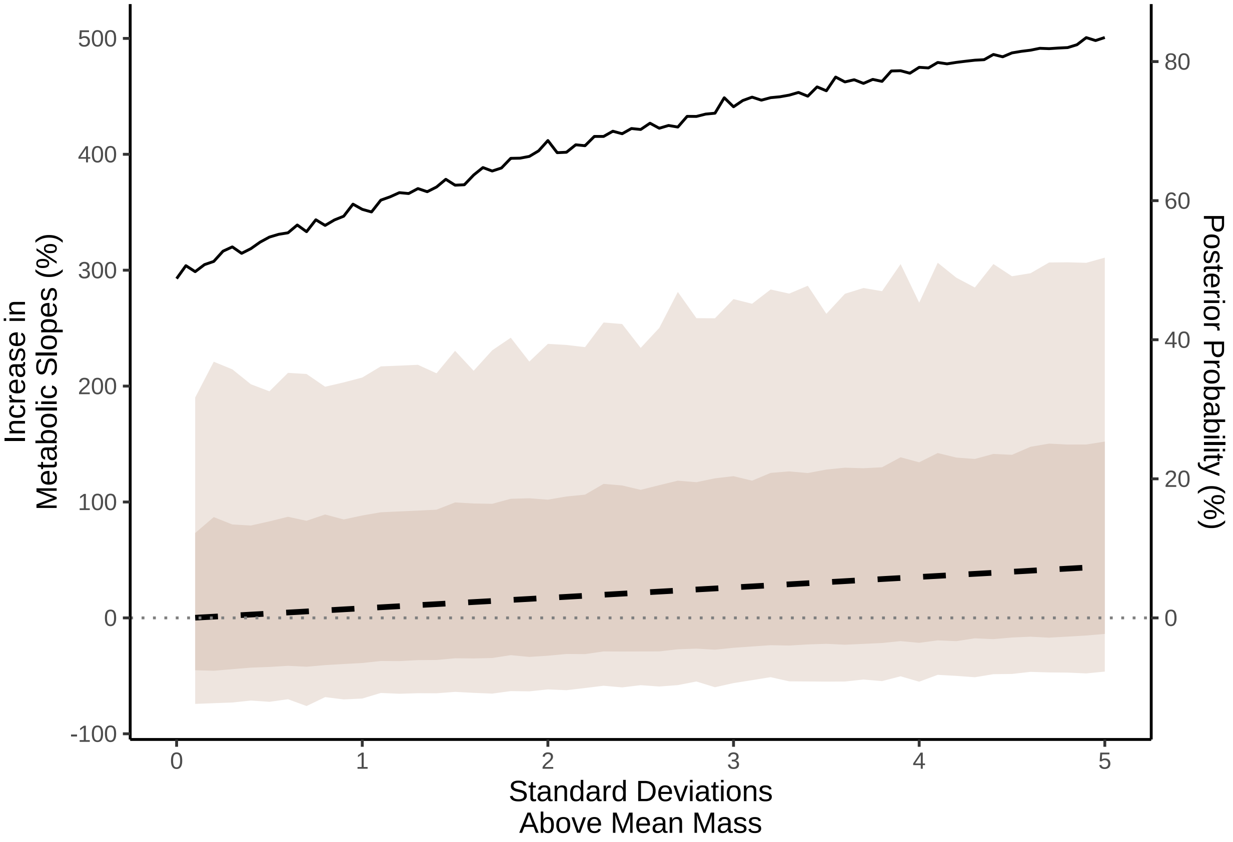

Figure 140: *Effect of increasing mass (in standard deviations) above the average on the percent change of metabolic slope above 40°C in mature Japanese quail. Effects are estimated from a Bayesian path analysis. The dark ribbon represents 50% credible intervals around the estimated change in metabolic slope and the light ribbon represents 95% credible intervals around the predicted change. The dashed line represents the predicted relationship between the change in metabolic slope and relative increase in body mass above the average. The solid line indicate the posterior probability of an increase in metabolic slope among individuals of a given size above average. All predictions assume rearing in mild conditions (20°C) and average tarsus length. The horizontal dotted line indicates 0.*

```
# Last, it's important to note that having a large body size and average sized
# appendages is unlikely. Below, we calculate the odds that body mass is two
# standard deviations above the mean, but tarsus length or bill length are less
# than one quarter of a standard deviation above the mean

hyp <- paste0(
  "tarsus_mass*",
  2 * sd(functionModel8WeeksHot$data$mass),
```

```
  " < ",
  0.25 * sd(functionModel8WeeksHot$data$tarsus)
)
hypothesis(hypothesis = hyp, functionModel8WeeksHot)$hypothesis$Post.Prob
```

```
## [1] 0.0065
```

```
hyp <- paste0(
  "bill_mass*",
  2 * sd(functionModel8WeeksHot$data$mass),
  " < ",
  0.25 * sd(functionModel8WeeksHot$data$bill)
)
hypothesis(hypothesis = hyp, functionModel8WeeksHot)$hypothesis$Post.Prob
```

```
## [1] 0.9
```

```
# Probability is low for tarsus length. As such, we calculate the assumed costs
# of mismatching body size when allometry is accounted for

data.frame(
  "Size" = c("Average", "Large (2x s.d. > mean)"),
  "pretreatment" = "B",
  "mass" = c(
    mean(functionModel8WeeksHot$data$mass, na.rm = T),
    mean(functionModel8WeeksHot$data$mass, na.rm = T) +
      2 * sd(functionModel8WeeksHot$data$mass, na.rm = T)
  )
) %>%
  mutate(
    "tarsus" = predict(functionModel8WeeksHot,
      newdata = .,
      resp = "tarsus", re_form = NA,
      robust = TRUE
    )[, "Estimate"],
    "bill" = predict(functionModel8WeeksHot,
      newdata = .,
      resp = "bill", re_form = NA,
      robust = TRUE
    )[, "Estimate"]
  ) %>%
  mutate("slope" = predict(functionModel8WeeksHot,
    newdata = .,
    resp = "slope", re_form = NA,
    robust = TRUE
  )[, "Estimate"]) %>%
  mutate(slope = slope +
    mean(slopeDataHot8$slope, na.rm = T)) %>%
  dplyr::select(
    "Body Mass" = Size,
    "Metabolic Slope" = slope
  )
```

```
## # A tibble: 2 x 2
##   `Body Mass`            `Metabolic Slope`
##   <chr>                              <dbl>
## 1 Average                            0.110
## 2 Large (2x s.d. > mean)             0.125
```

```
mismatchDFMassAllometric <- data.frame(
  "Size" = c("Average", "Large (2x s.d. > mean)"),
  "pretreatment" = "B",
  "mass" = c(
    mean(functionModel8WeeksHot$data$mass, na.rm = T),
    mean(functionModel8WeeksHot$data$mass, na.rm = T) +
      2 * sd(functionModel8WeeksHot$data$mass, na.rm = T)
  )
) %>%
  mutate(
```

```
    "tarsus" = predict(functionModel8WeeksHot,
      newdata = .,
      robust = TRUE, re_form = NA,
      resp = "tarsus", summary = TRUE
    )[, "Estimate"],
    "bill" = predict(functionModel8WeeksHot,
      newdata = .,
      robust = TRUE, re_form = NA,
      resp = "bill", summary = TRUE
    )[, "Estimate"]
  ) %>%
  predict(functionModel8WeeksHot,
    newdata = .,
    robust = TRUE, re_form = NA,
    resp = "slope", summary = FALSE
  ) %>%
  as.data.frame() %>%
  rename("Average" = "V1", "Large" = "V2") %>%
  mutate_all(.funs = function(x) {
    x + mean(slopeDataHot8$slope, na.rm = T)
  })

mismatchDFMassTestAllometric <- build_hdf(
  vars = list(
    mismatchDFMassAllometric$Average,
    mismatchDFMassAllometric$Large
  ),
  priors = list(
    rnorm(nrow(mismatchDFMassAllometric), 0, 0.01),
    rnorm(nrow(mismatchDFMassAllometric), 0, 0.01)
  ),
  names = c("Average", "Large")
)

mismatchTestMassAllometric <- hypothesis_df("Large > Average",
  mismatchDFMassTestAllometric,
  class = "b", alpha = 0.05
)

mismatchTestMassAllometric$hypothesis$Hypothesis <-
  "Large Metabolic Slope > Average Slope"

# And summarising

caption <- paste0(
  "Results of a non-linear hypothesis ",
  "tests comparing predicted metabolic slopes in the heat (30°C - 40°C) ",
  "among eight week old Japanese quail of average or atypically large ",
  "body size (mean + 2 standard deviations), while assuming ",
  "that appendage lengths scale allometrically . Posterior probabilities are ",
  "calculated using the Savage-Dickey ",
  "density ratio method."
)

mismatchTestMassTable <- mismatchTestMassAllometric$hypothesis %>%
  mutate(Hypothesis = c(
    "Large Metabolic Slope > Average Slope"
  )) %>%
  dplyr::select(-c(Evid.Ratio, Star)) %>%
  mutate(
    Estimate = round(Estimate, digits = 4),
    "Est.Error" = round(Est.Error, digits = 4),
    "CI.Lower" = round(CI.Lower, digits = 4),
    "CI.Upper" = round(CI.Upper, digits = 4)
  ) %>%
  rename(
    "Difference Between Metabolic Slopes" = Estimate,
```

```
    "Standard Error" = Est.Error,
    `2.5 % CI` = "CI.Lower",
    `97.5 % CI` = "CI.Upper",
    "Posterior Probability" = Post.Prob
  ) %>%
  kbl(.,
    longtable = T, booktabs = T, format = "latex",
    caption = caption
  ) %>%
  column_spec(column = c(1:2), width = "2.5cm") %>%
  column_spec(column = c(3:10), width = "2cm") %>%
  kable_styling(latex_options = "striped")

mismatchTestMassTable
```

Table 87: *Results of a non-linear hypothesis tests comparing predicted metabolic slopes in the heat (30°C - 40°C) among eight week old Japanese quail of average or atypically large body size (mean + 2 standard deviations), while assuming that appendage lengths scale allometrically . Posterior probabilities are calculated using the Savage-Dickey density ratio method.*

Hypothesis	Difference Between Metabolic Slopes	Standard Error	2.5 % CI	97.5 % CI	Posterior Probability
Large Metabolic Slope > Average Slope	0.0151	0.0534	-0.0699	0.0975	0.63625

We now scale model coefficients to represent the effects of changing a predictor by one standard deviation (or categorical level) on standard deviations in the response variable. After, we use these transformed coefficients to visualise direct, indirect, and total effect of individual predictors on metabolic slopes in the heat among mature quail.

```
scaledBetasHot3 <- as.data.frame(functionModel3WeeksHot) %>%
  mutate(
    b_mass_pretreatmentA = b_mass_pretreatmentA /
      sd(functionModel3WeeksHot$data$mass),
    b_mass_pretreatmentC = b_mass_pretreatmentC /
      sd(functionModel3WeeksHot$data$mass),
    b_tarsus_pretreatmentA = b_tarsus_pretreatmentA /
      sd(functionModel3WeeksHot$data$tarsus),
    b_tarsus_pretreatmentC = b_tarsus_pretreatmentC /
      sd(functionModel3WeeksHot$data$tarsus),
    b_tarsus_mass =
      (b_tarsus_mass * sd(functionModel3WeeksHot$data$mass)) /
        sd(functionModel3WeeksHot$data$tarsus),
    b_bill_pretreatmentA = b_bill_pretreatmentA /
      sd(functionModel3WeeksHot$data$bill),
    b_bill_pretreatmentC = b_tarsus_pretreatmentC /
      sd(functionModel3WeeksHot$data$bill),
    b_bill_mass =
      (b_bill_mass * sd(functionModel3WeeksHot$data$mass)) /
        sd(functionModel3WeeksHot$data$bill),
    b_slope_mass =
      (b_slope_mass * sd(functionModel3WeeksHot$data$mass)) /
        sd(functionModel3WeeksHot$data$slope),
    b_slope_tarsus =
      (b_slope_tarsus * sd(functionModel3WeeksHot$data$tarsus)) /
        sd(functionModel3WeeksHot$data$slope),
    b_slope_bill =
      (b_slope_bill * sd(functionModel3WeeksHot$data$bill)) /
        sd(functionModel3WeeksHot$data$slope),
    b_slope_pretreatmentA = b_slope_pretreatmentA /
      sd(functionModel3WeeksHot$data$slope),
    b_slope_pretreatmentC = b_slope_pretreatmentC /
```

```
    sd(functionModel3WeeksHot$data$slope),
  )

fullEffectThreeWeeksHotBetas <- scaledBetasHot3 %>%
  mutate("Effects" = "Direct Effects") %>%
  mutate(
    "Body Mass" = b_slope_mass,
    "Tarsus Length" = b_slope_tarsus,
    "Bill Length" = b_slope_bill,
    "Cold Rearing\n(10°C)" = b_slope_pretreatmentA,
    "Warm Rearing\n(30°C)" = b_slope_pretreatmentC
  ) %>%
  dplyr::select(
    Effects, `Body Mass`, `Tarsus Length`, `Bill Length`,
    `Cold Rearing\n(10°C)`,
    `Warm Rearing\n(30°C)`
  ) %>%
  rbind(
    .,
    scaledBetasHot3 %>%
      mutate("Effects" = "Indirect Effects") %>%
      mutate(
        "Body Mass" = b_tarsus_mass *
          b_slope_tarsus +
          b_bill_mass *
            b_slope_bill,
        "Tarsus Length" = NA,
        "Bill Length" = NA,
        "Cold Rearing\n(10°C)" =
          b_mass_pretreatmentA * b_slope_mass +
            b_tarsus_pretreatmentA * b_slope_tarsus +
            b_tarsus_pretreatmentA * b_slope_bill +
            b_mass_pretreatmentA * b_tarsus_mass *
              b_slope_tarsus +
            b_mass_pretreatmentA * b_bill_mass *
              b_slope_bill,
        "Warm Rearing\n(30°C)" =
          b_mass_pretreatmentC * b_slope_mass +
            b_tarsus_pretreatmentC * b_slope_tarsus +
            b_tarsus_pretreatmentC * b_slope_bill +
            b_mass_pretreatmentC * b_tarsus_mass *
              b_slope_tarsus +
            b_mass_pretreatmentC * b_bill_mass *
              b_slope_bill
      ) %>%
      dplyr::select(
        Effects, `Body Mass`, `Tarsus Length`, `Bill Length`,
        `Cold Rearing\n(10°C)`, `Warm Rearing\n(30°C)`
      )
  ) %>%
  rbind(., scaledBetasHot3 %>%
    mutate("Effects" = "Total Effects") %>%
    mutate(
      "Body Mass" =
        b_slope_mass +
          b_tarsus_mass * b_slope_tarsus +
          b_bill_mass * b_slope_bill,
      "Tarsus Length" =
        b_slope_tarsus,
      "Bill Length" =
        b_slope_bill,
      "Cold Rearing\n(10°C)" =
        b_slope_pretreatmentA +
          b_mass_pretreatmentA *
            b_slope_mass +
          b_tarsus_pretreatmentA *
            b_slope_tarsus +
```

```
          b_bill_pretreatmentA *
            b_slope_bill +
          b_mass_pretreatmentA *
            b_tarsus_mass * b_slope_tarsus +
          b_mass_pretreatmentA *
            b_bill_mass * b_slope_bill,
      "Warm Rearing\n(30°C)" =
        b_slope_pretreatmentC +
          b_mass_pretreatmentC *
            b_slope_mass +
          b_tarsus_pretreatmentC *
            b_slope_tarsus +
          b_bill_pretreatmentC *
            b_slope_bill +
          b_mass_pretreatmentC *
            b_tarsus_mass * b_slope_tarsus +
          b_mass_pretreatmentC *
            b_bill_mass * b_slope_bill,
    ) %>%
    dplyr::select(
      Effects, `Body Mass`, `Tarsus Length`, `Bill Length`,
      `Cold Rearing\n(10°C)`, `Warm Rearing\n(30°C)`
    )) %>%
  pivot_longer(c(-Effects), names_to = "var", values_to = "values") %>%
  mutate(var = factor(var,
    levels = c(
      "Body Mass",
      "Tarsus Length",
      "Bill Length",
      "Warm Rearing\n(30°C)",
      "Cold Rearing\n(10°C)"
    )
  ))


fullEffectThreeWeeksHot <- fullEffectThreeWeeksHotBetas %>%
  ggplot(aes(x = values, y = var, fill = var)) +
  facet_wrap(~Effects) +
  stat_halfeye(normalize = "xy", colour = "black", alpha = 0.7) +
  geom_vline(xintercept = 0, linetype = "dashed",
             colour = "black") +
  xlab(".                   Effect on Metabolic Slope (standard deviations)") +
  scale_fill_manual(values = c("black", "grey20","grey80","#CD5C5C", "#7BB4E3")) +
  theme_classic() +
  theme(
    legend.position = "none", axis.title.y = element_blank(),
    axis.text.y = element_text(size = 11, colour = "black",
                               family = "Noto Sans"),
    axis.title.x = element_text(family = "Noto Sans", hjust = -0.005),
    axis.text.x = element_text(family = "Noto Sans")
  )

showtext_auto(enable = TRUE)

ggsave("./plots/indirectEffectsOnResistance3WeeksHot_3ScaledFold.pdf",
       fullEffectThreeWeeksHot,
  dpi = 800, width = 8, height = 6
)
showtext_auto(enable = FALSE)

# Summarising results

caption <- paste0(
  "Direct, indirect, and total effects of morphology and ",
  "rearing temperature on metabolic slope ",
  "(fold metabolism at thermoneutrality/°C) ",
  "of three week old Japanese quail. Metabolic slopes ",
```

```
  "represent those observed above thermoneutrality ",
  "(30°C - 40°C). Effects are derived from ",
  "a Bayesian path analysis and represent those predicted for a ",
  "change in one standard deviation (or categorical level) of ",
  "a given predictor on the standard deviation of metabolic ",
  "slopes. Estimates indicate posterior medians and credible ",
  "intervels (CIs) indicate quantile intervals."
)

week3ResultsScaledHot <-
  fullEffectThreeWeeksHotBetas %>%
  filter(!is.na(values) & !is.nan(values)) %>%
  group_by(Effects, var) %>%
  summarise(
    "Estimate" = median(values),
    "50\\% CIs" = paste0(
      "[",
      round(
        quantile(values, probs = 0.1, type = 8),
        digits = 4
      ),
      ", ",
      round(
        quantile(values, probs = 0.9, type = 8),
        digits = 4
      ),
      "]"
    ),
    "95\\% CIs" = paste0(
      "[",
      round(
        quantile(values, probs = 0.025, type = 8),
        digits = 4
      ),
      ", ",
      round(
        quantile(values, probs = 0.975, type = 8),
        digits = 4
      ),
      "]"
    )
  ) %>%
  mutate("Effects" = gsub("[[:space:]].*", "", Effects)) %>%
  dplyr::select(
    "Predictor" = "var", "Effect Level" = "Effects",
    Estimate, `50\\% CIs`, `95\\% CIs`
  ) %>%
  arrange(Predictor, `Effect Level`) %>%
  kbl(.,
    longtable = T, booktabs = T, format = "latex", escape = FALSE,
    caption = caption
  ) %>%
  column_spec(column = c(1:2), width = "2.2cm") %>%
  column_spec(column = c(3:10), width = "1.9cm") %>%
  kable_styling(latex_options = "striped")

week3ResultsScaledHot
```

Table 88: *Direct, indirect, and total effects of morphology and rearing temperature on metabolic slope (fold metabolism at thermoneutrality/°C) of three week old Japanese quail. Metabolic slopes represent those observed above thermoneutrality (30°C - 40°C). Effects are derived from a Bayesian path analysis and represent those predicted for a change in one standard deviation (or categorical level) of a given predictor on the standard deviation of metabolic slopes. Estimates indicate posterior medians and credible intervels (CIs) indicate quantile intervals.*

Predictor	Effect Level	Estimate	50% CIs	95% CIs
Body Mass	Direct	0.1420926	[0.0256, 0.2446]	[-0.0392, 0.2989]
Body Mass	Indirect	-0.0289988	[-0.0732, 0.0136]	[-0.1015, 0.039]
Body Mass	Total	0.1106714	[0.0098, 0.2057]	[-0.0451, 0.258]
Tarsus Length	Direct	-0.0588988	[-0.1544, 0.0499]	[-0.2059, 0.1137]
Tarsus Length	Total	-0.0588988	[-0.1544, 0.0499]	[-0.2059, 0.1137]
Bill Length	Direct	-0.1931932	[-0.3294, -0.0458]	[-0.4018, 0.0341]
Bill Length	Total	-0.1931932	[-0.3294, -0.0458]	[-0.4018, 0.0341]
Warm Rearing (30°C)	Direct	-0.3513767	[-0.5788, -0.1103]	[-0.6974, 0.0395]
Warm Rearing (30°C)	Indirect	-0.1295312	[-0.3046, -0.0093]	[-0.4255, 0.0431]
Warm Rearing (30°C)	Total	-0.5101345	[-0.7717, -0.248]	[-0.9255, -0.0952]
Cold Rearing (10°C)	Direct	-0.1530240	[-0.3662, 0.0609]	[-0.4973, 0.1748]
Cold Rearing (10°C)	Indirect	-0.0872770	[-0.2256, 0.0093]	[-0.3257, 0.0626]
Cold Rearing (10°C)	Total	-0.1415461	[-0.3707, 0.0861]	[-0.4976, 0.2103]

```
# Continuing for data from mature quail

scaledBetasHot8 <- as.data.frame(functionModel8WeeksHot) %>%
  mutate(
    b_mass_pretreatmentA = b_mass_pretreatmentA /
      sd(functionModel8WeeksHot$data$mass),
    b_mass_pretreatmentC = b_mass_pretreatmentC /
      sd(functionModel8WeeksHot$data$mass),
    b_tarsus_pretreatmentA = b_tarsus_pretreatmentA /
      sd(functionModel8WeeksHot$data$tarsus),
    b_tarsus_pretreatmentC = b_tarsus_pretreatmentC /
      sd(functionModel8WeeksHot$data$tarsus),
    b_tarsus_mass =
      (b_tarsus_mass * sd(functionModel8WeeksHot$data$mass)) /
        sd(functionModel8WeeksHot$data$tarsus),
    b_bill_pretreatmentA = b_bill_pretreatmentA /
      sd(functionModel8WeeksHot$data$bill),
    b_bill_pretreatmentC = b_tarsus_pretreatmentC /
      sd(functionModel8WeeksHot$data$bill),
    b_bill_mass =
      (b_bill_mass * sd(functionModel8WeeksHot$data$mass)) /
        sd(functionModel8WeeksHot$data$bill),
    b_slope_mass =
      (b_slope_mass * sd(functionModel8WeeksHot$data$mass)) /
        sd(functionModel8WeeksHot$data$slope),
    b_slope_tarsus =
      (b_slope_tarsus * sd(functionModel8WeeksHot$data$tarsus)) /
        sd(functionModel8WeeksHot$data$slope),
    b_slope_bill =
      (b_slope_bill * sd(functionModel8WeeksHot$data$bill)) /
        sd(functionModel8WeeksHot$data$slope),
    b_slope_pretreatmentA = b_slope_pretreatmentA /
      sd(functionModel8WeeksHot$data$slope),
    b_slope_pretreatmentC = b_slope_pretreatmentC /
      sd(functionModel8WeeksHot$data$slope),
  )
```

```
fullEffectEightWeeksHotBetas <- scaledBetasHot8 %>%
  mutate("Effects" = "Direct Effects") %>%
  mutate(
    "Body Mass" = b_slope_mass,
    "Tarsus Length" = b_slope_tarsus,
    "Bill Length" = b_slope_bill,
    "Cold Rearing\n(10°C)" = b_slope_pretreatmentA,
    "Warm Rearing\n(30°C)" = b_slope_pretreatmentC
  ) %>%
  dplyr::select(
    Effects, `Body Mass`, `Tarsus Length`, `Bill Length`,
    `Cold Rearing\n(10°C)`,
    `Warm Rearing\n(30°C)`
  ) %>%
  rbind(
    .,
    scaledBetasHot8 %>%
      mutate("Effects" = "Indirect Effects") %>%
      mutate(
        "Body Mass" = b_tarsus_mass *
          b_slope_tarsus +
          b_bill_mass *
            b_slope_bill,
        "Tarsus Length" = NA,
        "Bill Length" = NA,
        "Cold Rearing\n(10°C)" =
          b_mass_pretreatmentA * b_slope_mass +
            b_tarsus_pretreatmentA * b_slope_tarsus +
            b_tarsus_pretreatmentA * b_slope_bill +
            b_mass_pretreatmentA * b_tarsus_mass *
              b_slope_tarsus +
            b_mass_pretreatmentA * b_bill_mass *
              b_slope_bill,
        "Warm Rearing\n(30°C)" =
          b_mass_pretreatmentC * b_slope_mass +
            b_tarsus_pretreatmentC * b_slope_tarsus +
            b_tarsus_pretreatmentC * b_slope_bill +
            b_mass_pretreatmentC * b_tarsus_mass *
              b_slope_tarsus +
            b_mass_pretreatmentC * b_bill_mass *
              b_slope_bill
      ) %>%
      dplyr::select(
        Effects, `Body Mass`, `Tarsus Length`, `Bill Length`,
        `Cold Rearing\n(10°C)`, `Warm Rearing\n(30°C)`
      )
  ) %>%
  rbind(., scaledBetasHot8 %>%
    mutate("Effects" = "Total Effects") %>%
    mutate(
      "Body Mass" =
        b_slope_mass +
          b_tarsus_mass * b_slope_tarsus +
          b_bill_mass * b_slope_bill,
      "Tarsus Length" =
        b_slope_tarsus,
      "Bill Length" =
        b_slope_bill,
      "Cold Rearing\n(10°C)" =
        b_slope_pretreatmentA +
          b_mass_pretreatmentA *
            b_slope_mass +
          b_tarsus_pretreatmentA *
            b_slope_tarsus +
          b_bill_pretreatmentA *
            b_slope_bill +
          b_mass_pretreatmentA *
```

```
            b_tarsus_mass * b_slope_tarsus +
          b_mass_pretreatmentA *
            b_bill_mass * b_slope_bill,
      "Warm Rearing\n(30°C)" =
        b_slope_pretreatmentC +
          b_mass_pretreatmentC *
            b_slope_mass +
          b_tarsus_pretreatmentC *
            b_slope_tarsus +
          b_bill_pretreatmentC *
            b_slope_bill +
          b_mass_pretreatmentC *
            b_tarsus_mass * b_slope_tarsus +
          b_mass_pretreatmentC *
            b_bill_mass * b_slope_bill,
    ) %>%
    dplyr::select(
      Effects, `Body Mass`, `Tarsus Length`, `Bill Length`,
      `Cold Rearing\n(10°C)`, `Warm Rearing\n(30°C)`
    )) %>%
  pivot_longer(c(-Effects), names_to = "var", values_to = "values") %>%
  mutate(var = factor(var,
    levels = c(
      "Body Mass",
      "Tarsus Length",
      "Bill Length",
      "Warm Rearing\n(30°C)",
      "Cold Rearing\n(10°C)"
    )
  ))

fullEffectEightWeeksHot <- fullEffectEightWeeksHotBetas %>%
  ggplot(aes(x = values, y = var, fill = var)) +
  facet_wrap(~Effects) +
  stat_halfeye(normalize = "xy", colour = "black", alpha = 0.7) +
  geom_vline(xintercept = 0, linetype = "dashed",
             colour = "black") +
  xlab(".                   Effect on Metabolic Slope (standard deviations)") +
  scale_fill_manual(values = c("black", "grey20","grey80","#CD5C5C", "#7BB4E3")) +
  theme_classic() +
  theme(
    legend.position = "none", axis.title.y = element_blank(),
    axis.text.y = element_text(size = 11, colour = "black",
                               family = "Noto Sans"),
    axis.title.x = element_text(family = "Noto Sans", hjust = -0.005),
    axis.text.x = element_text(family = "Noto Sans")
  )

showtext_auto(enable = TRUE)

ggsave("./plots/indirectEffectsOnResistance8WeeksHot_3ScaledFold.pdf",
       fullEffectEightWeeksHot,
  dpi = 800, width = 8, height = 6
)
showtext_auto(enable = FALSE)

# Summarising results

caption <- paste0(
  "Direct, indirect, and total effects of morphology and ",
  "rearing temperature on metabolic slope ",
  "(fold metabolism at thermoneutrality/°C) ",
  "of eight week old Japanese quail. Metabolic slopes ",
  "represent those observed above thermoneutrality ",
  "(30°C - 40°C). Effects are derived from ",
  "a Bayesian path analysis and represent those predicted for a ",
  "change in one standard deviation (or categorical level) of ",
```

```
  "a given predictor on the standard deviation of metabolic ",
  "slopes. Estimates indicate posterior medians and credible ",
  "intervels (CIs) indicate quantile intervals."
)

week8ResultsScaledHot <-
  fullEffectEightWeeksHotBetas %>%
  filter(!is.na(values) & !is.nan(values)) %>%
  group_by(Effects, var) %>%
  summarise(
    "Estimate" = median(values),
    "50\\% CIs" = paste0(
      "[",
      round(
        quantile(values, probs = 0.1, type = 8),
        digits = 4
      ),
      ", ",
      round(
        quantile(values, probs = 0.9, type = 8),
        digits = 4
      ),
      "]"
    ),
    "95\\% CIs" = paste0(
      "[",
      round(
        quantile(values, probs = 0.025, type = 8),
        digits = 4
      ),
      ", ",
      round(
        quantile(values, probs = 0.975, type = 8),
        digits = 4
      ),
      "]"
    )
  ) %>%
  mutate("Effects" = gsub("[[:space:]].*", "", Effects)) %>%
  dplyr::select(
    "Predictor" = "var", "Effect Level" = "Effects",
    Estimate, `50\\% CIs`, `95\\% CIs`
  ) %>%
  arrange(Predictor, `Effect Level`) %>%
  kbl(.,
    longtable = T, booktabs = T, format = "latex", escape = FALSE,
    caption = caption
  ) %>%
  column_spec(column = c(1:2), width = "2.2cm") %>%
  column_spec(column = c(3:10), width = "1.9cm") %>%
  kable_styling(latex_options = "striped")

week8ResultsScaledHot
```

Table 89: *Direct, indirect, and total effects of morphology and rearing temperature on metabolic slope (fold metabolism at thermoneutrality/°C) of eight week old Japanese quail. Metabolic slopes represent those observed above thermoneutrality (30°C - 40°C). Effects are derived from a Bayesian path analysis and represent those predicted for a change in one standard deviation (or categorical level) of a given predictor on the standard deviation of metabolic slopes. Estimates indicate posterior medians and credible intervels (CIs) indicate quantile intervals.*

Predictor	Effect Level	Estimate	50% CIs	95% CIs
Body Mass	Direct	0.2012866	[0.085, 0.3254]	[0.0209, 0.3889]
Body Mass	Indirect	-0.0415416	[-0.0994, 0.0075]	[-0.1364, 0.0353]

Body Mass	Total	0.1566445	[0.05, 0.2732]	[-0.0075, 0.3312]
Tarsus Length	Direct	-0.1203981	[-0.2496, 0.0082]	[-0.3219, 0.0708]
Tarsus Length	Total	-0.1203981	[-0.2496, 0.0082]	[-0.3219, 0.0708]
Bill Length	Direct	-0.0799589	[-0.1947, 0.0384]	[-0.2505, 0.1029]
Bill Length	Total	-0.0799589	[-0.1947, 0.0384]	[-0.2505, 0.1029]
Warm Rearing (30°C)	Direct	0.1201766	[-0.1521, 0.3763]	[-0.2979, 0.5084]
Warm Rearing (30°C)	Indirect	-0.0050046	[-0.1221, 0.1009]	[-0.2062, 0.168]
Warm Rearing (30°C)	Total	0.0979275	[-0.1931, 0.3794]	[-0.3505, 0.522]
Cold Rearing (10°C)	Direct	-0.0197938	[-0.2796, 0.2603]	[-0.4126, 0.3995]
Cold Rearing (10°C)	Indirect	-0.0180657	[-0.1264, 0.075]	[-0.2013, 0.1389]
Cold Rearing (10°C)	Total	0.0041217	[-0.2586, 0.2844]	[-0.3997, 0.4314]

Again, the above path analyses are repeated but while relativising tarsus length and bill length measurements by body mass. This is achieved by calculating length residuals from simple regressions of tarsus length or bill length by body mass (tarsus length priors for models with three week data: intercept = $\mathcal{N}[30, 2.5]$, body mass = $S\mathcal{N}[0, 0.25, 5]$; models with eight week data: intercept = $\mathcal{N}[37.5, 2.5]$, body mass = $S\mathcal{N}[0, 0.25, 5]$; bill length priors for models with three week data: intercept = $\mathcal{N}[9, 1]$, body mass = $S\mathcal{N}[0, 0.25, 5]$; priors for models with eight week data: intercept = $\mathcal{N}[10, 1]$, body mass = $S\mathcal{N}[0, 0.25, 5]$), then loading these residuals into the full analysis. Priors for these path analyses remain the same as those used previously with response variables unscaled.

Given that these adjusted path analyses are conducted merely for comparative purposes (i.e. for comparison with models analyses where tarsus length is not relativised), residuals are not rigorously scrutinised, nor are model coefficients plotted in detail. Instead, final model outcomes are printed after checking for prior suitability and chain convergence.

```
resModData3 <- filter(slopeDataHot3, week == "3") %>%
    mutate(
      massCentred = mass - mean(mass, na.rm = T),
      pretreatment = ifelse(pretreatment == "cold", "A",
        ifelse(pretreatment == "neutral", "B", "C")
      )
    ) %>%
    mutate(pretreatment = factor(pretreatment, levels = c("B", "A", "C"))) %>%
    dplyr::select(-mass) %>%
    rename("mass" = massCentred) %>%
    drop_na() %>%
    merge(., data %>%
      dplyr::select(ring, "batch" = exp) %>%
      distinct(),
    by = "ring", all.x = TRUE
    )

residTarsusModel3 <- brm(
  bf(tarsusLengthMean ~ mass),
  data = resModData3,
  prior = c(
    set_prior("skew_normal(0, 0.25, 5)",
      class = "b"
    ),
    set_prior("normal(30, 2.5)",
```

```
      class = "Intercept"
    )
  ),
  family = "gaussian",
  iter = 50000, warmup = 10000, cores = 1,
  chains = 4, thin = 20,
  silent = TRUE, refresh = 0,
  file = "./models/_tarsusResidualsModel3WeeksHot.Rds"
)

residBillModel3 <- brm(
  bf(billLengthMean ~ mass),
  data = resModData3,
  prior = c(
    set_prior("skew_normal(0, 0.25, 5)",
      class = "b"
    ),
    set_prior("normal(9, 1)",
      class = "Intercept"
    )
  ),
  family = "gaussian",
  iter = 50000, warmup = 10000, cores = 1,
  chains = 4, thin = 20,
  silent = TRUE, refresh = 0,
  file = "./models/_billResidualsModel3WeeksHot.Rds"
)

functionModel3WeeksResidualPPCheck <- brm(
  data = resModData3 %>%
    mutate(
      "residualTarsus" =
        residuals(residTarsusModel3)[,"Estimate"],
      "residualBill" =
        residuals(residBillModel3)[,"Estimate"]
    ),
  family = "gaussian",
  bf(mass ~ pretreatment + (1 | batch), family = "gaussian") +
    bf(residualTarsus ~ pretreatment + (1 | batch), family = "gaussian") +
    bf(residualBill ~ pretreatment + (1 | batch), family = "gaussian") +
    bf(slope ~ mass + residualTarsus + residualBill + pretreatment +
        (1 | batch), sigma ~ batch, family = "student") +
    set_rescor(rescor = FALSE),
    prior = c(
      set_prior("normal(0, 5)",
        class = "Intercept",
        resp = "mass"
      ),
      set_prior("normal(0, 15)",
        class = "b",
        coef = "pretreatmentA",
        resp = "mass"
      ),
      set_prior("normal(0, 15)",
        class = "b",
        coef = "pretreatmentC",
        resp = "mass"
      ),
      set_prior("exponential(1.5)",
        class = "sd",
        group = "batch",
        resp = "mass"
      ),
      set_prior("exponential(0.15)",
        class = "sigma",
        resp = "mass"
      ),
```

```
    set_prior("normal(0, 2.5)",
      class = "Intercept",
      resp = "residualTarsus"
    ),
    set_prior("normal(0, 2.5)",
      class = "b",
      coef = "pretreatmentA",
      resp = "residualTarsus"
    ),
    set_prior("normal(0, 2.5)",
      class = "b",
      coef = "pretreatmentC",
      resp = "residualTarsus"
    ),
    set_prior("exponential(2)",
      class = "sd",
      group = "batch",
      resp = "residualTarsus"
    ),
    set_prior("exponential(1)",
      class = "sigma",
      resp = "residualTarsus"
    ),
    set_prior("normal(0, 1)",
      class = "Intercept",
      resp = "residualBill"
    ),
    set_prior("normal(0, 0.5)",
      class = "b",
      coef = "pretreatmentA",
      resp = "residualBill"
    ),
    set_prior("normal(0, 0.5)",
      class = "b",
      coef = "pretreatmentC",
      resp = "residualBill"
    ),
    set_prior("exponential(5)",
      class = "sd",
      group = "batch",
      resp = "residualBill"
    ),
    set_prior("exponential(2.5)",
      class = "sigma",
      resp = "residualBill"
    ),
    set_prior("normal(0, 0.125)",
      class = "Intercept",
      resp = "slope"
    ),
    set_prior("normal(0, 0.125)",
      class = "b",
      coef = "pretreatmentA",
      resp = "slope"
    ),
    set_prior("normal(0, 0.125)",
      class = "b",
      coef = "pretreatmentC",
      resp = "slope"
    ),
    set_prior("normal(0, 0.004)",
      class = "b",
      coef = "mass",
      resp = "slope"
    ),
    set_prior("normal(0, 0.015)",
      class = "b",
```

```
        coef = "residualTarsus",
        resp = "slope"
      ),
      set_prior("normal(0, 0.06)",
        class = "b",
        coef = "residualBill",
        resp = "slope"
      ),
      set_prior("exponential(50)",
        class = "sd",
        group = "batch",
        resp = "slope"
      ),
      set_prior("normal(-3, 1.5)",
        dpar = "sigma",
        class = "Intercept",
        resp = "slope"
      ),
      set_prior("normal(0, 0.5)",
        dpar = "sigma",
        class = "b",
        coef = "batchB",
        resp = "slope"
      ),
      set_prior("normal(1, 1.5)",
        dpar = "sigma",
        class = "b",
        coef = "batchC",
        resp = "slope"
      ),
      set_prior("gamma(5, 1)",
        class = "nu",
        resp = "slope"
      )
    ),
  iter = 50000, warmup = 10000, cores = 4, chains = 4, thin = 20,
  control = list(adapt_delta = .96),
  silent = TRUE, refresh = 0,
  sample_prior = "only",
  file = "./models/_threeWeekFunctionModelResidualHotPPCheck.Rds"
)

resModData8 <- filter(slopeDataHot8, week == "8") %>%
    mutate(
      massCentred = mass - mean(mass, na.rm = T),
      pretreatment = ifelse(pretreatment == "cold", "A",
        ifelse(pretreatment == "neutral", "B", "C")
      )
    ) %>%
    mutate(pretreatment = factor(pretreatment, levels = c("B", "A", "C"))) %>%
    dplyr::select(-mass) %>%
    rename("mass" = massCentred) %>%
    drop_na() %>%
    merge(., data %>%
      dplyr::select(ring, "batch" = exp) %>%
      distinct(),
    by = "ring", all.x = TRUE
    )

residTarsusModel8 <- brm(
  bf(tarsusLengthMean ~ mass),
  data = resModData8,
  prior = c(
    set_prior("skew_normal(0, 0.25, 5)",
      class = "b"
    ),
    set_prior("normal(37.5, 2.5)",
```

```
      class = "Intercept"
    )
  ),
  family = "gaussian",
  iter = 50000, warmup = 10000, cores = 1,
  chains = 4, thin = 20,
  silent = TRUE, refresh = 0,
  file = "./models/_tarsusResidualsModel8WeeksHot.Rds"
)

residBillModel8 <- brm(
  bf(billLengthMean ~ mass),
  data = resModData8,
  prior = c(
    set_prior("skew_normal(0, 0.25, 5)",
      class = "b"
    ),
    set_prior("normal(10, 1)",
      class = "Intercept"
    )
  ),
  family = "gaussian",
  iter = 50000, warmup = 10000, cores = 1,
  chains = 4, thin = 20,
  silent = TRUE, refresh = 0,
  file = "./models/_billResidualsModel8WeeksHot.Rds"
)

functionModel8WeeksResidualPPCheck <- brm(
  data = resModData8 %>%
    mutate(
      "residualTarsus" =
        residuals(residTarsusModel8)[,"Estimate"],
      "residualBill" =
        residuals(residBillModel8)[,"Estimate"]
    ),
  family = "gaussian",
  bf(mass ~ pretreatment + (1 | batch), family = "gaussian") +
    bf(residualTarsus ~ pretreatment + (1 | batch), family = "gaussian") +
    bf(residualBill ~ pretreatment + (1 | batch), family = "gaussian") +
    bf(slope ~ mass + residualTarsus + residualBill + pretreatment +
         (1 | batch), family = "student") +
    set_rescor(rescor = FALSE),
    prior = c(
      set_prior("normal(0, 10)",
        class = "Intercept",
        resp = "mass"
      ),
      set_prior("normal(0, 25)",
        class = "b",
        coef = "pretreatmentA",
        resp = "mass"
      ),
      set_prior("normal(0, 25)",
        class = "b",
        coef = "pretreatmentC",
        resp = "mass"
      ),
      set_prior("exponential(1.5)",
        class = "sd",
        group = "batch",
        resp = "mass"
      ),
      set_prior("exponential(0.05)",
        class = "sigma",
        resp = "mass"
      ),
```

```
    set_prior("normal(0, 3)",
      class = "Intercept",
      resp = "residualTarsus"
    ),
    set_prior("normal(0, 3)",
      class = "b",
      coef = "pretreatmentA",
      resp = "residualTarsus"
    ),
    set_prior("normal(0, 3)",
      class = "b",
      coef = "pretreatmentC",
      resp = "residualTarsus"
    ),
    set_prior("exponential(2)",
      class = "sd",
      group = "batch",
      resp = "residualTarsus"
    ),
    set_prior("exponential(0.75)",
      class = "sigma",
      resp = "residualTarsus"
    ),
    set_prior("normal(0, 1)",
      class = "Intercept",
      resp = "residualBill"
    ),
    set_prior("normal(0, 0.5)",
      class = "b",
      coef = "pretreatmentA",
      resp = "residualBill"
    ),
    set_prior("normal(0, 0.5)",
      class = "b",
      coef = "pretreatmentC",
      resp = "residualBill"
    ),
    set_prior("exponential(5)",
      class = "sd",
      group = "batch",
      resp = "residualBill"
    ),
    set_prior("exponential(2.5)",
      class = "sigma",
      resp = "residualBill"
    ),
    set_prior("normal(0, 0.1)",
      class = "Intercept",
      resp = "slope"
    ),
    set_prior("normal(0, 0.1)",
      class = "b",
      coef = "pretreatmentA",
      resp = "slope"
    ),
    set_prior("normal(0, 0.1)",
      class = "b",
      coef = "pretreatmentC",
      resp = "slope"
    ),
    set_prior("normal(0, 0.0025)",
      class = "b",
      coef = "mass",
      resp = "slope"
    ),
    set_prior("normal(0, 0.03)",
      class = "b",
```

```
        coef = "residualTarsus",
        resp = "slope"
      ),
      set_prior("normal(0, 0.1)",
        class = "b",
        coef = "residualBill",
        resp = "slope"
      ),
      set_prior("exponential(50)",
        class = "sd",
        group = "batch",
        resp = "slope"
      ),
      set_prior("exponential(10)",
        class = "sigma",
        resp = "slope"
      ),
      set_prior("gamma(10, 1)",
                class = "nu",
                resp = "slope"
      )
    ),
  iter = 50000, warmup = 10000, cores = 4, chains = 4, thin = 20,
  control = list(adapt_delta = .96),
  silent = TRUE, refresh = 0,
  sample_prior = "only",
  file = "./models/_eightWeekFunctionModelResidualHotPPCheck.Rds"
)

p1 <- pp_check2(functionModel3WeeksResidualPPCheck,
  resp = "slope",
  xlab = paste0("Metabolic Slope (Fold Metabolism\n",
                "at Thermoneutrality/°C)")
)

p2 <- pp_check2(functionModel8WeeksResidualPPCheck,
  resp = "slope",
  xlab = paste0("Metabolic Slope (Fold Metabolism\n",
                "at Thermoneutrality/°C)")
  )

(p1 + p2) + plot_annotation(tag_levels = "A")
```

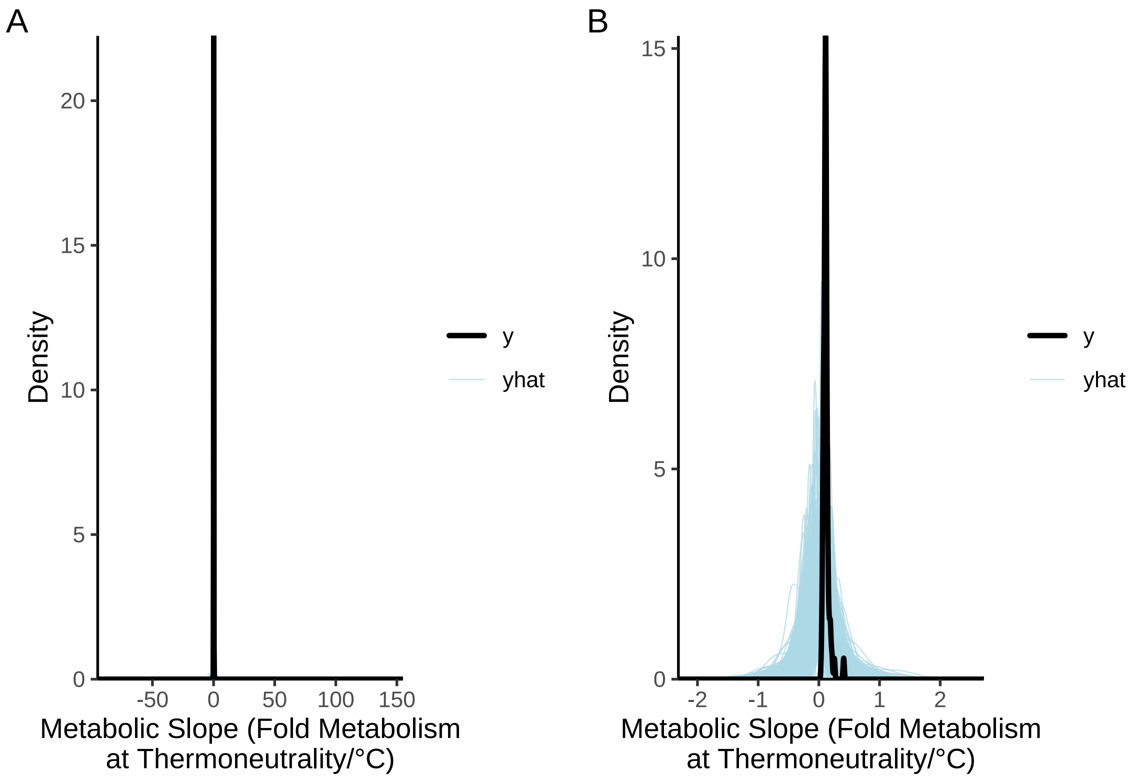

Figure 141: *Prior predictive checks for two Bayesian path analyses predicting physiological responses to heat in three and eight week old Japanese quail. Tarsus length and bill length in these models are relativised by body mass (i.e. as ordinary residuals from regressions against body mass). Black lines represent true densities of metabolic slopes (fold metabolism at thermoneutrality/°C) while blue lines represent densities estimated from model priors alone.*

```
# Priors are wide but capture true distributions nonetheless
```

```
functionModel3WeeksResidualHot <- brm(
  data = resModData3 %>%
    mutate(
      "residualTarsus" =
        residuals(residTarsusModel3)[,"Estimate"],
      "residualBill" =
        residuals(residBillModel3)[,"Estimate"]
    ),
  family = "gaussian",
  bf(mass ~ pretreatment + (1 | batch), family = "gaussian") +
    bf(residualTarsus ~ pretreatment + (1 | batch), family = "gaussian") +
    bf(residualBill ~ pretreatment + (1 | batch), family = "gaussian") +
    bf(slope ~ mass + residualTarsus + residualBill + pretreatment +
         (1 | batch), sigma ~ batch, family = "student") +
    set_rescor(rescor = FALSE),
    prior = c(
      set_prior("normal(0, 5)",
        class = "Intercept",
        resp = "mass"
      ),
      set_prior("normal(0, 15)",
        class = "b",
        coef = "pretreatmentA",
        resp = "mass"
      ),
      set_prior("normal(0, 15)",
        class = "b",
```

```
      coef = "pretreatmentC",
      resp = "mass"
    ),
    set_prior("exponential(1.5)",
      class = "sd",
      group = "batch",
      resp = "mass"
    ),
    set_prior("exponential(0.15)",
      class = "sigma",
      resp = "mass"
    ),
    set_prior("normal(0, 2.5)",
      class = "Intercept",
      resp = "residualTarsus"
    ),
    set_prior("normal(0, 2.5)",
      class = "b",
      coef = "pretreatmentA",
      resp = "residualTarsus"
    ),
    set_prior("normal(0, 2.5)",
      class = "b",
      coef = "pretreatmentC",
      resp = "residualTarsus"
    ),
    set_prior("exponential(2)",
      class = "sd",
      group = "batch",
      resp = "residualTarsus"
    ),
    set_prior("exponential(1)",
      class = "sigma",
      resp = "residualTarsus"
    ),
    set_prior("normal(0, 1)",
      class = "Intercept",
      resp = "residualBill"
    ),
    set_prior("normal(0, 0.5)",
      class = "b",
      coef = "pretreatmentA",
      resp = "residualBill"
    ),
    set_prior("normal(0, 0.5)",
      class = "b",
      coef = "pretreatmentC",
      resp = "residualBill"
    ),
    set_prior("exponential(5)",
      class = "sd",
      group = "batch",
      resp = "residualBill"
    ),
    set_prior("exponential(2.5)",
      class = "sigma",
      resp = "residualBill"
    ),
    set_prior("normal(0, 0.125)",
      class = "Intercept",
      resp = "slope"
    ),
    set_prior("normal(0, 0.125)",
      class = "b",
      coef = "pretreatmentA",
      resp = "slope"
    ),
```

```
      set_prior("normal(0, 0.125)",
        class = "b",
        coef = "pretreatmentC",
        resp = "slope"
      ),
      set_prior("normal(0, 0.004)",
        class = "b",
        coef = "mass",
        resp = "slope"
      ),
      set_prior("normal(0, 0.015)",
        class = "b",
        coef = "residualTarsus",
        resp = "slope"
      ),
      set_prior("normal(0, 0.06)",
        class = "b",
        coef = "residualBill",
        resp = "slope"
      ),
      set_prior("exponential(50)",
        class = "sd",
        group = "batch",
        resp = "slope"
      ),
      set_prior("normal(-3, 1.5)",
        dpar = "sigma",
        class = "Intercept",
        resp = "slope"
      ),
      set_prior("normal(0, 0.5)",
        dpar = "sigma",
        class = "b",
        coef = "batchB",
        resp = "slope"
      ),
      set_prior("normal(1, 1.5)",
        dpar = "sigma",
        class = "b",
        coef = "batchC",
        resp = "slope"
      ),
      set_prior("gamma(5, 1)",
        class = "nu",
        resp = "slope"
      )
    ),
  iter = 50000, warmup = 10000, cores = 4, chains = 4, thin = 20,
  control = list(adapt_delta = .98),
  silent = TRUE, refresh = 0,
  file = "./models/_threeWeekFunctionModelResidualHot.Rds"
)

functionModel8WeeksResidualHot<- brm(
  data = resModData8 %>%
    mutate(
      "residualTarsus" =
        residuals(residTarsusModel8)[,"Estimate"],
      "residualBill" =
        residuals(residBillModel8)[,"Estimate"]
    ),
  family = "gaussian",
  bf(mass ~ pretreatment + (1 | batch), family = "gaussian") +
    bf(residualTarsus ~ pretreatment + (1 | batch), family = "gaussian") +
    bf(residualBill ~ pretreatment + (1 | batch), family = "gaussian") +
    bf(slope ~ mass + residualTarsus + residualBill + pretreatment +
         (1 | batch), family = "student") +
```

```
    set_rescor(rescor = FALSE),
    prior = c(
      set_prior("normal(0, 10)",
        class = "Intercept",
        resp = "mass"
      ),
      set_prior("normal(0, 25)",
        class = "b",
        coef = "pretreatmentA",
        resp = "mass"
      ),
      set_prior("normal(0, 25)",
        class = "b",
        coef = "pretreatmentC",
        resp = "mass"
      ),
      set_prior("exponential(1.5)",
        class = "sd",
        group = "batch",
        resp = "mass"
      ),
      set_prior("exponential(0.05)",
        class = "sigma",
        resp = "mass"
      ),
      set_prior("normal(0, 3)",
        class = "Intercept",
        resp = "residualTarsus"
      ),
      set_prior("normal(0, 3)",
        class = "b",
        coef = "pretreatmentA",
        resp = "residualTarsus"
      ),
      set_prior("normal(0, 3)",
        class = "b",
        coef = "pretreatmentC",
        resp = "residualTarsus"
      ),
      set_prior("exponential(2)",
        class = "sd",
        group = "batch",
        resp = "residualTarsus"
      ),
      set_prior("exponential(0.75)",
        class = "sigma",
        resp = "residualTarsus"
      ),
      set_prior("normal(0, 1)",
        class = "Intercept",
        resp = "residualBill"
      ),
      set_prior("normal(0, 0.5)",
        class = "b",
        coef = "pretreatmentA",
        resp = "residualBill"
      ),
      set_prior("normal(0, 0.5)",
        class = "b",
        coef = "pretreatmentC",
        resp = "residualBill"
      ),
      set_prior("exponential(5)",
        class = "sd",
        group = "batch",
        resp = "residualBill"
      ),
```

```
      set_prior("exponential(2.5)",
        class = "sigma",
        resp = "residualBill"
      ),
      set_prior("normal(0, 0.1)",
        class = "Intercept",
        resp = "slope"
      ),
      set_prior("normal(0, 0.1)",
        class = "b",
        coef = "pretreatmentA",
        resp = "slope"
      ),
      set_prior("normal(0, 0.1)",
        class = "b",
        coef = "pretreatmentC",
        resp = "slope"
      ),
      set_prior("normal(0, 0.0025)",
        class = "b",
        coef = "mass",
        resp = "slope"
      ),
      set_prior("normal(0, 0.03)",
        class = "b",
        coef = "residualTarsus",
        resp = "slope"
      ),
      set_prior("normal(0, 0.1)",
        class = "b",
        coef = "residualBill",
        resp = "slope"
      ),
      set_prior("exponential(50)",
        class = "sd",
        group = "batch",
        resp = "slope"
      ),
      set_prior("exponential(10)",
        class = "sigma",
        resp = "slope"
      ),
      set_prior("gamma(10, 1)",
                class = "nu",
                resp = "slope"
      )
    ),
  iter = 50000, warmup = 10000, cores = 4, chains = 4, thin = 20,
  control = list(adapt_delta = .98),
  silent = TRUE, refresh = 0,
  file = "./models/_eightWeekFunctionModelResidualHot.Rds"
)

# Minimal divergent transitions in both models.

p1 <- mcmc_neff(neff_ratio(functionModel3WeeksResidualHot)) +
  xlab(
    TeX("$Three~Week~N_{eff}/N$")
  ) +
  theme_classic() +
  theme(
    axis.text.y = element_blank(),
    axis.ticks.y = element_blank(),
    legend.position = "none"
  )

p2 <- mcmc_rhat(rhat(functionModel3WeeksResidualHot)) +
```

```
  xlab(
    TeX("$\\hat{R}~(Three~Weeks)$")
  ) +
  theme_classic() +
  theme(
    axis.text.y = element_blank(),
    axis.ticks.y = element_blank(),
    legend.position = "none"
  )

p3 <- functionModel3WeeksResidualHot$data %>%
  mutate(
    "Residuals" =
      residuals(functionModel3WeeksResidualHot,
        type = "ordinary",
        resp = "slope",
        robust = TRUE
      )[, "Estimate"]
  ) %>%
  ggplot(aes(x = Residuals)) +
  geom_density() +
  xlab("Three Week Metabolic\nSlope Residuals") +
  ylab("Density") +
  theme_classic()

p4 <- mcmc_neff(neff_ratio(functionModel8WeeksResidualHot)) +
  xlab(
    TeX("$Eight~Week~N_{eff}/N$")
  ) +
  theme_classic() +
  theme(
    axis.text.y = element_blank(),
    axis.ticks.y = element_blank(),
    legend.position = "none"
  )

p5 <- mcmc_rhat(rhat(functionModel8WeeksResidualHot)) +
  xlab(
    TeX("$\\hat{R}~(Eight~Weeks)$")
  ) +
  theme_classic() +
  theme(
    axis.text.y = element_blank(),
    axis.ticks.y = element_blank(),
    legend.position = "none"
  )

p6 <- functionModel8WeeksResidualHot$data %>%
  mutate(
    "Residuals" =
      residuals(functionModel8WeeksResidualHot,
        type = "ordinary",
        resp = "slope",
        robust = TRUE
      )[, "Estimate"]
  ) %>%
  ggplot(aes(x = Residuals)) +
  geom_density() +
  xlab("Eight Week Metabolic\nSlope Residuals") +
  ylab("Density") +
  theme_classic()

(p1 + p2 + p3) / (p4 + p5 + p6) +
  plot_annotation(tag_levels = "A")
```

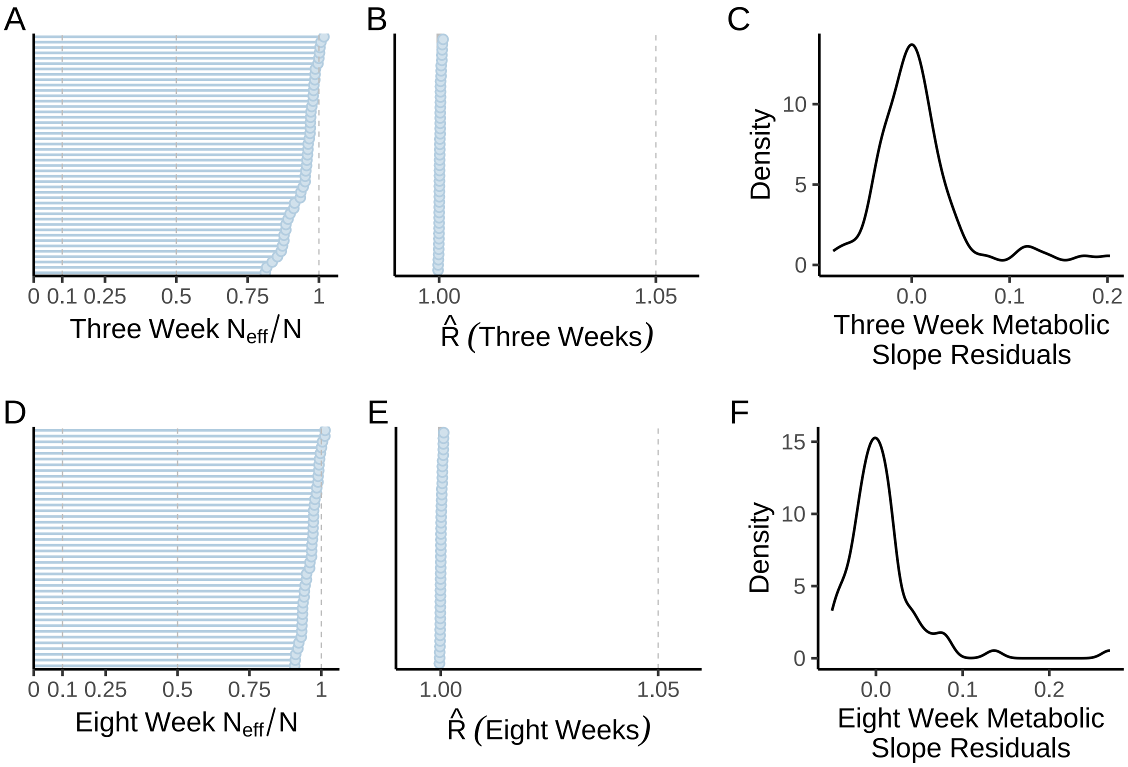

Figure 142: *Gelman-Rubin ($\hat{R}$) statistics, ratio of effective sample sizes to sample sizes (N_{eff}/N), and residual densities from Bayesian path analyses predicting physiological responses to heat in three and eight week old Japanese quail. Tarsus length and bill length in these models are relativised by body mass (i.e. as ordinary residuals from a regression against body mass). In panels A, B, D and E, blue dots indicate Rhat or Neff/N values for a model parameter.*

```
# Chains evidently converged, autocorrelation between
# draws appears limited, and residuals reasonably normal.
# Extracting model outcomes for comparison.

caption <- paste0(
  "Results from a Bayesian path analysis ",
  "predicting metabolic responses to heat (metabolic slopes ",
  "[fold metabolism at thermoneutrality/",
  "°C] as a function of body mass (g), relative tarsus length ",
  "(residuals; mm), relative bill length (residuals; mm) and ",
  "rearing conditions in three ",
  "week old Japanese quail. Physiological measurements ",
  "are made at ambient temperatures between ",
  "30°C and 40°C. Cold rearing indicates ",
  "post-hatch rearing at 10°C, relative to 20°C (intercept), ",
  "or 30°C ('warm rearing'). CI indicates quantile ",
  "intervals and BF indicates Bayes Factors."
)

week3ResultsHeatResiduals <-
  as.data.frame(functionModel3WeeksResidualHot) %>%
  summarise_all(., .funs = median) %>%
  pivot_longer(everything(),
    names_to = "Parameter",
    values_to = "Estimate"
  ) %>%
  merge(., quantileCIs(functionModel3WeeksResidualHot, cis = c(50, 95)),
    by = "Parameter", all.x = TRUE
  ) %>%
```

```
filter(grepl("b_|sd_", Parameter)) %>%
rowwise() %>%
mutate("BF" = ifelse(Estimate < 0,
  (2 * mean(as.data.frame(
    functionModel3WeeksResidualHot
  )[, Parameter] <= 0)) /
    (2 * mean(as.data.frame(
      functionModel3WeeksResidualHot
    )[, Parameter] >= 0)),
  (2 * mean(as.data.frame(
    functionModel3WeeksResidualHot
  )[, Parameter] >= 0)) /
    (2 * mean(as.data.frame(
      functionModel3WeeksResidualHot
    )[, Parameter] <= 0))
)) %>%
ungroup() %>%
mutate(
  "Estimate" = round(Estimate, digits = 4),
  "BF" = round(BF, digits = 4),
  "N" = nrow(functionModel3WeeksResidualHot$data)
) %>%
mutate("Parameter" = ifelse(grepl("b_", Parameter),
  gsub("b_", "", Parameter),
  gsub(
    "Intercept", "batch",
    gsub(".*__", "", Parameter)
  )
)) %>%
mutate(
  "Response" = gsub("_.*", "", Parameter),
  "Parameter" = gsub(".*_", "", Parameter)
) %>%
merge(., tribble(
  ~Response, ~response, ~level,
  "mass", "Body Mass (g)", "A",
  "residualTarsus", "Residual Tarsus Length (mm)", "B",
  "residualBill", "Residual Bill Length (mm)", "C",
  "slope",
  "Metabolic Slope", "D"
),
by = "Response"
) %>%
merge(., tribble(
  ~Parameter, ~parameter, ~number,
  "Intercept", "Intercept", "1",
  "mass", "Body Mass (g)", "4",
  "residualTarsus", "Residual Tarsus Length (mm)", "5",
  "residualBill", "Residual Bill Length (mm)", "6",
  "pretreatmentA", "Cold Rearing", "2",
  "pretreatmentC", "Warm Rearing", "3",
  "batch", "Egg Batch [mu]", "7"
),
by = "Parameter"
) %>%
mutate(
  `50\\% HDI` = paste0("(", paste(
    round(Low_CI_50, digits = 4),
    round(High_CI_50, digits = 4),
    sep = ", "
  ), ")"),
  `95\\% HDI` = paste0("(", paste(
    round(Low_CI_95, digits = 4),
    round(High_CI_95, digits = 4),
    sep = ", "
  ), ")")
) %>%
```

```
  dplyr::select(-c(Low_CI_50, High_CI_50, Low_CI_95, High_CI_95)) %>%
  dplyr::select(
    "Response" = "response", "Parameter" = "parameter", N,
    Estimate, `50\\% HDI`, `95\\% HDI`, BF, level, number
  ) %>%
  arrange(level, number) %>%
  dplyr::select(-c(level, number)) %>%
  kbl(.,
    longtable = T, booktabs = T, format = "latex", escape = FALSE,
    caption = caption
  ) %>%
  column_spec(column = c(1:2), width = "2.2cm") %>%
  column_spec(column = c(3:10), width = "1.9cm") %>%
  kable_styling(latex_options = "striped")

week3ResultsHeatResiduals
```

Table 90: *Results from a Bayesian path analysis predicting metabolic responses to heat (metabolic slopes [fold metabolism at thermoneutrality/°C] as a function of body mass (g), relative tarsus length (residuals; mm), relative bill length (residuals; mm) and rearing conditions in three week old Japanese quail. Physiological measurements are made at ambient temperatures between 30°C and 40°C. Cold rearing indicates post-hatch rearing at 10°C, relative to 20°C (intercept), or 30°C ('warm rearing'). CI indicates quantile intervals and BF indicates Bayes Factors.*

Response	Parameter	N	Estimate	50% HDI	95% HDI	BF
Body Mass (g)	Intercept	66	3.6440	(1.3456, 6.0223)	(-2.9937, 10.6466)	5.9444
Body Mass (g)	Cold Rearing	66	-8.6411	(-11.5684, -5.6703)	(-17.1851, -0.0386)	40.0256
Body Mass (g)	Warm Rearing	66	-0.0943	(-3.0373, 2.9026)	(-9.0094, 8.8486)	1.0367
Body Mass (g)	Egg Batch [mu]	66	0.4381	(0.183, 0.8491)	(0.0159, 2.1194)	Inf
Residual Tarsus Length (mm)	Intercept	66	-0.6776	(-1.2285, -0.1237)	(-2.385, 1.078)	3.8048
Residual Tarsus Length (mm)	Cold Rearing	66	0.5570	(0.0507, 1.0632)	(-0.9039, 2.068)	3.3455
Residual Tarsus Length (mm)	Warm Rearing	66	1.7784	(1.1984, 2.3508)	(0.1083, 3.4965)	53.7945
Residual Tarsus Length (mm)	Egg Batch [mu]	66	0.9709	(0.6974, 1.3195)	(0.1828, 2.2606)	Inf
Residual Bill Length (mm)	Intercept	66	0.0424	(-0.155, 0.2322)	(-0.6042, 0.6639)	1.2747
Residual Bill Length (mm)	Cold Rearing	66	-0.3300	(-0.4693, -0.1942)	(-0.7426, 0.0754)	16.9775
Residual Bill Length (mm)	Warm Rearing	66	0.4118	(0.2538, 0.566)	(-0.0487, 0.8584)	24.3165
Residual Bill Length (mm)	Egg Batch [mu]	66	0.4207	(0.3271, 0.5488)	(0.2006, 0.9089)	Inf
Metabolic Slope	Intercept	66	0.1431	(0.1378, 0.1486)	(0.1228, 0.1655)	Inf
Metabolic Slope	Cold Rearing	66	-0.0074	(-0.0127, -0.002)	(-0.0236, 0.0084)	4.7513
Metabolic Slope	Warm Rearing	66	-0.0170	(-0.0227, -0.011)	(-0.034, 0.0016)	26.0270
Metabolic Slope	Body Mass (g)	66	0.0003	(1e-04, 5e-04)	(-2e-04, 7e-04)	9.3359
Metabolic Slope	Residual Tarsus Length (mm)	66	-0.0010	(-0.0018, -1e-04)	(-0.0034, 0.002)	3.4420
Metabolic Slope	Residual Bill Length (mm)	66	-0.0108	(-0.0151, -0.0064)	(-0.0232, 0.0019)	19.9974
Metabolic Slope	Egg Batch [mu]	66	0.0073	(0.0031, 0.0143)	(3e-04, 0.0395)	Inf

```
# save_kable(week3ResultsHeatResiduals,
# "../tables/pathAnalysisInHeat3WeeksResiduals.html")

caption <- paste0(
  "Results from a Bayesian path analysis ",
  "predicting metabolic responses to heat (metabolic slopes ",
  "[fold metabolism at thermoneutrality/",
  "°C] as a function of body mass (g), relative tarsus length ",
  "(residuals; mm), relative bill length (residuals; mm) ",
  "and rearing conditions in eight ",
  "week old Japanese quail. Physiological measurements ",
  "are made at ambient temperatures between 30°C and ",
  "40°C. Cold rearing indicates post-hatch rearing at 10°C, ",
  "relative to 20°C (intercept), or 30°C ('warm rearing'). ",
  "CI indicates quantile intervals and BF indicates Bayes Factors."
)

week8ResultsHeatResiduals <-
  as.data.frame(functionModel8WeeksResidualHot) %>%
  summarise_all(., .funs = median) %>%
  pivot_longer(everything(),
    names_to = "Parameter",
    values_to = "Estimate"
  ) %>%
  merge(., quantileCIs(functionModel8WeeksResidualHot, cis = c(50, 95)),
    by = "Parameter", all.x = TRUE
  ) %>%
  filter(grepl("b_|sd_", Parameter)) %>%
  rowwise() %>%
  mutate("BF" = ifelse(Estimate < 0,
    (2 * mean(as.data.frame(
      functionModel8WeeksResidualHot
    )[, Parameter] <= 0)) /
      (2 * mean(as.data.frame(
        functionModel8WeeksResidualHot
      )[, Parameter] >= 0)),
    (2 * mean(as.data.frame(
      functionModel8WeeksResidualHot
    )[, Parameter] >= 0)) /
      (2 * mean(as.data.frame(
        functionModel8WeeksResidualHot
      )[, Parameter] <= 0))
  )) %>%
  ungroup() %>%
  mutate(
    "Estimate" = round(Estimate, digits = 4),
    "BF" = round(BF, digits = 4),
    "N" = nrow(functionModel8WeeksResidualHot$data)
  ) %>%
  mutate("Parameter" = ifelse(grepl("b_", Parameter),
    gsub("b_", "", Parameter),
    gsub(
      "Intercept", "batch",
      gsub(".*__", "", Parameter)
    )
  )) %>%
  mutate(
    "Response" = gsub("_.*", "", Parameter),
    "Parameter" = gsub(".*_", "", Parameter)
  ) %>%
  merge(., tribble(
    ~Response, ~response, ~level,
    "mass", "Body Mass (g)", "A",
    "residualTarsus", "Residual Tarsus Length (mm)", "B",
    "residualBill", "Residual Bill Length (mm)", "C",
    "slope",
    "Metabolic Slope", "D"
```

```
  ),
  by = "Response"
  ) %>%
  merge(., tribble(
    ~Parameter, ~parameter, ~number,
    "Intercept", "Intercept", "1",
    "mass", "Body Mass (g)", "4",
    "residualTarsus", "Residual Tarsus Length (mm)", "5",
    "residualBill", "Residual Bill Length (mm)", "6",
    "pretreatmentA", "Cold Rearing", "2",
    "pretreatmentC", "Warm Rearing", "3",
    "batch", "Egg Batch [mu]", "7"
  ),
  by = "Parameter"
  ) %>%
  mutate(
    `50\\% HDI` = paste0("(", paste(
      round(Low_CI_50, digits = 4),
      round(High_CI_50, digits = 4),
      sep = ", "
    ), ")"),
    `95\\% HDI` = paste0("(", paste(
      round(Low_CI_95, digits = 4),
      round(High_CI_95, digits = 4),
      sep = ", "
    ), ")")
  ) %>%
  dplyr::select(-c(Low_CI_50, High_CI_50, Low_CI_95, High_CI_95)) %>%
  dplyr::select(
    "Response" = "response", "Parameter" = "parameter", N,
    Estimate, `50\\% HDI`, `95\\% HDI`, BF, level, number
  ) %>%
  arrange(level, number) %>%
  dplyr::select(-c(level, number)) %>%
  kbl(.,
    longtable = T, booktabs = T, format = "latex", escape = FALSE,
    caption = caption
  ) %>%
  column_spec(column = c(1:2), width = "2.2cm") %>%
  column_spec(column = c(3:10), width = "1.9cm") %>%
  kable_styling(latex_options = "striped")

week8ResultsHeatResiduals
```

Table 91: *Results from a Bayesian path analysis predicting metabolic responses to heat (metabolic slopes [fold metabolism at thermoneutrality/°C] as a function of body mass (g), relative tarsus length (residuals; mm), relative bill length (residuals; mm) and rearing conditions in eight week old Japanese quail. Physiological measurements are made at ambient temperatures between 30°C and 40°C. Cold rearing indicates post-hatch rearing at 10°C, relative to 20°C (intercept), or 30°C ('warm rearing'). CI indicates quantile intervals and BF indicates Bayes Factors.*

Response	Parameter	N	Estimate	50% HDI	95% HDI	BF
Body Mass (g)	Intercept	80	-4.4747	(-8.3797, -0.5584)	(-15.8464, 7.2738)	3.5326
Body Mass (g)	Cold Rearing	80	4.5232	(-0.9892, 10.0015)	(-11.7903, 20.5327)	2.4086
Body Mass (g)	Warm Rearing	80	11.9884	(6.5242, 17.4533)	(-4.0043, 28.0663)	13.2349
Body Mass (g)	Egg Batch [mu]	80	0.4960	(0.2033, 0.9926)	(0.0164, 2.5452)	Inf
Residual Tarsus Length (mm)	Intercept	80	-0.5744	(-0.9407, -0.1722)	(-1.6868, 0.7477)	4.8097
Residual Tarsus Length (mm)	Cold Rearing	80	0.6166	(0.1706, 1.0614)	(-0.6638, 1.8948)	4.6259

Residual Tarsus Length (mm)	Warm Rearing	80	0.8432	(0.4145, 1.2701)	(-0.4685, 2.095)	9.0503
Residual Tarsus Length (mm)	Egg Batch [mu]	80	0.5358	(0.2964, 0.8275)	(0.0312, 1.6458)	Inf
Residual Bill Length (mm)	Intercept	80	0.0281	(-0.1022, 0.1607)	(-0.4255, 0.4402)	1.2903
Residual Bill Length (mm)	Cold Rearing	80	-0.2731	(-0.393, -0.1515)	(-0.6291, 0.0933)	13.7874
Residual Bill Length (mm)	Warm Rearing	80	0.2018	(0.068, 0.3327)	(-0.1819, 0.5782)	5.5735
Residual Bill Length (mm)	Egg Batch [mu]	80	0.2466	(0.1647, 0.354)	(0.034, 0.6806)	Inf
Metabolic Slope	Intercept	80	0.1073	(0.1011, 0.1135)	(0.0832, 0.1304)	Inf
Metabolic Slope	Cold Rearing	80	-0.0009	(-0.0076, 0.006)	(-0.0197, 0.0191)	1.1465
Metabolic Slope	Warm Rearing	80	0.0053	(-0.0016, 0.012)	(-0.0152, 0.0249)	2.3181
Metabolic Slope	Body Mass (g)	80	0.0002	(1e-04, 3e-04)	(0, 5e-04)	30.1284
Metabolic Slope	Residual Tarsus Length (mm)	80	-0.0024	(-0.0037, -0.0011)	(-0.0063, 0.0014)	8.5352
Metabolic Slope	Residual Bill Length (mm)	80	-0.0055	(-0.0097, -0.0012)	(-0.0175, 0.0072)	4.1249
Metabolic Slope	Egg Batch [mu]	80	0.0104	(0.0052, 0.0178)	(5e-04, 0.0435)	Inf

```
#save_kable(week8ResultsHeatResiduals,
# "../tables/pathAnalysisInHeat8WeeksResiduals.html")
```

4.0 Effect of morphology on evaporative cooling

Overview

In desert bird species, body mass has been shown to influence the rate at which individuals lose water to evaporation in the heat (McKechnie et al, 2021). Similarly, in male great tits (*Parus major*), tarsus surface area appears correlated with evaporative cooling efficiency at temperatures above thermoneutrality (Playà-Montmany et al, 2021). In light of these findings, we sought to test whether alignment with Bergmann's and Allen's rules may benefit individuals by either increasing their capacity to contend with metabolic heat production through evaporative cooling, or decreasing their demand for evaporative cooling in the heat. Below, we collate measurements of evaporative water loss in developing (three weeks of age) and adult (eight weeks of age) Japanese quail, visualise them for evidence of oddities/errors, use them to estimate rates of evaporative heat loss (described in the main text of Tabh et al, 2025), then combine them with resting metabolic measurements to estimate evaporative cooling efficiency per individual (here, represented as evaporative heat loss/resting metabolism, each in Watts). Once complete, we test whether: (1) increases in evaporative heat loss between thermoneutrality (30°C) and hot environments (40°C), and (2) evaporative cooling efficiency, vary by body mass and appendage length (here, tarsus and bill length) in our quail.

Data import, collation, and filtration

We begin this document by loading in R packages and functions necessary for data collation, visualisation, and analysis. We then import our data, inspect it for oddities and errors, and use it to visualise raw trends. Last, models testing effects of morphology on evaporative heat loss and cooling efficiency are produced for evaluation.

```
library("tidyverse")
library("easypackages")

packageList <- c("bayesplot", "brms", "brmsMethods",
                 "doParallel", "foreach", "ggpubr",
                 "kableExtra", "latex2exp", "patchwork",
                 "priorsense", "showtext", "tidybayes",
                 "wesanderson")

libraries(packageList)

caption <- paste0("R packages and their respective versions used for",
                  " data organisation and analysis in this study."
)

sapply(packageList, function(x) {
  y <- as.character(packageVersion(x))
  return(y)
}, simplify = FALSE) %>%
  enframe(., name = "Package", value = "Version") %>%
  as.data.frame(.) %>%
  kbl(.,
    longtable = T, booktabs = T,
    caption = caption
  ) %>%
  kable_styling(latex_options = "striped")
```

Table 92: *R packages and their respective versions used for data organisation and analysis in this study.*

Package	Version
bayesplot	1.11.1
brms	2.22.7
brmsMethods	0.0.0.9000
doParallel	1.0.17
foreach	1.5.2
ggpubr	0.6.0
kableExtra	1.4.0
latex2exp	0.9.6

patchwork	1.2.0
priorsense	1.0.2
showtext	0.9.7
tidybayes	3.0.6
wesanderson	0.3.7

```
# Loading additional functions

pp_check2 <- function(model, resp = NA, ndraws = 500,
                      xlab = "label", colour = "lightblue") {
  require(brms)
  require(ggplot2)
  stopifnot("Model must be a brmsfit object" = is.brmsfit(model))

  if (is.na(resp)) {
    resp <- model$formula$resp
  }

  p1 <- brms::pp_check(model, ndraws = ndraws, resp = resp) +
    scale_colour_manual(
      values = c("black", colour),
      labels = c("y", "yhat"),
      name = NULL
    ) +
    xlab(xlab) +
    ylab("Density") +
    theme_classic()
  return(p1)
}

chainCheck <- function(model, rDig = 3) {
  require(brms)
  stopifnot("Model must be a brmsfit object" = is.brmsfit(model))

  Rhat <- paste0(
    "Rhat range: ",
    round(min(rhat(model)), digits = rDig),
    " - ",
    round(max(rhat(model)), digits = rDig)
  )
  Neff <- paste0(
    "Neff/N range: ",
    round(min(neff_ratio(model)), digits = rDig),
    " - ",
    round(max(neff_ratio(model)), digits = rDig)
  )
  cat(paste0(Rhat, "\n", Neff))
}

quantileCIs <- function(x, rnd = 3, cis = c(50, 95), sci_note = FALSE) {
  require(tidyverse)

  if (class(x)[1] != "brmsfit") {
    return("x must be a brmsfit object.")
  }
  if (length(cis) != 2) {
    return("cis must be a vector of integers with length 2")
  }

  prbs = c()
  nColNames = c()
  for (i in 1:length(cis)){
    prbs = c(prbs, c(0.5 - (cis[i]/100)/2, 0.5 + (cis[i]/100)/2))
    nColNames = c(nColNames,
                  paste0("Low_CI_", cis[i]),
                  paste0("High_CI_", cis[i])
```

```
    )
  }

  modelFrame = as.data.frame(x)

  Results <- apply(modelFrame, MARGIN = 2, FUN = quantile,
        probs = prbs, type = 8) %>%
    t() %>%
    as.data.frame() %>%
    rownames_to_column(var = "par") %>%
    `colnames<-`(c("Parameter", nColNames))

  if (sci_note == FALSE) {
    Results <- apply(modelFrame, MARGIN = 2, FUN = quantile,
        probs = prbs, type = 8) %>%
    t() %>%
    as.data.frame() %>%
    rownames_to_column(var = "par") %>%
    `colnames<-`(c("Parameter", nColNames))

  } else if (sci_note == TRUE) {
    Results <- apply(modelFrame, MARGIN = 2, FUN = quantile,
        probs = prbs, type = 8) %>%
    t() %>%
    as.data.frame() %>%
    rownames_to_column(var = "par") %>%
    `colnames<-`(c("Parameter", nColNames)) %>%
    mutate_at(.vars = vars(-Parameter),
              .funs = function(x){
                return(format(x, scientific = TRUE))
              }
    )
  }

  return(Results)
}

# Installing font

font_add_google(name = "Noto Sans", family = "Noto Sans")

# Setting our working directory

setwd("/Users/joshuatabh/analyses")
```

Here, our evaporative water loss, morphometry, and resting metabolism data are imported and bound. Collation and filtration of morphometric and metabolic data are described in the previous sections of this document (sections 2.0 and 3.0 respectively). Evaporative heat loss and evaporative cooling efficiency data are then visualised for potential errors using Cleveland dotplots.

```
# Morphometric data are loaded.

data <- read.csv("compiledDataFull.csv")

# Next, metabolic data are loaded and filtered as previously described.

{
  vo2Data <- bind_rows(
    read.csv("exp1VO2.csv"),
    read.csv("exp2VO2.csv"),
    read.csv("exp3VO2.csv")
  ) %>%
    dplyr::select(-c(startTime, endTime, initialTb, fileName)) %>%
    mutate(pretreatment = ifelse(pretreatment == "control",
      "neutral", pretreatment
    ))
```

```
  all <- merge(
    data %>%
      dplyr::select(-Ta),
    vo2Data %>%
      distinct() %>%
      dplyr::select(-c(pretreatment, posttreatment, birdID)),
    by = c("ring", "week", "exp"),
    all.x = TRUE
  ) %>%
    distinct()

  all <- all %>%
    dplyr::select(
      ring, birdID, sex, exp, week, pretreatment,
      posttreatment, treatment, Ta, mass, wingLength,
      meanTb, tarsusLengthMean, tarsusLengthSD,
      tarsusCalibration, billLengthMean, billLengthSD,
      billCalibration, VO2, RMR
    )

  all <- all %>%
    mutate(Ta = ifelse(exp == "C" & Ta > 28 & Ta < 31, 30, Ta)) %>%
    mutate(Ta = ifelse(exp == "C" & Ta > 38 & Ta < 41, 40, Ta)) %>%
    filter(Ta %in% c(10, 20, 30, 40))

  all <- rbind(
    all %>%
      filter(ring == "B14" & week == "3") %>%
      group_by(Ta) %>%
      mutate(
        "tarsusLengthMean" = mean(tarsusLengthMean, na.rm = T),
        "billLengthMean" = mean(billLengthMean, na.rm = T)
      ) %>%
      mutate("n" = 1:2) %>%
      ungroup() %>%
      arrange(n, Ta) %>%
      slice(1:4) %>%
      dplyr::select(-n),
    all %>%
      filter(!(ring == "B14" & week == "3"))
  ) %>%
    arrange(exp, week, ring, Ta)
}

# Evaporative water loss data are now imported and bound with all other data.

vh2o = all %>%
  merge(.,
        bind_rows(
          read.csv("exp1VH2O.csv"),
          read.csv("exp2VH2O.csv"),
          read.csv("exp3VH2O.csv") %>%
            mutate(Ta = ifelse(Ta > 28 & Ta < 31, 30, Ta)) %>%
            mutate(Ta = ifelse(Ta > 38 & Ta < 41, 40, Ta)) %>%
            mutate(week = ifelse(week == 4, 3, 8))
        ), by =
          c("ring", "birdID", "exp", "week",
            "pretreatment", "posttreatment", "Ta"),
        all.x = TRUE
  ) %>% filter(Ta %in% c(10, 20, 30, 40)) %>%
  mutate(ehl = (VH2O*2.406)/60) %>%
  mutate(ecc = ehl/RMR)

# With data loaded, we check the spread of evaporative heat loss measures by
# ambient temperature

ggplot(
```

```
  vh2o %>%
    filter(week %in% c(3, 8)) %>%
    group_by(week) %>%
    mutate("ID" = 1:n()) %>%
    ungroup() %>%
    mutate(
      "week" = paste0("Age = ", week, " weeks"),
      "Ta" = paste0(Ta, "°C")
    ),
  aes(x = ID, y = ehl)
) +
  facet_grid(week ~ Ta, scales = "free") +
  geom_point(size = 2, colour = "black",
             fill = "slateblue", alpha = 0.7) +
  geom_rect(
    data = vh2o %>%
      filter(week %in% c(3, 8)) %>%
      group_by(week, Ta) %>%
      summarise(
        "Mean" = mean(ehl, na.rm = T),
        "LCL" = Mean - 4 * sd(ehl, na.rm = T),
        "UCL" = Mean + 4 * sd(ehl, na.rm = T),
        "ID" = 1, "ehl" = 1
      ) %>%
      ungroup() %>%
      mutate(
        "week" = paste0("Age = ", week, " weeks"),
        "Ta" = paste0(Ta, "°C")
      ) %>%
      as.data.frame(),
    aes(xmin = -Inf, xmax = Inf, ymin = LCL, ymax = UCL),
    colour = "black", fill = "grey70", alpha = 0.5
  ) +
  theme_classic() +
  xlab("Sample Number") +
  ylab("Evaporative Heat Loss (W)")
```

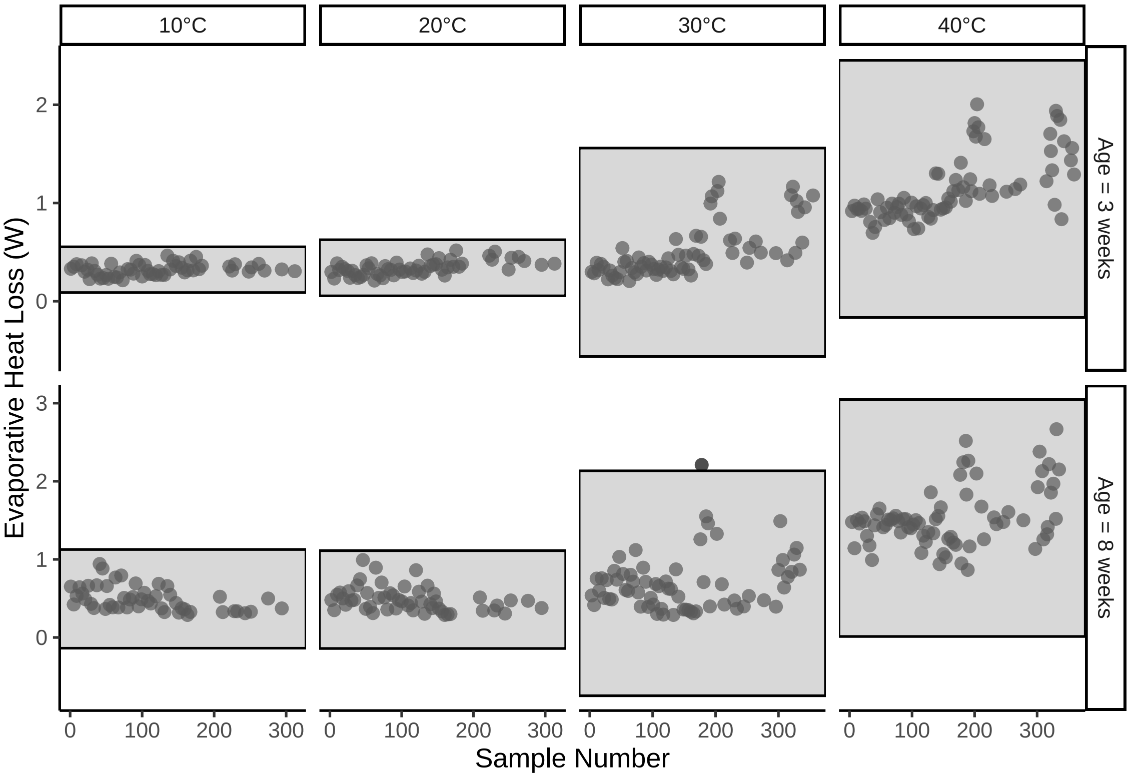

Figure 143: *Cleveland dotplot of evaporative heat loss (W) by ambient temperature of collection, each drawn from adult Japanese quail. Dots represent raw data points. Grey rectangles indicate areas captured by means +/- 4 standard deviations.*

```
ggplot(
  vh2o %>%
    filter(week %in% c(3, 8)) %>%
    group_by(week) %>%
    mutate("ID" = 1:n()) %>%
    ungroup() %>%
    mutate(
      "week" = paste0("Age = ", week, " weeks"),
      "Ta" = paste0(Ta, "°C")
    ),
  aes(x = ID, y = ecc)
) +
  facet_grid(week ~ Ta, scales = "free") +
  geom_point(size = 2, colour = "black",
             fill = "slateblue", alpha = 0.7) +
  geom_rect(
    data = vh2o %>%
      filter(week %in% c(3, 8)) %>%
      group_by(week, Ta) %>%
      summarise(
        "Mean" = mean(ecc, na.rm = T),
        "LCL" = Mean - 4 * sd(ecc, na.rm = T),
        "UCL" = Mean + 4 * sd(ecc, na.rm = T),
        "ID" = 1, "ecc" = 1
      ) %>%
      ungroup() %>%
      mutate(
        "week" = paste0("Age = ", week, " weeks"),
        "Ta" = paste0(Ta, "°C")
      ) %>%
      as.data.frame(),
```

```
    aes(xmin = -Inf, xmax = Inf, ymin = LCL, ymax = UCL),
    colour = "black", fill = "grey70", alpha = 0.5
  ) +
  theme_classic() +
  xlab("Sample Number") +
  ylab("Evaporative Cooling Efficiency (EHL/RMR)")
```

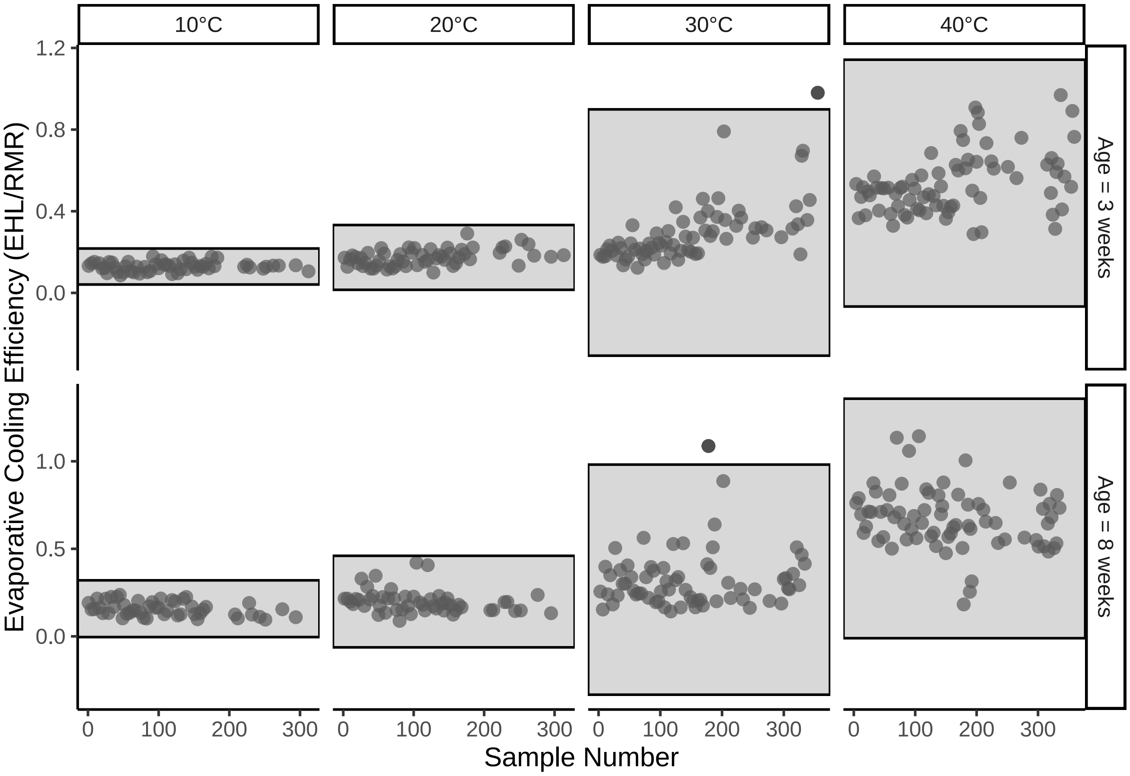

Figure 144: *Cleveland dotplot of evaporative cooling efficiency (evaporative heat loss/resting metabolism, each in watts) by ambient temperature of collection, each drawn from adult Japanese quail. Dots represent raw data points. Grey rectangles indicate areas captured by means +/- 4 standard deviations.*

Some measurements of evaporative heat loss and evaporative cooling efficiency are notably high at 30°C, however, these values still fit within the distribution of biologically realistic measurements (i.e. by comparison with measurements drawn at 40°C). For this reason, these measurements are retained and we proceed to visualing raw trends in our data.

```
p1 <- vh2o %>%
  filter(week %in% c(3,8)) %>%
  mutate(week = paste0(week, " Weeks")) %>%
  ggplot(aes(x = Ta, y = ehl)) +
  facet_wrap(~week) +
  geom_line(aes(group = ring), colour = "grey50", alpha = 0.3) +
  geom_point(pch = 21, size = 1.5, colour = "black", fill = "grey50",
             alpha = 0.3) +
  stat_summary(geom = "line", fun = "mean", colour = "black") +
  stat_summary(geom = "errorbar", fun.data = "mean_cl_boot", width = 2,
               colour = "black") +
  stat_summary(geom = "point", fun = "mean", pch = 21, size = 4,
               colour = "black", fill = "lightblue3") +
  xlab("Ambient Temperature (°C)") +
  ylab("Evaporative Heat\nLoss (W)") +
  theme_classic()
```

```
p2 <- vh2o %>%
  filter(week %in% c(3,8)) %>%
  mutate(week = paste0(week, " Weeks")) %>%
  ggplot(aes(x = Ta, y = ecc)) +
  facet_wrap(~week) +
  geom_line(aes(group = ring), colour = "grey50", alpha = 0.3) +
  geom_point(pch = 21, size = 1.5, colour = "black", fill = "grey50",
             alpha = 0.3) +
  stat_summary(geom = "line", fun = "mean", colour = "black") +
  stat_summary(geom = "errorbar", fun.data = "mean_cl_boot", width = 2,
               colour = "black") +
  stat_summary(geom = "point", fun = "mean", pch = 21, size = 4,
               colour = "black", fill = "lightblue3") +
  xlab("Ambient Temperature (°C)") +
  ylab("Evaporative Cooling\nEfficiency (EHL/RMR)") +
  theme_classic()

p1/p2 + plot_annotation(tag_levels = "A")
```

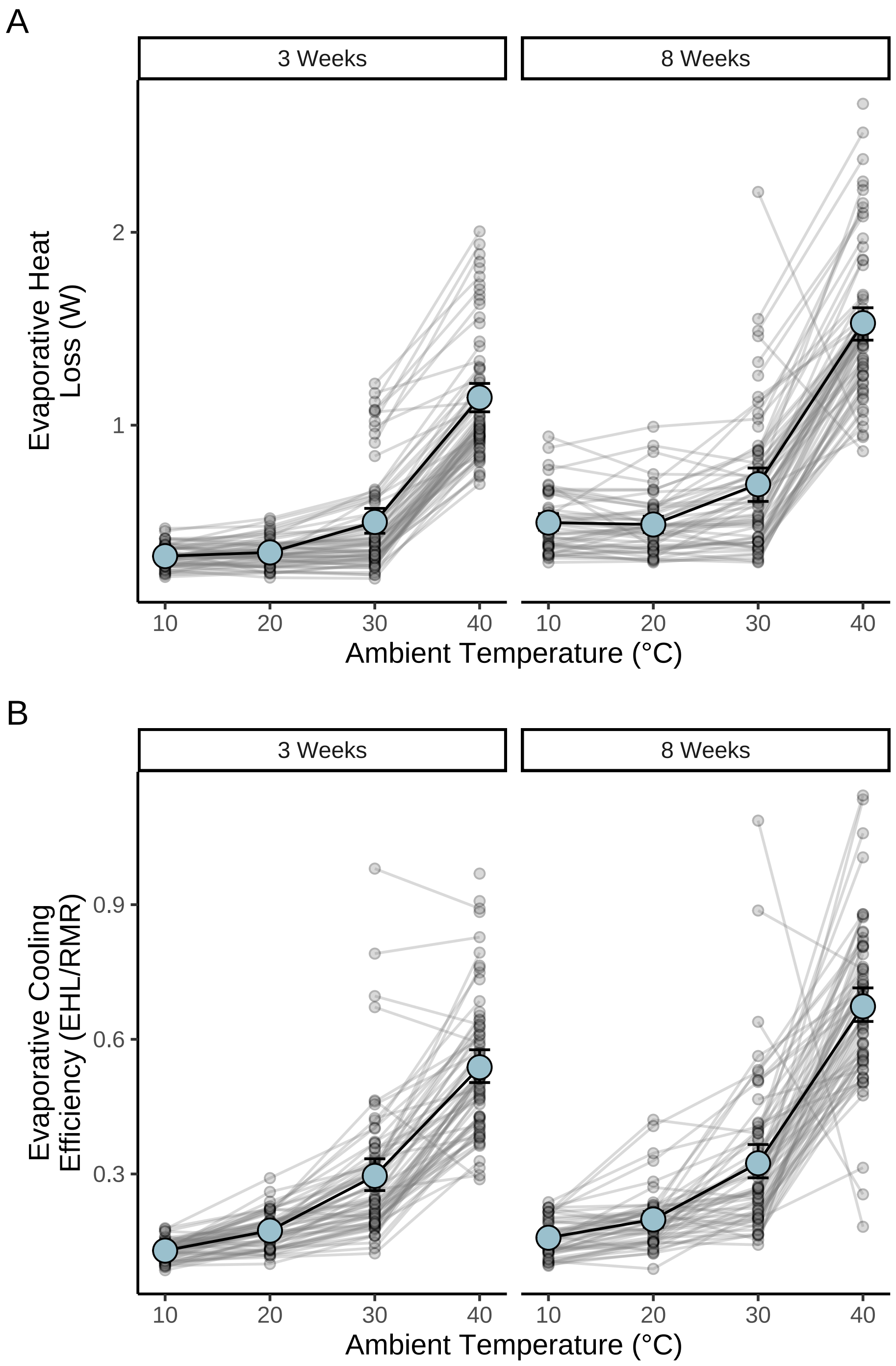

Figure 145: *Effect of ambient temperature (°C) on evaporative heat production and cooling efficiency in developing (3 weeks) and adult (8 weeks) Japanese quail. Evaporative cooling efficiency represents the ratio of evaporative heat loss (W) to metabolic heat production (W) at a given ambient temperature. Large dots represent means and errorbars indicate +/- one standard error. Small dots represent raw values and lines connect measurements drawn from the same individual.*

Next, we explore raw correlations between morphology (i.e. body mass [g], tarsus length [mm], bill length [mm]) and both evaporative heat loss and evaporative cooling efficiency at 40°C. Raw effects of rearing condition on evaporative heat loss and cooling efficiency are also visualised.

```
p1 <- vh2o %>%
  filter(week %in% c(3,8) & Ta == 40) %>%
  mutate(week = paste0(week, " Weeks")) %>%
  ggplot(aes(x = mass, y = ehl)) +
  facet_wrap(~week, scales = "free") +
  geom_point(pch = 21, size = 2, colour = "black", fill = "grey50",
             alpha = 0.3) +
  xlab("Body Mass (g)") +
  ylab("Evaporative Heat\nLoss (W)") +
  theme_classic()

p2 <- vh2o %>%
  filter(week %in% c(3,8) & Ta == 40) %>%
  mutate(week = paste0(week, " Weeks")) %>%
  ggplot(aes(x = mass, y = ecc)) +
  facet_wrap(~week, scales = "free") +
  geom_point(pch = 21, size = 2, colour = "black", fill = "grey50",
             alpha = 0.3) +
  xlab("Body Mass (g)") +
  ylab("Evaporative Cooling\nEfficiency (EHL/RMR)") +
  theme_classic()

p1/p2
```

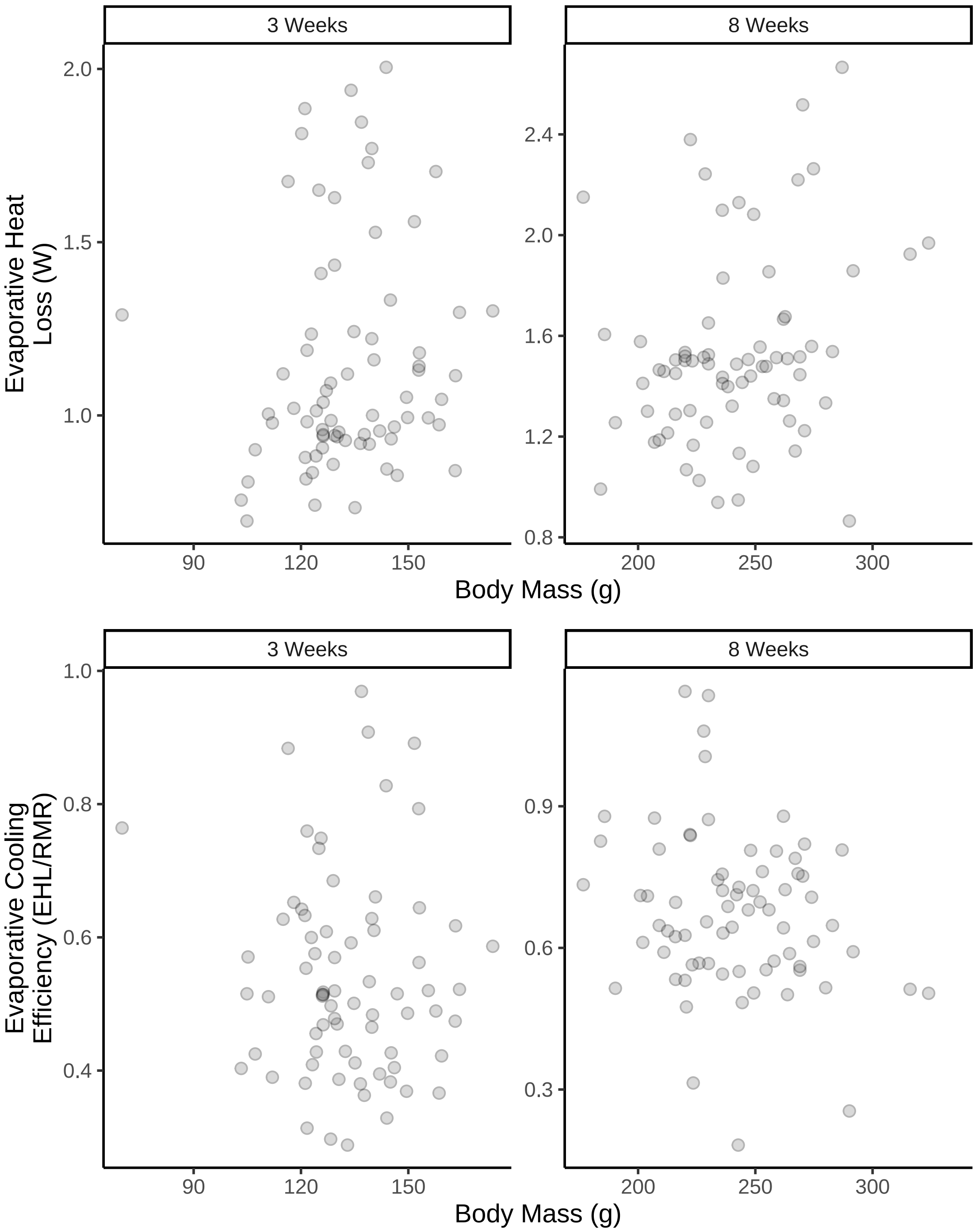

Figure 146: *Evaporative heat loss and cooling efficiency as a function of body mass in developing (3 weeks) and adult (8 weeks) Japanese quail (n = 82). Small dots represent raw values. Quail were reared at either 10°C, 20°C, or 30°C until at least three weeks of age.*

```
p1 <- vh2o %>%
  filter(week %in% c(3,8) & Ta == 40) %>%
  mutate(week = paste0(week, " Weeks")) %>%
  ggplot(aes(x = tarsusLengthMean, y = ehl)) +
  facet_wrap(~week, scales = "free") +
  geom_point(pch = 21, size = 2, colour = "black", fill = "grey50",
             alpha = 0.3) +
```

```
  xlab("Tarsus Length (mm)") +
  ylab("Evaporative Heat Loss (W)") +
  theme_classic()

p2 <- vh2o %>%
  filter(week %in% c(3,8) & Ta == 40) %>%
  mutate(week = paste0(week, " Weeks")) %>%
  ggplot(aes(x = tarsusLengthMean, y = ecc)) +
  facet_wrap(~week, scales = "free") +
  geom_point(pch = 21, size = 2, colour = "black", fill = "grey50",
             alpha = 0.3) +
  xlab("Tarsus Length (mm)") +
  ylab("Evaporative Cooling\nEfficiency (EHL/RMR)") +
  theme_classic()

p1/p2
```

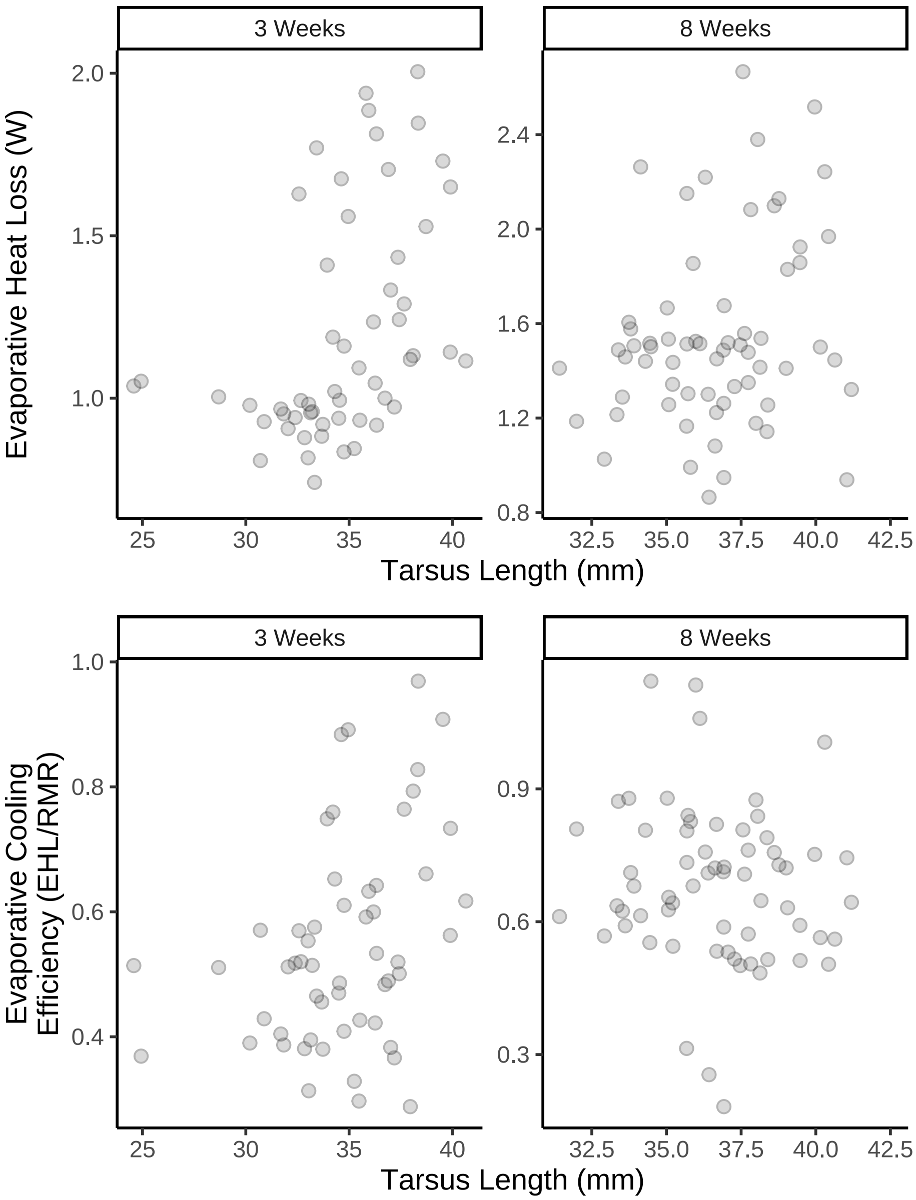

Figure 147: *Evaporative heat loss and cooling efficiency as a function of tarsus length (mm) in developing (3 weeks) and adult (8 weeks) Japanese quail (n = 82). Small dots represent raw values. Quail were reared at either 10°C, 20°C, or 30°C until at least three weeks of age.*

```
p1 <- vh2o %>%
  filter(week %in% c(3,8) & Ta == 40) %>%
  mutate(week = paste0(week, " Weeks")) %>%
  ggplot(aes(x = billLengthMean, y = ehl)) +
  facet_wrap(~week, scales = "free") +
  geom_point(pch = 21, size = 2, colour = "black", fill = "grey50",
             alpha = 0.3) +
  xlab("Bill Length (mm)") +
  ylab("Evaporative Heat Loss (W)") +
  theme_classic()

p2 <- vh2o %>%
  filter(week %in% c(3,8) & Ta == 40) %>%
  mutate(week = paste0(week, " Weeks")) %>%
```

```
  ggplot(aes(x = billLengthMean, y = ecc)) +
  facet_wrap(~week, scales = "free") +
  geom_point(pch = 21, size = 2, colour = "black", fill = "grey50",
             alpha = 0.3) +
  xlab("Bill Length (mm)") +
  ylab("Evaporative Cooling\nEfficiency (EHL/RMR)") +
  theme_classic()

p1/p2
```

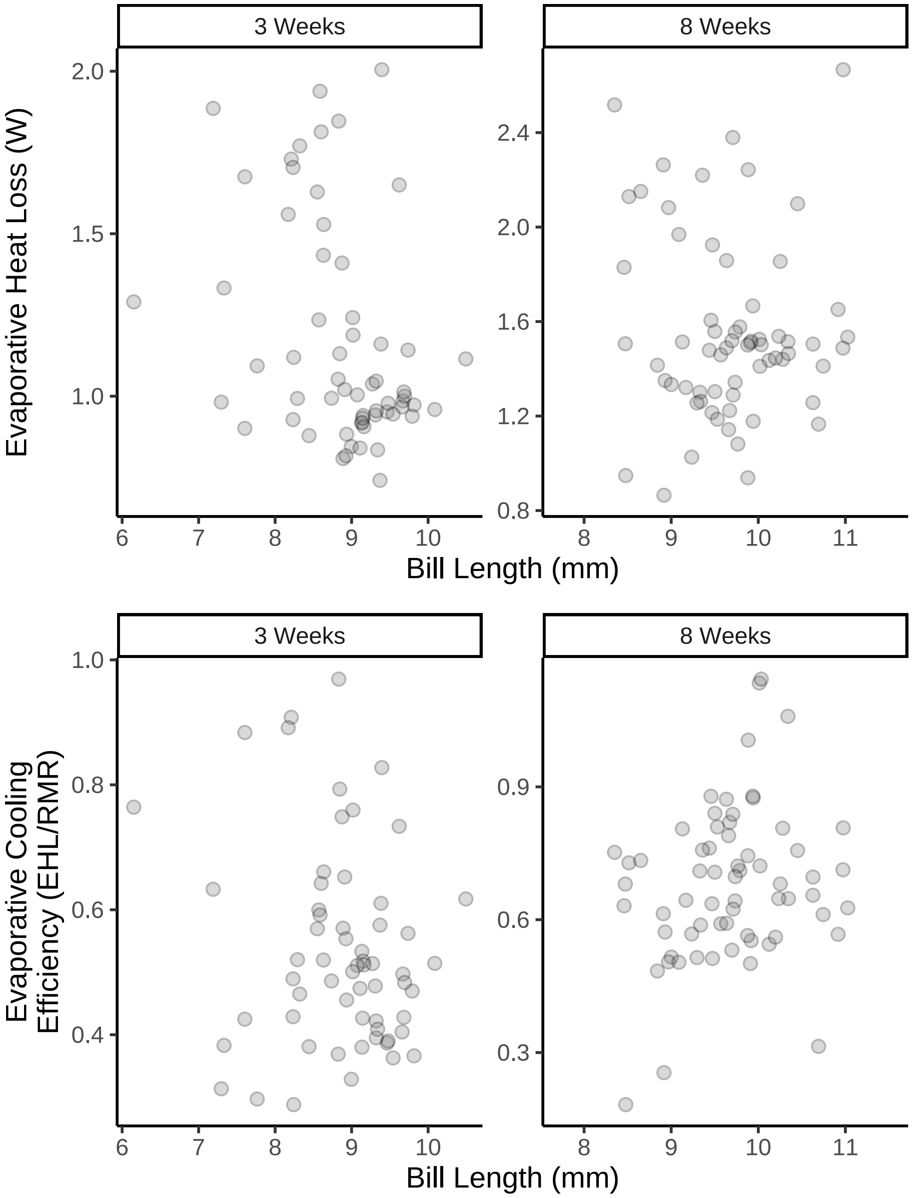

Figure 148: *Evaporative heat loss and cooling efficiency as a function of bill length (mm) in developing (3 weeks) and adult (8 weeks) Japanese quail (n = 82). Small dots represent raw values. Quail were reared at either 10°C, 20°C, or 30°C until at least three weeks of age.*

```
p1 <- vh2o %>%
  filter(week %in% c(3, 8) & Ta == 40) %>%
  mutate(week = paste0(week, " Weeks")) %>%
```

```
  ggplot(aes(x = ehl, fill = pretreatment)) +
  facet_wrap(~week, scales = "free") +
  geom_density(alpha = 0.3) +
  xlab("Evaporative Heat Loss (W)") +
  ylab("Density") +
  scale_fill_manual(
    values = c("#7BB4E3", "black", "#CD5C5C"),
    name = "Rearing\nTreatment",
    labels = c(
      "Cold (10°C)",
      "Mild (20°C)",
      "Warm (30°C)"
    )
  ) +
  theme_classic() +
  theme(legend.position = "bottom")

p2 <- vh2o %>%
  filter(week %in% c(3, 8) & Ta == 40) %>%
  mutate(week = paste0(week, " Weeks")) %>%
  ggplot(aes(x = ecc, fill = pretreatment)) +
  facet_wrap(~week, scales = "free") +
  geom_density(alpha = 0.3) +
  xlab("Evaporative Cooling Efficiency (EHL/RMR)") +
  ylab("Density") +
  scale_fill_manual(
    values = c("#7BB4E3", "black", "#CD5C5C"),
    name = "Rearing\nTreatment",
    labels = c(
      "Cold (10°C)",
      "Mild (20°C)",
      "Warm (30°C)"
    )
  ) +
  theme_classic() +
  theme(legend.position = "bottom")

p1 / p2
```

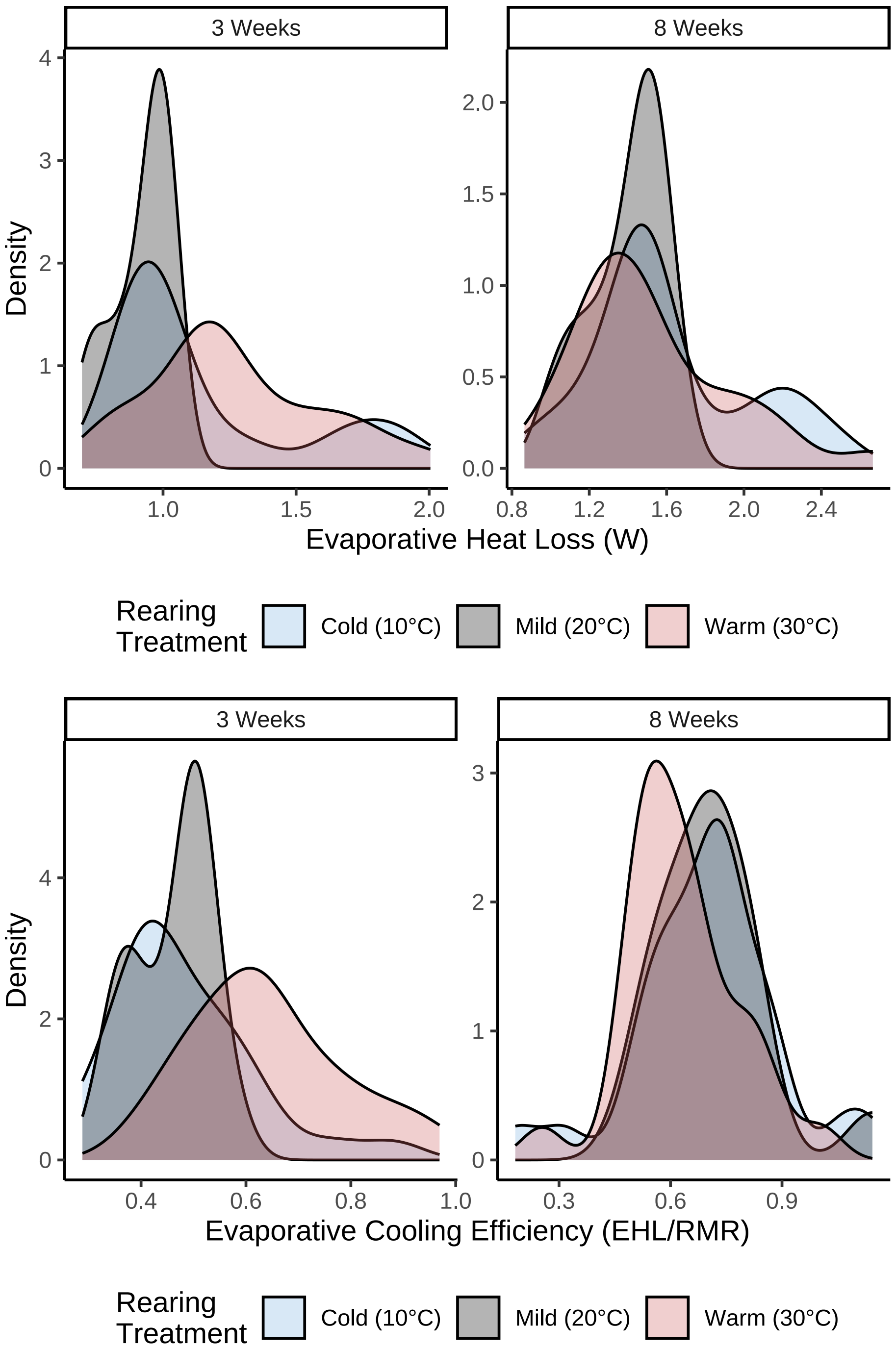

Figure 149: *Density of evaporative heat loss and cooling efficiency measurements among Japanese quail reared at different temperatures (10°C, 20°C, or 30°C) until at least 3 weeks of age (n = 82). Measurements were taken at 40°C.*

Variance in body evaporative heat loss (W) and evaporative cooling efficiency may differ subtly among rearing treatments. Model residuals are therefore scrutinised below to assess whether remaining variance is indeed treatment-dependent. Last, we check whether variance in each metric differs between experimental batch 3 and experimental batches 1 and 2 owing to increased flow rates at this time.

```
p1 <- vh2o %>%
  rename("batch" = exp) %>%
  filter(week %in% c(3, 8) & Ta == 40) %>%
  mutate(week = paste0(week, " Weeks")) %>%
  ggplot(aes(x = ehl, fill = pretreatment)) +
  facet_wrap(~week, scales = "free") +
  geom_density(alpha = 0.3) +
  xlab("Evaporative Heat Loss (W)") +
  ylab("Density") +
  scale_fill_manual(
    values = c("#7BB4E3", "black", "#CD5C5C"),
    name = "Experimental\nBatch",
    labels = c(
      "1",
      "2",
      "3"
    )
  ) +
  theme_classic() +
  theme(legend.position = "bottom")

p2 <- vh2o %>%
  rename("batch" = exp) %>%
  filter(week %in% c(3, 8) & Ta == 40) %>%
  mutate(week = paste0(week, " Weeks")) %>%
  ggplot(aes(x = ecc, fill = pretreatment)) +
  facet_wrap(~week, scales = "free") +
  geom_density(alpha = 0.3) +
  xlab("Evaporative Cooling Efficiency (EHL/RMR)") +
  ylab("Density") +
  scale_fill_manual(
    values = c("#7BB4E3", "black", "#CD5C5C"),
    name = "Experimental\nBatch",
    labels = c(
      "1",
      "2",
      "3"
    )
  ) +
  theme_classic() +
  theme(legend.position = "bottom")

p1 / p2
```

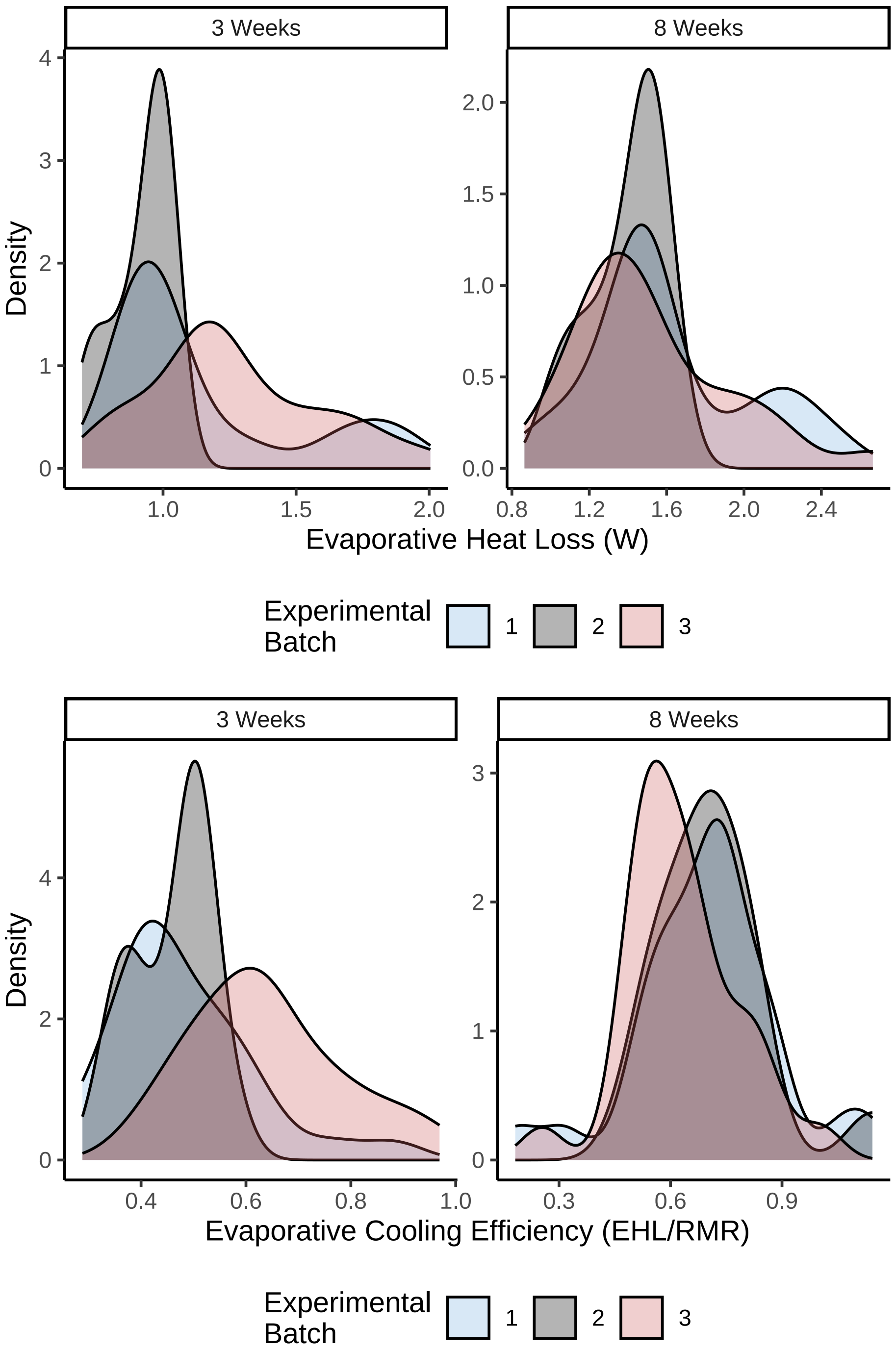

Figure 150: *Density of evaporative heat loss and cooling efficiency measurements among Japanese quail derived from three different egg (or experimental) batches. Measurements were taken at 40°C.*

Variance appears particular large among experimental batch 3 for measures of evaporative cooling efficiency at three weeks of age. Given this, and that evaporative cooling efficiency was calculated using resting metabolism values which also appeared to vary considerably in experimental batch 3 at this age (refer to section 2.0), our error term for our model predicting evaporative cooling efficiency during development (below) is permitted to vary by experimental batch.

Testing effects of morphology and thermal history on evaporative cooling

In our study (Tabh et al, 2025) we show that rearing temperature influences developmental trajectories and mature morphology of Japanese quail. However, in a previous study, we have also shown that rearing temperatures can directly effect evaporative heat loss and cooling capacities in this species (Persson et al, 2024). As such, to evaluate direct effects of morphology on evaporative heat loss responses and cooling efficiency while controlling for known effects of rearing conditions on each, we constructed two Bayesian path analyses depicted in the figure below.

```
data.frame(x = c(-5:5), y = c(-5:5)) %>%
  ggplot(aes(x = x, y = y)) +
  annotate("text", x = 0, y = 5, label = paste0(
    "Developmental, ",
    "Thermal\nEnvironment"
  ), colour = "black") +
  annotate("text",
    x = -2.5, y = 2.5, label = "Body Mass (g)",
    colour = "black"
  ) +
  annotate("text",
    x = 2.5, y = 2.5, label = "Appendage Length\n(mm)",
    colour = "black"
  ) +
  annotate("text",
    x = 0, y = -0.4,
    label = paste0(
      "[1] Evaporative Heat Loss\n",
      "Response OR\n",
      "[2] Evaporative Cooling\n",
      "Efficiency"
    ),
    colour = "black"
  ) +
  geom_segment(
    lineend = "round", linejoin = "round",
    size = 0.3, linetype = "solid", colour = "grey10",
    aes(x = -0.25, y = 4.6, xend = -2.5, yend = 2.75),
    arrow = arrow(length = unit(0.2, "cm"))
  ) +
  geom_segment(
    lineend = "round", linejoin = "round",
    size = 0.3, linetype = "solid", colour = "grey10",
    aes(x = 0.25, y = 4.6, xend = 2.5, yend = 2.75),
    arrow = arrow(length = unit(0.2, "cm"))
  ) +
  geom_segment(
    lineend = "round", linejoin = "round",
    size = 0.3, linetype = "solid", colour = "grey10",
    aes(x = -1.25, y = 2.5, xend = 1, yend = 2.5),
    arrow = arrow(length = unit(0.2, "cm"))
  ) +
  geom_segment(
    lineend = "round", linejoin = "round",
    size = 0.3, linetype = "dotted", colour = "grey10",
    aes(x = -2.5, y = 2.25, xend = -0.25, yend = 0.25),
    arrow = arrow(length = unit(0.2, "cm"))
  ) +
  geom_segment(
    lineend = "round", linejoin = "round",
    size = 0.3, linetype = "longdash", colour = "grey10",
    aes(x = 2.5, y = 2.25, xend = 0.25, yend = 0.25),
    arrow = arrow(length = unit(0.2, "cm"))
  ) +
  geom_curve(
    lineend = "round",
    size = 0.3, linetype = "solid", colour = "grey10",
```

```
  aes(x = -1.75, y = 4.75, xend = -1.75, yend = 0.25),
  arrow = arrow(length = unit(0.2, "cm"))
) +
xlim(c(-6, 6)) +
ylim(c(-0.7, 6)) +
theme_void()
```

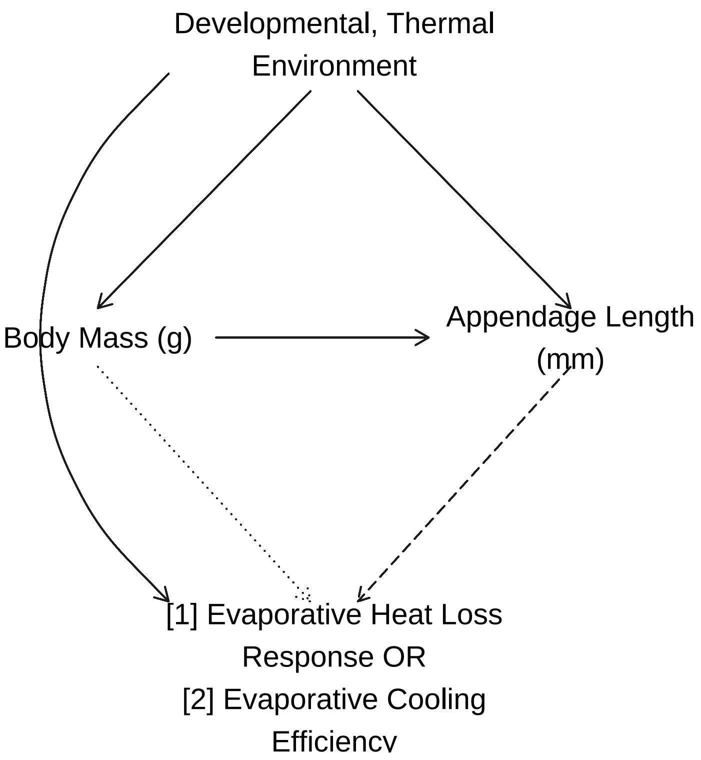

Figure 151: *Flow-chart describing expected effects of developmental, thermal environment on morphology, and subsequently, thermal physiology of Japanese quail. Dashed and dotted lines represent effects predicted under a adaptive hypotheses of Allen's rule and Bergmann's rule respectively.*

Similar to our previous analyses (described in section 3.0 above), equations within these path analyses were as follows:

$$Body\ Mass_j \sim \beta_{a0} + \beta_{a1} \cdot Cold\ Rearing_j + \beta_{a2} \cdot Warm\ Rearing_j + \mu_{0a} + \epsilon_a$$

$$Tarsus\ Length_j \sim \beta_{b0} + \beta_{b1} \cdot Cold\ Rearing_j + \beta_{b2} \cdot Warm\ Rearing_j + \beta_{b3} \cdot Body\ Mass_j + \mu_{0b} + \epsilon_b$$

$$Bill\ Length_j \sim \beta_{c0} + \beta_{c1} \cdot Cold\ Rearing_j + \beta_{c2} \cdot Warm\ Rearing_j + \beta_{c3} \cdot Body\ Mass_j + \mu_{0c} + \epsilon_c$$

and either:

$$Fold\ Evaporative\ Heat\ Loss_j \sim \beta_{d0} + \beta_{d1} \cdot Cold\ Rearing_j + \beta_{d2} \cdot Warm\ Rearing_j + \beta_{d3} \cdot Body\ Mass_j + \beta_{d4} \cdot Tarsus\ Length_j + \beta_{d5} \cdot Bill\ Length_j + \mu_{0d} + \epsilon_d$$

where:

$$Fold\ Evaporative\ Heat\ Loss_j = \frac{Evaporative\ Heat\ Loss_{40°\mathrm{C}j}}{Evaporative\ Heat\ Loss_{30°\mathrm{C}j}}$$

or:

$$Evaporative\ Cooling\ Efficiency_j \sim \beta_{d0} + \beta_{d1} \cdot Cold\ Rearing_j + \beta_{d2} \cdot Warm\ Rearing_j + \beta_{d3} \cdot Body\ Mass_j + \beta_{d4} \cdot Tarsus\ Length_j + \beta_{d5} \cdot Tarsus\ Length_j + \mu_{0d} + \epsilon_{dj}$$

where evaporative cooling efficiency is measured as:

$$Evaporative\ Cooling\ Efficiency_j = \frac{Evaporative\ Heat\ Loss_{40°\mathrm{C}j}}{Metabolic\ Heat\ Production_{40°\mathrm{C}j}}$$

and:

$$\epsilon_{dj} \sim e^{(\tau_0 + \tau_1 \cdot Batch_{2j} + \tau_2 \cdot Batch_{3j})}$$

In all cases, j represents individual (and thus, observation) identity, "warm rearing" and "cold rearing" represent binomial parameters with "0" corresponding to "false" and "1" corresponding to "true", μ_{0a} - μ_{0c} represent group-level intercepts of egg batch on each response variable, and body mass, tarsus length and bill length are mean-centred to improve interpretability of model intercepts(β_0 terms). For models predicting body mass, tarsus length, and bill length ϵ is assumed to be normally distributed and centred at 0. For model 3 (predicting evaproative cooling efficiency) epsilon is allowed to vary by experimental batch. There, τ_0 indicates the natural log-transformed error term for experimental batch 1, τ_1 indicates the change in error associated with measures derived from experimental batch 2, and τ_2 indicates the change in error associated with measures derived from experimental batch 3; $Batch_{2j}$ and $Batch_{3j}$ indicate binomial variables pertaining to whether a slope from individual j was derived from experimental batch 2 or 3 respectively (0 = no; 1 = yes). Last, residuals are assumed to be uncorrelated across models.

Modelling for juveniles

All measurement values refer to those collected during development (3 weeks of age).

For our first two equations described above (i.e. those predicting mean-centred body mass, tarsus length and bill length), priors were as follows (regardless of which path analyses they were encompassed within):

$$\beta_{a0} \sim \mathcal{N}(0, 5)$$
$$\beta_{a1} \sim \mathcal{N}(0, 15)$$
$$\beta_{a2} \sim \mathcal{N}(0, 15)$$
$$\mu_{0a} \sim \exp(2.5)$$
$$\epsilon_a \sim \exp(0.15)$$

$$\beta_{b0} \sim \mathcal{N}(0, 2.5)$$
$$\beta_{b1} \sim \mathcal{N}(0, 2.5)$$
$$\beta_{b2} \sim \mathcal{N}(0, 2.5)$$
$$\beta_{b3} \sim S\mathcal{N}(0, 0.25, 5)$$
$$\mu_{0b} \sim \exp(2)$$

$$\epsilon_b \sim \exp(1)$$

$$\begin{aligned}
\beta_{c0} &\sim \mathcal{N}(0, 1) \\
\beta_{c1} &\sim \mathcal{N}(0, 0.5) \\
\beta_{c2} &\sim \mathcal{N}(0, 0.5) \\
\beta_{c3} &\sim S\mathcal{N}(0, 0.25, 5) \\
\mu_{0c} &\sim \exp(5) \\
\epsilon_c &\sim \exp(2.5)
\end{aligned}$$

Development and justification of these priors is described in our previous supplement (Metabolic Slope and Repeatability Analyses"). For our equation predicting evaporative heat loss responses at 40°C, priors were informed from Persson et al (2024) and were as follows:

$$\begin{aligned}
\beta_{d0} &\sim \mathcal{N}(2.5, 1) \\
\beta_{d1} &\sim \mathcal{N}(0, 0.5) \\
\beta_{d2} &\sim \mathcal{N}(0, 0.5) \\
\beta_{d3} &\sim \mathcal{N}(0, 0.025) \\
\beta_{d4} &\sim \mathcal{N}(0, 0.1) \\
\beta_{d5} &\sim \mathcal{N}(0, 0.4) \\
\mu_{0d} &\sim \exp(10) \\
\epsilon_d &\sim \exp(5)
\end{aligned}$$

Those for our equation predicting evaporative cooling efficiency were also informed by Persson et al (2024) and were as follows:

$$\begin{aligned}
\beta_{d0} &\sim \mathcal{N}(0.5, 0.2) \\
\beta_{d1} &\sim \mathcal{N}(0, 0.25) \\
\beta_{d2} &\sim \mathcal{N}(0, 0.25) \\
\beta_{d3} &\sim \mathcal{N}(0, 0.01) \\
\beta_{d4} &\sim \mathcal{N}(0, 0.02) \\
\beta_{d5} &\sim \mathcal{N}(0, 0.075) \\
\mu_{0d} &\sim \exp(15) \\
\tau_0 &\sim \mathcal{N}(-2, 1) \\
\tau_1 &\sim \mathcal{N}(0, 0.5) \\
\tau_2 &\sim \mathcal{N}(1, 1)
\end{aligned}$$

To ensure suitability of our priors, we first draw posterior predictions from prior distributions alone. These predictions are then compared against true data distributions (i.e. as a “prior predictive check”).

```
ehlModel3WeeksPPCheck <- brm(
  data = vh2o %>%
    filter(week == "3" & Ta %in% c(30, 40)) %>%
    dplyr::select(Ta, ring, pretreatment,
           mass, tarsusLengthMean, billLengthMean, ehl,
           "batch" = exp) %>%
    pivot_wider(
      id_cols = c("ring", "batch",
                  "pretreatment", "mass",
                  "tarsusLengthMean", "billLengthMean"),
      values_from = "ehl",
      names_from = "Ta"
    ) %>%
    mutate("foldEhl" = `40` / `30`) %>%
    mutate(pretreatment = ifelse(pretreatment == "neutral", "B",
      ifelse(pretreatment == "cold", "A", "C")
    )) %>%
    mutate(pretreatment = factor(pretreatment,
      levels = c("B", "A", "C")
    )) %>%
    distinct() %>%
    mutate(
      mass = mass - mean(mass, na.rm = T),
      tarsus = tarsusLengthMean - mean(tarsusLengthMean, na.rm = T),
      bill = billLengthMean - mean(billLengthMean, na.rm = T)
    ) %>%
    drop_na(),
  family = "gaussian",
  bf(mass ~ pretreatment + (1 | batch)) +
    bf(tarsus ~ mass + pretreatment + (1 | batch)) +
    bf(bill ~ mass + pretreatment + (1 | batch)) +
    bf(foldEhl ~ tarsus + mass + bill + pretreatment + (1 | batch)) +
    set_rescor(FALSE),
  prior = c(
    set_prior("normal(0, 5)",
      class = "Intercept",
      resp = "mass"
    ),
    set_prior("normal(0, 15)",
      class = "b",
      coef = "pretreatmentA",
      resp = "mass"
    ),
    set_prior("normal(0, 15)",
      class = "b",
      coef = "pretreatmentC",
      resp = "mass"
    ),
    set_prior("exponential(2.5)",
      class = "sd",
      group = "batch",
      resp = "mass"
    ),
    set_prior("exponential(0.15)",
      class = "sigma",
      resp = "mass"
    ),
    set_prior("normal(0, 2.5)",
      class = "Intercept",
      resp = "tarsus"
    ),
    set_prior("normal(0, 2.5)",
      class = "b",
      coef = "pretreatmentA",
      resp = "tarsus"
    ),
    set_prior("normal(0, 2.5)",
```

```
      class = "b",
      coef = "pretreatmentC",
      resp = "tarsus"
    ),
    set_prior("skew_normal(0, 0.25, 5)",
      class = "b",
      coef = "mass",
      resp = "tarsus"
    ),
    set_prior("exponential(2)",
      class = "sd",
      group = "batch",
      resp = "tarsus"
    ),
    set_prior("exponential(1)",
      class = "sigma",
      resp = "tarsus"
    ),
    set_prior("normal(0, 1)",
      class = "Intercept",
      resp = "bill"
    ),
    set_prior("normal(0, 0.5)",
      class = "b",
      coef = "pretreatmentA",
      resp = "bill"
    ),
    set_prior("normal(0, 0.5)",
      class = "b",
      coef = "pretreatmentC",
      resp = "bill"
    ),
    set_prior("skew_normal(0, 0.25, 5)",
      class = "b",
      coef = "mass",
      resp = "bill"
    ),
    set_prior("exponential(5)",
      class = "sd",
      group = "batch",
      resp = "bill"
    ),
    set_prior("exponential(2.5)",
      class = "sigma",
      resp = "bill"
    ),
    set_prior("normal(2.5, 1)",
      class = "Intercept",
      resp = "foldEhl"
    ),
    set_prior("normal(0, 0.5)",
      class = "b",
      coef = "pretreatmentA",
      resp = "foldEhl"
    ),
    set_prior("normal(0, 0.5)",
      class = "b",
      coef = "pretreatmentC",
      resp = "foldEhl"
    ),
    set_prior("normal(0, 0.025)",
      class = "b",
      coef = "mass",
      resp = "foldEhl"
    ),
    set_prior("normal(0, 0.1)",
      class = "b",
```

```
      coef = "tarsus",
      resp = "foldEhl"
    ),
    set_prior("normal(0, 0.4)",
      class = "b",
      coef = "bill",
      resp = "foldEhl"
    ),
    set_prior("exponential(10)",
      class = "sd",
      group = "batch",
      resp = "foldEhl"
    ),
    set_prior("exponential(5)",
      class = "sigma",
      resp = "foldEhl"
    )
  ),
  iter = 50000, warmup = 10000, cores = 4, chains = 4, thin = 20,
  control = list(adapt_delta = .98, max_treedepth = 14),
  sample_prior = "only",
  silent = TRUE, refresh = 0,
  file = "./models/_heatLossModel3WeeksPPCheck.Rds",
)

pp1 <- pp_check2(ehlModel3WeeksPPCheck, resp = "foldEhl",
                 xlab = "Evaporative Heat\nLoss Response (Fold From 30°C)") +
  theme(legend.position = "none")

efficiencyModel3WeeksPPCheck <- brm(
  data = vh2o %>%
    filter(week == "3" & Ta == 40) %>%
    dplyr::select(Ta, ring, pretreatment,
      mass, tarsusLengthMean, billLengthMean, ecc,
      "batch" = exp
    ) %>%
    mutate(
      pretreatment =
        ifelse(pretreatment == "neutral", "B",
          ifelse(pretreatment == "cold", "A", "C")
        )
    ) %>%
    mutate(pretreatment = factor(pretreatment,
      levels = c("B", "A", "C")
    )) %>%
    distinct() %>%
    mutate(
      mass = mass - mean(mass, na.rm = T),
      tarsus = tarsusLengthMean -
        mean(tarsusLengthMean, na.rm = T),
      bill = billLengthMean -
        mean(billLengthMean, na.rm = T)
    ) %>%
    drop_na(),
  family = "gaussian",
  bf(mass ~ pretreatment + (1 | batch)) +
    bf(tarsus ~ mass + pretreatment + (1 | batch)) +
    bf(bill ~ mass + pretreatment + (1 | batch)) +
    bf(
      ecc ~ mass + tarsus + bill + pretreatment + (1 | batch),
      sigma ~ batch
    ) +
    set_rescor(FALSE),
  prior = c(
    set_prior("normal(0, 5)",
      class = "Intercept",
      resp = "mass"
```

```
    ),
    set_prior("normal(0, 15)",
      class = "b",
      coef = "pretreatmentA",
      resp = "mass"
    ),
    set_prior("normal(0, 15)",
      class = "b",
      coef = "pretreatmentC",
      resp = "mass"
    ),
    set_prior("exponential(2.5)",
      class = "sd",
      group = "batch",
      resp = "mass"
    ),
    set_prior("exponential(0.15)",
      class = "sigma",
      resp = "mass"
    ),
    set_prior("normal(0, 2.5)",
      class = "Intercept",
      resp = "tarsus"
    ),
    set_prior("normal(0, 2.5)",
      class = "b",
      coef = "pretreatmentA",
      resp = "tarsus"
    ),
    set_prior("normal(0, 2.5)",
      class = "b",
      coef = "pretreatmentC",
      resp = "tarsus"
    ),
    set_prior("skew_normal(0, 0.25, 5)",
      class = "b",
      coef = "mass",
      resp = "tarsus"
    ),
    set_prior("exponential(2)",
      class = "sd",
      group = "batch",
      resp = "tarsus"
    ),
    set_prior("exponential(1)",
      class = "sigma",
      resp = "tarsus"
    ),
    set_prior("normal(0, 1)",
      class = "Intercept",
      resp = "bill"
    ),
    set_prior("normal(0, 0.5)",
      class = "b",
      coef = "pretreatmentA",
      resp = "bill"
    ),
    set_prior("normal(0, 0.5)",
      class = "b",
      coef = "pretreatmentC",
      resp = "bill"
    ),
    set_prior("skew_normal(0, 0.25, 5)",
      class = "b",
      coef = "mass",
      resp = "bill"
    ),
```

```
    set_prior("exponential(5)",
      class = "sd",
      group = "batch",
      resp = "bill"
    ),
    set_prior("exponential(2.5)",
      class = "sigma",
      resp = "bill"
    ),
    set_prior("normal(0.5, 0.2)",
      class = "Intercept",
      resp = "ecc"
    ),
    set_prior("normal(0, 0.25)",
      class = "b",
      coef = "pretreatmentA",
      resp = "ecc"
    ),
    set_prior("normal(0, 0.25)",
      class = "b",
      coef = "pretreatmentC",
      resp = "ecc"
    ),
    set_prior("normal(0, 0.01)",
      class = "b",
      coef = "mass",
      resp = "ecc"
    ),
    set_prior("normal(0, 0.02)",
      class = "b",
      coef = "tarsus",
      resp = "ecc"
    ),
    set_prior("normal(0, 0.075)",
      class = "b",
      coef = "bill",
      resp = "ecc"
    ),
    set_prior("exponential(15)",
      class = "sd",
      group = "batch",
      resp = "ecc"
    ),
    set_prior("normal(-2, 1)",
      dpar = "sigma",
      class = "Intercept",
      resp = "ecc"
    ),
    set_prior("normal(0, 0.5)",
      dpar = "sigma",
      class = "b",
      coef = "batchB",
      resp = "ecc"
    ),
    set_prior("normal(1, 1)",
      dpar = "sigma",
      class = "b",
      coef = "batchC",
      resp = "ecc"
    )
  ),
  iter = 50000, warmup = 10000, cores = 4, chains = 4, thin = 20,
  control = list(adapt_delta = .98, max_treedepth = 14),
  silent = TRUE, refresh = 0,
  sample_prior = "only",
  file = "./models/_efficiencyModel3WeeksPPCheck.Rds"
)
```

```
pp2 <- pp_check2(efficiencyModel3WeeksPPCheck, resp = "ecc",
                 xlab = "Evaporative Cooling\nEfficiency (EHL/RMR)")

pp1 + pp2 + plot_annotation(tag_levels = "A")
```

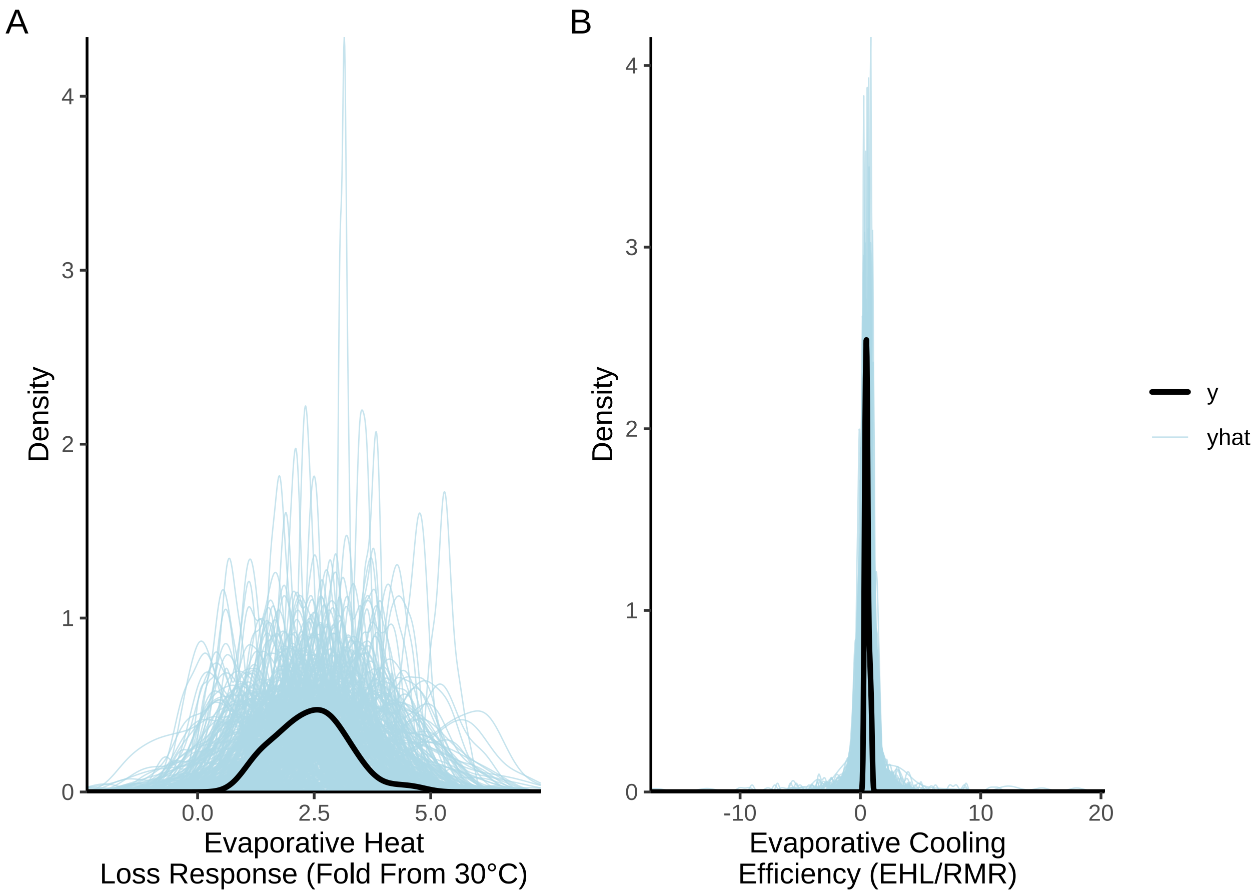

Figure 152: *Prior predictive checks for two Bayesian path analyses ultimately predicting evaporative heat loss responses ('EHL'; fold from that observed at 30°C in W) and evaporative cooling efficiency ('ECE'; the ratio of evaporative heat-loss to metabolic heat production, each in W) in three week old Japanese quail. Black lines represent true EHL and ECE densities while blue lines represent densities estimated from model priors alone.*

For both analyses, priors are unlikely to be constrainin and capture the centrality of our response variables well. We therefore proceed with constructing our complete path analyses below. After construction, we: (1) check for evidence of adequate chain mixing (i.e. a Gelman-Rubin statistic near 1), (2) check for evidence of sequentially independent chain sampling within parameters (effective sample size to sample size ratio near 1), and (3) visually compare our posterior predictions against true data distributions.

```
ehlModel3Weeks <- brm(
  data = vh2o %>%
    filter(week == "3" & Ta %in% c(30, 40)) %>%
    dplyr::select(Ta, ring, pretreatment,
           mass, tarsusLengthMean, billLengthMean, ehl,
           "batch" = exp) %>%
    pivot_wider(
      id_cols = c("ring", "batch",
                  "pretreatment", "mass",
```

```
                  "tarsusLengthMean", "billLengthMean"),
      values_from = "ehl",
      names_from = "Ta"
    ) %>%
    mutate("foldEhl" = `40` / `30`) %>%
    mutate(pretreatment = ifelse(pretreatment == "neutral", "B",
      ifelse(pretreatment == "cold", "A", "C")
    )) %>%
    mutate(pretreatment = factor(pretreatment,
      levels = c("B", "A", "C")
    )) %>%
    distinct() %>%
    mutate(
      mass = mass - mean(mass, na.rm = T),
      tarsus = tarsusLengthMean - mean(tarsusLengthMean, na.rm = T),
      bill = billLengthMean - mean(billLengthMean, na.rm = T)
    ) %>%
    drop_na(),
  family = "gaussian",
  bf(mass ~ pretreatment + (1 | batch)) +
    bf(tarsus ~ mass + pretreatment + (1 | batch)) +
    bf(bill ~ mass + pretreatment + (1 | batch)) +
    bf(foldEhl ~ tarsus + mass + bill + pretreatment + (1 | batch)) +
    set_rescor(FALSE),
  prior = c(
    set_prior("normal(0, 5)",
      class = "Intercept",
      resp = "mass"
    ),
    set_prior("normal(0, 15)",
      class = "b",
      coef = "pretreatmentA",
      resp = "mass"
    ),
    set_prior("normal(0, 15)",
      class = "b",
      coef = "pretreatmentC",
      resp = "mass"
    ),
    set_prior("exponential(2.5)",
      class = "sd",
      group = "batch",
      resp = "mass"
    ),
    set_prior("exponential(0.15)",
      class = "sigma",
      resp = "mass"
    ),
    set_prior("normal(0, 2.5)",
      class = "Intercept",
      resp = "tarsus"
    ),
    set_prior("normal(0, 2.5)",
      class = "b",
      coef = "pretreatmentA",
      resp = "tarsus"
    ),
    set_prior("normal(0, 2.5)",
      class = "b",
      coef = "pretreatmentC",
      resp = "tarsus"
    ),
    set_prior("skew_normal(0, 0.25, 5)",
      class = "b",
      coef = "mass",
      resp = "tarsus"
    ),
```

```
    set_prior("exponential(2)",
      class = "sd",
      group = "batch",
      resp = "tarsus"
    ),
    set_prior("exponential(1)",
      class = "sigma",
      resp = "tarsus"
    ),
    set_prior("normal(0, 1)",
      class = "Intercept",
      resp = "bill"
    ),
    set_prior("normal(0, 0.5)",
      class = "b",
      coef = "pretreatmentA",
      resp = "bill"
    ),
    set_prior("normal(0, 0.5)",
      class = "b",
      coef = "pretreatmentC",
      resp = "bill"
    ),
    set_prior("skew_normal(0, 0.25, 5)",
      class = "b",
      coef = "mass",
      resp = "bill"
    ),
    set_prior("exponential(5)",
      class = "sd",
      group = "batch",
      resp = "bill"
    ),
    set_prior("exponential(2.5)",
      class = "sigma",
      resp = "bill"
    ),
    set_prior("normal(2.5, 1)",
      class = "Intercept",
      resp = "foldEhl"
    ),
    set_prior("normal(0, 0.5)",
      class = "b",
      coef = "pretreatmentA",
      resp = "foldEhl"
    ),
    set_prior("normal(0, 0.5)",
      class = "b",
      coef = "pretreatmentC",
      resp = "foldEhl"
    ),
    set_prior("normal(0, 0.025)",
      class = "b",
      coef = "mass",
      resp = "foldEhl"
    ),
    set_prior("normal(0, 0.1)",
      class = "b",
      coef = "tarsus",
      resp = "foldEhl"
    ),
    set_prior("normal(0, 0.4)",
      class = "b",
      coef = "bill",
      resp = "foldEhl"
    ),
    set_prior("exponential(10)",
```

```
      class = "sd",
      group = "batch",
      resp = "foldEhl"
    ),
    set_prior("exponential(5)",
      class = "sigma",
      resp = "foldEhl"
    )
  ),
  iter = 50000, warmup = 10000, cores = 4, chains = 4, thin = 20,
  control = list(adapt_delta = .98, max_treedepth = 14),
  silent = TRUE, refresh = 0,
  file = "./models/_heatLossModel3Weeks.Rds",
)

pp1a <- mcmc_neff(neff_ratio(ehlModel3Weeks)) +
  xlab(
    TeX("$\\overset{Evaporative~Heat~Loss}{Response~(N_{eff}/N)}$")
  ) +
  theme_classic() +
  theme(
    axis.text.y = element_blank(),
    axis.ticks.y = element_blank(),
    legend.position = "none"
  )

pp1b <- mcmc_rhat(rhat(ehlModel3Weeks)) +
  xlab(
    TeX("$\\overset{Evaporative~Heat~Loss}{Response~(\\hat{R})}$")
  ) +
  theme_classic() +
  theme(
    axis.text.y = element_blank(),
    axis.ticks.y = element_blank(),
    legend.position = "none"
  )

pp1c <- pp_check2(ehlModel3Weeks,
  resp = "foldEhl",
  xlab = "Evaporative Heat\nLoss Response (Fold From 30°C)"
) +
  theme(legend.position = "none")

pp1d <- ehlModel3Weeks$data %>%
  mutate(
    "Fit" = fitted(ehlModel3Weeks, resp = "foldEhl")[, "Estimate"],
    "FitSE" = fitted(ehlModel3Weeks, resp = "foldEhl")[, "Est.Error"]
  ) %>%
  ggplot(aes(x = Fit, y = foldEhl)) +
  geom_errorbarh(aes(xmin = Fit - FitSE, xmax = Fit + FitSE),
    height = 0.25, colour = "black", alpha = 0.8
  ) +
  geom_point(
    size = 2, pch = 21, colour = "black", fill = "lightblue2",
    alpha = 0.8
  ) +
  geom_smooth(
    method = "lm", colour = "black", formula = y ~ 0 + x,
    linetype = "dashed", se = FALSE
  ) +
  xlab("Fitted Evaporative Heat\nLoss (Fold from 30°C)") +
  ylab("Evaporative Heat Loss\n(Fold from 30°C)") +
  theme_classic()

efficiencyModel3Weeks <- brm(
  data = vh2o %>%
    filter(week == "3" & Ta == 40) %>%
```

```
    dplyr::select(Ta, ring, pretreatment,
      mass, tarsusLengthMean, billLengthMean, ecc,
      "batch" = exp
    ) %>%
    mutate(
      pretreatment =
        ifelse(pretreatment == "neutral", "B",
          ifelse(pretreatment == "cold", "A", "C")
        )
    ) %>%
    mutate(pretreatment = factor(pretreatment,
      levels = c("B", "A", "C")
    )) %>%
    distinct() %>%
    mutate(
      mass = mass - mean(mass, na.rm = T),
      tarsus = tarsusLengthMean -
        mean(tarsusLengthMean, na.rm = T),
      bill = billLengthMean -
        mean(billLengthMean, na.rm = T)
    ) %>%
    drop_na(),
  family = "gaussian",
  bf(mass ~ pretreatment + (1 | batch)) +
    bf(tarsus ~ mass + pretreatment + (1 | batch)) +
    bf(bill ~ mass + pretreatment + (1 | batch)) +
    bf(
      ecc ~ mass + tarsus + bill + pretreatment + (1 | batch),
      sigma ~ batch
    ) +
    set_rescor(FALSE),
  prior = c(
    set_prior("normal(0, 5)",
      class = "Intercept",
      resp = "mass"
    ),
    set_prior("normal(0, 15)",
      class = "b",
      coef = "pretreatmentA",
      resp = "mass"
    ),
    set_prior("normal(0, 15)",
      class = "b",
      coef = "pretreatmentC",
      resp = "mass"
    ),
    set_prior("exponential(2.5)",
      class = "sd",
      group = "batch",
      resp = "mass"
    ),
    set_prior("exponential(0.15)",
      class = "sigma",
      resp = "mass"
    ),
    set_prior("normal(0, 2.5)",
      class = "Intercept",
      resp = "tarsus"
    ),
    set_prior("normal(0, 2.5)",
      class = "b",
      coef = "pretreatmentA",
      resp = "tarsus"
    ),
    set_prior("normal(0, 2.5)",
      class = "b",
      coef = "pretreatmentC",
```

```
    resp = "tarsus"
  ),
  set_prior("skew_normal(0, 0.25, 5)",
    class = "b",
    coef = "mass",
    resp = "tarsus"
  ),
  set_prior("exponential(2)",
    class = "sd",
    group = "batch",
    resp = "tarsus"
  ),
  set_prior("exponential(1)",
    class = "sigma",
    resp = "tarsus"
  ),
  set_prior("normal(0, 1)",
    class = "Intercept",
    resp = "bill"
  ),
  set_prior("normal(0, 0.5)",
    class = "b",
    coef = "pretreatmentA",
    resp = "bill"
  ),
  set_prior("normal(0, 0.5)",
    class = "b",
    coef = "pretreatmentC",
    resp = "bill"
  ),
  set_prior("skew_normal(0, 0.25, 5)",
    class = "b",
    coef = "mass",
    resp = "bill"
  ),
  set_prior("exponential(5)",
    class = "sd",
    group = "batch",
    resp = "bill"
  ),
  set_prior("exponential(2.5)",
    class = "sigma",
    resp = "bill"
  ),
  set_prior("normal(0.5, 0.2)",
    class = "Intercept",
    resp = "ecc"
  ),
  set_prior("normal(0, 0.25)",
    class = "b",
    coef = "pretreatmentA",
    resp = "ecc"
  ),
  set_prior("normal(0, 0.25)",
    class = "b",
    coef = "pretreatmentC",
    resp = "ecc"
  ),
  set_prior("normal(0, 0.01)",
    class = "b",
    coef = "mass",
    resp = "ecc"
  ),
  set_prior("normal(0, 0.02)",
    class = "b",
    coef = "tarsus",
    resp = "ecc"
```

```
    ),
    set_prior("normal(0, 0.075)",
      class = "b",
      coef = "bill",
      resp = "ecc"
    ),
    set_prior("exponential(15)",
      class = "sd",
      group = "batch",
      resp = "ecc"
    ),
    set_prior("normal(-2, 1)",
      dpar = "sigma",
      class = "Intercept",
      resp = "ecc"
    ),
    set_prior("normal(0, 0.5)",
      dpar = "sigma",
      class = "b",
      coef = "batchB",
      resp = "ecc"
    ),
    set_prior("normal(1, 1)",
      dpar = "sigma",
      class = "b",
      coef = "batchC",
      resp = "ecc"
    )
  ),
  iter = 50000, warmup = 10000, cores = 4, chains = 4, thin = 20,
  control = list(adapt_delta = .98, max_treedepth = 14),
  silent = TRUE, refresh = 0,
  file = "./models/_efficiencyModel3Weeks.Rds"
)

pp2a <- mcmc_neff(neff_ratio(efficiencyModel3Weeks)) +
  xlab(
    TeX("$\\overset{Evaporative~Cooling}{Efficiency~N_{eff}/N}$")
  ) +
  theme_classic() +
  theme(
    axis.text.y = element_blank(),
    axis.ticks.y = element_blank(),
    legend.position = "none"
  )

pp2b <- mcmc_rhat(rhat(efficiencyModel3Weeks)) +
  xlab(
    TeX("$\\overset{Evaporative~Cooling~Efficiency}{\\hat{R}}$")
  ) +
  theme_classic() +
  theme(
    axis.text.y = element_blank(),
    axis.ticks.y = element_blank(),
    legend.position = "none"
  )

pp2c <- pp_check2(efficiencyModel3Weeks,
  resp = "ecc",
  xlab = "Evaporative Cooling\nEfficiency (EHL/RMR)"
) + scale_x_continuous(n.breaks = 3)

pp2d <- efficiencyModel3Weeks$data %>%
  mutate(
    "Fit" = fitted(efficiencyModel3Weeks,
      resp = "ecc"
    )[, "Estimate"],
```

```
    "FitSE" = fitted(efficiencyModel3Weeks,
      resp = "ecc"
    )[, "Est.Error"]
  ) %>%
  ggplot(aes(x = Fit, y = ecc)) +
  geom_errorbarh(aes(xmin = Fit - FitSE, xmax = Fit + FitSE),
    height = 0.05, colour = "black", alpha = 0.8
  ) +
  geom_point(
    size = 2, pch = 21, colour = "black", fill = "lightblue2",
    alpha = 0.8
  ) +
  geom_smooth(
    method = "lm", colour = "black", formula = y ~ 0 + x,
    linetype = "dashed", se = FALSE
  ) +
  xlab("Fitted Evaporative\nCooling Efficiency (EHL/RMR)") +
  ylab("Evaporative Cooling\nEfficiency (EHL/RMR)") +
  theme_classic()

((pp1a + pp1b) /
  (pp1c + pp1d) /
  (pp2a + pp2b) /
  (pp2c + pp2d)) +
  plot_annotation(tag_levels = "A")
```

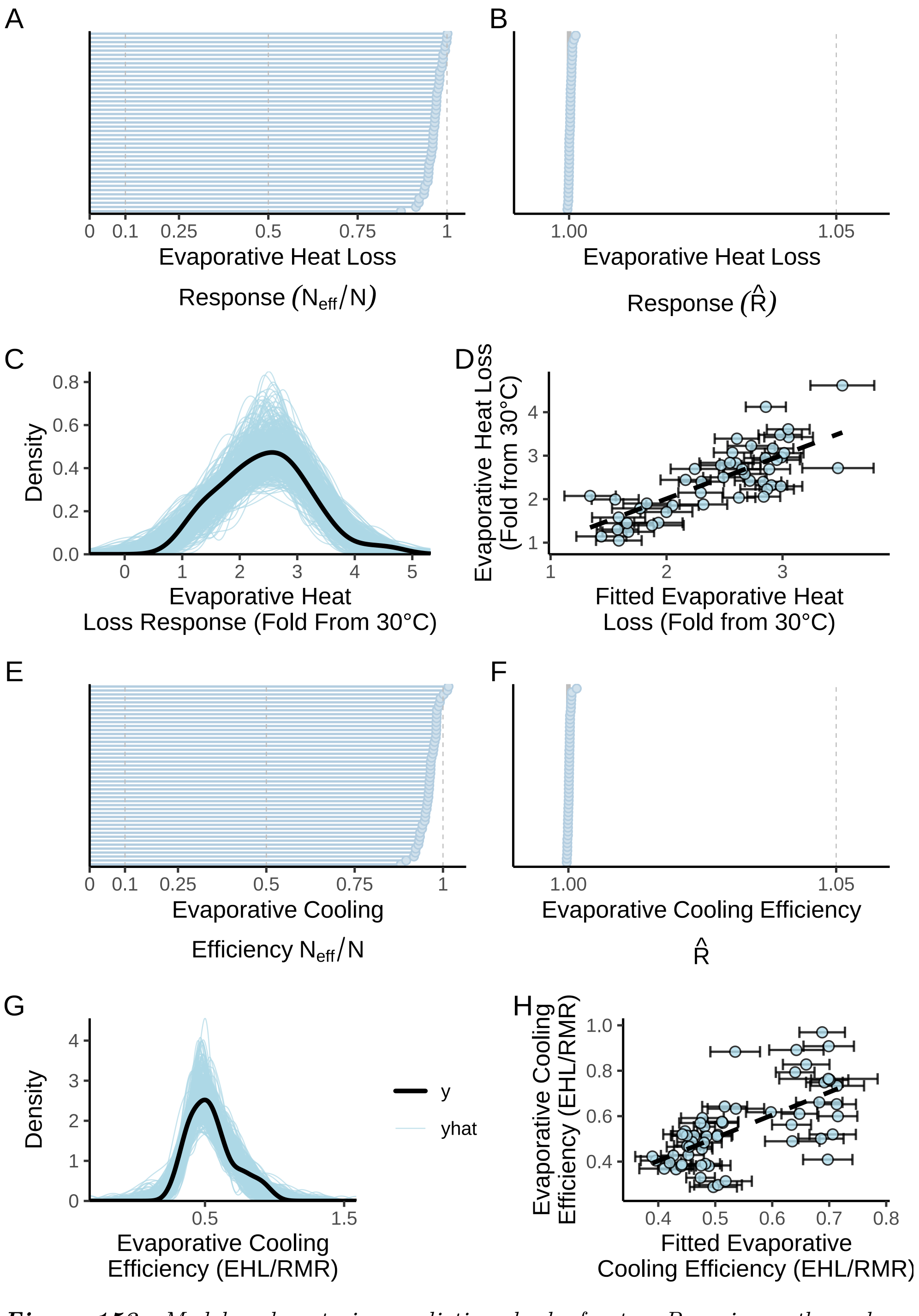

Figure 153: *Model and posterior predictive checks for two Bayesian path analyses predicting, ultimately, evaporative heat loss ('EHL'; fold from that observed at 30°C in W) and evaporative cooling efficiency ('ECE'; the ratio of evaporative heat-loss to metabolic heat production, each in W) in three week old Japanese quail. Panels A and E display parameter-specific effective sample size to sample size ratios (N_{eff}/N) for each model, while panels B and F display parameter specific Gelman-Rubin statistics ($\hat{R}$). Panels C and G display posterior predictive checks; black lines represent true EHL or ECE densities while blue lines represent densities estimated from model posteriors. In panels D and H, dots represent individual predictions plotted against their true values. Errorbars indicate +/- one standard error around predictions and dashed black lines indicate lines of best fit estimated by ggplot2 (Wickham, 2011) while assuming a y-intercept at 0.*

Next, we visualise the spread of model residuals (here, as medians) against model predictors and expectations. After, we check posterior densities per model predictor for evidence of skewing or kurtosis.

```
rp1 <- ehlModel3Weeks$data %>%
  mutate(
    "Res" =
      residuals(ehlModel3Weeks,
        resp = "foldEhl",
        robust = TRUE
      )[, "Estimate"]
  ) %>%
  ggplot(aes(sample = Res)) +
  stat_qq(colour = "grey50") +
  stat_qq_line() +
  xlab("Theoretical EHL\nResidual Quantiles") +
  ylab("Sample ELH\nResidual Quantiles") +
  theme_classic()

rp2 <- ehlModel3Weeks$data %>%
  mutate(
    "Res" =
      residuals(ehlModel3Weeks,
        resp = "foldEhl",
        robust = TRUE
      )[, "Estimate"]
  ) %>%
  mutate(mass = mass + mean(subset(vh2o, week == "3")$mass, na.rm = T)) %>%
  ggplot(aes(x = mass, y = Res)) +
  geom_point(pch = 21, colour = "black", fill = "grey20", alpha = 0.5) +
  xlab("Body Mass (g)") +
  ylab("Evaporative Heat\nLoss Residuals") +
  theme_classic()

rp3 <- ehlModel3Weeks$data %>%
  mutate(
    "Res" =
      residuals(ehlModel3Weeks,
        resp = "foldEhl",
        robust = TRUE
      )[, "Estimate"]
  ) %>%
  mutate(tarsus = tarsus +
    mean(subset(vh2o, week == "3")$tarsusLengthMean, na.rm = T)) %>%
  ggplot(aes(x = tarsus, y = Res)) +
  geom_point(pch = 21, colour = "black", fill = "grey20", alpha = 0.5) +
  xlab("Tarsus Length (mm)") +
  ylab("Evaporative Heat\nLoss Residuals") +
  theme_classic()

rp4 <- ehlModel3Weeks$data %>%
  mutate(
    "Res" =
      residuals(ehlModel3Weeks,
        resp = "foldEhl",
        robust = TRUE
      )[, "Estimate"]
  ) %>%
  mutate(bill = bill +
    mean(subset(vh2o, week == "3")$billLengthMean, na.rm = T)) %>%
  ggplot(aes(x = tarsus, y = Res)) +
  geom_point(pch = 21, colour = "black", fill = "grey20", alpha = 0.5) +
  xlab("Bill Length (mm)") +
  ylab("Evaporative Heat\nLoss Residuals") +
  theme_classic()

rp5 <- ehlModel3Weeks$data %>%
  mutate(
```

```
    "Res" =
      residuals(ehlModel3Weeks,
        resp = "foldEhl",
        robust = TRUE
      )[, "Estimate"]
  ) %>%
  mutate(pretreatment = factor(pretreatment, levels = c("A", "B", "C"))) %>%
  ggplot(aes(x = pretreatment, y = Res)) +
  geom_boxplot(fill = "lightblue2") +
  geom_point(size = 2, position = position_jitter(width = 0.25)) +
  scale_x_discrete(
    name = "Rearing Treatment",
    labels = c(
      "Cold\n(10°C)",
      "Mild\n(20°C)",
      "Warm\n(30°C)"
    )
  ) +
  xlab("Rearing Treatment") +
  ylab("Evaporative Heat\nLoss Residuals") +
  theme_classic()

rp6 <- efficiencyModel3Weeks$data %>%
  mutate(
    "Res" =
      residuals(efficiencyModel3Weeks,
        resp = "ecc",
        robust = TRUE
      )[, "Estimate"]
  ) %>%
  ggplot(aes(sample = Res)) +
  stat_qq(colour = "grey50") +
  stat_qq_line() +
  xlab("Theoretical ECE\nResidual Quantiles") +
  ylab("Sample ECE\nResidual Quantiles") +
  theme_classic()

rp7 <- efficiencyModel3Weeks$data %>%
  mutate(
    "Res" =
      residuals(efficiencyModel3Weeks,
        resp = "ecc",
        robust = TRUE
      )[, "Estimate"]
  ) %>%
  mutate(mass = mass + mean(subset(vh2o, week == "3")$mass, na.rm = T)) %>%
  ggplot(aes(x = mass, y = Res)) +
  geom_point(pch = 21, colour = "black", fill = "grey20", alpha = 0.5) +
  xlab("Body Mass (g)") +
  ylab("Evaporative Cooling\nEfficiency Residuals") +
  theme_classic()

rp8 <- efficiencyModel3Weeks$data %>%
  mutate(
    "Res" =
      residuals(efficiencyModel3Weeks,
        resp = "ecc",
        robust = TRUE
      )[, "Estimate"]
  ) %>%
  mutate(tarsus = tarsus +
    mean(subset(vh2o, week == "3")$tarsusLengthMean, na.rm = T)) %>%
  ggplot(aes(x = tarsus, y = Res)) +
  geom_point(pch = 21, colour = "black", fill = "grey20", alpha = 0.5) +
  xlab("Tarsus Length (mm)") +
  ylab("Evaporative Cooling\nEfficiency Residuals") +
  theme_classic()
```

```
rp9 <- efficiencyModel3Weeks$data %>%
  mutate(
    "Res" =
      residuals(efficiencyModel3Weeks,
        resp = "ecc",
        robust = TRUE
      )[, "Estimate"]
  ) %>%
  mutate(bill = bill +
    mean(subset(vh2o, week == "3")$billLengthMean, na.rm = T)) %>%
  ggplot(aes(x = tarsus, y = Res)) +
  geom_point(pch = 21, colour = "black", fill = "grey20", alpha = 0.5) +
  xlab("Bill Length (mm)") +
  ylab("Evaporative Cooling\nEfficiency Residuals") +
  theme_classic()

rp10 <- efficiencyModel3Weeks$data %>%
  mutate(
    "Res" =
      residuals(efficiencyModel3Weeks,
        resp = "ecc",
        robust = TRUE
      )[, "Estimate"]
  ) %>%
  mutate(pretreatment = factor(pretreatment, levels = c("A", "B", "C"))) %>%
  ggplot(aes(x = pretreatment, y = Res)) +
  geom_boxplot(fill = "lightblue2") +
  geom_point(size = 2, position = position_jitter(width = 0.25)) +
  scale_x_discrete(
    name = "Rearing Treatment",
    labels = c(
      "Cold\n(10°C)",
      "Mild\n(20°C)",
      "Warm\n(30°C)"
    )
  ) +
  xlab("Rearing Treatment") +
  ylab("Evaporative Cooling\nEfficiency Residuals") +
  theme_classic()

((rp1 + rp6 + rp2)/
  (rp7 + rp3 + rp8)/
  (rp4 + rp9 + rp5)/
    ((rp10) + plot_spacer() + plot_spacer())
) + plot_annotation(tag_levels = "A")
```

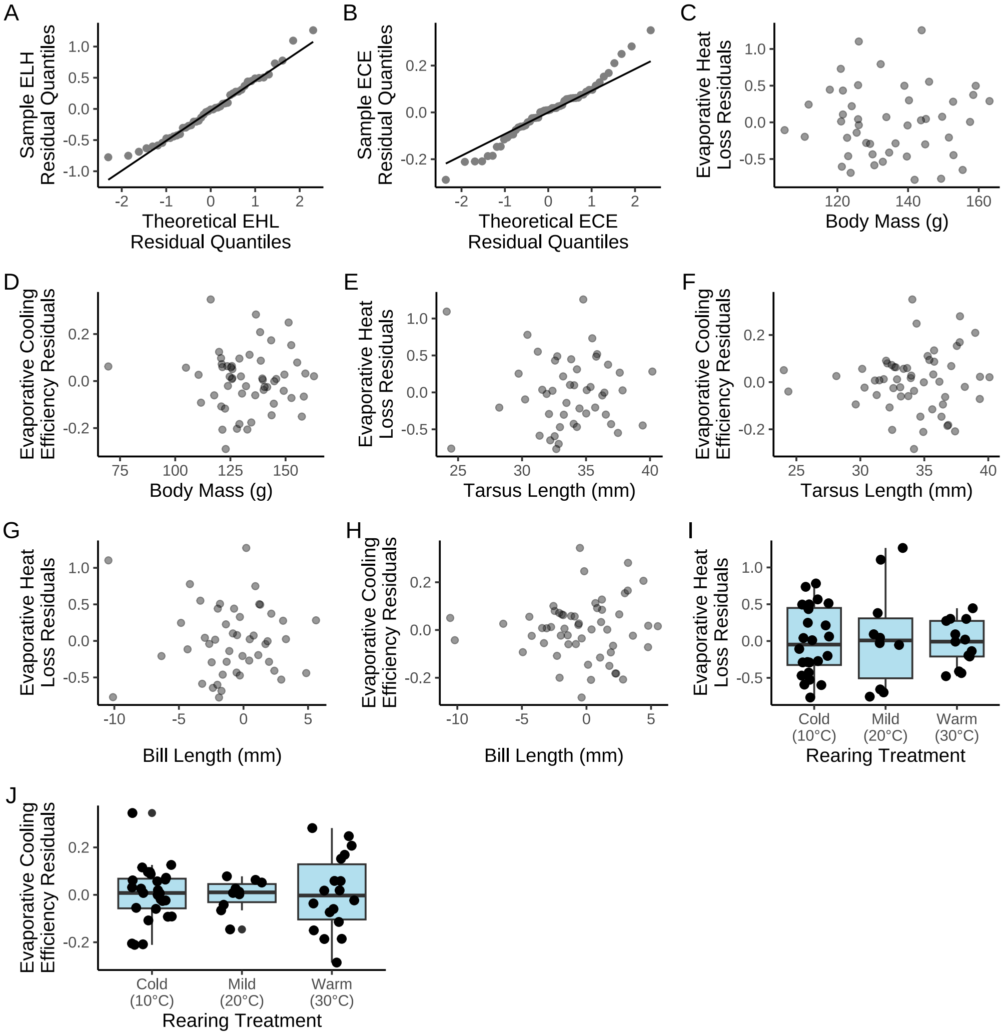

Figure 154: *Residual diagnostics from two Bayesian path analyses predicting, ultimately, evaporative heat loss ('EHL'; fold from that observed at 30°C in W) and evaporative cooling efficiency ('ECE'; the ratio of evaporative heat-loss to metabolic heat production, each in W) in three week old Japanese quail. Panels A and B display traditional 'qq-plots', with theoretic and sample residual quantiles regressed against each other. Dots represent individual samples. Remaining panels display median residual, per raw data point (small dots) by model predictors (here, body mass [g], tarsus length [mm], bill length [mm] and rearing condition (10°C, 20°C, or 30°C until the time of measurement). Boxplots in panels I and J display medians (centre horizontal bar), first and third quantiles (lower and upper limits of boxes respectively) and ranges excluding outliers (whiskers). 'EHL' indicates evaporative heat loss in watts, and ECE indicates evaporative cooling efficiency (the ratio of evaporative heat loss by metabolic heat production, each in watts).*

Residual spreads are largely homogenous and normal. However, one individual with an extremely low mass is evident in our efficiency analysis but not our relative heat loss analysis. This individual is therefore removed from our efficiency analysis and our analysis re-run.

```
efficiencyModel3Weeks <- brm(
  data = vh2o %>%
    filter(week == "3" & Ta == 40) %>%
    dplyr::select(Ta, ring, pretreatment,
      mass, tarsusLengthMean, billLengthMean, ecc,
      "batch" = exp
```

```
  ) %>%
  filter(mass > 75) %>%
  mutate(
    pretreatment =
      ifelse(pretreatment == "neutral", "B",
        ifelse(pretreatment == "cold", "A", "C")
      )
  ) %>%
  mutate(pretreatment = factor(pretreatment,
    levels = c("B", "A", "C")
  )) %>%
  distinct() %>%
  mutate(
    mass = mass - mean(mass, na.rm = T),
    tarsus = tarsusLengthMean -
      mean(tarsusLengthMean, na.rm = T),
    bill = billLengthMean -
      mean(billLengthMean, na.rm = T)
  ) %>%
  drop_na(),
family = "gaussian",
bf(mass ~ pretreatment + (1 | batch)) +
  bf(tarsus ~ mass + pretreatment + (1 | batch)) +
  bf(bill ~ mass + pretreatment + (1 | batch)) +
  bf(
    ecc ~ mass + tarsus + bill + pretreatment + (1 | batch),
    sigma ~ batch
  ) +
  set_rescor(FALSE),
prior = c(
  set_prior("normal(0, 5)",
    class = "Intercept",
    resp = "mass"
  ),
  set_prior("normal(0, 15)",
    class = "b",
    coef = "pretreatmentA",
    resp = "mass"
  ),
  set_prior("normal(0, 15)",
    class = "b",
    coef = "pretreatmentC",
    resp = "mass"
  ),
  set_prior("exponential(2.5)",
    class = "sd",
    group = "batch",
    resp = "mass"
  ),
  set_prior("exponential(0.15)",
    class = "sigma",
    resp = "mass"
  ),
  set_prior("normal(0, 2.5)",
    class = "Intercept",
    resp = "tarsus"
  ),
  set_prior("normal(0, 2.5)",
    class = "b",
    coef = "pretreatmentA",
    resp = "tarsus"
  ),
  set_prior("normal(0, 2.5)",
    class = "b",
    coef = "pretreatmentC",
    resp = "tarsus"
  ),
```

```
  set_prior("skew_normal(0, 0.25, 5)",
    class = "b",
    coef = "mass",
    resp = "tarsus"
  ),
  set_prior("exponential(2)",
    class = "sd",
    group = "batch",
    resp = "tarsus"
  ),
  set_prior("exponential(1)",
    class = "sigma",
    resp = "tarsus"
  ),
  set_prior("normal(0, 1)",
    class = "Intercept",
    resp = "bill"
  ),
  set_prior("normal(0, 0.5)",
    class = "b",
    coef = "pretreatmentA",
    resp = "bill"
  ),
  set_prior("normal(0, 0.5)",
    class = "b",
    coef = "pretreatmentC",
    resp = "bill"
  ),
  set_prior("skew_normal(0, 0.25, 5)",
    class = "b",
    coef = "mass",
    resp = "bill"
  ),
  set_prior("exponential(5)",
    class = "sd",
    group = "batch",
    resp = "bill"
  ),
  set_prior("exponential(2.5)",
    class = "sigma",
    resp = "bill"
  ),
  set_prior("normal(0.5, 0.2)",
    class = "Intercept",
    resp = "ecc"
  ),
  set_prior("normal(0, 0.25)",
    class = "b",
    coef = "pretreatmentA",
    resp = "ecc"
  ),
  set_prior("normal(0, 0.25)",
    class = "b",
    coef = "pretreatmentC",
    resp = "ecc"
  ),
  set_prior("normal(0, 0.01)",
    class = "b",
    coef = "mass",
    resp = "ecc"
  ),
  set_prior("normal(0, 0.02)",
    class = "b",
    coef = "tarsus",
    resp = "ecc"
  ),
  set_prior("normal(0, 0.075)",
```

```
      class = "b",
      coef = "bill",
      resp = "ecc"
    ),
    set_prior("exponential(15)",
      class = "sd",
      group = "batch",
      resp = "ecc"
    ),
    set_prior("normal(-2, 1)",
      dpar = "sigma",
      class = "Intercept",
      resp = "ecc"
    ),
    set_prior("normal(0, 0.5)",
      dpar = "sigma",
      class = "b",
      coef = "batchB",
      resp = "ecc"
    ),
    set_prior("normal(1, 1)",
      dpar = "sigma",
      class = "b",
      coef = "batchC",
      resp = "ecc"
    )
  ),
  iter = 50000, warmup = 10000, cores = 4, chains = 4, thin = 20,
  control = list(adapt_delta = .98, max_treedepth = 14),
  silent = TRUE, refresh = 0,
  file = "./models/_efficiencyModel3WeeksRevised.Rds"
)

ppa <- mcmc_neff(neff_ratio(efficiencyModel3Weeks)) +
  xlab(
    TeX("$\\overset{Evaporative~Cooling}{Efficiency~N_{eff}/N}$")
  ) +
  theme_classic() +
  theme(
    axis.text.y = element_blank(),
    axis.ticks.y = element_blank(),
    legend.position = "none"
  )

ppb <- mcmc_rhat(rhat(efficiencyModel3Weeks)) +
  xlab(
    TeX("$\\overset{Evaporative~Cooling~Efficiency}{\\hat{R}}$")
  ) +
  theme_classic() +
  theme(
    axis.text.y = element_blank(),
    axis.ticks.y = element_blank(),
    legend.position = "none"
  )

ppc <- pp_check2(efficiencyModel3Weeks,
  resp = "ecc",
  xlab = "Evaporative Cooling\nEfficiency (EHL/RMR)"
) + scale_x_continuous(n.breaks = 3)

(ppa + ppb)/ppc + plot_annotation(tag_levels = "A")
```

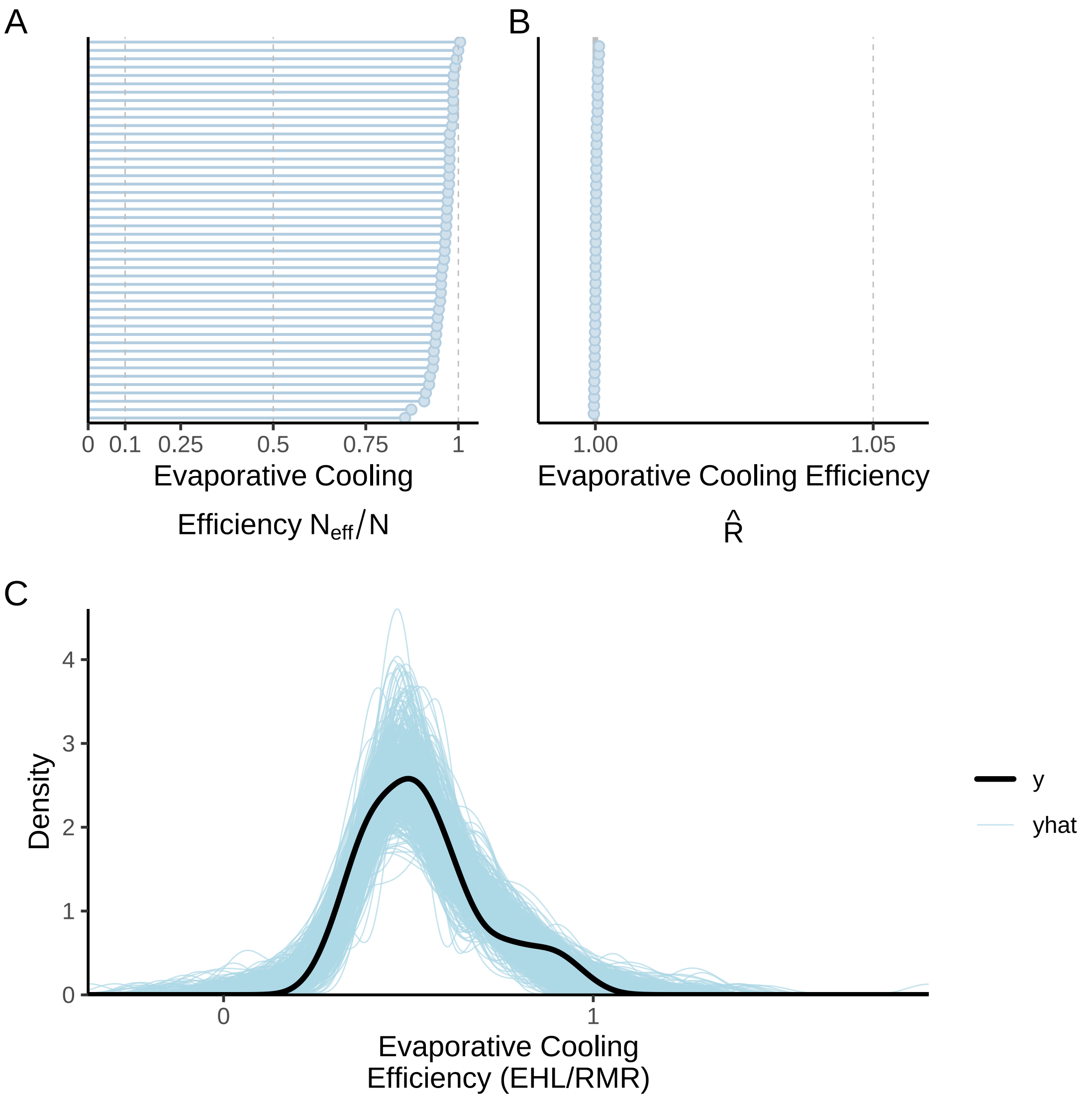

Figure 155: *Model and posterior predictive checks for a Bayesian path analysis predicting evaporative cooling efficiency ('ECE'; the ratio of evaporative heat-loss to metabolic heat production, each in W) in three week old Japanese quail. Panel A displays parameter-specific effective sample size to sample size ratios (N_{eff}/N), panel B displays parameter specific Gelman-Rubin statistics ($\hat{R}$), and panel panels C displays a posterior predictive check; black lines represent true ECE densities while blue lines represent densities estimated from model posteriors.*

Below, we proceed to plotting model coefficients.

```
as.data.frame(ehlModel3Weeks) %>%
  pivot_longer(everything(), names_to = "par", values_to = "values") %>%
  filter(grepl("b_|sd_", par)) %>%
  merge(., tribble(
    ~par, ~Par, ~Order,
    "b_mass_Intercept", "Mass\nIntercept", "A",
    "b_mass_pretreatmentA", "Mass ~\nCold Rearing", "B",
    "b_mass_pretreatmentC", "Mass ~\nWarm Rearing", "C",
    "b_tarsus_Intercept", "Tarsus\nIntercept", "D",
    "b_tarsus_pretreatmentA", "Tarsus ~\nCold Rearing", "E",
    "b_tarsus_pretreatmentC", "Tarsus ~\nWarm Rearing", "F",
```

```
    "b_tarsus_mass", "Tarsus ~\nMass", "G",
    "sd_batch__tarsus_Intercept", "Tarsus ~\nBatch", "H",
    "b_bill_Intercept", "Bill\nIntercept", "I",
    "b_bill_pretreatmentA", "Bill ~\nCold Rearing", "J",
    "b_bill_pretreatmentC", "Bill ~\nWarm Rearing", "K",
    "b_bill_mass", "Bill ~\nMass", "K",
    "sd_batch__bill_Intercept", "Bill ~\nBatch", "M",
    "b_foldEhl_Intercept", "EHL\nIntercept", "N",
    "b_foldEhl_pretreatmentA", "EHL ~\nCold Rearing", "O",
    "b_foldEhl_pretreatmentC", "EHL ~\nWarm Rearing", "P",
    "b_foldEhl_mass", "EHL ~ Mass", "Q",
    "b_foldEhl_tarsus", "EHL ~\nTarsus", "R",
    "b_foldEhl_tarsus", "EHL ~\nBill", "S",
    "sd_batch__foldEhl_Intercept", "EHL ~\nBatch", "T"
  ),
  by = "par", all.x = TRUE
  ) %>%
  drop_na() %>%
  arrange(Order) %>%
  mutate(Par = factor(Par, levels = unique(Par))) %>%
  ggplot(aes(x = values)) +
  facet_wrap(~Par, scales = "free") +
  geom_density(colour = "black", fill = "white") +
  geom_vline(xintercept = 0, colour = "darkred", linetype = "solid") +
  scale_x_continuous(n.breaks = 3) +
  scale_y_continuous(n.breaks = 3) +
  xlab("Values") +
  ylab("Density") +
  theme_classic()
```

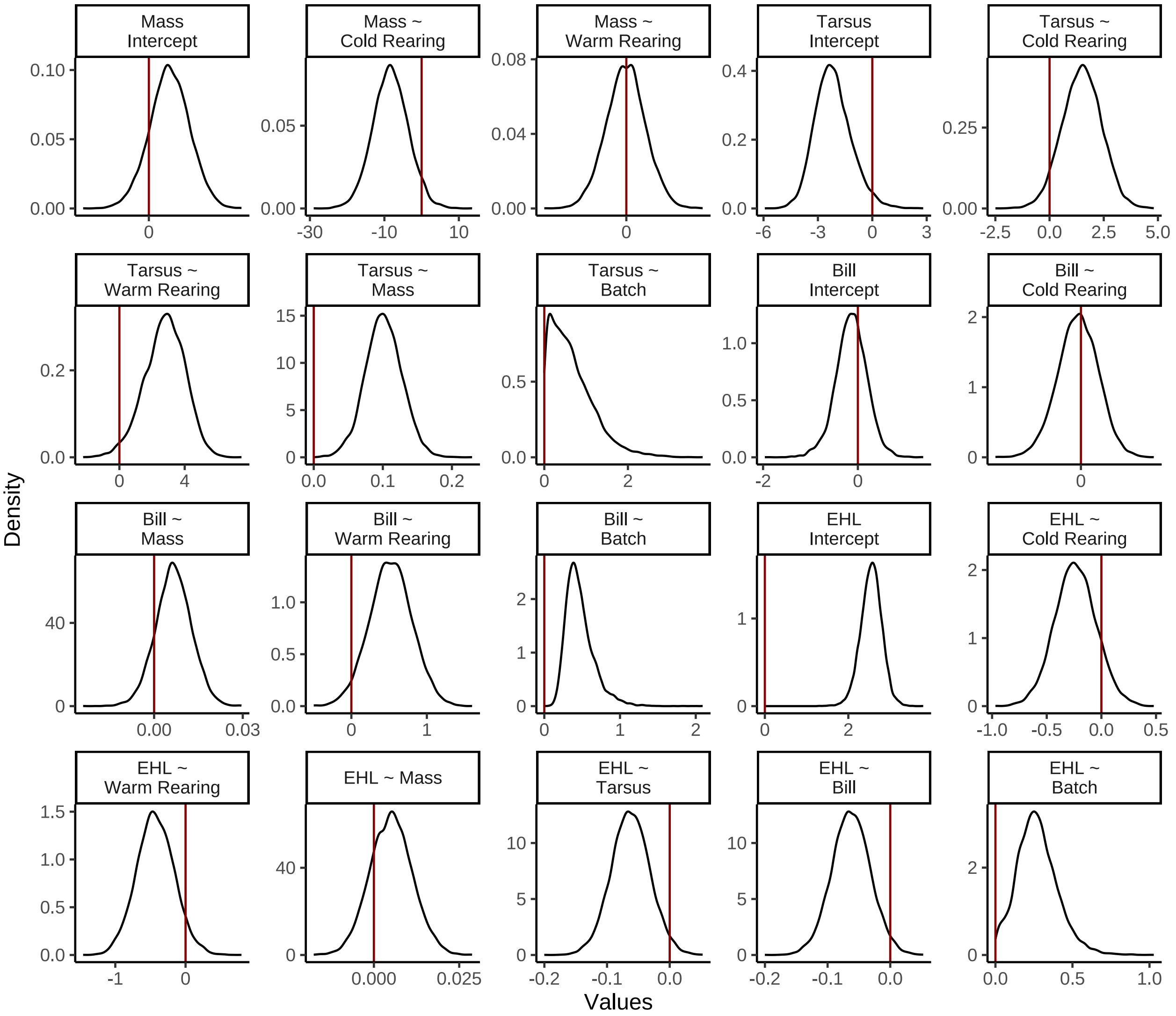

Figure 156: *Posterior densities for coefficients from a Bayesian path analysis predicting body mass (g), tarsus length (mm), bill length (mm) and evaporative heat loss responses ("EHL"; fold from that observed at 30°C in W) in three week old Japanese quail. Red vertical lines label 0. Response and predictor variables for which densities refer are indicated on the left side and right side of tildes respectively. "Cold Rearing" indicates rearing at 10°C, "Warm Rearing" indicates rearing at 30°C, and "Batch" indicates the batch of eggs from which an individual was derived. All coefficients except batch are population level.*

```
as.data.frame(efficiencyModel3Weeks) %>%
  pivot_longer(everything(), names_to = "par", values_to = "values") %>%
  filter(grepl("b_|sd_", par)) %>%
  merge(., tribble(
    ~par, ~Par, ~Order,
    "b_mass_Intercept", "Mass\nIntercept", "A",
    "b_mass_pretreatmentA", "Mass ~\nCold Rearing", "B",
    "b_mass_pretreatmentC", "Mass ~\nWarm Rearing", "C",
    "b_tarsus_Intercept", "Tarsus\nIntercept", "D",
    "b_tarsus_pretreatmentA", "Tarsus ~\nCold Rearing", "E",
    "b_tarsus_pretreatmentC", "Tarsus ~\nWarm Rearing", "F",
    "b_tarsus_mass", "Tarsus ~\nMass", "G",
    "sd_batch__tarsus_Intercept", "Tarsus ~\nBatch", "H",
    "b_bill_Intercept", "Bill\nIntercept", "I",
    "b_bill_pretreatmentA", "Bill ~\nCold Rearing", "J",
```

```
    "b_bill_pretreatmentC", "Bill ~\nWarm Rearing", "K",
    "b_bill_mass", "Bill ~\nMass", "K",
    "sd_batch__bill_Intercept", "Bill ~\nBatch", "M",
    "b_ecc_Intercept", "ECE\nIntercept", "N",
    "b_ecc_pretreatmentA", "ECE ~\nCold Rearing", "O",
    "b_ecc_pretreatmentC", "ECE ~\nWarm Rearing", "P",
    "b_ecc_mass", "ECE ~ Mass", "Q",
    "b_ecc_tarsus", "ECE ~\nTarsus", "R",
    "b_ecc_tarsus", "ECE ~\nBill", "S",
    "sd_batch__ecc_Intercept", "ECE ~\nBatch", "T"
  ),
  by = "par", all.x = TRUE
  ) %>%
  drop_na() %>%
  arrange(Order) %>%
  mutate(Par = factor(Par, levels = unique(Par))) %>%
  ggplot(aes(x = values)) +
  facet_wrap(~Par, scales = "free") +
  geom_density(colour = "black", fill = "white") +
  geom_vline(xintercept = 0, colour = "darkred", linetype = "solid") +
  scale_x_continuous(n.breaks = 3) +
  scale_y_continuous(n.breaks = 3) +
  xlab("Values") +
  ylab("Density") +
  theme_classic()
```

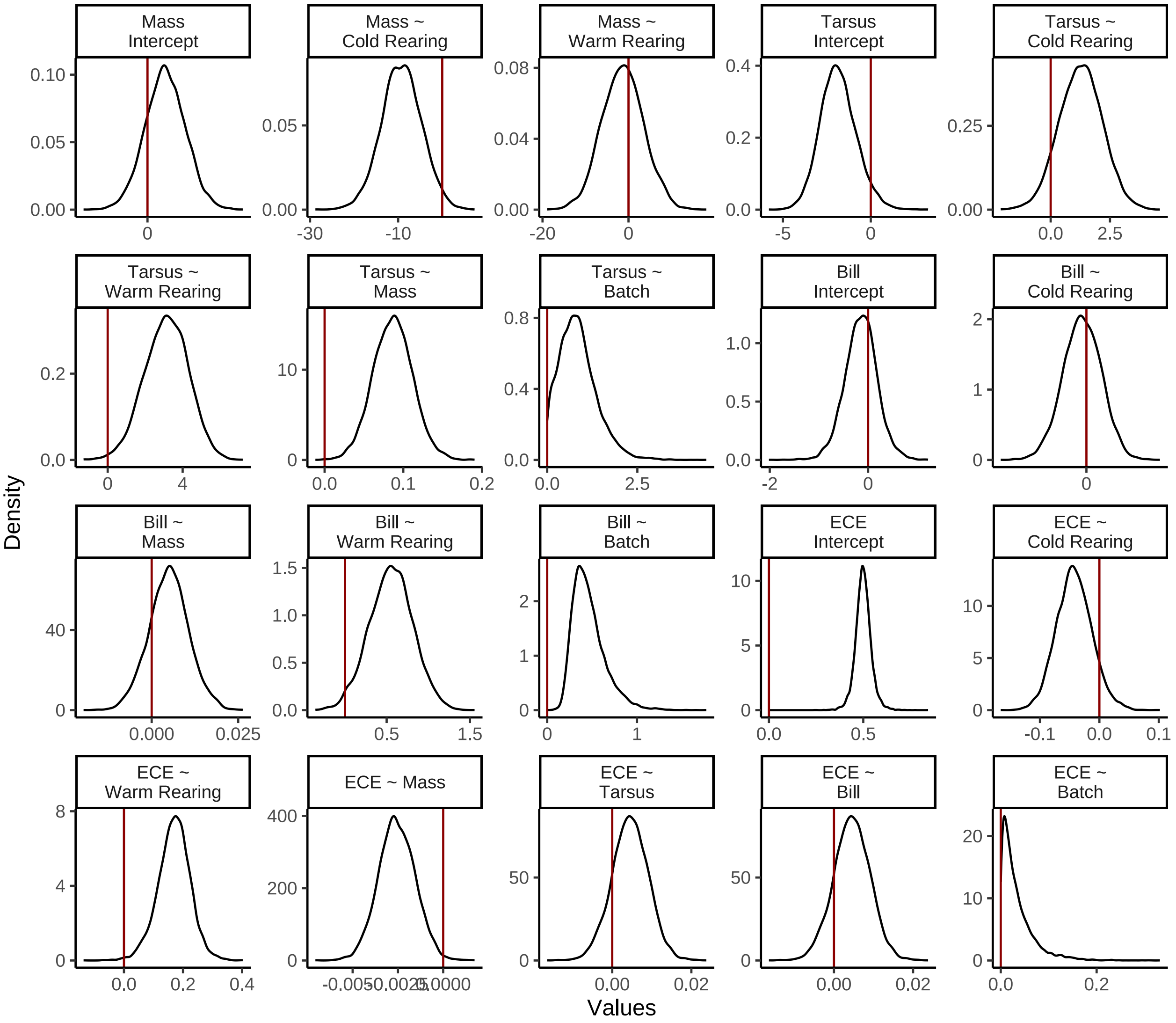

Figure 157: *Posterior densities for coefficients from a Bayesian path analysis predicting body mass (g), tarsus length (mm), bill length (mm) and evaporative cooling efficiency ("ECE", the ratio of evaporative heat loss in watts to evaporative heat production in watts) in three week old Japanese quail. Red vertical lines label 0. Response and predictor variables for which densities refer are indicated on the left side and right side of tildes respectively. "Cold Rearing" indicates rearing at 10°C, "Warm Rearing" indicates rearing at 30°C, and "Batch" indicates the batch of eggs from which an individual was derived. All coefficients except batch are population level.*

Given that some densities are slightly skewed or leptokurtic, we continue to summarise posteriors at their medians and estimate error around these medians using quantile-based credible intervals. Following this summarisation, we estimate variance explained by our total models (using Bayesian R^2 values) and by morphometric predictors alone (using partial Bayesian R^2 values).

```
caption <- paste0(
  "Results of Bayesian path analysis testing the ",
  "effects of morphology and rearing temperature on relative evaporative ",
  "heat loss at 40°C in three week old Japanese quail. Relative evaporative ",
  "heat loss represents the ",
  "fold change from 30°C, per individual. Estimates indicate posterior ",
  "medians and credible intervels (CIs) indicate quantile intervals."
```

```
)

heatLoss3WeeksResults <-
  as.data.frame(ehlModel3Weeks) %>%
  summarise_all(., .funs = median) %>%
  pivot_longer(everything(),
    names_to = "Parameter",
    values_to = "Estimate"
  ) %>%
  merge(., quantileCIs(ehlModel3Weeks, cis = c(50, 95)),
    by = "Parameter", all.x = TRUE
  ) %>%
  filter(grepl("b_|sd_", Parameter)) %>%
  rowwise() %>%
  mutate("BF" = ifelse(Estimate < 0,
    (2 * mean(as.data.frame(
      ehlModel3Weeks
    )[, Parameter] <= 0)) /
      (2 * mean(as.data.frame(
        ehlModel3Weeks
      )[, Parameter] >= 0)),
    (2 * mean(as.data.frame(
      ehlModel3Weeks
    )[, Parameter] >= 0)) /
      (2 * mean(as.data.frame(
        ehlModel3Weeks
      )[, Parameter] <= 0))
  )) %>%
  ungroup() %>%
  mutate(
    "Estimate" = round(Estimate, digits = 4),
    "BF" = round(BF, digits = 4),
    "N" = nrow(ehlModel3Weeks$data)
  ) %>%
  mutate("Parameter" = ifelse(grepl("b_", Parameter),
    gsub("b_", "", Parameter),
    gsub(
      "Intercept", "batch",
      gsub(".*__", "", Parameter)
    )
  )) %>%
  mutate(
    "Response" = gsub("_.*", "", Parameter),
    "Parameter" = gsub(".*_", "", Parameter)
  ) %>%
  merge(., tribble(
    ~Response, ~response, ~level,
    "mass", "Body Mass (g)", "A",
    "tarsus", "Tarsus Length (mm)", "B",
    "bill", "Bill Length (mm)", "C",
    "foldEhl", "Evaporative Heat Loss (fold from 30°C)", "D"
  ),
  by = "Response"
  ) %>%
  merge(., tribble(
    ~Parameter, ~parameter, ~number,
    "Intercept", "Intercept", "1",
    "mass", "Body Mass (g)", "4",
    "tarsus", "Tarsus Length (mm)", "5",
    "bill", "Bill Length (mm)", "6",
    "pretreatmentA", "Cold Rearing", "2",
    "pretreatmentC", "Warm Rearing", "3",
    "batch", "Egg Batch [mu]", "7"
  ),
  by = "Parameter"
  ) %>%
  mutate(
```

```
    `50\\% CI` = paste0("(", paste(
      round(Low_CI_50, digits = 4),
      round(High_CI_50, digits = 4),
      sep = ", "
    ), ")"),
    `95\\% CI` = paste0("(", paste(
      round(Low_CI_95, digits = 4),
      round(High_CI_95, digits = 4),
      sep = ", "
    ), ")")
  ) %>%
  dplyr::select(-c(Low_CI_50, High_CI_50, Low_CI_95, High_CI_95)) %>%
  dplyr::select(
    "Response" = "response", "Parameter" = "parameter", N,
    Estimate, `50\\% CI`, `95\\% CI`, BF, level, number
  ) %>%
  arrange(level, number) %>%
  dplyr::select(-c(level, number)) %>%
  kbl(.,
    longtable = T, booktabs = T, format = "latex", escape = FALSE,
    caption = caption
  ) %>%
  column_spec(column = c(1:2), width = "2.1cm") %>%
  column_spec(column = c(3:10), width = "1.8cm") %>%
  kable_styling(latex_options = "striped")

heatLoss3WeeksResults
```

Table 93: *Results of Bayesian path analysis testing the effects of morphology and rearing temperature on relative evaporative heat loss at 40°C in three week old Japanese quail. Relative evaporative heat loss represents the fold change from 30°C, per individual. Estimates indicate posterior medians and credible intervels (CIs) indicate quantile intervals.*

Response	Parameter	N	Estimate	50% CI	95% CI	BF
Body Mass (g)	Intercept	47	4.1027	(1.5136, 6.7622)	(-3.8083, 11.8924)	5.9264
Body Mass (g)	Cold Rearing	47	-8.3850	(-11.4949, -5.2027)	(-17.6433, 0.9857)	22.3918
Body Mass (g)	Warm Rearing	47	-0.1074	(-3.5597, 3.2914)	(-10.4586, 10.1126)	1.0284
Body Mass (g)	Egg Batch [mu]	47	0.2807	(0.1123, 0.5504)	(0.0123, 1.4098)	Inf
Tarsus Length (mm)	Intercept	47	-2.2339	(-2.8554, -1.5417)	(-3.9521, 0.0339)	36.5587
Tarsus Length (mm)	Cold Rearing	47	1.5005	(0.8894, 2.1081)	(-0.2951, 3.2144)	19.3562
Tarsus Length (mm)	Warm Rearing	47	2.8238	(1.9871, 3.6362)	(0.1575, 5.0255)	50.6129
Tarsus Length (mm)	Body Mass (g)	47	0.1009	(0.0835, 0.1187)	(0.0477, 0.1529)	Inf
Tarsus Length (mm)	Egg Batch [mu]	47	0.5465	(0.2465, 0.9378)	(0.0205, 1.9654)	Inf
Bill Length (mm)	Intercept	47	-0.1346	(-0.3444, 0.0798)	(-0.8267, 0.4963)	2.0315
Bill Length (mm)	Cold Rearing	47	-0.0263	(-0.1567, 0.1056)	(-0.4196, 0.3494)	1.2428
Bill Length (mm)	Warm Rearing	47	0.5210	(0.3356, 0.7097)	(-0.0403, 1.069)	28.5203
Bill Length (mm)	Body Mass (g)	47	0.0066	(0.0026, 0.0106)	(-0.0056, 0.0184)	6.3193
Bill Length (mm)	Egg Batch [mu]	47	0.4345	(0.3402, 0.5592)	(0.2058, 0.9403)	Inf
Evaporative Heat Loss (fold from 30°C)	Intercept	47	2.5547	(2.3873, 2.7131)	(2.0297, 3.0231)	Inf

Evaporative Heat Loss (fold from 30°C)	Cold Rearing	47	-0.2399	(-0.3631, -0.1124)	(-0.6123, 0.1411)	8.3677
Evaporative Heat Loss (fold from 30°C)	Warm Rearing	47	-0.4329	(-0.6094, -0.2476)	(-0.9524, 0.1151)	16.5439
Evaporative Heat Loss (fold from 30°C)	Body Mass (g)	47	0.0051	(9e-04, 0.0091)	(-0.007, 0.0175)	3.8544
Evaporative Heat Loss (fold from 30°C)	Tarsus Length (mm)	47	-0.0627	(-0.0832, -0.042)	(-0.1235, -7e-04)	41.7807
Evaporative Heat Loss (fold from 30°C)	Bill Length (mm)	47	0.2948	(0.1954, 0.3978)	(0.0063, 0.6006)	42.7158
Evaporative Heat Loss (fold from 30°C)	Egg Batch [mu]	47	0.2611	(0.1804, 0.3494)	(0.035, 0.5648)	Inf

```r
caption <- paste0(
  "Results of Bayesian path analysis testing the ",
  "effects of morphology and rearing temperature on evaporative ",
  "cooling efficiency at 40°C in three week old Japanese quail. ",
  "Evaporative cooling efficiency represents the quotient of ",
  "evaporative heat loss (in W) over metabolic heat production ",
  "(again, in W) and is therefore unitless. Estimates indicate posterior ",
  "medians and credible intervels (CIs) indicate quantile intervals."
)

efficiency3WeeksResults <-
  as.data.frame(efficiencyModel3Weeks) %>%
  summarise_all(., .funs = median) %>%
  pivot_longer(everything(),
    names_to = "Parameter",
    values_to = "Estimate"
  ) %>%
  merge(., quantileCIs(efficiencyModel3Weeks,
                       cis = c(50, 95)),
    by = "Parameter", all.x = TRUE
  ) %>%
  filter(grepl("b_|sd_", Parameter)) %>%
  rowwise() %>%
  mutate("BF" = ifelse(Estimate < 0,
    (2 * mean(as.data.frame(
      efficiencyModel3Weeks
    )[, Parameter] <= 0)) /
      (2 * mean(as.data.frame(
        efficiencyModel3Weeks
      )[, Parameter] >= 0)),
    (2 * mean(as.data.frame(
      efficiencyModel3Weeks
    )[, Parameter] >= 0)) /
      (2 * mean(as.data.frame(
        efficiencyModel3Weeks
      )[, Parameter] <= 0))
  )) %>%
  ungroup() %>%
  mutate(
    "Estimate" = round(Estimate, digits = 4),
    "BF" = round(BF, digits = 4),
    "N" = nrow(efficiencyModel3Weeks$data)
  ) %>%
  mutate("Parameter" = ifelse(grepl("b_", Parameter),
    gsub("b_", "", Parameter),
    gsub(
      "Intercept", "batch",
```

```
    gsub(".*__", "", Parameter)
  )
)) %>%
mutate(
  "Response" = gsub("_.*", "", Parameter),
  "Parameter" = gsub(".*_", "", Parameter)
) %>%
merge(., tribble(
  ~Response, ~response, ~level,
  "mass", "Body Mass (g)", "A",
  "tarsus", "Tarsus Length (mm)", "B",
  "bill", "Bill Length (mm)", "C",
  "ecc", "Evaporative Cooling Efficiency", "D"
),
by = "Response"
) %>%
merge(., tribble(
  ~Parameter, ~parameter, ~number,
  "Intercept", "Intercept", "1",
  "mass", "Body Mass (g)", "4",
  "tarsus", "Tarsus Length (mm)", "5",
  "bill", "Bill Length (mm)", "6",
  "pretreatmentA", "Cold Rearing", "2",
  "pretreatmentC", "Warm Rearing", "3",
  "batch", "Egg Batch [mu]", "7"
),
by = "Parameter"
) %>%
mutate(
  `50\\% CI` = paste0("(", paste(
    round(Low_CI_50, digits = 4),
    round(High_CI_50, digits = 4),
    sep = ", "
  ), ")"),
  `95\\% CI` = paste0("(", paste(
    round(Low_CI_95, digits = 4),
    round(High_CI_95, digits = 4),
    sep = ", "
  ), ")")
) %>%
dplyr::select(-c(Low_CI_50, High_CI_50, Low_CI_95, High_CI_95)) %>%
dplyr::select(
  "Response" = "response", "Parameter" = "parameter", N,
  Estimate, `50\\% CI`, `95\\% CI`, BF, level, number
) %>%
arrange(level, number) %>%
dplyr::select(-c(level, number)) %>%
kbl(.,
  longtable = T, booktabs = T, format = "latex", escape = FALSE,
  caption = caption
) %>%
column_spec(column = c(1:2), width = "2.1cm") %>%
column_spec(column = c(3:10), width = "1.8cm") %>%
kable_styling(latex_options = "striped")

efficiency3WeeksResults
```

Table 94: *Results of Bayesian path analysis testing the effects of morphology and rearing temperature on evaporative cooling efficiency at 40°C in three week old Japanese quail. Evaporative cooling efficiency represents the quotient of evaporative heat loss (in W) over metabolic heat production (again, in W) and is therefore unitless. Estimates indicate posterior medians and credible intervels (CIs) indicate quantile intervals.*

Response	Parameter	N	Estimate	50% CI	95% CI	BF
Body Mass (g)	Intercept	54	3.4025	(0.8444, 5.9714)	(-4.1398, 11.1602)	4.3583

Body Mass (g)	Cold Rearing	54	-9.2375	(-12.2493, -6.2047)	(-18.3804, -0.3214)	46.9042
Body Mass (g)	Warm Rearing	54	-1.3360	(-4.6258, 1.9119)	(-10.6209, 8.3573)	1.5592
Body Mass (g)	Egg Batch [mu]	54	0.2695	(0.1114, 0.5331)	(0.0094, 1.4146)	Inf
Tarsus Length (mm)	Intercept	54	-1.8947	(-2.55, -1.201)	(-3.7441, 0.3358)	21.7920
Tarsus Length (mm)	Cold Rearing	54	1.2752	(0.6542, 1.8858)	(-0.5194, 3.0289)	11.1766
Tarsus Length (mm)	Warm Rearing	54	3.1572	(2.3416, 3.9474)	(0.7625, 5.3357)	199.0000
Tarsus Length (mm)	Body Mass (g)	54	0.0868	(0.0696, 0.1032)	(0.0377, 0.1367)	1999.0000
Tarsus Length (mm)	Egg Batch [mu]	54	0.7992	(0.4766, 1.1459)	(0.0542, 2.0557)	Inf
Bill Length (mm)	Intercept	54	-0.1397	(-0.3577, 0.0645)	(-0.8173, 0.5072)	2.0511
Bill Length (mm)	Cold Rearing	54	-0.0419	(-0.1717, 0.0894)	(-0.4284, 0.3269)	1.4067
Bill Length (mm)	Warm Rearing	54	0.5575	(0.3783, 0.7286)	(0.0295, 1.0658)	58.7015
Bill Length (mm)	Body Mass (g)	54	0.0050	(0.0012, 0.0087)	(-0.0066, 0.0166)	4.2736
Bill Length (mm)	Egg Batch [mu]	54	0.4310	(0.3355, 0.5557)	(0.2096, 0.9134)	Inf
Evaporative Cooling Efficiency	Intercept	54	0.4991	(0.4751, 0.524)	(0.416, 0.5884)	Inf
Evaporative Cooling Efficiency	Cold Rearing	54	-0.0430	(-0.0624, -0.0228)	(-0.1, 0.0193)	11.7389
Evaporative Cooling Efficiency	Warm Rearing	54	0.1714	(0.1357, 0.2046)	(0.0546, 0.2732)	249.0000
Evaporative Cooling Efficiency	Body Mass (g)	54	-0.0026	(-0.0032, -0.0019)	(-0.0045, -6e-04)	141.8571
Evaporative Cooling Efficiency	Tarsus Length (mm)	54	0.0045	(0.0015, 0.0076)	(-0.0047, 0.0135)	5.3291
Evaporative Cooling Efficiency	Bill Length (mm)	54	-0.0148	(-0.0298, 1e-04)	(-0.0593, 0.0293)	2.9900
Evaporative Cooling Efficiency	Egg Batch [mu]	54	0.0230	(0.01, 0.0455)	(9e-04, 0.1286)	Inf

Partial R^2 values are here calculated by removing the focal predictor (i.e. either body mass, tarsus length, bill length, or both tarsus and bill length) from models within path analyses predicting evaporative heat loss or cooling measurements. Models are then re-run and total R^2 values compared between original and parameter-excluding models. The extent to which R^2 values are improved by inclusion of the target predictor (or predictors) is assumed as its partial R^2.

```
## Calculating total R2 values

caption <- paste0(
  "Estimates of fit for each element of a Bayesian path ",
  "analysis predicting evaporative heat loss responses ",
  "(fold from 30°C; Model 'A') and evaporative cooling ",
  "efficiency (Model 'B') in developing Japanese quail ",
  "(3 weeks of age). Response variables refer to those ",
  "measured at 40°C. Credible intervals are quantile intervals."
```

```
)

brms::bayes_R2(ehlModel3Weeks,
  ndraws = 1000,
  robust = TRUE
) %>%
  as.data.frame() %>%
  rownames_to_column("var") %>%
  merge(., tribble(
    ~var, ~Var, ~Order,
    "R2mass", "Body Mass (g)", "A",
    "R2tarsus", "Tarsus Length (mm)", "B",
    "R2bill", "Bill Length (mm)", "C",
    "R2foldEhl",
    "Evaporative Heat Loss", "D"
  ), by = c("var")) %>%
  mutate(
    Estimate = round(Estimate, digits = 4),
    Est.Error = round(Est.Error, digits = 4),
    "95\\% CI" = paste0(
      "[", round(Q2.5, digits = 4),
      ", ", round(Q97.5, digits = 4),
      "]"
    ),
    "Model" = "A"
  ) %>%
  rbind(., brms::bayes_R2(efficiencyModel3Weeks,
    ndraws = 1000,
    robust = TRUE
  ) %>%
    as.data.frame() %>%
    rownames_to_column("var") %>%
    merge(., tribble(
      ~var, ~Var, ~Order,
      "R2mass", "Body Mass (g)", "E",
      "R2tarsus", "Tarsus Length (mm)", "F",
      "R2bill", "Bill Length (mm)", "G",
      "R2ecc",
      "Evaporative Cooling Efficiency", "H"
    ), by = c("var")) %>%
    mutate(
      Estimate = round(Estimate, digits = 4),
      Est.Error = round(Est.Error, digits = 4),
      "95\\% CI" = paste0(
        "[", round(Q2.5, digits = 4),
        ", ", round(Q97.5, digits = 4),
        "]"
      ),
      "Model" = "B"
    )) %>%
  arrange(Order) %>%
  dplyr::select(
    Model,
    "Response" = Var, "R\\textsuperscript{2}" = Estimate,
    "Standard Error" = Est.Error,
    `95\\% CI`
  ) %>%
  kbl(.,
    longtable = T, booktabs = T, format = "latex",
    caption = caption, escape = FALSE
  ) %>%
  column_spec(column = c(1:10), width = "2.5cm") %>%
  kable_styling(latex_options = "striped")
```

Table 95: *Estimates of fit for each element of a Bayesian path analysis predicting evaporative heat loss responses (fold from 30°C; Model 'A') and evaporative cooling efficiency (Model 'B') in developing Japanese quail (3 weeks of age). Response variables refer to those measured at 40°C. Credible intervals are quantile intervals.*

Model	Response	R^2	Standard Error	95% CI
A	Body Mass (g)	0.1005	0.0687	[0.0117, 0.2565]
A	Tarsus Length (mm)	0.4182	0.0867	[0.2321, 0.5444]
A	Bill Length (mm)	0.4142	0.0892	[0.1857, 0.5479]
A	Evaporative Heat Loss	0.5785	0.0679	[0.3994, 0.6747]
B	Body Mass (g)	0.1126	0.0716	[0.0125, 0.2546]
B	Tarsus Length (mm)	0.4431	0.0832	[0.2477, 0.5788]
B	Bill Length (mm)	0.4141	0.0903	[0.2126, 0.5373]
B	Evaporative Cooling Efficiency	0.4080	0.0615	[0.2631, 0.5178]

```
# Calculating partial R2 values.
## Beginning with heat loss models

{
  ehl3WeekData <-
    vh2o %>%
    filter(week == "3" & Ta %in% c(30, 40)) %>%
    dplyr::select(Ta, ring, pretreatment,
      mass, tarsusLengthMean, billLengthMean, ehl,
      "batch" = exp
    ) %>%
    pivot_wider(
      id_cols = c(
        "ring", "batch",
        "pretreatment", "mass",
        "tarsusLengthMean", "billLengthMean"
      ),
      values_from = "ehl",
      names_from = "Ta"
    ) %>%
    mutate("foldEhl" = `40` / `30`) %>%
    mutate(pretreatment = ifelse(pretreatment == "neutral", "B",
      ifelse(pretreatment == "cold", "A", "C")
    )) %>%
    mutate(pretreatment = factor(pretreatment,
      levels = c("B", "A", "C")
    )) %>%
    distinct() %>%
    mutate(
      mass = mass - mean(mass, na.rm = T),
      tarsus = tarsusLengthMean - mean(tarsusLengthMean, na.rm = T),
      bill = billLengthMean - mean(billLengthMean, na.rm = T)
    ) %>%
    drop_na()

  ehlModel3WeeksMassR2 <- brm(
    data = ehl3WeekData,
    family = "gaussian",
    bf(mass ~ pretreatment + (1 | batch)) +
      bf(tarsus ~ mass + pretreatment + (1 | batch)) +
      bf(bill ~ mass + pretreatment + (1 | batch)) +
      bf(foldEhl ~ tarsus + bill + pretreatment + (1 | batch)) +
      set_rescor(FALSE),
    prior = c(
      set_prior("normal(0, 5)",
        class = "Intercept",
```

```
    resp = "mass"
  ),
  set_prior("normal(0, 15)",
    class = "b",
    coef = "pretreatmentA",
    resp = "mass"
  ),
  set_prior("normal(0, 15)",
    class = "b",
    coef = "pretreatmentC",
    resp = "mass"
  ),
  set_prior("exponential(2.5)",
    class = "sd",
    group = "batch",
    resp = "mass"
  ),
  set_prior("exponential(0.15)",
    class = "sigma",
    resp = "mass"
  ),
  set_prior("normal(0, 2.5)",
    class = "Intercept",
    resp = "tarsus"
  ),
  set_prior("normal(0, 2.5)",
    class = "b",
    coef = "pretreatmentA",
    resp = "tarsus"
  ),
  set_prior("normal(0, 2.5)",
    class = "b",
    coef = "pretreatmentC",
    resp = "tarsus"
  ),
  set_prior("skew_normal(0, 0.25, 5)",
    class = "b",
    coef = "mass",
    resp = "tarsus"
  ),
  set_prior("exponential(2)",
    class = "sd",
    group = "batch",
    resp = "tarsus"
  ),
  set_prior("exponential(1)",
    class = "sigma",
    resp = "tarsus"
  ),
  set_prior("normal(0, 1)",
    class = "Intercept",
    resp = "bill"
  ),
  set_prior("normal(0, 0.5)",
    class = "b",
    coef = "pretreatmentA",
    resp = "bill"
  ),
  set_prior("normal(0, 0.5)",
    class = "b",
    coef = "pretreatmentC",
    resp = "bill"
  ),
  set_prior("skew_normal(0, 0.25, 5)",
    class = "b",
    coef = "mass",
    resp = "bill"
```

```
    ),
    set_prior("exponential(5)",
      class = "sd",
      group = "batch",
      resp = "bill"
    ),
    set_prior("exponential(2.5)",
      class = "sigma",
      resp = "bill"
    ),
    set_prior("normal(2.5, 1)",
      class = "Intercept",
      resp = "foldEhl"
    ),
    set_prior("normal(0, 0.5)",
      class = "b",
      coef = "pretreatmentA",
      resp = "foldEhl"
    ),
    set_prior("normal(0, 0.5)",
      class = "b",
      coef = "pretreatmentC",
      resp = "foldEhl"
    ),
    set_prior("normal(0, 0.1)",
      class = "b",
      coef = "tarsus",
      resp = "foldEhl"
    ),
    set_prior("normal(0, 0.4)",
      class = "b",
      coef = "bill",
      resp = "foldEhl"
    ),
    set_prior("exponential(10)",
      class = "sd",
      group = "batch",
      resp = "foldEhl"
    ),
    set_prior("exponential(5)",
      class = "sigma",
      resp = "foldEhl"
    )
  ),
  iter = 50000, warmup = 10000, cores = 4, chains = 4, thin = 20,
  control = list(adapt_delta = .98, max_treedepth = 14),
  silent = TRUE, refresh = 0,
  file = "./models/_heatLossModel3WeeksMassR2.Rds",
)

ehlModel3WeeksTarsusR2 <- brm(
  data = ehl3WeekData,
  family = "gaussian",
  bf(mass ~ pretreatment + (1 | batch)) +
    bf(tarsus ~ mass + pretreatment + (1 | batch)) +
    bf(bill ~ mass + pretreatment + (1 | batch)) +
    bf(foldEhl ~ mass + bill + pretreatment + (1 | batch)) +
    set_rescor(FALSE),
  prior = c(
    set_prior("normal(0, 5)",
      class = "Intercept",
      resp = "mass"
    ),
    set_prior("normal(0, 15)",
      class = "b",
      coef = "pretreatmentA",
      resp = "mass"
```

```
    ),
    set_prior("normal(0, 15)",
      class = "b",
      coef = "pretreatmentC",
      resp = "mass"
    ),
    set_prior("exponential(2.5)",
      class = "sd",
      group = "batch",
      resp = "mass"
    ),
    set_prior("exponential(0.15)",
      class = "sigma",
      resp = "mass"
    ),
    set_prior("normal(0, 2.5)",
      class = "Intercept",
      resp = "tarsus"
    ),
    set_prior("normal(0, 2.5)",
      class = "b",
      coef = "pretreatmentA",
      resp = "tarsus"
    ),
    set_prior("normal(0, 2.5)",
      class = "b",
      coef = "pretreatmentC",
      resp = "tarsus"
    ),
    set_prior("skew_normal(0, 0.25, 5)",
      class = "b",
      coef = "mass",
      resp = "tarsus"
    ),
    set_prior("exponential(2)",
      class = "sd",
      group = "batch",
      resp = "tarsus"
    ),
    set_prior("exponential(1)",
      class = "sigma",
      resp = "tarsus"
    ),
    set_prior("normal(0, 1)",
      class = "Intercept",
      resp = "bill"
    ),
    set_prior("normal(0, 0.5)",
      class = "b",
      coef = "pretreatmentA",
      resp = "bill"
    ),
    set_prior("normal(0, 0.5)",
      class = "b",
      coef = "pretreatmentC",
      resp = "bill"
    ),
    set_prior("skew_normal(0, 0.25, 5)",
      class = "b",
      coef = "mass",
      resp = "bill"
    ),
    set_prior("exponential(5)",
      class = "sd",
      group = "batch",
      resp = "bill"
    ),
```

```
    set_prior("exponential(2.5)",
      class = "sigma",
      resp = "bill"
    ),
    set_prior("normal(2.5, 1)",
      class = "Intercept",
      resp = "foldEhl"
    ),
    set_prior("normal(0, 0.5)",
      class = "b",
      coef = "pretreatmentA",
      resp = "foldEhl"
    ),
    set_prior("normal(0, 0.5)",
      class = "b",
      coef = "pretreatmentC",
      resp = "foldEhl"
    ),
    set_prior("normal(0, 0.025)",
      class = "b",
      coef = "mass",
      resp = "foldEhl"
    ),
    set_prior("normal(0, 0.4)",
      class = "b",
      coef = "bill",
      resp = "foldEhl"
    ),
    set_prior("exponential(10)",
      class = "sd",
      group = "batch",
      resp = "foldEhl"
    ),
    set_prior("exponential(5)",
      class = "sigma",
      resp = "foldEhl"
    )
  ),
  iter = 50000, warmup = 10000, cores = 4, chains = 4, thin = 20,
  control = list(adapt_delta = .98, max_treedepth = 14),
  silent = TRUE, refresh = 0,
  file = "./models/_heatLossModel3WeeksTarsusR2.Rds",
)

ehlModel3WeeksBillR2 <- brm(
  data = ehl3WeekData,
  family = "gaussian",
  bf(mass ~ pretreatment + (1 | batch)) +
    bf(tarsus ~ mass + pretreatment + (1 | batch)) +
    bf(bill ~ mass + pretreatment + (1 | batch)) +
    bf(foldEhl ~ mass + tarsus + pretreatment + (1 | batch)) +
    set_rescor(FALSE),
  prior = c(
    set_prior("normal(0, 5)",
      class = "Intercept",
      resp = "mass"
    ),
    set_prior("normal(0, 15)",
      class = "b",
      coef = "pretreatmentA",
      resp = "mass"
    ),
    set_prior("normal(0, 15)",
      class = "b",
      coef = "pretreatmentC",
      resp = "mass"
    ),
```

```
    set_prior("exponential(2.5)",
      class = "sd",
      group = "batch",
      resp = "mass"
    ),
    set_prior("exponential(0.15)",
      class = "sigma",
      resp = "mass"
    ),
    set_prior("normal(0, 2.5)",
      class = "Intercept",
      resp = "tarsus"
    ),
    set_prior("normal(0, 2.5)",
      class = "b",
      coef = "pretreatmentA",
      resp = "tarsus"
    ),
    set_prior("normal(0, 2.5)",
      class = "b",
      coef = "pretreatmentC",
      resp = "tarsus"
    ),
    set_prior("skew_normal(0, 0.25, 5)",
      class = "b",
      coef = "mass",
      resp = "tarsus"
    ),
    set_prior("exponential(2)",
      class = "sd",
      group = "batch",
      resp = "tarsus"
    ),
    set_prior("exponential(1)",
      class = "sigma",
      resp = "tarsus"
    ),
    set_prior("normal(0, 1)",
      class = "Intercept",
      resp = "bill"
    ),
    set_prior("normal(0, 0.5)",
      class = "b",
      coef = "pretreatmentA",
      resp = "bill"
    ),
    set_prior("normal(0, 0.5)",
      class = "b",
      coef = "pretreatmentC",
      resp = "bill"
    ),
    set_prior("skew_normal(0, 0.25, 5)",
      class = "b",
      coef = "mass",
      resp = "bill"
    ),
    set_prior("exponential(5)",
      class = "sd",
      group = "batch",
      resp = "bill"
    ),
    set_prior("exponential(2.5)",
      class = "sigma",
      resp = "bill"
    ),
    set_prior("normal(2.5, 1)",
      class = "Intercept",
```

```
      resp = "foldEhl"
    ),
    set_prior("normal(0, 0.5)",
      class = "b",
      coef = "pretreatmentA",
      resp = "foldEhl"
    ),
    set_prior("normal(0, 0.5)",
      class = "b",
      coef = "pretreatmentC",
      resp = "foldEhl"
    ),
    set_prior("normal(0, 0.025)",
      class = "b",
      coef = "mass",
      resp = "foldEhl"
    ),
    set_prior("normal(0, 0.1)",
      class = "b",
      coef = "tarsus",
      resp = "foldEhl"
    ),
    set_prior("exponential(10)",
      class = "sd",
      group = "batch",
      resp = "foldEhl"
    ),
    set_prior("exponential(5)",
      class = "sigma",
      resp = "foldEhl"
    )
  ),
  iter = 50000, warmup = 10000, cores = 4, chains = 4, thin = 20,
  control = list(adapt_delta = .98, max_treedepth = 14),
  silent = TRUE, refresh = 0,
  file = "./models/_heatLossModel3WeeksBillR2.Rds",
)

ehlModel3WeeksAppendageR2 <- brm(
  data = ehl3WeekData,
  family = "gaussian",
  bf(mass ~ pretreatment + (1 | batch)) +
    bf(tarsus ~ mass + pretreatment + (1 | batch)) +
    bf(bill ~ mass + pretreatment + (1 | batch)) +
    bf(foldEhl ~ mass + pretreatment + (1 | batch)) +
    set_rescor(FALSE),
  prior = c(
    set_prior("normal(0, 5)",
      class = "Intercept",
      resp = "mass"
    ),
    set_prior("normal(0, 15)",
      class = "b",
      coef = "pretreatmentA",
      resp = "mass"
    ),
    set_prior("normal(0, 15)",
      class = "b",
      coef = "pretreatmentC",
      resp = "mass"
    ),
    set_prior("exponential(2.5)",
      class = "sd",
      group = "batch",
      resp = "mass"
    ),
    set_prior("exponential(0.15)",
```

```
        class = "sigma",
        resp = "mass"
      ),
      set_prior("normal(0, 2.5)",
        class = "Intercept",
        resp = "tarsus"
      ),
      set_prior("normal(0, 2.5)",
        class = "b",
        coef = "pretreatmentA",
        resp = "tarsus"
      ),
      set_prior("normal(0, 2.5)",
        class = "b",
        coef = "pretreatmentC",
        resp = "tarsus"
      ),
      set_prior("skew_normal(0, 0.25, 5)",
        class = "b",
        coef = "mass",
        resp = "tarsus"
      ),
      set_prior("exponential(2)",
        class = "sd",
        group = "batch",
        resp = "tarsus"
      ),
      set_prior("exponential(1)",
        class = "sigma",
        resp = "tarsus"
      ),
      set_prior("normal(0, 1)",
        class = "Intercept",
        resp = "bill"
      ),
      set_prior("normal(0, 0.5)",
        class = "b",
        coef = "pretreatmentA",
        resp = "bill"
      ),
      set_prior("normal(0, 0.5)",
        class = "b",
        coef = "pretreatmentC",
        resp = "bill"
      ),
      set_prior("skew_normal(0, 0.25, 5)",
        class = "b",
        coef = "mass",
        resp = "bill"
      ),
      set_prior("exponential(5)",
        class = "sd",
        group = "batch",
        resp = "bill"
      ),
      set_prior("exponential(2.5)",
        class = "sigma",
        resp = "bill"
      ),
      set_prior("normal(2.5, 1)",
        class = "Intercept",
        resp = "foldEhl"
      ),
      set_prior("normal(0, 0.5)",
        class = "b",
        coef = "pretreatmentA",
        resp = "foldEhl"
```

```
      ),
      set_prior("normal(0, 0.5)",
        class = "b",
        coef = "pretreatmentC",
        resp = "foldEhl"
      ),
      set_prior("normal(0, 0.025)",
        class = "b",
        coef = "mass",
        resp = "foldEhl"
      ),
      set_prior("exponential(10)",
        class = "sd",
        group = "batch",
        resp = "foldEhl"
      ),
      set_prior("exponential(5)",
        class = "sigma",
        resp = "foldEhl"
      )
    ),
    iter = 50000, warmup = 10000, cores = 4, chains = 4, thin = 20,
    control = list(adapt_delta = .98, max_treedepth = 14),
    silent = TRUE, refresh = 0,
    file = "./models/_heatLossModel3WeeksAppendageR2.Rds",
  )
}

## Proceeding to evaporative cooling efficiency models

{
  efficiencyData3Weeks <-
    vh2o %>%
    filter(week == "3" & Ta == 40) %>%
    dplyr::select(Ta, ring, pretreatment,
      mass, tarsusLengthMean, billLengthMean, ecc,
      "batch" = exp
    ) %>%
    filter(mass > 75) %>%
    mutate(
      pretreatment =
        ifelse(pretreatment == "neutral", "B",
          ifelse(pretreatment == "cold", "A", "C")
        )
    ) %>%
    mutate(pretreatment = factor(pretreatment,
      levels = c("B", "A", "C")
    )) %>%
    distinct() %>%
    mutate(
      mass = mass - mean(mass, na.rm = T),
      tarsus = tarsusLengthMean -
        mean(tarsusLengthMean, na.rm = T),
      bill = billLengthMean -
        mean(billLengthMean, na.rm = T)
    ) %>%
    drop_na()

  efficiencyModel3WeeksMassR2 <- brm(
    data = efficiencyData3Weeks,
    family = "gaussian",
    bf(mass ~ pretreatment + (1 | batch)) +
      bf(tarsus ~ mass + pretreatment + (1 | batch)) +
      bf(bill ~ mass + pretreatment + (1 | batch)) +
      bf(
        ecc ~ tarsus + bill + pretreatment + (1 | batch),
        sigma ~ batch
```

```
    ) +
    set_rescor(FALSE),
  prior = c(
    set_prior("normal(0, 5)",
      class = "Intercept",
      resp = "mass"
    ),
    set_prior("normal(0, 15)",
      class = "b",
      coef = "pretreatmentA",
      resp = "mass"
    ),
    set_prior("normal(0, 15)",
      class = "b",
      coef = "pretreatmentC",
      resp = "mass"
    ),
    set_prior("exponential(2.5)",
      class = "sd",
      group = "batch",
      resp = "mass"
    ),
    set_prior("exponential(0.15)",
      class = "sigma",
      resp = "mass"
    ),
    set_prior("normal(0, 2.5)",
      class = "Intercept",
      resp = "tarsus"
    ),
    set_prior("normal(0, 2.5)",
      class = "b",
      coef = "pretreatmentA",
      resp = "tarsus"
    ),
    set_prior("normal(0, 2.5)",
      class = "b",
      coef = "pretreatmentC",
      resp = "tarsus"
    ),
    set_prior("skew_normal(0, 0.25, 5)",
      class = "b",
      coef = "mass",
      resp = "tarsus"
    ),
    set_prior("exponential(2)",
      class = "sd",
      group = "batch",
      resp = "tarsus"
    ),
    set_prior("exponential(1)",
      class = "sigma",
      resp = "tarsus"
    ),
    set_prior("normal(0, 1)",
      class = "Intercept",
      resp = "bill"
    ),
    set_prior("normal(0, 0.5)",
      class = "b",
      coef = "pretreatmentA",
      resp = "bill"
    ),
    set_prior("normal(0, 0.5)",
      class = "b",
      coef = "pretreatmentC",
      resp = "bill"
```

```
    ),
    set_prior("skew_normal(0, 0.25, 5)",
      class = "b",
      coef = "mass",
      resp = "bill"
    ),
    set_prior("exponential(5)",
      class = "sd",
      group = "batch",
      resp = "bill"
    ),
    set_prior("exponential(2.5)",
      class = "sigma",
      resp = "bill"
    ),
    set_prior("normal(0.5, 0.2)",
      class = "Intercept",
      resp = "ecc"
    ),
    set_prior("normal(0, 0.25)",
      class = "b",
      coef = "pretreatmentA",
      resp = "ecc"
    ),
    set_prior("normal(0, 0.25)",
      class = "b",
      coef = "pretreatmentC",
      resp = "ecc"
    ),
    set_prior("normal(0, 0.02)",
      class = "b",
      coef = "tarsus",
      resp = "ecc"
    ),
    set_prior("normal(0, 0.075)",
      class = "b",
      coef = "bill",
      resp = "ecc"
    ),
    set_prior("exponential(15)",
      class = "sd",
      group = "batch",
      resp = "ecc"
    ),
    set_prior("normal(-2, 1)",
      dpar = "sigma",
      class = "Intercept",
      resp = "ecc"
    ),
    set_prior("normal(0, 0.5)",
      dpar = "sigma",
      class = "b",
      coef = "batchB",
      resp = "ecc"
    ),
    set_prior("normal(1, 1)",
      dpar = "sigma",
      class = "b",
      coef = "batchC",
      resp = "ecc"
    )
  ),
  iter = 50000, warmup = 10000, cores = 4, chains = 4, thin = 20,
  control = list(adapt_delta = .98, max_treedepth = 14),
  silent = TRUE, refresh = 0,
  file = "./models/_efficiencyModel3WeeksMassR2.Rds"
)
```

```
efficiencyModel3WeeksTarsusR2 <- brm(
  data = efficiencyData3Weeks,
  family = "gaussian",
  bf(mass ~ pretreatment + (1 | batch)) +
    bf(tarsus ~ mass + pretreatment + (1 | batch)) +
    bf(bill ~ mass + pretreatment + (1 | batch)) +
    bf(
      ecc ~ mass + bill + pretreatment + (1 | batch),
      sigma ~ batch
    ) +
    set_rescor(FALSE),
  prior = c(
    set_prior("normal(0, 5)",
      class = "Intercept",
      resp = "mass"
    ),
    set_prior("normal(0, 15)",
      class = "b",
      coef = "pretreatmentA",
      resp = "mass"
    ),
    set_prior("normal(0, 15)",
      class = "b",
      coef = "pretreatmentC",
      resp = "mass"
    ),
    set_prior("exponential(2.5)",
      class = "sd",
      group = "batch",
      resp = "mass"
    ),
    set_prior("exponential(0.15)",
      class = "sigma",
      resp = "mass"
    ),
    set_prior("normal(0, 2.5)",
      class = "Intercept",
      resp = "tarsus"
    ),
    set_prior("normal(0, 2.5)",
      class = "b",
      coef = "pretreatmentA",
      resp = "tarsus"
    ),
    set_prior("normal(0, 2.5)",
      class = "b",
      coef = "pretreatmentC",
      resp = "tarsus"
    ),
    set_prior("skew_normal(0, 0.25, 5)",
      class = "b",
      coef = "mass",
      resp = "tarsus"
    ),
    set_prior("exponential(2)",
      class = "sd",
      group = "batch",
      resp = "tarsus"
    ),
    set_prior("exponential(1)",
      class = "sigma",
      resp = "tarsus"
    ),
    set_prior("normal(0, 1)",
      class = "Intercept",
      resp = "bill"
    ),
```

```
    set_prior("normal(0, 0.5)",
      class = "b",
      coef = "pretreatmentA",
      resp = "bill"
    ),
    set_prior("normal(0, 0.5)",
      class = "b",
      coef = "pretreatmentC",
      resp = "bill"
    ),
    set_prior("skew_normal(0, 0.25, 5)",
      class = "b",
      coef = "mass",
      resp = "bill"
    ),
    set_prior("exponential(5)",
      class = "sd",
      group = "batch",
      resp = "bill"
    ),
    set_prior("exponential(2.5)",
      class = "sigma",
      resp = "bill"
    ),
    set_prior("normal(0.5, 0.2)",
      class = "Intercept",
      resp = "ecc"
    ),
    set_prior("normal(0, 0.25)",
      class = "b",
      coef = "pretreatmentA",
      resp = "ecc"
    ),
    set_prior("normal(0, 0.25)",
      class = "b",
      coef = "pretreatmentC",
      resp = "ecc"
    ),
    set_prior("normal(0, 0.01)",
      class = "b",
      coef = "mass",
      resp = "ecc"
    ),
    set_prior("normal(0, 0.075)",
      class = "b",
      coef = "bill",
      resp = "ecc"
    ),
    set_prior("exponential(15)",
      class = "sd",
      group = "batch",
      resp = "ecc"
    ),
    set_prior("normal(-2, 1)",
      dpar = "sigma",
      class = "Intercept",
      resp = "ecc"
    ),
    set_prior("normal(0, 0.5)",
      dpar = "sigma",
      class = "b",
      coef = "batchB",
      resp = "ecc"
    ),
    set_prior("normal(1, 1)",
      dpar = "sigma",
      class = "b",
```

```
      coef = "batchC",
      resp = "ecc"
    )
  ),
  iter = 50000, warmup = 10000, cores = 4, chains = 4, thin = 20,
  control = list(adapt_delta = .98, max_treedepth = 14),
  silent = TRUE, refresh = 0,
  file = "./models/_efficiencyModel3WeeksTarsusR2.Rds"
)

efficiencyModel3WeeksBillR2 <- brm(
  data = efficiencyData3Weeks,
  family = "gaussian",
  bf(mass ~ pretreatment + (1 | batch)) +
    bf(tarsus ~ mass + pretreatment + (1 | batch)) +
    bf(bill ~ mass + pretreatment + (1 | batch)) +
    bf(
      ecc ~ mass + tarsus + pretreatment + (1 | batch),
      sigma ~ batch
    ) +
    set_rescor(FALSE),
  prior = c(
    set_prior("normal(0, 5)",
      class = "Intercept",
      resp = "mass"
    ),
    set_prior("normal(0, 15)",
      class = "b",
      coef = "pretreatmentA",
      resp = "mass"
    ),
    set_prior("normal(0, 15)",
      class = "b",
      coef = "pretreatmentC",
      resp = "mass"
    ),
    set_prior("exponential(2.5)",
      class = "sd",
      group = "batch",
      resp = "mass"
    ),
    set_prior("exponential(0.15)",
      class = "sigma",
      resp = "mass"
    ),
    set_prior("normal(0, 2.5)",
      class = "Intercept",
      resp = "tarsus"
    ),
    set_prior("normal(0, 2.5)",
      class = "b",
      coef = "pretreatmentA",
      resp = "tarsus"
    ),
    set_prior("normal(0, 2.5)",
      class = "b",
      coef = "pretreatmentC",
      resp = "tarsus"
    ),
    set_prior("skew_normal(0, 0.25, 5)",
      class = "b",
      coef = "mass",
      resp = "tarsus"
    ),
    set_prior("exponential(2)",
      class = "sd",
      group = "batch",
```

```
    resp = "tarsus"
  ),
  set_prior("exponential(1)",
    class = "sigma",
    resp = "tarsus"
  ),
  set_prior("normal(0, 1)",
    class = "Intercept",
    resp = "bill"
  ),
  set_prior("normal(0, 0.5)",
    class = "b",
    coef = "pretreatmentA",
    resp = "bill"
  ),
  set_prior("normal(0, 0.5)",
    class = "b",
    coef = "pretreatmentC",
    resp = "bill"
  ),
  set_prior("skew_normal(0, 0.25, 5)",
    class = "b",
    coef = "mass",
    resp = "bill"
  ),
  set_prior("exponential(5)",
    class = "sd",
    group = "batch",
    resp = "bill"
  ),
  set_prior("exponential(2.5)",
    class = "sigma",
    resp = "bill"
  ),
  set_prior("normal(0.5, 0.2)",
    class = "Intercept",
    resp = "ecc"
  ),
  set_prior("normal(0, 0.25)",
    class = "b",
    coef = "pretreatmentA",
    resp = "ecc"
  ),
  set_prior("normal(0, 0.25)",
    class = "b",
    coef = "pretreatmentC",
    resp = "ecc"
  ),
  set_prior("normal(0, 0.01)",
    class = "b",
    coef = "mass",
    resp = "ecc"
  ),
  set_prior("normal(0, 0.02)",
    class = "b",
    coef = "tarsus",
    resp = "ecc"
  ),
  set_prior("exponential(15)",
    class = "sd",
    group = "batch",
    resp = "ecc"
  ),
  set_prior("normal(-2, 1)",
    dpar = "sigma",
    class = "Intercept",
    resp = "ecc"
```

```
    ),
    set_prior("normal(0, 0.5)",
      dpar = "sigma",
      class = "b",
      coef = "batchB",
      resp = "ecc"
    ),
    set_prior("normal(1, 1)",
      dpar = "sigma",
      class = "b",
      coef = "batchC",
      resp = "ecc"
    )
  ),
  iter = 50000, warmup = 10000, cores = 4, chains = 4, thin = 20,
  control = list(adapt_delta = .98, max_treedepth = 14),
  silent = TRUE, refresh = 0,
  file = "./models/_efficiencyModel3WeeksBillR2.Rds"
)

efficiencyModel3WeeksAppendageR2 <- brm(
  data = efficiencyData3Weeks,
  family = "gaussian",
  bf(mass ~ pretreatment + (1 | batch)) +
    bf(tarsus ~ mass + pretreatment + (1 | batch)) +
    bf(bill ~ mass + pretreatment + (1 | batch)) +
    bf(
      ecc ~ mass + pretreatment + (1 | batch),
      sigma ~ batch
    ) +
    set_rescor(FALSE),
  prior = c(
    set_prior("normal(0, 5)",
      class = "Intercept",
      resp = "mass"
    ),
    set_prior("normal(0, 15)",
      class = "b",
      coef = "pretreatmentA",
      resp = "mass"
    ),
    set_prior("normal(0, 15)",
      class = "b",
      coef = "pretreatmentC",
      resp = "mass"
    ),
    set_prior("exponential(2.5)",
      class = "sd",
      group = "batch",
      resp = "mass"
    ),
    set_prior("exponential(0.15)",
      class = "sigma",
      resp = "mass"
    ),
    set_prior("normal(0, 2.5)",
      class = "Intercept",
      resp = "tarsus"
    ),
    set_prior("normal(0, 2.5)",
      class = "b",
      coef = "pretreatmentA",
      resp = "tarsus"
    ),
    set_prior("normal(0, 2.5)",
      class = "b",
      coef = "pretreatmentC",
```

```
      resp = "tarsus"
    ),
    set_prior("skew_normal(0, 0.25, 5)",
      class = "b",
      coef = "mass",
      resp = "tarsus"
    ),
    set_prior("exponential(2)",
      class = "sd",
      group = "batch",
      resp = "tarsus"
    ),
    set_prior("exponential(1)",
      class = "sigma",
      resp = "tarsus"
    ),
    set_prior("normal(0, 1)",
      class = "Intercept",
      resp = "bill"
    ),
    set_prior("normal(0, 0.5)",
      class = "b",
      coef = "pretreatmentA",
      resp = "bill"
    ),
    set_prior("normal(0, 0.5)",
      class = "b",
      coef = "pretreatmentC",
      resp = "bill"
    ),
    set_prior("skew_normal(0, 0.25, 5)",
      class = "b",
      coef = "mass",
      resp = "bill"
    ),
    set_prior("exponential(5)",
      class = "sd",
      group = "batch",
      resp = "bill"
    ),
    set_prior("exponential(2.5)",
      class = "sigma",
      resp = "bill"
    ),
    set_prior("normal(0.5, 0.2)",
      class = "Intercept",
      resp = "ecc"
    ),
    set_prior("normal(0, 0.25)",
      class = "b",
      coef = "pretreatmentA",
      resp = "ecc"
    ),
    set_prior("normal(0, 0.25)",
      class = "b",
      coef = "pretreatmentC",
      resp = "ecc"
    ),
    set_prior("normal(0, 0.01)",
      class = "b",
      coef = "mass",
      resp = "ecc"
    ),
    set_prior("exponential(15)",
      class = "sd",
      group = "batch",
      resp = "ecc"
```

```
      ),
      set_prior("normal(-2, 1)",
        dpar = "sigma",
        class = "Intercept",
        resp = "ecc"
      ),
      set_prior("normal(0, 0.5)",
        dpar = "sigma",
        class = "b",
        coef = "batchB",
        resp = "ecc"
      ),
      set_prior("normal(1, 1)",
        dpar = "sigma",
        class = "b",
        coef = "batchC",
        resp = "ecc"
      )
    ),
    iter = 50000, warmup = 10000, cores = 4, chains = 4, thin = 20,
    control = list(adapt_delta = .98, max_treedepth = 14),
    silent = TRUE, refresh = 0,
    file = "./models/_efficiencyModel3WeeksAppendageR2.Rds"
  )
}

pR2Pull <- function(baseModel, response, x) {
  baseR2 <- as.data.frame(
    brms::bayes_R2(baseModel,
      ndraws = 1000,
      resp = response, summary = FALSE,
      robust = TRUE
    )
  )[,1]
  redR2 <- as.data.frame(
    brms::bayes_R2(x,
      ndraws = 1000,
      resp = response, summary = FALSE,
      robust = TRUE
    )
  )[,1]
  pR2 <- round(baseR2 - redR2, digits = 3)

  ciFrame <- t(
    quantile(pR2, probs = c(0.025, 0.975), type = 8)
  ) %>% as.data.frame()

  ciFrame <- ciFrame %>%
    mutate(`2.5%` = ifelse(`2.5%` < 0, 0, `2.5%`)) %>%
    mutate("95\\% CI" = paste0(
      "[",
      round(`2.5%`, digits = 3),
      ",",
      round(`97.5%`, digits = 3),
      "]"
    )) %>%
    mutate("Partial R2" = round(median(pR2), digits = 3)) %>%
    mutate(`Partial R2` = ifelse(`Partial R2` < 0, 0, `Partial R2`)) %>%
    dplyr::select("Partial R\\textsuperscript{2}" = `Partial R2`, "95\\% CI")

  return(ciFrame)
}

caption = paste0(
  'Variance in evaporative heat loss ("EHL", fold change ',
  'from 30°C) and evaporative cooling efficiency ("ECE", the ratio of evaporative ',
  'heat loss to metabolic heat production) at at 40°C explained by ',
```

```
  'morphometry in three week old Japanese quail.'
  )

models <- list(ehlModel3WeeksMassR2, ehlModel3WeeksTarsusR2,
            ehlModel3WeeksBillR2, ehlModel3WeeksAppendageR2)
hold <- bind_rows(
  lapply(models, pR2Pull, baseModel = ehlModel3Weeks, response = "foldEhl")
  ) %>%
  mutate("Variable" = c("Body Mass", "Tarsus Length",
                        "Bill Length", "Appendage Length"),
         "Response" = "EHL"
         ) %>%
  dplyr::select(
    Response, Variable, "Partial R\\textsuperscript{2}", `95\\% CI`
  )

models <- list(efficiencyModel3WeeksMassR2, efficiencyModel3WeeksTarsusR2,
            efficiencyModel3WeeksBillR2, efficiencyModel3WeeksAppendageR2)
bind_rows(
  lapply(models, pR2Pull, baseModel = efficiencyModel3Weeks,
         response = "ecc")
  ) %>%
  mutate("Variable" = c("Body Mass", "Tarsus Length",
                        "Bill Length", "Appendage Length"),
         "Response" = "ECE") %>%
  dplyr::select(
    Response, Variable, "Partial R\\textsuperscript{2}", `95\\% CI`
  ) %>%
  rbind(hold, .) %>%
  kbl(.,
    longtable = T, booktabs = T, format = "latex",
    caption = caption, escape = FALSE
  ) %>%
  column_spec(column = c(1:10), width = "2.5cm")  %>%
  kable_styling(latex_options = "striped")
```

Table 96: *Variance in evaporative heat loss ("EHL", fold change from 30°C) and evaporative cooling efficiency ("ECE", the ratio of evaporative heat loss to metabolic heat production) at at 40°C explained by morphometry in three week old Japanese quail.*

Response	Variable	Partial R^2	95% CI
EHL	Body Mass	0.004	[0,0.242]
EHL	Tarsus Length	0.028	[0,0.248]
EHL	Bill Length	0.011	[0,0.229]
EHL	Appendage Length	0.024	[0,0.278]
ECE	Body Mass	0.026	[0,0.228]
ECE	Tarsus Length	0.007	[0,0.199]
ECE	Bill Length	0.005	[0,0.196]
ECE	Appendage Length	0.008	[0,0.201]

```
rm(models)
```

Next, we visualise conditional effects of morphology and rearing conditions on evaporative heat loss responses and evaporative cooling efficiency in our developing quail. Conditional effects are estimated from posteriors of our path analysis while assuming that all other parameters lay at their average.

```
## Producing morphology plots

heatLoss3WeeksMassPlot <-
  expand.grid(
    "mass" = with(
      ehlModel3Weeks$data,
      seq(min(mass), max(mass), by = 1)
```

```
    ),
    "tarsus" = 0,
    "bill" = 0,
    "pretreatment" = "B"
  ) %>%
  mutate(
    "foldEhl" = predict(ehlModel3Weeks,
      newdata = .,
      re_form = NA, robust = TRUE,
      resp = "foldEhl"
    )[, "Estimate"],
    "SE" = predict(ehlModel3Weeks,
      newdata = .,
      re_form = NA, robust = TRUE,
      resp = "foldEhl"
    )[, "Est.Error"]
  ) %>%
  mutate(mass = mass + mean(subset(vh2o, week == 3)$mass, na.rm = T)) %>%
  ggplot(aes(x = mass, y = foldEhl)) +
  geom_ribbon(aes(ymin = foldEhl - SE, ymax = foldEhl + SE),
    alpha = 0.5, fill = "#DECCC1"
  ) +
  geom_point(
    data = ehlModel3Weeks$data %>%
      mutate(mass = mass +
        mean(subset(vh2o, week == 3)$mass, na.rm = T)),
    aes(x = mass, y = foldEhl),
    alpha = 0.5
  ) +
  geom_smooth(
    method = "lm", colour = "black",
    linetype = "dashed", se = FALSE
  ) +
  xlab("Body Mass (g)") +
  ylab("Evaporative Heat Loss\n(Fold From 30°C)") +
  theme_classic() +
  theme(
    axis.title = element_text(family = "Noto Sans"),
    axis.text = element_text(family = "Noto Sans")
  )

heatLoss3WeeksTarsusPlot <-
  expand.grid(
    "tarsus" = with(
      ehlModel3Weeks$data,
      seq(min(tarsus), max(tarsus), by = 1)
    ),
    "mass" = 0,
    "bill" = 0,
    "pretreatment" = "B"
  ) %>%
  mutate(
    "foldEhl" = predict(ehlModel3Weeks,
      newdata = .,
      re_form = NA, robust = TRUE,
      resp = "foldEhl"
    )[, "Estimate"],
    "SE" = predict(ehlModel3Weeks,
      newdata = .,
      re_form = NA, robust = TRUE,
      resp = "foldEhl"
    )[, "Est.Error"]
  ) %>%
  mutate(tarsus = tarsus +
           mean(subset(vh2o, week == 3)$tarsusLengthMean, na.rm = T)) %>%
  ggplot(aes(x = tarsus, y = foldEhl)) +
  geom_ribbon(aes(ymin = foldEhl - SE, ymax = foldEhl + SE),
```

```
    alpha = 0.5, fill = "#DECCC1"
  ) +
  geom_point(
    data = ehlModel3Weeks$data %>%
      mutate(tarsus = tarsus +
        mean(subset(vh2o, week == 3)$tarsusLengthMean, na.rm = T)),
    aes(x = tarsus, y = foldEhl),
    alpha = 0.5
  ) +
  geom_smooth(
    method = "lm", colour = "black",
    linetype = "dashed", se = FALSE
  ) +
  xlab("Tarsus Length (mm)") +
  ylab("Evaporative Heat Loss\n(Fold From 30°C)") +
  theme_classic() +
  theme(
    axis.title = element_text(family = "Noto Sans"),
    axis.text = element_text(family = "Noto Sans")
  )

heatLoss3WeeksBillPlot <-
  expand.grid(
    "bill" = with(
      ehlModel3Weeks$data,
      seq(min(bill), max(bill), by = 1)
    ),
    "mass" = 0,
    "tarsus" = 0,
    "pretreatment" = "B"
  ) %>%
  mutate(
    "foldEhl" = predict(ehlModel3Weeks,
      newdata = .,
      re_form = NA, robust = TRUE,
      resp = "foldEhl"
    )[, "Estimate"],
    "SE" = predict(ehlModel3Weeks,
      newdata = .,
      re_form = NA, robust = TRUE,
      resp = "foldEhl"
    )[, "Est.Error"]
  ) %>%
  mutate(bill = bill +
           mean(subset(vh2o, week == 3)$billLengthMean, na.rm = T)) %>%
  ggplot(aes(x = bill, y = foldEhl)) +
  geom_ribbon(aes(ymin = foldEhl - SE, ymax = foldEhl + SE),
    alpha = 0.5, fill = "#DECCC1"
  ) +
  geom_point(
    data = ehlModel3Weeks$data %>%
      mutate(bill = bill +
        mean(subset(vh2o, week == 3)$billLengthMean, na.rm = T)),
    aes(x = bill, y = foldEhl),
    alpha = 0.5
  ) +
  geom_smooth(
    method = "lm", colour = "black",
    linetype = "dashed", se = FALSE
  ) +
  xlab("Bill Length (mm)") +
  ylab("Evaporative Heat Loss\n(Fold From 30°C)") +
  theme_classic() +
  theme(
    axis.title = element_text(family = "Noto Sans"),
    axis.text = element_text(family = "Noto Sans")
  )
```

```
efficiency3WeeksMassPlot <-
  expand.grid(
    "mass" = with(
      efficiencyModel3Weeks$data,
      seq(min(mass), max(mass), by = 1)
    ),
    "tarsus" = 0,
    "bill" = 0,
    "pretreatment" = "B",
    "batch" = "A"
  ) %>%
  mutate(
    "ecc" = predict(efficiencyModel3Weeks,
      newdata = .,
      re_form = NA, robust = TRUE,
      resp = "ecc"
    )[, "Estimate"],
    "SE" = predict(efficiencyModel3Weeks,
      newdata = .,
      re_form = NA, robust = TRUE,
      resp = "ecc"
    )[, "Est.Error"]
  ) %>%
  mutate(mass = mass + mean(subset(vh2o, week == 3)$mass, na.rm = T)) %>%
  ggplot(aes(x = mass, y = ecc)) +
  geom_ribbon(aes(ymin = ecc - SE, ymax = ecc + SE),
    alpha = 0.5, fill = "#DECCC1"
  ) +
  geom_point(
    data = efficiencyModel3Weeks$data %>%
      mutate(mass = mass +
        mean(subset(vh2o, week == 3)$mass, na.rm = T)),
    aes(x = mass, y = ecc),
    alpha = 0.5
  ) +
  geom_smooth(
    method = "lm", colour = "black",
    linetype = "dashed", se = FALSE
  ) +
  xlab("Body Mass (g)") +
  ylab("Evaporative Cooling\nEfficiency (EHL/RMR)") +
  theme_classic() +
  theme(
    axis.title = element_text(family = "Noto Sans"),
    axis.text = element_text(family = "Noto Sans")
  )

efficiency3WeeksTarsusPlot <-
  expand.grid(
    "tarsus" = with(
      efficiencyModel3Weeks$data,
      seq(min(tarsus), max(tarsus), by = 1)
    ),
    "mass" = 0,
    "bill" = 0,
    "pretreatment" = "B",
    "batch" = "A"
  ) %>%
  mutate(
    "ecc" = predict(efficiencyModel3Weeks,
      newdata = .,
      re_form = NA, robust = TRUE,
      resp = "ecc"
    )[, "Estimate"],
    "SE" = predict(efficiencyModel3Weeks,
      newdata = .,
      re_form = NA, robust = TRUE,
```

```
      resp = "ecc"
    )[, "Est.Error"]
  ) %>%
  mutate(tarsus = tarsus +
           mean(subset(vh2o, week == 3)$tarsusLengthMean, na.rm = T)) %>%
  ggplot(aes(x = tarsus, y = ecc)) +
  geom_ribbon(aes(ymin = ecc - SE, ymax = ecc + SE),
    alpha = 0.5, fill = "#DECCC1"
  ) +
  geom_point(
    data = efficiencyModel3Weeks$data %>%
      mutate(tarsus = tarsus +
        mean(subset(vh2o, week == 3)$tarsusLengthMean, na.rm = T)),
    aes(x = tarsus, y = ecc),
    alpha = 0.5
  ) +
  geom_smooth(
    method = "lm", colour = "black",
    linetype = "dashed", se = FALSE
  ) +
  xlab("Tarsus Length (mm)") +
  ylab("Evaporative Cooling\nEfficiency (EHL/RMR)") +
  theme_classic() +
  theme(
    axis.title = element_text(family = "Noto Sans"),
    axis.text = element_text(family = "Noto Sans")
  )

efficiency3WeeksBillPlot <-
  expand.grid(
    "bill" = with(
      efficiencyModel3Weeks$data,
      seq(min(bill), max(bill), by = 1)
    ),
    "mass" = 0,
    "tarsus" = 0,
    "pretreatment" = "B",
    "batch" = "A"
  ) %>%
  mutate(
    "ecc" = predict(efficiencyModel3Weeks,
      newdata = .,
      re_form = NA, robust = TRUE,
      resp = "ecc"
    )[, "Estimate"],
    "SE" = predict(efficiencyModel3Weeks,
      newdata = .,
      re_form = NA, robust = TRUE,
      resp = "ecc"
    )[, "Est.Error"]
  ) %>%
  mutate(bill = bill +
           mean(subset(vh2o, week == 3)$billLengthMean, na.rm = T)) %>%
  ggplot(aes(x = bill, y = ecc)) +
  geom_ribbon(aes(ymin = ecc - SE, ymax = ecc + SE),
    alpha = 0.5, fill = "#DECCC1"
  ) +
  geom_point(
    data = efficiencyModel3Weeks$data %>%
      mutate(bill = bill +
        mean(subset(vh2o, week == 3)$billLengthMean, na.rm = T)),
    aes(x = bill, y = ecc),
    alpha = 0.5
  ) +
  geom_smooth(
    method = "lm", colour = "black",
    linetype = "dashed", se = FALSE
```

```
  ) +
  xlab("Bill Length (mm)") +
  ylab("Evaporative Cooling\nEfficiency (EHL/RMR)") +
  theme_classic() +
  theme(
    axis.title = element_text(family = "Noto Sans"),
    axis.text = element_text(family = "Noto Sans")
  )

(heatLoss3WeeksMassPlot + heatLoss3WeeksTarsusPlot + heatLoss3WeeksBillPlot) /
  (efficiency3WeeksMassPlot + efficiency3WeeksTarsusPlot + efficiency3WeeksBillPlot) +
  plot_annotation(tag_level = "A")
```

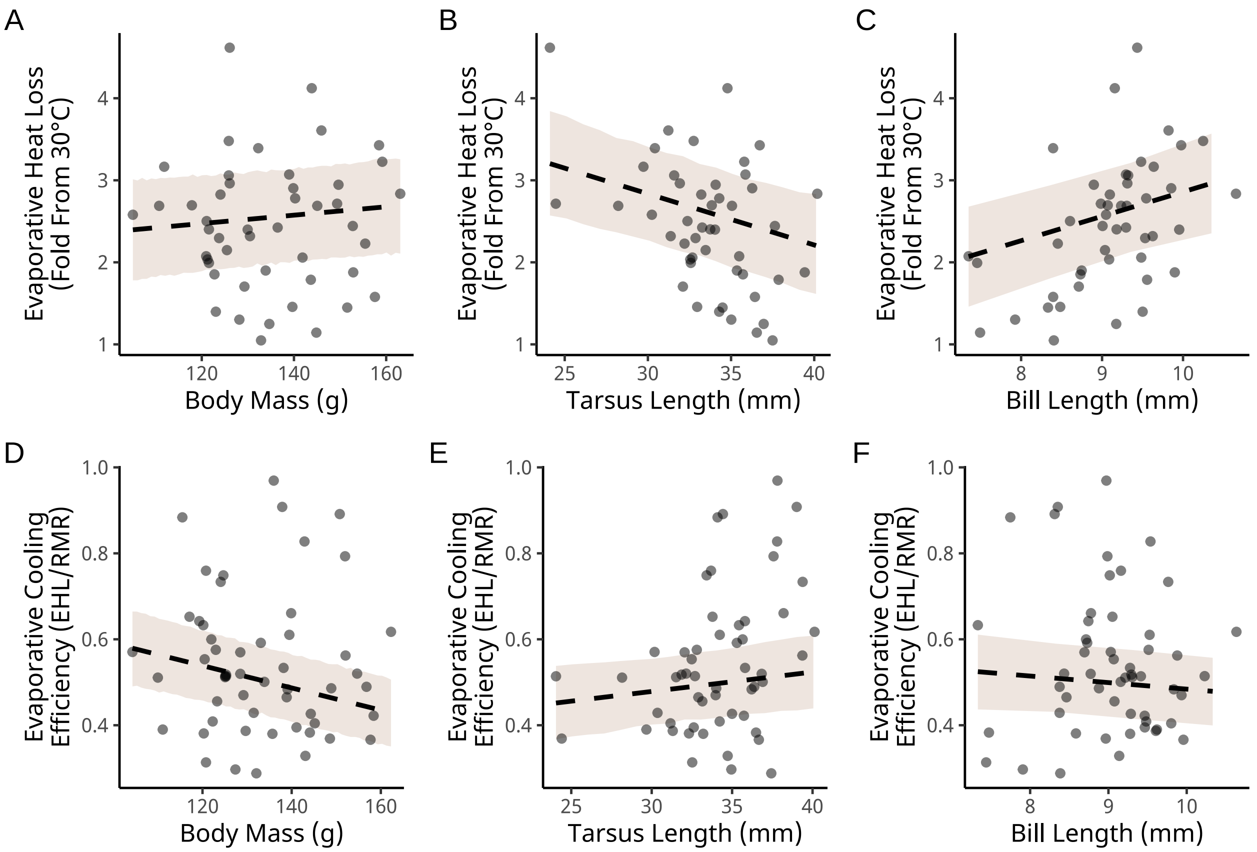

Figure 158: *Effects of morphology on evaporative heat loss responses (panels A to C) and evaporative cooling efficiency (panels D to F) in developing Japanese quail (three weeks of age; n = 48). Evaporative heat loss responses represent fold increases in evaporative heat loss, in watts, between 30°C and 40°C. Evaporative cooling efficiency represents the ratio between evaporative heat loss (in watts) and metabolic heat production (in watts). Dashed lines indicate predicted relationships, as estimated from Bayesian path analyses. Ribbons represents +/- one standard error around predicted lines of best fit. Small dots indicate raw data points.*

```
heatLoss3WeeksTreatmentPlot <-
  expand.grid(
    "mass" = 0,
    "tarsus" = 0,
    "bill" = 0,
    "pretreatment" = c("A", "B", "C")
  ) %>%
  mutate(
    "foldEhl" = predict(ehlModel3Weeks,
```

```
      newdata = .,
      re_form = NA, robust = TRUE,
      resp = "foldEhl"
    )[, "Estimate"],
    "SE" = predict(ehlModel3Weeks,
      newdata = .,
      re_form = NA, robust = TRUE,
      resp = "foldEhl"
    )[, "Est.Error"]
  ) %>%
  mutate(pretreatment = factor(pretreatment, levels = c("A", "B", "C"))) %>%
  ggplot(aes(x = pretreatment, y = foldEhl, fill = pretreatment)) +
  geom_point(
    data = ehlModel3Weeks$data %>%
      mutate(pretreatment = factor(pretreatment, levels = c("A", "B", "C"))),
    aes(x = pretreatment, y = foldEhl),
    alpha = 0.5, position = position_jitter(width = 0.25)
  ) +
  geom_errorbar(aes(ymin = foldEhl - SE, ymax = foldEhl + SE),
    colour = "black", width = 0.25
  ) +
  geom_point(
    pch = 21, colour = "black", size = 4
  ) +
  scale_x_discrete(name = "Rearing Treatment",
                   labels = c("Cold (10°C)",
                              "Mild (20°C)",
                              "Warm (30°C)")
                   ) +
  ylab("Evaporative Heat Loss\nResponse (Fold From 30°C)") +
  scale_fill_manual(values = c("#7BB4E3", "black", "#CD5C5C")) +
  theme_classic() +
  theme(
    axis.title = element_text(family = "Noto Sans"),
    axis.text = element_text(family = "Noto Sans"),
    legend.position = "none"
  )

efficiency3WeeksTreatmentPlot <-
  expand.grid(
    "mass" = 0,
    "tarsus" = 0,
    "bill" = 0,
    "pretreatment" = c("A", "B", "C"),
    "batch" = "A"
  ) %>%
  mutate(
    "ecc" = predict(efficiencyModel3Weeks,
      newdata = .,
      re_form = NA, robust = TRUE,
      resp = "ecc"
    )[, "Estimate"],
    "SE" = predict(efficiencyModel3Weeks,
      newdata = .,
      re_form = NA, robust = TRUE,
      resp = "ecc"
    )[, "Est.Error"]
  ) %>%
  mutate(pretreatment = factor(pretreatment, levels = c("A", "B", "C"))) %>%
  ggplot(aes(x = pretreatment, y = ecc, fill = pretreatment)) +
  geom_point(
    data = efficiencyModel3Weeks$data %>%
      mutate(pretreatment = factor(pretreatment, levels = c("A", "B", "C"))),
    aes(x = pretreatment, y = ecc),
    alpha = 0.5, position = position_jitter(width = 0.25)
  ) +
  geom_errorbar(aes(ymin = ecc - SE, ymax = ecc + SE),
```

```
    colour = "black", width = 0.25
  ) +
  geom_point(
    pch = 21, colour = "black", size = 4
  ) +
  scale_x_discrete(name = "Rearing Treatment",
                   labels = c("Cold (10°C)",
                              "Mild (20°C)",
                              "Warm (30°C)")
                   ) +
  ylab("Evaporative Cooling\nEfficiency (EHL/RMR)") +
  scale_fill_manual(values = c("#7BB4E3", "black", "#CD5C5C")) +
  theme_classic() +
  theme(
    axis.title = element_text(family = "Noto Sans"),
    axis.text = element_text(family = "Noto Sans"),
    legend.position = "none"
  )

(heatLoss3WeeksTreatmentPlot / efficiency3WeeksTreatmentPlot) +
  plot_annotation(tag_levels = "A")
```

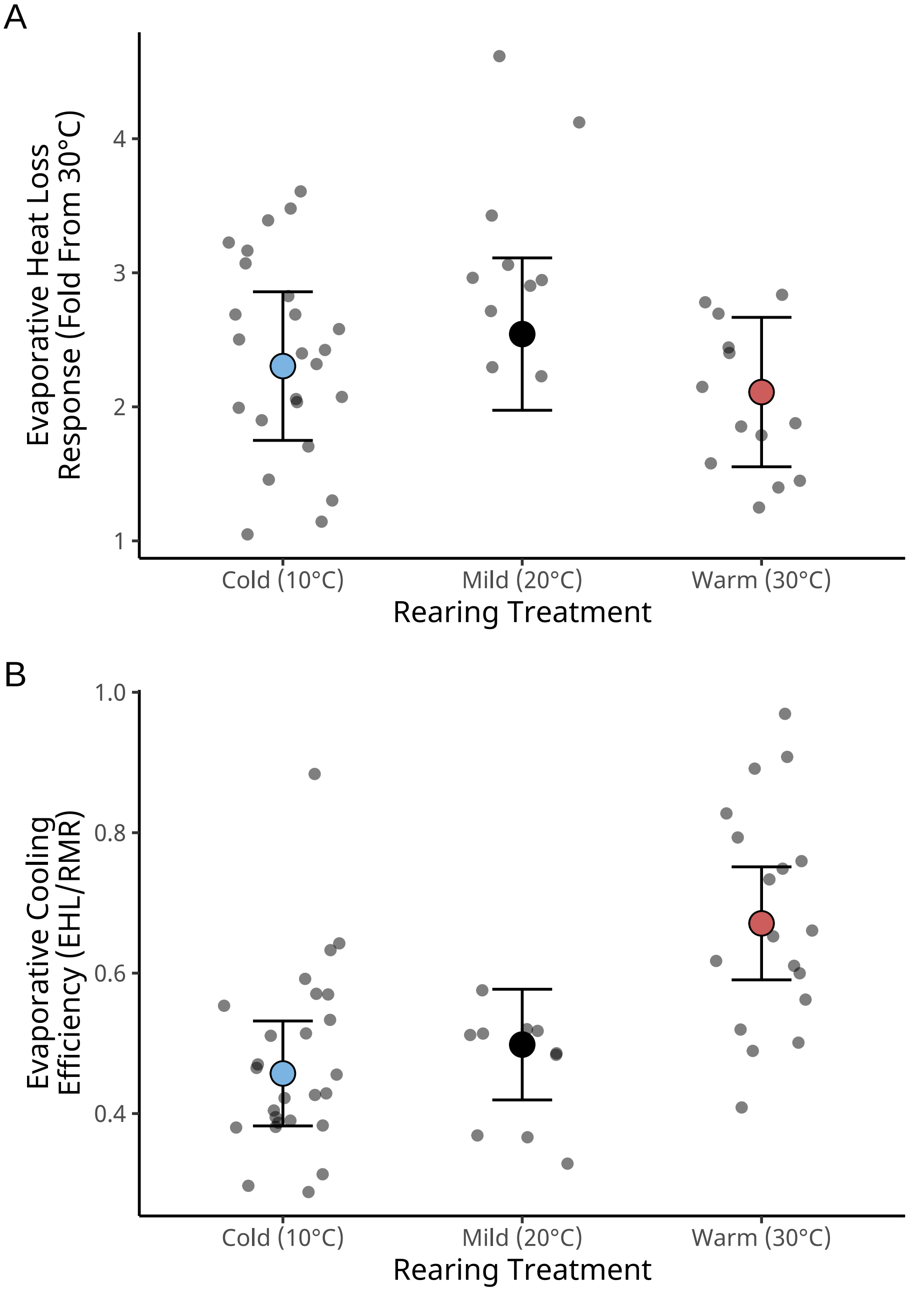

Figure 159: *Effects of rearing temperature on evaporative heat loss responses (panel A) and evaporative cooling efficiency (panel B) in developing Japanese quail (three weeks of age; n = 48). Evaporative heat loss responses represent fold increases in evaporative heat loss, in watts, between 30°C and 40°C. Evaporative cooling efficiency represents the ratio between evaporative heat loss (in watts) and metabolic heat production (in watts). Dashed lines indicate predicted relationships, as estimated from Bayesian path analyses. Ribbons represents +/- one standard error around predicted lines of best fit. Small dots indicate raw data points.*

To quantify direct and indirect effects of morphology or rearing treatment on final evaporative cooling phenotypes, we combine posterior estimates from each model within our path analyses. To ease interpretation and cross-comparability of effects, however, we first scale individual coefficients to represent the effect of changing a given predictor variable by one standard deviation (or categorical level) on a change, in standard deviations, of the response variable. Direct and indirect effects are both visualised as densities and

summarised at medians (± quantile intervals).

```
# Scaling coefficients

scaledBetasHeatLoss3 <- as.data.frame(ehlModel3Weeks) %>%
  mutate(
    b_mass_pretreatmentA = b_mass_pretreatmentA /
      sd(ehlModel3Weeks$data$mass),
    b_mass_pretreatmentC = b_mass_pretreatmentC /
      sd(ehlModel3Weeks$data$mass),
    b_tarsus_pretreatmentA = b_tarsus_pretreatmentA /
      sd(ehlModel3Weeks$data$tarsus),
    b_tarsus_pretreatmentC = b_tarsus_pretreatmentC /
      sd(ehlModel3Weeks$data$tarsus),
    b_tarsus_mass =
      (b_tarsus_mass * sd(ehlModel3Weeks$data$mass)) /
        sd(ehlModel3Weeks$data$tarsus),
    b_bill_pretreatmentA = b_bill_pretreatmentA /
      sd(ehlModel3Weeks$data$bill),
    b_tarsus_pretreatmentC = b_tarsus_pretreatmentC /
      sd(ehlModel3Weeks$data$bill),
    b_bill_mass =
      (b_bill_mass * sd(ehlModel3Weeks$data$mass)) /
        sd(ehlModel3Weeks$data$bill),
    b_foldEhl_mass =
      (b_foldEhl_mass * sd(ehlModel3Weeks$data$mass)) /
        sd(ehlModel3Weeks$data$foldEhl),
    b_foldEhl_tarsus =
      (b_foldEhl_tarsus * sd(ehlModel3Weeks$data$tarsus)) /
        sd(ehlModel3Weeks$data$foldEhl),
    b_foldEhl_bill =
      (b_foldEhl_bill * sd(ehlModel3Weeks$data$bill)) /
        sd(ehlModel3Weeks$data$foldEhl),
    b_foldEhl_pretreatmentA = b_foldEhl_pretreatmentA /
      sd(ehlModel3Weeks$data$foldEhl),
    b_foldEhl_pretreatmentC = b_foldEhl_pretreatmentC /
      sd(ehlModel3Weeks$data$foldEhl),
  )

scaledBetasEfficiency3 <- as.data.frame(efficiencyModel3Weeks) %>%
  mutate(
    b_mass_pretreatmentA = b_mass_pretreatmentA /
      sd(efficiencyModel3Weeks$data$mass),
    b_mass_pretreatmentC = b_mass_pretreatmentC /
      sd(efficiencyModel3Weeks$data$mass),
    b_tarsus_pretreatmentA = b_tarsus_pretreatmentA /
      sd(efficiencyModel3Weeks$data$tarsus),
    b_tarsus_pretreatmentC = b_tarsus_pretreatmentC /
      sd(efficiencyModel3Weeks$data$tarsus),
    b_tarsus_mass =
      (b_tarsus_mass * sd(efficiencyModel3Weeks$data$mass)) /
        sd(efficiencyModel3Weeks$data$tarsus),
    b_bill_pretreatmentA = b_bill_pretreatmentA /
      sd(efficiencyModel3Weeks$data$bill),
    b_bill_pretreatmentC = b_bill_pretreatmentC /
      sd(efficiencyModel3Weeks$data$bill),
    b_bill_mass =
      (b_bill_mass * sd(efficiencyModel3Weeks$data$mass)) /
        sd(efficiencyModel3Weeks$data$bill),
    b_ecc_mass =
      (b_ecc_mass * sd(efficiencyModel3Weeks$data$mass)) /
        sd(efficiencyModel3Weeks$data$ecc),
    b_ecc_tarsus =
      (b_ecc_tarsus * sd(efficiencyModel3Weeks$data$tarsus)) /
        sd(efficiencyModel3Weeks$data$ecc),
    b_ecc_bill =
      (b_ecc_bill * sd(efficiencyModel3Weeks$data$bill)) /
```

```
        sd(efficiencyModel3Weeks$data$ecc),
    b_ecc_pretreatmentA = b_ecc_pretreatmentA /
      sd(efficiencyModel3Weeks$data$ecc),
    b_ecc_pretreatmentC = b_ecc_pretreatmentC /
      sd(efficiencyModel3Weeks$data$ecc),
  )

# Calculating effects

fullEffectHeatLoss3 <- scaledBetasHeatLoss3 %>%
  mutate("Effects" = "Direct Effects") %>%
  mutate(
    "Body Mass" = b_foldEhl_mass,
    "Tarsus Length" = b_foldEhl_tarsus,
    "Bill Length" = b_foldEhl_bill,
    "Cold Rearing\n(10°C)" = b_foldEhl_pretreatmentA,
    "Warm Rearing\n(30°C)" = b_foldEhl_pretreatmentC
  ) %>%
  dplyr::select(
    Effects, `Body Mass`, `Tarsus Length`, `Bill Length`,
    `Cold Rearing\n(10°C)`, `Warm Rearing\n(30°C)`
  ) %>%
  rbind(
    .,
    scaledBetasHeatLoss3 %>%
      mutate("Effects" = "Indirect Effects") %>%
      mutate(
        "Body Mass" = (b_tarsus_mass * b_foldEhl_tarsus) +
          (b_bill_mass * b_foldEhl_bill),
        "Tarsus Length" = NA,
        "Bill Length" = NA,
        "Cold Rearing\n(10°C)" =
          (b_mass_pretreatmentA * b_foldEhl_mass) +
            (b_tarsus_pretreatmentA * b_foldEhl_tarsus) +
            (b_bill_pretreatmentA * b_foldEhl_bill) +
            (b_mass_pretreatmentA * b_tarsus_mass * b_foldEhl_tarsus) +
            (b_mass_pretreatmentA * b_bill_mass * b_foldEhl_bill),
        "Warm Rearing\n(30°C)" =
          (b_mass_pretreatmentC * b_foldEhl_mass) +
            (b_tarsus_pretreatmentC * b_foldEhl_tarsus) +
            (b_bill_pretreatmentC * b_foldEhl_bill) +
            (b_mass_pretreatmentC * b_tarsus_mass * b_foldEhl_tarsus) +
            (b_mass_pretreatmentC * b_bill_mass * b_foldEhl_bill),
      ) %>%
      dplyr::select(
        Effects, `Body Mass`, `Tarsus Length`, `Bill Length`,
        `Cold Rearing\n(10°C)`, `Warm Rearing\n(30°C)`
      )
  ) %>%
  rbind(., scaledBetasHeatLoss3 %>%
    mutate("Effects" = "Total Effects") %>%
    mutate(
      "Body Mass" =
        b_foldEhl_mass +
          b_tarsus_mass * b_foldEhl_tarsus,
      "Tarsus Length" =
        b_foldEhl_tarsus,
      "Bill Length" =
        b_foldEhl_bill,
      "Cold Rearing\n(10°C)" =
        (b_foldEhl_pretreatmentA) +
          (b_mass_pretreatmentA * b_foldEhl_mass) +
          (b_tarsus_pretreatmentA * b_foldEhl_tarsus) +
          (b_bill_pretreatmentA * b_foldEhl_bill) +
          (b_mass_pretreatmentA * b_tarsus_mass * b_foldEhl_tarsus) +
          (b_mass_pretreatmentA * b_bill_mass * b_foldEhl_bill),
      "Warm Rearing\n(30°C)" =
```

```
        (b_foldEhl_pretreatmentC) +
          (b_mass_pretreatmentC * b_foldEhl_mass) +
          (b_tarsus_pretreatmentC * b_foldEhl_tarsus) +
          (b_bill_pretreatmentC * b_foldEhl_bill) +
          (b_mass_pretreatmentC * b_tarsus_mass * b_foldEhl_tarsus) +
          (b_mass_pretreatmentC * b_bill_mass * b_foldEhl_bill),
    ) %>%
    dplyr::select(
      Effects, `Body Mass`, `Tarsus Length`, `Bill Length`,
      `Cold Rearing\n(10°C)`, `Warm Rearing\n(30°C)`
    )) %>%
  pivot_longer(c(-Effects), names_to = "var", values_to = "values") %>%
  mutate(var = factor(var,
    levels = c(
      "Body Mass",
      "Tarsus Length",
      "Bill Length",
      "Warm Rearing\n(30°C)",
      "Cold Rearing\n(10°C)"
    )
  ))

fullEffectEfficiency3 <- scaledBetasEfficiency3 %>%
  mutate("Effects" = "Direct Effects") %>%
  mutate(
    "Body Mass" = b_ecc_mass,
    "Tarsus Length" = b_ecc_tarsus,
    "Bill Length" = b_ecc_bill,
    "Cold Rearing\n(10°C)" = b_ecc_pretreatmentA,
    "Warm Rearing\n(30°C)" = b_ecc_pretreatmentC
  ) %>%
  dplyr::select(
    Effects, `Body Mass`, `Tarsus Length`, `Bill Length`,
    `Cold Rearing\n(10°C)`, `Warm Rearing\n(30°C)`
  ) %>%
  rbind(
    .,
    scaledBetasEfficiency3 %>%
      mutate("Effects" = "Indirect Effects") %>%
      mutate(
        "Body Mass" = (b_tarsus_mass * b_ecc_tarsus) +
          (b_bill_mass * b_ecc_bill),
        "Tarsus Length" = NA,
        "Bill Length" = NA,
        "Cold Rearing\n(10°C)" =
          (b_mass_pretreatmentA * b_ecc_mass) +
            (b_tarsus_pretreatmentA * b_ecc_tarsus) +
            (b_bill_pretreatmentA * b_ecc_bill) +
            (b_mass_pretreatmentA * b_tarsus_mass * b_ecc_tarsus) +
            (b_mass_pretreatmentA * b_bill_mass * b_ecc_bill),
        "Warm Rearing\n(30°C)" =
          (b_mass_pretreatmentC * b_ecc_mass) +
            (b_tarsus_pretreatmentC * b_ecc_tarsus) +
            (b_bill_pretreatmentC * b_ecc_bill) +
            (b_mass_pretreatmentC * b_tarsus_mass * b_ecc_tarsus) +
            (b_mass_pretreatmentC * b_bill_mass * b_ecc_bill),
      ) %>%
      dplyr::select(
        Effects, `Body Mass`, `Tarsus Length`, `Bill Length`,
        `Cold Rearing\n(10°C)`, `Warm Rearing\n(30°C)`
      )
  ) %>%
  rbind(., scaledBetasEfficiency3 %>%
    mutate("Effects" = "Total Effects") %>%
    mutate(
      "Body Mass" =
        b_ecc_mass +
```

```
          b_tarsus_mass * b_ecc_tarsus,
      "Tarsus Length" =
        b_ecc_tarsus,
      "Bill Length" =
        b_ecc_bill,
      "Cold Rearing\n(10°C)" =
        (b_ecc_pretreatmentA) +
          (b_mass_pretreatmentA * b_ecc_mass) +
          (b_tarsus_pretreatmentA * b_ecc_tarsus) +
          (b_bill_pretreatmentA * b_ecc_bill) +
          (b_mass_pretreatmentA * b_tarsus_mass * b_ecc_tarsus) +
          (b_mass_pretreatmentA * b_bill_mass * b_ecc_bill),
      "Warm Rearing\n(30°C)" =
        (b_ecc_pretreatmentC) +
          (b_mass_pretreatmentC * b_ecc_mass) +
          (b_tarsus_pretreatmentC * b_ecc_tarsus) +
          (b_bill_pretreatmentC * b_ecc_bill) +
          (b_mass_pretreatmentC * b_tarsus_mass * b_ecc_tarsus) +
          (b_mass_pretreatmentC * b_bill_mass * b_ecc_bill),
    ) %>%
    dplyr::select(
      Effects, `Body Mass`, `Tarsus Length`, `Bill Length`,
      `Cold Rearing\n(10°C)`, `Warm Rearing\n(30°C)`
    )) %>%
  pivot_longer(c(-Effects), names_to = "var", values_to = "values") %>%
  mutate(var = factor(var,
    levels = c(
      "Body Mass",
      "Tarsus Length",
      "Bill Length",
      "Warm Rearing\n(30°C)",
      "Cold Rearing\n(10°C)"
    )
  ))

# Plotting effects

fullEffectHeatLoss3Plot <- fullEffectHeatLoss3 %>%
  ggplot(aes(x = values, y = var, fill = var)) +
  facet_wrap(~Effects) +
  stat_halfeye(normalize = "xy", colour = "black", alpha = 0.7) +
  geom_vline(
    xintercept = 0, linetype = "dashed",
    colour = "black"
  ) +
  xlab("Effect on Fold Evaporative\nHeat Loss (standard deviations)") +
  scale_fill_manual(
    values =
      c("black", "grey25", "grey75", "#CD5C5C", "#7BB4E3")
  ) +
  theme_classic() +
  theme(
    legend.position = "none", axis.title.y = element_blank(),
    axis.text.y = element_text(
      size = 11, colour = "black",
      family = "Noto Sans"
    ),
    axis.title.x = element_text(family = "Noto Sans", hjust = -0.005),
    axis.text.x = element_text(family = "Noto Sans")
  )

fullEffectEfficiency3Plot <- fullEffectEfficiency3 %>%
  ggplot(aes(x = values, y = var, fill = var)) +
  facet_wrap(~Effects) +
  stat_halfeye(normalize = "xy", colour = "black", alpha = 0.7) +
  geom_vline(
    xintercept = 0, linetype = "dashed",
```

```
    colour = "black"
  ) +
  xlab("Effect on Evaporative Cooling\nEfficiency (standard deviations)") +
  scale_fill_manual(
    values =
      c("black", "grey25", "grey75", "#CD5C5C", "#7BB4E3")
  ) +
  theme_classic() +
  theme(
    legend.position = "none", axis.title.y = element_blank(),
    axis.text.y = element_text(
      size = 11, colour = "black",
      family = "Noto Sans"
    ),
    axis.title.x = element_text(family = "Noto Sans", hjust = -0.005),
    axis.text.x = element_text(family = "Noto Sans")
  )

(fullEffectHeatLoss3Plot /
  fullEffectEfficiency3Plot) +
  plot_annotation(tag_level = "A")
```

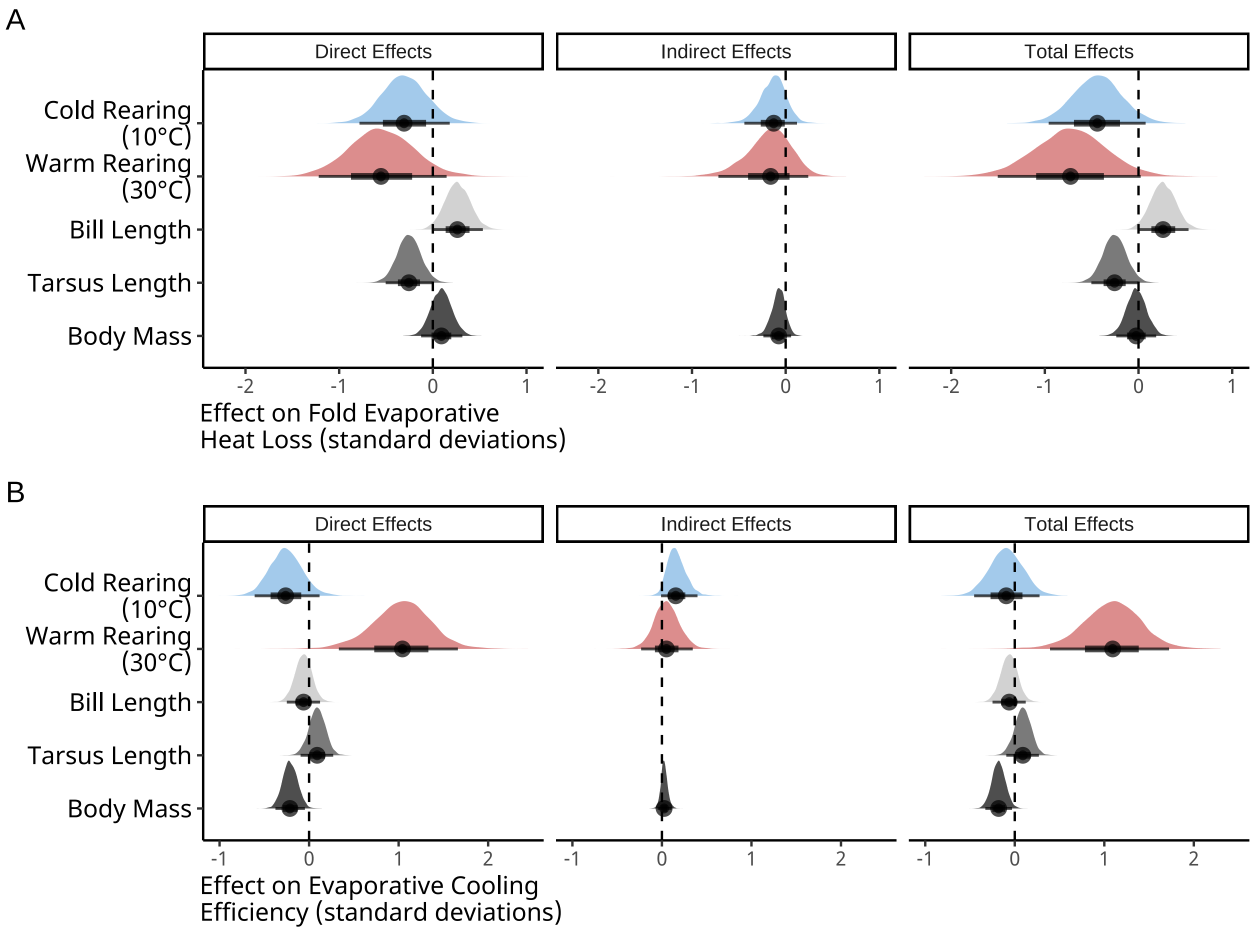

Figure 160: *Direct and indirect effects of rearing temperature and morphology (here, body mass, tarsus length and bill length) on evaporative heat loss responses (panel A) and evaporative cooling efficiency (panel B) in developing Japanese quail (three weeks of age; $n = 48$). Evaporative heat loss responses represent fold increases in evaporative heat loss, in watts, between 30°C and 40°C. Evaporative cooling efficiency represents the ratio between evaporative heat loss (in watts) and metabolic heat production (in watts). Effects indicate how much, in standard deviations, a response variables would be altered by changing a predictor variable by one standard deviation. Densities are derived from posteriors of Bayesian path analyses. Dashed lines indicate 0.*

```
# And printing outcomes

caption <- paste0("Direct, indirect, and total effects of morphology and ",
                  "rearing temperature on fold evaporative heat loss at 40°C",
                  "(relative to 30°C) of three week old Japanese quail. ",
                  "Effects are derived from ",
                  "a Bayesian path analysis and represent those predicted for a ",
                  "change in one standard deviation (or categorical level) of ",
                  "a given predictor on the standard deviation of metabolic ",
                  "slopes. Estimates indicate posterior medians and credible ",
                  "intervels (CIs) indicate quantile intervals."
)

heatLossResultsScaled3 <-
  fullEffectHeatLoss3 %>%
  filter(!is.na(values) & !is.nan(values)) %>%
  group_by(Effects, var) %>%
  summarise(
    "Estimate" = median(values),
```

```
    "50\\% CIs" = paste0(
      "[",
      round(
        quantile(values, probs = 0.1, type = 8),
        digits = 4
      ),
      ", ",
      round(
        quantile(values, probs = 0.9, type = 8),
        digits = 4
      ),
      "]"
    ),
    "95\\% CIs" = paste0(
      "[",
      round(
        quantile(values, probs = 0.025, type = 8),
        digits = 4
      ),
      ", ",
      round(
        quantile(values, probs = 0.975, type = 8),
        digits = 4
      ),
      "]"
    )
  ) %>%
  mutate("Effects" = gsub("[[:space:]].*", "", Effects)) %>%
  dplyr::select(
    "Predictor" = "var", "Effect Level" = "Effects",
    Estimate, `50\\% CIs`, `95\\% CIs`
  ) %>%
  arrange(Predictor, `Effect Level`) %>%
  kbl(.,
    longtable = T, booktabs = T, format = "latex", escape = FALSE,
    caption = caption
  ) %>%
  column_spec(column = c(1:2), width = "2.2cm") %>%
  column_spec(column = c(3:10), width = "1.9cm") %>%
  kable_styling(latex_options = "striped")

heatLossResultsScaled3
```

Table 97: *Direct, indirect, and total effects of morphology and rearing temperature on fold evaporative heat loss at 40°C(relative to 30°C) of three week old Japanese quail. Effects are derived from a Bayesian path analysis and represent those predicted for a change in one standard deviation (or categorical level) of a given predictor on the standard deviation of metabolic slopes. Estimates indicate posterior medians and credible intervels (CIs) indicate quantile intervals.*

Predictor	Effect Level	Estimate	50% CIs	95% CIs
Body Mass	Direct	0.0916735	[-0.0501, 0.2331]	[-0.1257, 0.3155]
Body Mass	Indirect	-0.0744355	[-0.1759, 0.011]	[-0.238, 0.0581]
Body Mass	Total	-0.0241831	[-0.1579, 0.1148]	[-0.2379, 0.1887]
Tarsus Length	Direct	-0.2555074	[-0.4159, -0.0939]	[-0.5035, -0.0028]
Tarsus Length	Total	-0.2555074	[-0.4159, -0.0939]	[-0.5035, -0.0028]
Bill Length	Direct	0.2621845	[0.0955, 0.4356]	[0.0056, 0.5342]
Bill Length	Total	0.2621845	[0.0955, 0.4356]	[0.0056, 0.5342]

Warm Rearing (30°C)	Direct	-0.5538577	[-0.9859, -0.1085]	[-1.2185, 0.1472]
Warm Rearing (30°C)	Indirect	-0.1609801	[-0.5021, 0.1077]	[-0.7187, 0.2419]
Warm Rearing (30°C)	Total	-0.7261611	[-1.2233, -0.2425]	[-1.5028, 0.0247]
Cold Rearing (10°C)	Direct	-0.3069252	[-0.6071, 0.0084]	[-0.7834, 0.1805]
Cold Rearing (10°C)	Indirect	-0.1277798	[-0.3209, 0.0311]	[-0.4411, 0.121]
Cold Rearing (10°C)	Total	-0.4395101	[-0.7738, -0.1076]	[-0.9593, 0.0761]

```
caption <- paste0("Direct, indirect, and total effects of morphology and ",
                  "rearing temperature on evaporative cooling efficiency ",
                  "at 40°C  of three week old Japanese quail. ",
                  "Evaporative cooling efficiency represents the ratio of ",
                  "evaporative heat loss (in W) to metabolic heat production ",
                  "(again, in W). Effects are derived from ",
                  "a Bayesian path analysis and represent those predicted for a ",
                  "change in one standard deviation (or categorical level) of ",
                  "a given predictor on the standard deviation of metabolic ",
                  "slopes. Estimates indicate posterior medians and credible ",
                  "intervels (CIs) indicate quantile intervals."
)

efficiencyResultsScaled3 <-
  fullEffectEfficiency3 %>%
  filter(!is.na(values) & !is.nan(values)) %>%
  group_by(Effects, var) %>%
  summarise(
    "Estimate" = median(values),
    "50\\% CIs" = paste0(
      "[",
      round(
        quantile(values, probs = 0.1, type = 8),
        digits = 4
      ),
      ", ",
      round(
        quantile(values, probs = 0.9, type = 8),
        digits = 4
      ),
      "]"
    ),
    "95\\% CIs" = paste0(
      "[",
      round(
        quantile(values, probs = 0.025, type = 8),
        digits = 4
      ),
      ", ",
      round(
        quantile(values, probs = 0.975, type = 8),
        digits = 4
      ),
      "]"
    )
  ) %>%
  mutate("Effects" = gsub("[[:space:]].*", "", Effects)) %>%
  dplyr::select(
    "Predictor" = "var", "Effect Level" = "Effects",
    Estimate, `50\\% CIs`, `95\\% CIs`
  ) %>%
  arrange(Predictor, `Effect Level`) %>%
  kbl(.,
```

```
    longtable = T, booktabs = T, format = "latex", escape = FALSE,
    caption = caption
  ) %>%
  column_spec(column = c(1:2), width = "2.2cm") %>%
  column_spec(column = c(3:10), width = "1.9cm") %>%
  kable_styling(latex_options = "striped")

efficiencyResultsScaled3
```

Table 98: *Direct, indirect, and total effects of morphology and rearing temperature on evaporative cooling efficiency at 40°C of three week old Japanese quail. Evaporative cooling efficiency represents the ratio of evaporative heat loss (in W) to metabolic heat production (again, in W). Effects are derived from a Bayesian path analysis and represent those predicted for a change in one standard deviation (or categorical level) of a given predictor on the standard deviation of metabolic slopes. Estimates indicate posterior medians and credible intervels (CIs) indicate quantile intervals.*

Predictor	Effect Level	Estimate	50% CIs	95% CIs
Body Mass	Direct	-0.2144580	[-0.3176, -0.1043]	[-0.376, -0.0464]
Body Mass	Indirect	0.0244689	[-0.0197, 0.074]	[-0.0485, 0.1082]
Body Mass	Total	-0.1801429	[-0.2754, -0.0839]	[-0.3291, -0.0303]
Tarsus Length	Direct	0.0900544	[-0.0278, 0.2052]	[-0.0948, 0.2694]
Tarsus Length	Total	0.0900544	[-0.0278, 0.2052]	[-0.0948, 0.2694]
Bill Length	Direct	-0.0618969	[-0.1826, 0.0546]	[-0.2487, 0.1227]
Bill Length	Total	-0.0618969	[-0.1826, 0.0546]	[-0.2487, 0.1227]
Warm Rearing (30°C)	Direct	1.0426860	[0.6129, 1.4302]	[0.3323, 1.6625]
Warm Rearing (30°C)	Indirect	0.0499558	[-0.1232, 0.2369]	[-0.2326, 0.3444]
Warm Rearing (30°C)	Total	1.0938602	[0.6715, 1.4778]	[0.3936, 1.7238]
Cold Rearing (10°C)	Direct	-0.2615054	[-0.4885, -0.0244]	[-0.6085, 0.1177]
Cold Rearing (10°C)	Indirect	0.1544234	[0.0408, 0.3031]	[-0.0099, 0.3975]
Cold Rearing (10°C)	Total	-0.0950498	[-0.3343, 0.1463]	[-0.4539, 0.277]

Finally, we estimate the tentative consequences of misalignment with Bergmann's and Allen's rule in the heat (i.e. by having an atypically large body or atypically short appendages). Consequences here represent changes in evaporative heat loss responses and evaporative cooling efficiency at 40°C relative to average.

```
data.frame(
  "Size" = c("Average", "Large (2x s.d. > mean)"),
  "pretreatment" = "B",
  "mass" = c(
    mean(ehlModel3Weeks$data$mass, na.rm = T),
    mean(ehlModel3Weeks$data$mass, na.rm = T) +
      2 * sd(ehlModel3Weeks$data$mass, na.rm = T)
  ),
  "tarsus" = 0,
  "bill" = 0,
  "batch" = "A"
) %>%
  mutate(
```

```
    "ehl" = predict(ehlModel3Weeks,
      newdata = .,
      resp = "foldEhl",
      re_form = NA,
      robust = TRUE
    )[, "Estimate"],
    "se1" = predict(ehlModel3Weeks,
      newdata = .,
      resp = "foldEhl",
      re_form = NA,
      robust = TRUE
    )[, "Est.Error"],
    "ecc" = predict(efficiencyModel3Weeks,
      newdata = .,
      resp = "ecc",
      re_form = NA,
      robust = TRUE
    )[, "Estimate"],
    "se2" = predict(efficiencyModel3Weeks,
      newdata = .,
      resp = "ecc",
      re_form = NA,
      robust = TRUE
    )[, "Est.Error"]
  ) %>%
  mutate_if(is.numeric, round, digits = 4) %>%
  mutate(
    "Body Size" = Size,
    "Evaporative Heat Loss" =
      paste0(ehl, " [", se1, "]"),
    "Evaporative Cooling Efficiency" =
      paste0(ecc, " [", se2, "]")
  ) %>%
  dplyr::select(
    `Body Size`, `Evaporative Heat Loss`, `Evaporative Cooling Efficiency`
  ) %>%
  kbl(.,
    longtable = T, booktabs = T, format = "latex",
    caption = paste0("Comparison of estimated evaporative heat loss rate ",
                     "and evaporative cooling efficiency among three week old ",
                     "Japanese quail at 40°C and varying body masses."),
    escape = FALSE
  ) %>%
  column_spec(column = c(1:10), width = "2.5cm")  %>%
  kable_styling(latex_options = "striped")
```

Table 99: *Comparison of estimated evaporative heat loss rate and evaporative cooling efficiency among three week old Japanese quail at 40°C and varying body masses.*

Body Size	Evaporative Heat Loss	Evaporative Cooling Efficiency
Average	2.5531 [0.5551]	0.5 [0.0769]
Large (2x s.d. > mean)	2.6961 [0.5775]	0.4281 [0.0805]

```
data.frame(
  "Size" = c("Average", "Short (2x s.d. < mean)"),
  "pretreatment" = "B",
  "tarsus" = c(
    mean(ehlModel3Weeks$data$tarsus, na.rm = T),
    mean(ehlModel3Weeks$data$tarsus, na.rm = T) -
      2 * sd(ehlModel3Weeks$data$tarsus, na.rm = T)
  ),
  "mass" = 0,
  "bill" = 0,
```

```
  "batch" = "A"
) %>%
  mutate(
    "ehl" = predict(ehlModel3Weeks,
      newdata = .,
      resp = "foldEhl",
      re_form = NA,
      robust = TRUE
    )[, "Estimate"],
    "se1" = predict(ehlModel3Weeks,
      newdata = .,
      resp = "foldEhl",
      re_form = NA,
      robust = TRUE
    )[, "Est.Error"],
    "ecc" = predict(efficiencyModel3Weeks,
      newdata = .,
      resp = "ecc",
      re_form = NA,
      robust = TRUE
    )[, "Estimate"],
    "se2" = predict(efficiencyModel3Weeks,
      newdata = .,
      resp = "ecc",
      re_form = NA,
      robust = TRUE
    )[, "Est.Error"]
  ) %>%
  mutate_if(is.numeric, round, digits = 4) %>%
  mutate(
    "Tarsus Length" = Size,
    "Evaporative Heat Loss" =
      paste0(ehl, " [", se1, "]"),
    "Evaporative Cooling Efficiency" =
      paste0(ecc, " [", se2, "]")
  ) %>%
  dplyr::select(`Tarsus Length`, `Evaporative Heat Loss`,
         `Evaporative Cooling Efficiency`) %>%
  kbl(.,
    longtable = T, booktabs = T, format = "latex",
    caption = paste0("Comparison of estimated evaporative heat loss rate ",
                     "and evaporative cooling efficiency among three week old ",
                     "Japanese quail at 40°C and varying tarsus lengths."),
    escape = FALSE
  ) %>%
  column_spec(column = c(1:10), width = "2.5cm")  %>%
  kable_styling(latex_options = "striped")
```

Table 100: *Comparison of estimated evaporative heat loss rate and evaporative cooling efficiency among three week old Japanese quail at 40°C and varying tarsus lengths.*

Tarsus Length	Evaporative Heat Loss	Evaporative Cooling Efficiency
Average	2.5996 [0.5625]	0.495 [0.0771]
Short (2x s.d. < mean)	2.9902 [0.5868]	0.4649 [0.0808]

```
data.frame(
  "Size" = c("Average", "Short (2x s.d. < mean)"),
  "pretreatment" = "B",
  "bill" = c(
    mean(ehlModel3Weeks$data$bill, na.rm = T),
    mean(ehlModel3Weeks$data$bill, na.rm = T) -
      2 * sd(ehlModel3Weeks$data$bill, na.rm = T)
  ),
```

```
  "mass" = 0,
  "tarsus" = 0,
  "batch" = "A"
) %>%
  mutate(
    "ehl" = predict(ehlModel3Weeks,
      newdata = .,
      resp = "foldEhl",
      re_form = NA,
      robust = TRUE
    )[, "Estimate"],
    "se1" = predict(ehlModel3Weeks,
      newdata = .,
      resp = "foldEhl",
      re_form = NA,
      robust = TRUE
    )[, "Est.Error"],
    "ecc" = predict(efficiencyModel3Weeks,
      newdata = .,
      resp = "ecc",
      re_form = NA,
      robust = TRUE
    )[, "Estimate"],
    "se2" = predict(efficiencyModel3Weeks,
      newdata = .,
      resp = "ecc",
      re_form = NA,
      robust = TRUE
    )[, "Est.Error"]
  ) %>%
  mutate_if(is.numeric, round, digits = 4) %>%
  mutate(
    "Bill Length" = Size,
    "Evaporative Heat Loss" =
      paste0(ehl, " [", se1, "]"),
    "Evaporative Cooling Efficiency" =
      paste0(ecc, " [", se2, "]")
  ) %>%
  dplyr::select(`Bill Length`, `Evaporative Heat Loss`,
         `Evaporative Cooling Efficiency`) %>%
  kbl(.,
    longtable = T, booktabs = T, format = "latex",
    caption = paste0("Comparison of estimated evaporative heat loss rate ",
                     "and evaporative cooling efficiency among three week old ",
                     "Japanese quail at 40°C and varying bill lengths."),
    escape = FALSE
  ) %>%
  column_spec(column = c(1:10), width = "2.5cm")  %>%
  kable_styling(latex_options = "striped")
```

Table 101: *Comparison of estimated evaporative heat loss rate and evaporative cooling efficiency among three week old Japanese quail at 40°C and varying bill lengths.*

Bill Length	Evaporative Heat Loss	Evaporative Cooling Efficiency
Average	2.586 [0.5737]	0.4966 [0.0799]
Short (2x s.d. < mean)	2.1801 [0.5796]	0.5197 [0.0845]

Modelling for adults

In this subsection, we evaluate effects of rearing temperature and morphology on evaporative heat loss responses and evaporative cooling efficiency in mature Japanese quail (here, eight weeks of age). To do so, we repeat our above described path analyses but while: (1) only using data obtained from our mature quail,

and (2) broadening and shifting our model priors to account for changes in morphology and physiology that have been observed between early development and maturity (see Persson et al, 2024). Model equations are repeated here for convenience.

$$Body\ Mass_j \sim \beta_{a0} + \beta_{a1} \cdot Cold\ Rearing_j + \beta_{a2} \cdot Warm\ Rearing_j + \mu_{0a} + \epsilon_a$$

$$Tarsus\ Length_j \sim \beta_{b0} + \beta_{b1} \cdot Cold\ Rearing_j + \beta_{b2} \cdot Warm\ Rearing_j + \beta_{b3} \cdot Body\ Mass_j + \mu_{0b} + \epsilon_b$$

$$Bill\ Length_j \sim \beta_{c0} + \beta_{c1} \cdot Cold\ Rearing_j + \beta_{c2} \cdot Warm\ Rearing_j + \beta_{c3} \cdot Body\ Mass_j + \mu_{0c} + \epsilon_c$$

and either:

$$Fold\ Evaporative\ Heat\ Loss_j \sim \beta_{d0} + \beta_{d1} \cdot Cold\ Rearing_j + \beta_{d2} \cdot Warm\ Rearing_j + \beta_{d3} \cdot Body\ Mass_j + \beta_{d4} \cdot Tarsus\ Length_j + \beta_{d5} \cdot Bill\ Length_j + \mu_{0d} + \epsilon_d$$

where:

$$Fold\ Evaporative\ Heat\ Loss_j = \frac{Evaporative\ Heat\ Loss_{40^\circ \mathrm{C}j}}{Evaporative\ Heat\ Loss_{30^\circ \mathrm{C}j}}$$

or:

$$Evaporative\ Cooling\ Efficiency_j \sim \beta_{d0} + \beta_{d1} \cdot Cold\ Rearing_j + \beta_{d2} \cdot Warm\ Rearing_j + \beta_{d3} \cdot Body\ Mass_j + \beta_{d4} \cdot Tarsus\ Length_j + \beta_{d5} \cdot Bill\ Length_j + \mu_{0d} + \epsilon_d$$

where evaporative cooling efficiency is measured as:

$$Evaporative\ Cooling\ Efficiency_j = \frac{Evaporative\ Heat\ Loss_{40^\circ \mathrm{C}j}}{Metabolic\ Heat\ Production_{40^\circ \mathrm{C}j}}$$

Model terms remain as previously described.

Priors for our new path analyses are therefore as follows:

$$\beta_{a0} \sim \mathcal{N}(0, 10)$$
$$\beta_{a1} \sim \mathcal{N}(0, 25)$$
$$\beta_{a2} \sim \mathcal{N}(0, 25)$$
$$\mu_{0a} \sim \exp(2.5)$$
$$\epsilon_a \sim \exp(0.15)$$

$$\beta_{b0} \sim \mathcal{N}(0, 2.5)$$
$$\beta_{b1} \sim \mathcal{N}(0, 2.5)$$

$$\beta_{b2} \sim \mathcal{N}(0, 2.5)$$
$$\beta_{b3} \sim S\mathcal{N}(0, 0.25, 5)$$
$$\mu_{0b} \sim \exp(2)$$
$$\epsilon_b \sim \exp(1)$$

$$\beta_{c0} \sim \mathcal{N}(0, 1)$$
$$\beta_{c1} \sim \mathcal{N}(0, 0.5)$$
$$\beta_{c2} \sim \mathcal{N}(0, 0.5)$$
$$\beta_{c3} \sim S\mathcal{N}(0, 0.25, 5)$$
$$\mu_{0c} \sim \exp(5)$$
$$\epsilon_c \sim \exp(2.5)$$

and either:

$$\beta_{d0} \sim \mathcal{N}(2.5, 1)$$
$$\beta_{d1} \sim \mathcal{N}(0, 0.5)$$
$$\beta_{d2} \sim \mathcal{N}(0, 0.5)$$
$$\beta_{d3} \sim \mathcal{N}(0, 0.015)$$
$$\beta_{d4} \sim \mathcal{N}(0, 0.2)$$
$$\beta_{d5} \sim \mathcal{N}(0, 0.5)$$
$$\mu_{0d} \sim \exp(5)$$
$$\epsilon_d \sim \exp(5)$$

for parameters predicting evaporative heat loss responses, and:

$$\beta_{d0} \sim \mathcal{N}(0.75, 0.2)$$
$$\beta_{d1} \sim \mathcal{N}(0, 0.25)$$
$$\beta_{d2} \sim \mathcal{N}(0, 0.25)$$
$$\beta_{d3} \sim \mathcal{N}(0, 0.01)$$
$$\beta_{d4} \sim \mathcal{N}(0, 0.1)$$
$$\mu_{0d} \sim \exp(15)$$
$$\epsilon_d \sim \exp(5)$$

for parameters predicting evaporative cooling efficiency.

Similar to our above analyses, suitability of priors is first assessed using prior predictive checks.

```
ehlModel8WeeksPPCheck <- brm(
  data = vh2o %>%
    filter(week == "8" & Ta %in% c(30, 40)) %>%
    dplyr::select(Ta, ring, pretreatment,
           mass, tarsusLengthMean, billLengthMean,
           ehl, "batch" = exp) %>%
    pivot_wider(
      id_cols = c("ring", "batch",
                  "pretreatment", "mass",
                  "tarsusLengthMean", "billLengthMean"),
      values_from = "ehl",
      names_from = "Ta"
    ) %>%
    mutate("foldEhl" = `40` / `30`) %>%
    mutate(pretreatment = ifelse(pretreatment == "neutral", "B",
      ifelse(pretreatment == "cold", "A", "C")
    )) %>%
    mutate(pretreatment = factor(pretreatment,
      levels = c("B", "A", "C")
    )) %>%
    distinct() %>%
    mutate(
      mass = mass - mean(mass, na.rm = T),
      tarsus = tarsusLengthMean - mean(tarsusLengthMean, na.rm = T),
      bill = billLengthMean - mean(billLengthMean, na.rm = T)
    ),
  family = "gaussian",
  bf(mass ~ pretreatment + (1 | batch)) +
    bf(tarsus ~ mass + pretreatment + (1 | batch)) +
    bf(bill ~ mass + pretreatment + (1 | batch)) +
    bf(foldEhl ~ mass + tarsus + bill + pretreatment + (1 | batch)) +
    set_rescor(FALSE),
  prior = c(
    set_prior("normal(0, 10)",
        class = "Intercept",
        resp = "mass"
      ),
      set_prior("normal(0, 25)",
        class = "b",
        coef = "pretreatmentA",
        resp = "mass"
      ),
      set_prior("normal(0, 25)",
        class = "b",
        coef = "pretreatmentC",
        resp = "mass"
      ),
      set_prior("exponential(2.5)",
        class = "sd",
        group = "batch",
        resp = "mass"
      ),
      set_prior("exponential(0.05)",
        class = "sigma",
        resp = "mass"
      ),
      set_prior("normal(0, 3)",
        class = "Intercept",
        resp = "tarsus"
      ),
      set_prior("normal(0, 3)",
        class = "b",
        coef = "pretreatmentA",
        resp = "tarsus"
      ),
      set_prior("normal(0, 3)",
        class = "b",
```

```
      coef = "pretreatmentC",
      resp = "tarsus"
    ),
    set_prior("skew_normal(0, 0.25, 5)",
      class = "b",
      coef = "mass",
      resp = "tarsus"
    ),
    set_prior("exponential(2)",
      class = "sd",
      group = "batch",
      resp = "tarsus"
    ),
    set_prior("exponential(0.75)",
      class = "sigma",
      resp = "tarsus"
    ),
    set_prior("normal(0, 1)",
      class = "Intercept",
      resp = "bill"
    ),
   set_prior("normal(0, 0.5)",
      class = "b",
      coef = "pretreatmentA",
      resp = "bill"
    ),
    set_prior("normal(0, 0.5)",
      class = "b",
      coef = "pretreatmentC",
      resp = "bill"
    ),
    set_prior("skew_normal(0, 0.25, 5)",
      class = "b",
      coef = "mass",
      resp = "bill"
    ),
    set_prior("exponential(5)",
      class = "sd",
      group = "batch",
      resp = "bill"
    ),
    set_prior("exponential(2.5)",
      class = "sigma",
      resp = "bill"
    ),
  set_prior("normal(2.5, 1)",
    class = "Intercept",
    resp = "foldEhl"
  ),
  set_prior("normal(0, 0.5)",
    class = "b",
    coef = "pretreatmentA",
    resp = "foldEhl"
  ),
  set_prior("normal(0, 0.5)",
    class = "b",
    coef = "pretreatmentC",
    resp = "foldEhl"
  ),
  set_prior("normal(0, 0.015)",
    class = "b",
    coef = "mass",
    resp = "foldEhl"
  ),
  set_prior("normal(0, 0.2)",
    class = "b",
    coef = "tarsus",
```

```
      resp = "foldEhl"
    ),
    set_prior("normal(0, 0.5)",
      class = "b",
      coef = "bill",
      resp = "foldEhl"
    ),
    set_prior("exponential(5)",
      class = "sd",
      group = "batch",
      resp = "foldEhl"
    ),
    set_prior("exponential(5)",
      class = "sigma",
      resp = "foldEhl"
    )
  ),
  iter = 50000, warmup = 10000, cores = 4, chains = 4, thin = 20,
  control = list(adapt_delta = .98, max_treedepth = 14),
  sample_prior = "only",
  silent = TRUE, refresh = 0,
  file = "./models/_heatLossModel8WeeksPPCheck.Rds",
)

pp1 <- pp_check2(
  ehlModel8WeeksPPCheck, resp = "foldEhl",
  xlab = "Evaporative Heat\nLoss Responses (Fold From 30°C)"
  ) +
  theme(legend.position = "none")

efficiencyModel8WeeksPPCheck <- brm(
  data = vh2o %>%
    filter(week == "8" & Ta == 40) %>%
    dplyr::select(Ta, ring, pretreatment,
      mass, tarsusLengthMean, billLengthMean, ecc,
      "batch" = exp
    ) %>%
    mutate(
      pretreatment =
        ifelse(pretreatment == "neutral", "B",
          ifelse(pretreatment == "cold", "A", "C")
        )
    ) %>%
    mutate(pretreatment = factor(pretreatment,
      levels = c("B", "A", "C")
    )) %>%
    distinct() %>%
    mutate(
      mass = mass - mean(mass, na.rm = T),
      tarsus = tarsusLengthMean -
        mean(tarsusLengthMean, na.rm = T),
      bill = billLengthMean -
        mean(billLengthMean, na.rm = T)
    ),
  family = "gaussian",
  bf(mass ~ pretreatment + (1 | batch)) +
    bf(tarsus ~ mass + pretreatment + (1 | batch)) +
    bf(bill ~ mass + pretreatment + (1 | batch)) +
    bf(ecc ~ mass + tarsus + bill + pretreatment + (1 | batch)) +
    set_rescor(FALSE),
  prior = c(
    set_prior("normal(0, 10)",
        class = "Intercept",
        resp = "mass"
      ),
      set_prior("normal(0, 25)",
        class = "b",
```

```
      coef = "pretreatmentA",
      resp = "mass"
    ),
    set_prior("normal(0, 25)",
      class = "b",
      coef = "pretreatmentC",
      resp = "mass"
    ),
    set_prior("exponential(2.5)",
      class = "sd",
      group = "batch",
      resp = "mass"
    ),
    set_prior("exponential(0.05)",
      class = "sigma",
      resp = "mass"
    ),
    set_prior("normal(0, 3)",
      class = "Intercept",
      resp = "tarsus"
    ),
    set_prior("normal(0, 3)",
      class = "b",
      coef = "pretreatmentA",
      resp = "tarsus"
    ),
    set_prior("normal(0, 3)",
      class = "b",
      coef = "pretreatmentC",
      resp = "tarsus"
    ),
    set_prior("skew_normal(0, 0.25, 5)",
      class = "b",
      coef = "mass",
      resp = "tarsus"
    ),
    set_prior("exponential(2)",
      class = "sd",
      group = "batch",
      resp = "tarsus"
    ),
    set_prior("exponential(0.75)",
      class = "sigma",
      resp = "tarsus"
    ),
    set_prior("normal(0, 1)",
      class = "Intercept",
      resp = "bill"
    ),
   set_prior("normal(0, 0.5)",
      class = "b",
      coef = "pretreatmentA",
      resp = "bill"
    ),
    set_prior("normal(0, 0.5)",
      class = "b",
      coef = "pretreatmentC",
      resp = "bill"
    ),
    set_prior("skew_normal(0, 0.25, 5)",
      class = "b",
      coef = "mass",
      resp = "bill"
    ),
    set_prior("exponential(5)",
      class = "sd",
      group = "batch",
```

```
        resp = "bill"
      ),
      set_prior("exponential(2.5)",
        class = "sigma",
        resp = "bill"
      ),
    set_prior("normal(0.75, 0.2)",
      class = "Intercept",
      resp = "ecc"
    ),
    set_prior("normal(0, 0.25)",
      class = "b",
      coef = "pretreatmentA",
      resp = "ecc"
    ),
    set_prior("normal(0, 0.25)",
      class = "b",
      coef = "pretreatmentC",
      resp = "ecc"
    ),
    set_prior("normal(0, 0.01)",
      class = "b",
      coef = "mass",
      resp = "ecc"
    ),
    set_prior("normal(0, 0.1)",
      class = "b",
      coef = "tarsus",
      resp = "ecc"
    ),
    set_prior("normal(0, 0.15)",
      class = "b",
      coef = "bill",
      resp = "ecc"
    ),
    set_prior("exponential(15)",
      class = "sd",
      group = "batch",
      resp = "ecc"
    ),
    set_prior("exponential(5)",
      class = "sigma",
      resp = "ecc"
    )
  ),
  iter = 50000, warmup = 10000, cores = 4, chains = 4, thin = 20,
  control = list(adapt_delta = .98, max_treedepth = 14),
  silent = TRUE, refresh = 0,
  sample_prior = "only",
  file = "./models/_efficiencyModel8WeeksPPCheck.Rds"
)

pp2 <- pp_check2(efficiencyModel8WeeksPPCheck, resp = "ecc",
                 xlab = "Evaporative Cooling\nEfficiency (EHL/RMR)")

pp1 + pp2 + plot_annotation(tag_levels = "A")
```

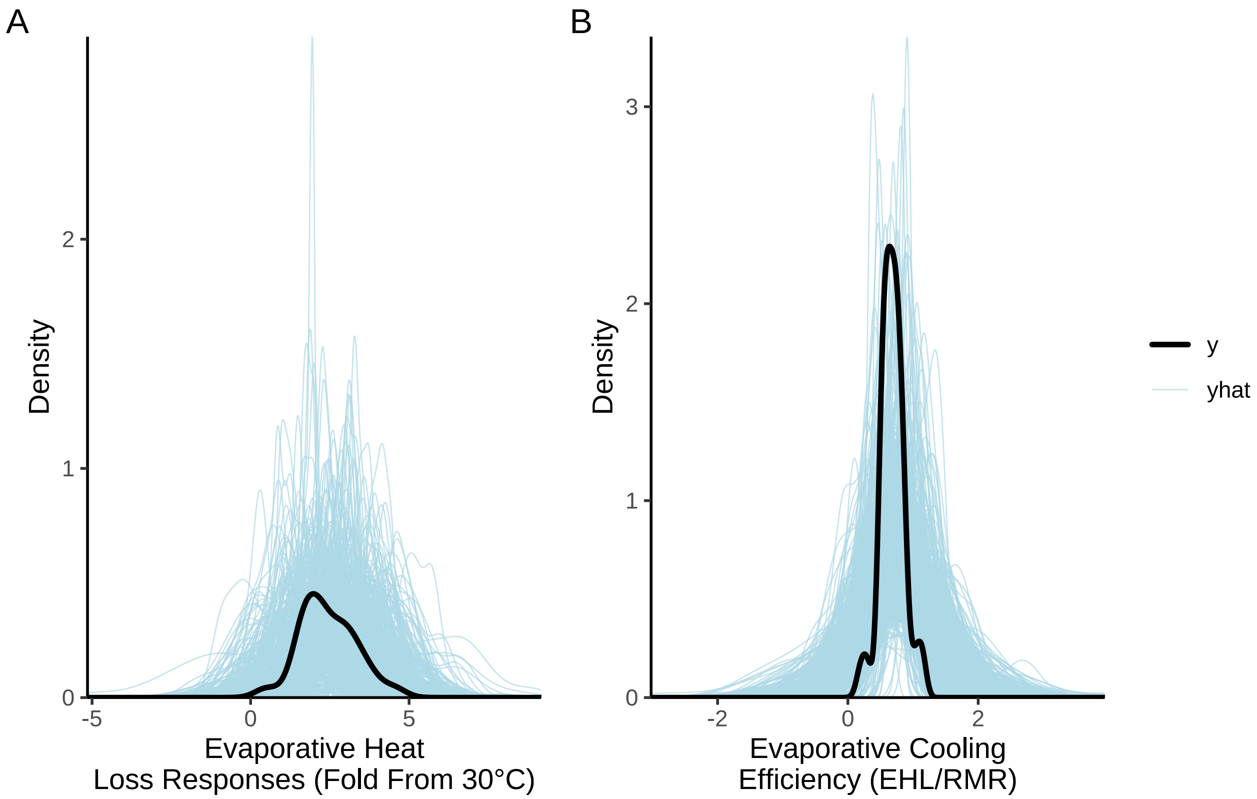

Figure 161: *Prior predictive checks for two Bayesian path analyses ultimately predicting evaporative heat loss ('EHL'; fold from that observed at 30°C in W) and evaporative cooling efficiency ('ECE'; the ratio of evaporative heat-loss to metabolic heat production, each in W) in eight week old Japanese quail. Black lines represent true EHL and ECE densities while blue lines represent densities estimated from model priors alone.*

Predictions from priors generally capture true data densities well. We thus proceed with their use in the construction of full models below.

```
ehlModel8Weeks <- brm(
  data = vh2o %>%
    filter(week == "8" & Ta %in% c(30, 40)) %>%
    dplyr::select(Ta, ring, pretreatment,
           mass, tarsusLengthMean, billLengthMean,
           ehl, "batch" = exp) %>%
    pivot_wider(
      id_cols = c("ring", "batch",
                  "pretreatment", "mass",
                  "tarsusLengthMean", "billLengthMean"),
      values_from = "ehl",
      names_from = "Ta"
    ) %>%
    mutate("foldEhl" = `40` / `30`) %>%
    mutate(pretreatment = ifelse(pretreatment == "neutral", "B",
      ifelse(pretreatment == "cold", "A", "C")
    )) %>%
    mutate(pretreatment = factor(pretreatment,
      levels = c("B", "A", "C")
    )) %>%
    distinct() %>%
    mutate(
      mass = mass - mean(mass, na.rm = T),
      tarsus = tarsusLengthMean - mean(tarsusLengthMean, na.rm = T),
      bill = billLengthMean - mean(billLengthMean, na.rm = T)
```

```
    ),
  family = "gaussian",
  bf(mass ~ pretreatment + (1 | batch)) +
    bf(tarsus ~ mass + pretreatment + (1 | batch)) +
    bf(bill ~ mass + pretreatment + (1 | batch)) +
    bf(foldEhl ~ mass + tarsus + bill + pretreatment + (1 | batch)) +
    set_rescor(FALSE),
  prior = c(
    set_prior("normal(0, 10)",
        class = "Intercept",
        resp = "mass"
      ),
      set_prior("normal(0, 25)",
        class = "b",
        coef = "pretreatmentA",
        resp = "mass"
      ),
      set_prior("normal(0, 25)",
        class = "b",
        coef = "pretreatmentC",
        resp = "mass"
      ),
      set_prior("exponential(2.5)",
        class = "sd",
        group = "batch",
        resp = "mass"
      ),
      set_prior("exponential(0.05)",
        class = "sigma",
        resp = "mass"
      ),
      set_prior("normal(0, 3)",
        class = "Intercept",
        resp = "tarsus"
      ),
      set_prior("normal(0, 3)",
        class = "b",
        coef = "pretreatmentA",
        resp = "tarsus"
      ),
      set_prior("normal(0, 3)",
        class = "b",
        coef = "pretreatmentC",
        resp = "tarsus"
      ),
      set_prior("skew_normal(0, 0.25, 5)",
        class = "b",
        coef = "mass",
        resp = "tarsus"
      ),
      set_prior("exponential(2)",
        class = "sd",
        group = "batch",
        resp = "tarsus"
      ),
      set_prior("exponential(0.75)",
        class = "sigma",
        resp = "tarsus"
      ),
      set_prior("normal(0, 1)",
        class = "Intercept",
        resp = "bill"
      ),
     set_prior("normal(0, 0.5)",
        class = "b",
        coef = "pretreatmentA",
        resp = "bill"
```

```
      ),
      set_prior("normal(0, 0.5)",
        class = "b",
        coef = "pretreatmentC",
        resp = "bill"
      ),
      set_prior("skew_normal(0, 0.25, 5)",
        class = "b",
        coef = "mass",
        resp = "bill"
      ),
      set_prior("exponential(5)",
        class = "sd",
        group = "batch",
        resp = "bill"
      ),
      set_prior("exponential(2.5)",
        class = "sigma",
        resp = "bill"
      ),
    set_prior("normal(2.5, 1)",
      class = "Intercept",
      resp = "foldEhl"
    ),
    set_prior("normal(0, 0.5)",
      class = "b",
      coef = "pretreatmentA",
      resp = "foldEhl"
    ),
    set_prior("normal(0, 0.5)",
      class = "b",
      coef = "pretreatmentC",
      resp = "foldEhl"
    ),
    set_prior("normal(0, 0.015)",
      class = "b",
      coef = "mass",
      resp = "foldEhl"
    ),
    set_prior("normal(0, 0.2)",
      class = "b",
      coef = "tarsus",
      resp = "foldEhl"
    ),
    set_prior("normal(0, 0.5)",
      class = "b",
      coef = "bill",
      resp = "foldEhl"
    ),
    set_prior("exponential(5)",
      class = "sd",
      group = "batch",
      resp = "foldEhl"
    ),
    set_prior("exponential(5)",
      class = "sigma",
      resp = "foldEhl"
    )
  ),
  iter = 50000, warmup = 10000, cores = 4, chains = 4, thin = 20,
  control = list(adapt_delta = .97, max_treedepth = 14),
  silent = TRUE, refresh = 0,
  file = "./models/_heatLossModel8Weeks.Rds",
)

pp1a <- mcmc_neff(neff_ratio(ehlModel8Weeks)) +
  xlab(
```

```
    TeX("$\\overset{Evaporative~Heat~Loss}{Response~(N_{eff}/N})$")
  ) +
  theme_classic() +
  theme(
    axis.text.y = element_blank(),
    axis.ticks.y = element_blank(),
    legend.position = "none"
  )

pp1b <- mcmc_rhat(rhat(ehlModel8Weeks)) +
  xlab(
    TeX("$\\overset{Evaporative~Heat~Loss}{Response~(\\hat{R}})$")
  ) +
  theme_classic() +
  theme(
    axis.text.y = element_blank(),
    axis.ticks.y = element_blank(),
    legend.position = "none"
  )

pp1c <- pp_check2(ehlModel8Weeks,
  resp = "foldEhl",
  xlab = "Evaporative Heat\nLoss Response (Fold From 30°C)"
) +
  theme(legend.position = "none")

pp1d <- ehlModel8Weeks$data %>%
  mutate(
    "Fit" = fitted(ehlModel8Weeks, resp = "foldEhl")[, "Estimate"],
    "FitSE" = fitted(ehlModel8Weeks, resp = "foldEhl")[, "Est.Error"]
  ) %>%
  ggplot(aes(x = Fit, y = foldEhl)) +
  geom_errorbarh(aes(xmin = Fit - FitSE, xmax = Fit + FitSE),
    height = 0.25, colour = "black", alpha = 0.8
  ) +
  geom_point(
    size = 2, pch = 21, colour = "black", fill = "lightblue2",
    alpha = 0.8
  ) +
  geom_smooth(
    method = "lm", colour = "black", formula = y ~ 0 + x,
    linetype = "dashed", se = FALSE
  ) +
  xlab("Fitted Evaporative Heat\nLoss Response (Fold from 30°C)") +
  ylab("Evaporative Heat Loss\nResponse(Fold from 30°C)") +
  theme_classic()

efficiencyModel8Weeks <- brm(
  data = vh2o %>%
    filter(week == "8" & Ta == 40) %>%
    dplyr::select(Ta, ring, pretreatment,
      mass, tarsusLengthMean, billLengthMean, ecc,
      "batch" = exp
    ) %>%
    mutate(
      pretreatment =
        ifelse(pretreatment == "neutral", "B",
          ifelse(pretreatment == "cold", "A", "C")
        )
    ) %>%
    mutate(pretreatment = factor(pretreatment,
      levels = c("B", "A", "C")
    )) %>%
    distinct() %>%
    mutate(
      mass = mass - mean(mass, na.rm = T),
      tarsus = tarsusLengthMean -
```

```
      mean(tarsusLengthMean, na.rm = T),
    bill = billLengthMean -
      mean(billLengthMean, na.rm = T)
  ),
  family = "gaussian",
  bf(mass ~ pretreatment + (1 | batch)) +
    bf(tarsus ~ mass + pretreatment + (1 | batch)) +
    bf(bill ~ mass + pretreatment + (1 | batch)) +
    bf(ecc ~ mass + tarsus + bill + pretreatment + (1 | batch)) +
    set_rescor(FALSE),
  prior = c(
    set_prior("normal(0, 10)",
        class = "Intercept",
        resp = "mass"
      ),
      set_prior("normal(0, 25)",
        class = "b",
        coef = "pretreatmentA",
        resp = "mass"
      ),
      set_prior("normal(0, 25)",
        class = "b",
        coef = "pretreatmentC",
        resp = "mass"
      ),
      set_prior("exponential(2.5)",
        class = "sd",
        group = "batch",
        resp = "mass"
      ),
      set_prior("exponential(0.05)",
        class = "sigma",
        resp = "mass"
      ),
      set_prior("normal(0, 3)",
        class = "Intercept",
        resp = "tarsus"
      ),
      set_prior("normal(0, 3)",
        class = "b",
        coef = "pretreatmentA",
        resp = "tarsus"
      ),
      set_prior("normal(0, 3)",
        class = "b",
        coef = "pretreatmentC",
        resp = "tarsus"
      ),
      set_prior("skew_normal(0, 0.25, 5)",
        class = "b",
        coef = "mass",
        resp = "tarsus"
      ),
      set_prior("exponential(2)",
        class = "sd",
        group = "batch",
        resp = "tarsus"
      ),
      set_prior("exponential(0.75)",
        class = "sigma",
        resp = "tarsus"
      ),
      set_prior("normal(0, 1)",
        class = "Intercept",
        resp = "bill"
      ),
     set_prior("normal(0, 0.5)",
```

```
        class = "b",
        coef = "pretreatmentA",
        resp = "bill"
      ),
      set_prior("normal(0, 0.5)",
        class = "b",
        coef = "pretreatmentC",
        resp = "bill"
      ),
      set_prior("skew_normal(0, 0.25, 5)",
        class = "b",
        coef = "mass",
        resp = "bill"
      ),
      set_prior("exponential(5)",
        class = "sd",
        group = "batch",
        resp = "bill"
      ),
      set_prior("exponential(2.5)",
        class = "sigma",
        resp = "bill"
      ),
    set_prior("normal(0.75, 0.2)",
      class = "Intercept",
      resp = "ecc"
    ),
    set_prior("normal(0, 0.25)",
      class = "b",
      coef = "pretreatmentA",
      resp = "ecc"
    ),
    set_prior("normal(0, 0.25)",
      class = "b",
      coef = "pretreatmentC",
      resp = "ecc"
    ),
    set_prior("normal(0, 0.01)",
      class = "b",
      coef = "mass",
      resp = "ecc"
    ),
    set_prior("normal(0, 0.1)",
      class = "b",
      coef = "tarsus",
      resp = "ecc"
    ),
    set_prior("normal(0, 0.15)",
      class = "b",
      coef = "bill",
      resp = "ecc"
    ),
    set_prior("exponential(15)",
      class = "sd",
      group = "batch",
      resp = "ecc"
    ),
    set_prior("exponential(5)",
      class = "sigma",
      resp = "ecc"
    )
  ),
  iter = 50000, warmup = 10000, cores = 4, chains = 4, thin = 20,
  control = list(adapt_delta = .98, max_treedepth = 14),
  silent = TRUE, refresh = 0,
  file = "./models/_efficiencyModel8Weeks.Rds"
)
```

```
pp2a <- mcmc_neff(neff_ratio(efficiencyModel8Weeks)) +
  xlab(
    TeX("$\\overset{Evaporative~Cooling}{Efficiency~N_{eff}/N}$")
  ) +
  theme_classic() +
  theme(
    axis.text.y = element_blank(),
    axis.ticks.y = element_blank(),
    legend.position = "none"
  )

pp2b <- mcmc_rhat(rhat(efficiencyModel8Weeks)) +
  xlab(
    TeX("$\\overset{Evaporative~Cooling~Efficiency}{\\hat{R}}$")
  ) +
  theme_classic() +
  theme(
    axis.text.y = element_blank(),
    axis.ticks.y = element_blank(),
    legend.position = "none"
  )

pp2c <- pp_check2(efficiencyModel8Weeks,
  resp = "ecc",
  xlab = "Evaporative Cooling\nEfficiency (EHL/RMR)"
)

pp2d <- efficiencyModel8Weeks$data %>%
  mutate(
    "Fit" = fitted(efficiencyModel8Weeks,
      resp = "ecc"
    )[, "Estimate"],
    "FitSE" = fitted(efficiencyModel8Weeks,
      resp = "ecc"
    )[, "Est.Error"]
  ) %>%
  ggplot(aes(x = Fit, y = ecc)) +
  geom_errorbarh(aes(xmin = Fit - FitSE, xmax = Fit + FitSE),
    height = 0.05, colour = "black", alpha = 0.8
  ) +
  geom_point(
    size = 2, pch = 21, colour = "black", fill = "lightblue2",
    alpha = 0.8
  ) +
  geom_smooth(
    method = "lm", colour = "black", formula = y ~ 0 + x,
    linetype = "dashed", se = FALSE
  ) +
  xlab("Fitted Evaporative\nCooling Efficiency (EHL/RMR)") +
  ylab("Evaporative Cooling\nEfficiency (EHL/RMR)") +
  theme_classic()

((pp1a + pp1b) /
  (pp1c + pp1d) /
  (pp2a + pp2b) /
  (pp2c + pp2d)) +
  plot_annotation(tag_levels = "A")
```

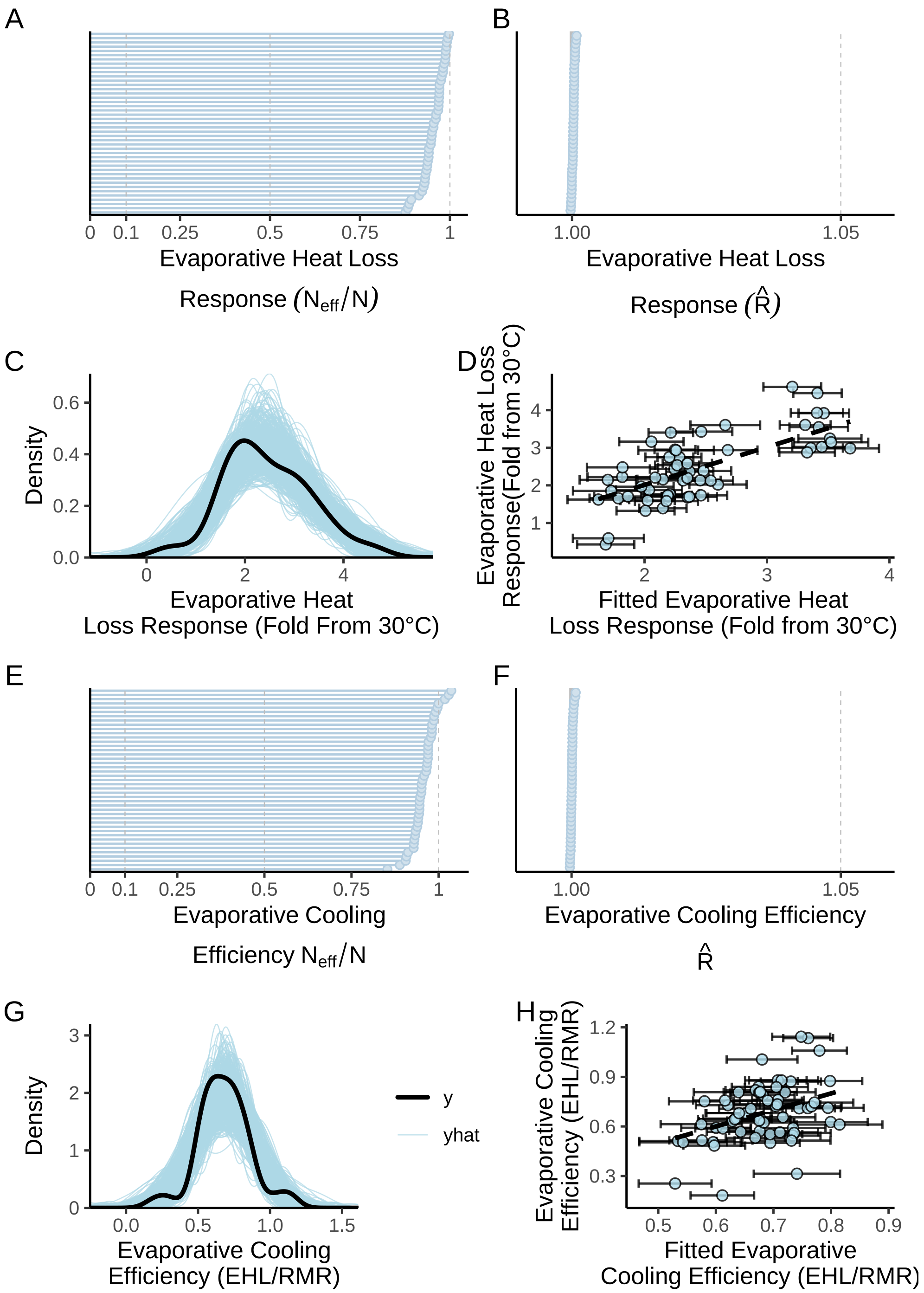

Figure 162: *Model and posterior predictive checks for two Bayesian path analyses predicting, ultimately, evaporative heat loss ('EHL'; fold from that observed at 30°C in W) and evaporative cooling efficiency ('ECE'; the ratio of evaporative heat-loss to metabolic heat production, each in W) in eight week old Japanese quail. Panels A and E display parameter-specific effective sample size to sample size ratios (N_{eff}/N) for each model, while panels B and F display parameter specific Gelman-Rubin statistics ($\hat{R}$). Panels C and G display posterior predictive checks; black lines represent true EHL or ECE densities while blue lines represent densities estimated from model posteriors. In panels D and H, dots represent individual predictions plotted against their true values. Errorbars indicate +/- one standard error around predictions and dashed black lines indicate lines of best fit estimated by ggplot2 (Wickham, 2011) while assuming a y-intercept at 0.*

Chains are clearly well mixed and posterior estimates are unlikely to be biased by autocorrelation between chain draws. As such, we next plot model residuals (here, as medians) against model predictors and expectations to check for evidence of outlaying samples or overt heteroskedasticity.

```
rp1 <- ehlModel8Weeks$data %>%
  mutate(
    "Res" =
      residuals(ehlModel8Weeks,
        resp = "foldEhl",
        robust = TRUE
      )[, "Estimate"]
  ) %>%
  ggplot(aes(sample = Res)) +
  stat_qq(colour = "grey50") +
  stat_qq_line() +
  xlab("Theoretical EHL\nResidual Quantiles") +
  ylab("Sample ELH\nResidual Quantiles") +
  theme_classic()

rp2 <- ehlModel8Weeks$data %>%
  mutate(
    "Res" =
      residuals(ehlModel8Weeks,
        resp = "foldEhl",
        robust = TRUE
      )[, "Estimate"]
  ) %>%
  mutate(mass = mass + mean(subset(vh2o, week == "8")$mass, na.rm = T)) %>%
  ggplot(aes(x = mass, y = Res)) +
  geom_point(pch = 21, colour = "black", fill = "grey20", alpha = 0.5) +
  xlab("Body Mass (g)") +
  ylab("Evaporative Heat\nLoss Residuals") +
  theme_classic()

rp3 <- ehlModel8Weeks$data %>%
  mutate(
    "Res" =
      residuals(ehlModel8Weeks,
        resp = "foldEhl",
        robust = TRUE
      )[, "Estimate"]
  ) %>%
  mutate(tarsus = tarsus +
    mean(subset(vh2o, week == "8")$tarsusLengthMean, na.rm = T)) %>%
  ggplot(aes(x = tarsus, y = Res)) +
  geom_point(pch = 21, colour = "black", fill = "grey20", alpha = 0.5) +
  xlab("Tarsus Length (mm)") +
  ylab("Evaporative Heat\nLoss Residuals") +
  theme_classic()

rp4 <- ehlModel8Weeks$data %>%
  mutate(
    "Res" =
      residuals(ehlModel8Weeks,
        resp = "foldEhl",
        robust = TRUE
      )[, "Estimate"]
  ) %>%
  mutate(bill = bill +
    mean(subset(vh2o, week == "8")$billLengthMean, na.rm = T)) %>%
  ggplot(aes(x = tarsus, y = Res)) +
  geom_point(pch = 21, colour = "black", fill = "grey20", alpha = 0.5) +
  xlab("Bill Length (mm)") +
  ylab("Evaporative Heat\nLoss Residuals") +
  theme_classic()

rp5 <- ehlModel8Weeks$data %>%
```

```
  mutate(
    "Res" =
      residuals(ehlModel8Weeks,
        resp = "foldEhl",
        robust = TRUE
      )[, "Estimate"]
  ) %>%
  mutate(pretreatment = factor(pretreatment, levels = c("A", "B", "C"))) %>%
  ggplot(aes(x = pretreatment, y = Res)) +
  geom_boxplot(fill = "lightblue2") +
  geom_point(size = 2, position = position_jitter(width = 0.25)) +
  scale_x_discrete(
    name = "Rearing Treatment",
    labels = c(
      "Cold\n(10°C)",
      "Mild\n(20°C)",
      "Warm\n(30°C)"
    )
  ) +
  xlab("Rearing Treatment") +
  ylab("Evaporative Heat\nLoss Residuals") +
  theme_classic()

rp6 <- efficiencyModel8Weeks$data %>%
  mutate(
    "Res" =
      residuals(efficiencyModel8Weeks,
        resp = "ecc",
        robust = TRUE
      )[, "Estimate"]
  ) %>%
  ggplot(aes(sample = Res)) +
  stat_qq(colour = "grey50") +
  stat_qq_line() +
  xlab("Theoretical ECE\nResidual Quantiles") +
  ylab("Sample ECE\nResidual Quantiles") +
  theme_classic()

rp7 <- efficiencyModel8Weeks$data %>%
  mutate(
    "Res" =
      residuals(efficiencyModel8Weeks,
        resp = "ecc",
        robust = TRUE
      )[, "Estimate"]
  ) %>%
  mutate(mass = mass + mean(subset(vh2o, week == "8")$mass, na.rm = T)) %>%
  ggplot(aes(x = mass, y = Res)) +
  geom_point(pch = 21, colour = "black", fill = "grey20", alpha = 0.5) +
  xlab("Body Mass (g)") +
  ylab("Evaporative Cooling\nEfficiency Residuals") +
  theme_classic()

rp8 <- efficiencyModel8Weeks$data %>%
  mutate(
    "Res" =
      residuals(efficiencyModel8Weeks,
        resp = "ecc",
        robust = TRUE
      )[, "Estimate"]
  ) %>%
  mutate(tarsus = tarsus +
    mean(subset(vh2o, week == "8")$tarsusLengthMean, na.rm = T)) %>%
  ggplot(aes(x = tarsus, y = Res)) +
  geom_point(pch = 21, colour = "black", fill = "grey20", alpha = 0.5) +
  xlab("Tarsus Length (mm)") +
  ylab("Evaporative Cooling\nEfficiency Residuals") +
```

```
  theme_classic()

rp9 <- efficiencyModel8Weeks$data %>%
  mutate(
    "Res" =
      residuals(efficiencyModel8Weeks,
        resp = "ecc",
        robust = TRUE
      )[, "Estimate"]
  ) %>%
  mutate(bill = bill +
    mean(subset(vh2o, week == "8")$billLengthMean, na.rm = T)) %>%
  ggplot(aes(x = tarsus, y = Res)) +
  geom_point(pch = 21, colour = "black", fill = "grey20", alpha = 0.5) +
  xlab("Bill Length (mm)") +
  ylab("Evaporative Cooling\nEfficiency Residuals") +
  theme_classic()

rp10 <- efficiencyModel8Weeks$data %>%
  mutate(
    "Res" =
      residuals(efficiencyModel8Weeks,
        resp = "ecc",
        robust = TRUE
      )[, "Estimate"]
  ) %>%
  mutate(pretreatment = factor(pretreatment, levels = c("A", "B", "C"))) %>%
  ggplot(aes(x = pretreatment, y = Res)) +
  geom_boxplot(fill = "lightblue2") +
  geom_point(size = 2, position = position_jitter(width = 0.25)) +
  scale_x_discrete(
    name = "Rearing Treatment",
    labels = c(
      "Cold\n(10°C)",
      "Mild\n(20°C)",
      "Warm\n(30°C)"
    )
  ) +
  xlab("Rearing Treatment") +
  ylab("Evaporative Cooling\nEfficiency Residuals") +
  theme_classic()

((rp1 + rp6 + rp2)/
  (rp7 + rp3 + rp8)/
  (rp4 + rp9 + rp5)/
    ((rp10) + plot_spacer() + plot_spacer())
) + plot_annotation(tag_levels = "A")
```

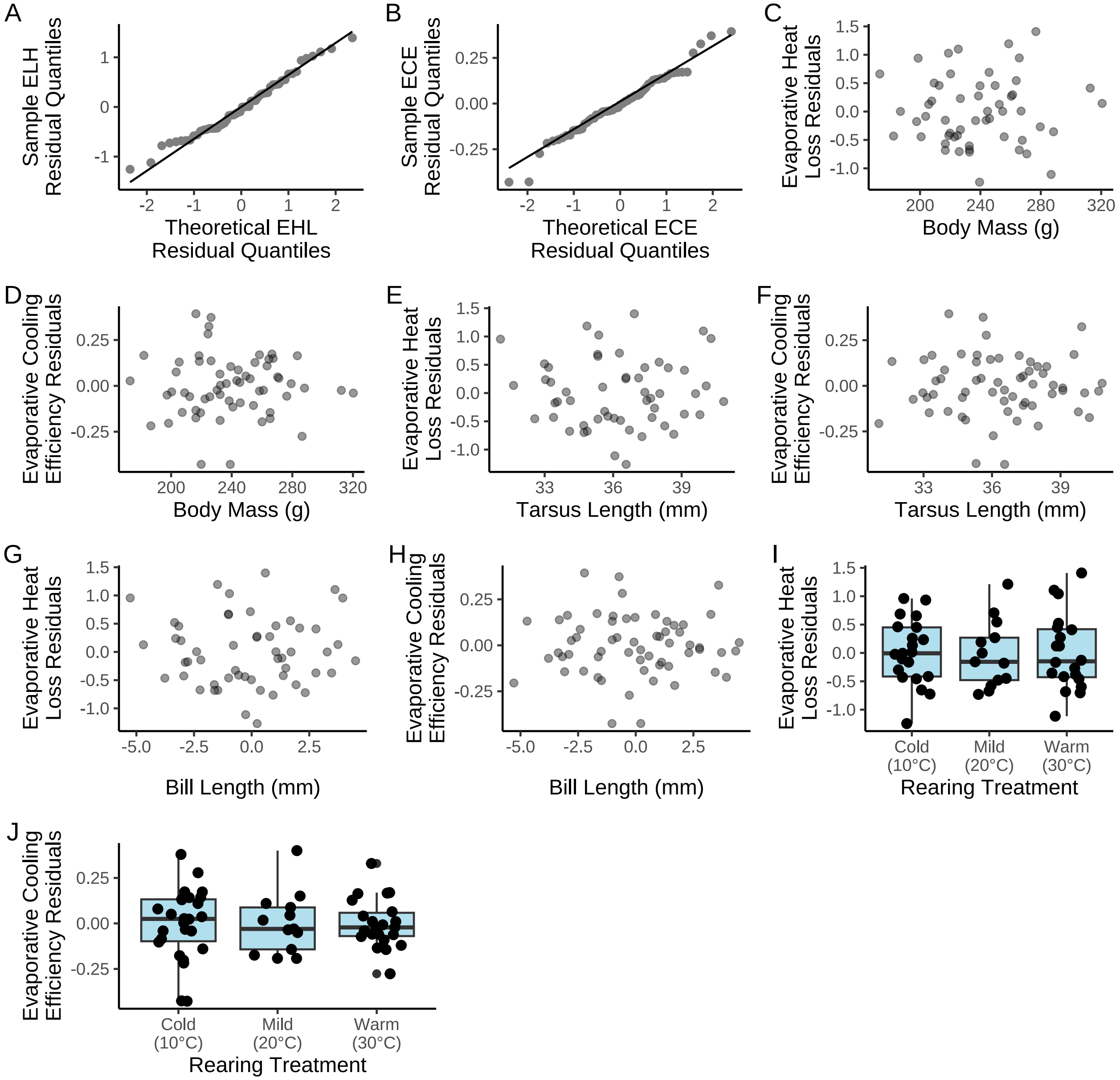

Figure 163: *Residual diagnostics from two Bayesian path analyses predicting evaporative heat loss ('EHL'; fold from that observed at 30°C in W) and evaporative cooling efficiency ('ECE'; the ratio of evaporative heat-loss to metabolic heat production, each in W) in eight week old Japanese quail. Panels A and B display traditional 'qq-plots', with theoretic and sample residual quantiles regressed against each other. Dots represent individual samples. Remaining panels display median residual, per raw data point (small dots) by model predictors (here, body mass [g], tarsus length [mm], bill length [mm] and rearing condition (10°C, 20°C, or 30°C until the time of measurement). Boxplots in panels I and J display medians (centre horizontal bar), first and third quantiles (lower and upper limits of boxes respectively) and ranges excluding outliers (whiskers). 'EHL' indicates evaporative heat loss in watts, and ECE indicates evaporative cooling efficiency (the ratio of evaporative heat loss by metabolic heat production, each in watts).*

Residuals appear both normally- and evenly-distributed (by predictor variables). Next, we plot model coefficient density to evaluate possible skewing, multimodality etc.

```
as.data.frame(ehlModel8Weeks) %>%
  pivot_longer(everything(), names_to = "par", values_to = "values") %>%
  filter(grepl("b_|sd_", par)) %>%
  merge(., tribble(
    ~par, ~Par, ~Order,
    "b_mass_Intercept", "Mass\nIntercept", "A",
    "b_mass_pretreatmentA", "Mass ~\nCold Rearing", "B",
    "b_mass_pretreatmentC", "Mass ~\nWarm Rearing", "C",
    "b_tarsus_Intercept", "Tarsus\nIntercept", "D",
    "b_tarsus_pretreatmentA", "Tarsus ~\nCold Rearing", "E",
    "b_tarsus_pretreatmentC", "Tarsus ~\nWarm Rearing", "F",
    "b_tarsus_mass", "Tarsus ~\nMass", "G",
    "sd_batch__tarsus_Intercept", "Tarsus ~\nBatch", "H",
    "b_bill_Intercept", "Bill\nIntercept", "I",
    "b_bill_pretreatmentA", "Bill ~\nCold Rearing", "J",
    "b_bill_pretreatmentC", "Bill ~\nWarm Rearing", "K",
    "b_bill_mass", "Bill ~\nMass", "L",
    "sd_batch__bill_Intercept", "Bill ~\nBatch", "M",
    "b_foldEhl_Intercept", "EHL\nIntercept", "N",
    "b_foldEhl_pretreatmentA", "EHL ~\nCold Rearing", "O",
    "b_foldEhl_pretreatmentC", "EHL ~\nWarm Rearing", "P",
    "b_foldEhl_mass", "EHL ~ Mass", "Q",
    "b_foldEhl_tarsus", "EHL ~\nTarsus", "R",
    "b_foldEhl_bill", "EHL ~\nBill", "S",
    "sd_batch__foldEhl_Intercept", "EHL ~\nBatch", "T"
  ),
  by = "par", all.x = TRUE
  ) %>%
  arrange(Order) %>%
  mutate(Par = factor(Par, levels = unique(Par))) %>%
  drop_na() %>%
  ggplot(aes(x = values)) +
  facet_wrap(~Par, scales = "free") +
  geom_density(colour = "black", fill = "white") +
  geom_vline(xintercept = 0, colour = "darkred", linetype = "solid") +
  scale_x_continuous(n.breaks = 3) +
  scale_y_continuous(n.breaks = 3) +
  xlab("Values") +
  ylab("Density") +
  theme_classic()
```

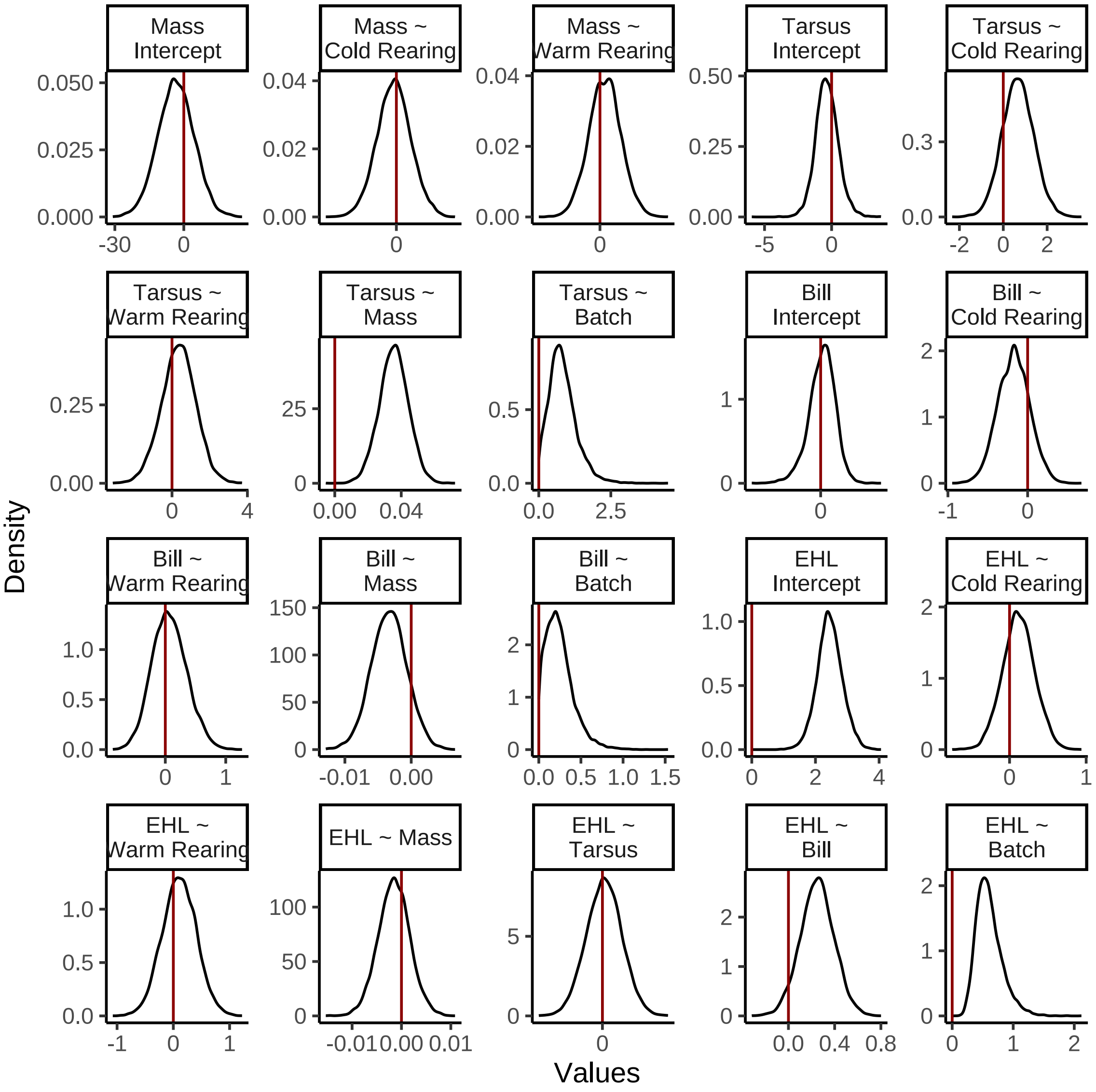

Figure 164: *Posterior densities for coefficients from a Bayesian path analysis predicting body mass (g), tarsus length (mm), bill length (mm) and evaporative heat loss responses ("EHL"; fold from that observed at 30°C in W) in eight week old Japanese quail. Red vertical lines label 0. Response and predictor variables for which densities refer are indicated on the left side and right side of tildes respectively. "Cold Rearing" indicates rearing at 10°C, "Warm Rearing" indicates rearing at 30°C, and "Batch" indicates the batch of eggs from which an individual was derived. All coefficients except batch are population level.*

```
as.data.frame(efficiencyModel8Weeks) %>%
  pivot_longer(everything(), names_to = "par", values_to = "values") %>%
  filter(grepl("b_|sd_", par)) %>%
  merge(., tribble(
    ~par, ~Par, ~Order,
    "b_mass_Intercept", "Mass\nIntercept", "A",
    "b_mass_pretreatmentA", "Mass ~\nCold Rearing", "B",
    "b_mass_pretreatmentC", "Mass ~\nWarm Rearing", "C",
    "b_tarsus_Intercept", "Tarsus\nIntercept", "D",
    "b_tarsus_pretreatmentA", "Tarsus ~\nCold Rearing", "E",
    "b_tarsus_pretreatmentC", "Tarsus ~\nWarm Rearing", "F",
    "b_tarsus_mass", "Tarsus ~\nMass", "G",
    "sd_batch__tarsus_Intercept", "Tarsus ~\nBatch", "H",
```

```
    "b_bill_Intercept", "Bill\nIntercept", "I",
    "b_bill_pretreatmentA", "Bill ~\nCold Rearing", "J",
    "b_bill_pretreatmentC", "Bill ~\nWarm Rearing", "K",
    "b_bill_mass", "Bill ~\nMass", "L",
    "sd_batch__bill_Intercept", "Bill ~\nBatch", "M",
    "b_ecc_Intercept", "ECE\nIntercept", "N",
    "b_ecc_pretreatmentA", "ECE ~\nCold Rearing", "O",
    "b_ecc_pretreatmentC", "ECE ~\nWarm Rearing", "P",
    "b_ecc_mass", "ECE ~ Mass", "Q",
    "b_ecc_tarsus", "ECE ~\nTarsus", "R",
    "b_ecc_bill", "ECE ~\nBill", "S",
    "sd_batch__ecc_Intercept", "ECE ~\nBatch", "T"
  ),
  by = "par", all.x = TRUE
  ) %>%
  drop_na() %>%
  arrange(Order) %>%
  mutate(Par = factor(Par, levels = unique(Par))) %>%
  ggplot(aes(x = values)) +
  facet_wrap(~Par, scales = "free") +
  geom_density(colour = "black", fill = "white") +
  geom_vline(xintercept = 0, colour = "darkred", linetype = "solid") +
  scale_x_continuous(n.breaks = 3) +
  scale_y_continuous(n.breaks = 3) +
  xlab("Values") +
  ylab("Density") +
  theme_classic()
```

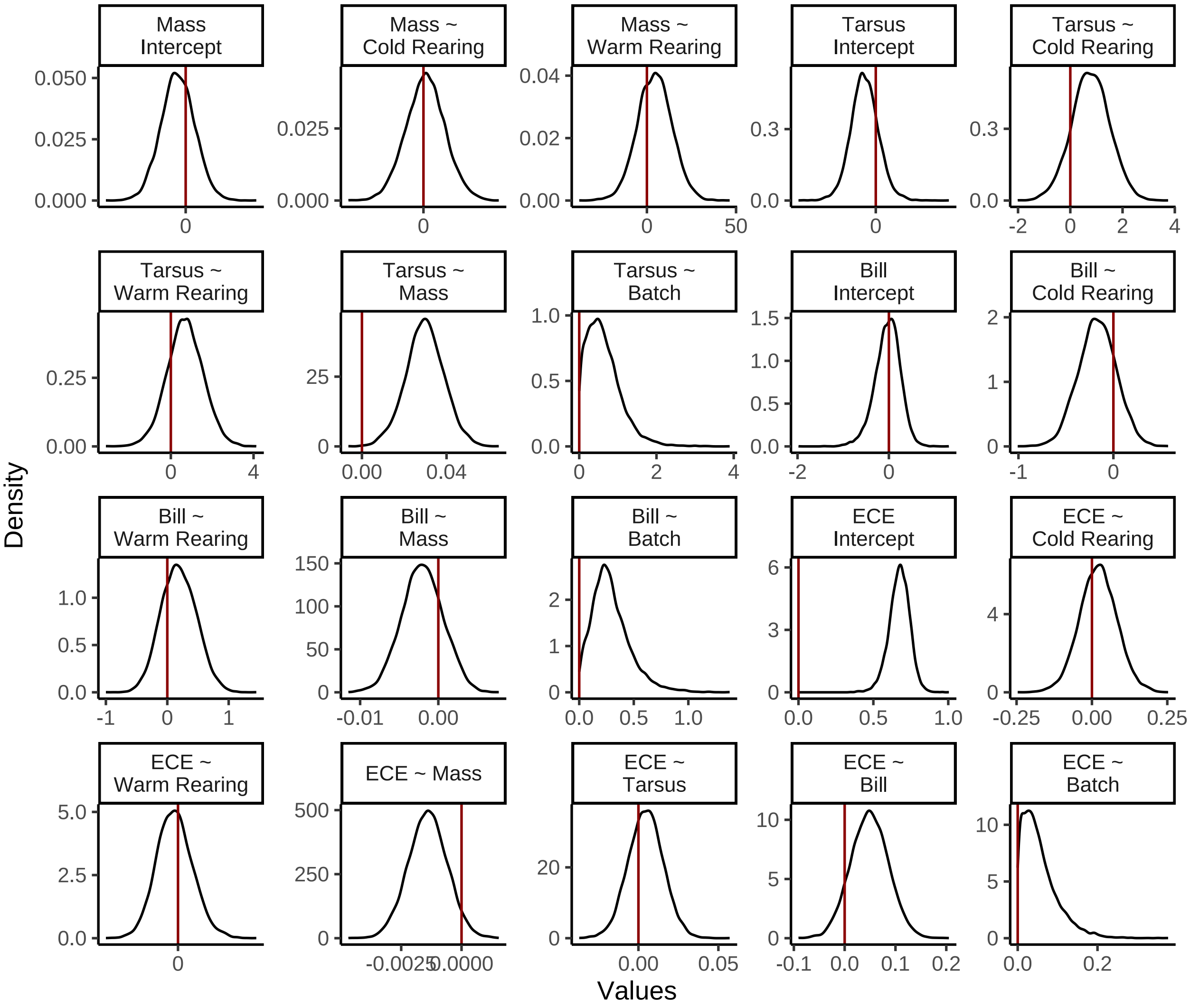

Figure 165: *Posterior densities for coefficients from a Bayesian path analysis predicting body mass (g), tarsus length (mm), bill length (mm) and evaporative cooling efficiency ("ECE", the ratio of evaporative heat loss in watts to evaporative heat production in watts) in eight week old Japanese quail. Red vertical lines label 0. Response and predictor variables for which densities refer are indicated on the left side and right side of tildes respectively. "Cold Rearing" indicates rearing at 10°C, "Warm Rearing" indicates rearing at 30°C, and "Batch" indicates the batch of eggs from which an individual was derived. All coefficients except batch are population level.*

As before, we proceed by summarising centrality of posteriors with medians. Credible intervals around medians are estimated using quantiles.

```
caption <- paste0(
  "Results of Bayesian path analysis testing the ",
  "effects of morphology and rearing temperature on relative evaporative ",
  "heat loss at 40°C in eight week old Japanese quail. Relative evapoative ",
  "heat loss represents the Evaporative cooling efficiency ",
  "fold change from 30°C, per individual. Estimates indicate posterior ",
  "medians and credible intervels (CIs) indicate quantile intervals."
)

heatLoss8WeeksResults <-
```

```
as.data.frame(ehlModel8Weeks) %>%
summarise_all(., .funs = median) %>%
pivot_longer(everything(),
  names_to = "Parameter",
  values_to = "Estimate"
) %>%
merge(., quantileCIs(ehlModel8Weeks, cis = c(50, 95)),
  by = "Parameter", all.x = TRUE
) %>%
filter(grepl("b_|sd_", Parameter)) %>%
rowwise() %>%
mutate("BF" = ifelse(Estimate < 0,
  (2 * mean(as.data.frame(
    ehlModel8Weeks
  )[, Parameter] <= 0)) /
    (2 * mean(as.data.frame(
      ehlModel8Weeks
    )[, Parameter] >= 0)),
  (2 * mean(as.data.frame(
    ehlModel8Weeks
  )[, Parameter] >= 0)) /
    (2 * mean(as.data.frame(
      ehlModel8Weeks
    )[, Parameter] <= 0))
)) %>%
ungroup() %>%
mutate(
  "Estimate" = round(Estimate, digits = 4),
  "BF" = round(BF, digits = 4),
  "N" = nrow(ehlModel3Weeks$data)
) %>%
mutate("Parameter" = ifelse(grepl("b_", Parameter),
  gsub("b_", "", Parameter),
  gsub(
    "Intercept", "batch",
    gsub(".*__", "", Parameter)
  )
)) %>%
mutate(
  "Response" = gsub("_.*", "", Parameter),
  "Parameter" = gsub(".*_", "", Parameter)
) %>%
merge(., tribble(
  ~Response, ~response, ~level,
  "mass", "Body Mass (g)", "A",
  "tarsus", "Tarsus Length (mm)", "B",
  "bill", "Bill Length (mm)", "C",
  "foldEhl", "Evaporative Heat Loss (fold from 30°C)", "D"
),
by = "Response"
) %>%
merge(., tribble(
  ~Parameter, ~parameter, ~number,
  "Intercept", "Intercept", "1",
  "pretreatmentA", "Cold Rearing", "2",
  "pretreatmentC", "Warm Rearing", "3",
  "mass", "Body Mass (g)", "4",
  "tarsus", "Tarsus Length (mm)", "5",
  "bill", "Bill Length (mm)", "6",
  "batch", "Egg Batch [mu]", "7"
),
by = "Parameter"
) %>%
mutate(
  `50\\% CI` = paste0("(", paste(
    round(Low_CI_50, digits = 4),
    round(High_CI_50, digits = 4),
```

```
    sep = ", "
  ), ")"),
  `95\\% CI` = paste0("(", paste(
    round(Low_CI_95, digits = 4),
    round(High_CI_95, digits = 4),
    sep = ", "
  ), ")")
) %>%
dplyr::select(-c(Low_CI_50, High_CI_50, Low_CI_95, High_CI_95)) %>%
dplyr::select(
  "Response" = "response", "Parameter" = "parameter", N,
  Estimate, `50\\% CI`, `95\\% CI`, BF, level, number
) %>%
arrange(level, number) %>%
dplyr::select(-c(level, number)) %>%
kbl(.,
  longtable = T, booktabs = T, format = "latex", escape = FALSE,
  caption = caption
) %>%
column_spec(column = c(1:2), width = "2.1cm") %>%
column_spec(column = c(3:10), width = "1.8cm") %>%
kable_styling(latex_options = "striped")

heatLoss8WeeksResults
```

Table 102: *Results of Bayesian path analysis testing the effects of morphology and rearing temperature on relative evaporative heat loss at 40°C in eight week old Japanese quail. Relative evapoative heat loss represents the Evaporative cooling efficiency fold change from 30°C, per individual. Estimates indicate posterior medians and credible intervels (CIs) indicate quantile intervals.*

Response	Parameter	N	Estimate	50% CI	95% CI	BF
Body Mass (g)	Intercept	47	-3.4143	(-8.6124, 1.8598)	(-18.8191, 11.8185)	2.0189
Body Mass (g)	Cold Rearing	47	-1.3354	(-8.0887, 5.2069)	(-21.2528, 18.4401)	1.2637
Body Mass (g)	Warm Rearing	47	3.1313	(-3.6143, 9.6434)	(-16.5329, 23.0735)	1.6264
Body Mass (g)	Egg Batch [mu]	47	0.2728	(0.1148, 0.5647)	(0.0098, 1.5079)	Inf
Tarsus Length (mm)	Intercept	47	-0.3738	(-0.8879, 0.1861)	(-1.9403, 1.4109)	2.0523
Tarsus Length (mm)	Cold Rearing	47	0.6606	(0.1876, 1.1439)	(-0.7254, 2.0983)	4.5983
Tarsus Length (mm)	Warm Rearing	47	0.3633	(-0.2411, 0.9531)	(-1.4698, 2.1117)	1.9059
Tarsus Length (mm)	Body Mass (g)	47	0.0356	(0.0297, 0.0414)	(0.0181, 0.0524)	7999.0000
Tarsus Length (mm)	Egg Batch [mu]	47	0.7678	(0.4982, 1.0873)	(0.0783, 1.9566)	Inf
Bill Length (mm)	Intercept	47	0.0441	(-0.1307, 0.2)	(-0.5458, 0.4918)	1.3242
Bill Length (mm)	Cold Rearing	47	-0.1736	(-0.3124, -0.0402)	(-0.5632, 0.22)	4.2875
Bill Length (mm)	Warm Rearing	47	0.0589	(-0.1324, 0.2586)	(-0.4702, 0.6563)	1.3981
Bill Length (mm)	Body Mass (g)	47	-0.0033	(-0.0051, -0.0015)	(-0.0087, 0.002)	8.1848
Bill Length (mm)	Egg Batch [mu]	47	0.2176	(0.1213, 0.3308)	(0.012, 0.6586)	Inf
Evaporative Heat Loss (fold from 30°C)	Intercept	47	2.4388	(2.1935, 2.7028)	(1.6681, 3.2497)	Inf
Evaporative Heat Loss (fold from 30°C)	Cold Rearing	47	0.1165	(-0.0207, 0.2577)	(-0.2953, 0.527)	2.5746

Evaporative Heat Loss (fold from 30°C)	Warm Rearing	47	0.1051	(-0.0974, 0.313)	(-0.489, 0.7016)	1.7416
Evaporative Heat Loss (fold from 30°C)	Body Mass (g)	47	-0.0015	(-0.0036, 6e-04)	(-0.0078, 0.0049)	2.1262
Evaporative Heat Loss (fold from 30°C)	Tarsus Length (mm)	47	0.0046	(-0.0266, 0.0355)	(-0.0866, 0.0954)	1.1775
Evaporative Heat Loss (fold from 30°C)	Bill Length (mm)	47	0.2499	(0.1521, 0.3455)	(-0.0407, 0.5366)	20.3904
Evaporative Heat Loss (fold from 30°C)	Egg Batch [mu]	47	0.5758	(0.4572, 0.7223)	(0.2799, 1.1083)	Inf

```
caption <- paste0(
  "Results of Bayesian path analysis testing the ",
  "effects of morphology and rearing temperature on evaporative ",
  "cooling efficiency at 40°C in eight week old Japanese quail. ",
  "Evaporative cooling efficiency represents the ratio of ",
  "evaporative heat loss (in W) to metabolic heat production ",
  "(again, in W). Estimates indicate posterior medians and credible ",
  "intervels (CIs) indicate quantile intervals."
)

efficiency8WeeksResults <-
  as.data.frame(efficiencyModel8Weeks) %>%
  summarise_all(., .funs = median) %>%
  pivot_longer(everything(),
    names_to = "Parameter",
    values_to = "Estimate"
  ) %>%
  merge(., quantileCIs(efficiencyModel8Weeks,
                       cis = c(50, 95)),
    by = "Parameter", all.x = TRUE
  ) %>%
  filter(grepl("b_|sd_", Parameter)) %>%
  rowwise() %>%
  mutate("BF" = ifelse(Estimate < 0,
    (2 * mean(as.data.frame(
      efficiencyModel8Weeks
    )[, Parameter] <= 0)) /
      (2 * mean(as.data.frame(
        efficiencyModel8Weeks
      )[, Parameter] >= 0)),
    (2 * mean(as.data.frame(
      efficiencyModel8Weeks
    )[, Parameter] >= 0)) /
      (2 * mean(as.data.frame(
        efficiencyModel8Weeks
      )[, Parameter] <= 0))
  )) %>%
  ungroup() %>%
  mutate(
    "Estimate" = round(Estimate, digits = 4),
    "BF" = round(BF, digits = 4),
    "N" = nrow(efficiencyModel8Weeks$data)
  ) %>%
  mutate("Parameter" = ifelse(grepl("b_", Parameter),
    gsub("b_", "", Parameter),
    gsub(
      "Intercept", "batch",
      gsub(".*__", "", Parameter)
    )
  )) %>%
```

```
  mutate(
    "Response" = gsub("_.*", "", Parameter),
    "Parameter" = gsub(".*_", "", Parameter)
  ) %>%
  merge(., tribble(
    ~Response, ~response, ~level,
    "mass", "Body Mass (g)", "A",
    "tarsus", "Tarsus Length (mm)", "B",
    "bill", "Bill Length (mm)", "C",
    "ecc", "Evaporative Cooling Efficiency", "D"
  ),
  by = "Response"
  ) %>%
  merge(., tribble(
    ~Parameter, ~parameter, ~number,
    "Intercept", "Intercept", "1",
    "pretreatmentA", "Cold Rearing", "2",
    "pretreatmentC", "Warm Rearing", "3",
    "mass", "Body Mass (g)", "4",
    "tarsus", "Tarsus Length (mm)", "5",
    "bill", "Bill Length (mm)", "6",
    "batch", "Egg Batch [mu]", "7"
  ),
  by = "Parameter"
  ) %>%
  mutate(
    `50\\% CI` = paste0("(", paste(
      round(Low_CI_50, digits = 4),
      round(High_CI_50, digits = 4),
      sep = ", "
    ), ")"),
    `95\\% CI` = paste0("(", paste(
      round(Low_CI_95, digits = 4),
      round(High_CI_95, digits = 4),
      sep = ", "
    ), ")")
  ) %>%
  dplyr::select(-c(Low_CI_50, High_CI_50, Low_CI_95, High_CI_95)) %>%
  dplyr::select(
    "Response" = "response", "Parameter" = "parameter", N,
    Estimate, `50\\% CI`, `95\\% CI`, BF, level, number
  ) %>%
  arrange(level, number) %>%
  dplyr::select(-c(level, number)) %>%
  kbl(.,
    longtable = T, booktabs = T, format = "latex", escape = FALSE,
    caption = caption
  ) %>%
  column_spec(column = c(1:2), width = "2.1cm") %>%
  column_spec(column = c(3:10), width = "1.8cm") %>%
  kable_styling(latex_options = "striped")

efficiency8WeeksResults
```

Table 103: *Results of Bayesian path analysis testing the effects of morphology and rearing temperature on evaporative cooling efficiency at 40°C in eight week old Japanese quail. Evaporative cooling efficiency represents the ratio of evaporative heat loss (in W) to metabolic heat production (again, in W). Estimates indicate posterior medians and credible intervels (CIs) indicate quantile intervals.*

Response	Parameter	N	Estimate	50% CI	95% CI	BF
Body Mass (g)	Intercept	61	-3.5996	(-8.5926, 1.5126)	(-18.3531, 11.4829)	2.0840
Body Mass (g)	Cold Rearing	61	1.0191	(-5.242, 7.0683)	(-17.3149, 19.6281)	1.2063
Body Mass (g)	Warm Rearing	61	4.4088	(-2.1603, 10.7721)	(-14.5858, 23.2362)	2.0269

Body Mass (g)	Egg Batch [mu]	61	0.2813	(0.1169, 0.5578)	(0.0106, 1.462)	Inf
Tarsus Length (mm)	Intercept	61	-0.6408	(-1.1287, -0.1296)	(-2.1552, 0.9588)	3.9782
Tarsus Length (mm)	Cold Rearing	61	0.7777	(0.2953, 1.2669)	(-0.673, 2.2005)	6.1942
Tarsus Length (mm)	Warm Rearing	61	0.6870	(0.1148, 1.2699)	(-1.0511, 2.3997)	3.7790
Tarsus Length (mm)	Body Mass (g)	61	0.0297	(0.0239, 0.0357)	(0.0114, 0.0478)	1141.8571
Tarsus Length (mm)	Egg Batch [mu]	61	0.5545	(0.2958, 0.8699)	(0.0264, 1.7234)	Inf
Bill Length (mm)	Intercept	61	-0.0125	(-0.2002, 0.1573)	(-0.6286, 0.4711)	1.0790
Bill Length (mm)	Cold Rearing	61	-0.1684	(-0.3016, -0.0345)	(-0.5543, 0.2163)	4.0473
Bill Length (mm)	Warm Rearing	61	0.1800	(-0.0156, 0.3834)	(-0.3591, 0.7604)	2.7612
Bill Length (mm)	Body Mass (g)	61	-0.0020	(-0.0038, -2e-04)	(-0.0072, 0.0031)	3.4944
Bill Length (mm)	Egg Batch [mu]	61	0.2642	(0.1697, 0.3818)	(0.0301, 0.7189)	Inf
Evaporative Cooling Efficiency	Intercept	61	0.6759	(0.6304, 0.7194)	(0.5273, 0.7995)	Inf
Evaporative Cooling Efficiency	Cold Rearing	61	0.0207	(-0.0206, 0.062)	(-0.101, 0.1437)	1.7073
Evaporative Cooling Efficiency	Warm Rearing	61	-0.0170	(-0.0687, 0.0366)	(-0.1632, 0.149)	1.4104
Evaporative Cooling Efficiency	Body Mass (g)	61	-0.0014	(-0.0019, -9e-04)	(-0.003, 2e-04)	23.4648
Evaporative Cooling Efficiency	Tarsus Length (mm)	61	0.0046	(-0.0028, 0.0117)	(-0.017, 0.0262)	1.9674
Evaporative Cooling Efficiency	Bill Length (mm)	61	0.0488	(0.0235, 0.074)	(-0.0237, 0.1214)	9.6101
Evaporative Cooling Efficiency	Egg Batch [mu]	61	0.0442	(0.0216, 0.0756)	(0.002, 0.1688)	Inf

Our results suggest that morphology (in particular, body mass) may influence: (1) the rate at which individuals increase their evaporative heat loss above 40°C, and (2) the efficiency by which evaporative cooling is achieved at maturity. To evaluate the extent to which variance in these two response variables are explained by morphology, we below calculate partial R^2 values for body mass, tarsus length and bill length from our above path analyses. Calculation of partial R^2 values is described above (subsection "Effects in developing individuals").

```
## Calculating total R2 values

caption <- paste0(
  "Estimates of fit for each element of a Bayesian path ",
  "analysis predicting evaporative heat loss responses ",
  "(fold from 30°C; Model 'A') and evaporative cooling ",
  "efficiency (Model 'B') in mature Japanese quail ",
  "(8 weeks of age). Response variables refer to those ",
  "measured at 40°C. Credible intervals are quantile intervals."
)
```

```
brms::bayes_R2(ehlModel8Weeks,
  ndraws = 1000,
  robust = TRUE
) %>%
  as.data.frame() %>%
  rownames_to_column("var") %>%
  merge(., tribble(
    ~var, ~Var,
    "R2mass", "Body Mass (g)",
    "R2tarsus", "Tarsus Length (mm)",
    "R2bill", "Bill Length (mm)",
    "R2foldEhl",
    "Evaporative Heat Loss"
  ), by = c("var")) %>%
  mutate(
    Estimate = round(Estimate, digits = 4),
    Est.Error = round(Est.Error, digits = 4),
    "95\\% CI" = paste0(
      "[", round(Q2.5, digits = 4),
      ", ", round(Q97.5, digits = 4),
      "]"
    ),
    "Model" = "A"
  ) %>%
  rbind(., brms::bayes_R2(efficiencyModel8Weeks,
    ndraws = 1000,
    robust = TRUE
  ) %>%
    as.data.frame() %>%
    rownames_to_column("var") %>%
    merge(., tribble(
      ~var, ~Var,
      "R2mass", "Body Mass (g)",
      "R2tarsus", "Tarsus Length (mm)",
      "R2bill", "Bill Length (mm)",
      "R2ecc",
      "Evaporative Cooling Efficiency"
    ), by = c("var")) %>%
    mutate(
      Estimate = round(Estimate, digits = 4),
      Est.Error = round(Est.Error, digits = 4),
      "95\\% CI" = paste0(
        "[", round(Q2.5, digits = 4),
        ", ", round(Q97.5, digits = 4),
        "]"
      ),
      "Model" = "B"
    )) %>%
  dplyr::select(
    Model,
    "Response" = Var, "R\\textsuperscript{2}" = Estimate,
    "Standard Error" = Est.Error,
    `95\\% CI`
  ) %>%
  kbl(.,
    longtable = T, booktabs = T, format = "latex",
    caption = caption, escape = FALSE
  ) %>%
  column_spec(column = c(1:10), width = "2.5cm") %>%
  kable_styling(latex_options = "striped")
```

Table 104: *Estimates of fit for each element of a Bayesian path analysis predicting evaporative heat loss responses (fold from 30°C; Model 'A') and evaporative cooling efficiency (Model 'B') in mature Japanese quail (8 weeks of age). Response variables refer to those measured at 40°C. Credible intervals are quantile intervals.*

Model	Response	R^2	Standard Error	95% CI
A	Bill Length (mm)	0.1436	0.0833	[0.0255, 0.3042]
A	Evaporative Heat Loss	0.4871	0.0755	[0.2963, 0.597]
A	Body Mass (g)	0.0236	0.0233	[8e-04, 0.1056]
A	Tarsus Length (mm)	0.3612	0.0789	[0.1777, 0.4953]
B	Bill Length (mm)	0.1572	0.0785	[0.0354, 0.3063]
B	Evaporative Cooling Efficiency	0.1990	0.0693	[0.068, 0.3259]
B	Body Mass (g)	0.0215	0.0217	[0.001, 0.1062]
B	Tarsus Length (mm)	0.2513	0.0824	[0.098, 0.3847]

```
# Partial R2 calculations

{
  ehl8WeekData <-
    vh2o %>%
    filter(week == "8" & Ta %in% c(30, 40)) %>%
    dplyr::select(Ta, ring, pretreatment,
      mass, tarsusLengthMean, billLengthMean,
      ehl,
      "batch" = exp
    ) %>%
    pivot_wider(
      id_cols = c(
        "ring", "batch",
        "pretreatment", "mass",
        "tarsusLengthMean", "billLengthMean"
      ),
      values_from = "ehl",
      names_from = "Ta"
    ) %>%
    mutate("foldEhl" = `40` / `30`) %>%
    mutate(pretreatment = ifelse(pretreatment == "neutral", "B",
      ifelse(pretreatment == "cold", "A", "C")
    )) %>%
    mutate(pretreatment = factor(pretreatment,
      levels = c("B", "A", "C")
    )) %>%
    distinct() %>%
    mutate(
      mass = mass - mean(mass, na.rm = T),
      tarsus = tarsusLengthMean - mean(tarsusLengthMean, na.rm = T),
      bill = billLengthMean - mean(billLengthMean, na.rm = T)
    )

  ehlModel8WeeksMassR2 <- brm(
    data = ehl8WeekData,
    family = "gaussian",
    bf(mass ~ pretreatment + (1 | batch)) +
      bf(tarsus ~ mass + pretreatment + (1 | batch)) +
      bf(bill ~ mass + pretreatment + (1 | batch)) +
      bf(foldEhl ~ tarsus + bill + pretreatment + (1 | batch)) +
      set_rescor(FALSE),
    prior = c(
      set_prior("normal(0, 10)",
        class = "Intercept",
        resp = "mass"
      ),
      set_prior("normal(0, 25)",
        class = "b",
        coef = "pretreatmentA",
        resp = "mass"
      ),
```

```
    set_prior("normal(0, 25)",
      class = "b",
      coef = "pretreatmentC",
      resp = "mass"
    ),
    set_prior("exponential(2.5)",
      class = "sd",
      group = "batch",
      resp = "mass"
    ),
    set_prior("exponential(0.05)",
      class = "sigma",
      resp = "mass"
    ),
    set_prior("normal(0, 3)",
      class = "Intercept",
      resp = "tarsus"
    ),
    set_prior("normal(0, 3)",
      class = "b",
      coef = "pretreatmentA",
      resp = "tarsus"
    ),
    set_prior("normal(0, 3)",
      class = "b",
      coef = "pretreatmentC",
      resp = "tarsus"
    ),
    set_prior("skew_normal(0, 0.25, 5)",
      class = "b",
      coef = "mass",
      resp = "tarsus"
    ),
    set_prior("exponential(2)",
      class = "sd",
      group = "batch",
      resp = "tarsus"
    ),
    set_prior("exponential(0.75)",
      class = "sigma",
      resp = "tarsus"
    ),
    set_prior("normal(0, 1)",
      class = "Intercept",
      resp = "bill"
    ),
    set_prior("normal(0, 0.5)",
      class = "b",
      coef = "pretreatmentA",
      resp = "bill"
    ),
    set_prior("normal(0, 0.5)",
      class = "b",
      coef = "pretreatmentC",
      resp = "bill"
    ),
    set_prior("skew_normal(0, 0.25, 5)",
      class = "b",
      coef = "mass",
      resp = "bill"
    ),
    set_prior("exponential(5)",
      class = "sd",
      group = "batch",
      resp = "bill"
    ),
    set_prior("exponential(2.5)",
```

```
      class = "sigma",
      resp = "bill"
    ),
    set_prior("normal(2.5, 1)",
      class = "Intercept",
      resp = "foldEhl"
    ),
    set_prior("normal(0, 0.5)",
      class = "b",
      coef = "pretreatmentA",
      resp = "foldEhl"
    ),
    set_prior("normal(0, 0.5)",
      class = "b",
      coef = "pretreatmentC",
      resp = "foldEhl"
    ),
    set_prior("normal(0, 0.2)",
      class = "b",
      coef = "tarsus",
      resp = "foldEhl"
    ),
    set_prior("normal(0, 0.5)",
      class = "b",
      coef = "bill",
      resp = "foldEhl"
    ),
    set_prior("exponential(5)",
      class = "sd",
      group = "batch",
      resp = "foldEhl"
    ),
    set_prior("exponential(5)",
      class = "sigma",
      resp = "foldEhl"
    )
  ),
  iter = 50000, warmup = 10000, cores = 4, chains = 4, thin = 20,
  control = list(adapt_delta = .98, max_treedepth = 14),
  silent = TRUE, refresh = 0,
  file = "./models/_heatLossModel8WeeksMassR2.Rds",
)

ehlModel8WeeksTarsusR2 <- brm(
  data = ehl8WeekData,
  family = "gaussian",
  bf(mass ~ pretreatment + (1 | batch)) +
    bf(tarsus ~ mass + pretreatment + (1 | batch)) +
    bf(bill ~ mass + pretreatment + (1 | batch)) +
    bf(foldEhl ~ mass + bill + pretreatment + (1 | batch)) +
    set_rescor(FALSE),
  prior = c(
    set_prior("normal(0, 10)",
      class = "Intercept",
      resp = "mass"
    ),
    set_prior("normal(0, 25)",
      class = "b",
      coef = "pretreatmentA",
      resp = "mass"
    ),
    set_prior("normal(0, 25)",
      class = "b",
      coef = "pretreatmentC",
      resp = "mass"
    ),
    set_prior("exponential(2.5)",
```

```
    class = "sd",
    group = "batch",
    resp = "mass"
  ),
  set_prior("exponential(0.05)",
    class = "sigma",
    resp = "mass"
  ),
  set_prior("normal(0, 3)",
    class = "Intercept",
    resp = "tarsus"
  ),
  set_prior("normal(0, 3)",
    class = "b",
    coef = "pretreatmentA",
    resp = "tarsus"
  ),
  set_prior("normal(0, 3)",
    class = "b",
    coef = "pretreatmentC",
    resp = "tarsus"
  ),
  set_prior("skew_normal(0, 0.25, 5)",
    class = "b",
    coef = "mass",
    resp = "tarsus"
  ),
  set_prior("exponential(2)",
    class = "sd",
    group = "batch",
    resp = "tarsus"
  ),
  set_prior("exponential(0.75)",
    class = "sigma",
    resp = "tarsus"
  ),
  set_prior("normal(0, 1)",
    class = "Intercept",
    resp = "bill"
  ),
  set_prior("normal(0, 0.5)",
    class = "b",
    coef = "pretreatmentA",
    resp = "bill"
  ),
  set_prior("normal(0, 0.5)",
    class = "b",
    coef = "pretreatmentC",
    resp = "bill"
  ),
  set_prior("skew_normal(0, 0.25, 5)",
    class = "b",
    coef = "mass",
    resp = "bill"
  ),
  set_prior("exponential(5)",
    class = "sd",
    group = "batch",
    resp = "bill"
  ),
  set_prior("exponential(2.5)",
    class = "sigma",
    resp = "bill"
  ),
  set_prior("normal(2.5, 1)",
    class = "Intercept",
    resp = "foldEhl"
```

```
    ),
    set_prior("normal(0, 0.5)",
      class = "b",
      coef = "pretreatmentA",
      resp = "foldEhl"
    ),
    set_prior("normal(0, 0.5)",
      class = "b",
      coef = "pretreatmentC",
      resp = "foldEhl"
    ),
    set_prior("normal(0, 0.015)",
      class = "b",
      coef = "mass",
      resp = "foldEhl"
    ),
    set_prior("normal(0, 0.5)",
      class = "b",
      coef = "bill",
      resp = "foldEhl"
    ),
    set_prior("exponential(5)",
      class = "sd",
      group = "batch",
      resp = "foldEhl"
    ),
    set_prior("exponential(5)",
      class = "sigma",
      resp = "foldEhl"
    )
  ),
  iter = 50000, warmup = 10000, cores = 4, chains = 4, thin = 20,
  control = list(adapt_delta = .98, max_treedepth = 14),
  silent = TRUE, refresh = 0,
  file = "./models/_heatLossModel8WeeksTarsusR2.Rds",
)

ehlModel8WeeksBillR2 <- brm(
  data = ehl8WeekData,
  family = "gaussian",
  bf(mass ~ pretreatment + (1 | batch)) +
    bf(tarsus ~ mass + pretreatment + (1 | batch)) +
    bf(bill ~ mass + pretreatment + (1 | batch)) +
    bf(foldEhl ~ mass + tarsus + pretreatment + (1 | batch)) +
    set_rescor(FALSE),
  prior = c(
    set_prior("normal(0, 10)",
      class = "Intercept",
      resp = "mass"
    ),
    set_prior("normal(0, 25)",
      class = "b",
      coef = "pretreatmentA",
      resp = "mass"
    ),
    set_prior("normal(0, 25)",
      class = "b",
      coef = "pretreatmentC",
      resp = "mass"
    ),
    set_prior("exponential(2.5)",
      class = "sd",
      group = "batch",
      resp = "mass"
    ),
    set_prior("exponential(0.05)",
      class = "sigma",
```

```
    resp = "mass"
  ),
  set_prior("normal(0, 3)",
    class = "Intercept",
    resp = "tarsus"
  ),
  set_prior("normal(0, 3)",
    class = "b",
    coef = "pretreatmentA",
    resp = "tarsus"
  ),
  set_prior("normal(0, 3)",
    class = "b",
    coef = "pretreatmentC",
    resp = "tarsus"
  ),
  set_prior("skew_normal(0, 0.25, 5)",
    class = "b",
    coef = "mass",
    resp = "tarsus"
  ),
  set_prior("exponential(2)",
    class = "sd",
    group = "batch",
    resp = "tarsus"
  ),
  set_prior("exponential(0.75)",
    class = "sigma",
    resp = "tarsus"
  ),
  set_prior("normal(0, 1)",
    class = "Intercept",
    resp = "bill"
  ),
  set_prior("normal(0, 0.5)",
    class = "b",
    coef = "pretreatmentA",
    resp = "bill"
  ),
  set_prior("normal(0, 0.5)",
    class = "b",
    coef = "pretreatmentC",
    resp = "bill"
  ),
  set_prior("skew_normal(0, 0.25, 5)",
    class = "b",
    coef = "mass",
    resp = "bill"
  ),
  set_prior("exponential(5)",
    class = "sd",
    group = "batch",
    resp = "bill"
  ),
  set_prior("exponential(2.5)",
    class = "sigma",
    resp = "bill"
  ),
  set_prior("normal(2.5, 1)",
    class = "Intercept",
    resp = "foldEhl"
  ),
  set_prior("normal(0, 0.5)",
    class = "b",
    coef = "pretreatmentA",
    resp = "foldEhl"
  ),
```

```
    set_prior("normal(0, 0.5)",
      class = "b",
      coef = "pretreatmentC",
      resp = "foldEhl"
    ),
    set_prior("normal(0, 0.015)",
      class = "b",
      coef = "mass",
      resp = "foldEhl"
    ),
    set_prior("normal(0, 0.2)",
      class = "b",
      coef = "tarsus",
      resp = "foldEhl"
    ),
    set_prior("exponential(5)",
      class = "sd",
      group = "batch",
      resp = "foldEhl"
    ),
    set_prior("exponential(5)",
      class = "sigma",
      resp = "foldEhl"
    )
  ),
  iter = 50000, warmup = 10000, cores = 4, chains = 4, thin = 20,
  control = list(adapt_delta = .98, max_treedepth = 14),
  silent = TRUE, refresh = 0,
  file = "./models/_heatLossModel8WeeksBillR2.Rds",
)

ehlModel8WeeksAppendageR2 <- brm(
  data = ehl8WeekData,
  family = "gaussian",
  bf(mass ~ pretreatment + (1 | batch)) +
    bf(tarsus ~ mass + pretreatment + (1 | batch)) +
    bf(bill ~ mass + pretreatment + (1 | batch)) +
    bf(foldEhl ~ mass + pretreatment + (1 | batch)) +
    set_rescor(FALSE),
  prior = c(
    set_prior("normal(0, 10)",
      class = "Intercept",
      resp = "mass"
    ),
    set_prior("normal(0, 25)",
      class = "b",
      coef = "pretreatmentA",
      resp = "mass"
    ),
    set_prior("normal(0, 25)",
      class = "b",
      coef = "pretreatmentC",
      resp = "mass"
    ),
    set_prior("exponential(2.5)",
      class = "sd",
      group = "batch",
      resp = "mass"
    ),
    set_prior("exponential(0.05)",
      class = "sigma",
      resp = "mass"
    ),
    set_prior("normal(0, 3)",
      class = "Intercept",
      resp = "tarsus"
    ),
```

```
    set_prior("normal(0, 3)",
      class = "b",
      coef = "pretreatmentA",
      resp = "tarsus"
    ),
    set_prior("normal(0, 3)",
      class = "b",
      coef = "pretreatmentC",
      resp = "tarsus"
    ),
    set_prior("skew_normal(0, 0.25, 5)",
      class = "b",
      coef = "mass",
      resp = "tarsus"
    ),
    set_prior("exponential(2)",
      class = "sd",
      group = "batch",
      resp = "tarsus"
    ),
    set_prior("exponential(0.75)",
      class = "sigma",
      resp = "tarsus"
    ),
    set_prior("normal(0, 1)",
      class = "Intercept",
      resp = "bill"
    ),
    set_prior("normal(0, 0.5)",
      class = "b",
      coef = "pretreatmentA",
      resp = "bill"
    ),
    set_prior("normal(0, 0.5)",
      class = "b",
      coef = "pretreatmentC",
      resp = "bill"
    ),
    set_prior("skew_normal(0, 0.25, 5)",
      class = "b",
      coef = "mass",
      resp = "bill"
    ),
    set_prior("exponential(5)",
      class = "sd",
      group = "batch",
      resp = "bill"
    ),
    set_prior("exponential(2.5)",
      class = "sigma",
      resp = "bill"
    ),
    set_prior("normal(2.5, 1)",
      class = "Intercept",
      resp = "foldEhl"
    ),
    set_prior("normal(0, 0.5)",
      class = "b",
      coef = "pretreatmentA",
      resp = "foldEhl"
    ),
    set_prior("normal(0, 0.5)",
      class = "b",
      coef = "pretreatmentC",
      resp = "foldEhl"
    ),
    set_prior("normal(0, 0.015)",
```

```
        class = "b",
        coef = "mass",
        resp = "foldEhl"
      ),
      set_prior("exponential(5)",
        class = "sd",
        group = "batch",
        resp = "foldEhl"
      ),
      set_prior("exponential(5)",
        class = "sigma",
        resp = "foldEhl"
      )
    ),
    iter = 50000, warmup = 10000, cores = 4, chains = 4, thin = 20,
    control = list(adapt_delta = .98, max_treedepth = 14),
    silent = TRUE, refresh = 0,
    file = "./models/_heatLossModel8WeeksAppendageR2.Rds",
  )
}

## Evaporative cooling efficiency models

{
  efficiencyData8Weeks <-
    vh2o %>%
    filter(week == "8" & Ta == 40) %>%
    dplyr::select(Ta, ring, pretreatment,
      mass, tarsusLengthMean, billLengthMean, ecc,
      "batch" = exp
    ) %>%
    mutate(
      pretreatment =
        ifelse(pretreatment == "neutral", "B",
          ifelse(pretreatment == "cold", "A", "C")
        )
    ) %>%
    mutate(pretreatment = factor(pretreatment,
      levels = c("B", "A", "C")
    )) %>%
    distinct() %>%
    mutate(
      mass = mass - mean(mass, na.rm = T),
      tarsus = tarsusLengthMean -
        mean(tarsusLengthMean, na.rm = T),
      bill = billLengthMean -
        mean(billLengthMean, na.rm = T)
    )

  efficiencyModel8WeeksMassR2 <- brm(
    data = efficiencyData8Weeks,
    family = "gaussian",
    bf(mass ~ pretreatment + (1 | batch)) +
      bf(tarsus ~ mass + pretreatment + (1 | batch)) +
      bf(bill ~ mass + pretreatment + (1 | batch)) +
      bf(
        ecc ~ tarsus + bill + pretreatment + (1 | batch)
      ) +
      set_rescor(FALSE),
    prior = c(
      set_prior("normal(0, 10)",
        class = "Intercept",
        resp = "mass"
      ),
      set_prior("normal(0, 25)",
        class = "b",
        coef = "pretreatmentA",
```

```
    resp = "mass"
  ),
  set_prior("normal(0, 25)",
    class = "b",
    coef = "pretreatmentC",
    resp = "mass"
  ),
  set_prior("exponential(2.5)",
    class = "sd",
    group = "batch",
    resp = "mass"
  ),
  set_prior("exponential(0.05)",
    class = "sigma",
    resp = "mass"
  ),
  set_prior("normal(0, 3)",
    class = "Intercept",
    resp = "tarsus"
  ),
  set_prior("normal(0, 3)",
    class = "b",
    coef = "pretreatmentA",
    resp = "tarsus"
  ),
  set_prior("normal(0, 3)",
    class = "b",
    coef = "pretreatmentC",
    resp = "tarsus"
  ),
  set_prior("skew_normal(0, 0.25, 5)",
    class = "b",
    coef = "mass",
    resp = "tarsus"
  ),
  set_prior("exponential(2)",
    class = "sd",
    group = "batch",
    resp = "tarsus"
  ),
  set_prior("exponential(0.75)",
    class = "sigma",
    resp = "tarsus"
  ),
  set_prior("normal(0, 1)",
    class = "Intercept",
    resp = "bill"
  ),
  set_prior("normal(0, 0.5)",
    class = "b",
    coef = "pretreatmentA",
    resp = "bill"
  ),
  set_prior("normal(0, 0.5)",
    class = "b",
    coef = "pretreatmentC",
    resp = "bill"
  ),
  set_prior("skew_normal(0, 0.25, 5)",
    class = "b",
    coef = "mass",
    resp = "bill"
  ),
  set_prior("exponential(5)",
    class = "sd",
    group = "batch",
    resp = "bill"
```

```
    ),
    set_prior("exponential(2.5)",
      class = "sigma",
      resp = "bill"
    ),
    set_prior("normal(0.75, 0.2)",
      class = "Intercept",
      resp = "ecc"
    ),
    set_prior("normal(0, 0.25)",
      class = "b",
      coef = "pretreatmentA",
      resp = "ecc"
    ),
    set_prior("normal(0, 0.25)",
      class = "b",
      coef = "pretreatmentC",
      resp = "ecc"
    ),
    set_prior("normal(0, 0.1)",
      class = "b",
      coef = "tarsus",
      resp = "ecc"
    ),
    set_prior("normal(0, 0.15)",
      class = "b",
      coef = "bill",
      resp = "ecc"
    ),
    set_prior("exponential(15)",
      class = "sd",
      group = "batch",
      resp = "ecc"
    ),
    set_prior("exponential(5)",
      class = "sigma",
      resp = "ecc"
    )
  ),
  iter = 50000, warmup = 10000, cores = 4, chains = 4, thin = 20,
  control = list(adapt_delta = .98, max_treedepth = 14),
  silent = TRUE, refresh = 0,
  file = "./models/_efficiencyModel8WeeksMassR2.Rds"
)

efficiencyModel8WeeksTarsusR2 <- brm(
  data = efficiencyData8Weeks,
  family = "gaussian",
  bf(mass ~ pretreatment + (1 | batch)) +
    bf(tarsus ~ mass + pretreatment + (1 | batch)) +
    bf(bill ~ mass + pretreatment + (1 | batch)) +
    bf(
      ecc ~ mass + bill + pretreatment + (1 | batch)
    ) +
    set_rescor(FALSE),
  prior = c(
    set_prior("normal(0, 10)",
      class = "Intercept",
      resp = "mass"
    ),
    set_prior("normal(0, 25)",
      class = "b",
      coef = "pretreatmentA",
      resp = "mass"
    ),
    set_prior("normal(0, 25)",
      class = "b",
```

```
      coef = "pretreatmentC",
      resp = "mass"
    ),
    set_prior("exponential(2.5)",
      class = "sd",
      group = "batch",
      resp = "mass"
    ),
    set_prior("exponential(0.05)",
      class = "sigma",
      resp = "mass"
    ),
    set_prior("normal(0, 3)",
      class = "Intercept",
      resp = "tarsus"
    ),
    set_prior("normal(0, 3)",
      class = "b",
      coef = "pretreatmentA",
      resp = "tarsus"
    ),
    set_prior("normal(0, 3)",
      class = "b",
      coef = "pretreatmentC",
      resp = "tarsus"
    ),
    set_prior("skew_normal(0, 0.25, 5)",
      class = "b",
      coef = "mass",
      resp = "tarsus"
    ),
    set_prior("exponential(2)",
      class = "sd",
      group = "batch",
      resp = "tarsus"
    ),
    set_prior("exponential(0.75)",
      class = "sigma",
      resp = "tarsus"
    ),
    set_prior("normal(0, 1)",
      class = "Intercept",
      resp = "bill"
    ),
    set_prior("normal(0, 0.5)",
      class = "b",
      coef = "pretreatmentA",
      resp = "bill"
    ),
    set_prior("normal(0, 0.5)",
      class = "b",
      coef = "pretreatmentC",
      resp = "bill"
    ),
    set_prior("skew_normal(0, 0.25, 5)",
      class = "b",
      coef = "mass",
      resp = "bill"
    ),
    set_prior("exponential(5)",
      class = "sd",
      group = "batch",
      resp = "bill"
    ),
    set_prior("exponential(2.5)",
      class = "sigma",
      resp = "bill"
```

```
    ),
    set_prior("normal(0.75, 0.2)",
      class = "Intercept",
      resp = "ecc"
    ),
    set_prior("normal(0, 0.25)",
      class = "b",
      coef = "pretreatmentA",
      resp = "ecc"
    ),
    set_prior("normal(0, 0.25)",
      class = "b",
      coef = "pretreatmentC",
      resp = "ecc"
    ),
    set_prior("normal(0, 0.01)",
      class = "b",
      coef = "mass",
      resp = "ecc"
    ),
    set_prior("normal(0, 0.15)",
      class = "b",
      coef = "bill",
      resp = "ecc"
    ),
    set_prior("exponential(15)",
      class = "sd",
      group = "batch",
      resp = "ecc"
    ),
    set_prior("exponential(5)",
      class = "sigma",
      resp = "ecc"
    )
  ),
  iter = 50000, warmup = 10000, cores = 4, chains = 4, thin = 20,
  control = list(adapt_delta = .98, max_treedepth = 14),
  silent = TRUE, refresh = 0,
  file = "./models/_efficiencyModel8WeeksTarsusR2.Rds"
)

efficiencyModel8WeeksBillR2 <- brm(
  data = efficiencyData8Weeks,
  family = "gaussian",
  bf(mass ~ pretreatment + (1 | batch)) +
    bf(tarsus ~ mass + pretreatment + (1 | batch)) +
    bf(bill ~ mass + pretreatment + (1 | batch)) +
    bf(
      ecc ~ mass + tarsus + pretreatment + (1 | batch)
    ) +
    set_rescor(FALSE),
  prior = c(
    set_prior("normal(0, 10)",
      class = "Intercept",
      resp = "mass"
    ),
    set_prior("normal(0, 25)",
      class = "b",
      coef = "pretreatmentA",
      resp = "mass"
    ),
    set_prior("normal(0, 25)",
      class = "b",
      coef = "pretreatmentC",
      resp = "mass"
    ),
    set_prior("exponential(2.5)",
```

```
      class = "sd",
      group = "batch",
      resp = "mass"
    ),
    set_prior("exponential(0.05)",
      class = "sigma",
      resp = "mass"
    ),
    set_prior("normal(0, 3)",
      class = "Intercept",
      resp = "tarsus"
    ),
    set_prior("normal(0, 3)",
      class = "b",
      coef = "pretreatmentA",
      resp = "tarsus"
    ),
    set_prior("normal(0, 3)",
      class = "b",
      coef = "pretreatmentC",
      resp = "tarsus"
    ),
    set_prior("skew_normal(0, 0.25, 5)",
      class = "b",
      coef = "mass",
      resp = "tarsus"
    ),
    set_prior("exponential(2)",
      class = "sd",
      group = "batch",
      resp = "tarsus"
    ),
    set_prior("exponential(0.75)",
      class = "sigma",
      resp = "tarsus"
    ),
    set_prior("normal(0, 1)",
      class = "Intercept",
      resp = "bill"
    ),
    set_prior("normal(0, 0.5)",
      class = "b",
      coef = "pretreatmentA",
      resp = "bill"
    ),
    set_prior("normal(0, 0.5)",
      class = "b",
      coef = "pretreatmentC",
      resp = "bill"
    ),
    set_prior("skew_normal(0, 0.25, 5)",
      class = "b",
      coef = "mass",
      resp = "bill"
    ),
    set_prior("exponential(5)",
      class = "sd",
      group = "batch",
      resp = "bill"
    ),
    set_prior("exponential(2.5)",
      class = "sigma",
      resp = "bill"
    ),
    set_prior("normal(0.75, 0.2)",
      class = "Intercept",
      resp = "ecc"
```

```
    ),
    set_prior("normal(0, 0.25)",
      class = "b",
      coef = "pretreatmentA",
      resp = "ecc"
    ),
    set_prior("normal(0, 0.25)",
      class = "b",
      coef = "pretreatmentC",
      resp = "ecc"
    ),
    set_prior("normal(0, 0.01)",
      class = "b",
      coef = "mass",
      resp = "ecc"
    ),
    set_prior("normal(0, 0.1)",
      class = "b",
      coef = "tarsus",
      resp = "ecc"
    ),
    set_prior("exponential(15)",
      class = "sd",
      group = "batch",
      resp = "ecc"
    ),
    set_prior("exponential(5)",
      class = "sigma",
      resp = "ecc"
    )
  ),
  iter = 50000, warmup = 10000, cores = 4, chains = 4, thin = 20,
  control = list(adapt_delta = .98, max_treedepth = 14),
  silent = TRUE, refresh = 0,
  file = "./models/_efficiencyModel8WeeksBillR2.Rds"
)

efficiencyModel8WeeksAppendageR2 <- brm(
  data = efficiencyData8Weeks,
  family = "gaussian",
  bf(mass ~ pretreatment + (1 | batch)) +
    bf(tarsus ~ mass + pretreatment + (1 | batch)) +
    bf(bill ~ mass + pretreatment + (1 | batch)) +
    bf(
      ecc ~ mass + pretreatment + (1 | batch)
    ) +
    set_rescor(FALSE),
  prior = c(
    set_prior("normal(0, 10)",
      class = "Intercept",
      resp = "mass"
    ),
    set_prior("normal(0, 25)",
      class = "b",
      coef = "pretreatmentA",
      resp = "mass"
    ),
    set_prior("normal(0, 25)",
      class = "b",
      coef = "pretreatmentC",
      resp = "mass"
    ),
    set_prior("exponential(2.5)",
      class = "sd",
      group = "batch",
      resp = "mass"
    ),
```

```
    set_prior("exponential(0.05)",
      class = "sigma",
      resp = "mass"
    ),
    set_prior("normal(0, 3)",
      class = "Intercept",
      resp = "tarsus"
    ),
    set_prior("normal(0, 3)",
      class = "b",
      coef = "pretreatmentA",
      resp = "tarsus"
    ),
    set_prior("normal(0, 3)",
      class = "b",
      coef = "pretreatmentC",
      resp = "tarsus"
    ),
    set_prior("skew_normal(0, 0.25, 5)",
      class = "b",
      coef = "mass",
      resp = "tarsus"
    ),
    set_prior("exponential(2)",
      class = "sd",
      group = "batch",
      resp = "tarsus"
    ),
    set_prior("exponential(0.75)",
      class = "sigma",
      resp = "tarsus"
    ),
    set_prior("normal(0, 1)",
      class = "Intercept",
      resp = "bill"
    ),
    set_prior("normal(0, 0.5)",
      class = "b",
      coef = "pretreatmentA",
      resp = "bill"
    ),
    set_prior("normal(0, 0.5)",
      class = "b",
      coef = "pretreatmentC",
      resp = "bill"
    ),
    set_prior("skew_normal(0, 0.25, 5)",
      class = "b",
      coef = "mass",
      resp = "bill"
    ),
    set_prior("exponential(5)",
      class = "sd",
      group = "batch",
      resp = "bill"
    ),
    set_prior("exponential(2.5)",
      class = "sigma",
      resp = "bill"
    ),
    set_prior("normal(0.75, 0.2)",
      class = "Intercept",
      resp = "ecc"
    ),
    set_prior("normal(0, 0.25)",
      class = "b",
      coef = "pretreatmentA",
```

```
        resp = "ecc"
      ),
      set_prior("normal(0, 0.25)",
        class = "b",
        coef = "pretreatmentC",
        resp = "ecc"
      ),
      set_prior("normal(0, 0.01)",
        class = "b",
        coef = "mass",
        resp = "ecc"
      ),
      set_prior("exponential(15)",
        class = "sd",
        group = "batch",
        resp = "ecc"
      ),
      set_prior("exponential(5)",
        class = "sigma",
        resp = "ecc"
      )
    ),
    iter = 50000, warmup = 10000, cores = 4, chains = 4, thin = 20,
    control = list(adapt_delta = .98, max_treedepth = 14),
    silent = TRUE, refresh = 0,
    file = "./models/_efficiencyModel8WeeksAppendageR2.Rds"
  )
}

pR2Pull <- function(baseModel, response, x) {
  baseR2 <- as.data.frame(
    brms::bayes_R2(baseModel,
      ndraws = 1000,
      resp = response, summary = FALSE,
      robust = TRUE
    )
  )[,1]
  redR2 <- as.data.frame(
    brms::bayes_R2(x,
      ndraws = 1000,
      resp = response, summary = FALSE,
      robust = TRUE
    )
  )[,1]
  pR2 <- round(baseR2 - redR2, digits = 3)

  ciFrame <- t(
    quantile(pR2, probs = c(0.025, 0.975), type = 8)
  ) %>% as.data.frame()

  ciFrame <- ciFrame %>%
    mutate(`2.5%` = ifelse(`2.5%` < 0, 0, `2.5%`)) %>%
    mutate("95\\% CI" = paste0(
      "[",
      round(`2.5%`, digits = 3),
      ",",
      round(`97.5%`, digits = 3),
      "]"
    )) %>%
    mutate("Partial R2" = round(median(pR2), digits = 3)) %>%
    mutate(`Partial R2` = ifelse(`Partial R2` < 0, 0, `Partial R2`)) %>%
    dplyr::select("Partial R\\textsuperscript{2}" = `Partial R2`, "95\\% CI")

  return(ciFrame)
}

caption = paste0(
```

```
  'Variance in evaporative heat loss ("EHL", fold change ',
  'from 30°C) and evaporative cooling efficiency ("ECE", the ratio of evaporative ',
  'heat loss to metabolic heat production) at at 40°C explained by ',
  'morphometry in eight week old Japanese quail.'
  )

models <- list(ehlModel8WeeksMassR2, ehlModel8WeeksTarsusR2,
            ehlModel8WeeksBillR2, ehlModel8WeeksAppendageR2)
hold <- bind_rows(
  lapply(models, pR2Pull, baseModel = ehlModel3Weeks, response = "foldEhl")
  ) %>%
  mutate("Variable" = c("Body Mass", "Tarsus Length",
                        "Bill Length", "Appendage Length"),
         "Response" = "EHL"
         ) %>%
  dplyr::select(
    Response, Variable, "Partial R\\textsuperscript{2}", `95\\% CI`
  )

models <- list(efficiencyModel8WeeksMassR2, efficiencyModel8WeeksTarsusR2,
            efficiencyModel8WeeksBillR2, efficiencyModel8WeeksAppendageR2)
bind_rows(
  lapply(models, pR2Pull, baseModel = efficiencyModel8Weeks,
         response = "ecc")
  ) %>%
  mutate("Variable" = c("Body Mass", "Tarsus Length",
                        "Bill Length", "Appendage Length"),
         "Response" = "ECE") %>%
  dplyr::select(
    Response, Variable, "Partial R\\textsuperscript{2}", `95\\% CI`
  ) %>%
  rbind(hold, .) %>%
  kbl(.,
    longtable = T, booktabs = T, format = "latex",
    caption = caption, escape = FALSE
  ) %>%
  column_spec(column = c(1:10), width = "2.5cm")  %>%
  kable_styling(latex_options = "striped")
```

Table 105: *Variance in evaporative heat loss ("EHL", fold change from 30°C) and evaporative cooling efficiency ("ECE", the ratio of evaporative heat loss to metabolic heat production) at at 40°C explained by morphometry in eight week old Japanese quail.*

Response	Variable	Partial R^2	95% CI
EHL	Body Mass	0.088	[0,0.285]
EHL	Tarsus Length	0.092	[0,0.308]
EHL	Bill Length	0.105	[0,0.341]
EHL	Appendage Length	0.120	[0,0.341]
ECE	Body Mass	0.045	[0,0.226]
ECE	Tarsus Length	0.011	[0,0.197]
ECE	Bill Length	0.030	[0,0.218]
ECE	Appendage Length	0.035	[0,0.236]

```
rm(models)
```

Below, conditional effects of body mass, tarsus length, bill length, and rearing treatment on both evaporative heat loss responses and evaporative cooling efficiency (both at 40°C) are plotted. All plots assume otherwise average morphology or mild rearing (20°C).

```
heatLoss8WeeksMassPlot <-
  expand.grid(
    "mass" = with(
      ehlModel8Weeks$data,
```

```
      seq(min(mass), max(mass), by = 1)
    ),
    "tarsus" = 0,
    "bill" = 0,
    "pretreatment" = "B"
  ) %>%
  mutate(
    "foldEhl" = predict(ehlModel8Weeks,
      newdata = .,
      re_form = NA, robust = TRUE,
      resp = "foldEhl"
    )[, "Estimate"],
    "SE" = predict(ehlModel8Weeks,
      newdata = .,
      re_form = NA, robust = TRUE,
      resp = "foldEhl"
    )[, "Est.Error"]
  ) %>%
  mutate(mass = mass + mean(subset(vh2o, week == 8)$mass, na.rm = T)) %>%
  ggplot(aes(x = mass, y = foldEhl)) +
  geom_ribbon(aes(ymin = foldEhl - SE, ymax = foldEhl + SE),
    alpha = 0.5, fill = "#B35050"
  ) +
  geom_point(
    data = ehlModel8Weeks$data %>%
      mutate(mass = mass +
        mean(subset(vh2o, week == 8)$mass, na.rm = T)),
    aes(x = mass, y = foldEhl),
    alpha = 0.9
  ) +
  geom_smooth(
    method = "lm", colour = "black",
    linetype = "dashed", se = FALSE
  ) +
  xlab("Body Mass (g)") +
  ylab("Evaporative Heat Loss\n(Fold From 30°C)") +
  theme_classic() +
  theme(
    axis.title = element_text(family = "Noto Sans"),
    axis.text = element_text(family = "Noto Sans")
  )

heatLoss8WeeksTarsusPlot <-
  expand.grid(
    "tarsus" = with(
      ehlModel8Weeks$data,
      seq(min(tarsus), max(tarsus), by = 1)
    ),
    "mass" = 0,
    "bill" = 0,
    "pretreatment" = "B"
  ) %>%
  mutate(
    "foldEhl" = predict(ehlModel8Weeks,
      newdata = .,
      re_form = NA, robust = TRUE,
      resp = "foldEhl"
    )[, "Estimate"],
    "SE" = predict(ehlModel8Weeks,
      newdata = .,
      re_form = NA, robust = TRUE,
      resp = "foldEhl"
    )[, "Est.Error"]
  ) %>%
  mutate(tarsus = tarsus +
           mean(subset(vh2o, week == 8)$tarsusLengthMean, na.rm = T)) %>%
  ggplot(aes(x = tarsus, y = foldEhl)) +
```

```
  geom_ribbon(aes(ymin = foldEhl - SE, ymax = foldEhl + SE),
    alpha = 0.5, fill = "#B35050"
  ) +
  geom_point(
    data = ehlModel8Weeks$data %>%
      mutate(tarsus = tarsus +
        mean(subset(vh2o, week == 8)$tarsusLengthMean, na.rm = T)),
    aes(x = tarsus, y = foldEhl),
    alpha = 0.5
  ) +
  geom_smooth(
    method = "lm", colour = "black",
    linetype = "dashed", se = FALSE
  ) +
  xlab("Tarsus Length (mm)") +
  ylab("Evaporative Heat Loss\n(Fold From 30°C)") +
  theme_classic() +
  theme(
    axis.title = element_text(family = "Noto Sans"),
    axis.text = element_text(family = "Noto Sans")
  )

heatLoss8WeeksBillPlot <-
  expand.grid(
    "bill" = with(
      ehlModel8Weeks$data,
      seq(min(bill), max(bill), by = 1)
    ),
    "mass" = 0,
    "tarsus" = 0,
    "pretreatment" = "B"
  ) %>%
  mutate(
    "foldEhl" = predict(ehlModel8Weeks,
      newdata = .,
      re_form = NA, robust = TRUE,
      resp = "foldEhl"
    )[, "Estimate"],
    "SE" = predict(ehlModel8Weeks,
      newdata = .,
      re_form = NA, robust = TRUE,
      resp = "foldEhl"
    )[, "Est.Error"]
  ) %>%
  mutate(bill = bill +
           mean(subset(vh2o, week == 8)$billLengthMean, na.rm = T)) %>%
  ggplot(aes(x = bill, y = foldEhl)) +
  geom_ribbon(aes(ymin = foldEhl - SE, ymax = foldEhl + SE),
    alpha = 0.5, fill = "#B35050"
  ) +
  geom_point(
    data = ehlModel8Weeks$data %>%
      mutate(bill = bill +
        mean(subset(vh2o, week == 8)$billLengthMean, na.rm = T)),
    aes(x = bill, y = foldEhl),
    alpha = 0.5
  ) +
  geom_smooth(
    method = "lm", colour = "black",
    linetype = "dashed", se = FALSE
  ) +
  xlab("Bill Length (mm)") +
  ylab("Evaporative Heat Loss\n(Fold From 30°C)") +
  theme_classic() +
  theme(
    axis.title = element_text(family = "Noto Sans"),
    axis.text = element_text(family = "Noto Sans")
```

```
  )

efficiency8WeeksMassPlot <-
  expand.grid(
    "mass" = with(
      efficiencyModel8Weeks$data,
      seq(min(mass), max(mass), by = 1)
    ),
    "tarsus" = 0,
    "bill" = 0,
    "pretreatment" = "B"
  ) %>%
  mutate(
    "ecc" = predict(efficiencyModel8Weeks,
      newdata = .,
      re_form = NA, robust = TRUE,
      resp = "ecc"
    )[, "Estimate"],
    "SE" = predict(efficiencyModel8Weeks,
      newdata = .,
      re_form = NA, robust = TRUE,
      resp = "ecc"
    )[, "Est.Error"]
  ) %>%
  mutate(mass = mass + mean(subset(vh2o, week == 8)$mass, na.rm = T)) %>%
  ggplot(aes(x = mass, y = ecc)) +
  geom_ribbon(aes(ymin = ecc - SE, ymax = ecc + SE),
    alpha = 0.5, fill = "#DECCC1"
  ) +
  geom_point(
    data = efficiencyModel8Weeks$data %>%
      mutate(mass = mass +
        mean(subset(vh2o, week == 8)$mass, na.rm = T)),
    aes(x = mass, y = ecc)
  ) +
  geom_smooth(
    method = "lm", colour = "black",
    linetype = "dashed", se = FALSE
  ) +
  xlab("Body Mass (g)") +
  ylab("Evaporative Cooling\nEfficiency (EHL/RMR)") +
  theme_classic() +
  theme(
    axis.title = element_text(family = "Noto Sans"),
    axis.text = element_text(family = "Noto Sans")
  )

efficiency8WeeksTarsusPlot <-
  expand.grid(
    "tarsus" = with(
      efficiencyModel8Weeks$data,
      seq(min(tarsus), max(tarsus), by = 1)
    ),
    "mass" = 0,
    "bill" = 0,
    "pretreatment" = "B"
  ) %>%
  mutate(
    "ecc" = predict(efficiencyModel8Weeks,
      newdata = .,
      re_form = NA, robust = TRUE,
      resp = "ecc"
    )[, "Estimate"],
    "SE" = predict(efficiencyModel8Weeks,
      newdata = .,
      re_form = NA, robust = TRUE,
      resp = "ecc"
```

```
    )[, "Est.Error"]
  ) %>%
  mutate(tarsus = tarsus +
           mean(subset(vh2o, week == 8)$tarsusLengthMean, na.rm = T)) %>%
  ggplot(aes(x = tarsus, y = ecc)) +
  geom_ribbon(aes(ymin = ecc - SE, ymax = ecc + SE),
    alpha = 0.5, fill = "#DECCC1"
  ) +
  geom_point(
    data = efficiencyModel8Weeks$data %>%
      mutate(tarsus = tarsus +
        mean(subset(vh2o, week == 8)$tarsusLengthMean, na.rm = T)),
    aes(x = tarsus, y = ecc),
    alpha = 0.5
  ) +
  geom_smooth(
    method = "lm", colour = "black",
    linetype = "dashed", se = FALSE
  ) +
  xlab("Tarsus Length (mm)") +
  ylab("Evaporative Cooling\nEfficiency (EHL/RMR)") +
  theme_classic() +
  theme(
    axis.title = element_text(family = "Noto Sans"),
    axis.text = element_text(family = "Noto Sans")
  )

efficiency8WeeksBillPlot <-
  expand.grid(
    "bill" = with(
      efficiencyModel8Weeks$data,
      seq(min(bill), max(bill), by = 1)
    ),
    "mass" = 0,
    "tarsus" = 0,
    "pretreatment" = "B"
  ) %>%
  mutate(
    "ecc" = predict(efficiencyModel8Weeks,
      newdata = .,
      re_form = NA, robust = TRUE,
      resp = "ecc"
    )[, "Estimate"],
    "SE" = predict(efficiencyModel8Weeks,
      newdata = .,
      re_form = NA, robust = TRUE,
      resp = "ecc"
    )[, "Est.Error"]
  ) %>%
  mutate(bill = bill +
           mean(subset(vh2o, week == 8)$billLengthMean, na.rm = T)) %>%
  ggplot(aes(x = bill, y = ecc)) +
  geom_ribbon(aes(ymin = ecc - SE, ymax = ecc + SE),
    alpha = 0.5, fill = "#DECCC1"
  ) +
  geom_point(
    data = efficiencyModel8Weeks$data %>%
      mutate(bill = bill +
        mean(subset(vh2o, week == 8)$billLengthMean, na.rm = T)),
    aes(x = bill, y = ecc),
    alpha = 0.5
  ) +
  geom_smooth(
    method = "lm", colour = "black",
    linetype = "dashed", se = FALSE
  ) +
  xlab("Bill Length (mm)") +
```

```
  ylab("Evaporative Cooling\nEfficiency (EHL/RMR)") +
  theme_classic() +
  theme(
    axis.title = element_text(family = "Noto Sans"),
    axis.text = element_text(family = "Noto Sans")
  )

(
  heatLoss8WeeksMassPlot + heatLoss8WeeksTarsusPlot + heatLoss8WeeksBillPlot
) / (
  efficiency8WeeksMassPlot + efficiency8WeeksTarsusPlot + efficiency8WeeksBillPlot
) +
  plot_annotation(tag_level = "A")
```

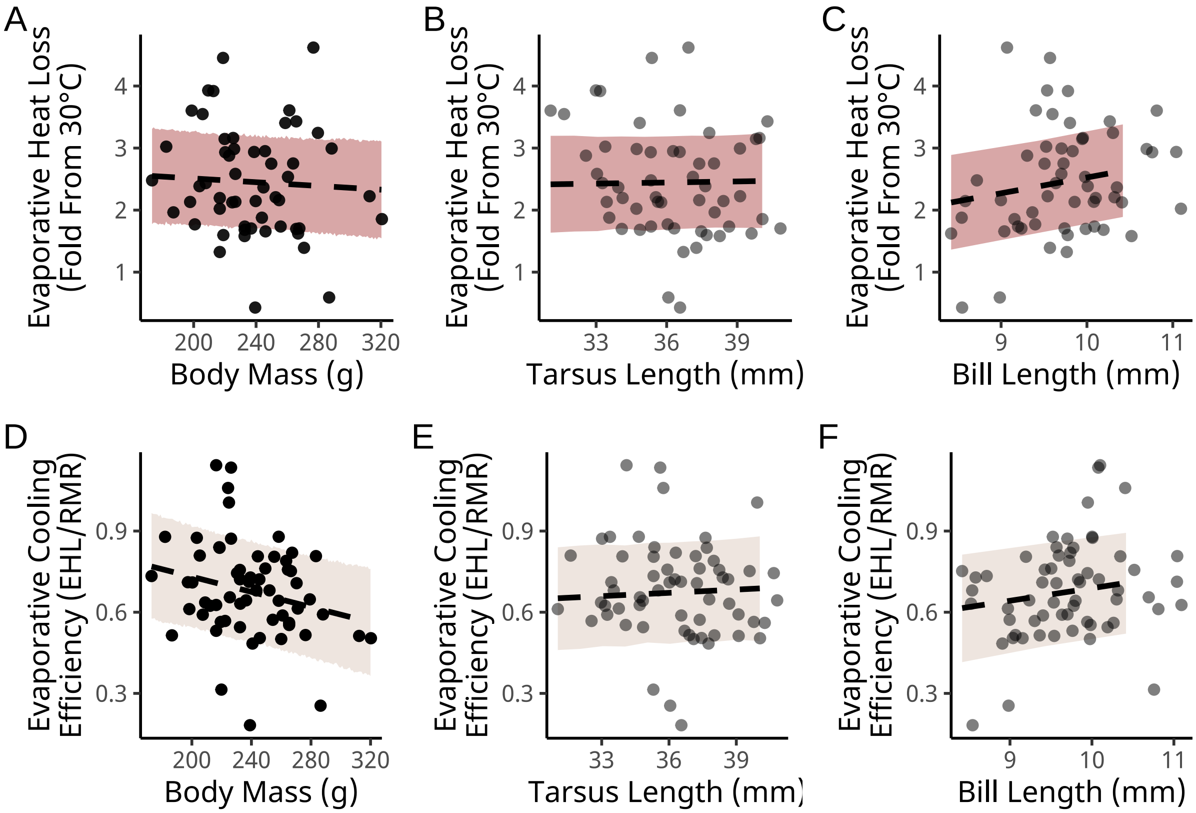

Figure 166: *Effects of morphology on evaporative heat loss responses (panels A to C and evaporative cooling efficiency (panels D to F) in mature Japanese quail (eight weeks of age; n = 57). Evaporative heat loss responses represent fold increases in evaporative heat loss, in watts, between 30°C and 40°C. Evaporative cooling efficiency represents the ratio between evaporative heat loss (in watts) and metabolic heat production (in Watts). Dashed lines indicate predicted relationships, as estimated from Bayesian path analyses. Ribbons represents +/- one standard error around predicted lines of best fit. Small dots indicate raw data points.*

```
heatLoss8WeeksTreatmentPlot <-
  expand.grid(
    "mass" = 0,
    "tarsus" = 0,
    "bill" = 0,
    "pretreatment" = c("A", "B", "C")
  ) %>%
  mutate(
    "foldEhl" = predict(ehlModel8Weeks,
      newdata = .,
```

```
      re_form = NA, robust = TRUE,
      resp = "foldEhl"
    )[, "Estimate"],
    "SE" = predict(ehlModel8Weeks,
      newdata = .,
      re_form = NA, robust = TRUE,
      resp = "foldEhl"
    )[, "Est.Error"]
  ) %>%
  mutate(pretreatment = factor(pretreatment, levels = c("A", "B", "C"))) %>%
  ggplot(aes(x = pretreatment, y = foldEhl, fill = pretreatment)) +
  geom_point(
    data = ehlModel8Weeks$data %>%
      mutate(pretreatment = factor(pretreatment, levels = c("A", "B", "C"))),
    aes(x = pretreatment, y = foldEhl),
    alpha = 0.5, position = position_jitter(width = 0.25)
  ) +
  geom_errorbar(aes(ymin = foldEhl - SE, ymax = foldEhl + SE),
    colour = "black", width = 0.25
  ) +
  geom_point(
    pch = 21, colour = "black", size = 4
  ) +
  scale_x_discrete(name = "Rearing Treatment",
                   labels = c("Cold (10°C)",
                              "Mild (20°C)",
                              "Warm (30°C)")
                   ) +
  ylab("Evaporative Heat Loss\nResponse (Fold From 30°C)") +
  scale_fill_manual(values = c("#7BB4E3", "black", "#CD5C5C")) +
  theme_classic() +
  theme(
    axis.title = element_text(family = "Noto Sans"),
    axis.text = element_text(family = "Noto Sans"),
    legend.position = "none"
  )

efficiency8WeeksTreatmentPlot <-
  expand.grid(
    "mass" = 0,
    "tarsus" = 0,
    "bill" = 0,
    "pretreatment" = c("A", "B", "C")
  ) %>%
  mutate(
    "ecc" = predict(efficiencyModel8Weeks,
      newdata = .,
      re_form = NA, robust = TRUE,
      resp = "ecc"
    )[, "Estimate"],
    "SE" = predict(efficiencyModel8Weeks,
      newdata = .,
      re_form = NA, robust = TRUE,
      resp = "ecc"
    )[, "Est.Error"]
  ) %>%
  mutate(pretreatment = factor(pretreatment, levels = c("A", "B", "C"))) %>%
  ggplot(aes(x = pretreatment, y = ecc, fill = pretreatment)) +
  geom_point(
    data = efficiencyModel8Weeks$data %>%
      mutate(pretreatment = factor(pretreatment, levels = c("A", "B", "C"))),
    aes(x = pretreatment, y = ecc),
    alpha = 0.5, position = position_jitter(width = 0.25)
  ) +
  geom_errorbar(aes(ymin = ecc - SE, ymax = ecc + SE),
    colour = "black", width = 0.25
  ) +
```

```
  geom_point(
    pch = 21, colour = "black", size = 4
  ) +
  scale_x_discrete(name = "Rearing Treatment",
                   labels = c("Cold (10°C)",
                              "Mild (20°C)",
                              "Warm (30°C)")
                   ) +
  ylab("Evaporative Cooling\nEfficiency (EHL/RMR)") +
  scale_fill_manual(values = c("#7BB4E3", "black", "#CD5C5C")) +
  theme_classic() +
  theme(
    axis.title = element_text(family = "Noto Sans"),
    axis.text = element_text(family = "Noto Sans"),
    legend.position = "none"
  )

(heatLoss8WeeksTreatmentPlot / efficiency8WeeksTreatmentPlot) +
  plot_annotation(tag_levels = "A")
```

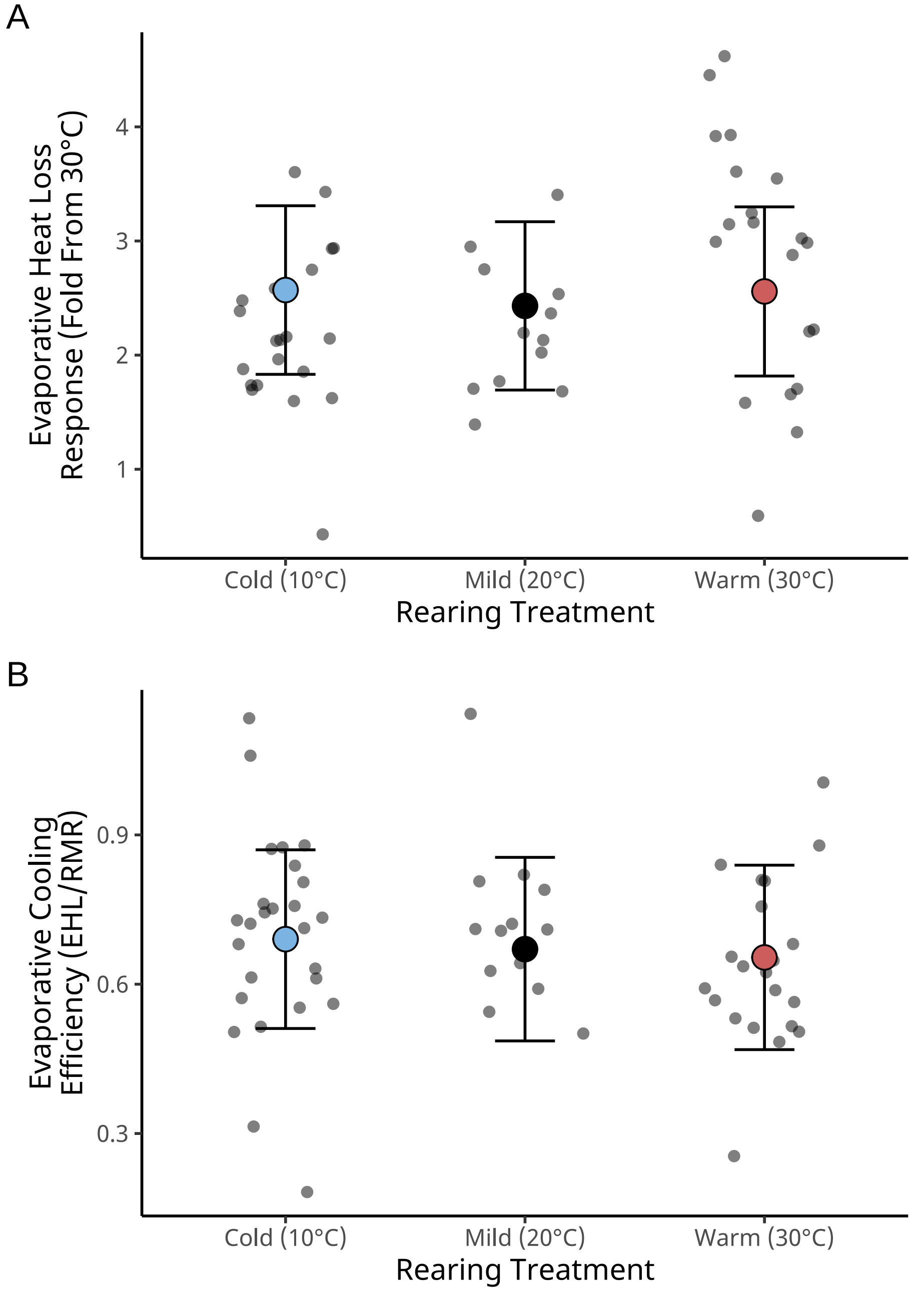

Figure 167: *Effects of rearing temperature on evaporative heat loss responses (panel A) and evaporative cooling efficiency (panel B) in mature Japanese quail (eight weeks of age; n = 57). Evaporative heat loss responses represent fold increases in evaporative heat loss, in Watts, between 30°C and 40°C. Evaporative cooling efficiency represents the ratio between evaporative heat loss (in watts) and metabolic heat production (in watts). Dashed lines indicate predicted relationships, as estimated from Bayesian path analyses. Ribbons represents +/- one standard error around predicted lines of best fit. Small dots indicate raw data points.*

Last, we again estimate and visualise the direct and indirect effects of rearing conditions (and body mass) on evaporative heat loss responses and evaporative cooling efficiency (both at 40°C). Similar to our estimations for developing quail, model coefficients are first scaled to represent the effect of changing a given predictor variable by one standard deviation (or categorical level) on a change, in standard deviations, of the response variable.

```
# Coefficients are first scaled

scaledBetasHeatLoss8 <- as.data.frame(ehlModel8Weeks) %>%
  mutate(
    b_mass_pretreatmentA = b_mass_pretreatmentA /
      sd(ehlModel8Weeks$data$mass),
    b_mass_pretreatmentC = b_mass_pretreatmentC /
      sd(ehlModel8Weeks$data$mass),
    b_tarsus_pretreatmentA = b_tarsus_pretreatmentA /
      sd(ehlModel8Weeks$data$tarsus),
    b_tarsus_pretreatmentC = b_tarsus_pretreatmentC /
      sd(ehlModel8Weeks$data$tarsus),
    b_tarsus_mass =
      (b_tarsus_mass * sd(ehlModel8Weeks$data$mass)) /
        sd(ehlModel8Weeks$data$tarsus),
    b_bill_pretreatmentA = b_bill_pretreatmentA /
      sd(ehlModel8Weeks$data$bill),
    b_tarsus_pretreatmentC = b_tarsus_pretreatmentC /
      sd(ehlModel8Weeks$data$bill),
    b_bill_mass =
      (b_bill_mass * sd(ehlModel8Weeks$data$mass)) /
        sd(ehlModel8Weeks$data$bill),
    b_foldEhl_mass =
      (b_foldEhl_mass * sd(ehlModel8Weeks$data$mass)) /
        sd(ehlModel8Weeks$data$foldEhl),
    b_foldEhl_tarsus =
      (b_foldEhl_tarsus * sd(ehlModel8Weeks$data$tarsus)) /
        sd(ehlModel8Weeks$data$foldEhl),
    b_foldEhl_bill =
      (b_foldEhl_bill * sd(ehlModel8Weeks$data$bill)) /
        sd(ehlModel8Weeks$data$foldEhl),
    b_foldEhl_pretreatmentA = b_foldEhl_pretreatmentA /
      sd(ehlModel8Weeks$data$foldEhl),
    b_foldEhl_pretreatmentC = b_foldEhl_pretreatmentC /
      sd(ehlModel8Weeks$data$foldEhl),
  )

scaledBetasEfficiency8 <- as.data.frame(efficiencyModel8Weeks) %>%
  mutate(
    b_mass_pretreatmentA = b_mass_pretreatmentA /
      sd(efficiencyModel8Weeks$data$mass),
    b_mass_pretreatmentC = b_mass_pretreatmentC /
      sd(efficiencyModel8Weeks$data$mass),
    b_tarsus_pretreatmentA = b_tarsus_pretreatmentA /
      sd(efficiencyModel8Weeks$data$tarsus),
    b_tarsus_pretreatmentC = b_tarsus_pretreatmentC /
      sd(efficiencyModel8Weeks$data$tarsus),
    b_tarsus_mass =
      (b_tarsus_mass * sd(efficiencyModel8Weeks$data$mass)) /
        sd(efficiencyModel8Weeks$data$tarsus),
    b_bill_pretreatmentA = b_bill_pretreatmentA /
      sd(efficiencyModel8Weeks$data$bill),
    b_bill_pretreatmentC = b_bill_pretreatmentC /
      sd(efficiencyModel8Weeks$data$bill),
    b_bill_mass =
      (b_bill_mass * sd(efficiencyModel8Weeks$data$mass)) /
        sd(efficiencyModel8Weeks$data$bill),
    b_ecc_mass =
      (b_ecc_mass * sd(efficiencyModel8Weeks$data$mass)) /
        sd(efficiencyModel8Weeks$data$ecc),
    b_ecc_tarsus =
      (b_ecc_tarsus * sd(efficiencyModel8Weeks$data$tarsus)) /
        sd(efficiencyModel8Weeks$data$ecc),
    b_ecc_bill =
      (b_ecc_bill * sd(efficiencyModel8Weeks$data$bill)) /
        sd(efficiencyModel8Weeks$data$ecc),
    b_ecc_pretreatmentA = b_ecc_pretreatmentA /
```

```
      sd(efficiencyModel8Weeks$data$ecc),
    b_ecc_pretreatmentC = b_ecc_pretreatmentC /
      sd(efficiencyModel8Weeks$data$ecc),
  )

# Calculating full effects

fullEffectHeatLoss8 <- scaledBetasHeatLoss8 %>%
  mutate("Effects" = "Direct Effects") %>%
  mutate(
    "Body Mass" = b_foldEhl_mass,
    "Tarsus Length" = b_foldEhl_tarsus,
    "Bill Length" = b_foldEhl_bill,
    "Cold Rearing\n(10°C)" = b_foldEhl_pretreatmentA,
    "Warm Rearing\n(30°C)" = b_foldEhl_pretreatmentC
  ) %>%
  dplyr::select(
    Effects, `Body Mass`, `Tarsus Length`, `Bill Length`,
    `Cold Rearing\n(10°C)`, `Warm Rearing\n(30°C)`
  ) %>%
  rbind(
    .,
    scaledBetasHeatLoss8 %>%
      mutate("Effects" = "Indirect Effects") %>%
      mutate(
        "Body Mass" = (b_tarsus_mass * b_foldEhl_tarsus) +
          (b_bill_mass * b_foldEhl_bill),
        "Tarsus Length" = NA,
        "Bill Length" = NA,
        "Cold Rearing\n(10°C)" =
          (b_mass_pretreatmentA * b_foldEhl_mass) +
            (b_tarsus_pretreatmentA * b_foldEhl_tarsus) +
            (b_bill_pretreatmentA * b_foldEhl_bill) +
            (b_mass_pretreatmentA * b_tarsus_mass * b_foldEhl_tarsus) +
            (b_mass_pretreatmentA * b_bill_mass * b_foldEhl_bill),
        "Warm Rearing\n(30°C)" =
          (b_mass_pretreatmentC * b_foldEhl_mass) +
            (b_tarsus_pretreatmentC * b_foldEhl_tarsus) +
            (b_bill_pretreatmentC * b_foldEhl_bill) +
            (b_mass_pretreatmentC * b_tarsus_mass * b_foldEhl_tarsus) +
            (b_mass_pretreatmentC * b_bill_mass * b_foldEhl_bill),
      ) %>%
      dplyr::select(
        Effects, `Body Mass`, `Tarsus Length`, `Bill Length`,
        `Cold Rearing\n(10°C)`, `Warm Rearing\n(30°C)`
      )
  ) %>%
  rbind(., scaledBetasHeatLoss8 %>%
    mutate("Effects" = "Total Effects") %>%
    mutate(
      "Body Mass" =
        b_foldEhl_mass +
          b_tarsus_mass * b_foldEhl_tarsus,
      "Tarsus Length" =
        b_foldEhl_tarsus,
      "Bill Length" =
        b_foldEhl_bill,
      "Cold Rearing\n(10°C)" =
        (b_foldEhl_pretreatmentA) +
          (b_mass_pretreatmentA * b_foldEhl_mass) +
          (b_tarsus_pretreatmentA * b_foldEhl_tarsus) +
          (b_bill_pretreatmentA * b_foldEhl_bill) +
          (b_mass_pretreatmentA * b_tarsus_mass * b_foldEhl_tarsus) +
          (b_mass_pretreatmentA * b_bill_mass * b_foldEhl_bill),
      "Warm Rearing\n(30°C)" =
        (b_foldEhl_pretreatmentC) +
          (b_mass_pretreatmentC * b_foldEhl_mass) +
```

```
          (b_tarsus_pretreatmentC * b_foldEhl_tarsus) +
          (b_bill_pretreatmentC * b_foldEhl_bill) +
          (b_mass_pretreatmentC * b_tarsus_mass * b_foldEhl_tarsus) +
          (b_mass_pretreatmentC * b_bill_mass * b_foldEhl_bill),
    ) %>%
    dplyr::select(
      Effects, `Body Mass`, `Tarsus Length`, `Bill Length`,
      `Cold Rearing\n(10°C)`, `Warm Rearing\n(30°C)`
    )) %>%
  pivot_longer(c(-Effects), names_to = "var", values_to = "values") %>%
  mutate(var = factor(var,
    levels = c(
      "Body Mass",
      "Tarsus Length",
      "Bill Length",
      "Warm Rearing\n(30°C)",
      "Cold Rearing\n(10°C)"
    )
  ))

fullEffectEfficiency8 <- scaledBetasEfficiency8 %>%
  mutate("Effects" = "Direct Effects") %>%
  mutate(
    "Body Mass" = b_ecc_mass,
    "Tarsus Length" = b_ecc_tarsus,
    "Bill Length" = b_ecc_bill,
    "Cold Rearing\n(10°C)" = b_ecc_pretreatmentA,
    "Warm Rearing\n(30°C)" = b_ecc_pretreatmentC
  ) %>%
  dplyr::select(
    Effects, `Body Mass`, `Tarsus Length`, `Bill Length`,
    `Cold Rearing\n(10°C)`, `Warm Rearing\n(30°C)`
  ) %>%
  rbind(
    .,
    scaledBetasEfficiency8 %>%
      mutate("Effects" = "Indirect Effects") %>%
      mutate(
        "Body Mass" = (b_tarsus_mass * b_ecc_tarsus) +
          (b_bill_mass * b_ecc_bill),
        "Tarsus Length" = NA,
        "Bill Length" = NA,
        "Cold Rearing\n(10°C)" =
          (b_mass_pretreatmentA * b_ecc_mass) +
            (b_tarsus_pretreatmentA * b_ecc_tarsus) +
            (b_bill_pretreatmentA * b_ecc_bill) +
            (b_mass_pretreatmentA * b_tarsus_mass * b_ecc_tarsus) +
            (b_mass_pretreatmentA * b_bill_mass * b_ecc_bill),
        "Warm Rearing\n(30°C)" =
          (b_mass_pretreatmentC * b_ecc_mass) +
            (b_tarsus_pretreatmentC * b_ecc_tarsus) +
            (b_bill_pretreatmentC * b_ecc_bill) +
            (b_mass_pretreatmentC * b_tarsus_mass * b_ecc_tarsus) +
            (b_mass_pretreatmentC * b_bill_mass * b_ecc_bill),
      ) %>%
      dplyr::select(
        Effects, `Body Mass`, `Tarsus Length`, `Bill Length`,
        `Cold Rearing\n(10°C)`, `Warm Rearing\n(30°C)`
      )
  ) %>%
  rbind(., scaledBetasEfficiency8 %>%
    mutate("Effects" = "Total Effects") %>%
    mutate(
      "Body Mass" =
        b_ecc_mass +
          b_tarsus_mass * b_ecc_tarsus,
      "Tarsus Length" =
```

```
        b_ecc_tarsus,
      "Bill Length" =
        b_ecc_bill,
      "Cold Rearing\n(10°C)" =
        (b_ecc_pretreatmentA) +
          (b_mass_pretreatmentA * b_ecc_mass) +
          (b_tarsus_pretreatmentA * b_ecc_tarsus) +
          (b_bill_pretreatmentA * b_ecc_bill) +
          (b_mass_pretreatmentA * b_tarsus_mass * b_ecc_tarsus) +
          (b_mass_pretreatmentA * b_bill_mass * b_ecc_bill),
      "Warm Rearing\n(30°C)" =
        (b_ecc_pretreatmentC) +
          (b_mass_pretreatmentC * b_ecc_mass) +
          (b_tarsus_pretreatmentC * b_ecc_tarsus) +
          (b_bill_pretreatmentC * b_ecc_bill) +
          (b_mass_pretreatmentC * b_tarsus_mass * b_ecc_tarsus) +
          (b_mass_pretreatmentC * b_bill_mass * b_ecc_bill),
    ) %>%
    dplyr::select(
      Effects, `Body Mass`, `Tarsus Length`, `Bill Length`,
      `Cold Rearing\n(10°C)`, `Warm Rearing\n(30°C)`
    )) %>%
  pivot_longer(c(-Effects), names_to = "var", values_to = "values") %>%
  mutate(var = factor(var,
    levels = c(
      "Body Mass",
      "Tarsus Length",
      "Bill Length",
      "Warm Rearing\n(30°C)",
      "Cold Rearing\n(10°C)"
    )
  ))

# Effects are plotted

fullEffectHeatLoss8Plot <- fullEffectHeatLoss8 %>%
  ggplot(aes(x = values, y = var, fill = var)) +
  facet_wrap(~Effects) +
  stat_halfeye(normalize = "xy", colour = "black", alpha = 0.7) +
  geom_vline(
    xintercept = 0, linetype = "dashed",
    colour = "black"
  ) +
  xlab("Effect on Fold Evaporative\nHeat Loss (standard deviations)") +
  scale_fill_manual(values = c("black", "grey25", "grey75", "#CD5C5C", "#7BB4E3")) +
  theme_classic() +
  theme(
    legend.position = "none", axis.title.y = element_blank(),
    axis.text.y = element_text(
      size = 11, colour = "black",
      family = "Noto Sans"
    ),
    axis.title.x = element_text(family = "Noto Sans", hjust = -0.005),
    axis.text.x = element_text(family = "Noto Sans")
  )

fullEffectEfficiency8Plot <- fullEffectEfficiency8 %>%
  ggplot(aes(x = values, y = var, fill = var)) +
  facet_wrap(~Effects) +
  stat_halfeye(normalize = "xy", colour = "black", alpha = 0.7) +
  geom_vline(
    xintercept = 0, linetype = "dashed",
    colour = "black"
  ) +
  xlab("Effect on Evaporative Cooling\nEfficiency (standard deviations)") +
  scale_fill_manual(values = c("black", "grey25", "grey75", "#CD5C5C", "#7BB4E3")) +
  theme_classic() +
```

```
  theme(
    legend.position = "none", axis.title.y = element_blank(),
    axis.text.y = element_text(
      size = 11, colour = "black",
      family = "Noto Sans"
    ),
    axis.title.x = element_text(family = "Noto Sans", hjust = -0.005),
    axis.text.x = element_text(family = "Noto Sans")
  )

(fullEffectHeatLoss8Plot /
  fullEffectEfficiency8Plot) +
  plot_annotation(tag_level = "A")
```

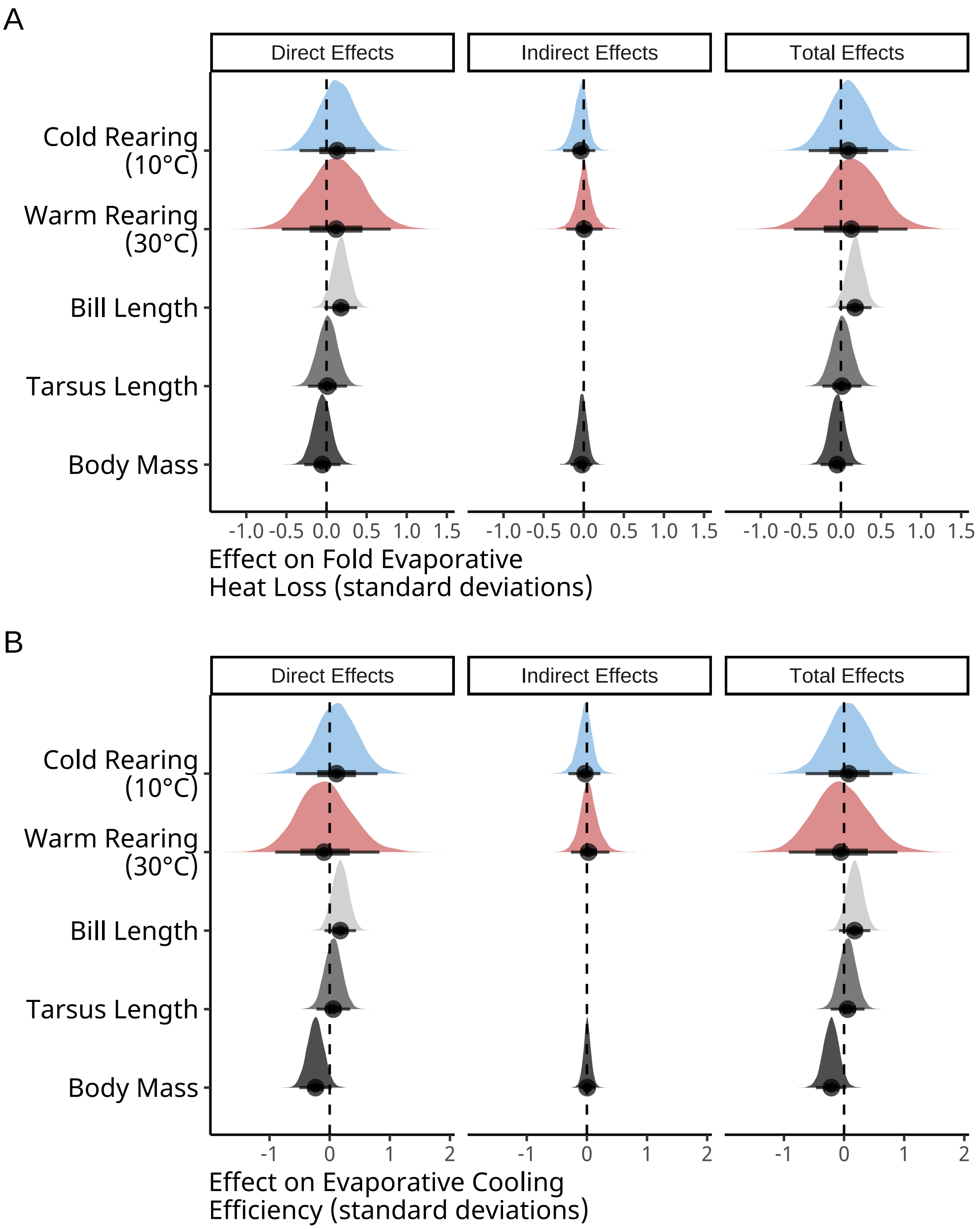

Figure 168: *Direct and indirect effects of rearing temperature and morphology (here, body mass and tarsus length) on evaporative heat loss responses (panel A) and evaporative cooling efficiency (panel B) in developing Japanese quail (eight weeks of age; n = 57). Evaporative heat loss responses represent fold increases in evaporative heat loss, in watts, between 30°C and 40°C. Evaporative cooling efficiency represents the ratio between evaporative heat loss (in watts) and metabolic heat production (in watts). Effects indicate how much, in standard deviations, a response variables would be altered by changing a predictor variable by one standard deviation. Densities are derived from posteriors of Bayesian path analyses. Dashed lines indicate 0.*

```
# Effects are summarised below
```

```
caption <- paste0("Direct, indirect, and total effects of morphology and ",
                  "rearing temperature on fold evaporative heat loss at 40°C",
                  "(relative to 30°C) of eight week old Japanese quail. ",
                  "Effects are derived from ",
                  "a Bayesian path analysis and represent those predicted for a ",
                  "change in one standard deviation (or categorical level) of ",
                  "a given predictor on the standard deviation of metabolic ",
                  "slopes. Estimates indicate posterior medians and credible ",
                  "intervels (CIs) indicate quantile intervals."
)

heatLossResultsScaled8 <-
  fullEffectHeatLoss8 %>%
  filter(!is.na(values) & !is.nan(values)) %>%
  group_by(Effects, var) %>%
  summarise(
    "Estimate" = median(values),
    "50\\% CIs" = paste0(
      "[",
      round(
        quantile(values, probs = 0.1, type = 8),
        digits = 4
      ),
      ", ",
      round(
        quantile(values, probs = 0.9, type = 8),
        digits = 4
      ),
      "]"
    ),
    "95\\% CIs" = paste0(
      "[",
      round(
        quantile(values, probs = 0.025, type = 8),
        digits = 4
      ),
      ", ",
      round(
        quantile(values, probs = 0.975, type = 8),
        digits = 4
      ),
      "]"
    )
  ) %>%
  mutate("Effects" = gsub("[[:space:]].*", "", Effects)) %>%
  dplyr::select(
    "Predictor" = "var", "Effect Level" = "Effects",
    Estimate, `50\\% CIs`, `95\\% CIs`
  ) %>%
  arrange(Predictor, `Effect Level`) %>%
  kbl(.,
    longtable = T, booktabs = T, format = "latex", escape = FALSE,
    caption = caption
  ) %>%
  column_spec(column = c(1:2), width = "2.2cm") %>%
  column_spec(column = c(3:10), width = "1.9cm") %>%
  kable_styling(latex_options = "striped")

heatLossResultsScaled8
```

Table 106: *Direct, indirect, and total effects of morphology and rearing temperature on fold evaporative heat loss at 40°C(relative to 30°C) of eight week old Japanese quail. Effects are derived from a Bayesian path analysis and represent those predicted for a change in one standard deviation (or categorical level) of a given predictor on the standard deviation of metabolic slopes. Estimates indicate posterior medians and credible intervels (CIs) indicate quantile intervals.*

Predictor	Effect Level	Estimate	50% CIs	95% CIs
Body Mass	Direct	-0.0526496	[-0.1985, 0.0886]	[-0.2773, 0.1736]
Body Mass	Indirect	-0.0222728	[-0.1108, 0.0593]	[-0.1638, 0.1104]
Body Mass	Total	-0.0464246	[-0.1753, 0.0804]	[-0.2521, 0.1489]
Tarsus Length	Direct	0.0123829	[-0.1484, 0.1705]	[-0.2321, 0.2556]
Tarsus Length	Total	0.0123829	[-0.1484, 0.1705]	[-0.2321, 0.2556]
Bill Length	Direct	0.1779220	[0.0449, 0.3116]	[-0.029, 0.3821]
Bill Length	Total	0.1779220	[0.0449, 0.3116]	[-0.029, 0.3821]
Warm Rearing (30°C)	Direct	0.1200107	[-0.3283, 0.5589]	[-0.5581, 0.8007]
Warm Rearing (30°C)	Indirect	0.0044564	[-0.1217, 0.1345]	[-0.2186, 0.235]
Warm Rearing (30°C)	Total	0.1295758	[-0.3341, 0.5701]	[-0.5887, 0.8297]
Cold Rearing (10°C)	Direct	0.1330169	[-0.1712, 0.4446]	[-0.3371, 0.6015]
Cold Rearing (10°C)	Indirect	-0.0353919	[-0.1676, 0.0684]	[-0.2565, 0.1432]
Cold Rearing (10°C)	Total	0.0916087	[-0.2328, 0.409]	[-0.4018, 0.5909]

```r
caption <- paste0(
  "Direct, indirect, and total effects of morphology and ",
  "rearing temperature on evaporative cooling efficiency ",
  "at 40°C  of eight week old Japanese quail. ",
  "Evaporative cooling efficiency represents the ratio of ",
  "evaporative heat loss (in W) to metabolic heat production ",
  "(again, in W). Effects are derived from ",
  "a Bayesian path analysis and represent those predicted for a ",
  "change in one standard deviation (or categorical level) of ",
  "a given predictor on the standard deviation of metabolic ",
  "slopes. Estimates indicate posterior medians and credible ",
  "intervels (CIs) indicate quantile intervals."
)

efficiencyResultsScaled8 <-
  fullEffectEfficiency8 %>%
  filter(!is.na(values) & !is.nan(values)) %>%
  group_by(Effects, var) %>%
  summarise(
    "Estimate" = median(values),
    "50\\% CIs" = paste0(
      "[",
      round(
        quantile(values, probs = 0.1, type = 8),
        digits = 4
      ),
      ", ",
      round(
        quantile(values, probs = 0.9, type = 8),
        digits = 4
```

```
      ),
      "]"
    ),
    "95\\% CIs" = paste0(
      "[",
      round(
        quantile(values, probs = 0.025, type = 8),
        digits = 4
      ),
      ", ",
      round(
        quantile(values, probs = 0.975, type = 8),
        digits = 4
      ),
      "]"
    )
  ) %>%
  mutate("Effects" = gsub("[[:space:]].*", "", Effects)) %>%
  dplyr::select(
    "Predictor" = "var", "Effect Level" = "Effects",
    Estimate, `50\\% CIs`, `95\\% CIs`
  ) %>%
  arrange(Predictor, `Effect Level`) %>%
  kbl(.,
    longtable = T, booktabs = T, format = "latex", escape = FALSE,
    caption = caption
  ) %>%
  column_spec(column = c(1:2), width = "2.2cm") %>%
  column_spec(column = c(3:10), width = "1.9cm") %>%
  kable_styling(latex_options = "striped")

efficiencyResultsScaled8
```

Table 107: *Direct, indirect, and total effects of morphology and rearing temperature on evaporative cooling efficiency at 40°C of eight week old Japanese quail. Evaporative cooling efficiency represents the ratio of evaporative heat loss (in W) to metabolic heat production (again, in W). Effects are derived from a Bayesian path analysis and represent those predicted for a change in one standard deviation (or categorical level) of a given predictor on the standard deviation of metabolic slopes. Estimates indicate posterior medians and credible intervels (CIs) indicate quantile intervals.*

Predictor	Effect Level	Estimate	50% CIs	95% CIs
Body Mass	Direct	-0.2360128	[-0.4083, -0.0617]	[-0.5035, 0.0321]
Body Mass	Indirect	0.0057005	[-0.0744, 0.087]	[-0.1245, 0.1379]
Body Mass	Total	-0.2121878	[-0.3768, -0.0507]	[-0.4693, 0.0385]
Tarsus Length	Direct	0.0595915	[-0.1232, 0.2388]	[-0.2213, 0.3409]
Tarsus Length	Total	0.0595915	[-0.1232, 0.2388]	[-0.2213, 0.3409]
Bill Length	Direct	0.1758352	[0.0039, 0.3471]	[-0.0854, 0.4369]
Bill Length	Total	0.1758352	[0.0039, 0.3471]	[-0.0854, 0.4369]
Warm Rearing (30°C)	Direct	-0.0946723	[-0.6179, 0.4908]	[-0.9066, 0.8277]
Warm Rearing (30°C)	Indirect	0.0266270	[-0.1477, 0.2315]	[-0.2634, 0.3741]
Warm Rearing (30°C)	Total	-0.0554991	[-0.6234, 0.5429]	[-0.9184, 0.8882]
Cold Rearing (10°C)	Direct	0.1147443	[-0.3219, 0.5487]	[-0.561, 0.7981]

Cold Rearing (10°C)	Indirect	-0.0307586	[-0.1958, 0.1194]	[-0.3083, 0.2246]
Cold Rearing (10°C)	Total	0.0770885	[-0.3808, 0.5408]	[-0.6387, 0.8074]

Again, we estimate the consequences of misalignment with Allen's and Bergmann's rule on evaporative heat loss responses and evaporative cooling efficiency at 40°C. Misalignment here represents have a relative tarsus length two standard deviations below average (Allen's Rule) or a body mass two standard deviations above average (Bergmann's rule).

```
data.frame(
  "Size" = c("Average", "Large (2x s.d. > mean)"),
  "pretreatment" = "B",
  "mass" = c(
    mean(ehlModel8Weeks$data$mass, na.rm = T),
    mean(ehlModel8Weeks$data$mass, na.rm = T) +
      2 * sd(ehlModel8Weeks$data$mass, na.rm = T)
  ),
  "tarsus" = 0,
  "bill" = 0,
  "batch" = "A"
) %>%
  mutate(
    "ehl" = predict(ehlModel8Weeks,
      newdata = .,
      resp = "foldEhl",
      re_form = NA,
      robust = TRUE
    )[, "Estimate"],
    "se1" = predict(ehlModel8Weeks,
      newdata = .,
      resp = "foldEhl",
      re_form = NA,
      robust = TRUE
    )[, "Est.Error"],
    "ecc" = predict(efficiencyModel8Weeks,
      newdata = .,
      resp = "ecc",
      re_form = NA,
      robust = TRUE
    )[, "Estimate"],
    "se2" = predict(efficiencyModel8Weeks,
      newdata = .,
      resp = "ecc",
      re_form = NA,
      robust = TRUE
    )[, "Est.Error"]
  ) %>%
  mutate_if(is.numeric, round, digits = 4) %>%
  mutate(
    "Body Size" = Size,
    "Evaporative Heat Loss" =
      paste0(ehl, " [", se1, "]"),
    "Evaporative Cooling Efficiency" =
      paste0(ecc, " [", se2, "]")
  ) %>%
  dplyr::select(
    `Body Size`, `Evaporative Heat Loss`, `Evaporative Cooling Efficiency`
  ) %>%
  kbl(.,
    longtable = T, booktabs = T, format = "latex",
    caption = paste0("Comparison of estimated evaporative heat loss rate ",
                     "and evaporative cooling efficiency among eight week old ",
                     "Japanese quail at 40°C and varying body masses."),
    escape = FALSE
```

```
  ) %>%
  column_spec(column = c(1:10), width = "2.5cm")  %>%
  kable_styling(latex_options = "striped")
```

Table 108: *Comparison of estimated evaporative heat loss rate and evaporative cooling efficiency among eight week old Japanese quail at 40°C and varying body masses.*

Body Size	Evaporative Heat Loss	Evaporative Cooling Efficiency
Average	2.4458 [0.7321]	0.6743 [0.1888]
Large (2x s.d. > mean)	2.3615 [0.7648]	0.5898 [0.1994]

```
data.frame(
  "Size" = c("Average", "Short (2x s.d. < mean)"),
  "pretreatment" = "B",
  "tarsus" = c(
    mean(ehlModel8Weeks$data$tarsus, na.rm = T),
    mean(ehlModel8Weeks$data$tarsus, na.rm = T) -
      2 * sd(ehlModel8Weeks$data$tarsus, na.rm = T)
  ),
  "mass" = 0,
  "bill" = 0,
  "batch" = "A"
) %>%
  mutate(
    "ehl" = predict(ehlModel8Weeks,
      newdata = .,
      resp = "foldEhl",
      re_form = NA,
      robust = TRUE
    )[, "Estimate"],
    "se1" = predict(ehlModel8Weeks,
      newdata = .,
      resp = "foldEhl",
      re_form = NA,
      robust = TRUE
    )[, "Est.Error"],
    "ecc" = predict(efficiencyModel8Weeks,
      newdata = .,
      resp = "ecc",
      re_form = NA,
      robust = TRUE
    )[, "Estimate"],
    "se2" = predict(efficiencyModel8Weeks,
      newdata = .,
      resp = "ecc",
      re_form = NA,
      robust = TRUE
    )[, "Est.Error"]
  ) %>%
  mutate_if(is.numeric, round, digits = 4) %>%
  mutate(
    "Tarsus Length" = Size,
    "Evaporative Heat Loss" =
      paste0(ehl, " [", se1, "]"),
    "Evaporative Cooling Efficiency" =
      paste0(ecc, " [", se2, "]")
  ) %>%
  dplyr::select(`Tarsus Length`, `Evaporative Heat Loss`,
         `Evaporative Cooling Efficiency`) %>%
  kbl(.,
    longtable = T, booktabs = T, format = "latex",
    caption = paste0("Comparison of estimated evaporative heat loss rate ",
                     "and evaporative cooling efficiency among eight week old ",
```

```
                     "Japanese quail at 40°C and varying tarsus lengths."),
    escape = FALSE
  ) %>%
  column_spec(column = c(1:10), width = "2.5cm")  %>%
  kable_styling(latex_options = "striped")
```

Table 109: *Comparison of estimated evaporative heat loss rate and evaporative cooling efficiency among eight week old Japanese quail at 40°C and varying tarsus lengths.*

Tarsus Length	Evaporative Heat Loss	Evaporative Cooling Efficiency
Average	2.4445 [0.7507]	0.6718 [0.1871]
Short (2x s.d. < mean)	2.4394 [0.7851]	0.649 [0.1922]

```
data.frame(
  "Size" = c("Average", "Short (2x s.d. < mean)"),
  "pretreatment" = "B",
  "bill" = c(
    mean(ehlModel8Weeks$data$bill, na.rm = T),
    mean(ehlModel8Weeks$data$bill, na.rm = T) -
      2 * sd(ehlModel8Weeks$data$bill, na.rm = T)
  ),
  "mass" = 0,
  "tarsus" = 0,
  "batch" = "A"
) %>%
  mutate(
    "ehl" = predict(ehlModel8Weeks,
      newdata = .,
      resp = "foldEhl",
      re_form = NA,
      robust = TRUE
    )[, "Estimate"],
    "se1" = predict(ehlModel8Weeks,
      newdata = .,
      resp = "foldEhl",
      re_form = NA,
      robust = TRUE
    )[, "Est.Error"],
    "ecc" = predict(efficiencyModel8Weeks,
      newdata = .,
      resp = "ecc",
      re_form = NA,
      robust = TRUE
    )[, "Estimate"],
    "se2" = predict(efficiencyModel8Weeks,
      newdata = .,
      resp = "ecc",
      re_form = NA,
      robust = TRUE
    )[, "Est.Error"]
  ) %>%
  mutate_if(is.numeric, round, digits = 4) %>%
  mutate(
    "Bill Length" = Size,
    "Evaporative Heat Loss" =
      paste0(ehl, " [", se1, "]"),
    "Evaporative Cooling Efficiency" =
      paste0(ecc, " [", se2, "]")
  ) %>%
  dplyr::select(`Bill Length`, `Evaporative Heat Loss`,
         `Evaporative Cooling Efficiency`) %>%
  kbl(.,
    longtable = T, booktabs = T, format = "latex",
```

```
  caption = paste0("Comparison of estimated evaporative heat loss rate ",
                   "and evaporative cooling efficiency among eight week old ",
                   "Japanese quail at 40°C and varying bill lengths."),
  escape = FALSE
) %>%
column_spec(column = c(1:10), width = "2.5cm")  %>%
kable_styling(latex_options = "striped")
```

Table 110: *Comparison of estimated evaporative heat loss rate and evaporative cooling efficiency among eight week old Japanese quail at 40°C and varying bill lengths.*

Bill Length	Evaporative Heat Loss	Evaporative Cooling Efficiency
Average	2.4516 [0.7187]	0.676 [0.1858]
Short (2x s.d. < mean)	2.1503 [0.754]	0.6132 [0.1936]

5.0 Estimating lower critical temperature

Overview

Previous studies have argued that alignment with Allen's and Bergmann's rules provide thermal advantages in the cold by decreasing an individual's lower critical temperature (e.g. Kendeigh, 1969). In this document, we test whether: (1) lower critical temperature evidently varies among adult quail from our sample population, and (2) whether lower critical temperature does indeed co-vary with adult body mass and/or limb length (here, tarsus length; mm), as might be predicted by Allen's and Bergmann's rules.

All analyses conducted here for first completed using R Statistical Software (2023; version 4.2.3) on a linux platform (kernel: 5.15.0-46-generic).

General methods

To estimate mean lower critical temperature in our quail, we first measured resting metabolism (mL O_2/min) in a subsample of adult birds (n = 40; age 20 weeks) at 5°C intervals ranging from 0°C to 35°C. Measurements were obtained by flow-through respirometry following methods described for adult quail in the main text of Tabh et al (2025). Once collected, resting metabolism values were then used to build Scholander–Irving curves per individual (here, via Bayesian piece-wise regression described below), from which lower critical temperatures (in °C) were extracted. Body mass of individuals was measured using a digital scale immediately before respirometry; tarsus length was measured digitally (described in Tabh et al 2025) at 12 weeks of age. To evaluate whether lower critical temperatures varied by morphology, we modelling lower critical temperatures (°C) per individual as a function of their body mass (g) and tarsus length (mm) using a simple, Bayesian, linear model (described in detail below below).

Data import, collation, and filtration

Below, we begin by loading in R-packages required for analysis, then importing and checking data for anomalies.

```
# First loading in packages

library("tidyverse")
library("easypackages")

packageList <- c("bayesplot", "brms", "brmsMethods",
                 "doParallel", "foreach", "ggpubr",
                 "kableExtra", "latex2exp", "patchwork",
                 "priorsense", "showtext", "tidybayes",
                 "wesanderson")

libraries(packageList)

caption <- paste0("R packages and their respective versions used for",
                  " data organisation and analysis in this study."
)

sapply(packageList, function(x) {
  y <- as.character(packageVersion(x))
  return(y)
}, simplify = FALSE) %>%
  enframe(., name = "Package", value = "Version") %>%
  as.data.frame(.) %>%
  kbl(.,
    longtable = T, booktabs = T,
    caption = caption
  ) %>%
  kable_styling(latex_options = "striped")
```

Table 111: *R packages and their respective versions used for data organisation and analysis in this study.*

Package	Version

bayesplot	1.11.1
brms	2.22.7
brmsMethods	0.0.0.9000
doParallel	1.0.17
foreach	1.5.2
ggpubr	0.6.0
kableExtra	1.4.0
latex2exp	0.9.6
patchwork	1.2.0
priorsense	1.0.2
showtext	0.9.7
tidybayes	3.0.6
wesanderson	0.3.7

```
# Loading additional functions

pp_check2 <- function(model, resp = NA, ndraws = 500,
                      xlab = "label", colour = "lightblue") {
  require(brms)
  require(ggplot2)
  stopifnot("Model must be a brmsfit object" = is.brmsfit(model))

  if (is.na(resp)) {
    resp <- model$formula$resp
  }

  p1 <- brms::pp_check(model, ndraws = ndraws, resp = resp) +
    scale_colour_manual(
      values = c("black", colour),
      labels = c("y", "yhat"),
      name = NULL
    ) +
    xlab(xlab) +
    ylab("Density") +
    theme_classic()
  return(p1)
}

chainCheck <- function(model, rDig = 3) {
  require(brms)
  stopifnot("Model must be a brmsfit object" = is.brmsfit(model))

  Rhat <- paste0(
    "Rhat range: ",
    round(min(rhat(model)), digits = rDig),
    " - ",
    round(max(rhat(model)), digits = rDig)
  )
  Neff <- paste0(
    "Neff/N range: ",
    round(min(neff_ratio(model)), digits = rDig),
    " - ",
    round(max(neff_ratio(model)), digits = rDig)
  )
  cat(paste0(Rhat, "\n", Neff))
}

quantileCIs <- function(x, rnd = 3, cis = c(50, 95), sci_note = FALSE) {
  require(tidyverse)

  if (class(x)[1] != "brmsfit") {
    return("x must be a brmsfit object.")
  }
  if (length(cis) != 2) {
    return("cis must be a vector of integers with length 2")
  }
```

```
  prbs = c()
  nColNames = c()
  for (i in 1:length(cis)){
    prbs = c(prbs, c(0.5 - (cis[i]/100)/2, 0.5 + (cis[i]/100)/2))
    nColNames = c(nColNames,
                  paste0("Low_CI_", cis[i]),
                  paste0("High_CI_", cis[i])
    )
  }

  modelFrame = as.data.frame(x)

  Results <- apply(modelFrame, MARGIN = 2, FUN = quantile,
        probs = prbs, type = 8) %>%
    t() %>%
    as.data.frame() %>%
    rownames_to_column(var = "par") %>%
    `colnames<-`(c("Parameter", nColNames))

  if (sci_note == FALSE) {
    Results <- apply(modelFrame, MARGIN = 2, FUN = quantile,
        probs = prbs, type = 8) %>%
    t() %>%
    as.data.frame() %>%
    rownames_to_column(var = "par") %>%
    `colnames<-`(c("Parameter", nColNames))

  } else if (sci_note == TRUE) {
    Results <- apply(modelFrame, MARGIN = 2, FUN = quantile,
        probs = prbs, type = 8) %>%
    t() %>%
    as.data.frame() %>%
    rownames_to_column(var = "par") %>%
    `colnames<-`(c("Parameter", nColNames)) %>%
    mutate_at(.vars = vars(-Parameter),
              .funs = function(x){
                return(format(x, scientific = TRUE))
              }
    )
  }

  return(Results)
}

# Installing font

font_add_google(name = "Noto Sans", family = "Noto Sans")

# Setting working directory

setwd("/Users/joshuatabh/analyses")

# Loading in and restructuring metabolism data for use

rmrData <- read.csv("lctMetabolismData.csv") %>%
  rename("fileName" = "FileName") %>%
  mutate(fileName =
           gsub("_.*", "",
             gsub("mod-", "", fileName)
             )
         ) %>%
  merge(., read.csv("lctRMRBirdIDs.csv") %>%
    select(
      "ring" = Ring, "sex" = Sex, Chamber,
      fileName = "respirometry_file"
    ),
  by = c("fileName", "Chamber"), all.x = TRUE
```

```
  )  %>%
  merge(., data.frame(
    "FileNumber" = seq(2, 9, by = 1),
    "Ta" = seq(35, 0, by = -5)
  ),
  by = "FileNumber", all.x = TRUE
  ) %>%
  select(ring, sex,
    "chamber" = Chamber,
    Ta, VO2,, fileName
  ) %>%
  filter(Ta <= 35)

# Producing dotplot to inspect data visually

rmrData %>%
  group_by(Ta) %>%
  mutate("Row" = 1:n()) %>%
  mutate(Ta = paste0(Ta, "°C")) %>%
  ungroup() %>%
  ggplot(
  aes(x = Row, y = VO2)) +
  facet_wrap(~Ta, scales = "free") +
  geom_point() +
  xlab("Sample Number") +
  ylab(bquote(Resting ~ Metabolism ~ (mL ~ O[2] / min))) +
  theme_classic() +
  theme(legend.position = "none")
```

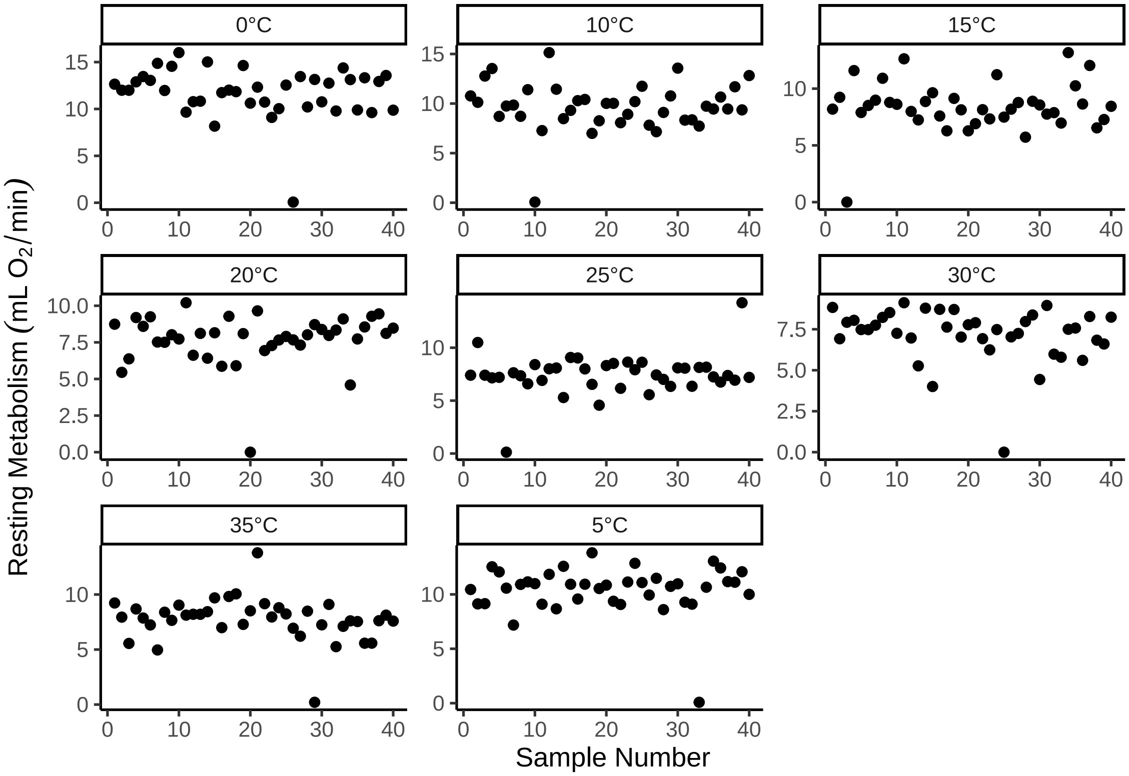

Figure 169: *Cleveland dotplot of resting metabolism values by ambient temperature of collection, each drawn from adult Japanese quail. Dots represent raw data points.*

```
# Several points indicating an unrealistic resting metabolism of 0.
# Filtering these points out and proceeding.
```

```
rmrData = rmrData %>%
  filter(VO2 > 3)
```

Next, all resting metabolism values are plotted by ambient temperature.

```
ggplot(rmrData,
  aes(x = Ta, y = VO2)) +
  geom_point(pch = 21, colour = "black", fill = "black",
             alpha = 0.5) +
  stat_summary(
    fun.data = "mean_se", geom = "errorbar",
    colour = "black", width = 1
  ) +
  stat_summary(
    fun.y = "mean", geom = "point", size = 4,
    pch = 21, colour = "black", fill = "grey50", alpha = 0.7) +
  xlab("Ambient Temperature (°C)") +
  ylab(bquote(Resting ~ Metabolism ~ (mL ~ O[2] / min))) +
  theme_classic()
```

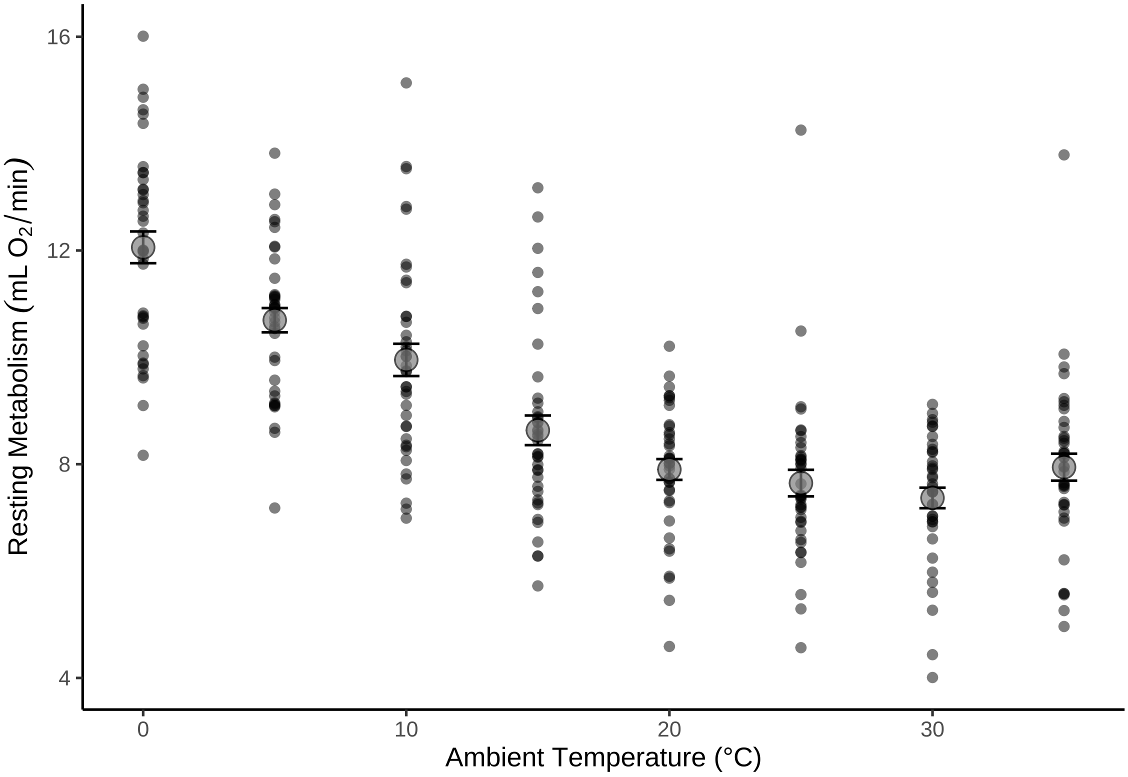

Figure 170: *Resting metabolism values (mL O_2/min) by ambient temperature (°C) of collection drawn from adult Japanese quail. Large dots represent averages at given ambient temperatures, represent standard errors around these averages, and small dots represent raw values.*

Evidently, 35°C lies above thermoneutrality in many of our sampled quail. Since we are only interested in defining lower critical temperatures among our quail, measurements at 35°C are excluded from analysis.

```
rmrData <- rmrData %>%
  filter(Ta < 35)

# Relativising ambient temperature measures with respect to known
```

```
# thermoneutrality

rmrData$relativeTa = abs(rmrData$Ta - 30)
```

Model construction

Next, we construct a Bayesian piece-wise regression (discussed above) with resting metabolism as the Gaussian-distributed response variable and ambient temperature (continuous) as the sole population-level predictor. Since we only considered data obtained within or below thermoneutrality for our quail (confirmed above), we assumed that resting metabolism was constant at ambient temperatures above the lower critical temperature (here defined as a break-point) and decreased at a constant linear rate (defined as "thermal resistance") below such. To evaluate the degree to which lower critical temperatures might differ by individual, our break-point, thermal resistance, and metabolism intercepts were allowed to vary by individual around a population mean (i.e. by inclusion of population; and group-level intercepts for each parameter). Further, to simplify model interpretation, we defined 30°C, an ambient temperature known to fall within thermoneutrality for quail (Ben-Hamo et al, 2010), as our regression intercept and relativised remaining temperature values as absolute deviations from 30°C (e.g. 30°C = 0°C, 0°C = 30°C). By doing so, we defined metabolism at thermoneutrality as our model "reference point".

Priors for our model break-point and and resting metabolism at thermoneutrality (model intercept) were skew-normal with ξ, ω, and α values of 0.65 and 4.5, 2.5 and 2.5, and 2.5 and 4 respectively (setting mean lower critical temperatures at approximately 27.5°C and 6 mL O_2/min; see Atchley et al, 2008; Ben-Hamo et al, 2010; Persson et al, 2024), while that for thermal resistance was also skew-normal ($\xi = 0.07$, $\omega = 0.1$, $\alpha = 9$; informed by Saarela and Heldmaier, 1978; Persson et al, 2024). Priors for individual variation around break-points, resting metabolism at thermoneutrality, and thermal resistance were exponential with lambda (λ) values of 0.25, 0.5, and 10 respectively.

Below, we check the suitability of our piece-wise regression priors. This is achieved by estimating possible resting metabolism values from our priors alone, then comparing these values against true resting metabolism measurements.

```
# First constructing model formula

bform <- bf(
 VO2 ~ Intercept +
   (resistance * (relativeTa - breakPoint)) *
   stepFunction(relativeTa - breakPoint),
 Intercept + resistance + breakPoint ~ 1 + (1|ring),
 nl = TRUE
)

stan_funs <- "
  real stepFunction(real x) {
    return step(x);
  }
"

stanvars <- stanvar(scode = stan_funs, block = "functions")

# Constructing model priors

breakPriors <- c(
  set_prior("skew_normal(4.5, 2, 4)", nlpar = "Intercept"),
  set_prior("skew_normal(0.65, 2.5, 2.5)", nlpar = "breakPoint"),
  set_prior("skew_normal(0.07, 0.01, 9)", nlpar = "resistance"),
  set_prior("exponential(0.5)", nlpar = "Intercept", class = "sd"),
  set_prior("exponential(0.25)", nlpar = "breakPoint", class = "sd"),
  set_prior("exponential(5)", nlpar = "resistance", class = "sd")
)

# Fitting model but only sampling from priors
```

```
priorCheck <- brm(bform, data = rmrData,
            prior = breakPriors,
            iter = 50000, warmup = 10000, thin = 10,
            cores = 4, chains = 4,
            silent = TRUE, refresh = 0,
            stanvars = stanvars,
            sample_prior = "only",
            file = "./models/lctAnalysisPPCheck.Rds"
            )

expose_functions(priorCheck, vectorize = TRUE)
```

```
pp_check2(priorCheck,
  xlab = TeX('$Resting~Metabolism~(mL~O_{2}/min)$')) +
  xlim(c(0, 50))
```

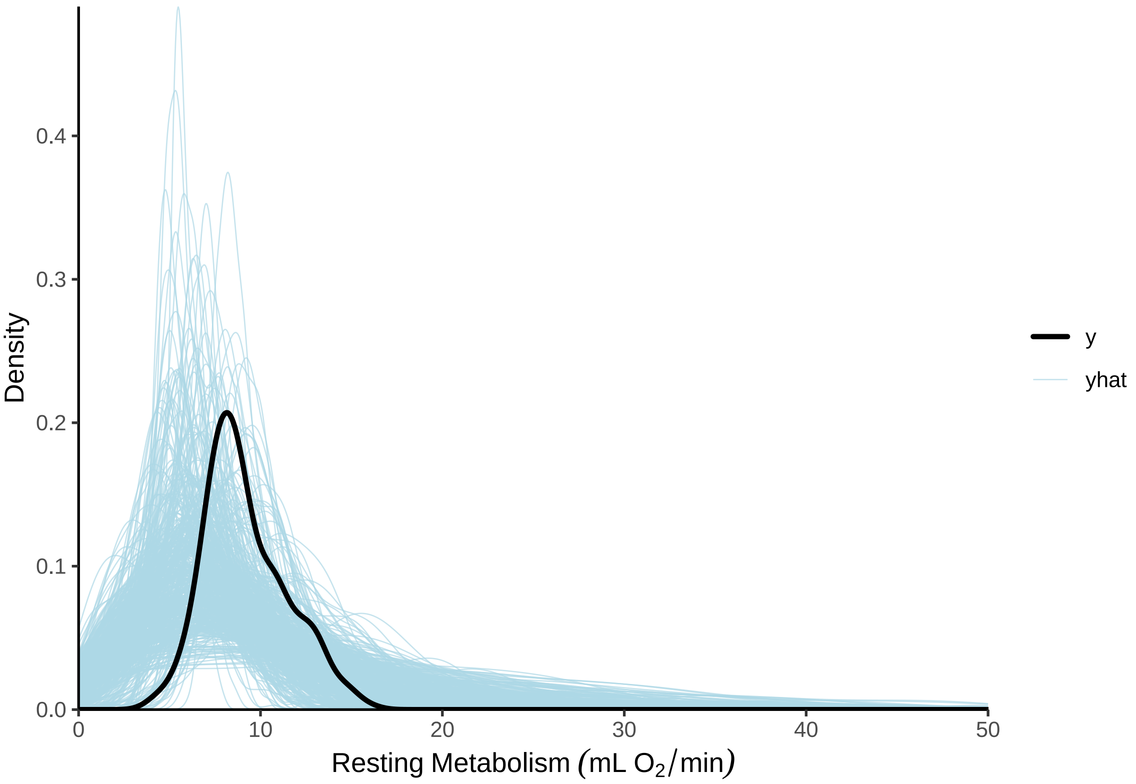

Figure 171: *Prior predictive check for a Bayesian piece-wise regression predicting resting metabolism (mL O_2/min) of adult Japanese quail by ambient temperature (raw ambient temperatures ranging from 0°C - 30°C). Resting metabolism is assumed to remain constant at ambient temperatures above an estimated breakpoint (the lower critical temperature) and increase linearly below that ambient temperature. Light blue lines represent densities of resting metabolism values as predicted by model priors alone. The dark blue line represents the true density of resting metabolism values. Clear overlap between the dark blue and light blue lines indicates that priors capture true data distributions well.*

Given the clear overlap between the density of true resting metabolism values and those derived from model priors, we proceed with executing our full model as parameterised above.

```
model <- brm(bform, data = rmrData,
            prior = breakPriors,
            iter = 100000, warmup = 10000, thin = 10,
            cores = 4, chains = 4,
```

```r
            silent = TRUE, refresh = 0,
            stanvars = stanvars,
            control = list(adapt_delta = .97, max_treedepth = 14),
            file = "./models/lctAnalysis.Rds"
            )

expose_functions(model, vectorize = TRUE)
```

```r
# Checking Gelman-Rubin statistics (Rhat values) and ratio of effective
# sample sizes to sample sizes.

chainCheck(model)
```

```
## Rhat range: 1 - 1
## Neff/N range: 0.93 - 1.007
```

```r
# Rhat values all close to 1 indicating clear chain mixing.
# Also, ratio of effective sample sizes to sample sizes all near 1,
# suggesting little autocorrelation within sample draws.

# Viewing posterior densities and fitted trends

pp_check2(model,
  xlab = TeX('$Resting~Metabolism~(mL~O_{2}/min)$')) +
  xlim(c(0, 50))
```

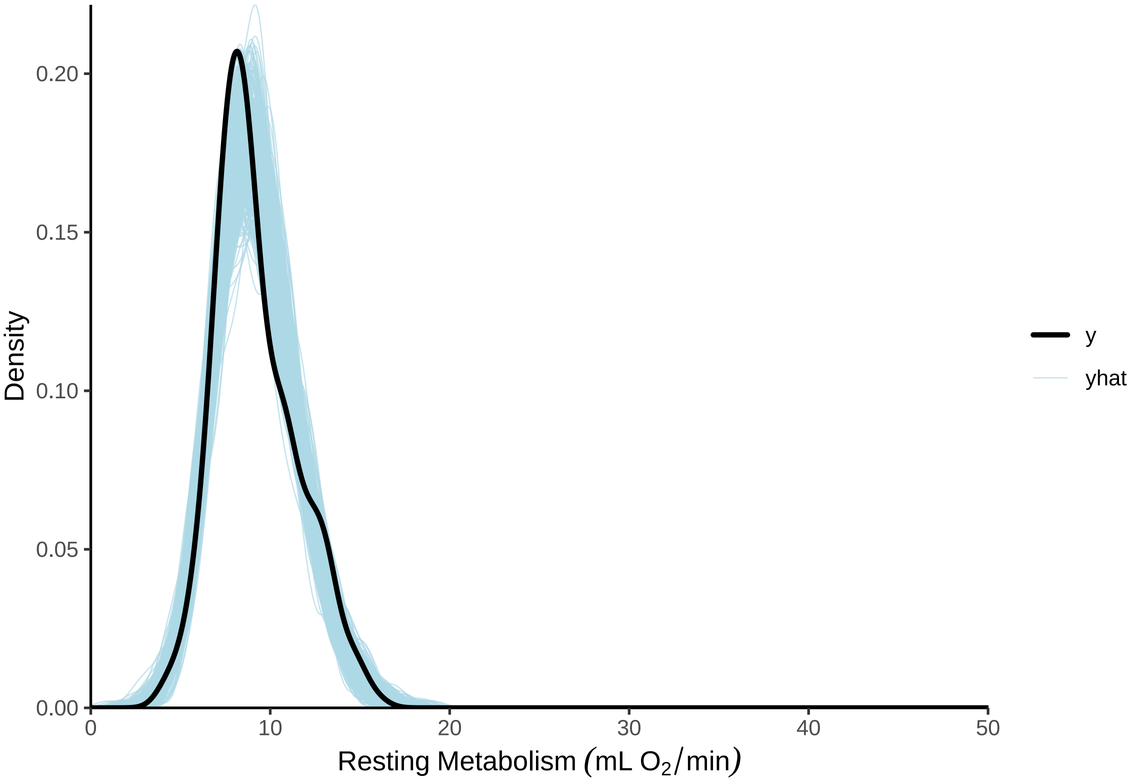

Figure 172: *Posterior predictive check for a Bayesian piece-wise regression predicting resting metabolism (mL O_2/min) of adult Japanese quail by ambient temperature (raw ambient temperatures ranging from 0°C - 30°C). Light blue lines represent densities of resting metabolism values as predicted by model posteriors. The dark blue line represents the true density of resting metabolism values.*

Below, we visualise the predicted outcomes of our model at the individual level.

```
model$data %>%
  mutate("fit" = fitted(model, robust = TRUE)[, "Estimate"]) %>%
  mutate(Ta = 30 - relativeTa) %>%
  ggplot(aes(x = Ta, y = fit, colour = ring)) +
  geom_point(data = rmrData, aes(x = Ta, y = VO2)) +
  geom_line() +
  facet_wrap(~ring) +
  scale_color_grey() +
  xlab("Ambient Temperature (°C)") +
  ylab(TeX('$Resting~Metabolism~(mL~O_{2}/min)$')) +
  theme_classic() +
  theme(legend.position = "none")
```

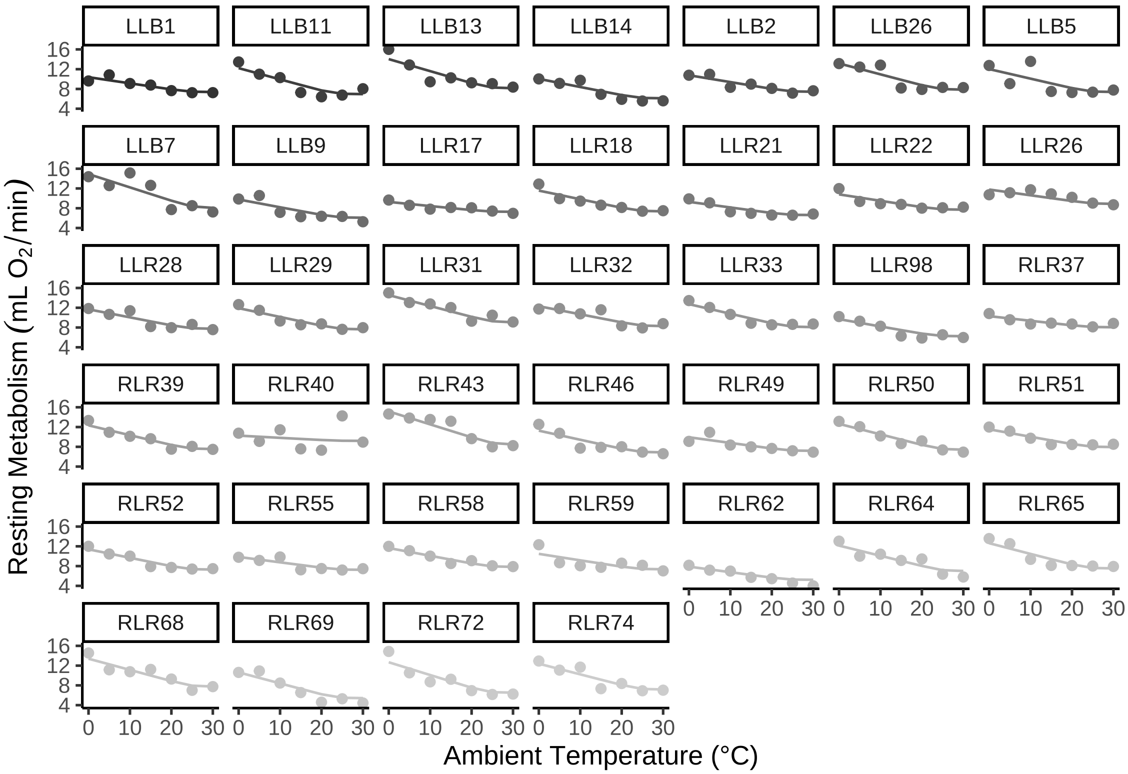

Figure 173: *Predictions from a Bayesian piece-wise regression correlating resting metabolism (mL O_2/min) with ambient temperature in adult Japanese quail. Dots represent raw values per individual and lines represent lines of best fit derived from regression. All dots and lines within a single panel represent those relevant to a single individual.*

Next, we check model residuals for abnormalities.

```
p1 = model$data %>%
  mutate("Residuals" = residuals(model, robust = TRUE)[,"Estimate"]) %>%
  ggplot(aes(x = Residuals)) +
  geom_density() +
  xlab("Ordinary Residuals") +
  ylab("Density") +
  theme_classic()

p2 = model$data %>%
  mutate("Residuals" = residuals(model, robust = TRUE)[,"Estimate"]) %>%
  mutate("Ta" = 30 - relativeTa) %>%
  ggplot(aes(x = Ta, y = Residuals)) +
  geom_point() +
```

```
  geom_smooth(colour = "black", linetype = "dashed", se = FALSE) +
  xlab("Ambient Temperature (°C)") +
  ylab("Ordinary Residuals") +
  theme_classic()

p3 = model$data %>%
  mutate("Residuals" = residuals(model, robust = TRUE)[,"Estimate"],
         "Fitted" = fitted(model, robust = TRUE)[,"Estimate"]) %>%
  ggplot(aes(x = Fitted, y = Residuals)) +
  geom_point() +
  xlab(TeX('$Fitted~Values~(mL~O_{2}/min)$')) +
  ylab("Ordinary Residuals") +
  theme_classic()

(p1 + p2)/p3 + plot_annotation(tag_levels = "A")
```

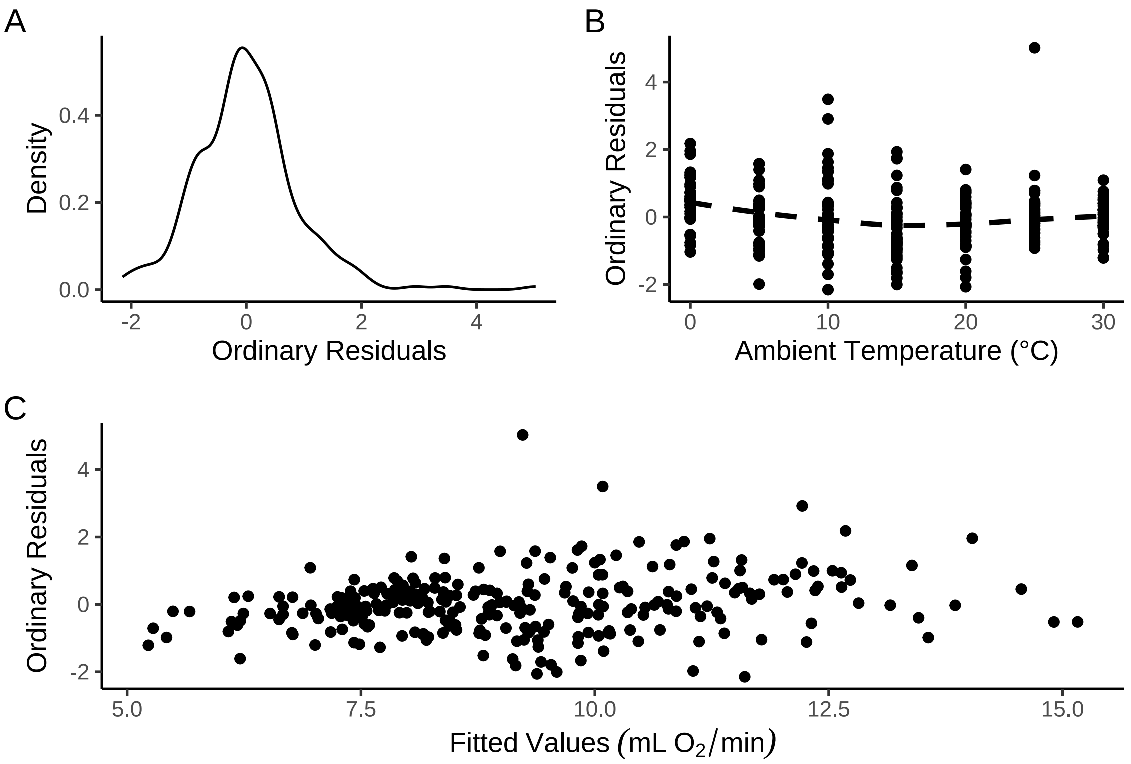

Figure 174: *Spread of ordinary residuals (here, using medians as measures of central tendancy in posteriors) from a Bayesian piece-wise regression predicting resting metabolism (mL O_2/min) of adult Japanese quail across ambient temperature (°C). Dots represent individual data points. In panel B, the dotted line represents a loess line of best fit as estimated by the R package ggplot2 (Wickham, 2011). Fitted values in panel C represent posterior medians.*

Residuals are largely homoskedastic but one extreme point is identified (absolute ordinary residual value exceeding 4). This value is therefore identified and scrutinised.

```
caption <- paste0(
  "List of resting metabolism values obtained from one mature Japanese Quail",
  " with one measurement labelled as 'extreme' owing to its high residual value."
  )

model$data %>%
  mutate("Residuals" = residuals(model, robust = TRUE)[,"Estimate"]) %>%
  mutate("Extreme" = ifelse(Residuals > 4, "Y", "N"),
```

```
        "Ta" = 30 - relativeTa) %>%
  group_by(ring) %>%
  mutate("containsExtreme" = ifelse(length(grep("Y", Extreme)) > 0, "Y", "N")) %>%
  ungroup() %>%
  filter(containsExtreme == "Y") %>%
  dplyr::select("Ring" = ring, "Ambient Temperature (°C)" = Ta,
              Extreme, "Metabolism (mL O2/min)" = VO2) %>%
  kbl(.,
    longtable = T, booktabs = T, format = "latex",
    caption = caption, escape = FALSE,
  ) %>%
  kable_styling(latex_options = "striped")
```

Table 112: *List of resting metabolism values obtained from one mature Japanese Quail with one measurement labelled as 'extreme' owing to its high residual value.*

Ring	Ambient Temperature (°C)	Extreme	Metabolism (mL O2/min)
RLR40	30	N	8.950793
RLR40	25	Y	14.252910
RLR40	20	N	7.314975
RLR40	15	N	7.586870
RLR40	10	N	11.443220
RLR40	5	N	9.099599
RLR40	0	N	10.757160

```
hold <- model$data %>%
  mutate("Residuals" = residuals(model, robust = TRUE)[,"Estimate"]) %>%
  filter(Residuals > 4) %>%
  pull(VO2)

model$data %>%
  filter(relativeTa == 5) %>%
  mutate("Ta" = 30 - relativeTa) %>%
  ggplot(aes(x = VO2)) +
  geom_density(colour = "black", fill = "grey80", alpha = 0.5) +
  geom_vline(xintercept = hold, colour = "black", linetype = "dashed") +
  xlab(TeX('$Resting~Metabolism~(mL~O_{2}/min)$')) +
  ylab("Density") +
  theme_classic()
```

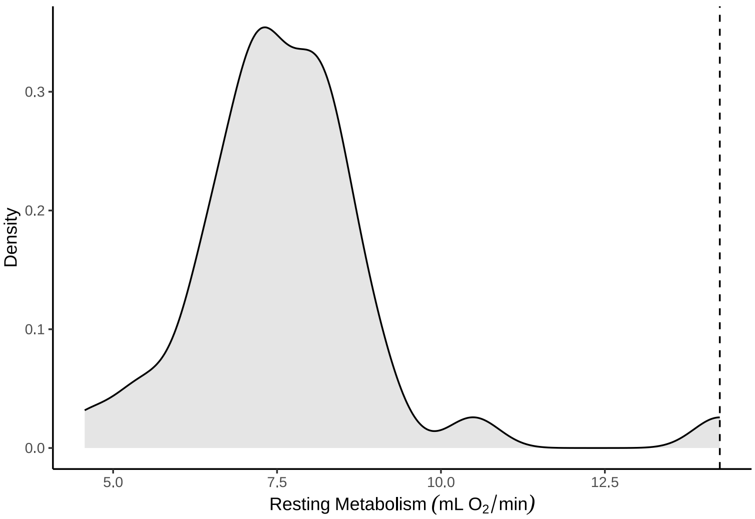

Figure 175: *Density of resting metabolism (mL O_2/min) of adult Japanese quail measured at 25°C. The dashed line indicates the resting metabolism value measured for an individual that appears extreme.*

Given the marked deviation of this resting metabolism value from others obtained at the equivalent ambient temperature, it is removed it from our dataset and re-execute our analyses.

```
rmrData <- rmrData %>%
  mutate(VO2 = ifelse(relativeTa == 5 & ring == "RLR40", NA, VO2))

model <- brm(bform, data = rmrData,
            prior = breakPriors,
            iter = 100000, warmup = 10000, thin = 10,
            cores = 4, chains = 4,
            silent = TRUE, refresh = 0,
            stanvars = stanvars,
            control = list(adapt_delta = .97, max_treedepth = 14),
            file = "./models/_lctAnalysis2.Rds"
            )

expose_functions(model, vectorize = TRUE)
chainCheck(model)

pp_check2(model,
  xlab = TeX('$Resting~Metabolism~(mL~O_{2}/min)$')) +
  xlim(c(0, 50))
```

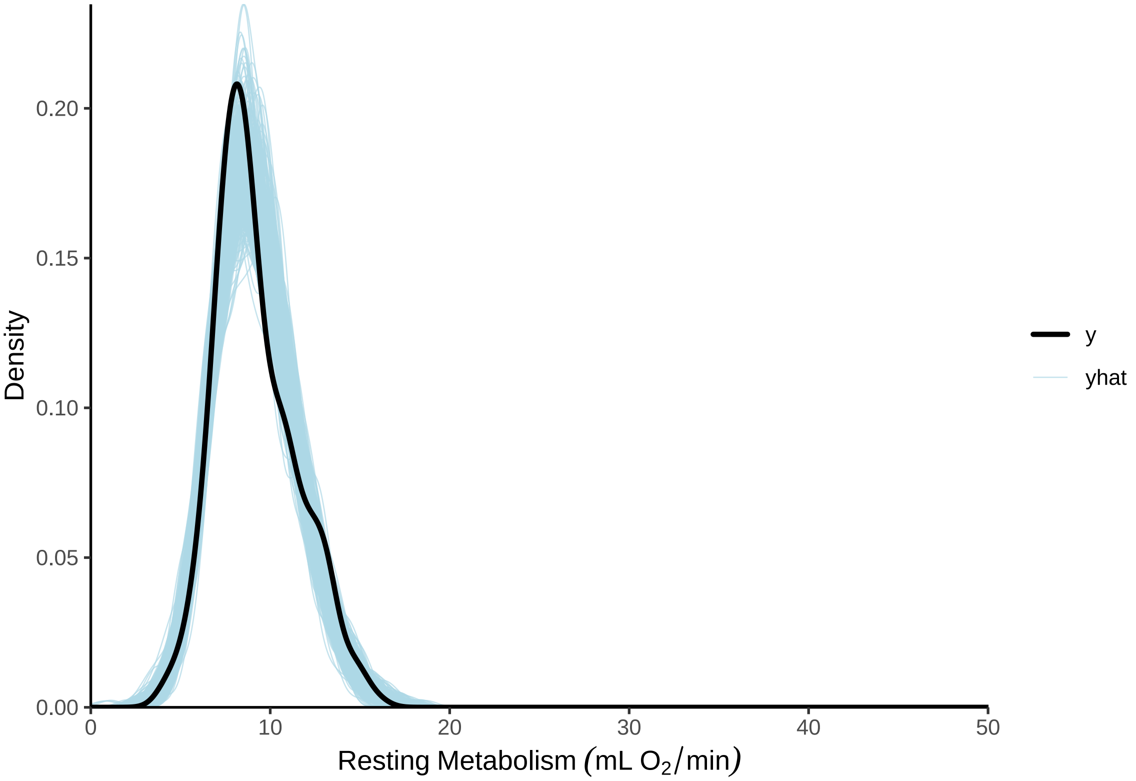

Figure 176: *Posterior predictive check for a revised Bayesian piece-wise regression predicting resting metabolism (mL O_2/min) of adult Japanese quail by ambient temperature (raw ambient temperatures ranging from 0°C - 30°C). One extreme value is excluded from this analysis. Light blue lines represent densities of resting metabolism values as predicted by model posteriors. The dark blue line represents the true density of resting metabolism values.*

Residuals of this updated model are visually appraised as above.

```
p1 = model$data %>%
  mutate("Residuals" = residuals(model, robust = TRUE)[,"Estimate"]) %>%
  ggplot(aes(x = Residuals)) +
  geom_density() +
  xlab("Ordinary Residuals") +
  ylab("Density") +
  theme_classic()

p2 = model$data %>%
  mutate("Residuals" = residuals(model, robust = TRUE)[,"Estimate"]) %>%
  mutate("Ta" = 30 - relativeTa) %>%
  ggplot(aes(x = Ta, y = Residuals)) +
  geom_point() +
  geom_smooth(colour = "black", linetype = "dashed", se = FALSE) +
  xlab("Ambient Temperature (°C)") +
  ylab("Ordinary Residuals") +
  theme_classic()

p3 = model$data %>%
  mutate("Residuals" = residuals(model, robust = TRUE)[,"Estimate"],
         "Fitted" = fitted(model, robust = TRUE)[,"Estimate"]) %>%
  ggplot(aes(x = Fitted, y = Residuals)) +
  geom_point() +
  xlab(TeX('$Fitted~Values~(mL~O_{2}/min)$')) +
  ylab("Ordinary Residuals") +
  theme_classic()

(p1 + p2)/p3 + plot_annotation(tag_levels = "A")
```

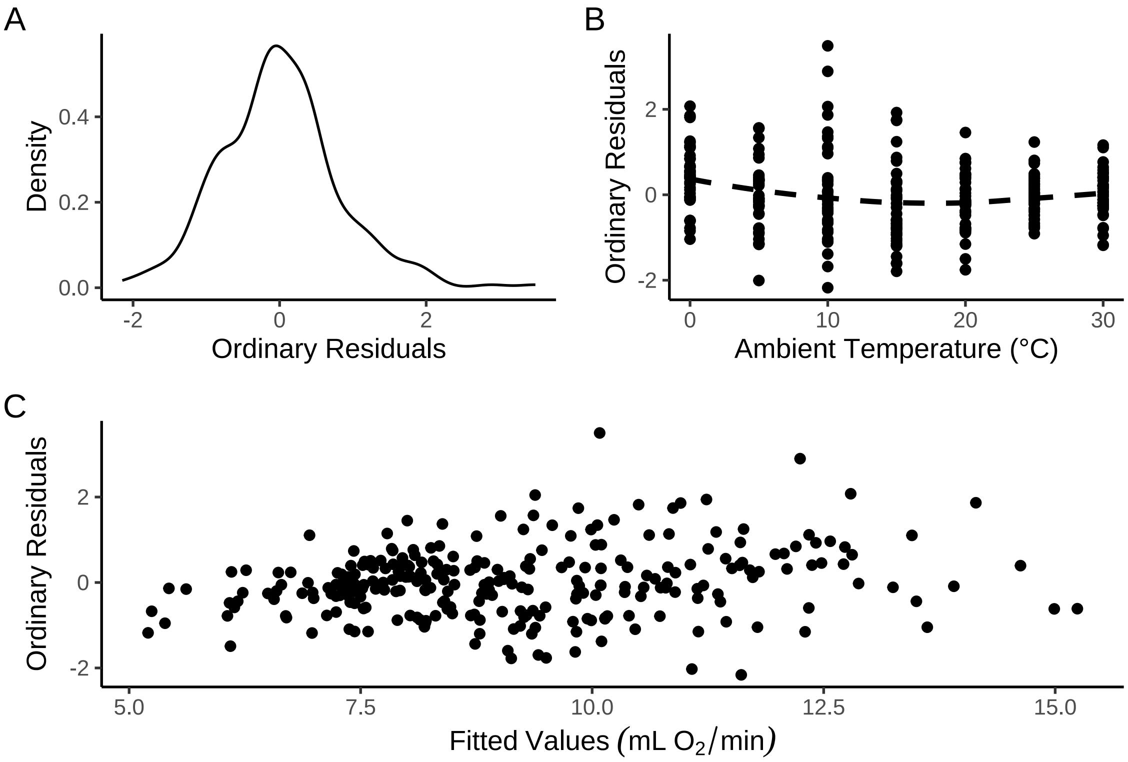

Figure 177: *Spread of ordinary residuals (here, using medians as measures of central tendancy in posteriors) from a revised Bayesian piece-wise regression predicting resting metabolism (mL O_2/min) of adult Japanese quail across ambient temperature (°C). One extreme value is excluded from this analysis. Dots represent individual data points. In panel B, the dotted line represents a loess line of best fit as estimated by the R package ggplot2 (Wickham, 2011). Fitted values in panel C represent posterior medians.*

Spread of residuals now appears more reasonable. Predicted trends across ambient temperature and by individual are repeated.

```
model$data %>%
  mutate("fit" = fitted(model, robust = TRUE)[, "Estimate"]) %>%
  mutate(Ta = 30 - relativeTa) %>%
  ggplot(aes(x = Ta, y = fit, colour = ring)) +
  geom_point(data = rmrData, aes(x = Ta, y = VO2)) +
  geom_line() +
  facet_wrap(~ring) +
  scale_color_grey() +
  xlab("Ambient Temperature (°C)") +
  ylab(TeX('$Resting~Metabolism~(mL~O_{2}/min)$')) +
  theme_classic() +
  theme(legend.position = "none")
```

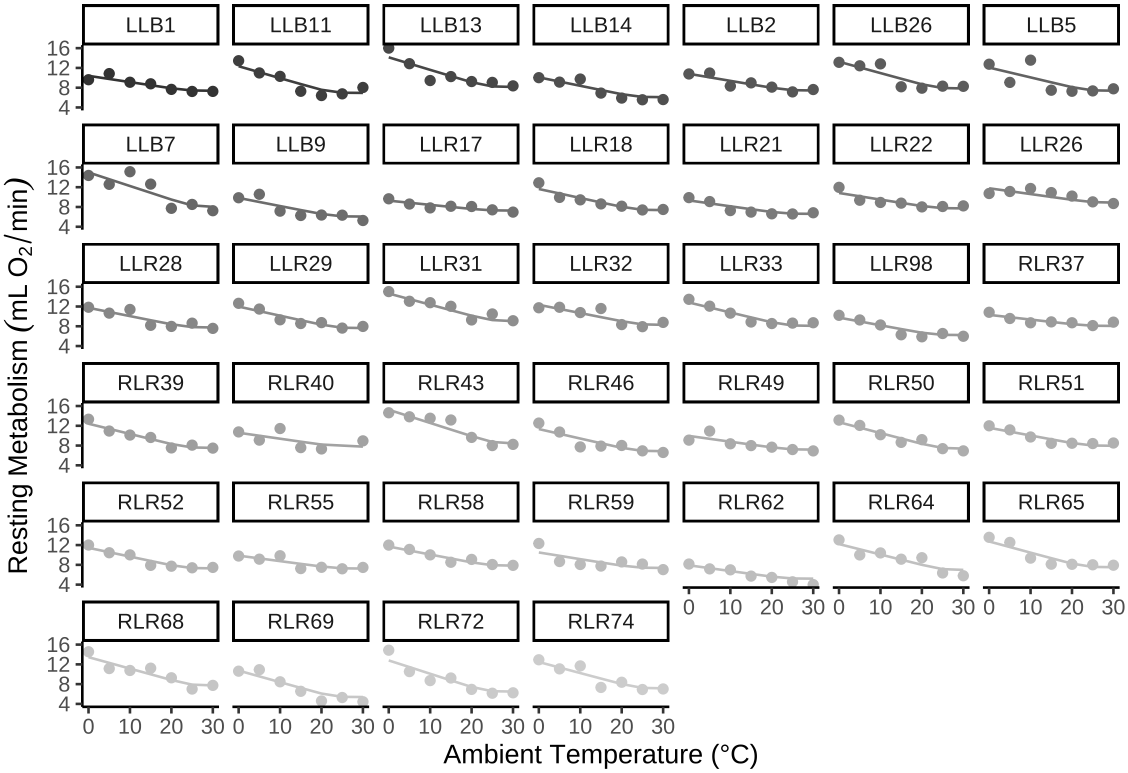

Figure 178: *Predictions from a revised Bayesian piece-wise regression correlating resting metabolism (mL O_2/min) with ambient temperature in adult Japanese quail. One extreme value is excluded from this analysis. Dots represent raw values per individual and lines represent lines of best fit derived from regression. All dots and lines within a single panel represent those relevant to a single individual.*

Next, densities of our model coefficients are below visualised.

```
as.data.frame(model) %>%
  pivot_longer(everything(), names_to = "par", values_to = "values") %>%
  filter(grepl("b_|sd_", par)) %>%
  mutate(values = ifelse(par == "b_breakPoint_Intercept",
                         30 - values, values),
         values = ifelse(par == "b_resistance_Intercept",
                         values*-1, values)) %>%
  merge(.,
        tribble(~par, ~Parameter,
```

```
                "b_Intercept_Intercept", "Metabolism at 30°C\n(mL O2/min)",
                "b_resistance_Intercept", "Thermal Resistance\n(mL O2/min/°C)",
                "b_breakPoint_Intercept", "Lower Critical Temperature\n(°C)",
                "sd_ring__Intercept_Intercept",
                "Group-level\nMetabolism Intercept",
                "sd_ring__resistance_Intercept",
                "Group-level\nResistance Intercept",
                "sd_ring__breakPoint_Intercept",
                "Group-level Lower\nCritical Temperature\nIntercept"),
        by = 'par', all.x = TRUE
  ) %>%
  ggplot(aes(x = values)) +
    facet_wrap(~Parameter, scales = "free", strip.position = "bottom") +
    geom_density() +
    ylab("Density") +
    theme_classic() +
    theme(axis.title.x = element_blank(),
          strip.background = element_blank(),
          strip.placement = "outside",
          strip.text.x = element_text(size = 10))
```

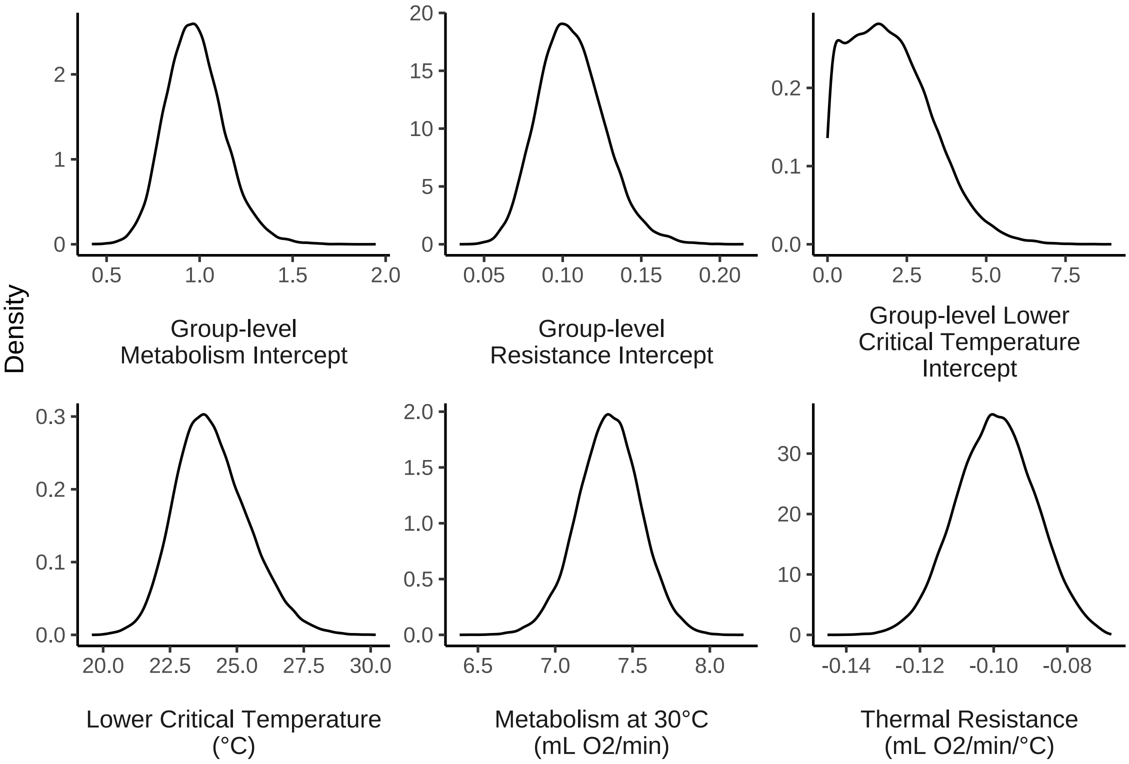

Figure 179: *Posterior densities of coefficients from a Bayesian piece-wise regression predicting resting metabolism (mL O_2/min) of adult Japanese quail across ambient temperature (°C).*

```
caption = paste0("Results of a Bayesian piece-wise regression ",
                 "predicting resting metabolism (mL O\\textsubscript{2}/min) ",
                 "of adult Japanese quail across ambient temperature ",
                 "(°C). Break-points (here, lower critical ",
                 "temperature), thermal resistance, and metabolism at ",
                 "thermoneutrality was allowed to vary by individual. ",
                 "Estimates represent posterior medians and credible ",
                 "intervals represent quantile intervals.")

as.data.frame(model) %>%
  mutate("b_breakPoint_Intercept" = b_breakPoint_Intercept,
```

```
        "b_resistance_Intercept" = b_resistance_Intercept*-1) %>%
  summarise_all(.funs = function(x) round(median(x), digits = 3)) %>%
  pivot_longer(everything(), names_to = "par", values_to = "median") %>%
  merge(.,
  apply(as.data.frame(model),
        FUN = quantile, probs = c(0.025, 0.1, 0.9, 0.975),
        MARGIN = 2) %>%
  t() %>%
  as.data.frame() %>%
  rownames_to_column(var = "par") %>%
  filter(grepl("b_|sd_|sigma", par)),
  by = "par",
  all.y = TRUE) %>%
  mutate(ten = 30 - `90%`,
         ninety = 30 - `10%`,
         twoPointFive = 30 - `97.5%`,
         ninetysevenPointFive = 30 - `2.5%`
         ) %>%
  mutate_if(is.numeric, .funs = round, digits = 3) %>%
  mutate(`50\\% CIs` = paste0("[", ten, ", ", ninety, "]"),
         `95\\% CIs` = paste0("[", twoPointFive, ", ", ninetysevenPointFive,
                              "]")
         ) %>%
  mutate(median = ifelse(par == "b_breakPoint_Intercept",
                         30 - median, median)
         ) %>%
  merge(.,
        tribble(~par, ~Parameter,
                "b_Intercept_Intercept",
                "Metabolism at 30°C (mL O\\textsubscript{2}/min)",
                "b_resistance_Intercept",
                "Thermal Resistance (mL O\\textsubscript{2}/min/°C)",
                "b_breakPoint_Intercept",
                "Lower Critical Temperature (°C)",
                "sd_ring__Intercept_Intercept",
                "Bird ID (Metabolism Intercept)",
                "sd_ring__resistance_Intercept",
                "Bird ID (Thermal Resistance)",
                "sd_ring__breakPoint_Intercept",
                "Bird ID (Lower Critical Temperature)",
                "sigma", "Sigma"),
        by = 'par', all.x = TRUE
  ) %>%
  dplyr::select(Parameter, "Estimate" = median, `50\\% CIs`, `95\\% CIs`) %>%
  kbl(.,
    longtable = T, booktabs = T, format = "latex",
    caption = caption, escape = FALSE
  ) %>%
  kable_styling(latex_options = "striped")
```

Table 113: *Results of a Bayesian piece-wise regression predicting resting metabolism (mL O_2/min) of adult Japanese quail across ambient temperature (°C). Break-points (here, lower critical temperature), thermal resistance, and metabolism at thermoneutrality was allowed to vary by individual. Estimates represent posterior medians and credible intervals represent quantile intervals.*

Parameter	Estimate	50% CIs	95% CIs
Lower Critical Temperature (°C)	23.979	[22.46, 25.932]	[21.72, 27.042]
Metabolism at 30°C (mL O_2/min)	7.350	[22.395, 22.912]	[22.254, 23.064]
Thermal Resistance (mL O_2/min/°C)	-0.099	[29.887, 29.915]	[29.88, 29.921]
Bird ID (Lower Critical Temperature)	1.853	[26.244, 29.631]	[25.137, 29.909]
Bird ID (Metabolism Intercept)	0.969	[28.819, 29.215]	[28.682, 29.304]
Bird ID (Thermal Resistance)	0.104	[29.867, 29.92]	[29.849, 29.931]
Sigma	0.949	[28.98, 29.114]	[28.939, 29.145]

Finally, we test whether individual body mass, tarsus length, and bill length predict lower critical temperature in quail. Here, for all population-level effects, use broad, normally-distributed priors mean-centred on zero as follows:

$$Intercept\ (\beta_0) \sim \mathcal{N}(27.5, 2.5)$$
$$Body\ mass\ (\beta_1) \sim \mathcal{N}(0, 0.2)$$
$$Tarsus\ length\ (\beta_2) \sim \mathcal{N}(0, 0.5)$$
$$Bill\ length\ (\beta_2) \sim \mathcal{N}(0, 0.7)$$

Body mass and tarsus length are both mean-centred to ease interpretation of our model intercept.

```
base = 30 - as.data.frame(model)$b_breakPoint_Intercept
grab = as.data.frame(model) %>%
  dplyr::select(starts_with("r_ring__breakPoint")) %>%
  mutate_all(.funs = function(x){return(base - x)}) %>%
  summarise_all(.funs = mean) %>%
  pivot_longer(everything(), names_to = "ring", values_to = "LCT") %>%
   mutate(ring = gsub("\\,.*", "",
    gsub(".*\\[", "", ring)
    )
  )

base <- 30 - as.data.frame(model)$b_breakPoint_Intercept

modData <- as.data.frame(model) %>%
  dplyr::select(starts_with("r_ring__breakPoint")) %>%
  mutate_all(.funs = function(x) {
    return(base - x)
  }) %>%
  summarise_all(.funs = mean) %>%
  pivot_longer(everything(), names_to = "ring", values_to = "LCT") %>%
  mutate(ring = gsub(
    "\\,.*", "",
    gsub(".*\\[", "", ring)
  )) %>%
  merge(., read.csv("lctRMRBirdIDs.csv") %>%
    mutate("mass" = (mass_in + as.numeric(mass_out)) / 2) %>%
    dplyr::select("ring" = Ring, mass),
  by = "ring", all.x = TRUE
  ) %>%
  merge(., read.csv("springWeek12Tarsus.csv"),
    by = "ring", all.x = TRUE
  ) %>%
  merge(., read.csv("springWeek12Bill.csv") %>%
    dplyr::select(-c(ventSex)),
  by = c("ring", "week"), all.x = TRUE
  ) %>%
  group_by(ring, week) %>%
  reframe(
    "sex" = ventSex[1],
    "LCT" = LCT[1],
    "mass" = mean(mass, na.rm = T),
    "tarsus" = mean(tarsus, na.rm = T),
    "bill" = mean(billLength, na.rm = T)
  )

modData <- modData %>%
  mutate(mass = mass - mean(mass, na.rm = T),
         tarsus = tarsus - mean(tarsus, na.rm = T),
         bill = bill - mean(bill, na.rm = T)
         )
```

```
lctModel <- brm(
  data = modData %>%
    dplyr::select(ring, LCT, mass, tarsus, bill) %>%
    drop_na(),
  bf(LCT ~ mass + tarsus + bill),
  family = "gaussian",
  prior = c(
    set_prior("normal(27.5, 2.5)", class = "Intercept"),
    set_prior("normal(0, 0.02)", class = "b", coef = "mass"),
    set_prior("normal(0, 0.5)", class = "b", coef = "tarsus"),
    set_prior("normal(0, 0.7)", class = "b", coef = "bill"),
    set_prior("exponential(0.5)", class = "sigma")
  ),
  iter = 100000, warmup = 50000, thin = 10,
  chains = 4, cores = 4,
  control = list(adapt_delta = .98, max_treedepth = 14),
  silent = TRUE, refresh = 0,
  file = "./models/_lctMorphometryModel2.Rds"
)

chainCheck(lctModel)
```

```
## Rhat range: 1 - 1
## Neff/N range: 0.94 - 1.003
```

```
# Clear chain convergence and draws appear sequentially independent.

pp_check2(lctModel, xlab = "Lower Critical Temperature (°C)")
```

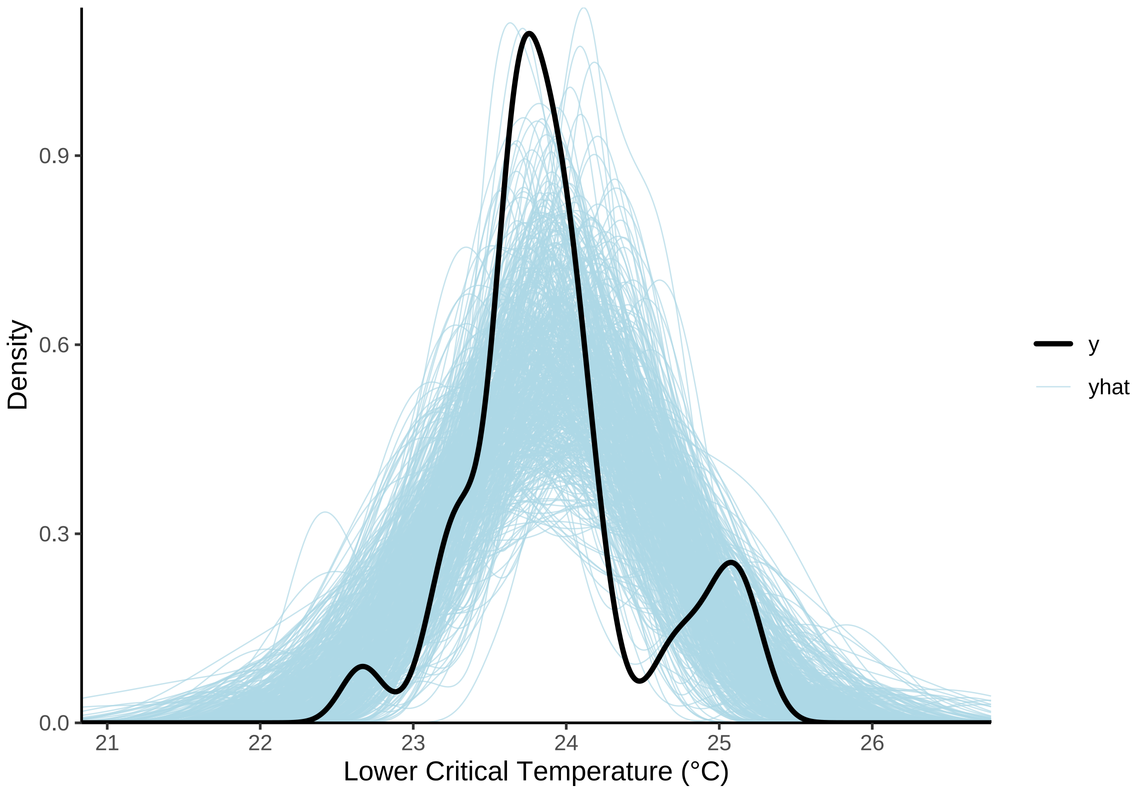

Figure 180: *Posterior predictive check for a Bayesian linear model predicting lower critical temperature (°C) of mature Japanese quail by their mean-centred body mass (g), tarsus length (mm) and bill length (mm). Light blue lines represent densities of lower critical temperature values as drawn from model posteriors. The dark blue line represent the true density of lower critical temperature values.*

As described above, we plot our model residuals to appraise structural errors and identify possible extreme values.

```
p1 <- lctModel$data %>%
  mutate("Residuals" = residuals(lctModel, robust = TRUE)[, "Estimate"]) %>%
  ggplot(aes(x = Residuals)) +
  geom_density() +
  xlab("Ordinary Residuals") +
  ylab("Density") +
  theme_classic()

p2 <- lctModel$data %>%
  mutate("Residuals" = residuals(lctModel, robust = TRUE)[, "Estimate"]) %>%
  ggplot(aes(sample = Residuals)) +
  stat_qq(colour = "grey50") +
  stat_qq_line() +
  xlab("Theoretical") +
  ylab("Sample") +
  theme_classic()


p3 <- lctModel$data %>%
  mutate("Residuals" = residuals(lctModel, robust = TRUE)[, "Estimate"]) %>%
  ggplot(aes(x = mass, y = Residuals)) +
  geom_point() +
  xlab("Body Mass (g; Mean-Centred)") +
  ylab("Ordinary Residuals") +
  theme_classic()

p4 <- lctModel$data %>%
  mutate("Residuals" = residuals(lctModel, robust = TRUE)[, "Estimate"]) %>%
  ggplot(aes(x = tarsus, y = Residuals)) +
  geom_point() +
  xlab("Tarsus Length (mm; Mean-Centred)") +
  ylab("Ordinary Residuals") +
  theme_classic()

p5 <- lctModel$data %>%
  mutate("Residuals" = residuals(lctModel, robust = TRUE)[, "Estimate"]) %>%
  ggplot(aes(x = bill, y = Residuals)) +
  geom_point() +
  xlab("Bill Length (mm; Mean-Centred)") +
  ylab("Ordinary Residuals") +
  theme_classic()

p6 <- lctModel$data %>%
  mutate(
    "Residuals" = residuals(lctModel, robust = TRUE)[, "Estimate"],
    "Fitted" = fitted(lctModel, robust = TRUE)[, "Estimate"]
  ) %>%
  ggplot(aes(x = Fitted, y = Residuals)) +
  geom_point() +
  xlab("Fitted Lower Critical\nTemperature (°C)") +
  ylab("Ordinary Residuals") +
  theme_classic()


((p1 + p2 + p3) / (p4 + p5 + p6)) + plot_annotation(tag_levels = "A")
```

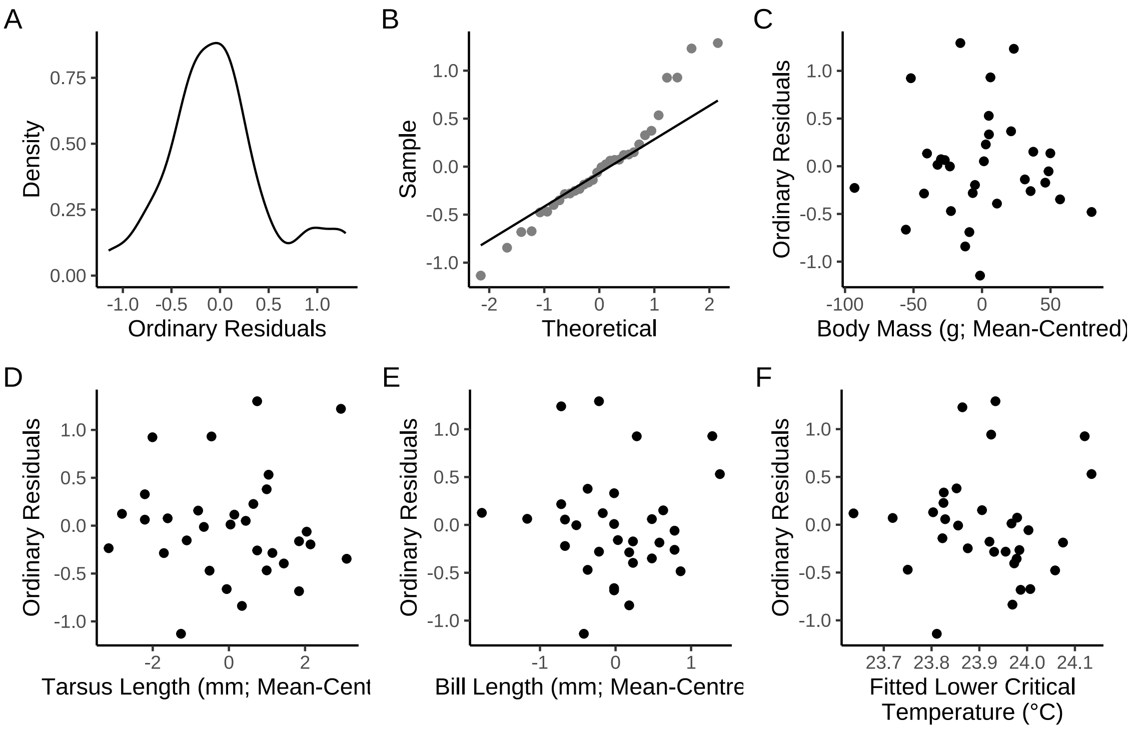

Figure 181: *Spread of ordinary residuals (here, using medians as measures of central tendancy in posteriors) from a simple Bayesian regression predicting lower critical temperature (°C) of adult Japanese quail by their mean-centred body mass (g), tarsus length (mm) and bill length (mm). Panels A and B display the distribution of residual values and their theoretical vs observed values by quantiles respectively. Panels C to E display individual residual values according to predictor values. Panel F displays residual values according to the corresponding fitted value for that individual. Dots represent individual data points.*

Some residual values appear slightly high relative to expectations, however, the spread of this particular values does not appear biased according to certain ranges of predictor variables or fitted values. We therefore proceed to summarising our model posteriors.

```
as.data.frame(lctModel) %>%
  summarise_all(., .funs = median) %>%
  pivot_longer(everything(), names_to = "Parameter",
               values_to = "Estimate") %>%
  merge(., quantileCIs(lctModel, cis = c(50, 95)),
        by = "Parameter", all.x = TRUE) %>%
  filter(grepl("b_|sd_", Parameter)) %>%
  rowwise() %>%
  mutate("BF" = ifelse(Estimate < 0,
                       (2 * mean(as.data.frame(
                         lctModel)[, Parameter] <= 0)) /
    (2 * mean(as.data.frame(
      lctModel)[, Parameter] >= 0)),
  (2 * mean(as.data.frame(
    lctModel)[, Parameter] >= 0)) /
    (2 * mean(as.data.frame(
      lctModel)[, Parameter] <= 0))
  )) %>%
  ungroup() %>%
  mutate(
    "Estimate" = round(Estimate, digits = 4),
    "BF" = round(BF, digits = 4),
    "N" = nrow(lctModel$data)
  ) %>%
```

```
  mutate("Parameter" = gsub("b_|sd_", "",
                            Parameter)) %>%
  mutate(
    "Response" = "Lower Critical Temperature",
    "Parameter" = gsub(".*_", "", Parameter)
  ) %>%
  merge(., tribble(
    ~Parameter, ~parameter, ~number,
    "Intercept", "Intercept", "1",
    "mass", "Body Mass (g)", "2",
    "tarsus", "Tarsus Length (mm)", "3",
    "bill", "Bill Length (mm)", "4",
  ),
  by = "Parameter"
  ) %>%
  mutate(
    `50\\% CI` = paste0("(", paste(
      round(Low_CI_50, digits = 4),
      round(High_CI_50, digits = 4),
      sep = ", "
    ), ")"),
    `95\\% CI` = paste0("(", paste(
      round(Low_CI_95, digits = 4),
      round(High_CI_95, digits = 4),
      sep = ", "
    ), ")")
  ) %>%
  dplyr::select(-c(Low_CI_50, High_CI_50, Low_CI_95, High_CI_95)) %>%
  dplyr::select(
    Response, "Parameter" = "parameter", N,
    Estimate, `50\\% CI`, `95\\% CI`, BF, number
  ) %>%
  arrange(number) %>%
  dplyr::select(-c(number)) %>%
  kbl(.,
    longtable = T, booktabs = T, format = "latex",
    caption = caption, escape = FALSE
  ) %>%
  column_spec(column = c(1:10), width = "2cm")  %>%
  kable_styling(latex_options = "striped")
```

Table 114: *Results of a Bayesian piece-wise regression predicting resting metabolism (mL O_2/min) of adult Japanese quail across ambient temperature (°C). Break-points (here, lower critical temperature), thermal resistance, and metabolism at thermoneutrality was allowed to vary by individual. Estimates represent posterior medians and credible intervals represent quantile intervals.*

Response	Parameter	N	Estimate	50% CI	95% CI	BF
Lower Critical Temperature	Intercept	32	23.9107	(23.8384, 23.9838)	(23.6963, 24.1283)	Inf
Lower Critical Temperature	Body Mass (g)	32	-0.0018	(-0.0042, 6e-04)	(-0.009, 0.0054)	2.2248
Lower Critical Temperature	Tarsus Length (mm)	32	0.0331	(-0.0226, 0.0888)	(-0.1346, 0.2008)	1.9078
Lower Critical Temperature	Bill Length (mm)	32	0.1431	(0.0374, 0.25)	(-0.1782, 0.4627)	4.4810

```
showtext_auto()

lctPlotMass <- ggplot(lctModel$data, aes(x = mass, y = LCT)) +
  geom_ribbon(
    data = expand.grid(
      "mass" = seq(min(lctModel$data$mass, na.rm = T),
        max(lctModel$data$mass, na.rm = T),
        by = 1
```

```
      ),
      "tarsus" = 0,
      "bill" = 0
    ) %>%
      mutate(
        "LCT" = predict(lctModel, newdata = ., robust = TRUE)[, "Estimate"],
        "SE" = predict(lctModel, newdata = ., robust = TRUE)[, "Est.Error"]
      ) %>%
      mutate(
        "LCL" = LCT - SE,
        "UCL" = LCT + SE
      ),
    aes(x = mass, ymin = LCL, ymax = UCL),
    fill = "#DECCC1", alpha = 0.4
  ) +
  geom_point(pch = 21, size = 2, colour = "black", fill = "#DECCC1", alpha = 0.8) +
  geom_line(
    data = expand.grid(
      "mass" = seq(min(lctModel$data$mass, na.rm = T),
        max(lctModel$data$mass, na.rm = T),
        by = 1
      ),
      "tarsus" = 0,
      "bill" = 0
    ) %>%
      mutate(
        "LCT" = predict(lctModel, newdata = ., robust = TRUE)[, "Estimate"],
        "LCL" = predict(lctModel, newdata = ., robust = TRUE)[, "Q2.5"],
        "UCL" = predict(lctModel, newdata = ., robust = TRUE)[, "Q97.5"]
      ),
    aes(x = mass, y = LCT),
    colour = "black", linetype = "dashed"
  ) +
  xlab("Body Mass (g)") +
  ylab("Lower Critical Temperature (°C)") +
  ylim(c(22, 26)) +
  theme_classic() +
  theme(
    axis.text = element_text(family = "Noto Sans"),
    axis.title = element_text(family = "Noto Sans")
  )

lctPlotBill <- ggplot(lctModel$data, aes(x = bill, y = LCT)) +
  geom_ribbon(
    data = expand.grid(
      "mass" = 0,
      "tarsus" = 0,
      "bill" = seq(min(lctModel$data$bill, na.rm = T),
        max(lctModel$data$bill, na.rm = T),
        by = 0.1
      )
    ) %>%
      mutate(
        "LCT" = predict(lctModel, newdata = ., robust = TRUE)[, "Estimate"],
        "SE" = predict(lctModel, newdata = ., robust = TRUE)[, "Est.Error"]
      ) %>%
      mutate(
        "LCL" = LCT - SE,
        "UCL" = LCT + SE
      ),
    aes(x = bill, ymin = LCL, ymax = UCL),
    fill = "#DECCC1", alpha = 0.4
  ) +
  geom_point(pch = 21, size = 2, colour = "black", fill = "#DECCC1", alpha = 0.8) +
  geom_line(
    data = expand.grid(
      "mass" = 0,
```

```
      "tarsus" = 0,
      "bill" = seq(min(lctModel$data$bill, na.rm = T),
        max(lctModel$data$bill, na.rm = T),
        by = 0.1
      )
    ) %>%
      mutate(
        "LCT" = predict(lctModel, newdata = ., robust = TRUE)[, "Estimate"],
        "LCL" = predict(lctModel, newdata = ., robust = TRUE)[, "Q2.5"],
        "UCL" = predict(lctModel, newdata = ., robust = TRUE)[, "Q97.5"]
      ),
    aes(x = bill, y = LCT),
    colour = "black", linetype = "dashed"
  ) +
  xlab("Bill Length (mm)") +
  ylab("Lower Critical Temperature (°C)") +
  ylim(c(22, 26)) +
  theme_classic() +
  theme(
    axis.text = element_text(family = "Noto Sans"),
    axis.title = element_text(family = "Noto Sans")
  )

lctPlot <- lctPlotMass + lctPlotBill + plot_annotation(tag_levels = "A")
lctPlot
```

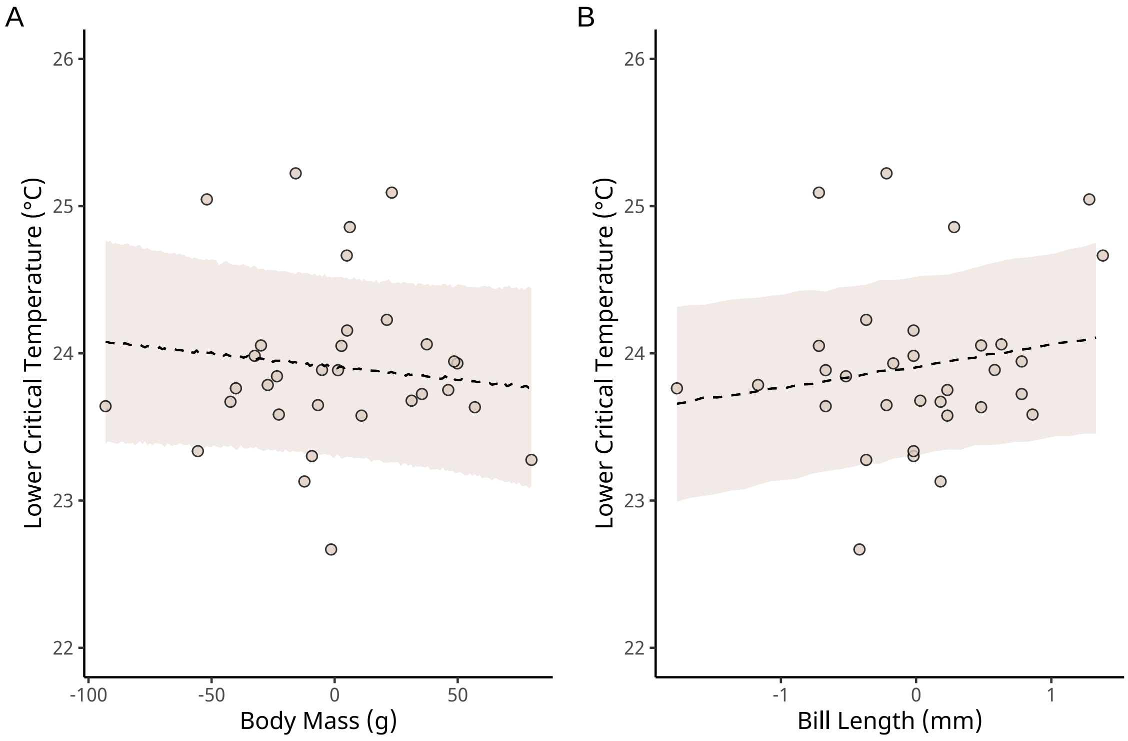

Figure 182: *Lower critical temperature (°C) of adult Japanese quail as a function of mean-centred body mass (g; panel A) and mean-centred bill length (mm; panel B). Lower critical temperature is estimate from a Bayesian mixed effects break-point regression with resting metabolism (ml O_2/min) as the response variable and ambient temperature (°C) as the sole population-level predictor. Dots represent individual values and the dashed line represents the predicted line of best fit, as estimated from a Bayesian linear model. The ribbon indicates +/- one standard error around the line of best fit.*

```
ggsave("./plots/lctPlot.pdf", dpi = 800,
       width = 7.5, height = 5,
```

```
       lctPlot)

showtext_auto(enable = FALSE)
```

6.0 Morphology and body temperature responses to heat and cold

Overview

Contrasting long-standing assumptions, we found that neither body size (proxied by body mass) nor appendage length influenced the rate at which adult Japanese quail increased there metabolism across a cold challenge (from 30°C to 10°C; see main text). This finding suggests that neither variable detectably affected the rates at which body heat was lost to the environment, and thus, the rates at which heat compensatory heat production was demanded. However, it is also possible that body size and appendage length did indeed influence rates of body heat loss, and those with highest rates of heat loss experienced declines in body temperature owing to insufficient rates of compensatory heat production. To evaluate this possibility, we tested whether body mass or appendage length (here, both tarsus and bill length) influenced the degree to which core body temperature of our quail changed between thermoneutral conditions (30°C) and our lowest temperature exposure (10°C).

Body temperature measurement

To measure core body temperatures of adult quail throughout cold exposures, we used remotely-monitored, temperature sensitive passive integrated transponder (PIT) tags (LifeChip BioTherm, Destron Fearing, South St. Paul, MN, USA; 2.1 × 12 mm; < 0.5% of body mass) implanted in the peritoneum. Tags were calibrated and implanted between 13 and 16 days of age following methods described in Persson et al (2024). During cold exposures, temperatures from tags were read passively but at a frequency of approximately 5 reads/min. For our analyses, we quantified cold-induced changes in body temperature as the difference in mean body temperature (°C) displayed at 10°C and 30°C, per individual.

Description of analyses

To analyse whether and how body mass and appendage length influenced body temperature responses to the cold, we used a Bayesian path analysis similar to those described in our main text (Tabh et al, 2025). Using a path analysis allowed us to control for, and measure, direct and indirect effects of rearing conditions (here, 10°C, 20°C, or 30°C) on morphology and body temperature responses. Here, our path analysis was composed of four models with uncorrelated residuals as follows:

$$Body\ Mass_j \sim \beta_{a0} + \beta_{a1} \cdot Cold\ Rearing_j + \beta_{a2} \cdot Warm\ Rearing_j + \\ \mu_{0a} + \epsilon_a$$

$$Tarsus\ Length_j \sim \beta_{b0} + \beta_{b1} \cdot Cold\ Rearing_j + \beta_{b2} \cdot Warm\ Rearing_j + \beta_{b3} \cdot Body\ Mass_j + \\ \mu_{0b} + \epsilon_b$$

$$Bill\ Length_j \sim \beta_{c0} + \beta_{c1} \cdot Cold\ Rearing_j + \beta_{c2} \cdot Warm\ Rearing_j + \beta_{c3} \cdot Body\ Mass_j + \\ \mu_{0c} + \epsilon_c$$

and:

$$Delta\ Tb_j \sim \beta_{d0} + \beta_{d1} \cdot Cold\ Rearing_j + \beta_{d2} \cdot Warm\ Rearing_j + \beta_{d3} \cdot Body\ Mass_j + \\ \beta_{d4} \cdot Tarsus\ Length_j + \beta_{d5} \cdot Bill\ Length_j + \mu_{0d} + \epsilon_d$$

where *j* represents individual (or, observation) identity, "warm rearing" and "cold rearing" represent binomial terms with "0" implying "false" and "1" implying"true", μ_{0a} -μ_{0c} representing group-level intercepts, per response variable, of egg batch, *Delta Tb* indicating the change in mean body temperature observed at 10°C and 30°C, and body mass and tarsus length measures being mean-centred to simplify model intercepts (β_0 terms). Error terms (ϵ) were normally distributed with a mean of zero.

Priors for the first two models in our path analysis, including their derivations, are described in the main text and in supplemental files 2 and 3. Briefly, these priors were as follows:

$$\beta_{a0} \sim \mathcal{N}(0, 10)$$
$$\beta_{a1} \sim \mathcal{N}(0, 25)$$
$$\beta_{a2} \sim \mathcal{N}(0, 25)$$
$$\mu_{0a} \sim \exp(2.5)$$
$$\epsilon_a \sim \exp(0.15)$$

$$\beta_{b0} \sim \mathcal{N}(0, 2.5)$$
$$\beta_{b1} \sim \mathcal{N}(0, 2.5)$$
$$\beta_{b2} \sim \mathcal{N}(0, 2.5)$$
$$\beta_{b3} \sim S\mathcal{N}(0, 0.25, 5)$$
$$\mu 0b \sim \exp(2)$$
$$\epsilon_b \sim \exp(1)$$

$$\beta_{c0} \sim \mathcal{N}(0, 1)$$
$$\beta_{c1} \sim \mathcal{N}(0, 0.5)$$
$$\beta_{c2} \sim \mathcal{N}(0, 0.5)$$
$$\beta_{c3} \sim S\mathcal{N}(0, 0.25, 5)$$
$$\mu 0c \sim \exp(5)$$
$$\epsilon_c \sim \exp(2.5)$$

For the fourth model in our path analysis, priors were weak and moderately informed by Persson et al (2024).

For the effects of body mass, tarsus length and bill length on body temperature responses, we assumed that effects larger than the range of body temperature responses divided by the range of the given morphological variable were unlikely (here, standard deviation of a normally-distributed prior $= 0.5 \cdot \frac{Range[Delta\ Tb]}{Range[Mass,\ Tarsus,\ Bill]}$). Priors for this model were therefore as follows:

$$\beta_{d0} \sim \mathcal{N}(1, 1)$$
$$\beta_{d1} \sim \mathcal{N}(0, 1)$$
$$\beta_{d2} \sim \mathcal{N}(0, 1)$$
$$\beta_{d3} \sim \mathcal{N}(0, 0.01)$$
$$\beta_{d4} \sim \mathcal{N}(0, 0.1)$$
$$\beta_{d5} \sim \mathcal{N}(0, 0.4)$$
$$\mu 0d \sim \exp(2.5)$$
$$\epsilon_d \sim \exp(2.5)$$

Data import, collation, and filtration

Below, we begin by importing all packages and functions required for this analysis. We then set our working directly to simplify import and collation of our data.

```
library("tidyverse")
library("easypackages")

packageList <- c("bayesplot", "brms", "doParallel",
                 "foreach", "ggpubr", "kableExtra",
                 "latex2exp", "patchwork", "priorsense",
                 "showtext", "tidybayes", "wesanderson")

libraries(packageList)
library("brmsMethods")

# Printing package version numbers

caption <- paste0("R packages and their respective versions used for",
                  " data organisation and analysis in this study."
)

sapply(packageList, function(x) {
  y <- as.character(packageVersion(x))
  return(y)
}, simplify = FALSE) %>%
  enframe(., name = "Package", value = "Version") %>%
  as.data.frame(.) %>%
  kbl(.,
    longtable = T, booktabs = T,
    caption = caption
  ) %>%
  kable_styling(latex_options = "striped")
```

Table 115: *R packages and their respective versions used for data organisation and analysis in this study.*

Package	Version
bayesplot	1.11.1
brms	2.22.7
doParallel	1.0.17
foreach	1.5.2
ggpubr	0.6.0
kableExtra	1.4.0
latex2exp	0.9.6
patchwork	1.2.0
priorsense	1.0.2
showtext	0.9.7
tidybayes	3.0.6
wesanderson	0.3.7

```
# Adding custom functions

## A function to calculate the mode of a vector

md <- function(x) {
  all_values <- unique(x)
  all_values[which.max(tabulate(match(x, all_values)))]
}

# A function to cleanly view autocorrelation between posterior
# draws of specified coefficients/variables

clean_ac <- function(x, prs = NA, names = NA) {
  require("rstan")

  if (class(x)[1] != "brmsfit") {
    return("x must be a brmsfit object.")
  }
```

```
  if (is.na(prs[1])) {
    return(stan_ac(x$fit))
  }

  if (!is.na(prs[1]) & is.na(names[1])) {
    return(stan_ac(x$fit, pars = prs))
  }

  if (length(prs) != length(names)) {
    return("Length of pars and names must be equal.")
  }

  Base_plot <- stan_ac(x$fit, pars = prs, fill = nice_pink)
  Base_plot$data$parameters <- as.character(Base_plot$data$parameters)

  for (i in 1:length(prs)) {
    Base_plot$data$parameters[c(which(Base_plot$data$parameters == prs[i]))] <-
      names[i]
  }
  Base_plot$data$parameters <- as.factor(Base_plot$data$parameters)

  return(Base_plot)
}

# A function to simplify the output of bayestestR's hdi function.

simple_hdi <- function(x, rnd = 3, cis = c(50, 95), sci_note = FALSE) {
  if (class(x)[1] != "brmsfit") {
    return("x must be a brmsfit object.")
  }
  if (length(cis) != 2) {
    return("cis must be a vector of integers with length 2")
  }

  HDI_low <- bayestestR::hdi(x, effects = "all", ci = min(cis) / 100)
  HDI_high <- bayestestR::hdi(x, effects = "all", ci = max(cis) / 100)

  if (sci_note == FALSE) {
    Results <- data.frame(
      "Parameter" = HDI_low$Parameter,
      "1" = round(HDI_low$CI_low, rnd),
      "2" = round(HDI_low$CI_high, rnd),
      "3" = round(HDI_high$CI_low, rnd),
      "4" = round(HDI_high$CI_high, rnd)
    )
    colnames(Results)[c(2:5)] <-
      c(paste0("Low_HDI_", min(cis)), paste0("High_HDI_", min(cis)),
        paste0("Low_HDI_", max(cis)), paste0("High_HDI_", max(cis)))
  } else if (sci_note == TRUE) {
    Results <- data.frame(
      "Parameter" = HDI_low$Parameter,
      "1" = format(round(HDI_low$CI_low, rnd), scientific = TRUE),
      "2" = format(round(HDI_low$CI_high, rnd), scientific = TRUE),
      "3" = format(round(HDI_high$CI_low, rnd), scientific = TRUE),
      "4" = format(round(HDI_high$CI_high, rnd), scientific = TRUE)
    )
    colnames(Results)[c(2:5)] <- c(paste0("Low_HDI_", min(cis)),
                                   paste0("High_HDI_", min(cis)),
                                   paste0("Low_HDI_", max(cis)),
                                   paste0("High_HDI_", max(cis)))
  }

  return(Results)
}

modeHDI <- function(x, cis = c(50, 95), rnd = 4, collapse = FALSE){
  stopifnot("x must be a 'brmsfit' object" = class(x) == "brmsfit",
```

```
            "collapse must be logical TRUE/FALSE" = is.logical(collapse))

  out <- lapply(X = as.data.frame(x), MARGIN = 2, FUN = ggdist::mode_hdi,
                .width = c(cis/100))
  hold <- names(out)
  out <- out %>%
    map2(hold, ~mutate(.x, name = .y)) %>%
    bind_rows() %>%
    mutate(y = round(y, digits = rnd),
           ymin = round(ymin, digits = rnd),
           ymax = round(ymax, digits = rnd)) %>%
    select("par" = name, "mode" = y, "lcl" = ymin,
           "ucl"= ymax, "confidenceLevel" = .width)

  if (collapse == FALSE){
    return(out)
  } else if (collapse == TRUE){
    out <- out %>%
      mutate("cis" = paste0("[", lcl, ", ", ucl, "]")) %>%
      select(-c(lcl, ucl))

    return(out)
  }
}


# A function to calculate quantile intervals from a brmsfit object

quantileCIs <- function(x, rnd = 3, cis = c(50, 95), sci_note = FALSE) {
  require(tidyverse)

  if (class(x)[1] != "brmsfit") {
    return("x must be a brmsfit object.")
  }
  if (length(cis) != 2) {
    return("cis must be a vector of integers with length 2")
  }

  prbs = c()
  nColNames = c()
  for (i in 1:length(cis)){
    prbs = c(prbs, c(0.5 - (cis[i]/100)/2, 0.5 + (cis[i]/100)/2))
    nColNames = c(nColNames,
                  paste0("Low_CI_", cis[i]),
                  paste0("High_CI_", cis[i])
    )
  }

  modelFrame = as.data.frame(x)

  Results <- apply(modelFrame, MARGIN = 2, FUN = quantile,
        probs = prbs, type = 8) %>%
    t() %>%
    as.data.frame() %>%
    rownames_to_column(var = "par") %>%
    `colnames<-`(c("Parameter", nColNames))

  if (sci_note == FALSE) {
    Results <- apply(modelFrame, MARGIN = 2, FUN = quantile,
        probs = prbs, type = 8) %>%
    t() %>%
    as.data.frame() %>%
    rownames_to_column(var = "par") %>%
    `colnames<-`(c("Parameter", nColNames))

  } else if (sci_note == TRUE) {
    Results <- apply(modelFrame, MARGIN = 2, FUN = quantile,
```

```
        probs = prbs, type = 8) %>%
    t() %>%
    as.data.frame() %>%
    rownames_to_column(var = "par") %>%
    `colnames<-`(c("Parameter", nColNames)) %>%
    mutate_at(.vars = vars(-Parameter),
              .funs = function(x){
                return(format(x, scientific = TRUE))
              }
    )
  }

  return(Results)
}

# A function that allows users to assign multiple objects
# to different variable names at once. The below is reported by "ellbur" at
# https://strugglingthroughproblems.wordpress.com/author/ellbur/page/3/.

{
  "%=%" <- function(l, r, ...) UseMethod("%=%")

  "%=%.lbunch" <- function(l, r, ..., List = NA) {
    Envir <- as.environment(-1)

    if (!is.na(List)) {
      l <- List[[1]]
      r <- List[[2]]
    }

    if (length(r) > length(l)) {
      warning("RHS has more args than LHS. Only first",
              length(l), "used.")
    }

    if (length(l) > length(r)) {
      warning("LHS has more args than RHS. RHS will be repeated.")
      r <- extendToMatch(r, l)
    }

    for (II in 1:length(l)) {
      do.call("<-", list(l[[II]], r[[II]]), envir = Envir)
    }
  }

  extendToMatch <- function(source, destin) {
    s <- length(source)
    d <- length(destin)

    if (d == 1 && s > 1 && !is.null(as.numeric(destin))) {
      d <- destin
    }

    dif <- d - s
    if (dif > 0) {
      source <- rep(source, ceiling(d / s))[1:d]
    }
    return(source)
  }

  g <- function(...) {
    List <- as.list(substitute(list(...)))[-1L]
    class(List) <- "lbunch"
    return(List)
  }
}
```

```
# A function to simplify output of bayes_R2 from brms

simpleR2 <- function(x, ndraws = 1000, roundDigits = 5,
                     robust = TRUE) {
  stopifnot("x must be a 'brmsfit' object" = class(x) == "brmsfit",
            "robust must be logical (TRUE/FALSE)" = is.logical(robust))
  grab <- brms::bayes_R2(x, ndraws = ndraws, robust = robust)
  toPrint <- paste0(
    "R2 = ", round(grab[, "Estimate"], digits = roundDigits),
    " [",
    round(grab[, "Q2.5"], digits = roundDigits),
    ",",
    round(grab[, "Q97.5"], digits = roundDigits),
    "]"
  )
  cat(toPrint)
}

# A function to calculate the position of a skew-normal
# distribution given its mean, omega, and alpha values

skewxi <- function(mean, omega, alpha) {
  delta <- alpha / (sqrt(1 + alpha^2))
  xi <- mean - omega * delta * sqrt(2 / pi)
  return(xi)
}

# A function to produce clean posterior or prior
# predictive checks, based upon "pp_check" from the R-package 'brms'.

pp_check2 <- function(model, resp = NA, ndraws = 500,
                      xlab = "label", colour = "lightblue") {
  require(brms)
  require(ggplot2)
  stopifnot("Model must be a brmsfit object" = is.brmsfit(model))

  if (is.na(resp)) {
    resp <- model$formula$resp
  }

  p1 <- brms::pp_check(model, ndraws = ndraws, resp = resp) +
    scale_colour_manual(
      values = c("black", colour),
      labels = c("y", "yhat"),
      name = NULL
    ) +
    xlab(xlab) +
    ylab("Density") +
    theme_classic()
  return(p1)
}

# A function to summarise and print Gelman-Rubin statistics and effective
# sample sizes to sample sizes for brmsfit objects.

neffBase <- function(x){
  stopifnot("Model must be a brmsfit object" = is.brmsfit(x))
  out <- as.data.frame(brms::neff_ratio(x)) %>%
    rownames_to_column(var = "var") %>%
    filter(!(var %in% c("lprior", "lp__"))) %>%
    pull(.)
  return(out)
}

chainCheck <- function(model, rDig = 3) {
  require(brms)
  stopifnot("Model must be a brmsfit object" = is.brmsfit(model))
```

```
  Rhat <- paste0(
    "Rhat range: ",
    round(min(rhat(model)), digits = rDig),
    " - ",
    round(max(rhat(model)), digits = rDig)
  )
  Neff <- paste0(
    "Neff/N range: ",
    round(min(neffBase(model)), digits = rDig),
    " - ",
    round(max(neffBase(model)), digits = rDig)
  )
  cat(paste0(Rhat, "\n", Neff))
}

# Setting working directory.

setwd("/Users/joshuatabh/analyses")
```

Next, we import and organise data pertaining to body temperature measurements. Raw body temperature means are then plotted to check for erroneous measurements using a Cleveland dot plot.

```
# Loading and binding data as described in section 3.0

{
  all <- merge(
    read.csv("compiledDataFull.csv") %>%
      dplyr::select(-Ta),
    bind_rows(
      read.csv("exp1VO2.csv"),
      read.csv("exp2VO2.csv"),
      read.csv("exp3VO2.csv")
    ) %>%
      dplyr::select(-c(startTime, endTime, initialTb, fileName)) %>%
      mutate(pretreatment = ifelse(pretreatment == "control",
        "neutral", pretreatment
      )) %>%
      distinct() %>%
      dplyr::select(-c(pretreatment, posttreatment, birdID)),
    by = c("ring", "week", "exp"),
    all.x = TRUE
  ) %>%
    distinct() %>%
    dplyr::select(
      ring, birdID, sex, exp, week, pretreatment,
      posttreatment, treatment, Ta, mass, wingLength,
      meanTb, tarsusLengthMean, tarsusLengthSD,
      billLengthMean, billLengthSD, VO2, RMR
    ) %>%
    mutate(Ta = ifelse(exp == "C" & Ta > 28 & Ta < 31, 30, Ta)) %>%
    mutate(Ta = ifelse(exp == "C" & Ta > 38 & Ta < 41, 40, Ta)) %>%
    filter(Ta %in% c(10, 20, 30, 40))

  all <- rbind(
    all %>%
      filter(ring == "B14" & week == "3") %>%
      group_by(Ta) %>%
      mutate(
        "tarsusLengthMean" = mean(tarsusLengthMean, na.rm = T),
        "billLengthMean" = mean(billLengthMean, na.rm = T)
      ) %>%
      mutate("n" = 1:2) %>%
      ungroup() %>%
      arrange(n, Ta) %>%
      slice(1:4) %>%
      dplyr::select(-n),
```

```
    all %>%
      filter(!(ring == "B14" & week == "3"))
  ) %>%
    arrange(exp, week, ring, Ta)

  # Splitting out experiment 3 data and adding in body temperature data

  all <- all %>%
    filter(exp == "C") %>%
    dplyr::select(-meanTb) %>%
    merge(.,
      read.csv("exp3Tb.csv") %>%
        dplyr::select(-X) %>%
        mutate(Ta = ifelse(Ta > 28 & Ta < 31, 30, Ta)) %>%
        mutate(
          Ta = ifelse(Ta > 38 & Ta < 41, 40, Ta),
          week = ifelse(week == 4, "3",
            ifelse(week == 9, "8",
              "12"
            )
          )
        ) %>%
        filter(Ta %in% c(30, 40) & week %in% c(3, 8)) %>%
        dplyr::select(ring, week, Ta, meanTb),
      by = c("ring", "week", "Ta"),
      all.x = TRUE
    ) %>%
    bind_rows(
      .,
      all %>%
        filter(exp != "C")
    ) %>%
    arrange(exp, week, ring) %>%
    filter(week == 8)
}

# Plotting body temperature with Cleveland dotplot to check for erroneous
# measurements

lims <- all %>%
  filter(!is.na(meanTb)) %>%
  mutate(
    "Ta" = paste0(Ta, "°C")
  ) %>%
  group_by(Ta) %>%
  summarise(
    "LCL" = mean(meanTb, na.rm = T) -
      3.5 * sd(meanTb, na.rm = T),
    "UCL" = mean(meanTb, na.rm = T) +
      3.5 * sd(meanTb, na.rm = T),
    meanTb = mean(meanTb, na.rm = T)
  )

all %>%
  filter(!is.na(meanTb)) %>%
  mutate(
    "Ta" = paste0(Ta, "°C")
  ) %>%
  ggplot(aes(x = 1:nrow(.), y = meanTb)) +
  facet_wrap(~ Ta) +
  geom_rect(
    data = lims,
    colour = "black", fill = "grey80", alpha = 0.5,
    aes(
      group = Ta,
      xmin = -Inf, xmax = Inf,
      ymin = LCL, ymax = UCL),
```

```
    inherit.aes = FALSE
  ) +
  geom_point(size = 2, colour = "black", alpha = 0.7) +
  theme_classic() +
  xlab("Sample Number") +
  ylab("Mean Body Temperature (°C)")
```

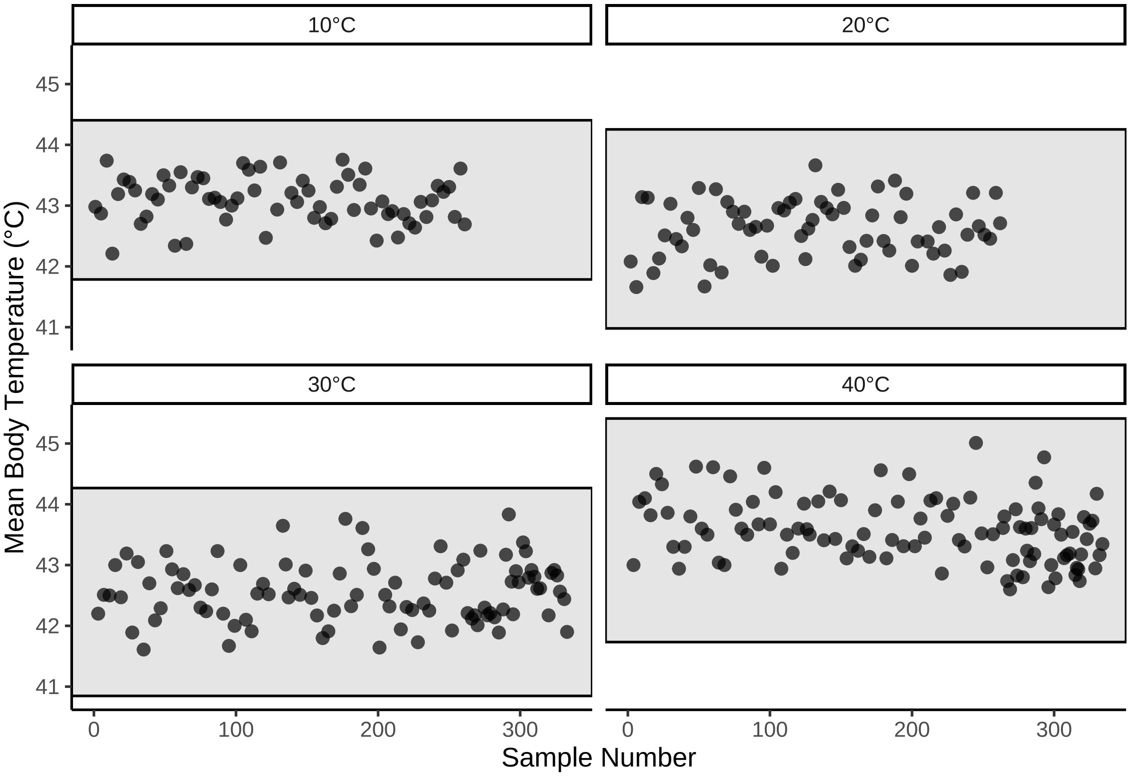

Figure 183: *Cleveland dotplot of mean body temperature values (°C) by ambient temperature of collection, each drawn from adult Japanese quail. Dots represent raw data points. Rectangles indicate mean body temperatures +/- 3.5 times the standard deviation at a given ambient temperature.*

No extreme values are evident from our dotplot. Next, we calculate body temperature responses from 30°C to 10°C and visualise these values for oddities.

```
tbResponse <- rbind(
  all %>%
    filter(Ta %in% c(10, 30)) %>%
    rename(
      "batch" = exp,
      "tarsus" = tarsusLengthMean,
      "bill" = billLengthMean
    ) %>%
    pivot_wider(
      id_cols = c(
        "ring", "batch", "week", "pretreatment",
        "mass", "tarsus", "bill"
      ), names_from = "Ta",
      values_from = "meanTb"
    ) %>%
    mutate(deltaTb = `10` - `30`) %>%
    dplyr::select(-c(`10`, `30`)) %>%
    mutate("challenge" = "cold"),
```

```
  all %>%
    filter(Ta %in% c(30, 40)) %>%
    rename(
      "batch" = exp,
      "tarsus" = tarsusLengthMean,
      "bill" = billLengthMean
    ) %>%
    pivot_wider(
      id_cols = c(
        "ring", "batch", "week", "pretreatment",
        "mass", "tarsus", "bill"
      ), names_from = "Ta",
      values_from = "meanTb"
    ) %>%
    mutate(deltaTb = `40` - `30`) %>%
    dplyr::select(-c(`30`, `40`)) %>%
    mutate("challenge" = "warm")
)

tbResponse %>%
  filter(!is.na(deltaTb) & challenge == "cold") %>%
  ggplot(aes(x = 1:nrow(.), y = deltaTb)) +
  geom_rect(aes(xmin = -Inf, xmax = Inf,
                ymin = mean(deltaTb) - 3.5*sd(deltaTb),
                ymax = mean(deltaTb) + 3.5*sd(deltaTb)
                ),
            fill = "grey80", alpha = 0.5, colour = "black") +
  geom_hline(yintercept = 0, linetype = "dashed", colour = "black") +
  geom_point(size = 2, colour = "black", alpha = 0.7) +
  scale_y_continuous(sec.axis = sec_axis( trans=~., name="Second Axis",
                                          breaks = c(1.5, -0.5),
                                          labels = c("Increasing Tb",
                                                     "Decreasing Tb"))
                     ) +
  theme_classic() +
  theme(axis.text.y.right = element_text(angle = 270),
        axis.ticks.y.right = element_blank(),
        axis.title.y.right = element_blank()) +
  xlab("Sample Number") +
  ylab("Change in\nBody Temperature (°C)")
```

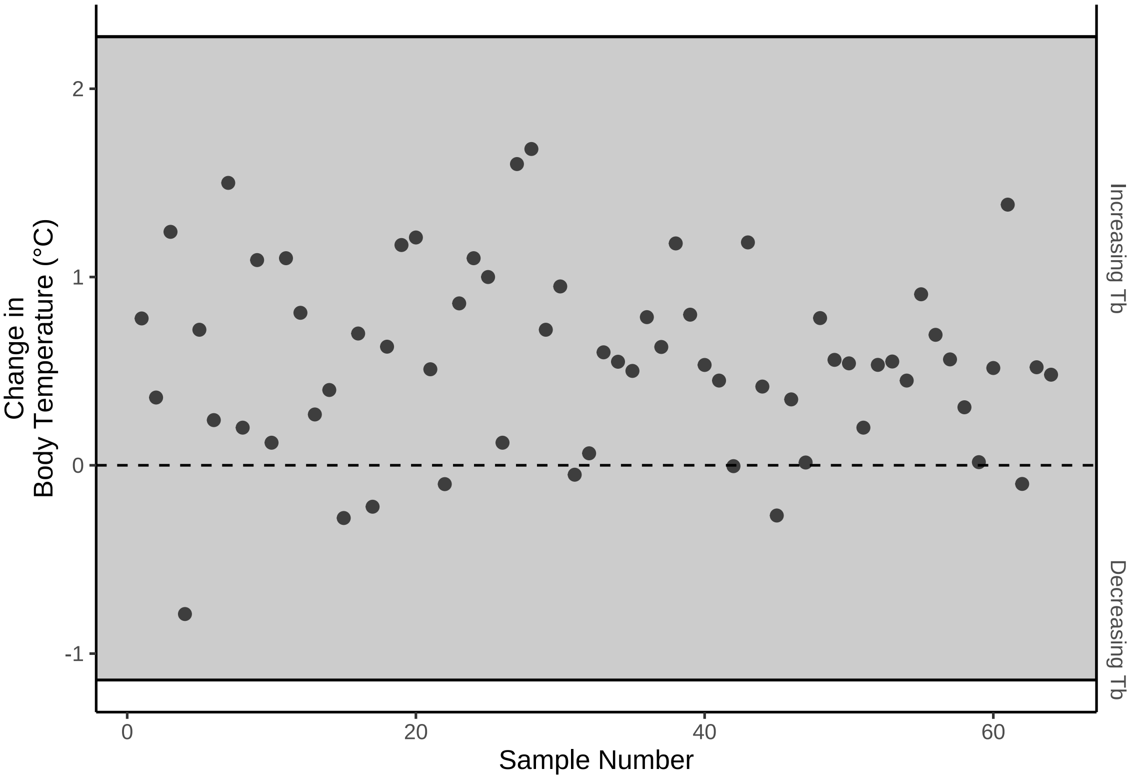

Figure 184: *Cleveland dotplot of mean changes in body temperature (°C) between 10°C and 30°C, each drawn from adult Japanese quail. Again, dots represent raw data points and rectangles indicate means +/- 3.5 times the standard deviation at a given ambient temperature. The dashed line indicates no change in body temperature.*

Raw effects of morphology and rearing conditions on body temperature responses to cold are visualised to build information for model design, if needed.

```
p1 <- tbResponse %>%
  filter(!is.na(deltaTb) & challenge == "cold") %>%
  ggplot(aes(x = mass, y = deltaTb)) +
  geom_point(size = 2, colour = "black", alpha = 0.7) +
  geom_smooth(
    method = "lm", se = FALSE, linetype = "dashed",
    colour = "black"
  ) +
  geom_hline(yintercept = 0, linetype = "dashed", colour = "firebrick4") +
  theme_classic() +
  xlab("Body Mass (g)") +
  ylab("Change in\nBody Temperature (°C)")

p2 <- tbResponse %>%
  filter(!is.na(deltaTb) & challenge == "cold") %>%
  ggplot(aes(x = tarsus, y = deltaTb)) +
  geom_point(size = 2, colour = "black", alpha = 0.7) +
  geom_smooth(
    method = "lm", se = FALSE, linetype = "dashed",
    colour = "black"
  ) +
  geom_hline(yintercept = 0, linetype = "dashed", colour = "firebrick4") +
  theme_classic() +
  xlab("Tarsus Length (mm)") +
  ylab("Change in\nBody Temperature (°C)")
```

```r
p3 <- tbResponse %>%
  filter(!is.na(deltaTb) & challenge == "cold") %>%
  ggplot(aes(x = bill, y = deltaTb)) +
  geom_point(size = 2, colour = "black", alpha = 0.7) +
  geom_smooth(
    method = "lm", se = FALSE, linetype = "dashed",
    colour = "black"
  ) +
  geom_hline(yintercept = 0, linetype = "dashed", colour = "firebrick4") +
  theme_classic() +
  xlab("Bill Length (mm)") +
  ylab("Change in\nBody Temperature (°C)")

p4 <- tbResponse %>%
  filter(!is.na(deltaTb) & challenge == "cold") %>%
  mutate(pretreatment = ifelse(pretreatment == "cold",
    "Cold (10°C)",
    ifelse(pretreatment == "neutral",
      "Mild (20°C)", "Warm (30°C)"
    )
  )) %>%
  ggplot(aes(x = pretreatment, y = deltaTb, fill = pretreatment)) +
  geom_point(
    size = 1, colour = "black", alpha = 0.5,
    position = position_jitter(width = 0.25),
    pch = 21
  ) +
  stat_summary(
    geom = "errorbar", fun.data = "mean_se",
    colour = "black", width = 0.25,
    position = position_dodge(width = 0.35)
  ) +
  stat_summary(
    geom = "point", fun = "mean", pch = 21,
    colour = "black", size = 3,
    position = position_dodge(width = 0.35)
  ) +
  geom_hline(yintercept = 0, linetype = "dashed", colour = "firebrick4") +
  scale_fill_manual(values = c("#7BB4E3", "black", "#CD5C5C")) +
  theme_classic() +
  theme(
    axis.title.x = element_blank(),
    legend.position = "none"
  ) +
  ylab("Change in\nBody Temperature (°C)")

(p1 + p2) / (p3 + p4) + plot_annotation(tag_levels = "A")
```

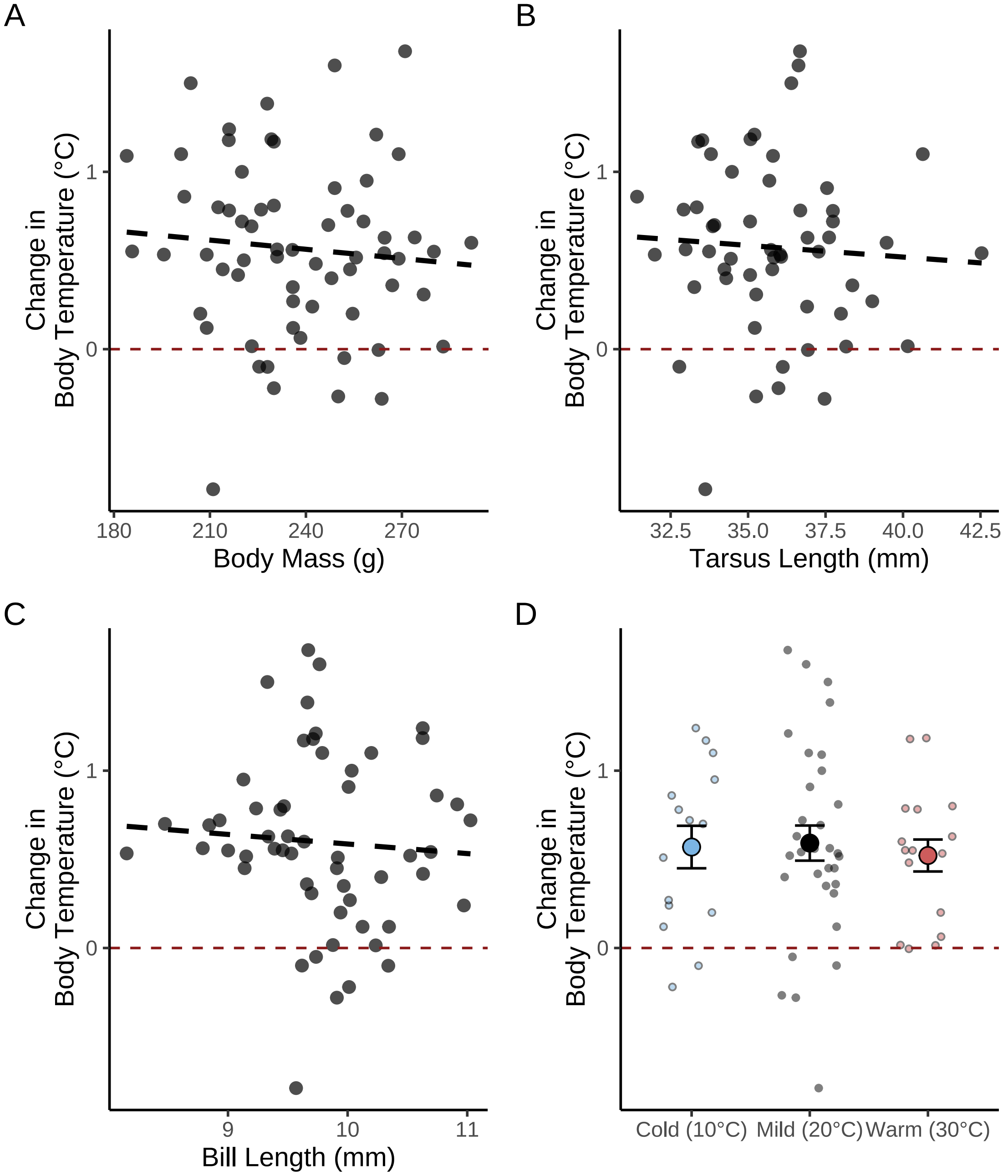

Figure 185: *Effects of body mass (g), tarsus length (mm), bill length (mm) and rearing treatment on changes in body temperature (°C) in responses to a cold exposure (10°C) in adult Japanese quail. Baseline body temperatures represent those measured at thermoneutrality (30°C). Small dots represent raw values per individual. Horizontal and dashed red lines indicate no change in body temperature. Black dashed lines represent raw lines of best fit estimated from the R package ggplot2 (Wickham, 2011). In panel D, large dots indicate means per rearing treatment and errorbars indicate standard errors.*

Last, we visually check whether variance in body temperature responses differ between rearing treatments or sources of individuals (here, egg batches).

```
# Checking whether variance in responses by treatment temperature
```

```
p1 <- tbResponse %>%
  filter(!is.na(deltaTb) & challenge == "cold") %>%
  ggplot(aes(x = deltaTb, fill = pretreatment)) +
  geom_density(colour = "black", alpha = 0.5) +
  scale_fill_manual(name = "Rearing\nTreatment",
                    labels = c("Cold\n(10°C)",
                               "Mild\n(20°C)",
                               "Warm\n(30°C)"),
                    values = c("#7BB4E3", "black", "#CD5C5C")
                    ) +
  theme_classic() +
  theme(axis.title.x = element_blank(),
        legend.position = "bottom") +
  ylab("Change in\nBody Temperature (°C)")

p2 <- tbResponse %>%
  filter(!is.na(deltaTb) & challenge == "cold") %>%
  ggplot(aes(x = deltaTb, fill = batch)) +
  geom_density(colour = "black", alpha = 0.5) +
  scale_fill_manual(name = "Egg\nBatch",
                    values = c("#7BB4E3", "black")
                    ) +
  theme_classic() +
  theme(axis.title.x = element_blank(),
        legend.position = "bottom") +
  ylab("Change in\nBody Temperature (°C)")

(p1 + p2) + plot_annotation(tag_levels = "A")
```

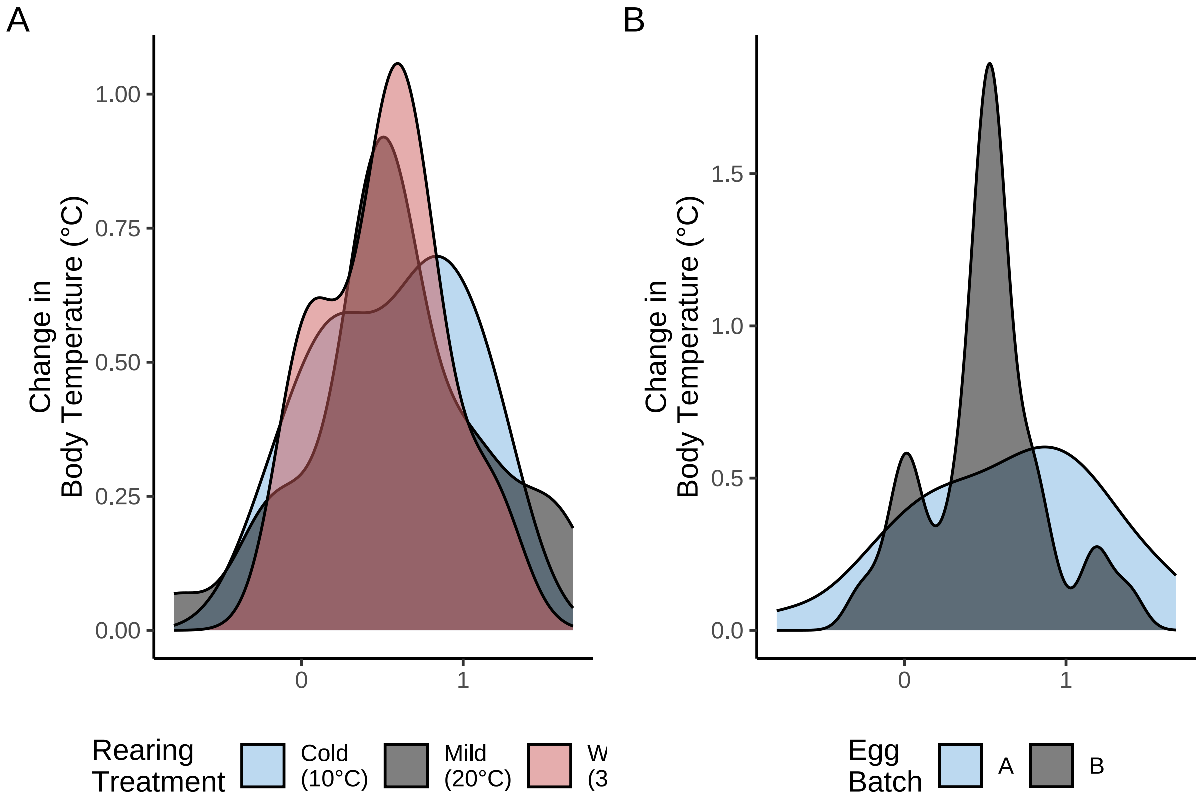

Figure 186: *Density of body temperature responses (°C) to cold exposure (10°C) in adult Japanese quail from different rearing conditions or egg batches. Baseline body temperatures represent those measured at thermoneutrality (30°C).*

Evidence for heteroskedasticity is very limited. Variance between groups is therefore treated as equal going forward.

Analysing body temperature responses to cold

We now proceed to constructing our path analysis as described previously. To test the suitability of our model priors, we first check how reasonably predictions from our priors alone overlay with the true density of our body temperature response values.

```
tbResponseCold8WeeksPPCheck <-
  brm(
    data = tbResponse %>%
      filter(challenge == "cold") %>%
      mutate(
        mass = mass - mean(mass, na.rm = T),
        tarsus = tarsus - mean(tarsus, na.rm = T),
        bill = bill - mean(bill, na.rm = T),
        pretreatment = ifelse(pretreatment == "cold", "A",
          ifelse(pretreatment == "neutral", "B", "C")
        )
      ) %>%
      mutate(pretreatment = factor(pretreatment,
        levels = c("B", "A", "C")
      )) %>%
      drop_na(),
    family = "gaussian",
    bf(mass ~ pretreatment + (1 | batch)) +
      bf(tarsus ~ mass + pretreatment + (1 | batch)) +
      bf(bill ~ mass + pretreatment + (1 | batch)) +
      bf(deltaTb ~ mass + tarsus + bill + pretreatment + (1 | batch)) +
      set_rescor(FALSE),
    prior = c(
      set_prior("normal(0, 10)",
        class = "Intercept",
        resp = "mass"
      ),
      set_prior("normal(0, 25)",
        class = "b",
        coef = "pretreatmentA",
        resp = "mass"
      ),
      set_prior("normal(0, 25)",
        class = "b",
        coef = "pretreatmentC",
        resp = "mass"
      ),
      set_prior("exponential(2.5)",
        class = "sd",
        group = "batch",
        resp = "mass"
      ),
      set_prior("exponential(0.15)",
        class = "sigma",
        resp = "mass"
      ),
      set_prior("normal(0, 2.5)",
        class = "Intercept",
        resp = "tarsus"
      ),
      set_prior("normal(0, 2.5)",
        class = "b",
        coef = "pretreatmentA",
        resp = "tarsus"
      ),
      set_prior("normal(0, 2.5)",
        class = "b",
```

```
      coef = "pretreatmentC",
      resp = "tarsus"
    ),
    set_prior("skew_normal(0, 0.25, 5)",
      class = "b",
      coef = "mass",
      resp = "tarsus"
    ),
    set_prior("exponential(2)",
      class = "sd",
      group = "batch",
      resp = "tarsus"
    ),
    set_prior("exponential(1)",
      class = "sigma",
      resp = "tarsus"
    ),
    set_prior("normal(0, 1)",
      class = "Intercept",
      resp = "bill"
    ),
    set_prior("normal(0, 0.5)",
      class = "b",
      coef = "pretreatmentA",
      resp = "bill"
    ),
    set_prior("normal(0, 0.5)",
      class = "b",
      coef = "pretreatmentC",
      resp = "bill"
    ),
    set_prior("skew_normal(0, 0.25, 5)",
      class = "b",
      coef = "mass",
      resp = "bill"
    ),
    set_prior("exponential(5)",
      class = "sd",
      group = "batch",
      resp = "bill"
    ),
    set_prior("exponential(2.5)",
      class = "sigma",
      resp = "bill"
    ),
    set_prior("normal(1, 1)",
      class = "Intercept",
      resp = "deltaTb"
    ),
    set_prior("normal(0, 1)",
      class = "b",
      coef = "pretreatmentA",
      resp = "deltaTb"
    ),
    set_prior("normal(0, 1)",
      class = "b",
      coef = "pretreatmentC",
      resp = "deltaTb"
    ),
    set_prior("normal(0, 0.01)",
      class = "b",
      coef = "mass",
      resp = "deltaTb"
    ),
    set_prior("normal(0, 0.1)",
      class = "b",
      coef = "tarsus",
```

```
      resp = "deltaTb"
    ),
    set_prior("normal(0, 0.4)",
      class = "b",
      coef = "bill",
      resp = "deltaTb"
    ),
    set_prior("exponential(2.5)",
      class = "sd",
      group = "batch",
      resp = "deltaTb"
    ),
    set_prior("exponential(2.5)",
      class = "sigma",
      resp = "deltaTb"
    )
  ),
  iter = 50000, warmup = 10000, cores = 4, chains = 4, thin = 20,
  control = list(adapt_delta = .97, max_treedepth = 14),
  silent = TRUE, refresh = 0,
  sample_prior = "only",
  file = "./models/_tbResponseToColdPPCheck.Rds"
)

pp_check2(tbResponseCold8WeeksPPCheck,
          xlab = "Change in\nBody Temperature (°C)",
          resp = "deltaTb"
          )
```

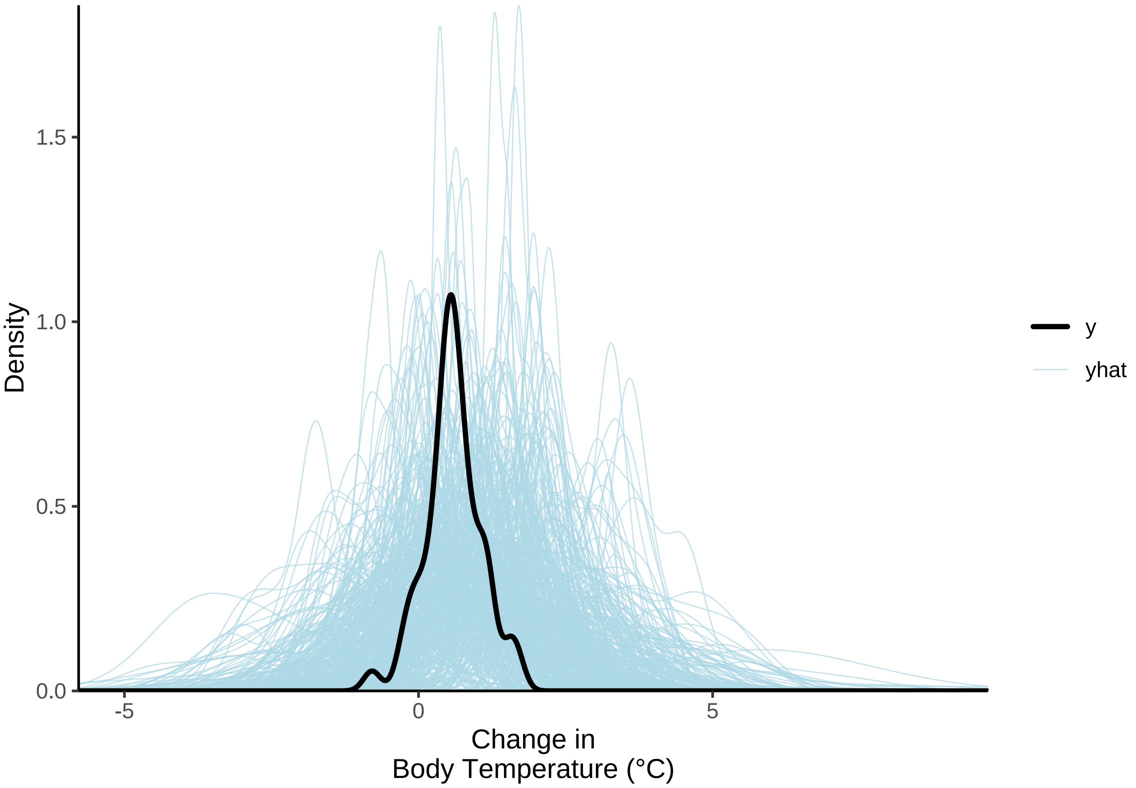

Figure 187: *Overlay of predicted (blue) and true (black) body temperature response (°C) densities, where predicted densities are derived from priors in a Bayesian path analyses. Clear overlap betwen the black and blue lines suggests that model priors are reasonable with respect to the data.*

Priors chosen for this model clearly capture the density of true body temperature responses without obvious constraints. We therefore proceed to constructing and evaluating our full path analysis as described above.

```
tbResponseCold8Weeks <-
  brm(
    data = tbResponse %>%
      filter(challenge == "cold") %>%
      mutate(
        mass = mass - mean(mass, na.rm = T),
        tarsus = tarsus - mean(tarsus, na.rm = T),
        bill = bill - mean(bill, na.rm = T),
        pretreatment = ifelse(pretreatment == "cold", "A",
          ifelse(pretreatment == "neutral", "B", "C")
        )
      ) %>%
      mutate(pretreatment = factor(pretreatment,
        levels = c("B", "A", "C")
      )) %>%
      drop_na(),
    family = "gaussian",
    bf(mass ~ pretreatment + (1 | batch)) +
      bf(tarsus ~ mass + pretreatment + (1 | batch)) +
      bf(bill ~ mass + pretreatment + (1 | batch)) +
      bf(deltaTb ~ mass + tarsus + bill + pretreatment + (1 | batch)) +
      set_rescor(FALSE),
    prior = c(
      set_prior("normal(0, 10)",
        class = "Intercept",
        resp = "mass"
      ),
      set_prior("normal(0, 25)",
        class = "b",
        coef = "pretreatmentA",
        resp = "mass"
      ),
      set_prior("normal(0, 25)",
        class = "b",
        coef = "pretreatmentC",
        resp = "mass"
      ),
      set_prior("exponential(2.5)",
        class = "sd",
        group = "batch",
        resp = "mass"
      ),
      set_prior("exponential(0.15)",
        class = "sigma",
        resp = "mass"
      ),
      set_prior("normal(0, 2.5)",
        class = "Intercept",
        resp = "tarsus"
      ),
      set_prior("normal(0, 2.5)",
        class = "b",
        coef = "pretreatmentA",
        resp = "tarsus"
      ),
      set_prior("normal(0, 2.5)",
        class = "b",
        coef = "pretreatmentC",
        resp = "tarsus"
      ),
      set_prior("skew_normal(0, 0.25, 5)",
        class = "b",
        coef = "mass",
        resp = "tarsus"
```

```
    ),
    set_prior("exponential(2)",
      class = "sd",
      group = "batch",
      resp = "tarsus"
    ),
    set_prior("exponential(1)",
      class = "sigma",
      resp = "tarsus"
    ),
    set_prior("normal(0, 1)",
      class = "Intercept",
      resp = "bill"
    ),
    set_prior("normal(0, 0.5)",
      class = "b",
      coef = "pretreatmentA",
      resp = "bill"
    ),
    set_prior("normal(0, 0.5)",
      class = "b",
      coef = "pretreatmentC",
      resp = "bill"
    ),
    set_prior("skew_normal(0, 0.25, 5)",
      class = "b",
      coef = "mass",
      resp = "bill"
    ),
    set_prior("exponential(5)",
      class = "sd",
      group = "batch",
      resp = "bill"
    ),
    set_prior("exponential(2.5)",
      class = "sigma",
      resp = "bill"
    ),
    set_prior("normal(1, 1)",
      class = "Intercept",
      resp = "deltaTb"
    ),
    set_prior("normal(0, 1)",
      class = "b",
      coef = "pretreatmentA",
      resp = "deltaTb"
    ),
    set_prior("normal(0, 1)",
      class = "b",
      coef = "pretreatmentC",
      resp = "deltaTb"
    ),
    set_prior("normal(0, 0.01)",
      class = "b",
      coef = "mass",
      resp = "deltaTb"
    ),
    set_prior("normal(0, 0.1)",
      class = "b",
      coef = "tarsus",
      resp = "deltaTb"
    ),
    set_prior("normal(0, 0.4)",
      class = "b",
      coef = "bill",
      resp = "deltaTb"
    ),
```

```
      set_prior("exponential(2.5)",
        class = "sd",
        group = "batch",
        resp = "deltaTb"
      ),
      set_prior("exponential(2.5)",
        class = "sigma",
        resp = "deltaTb"
      )
    ),
    iter = 50000, warmup = 10000, cores = 4, chains = 4, thin = 20,
    control = list(adapt_delta = .98, max_treedepth = 14),
    silent = TRUE, refresh = 0,
    file = "./models/_tbResponseToCold.Rds"
  )
```

A few (n = 19) divergent transitions were detected during our Hamiltonian Monte Carlo (HMC) chain sampling. These divergences are identified and visualised with respect to all samples to determine their cause.

```
grab <- paste0(
  grep("b_|sd_|sigma_",
    get_variables(tbResponseCold8Weeks),
    value = TRUE
  ),
  collapse = "|"
)

posteriorDraws <- as.array(tbResponseCold8Weeks)
divergences <- nuts_params(tbResponseCold8Weeks) %>%
  filter(Parameter == "divergent__" & Value == "1") %>%
  dplyr::select(".chain" = Chain, ".iteration" = Iteration) %>%
  mutate("divergent" = "TRUE")

spread_draws(tbResponseCold8Weeks, !!sym(grab), regex = TRUE) %>%
  dplyr::select(-c(.draw)) %>%
  pivot_longer(-c(".chain", ".iteration"),
    names_to = "par", values_to = "values"
  ) %>%
  mutate("chain" = paste0("Chain ", .chain)) %>%
  merge(.,
    tribble(
      ~par, ~Par,
      "b_mass_Intercept", " a0",
      "b_mass_pretreatmentA", " a1",
      "b_mass_pretreatmentC", " a2",
      "sd_batch__mass_Intercept", " 0a",
      "sigma_mass", " a",
      "b_tarsus_Intercept", " b0",
      "b_tarsus_pretreatmentA", " b1",
      "b_tarsus_pretreatmentC", " b2",
      "b_tarsus_mass", " b3",
      "sd_batch__tarsus_Intercept", " 0b",
      "sigma_tarsus", " b",
      "b_bill_Intercept", " c0",
      "b_bill_pretreatmentA", " c1",
      "b_bill_pretreatmentC", " c2",
      "b_bill_mass", " c3",
      "sd_batch__bill_Intercept", " 0c",
      "sigma_bill", " c"
    ),
    by = "par", all.x = TRUE
  ) %>%
  merge(., divergences, by = c(".chain", ".iteration"), all.x = TRUE) %>%
  mutate(divergent = ifelse(is.na(divergent), "FALSE", divergent)) %>%
  drop_na(Par) %>%
```

```r
ggplot(aes(
  x = .iteration, y = values,
  fill = divergent, alpha = divergent
)) +
facet_grid(Par ~ chain, scales = "free") +
geom_point(pch = 21, colour = "black") +
scale_fill_manual(
  values = c("grey90", "darkred"),
  name = "Divergent"
) +
scale_alpha_manual(values = c(0.2, 1), name = "Divergent") +
scale_x_continuous(breaks = c(0,1000,2000)) +
xlab("Iteration") +
ylab("Values") +
theme_classic() +
theme(
  axis.text.y = element_blank(),
  axis.ticks.y = element_blank()
)
```

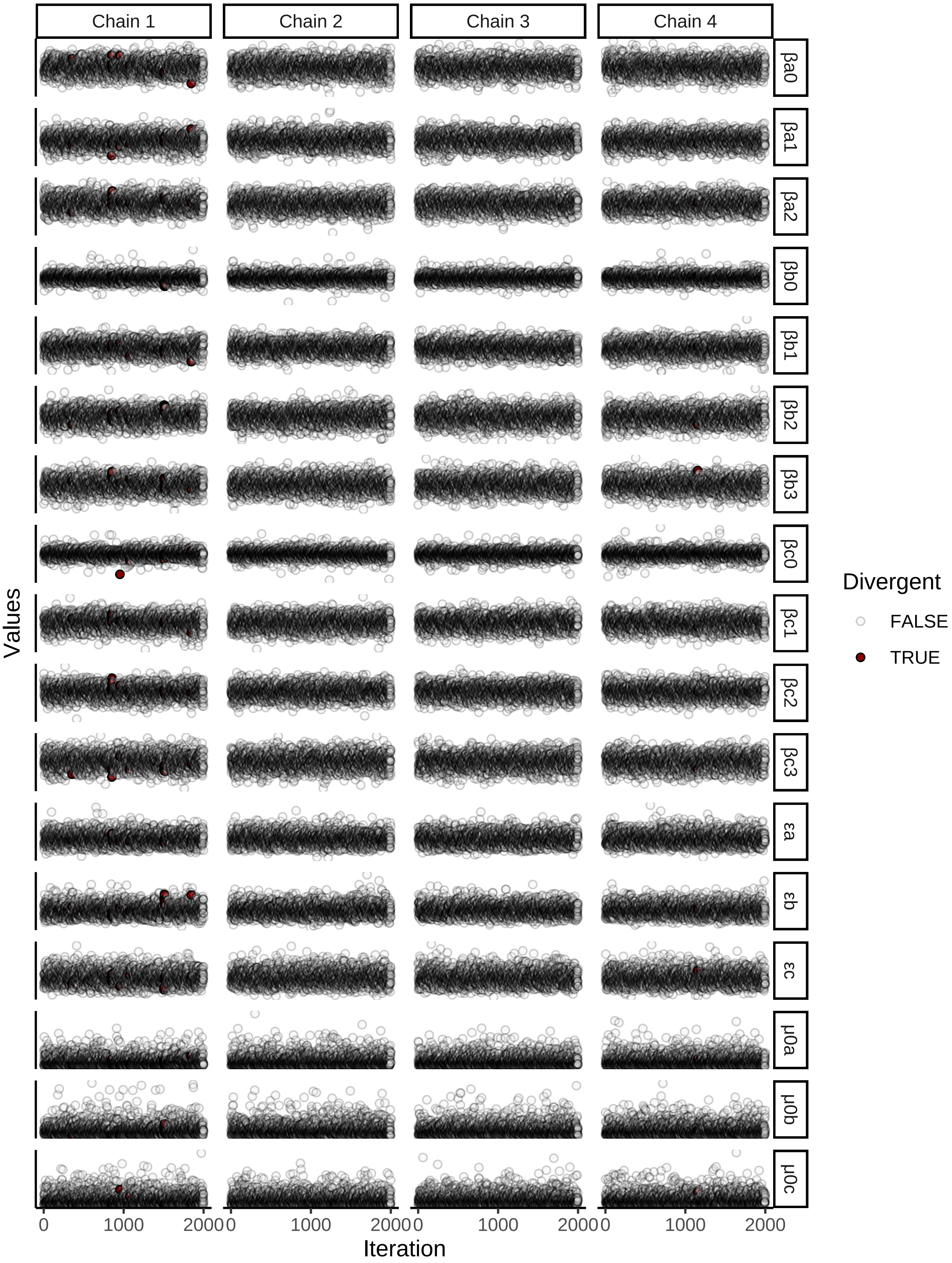

Figure 188: *Posterior draws from Bayesian path analysis predicting body temperature responses (°C) to the cold (10°C) as a direct and indirect function of body mass (g), tarsus length (mm), and bill length (mm) in adult old Japanese quail. Draws are seperated by model parameter and Hamiltonian Monte Carlo (HMC) chains. Part 1.*

```
spread_draws(tbResponseCold8Weeks, !!sym(grab), regex = TRUE) %>%
  dplyr::select(-c(.draw)) %>%
  pivot_longer(-c(".chain", ".iteration"),
    names_to = "par", values_to = "values"
  ) %>%
  mutate("chain" = paste0("Chain ", .chain)) %>%
  merge(.,
    tribble(
      ~par, ~Par,
      "b_deltaTb_Intercept", " d0",
      "b_deltaTb_pretreatmentA", " d1",
      "b_deltaTb_pretreatmentC", " d2",
      "b_deltaTb_mass", " d3",
      "b_deltaTb_tarsus", " d4",
      "b_deltaTb_bill", " d5",
      "sd_batch__deltaTb_Intercept", " 0d",
      "sigma_deltaTb", " d"
    ),
    by = "par", all.x = TRUE
  ) %>%
  merge(., divergences, by = c(".chain", ".iteration"), all.x = TRUE) %>%
  mutate(divergent = ifelse(is.na(divergent), "FALSE", divergent)) %>%
  drop_na(Par) %>%
  ggplot(aes(
    x = .iteration, y = values,
    fill = divergent, alpha = divergent
  )) +
  facet_grid(Par ~ chain, scales = "free") +
  geom_point(pch = 21, colour = "black") +
  scale_fill_manual(
    values = c("grey90", "darkred"),
    name = "Divergent"
  ) +
  scale_alpha_manual(values = c(0.2, 1), name = "Divergent") +
  scale_x_continuous(breaks = c(0,1000,2000)) +
  xlab("Iteration") +
  ylab("Values") +
  theme_classic() +
  theme(
    axis.text.y = element_blank(),
    axis.ticks.y = element_blank()
  )
```

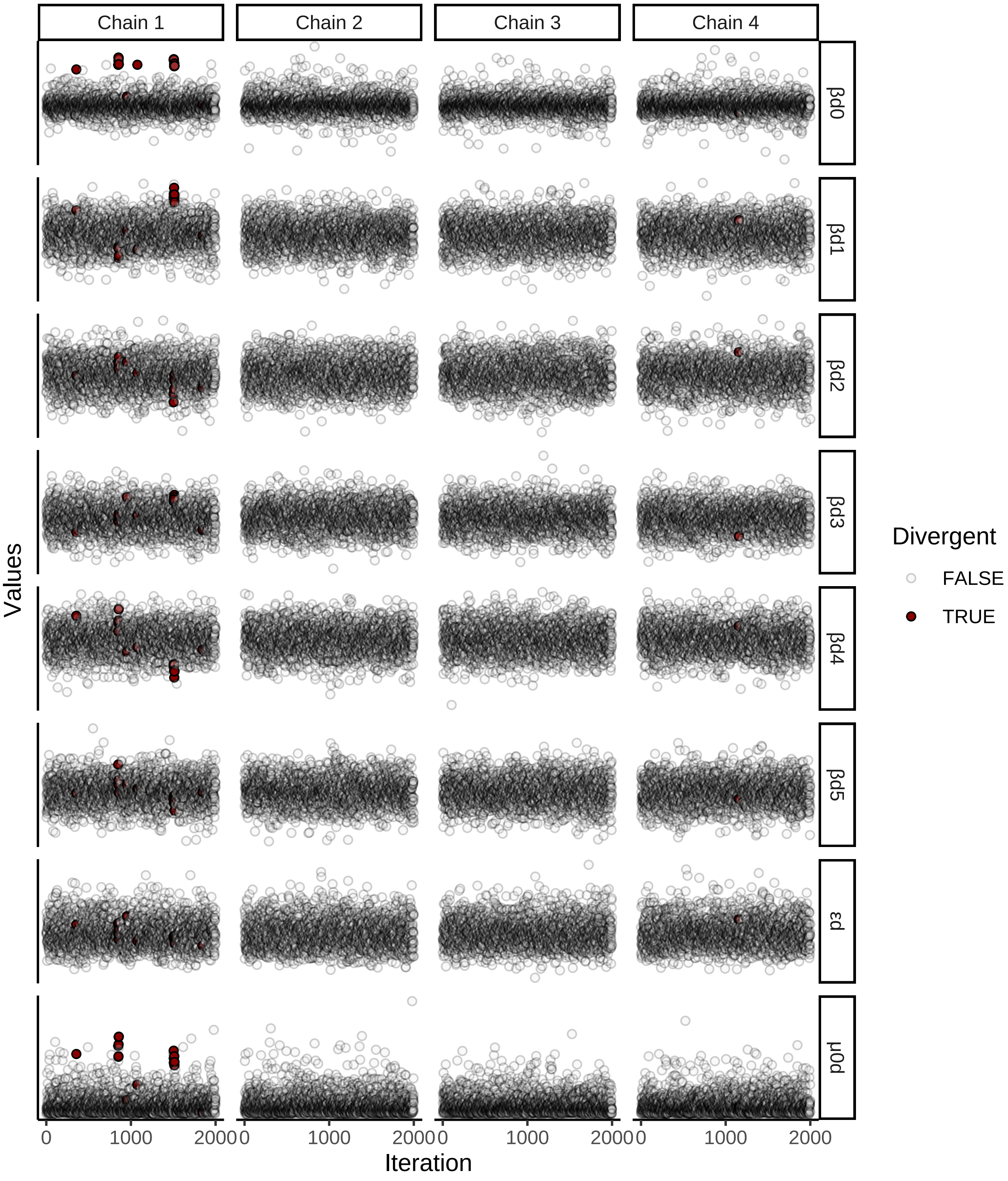

Figure 189: *Posterior draws from Bayesian path analysis predicting body temperature responses (°C) to the cold (10°C) as a direct and indirect function of body mass (g), tarsus length (mm), and bill length (mm) in adult old Japanese quail. Draws are seperated by model parameter and Hamiltonian Monte Carlo (HMC) chains. Part 2.*

Divergent transitions appear to occur when both: (1) the value for our intercept on body temperature responses, and (2) the effect of egg batch on that intercept are large Below, we assess where these divergences lie on a numeric scale.

```
p1 <- spread_draws(tbResponseCold8Weeks, !!sym(grab), regex = TRUE) %>%
  dplyr::select(-c(.draw)) %>%
  pivot_longer(-c(".chain", ".iteration"),
               names_to = "par", values_to = "values") %>%
  mutate("chain" = paste0("Chain ", .chain)) %>%
  filter(par == "b_deltaTb_Intercept") %>%
  merge(., divergences, by = c(".chain", ".iteration"), all.x = TRUE) %>%
  mutate(divergent = ifelse(is.na(divergent), "FALSE", divergent)) %>%
  ggplot(aes(x = .iteration, y = values,
             fill = divergent, alpha = divergent)) +
  geom_point(pch = 21, colour = "black") +
  geom_hline(yintercept = -1, linetype = "dashed", colour = "firebrick4") +
  geom_hline(yintercept = 3, linetype = "dashed", colour = "firebrick4") +
  scale_fill_manual(values = c("grey90", "darkred"),
                    name = "Divergent") +
  scale_alpha_manual(values = c(0.2, 1), name = "Divergent") +
  xlab("Iteration") +
  ylab("Body Temperature Response (°C)\nIntercept") +
  theme_classic() +
  theme(legend.position = "none")

p2 <- spread_draws(tbResponseCold8Weeks, !!sym(grab), regex = TRUE) %>%
  dplyr::select(-c(.draw)) %>%
  pivot_longer(-c(".chain", ".iteration"),
               names_to = "par", values_to = "values") %>%
  mutate("chain" = paste0("Chain ", .chain)) %>%
  filter(par == "sd_batch__deltaTb_Intercept") %>%
  merge(., divergences, by = c(".chain", ".iteration"), all.x = TRUE) %>%
  mutate(divergent = ifelse(is.na(divergent), "FALSE", divergent)) %>%
  ggplot(aes(x = .iteration, y = values,
             fill = divergent, alpha = divergent)) +
  geom_point(pch = 21, colour = "black") +
  geom_hline(yintercept = -1, linetype = "dashed", colour = "firebrick4") +
  geom_hline(yintercept = 3, linetype = "dashed", colour = "firebrick4") +
  scale_fill_manual(values = c("grey90", "darkred"),
                    name = "Divergent") +
  scale_alpha_manual(values = c(0.2, 1), name = "Divergent") +
  xlab("Iteration") +
  ylab("Batch Effect on\nBody Temperature Response\nIntercept") +
  theme_classic()

(p1 + p2)
```

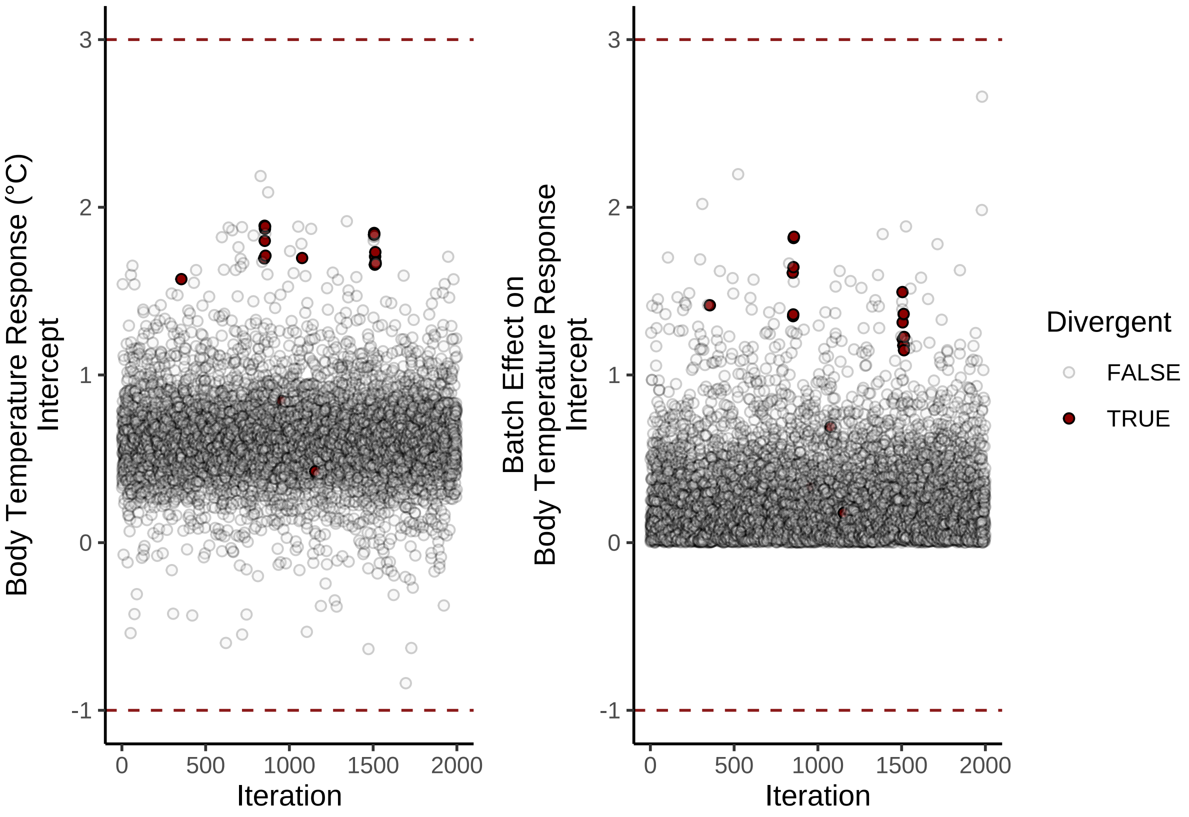

Figure 190: *Posterior draws from Bayesian path analysis predicting body temperature responses (°C) to the cold (10°C) as a direct and indirect function of body mass (g), tarsus length (mm), bill length (mm) in adult old Japanese quail. Estimate body temperature response intercepts from draws are plotted against sample iteration. Dashed red lines indicate 95% limits of our prior on the body temperature response intercept.*

Our priors on both effects appears slightly too generous and mismatched with our data. We therefore adjust these priors as follows:

$$Body\ Temperature\ Response\ Intercept\ (\beta_{d0}) \sim \mathcal{N}(1, 0.5)$$

$$Egg\ Batch\ Intercept\ (\mu_{d0}) \sim exp(3)$$

Our model is then rerun after these adjustments.

```
tbResponseCold8WeeksB <-
  brm(
    data = tbResponse %>%
      filter(challenge == "cold") %>%
      mutate(
        mass = mass - mean(mass, na.rm = T),
        tarsus = tarsus - mean(tarsus, na.rm = T),
        bill = bill - mean(bill, na.rm = T),
        pretreatment = ifelse(pretreatment == "cold", "A",
          ifelse(pretreatment == "neutral", "B", "C")
        )
      ) %>%
      mutate(pretreatment = factor(pretreatment,
        levels = c("B", "A", "C")
      )) %>%
      drop_na(),
    family = "gaussian",
```

```
    bf(mass ~ pretreatment + (1 | batch)) +
      bf(tarsus ~ mass + pretreatment + (1 | batch)) +
      bf(bill ~ mass + pretreatment + (1 | batch)) +
      bf(deltaTb ~ mass + tarsus + bill + pretreatment + (1 | batch)) +
      set_rescor(FALSE),
    prior = c(
      set_prior("normal(0, 10)",
        class = "Intercept",
        resp = "mass"
      ),
      set_prior("normal(0, 25)",
        class = "b",
        coef = "pretreatmentA",
        resp = "mass"
      ),
      set_prior("normal(0, 25)",
        class = "b",
        coef = "pretreatmentC",
        resp = "mass"
      ),
      set_prior("exponential(2.5)",
        class = "sd",
        group = "batch",
        resp = "mass"
      ),
      set_prior("exponential(0.15)",
        class = "sigma",
        resp = "mass"
      ),
      set_prior("normal(0, 2.5)",
        class = "Intercept",
        resp = "tarsus"
      ),
      set_prior("normal(0, 2.5)",
        class = "b",
        coef = "pretreatmentA",
        resp = "tarsus"
      ),
      set_prior("normal(0, 2.5)",
        class = "b",
        coef = "pretreatmentC",
        resp = "tarsus"
      ),
      set_prior("skew_normal(0, 0.25, 5)",
        class = "b",
        coef = "mass",
        resp = "tarsus"
      ),
      set_prior("exponential(2)",
        class = "sd",
        group = "batch",
        resp = "tarsus"
      ),
      set_prior("exponential(1)",
        class = "sigma",
        resp = "tarsus"
      ),
      set_prior("normal(0, 1)",
        class = "Intercept",
        resp = "bill"
      ),
      set_prior("normal(0, 0.5)",
        class = "b",
        coef = "pretreatmentA",
        resp = "bill"
      ),
      set_prior("normal(0, 0.5)",
```

```
      class = "b",
      coef = "pretreatmentC",
      resp = "bill"
    ),
    set_prior("skew_normal(0, 0.25, 5)",
      class = "b",
      coef = "mass",
      resp = "bill"
    ),
    set_prior("exponential(5)",
      class = "sd",
      group = "batch",
      resp = "bill"
    ),
    set_prior("exponential(2.5)",
      class = "sigma",
      resp = "bill"
    ),
    set_prior("normal(1, 0.5)",
      class = "Intercept",
      resp = "deltaTb"
    ),
    set_prior("normal(0, 1)",
      class = "b",
      coef = "pretreatmentA",
      resp = "deltaTb"
    ),
    set_prior("normal(0, 1)",
      class = "b",
      coef = "pretreatmentC",
      resp = "deltaTb"
    ),
    set_prior("normal(0, 0.01)",
      class = "b",
      coef = "mass",
      resp = "deltaTb"
    ),
    set_prior("normal(0, 0.1)",
      class = "b",
      coef = "tarsus",
      resp = "deltaTb"
    ),
    set_prior("normal(0, 0.4)",
      class = "b",
      coef = "bill",
      resp = "deltaTb"
    ),
    set_prior("exponential(3.0)",
      class = "sd",
      group = "batch",
      resp = "deltaTb"
    ),
    set_prior("exponential(2.5)",
      class = "sigma",
      resp = "deltaTb"
    )
  ),
  iter = 50000, warmup = 10000, cores = 4, chains = 4, thin = 20,
  control = list(adapt_delta = .98, max_treedepth = 14),
  silent = TRUE, refresh = 0,
  file = "./models/_tbResponseToColdRevised.Rds"
)
```

Divergent transitions are almost entirely resolved. We therefore proceed to assessing: (1) convergence of our HMC chains (here, using Gelman-Rubin statistics), (2) evidence of within chain autocorrelation (here, using the ratio of effective sample sizes to sample sizes), and (3) the capacity of our model to predict true densities and individual measures of body temperature responses to cold.

```
p1 <- mcmc_rhat(rhat(tbResponseCold8WeeksB)) +
  theme(legend.position = "none") +
  xlab(
    TeX('$\\hat{R}$')
  )

p2 <- mcmc_neff(neff_ratio(tbResponseCold8WeeksB), size = 2) +
  theme(legend.position = "none") +
  xlab(
    TeX('$N_{eff}/N~Ratio$')
  )

p3 <- pp_check2(tbResponseCold8WeeksB,
          xlab = "Change in\nBody Temperature (°C)",
          resp = "deltaTb"
          )

p4 <- tbResponseCold8WeeksB$data %>%
  mutate("fit" = fitted(tbResponseCold8WeeksB, resp = "deltaTb",
                        robust = TRUE)[,"Estimate"]) %>%
  ggplot(aes(x = fit, y = deltaTb)) +
  geom_point(size = 2, colour = "black", pch = 21, fill = "grey70") +
  geom_smooth(method = "lm", linetype = "dashed",
              colour = "black", se = FALSE) +
  xlab("Fitted Body Temperature\nResponse (°C)") +
  ylab("True Body Temperature\nResponse (°C)") +
  theme_classic()

(p1 + p2)/(p3 + p4) + plot_annotation(tag_levels = "A")
```

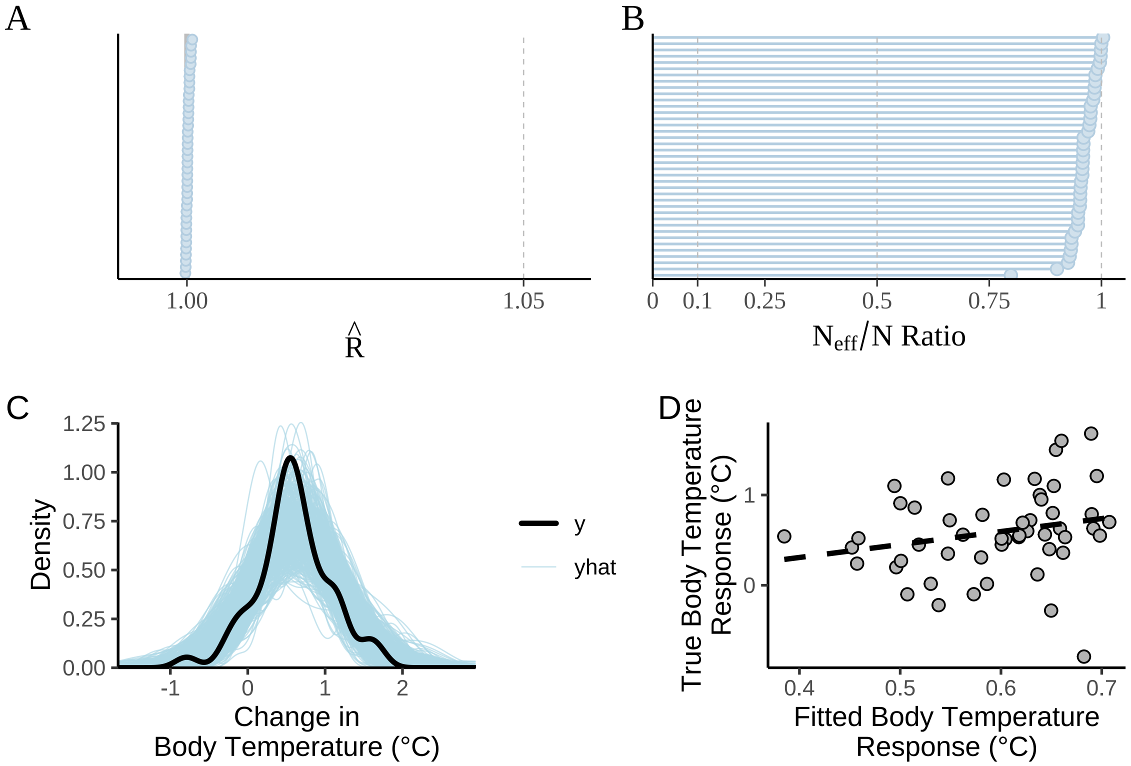

Figure 191: *Validations for a Bayesian path analysis ultimately predicting body temperature responses (°C) to cold (10°C) in eight week old Japanese quail. Baseline body temperatures represent those measured at 30°C. Panel A displays displays Gelman-Rubin statistics for all model parameters. Panel B displays the ratio of effective sample sizes to sample sizes, again, for each model parameter. Panel C displays densities of true (y) and predicted (yhat) values of body temperature responses to cold. Panel D displays true and fitted body temperature responses. The dashed line in panel D represents that line of best fit as estimated by the R package ggplot2 (Wickham 2011).*

Chains have evidently converged and we see no evidence of problematic within-chain autocorrelation. Predictions from our model also reasonably predict true body temperature response values. Next, we visualise model residuals to check for evidence of heteroskedasticity or high-influence measures.

```
g(p1, p2, p3, p4, p5, p6) %=% list(
  ggplot(
    tbResponseCold8WeeksB$data %>%
      mutate(
        "residuals" =
          residuals(tbResponseCold8WeeksB,
            type = "ordinary",
            robust = TRUE,
            resp = "deltaTb"
          )[, "Estimate"]
      ), aes(sample = residuals)
  ) +
    stat_qq(colour = "grey50") +
    stat_qq_line() +
    xlab("Theoretical") +
    ylab("Sample") +
    theme_classic(),

  ggplot(
    tbResponseCold8WeeksB$data %>%
      mutate(
```

```
      "residuals" =
        residuals(tbResponseCold8WeeksB,
          type = "ordinary",
          robust = TRUE,
          resp = "deltaTb"
        )[, "Estimate"],
      "resSE" = residuals(tbResponseCold8WeeksB,
        type = "ordinary",
        robust = TRUE,
        resp = "deltaTb"
      )[, "Est.Error"]
    ),
  aes(x = mass, y = residuals)
) +
  geom_errorbar(
    aes(
      x = mass, ymin = residuals - resSE,
      ymax = residuals + resSE
    ),
    colour = "black", width = 2
  ) +
  geom_point(
    size = 2, pch = 21, colour = "black",
    fill = "grey70"
  ) +
  theme_classic() +
  xlab("Body Mass\n(g; Mean-Centred)") +
  ylab("Ordinary Residuals"),

ggplot(
  tbResponseCold8WeeksB$data %>%
    mutate(
      "residuals" =
        residuals(tbResponseCold8WeeksB,
          type = "ordinary",
          robust = TRUE,
          resp = "deltaTb"
        )[, "Estimate"],
      "resSE" = residuals(tbResponseCold8WeeksB,
        type = "ordinary",
        robust = TRUE,
        resp = "deltaTb"
      )[, "Est.Error"]
    ),
  aes(x = tarsus, y = residuals)
) +
  geom_errorbar(
    aes(
      x = tarsus, ymin = residuals - resSE,
      ymax = residuals + resSE
    ),
    colour = "black", width = 2
  ) +
  geom_point(
    size = 2, pch = 21, colour = "black",
    fill = "grey70"
  ) +
  theme_classic() +
  xlab("Tarsus Length\n(mm; Mean-Centred)") +
  ylab("Ordinary Residuals"),

ggplot(
  tbResponseCold8WeeksB$data %>%
    mutate(
      "residuals" =
        residuals(tbResponseCold8WeeksB,
          type = "ordinary",
```

```
          robust = TRUE,
          resp = "deltaTb"
        )[, "Estimate"],
      "resSE" = residuals(tbResponseCold8WeeksB,
        type = "ordinary",
        robust = TRUE,
        resp = "deltaTb"
      )[, "Est.Error"]
    ),
  aes(x = bill, y = residuals)
) +
  geom_errorbar(
    aes(
      x = bill, ymin = residuals - resSE,
      ymax = residuals + resSE
    ),
    colour = "black", width = 2
  ) +
  geom_point(
    size = 2, pch = 21, colour = "black",
    fill = "grey70"
  ) +
  theme_classic() +
  xlab("Bill Length\n(mm; Mean-Centred)") +
  ylab("Ordinary Residuals"),

ggplot(
  tbResponseCold8WeeksB$data %>%
    mutate(
      "residuals" =
        residuals(tbResponseCold8WeeksB,
          type = "ordinary",
          robust = TRUE,
          resp = "deltaTb"
        )[, "Estimate"],
      pretreatment = ifelse(pretreatment == "B",
        "Mild (20°C)",
        ifelse(pretreatment == "A",
          "Cold (10°C)", "Warm (30°C)"
        )
      )
    ),
  aes(x = pretreatment, y = residuals)
) +
  geom_boxplot(colour = "black", alpha = 0.5, fill = "grey70") +
  geom_point(
    size = 1.5, colour = "black",
    position = position_jitter(width = 0.25)
  ) +
  theme_classic() +
  xlab("Rearing Treatment") +
  ylab("Ordinary Residuals"),

ggplot(
  tbResponseCold8WeeksB$data %>%
    mutate(
      "residuals" =
        residuals(tbResponseCold8WeeksB,
          type = "ordinary",
          robust = TRUE,
          resp = "deltaTb"
        )[, "Estimate"]
    ),
  aes(x = batch, y = residuals, fill = batch)
) +
  geom_boxplot(colour = "black", alpha = 0.5) +
  scale_fill_manual(
```

```
      values = c("black", "grey70"),
      name = "Egg Batch"
    ) +
    theme_classic() +
    xlab("Ordinary Residuals") +
    ylab("Density")
)

(p1 + p2) / (p3 + p4) / (p5 + p6) +
  plot_annotation(tag_levels = "A")
```

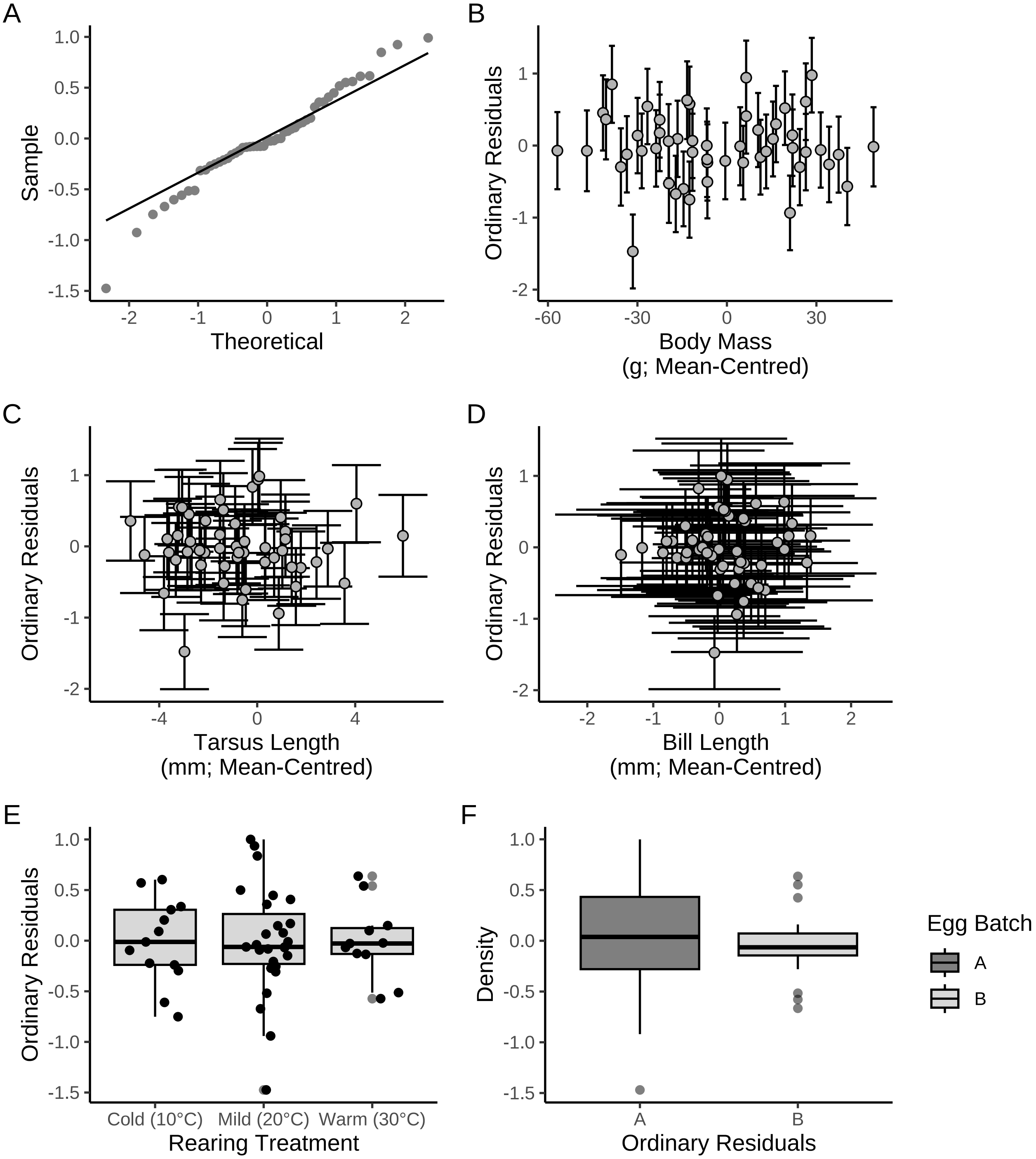

Figure 192: *Ordinary residuals from a Bayesian path analysis ultimately predicting body temperature responses (°C) to cold (10°C) in eight week old Japanese quail. Panel A displays theoretical against sample residuals (qq-plot), panels B-D displays median residuals morphometric predictors, panel E displays median residuals against rearing treatment, and panel F displays median residuals against egg source number (or batch). Errorbars in panels B and D indicate one median absolute deviation around median residuals.*

Heterskedasticity is evident across egg batches. Our path-analysis is therefore reconstructed while allowing the error term for our model predicting body temperature responses to vary by egg batch (here, on the natural log scale). These adjustments are therefore as follows:

$$\epsilon_d \sim e^{\tau_0 + Batch \cdot \tau_1}$$

where “Batch” indicates a Bernoulli value, with “0” representing egg-batch A and “1” representing egg-batch B.

Priors for our error term and batch effect on that error term are conservative and as follows:

$$\tau_0 \sim \mathcal{N}(-3, 1)$$
$$\tau_1 \sim \mathcal{N}(0, 0.25)$$

```
tbResponseCold8WeeksC <-
  brm(
    data = tbResponse %>%
      filter(challenge == "cold") %>%
      mutate(
        mass = mass - mean(mass, na.rm = T),
        tarsus = tarsus - mean(tarsus, na.rm = T),
        bill = bill - mean(bill, na.rm = T),
        pretreatment = ifelse(pretreatment == "cold", "A",
          ifelse(pretreatment == "neutral", "B", "C")
        )
      ) %>%
      mutate(pretreatment = factor(pretreatment,
        levels = c("B", "A", "C")
      )) %>%
      drop_na(),
    family = "gaussian",
    bf(mass ~ pretreatment + (1 | batch)) +
      bf(tarsus ~ mass + pretreatment + (1 | batch)) +
      bf(bill ~ mass + pretreatment + (1 | batch)) +
      bf(deltaTb ~ mass + tarsus + bill + pretreatment + (1 | batch),
         sigma ~ batch) +
      set_rescor(FALSE),
    prior = c(
      set_prior("normal(0, 10)",
        class = "Intercept",
        resp = "mass"
      ),
      set_prior("normal(0, 25)",
        class = "b",
        coef = "pretreatmentA",
        resp = "mass"
      ),
      set_prior("normal(0, 25)",
        class = "b",
        coef = "pretreatmentC",
        resp = "mass"
      ),
      set_prior("exponential(2.5)",
        class = "sd",
        group = "batch",
        resp = "mass"
      ),
      set_prior("exponential(0.15)",
        class = "sigma",
        resp = "mass"
      ),
      set_prior("normal(0, 2.5)",
        class = "Intercept",
        resp = "tarsus"
      ),
      set_prior("normal(0, 2.5)",
        class = "b",
        coef = "pretreatmentA",
        resp = "tarsus"
```

```
    ),
    set_prior("normal(0, 2.5)",
      class = "b",
      coef = "pretreatmentC",
      resp = "tarsus"
    ),
    set_prior("skew_normal(0, 0.25, 5)",
      class = "b",
      coef = "mass",
      resp = "tarsus"
    ),
    set_prior("exponential(2)",
      class = "sd",
      group = "batch",
      resp = "tarsus"
    ),
    set_prior("exponential(1)",
      class = "sigma",
      resp = "tarsus"
    ),
    set_prior("normal(0, 1)",
      class = "Intercept",
      resp = "bill"
    ),
    set_prior("normal(0, 0.5)",
      class = "b",
      coef = "pretreatmentA",
      resp = "bill"
    ),
    set_prior("normal(0, 0.5)",
      class = "b",
      coef = "pretreatmentC",
      resp = "bill"
    ),
    set_prior("skew_normal(0, 0.25, 5)",
      class = "b",
      coef = "mass",
      resp = "bill"
    ),
    set_prior("exponential(5)",
      class = "sd",
      group = "batch",
      resp = "bill"
    ),
    set_prior("exponential(2.5)",
      class = "sigma",
      resp = "bill"
    ),
    set_prior("normal(1, 0.5)",
      class = "Intercept",
      resp = "deltaTb"
    ),
    set_prior("normal(0, 1)",
      class = "b",
      coef = "pretreatmentA",
      resp = "deltaTb"
    ),
    set_prior("normal(0, 1)",
      class = "b",
      coef = "pretreatmentC",
      resp = "deltaTb"
    ),
    set_prior("normal(0, 0.01)",
      class = "b",
      coef = "mass",
      resp = "deltaTb"
    ),
```

```
      set_prior("normal(0, 0.1)",
        class = "b",
        coef = "tarsus",
        resp = "deltaTb"
      ),
      set_prior("normal(0, 0.4)",
        class = "b",
        coef = "bill",
        resp = "deltaTb"
      ),
      set_prior("exponential(3.0)",
        class = "sd",
        group = "batch",
        resp = "deltaTb"
      ),
      set_prior("normal(-3,1)",
        dpar = "sigma",
        class = "Intercept",
        resp = "deltaTb"
      ),
      set_prior("normal(0,0.5)",
        dpar = "sigma",
        class = "b",
        resp = "deltaTb"
      )
    ),
    iter = 50000, warmup = 10000, cores = 4, chains = 4, thin = 20,
    control = list(adapt_delta = .98, max_treedepth = 15),
    silent = TRUE, refresh = 0,
    file = "./models/_tbResponseToColdRevised2.Rds"
  )
```

Model residuals are assessed again below.

```
g(p1, p2, p3, p4, p5, p6) %=% list(
  ggplot(
    tbResponseCold8WeeksC$data %>%
      mutate(
        "residuals" =
          residuals(tbResponseCold8WeeksC,
            type = "pearson",
            robust = TRUE,
            resp = "deltaTb"
          )[, "Estimate"]
      ), aes(sample = residuals)
  ) +
    stat_qq(colour = "grey50") +
    stat_qq_line() +
    xlab("Theoretical") +
    ylab("Sample") +
    theme_classic(),

  ggplot(
    tbResponseCold8WeeksC$data %>%
      mutate(
        "residuals" =
          residuals(tbResponseCold8WeeksC,
            type = "pearson",
            robust = TRUE,
            resp = "deltaTb"
          )[, "Estimate"],
        "resSE" = residuals(tbResponseCold8WeeksC,
          type = "pearson",
          robust = TRUE,
          resp = "deltaTb"
        )[, "Est.Error"]
      ),
```

```
    aes(x = mass, y = residuals)
  ) +
    geom_errorbar(
      aes(
        x = mass, ymin = residuals - resSE,
        ymax = residuals + resSE
      ),
      colour = "black", width = 2
    ) +
    geom_point(
      size = 2, pch = 21, colour = "black",
      fill = "grey70"
    ) +
    theme_classic() +
    xlab("Body Mass\n(g; Mean-Centred)") +
    ylab("Pearson Residuals"),

  ggplot(
    tbResponseCold8WeeksC$data %>%
      mutate(
        "residuals" =
          residuals(tbResponseCold8WeeksC,
            type = "pearson",
            robust = TRUE,
            resp = "deltaTb"
          )[, "Estimate"],
        "resSE" = residuals(tbResponseCold8WeeksC,
          type = "pearson",
          robust = TRUE,
          resp = "deltaTb"
        )[, "Est.Error"]
      ),
    aes(x = tarsus, y = residuals)
  ) +
    geom_errorbar(
      aes(
        x = tarsus, ymin = residuals - resSE,
        ymax = residuals + resSE
      ),
      colour = "black", width = 2
    ) +
    geom_point(
      size = 2, pch = 21, colour = "black",
      fill = "grey70"
    ) +
    theme_classic() +
    xlab("Tarsus Length\n(mm; Mean-Centred)") +
    ylab("Pearson Residuals"),

  ggplot(
    tbResponseCold8WeeksC$data %>%
      mutate(
        "residuals" =
          residuals(tbResponseCold8WeeksC,
            type = "pearson",
            robust = TRUE,
            resp = "deltaTb"
          )[, "Estimate"],
        "resSE" = residuals(tbResponseCold8WeeksC,
          type = "pearson",
          robust = TRUE,
          resp = "deltaTb"
        )[, "Est.Error"]
      ),
    aes(x = bill, y = residuals)
  ) +
    geom_errorbar(
```

```
      aes(
        x = bill, ymin = residuals - resSE,
        ymax = residuals + resSE
      ),
      colour = "black", width = 2
    ) +
    geom_point(
      size = 2, pch = 21, colour = "black",
      fill = "grey70"
    ) +
    theme_classic() +
    xlab("Bill Length\n(mm; Mean-Centred)") +
    ylab("Pearson Residuals"),

  ggplot(
    tbResponseCold8WeeksC$data %>%
      mutate(
        "residuals" =
          residuals(tbResponseCold8WeeksC,
            type = "pearson",
            robust = TRUE,
            resp = "deltaTb"
          )[, "Estimate"],
        pretreatment = ifelse(pretreatment == "B",
          "Mild (20°C)",
          ifelse(pretreatment == "A",
            "Cold (10°C)", "Warm (30°C)"
          )
        )
      ),
    aes(x = pretreatment, y = residuals)
  ) +
    geom_boxplot(colour = "black", alpha = 0.5, fill = "grey70") +
    geom_point(
      size = 1.5, colour = "black",
      position = position_jitter(width = 0.25)
    ) +
    theme_classic() +
    xlab("Rearing Treatment") +
    ylab("Pearson Residuals"),

  ggplot(
    tbResponseCold8WeeksC$data %>%
      mutate(
        "residuals" =
          residuals(tbResponseCold8WeeksC,
            type = "pearson",
            robust = TRUE,
            resp = "deltaTb"
          )[, "Estimate"]
      ),
    aes(x = batch, y = residuals, fill = batch)
  ) +
    geom_boxplot(colour = "black", alpha = 0.5) +
    scale_fill_manual(
      values = c("black", "grey70"),
      name = "Egg Batch"
    ) +
    theme_classic() +
    xlab("Pearson Residuals") +
    ylab("Density")
)

(p1 + p2) / (p3 + p4) / (p5 + p6) +
  plot_annotation(tag_levels = "A")
```

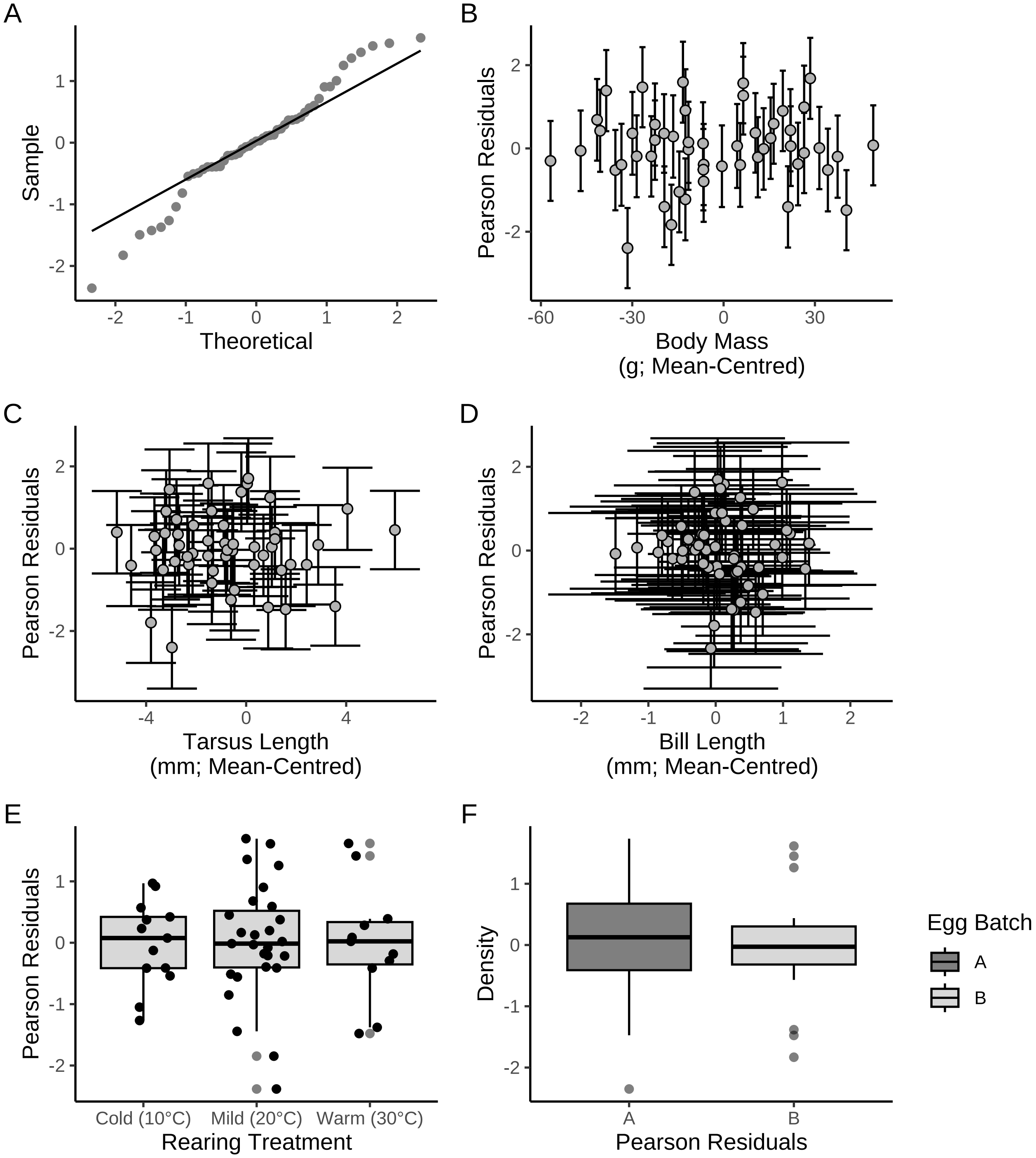

Figure 193: *Pearson residuals from a Bayesian path analysis ultimately predicting body temperature responses (°C) to cold (10°C) in eight week old Japanese quail. Here, error around body temperature responses to cold are allowed to vary by bird origin (egg batch). Panel A displays theoretical against sample residuals (qq-plot), panels B-D displays median residuals morphometric predictors, panel E displays median residuals against rearing treatment, and panel F displays median residuals against egg source number (or batch). Errorbars in panels B and C indicate one median absolute deviation around median residuals.*

Variance in residuals is now more balanced between egg batches. We proceed by calculating Bayesian R^2 for models within our path analysis, then plotting posterior coefficients to check for evidence of skewing.

```r
# Renaming model for simplicity

tbResponseCold8Weeks <- tbResponseCold8WeeksC

# Checking R2 values

caption <- paste0(
  "R\\textsuperscript{2} for Bayesian path analysis ",
  "ultimate predicting body temperature responses (°C) to cold ",
  "(10°C) in eight week old Japanese quail. Baseline body ",
  "temperatures represent those measured at thermoneutrality ",
  "(30°C)"
)

brms::bayes_R2(tbResponseCold8Weeks,
  robust = TRUE, ndraws = 1000
) %>%
  as.data.frame() %>%
  rownames_to_column(var = "Response") %>%
  left_join(., tribble(~Response, ~response,
                      "R2mass", "Body Mass (g)",
                      "R2tarsus", "Tarsus Length (mm)",
                      "R2bill", "Bill Length (mm)",
                      "R2deltaTb", "Change in Tb (°C)"),
            by = "Response") %>%
  dplyr::select(-Response) %>%
  mutate(
    Estimate = round(Estimate, digits = 4),
    Est.Error = round(Est.Error, digits = 4),
    Q2.5 = round(Q2.5, digits = 4),
    Q97.5 = round(Q97.5, digits = 4)
  ) %>%
  dplyr::select(
    "Response" = response,
    "R\\textsuperscript{2}" = Estimate,
    "Standard Error" = Est.Error,
    `2.5\\%CI` = "Q2.5", `97.5\\% CI` = "Q97.5"
  ) %>%
  kbl(.,
    longtable = T, booktabs = T, format = "latex",
    caption = caption, escape = FALSE
  ) %>%
  kable_styling(latex_options = "striped")
```

Table 116: *R^2 for Bayesian path analysis ultimate predicting body temperature responses (°C) to cold (10°C) in eight week old Japanese quail. Baseline body temperatures represent those measured at thermoneutrality (30°C)*

Response	R^2	Standard Error	2.5%CI	97.5% CI
Body Mass (g)	0.0248	0.0234	0.0015	0.1022
Tarsus Length (mm)	0.3217	0.0805	0.1380	0.4597
Bill Length (mm)	0.0594	0.0419	0.0069	0.1791
Change in Tb (°C)	0.0830	0.0452	0.0214	0.1993

```r
as.data.frame(tbResponseCold8Weeks) %>%
    dplyr::select(contains("b_deltaTb"), contains("__deltaTb_Intercept")) %>%
    pivot_longer(everything(), names_to = "Par",
                 values_to = "Coefs") %>%
    left_join(., tribble(~Par, ~par, ~Order,
                        "b_deltaTb_Intercept", "Intercept", "A",
                        "b_deltaTb_mass", "Body Mass (g)", "B",
                        "b_deltaTb_tarsus", "Tarsus Length\n(mm)", "C",
                        "b_deltaTb_bill", "Bill Length\n(mm)", "D",
                        "b_deltaTb_pretreatmentA", "Cold Rearing\n(10°C)", "E",
                        "b_deltaTb_pretreatmentC", "Warm Rearing\n(30°C)", "F",
```

```
                          "sd_batch__deltaTb_Intercept", "Egg Batch (mu)", "G"
                          ),
              by = "Par")  %>%
    dplyr::select("Par" = par, Coefs, Order) %>%
    arrange(Order) %>%
    mutate(Par = factor(Par, levels = unique(Par))) %>%
    ggplot(aes(x = Coefs)) +
    facet_wrap(~Par, scales = "free", ncol = 2) +
    geom_density(colour = "black", alpha = 0.5, fill = "grey70") +
    geom_vline(xintercept = 0, linetype = "dashed", colour = "black") +
    ylab("Density") +
    theme_classic() +
    theme(axis.title.x = element_blank())
```

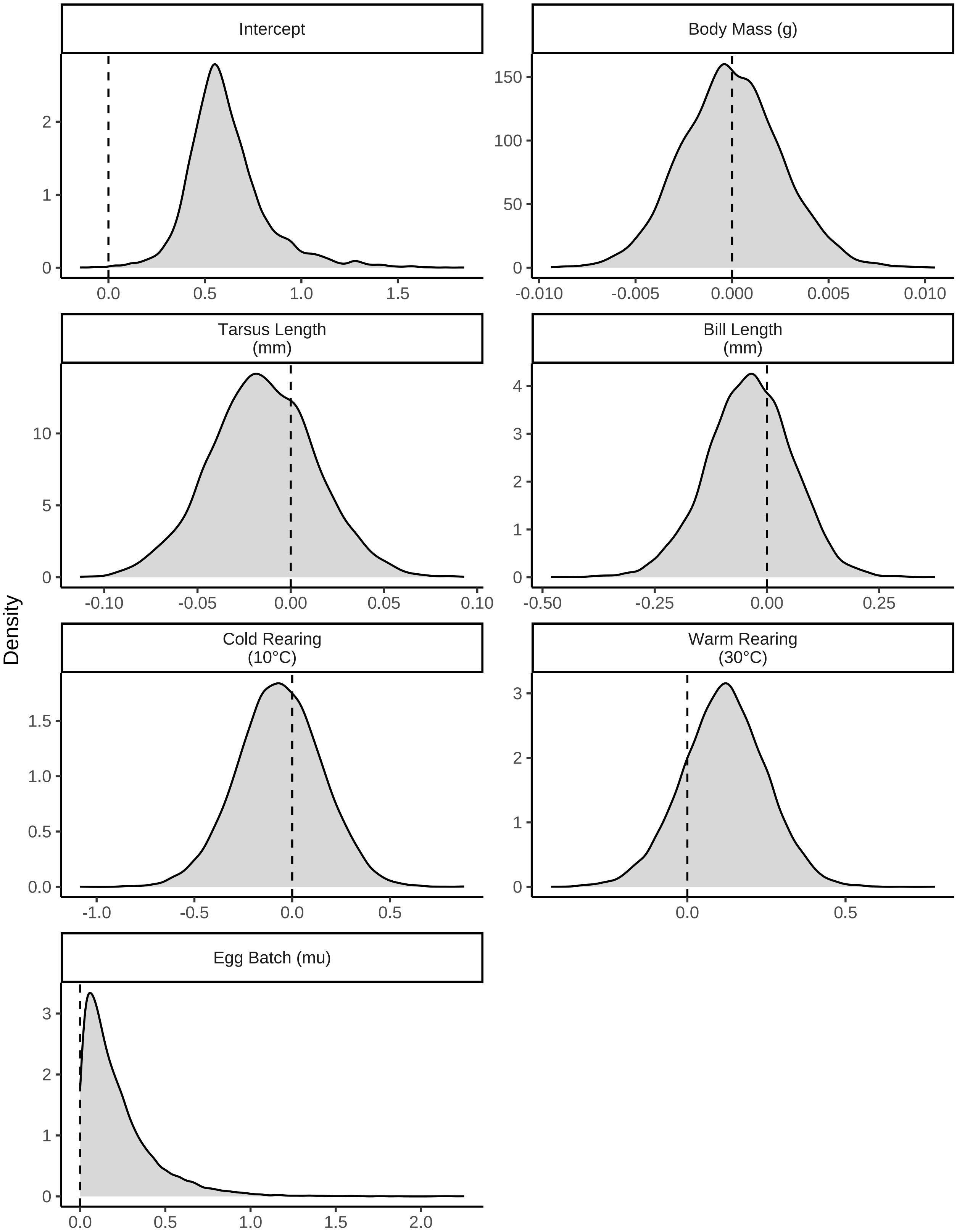

Figure 194: *Posterior densities for model coefficients derived from a Bayesian path analysis ultimately predicting body temperature responses (°C) to cold (10°C) in eight week old Japanese quail. Densities are split by their respective response variables (indicated with titles). Vertical dashed lines indicate 0.*

```
# Subtle skewing in intercept
```

We now summarise estimates from our path analysis, with central estimates of our coefficients being calculated as their medians and credible intervals as quantile intervals around medians.

```
caption <- paste0(
  "Results from a Bayesian path analysis ",
  "ultimately predicting body temperature responses to a ",
  "cold exposure (10°C) relative to thermoneutrality (30°C)",
  "in adult Japanese quail. Body temperature responses are ",
  "predicted as a function of body mass (g), tarsus length (mm), bill length ",
  "(mm) and rearing treatment. ",
  "Cold rearing indicates post-hatch rearing at ",
  "10°C, relative to ",
  "20°C (intercept), or ",
  "30°C ('warm rearing'). ",
  "Coefficients represent medians and credible intervals ",
  "(CIs) represent quantile intervals. ",
  "BF indicates Bayes Factors."
)

as.data.frame(tbResponseCold8Weeks) %>%
  summarise_all(., .funs = median) %>%
  pivot_longer(everything(),
    names_to = "Parameter",
    values_to = "Estimate"
  ) %>%
  merge(., quantileCIs(tbResponseCold8Weeks, cis = c(50, 95)),
    by = "Parameter", all.x = TRUE
  ) %>%
  filter(grepl("b_|sd_", Parameter)) %>%
  rowwise() %>%
  mutate("BF" = ifelse(Estimate < 0,
    (2 * mean(as.data.frame(
      tbResponseCold8Weeks
    )[, Parameter] <= 0)) /
      (2 * mean(as.data.frame(
        tbResponseCold8Weeks
      )[, Parameter] >= 0)),
    (2 * mean(as.data.frame(
      tbResponseCold8Weeks
    )[, Parameter] >= 0)) /
      (2 * mean(as.data.frame(
        tbResponseCold8Weeks
      )[, Parameter] <= 0))
  )) %>%
  ungroup() %>%
  mutate(
    "Estimate" = round(Estimate, digits = 4),
    "BF" = round(BF, digits = 4),
    "N" = nrow(tbResponseCold8Weeks$data)
  ) %>%
  rowwise() %>%
  mutate(Parameter = gsub("deltaTb", "deltaT", Parameter)) %>%
  ungroup() %>%
  mutate("Parameter" = ifelse(grepl("b_", Parameter),
                             gsub("b_", "", Parameter),
                             gsub("Intercept", "batch",
                                  gsub(".*__", "", Parameter)
                                  )
                             )
         ) %>%
  mutate(
    "Response" = gsub("_.*", "", Parameter),
    "Parameter" = gsub(".*_", "", Parameter)
  ) %>%
```

```
merge(., tribble(
  ~Response, ~response, ~level,
  "mass", "Body Mass (g)", "A",
  "tarsus", "Tarsus Length (mm)", "B",
  "bill", "Bill Length (mm)", "C",
  "deltaT", "Delta Body Temperature (°C)", "D"
),
by = "Response"
) %>%
merge(., tribble(
  ~Parameter, ~parameter, ~number,
  "Intercept", "Intercept", "1",
  "mass", "Body Mass (g)", "4",
  "tarsus", "Tarsus Length (mm)", "5",
  "bill", "Bill Length (mm)", "6",
  "pretreatmentA", "Cold Rearing", "2",
  "pretreatmentC", "Warm Rearing", "3",
  "batch", "Egg Batch [mu]", "7"
),
by = "Parameter"
) %>%
mutate(
  `50\\% CI` = paste0("(", paste(
    round(Low_CI_50, digits = 4),
    round(High_CI_50, digits = 4),
    sep = ", "
  ), ")"),
  `95\\% CI` = paste0("(", paste(
    round(Low_CI_95, digits = 4),
    round(High_CI_95, digits = 4),
    sep = ", "
  ), ")")
) %>%
dplyr::select(-c(Low_CI_50, High_CI_50, Low_CI_95, High_CI_95)) %>%
dplyr::select(
  "Response" = "response", "Parameter" = "parameter", N,
  Estimate, `50\\% CI`, `95\\% CI`, BF, level, number
) %>%
arrange(level, number) %>%
dplyr::select(-c(level, number)) %>%
kbl(.,
  longtable = T, booktabs = T, format = "latex", escape = FALSE,
  caption = caption
) %>%
column_spec(column = c(1:2), width = "2.2cm") %>%
column_spec(column = c(3:10), width = "1.9cm") %>%
kable_styling(latex_options = "striped")
```

Table 117: *Results from a Bayesian path analysis ultimately predicting body temperature responses to a cold exposure (10°C) relative to thermoneutrality (30°C)in adult Japanese quail. Body temperature responses are predicted as a function of body mass (g), tarsus length (mm), bill length (mm) and rearing treatment. Cold rearing indicates post-hatch rearing at 10°C, relative to 20°C (intercept), or 30°C ('warm rearing'). Coefficients represent medians and credible intervals (CIs) represent quantile intervals. BF indicates Bayes Factors.*

Response	Parameter	N	Estimate	50% CI	95% CI	BF
Body Mass (g)	Intercept	51	-3.7283	(-6.8731, -0.642)	(-12.6167, 5.3554)	3.8193
Body Mass (g)	Cold Rearing	51	2.1974	(-3.3727, 7.745)	(-13.6806, 18.2863)	1.5357
Body Mass (g)	Warm Rearing	51	-0.3593	(-5.9455, 5.562)	(-16.8906, 16.6593)	1.0774
Body Mass (g)	Egg Batch [mu]	51	0.2741	(0.116, 0.5397)	(0.0104, 1.4247)	Inf

Tarsus Length (mm)	Intercept	51	-0.7187	(-1.0211, -0.4137)	(-1.7178, 0.3444)	13.7601
Tarsus Length (mm)	Cold Rearing	51	0.4360	(-0.0107, 0.8799)	(-0.9115, 1.7733)	2.8929
Tarsus Length (mm)	Warm Rearing	51	0.0611	(-0.3997, 0.537)	(-1.348, 1.4879)	1.1534
Tarsus Length (mm)	Body Mass (g)	51	0.0497	(0.0424, 0.057)	(0.0275, 0.0715)	Inf
Tarsus Length (mm)	Egg Batch [mu]	51	0.2497	(0.1066, 0.4951)	(0.0097, 1.338)	Inf
Bill Length (mm)	Intercept	51	0.0949	(-0.0104, 0.2008)	(-0.2679, 0.4491)	2.6680
Bill Length (mm)	Cold Rearing	51	0.0375	(-0.1033, 0.1778)	(-0.3755, 0.4366)	1.3371
Bill Length (mm)	Warm Rearing	51	-0.0413	(-0.1801, 0.1048)	(-0.4585, 0.3776)	1.3606
Bill Length (mm)	Body Mass (g)	51	-0.0005	(-0.0029, 0.0018)	(-0.0071, 0.0062)	1.2734
Bill Length (mm)	Egg Batch [mu]	51	0.1148	(0.0508, 0.2161)	(0.0046, 0.5452)	Inf
Delta Body Temperature (°C)	Intercept	51	0.5802	(0.4868, 0.6978)	(0.2839, 1.1269)	999.0000
Delta Body Temperature (°C)	Cold Rearing	51	-0.0637	(-0.2058, 0.0792)	(-0.4919, 0.3514)	1.6195
Delta Body Temperature (°C)	Warm Rearing	51	0.1167	(0.0296, 0.2034)	(-0.1514, 0.3722)	4.3191
Delta Body Temperature (°C)	Body Mass (g)	51	-0.0001	(-0.0018, 0.0016)	(-0.005, 0.0051)	1.0571
Delta Body Temperature (°C)	Tarsus Length (mm)	51	-0.0153	(-0.0338, 0.004)	(-0.0711, 0.0413)	2.3361
Delta Body Temperature (°C)	Bill Length (mm)	51	-0.0370	(-0.1001, 0.0253)	(-0.2287, 0.1428)	1.8767
Delta Body Temperature (°C)	Egg Batch [mu]	51	0.1579	(0.0721, 0.2977)	(0.0062, 0.8056)	Inf

Analysing body temperature responses to heat

Unlike metabolic responses to cold exposure, we found that metabolic responses to heat exposure (40°C) were indeed influenced by body size and appendage length among adult Japanese quail. Specifically, adult quail with relatively large body sizes and short tarsi displayed larger increases in metabolism during a heat exposure than those with relatively small body sizes and long tarsi. However, the magnitude of these effects were small, limiting their biological implications. Beyond these effects, however, it is still possible that body size and tarsus length also influenced how body temperature responded to heat exposure, potentially increasing the biological implications of being large with atypically short appendages in the heat. To test this, we again evaluated whether body temperature responses to a heat exposure varied by body mass, tarsus length and bill length, while controlling for prior temperature exposure. This was achieved by quantifying the mean change in body temperature displayed between that measured in the heat (40°C) and that measured at thermoneutrality (30°C), then modelling this change as a function of body mass, tarsus length, bill length, and rearing condition. Given that rearing condition also influences body mass and appendage length in our quail (Tabh et al 2025), however, this model was nested with a path analysis identical to that described for body temperature responses to the cold above, but with body temperature responses to heat (°C) as the ultimate response variable.

For this analysis, priors for our models predicting body mass, tarsus length and bill length on their own

remained the same as those described previously (i.e. for our path analysis predicting body temperature responses to the cold). Priors for our model predicting body temperature responses to heat, however, differed slightly, but were equally informed by Persson et al (2024) and the observed ranges of body temperature responses and morphometric measurements. For the effects of body mass and both tarsus and bill length on body temperature responses, we again assumed that effects larger than the range of body temperature responses divided by the range of the given morphological variable were unlikely (standard deviation of a normally-distributed prior $= 0.5 \cdot \frac{Range[Delta\ Tb]}{Range[Mass,\ Tarsus,\ Bill]}$). Priors for our model predicting body temperature responses to heat were therefore as follows:

$$\beta_{d0} \sim \mathcal{N}(1, 1)$$
$$\beta_{d1} \sim \mathcal{N}(0, 1)$$
$$\beta_{d2} \sim \mathcal{N}(0, 1)$$
$$\beta_{d3} \sim \mathcal{N}(0, 0.01)$$
$$\beta_{d4} \sim \mathcal{N}(0, 0.15)$$
$$\beta_{d5} \sim \mathcal{N}(0, 0.4)$$
$$\mu 0d \sim \exp(2.5)$$
$$\epsilon_d \sim \exp(2.5)$$

Before constructing our path analysis, we check raw body temperature response values for oddities, then assess suitability of our priors using a prior predictive check (as described above).

```
tbResponse %>%
  filter(!is.na(deltaTb) & challenge == "warm") %>%
  ggplot(aes(x = 1:nrow(.), y = deltaTb)) +
  geom_rect(aes(xmin = -Inf, xmax = Inf,
                ymin = mean(deltaTb) - 3.5*sd(deltaTb),
                ymax = mean(deltaTb) + 3.5*sd(deltaTb)
                ),
            fill = "grey80", alpha = 0.5, colour = "black") +
  geom_hline(yintercept = 0, linetype = "dashed", colour = "black") +
  geom_point(size = 2, colour = "black", alpha = 0.7) +
  scale_y_continuous(sec.axis = sec_axis( trans=~., name="Second Axis",
                                          breaks = c(1.5, -0.2),
                                          labels = c("Increasing Tb",
                                                     "Decreasing Tb"))
                     ) +
  theme_classic() +
  theme(axis.text.y.right = element_text(angle = 270),
        axis.ticks.y.right = element_blank(),
        axis.title.y.right = element_blank()) +
  xlab("Sample Number") +
  ylab("Change in\nBody Temperature (°C)")
```

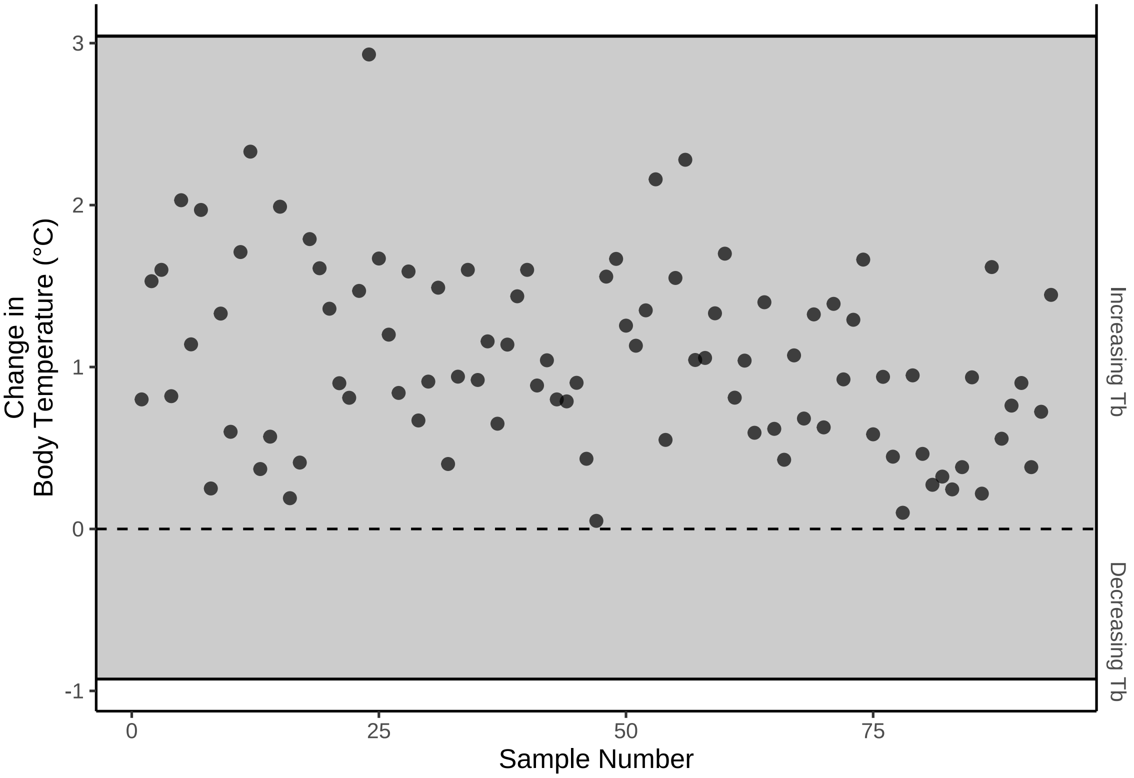

Figure 195: *Cleveland dotplot of mean changes in body temperature (°C) between 40°C and 30°C, each drawn from adult Japanese quail. Again, dots represent raw data points and rectangles indicate means 3.5 times the standard deviation at a given ambient temperature. The dashed line indicates no change in body temperature.*

```
tbResponseHot8WeeksPPCheck <-
  brm(
    data = tbResponse %>%
      filter(week == "8" & challenge == "warm") %>%
      mutate(
        mass = mass - mean(mass, na.rm = T),
        tarsus = tarsus - mean(tarsus, na.rm = T),
        bill = bill - mean(bill, na.rm = T),
        pretreatment = ifelse(pretreatment == "cold", "A",
          ifelse(pretreatment == "neutral", "B", "C")
        )
      ) %>%
      mutate(pretreatment = factor(pretreatment,
                                   levels = c("B", "A", "C"))
             ) %>%
      drop_na(),
    family = "gaussian",
    bf(mass ~ pretreatment + (1 | batch)) +
      bf(tarsus ~ mass + pretreatment + (1 | batch)) +
      bf(bill ~ mass + pretreatment + (1 | batch)) +
      bf(deltaTb ~ mass + tarsus + bill + pretreatment + (1 | batch)) +
      set_rescor(FALSE),
    prior = c(
      set_prior("normal(0, 10)",
        class = "Intercept",
        resp = "mass"
      ),
      set_prior("normal(0, 25)",
        class = "b",
        coef = "pretreatmentA",
```

```
      resp = "mass"
    ),
    set_prior("normal(0, 25)",
      class = "b",
      coef = "pretreatmentC",
      resp = "mass"
    ),
    set_prior("exponential(2.5)",
      class = "sd",
      group = "batch",
      resp = "mass"
    ),
    set_prior("exponential(0.15)",
      class = "sigma",
      resp = "mass"
    ),
    set_prior("normal(0, 2.5)",
      class = "Intercept",
      resp = "tarsus"
    ),
    set_prior("normal(0, 2.5)",
      class = "b",
      coef = "pretreatmentA",
      resp = "tarsus"
    ),
    set_prior("normal(0, 2.5)",
      class = "b",
      coef = "pretreatmentC",
      resp = "tarsus"
    ),
    set_prior("skew_normal(0, 0.25, 5)",
      class = "b",
      coef = "mass",
      resp = "tarsus"
    ),
    set_prior("exponential(2)",
      class = "sd",
      group = "batch",
      resp = "tarsus"
    ),
    set_prior("exponential(1)",
      class = "sigma",
      resp = "tarsus"
    ),
    set_prior("normal(0, 1)",
      class = "Intercept",
      resp = "bill"
    ),
    set_prior("normal(0, 0.5)",
      class = "b",
      coef = "pretreatmentA",
      resp = "bill"
    ),
    set_prior("normal(0, 0.5)",
      class = "b",
      coef = "pretreatmentC",
      resp = "bill"
    ),
    set_prior("skew_normal(0, 0.25, 5)",
      class = "b",
      coef = "mass",
      resp = "bill"
    ),
    set_prior("exponential(5)",
      class = "sd",
      group = "batch",
      resp = "bill"
```

```
      ),
      set_prior("exponential(2.5)",
        class = "sigma",
        resp = "bill"
      ),
      set_prior("normal(1, 1)",
        class = "Intercept",
        resp = "deltaTb"
      ),
      set_prior("normal(0, 1)",
        class = "b",
        coef = "pretreatmentA",
        resp = "deltaTb"
      ),
      set_prior("normal(0, 1)",
        class = "b",
        coef = "pretreatmentC",
        resp = "deltaTb"
      ),
      set_prior("normal(0, 0.01)",
        class = "b",
        coef = "mass",
        resp = "deltaTb"
      ),
      set_prior("normal(0, 0.15)",
        class = "b",
        coef = "tarsus",
        resp = "deltaTb"
      ),
      set_prior("normal(0, 0.4)",
        class = "b",
        coef = "bill",
        resp = "deltaTb"
      ),
      set_prior("exponential(2.5)",
        class = "sd",
        group = "batch",
        resp = "deltaTb"
      ),
      set_prior("exponential(2.5)",
        class = "sigma",
        resp = "deltaTb"
      )
    ),
    iter = 50000, warmup = 10000, cores = 4, chains = 4, thin = 20,
    control = list(adapt_delta = .98, max_treedepth = 14),
    silent = TRUE, refresh = 0,
    sample_prior = "only",
    file = "./models/_bodyTemperatureResponsesToHeatPPCheck.Rds"
  )

pp_check2(tbResponseHot8WeeksPPCheck,
          xlab = "Change in\nBody Temperature (°C)",
          resp = "deltaTb"
          )
```

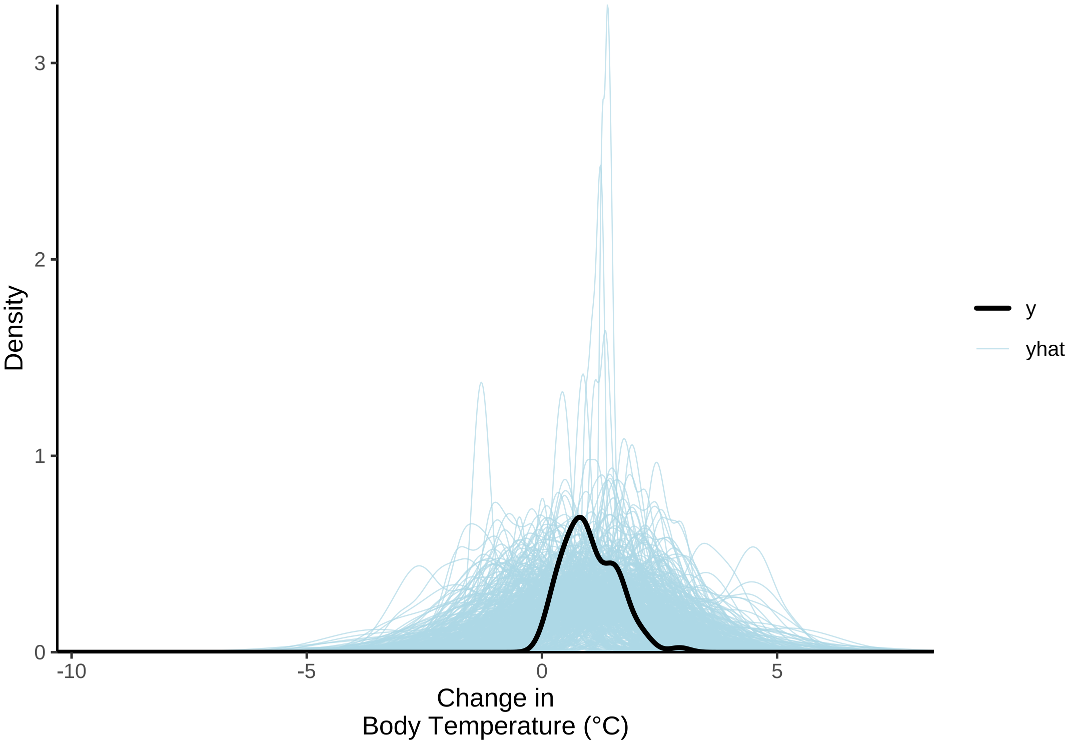

Figure 196: *Overlay of predicted (blue) and true (black) body temperature response (°C) densities, where predicted densities are derived from priors in a Bayesian path analyses. Clear overlap betwen the black and blue lines suggests that model priors are reasonable with respect to the data.*

Priors appear suitable. Our full analysis is therefore constructed and chains checked for convergence and evidence of autocorrelation. Further, the capacity of analysis to predict true body temperature responses to heat is visually assessed.

```
tbResponseHot8Weeks <-
  brm(
    data = tbResponse %>%
      filter(week == "8" & challenge == "warm") %>%
      mutate(
        mass = mass - mean(mass, na.rm = T),
        tarsus = tarsus - mean(tarsus, na.rm = T),
        bill = bill - mean(bill, na.rm = T),
        pretreatment = ifelse(pretreatment == "cold", "A",
          ifelse(pretreatment == "neutral", "B", "C")
        )
      ) %>%
      mutate(pretreatment = factor(pretreatment,
                                   levels = c("B", "A", "C"))
             ) %>%
      drop_na(),
    family = "gaussian",
    bf(mass ~ pretreatment + (1 | batch)) +
      bf(tarsus ~ mass + pretreatment + (1 | batch)) +
      bf(bill ~ mass + pretreatment + (1 | batch)) +
      bf(deltaTb ~ mass + tarsus + bill + pretreatment + (1 | batch)) +
      set_rescor(FALSE),
    prior = c(
      set_prior("normal(0, 10)",
        class = "Intercept",
        resp = "mass"
      ),
```

```
    set_prior("normal(0, 25)",
      class = "b",
      coef = "pretreatmentA",
      resp = "mass"
    ),
    set_prior("normal(0, 25)",
      class = "b",
      coef = "pretreatmentC",
      resp = "mass"
    ),
    set_prior("exponential(2.5)",
      class = "sd",
      group = "batch",
      resp = "mass"
    ),
    set_prior("exponential(0.15)",
      class = "sigma",
      resp = "mass"
    ),
    set_prior("normal(0, 2.5)",
      class = "Intercept",
      resp = "tarsus"
    ),
    set_prior("normal(0, 2.5)",
      class = "b",
      coef = "pretreatmentA",
      resp = "tarsus"
    ),
    set_prior("normal(0, 2.5)",
      class = "b",
      coef = "pretreatmentC",
      resp = "tarsus"
    ),
    set_prior("skew_normal(0, 0.25, 5)",
      class = "b",
      coef = "mass",
      resp = "tarsus"
    ),
    set_prior("exponential(2)",
      class = "sd",
      group = "batch",
      resp = "tarsus"
    ),
    set_prior("exponential(1)",
      class = "sigma",
      resp = "tarsus"
    ),
    set_prior("normal(0, 1)",
      class = "Intercept",
      resp = "bill"
    ),
    set_prior("normal(0, 0.5)",
      class = "b",
      coef = "pretreatmentA",
      resp = "bill"
    ),
    set_prior("normal(0, 0.5)",
      class = "b",
      coef = "pretreatmentC",
      resp = "bill"
    ),
    set_prior("skew_normal(0, 0.25, 5)",
      class = "b",
      coef = "mass",
      resp = "bill"
    ),
    set_prior("exponential(5)",
```

```
        class = "sd",
        group = "batch",
        resp = "bill"
      ),
      set_prior("exponential(2.5)",
        class = "sigma",
        resp = "bill"
      ),
      set_prior("normal(1, 1)",
        class = "Intercept",
        resp = "deltaTb"
      ),
      set_prior("normal(0, 1)",
        class = "b",
        coef = "pretreatmentA",
        resp = "deltaTb"
      ),
      set_prior("normal(0, 1)",
        class = "b",
        coef = "pretreatmentC",
        resp = "deltaTb"
      ),
      set_prior("normal(0, 0.01)",
        class = "b",
        coef = "mass",
        resp = "deltaTb"
      ),
      set_prior("normal(0, 0.15)",
        class = "b",
        coef = "tarsus",
        resp = "deltaTb"
      ),
      set_prior("normal(0, 0.4)",
        class = "b",
        coef = "bill",
        resp = "deltaTb"
      ),
      set_prior("exponential(2.5)",
        class = "sd",
        group = "batch",
        resp = "deltaTb"
      ),
      set_prior("exponential(2.5)",
        class = "sigma",
        resp = "deltaTb"
      )
    ),
    iter = 50000, warmup = 10000, cores = 4, chains = 4, thin = 20,
    control = list(adapt_delta = .98, max_treedepth = 14),
    silent = TRUE, refresh = 0,
    file = "./models/_bodyTemperatureResponsesToHeat.Rds"
  )

p1 <- mcmc_rhat(rhat(tbResponseHot8Weeks)) +
  theme(legend.position = "none") +
  xlab(
    TeX('$\\hat{R}$')
  )

p2 <- mcmc_neff(neff_ratio(tbResponseHot8Weeks), size = 2) +
  theme(legend.position = "none") +
  xlab(
    TeX('$N_{eff}/N~Ratio$')
  )

p3 <- pp_check2(tbResponseHot8Weeks,
          xlab = "Change in\nBody Temperature (°C)",
```

```
          resp = "deltaTb"
          )
p4 <- tbResponseHot8Weeks$data %>%
  mutate("fit" = fitted(tbResponseHot8Weeks, resp = "deltaTb",
                        robust = TRUE)[,"Estimate"]) %>%
  ggplot(aes(x = fit, y = deltaTb)) +
  geom_point(size = 2, colour = "black", pch = 21, fill = "grey70") +
  geom_smooth(method = "lm", linetype = "dashed",
              colour = "black", se = FALSE) +
  xlab("Fitted Body Temperature\nResponse (°C)") +
  ylab("True Body Temperature\nResponse (°C)") +
  theme_classic()

(p1 + p2)/(p3 + p4) + plot_annotation(tag_levels = "A")
```

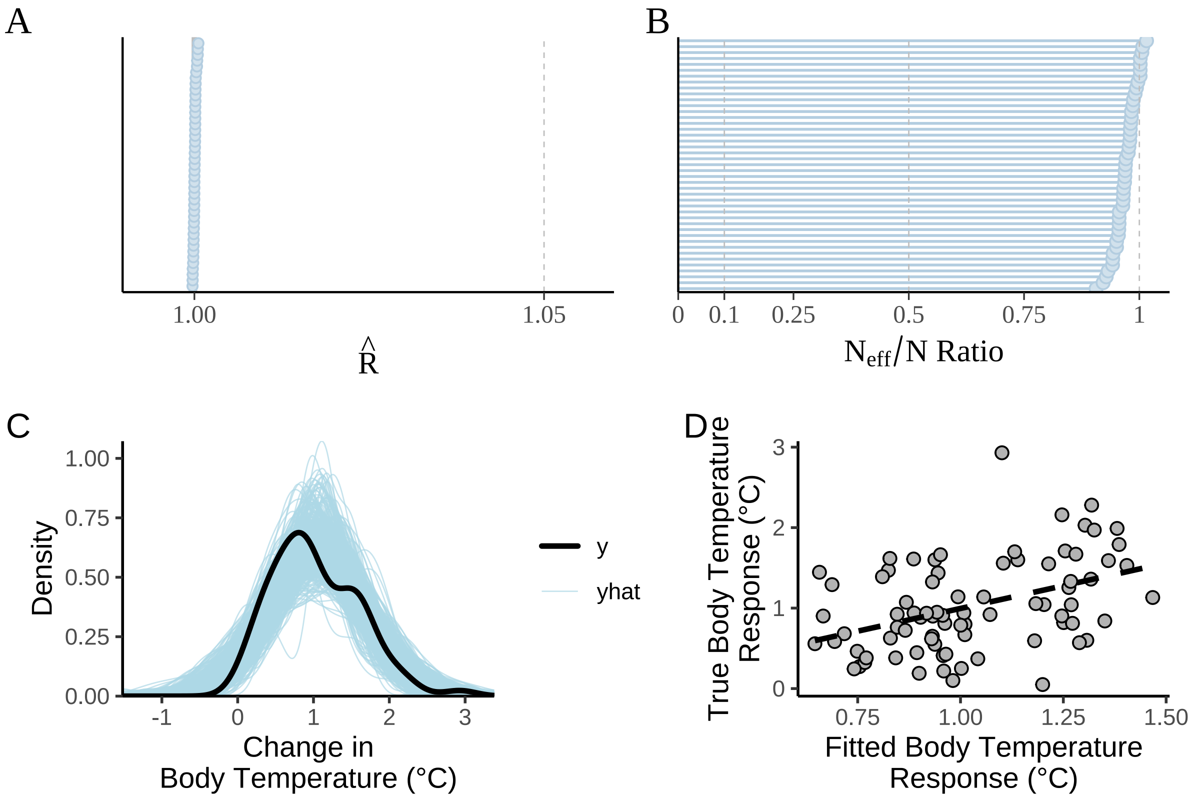

Figure 197: *Validations for a Bayesian path analysis ultimately predicting body temperature responses (°C) to heat (40°C) in eight week old Japanese quail. Baseline body temperatures represent those measured at 30°C. Panel A displays Gelman-Rubin statistics for all model parameters. Panel B displays the ratio of effective sample sizes to sample sizes, again, for each model parameter. Panel C displays densities of true (y) and predicted (yhat) values of body temperature responses to cold. Panel D displays true and fitted body temperature responses. The dashed line in panel D represents that line of best fit as estimated by the R package ggplot2 (Wickham 2011).*

Again, our HMC chains appear well converged with little evidence of autocorrelation, and predictions from our analysis reasonably overlay with true body temperature responses. Next, distributions of residuals from our analysis are visually diagnosed.

```
g(p1, p2, p3, p4, p5, p6) %=% list(
  ggplot(
    tbResponseHot8Weeks$data %>%
      mutate(
        "residuals" =
```

```
        residuals(tbResponseHot8Weeks,
          type = "ordinary",
          robust = TRUE,
          resp = "deltaTb"
        )[, "Estimate"]
    ), aes(sample = residuals)
) +
  stat_qq(colour = "grey50") +
  stat_qq_line() +
  xlab("Theoretical") +
  ylab("Sample") +
  theme_classic(),

ggplot(
  tbResponseHot8Weeks$data %>%
    mutate(
      "residuals" =
        residuals(tbResponseHot8Weeks,
          type = "ordinary",
          robust = TRUE,
          resp = "deltaTb"
        )[, "Estimate"],
      "resSE" = residuals(tbResponseHot8Weeks,
        type = "ordinary",
        robust = TRUE,
        resp = "deltaTb"
      )[, "Est.Error"]
    ),
  aes(x = mass, y = residuals)
) +
  geom_errorbar(
    aes(
      x = mass, ymin = residuals - resSE,
      ymax = residuals + resSE
    ),
    colour = "black", width = 2
  ) +
  geom_point(
    size = 2, pch = 21, colour = "black",
    fill = "grey70"
  ) +
  theme_classic() +
  xlab("Body Mass\n(g; Mean-Centred)") +
  ylab("Ordinary Residuals"),

ggplot(
  tbResponseHot8Weeks$data %>%
    mutate(
      "residuals" =
        residuals(tbResponseHot8Weeks,
          type = "ordinary",
          robust = TRUE,
          resp = "deltaTb"
        )[, "Estimate"],
      "resSE" = residuals(tbResponseHot8Weeks,
        type = "ordinary",
        robust = TRUE,
        resp = "deltaTb"
      )[, "Est.Error"]
    ),
  aes(x = tarsus, y = residuals)
) +
  geom_errorbar(
    aes(
      x = tarsus, ymin = residuals - resSE,
      ymax = residuals + resSE
    ),
```

```
      colour = "black", width = 2
    ) +
    geom_point(
      size = 2, pch = 21, colour = "black",
      fill = "grey70"
    ) +
    theme_classic() +
    xlab("Tarsus Length\n(mm; Mean-Centred)") +
    ylab("Ordinary Residuals"),

  ggplot(
    tbResponseHot8Weeks$data %>%
      mutate(
        "residuals" =
          residuals(tbResponseHot8Weeks,
            type = "ordinary",
            robust = TRUE,
            resp = "deltaTb"
          )[, "Estimate"],
        "resSE" = residuals(tbResponseHot8Weeks,
          type = "ordinary",
          robust = TRUE,
          resp = "deltaTb"
        )[, "Est.Error"]
      ),
    aes(x = bill, y = residuals)
  ) +
    geom_errorbar(
      aes(
        x = bill, ymin = residuals - resSE,
        ymax = residuals + resSE
      ),
      colour = "black", width = 2
    ) +
    geom_point(
      size = 2, pch = 21, colour = "black",
      fill = "grey70"
    ) +
    theme_classic() +
    xlab("Bill Length\n(mm; Mean-Centred)") +
    ylab("Ordinary Residuals"),

  ggplot(
    tbResponseHot8Weeks$data %>%
      mutate(
        "residuals" =
          residuals(tbResponseHot8Weeks,
            type = "ordinary",
            robust = TRUE,
            resp = "deltaTb"
          )[, "Estimate"],
        pretreatment = ifelse(pretreatment == "B",
          "Mild (20°C)",
          ifelse(pretreatment == "A",
            "Cold (10°C)", "Warm (30°C)"
          )
        )
      ),
    aes(x = pretreatment, y = residuals)
  ) +
    geom_boxplot(colour = "black", alpha = 0.5, fill = "grey70") +
    geom_point(
      size = 1.5, colour = "black",
      position = position_jitter(width = 0.25)
    ) +
    theme_classic() +
    xlab("Rearing Treatment") +
```

```
    ylab("Ordinary Residuals"),

  ggplot(
    tbResponseHot8Weeks$data %>%
      mutate(
        "residuals" =
          residuals(tbResponseHot8Weeks,
            type = "ordinary",
            robust = TRUE,
            resp = "deltaTb"
          )[, "Estimate"]
      ),
    aes(x = residuals, fill = batch)
  ) +
    geom_density(colour = "black", alpha = 0.5) +
    scale_fill_manual(
      values = c("black", "grey40", "grey90"),
      name = "Egg Batch"
    ) +
    theme_classic() +
    xlab("Ordinary Residuals") +
    ylab("Density")
)

(p1 + p2) / (p3 + p4) / (p5 + p6) +
  plot_annotation(tag_levels = "A")
```

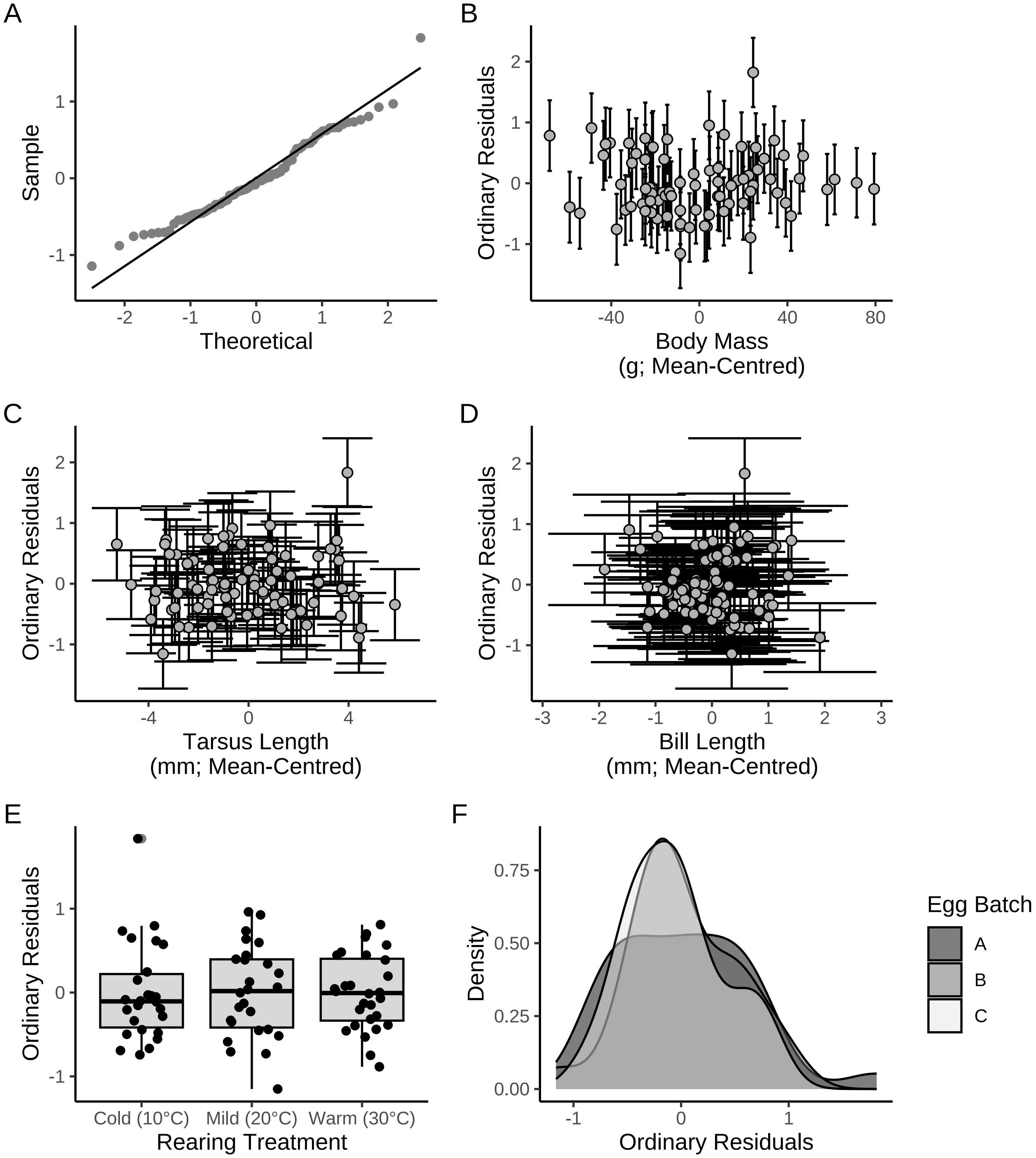

Figure 198: *Ordinary residuals from a Bayesian path analysis ultimately predicting body temperature responses (°C) to heat (40°C) in eight week old Japanese quail. Panel A displays theoretical residuals by quantiles against sampled residuals (qq-plot), panels B-D displays median residuals against mean-centred body mass (g), tarsus length (mm) and mean-centred bill length (mm). Panel E displays median residuals against rearing treatment, and panel F displays median residuals against egg source number (or batch). Errorbars in panels B-D indicate one median absolute deviation around median residuals.*

One data point appears to deviate from others and from expectations of error normality (obtained from a cold-reared individual with relatively long tarsi and slightly above average body mass). We check whether

this value has undue influence on model outcomes by using Pareto-smoothed, leave-one-out ("LOO") cross-validation (Vehtari et al, 2017). Influence values per data point (here, Pareto K) are then visualised for extremes.

```
loo(tbResponseHot8Weeks)$diagnostics$pareto_k %>%
  as_tibble() %>%
  ggplot(aes(x = 1:nrow(.), y = value)) +
  geom_point(pch = 21, colour = "black", fill = "grey50", size = 2) +
  geom_hline(yintercept = 0.5, colour = "black", linetype = "dashed") +
  geom_hline(yintercept = 0.7, colour = "red4", linetype = "dashed") +
  xlab("Sample") +
  ylab("Pareto K") +
  theme_classic()
```

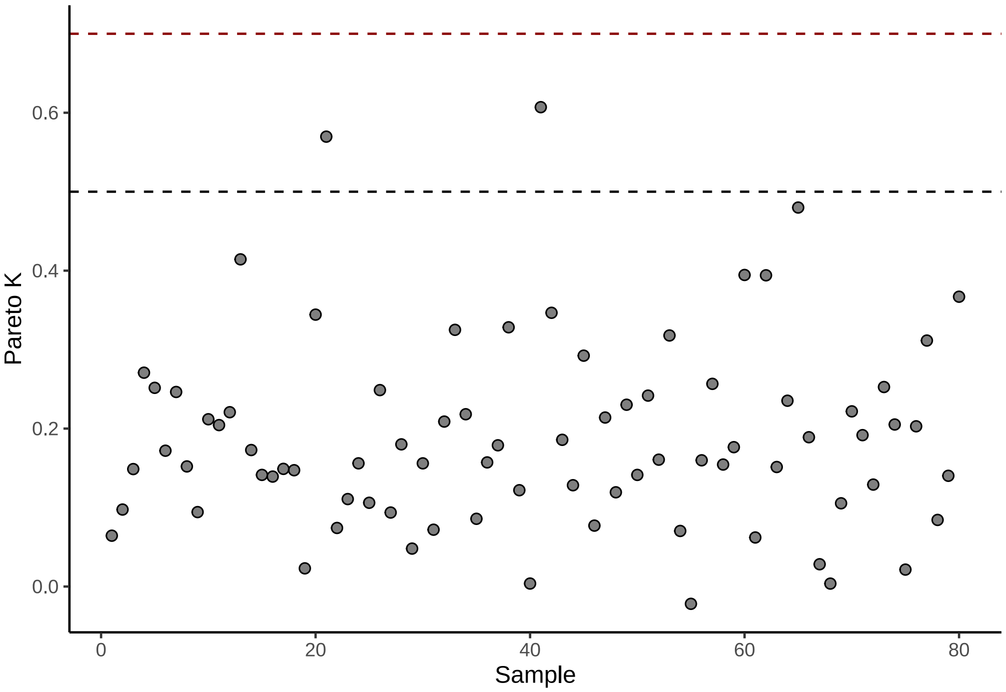

Figure 199: *Relative importance of individual body temperature response values (dots; derived from mature quail) on outcomes of our Bayesian path analysis, as estimated using leave-one-out cross validations. Importance is measured here as Pareto K (Vehtari et al, 2017). The black, dashed horizontal line indicated a Pareto K of 0.5 and the red horizontal dashed line indicates as Pareto K of 0.7, above which, samples hold significant importance on analysis outcomes.*

Two data points have a moderate degree of influence on our model (Pareto K $>$ 0.5). These values are identified and inspected below.

```
caption <- paste0("Potential body temperature response outliers ",
                  "among adult Japanese quail with ",
                  "body temperature measured at 30°C and 40°C."
)

tbResponseHot8Weeks$data %>%
  mutate("paretoK" = loo(tbResponseHot8Weeks)$diagnostics$pareto_k) %>%
  filter(paretoK > 0.5) %>%
  dplyr::select(-c(pretreatment, mass, tarsus, bill, batch)) %>%
  merge(., tbResponse %>%
    filter(challenge == "warm"),
  by = c("deltaTb"),
```

```
  all.x = TRUE
  ) %>%
  merge(., all %>%
    filter(Ta == 30) %>%
    dplyr::select(ring, week, meanTb) %>%
    mutate(meanTb = round(meanTb, digits = 2)) %>%
    rename("Tb at 30°C" = meanTb),
  by = c("ring", "week"),
  all.x = TRUE
  ) %>%
  merge(., all %>%
    filter(Ta == 40) %>%
    dplyr::select(ring, week, meanTb) %>%
    mutate(meanTb = round(meanTb, digits = 2)) %>%
    rename("Tb at 40°C" = meanTb),
  by = c("ring", "week"),
  all.x = TRUE
  ) %>%
  mutate(pretreatment = ifelse(pretreatment == "cold",
    "Cold (10°C)",
    ifelse(pretreatment == "neutral",
      "Mild (20°C)", "Warm (30°C)"
    )
  ),
  tarsus = round(tarsus, digits = 2),
  bill = round(bill, digits = 2),
  deltaTb = round(deltaTb, digits = 2),
  paretoK = round(paretoK, digits = 2)
  ) %>%
  dplyr::select(
    "Bird ID" = ring,
    "Rearing Treatment" = pretreatment,
    "Egg Batch" = batch,
    "Mass (g)" = mass,
    "Tarsus Length (mm)" = tarsus,
    "Bill Length (mm)" = bill,
    `Tb at 30°C`,
    `Tb at 40°C`,
    "Delta Tb (°C)" = deltaTb,
    "Pareto K" = paretoK
  ) %>%
  kbl(.,
    longtable = T, booktabs = T, format = "latex", escape = FALSE,
    caption = caption
  ) %>%
  column_spec(column = c(1:10), width = "1.2cm") %>%
  kable_styling(latex_options = "striped")
```

Table 118: *Potential body temperature response outliers among adult Japanese quail with body temperature measured at 30°C and 40°C.*

Bird ID	Rearing Treatment	Egg Batch	Mass (g)	Tarsus Length (mm)	Bill Length (mm)	Tb at 30°C	Tb at 40°C	Delta Tb (°C)	Pareto K
2RLG	Cold (10°C)	A	269.0	40.64	10.2	41.67	44.60	2.93	0.57
R20	Mild (20°C)	B	264.5	42.54	10.7	42.32	43.45	1.13	0.61

All measurements are within the range of expectations for adult Japanese quail. For this reason, these values are retained. However, our model is also re-run with a Student's-T distributed error term (prior on $\nu = \Gamma(2, 0.5)$) and posterior densities contrasted against those from our original model.

```r
tbResponseHot8WeeksStudent <-
  brm(
    data = tbResponse %>%
      filter(week == "8" & challenge == "warm") %>%
      mutate(
        mass = mass - mean(mass, na.rm = T),
        tarsus = tarsus - mean(tarsus, na.rm = T),
        bill = bill - mean(bill, na.rm = T),
        pretreatment = ifelse(pretreatment == "cold", "A",
          ifelse(pretreatment == "neutral", "B", "C")
        )
      ) %>%
      mutate(pretreatment = factor(pretreatment,
                                   levels = c("B", "A", "C"))
             ) %>%
      drop_na(),
    bf(mass ~ pretreatment + (1 | batch)) +
      bf(tarsus ~ mass + pretreatment + (1 | batch)) +
      bf(bill ~ mass + pretreatment + (1 | batch)) +
      bf(deltaTb ~ mass + tarsus + bill + pretreatment + (1 | batch),
         family = "student") +
      set_rescor(FALSE),
    prior = c(
      set_prior("normal(0, 10)",
        class = "Intercept",
        resp = "mass"
      ),
      set_prior("normal(0, 25)",
        class = "b",
        coef = "pretreatmentA",
        resp = "mass"
      ),
      set_prior("normal(0, 25)",
        class = "b",
        coef = "pretreatmentC",
        resp = "mass"
      ),
      set_prior("exponential(2.5)",
        class = "sd",
        group = "batch",
        resp = "mass"
      ),
      set_prior("exponential(0.15)",
        class = "sigma",
        resp = "mass"
      ),
      set_prior("normal(0, 2.5)",
        class = "Intercept",
        resp = "tarsus"
      ),
      set_prior("normal(0, 2.5)",
        class = "b",
        coef = "pretreatmentA",
        resp = "tarsus"
      ),
      set_prior("normal(0, 2.5)",
        class = "b",
        coef = "pretreatmentC",
        resp = "tarsus"
      ),
      set_prior("skew_normal(0, 0.25, 5)",
        class = "b",
        coef = "mass",
        resp = "tarsus"
      ),
      set_prior("exponential(2)",
        class = "sd",
```

```
      group = "batch",
      resp = "tarsus"
    ),
    set_prior("exponential(1)",
      class = "sigma",
      resp = "tarsus"
    ),
    set_prior("normal(0, 1)",
      class = "Intercept",
      resp = "bill"
    ),
    set_prior("normal(0, 0.5)",
      class = "b",
      coef = "pretreatmentA",
      resp = "bill"
    ),
    set_prior("normal(0, 0.5)",
      class = "b",
      coef = "pretreatmentC",
      resp = "bill"
    ),
    set_prior("skew_normal(0, 0.25, 5)",
      class = "b",
      coef = "mass",
      resp = "bill"
    ),
    set_prior("exponential(5)",
      class = "sd",
      group = "batch",
      resp = "bill"
    ),
    set_prior("exponential(2.5)",
      class = "sigma",
      resp = "bill"
    ),
    set_prior("normal(1, 1)",
      class = "Intercept",
      resp = "deltaTb"
    ),
    set_prior("normal(0, 1)",
      class = "b",
      coef = "pretreatmentA",
      resp = "deltaTb"
    ),
    set_prior("normal(0, 1)",
      class = "b",
      coef = "pretreatmentC",
      resp = "deltaTb"
    ),
    set_prior("normal(0, 0.01)",
      class = "b",
      coef = "mass",
      resp = "deltaTb"
    ),
    set_prior("normal(0, 0.15)",
      class = "b",
      coef = "tarsus",
      resp = "deltaTb"
    ),
    set_prior("normal(0, 0.4)",
      class = "b",
      coef = "bill",
      resp = "deltaTb"
    ),
    set_prior("exponential(2.5)",
      class = "sd",
      group = "batch",
```

```
        resp = "deltaTb"
      ),
      set_prior("exponential(2.5)",
        class = "sigma",
        resp = "deltaTb"
      ),
      set_prior("gamma(2, 0.5)",
        class = "nu",
        resp = "deltaTb"
      )
    ),
    iter = 50000, warmup = 10000, cores = 4, chains = 4, thin = 20,
    control = list(adapt_delta = .98, max_treedepth = 14),
    silent = TRUE, refresh = 0,
    file = "./models/_bodyTemperatureResponsesToHeatStudent.Rds"
  )

postDraws <- rbind(
  as.data.frame(tbResponseHot8Weeks) %>%
    dplyr::select(starts_with("b_"), starts_with("sd_")) %>%
    pivot_longer(everything(), names_to = "par", values_to = "Vals") %>%
    mutate("Model" = "Gaussian Error"),
  as.data.frame(tbResponseHot8WeeksStudent) %>%
    dplyr::select(starts_with("b_"), starts_with("sd_")) %>%
    pivot_longer(everything(), names_to = "par", values_to = "Vals") %>%
    mutate("Model" = "Student's T Error")
) %>%
  left_join(., tribble(
    ~par, ~Par, ~Order,
    "b_mass_Intercept", "Mass  0", "A",
    "b_mass_pretreatmentA", "Mass ~\nCold Rearing", "B",
    "b_mass_pretreatmentC", "Mass ~\nWarm Rearing", "C",
    "sd_batch__mass_Intercept", "Mass ~\nBatch", "D",
    "b_tarsus_Intercept", "Tarsus  0", "E",
    "b_tarsus_pretreatmentA", "Tarsus ~\nCold Rearing", "F",
    "b_tarsus_pretreatmentC", "Tarsus ~\nWarm Rearing", "G",
    "b_tarsus_mass", "Tarsus ~\nMass", "H",
    "sd_batch__tarsus_Intercept", "Tarsus ~\nBatch", "I",
    "b_bill_Intercept", "Bill  0", "J",
    "b_bill_pretreatmentA", "Bill ~\nCold Rearing", "K",
    "b_bill_pretreatmentC", "Bill ~\nWarm Rearing", "L",
    "b_bill_mass", "Bill ~\nMass", "M",
    "sd_batch__bill_Intercept", "Bill ~\nBatch", "N",
    "b_deltaTb_Intercept", "Delta Tb  0", "O",
    "b_deltaTb_pretreatmentA", "Delta Tb ~\nCold Rearing", "P",
    "b_deltaTb_pretreatmentC", "Delta Tb ~\nWarm Rearing", "Q",
    "b_deltaTb_mass", "Delta Tb ~\nMass", "R",
    "b_deltaTb_tarsus", "Delta Tb ~\nTarsus", "S",
    "b_deltaTb_bill", "Delta Tb ~\nBill", "T"
  ), by = "par") %>%
  dplyr::select(Model, Par, Vals, Order) %>%
  arrange(Order) %>%
  mutate(Par = factor(Par, levels = unique(Par)))

p1 <- postDraws %>%
  filter(grepl("Mass ", Par)) %>%
  ggplot(aes(x = Vals, fill = Model)) +
  facet_wrap(~Par, scales = "free") +
  geom_density(colour = "black", alpha = 0.5) +
  scale_fill_manual(values = c("grey30", "grey90")) +
  theme_classic() +
  theme(legend.position = "bottom") +
  ylab("Density") +
  xlab("Posterior Values")

p2 <- postDraws %>%
  filter(grepl("Tarsus ", Par)) %>%
```

```
  ggplot(aes(x = Vals, fill = Model)) +
  facet_wrap(~Par, scales = "free") +
  geom_density(colour = "black", alpha = 0.5) +
  scale_fill_manual(values = c("grey30", "grey90")) +
  xlab("Posterior Values") +
  ylab("Density") +
  theme_classic() +
  theme(legend.position = "none")

p3 <- postDraws %>%
  filter(grepl("Bill ", Par)) %>%
  ggplot(aes(x = Vals, fill = Model)) +
  facet_wrap(~Par, scales = "free") +
  geom_density(colour = "black", alpha = 0.5) +
  scale_fill_manual(values = c("grey30", "grey90")) +
  xlab("Posterior Values") +
  ylab("Density") +
  theme_classic() +
  theme(legend.position = "none")

((p1)/(p2)/(p3))
```

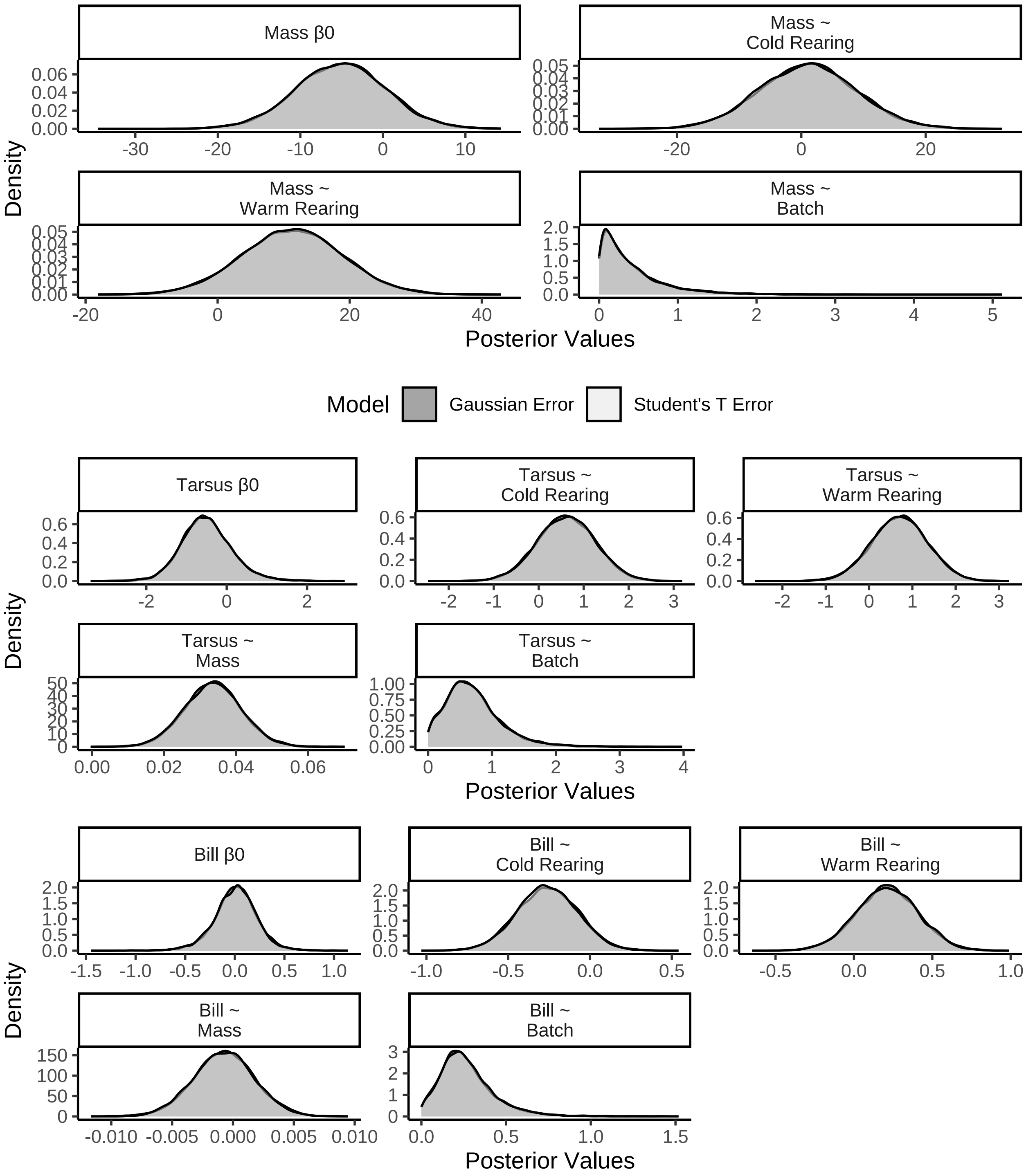

Figure 200: *Posterior densities, plotted by model parameter, or two path-analyses ultimately predicting body temperature responses to a heat exposure (40°C; as relative changes in °C from that observed at 30°C) among adult Japanese quail. One model assumes that error surrounding body temperature response values is Gaussian distributed while the other assumes that this error is Student's-T distributed. Part 1.*

```
postDraws %>%
  filter(grepl("Delta Tb ", Par)) %>%
  ggplot(aes(x = Vals, fill = Model)) +
  facet_wrap(~Par, scales = "free") +
```

```
  geom_density(colour = "black", alpha = 0.5) +
  scale_fill_manual(values = c("grey30", "grey90")) +
  xlab("Posterior Values") +
  ylab("Density") +
  theme_classic()
```

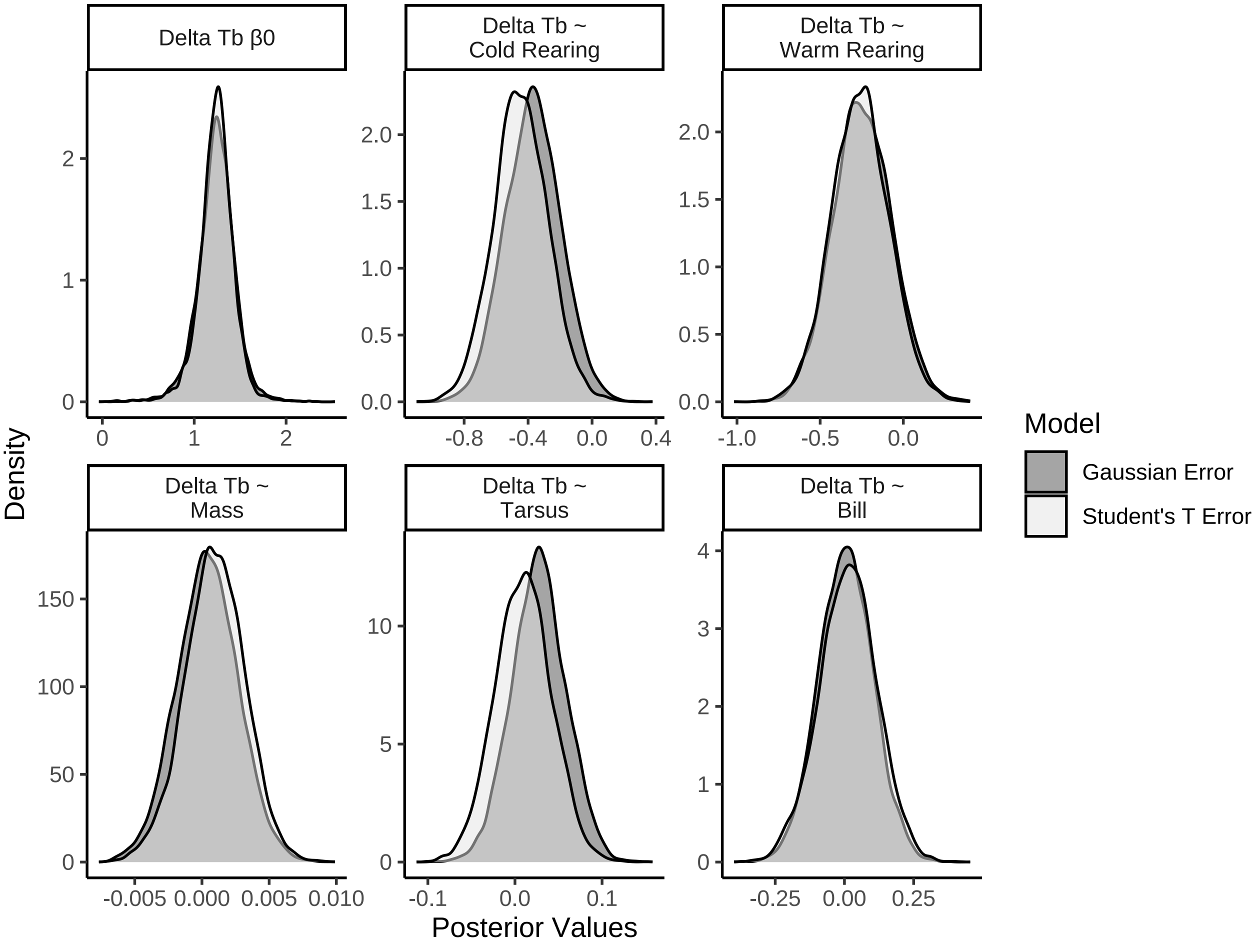

Figure 201: *Posterior densities, plotted by model parameter, or two path-analyses ultimately predicting body temperature responses to a heat exposure (40°C; as relative changes in °C from that observed at 30°C) among adult Japanese quail. One model assumes that error surrounding body temperature response values is Gaussian distributed while the other assumes that this error is Student's-T distributed. Part 2.*

Change in posteriors densities between models are subtle, but visible. We therefore proceed with our second path analysis (with the body temperature response error term assumed to be Student's-T distributed) and estimate variance in our data explained by this analysis.

```
# Checking R2 values

caption <- paste0(
  "R\\textsuperscript{2} for Bayesian path analysis ",
  "ultimate predicting body temperature responses (°C) to heat ",
  "(40°C) in eight week old Japanese quail. Baseline body ",
  "temperatures represent those measured at thermoneutrality ",
  "(30°C)"
)

brms::bayes_R2(tbResponseHot8WeeksStudent,
```

```
  robust = TRUE, ndraws = 1000
) %>%
  as.data.frame() %>%
  rownames_to_column(var = "response") %>%
  left_join(., tribble(~response, ~Response,
                      "R2mass", "Body Mass (g)",
                      "R2tarsus", "Tarsus Length (mm)",
                      "R2bill", "Bill Length (mm)",
                      "R2deltaTb", "Change in Tb (°C)"),
            by = "response") %>%
  mutate(
    Estimate = round(Estimate, digits = 4),
    Est.Error = round(Est.Error, digits = 4),
    Q2.5 = round(Q2.5, digits = 4),
    Q97.5 = round(Q97.5, digits = 4)
  ) %>%
  dplyr::select(
    Response,
    "R\\textsuperscript{2}" = Estimate,
    "Standard Error" = Est.Error,
    `2.5\\%CI` = "Q2.5", `97.5\\% CI` = "Q97.5"
  ) %>%
  kbl(.,
    longtable = T, booktabs = T, format = "latex",
    caption = caption, escape = FALSE
  ) %>%
  kable_styling(latex_options = "striped")
```

Table 119: *R^2 for Bayesian path analysis ultimate predicting body temperature responses (°C) to heat (40°C) in eight week old Japanese quail. Baseline body temperatures represent those measured at thermoneutrality (30°C)*

Response	R^2	Standard Error	2.5%CI	97.5% CI
Body Mass (g)	0.0392	0.0338	0.0019	0.1268
Tarsus Length (mm)	0.3285	0.0692	0.1787	0.4506
Bill Length (mm)	0.1282	0.0662	0.0276	0.2642
Change in Tb (°C)	0.1912	0.0657	0.0771	0.3091

Last, we summarise estimates from our path analysis while again using posterior medians to represent model coefficients and quantiles to estimate credible intervals around medians.

```
caption <- paste0(
  "Results from a Bayesian path analysis ",
  "ultimately predicting body temperature responses to a ",
  "heat exposure (40°C) relative to thermoneutrality (30°C)",
  "in adult Japanese quail. Body temperature responses are ",
  "predicted as a function of body mass (g), tarsus length (mm), ",
  "bill length (mm) and rearing treatment. ",
  "Cold rearing indicates post-hatch rearing at 10°C, relative to ",
  "20°C (intercept), or 30°C ('warm rearing'). ",
  "Coefficients represent medians and credible intervals ",
  "(CIs) represent quantile intervals. BF indicates Bayes Factors."
)

as.data.frame(tbResponseHot8WeeksStudent) %>%
  summarise_all(., .funs = median) %>%
  pivot_longer(everything(),
    names_to = "Parameter",
    values_to = "Estimate"
  ) %>%
  merge(., quantileCIs(tbResponseHot8WeeksStudent, cis = c(50, 95)),
    by = "Parameter", all.x = TRUE
  ) %>%
```

```
filter(grepl("b_|sd_", Parameter)) %>%
rowwise() %>%
mutate("BF" = ifelse(Estimate < 0,
  (2 * mean(as.data.frame(
    tbResponseHot8WeeksStudent
  )[, Parameter] <= 0)) /
    (2 * mean(as.data.frame(
      tbResponseHot8WeeksStudent
    )[, Parameter] >= 0)),
  (2 * mean(as.data.frame(
    tbResponseHot8WeeksStudent
  )[, Parameter] >= 0)) /
    (2 * mean(as.data.frame(
      tbResponseHot8WeeksStudent
    )[, Parameter] <= 0))
)) %>%
ungroup() %>%
mutate(
  "Estimate" = round(Estimate, digits = 4),
  "BF" = round(BF, digits = 4),
  "N" = nrow(tbResponseHot8WeeksStudent$data)
) %>%
rowwise() %>%
mutate(Parameter = gsub("deltaTb", "deltaT", Parameter)) %>%
ungroup() %>%
mutate("Parameter" = ifelse(grepl("b_", Parameter),
                            gsub("b_", "", Parameter),
                            gsub("Intercept", "batch",
                                 gsub(".*__", "", Parameter)
                                 )
                            )
       ) %>%
mutate(
  "Response" = gsub("_.*", "", Parameter),
  "Parameter" = gsub(".*_", "", Parameter)
) %>%
merge(., tribble(
  ~Response, ~response, ~level,
  "mass", "Body Mass (g)", "A",
  "tarsus", "Tarsus Length (mm)", "B",
  "bill", "Bill Length (mm)", "C",
  "deltaT", "Delta Body Temperature (°C)", "D",
),
by = "Response"
) %>%
merge(., tribble(
  ~Parameter, ~parameter, ~number,
  "Intercept", "Intercept", "1",
  "pretreatmentA", "Cold Rearing", "2",
  "pretreatmentC", "Warm Rearing", "3",
  "mass", "Body Mass (g)", "4",
  "tarsus", "Tarsus Length (mm)", "5",
  "bill", "Bill Length (mm)", "6",
  "batch", "Egg Batch [mu]", "7"
),
by = "Parameter"
) %>%
mutate(
  `50\\% CI` = paste0("(", paste(
    round(Low_CI_50, digits = 4),
    round(High_CI_50, digits = 4),
    sep = ", "
  ), ")"),
  `95\\% CI` = paste0("(", paste(
    round(Low_CI_95, digits = 4),
    round(High_CI_95, digits = 4),
    sep = ", "
```

```
  ), ")")
) %>%
dplyr::select(-c(Low_CI_50, High_CI_50, Low_CI_95, High_CI_95)) %>%
dplyr::select(
  "Response" = "response", "Parameter" = "parameter", N,
  Estimate, `50\\% CI`, `95\\% CI`, BF, level, number
) %>%
arrange(level, number) %>%
dplyr::select(-c(level, number)) %>%
kbl(.,
  longtable = T, booktabs = T, format = "latex", escape = FALSE,
  caption = caption
) %>%
column_spec(column = c(1:2), width = "2.2cm") %>%
column_spec(column = c(3:10), width = "1.9cm") %>%
kable_styling(latex_options = "striped")
```

Table 120: *Results from a Bayesian path analysis ultimately predicting body temperature responses to a heat exposure (40°C) relative to thermoneutrality (30°C)in adult Japanese quail. Body temperature responses are predicted as a function of body mass (g), tarsus length (mm), bill length (mm) and rearing treatment. Cold rearing indicates post-hatch rearing at 10°C, relative to 20°C (intercept), or 30°C ('warm rearing'). Coefficients represent medians and credible intervals (CIs) represent quantile intervals. BF indicates Bayes Factors.*

Response	Parameter	N	Estimate	50% CI	95% CI	BF
Body Mass (g)	Intercept	80	-5.0480	(-8.8067, -1.4431)	(-15.941, 5.7939)	4.6940
Body Mass (g)	Cold Rearing	80	1.1233	(-4.3161, 6.3091)	(-14.0137, 16.5567)	1.2554
Body Mass (g)	Warm Rearing	80	11.3597	(6.2034, 16.3911)	(-3.6598, 26.2296)	13.5455
Body Mass (g)	Egg Batch [mu]	80	0.2779	(0.1143, 0.5494)	(0.011, 1.513)	Inf
Tarsus Length (mm)	Intercept	80	-0.5273	(-0.9139, -0.1178)	(-1.6753, 0.8566)	4.0157
Tarsus Length (mm)	Cold Rearing	80	0.6199	(0.1777, 1.0515)	(-0.6488, 1.8759)	4.9041
Tarsus Length (mm)	Warm Rearing	80	0.7035	(0.2713, 1.1344)	(-0.5457, 1.9559)	6.2860
Tarsus Length (mm)	Body Mass (g)	80	0.0336	(0.0281, 0.0389)	(0.0178, 0.0491)	7999.0000
Tarsus Length (mm)	Egg Batch [mu]	80	0.6418	(0.4015, 0.9261)	(0.0514, 1.7829)	Inf
Bill Length (mm)	Intercept	80	0.0136	(-0.1249, 0.1429)	(-0.4579, 0.4257)	1.1209
Bill Length (mm)	Cold Rearing	80	-0.2638	(-0.3859, -0.1396)	(-0.6244, 0.0948)	11.8411
Bill Length (mm)	Warm Rearing	80	0.2113	(0.0787, 0.3454)	(-0.1799, 0.5912)	5.9991
Bill Length (mm)	Body Mass (g)	80	-0.0007	(-0.0024, 9e-04)	(-0.0058, 0.0041)	1.5999
Bill Length (mm)	Egg Batch [mu]	80	0.2443	(0.1602, 0.3496)	(0.0274, 0.654)	Inf
Delta Body Temperature (°C)	Intercept	80	1.2474	(1.1388, 1.3511)	(0.845, 1.5916)	7999.0000
Delta Body Temperature (°C)	Cold Rearing	80	-0.4475	(-0.5577, -0.3314)	(-0.7768, -0.1035)	147.1481
Delta Body Temperature (°C)	Warm Rearing	80	-0.2588	(-0.3762, -0.1442)	(-0.5905, 0.0828)	13.8423

Delta Body Temperature (°C)	Body Mass (g)	80	0.0010	(-5e-04, 0.0025)	(-0.0036, 0.0052)	2.0465
Delta Body Temperature (°C)	Tarsus Length (mm)	80	0.0096	(-0.0123, 0.0309)	(-0.053, 0.0728)	1.5991
Delta Body Temperature (°C)	Bill Length (mm)	80	0.0143	(-0.0567, 0.0827)	(-0.2006, 0.2166)	1.2542
Delta Body Temperature (°C)	Egg Batch [mu]	80	0.1629	(0.0825, 0.2843)	(0.0081, 0.7193)	Inf

7.0 References

Atchley, D.S., Foster, J.A. and Bavis, R.W. Thermoregulatory and metabolic responses of Japanese quail to hypoxia. *Comp. Biochem. Physiol. A: Mol. Integr. Physiol.* **151**, 641-650 (2008).

Ben-Hamo, M., Pinshow, B., McCue, M.D., McWilliams, S.R. and Bauchinger, U. Fasting triggers hypothermia, and ambient temperature modulates its depth in Japanese quail *Coturnix japonica*. *Comp. Biochem. Physiol. A: Mol. Integr. Physiol.* **156**, 84-91 (2010).

Burness, G, Huard, J.R., Malcolm, E. and Tattersall, G.J. Post-hatch heat warms adult beaks: irreversible physiological plasticity in Japanese quail. *Proc. Roy. Soc B: Biol. Sci.* **280**, 20131436 (2013).

Haqani, M.I., Kawamura, K., Takenouchi, A., Kabir, M.H., Nakamura, Y., Ishikawa, A. and Tsudzuki, M. A growth performance and nonlinear growth curve functions of large-and normal-sized Japanese Quail (*Coturnix japonica*). *J. Poul. Sci.* **58**, 88-96 (2021).

Kallioinen, N., Paananen, T., Bürkner, P.C. and Vehtari, A. Detecting and diagnosing prior and likelihood sensitivity with power-scaling. *Stat. Comp.* **34**, 57 (2024).

Kendeigh, S.C. Tolerance of cold and Bergmann's rule. *Auk* **86**, 13-25 (2021).

McKechnie, A.E., Gerson, A.R. and Wolf, B.O. Thermoregulation in desert birds: scaling and phylogenetic variation in heat tolerance and evaporative cooling. *J. Exp. Biol* **224**, jeb229211 (2021).

Narinc, D., Karaman, E., Firat, M.Z. and Aksoy, T. Comparison of non-linear growth models to describe the growth in Japanese quail. *J. Anim. Vet. Adv.* **9**, 1961-1966 (2010).

Persson, E., Ó Cuív, C. and Nord, A. Thermoregulatory consequences of growing up during a heatwave or a cold snap in Japanese quail. *J. Exp. Biol.* **227**, jeb246876 (2024).

Playà-Montmany, N., González-Medina, E., Cabello-Vergel, J., Parejo, M., Abad-Gómez, J.M., Sánchez-Guzmán, J.M. and Masero, J.A. The thermoregulatory role of relative bill and leg surface areas in a Mediterranean population of Great tit (*Parus major*). *Ecol. Evol.* **11**, 15936-15946 (2021).

R Core Team. R: A language and environment for statistical computing. R Foundation for Statistical Computing, Vienna, Austria. URL https://www.R-project.org/ (2023).

Saarela, S. and Heldmaier, G. Effect of photoperiod and melatonin on cold resistance, thermoregulation and shivering/nonshivering thermogenesis in Japanese quail. *J. Comp. Physiol. B* **157**, 625-633 (1987).

Schielzeth, H. and Nakagawa, S. Conditional repeatability and the variance explained by reaction norm variation in random slope models. *Methods Ecol. Evol.* **13**, 1214-1223 (1987).

Tabh, J.K., Mastromonaco, G.F. and Burness, G. Stress-induced changes in body surface temperature are repeatable, but do not differ between urban and rural birds. *Oecologia* **198**, 663-677 (2022).

Vehtari, A., Gelman, A. and Gabry, J. Practical Bayesian model evaluation using leave-one-out cross-validation and WAIC. *Stat. Comp.* **27**, 1413-1432 (2017).

Vehtari, A., Gabry, J., Magnusson, M., Yao, Y., Bürkner, P.C., Paananen, T. and Gelman, A. loo reference manual, *Version 2.3.1. https://CRAN.R-project.org/package=loo/loo.pdf* (2020).

Weeks, B.C., Willard, D.E., Zimova, M., Ellis, A.A., Witynski, M.L., Hennen, M. and Winger, B. M. Shared morphological consequences of global warming in North American migratory birds. *Ecol. Lett.* **23**, 316-325 (2020).

Wickham, H. ggplot2. *Wiley Interdiscip. Rev. Comput. Stat.* **3**, 180-185 (2011).
