# Supplementary material for: Limited evidence that body size shrinking and shape-shifting alleviate thermoregulatory pressures in a warmer world
Source: Commun Biol. 2025 May 7;8:707. doi: 10.1038/s42003-025-08131-7 (PMC12059039; doi:10.1038/s42003-025-08131-7)
Supplement: Supplementary file 3 — Description of Additional Supplementary Files [file 42003_2025_8131_MOESM3_ESM.docx]

Description of Additional Supplementary Files

**File name:** Supplemental Dataset 1

**Description:** The source data used for the generation of all results and graphs in this paper.
