# Supplementary figures and images for: Limited evidence that body size shrinking and shape-shifting alleviate thermoregulatory pressures in a warmer world
Source: Commun Biol. 2025 May 7;8:707. doi: 10.1038/s42003-025-08131-7 (PMC12059039; doi:10.1038/s42003-025-08131-7)

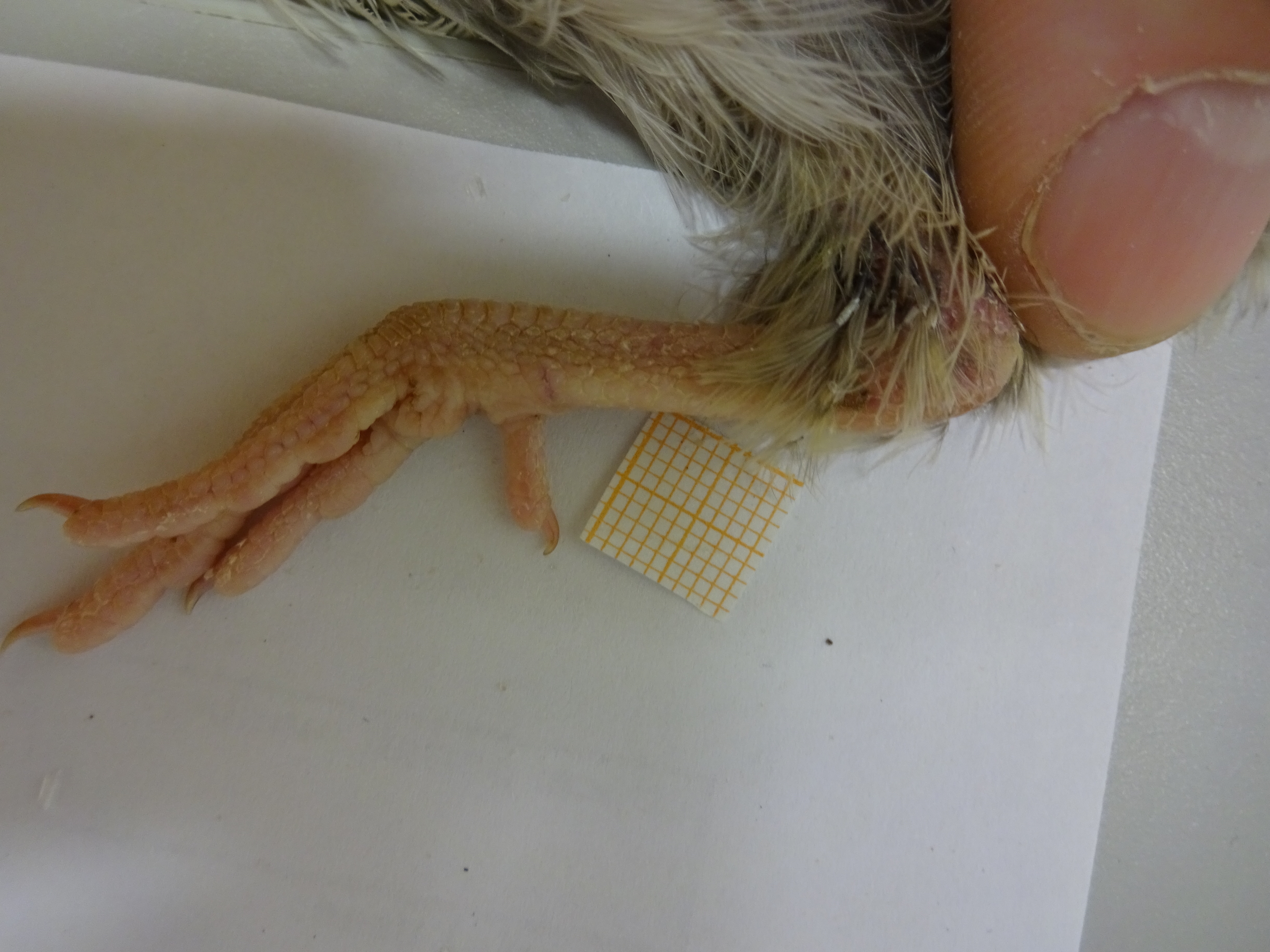

Supplement: Supplementary file 4 — Supplementary Dataset 1 [file 42003_2025_8131_MOESM4_ESM.zip › supplementalData/tarsusImage.jpg]
