# Supplementary material for: Limited evidence that body size shrinking and shape-shifting alleviate thermoregulatory pressures in a warmer world
Source: Commun Biol. 2025 May 7;8:707. doi: 10.1038/s42003-025-08131-7 (PMC12059039; doi:10.1038/s42003-025-08131-7)
Supplement: Supplementary file 5 — Reporting summary [file 42003_2025_8131_MOESM5_ESM.pdf]

nature portfolio

Corresponding author(s): Joshua K.R. Tabh

Last updated by author(s): 2025/04/16

Reporting Summary

Nature Portfolio wishes to improve the reproducibility of the work that we publish. This form provides structure for consistency and transparency in reporting. For further information on Nature Portfolio policies, see our Editorial Policies and the Editorial Policy Checklist.

Statistics

For all statistical analyses, confirm that the following items are present in the figure legend, table legend, main text, or Methods section.

n/a	Confirmed	
☐	☒	The exact sample size (*n*) for each experimental group/condition, given as a discrete number and unit of measurement
☐	☒	A statement on whether measurements were taken from distinct samples or whether the same sample was measured repeatedly
☐	☒	The statistical test(s) used AND whether they are one- or two-sided<br>*Only common tests should be described solely by name; describe more complex techniques in the Methods section.*
☐	☒	A description of all covariates tested
☐	☒	A description of any assumptions or corrections, such as tests of normality and adjustment for multiple comparisons
☐	☒	A full description of the statistical parameters including central tendency (e.g. means) or other basic estimates (e.g. regression coefficient) AND variation (e.g. standard deviation) or associated estimates of uncertainty (e.g. confidence intervals)
☐	☒	For null hypothesis testing, the test statistic (e.g. *F*, *t*, *r*) with confidence intervals, effect sizes, degrees of freedom and *P* value noted<br>*Give P values as exact values whenever suitable.*
☐	☒	For Bayesian analysis, information on the choice of priors and Markov chain Monte Carlo settings
☐	☒	For hierarchical and complex designs, identification of the appropriate level for tests and full reporting of outcomes
☐	☒	Estimates of effect sizes (e.g. Cohen's *d*, Pearson's *r*), indicating how they were calculated

Our web collection on statistics for biologists contains articles on many of the points above.

Software and code

Policy information about availability of computer code

Data collection	To measure the tarsus length of Japanese quail while minimising handling time, we flay-lay photographed the left leg or head of individuals on 1mmx1mm grid paper, then used ImageJ2 v2.9.0 to extract either the calibrated length between the angle and distal tarsometatarsus (leg length), or the straight-line length of the exposed culmen (bill length; see Methods for detailed information). For extraction of oxygen and water vapour readings from our flow-through respirometry system (here, used for resting metabolism and evaporative water loss estimation), we used ExpeData software (Sable Systems, USA). Last, for remote collection of body temperature readings used in supplemental analyses, we used a handheld, passive integrated transponder reader with integrated software (Biomark, USA).
Data analysis	All data organisation, analysis, and visualisations were conducted in R statistical software (v4.2.3). Bayesian models constructed in R were executed in Stan v2.32.2 via the R package brms (v2.21.1; Burkner, 2017).

For manuscripts utilizing custom algorithms or software that are central to the research but not yet described in published literature, software must be made available to editors and reviewers. We strongly encourage code deposition in a community repository (e.g. GitHub). See the Nature Portfolio guidelines for submitting code & software for further information.

Data

Policy information about availability of data

All manuscripts must include a data availability statement. This statement should provide the following information, where applicable:

- Accession codes, unique identifiers, or web links for publicly available datasets
- A description of any restrictions on data availability
- For clinical datasets or third party data, please ensure that the statement adheres to our policy

All data and analytical code used in the constructing of this manuscript are provided as supplemental information.

Research involving human participants, their data, or biological material

Policy information about studies with human participants or human data. See also policy information about sex, gender (identity/presentation), and sexual orientation and race, ethnicity and racism.

Reporting on sex and gender	N/A. This study did not involve the use of human participants, their data, or biological material.
Reporting on race, ethnicity, or other socially relevant groupings	N/A.
Population characteristics	N/A.
Recruitment	N/A.
Ethics oversight	N/A.

Note that full information on the approval of the study protocol must also be provided in the manuscript.

Field-specific reporting

Please select the one below that is the best fit for your research. If you are not sure, read the appropriate sections before making your selection.

☐ Life sciences ☐ Behavioural & social sciences ☒ Ecological, evolutionary & environmental sciences

For a reference copy of the document with all sections, see nature.com/documents/nr-reporting-summary-flat.pdf

Ecological, evolutionary & environmental sciences study design

All studies must disclose on these points even when the disclosure is negative.

Study description	For this study, we conducted a series of three replicate experiments, each involving manipulation of ambient temperature during rearing, then measurement of morphology (body mass, bill length and leg length), cold-induced resting metabolism, and heat-induced resting metabolism and water loss during development (three weeks of age) and at maturity (eight weeks of age). For each replicate experiment, we incubated locally-sourced Japanese quail eggs at a fixed temperature (37.5°C) and humidity (50%) until hatching, then allocated hatchlings evenly and semi-randomly (see "Randomization" below) among three possible environmental temperature treatments; 10°C (cold), 20°C (mild), or 30°C (warm). Hatchlings were reared in their defined temperature treatments until three weeks (replicates one and two) or eight weeks (replicate three) of age, after which, rearing temperatures were switched to 20°C until data collection for this study was complete. To enable us to test effects of morphology on thermoregulatory efficiency in our sample population, we measured body mass, bill length, tarsus length, resting metabolism during a cold challenge (replicates one and two) and both resting metabolism and evaporative water loss during a heat challenge (replicates one, two and three) at three and eight weeks of age. To enable us to test whether morphology changes in response to rearing temperatures, with possible direct consequences on thermoregulatory efficiency, body mass was measured weekly from hatch to maturity (eight weeks) and both bill and tarsus length measured weekly from hatch until three weeks, then again at maturity.
Research sample	Japanese quail of a single ("Jumbo") strain were obtained from a local breeder (Sigvard Månsgård, Åstorp, Sweden) as eggs and hatched in-house. Collection of quail from a single source population was chosen to enable tests of how morphology and rearing conditions may influence thermoregulatory efficiency in endotherms, without bias or "noise" arising from broad genetic differentiation.
Sampling strategy	Sampling for this study occurred across three consecutive experimental bouts (or batches). Sample sizes within each batch were defined by facility capacity and by comparison with previous studies using Japanese quail as a model for understanding endotherm thermoregulation (e.g. Burness et al, 2013). Repetition of our study across three experimental batches enabled us to not only ensure sufficient sampling replication at the individual level, but also at the experimental level.
Data collection	Body mass measurements and both bill and tarsus length photographs were collected by E.P.,C.O.C., M.C., and J.T. Estimation of bill and tarsus lengths from photographs was conducted by J.T. Resting metabolism measurements were collected by E.P., C.O.C., M.C.,

	and E.T. To measure body mass, we used a digital precision scale. To measure bill and tarsus length, we used standardised photography methods with lengths being extracted from calibrated photographs in ImageJ2 (see "Data Collection" under "Software and Code" above). Resting metabolism and evaporative water loss were measured using flow-through respirometry at 3 and 8 weeks of age. For respirometry, birds were placed in glass chambers (replicates 1-2: 3.3 L at 3 weeks and 8.0 L at 8 weeks; replicate 3: 13.0L for all ages) held in a climate controlled cabinet and ventilated with dried atmospheric air. Initial air temperature in the climate cabinet was set to either 10°C (for birds from our first two experimental batches) or 30°C (for birds from our third experimental batch), then increased by 10°C increments until 40°C. Oxygen and water vapour density from subsampled air were measured throughout the experiment and measurements derived from the most stable two minutes of readings at a given temperature (as determined used ExpeData; Sable Systems, USA) were harvested for our analyses.
Timing and spatial scale	All experiments were conducted at the Department of Biology, Lund University, Lund, Sweden. Rearing temperature manipulation experiments (n = 3) began in 2021 and ceased in 2022 with short gaps between each (approximately 3 months) for data collation and animal processing.
Data exclusions	Metabolic data from five adults and two juveniles was excluded from our analysis owing to clear signs of distress lasting >5 minutes upon entry to metabolic chambers.
Reproducibility	To best varify reproducibility of our findings, each rearing temperature treatment was repeated at least twice, with new hatchlings derived from distinct egg batchs being placed in each treatment bout. Further, resting metabolism and morphology measurements were collected by four researchers, thus allowing researcher bias to be minimised. Repeated metabolism measurements within individuals at a given lifestage (i.e. maturity or during development) was not conducted to minimise distress imposed on research animals.
Randomization	Allocation of hatchling quail among rearing temperature treatments was semi-random, with sequential allocation of hatchlings following a predefined, but randomly chosen order (e.g. allocation to cold, then mild, then warm treatment respectively). Randomisation of allocation orders also varied across our three experimental "batches" of eggs.
Blinding	Blinding in this study was partial. Animal care and handling were predominantly conducted by E.P., C.O.C. and M.C., while statistical analyses were conducted by J.T. Analyses were therefore conducted without knowledge of individual behaviours and characteristics. Further, J.T. was blind to individual identity during extraction of bill and tarsus length measurements from digital photographs. Blinding of individual identity was not possible during body mass and resting metabolism measurement.

Did the study involve field work? ☐ Yes ☒ No

Reporting for specific materials, systems and methods

We require information from authors about some types of materials, experimental systems and methods used in many studies. Here, indicate whether each material, system or method listed is relevant to your study. If you are not sure if a list item applies to your research, read the appropriate section before selecting a response.

Materials & experimental systems

n/a	Involved in the study	
☒	☐	Antibodies
☒	☐	Eukaryotic cell lines
☒	☐	Palaeontology and archaeology
☐	☒	Animals and other organisms
☒	☐	Clinical data
☒	☐	Dual use research of concern
☒	☐	Plants

Methods

n/a	Involved in the study	
☒	☐	ChIP-seq
☒	☐	Flow cytometry
☒	☐	MRI-based neuroimaging

Animals and other research organisms

Policy information about studies involving animals; ARRIVE guidelines recommended for reporting animal research, and Sex and Gender in Research

Laboratory animals	Japanese quail ("Jumbo" strain) of both sexes were used in this study, with morphometric and physiological measurements being collected between hatch and maturity (9 weeks of age). Quail were sourced as eggs, incubated in-house, from a local breeder (Sigvard Månsgård, Åstorp, Sweden).
Wild animals	No wild animals were used in this study.
Reporting on sex	We were interested in testing whether the morphology of endotherms influences thermoregulatory efficiency across temperatures, regardless of biological sex. For this reason, both females and males were included in our sample population at haphazard and roughly equal proportions (65 females, 54 males).
Field-collected samples	No field data collection occurred in this study.

Ethics oversight	All animal handling, measurements, and euthanasia for this study were approved by the Malmö/Lund Animal Ethics Committee (permit no. 9246-19).

Note that full information on the approval of the study protocol must also be provided in the manuscript.

Plants

Seed stocks	N/A. No plants were used in this research program.
Novel plant genotypes	N/A.
Authentication	N/A.
